# Impact of diet on human nutrition, immune response, gut microbiome, and cognition in an isolated and confined mission environment

**DOI:** 10.1038/s41598-022-21927-5

**Published:** 2022-12-15

**Authors:** Grace L. Douglas, Diane DeKerlegand, Holly Dlouhy, Nathan Dumont-Leblond, Eden Fields, Martina Heer, Stephanie Krieger, Satish Mehta, Bridgette V. Rooney, Manolito G. Torralba, Sara E. Whiting, Brian Crucian, Hernan Lorenzi, Scott M. Smith, Millennia Young, Sara R. Zwart

**Affiliations:** 1 Human Health and Performance Directorate (SF4), NASA Johnson Space Center, 2101 NASA Parkway, Houston, TX 77058 USA; 2 JES Tech, Houston, TX USA; 3 KBR, Houston, TX USA; 4 Centre de Recherche de L’Institut Universitaire de Cardiologie Et de Pneumologie de Québec, Quebec City, QC Canada; 5 IU International University of Applied Sciences and University of Bonn, Bonn, Germany; 6 GeoControl Company, Houston, TX USA; 7 J Craig Venter Institute, Rockville, MD USA; 8 University of Texas Medical Branch, Galveston, TX USA

**Keywords:** Physiology, Psychology

## Abstract

Long-duration spaceflight impacts human physiology, including well documented immune system dysregulation. The space food system has the potential to serve as a countermeasure to maladaptive physiological changes during spaceflight. However, the relationship between dietary requirements, the food system, and spaceflight adaptation requires further investigation to adequately define countermeasures and prioritize resources on future spaceflight missions. We evaluated the impact of an enhanced spaceflight diet, with increased quantity and variety of fruits, vegetables, fish, and other foods rich in flavonoids and omega-3 fatty acids, compared to a standard spaceflight diet on multiple health and performance outcomes in 16 subjects over four 45-day closed chamber missions in the NASA Human Exploration Research Analog (HERA). Subjects consuming the enhanced spaceflight diet had lower cholesterol levels, lower stress (i.e. cortisol levels), better cognitive speed, accuracy, and attention, and a more stable microbiome and metatranscriptome than subjects consuming the standard diet. Although no substantial changes were observed in the immune response, there were also no immune challenges, such as illness or infection, so the full benefits of the diet may not have been apparent in these analog missions. These results indicate that a spaceflight diet rich in fruits, vegetables, and omega-3 fatty acids produces significant health and performance benefits even over short durations. Further investigation is required to fully develop dietary countermeasures to physiological decrements observed during spaceflight. These results will have implications for food resource prioritization on spaceflight missions.

## Introduction

Immune system dysregulation and other concerning physiological decrements occur during human spaceflight missions^1,2^. Given that immune function and the gastrointestinal microbiome are interlinked with diet^3^, there is potential to optimize diet to provide beneficial impact to physiology during spaceflight adaptation. Given the many constraints on space vehicles (e.g. mass, power, volume), medical resources may be limited, and the protective and therapeutic potential of food may become even more important during space missions. However, the food system is also limited by space vehicle resource, environmental, safety, and shelf life requirements^4^.

Food variety, choice, and appeal become more important as mission duration increases^5^, and can conflict with vehicle resource limitations and constraints. Lack of variety and acceptable choice may impact intake and body mass^6^, and may exacerbate the physiological adaptations of astronauts and health and performance outcomes that are critical to mission success. Additionally, foods rich in bioactive compounds are often low in energy density and therefore suffer logistically in spaceflight trades when compared to denser/higher fat foods. The relationship between dietary requirements, the space food system, and benefits to spaceflight adaptation must be better defined so that resource trades between dietary and other countermeasures can be adequately prioritized for future spaceflight missions. These trades parallel food concerns on Earth as the population grows and available resources for food production are constrained.

The few spaceflight studies investigating nutritional effects on physiological systems to date have focused on a few nutrients and outcomes^7,8^. The impacts of the whole spaceflight food system on multiple interrelated aspects of crew health, cognition, behavior, and performance have not been investigated. In this study, we determined the impact of increasing the variety and availability of healthy, shelf-stable, space flight compatible foods, including fruits, vegetables, fish, and other foods rich in flavonoids and omega-3 fatty acids, on the immune system, gastrointestinal microbiome, nutritional status, and cognitive outcomes in high-performing subjects completing 45-day space mission simulations in NASA’s Human Exploration Research Analog (HERA) habitat. We report herein that subjects consuming this enhanced diet had positive outcomes including lower cholesterol, lower stress (as measured by cortisol), better cognitive speed, accuracy, and attention, and microbiome status consistent with improved dietary intake over the course of the 45-day confinement compared to subjects consuming the current spaceflight diet.

## Materials and methods

This study was one of several experiments implemented in the Human Exploration Research Analog (HERA) Campaign 4, which consisted of four 45-day missions (C4M1, M3, M4, and M5, between April 2017 and July 2018 at the NASA Johnson Space Center (JSC) in Houston, Texas^9^. HERA facilitates research on astronaut-like individuals and teams in a high-fidelity simulated spaceflight mission environment. The HERA analog was suitable to implement a pre-defined food system in terms of nutrient content and serving sizes, and it is closed, meaning that new foods are not introduced during the mission. The HERA analog also implemented key mission-realistic parameters and stressors including isolation, confined volume, prescribed daily exercise, mission tempo schedules and tasks, and sleep deprivation. There was no programmed sleep shift/circadian misalignment in the mission design. Timelines for each mission were identical to reduce confounds between missions associated with shifting timelines and experimental manipulations. HERA C4M2 was truncated on day 23 due to Hurricane Harvey and was not included in this analysis. HERA C4M2 was replaced by C4M5 to complete the initial 4-mission plan.

### Subjects

Sixteen HERA subjects (10 men and 6 women; age: 40 ± 9 y; BMI (kg/m^2^): 23.7 ± 2.8; mean ± SD) participated in the study. Each subject participated in one mission. Subjects received training to maintain dietary records, collect biological samples, and perform cognitive tests.

### Dietary implementation, recording and analysis

Food provisioning and dietary intake were tracked for each mission. Pre-Mission, subjects consumed their own diets, ad libitum. Subjects were instructed to log their food and fluid intake for 15 days prior to the study, which aligned with pre-mission training and the beginning of biological collections. Subjects were provided a logbook to record details (date, time, quantity eaten, food/beverage consumed and method of preparation). An ACCULAB 5001 portable electronic scale was provided for weighing foods.

In-mission, the four HERA missions in Campaign 4 were randomized to one of two menu-controlled diets (current ISS diet, or enhanced diet), with two missions on each diet. Subjects were blinded to which diet their mission was consuming. The current standard spaceflight diet (control diet) was designed for ISS to provide nutritionally complete meals. The enhanced diet was developed to provide > 6 servings fruits and vegetables per day, > 2 flavonoid-rich foods per day, 2–3 servings (8–12 oz) of fish per week, and > 5 tomato-based (lycopene rich foods) per week, with protein maintained between 1.2 and 1.7 g/kg/day, calcium between 1000 and 1200 mg/day, vitamin D at 800 IU/day, sodium around 2300 mg/day, and iron around 10 mg/day.

### Food provisioning

Subjects did not have the option to select any components of their menu, which is consistent with future exploration mission profiles, and trading was not permitted, as each menu was set to meet nutritional requirements. Subjects were informed that the evaluation would provide mission relevant data for the dietary relationship with health and performance, and that it was important for them to eat the food provided to accurately inform spaceflight dietary requirements. Missions were randomized to a diet rather than individual subjects to allow blinding to the menu they were receiving and to prevent crew discontent based on randomization to different diets that would be unrealistic within the same mission (i.e. astronauts on the same mission receive similar diets based on mission resources and the role of food in crew cohesion).

Foods and beverages were provided by the Space Food Systems Laboratory (JSC, Houston, TX) identical to those provided on International Space Station missions. All food was stowed at the beginning of the mission, according to each subject’s caloric needs, to simulate mission resource constraints. Each HERA subject received the same base menu in each mission, designed to provide required nutrients with approximately 2300 cal per day^10^. Calories were added, as necessary to meet individual estimated energy requirements, calculated using the Dietary Reference Intake predictive energy equations for active, normal weight adults^11^. A small caloric overage (100–200 cal per person per day) was provided to ensure adequate provisioning but minimal choice, consistent with mission profiles. Subjects were instructed to consume the base menu prior to consuming additional calories, and to record actual consumption after each meal using the International Space Station (ISS) Food Intake Tracker (FIT) iPad App, developed for and currently in use on the ISS, and used previously in HERA missions^6^. Use of antibiotics and medications were recorded. Weight and height were obtained as part of pre-study clinical evaluations. During the mission, subjects measured their pre-breakfast weight daily on a calibrated BalanceFrom Scale (Model # BFHA-B400ST).

After each mission concluded, the dietary intake files were obtained from the server and the reports were transferred to the NASA Nutritional Biochemistry Lab. Pre- and in-mission diet records were analyzed using Nutrition Data System for Research software version 2015, developed by the Nutrition Coordinating Center, University of Minnesota (Minneapolis, MN). Files were output from the Nutrition Data System for Research into Microsoft Excel and separated out into food group servings (pre-mission logs), food/beverage intake data (in-mission) and nutrients per day (pre-mission and in-mission).

### Biological sample collections

Biological samples (blood, stool, urine, and saliva) were collected from each subject at five time points (two pre-mission and three in-mission) (Table S1). In-mission samples were collected in the HERA chamber and were transferred out through a pass through with no/minimal contact between the crew and support team. Blood samples were collected by trained personnel, while the subjects had their arm extended through a curtain, simulating robotic blood collection to prevent a break in isolation and confinement. Samples were transported in coolers with or without ice packs, as appropriate, from the HERA facility to the Nutritional Biochemistry Lab or the Immunology Lab at JSC. Blood sample processing began within one hour after collection.

All urine and stool samples were stored in coolers with ice packs inside the HERA chamber in a passthrough until they were removed by ground support personnel. Samples were stored at 4 °C for a total time of up to 24 h prior to returning to the lab. Urine samples were stored at 4 °C until the entire 48-h collection was complete. Volume of urine voids was determined, a 24-h pool was created, and samples were aliquoted and frozen at − 80 °C until post-mission batched analysis.

Within 24 h of collection, stool samples were aliquoted into tubes containing 2.5–3.5 mm glass beads (Millipore-Sigma) and DNA/RNA Shield (Zymo Research), vortexed for five minutes, incubated at 4 °C for 18–72 h, and then frozen at − 80 °C. Stool samples were shipped to JCVI on dry-ice for genomic and transcriptomic analysis.

Saliva samples were collected using a synthetic polymer swab (Salimetrics LLC, State College, PA)^12^, transported in coolers with ice packs, and stored at − 80 °C until batch processing. The timing of the daily sampling was purposefully maintained throughout baseline and mission (morning) such that normal circadian variables in cortisol throughout a 24 h period would not be a confounding factor. All samples were analyzed simultaneously at the end of all C4 missions.

### Biochemical analysis

Nutritional markers (e.g. proteins, fatty acids, vitamins, minerals), hormones, bone markers, oxidative stress markers, and renal stone risk markers were analyzed from blood, urine, and fecal samples either real time (e.g. whole blood testing), in batches analyzed after each analog mission, or in larger batches where possible to minimize batch effects.

Unless otherwise stated, biochemical analyses were completed in the JSC Nutritional Biochemistry Laboratory, an ISO-9000 and OSHA-VPP certified laboratory. In general, all radioimmunoassay (RIA) techniques used a gamma counter (Perkin Elmer, Waltham, MA). ELISA techniques used a SpectraMax Plus 384 spectrophotometer (Molecular Diagnostics, Sunnyvale, CA). Colorimetric assays utilized an Ace Alera autoanalyzer (Alpha Wasserman, Inc., Caldwell, NJ). Standard laboratory techniques were used for biochemical analyses. Table S2 (blood) and Table S3 (urine) summarize the tests, sample types, time of testing, and method for all tests.

#### Vitamins and flavonoids

Fat soluble and B-vitamins and associated biochemical markers were analyzed by liquid chromatography with tandem mass spectrometry (LC–MS/MS) by a commercial laboratory (Bevital, Bergen, Norway). Urinary gamma-carboxyglutamic acid (GLA) was analyzed using HPLC (Agilent Technologies, Santa Clara, CA); within- and between-assay CVs were 4.0 and 6.5%. Vitamin C was analyzed in lithium heparin plasma using HPLC (Agilent Technologies, Santa Clara, CA); within- and between-assay CVs were 2.6 and 8.0%. Flavonoids were determined in plasma (EDTA) and urine and were analyzed using LCMSMS by a commercial laboratory (Brunswick Laboratories, Southborough, MA).

Red blood cell folate concentrations and serum vitamin B12 were analyzed using a commercially available RIA (MP Biomedical, Santa Ana, CA; within- and between-assay CVs for RBC folate were 5.3 and 10.9%, respectively, and for serum vitamin B12 were 5.6 and 9.1%, respectively).

#### Calcium and bone health

Bone-specific alkaline phosphatase (BSAP) was determined in serum by ELISA (Quidel, Inc., San Diego, CA; within- and between-assay CVs were 6.7 and 7.1%, respectively). Serum propeptide of type 1 procollagen (P1NP) was determined using RIA (IDS, Tyne & Wear, UK); within- and between-assay CVs were 7.37 and 9.86%.

Collagen crosslinks, markers of bone resorption, were determined in serum and urine by ELISA: Serum N-telopeptide (NTX); Abbott Laboratories, Abbott Park, IL; within- and between-assay CVs were 4.6 and 7.9%, respectively); serum C-telopeptide β (CTX-β; Immunodiagnostic Systems, Tyne & Wear, UK; within- and between-assay CVs were 4.0 and 5.9%, respectively); urine NTX (Abbott Laboratories, Abbott Park, IL; within- and between-assay CVs were 3.7 and 7.9%, respectively); urine pyridinium crosslinks (PYD, Quidel, San Diego, CA; within- and between-assay CVs were 6.0 and 9.5%, respectively); deoxypyridinoline (DPD, Quidel, San Diego, CA; within- and between-assay CVs were 5.2 and 11.8%, respectively). Urine CTX was determined using ELISA (Crosslaps® EIA, IBL International GmbH, Hamburg, Germany); within- and between-assay CVs were 4.8 and 5.9%, respectively).

Serum parathyroid hormone (PTH) was determined using RIA (Scantibodies, Santee, CA); within- and between-assay CVs were 2.5 and 4.0%, respectively). Serum total and undercarboxylated osteocalcin were determined using by RIA (Alpco, Salem, NH); within- and between-assay CVs were 2.1 and 10.6%, respectively). Serum sclerostin was determined by ELISA (Quidel, San Diego, CA); within- and between-assay CVs were 5.1 and 8.7%, respectively. Serum osteoprotegerin (OPG) and receptor activator of nuclear factor kappa-Β ligand (RANKL) were determined by ELISA (OPG: Alpco, Salem, NH; within- and between-assay CVs were 13.0 and 16.1%, respectively; RANKL: BioVendor, Asheville, NC; within- and between-assay CVs were 4.5 and 11.3, respectively).

Serum 25(OH)-vitamin D was analyzed by liquid chromatography/tandem mass spectrometry (LC/MSMS) techniques by a commercial laboratory (Bevital Inc, Bergen, Norway). 1, 25-(OH)_2_-vitamin D was analyzed using chemiluminescence techniques by a commercial laboratory (ARUP Laboratories, Salt Lake City, UT).

Calcium in serum and urine were determined using atomic absorption spectrophotometry (Analyst 200, Perkin Elmer, Waltham, MA); within- and between-assay CVs were 2.9 and 3.5% in serum and 2.9 and 3.3% in urine, as previously reported^13–15^. Other minerals were analyzed using inductively coupled plasma emission spectrometry (Perkin Elmer NexION 350D; Perkin Elmer, Inc., Waltham, MA); respective within- and between-assay CVs in serum were: iron: 3.7 and 6.2%, copper: 3.0 and 3.5%, zinc: 1.0 and 3.5%, iodine: 2.9 and 3.7%, and selenium: 3.0 and 5.9%. In urine, respective within- and between-assay CVs were: copper: 1.7 and 3.5%, zinc: 1.0 and 3.5%, iodine: 1.0 and 3.6%, and selenium: 3.0 and 5.9%, as previously reported^16,17^.

#### Cardiovascular and fluid regulation

Serum aldosterone was determined by RIA (IBL International/TECAN, Mannedorf, Switzerland); within and between-assay CVs were 7.6 and 10.8%, respectively. Plasma angiotensin II (Angio II) and atrial natriuretic peptide (ANP) were determined by RIA (Phoenix Pharmaceuticals, Inc., Burlingame, CA); within- and between-assay CVs for Angio II were 8.1 and 20.0%, respectively; within- and between-assay CVs for ANP were 9.5 and 16.5%, respectively. Plasma (EDTA) renin was determined using RIA (Cisbo, Codolet, France); within- and between-assay CVs were 3.6 and 5.0%, respectively).

#### Metabolic, oxidative stress, and reproductive hormones

Serum insulin, insulin-like growth factor-1 (IGF-1)^13^, and leptin were determined by RIA (Insulin: Millipore Sigma, Temecula, CA, within- and between-assay CVs were 3.3 and 3.9, respectively; IGF-1: Alpco, Salem, NH, within- and between-assay CVs were 6.2 and 9.9, respectively; Leptin: LINCO Millipore, Saint Charles, MO; within- and between-assay CVs were 4.5 and 19.23%, respectively).

Oxidized LDL was determined by ELISA (Mercodia, Winston Salem, NC); within- and between-assay CVs were 6.3 and 8.8%, respectively, as previously reported^18^. Glutathione (total and reduced forms) was determined on whole blood samples by HPLC (Eagle Biosciences, Amhurst, NH); within- and between-assay CVs were 2.6 and 9.9%, respectively, as previously reported^18^.

Total antioxidant capacity was determined colorimetrically (Randox Laboratories, Kearneysville, WV); within- and between-assay CVs were 5.0 and 10.0%, respectively, as previously reported^18^. Glutathione peroxidase (GPX) was determined on whole blood samples using a colorimetric assay (Randox Laboratories, Kearneysville, WV); within- and between-assay CVs were 1.6 and 10.0, respectively, as previously reported^18^. Superoxide dismutase (SOD) was determined on whole blood samples using an inhibition assay (Randox Laboratories, Kearneysville, WV); within- and between-assay CVs were 3.5 and 9.6, respectively, as previously reported^18^. Total lipid peroxides (LPO) were determined colorimetrically (Bioxytech LPO 586, Oxis Research, Burlingame, CA); within- and between-assay CVs were 7.0 and 10.4%, respectively, as previously reported^18^. Plasma (EDTA) heme was determined colorimetrically (BioAssay Systems, Hayword, CA); within- and between-assay CVs were 5.6 and 7.8%, respectively.

8-hydroxydeoxyguanosine (8-OHdG) was determined in urine using liquid chromatography/tandem mass spectrometry (LC/MSMS) techniques (Waters Quattro, Waters Inc, Milford, MA)^19^; within- and between-assay CVs were 7.23 and 8.11%, respectively, as previously reported^8,18^. Urine 8-iso-prostaglandin F2α (PGF2α) was determined using ELISA (Oxford Biomedical, Riviera Beach, FL); within- and between-assay CVs were 14.5 and 19.8%, respectively), as previously reported^18^.

Cortisol was determined in serum and urine using LC/MSMS techniques (Waters Quattro, Milford, MA)^20^; within- and between-assay CVs were 1.64 and 5.71%, respectively, as previously reported^18,21^.

In male crewmembers, testosterone was determined in serum and urine using LC/MSMS techniques (Waters Quattro, Milford, MA)^22^; within- and between-assay CVs for serum were 8.5 and 8.7%, respectively, and for urine within- and between-assay CVs for serum were 4.7 and 8.7%, respectively, as previously reported^21^. In female crewmembers, estradiol was determined by RIA; MP Biomedical, Salon, OH); within- and between-assay CVs were 6.6 and 9.1%, respectively. Dehydroepiandrosterone (DHEA) and DHEA-sulfate (DHEA-S) were analyzed by ELISA (IBL International); within- and between-assay CVs were 7.6 and 10.4%, and 6.9 and 7.5%, for DHEA and DHEA/S, respectively.

#### Iron status and general chemistry

A complete blood count was performed by the JSC Clinical Laboratory (Houston, TX), using a Sysmex XN 1000 analyzer (Sysmex, Kobe, Japan). A serum basic metabolic panel and a lipid panel were completed on a Vitros 5600 autoanalyzer (Ortho Clinical Diagnostics, Raritan, NJ) by the JSC Clinical Lab. These panels included: albumin, (total) alkaline phosphatase (ALP), alanine aminotransferase (ALT), aspartate aminotransferase (AST), calcium, creatinine, glucose, lactate dehydrogenase (LDH), magnesium, phosphorus, total protein, sodium, chloride, total cholesterol, triglycerides, low density lipoproteins, (LDL), urea, and uric acid. High sensitivity CRP was analyzed using nephelometry using a Siemens BNII analyzer (Siemens,Tarrytown NY); within- and between-assay CVs were 3.6 and 5.9%, respectively, as previously reported^8,18^. The BNII was also used to analyze serum transferrin (within- and between-assay CVs were 6.1 and 4.8%, respectively), serum transthyretin (within- and between-assay CVs were < 8.0% and 5.97%, respectively), serum ceruloplasmin (within- and between-assay CVs were 3.5 and 4.8%, respectively); and serum retinol binding protein (within- and between-assay CVs were < 4.0% and 4.8%, respectively).

Serum transferrin receptors were determined by ELISA (Ramco Laboratories, Stafford, TX); within- and between-assay CVs were 5.7 and 11.4%, respectively, as previously reported^8^. Serum hepcidin-25 was analyzed by ELISA (DRG International, Springfield, NJ); within- and between-assay CVs were 10.0 and 12.6%, respectively, as previously reported^8^.

Total serum lipids were determined colorimetrically on the Ace Alera (Alpha Wasserman, Caldwell, NJ) using a kit from WAKO Diagnostics (Mountain View, CA); within- and between-assay CVs were 2.8 and 4.5%, respectively). Short and long-chain fatty acid composition of serum^23^, red blood cells^24^, and fecal samples were determined by a send-out laboratory at Duke University (Durham, NC) using quadrupole mass spectrometry (Waters Xevo TQ-S, Milford, MA) based on the work of Han, et al.^25^, and it was implemented in the Duke Proteomics and Metabolomics Core Facility (DPMCF).

Real time whole blood chemistries were determined using a portable clinical analyzer (iSTAT, Abbott, Abbott Park, IL), as previously reported^26,27^.

Urine chemistries (e.g. specific gravity, sodium, potassium, uric acid, magnesium, calcium, phosphorus) were completed by the JSC Clinical Lab using a Vitros 5600 (Ortho Clinical Diagnostics, Raritan, NJ). 3-methylhistidine was determined using HPLC techniques on an amino acid analyzer (LA8080 Amino Acid Analyzer, Hitachi, Dallas, TX); within- and between-assay CVs were 1.5 and 12.7%, respectively). Urine nitrogen was determined using chemiluminescence (Multitek Analyzer, PAC, Houston, TX; within- and between-assay CVs were 3.3 and 6.5%, respectively).

Renal stone risk assessments were determined using previously described methods^28^ which evaluate metabolic (i.e., calcium, potassium, oxalate, uric acid, citrate, creatinine, and pH) and environmental (i.e., volume, sodium, sulfate, phosphorus, and magnesium) factors to calculate an estimate of the relative supersaturation risk of five types of renal stones: calcium oxalate, brushite, sodium urate, struvite, and uric acid^28,29^. In addition to testing described above, ion chromatography (Chem K5500 System, Thermo Fisher, Waltham, MA) was used for citrate, sulfate, and oxalate analyses; within- and between-assay CVs were all below 15%, as previously reported^13,17,29^.

### Immunological analysis

Immunological analysis were performed as described previously^1^. Peripheral blood leukocyte distribution and T cell function were measured by multi-parameter flow cytometry, and plasma cytokine profiles, mitogen stimulated early blastogenesis, and mitogen-stimulated cytokine profiles were measured by magnetic bead multiplex immunoassay.

#### Peripheral leukocyte distribution (flow cytometry)

A white blood cell count (WBC) and differential was performed using a Beckman-Coulter Hematology Analyzer. The peripheral immunophenotype consists of leukocyte differential, lymphocyte subsets, CD4/CD8 ratio and memory/naïve T cell subsets. Levels of cytotoxic T cells, central memory T cells, and NK cell/B cell/monocyte subsets were also assessed by multicolor flow cytometry. Cell surface markers were stained by first combining 100 µl of EDTA whole blood and 10 ug of each appropriate labeled monoclonal antibodies. Staining was performed by incubation at room temperature for 20 min. Red blood cells were lysed using Beckman-Coulter Optilyse as described by the manufacturer. Stained leukocytes were then fixed in 1.0% paraformaldehyde in PBS for 10 min and analyzed on a Beckman-Coulter Gallios flow cytometer.

#### Immunocyte function (24 h cell culture—T cell early activation)

T cell function was assessed by culturing whole blood (150 µl) for 24 h in the presence of antibodies which trigger the TCR (anti-CD3) and provide co-stimulation (anti-CD28) or Staphylococcus enterotoxin A and B. Cultures were performed by combining 150 µl of whole blood, 1.0 ml RPMI media, 10 µl each of anti-CD3 and anti-CD28 or 10 µl each of SEA and SEB. Cultures were incubated for 24 h at 37 °C. Following incubation, 800ul of supernatant was removed and discarded from the cell pellet. T cell progression through a full activation cycle was monitored by determining the expression of CD69 (early activation) and CD25 (mid-activation, receptor for IL-2 that requires new gene synthesis). A four-color staining of cell surface markers (CD25/FITC, CD69/PE, CD8/ECD and CD3/PC5) was performed as described above. Flow cytometry was being performed on a Beckman-Coulter Gallios flow cytometer. The gating strategy consisted of T cell resolution and separation into CD4 and CD8 subsets, followed by enumeration of total CD69 + and CD69 + /CD25 + dual positive events.

#### Mitogen-stimulated cytokine profiles

For analysis of secreted cytokine profiles, 150 µl of whole blood was cultured in 1.0 ml RPMI media. Mitogenic stimulation was performed by adding 10 µl each of anti-CD3/CD28 (to activate T cells only via the TCR), 10 ng/ml of PMA and 2 µg/ml of ionomycin (as a broader pharmacologic stimulus), or 20 µg/ml of LPS (monocyte activation). Cultures were incubated for 48 h. Following culture, supernatants were removed and frozen until analysis. The 13-plex magnetic bead multiplex immunoassay (EMD Millipore) was used to assess the cytokine concentrations. The samples were processed according to the manufacturer’s instructions in a 96-well plate. Briefly, 25 µl of supernatant was diluted and incubated with magnetic beads coated with capture antibody. The beads were then washed and incubated with a biotinylated detection antibody, specific for each cytokine. This mixture was then incubated with Streptavidin PE conjugate, which is the reporter molecule. The samples were analyzed using a Luminex MAGPIX. All subjects’ samples were batch-analyzed to control inter-assay variability. Data was recorded as mean fluorescence intensity (MFI) to show subject relative cytokine production alterations throughout the mission, which were then converted to relative pg/ml concentration and multiplied by the dilution factor.

#### Plasma cytokine concentration

The concentrations for 30 plasma cytokines representing several broad categories (pro-, anti-inflammatory, Th1/Th2, growth factors, chemokines, etc.) were determined simultaneously in duplicate using a commercially available multiplex magnetic bead immunoassay provided by EMD Millipore. Samples were processed according to the manufacturer’s instructions in a 96-well plate, as described above. The samples were analyzed using a Luminex MAGPIX. All samples were batch-analyzed to control inter-assay variability. Data was recorded as mean fluorescence intensity (MFI) to show subject relative cytokine production alterations throughout the mission, which were then converted to relative pg/ml concentration.

### Salivary viral and cortisol quantification

Viral quantification and cortisol measurements were performed as previously described^12,30^. Samples were thawed on ice and then centrifuged at 2000 × *g* for 30 min to separate the fluid from the swab. The swab was discarded and the fluid was further separated into aliquots for viral DNA real-time PCR (500 µl) and salivary cortisol (200 µl) analysis. For Epstein-Barr Virus (EBV), Herpes Simplex Virus-1 (HSV-1), and Varicella Zoster Virus (VZV) quantification, DNA was extracted from the sample with a QIA-Amp DNA kit (Qiagen; Germantown, MD). Quantitative real-time PCR was performed in a QuantStudio 3 Real-Time PCR System (Thermo Fisher Scientific, USA) using fluorescence-based amplification. Primer sequences and probes for the herpes viruses (EBV, VZV and HSV-1) along with the glyceraldehyde 6-phosphate dehydrogenase (GAPDH) DNA sequences have been published previously^12^. Viral DNA standards generated from each herpes virus ranging from 10^0^ to 10^6^ copies/ml were included in all reactions. Reactions were performed in triplicates. A sample was considered negative for virus if it had lower than 20 copies of viral DNA. Cortisol was evaluated using an EIA kit (No. 1–3002/1–3012, Salimetrics, LLC, State College, PA), as previously described^12^.

### Taxonomic profiling of the gut microbiome

#### Taxonomic profiling sample preparation

Total DNA was extracted from stool samples using the MOBio/Qiagen PowerSoil DNA Isolation kit (Cat. No. 12888–50) following manufacturer instructions. PCR amplicons (~ 250 bp) spanning the V4 variable region of the bacterial 16S rRNA gene were generated from purified DNA using barcoded universal primers at their 5’ ends and pooled together to build a single Illumina sequencing library. To ensure enough sequencing depth per sample, up to 300 samples were multiplexed in a single 2 × 250 bp MiSeq Illumina run. After sequencing, samples had a mean number of 24,023 reads, ranging from 9335 to 70,455 reads. Reads were separated (binned) into individual samples based on matching barcode sequences at the 5’ end of the reads. Reads containing low quality values or chimeras were discarded.

#### Taxonomic Profiling

For taxonomic profiling of the gut microbiome, high quality sequences were clustered using a UPARSE-based pipeline^31^ and Operational Taxonomic Units (OTUs) were identified based on sequence similarity to known bacterial 16S rRNA gene sequences from the SILVA 16S rRNA database^32^ using the standard 97% sequence identity. Afterwards, rarefaction curves were computed to verify that sequencing depth was large enough to detect most species present in the sample. For estimating alpha diversity of the microbial communities in the stool samples, the Shannon-Weiner diversity index was calculated, while for estimating the total number of species present in a sample, the richness index was used.

Assessment of statistically significant changes in alpha diversity and richness of microbial communities associated to each type of diet versus mean pre-mission baseline values were carried out using linear mixed models using subject-specific intercepts as the random effect with the function lmer from the R package lme4^33^.

Determination of in-mission changes of phylogenetic profiles under each diet regime compared with their mean baseline values (Bray–Curtis weighted and unweighted beta diversity) were performed with Permutational multivariate analysis of variance (PERMANOVA^34^) with the R package adonis.

Identification of individual taxonomic groups of bacteria whose relative abundance changed between every in-mission time point and their respective pre-mission mean abundance values for each of the two dietary groups was performed with the R package DESeq2^35^ using a paired design. In all cases, the p-values were corrected using false discovery rate (FDR) procedure to correct for multiple hypothesis testing. Because of the exploratory nature of this ground-based study, taxonomic groups having at least a two-fold change in their abundance for at least one in-mission time point with a FDR < 0.25 were considered for further interpretation.

#### Correlation analysis between nutritional intakes and bacterium relative abundance in the intestinal tract

Identification of bacterial taxa whose relative abundance correlated with nutritional intakes such as serum cholesterol and iron levels of participating subjects was carried out using linear regression mixed models using subject-specific intercepts as the random effect with the lme function of the R package nlme. Correlations are not based on time points, just intake levels. Correlations were excluded if they had too many points at zero or too many points at the tail, which skewed the slopes and were unusable.

### Metatranscriptomics of the gut microbiome

#### Metatranscriptomic Sample Preparation

Metatranscriptomics analysis was performed on the same fecal samples used for taxonomic profiling. Total RNA was extracted from stool samples and the bacterial mRNA fraction were enriched by depletion of human and bacterial rRNA with Ribo-Zero kit (Epicentre®). Human polyadenylated mRNA represents just a small fraction of the total mRNA in feces and hence, no specific removal was required. The enriched bacterial mRNA was used to prepare barcoded directional RNA-seq libraries that were sequenced using Illumina HiSeq at a rate of eight samples per lane. Sequencing reads were further depleted of remaining rRNA and human RNA sequences by mapping reads to a database of rRNA (SILVA rRNAdb^32^) and the human reference genome respectively. Next, mRNA reads were assembled into transcripts with the assembler rnaSPAdes^36^. RnaSPAdes also assigned abundance values to each transcript based on the number of reads that map to them. Abundance values were normalized by the length of each transcript.

#### Metatranscriptomic analysis

For metatranscriptomic analysis of the gut microbiome, Open Reading Frames (ORFs) were identified in each transcript with Prodigal^37^. For predicting gene functions, ORFs were compared against PFAM^38^ and KEGG^39^ databases and ORF abundances were extrapolated from their coding transcripts. Identification of gene function categories (KEGG Orthologous genes, enzyme commission [EC] numbers and Pfam domains) that significantly changed between pre-mission and in-mission timepoints were carried out with the R package DESeq2 using a paired design. Identification of metabolic pathways that were differentially perturbed between the gut microbiomes exposed to standard and enhanced diets was carried out with the R package GAGE^40^. Changes in functional categories or pathways with an associated FDR p-value < 0.05 were considered significative.

### Cognitive analysis

Vigilant attention was evaluated using the Psychomotor Vigilance Test (PVT), a well-validated computerized test^41^. A 3-min PVT was administered on calibrated computers twice pre-mission, and then three time per week in-mission as part of the broader Cognition test battery^42^ (Table S1). Subjects monitor an empty rectangular box and press the space bar as quickly as possible when a millisecond timer appears. Primary performance outcomes include attentional lapses (responses longer than 355 ms), response accuracy (1-errors/total number of responses), and reciprocal response time (1/RT [seconds]), which is more sensitive than raw response time to cognitive effects of sleep restriction^43^. The PVT has negligeable practice effects for repeated administrations^44^.

### Statistical analysis

All measures were analyzed with generalized-linear mixed models (assuming the appropriate distributional family for each measure—linear models for continuous, negative binomial for counts like cognition lapses, and logistic for binary like viral reactivation). Subject-specific random intercepts were used to account for the repeated measures within subjects, and Mission-specific random intercepts addressed the clustering of crew within missions.

For microbiome-related measures, the mission-specific random effect was dropped since the samples were limited to two per crew per mission. Menu (Enhanced or Standard) and time (pre, early, mid, or late) were incorporated as categorical fixed effects with models defined by the interaction of menu and time. Expected marginal means were used to estimate mean response for each time and menu combination, as well as to conduct statistical comparisons of interest—changes from pre-mission within each Menu group, comparisons between Menu groups at each time point, and contrasts of mission-related changes (from pre) between Menu groups. The primary contrast of interest for each data type could be different. For nutritional intake, blood, and urine, the primary contrast was between menu types at each timepoint. However, for immune response variables, the focus is on the individual-level changes (pre to in-mission) and contrasting those changes between menus.

Residual plots were visually inspected to ensure normality assumptions were not violated. Some measures required log-transformation. For those measures modeled with non-identity link functions (non-linear), the marginal means were inverted and reported on the raw scale. All models were fit in SAS v9.4 using the GLIMMIX procedure. Marginal mean estimation and testing was conducted through the LSMEANS and LSMESTIMATE statements.

We chose to treat those observations below the detectable level as missing in the analysis. In the reported marginal means tables, the number of subjects and observations included in the modeling are reported for each measure. For graphing purposes, those below detectable level were shown at zero.

To obtain robust estimates of weight loss for individual crew, change in bodyweight from Day 1 was modeled for each crew, and predictions obtained for Day 45 (end of mission). Inverse-variance weighted meta-analysis of the predictions was used to summarize and test for differences in weight change between diets.

From the analysis results, key features were identified and incorporated into an integrated analysis. Data were summarized and harmonized by estimating mean values for each mission phase—pre, early, mid, and late. Repeated measures correlation using R’s rmcorr package was used to estimate correlation between measures, and hierarchical clustering based on (1-abs (corr)) distances was used to cluster measures. The results are visualized through a dendogram and heatmap of the correlations.

### Ethical approval and consent to participate

This protocol (PRO1634) was reviewed and approved by the NASA JSC Institutional Review Board. Informed written consent was obtained from all subjects prior to participation in the study. All methods in the study were carried out in accordance with relevant guidelines and regulations.

## Results

### Nutritional intake was improved for subjects consuming the enhanced diet

**Figure 1:**
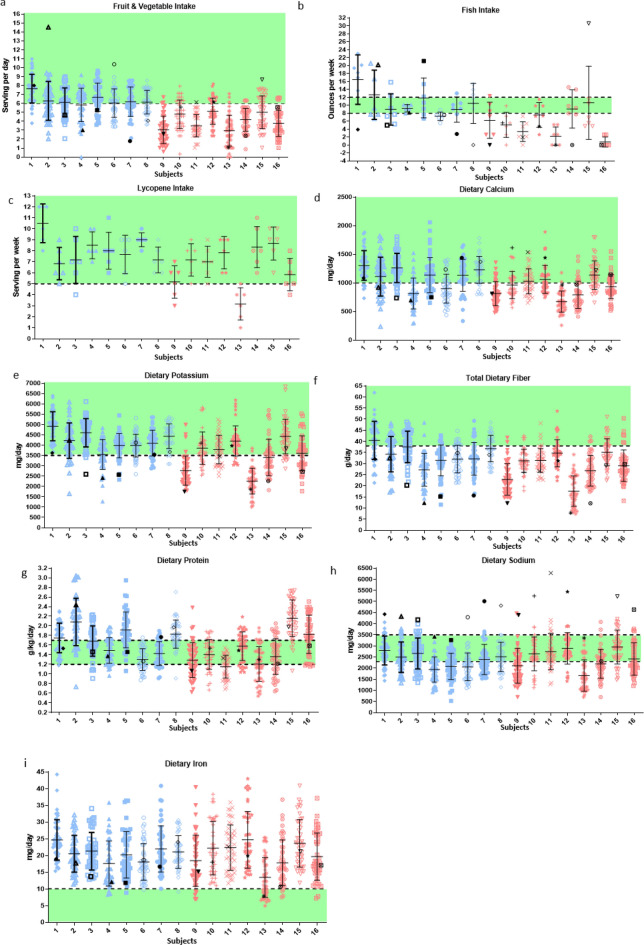
Daily consumption in-mission compared to pre-mission average. Each vertical set of samples represents one individual. (**a**) Fruit and vegetable, (**b**) fish, (**c**) lycopene, (**d**) calcium, (**e**) potassium, and (**f**) fiber intake were higher overall for subjects on the enhanced diet. (**g**) Protein (**h**) Sodium (**i**) and iron intake were similar on both diets. Black symbols denote pre-mission average, blue denotes daily enhanced diet intake, and red denotes daily standard diet intake. Green area represents target intake levels that met requirements. Subject numbers are consistent through all figures. Alternatively, for data over time in-mission, see SI Appendix*,* Tables S4,S5 for these and other nutrients.

HERA participants were provided one of two menu-based diets, either the current standard spaceflight menu diet or an enhanced menu diet with increased servings of fruits, vegetables, fish, and other foods rich in flavonoids and omega-3 fatty acids. Subjects tracked their intake daily both pre- and in- mission. On average, subjects under-consumed both diets (subjects consumed 84% of enhanced and 82% of standard caloric goals, P = 0.75) when compared to estimated requirements, with similar weight loss in both groups (enhanced menu subjects lost 0.70 kg ± 0.67 and standard menu subjects lost 1.14 kg ± 0.63, mean ± SE; P = 0.64), consistent with use of closed-menu spaceflight diets^45^. Although individual consumption varied (Fig. 1), on average, the enhanced menu subjects consumed 2.34 ± 0.34 more servings of fruits and vegetables per day (mean ± SE; P = 9.79E-12), 5.25 ± 1.52 more oz of fish per week (P = 0.00057), 1.25 ± 0.75 more servings of tomato-based, lycopene-rich foods per week (P = 0.098), 197 ± 75 more mg of calcium per day (P = 0.0087), 709 ± 272 more mg of potassium per day (P = 0.0092), and 5.58 ± 2.37 more g of fiber per day (*P* = 0.019) compared to standard menu subjects. Consumption of omega-3 fatty acids (ALA, EPA, and DHA) was greater on the enhanced diet (all *P* < 1E-10). Protein, sodium, and iron intake were similar on both diets. Iron intake was consistently higher than the target intake of < 10 mg/day both pre and in-mission, which was attributed to fortification in many commercially processed foods that are also used in the spaceflight diet (e.g. cereals, snack bars). Detailed nutritional intake data are available in SI Appendix, Tables S4,S5.

### General health status was similar between diets

General health outcomes, nutritional status, flavonoid levels, hormone levels, bone markers, and other outcomes of interest to spaceflight were evaluated at three time points during the 45-day study (early, mid, and late) from serum and urinary profiles. Serum and at least one 24-h urine sample was successfully obtained at each time point from each subject. Most markers did not vary between diets (SI Appendix, Tables S6,S7) over the 45-day analog duration. Serum ferritin concentrations decreased for most subjects during the mission, regardless of diet (SI Appendix, Fig. S1), even though dietary iron intake was above recommendations for all subjects through the mission (Fig. 1).

### Cholesterol status was improved for subjects consuming the enhanced diet

**Figure 2:**
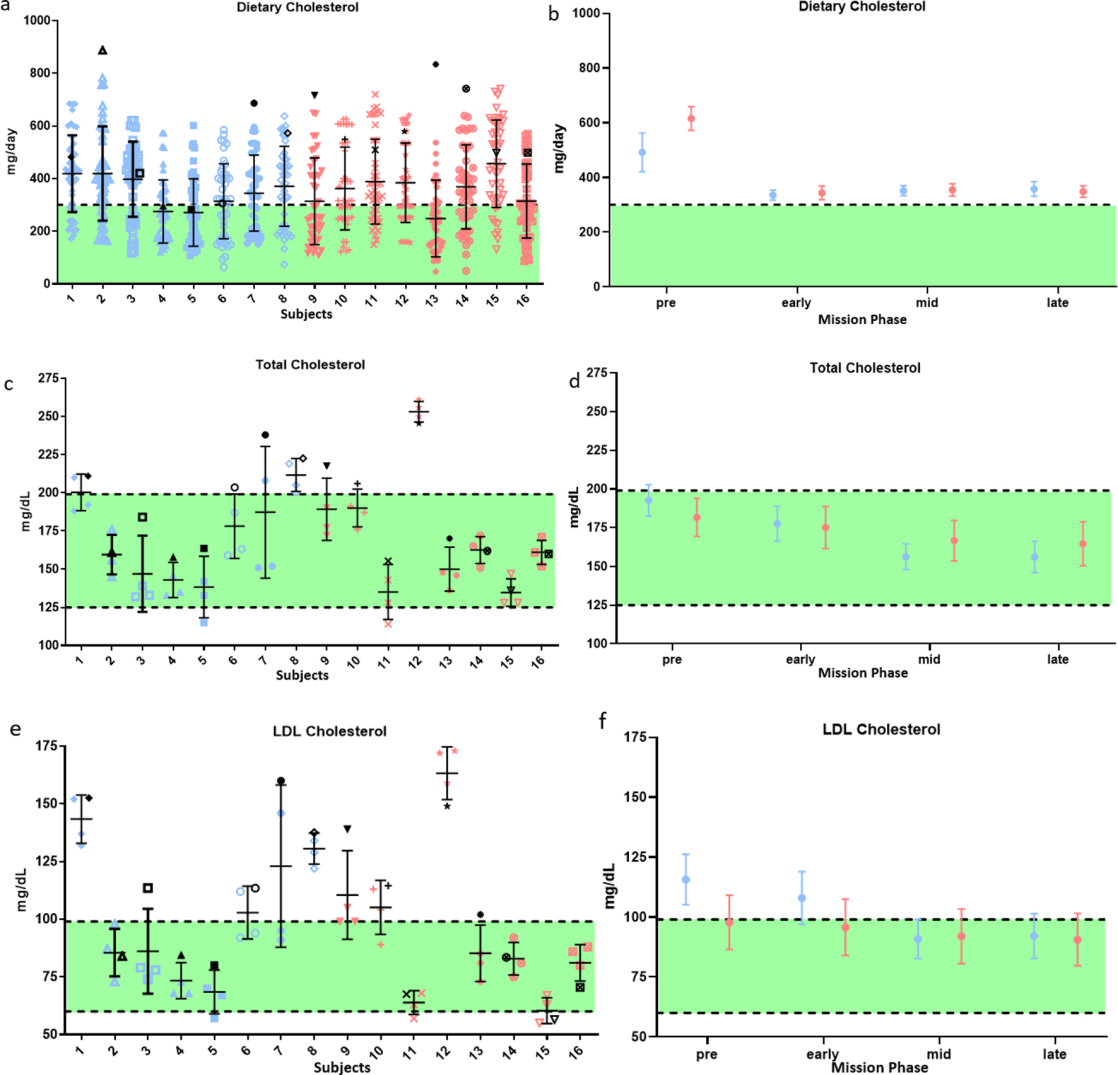
Cholesterol intake and serum status in-mission compared to pre-mission average. In a, c, and e each vertical set of samples represents one individual on the enhanced diet (blue) or the standard diet (red). Black symbols denote individual pre-mission average. In b, d, and f plots represent the mean ± SE of all subjects on the enhanced diet (blue) or the standard diet (red) during different phases of the mission. (**a,b**) Cholesterol intake was similar between diets. (**c,d**) Total cholesterol and (**e,f**) LDL cholesterol decreased more for subjects on the enhanced diet compared to the standard diet. Green area represents target intake levels that met requirements. More information is available in SI Appendix*,* Tables S5,S6.

Despite similar cholesterol and saturated fat intakes on both spaceflight diets, on average, subjects on the enhanced diet had a 16.6 ± 9.1 mg/dL greater reduction in total serum cholesterol (P = 0.07), and a 13.7 ± 8.3 mg/dL greater reduction in LDL cholesterol (P = 0.10), than subjects on the standard diet (Fig. 2). Only one of the eight enhanced diet subjects (subject 2) did not have a decrease in serum cholesterol compared to pre-mission. Dietary intake data indicate that subject 2 consumed the most fruit, vegetables, and fish before the mission (i.e., approximately 14 servings of fruit and vegetables per day compared to 6 on average during the mission), compared to all other subjects. Subject 2 also had the highest cholesterol intake pre-mission compared to other subjects.

### Physiological flavonoid concentrations increased for subjects consuming the enhanced diet

**Figure 3:**
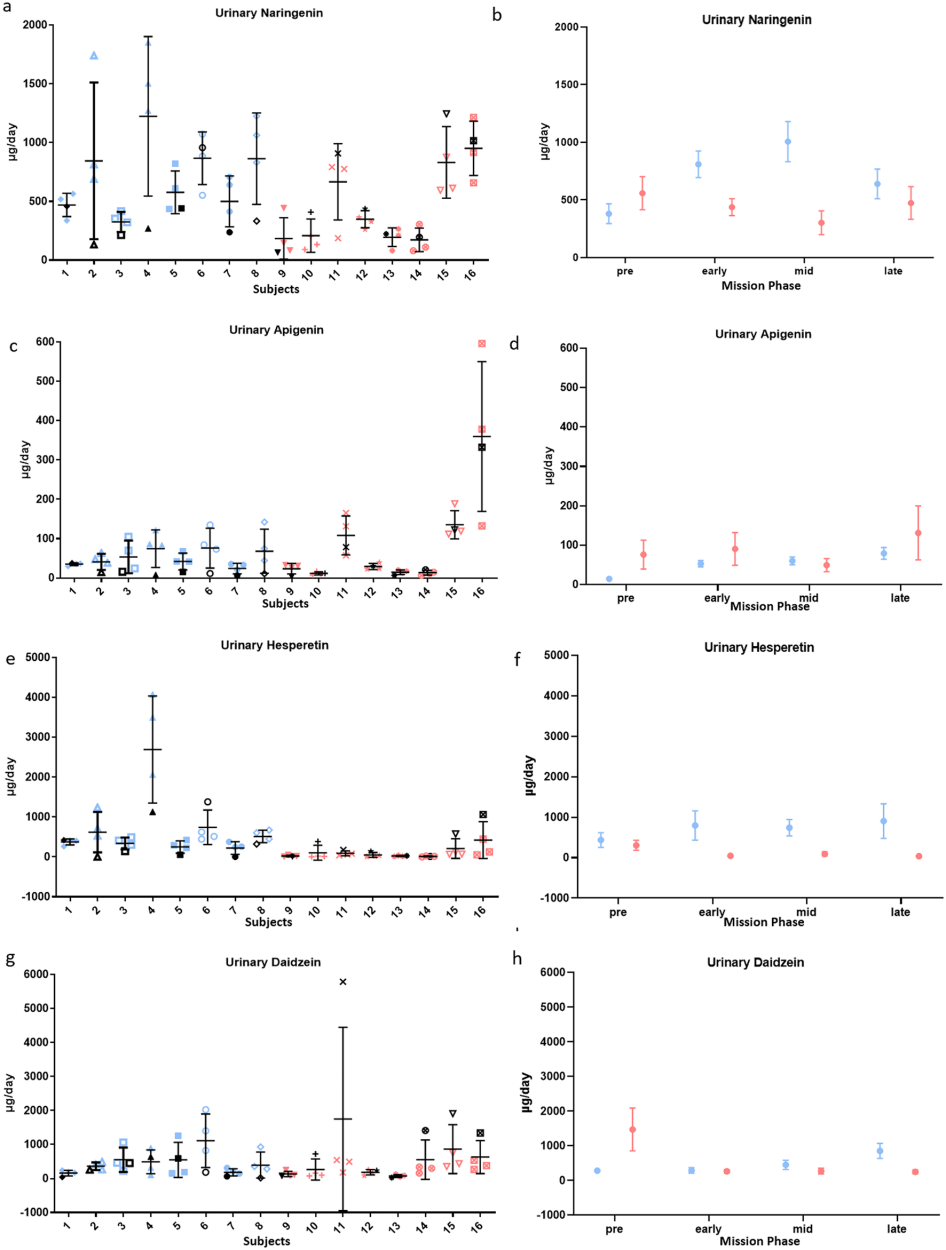

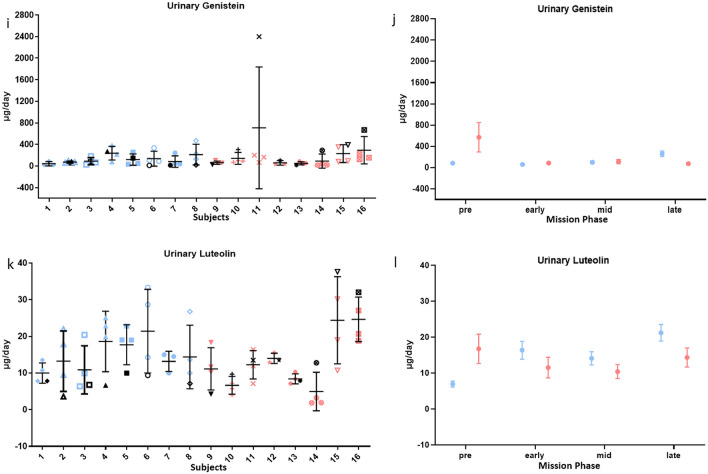
Urinary flavonoid status in-mission compared to pre-mission average. In (**a,c,e,g,i,k**) each vertical set of samples represents one individual on the enhanced diet (blue) or the standard diet (red). Black symbols denote individual pre-mission average. In (**b,d,f,h,j,l**) plots represent the mean ± SE of all subjects on the enhanced diet (blue) or the standard diet (red) during different phases of the mission. Urinary naringenin, apigenin, hesperetin, daidzein, genistein, and luteolin increased for subjects on the enhanced diet. More information is available in SI Appendix, Table S7.

Subjects consuming the enhanced diet had greater urinary excretion of several flavonoids, consistent with higher intake of fruits and vegetables. On average, subjects consuming the enhanced diet had a greater increase in urinary excretion of 6 of 13 urinary flavonoids in-mission compared to pre-mission than subjects on the standard diet. Subjects on the enhanced diet had 36.4 ± 13.6 µg/day more urinary apigenin (P = 0.0083), 1443 ± 609 µg/day more urinary daidzein (P = 0.019), 528 ± 276 µg/day more urinary genistein (P = 0.058), 624 ± 307 µg/day more urinary hesperetin (P = 0.044), 15.0 ± 3.6 µg/day more urinary luteolin (P = 0.00005), and 594 ± 178 µg/day more urinary naringenin (P = 0.0011), from pre-mission to in-mission than subjects on the standard diet (Fig. 3). Remaining urinary flavonoids did not show a substantial difference between diet groups or mission phase (P > 0.1).

Although some serum flavonoid differences appeared to be statistically significant, evaluation of raw data indicated a large portion of the data was below quantifiable limits, and there was no practical significance (SI Appendix, Table S7).

### Serum fatty acid concentrations were more stable for subjects consuming the enhanced diet

To determine if the increase in fish intake would alter fatty acid composition in the body, serum and red blood cell fatty acid profiles were measured (SI Appendix, Tables S8,S9). The concentrations of 9 out of 10 fatty acids increased in serum early in the mission for subjects on the standard diet but returned to pre-mission levels by the end of the mission. There were no substantial differences for subjects on the enhanced diet, nor differences in red blood cell fatty acid concentrations between diet groups or mission phase.

### Serum cortisol increased more for subjects consuming the standard diet

**Figure 4:**
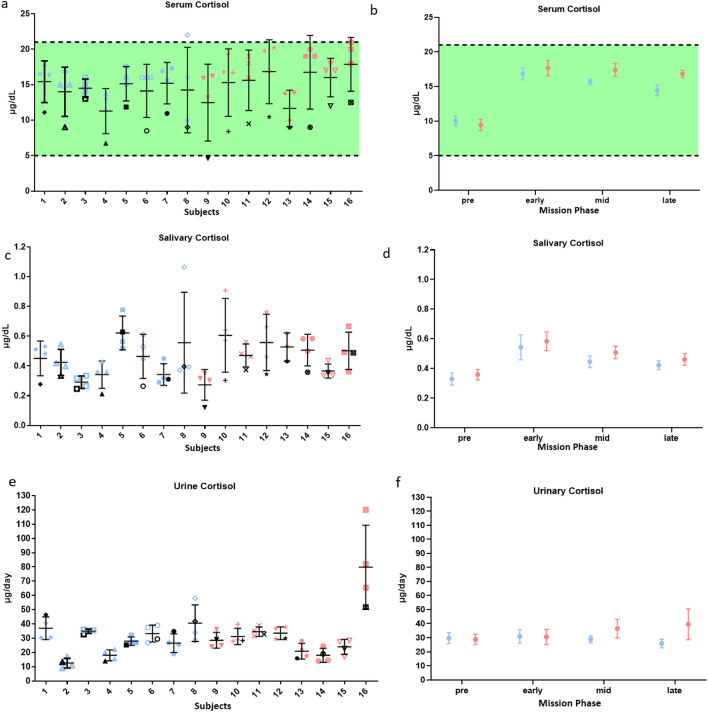
Serum, salivary, and urinary cortisol in-mission compared to pre-mission average. In (**a,c,e**) each vertical set of samples represents one individual on the enhanced diet (blue) or the standard diet (red). Black symbols denote individual pre-mission average. In (**b,d,f**) plots represent the mean ± SE of all subjects on the enhanced diet (blue) or the standard diet (red) during different phases of the mission. Serum cortisol increased more for subjects on the standard diet. Green area represents target intake levels that met requirements. More information is available in SI Appendix*,* Tables S6,S7,S10.

Cortisol, a measure of stress, was analyzed in serum, saliva, and urine (24 h pool). Serum and salivary cortisol increased for all subjects in-mission (Fig. 4, SI Appendix, Tables S6,S10). On average, serum cortisol increased more for subjects consuming the standard diet than the enhanced diet (2.22 ± 0.98 µg/dL; P = 0.027) (normal range = 5–21 µg/dL). On average, urinary cortisol was not different between dietary groups or mission phases for most subjects (Fig. 4, SI Appendix Table S7).

### No immune changes were associated with diet

Analysis of peripheral leukocyte distribution, T-cell function, plasma cytokine profiles, and mitogen-stimulated cytokine profiles shows that the majority of these parameters did not change due to diet or mission phase. A few markers were associated with some statistically significant alterations, although the health significance of the differences is considered minimal (SI Appendix*,* Tables S11–14).

EBV shedding was measured in the saliva of some subjects, but the outcomes did not associate by diet group, by mission phase, or with any changes in immunological outcomes (SI Appendix*,* Table S15). HSV-1 shedding was measured at low levels at one in-mission timepoint each in two standard diet subjects. One of the HSV-1 shedding subjects also had elevated plasma cytokines at all pre- and in-mission evaluations. No subjects shed VZV and no other adverse medical outcomes were recorded.

### Gut microbiome alpha-diversity and richness were reduced in-mission for subjects consuming the standard diet

**Figure 5:**
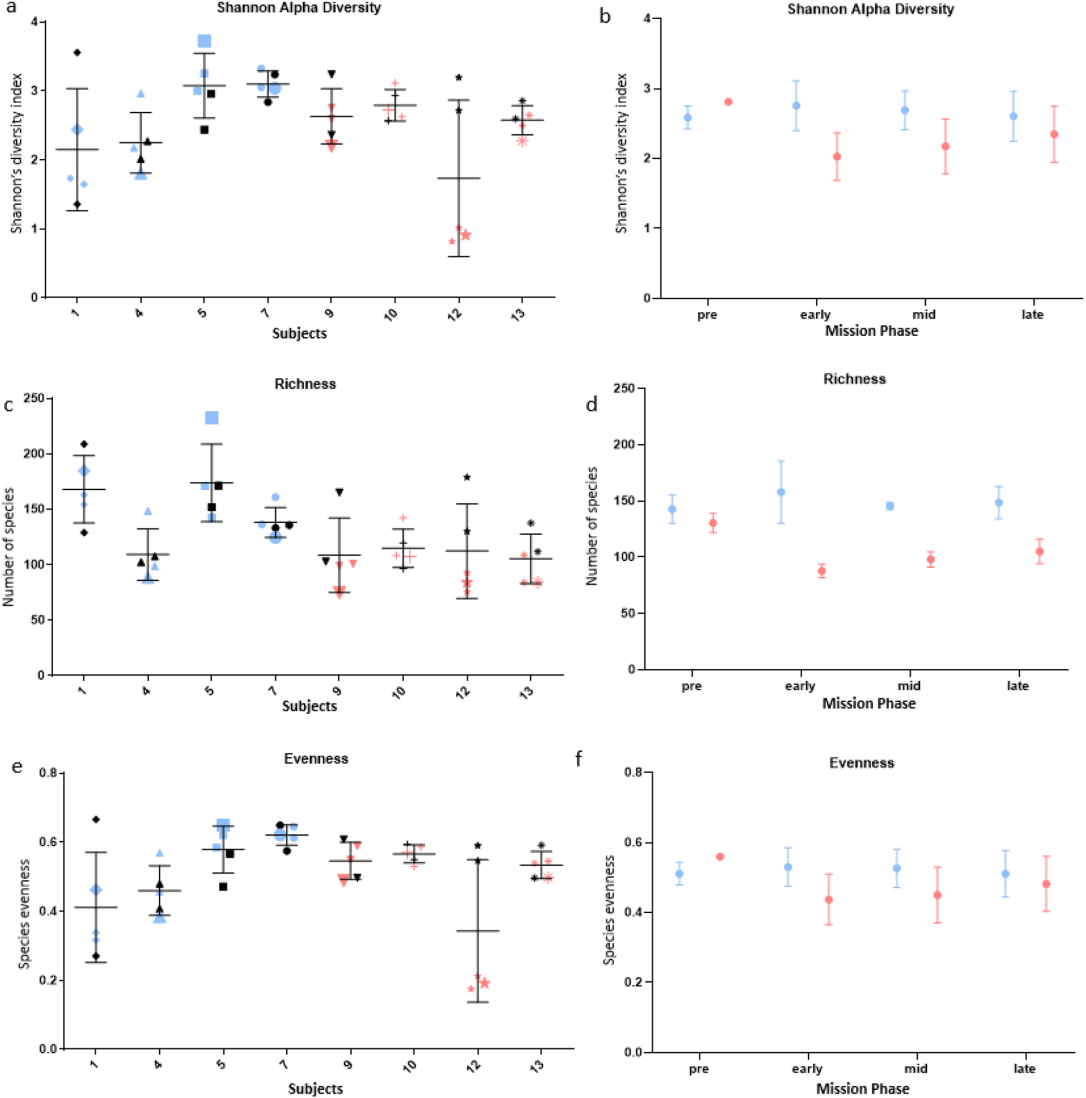
Variation in in-mission alpha diversity compared to pre-mission for a subset of subjects. Alpha diversity was more stable for subjects on the enhanced diet. Reductions in diversity (both richness and evenness) were significant early in the mission for subjects consuming the standard diet but returned to pre-mission levels by the end of the mission. In (**a,c,e**) each vertical set of samples represents one individual on the enhanced diet (blue) or the standard diet (red). Black symbols denote individual pre-mission average. Larger Red and Blue symbols indicate the early time point in-mission, with the most significant change for standard diet subjects. In (**b,d,f**) plots represent changes in alpha diversity mean ± SE of all subjects on the enhanced diet (blue) or the standard diet (red) during different phases of the mission. More information is available in SI Appendix, Table S16.

We next investigated the impact of HERA mission-associated stressors and diet on the diversity of the gut microbiome of a subset of subjects. This analysis showed that the mean alpha diversity, richness and evenness of the gut microbiota of the enhanced diet subjects was not statistically significantly different through the mission compared to their respective pre-mission values (for all comparisons, P > 0.4) (Fig. 5, SI Appendix*,* Table S16). However, the gut microbiome of subjects consuming the standard diet presented an early drop in Shannon’s alpha diversity (P = 0.05) and richness (P = 0.003) during the mission, followed by an ascending trend at later time points, fully recovering by mission day 43. For this group, microbial community evenness remained unchanged.

At the individual level, there were deviations from the patterns described above. Within the enhanced group, subject 1 showed a descending trend in Shannon’s alpha diversity during mid and late time points, while within the standard diet group, subject 10’s alpha diversity and richness remained unchanged throughout the mission.

### Gut microbiome beta-diversity was more stable for subjects consuming the enhanced diet

**Figure 6:**
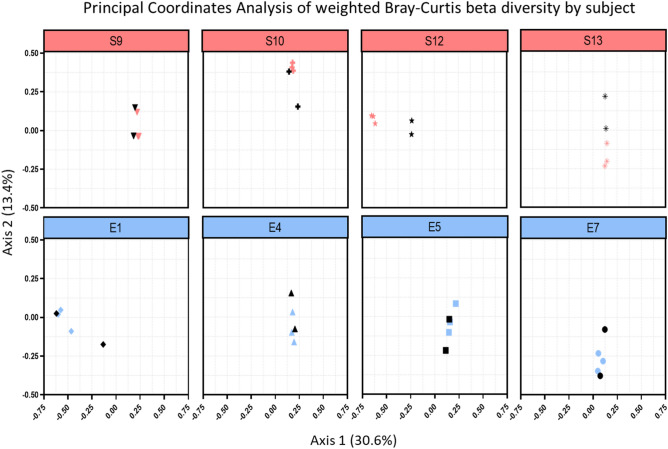
PCoA of weighted Bray–Curtis beta diversity in-mission compared to pre-mission for a subset of subjects. Subjects on the enhanced diet (blue) showed more stability in both richness and evenness between all pre- and in-mission samples compared to subjects on the standard diet (red). Black symbols denote pre-mission values.

To gain further insights into the effect of mission stressors and diet on the gut microbiota, we analyzed changes in the taxonomic composition of the bacterial communities of the gastrointestinal tract of a subset of subjects. Comparative analysis between pre- and in-mission time points did not identify any significant quantitative (weighted beta diversity, permanova R^2^ = 0.1, P = 0.15) (Fig. 6) nor qualitative (unweighted beta diversity, permanova R^2^ = 0.1, P = 0.09) (SI Appendix*,* Fig. S2) change in the microbial composition of the gut microbiome of subjects consuming the enhanced diet. On the contrary, the in-mission composition of the gut microbiome of subjects consuming the standard diet was moderately different from pre-mission samples, both quantitatively (weighted beta diversity, permanova R^2^ = 0.16, P = 0.003) (Fig. 6) and qualitatively (unweighted beta diversity, permanova R^2^ = 0.14, P = 0.008) (SI Appendix Fig. S2). This shift in microbial composition was particularly evident for subjects 12 and 13 within the standard diet group and was already noticeable five days within the mission. On the contrary, no in-mission associated change was apparent in subject 9.

Further analysis indicated that quantitative changes in the gut microbiota during the mission were 0.24 ± 0.02 (mean diff ± SE) smaller than natural variations before the mission for the standard diet group (P < 0.001), while no statistically significant difference was evident within the enhanced group (0.16 ± 0.11; P = 0.14). In addition, variations in the relative abundance of intestinal bacteria during the mission were smaller for subjects consuming the standard diet compared with those consuming the enhanced diet (0.10 ± 0.04; P = 0.02).

### Dietary and bacterial correlations were identified

**Figure 7:**
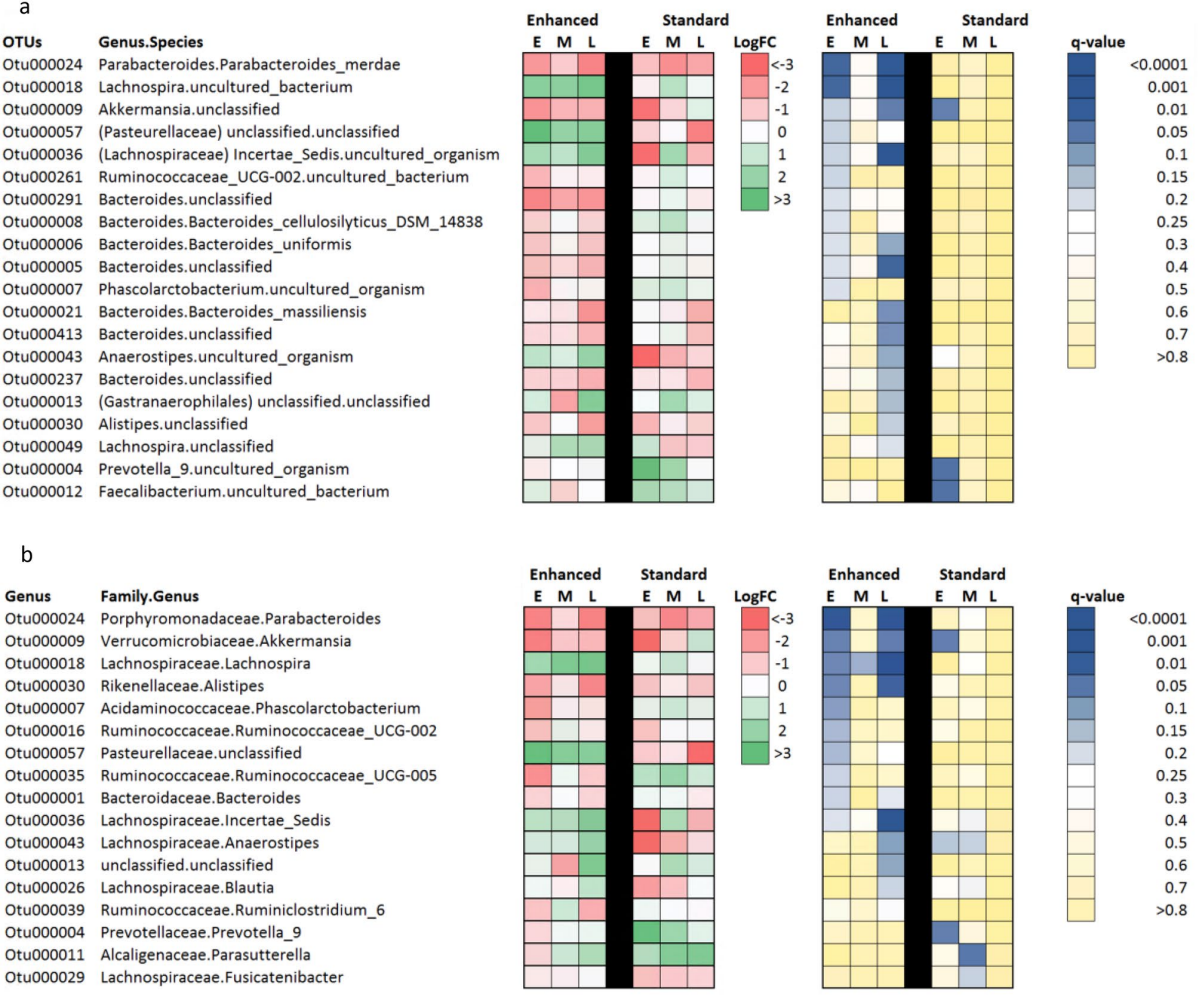
Associations between diet and OTUs. Differences were identified in 20 OTUS at the species level (**a**) and 17 at the Genus level (**b**). Both pre-mission timepoints were used as a baseline, FDR < 0.25. E = early, M = mid, and L = late time in mission. Changes in the abundance of taxonomic groups were estimated with the R package DESeq2 (v 1.28.1, https://bioconductor.org/packages/release/bioc/html/DESeq2.html) [PMID: 25516281] and heatmaps representing their associated Log2(Fold Change) and FDR (q-values) were generated in Microsoft Excel (v 16.63.1).

Within the same subset of subjects, a few significant associations were identified between changes in microbial abundance and diet. In the enhanced group, the abundance of 18 bacterial species significantly changed in-mission compared to preflight samples (q-value < 0.25). Most of these changes were more evident at early and/or late time points (Fig. 7). In contrast, changes in the abundance of only three species were observed in the standard group at the beginning of the mission. Further analysis revealed an overall in-mission reduction in the abundance of several *Bacteroides ssp.* and increases in four uncharacterized bacterial species of the Lachnospiraceae family in the enhanced group, while no differences were found for these species in the standard group. A bacterial species of the genus *Akkermansia* presented a significant drop in abundance in both dietary groups during the mission. These results were also evident at the genus level (Fig. 7b).

Further evaluation into associations between OTU changes and intake levels of specific nutrients by this subset of subjects revealed several positive and negative associations (Table 1). Among them, we found a positive association between intestinal *Bacteroides ssp.* abundance and cholesterol intake (SI Appendix*,* Fig. S3).

**Table 1:** Association between microbiome abundance at the species (top) or genus (bottom) level and nutrient intake concentration averaged over the 3 days prior to the biological collection.

OTU ID	Family. Genus. Species	Intake	p-value	R^2^
Otu000024	Porphyromonadaceae. Parabacteroides. Parabacteroides merdae	Cholesterol (mg/dL/day)	0.039	0.8
Otu000001	Bacteroidaceae. Bacteroides. unclassified	Omega.3.fatty.acids (g/day)	0.004	0.92
Otu000001	Bacteroidaceae. Bacteroides. unclassified	Calcium (mg/day)	0.023	0.87
Otu000001	Bacteroidaceae. Bacteroides. unclassified	Sodium (mg/day)	0.028	0.94
Otu000001	Bacteroidaceae. Bacteroides. unclassified	Cholesterol 3 days	0.002	0.93
Otu000413	Bacteroidaceae. Bacteroides. unclassified	Cholesterol 3 days	0.008	0.96
Otu000012	Ruminococcaceae. Faecalibacterium. uncultured_bacterium	Fruits and vegetables (servings/day)	0.022	0.9
Otu000226	Ruminococcaceae. Faecalibacterium. Unclassified	Fruits and vegetables (servings/day)	0.018	0.94

–	Family. Genus	Intake	p-value	R^2^
–	Porphyromonadaceae. Parabacteroides	Cholesterol (mg/dL/day)	0.036	0.8
–	Bacteroidaceae. Bacteroides	Sodium (mg/day)	0.019	0.88
–	Bacteroidaceae. Bacteroides	Cholesterol (mg/dL/day)	0.006	0.86

Three days has previously been shown to be adequate for dietary evaluation in relation to the microbiome^24^.

### Metatranscriptomic profiles of the gut microbiome were more stable for subjects consuming the enhanced diet

**Figure 8:**
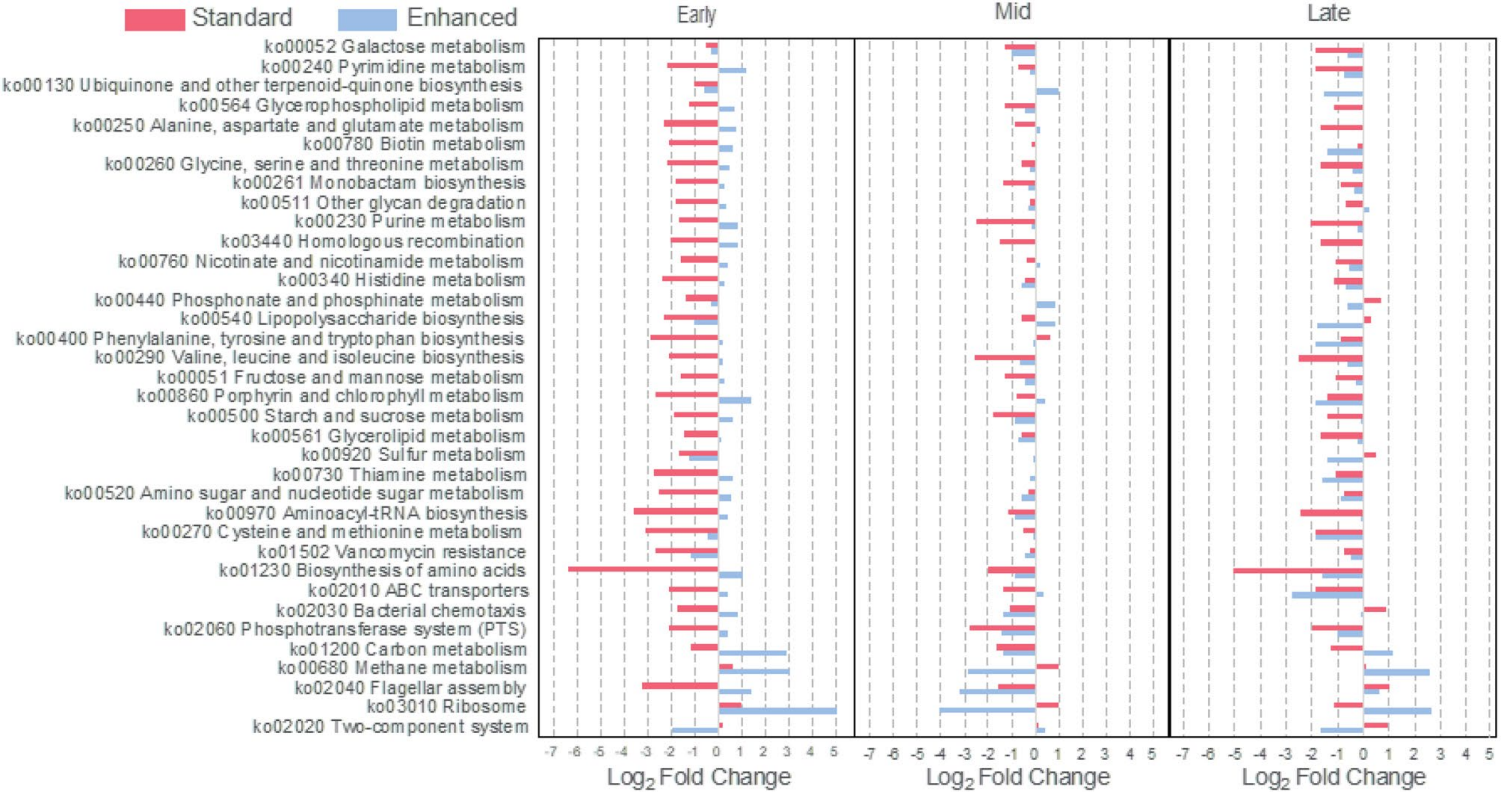
Metatranscriptomic changes in-mission compared to pre-mission for a subset of enhanced diet subjects (average fold changes in blue) and standard diet subjects (average fold changes in red). Several pathways were downregulated for standard diet subjects early in the mission but began to normalize by mid-mission. SI Appendix*,* Fig. S5 shows changes by individual for the ‘Early’ timepoint in the mission.

The gut microbiome of subjects consuming the enhanced diet had more stable metatranscriptomic profiles during the mission than subjects on the standard diet when considering overall number of significantly perturbed metabolic pathways (Fig. 8) or individual gene functions (SI Appendix*,* Fig. S4) compared to pre-mission samples. Several pathways for synthesis of amino acids, small sugars, lipid metabolism, membrane transport, and biosynthesis of biotin, vitamins B6 and B1 and bacterial vitamin K (menaquinone) were down-regulated in the microbiome of subjects consuming the standard diet by day 5 in-mission (Fig. 8). The metatranscriptomic profiles of the microbiome began to normalize by mid-mission towards the pre-mission baseline and remained more similar between diets through the end of the mission. Although there was individual variability in this trend (SI Appendix*,* Fig. S5), the down-regulation of the amino acid synthesis pathways was evident in the microbiome of most standard diet subjects. Similarly, the microbiome of subjects consuming the standard diet had a higher number of enzymatic gene functions, KEGG orthologs, and PFAM conserved protein domains perturbed than the enhanced diet subjects (SI Appendix*,* Fig. S4, Tables S17–19).

### No difference in short chain fatty acid concentration was associated with diet

Although we did not find any significant differences in short chain fatty acid pathways in the microbiome, we measured the concentration of short chain fatty acids from fecal samples to determine if the increase in fiber on the enhanced diet had any impact. Consistent with pathway data, although there were some minor changes, no trends or substantial increases were found (SI Appendix*,* Table S20).

### Cognitive speed, accuracy, and attention were better for subjects consuming the enhanced diet

**Figure 9:**
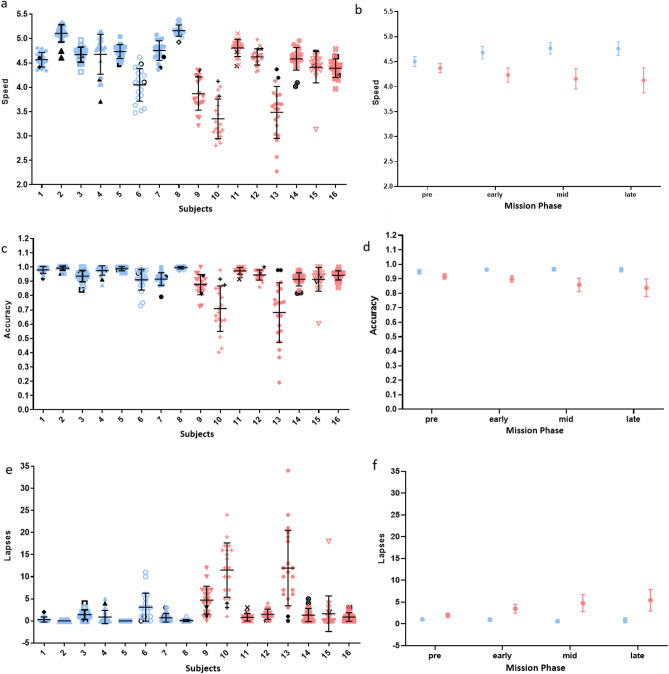
PVT speed, accuracy, and lapses in-mission compared to pre-mission. On average, subjects on the enhanced diet had improved speed compared to pre-mission, higher speed and accuracy compared to standard diet subjects, and fewer attention lapses compared to standard diet subjects. In (**a,c,e**) each vertical set of samples represents one individual on the enhanced diet (blue) or the standard diet (red). Black symbols denote individual pre-mission average. In (**b,d,f**) plots represent the mean ± SE of all subjects on the enhanced diet (blue) or the standard diet (red) during different phases of the mission. More information is available in SI Appendix, Table S21.

Scores on a Psychomotor Vigilance Test (PVT) (published previously^46^) were evaluated to determine cognitive outcomes in relation to dietary intake throughout the mission. There were no statistically significant PVT performance differences between diet groups pre-mission (SI Appendix*,* Table S21). Enhanced diet subjects performed better in evaluations of response speed and response accuracy than standard diet subjects throughout the mission (Fig. 9). For subjects consuming the enhanced diet, reaction speed was significantly improved over pre-mission, starting early in the mission (P = 0.024), and was higher than subjects consuming the standard diet throughout the mission (P = 0.014). Accuracy was also higher for enhanced diet subjects during the mission than standard diet subjects (P = 0.022). Finally, subjects consuming the enhanced diet experienced fewer attention lapses than subjects consuming the standard diet during the mission (P = 0.0047) (Fig. 9).

## Discussion

An enhanced diet resulted in improved nutritional outcomes, more stable intestinal microbial communities at both the taxonomic and metatranscriptomic level, and improved cognitive outcomes during 45-day HERA missions. The results indicate the importance and synergy of the whole diet, as improvements could not be attributed to individual nutrients.

All subjects consumed 83% of their estimated energy intake targets. This intake was higher than those observed in previous closed food system analogs^6^, and was consistent between enhanced and standard diet. This indicates that the higher proportion of vegetables, fruits, and fish in the enhanced diet was as acceptable as the standard diet, which alternatively had more availability of energy-dense desserts, snacks, and starch-heavy side dishes. However, intake may have been confounded by the sleep deprivation schedule associated with these missions, which allowed for 5 h of sleep per night for 5 nights each week and 8 h of sleep per night for two nights each week. Sleep deprivation has been associated with increased food consumption^47^, and may have improved food consumption in these missions. However, the subjects commented consistently in debriefs that despite not getting to choose their foods pre-mission, the variety and quality available was adequate for this mission length.

**Figure 10:**
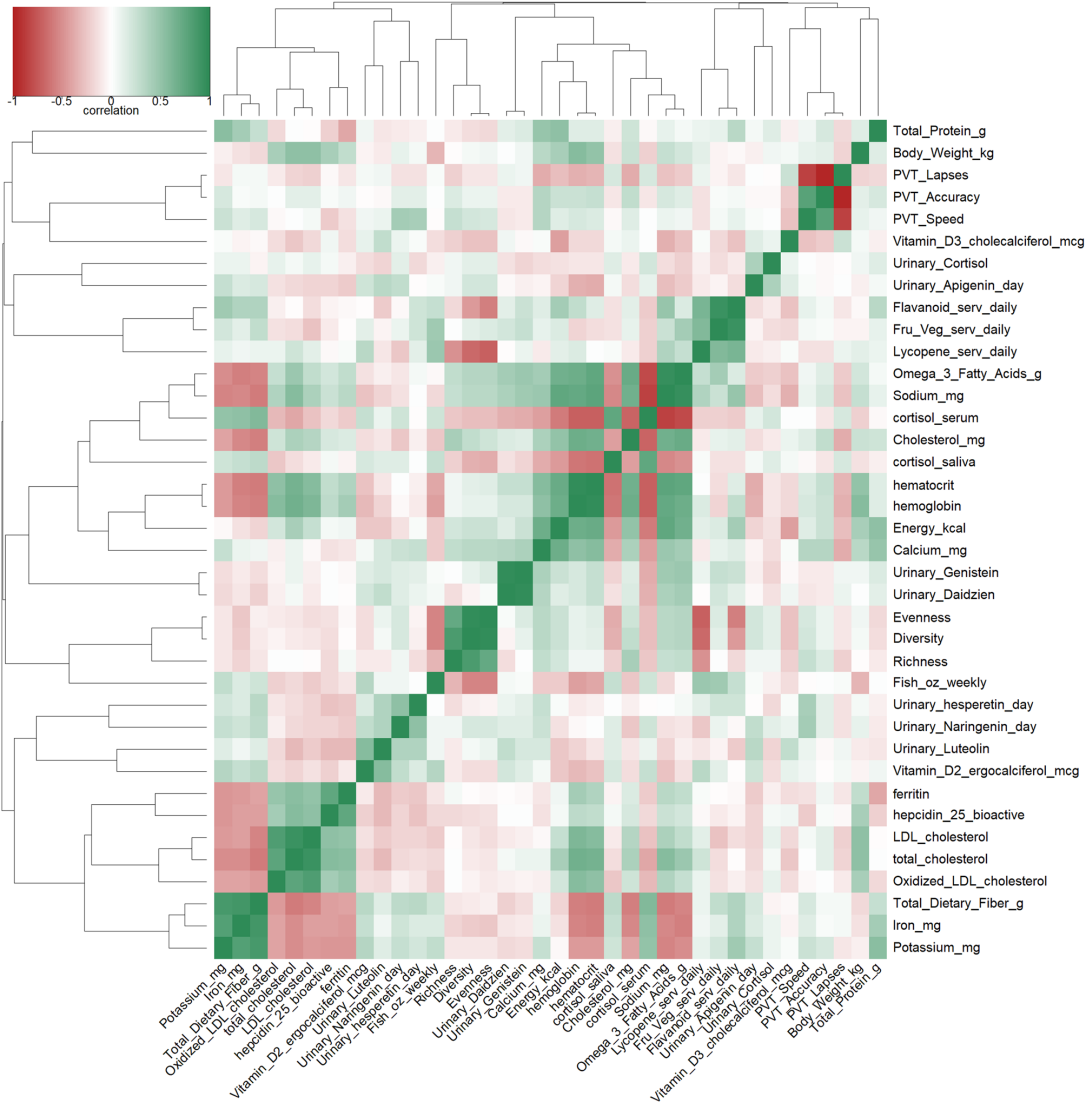
Correlations between dietary factors and physiological outcomes indicate a complex interaction of factors within a whole diet. Single nutritional factors may not always associate as expected with physiological outcomes, but overall, the enhanced diet resulted in beneficial physiological and cognitive outcomes. Data was analyzed and visualized in R v4.1.3 using the rmcorr, ggplot2, gplot2, dendextend, colorspace, and RColorBrewer packages. Graph was rendered with the heatmap.2() function within gplots.

Individual evaluation showed that during the analog some subjects had reduced access to and intake of some of the target foods, such as fruits, vegetables, and fish compared to pre-mission intakes although they met the dietary targets of the enhanced diet (Fig. 1). Additionally, even though provided with a specific set of foods during each mission, individual choice factored into nutritional intake, especially given underconsumption of available foods. However, the improvements in cholesterol intake and outcome levels on both diets and urinary flavonoid levels on the enhanced diet indicate that overall, subjects’ diets improved in-mission, even with the shift to a fully processed, shelf stable food system. These results indicate the complex interaction of factors within a whole diet, and single nutrients may not always associate as expected with physiological outcomes (Fig. 10).

The only subject on the enhanced diet (subject 2) that showed no change in cholesterol outcomes during the mission (Fig. 2) had regularly consumed more fruits and vegetables as well as cholesterol pre-mission than the enhanced target in-mission (Figs. 1,2). Pre-mission intakes may have reduced the impact of the diet in-mission for this subject compared to other subjects.

During orbital spaceflight numerous immune alterations are documented to persist throughout a 6-month mission. These include reductions in T and NK cell function, the persistent reactivation of several latent herpesviruses, and adverse clinical outcomes in some crewmembers (atopic dermatitis, atypical allergy, infections)^2,30,48^. Confinement in the HERA ground analog for 45 days does not simulate the radiation, microgravity, levels of danger, and associated stress of spaceflight. Although HERA subjects showed some statistically significant immunological changes and low levels of EBV and HSV1 shedding, they were below levels of clinical concern, and were lower than previous changes associated with spaceflight^2,30^. Both EBV and HSV1 are shed asymptomatically in the general population at levels similar to what was observed in the HERA subjects^49–51^.

There was an observed increase in the stress hormone, cortisol, in both saliva and serum. Dietary quality has previously been associated with stress and behavioral impacts^52^, and here serum cortisol increased more for subjects on the standard diet compared to the enhanced diet (Fig. 4). Although cortisol increases have previously been associated with immune changes and viral reactivation^53^, no association was found here. This may be due to differences in subject variability, small subject numbers, time of collection, whether samples were pooled, or other factors.

Although no substantial immunological changes were found in this study, there was also no analog-induced reduction in immune function, nor any immune challenge from infection, illness, or vaccination. In the absence of immunological deficit or challenge, there was also an absence of opportunity to reveal a difference in immune response related to diet. In a recent mouse study, immune challenge was necessary to induce immune response, and demonstrate the potential beneficial effects of dietary improvements^54^. It is possible that the limited length of these ground-based missions, the absence of spaceflight stressors such as radiation and microgravity, and the absence of a specific immune challenge, such as an infection, did not reveal the full potential of the enhanced diet as a countermeasure in this study.

Similarly, the increased urinary concentrations of several flavonoids are indicative of increased intake, but associated benefits may not have time to emerge over 45 days. Apigenin, found in multiple fruits, vegetables, and herbs, has antioxidant, anticancer, and neuroprotective mechanisms that may provide long term benefits in the radiation environment of deep space^55^. Luteolin, naringenin, and hesperetin commonly associated with citrus, have antioxidant and anti-inflammatory properties^56^. This could be beneficial given the oxidative damage documented in spaceflight^7^. Evidence is also beginning to emerge of the role of the microbiome in flavonoid metabolism, production of bioactive metabolites, and increased bioavailability^57^.

Of note when evaluating data for a small population, there was one subject with consistently elevated plasma cytokines at all pre- and in-mission evaluations. Although not clinically relevant, and not impacted by diet or mission phase in this study, it could indicate some inflammation. In combined analysis, this would influence in-mission comparisons (SI Appendix*,* Table S11), but contrasting the within-subject change from pre-mission levels between diets eliminated impacts such as these in our analysis.

Microbiome data further supported potential benefits associated with enhanced dietary intake. Similar to previous studies that demonstrated that diet can alter the microbiome within days^58^, a drop in alpha diversity was measured early in-mission for standard diet subjects. Drops in gut microbiome alpha diversity and richness have been associated with microbial dysbiosis and deleterious effects to human health^59^. However, the reductions measured early in-mission for subjects consuming the standard diet returned to pre-mission levels within 45 days, demonstrating some resilience with time. Similarly, beta-diversity significantly shifted for subjects consuming the standard diet from pre to in-mission but was stable during the mission. Alternatively, both alpha and beta-diversity were stable for subjects consuming the enhanced diet. This aligns with previous study results, in which consuming a more diverse diet, including fruits and vegetables, resulted in a more stable microbiome^60^.

Few taxonomic differences were noted between dietary groups. Although *Bacteroides* spp. associated with cholesterol level^61^, no species here were found to contain cholesterol metabolizing enzymes. Notably, both dietary groups showed a reduction in *Akkermansia* spp., associated with the intestinal anti-inflammatory response^62^. Although no substantial inflammatory changes were measured in this study, subjects did experience increases in cortisol, indicating an increase in stress. Reductions in *Akkermansia* spp. in relation to stress were similarly shown in a previous study with mice^63^. Alternatively, increases in *Akkermansia* spp. may have probiotic potential in stressful situations^64^.

Metatranscriptomics of the gut microbiome was performed to investigate if changes in microbial community structure were accompanied by more widespread shifts in gene expression of the gut microbiome. Many pathways for amino acid synthesis were down-regulated in the microbiome of subjects consuming the standard diet by day 5 in-mission. This may seem counterintuitive, as we might expect this more for the enhanced subjects, with greater dietary variety and nutritional substrate availability from the subjects’ diets requiring less of a need for substrate synthesis. However, this does support greater stability of the microbiome on the enhanced diet in general^60^. Overall, this could indicate a more robust microbiome, which may be less susceptible to perturbation. Although both diets provided similar amounts of basic macro-nutrients, including sugar and protein, they were combined differently with other components in the diet, which could trigger different responses in the microbiome.

Although it would be speculation at this point, it is possible that the significant unexpected increases in some serum fatty acids are similarly impacted by an integrated impact of the whole diet, and their interaction with the microbiome. In this study no metatranscriptomic differences were found in fatty acid synthesis pathways between diets or timepoints, and further investigation would be required with a larger subject number to investigate this possibility further. Similarly, short chain fatty acid concentrations can vary widely. Although differences were not revealed here, studies that show changes generally evaluate larger dietary differences or larger subject numbers^58,65,66^.

Research from this same HERA analog campaign demonstrated an overall adverse impact of sleep restriction on PVT performance throughout the mission, regardless of dietary condition^46^. The current findings offer evidence supporting a mitigation effect on attentional performance related to the enhanced diet. While the PVT is not a direct measure of operational performance, it does contribute to the prediction of simulated spacecraft docking performance^67^ and fitness-for-duty-like classifications for luggage screeners^68^, as well as correlating with rates of serious medical errors made by resident physicians^69^. Therefore, these findings support continued exploration of dietary countermeasures to mitigate behavioral health and performance risks of spaceflight.

Overall, subjects consuming the enhanced diet had improved nutritional intake that associated with several improvements in markers of health, stress, and cognitive performance, as well as greater stability in the microbiome and metatranscriptome compared to subjects consuming the standard diet. Although subject number is low in this study, the longitudinal experimental approach, the complete control of dietary availability during the mission, and collection of substantial pre-mission baseline data for each subject provided important insights into impacts from the complete spaceflight diet. This evaluation also addresses some of the limitations of previous studies, which have included evaluation of only one dietary factor at a time and use of food recall and general food frequency questionnaires instead of full dietary tracking with inability to access foods external to the evaluation^70^.

## Conclusions

This study provides evidence of health and cognitive performance benefits from an enhanced spaceflight diet starting early in a mission. This study also provides important ground-based baseline data for spaceflight diets to compare with spaceflight data that has added stressors such as microgravity and increased levels of radiation, where alterations in bone and oxidative stress markers have been demonstrated^7^. Further investigations are needed to define specific resource and risk trades between dietary and other potential spaceflight countermeasures. Food is the one potential countermeasure that will absolutely be included on future exploration-class space missions. Optimizing the benefits and minimizing any risks will enable safe exploration far beyond low-Earth orbit.

## Supplementary Information

Supplementary Information.

## Data Availability

All datasets generated and/or analyzed during this study are either included in this published article and its supplementary information files or can be requested through https://lsda.jsc.nasa.gov/.
