# Supplementary material for: Impact of diet on human nutrition, immune response, gut microbiome, and cognition in an isolated and confined mission environment
Source: Sci Rep. 2022 Dec 15;12:20847. doi: 10.1038/s41598-022-21927-5 (PMC9755260; doi:10.1038/s41598-022-21927-5)
Supplement: Supplementary file 1 — Supplementary Information. [file 41598_2022_21927_MOESM1_ESM.pdf]

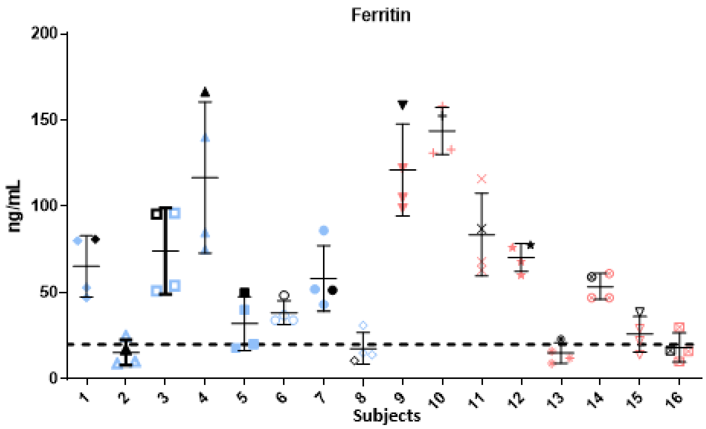

Fig. S1. Serum ferritin status in-mission compared to pre-mission average. Each vertical set of samples represents one individual. Serum ferritin decreased for 10 of 16 subjects in-mission, with no specific association with either diet. The dotted line indicates the lower limit. Black symbols = pre-mission average, Blue = enhanced diet subject, Red = standard diet subject. Alternatively, for data over time in-mission see Table S6.

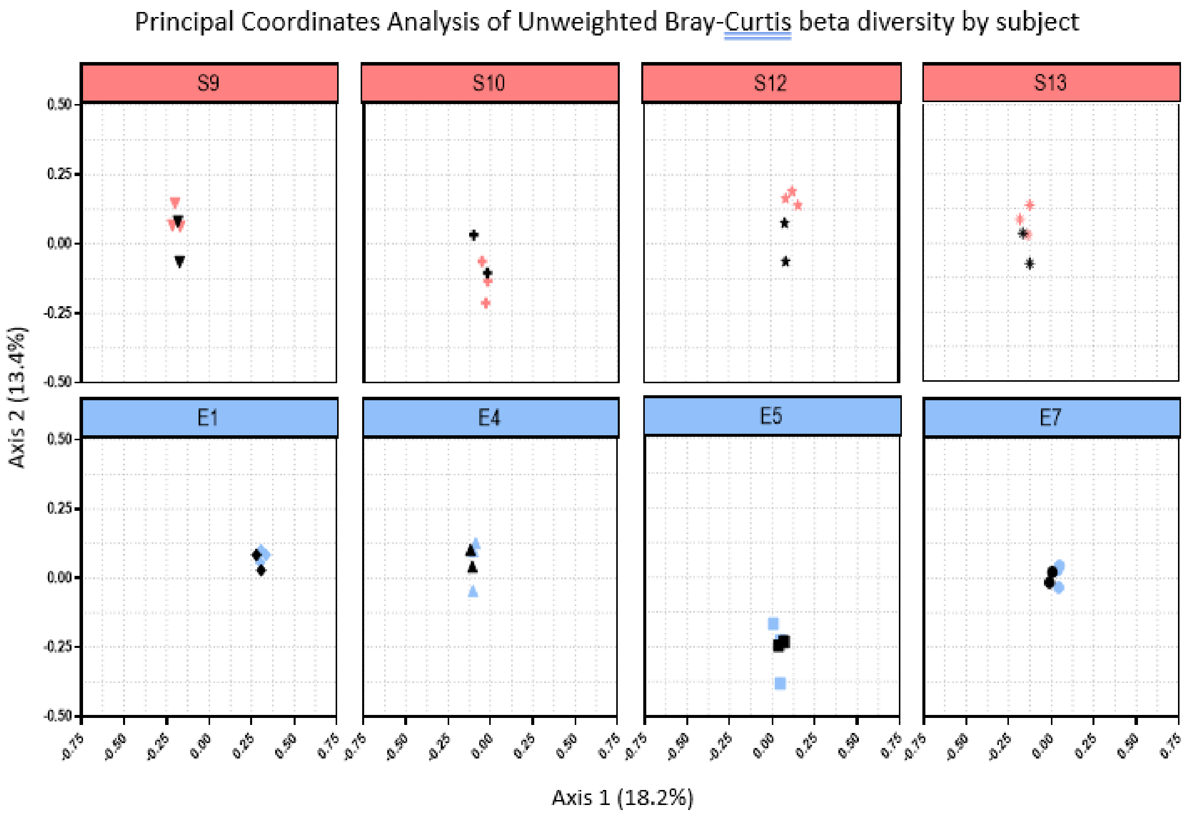

Fig. S2. PCA of unweighted Bray-Curtis beta diversity in-mission compared to pre-mission for a subset of subjects. Subjects on the enhanced diet (blue) showed more stability in richness between all pre- and in-mission samples compared to subjects on the standard diet (red). Black symbols = pre-mission values.

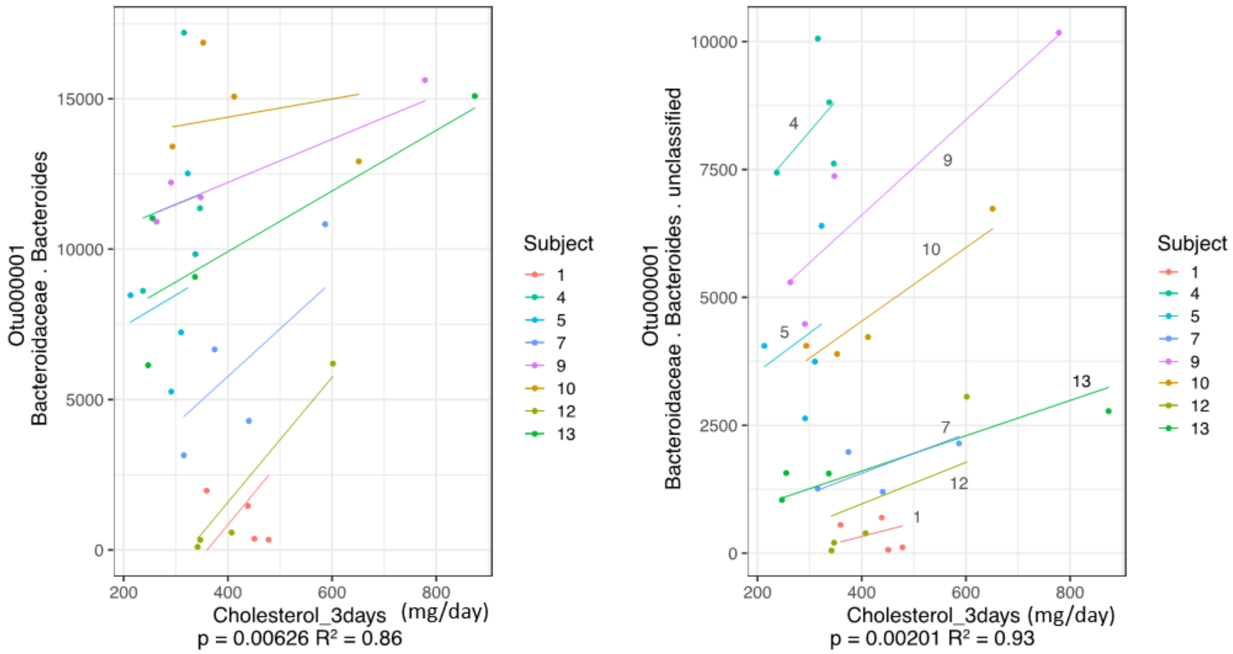

Fig. S3. Bacteroides OTU 000001 correlated with cholesterol intake for most subjects on both diets at both the genus (A) and species (B) level. Enhanced diet subjects are 1, 4, 5, 7, and standard diet subjects are 7, 10, 12, and 13.

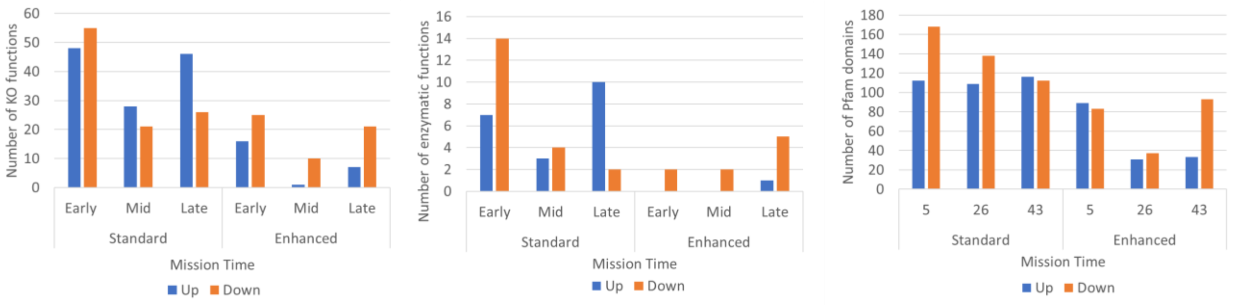

Fig. S4. Differential expression of KEGG Ortholog (KO) functions, enzymatic functions, and Pfam domains was greater for subjects consuming the standard diet than the enhanced diet in-mission compared to pre-mission.

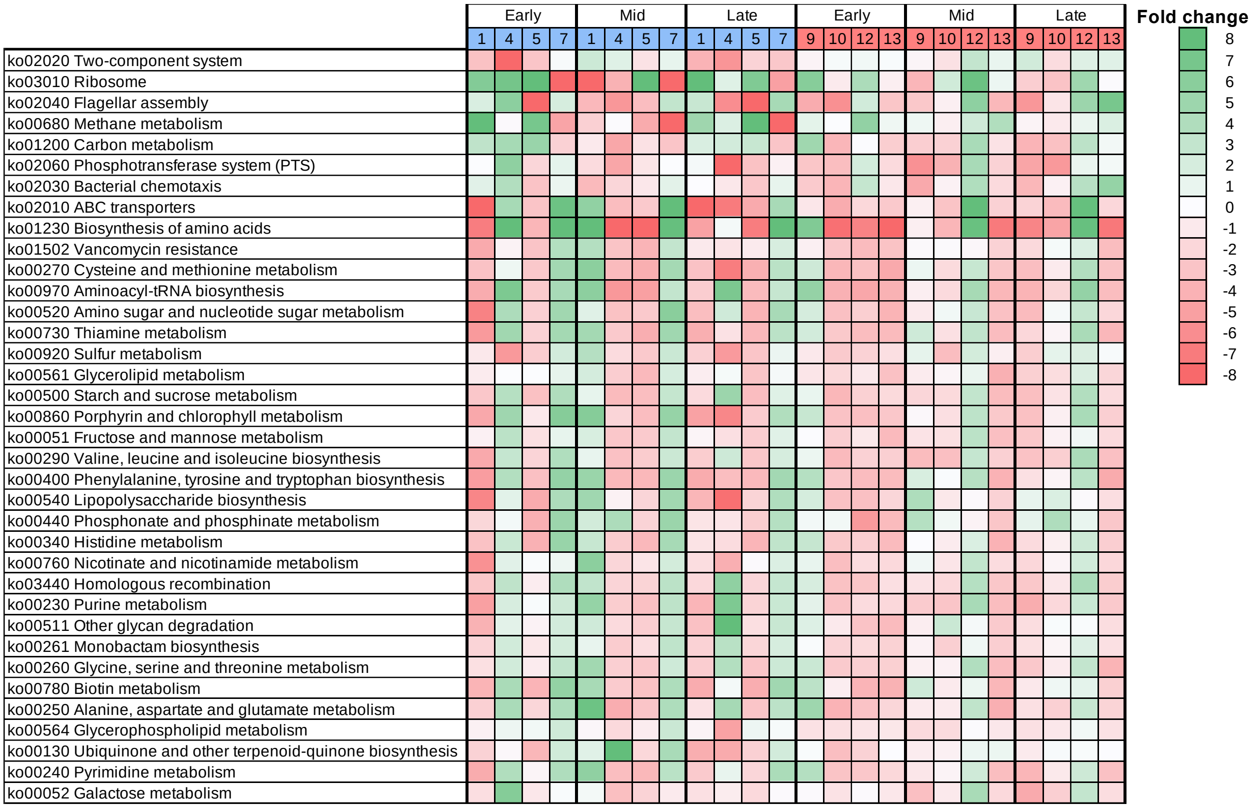

Fig. S5. Changes in the metatranscriptome early in the mission by individual. Although more downregulation was apparent for standard diet subjects (10, 12, 13) there was individual variability in this trend. Blue numbers = enhanced diet subjects; red numbers = standard diet subjects.

Table S1. HERA schedule for blood, urine, stool, saliva, and cognition testing in relation to sleep schedule

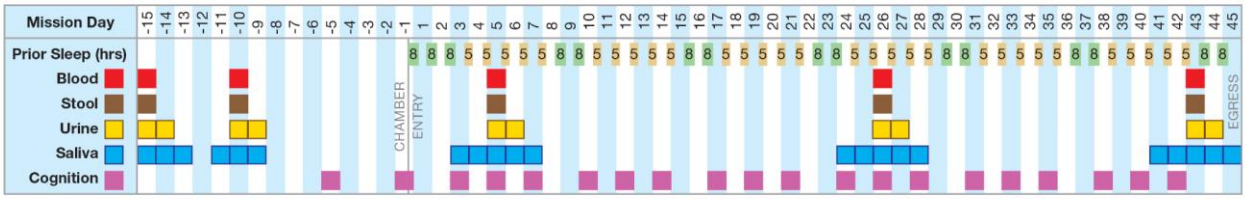

Key: Blood: Single blood collection used for immune and nutritional biomarker and metabolite analysis (41.7 ml total: 21 serum, 11 EDTA, 2.7 LiHep for nutritional analysis; 4.0 Heparin, 3.0 EDTA for immune). The nutritional EDTA and heparin tubes were placed on ice immediately after they are drawn, the remaining nutritional and immune tubes were kept at room temperature prior to analysis. Saliva: Single liquid saliva collection pre-breakfast, used for immune biomarker analysis; Stool: Single fecal collection, on day of blood draw or first collection after blood draw, used for microbiome taxonomic and metatranscriptomic profile and nutritional metabolite analysis. Urine: Collection of all urine over 48 hours, used for nutritional biomarker and metabolite analysis. All samples were nominally collected within a 24-hour timeframe on each selected mission day; urine collection proceeded for 48 hours, starting with the first void on the first day and ending with the first void on the third day. Cognition: administered after 12:00 pm; exact administration time was held constant within-subject throughout mission. Mission Day 3-7 corresponds to early, 24-28 corresponds to mid, and 41-45 corresponds to late time in mission for all figures and tables.

Table S2: Summary of blood testing, including sample type, timing of analyses, and method.

Test Name	Sample type	Timing	Method
1,25-Dihydroxy vitamin D	Serum	EOS*	Quantitative Chemiluminescent Immunoassay
A, B, D, E, K Vitamers	Serum	EOS	LC-MS/MS
Aldosterone	Serum	EOS	RIA
Alkyloid, Nicotine, Pteridine, Trp Metabolites	Serum	EOS	LC-MS/MS
Angiotensin II	Plasma	EOS	RIA
Atrial Natriuretic Peptide (ANP)	Plasma	EOS	RIA
Bone Specific Alkaline Phosphatase	Serum	EOS	ELISA
Calcium, Ionized	Heparin WB	EOS	CG*+ cartridge
Calcium, Serum	Serum	EOS	Atomic Absorption Spectroscopy
Carboxylic Acid	Serum	EOS	GC-MS/MS
CBC	EDTA WB	RT**	Sysmex XN-1000 analyzer
Ceruloplasmin	Serum	EOS	Nephelometry
Charged Amino Acids	Serum	EOS	GC-MS/MS
Comprehensive Metabolic Panel	Serum	EOS	Colorimetric and Potentiometric Ortho Vitros 5600
Copper	Serum	EOS	ICP-MS
Cortisol	Serum	EOS	LC-MS/MS
C-terminal telopeptides of type I collagen (CTX-1)	Serum	EOS	ELISA
Dehydroepiandrosterone (DHEA)	Serum	EOS	ELISA
DHEA-sulfate (DHEA-S)	Serum	EOS	ELISA
Essential Amino Acid	Serum	EOS	GC-MS/MS
Essential BCAA	Serum	EOS	LC-MS/MS
Flavonoids	Plasma	EOS	LC-MS/MS
Folate, RBC	EDTA WB	EOS	RIA
Folate, Serum	Serum	EOS	RIA
Glutathione	EDTA WB	RT	HPLC
Glutathione Peroxidase	Heparin WB	RT	Enzymatic
Glycation Products	Serum	EOS	GC-MS/MS
Heme	Plasma	EOS	Colorimetric
Hepcidin-25	Serum	EOS	ELISA
High Sensitivity C-Reactive Protein	Serum	EOS	Nephelometry
Hydrophobic Amino Acids	Serum	EOS	GC-MS/MS
Insulin	Serum	EOS	RIA
Insulin-like Growth Factor-1	Serum	EOS	RIA
Iodine	Serum	EOS	ICP-MS
Iron	Serum	EOS	ICP-MS
Keto Acids and Keto Bodies	Serum	EOS	GC-MS/MS
Leptin	Serum	EOS	RIA
Lys Metabolites	Serum	EOS	GC-MS/MS
Methylated Metabolites	Serum	EOS	GC-MS/MS
Osteocalcin	Serum	EOS	RIA
Osteoprotegerin (OPG)	Serum	EOS	ELISA
Oxidized Low Density Lipoproteins	Serum	EOS	ELISA
Parathyroid hormone	Serum	EOS	RIA
Polar Metabolites	Serum	EOS	GC-MS/MS
Procollagen type I N-terminal propeptide (P1NP)	Serum	EOS	RIA
Renin	Plasma	EOS	RIA
Retinol Binding Protein (RBP)	Serum	EOS	Nephelometry
Sclerostin	Serum	EOS	ELISA

Selenium	Serum	EOS	ICP-MS
RANKL	Serum	EOS	ELISA
Superoxide Dismutase (SOD)	Heparin WB	RT	Enzymatic
Testosterone, Bioavailable	Serum	EOS	LC-MS/MS
Testosterone, Free	Serum	EOS	LC-MS/MS
Testosterone, Sex hormone-binding globulin bound	Serum	EOS	LC-MS/MS
Testosterone, Total	Serum	EOS	LC-MS/MS
Thio Ether	Serum	EOS	GC-MS/MS
Throid Panel	Serum	EOS	Electrochemiluminescence Immunoassay
Total Antioxidant Capacity	Serum	EOS	Spectrophotometric Ortho Vitros 5600 Analyzer
Total Lipid Peroxides	Plasma	EOS	Colorimetry
Transferrin	Serum	EOS	Nephelometry
Transferrin Receptors	Serum	EOS	ELISA
Transthyretin	Serum	EOS	Nephelometry
Undercarboxylated Osteocalcin	Serum	EOS	RIA
Valine Metabolites	Serum	EOS	GC-MS/MS
Vitamin B12	Serum	EOS	RIA
Vitamin C	Plasma	EOS	HPLC
Zinc	Serum	EOS	ICP-MS

*EOS = End of study: samples kept frozen at -80°C until analysis after all campaigns were completed.
**RT = Real time: samples analyzed either at the time of collection or soon after based on known storage limitations for specific analyses.

Table S3: Urine analytical tests, sample types, timing of analysis, and general method.

Test Name	Sample type	Timing	Method
3-methylhistidine	Urine	EOS*	Ion-Exchange Chromatography
8-hydroxy-2'-deoxyguanosine	Urine	EOS	LC-MSM/MS
Renal Stone Profile	Urine	EOS	Ion Chromatography/Calculation
Calcium	Urine	EOS	Atomic Absorption Spectroscopy
Chemistry Panel	Urine	EOS	Colorimetric and Potentiometric Ortho Vitros 5600 Analyzer
Citrate	Urine	EOS	Ion Chromatography
Copper	Urine	EOS	ICP-MS
Cortisol (free)	Urine	EOS	LC-MS/MS
C-terminal telopeptides of Type I collagen (CTX-1)	Urine	EOS	ELISA
Deoxypyridinoline (DPD)	Urine	EOS	ELISA
Flavonoids	Urine	EOS	LC-MS/MS
γ-carboxyglutamatic acid	Urine	EOS	HPLC
Iodine	Urine	EOS	ICP-MS
Nitrogen	Urine	EOS	Pyrochemiluminescence
N-telopeptide (NTX)	Urine	EOS	ELISA
Oxalate	Urine	EOS	Ion Chromatography
Pyridinium Crosslinks	Urine	EOS	ELISA
PGF2-α (15-isoprostane F2t)	Urine	EOS	ELISA
pH	Urine	RT**	pH Probe
Selenium	Urine	EOS	ICP-MS
Specific gravity	Urine	EOS	Refractometry
Sulfate	Urine	EOS	Ion Chromatography
Testosterone	Urine	EOS	LC-MS/MS
Zinc	Urine	EOS	ICP-MS

*EOS = End of study: samples kept frozen at -80°C until analysis after all campaigns were completed.
**RT = Real time: samples analyzed either at the time of collection or soon after based on known storage limitations for specific analyses.

Table S4: Average Individual Intake of Nutritional Servings

Measurement	Units	Menu	Time	Estimate	StdErr	DF	Nsubjects	Nobs
Fish	oz per day	Standard	pre	0.790591947	0.504961366	950	16	972
Fish	oz per day	Standard	early	0.747335938	0.16891161	950	16	972
Fish	oz per day	Standard	mid	0.834583127	0.144588234	950	16	972
Fish	oz per day	Standard	late	0.778333333	0.155381207	950	16	972
Fish	oz per day	Enhanced	pre	1.171251351	0.332687163	950	16	972
Fish	oz per day	Enhanced	early	1.517351563	0.139324977	950	16	972
Fish	oz per day	Enhanced	mid	1.528166667	0.177307867	950	16	972
Fish	oz per day	Enhanced	late	1.628166667	0.228337644	950	16	972
Flavanoid	food items per day	Standard	early	4.325	0.222809554	700	16	720
Flavanoid	food items per day	Standard	mid	4.45	0.315072743	700	16	720
Flavanoid	food items per day	Standard	late	4.108333333	0.311121962	700	16	720
Flavanoid	food items per day	Enhanced	early	6.6	0.207498327	700	16	720
Flavanoid	food items per day	Enhanced	mid	6.733333333	0.232139805	700	16	720
Flavanoid	food items per day	Enhanced	late	6.708333333	0.230921313	700	16	720
Fru_Veg	servings per day	Standard	pre	4.853261355	0.884478949	950	16	972
Fru_Veg	servings per day	Standard	early	4.114976563	0.238728691	950	16	972
Fru_Veg	servings per day	Standard	mid	4.103924783	0.31674481	950	16	972
Fru_Veg	servings per day	Standard	late	3.821675	0.325735069	950	16	972
Fru_Veg	servings per day	Enhanced	pre	6.629970273	1.484496523	950	16	972
Fru_Veg	servings per day	Enhanced	early	6.163046875	0.185416639	950	16	972
Fru_Veg	servings per day	Enhanced	mid	6.433341667	0.228544978	950	16	972
Fru_Veg	servings per day	Enhanced	late	6.372916667	0.223721553	950	16	972
Lycopene	food items per day	Standard	early	0.933333333	0.079930525	700	16	720
Lycopene	food items per day	Standard	mid	1.05	0.107851493	700	16	720
Lycopene	food items per day	Standard	late	0.825	0.088142483	700	16	720
Lycopene	food items per day	Enhanced	early	1.141666667	0.049212549	700	16	720
Lycopene	food items per day	Enhanced	mid	1.083333333	0.067443392	700	16	720
Lycopene	food items per day	Enhanced	late	1.133333333	0.085796918	700	16	720

Table S5: Average Daily Individual Nutrient Intake

Measurement	Units	Menu	Time	Estimate	StdErr	DF	Nsubjects	Nobs
Acesulfame Potassium	mg	Standard	pre	0.68975	0.645202046	938	16	960
Acesulfame Potassium	mg	Standard	early	0.019	0.011635076	938	16	960
Acesulfame Potassium	mg	Standard	mid	0.0095	0.008886436	938	16	960
Acesulfame Potassium	mg	Standard	late	-7.01181E-16	#N/A	938	16	960
Acesulfame Potassium	mg	Enhanced	pre	2.311716667	1.650623263	938	16	960
Acesulfame Potassium	mg	Enhanced	early	3.325	3.110252703	938	16	960
Acesulfame Potassium	mg	Enhanced	mid	-1.22125E-15	#N/A	#N/A	16	960
Acesulfame Potassium	mg	Enhanced	late	-1.22125E-15	#N/A	#N/A	16	960
Added Sugars by Available Carbohydrate	g	Standard	pre	69.13643333	12.22429604	938	16	960
Added Sugars by Available Carbohydrate	g	Standard	early	83.60414167	5.74241092	938	16	960
Added Sugars by Available Carbohydrate	g	Standard	mid	78.909975	5.940754305	938	16	960
Added Sugars by Available Carbohydrate	g	Standard	late	81.05421667	8.523704916	938	16	960
Added Sugars by Available Carbohydrate	g	Enhanced	pre	66.64244167	9.704192629	938	16	960
Added Sugars by Available Carbohydrate	g	Enhanced	early	63.52734167	3.190165127	938	16	960
Added Sugars by Available Carbohydrate	g	Enhanced	mid	68.69435	3.728187917	938	16	960
Added Sugars by Available Carbohydrate	g	Enhanced	late	68.22451667	4.554176679	938	16	960
Added Sugars by Total Sugars	g	Standard	pre	59.28423333	9.166934095	938	16	960
Added Sugars by Total Sugars	g	Standard	early	15.134975	2.149146813	938	16	960
Added Sugars by Total Sugars	g	Standard	mid	12.06085	1.69873204	938	16	960
Added Sugars by Total Sugars	g	Standard	late	12.49414167	2.082640973	938	16	960
Added Sugars by Total Sugars	g	Enhanced	pre	60.22755	8.877735372	938	16	960
Added Sugars by Total Sugars	g	Enhanced	early	21.36056667	1.493859825	938	16	960
Added Sugars by Total Sugars	g	Enhanced	mid	24.3548	1.374046273	938	16	960
Added Sugars by Total Sugars	g	Enhanced	late	26.019725	2.096140759	938	16	960
Alanine	g	Standard	pre	4.868591667	0.438343146	938	16	960
Alanine	g	Standard	early	5.616541667	0.311803382	938	16	960
Alanine	g	Standard	mid	5.377116667	0.366152252	938	16	960
Alanine	g	Standard	late	5.454291667	0.332486616	938	16	960
Alanine	g	Enhanced	pre	5.666825	0.431894062	938	16	960
Alanine	g	Enhanced	early	5.468666667	0.207235498	938	16	960
Alanine	g	Enhanced	mid	5.762358333	0.285836714	938	16	960
Alanine	g	Enhanced	late	5.748958333	0.305335489	938	16	960
Alcohol	g	Standard	pre	10.55375833	3.43524403	938	16	960
Alcohol	g	Standard	early	0.610233333	0.022554111	938	16	960
Alcohol	g	Standard	mid	0.491	0.032487072	938	16	960
Alcohol	g	Standard	late	0.505291667	0.02561421	938	16	960
Alcohol	g	Enhanced	pre	13.75171667	3.62221815	938	16	960
Alcohol	g	Enhanced	early	0.470641667	0.025700302	938	16	960
Alcohol	g	Enhanced	mid	0.395566667	0.034196262	938	16	960
Alcohol	g	Enhanced	late	0.453808333	0.052972221	938	16	960
Alpha Carotene provitamin A carotenoid	mcg	Standard	pre	235.4957583	80.40547255	938	16	960
Alpha Carotene provitamin A carotenoid	mcg	Standard	early	837.3361	69.13031379	938	16	960
Alpha Carotene provitamin A carotenoid	mcg	Standard	mid	952.392775	102.4281231	938	16	960
Alpha Carotene provitamin A carotenoid	mcg	Standard	late	1170.294275	143.4848885	938	16	960
Alpha Carotene provitamin A carotenoid	mcg	Enhanced	pre	317.4621667	118.771273	938	16	960
Alpha Carotene provitamin A carotenoid	mcg	Enhanced	early	1472.209567	37.92679692	938	16	960
Alpha Carotene provitamin A carotenoid	mcg	Enhanced	mid	1565.157167	67.26212447	938	16	960
Alpha Carotene provitamin A carotenoid	mcg	Enhanced	late	1574.273867	97.88153234	938	16	960
Animal Protein	g	Standard	pre	73.96006667	5.484181813	938	16	960
Animal Protein	g	Standard	early	72.0818	3.997414575	938	16	960
Animal Protein	g	Standard	mid	69.33523333	4.73088161	938	16	960
Animal Protein	g	Standard	late	71.10793333	3.982362959	938	16	960
Animal Protein	g	Enhanced	pre	83.82008333	7.177340463	938	16	960
Animal Protein	g	Enhanced	early	70.95750833	2.965681626	938	16	960
Animal Protein	g	Enhanced	mid	75.86024167	4.003070199	938	16	960
Animal Protein	g	Enhanced	late	75.87326667	4.542358591	938	16	960
Arginine	g	Standard	pre	5.642183333	0.475224209	938	16	960
Arginine	g	Standard	early	6.927491667	0.420249502	938	16	960
Arginine	g	Standard	mid	6.6296	0.473268128	938	16	960
Arginine	g	Standard	late	6.773375	0.447264006	938	16	960
Arginine	g	Enhanced	pre	6.46785	0.534404125	938	16	960
Arginine	g	Enhanced	early	6.794375	0.257044035	938	16	960
Arginine	g	Enhanced	mid	7.039816667	0.34966982	938	16	960
Arginine	g	Enhanced	late	7.057675	0.369702226	938	16	960
Ash	g	Standard	pre	23.50080833	2.15495206	938	16	960
Ash	g	Standard	early	18.84346667	0.969984533	938	16	960
Ash	g	Standard	mid	18.266275	1.223583277	938	16	960
Ash	g	Standard	late	18.52284167	1.249188131	938	16	960
Ash	g	Enhanced	pre	23.26684167	1.35815516	938	16	960
Ash	g	Enhanced	early	19.66809167	0.757078626	938	16	960

Ash	g	Enhanced	mid	20.345225	0.927749865	938	16	960
Ash	g	Enhanced	late	20.019175	1.026289855	938	16	960
Aspartame	mg	Standard	pre	37.66999167	29.21248582	938	16	960
Aspartame	mg	Standard	early	1.88	1.758578972	938	16	960
Aspartame	mg	Standard	mid	2.35	2.198223715	938	16	960
Aspartame	mg	Standard	late	2.35	2.198223715	938	16	960
Aspartame	mg	Enhanced	pre	5.01295	4.117755479	938	16	960
Aspartame	mg	Enhanced	early	0.14	0.130958009	938	16	960
Aspartame	mg	Enhanced	mid	5.66214E-15	#N/A	938	16	960
Aspartame	mg	Enhanced	late	0.708666667	0.662896967	938	16	960
Aspartic Acid	g	Standard	pre	9.003083333	0.771103015	938	16	960
Aspartic Acid	g	Standard	early	10.27109167	0.598271254	938	16	960
Aspartic Acid	g	Standard	mid	9.799441667	0.683681678	938	16	960
Aspartic Acid	g	Standard	late	10.02886667	0.646235661	938	16	960
Aspartic Acid	g	Enhanced	pre	10.23641667	0.769990436	938	16	960
Aspartic Acid	g	Enhanced	early	10.37774167	0.391584424	938	16	960
Aspartic Acid	g	Enhanced	mid	10.85441667	0.547053266	938	16	960
Aspartic Acid	g	Enhanced	late	10.83809167	0.57337034	938	16	960
Available Carbohydrate	g	Standard	pre	249.3820333	28.6346009	938	16	960
Available Carbohydrate	g	Standard	early	244.2697167	13.21661142	938	16	960
Available Carbohydrate	g	Standard	mid	238.543175	17.29822311	938	16	960
Available Carbohydrate	g	Standard	late	231.5825167	20.14081027	938	16	960
Available Carbohydrate	g	Enhanced	pre	249.09515	16.02835471	938	16	960
Available Carbohydrate	g	Enhanced	early	240.5759583	8.745934631	938	16	960
Available Carbohydrate	g	Enhanced	mid	253.24895	9.927964217	938	16	960
Available Carbohydrate	g	Enhanced	late	251.5686417	9.961892002	938	16	960
Beta Carotene provitamin A carotenoid	mcg	Standard	pre	3306.781508	752.4002765	938	16	960
Beta Carotene provitamin A carotenoid	mcg	Standard	early	4386.944508	218.0148356	938	16	960
Beta Carotene provitamin A carotenoid	mcg	Standard	mid	4673.632908	385.4848558	938	16	960
Beta Carotene provitamin A carotenoid	mcg	Standard	late	4830.07105	544.2549859	938	16	960
Beta Carotene provitamin A carotenoid	mcg	Enhanced	pre	3319.927442	745.3456613	938	16	960
Beta Carotene provitamin A carotenoid	mcg	Enhanced	early	6163.460317	171.2392301	938	16	960
Beta Carotene provitamin A carotenoid	mcg	Enhanced	mid	6428.263958	211.7700593	938	16	960
Beta Carotene provitamin A carotenoid	mcg	Enhanced	late	6434.258317	310.046867	938	16	960
Beta Carotene Equivalents derived from provitamin A carotenoids	mcg	Standard	pre	3473.119858	803.0811547	938	16	960
Beta Carotene Equivalents derived from provitamin A carotenoids	mcg	Standard	early	4926.402842	253.6481388	938	16	960
Beta Carotene Equivalents derived from provitamin A carotenoids	mcg	Standard	mid	5253.447433	446.866392	938	16	960
Beta Carotene Equivalents derived from provitamin A carotenoids	mcg	Standard	late	5499.60395	623.5026633	938	16	960
Beta Carotene Equivalents derived from provitamin A carotenoids	mcg	Enhanced	pre	3545.80175	798.8261799	938	16	960
Beta Carotene Equivalents derived from provitamin A carotenoids	mcg	Enhanced	early	7097.711292	176.7163307	938	16	960
Beta Carotene Equivalents derived from provitamin A carotenoids	mcg	Enhanced	mid	7408.324083	238.9976027	938	16	960
Beta Carotene Equivalents derived from provitamin A carotenoids	mcg	Enhanced	late	7427.875483	357.1866556	938	16	960
Beta Cryptoxanthin provitamin A carotenoid	mcg	Standard	pre	97.1808	34.32747871	938	16	960
Beta Cryptoxanthin provitamin A carotenoid	mcg	Standard	early	234.854425	16.05213445	938	16	960
Beta Cryptoxanthin provitamin A carotenoid	mcg	Standard	mid	195.091	22.50557197	938	16	960
Beta Cryptoxanthin provitamin A carotenoid	mcg	Standard	late	183.6522917	23.19046375	938	16	960
Beta Cryptoxanthin provitamin A carotenoid	mcg	Enhanced	pre	134.28665	61.86729537	938	16	960
Beta Cryptoxanthin provitamin A carotenoid	mcg	Enhanced	early	387.3996417	9.805643934	938	16	960
Beta Cryptoxanthin provitamin A carotenoid	mcg	Enhanced	mid	386.5109833	9.751231941	938	16	960
Beta Cryptoxanthin provitamin A carotenoid	mcg	Enhanced	late	399.0202667	13.64305631	938	16	960
Beta Tocopherol	mg	Standard	pre	0.62835	0.0836574	938	16	960
Beta Tocopherol	mg	Standard	early	0.600533333	0.048856182	938	16	960
Beta Tocopherol	mg	Standard	mid	0.578208333	0.045942584	938	16	960
Beta Tocopherol	mg	Standard	late	0.583933333	0.054175854	938	16	960
Beta Tocopherol	mg	Enhanced	pre	0.802841667	0.149466913	938	16	960
Beta Tocopherol	mg	Enhanced	early	0.59645	0.044730458	938	16	960
Beta Tocopherol	mg	Enhanced	mid	0.596383333	0.062352011	938	16	960
Beta Tocopherol	mg	Enhanced	late	0.678175	0.079410585	938	16	960
Betaine	mg	Standard	pre	193.347825	35.75922919	938	16	960
Betaine	mg	Standard	early	156.4691167	5.549385349	938	16	960
Betaine	mg	Standard	mid	150.0505417	10.47004542	938	16	960
Betaine	mg	Standard	late	154.2662083	10.23973357	938	16	960
Betaine	mg	Enhanced	pre	219.7214083	45.12598348	938	16	960
Betaine	mg	Enhanced	early	209.6289583	9.815105115	938	16	960
Betaine	mg	Enhanced	mid	221.8448917	10.73135025	938	16	960
Betaine	mg	Enhanced	late	201.6153583	10.27599122	938	16	960
Biochanin A	mg	Standard	pre	0.337525	0.168693188	938	16	960
Biochanin A	mg	Standard	early	0.0518	0.003953339	938	16	960
Biochanin A	mg	Standard	mid	0.039558333	0.005660719	938	16	960
Biochanin A	mg	Standard	late	0.082641667	0.014574511	938	16	960
Biochanin A	mg	Enhanced	pre	0.0301	0.020116425	938	16	960
Biochanin A	mg	Enhanced	early	0.029866667	0.004289231	938	16	960

Biochanin A	mg	Enhanced	mid	0.036766667	0.005337459	938	16	960
Biochanin A	mg	Enhanced	late	0.031791667	0.004058281	938	16	960
CLA cis 9 trans 11	g	Standard	pre	0.147416667	0.016415358	938	16	960
CLA cis 9 trans 11	g	Standard	early	0.040875	0.002129959	938	16	960
CLA cis 9 trans 11	g	Standard	mid	0.0441	0.002164999	938	16	960
CLA cis 9 trans 11	g	Standard	late	0.0423	0.002754365	938	16	960
CLA cis 9 trans 11	g	Enhanced	pre	0.17575	0.030475919	938	16	960
CLA cis 9 trans 11	g	Enhanced	early	0.093541667	0.015663641	938	16	960
CLA cis 9 trans 11	g	Enhanced	mid	0.098991667	0.014394942	938	16	960
CLA cis 9 trans 11	g	Enhanced	late	0.125316667	0.018291861	938	16	960
CLA trans 10 cis 12	g	Standard	pre	0.028225	0.003325803	938	16	960
CLA trans 10 cis 12	g	Standard	early	0.007833333	0.000317761	938	16	960
CLA trans 10 cis 12	g	Standard	mid	0.007941667	0.000403533	938	16	960
CLA trans 10 cis 12	g	Standard	late	0.007816667	0.000500659	938	16	960
CLA trans 10 cis 12	g	Enhanced	pre	0.040625	0.00749307	938	16	960
CLA trans 10 cis 12	g	Enhanced	early	0.005983333	0.000336186	938	16	960
CLA trans 10 cis 12	g	Enhanced	mid	0.005458333	0.000392895	938	16	960
CLA trans 10 cis 12	g	Enhanced	late	0.006158333	0.000575656	938	16	960
Caffeine	mg	Standard	pre	154.4525833	27.59813823	938	16	960
Caffeine	mg	Standard	early	15.03495833	4.769036691	938	16	960
Caffeine	mg	Standard	mid	13.35456667	5.793961712	938	16	960
Caffeine	mg	Standard	late	16.91093333	7.085920592	938	16	960
Caffeine	mg	Enhanced	pre	155.234875	45.9134958	938	16	960
Caffeine	mg	Enhanced	early	35.26693333	12.1677158	938	16	960
Caffeine	mg	Enhanced	mid	44.76415833	15.01911314	938	16	960
Caffeine	mg	Enhanced	late	63.10969167	21.58515688	938	16	960
Calcium	mg	Standard	pre	1212.179417	97.29414266	938	16	960
Calcium	mg	Standard	early	933.2121833	48.29381804	938	16	960
Calcium	mg	Standard	mid	897.04265	53.5944254	938	16	960
Calcium	mg	Standard	late	922.4272583	58.14493206	938	16	960
Calcium	mg	Enhanced	pre	1027.40665	98.12034934	938	16	960
Calcium	mg	Enhanced	early	1106.750067	53.62670576	938	16	960
Calcium	mg	Enhanced	mid	1118.278742	56.63084996	938	16	960
Calcium	mg	Enhanced	late	1109.964508	69.49992789	938	16	960
Cholesterol	mg	Standard	pre	615.6996583	43.02554363	938	16	960
Cholesterol	mg	Standard	early	343.667025	24.91660803	938	16	960
Cholesterol	mg	Standard	mid	354.6127417	22.70534184	938	16	960
Cholesterol	mg	Standard	late	348.42815	21.00345845	938	16	960
Cholesterol	mg	Enhanced	pre	491.8274167	71.03867466	938	16	960
Cholesterol	mg	Enhanced	early	335.4300667	18.07292536	938	16	960
Cholesterol	mg	Enhanced	mid	351.6223417	18.25153433	938	16	960
Cholesterol	mg	Enhanced	late	358.006725	26.61668555	938	16	960
Cholesterol to Saturated Fatty Acid	Index	Standard	pre	70.63479167	3.002391111	938	16	960
Cholesterol to Saturated Fatty Acid	Index	Standard	early	43.8952	2.723252303	938	16	960
Cholesterol to Saturated Fatty Acid	Index	Standard	mid	43.96761667	2.778686896	938	16	960
Cholesterol to Saturated Fatty Acid	Index	Standard	late	43.67468333	2.653930859	938	16	960
Cholesterol to Saturated Fatty Acid	Index	Enhanced	pre	66.96083333	8.380271467	938	16	960
Cholesterol to Saturated Fatty Acid	Index	Enhanced	early	41.45413333	1.935600941	938	16	960
Cholesterol to Saturated Fatty Acid	Index	Enhanced	mid	42.42893333	2.152687177	938	16	960
Cholesterol to Saturated Fatty Acid	Index	Enhanced	late	42.97095833	2.726988477	938	16	960
Choline	mg	Standard	pre	611.830175	20.13241601	938	16	960
Choline	mg	Standard	early	406.42575	22.89266665	938	16	960
Choline	mg	Standard	mid	406.2131167	24.58813261	938	16	960
Choline	mg	Standard	late	405.1190667	24.33055911	938	16	960
Choline	mg	Enhanced	pre	545.0714167	60.59632457	938	16	960
Choline	mg	Enhanced	early	425.8289583	16.43993774	938	16	960
Choline	mg	Enhanced	mid	440.6416833	19.29869599	938	16	960
Choline	mg	Enhanced	late	439.2265583	23.77334998	938	16	960
Copper	mg	Standard	pre	1.643525	0.228672218	938	16	960
Copper	mg	Standard	early	1.963958333	0.124408844	938	16	960
Copper	mg	Standard	mid	2.014175	0.138689282	938	16	960
Copper	mg	Standard	late	2.019833333	0.145033588	938	16	960
Copper	mg	Enhanced	pre	1.693608333	0.158755796	938	16	960
Copper	mg	Enhanced	early	2.057066667	0.078244063	938	16	960
Copper	mg	Enhanced	mid	2.053483333	0.094375679	938	16	960
Copper	mg	Enhanced	late	2.048233333	0.103011977	938	16	960
Coumestrol	mg	Standard	pre	0.118225	0.02556329	938	16	960
Coumestrol	mg	Standard	early	0.00325	0.000740097	938	16	960
Coumestrol	mg	Standard	mid	0.001333333	0.000471405	938	16	960
Coumestrol	mg	Standard	late	0.00175	0.000633964	938	16	960
Coumestrol	mg	Enhanced	pre	0.119425	0.037564548	938	16	960
Coumestrol	mg	Enhanced	early	0.007583333	0.000570666	938	16	960

Coumestrol	mg	Enhanced	mid	0.008033333	0.000166249	938	16	960
Coumestrol	mg	Enhanced	late	0.007208333	0.00045299	938	16	960
Cystine	g	Standard	pre	1.434	0.088593845	938	16	960
Cystine	g	Standard	early	1.4724	0.079967693	938	16	960
Cystine	g	Standard	mid	1.4098	0.092597407	938	16	960
Cystine	g	Standard	late	1.420191667	0.087612973	938	16	960
Cystine	g	Enhanced	pre	1.499258333	0.132184824	938	16	960
Cystine	g	Enhanced	early	1.43245	0.0608793	938	16	960
Cystine	g	Enhanced	mid	1.507425	0.079345253	938	16	960
Cystine	g	Enhanced	late	1.498391667	0.082942105	938	16	960
Daidzein	mg	Standard	pre	1.354116667	0.543000553	938	16	960
Daidzein	mg	Standard	early	1.005141667	0.098100744	938	16	960
Daidzein	mg	Standard	mid	0.944533333	0.068073783	938	16	960
Daidzein	mg	Standard	late	0.738375	0.075106646	938	16	960
Daidzein	mg	Enhanced	pre	0.989125	0.251149832	938	16	960
Daidzein	mg	Enhanced	early	1.753641667	0.119769178	938	16	960
Daidzein	mg	Enhanced	mid	1.668075	0.105970267	938	16	960
Daidzein	mg	Enhanced	late	1.358675	0.112956864	938	16	960
Delta Tocopherol	mg	Standard	pre	5.776666667	0.933198405	938	16	960
Delta Tocopherol	mg	Standard	early	0.8462	0.074715622	938	16	960
Delta Tocopherol	mg	Standard	mid	0.9181	0.063586358	938	16	960
Delta Tocopherol	mg	Standard	late	0.807983333	0.082702642	938	16	960
Delta Tocopherol	mg	Enhanced	pre	3.289041667	0.335443598	938	16	960
Delta Tocopherol	mg	Enhanced	early	0.8842	0.043839379	938	16	960
Delta Tocopherol	mg	Enhanced	mid	0.8566	0.0490039	938	16	960
Delta Tocopherol	mg	Enhanced	late	0.831316667	0.057376367	938	16	960
Dietary Folate Equivalents	mcg	Standard	pre	527.533375	65.32477412	938	16	960
Dietary Folate Equivalents	mcg	Standard	early	595.4566333	22.79793906	938	16	960
Dietary Folate Equivalents	mcg	Standard	mid	544.5582667	36.96245834	938	16	960
Dietary Folate Equivalents	mcg	Standard	late	579.3221833	38.31971215	938	16	960
Dietary Folate Equivalents	mcg	Enhanced	pre	562.0670833	57.9460425	938	16	960
Dietary Folate Equivalents	mcg	Enhanced	early	590.38115	20.54396253	938	16	960
Dietary Folate Equivalents	mcg	Enhanced	mid	621.8809333	21.40624049	938	16	960
Dietary Folate Equivalents	mcg	Enhanced	late	622.0275667	22.31936363	938	16	960
Energy	kcal	Standard	pre	2630.746625	244.7114238	938	16	960
Energy	kcal	Standard	early	2312.074392	126.7161728	938	16	960
Energy	kcal	Standard	mid	2244.577667	149.6840433	938	16	960
Energy	kcal	Standard	late	2217.279983	162.6960603	938	16	960
Energy	kcal	Enhanced	pre	2705.001258	192.7847186	938	16	960
Energy	kcal	Enhanced	early	2277.686992	85.35625986	938	16	960
Energy	kcal	Enhanced	mid	2360.112858	103.9581113	938	16	960
Energy	kcal	Enhanced	late	2353.736933	108.5188371	938	16	960
Energy	kj	Standard	pre	11007.04147	1023.872241	938	16	960
Energy	kj	Standard	early	9673.716742	530.1798505	938	16	960
Energy	kj	Standard	mid	9391.310758	626.2774224	938	16	960
Energy	kj	Standard	late	9277.097233	680.7197982	938	16	960
Energy	kj	Enhanced	pre	11317.72196	806.6108834	938	16	960
Energy	kj	Enhanced	early	9529.840817	357.130232	938	16	960
Energy	kj	Enhanced	mid	9874.710017	434.9603387	938	16	960
Energy	kj	Enhanced	late	9848.03355	454.0424407	938	16	960
Erythritol	g	Standard	pre	0.000241667	9.3495E-05	938	16	960
Erythritol	g	Standard	early	-1.84314E-18	#N/A	938	16	960
Erythritol	g	Standard	mid	-1.84314E-18	#N/A	#N/A	16	960
Erythritol	g	Standard	late	-4.5672E-18	#N/A	#N/A	16	960
Erythritol	g	Enhanced	pre	0.08465	0.069317324	938	16	960
Erythritol	g	Enhanced	early	5.83333E-05	1.41299E-05	938	16	960
Erythritol	g	Enhanced	mid	9.16667E-05	2.61871E-05	938	16	960
Erythritol	g	Enhanced	late	5E-05	1.55902E-05	938	16	960
Formononetin	mg	Standard	pre	0.009341667	0.00605043	938	16	960
Formononetin	mg	Standard	early	0.000966667	0.000178341	938	16	960
Formononetin	mg	Standard	mid	0.000916667	0.000121192	938	16	960
Formononetin	mg	Standard	late	0.001641667	0.000289141	938	16	960
Formononetin	mg	Enhanced	pre	0.006508333	0.004106904	938	16	960
Formononetin	mg	Enhanced	early	0.001366667	0.000129636	938	16	960
Formononetin	mg	Enhanced	mid	0.001383333	0.00013731	938	16	960
Formononetin	mg	Enhanced	late	0.001425	0.000198584	938	16	960
Fructose	g	Standard	pre	18.555025	3.939020869	938	16	960
Fructose	g	Standard	early	14.91556667	0.888233909	938	16	960
Fructose	g	Standard	mid	15.15121667	1.395525397	938	16	960
Fructose	g	Standard	late	14.44944167	1.129422425	938	16	960
Fructose	g	Enhanced	pre	23.83100833	4.193264266	938	16	960
Fructose	g	Enhanced	early	24.364	1.01313379	938	16	960

Fructose	g	Enhanced	mid	24.51243333	0.825936138	938	16	960
Fructose	g	Enhanced	late	25.23894167	1.248634392	938	16	960
Galactose	g	Standard	pre	0.475725	0.096446682	938	16	960
Galactose	g	Standard	early	0.214783333	0.014447591	938	16	960
Galactose	g	Standard	mid	0.199533333	0.021246921	938	16	960
Galactose	g	Standard	late	0.217791667	0.020536605	938	16	960
Galactose	g	Enhanced	pre	0.283225	0.096409068	938	16	960
Galactose	g	Enhanced	early	0.230258333	0.010052007	938	16	960
Galactose	g	Enhanced	mid	0.222983333	0.008439505	938	16	960
Galactose	g	Enhanced	late	0.221458333	0.011233651	938	16	960
Gamma Tocopherol	mg	Standard	pre	22.69725833	2.993864894	938	16	960
Gamma Tocopherol	mg	Standard	early	9.864675	0.712225544	938	16	960
Gamma Tocopherol	mg	Standard	mid	10.344825	0.735061837	938	16	960
Gamma Tocopherol	mg	Standard	late	9.742308333	0.889386154	938	16	960
Gamma Tocopherol	mg	Enhanced	pre	15.69215833	1.524117078	938	16	960
Gamma Tocopherol	mg	Enhanced	early	9.589908333	0.411641819	938	16	960
Gamma Tocopherol	mg	Enhanced	mid	10.186725	0.574923186	938	16	960
Gamma Tocopherol	mg	Enhanced	late	9.781491667	0.484277422	938	16	960
Genistein	mg	Standard	pre	1.690883333	0.681960364	938	16	960
Genistein	mg	Standard	early	1.50495	0.149589278	938	16	960
Genistein	mg	Standard	mid	1.297458333	0.088624067	938	16	960
Genistein	mg	Standard	late	0.9456	0.088092306	938	16	960
Genistein	mg	Enhanced	pre	1.299708333	0.37422914	938	16	960
Genistein	mg	Enhanced	early	2.750033333	0.19104474	938	16	960
Genistein	mg	Enhanced	mid	2.601983333	0.172157707	938	16	960
Genistein	mg	Enhanced	late	2.120825	0.179420625	938	16	960
Glucose	g	Standard	pre	19.14514167	3.987912403	938	16	960
Glucose	g	Standard	early	16.04418333	1.026156197	938	16	960
Glucose	g	Standard	mid	15.85271667	1.4673892	938	16	960
Glucose	g	Standard	late	16.19989167	1.347252496	938	16	960
Glucose	g	Enhanced	pre	23.16320833	3.286035247	938	16	960
Glucose	g	Enhanced	early	25.421775	0.963318695	938	16	960
Glucose	g	Enhanced	mid	25.74448333	1.006245228	938	16	960
Glucose	g	Enhanced	late	26.89953333	1.199713442	938	16	960
Glutamic Acid	g	Standard	pre	19.63890833	2.05540876	938	16	960
Glutamic Acid	g	Standard	early	20.517475	1.111642384	938	16	960
Glutamic Acid	g	Standard	mid	19.49891667	1.30119611	938	16	960
Glutamic Acid	g	Standard	late	19.52676667	1.266751561	938	16	960
Glutamic Acid	g	Enhanced	pre	21.21604167	1.449682844	938	16	960
Glutamic Acid	g	Enhanced	early	20.06225833	0.788753669	938	16	960
Glutamic Acid	g	Enhanced	mid	21.08854167	1.06127335	938	16	960
Glutamic Acid	g	Enhanced	late	20.864	1.136028109	938	16	960
Glycemic Index bread	reference	Standard	pre	80.22330833	2.28227474	938	16	960
Glycemic Index bread	reference	Standard	early	6.179116667	0.284549941	938	16	960
Glycemic Index bread	reference	Standard	mid	4.350383333	0.201545491	938	16	960
Glycemic Index bread	reference	Standard	late	4.909683333	0.335481119	938	16	960
Glycemic Index bread	reference	Enhanced	pre	80.633925	1.559453958	938	16	960
Glycemic Index bread	reference	Enhanced	early	10.87926667	0.449366139	938	16	960
Glycemic Index bread	reference	Enhanced	mid	10.54140833	0.335763248	938	16	960
Glycemic Index bread	reference	Enhanced	late	10.71925	0.385226433	938	16	960
Glycemic Index glucose	reference	Standard	pre	56.12555	1.598559736	938	16	960
Glycemic Index glucose	reference	Standard	early	4.321891667	0.198980881	938	16	960
Glycemic Index glucose	reference	Standard	mid	3.042916667	0.141007674	938	16	960
Glycemic Index glucose	reference	Standard	late	3.434033333	0.23461588	938	16	960
Glycemic Index glucose	reference	Enhanced	pre	56.41319167	1.09235663	938	16	960
Glycemic Index glucose	reference	Enhanced	early	7.609666667	0.314371046	938	16	960
Glycemic Index glucose	reference	Enhanced	mid	7.373275	0.234871442	938	16	960
Glycemic Index glucose	reference	Enhanced	late	7.497816667	0.269454841	938	16	960
Glycemic Load bread	reference	Standard	pre	202.850325	24.37574982	938	16	960
Glycemic Load bread	reference	Standard	early	15.85855	1.158412748	938	16	960
Glycemic Load bread	reference	Standard	mid	10.50405833	0.80737426	938	16	960
Glycemic Load bread	reference	Standard	late	11.45391667	1.232631639	938	16	960
Glycemic Load bread	reference	Enhanced	pre	200.8038333	13.01185796	938	16	960
Glycemic Load bread	reference	Enhanced	early	26.33105833	1.885058446	938	16	960
Glycemic Load bread	reference	Enhanced	mid	26.770875	1.8977153	938	16	960
Glycemic Load bread	reference	Enhanced	late	26.98559167	1.827064589	938	16	960
Glycemic Load glucose	reference	Standard	pre	141.9225833	17.05732	938	16	960
Glycemic Load glucose	reference	Standard	early	11.091875	0.81010656	938	16	960
Glycemic Load glucose	reference	Standard	mid	7.347116667	0.564716852	938	16	960
Glycemic Load glucose	reference	Standard	late	8.011425	0.862023639	938	16	960
Glycemic Load glucose	reference	Enhanced	pre	140.485675	9.104832454	938	16	960
Glycemic Load glucose	reference	Enhanced	early	18.41768333	1.318635457	938	16	960

Glycemic Load glucose	reference	Enhanced	mid	18.72529167	1.327407995	938	16	960
Glycemic Load glucose	reference	Enhanced	late	18.87575833	1.277945897	938	16	960
Glycine	g	Standard	pre	4.157341667	0.413421297	938	16	960
Glycine	g	Standard	early	4.990558333	0.284276251	938	16	960
Glycine	g	Standard	mid	4.77065	0.33619944	938	16	960
Glycine	g	Standard	late	4.831283333	0.302525404	938	16	960
Glycine	g	Enhanced	pre	4.964125	0.386357018	938	16	960
Glycine	g	Enhanced	early	4.705433333	0.173063483	938	16	960
Glycine	g	Enhanced	mid	4.905966667	0.244172329	938	16	960
Glycine	g	Enhanced	late	4.934666667	0.252862448	938	16	960
Glycitein	mg	Standard	pre	0.332266667	0.159334473	938	16	960
Glycitein	mg	Standard	early	0.278258333	0.028219985	938	16	960
Glycitein	mg	Standard	mid	0.284766667	0.022614775	938	16	960
Glycitein	mg	Standard	late	0.181566667	0.019251266	938	16	960
Glycitein	mg	Enhanced	pre	0.217041667	0.068991048	938	16	960
Glycitein	mg	Enhanced	early	0.466258333	0.019723834	938	16	960
Glycitein	mg	Enhanced	mid	0.473133333	0.018281423	938	16	960
Glycitein	mg	Enhanced	late	0.402933333	0.021908382	938	16	960
Histidine	g	Standard	pre	2.839875	0.256542913	938	16	960
Histidine	g	Standard	early	3.081325	0.166440275	938	16	960
Histidine	g	Standard	mid	2.953791667	0.19379857	938	16	960
Histidine	g	Standard	late	2.993983333	0.181500503	938	16	960
Histidine	g	Enhanced	pre	3.27845	0.258187439	938	16	960
Histidine	g	Enhanced	early	3.007941667	0.113103933	938	16	960
Histidine	g	Enhanced	mid	3.187891667	0.156992273	938	16	960
Histidine	g	Enhanced	late	3.151041667	0.172508493	938	16	960
Inositol	g	Standard	pre	0.119941667	0.03053063	938	16	960
Inositol	g	Standard	early	0.119391667	0.006292472	938	16	960
Inositol	g	Standard	mid	0.126725	0.011184553	938	16	960
Inositol	g	Standard	late	0.134433333	0.009900323	938	16	960
Inositol	g	Enhanced	pre	0.113633333	0.051093671	938	16	960
Inositol	g	Enhanced	early	0.354216667	0.018699163	938	16	960
Inositol	g	Enhanced	mid	0.343266667	0.016193311	938	16	960
Inositol	g	Enhanced	late	0.3778	0.02153195	938	16	960
Insoluble Dietary Fiber	g	Standard	pre	14.99213333	2.36348579	938	16	960
Insoluble Dietary Fiber	g	Standard	early	20.35775833	1.334584048	938	16	960
Insoluble Dietary Fiber	g	Standard	mid	19.68088333	1.534319378	938	16	960
Insoluble Dietary Fiber	g	Standard	late	20.739725	1.90173123	938	16	960
Insoluble Dietary Fiber	g	Enhanced	pre	16.824775	2.466688745	938	16	960
Insoluble Dietary Fiber	g	Enhanced	early	21.76588333	0.732654923	938	16	960
Insoluble Dietary Fiber	g	Enhanced	mid	21.81458333	0.859502991	938	16	960
Insoluble Dietary Fiber	g	Enhanced	late	21.96181667	0.909278031	938	16	960
Iron	mg	Standard	pre	16.54241667	1.697020133	938	16	960
Iron	mg	Standard	early	20.84755	1.03378091	938	16	960
Iron	mg	Standard	mid	19.93348333	1.28145797	938	16	960
Iron	mg	Standard	late	20.233725	1.45159957	938	16	960
Iron	mg	Enhanced	pre	16.68036667	1.357657119	938	16	960
Iron	mg	Enhanced	early	20.11579167	0.569488369	938	16	960
Iron	mg	Enhanced	mid	21.39589167	0.87003502	938	16	960
Iron	mg	Enhanced	late	20.698475	0.859090708	938	16	960
Isoleucine	g	Standard	pre	4.765825	0.35673463	938	16	960
Isoleucine	g	Standard	early	4.949016667	0.272117677	938	16	960
Isoleucine	g	Standard	mid	4.733616667	0.309374286	938	16	960
Isoleucine	g	Standard	late	4.837208333	0.293853703	938	16	960
Isoleucine	g	Enhanced	pre	5.168541667	0.411300394	938	16	960
Isoleucine	g	Enhanced	early	4.956775	0.200780814	938	16	960
Isoleucine	g	Enhanced	mid	5.238858333	0.261000412	938	16	960
Isoleucine	g	Enhanced	late	5.167475	0.290300007	938	16	960
Lactose	g	Standard	pre	14.83365	3.726588647	938	16	960
Lactose	g	Standard	early	9.343741667	0.376772099	938	16	960
Lactose	g	Standard	mid	8.377866667	0.500208495	938	16	960
Lactose	g	Standard	late	9.384108333	0.607342868	938	16	960
Lactose	g	Enhanced	pre	13.890175	2.456112868	938	16	960
Lactose	g	Enhanced	early	12.19321667	1.007755258	938	16	960
Lactose	g	Enhanced	mid	13.42206667	1.089564144	938	16	960
Lactose	g	Enhanced	late	12.53675833	1.187244815	938	16	960
Leucine	g	Standard	pre	8.170891667	0.66900245	938	16	960
Leucine	g	Standard	early	8.515325	0.466164793	938	16	960
Leucine	g	Standard	mid	8.126433333	0.534573262	938	16	960
Leucine	g	Standard	late	8.258433333	0.510622373	938	16	960
Leucine	g	Enhanced	pre	8.901608333	0.645556849	938	16	960
Leucine	g	Enhanced	early	8.442891667	0.34394008	938	16	960

Leucine	g	Enhanced	mid	8.906108333	0.451649331	938	16	960
Leucine	g	Enhanced	late	8.819058333	0.496507146	938	16	960
Lutein Zeaxanthin	mcg	Standard	pre	2829.6471	456.2582471	938	16	960
Lutein Zeaxanthin	mcg	Standard	early	2216.071625	111.5941363	938	16	960
Lutein Zeaxanthin	mcg	Standard	mid	2113.26625	210.2385402	938	16	960
Lutein Zeaxanthin	mcg	Standard	late	2442.250617	217.4347411	938	16	960
Lutein Zeaxanthin	mcg	Enhanced	pre	3400.036367	953.2496865	938	16	960
Lutein Zeaxanthin	mcg	Enhanced	early	4588.010367	222.8732424	938	16	960
Lutein Zeaxanthin	mcg	Enhanced	mid	4559.331808	131.5302669	938	16	960
Lutein Zeaxanthin	mcg	Enhanced	late	4463.416342	239.8550635	938	16	960
Lycopene	mcg	Standard	pre	5402.254433	1186.104516	938	16	960
Lycopene	mcg	Standard	early	6090.086725	403.5661496	938	16	960
Lycopene	mcg	Standard	mid	6658.366017	490.2593495	938	16	960
Lycopene	mcg	Standard	late	5223.541842	477.306725	938	16	960
Lycopene	mcg	Enhanced	pre	3387.566817	233.2157784	938	16	960
Lycopene	mcg	Enhanced	early	6872.319375	154.3090862	938	16	960
Lycopene	mcg	Enhanced	mid	6950.429092	339.4045438	938	16	960
Lycopene	mcg	Enhanced	late	6892.9152	397.3840504	938	16	960
Lysine	g	Standard	pre	6.937991667	0.60390663	938	16	960
Lysine	g	Standard	early	7.751416667	0.435045769	938	16	960
Lysine	g	Standard	mid	7.413	0.505202433	938	16	960
Lysine	g	Standard	late	7.585008333	0.457390031	938	16	960
Lysine	g	Enhanced	pre	7.937791667	0.615960283	938	16	960
Lysine	g	Enhanced	early	7.685066667	0.304383226	938	16	960
Lysine	g	Enhanced	mid	8.176583333	0.411197624	938	16	960
Lysine	g	Enhanced	late	8.078025	0.449234224	938	16	960
MUFA 14 1 myristoleic acid	g	Standard	pre	0.144966667	0.019103723	938	16	960
MUFA 14 1 myristoleic acid	g	Standard	early	0.068825	0.002909084	938	16	960
MUFA 14 1 myristoleic acid	g	Standard	mid	0.06175	0.003106886	938	16	960
MUFA 14 1 myristoleic acid	g	Standard	late	0.06635	0.003390361	938	16	960
MUFA 14 1 myristoleic acid	g	Enhanced	pre	0.185616667	0.032442853	938	16	960
MUFA 14 1 myristoleic acid	g	Enhanced	early	0.038425	0.001755109	938	16	960
MUFA 14 1 myristoleic acid	g	Enhanced	mid	0.037025	0.002079528	938	16	960
MUFA 14 1 myristoleic acid	g	Enhanced	late	0.043908333	0.003086123	938	16	960
MUFA 16 1 palmitoleic acid	g	Standard	pre	1.708866667	0.154463581	938	16	960
MUFA 16 1 palmitoleic acid	g	Standard	early	1.872441667	0.119195861	938	16	960
MUFA 16 1 palmitoleic acid	g	Standard	mid	1.324458333	0.085344576	938	16	960
MUFA 16 1 palmitoleic acid	g	Standard	late	1.539933333	0.107381696	938	16	960
MUFA 16 1 palmitoleic acid	g	Enhanced	pre	2.012866667	0.235167527	938	16	960
MUFA 16 1 palmitoleic acid	g	Enhanced	early	1.863183333	0.151698571	938	16	960
MUFA 16 1 palmitoleic acid	g	Enhanced	mid	1.816633333	0.11688473	938	16	960
MUFA 16 1 palmitoleic acid	g	Enhanced	late	1.92705	0.200914979	938	16	960
MUFA 18 1 oleic acid	g	Standard	pre	39.48659167	3.440497303	938	16	960
MUFA 18 1 oleic acid	g	Standard	early	27.599575	1.886015551	938	16	960
MUFA 18 1 oleic acid	g	Standard	mid	26.11136667	1.727491671	938	16	960
MUFA 18 1 oleic acid	g	Standard	late	26.12990833	1.805345959	938	16	960
MUFA 18 1 oleic acid	g	Enhanced	pre	42.23956667	4.49228791	938	16	960
MUFA 18 1 oleic acid	g	Enhanced	early	27.5495	1.356234605	938	16	960
MUFA 18 1 oleic acid	g	Enhanced	mid	27.43803333	1.704054176	938	16	960
MUFA 18 1 oleic acid	g	Enhanced	late	27.93378333	1.846083027	938	16	960
MUFA 20 1 gadoleic acid	g	Standard	pre	0.404616667	0.041301853	938	16	960
MUFA 20 1 gadoleic acid	g	Standard	early	0.360091667	0.028759508	938	16	960
MUFA 20 1 gadoleic acid	g	Standard	mid	0.27475	0.021884897	938	16	960
MUFA 20 1 gadoleic acid	g	Standard	late	0.306608333	0.026161245	938	16	960
MUFA 20 1 gadoleic acid	g	Enhanced	pre	0.538958333	0.077702743	938	16	960
MUFA 20 1 gadoleic acid	g	Enhanced	early	0.381283333	0.02773325	938	16	960
MUFA 20 1 gadoleic acid	g	Enhanced	mid	0.375933333	0.026594253	938	16	960
MUFA 20 1 gadoleic acid	g	Enhanced	late	0.384741667	0.035058977	938	16	960
MUFA 22 1 erucic acid	g	Standard	pre	0.048225	0.016639653	938	16	960
MUFA 22 1 erucic acid	g	Standard	early	0.056258333	0.005162625	938	16	960
MUFA 22 1 erucic acid	g	Standard	mid	0.048225	0.00424097	938	16	960
MUFA 22 1 erucic acid	g	Standard	late	0.053683333	0.003866932	938	16	960
MUFA 22 1 erucic acid	g	Enhanced	pre	0.049925	0.009434021	938	16	960
MUFA 22 1 erucic acid	g	Enhanced	early	0.116191667	0.00658997	938	16	960
MUFA 22 1 erucic acid	g	Enhanced	mid	0.10515	0.007320223	938	16	960
MUFA 22 1 erucic acid	g	Enhanced	late	0.10125	0.009313854	938	16	960
Magnesium	mg	Standard	pre	351.4103833	40.2537145	938	16	960
Magnesium	mg	Standard	early	436.7338833	27.03135473	938	16	960
Magnesium	mg	Standard	mid	430.2973083	30.76503816	938	16	960
Magnesium	mg	Standard	late	440.7293583	30.82833214	938	16	960
Magnesium	mg	Enhanced	pre	411.9353333	32.26249549	938	16	960
Magnesium	mg	Enhanced	early	484.1126	16.38062279	938	16	960

Magnesium	mg	Enhanced	mid	486.1005167	21.50699475	938	16	960
Magnesium	mg	Enhanced	late	481.6359583	23.98654894	938	16	960
Maltitol	g	Standard	pre	3.33333E-05	1.66667E-05	938	16	960
Maltitol	g	Standard	early	-6.39143E-21	#N/A	#N/A	16	960
Maltitol	g	Standard	mid	-6.39143E-21	#N/A	#N/A	16	960
Maltitol	g	Standard	late	-5.06884E-21	#N/A	#N/A	16	960
Maltitol	g	Enhanced	pre	4.16667E-05	2.33854E-05	938	16	960
Maltitol	g	Enhanced	early	-1.13511E-20	#N/A	#N/A	16	960
Maltitol	g	Enhanced	mid	-7.87935E-21	#N/A	938	16	960
Maltitol	g	Enhanced	late	-6.34282E-20	#N/A	938	16	960
Maltose	g	Standard	pre	3.198966667	0.422215637	938	16	960
Maltose	g	Standard	early	1.915566667	0.11007456	938	16	960
Maltose	g	Standard	mid	2.065916667	0.144080684	938	16	960
Maltose	g	Standard	late	1.794141667	0.176170965	938	16	960
Maltose	g	Enhanced	pre	2.176241667	0.215044461	938	16	960
Maltose	g	Enhanced	early	1.653741667	0.091639155	938	16	960
Maltose	g	Enhanced	mid	1.93735	0.109382914	938	16	960
Maltose	g	Enhanced	late	1.880433333	0.112398111	938	16	960
Manganese	mg	Standard	pre	4.229516667	0.541607812	938	16	960
Manganese	mg	Standard	early	5.509441667	0.304368909	938	16	960
Manganese	mg	Standard	mid	5.254183333	0.386845611	938	16	960
Manganese	mg	Standard	late	5.4382	0.382029369	938	16	960
Manganese	mg	Enhanced	pre	4.318866667	0.58214437	938	16	960
Manganese	mg	Enhanced	early	5.972291667	0.196980066	938	16	960
Manganese	mg	Enhanced	mid	5.948991667	0.230648293	938	16	960
Manganese	mg	Enhanced	late	5.993666667	0.20850408	938	16	960
Mannitol	g	Standard	pre	0.2682	0.067148825	938	16	960
Mannitol	g	Standard	early	0.552016667	0.025925097	938	16	960
Mannitol	g	Standard	mid	0.582741667	0.051806707	938	16	960
Mannitol	g	Standard	late	0.436708333	0.042767552	938	16	960
Mannitol	g	Enhanced	pre	0.367266667	0.128629213	938	16	960
Mannitol	g	Enhanced	early	0.626925	0.020808834	938	16	960
Mannitol	g	Enhanced	mid	0.635391667	0.019283342	938	16	960
Mannitol	g	Enhanced	late	0.643266667	0.02401459	938	16	960
Methionine	g	Standard	pre	2.433558333	0.179557418	938	16	960
Methionine	g	Standard	early	2.51585	0.139157657	938	16	960
Methionine	g	Standard	mid	2.413233333	0.161193165	938	16	960
Methionine	g	Standard	late	2.450691667	0.144000166	938	16	960
Methionine	g	Enhanced	pre	2.671508333	0.213055235	938	16	960
Methionine	g	Enhanced	early	2.473375	0.098857393	938	16	960
Methionine	g	Enhanced	mid	2.6153	0.133456926	938	16	960
Methionine	g	Enhanced	late	2.5993	0.145351057	938	16	960
Natural Alpha Tocopherol RRR alpha tocopherol or d alpha tocopherol	mg	Standard	pre	10.782425	1.102511198	938	16	960
Natural Alpha Tocopherol RRR alpha tocopherol or d alpha tocopherol	mg	Standard	early	10.30739167	0.761250595	938	16	960
Natural Alpha Tocopherol RRR alpha tocopherol or d alpha tocopherol	mg	Standard	mid	10.14296667	0.699739177	938	16	960
Natural Alpha Tocopherol RRR alpha tocopherol or d alpha tocopherol	mg	Standard	late	10.14659167	0.688106247	938	16	960
Natural Alpha Tocopherol RRR alpha tocopherol or d alpha tocopherol	mg	Enhanced	pre	11.54738333	1.505527375	938	16	960
Natural Alpha Tocopherol RRR alpha tocopherol or d alpha tocopherol	mg	Enhanced	early	12.54645833	0.438640722	938	16	960
Natural Alpha Tocopherol RRR alpha tocopherol or d alpha tocopherol	mg	Enhanced	mid	11.99735833	0.766287603	938	16	960
Natural Alpha Tocopherol RRR alpha tocopherol or d alpha tocopherol	mg	Enhanced	late	12.74508333	0.78051321	938	16	960
Natural Folate food folate	mcg	Standard	pre	292.2769333	31.07083104	938	16	960
Natural Folate food folate	mcg	Standard	early	265.9745167	17.31517912	938	16	960
Natural Folate food folate	mcg	Standard	mid	249.318675	21.79918605	938	16	960
Natural Folate food folate	mcg	Standard	late	270.3433583	24.74599101	938	16	960
Natural Folate food folate	mcg	Enhanced	pre	309.1367	36.23836004	938	16	960
Natural Folate food folate	mcg	Enhanced	early	357.381125	12.56516426	938	16	960
Natural Folate food folate	mcg	Enhanced	mid	364.3235667	12.85375434	938	16	960
Natural Folate food folate	mcg	Enhanced	late	363.691925	12.73659605	938	16	960
Niacin vitamin B3	mg	Standard	pre	29.79580682	3.220000868	938	16	960
Niacin vitamin B3	mg	Standard	early	32.4103065	1.81467401	938	16	960
Niacin vitamin B3	mg	Standard	mid	31.0720319	2.211265827	938	16	960
Niacin vitamin B3	mg	Standard	late	31.81548992	2.140633525	938	16	960
Niacin vitamin B3	mg	Enhanced	pre	33.50532341	2.235835789	938	16	960
Niacin vitamin B3	mg	Enhanced	early	29.97328975	1.155947135	938	16	960
Niacin vitamin B3	mg	Enhanced	mid	33.17192292	1.943273924	938	16	960
Niacin vitamin B3	mg	Enhanced	late	32.24106492	1.725218038	938	16	960
Niacin Equivalents	mg	Standard	pre	50.79745	4.480859447	938	16	960
Niacin Equivalents	mg	Standard	early	53.00878333	2.981752277	938	16	960
Niacin Equivalents	mg	Standard	mid	50.93058333	3.531317712	938	16	960
Niacin Equivalents	mg	Standard	late	52.02030833	3.416898221	938	16	960
Niacin Equivalents	mg	Enhanced	pre	56.56903333	3.863516945	938	16	960
Niacin Equivalents	mg	Enhanced	early	50.9289	2.036761204	938	16	960

Niacin Equivalents	mg	Enhanced	mid	55.38290833	3.107829681	938	16	960
Niacin Equivalents	mg	Enhanced	late	53.932675	2.939225669	938	16	960
Nitrogen	g	Standard	pre	17.02068333	1.420672471	938	16	960
Nitrogen	g	Standard	early	5.258375	0.288743918	938	16	960
Nitrogen	g	Standard	mid	4.554933333	0.340021711	938	16	960
Nitrogen	g	Standard	late	5.184233333	0.299457305	938	16	960
Nitrogen	g	Enhanced	pre	18.76120833	1.356979618	938	16	960
Nitrogen	g	Enhanced	early	6.006108333	0.34182509	938	16	960
Nitrogen	g	Enhanced	mid	5.885108333	0.326269603	938	16	960
Nitrogen	g	Enhanced	late	6.418608333	0.356724675	938	16	960
Omega 3 Fatty Acids	g	Standard	pre	2.672341667	0.330547724	938	16	960
Omega 3 Fatty Acids	g	Standard	early	0.278816667	0.029874863	938	16	960
Omega 3 Fatty Acids	g	Standard	mid	0.257791667	0.022608871	938	16	960
Omega 3 Fatty Acids	g	Standard	late	0.241458333	0.023743688	938	16	960
Omega 3 Fatty Acids	g	Enhanced	pre	2.618516667	0.298950648	938	16	960
Omega 3 Fatty Acids	g	Enhanced	early	1.024125	0.052605844	938	16	960
Omega 3 Fatty Acids	g	Enhanced	mid	0.989575	0.051145773	938	16	960
Omega 3 Fatty Acids	g	Enhanced	late	1.094075	0.060963258	938	16	960
Oxalic Acid	mg	Standard	pre	303.0090667	46.20816028	938	16	960
Oxalic Acid	mg	Standard	early	276.4513667	18.80270966	938	16	960
Oxalic Acid	mg	Standard	mid	269.3497167	19.61225161	938	16	960
Oxalic Acid	mg	Standard	late	282.665225	21.21715023	938	16	960
Oxalic Acid	mg	Enhanced	pre	350.6391417	60.94622094	938	16	960
Oxalic Acid	mg	Enhanced	early	356.09815	8.992860285	938	16	960
Oxalic Acid	mg	Enhanced	mid	333.6939167	15.97054409	938	16	960
Oxalic Acid	mg	Enhanced	late	326.1984083	15.7978223	938	16	960
PUFA 18 2 linoleic acid	g	Standard	pre	23.43044167	2.884430737	938	16	960
PUFA 18 2 linoleic acid	g	Standard	early	12.34349167	0.900172492	938	16	960
PUFA 18 2 linoleic acid	g	Standard	mid	12.40245833	0.841036204	938	16	960
PUFA 18 2 linoleic acid	g	Standard	late	12.11049167	0.928324672	938	16	960
PUFA 18 2 linoleic acid	g	Enhanced	pre	21.1052	1.746367626	938	16	960
PUFA 18 2 linoleic acid	g	Enhanced	early	14.27143333	0.593806279	938	16	960
PUFA 18 2 linoleic acid	g	Enhanced	mid	14.05466667	0.884114127	938	16	960
PUFA 18 2 linoleic acid	g	Enhanced	late	14.49225833	0.874278703	938	16	960
PUFA 18 3 linolenic acid	g	Standard	pre	2.497708333	0.329129916	938	16	960
PUFA 18 3 linolenic acid	g	Standard	early	1.057241667	0.062677156	938	16	960
PUFA 18 3 linolenic acid	g	Standard	mid	1.0862	0.056464298	938	16	960
PUFA 18 3 linolenic acid	g	Standard	late	1.005366667	0.067160681	938	16	960
PUFA 18 3 linolenic acid	g	Enhanced	pre	2.2964	0.177248778	938	16	960
PUFA 18 3 linolenic acid	g	Enhanced	early	1.681775	0.051810922	938	16	960
PUFA 18 3 linolenic acid	g	Enhanced	mid	1.639258333	0.058277701	938	16	960
PUFA 18 3 linolenic acid	g	Enhanced	late	1.681458333	0.073950262	938	16	960
PUFA 18 3 n 3 alpha linolenic acid ALA	g	Standard	pre	2.425833333	0.324141652	938	16	960
PUFA 18 3 n 3 alpha linolenic acid ALA	g	Standard	early	0.13075	0.011265796	938	16	960
PUFA 18 3 n 3 alpha linolenic acid ALA	g	Standard	mid	0.103758333	0.008983024	938	16	960
PUFA 18 3 n 3 alpha linolenic acid ALA	g	Standard	late	0.108008333	0.008550182	938	16	960
PUFA 18 3 n 3 alpha linolenic acid ALA	g	Enhanced	pre	2.234175	0.173351296	938	16	960
PUFA 18 3 n 3 alpha linolenic acid ALA	g	Enhanced	early	0.670891667	0.034390571	938	16	960
PUFA 18 3 n 3 alpha linolenic acid ALA	g	Enhanced	mid	0.634658333	0.020368935	938	16	960
PUFA 18 3 n 3 alpha linolenic acid ALA	g	Enhanced	late	0.703375	0.038651324	938	16	960
PUFA 18 4 parinaric acid	g	Standard	pre	0.010741667	0.007375414	938	16	960
PUFA 18 4 parinaric acid	g	Standard	early	0.002658333	0.000596336	938	16	960
PUFA 18 4 parinaric acid	g	Standard	mid	0.001458333	0.000288299	938	16	960
PUFA 18 4 parinaric acid	g	Standard	late	0.002083333	0.000415624	938	16	960
PUFA 18 4 parinaric acid	g	Enhanced	pre	0.013966667	0.008808235	938	16	960
PUFA 18 4 parinaric acid	g	Enhanced	early	0.017566667	0.001994367	938	16	960
PUFA 18 4 parinaric acid	g	Enhanced	mid	0.018408333	0.002572192	938	16	960
PUFA 18 4 parinaric acid	g	Enhanced	late	0.020166667	0.00270973	938	16	960
PUFA 20 4 arachidonic acid	g	Standard	pre	0.3271	0.021885739	938	16	960
PUFA 20 4 arachidonic acid	g	Standard	early	0.179358333	0.011097359	938	16	960
PUFA 20 4 arachidonic acid	g	Standard	mid	0.187941667	0.012579185	938	16	960
PUFA 20 4 arachidonic acid	g	Standard	late	0.189266667	0.009938736	938	16	960
PUFA 20 4 arachidonic acid	g	Enhanced	pre	0.312666667	0.083600709	938	16	960
PUFA 20 4 arachidonic acid	g	Enhanced	early	0.168141667	0.00883608	938	16	960
PUFA 20 4 arachidonic acid	g	Enhanced	mid	0.173425	0.009573835	938	16	960
PUFA 20 4 arachidonic acid	g	Enhanced	late	0.174641667	0.012189561	938	16	960
PUFA 20 5 eicosapentaenoic acid EPA	g	Standard	pre	0.052141667	0.030906585	938	16	960
PUFA 20 5 eicosapentaenoic acid EPA	g	Standard	early	0.0415	0.008411839	938	16	960
PUFA 20 5 eicosapentaenoic acid EPA	g	Standard	mid	0.05145	0.007675464	938	16	960
PUFA 20 5 eicosapentaenoic acid EPA	g	Standard	late	0.034475	0.007826588	938	16	960
PUFA 20 5 eicosapentaenoic acid EPA	g	Enhanced	pre	0.092116667	0.037513231	938	16	960
PUFA 20 5 eicosapentaenoic acid EPA	g	Enhanced	early	0.116383333	0.009118439	938	16	960

PUFA 20 5 eicosapentaenoic acid EPA	g	Enhanced	mid	0.11445	0.010473311	938	16	960
PUFA 20 5 eicosapentaenoic acid EPA	g	Enhanced	late	0.12545	0.01031476	938	16	960
PUFA 22 5 docosapentaenoic acid DPA	g	Standard	pre	0.042508333	0.016836612	938	16	960
PUFA 22 5 docosapentaenoic acid DPA	g	Standard	early	0.023016667	0.001848676	938	16	960
PUFA 22 5 docosapentaenoic acid DPA	g	Standard	mid	0.020766667	0.001381776	938	16	960
PUFA 22 5 docosapentaenoic acid DPA	g	Standard	late	0.021416667	0.001595251	938	16	960
PUFA 22 5 docosapentaenoic acid DPA	g	Enhanced	pre	0.0587	0.022561081	938	16	960
PUFA 22 5 docosapentaenoic acid DPA	g	Enhanced	early	0.034316667	0.002083658	938	16	960
PUFA 22 5 docosapentaenoic acid DPA	g	Enhanced	mid	0.035558333	0.002473777	938	16	960
PUFA 22 5 docosapentaenoic acid DPA	g	Enhanced	late	0.035341667	0.002336974	938	16	960
PUFA 22 6 docosahexaenoic acid DHA	g	Standard	pre	0.141075	0.067401665	938	16	960
PUFA 22 6 docosahexaenoic acid DHA	g	Standard	early	0.080841667	0.012029835	938	16	960
PUFA 22 6 docosahexaenoic acid DHA	g	Standard	mid	0.0804	0.008982266	938	16	960
PUFA 22 6 docosahexaenoic acid DHA	g	Standard	late	0.0755	0.010471668	938	16	960
PUFA 22 6 docosahexaenoic acid DHA	g	Enhanced	pre	0.219608333	0.095096115	938	16	960
PUFA 22 6 docosahexaenoic acid DHA	g	Enhanced	early	0.184975	0.011551196	938	16	960
PUFA 22 6 docosahexaenoic acid DHA	g	Enhanced	mid	0.186475	0.01825077	938	16	960
PUFA 22 6 docosahexaenoic acid DHA	g	Enhanced	late	0.209716667	0.019647939	938	16	960
Pantothenic Acid	mg	Standard	pre	8.543558333	1.040684074	938	16	960
Pantothenic Acid	mg	Standard	early	5.742575	0.239851362	938	16	960
Pantothenic Acid	mg	Standard	mid	5.47225	0.311707587	938	16	960
Pantothenic Acid	mg	Standard	late	5.6478	0.329966342	938	16	960
Pantothenic Acid	mg	Enhanced	pre	7.11545	0.542841756	938	16	960
Pantothenic Acid	mg	Enhanced	early	6.298575	0.221962117	938	16	960
Pantothenic Acid	mg	Enhanced	mid	6.556666667	0.270167922	938	16	960
Pantothenic Acid	mg	Enhanced	late	6.247925	0.316957469	938	16	960
Pectins	g	Standard	pre	2.520183333	0.454570241	938	16	960
Pectins	g	Standard	early	3.524866667	0.215859387	938	16	960
Pectins	g	Standard	mid	3.427208333	0.278041633	938	16	960
Pectins	g	Standard	late	3.425725	0.285701012	938	16	960
Pectins	g	Enhanced	pre	3.118991667	0.557762806	938	16	960
Pectins	g	Enhanced	early	4.86445	0.170413822	938	16	960
Pectins	g	Enhanced	mid	4.961175	0.209784613	938	16	960
Pectins	g	Enhanced	late	5.009916667	0.220180925	938	16	960
Phenylalanine	g	Standard	pre	4.66405	0.361987505	938	16	960
Phenylalanine	g	Standard	early	4.818591667	0.267699255	938	16	960
Phenylalanine	g	Standard	mid	4.584658333	0.300956673	938	16	960
Phenylalanine	g	Standard	late	4.668875	0.296781756	938	16	960
Phenylalanine	g	Enhanced	pre	4.962083333	0.381735881	938	16	960
Phenylalanine	g	Enhanced	early	4.725583333	0.186303273	938	16	960
Phenylalanine	g	Enhanced	mid	4.943308333	0.245282535	938	16	960
Phenylalanine	g	Enhanced	late	4.901441667	0.26951165	938	16	960
Phosphorus	mg	Standard	pre	1684.855933	146.1733934	938	16	960
Phosphorus	mg	Standard	early	1802.517208	98.47763673	938	16	960
Phosphorus	mg	Standard	mid	1754.093358	118.9508849	938	16	960
Phosphorus	mg	Standard	late	1782.817908	125.0887613	938	16	960
Phosphorus	mg	Enhanced	pre	1711.22335	140.3699947	938	16	960
Phosphorus	mg	Enhanced	early	1878.506658	69.85980948	938	16	960
Phosphorus	mg	Enhanced	mid	1938.287617	88.74518862	938	16	960
Phosphorus	mg	Enhanced	late	1905.600008	101.0730289	938	16	960
Phytic Acid	mg	Standard	pre	785.5085917	131.1180841	938	16	960
Phytic Acid	mg	Standard	early	1232.581342	90.66979564	938	16	960
Phytic Acid	mg	Standard	mid	1223.372383	94.64218399	938	16	960
Phytic Acid	mg	Standard	late	1204.877067	92.71050044	938	16	960
Phytic Acid	mg	Enhanced	pre	900.9268667	136.9897777	938	16	960
Phytic Acid	mg	Enhanced	early	1268.211617	48.62891838	938	16	960
Phytic Acid	mg	Enhanced	mid	1187.991842	67.49345848	938	16	960
Phytic Acid	mg	Enhanced	late	1236.804425	75.13847221	938	16	960
Pinitol	g	Standard	pre	0.0257	0.009125871	938	16	960
Pinitol	g	Standard	early	0.003758333	0.000304352	938	16	960
Pinitol	g	Standard	mid	0.004966667	0.000639661	938	16	960
Pinitol	g	Standard	late	0.003525	0.000426295	938	16	960
Pinitol	g	Enhanced	pre	0.011025	0.002948515	938	16	960
Pinitol	g	Enhanced	early	0.0079	0.000649466	938	16	960
Pinitol	g	Enhanced	mid	0.007625	0.000392099	938	16	960
Pinitol	g	Enhanced	late	0.006141667	0.000485117	938	16	960
Polyunsaturated to Saturated Fat	Ratio	Standard	pre	0.709983333	0.050624316	938	16	960
Polyunsaturated to Saturated Fat	Ratio	Standard	early	0.547566667	0.017861353	938	16	960
Polyunsaturated to Saturated Fat	Ratio	Standard	mid	0.556066667	0.007661348	938	16	960
Polyunsaturated to Saturated Fat	Ratio	Standard	late	0.535108333	0.01123842	938	16	960
Polyunsaturated to Saturated Fat	Ratio	Enhanced	pre	0.656666667	0.054277402	938	16	960
Polyunsaturated to Saturated Fat	Ratio	Enhanced	early	0.691733333	0.013320478	938	16	960

Polyunsaturated to Saturated Fat	Ratio	Enhanced	mid	0.681216667	0.016125264	938	16	960
Polyunsaturated to Saturated Fat	Ratio	Enhanced	late	0.70085	0.030210745	938	16	960
Potassium	mg	Standard	pre	2997.2905	320.3201601	938	16	960
Potassium	mg	Standard	early	3610.228	195.3994618	938	16	960
Potassium	mg	Standard	mid	3481.392808	257.2782269	938	16	960
Potassium	mg	Standard	late	3556.959125	268.2711056	938	16	960
Potassium	mg	Enhanced	pre	3345.774367	239.0817619	938	16	960
Potassium	mg	Enhanced	early	4154.942083	108.7177229	938	16	960
Potassium	mg	Enhanced	mid	4301.398958	156.6787518	938	16	960
Potassium	mg	Enhanced	late	4267.830842	172.2459825	938	16	960
Proline	g	Standard	pre	6.724691667	0.715683176	938	16	960
Proline	g	Standard	early	6.289333333	0.312941597	938	16	960
Proline	g	Standard	mid	5.907916667	0.380197987	938	16	960
Proline	g	Standard	late	5.990008333	0.377756461	938	16	960
Proline	g	Enhanced	pre	6.918241667	0.48955773	938	16	960
Proline	g	Enhanced	early	6.097283333	0.254220387	938	16	960
Proline	g	Enhanced	mid	6.431491667	0.323354133	938	16	960
Proline	g	Enhanced	late	6.331841667	0.352339888	938	16	960
Refined Grains ounce	equivalents	Standard	pre	6.702683333	1.047589948	938	16	960
Refined Grains ounce	equivalents	Standard	early	0.662625	0.036703865	938	16	960
Refined Grains ounce	equivalents	Standard	mid	0.398291667	0.033437757	938	16	960
Refined Grains ounce	equivalents	Standard	late	0.381058333	0.02565502	938	16	960
Refined Grains ounce	equivalents	Enhanced	pre	5.675758333	0.559324479	938	16	960
Refined Grains ounce	equivalents	Enhanced	early	0.519716667	0.043012152	938	16	960
Refined Grains ounce	equivalents	Enhanced	mid	0.532575	0.043145622	938	16	960
Refined Grains ounce	equivalents	Enhanced	late	0.568325	0.059494872	938	16	960
Retinol	mcg	Standard	pre	802.3946083	163.9764456	938	16	960
Retinol	mcg	Standard	early	382.0961917	17.69736406	938	16	960
Retinol	mcg	Standard	mid	403.9731	24.04910269	938	16	960
Retinol	mcg	Standard	late	402.0361333	23.99015204	938	16	960
Retinol	mcg	Enhanced	pre	580.0972667	81.38800981	938	16	960
Retinol	mcg	Enhanced	early	447.8984667	27.49748686	938	16	960
Retinol	mcg	Enhanced	mid	473.5815167	23.74919458	938	16	960
Retinol	mcg	Enhanced	late	483.3298	34.0260601	938	16	960
Riboflavin vitamin B2	mg	Standard	pre	2.951833333	0.178337768	938	16	960
Riboflavin vitamin B2	mg	Standard	early	2.341325	0.10267299	938	16	960
Riboflavin vitamin B2	mg	Standard	mid	2.282125	0.127765696	938	16	960
Riboflavin vitamin B2	mg	Standard	late	2.3306	0.141777521	938	16	960
Riboflavin vitamin B2	mg	Enhanced	pre	2.636083333	0.246718276	938	16	960
Riboflavin vitamin B2	mg	Enhanced	early	2.443066667	0.092071958	938	16	960
Riboflavin vitamin B2	mg	Enhanced	mid	2.542025	0.111156329	938	16	960
Riboflavin vitamin B2	mg	Enhanced	late	2.5076	0.129099556	938	16	960
SFA 10 0 capric acid	g	Standard	pre	0.727958333	0.085689702	938	16	960
SFA 10 0 capric acid	g	Standard	early	0.642616667	0.031733258	938	16	960
SFA 10 0 capric acid	g	Standard	mid	0.648141667	0.041296562	938	16	960
SFA 10 0 capric acid	g	Standard	late	0.641975	0.043367954	938	16	960
SFA 10 0 capric acid	g	Enhanced	pre	0.847025	0.185616605	938	16	960
SFA 10 0 capric acid	g	Enhanced	early	0.640933333	0.023444956	938	16	960
SFA 10 0 capric acid	g	Enhanced	mid	0.616333333	0.031422519	938	16	960
SFA 10 0 capric acid	g	Enhanced	late	0.641441667	0.038786057	938	16	960
SFA 12 0 lauric acid	g	Standard	pre	1.582083333	0.349992032	938	16	960
SFA 12 0 lauric acid	g	Standard	early	1.550058333	0.101365026	938	16	960
SFA 12 0 lauric acid	g	Standard	mid	1.5825	0.11934023	938	16	960
SFA 12 0 lauric acid	g	Standard	late	1.6526	0.108475231	938	16	960
SFA 12 0 lauric acid	g	Enhanced	pre	1.732516667	0.483583104	938	16	960
SFA 12 0 lauric acid	g	Enhanced	early	1.749858333	0.063496186	938	16	960
SFA 12 0 lauric acid	g	Enhanced	mid	1.620916667	0.111778136	938	16	960
SFA 12 0 lauric acid	g	Enhanced	late	1.7976	0.143925216	938	16	960
SFA 14 0 myristic acid	g	Standard	pre	3.16355	0.383346783	938	16	960
SFA 14 0 myristic acid	g	Standard	early	2.5307	0.116785152	938	16	960
SFA 14 0 myristic acid	g	Standard	mid	2.485291667	0.150246919	938	16	960
SFA 14 0 myristic acid	g	Standard	late	2.491375	0.162071649	938	16	960
SFA 14 0 myristic acid	g	Enhanced	pre	3.521575	0.699069419	938	16	960
SFA 14 0 myristic acid	g	Enhanced	early	2.527725	0.096562037	938	16	960
SFA 14 0 myristic acid	g	Enhanced	mid	2.463275	0.119495291	938	16	960
SFA 14 0 myristic acid	g	Enhanced	late	2.547733333	0.159295343	938	16	960
SFA 16 0 palmitic acid	g	Standard	pre	21.378575	1.904182917	938	16	960
SFA 16 0 palmitic acid	g	Standard	early	12.47321667	0.637960391	938	16	960
SFA 16 0 palmitic acid	g	Standard	mid	11.98170833	0.702612516	938	16	960
SFA 16 0 palmitic acid	g	Standard	late	12.02501667	0.748048967	938	16	960
SFA 16 0 palmitic acid	g	Enhanced	pre	22.6047	2.837814456	938	16	960
SFA 16 0 palmitic acid	g	Enhanced	early	11.85920833	0.514753969	938	16	960

SFA 16 0 palmitic acid	g	Enhanced	mid	12.0799	0.589853024	938	16	960
SFA 16 0 palmitic acid	g	Enhanced	late	12.06050833	0.713829185	938	16	960
SFA 17 0 margaric acid	g	Standard	pre	0.192341667	0.016237249	938	16	960
SFA 17 0 margaric acid	g	Standard	early	0.1216	0.006225664	938	16	960
SFA 17 0 margaric acid	g	Standard	mid	0.118825	0.006500598	938	16	960
SFA 17 0 margaric acid	g	Standard	late	0.120441667	0.00724076	938	16	960
SFA 17 0 margaric acid	g	Enhanced	pre	0.197066667	0.029592517	938	16	960
SFA 17 0 margaric acid	g	Enhanced	early	0.099691667	0.004947922	938	16	960
SFA 17 0 margaric acid	g	Enhanced	mid	0.098141667	0.005462669	938	16	960
SFA 17 0 margaric acid	g	Enhanced	late	0.102866667	0.007042046	938	16	960
SFA 18 0 stearic acid	g	Standard	pre	9.871375	0.804991547	938	16	960
SFA 18 0 stearic acid	g	Standard	early	5.3987	0.282617322	938	16	960
SFA 18 0 stearic acid	g	Standard	mid	5.296308333	0.301428398	938	16	960
SFA 18 0 stearic acid	g	Standard	late	5.350908333	0.3164105	938	16	960
SFA 18 0 stearic acid	g	Enhanced	pre	10.30619167	1.462999036	938	16	960
SFA 18 0 stearic acid	g	Enhanced	early	4.656708333	0.20140456	938	16	960
SFA 18 0 stearic acid	g	Enhanced	mid	4.79815	0.240755161	938	16	960
SFA 18 0 stearic acid	g	Enhanced	late	4.746241667	0.286896141	938	16	960
SFA 20 0 arachidic acid	g	Standard	pre	0.220416667	0.02792458	938	16	960
SFA 20 0 arachidic acid	g	Standard	early	0.283058333	0.023661438	938	16	960
SFA 20 0 arachidic acid	g	Standard	mid	0.218641667	0.019532448	938	16	960
SFA 20 0 arachidic acid	g	Standard	late	0.2507	0.02290882	938	16	960
SFA 20 0 arachidic acid	g	Enhanced	pre	0.249983333	0.032133642	938	16	960
SFA 20 0 arachidic acid	g	Enhanced	early	0.272433333	0.02443578	938	16	960
SFA 20 0 arachidic acid	g	Enhanced	mid	0.253233333	0.021323383	938	16	960
SFA 20 0 arachidic acid	g	Enhanced	late	0.270858333	0.032157871	938	16	960
SFA 22 0 behenic acid	g	Standard	pre	0.168041667	0.037390757	938	16	960
SFA 22 0 behenic acid	g	Standard	early	0.190066667	0.018698284	938	16	960
SFA 22 0 behenic acid	g	Standard	mid	0.172608333	0.020924021	938	16	960
SFA 22 0 behenic acid	g	Standard	late	0.193458333	0.025455946	938	16	960
SFA 22 0 behenic acid	g	Enhanced	pre	0.206166667	0.050933845	938	16	960
SFA 22 0 behenic acid	g	Enhanced	early	0.178166667	0.014650611	938	16	960
SFA 22 0 behenic acid	g	Enhanced	mid	0.154425	0.017167548	938	16	960
SFA 22 0 behenic acid	g	Enhanced	late	0.160216667	0.018214946	938	16	960
SFA 4 0 butyric acid	g	Standard	pre	0.702875	0.097067445	938	16	960
SFA 4 0 butyric acid	g	Standard	early	0.698283333	0.029838823	938	16	960
SFA 4 0 butyric acid	g	Standard	mid	0.700033333	0.042105064	938	16	960
SFA 4 0 butyric acid	g	Standard	late	0.67905	0.046444828	938	16	960
SFA 4 0 butyric acid	g	Enhanced	pre	0.783358333	0.162506284	938	16	960
SFA 4 0 butyric acid	g	Enhanced	early	0.6478	0.026465638	938	16	960
SFA 4 0 butyric acid	g	Enhanced	mid	0.645025	0.027517093	938	16	960
SFA 4 0 butyric acid	g	Enhanced	late	0.646841667	0.042020736	938	16	960
SFA 6 0 caproic acid	g	Standard	pre	0.420191667	0.052945493	938	16	960
SFA 6 0 caproic acid	g	Standard	early	0.40985	0.018538012	938	16	960
SFA 6 0 caproic acid	g	Standard	mid	0.406458333	0.02557831	938	16	960
SFA 6 0 caproic acid	g	Standard	late	0.404683333	0.028841925	938	16	960
SFA 6 0 caproic acid	g	Enhanced	pre	0.513391667	0.117245714	938	16	960
SFA 6 0 caproic acid	g	Enhanced	early	0.387275	0.016252438	938	16	960
SFA 6 0 caproic acid	g	Enhanced	mid	0.381016667	0.017570765	938	16	960
SFA 6 0 caproic acid	g	Enhanced	late	0.382925	0.023892451	938	16	960
SFA 8 0 caprylic acid	g	Standard	pre	0.400391667	0.054842449	938	16	960
SFA 8 0 caprylic acid	g	Standard	early	0.393366667	0.021575211	938	16	960
SFA 8 0 caprylic acid	g	Standard	mid	0.39695	0.027463824	938	16	960
SFA 8 0 caprylic acid	g	Standard	late	0.406575	0.027013674	938	16	960
SFA 8 0 caprylic acid	g	Enhanced	pre	0.487466667	0.130527566	938	16	960
SFA 8 0 caprylic acid	g	Enhanced	early	0.420658333	0.013595814	938	16	960
SFA 8 0 caprylic acid	g	Enhanced	mid	0.393675	0.021746537	938	16	960
SFA 8 0 caprylic acid	g	Enhanced	late	0.418183333	0.026410214	938	16	960
Saccharin	mg	Standard	pre	-1.77636E-14	#N/A	938	16	960
Saccharin	mg	Standard	early	-1.5099E-14	#N/A	938	16	960
Saccharin	mg	Standard	mid	-1.33227E-14	#N/A	938	16	960
Saccharin	mg	Standard	late	-8.88178E-15	#N/A	938	16	960
Saccharin	mg	Enhanced	pre	1.06581E-14	#N/A	938	16	960
Saccharin	mg	Enhanced	early	1.15463E-14	#N/A	938	16	960
Saccharin	mg	Enhanced	mid	1.42109E-14	#N/A	#N/A	16	960
Saccharin	mg	Enhanced	late	6.104166667	5.709925075	938	16	960
Selenium	mcg	Standard	pre	162.49455	10.54296456	938	16	960
Selenium	mcg	Standard	early	139.2637583	8.346561865	938	16	960
Selenium	mcg	Standard	mid	145.1346083	9.26845399	938	16	960
Selenium	mcg	Standard	late	133.392925	7.986495868	938	16	960
Selenium	mcg	Enhanced	pre	155.199375	12.28589553	938	16	960
Selenium	mcg	Enhanced	early	145.6382417	6.246320913	938	16	960

Selenium	mcg	Enhanced	mid	154.4935583	8.047601413	938	16	960
Selenium	mcg	Enhanced	late	154.5119	8.198475045	938	16	960
Serine	g	Standard	pre	4.898725	0.362524879	938	16	960
Serine	g	Standard	early	4.986075	0.276354456	938	16	960
Serine	g	Standard	mid	4.753908333	0.310638254	938	16	960
Serine	g	Standard	late	4.837333333	0.30375284	938	16	960
Serine	g	Enhanced	pre	5.138508333	0.400743577	938	16	960
Serine	g	Enhanced	early	4.982425	0.202566282	938	16	960
Serine	g	Enhanced	mid	5.230758333	0.257834524	938	16	960
Serine	g	Enhanced	late	5.177608333	0.28892107	938	16	960
Sodium	mg	Standard	pre	4611.551983	417.3494023	938	16	960
Sodium	mg	Standard	early	2435.402275	108.3850872	938	16	960
Sodium	mg	Standard	mid	2429.770767	167.2150809	938	16	960
Sodium	mg	Standard	late	2343.850442	167.129638	938	16	960
Sodium	mg	Enhanced	pre	4217.935575	201.6381891	938	16	960
Sodium	mg	Enhanced	early	2274.693783	84.98592729	938	16	960
Sodium	mg	Enhanced	mid	2399.295717	107.301544	938	16	960
Sodium	mg	Enhanced	late	2297.66485	121.7185514	938	16	960
Solid Fats	g	Standard	pre	61.46554167	5.825315818	938	16	960
Solid Fats	g	Standard	early	5.343683333	0.358691697	938	16	960
Solid Fats	g	Standard	mid	6.737941667	0.377392135	938	16	960
Solid Fats	g	Standard	late	5.53475	0.692579299	938	16	960
Solid Fats	g	Enhanced	pre	61.28004167	10.27882834	938	16	960
Solid Fats	g	Enhanced	early	5.2254	0.337556018	938	16	960
Solid Fats	g	Enhanced	mid	4.783983333	0.458870332	938	16	960
Solid Fats	g	Enhanced	late	5.343458333	0.45800544	938	16	960
Soluble Dietary Fiber	g	Standard	pre	7.302966667	0.960213596	938	16	960
Soluble Dietary Fiber	g	Standard	early	7.124391667	0.328788318	938	16	960
Soluble Dietary Fiber	g	Standard	mid	6.906	0.458347548	938	16	960
Soluble Dietary Fiber	g	Standard	late	6.645725	0.474940788	938	16	960
Soluble Dietary Fiber	g	Enhanced	pre	7.574	0.801517235	938	16	960
Soluble Dietary Fiber	g	Enhanced	early	8.985425	0.448797054	938	16	960
Soluble Dietary Fiber	g	Enhanced	mid	9.246408333	0.50694053	938	16	960
Soluble Dietary Fiber	g	Enhanced	late	9.218975	0.507681149	938	16	960
Sorbitol	g	Standard	pre	0.179825	0.053750846	938	16	960
Sorbitol	g	Standard	early	0.878625	0.064845334	938	16	960
Sorbitol	g	Standard	mid	1.076	0.120392699	938	16	960
Sorbitol	g	Standard	late	0.8566	0.067588617	938	16	960
Sorbitol	g	Enhanced	pre	0.3247	0.102571803	938	16	960
Sorbitol	g	Enhanced	early	1.854133333	0.183436312	938	16	960
Sorbitol	g	Enhanced	mid	2.242725	0.213427944	938	16	960
Sorbitol	g	Enhanced	late	1.956216667	0.194375148	938	16	960
Starch	g	Standard	pre	122.5320583	19.51826352	938	16	960
Starch	g	Standard	early	111.5491167	5.730774185	938	16	960
Starch	g	Standard	mid	111.7429417	8.532238329	938	16	960
Starch	g	Standard	late	100.97645	9.169907549	938	16	960
Starch	g	Enhanced	pre	110.6445917	8.581003907	938	16	960
Starch	g	Enhanced	early	94.62625	3.84863464	938	16	960
Starch	g	Enhanced	mid	99.80283333	4.682833955	938	16	960
Starch	g	Enhanced	late	97.728675	4.200801819	938	16	960
Sucralose	mg	Standard	pre	0.51485	0.481598076	938	16	960
Sucralose	mg	Standard	early	-4.44089E-16	#N/A	#N/A	16	960
Sucralose	mg	Standard	mid	-2.22045E-16	#N/A	#N/A	16	960
Sucralose	mg	Standard	late	-1.11022E-16	#N/A	#N/A	16	960
Sucralose	mg	Enhanced	pre	1.508458333	1.101468667	938	16	960
Sucralose	mg	Enhanced	early	-6.66134E-16	#N/A	#N/A	16	960
Sucralose	mg	Enhanced	mid	-2.22045E-16	#N/A	#N/A	16	960
Sucralose	mg	Enhanced	late	0.583333333	0.545658369	938	16	960
Sucrose	g	Standard	pre	47.88801667	3.913820148	938	16	960
Sucrose	g	Standard	early	83.46964167	5.33502407	938	16	960
Sucrose	g	Standard	mid	77.87528333	5.546855225	938	16	960
Sucrose	g	Standard	late	80.05306667	7.96617492	938	16	960
Sucrose	g	Enhanced	pre	49.405775	7.201944608	938	16	960
Sucrose	g	Enhanced	early	69.960775	3.29155038	938	16	960
Sucrose	g	Enhanced	mid	74.53171667	3.691953223	938	16	960
Sucrose	g	Enhanced	late	74.585025	3.992229254	938	16	960
Synthetic Alpha Tocopherol all rac alpha tocopherol or dl alpha tocopherol	mg	Standard	pre	5.625	5.2617057	938	16	960
Synthetic Alpha Tocopherol all rac alpha tocopherol or dl alpha tocopherol	mg	Standard	early	1.5554	0.730682856	938	16	960
Synthetic Alpha Tocopherol all rac alpha tocopherol or dl alpha tocopherol	mg	Standard	mid	0.917516667	0.454140038	938	16	960
Synthetic Alpha Tocopherol all rac alpha tocopherol or dl alpha tocopherol	mg	Standard	late	1.566925	0.722190615	938	16	960
Synthetic Alpha Tocopherol all rac alpha tocopherol or dl alpha tocopherol	mg	Enhanced	pre	1.195466667	0.382108639	938	16	960
Synthetic Alpha Tocopherol all rac alpha tocopherol or dl alpha tocopherol	mg	Enhanced	early	1.606308333	0.317393834	938	16	960

Synthetic Alpha Tocopherol all rac alpha tocopherol or dl alpha tocopherol	mg	Enhanced	mid	1.576275	0.322291894	938	16	960
Synthetic Alpha Tocopherol all rac alpha tocopherol or dl alpha tocopherol	mg	Enhanced	late	1.159883333	0.169153683	938	16	960
Synthetic Folate folic acid	mcg	Standard	pre	138.2733583	22.19649371	938	16	960
Synthetic Folate folic acid	mcg	Standard	early	197.635875	7.851767491	938	16	960
Synthetic Folate folic acid	mcg	Standard	mid	179.1452167	11.82414265	938	16	960
Synthetic Folate folic acid	mcg	Standard	late	187.5812667	10.7563242	938	16	960
Synthetic Folate folic acid	mcg	Enhanced	pre	148.6982167	18.92675217	938	16	960
Synthetic Folate folic acid	mcg	Enhanced	early	141.5513083	5.985627743	938	16	960
Synthetic Folate folic acid	mcg	Enhanced	mid	156.2212833	6.315997722	938	16	960
Synthetic Folate folic acid	mcg	Enhanced	late	156.7671917	6.518385803	938	16	960
TRANS 16 1 trans hexadecenoic acid	g	Standard	pre	0.0733	0.011612433	938	16	960
TRANS 16 1 trans hexadecenoic acid	g	Standard	early	0.020575	0.001057377	938	16	960
TRANS 16 1 trans hexadecenoic acid	g	Standard	mid	0.023458333	0.001341198	938	16	960
TRANS 16 1 trans hexadecenoic acid	g	Standard	late	0.019266667	0.001221679	938	16	960
TRANS 16 1 trans hexadecenoic acid	g	Enhanced	pre	0.066741667	0.009021806	938	16	960
TRANS 16 1 trans hexadecenoic acid	g	Enhanced	early	0.01945	0.000704327	938	16	960
TRANS 16 1 trans hexadecenoic acid	g	Enhanced	mid	0.019016667	0.000899209	938	16	960
TRANS 16 1 trans hexadecenoic acid	g	Enhanced	late	0.019891667	0.0009467	938	16	960
TRANS 18 1 trans octadecenoic acid	g	Standard	pre	3.5967	0.514958695	938	16	960
TRANS 18 1 trans octadecenoic acid	g	Standard	early	1.595416667	0.099428664	938	16	960
TRANS 18 1 trans octadecenoic acid	g	Standard	mid	1.681666667	0.106043539	938	16	960
TRANS 18 1 trans octadecenoic acid	g	Standard	late	1.528291667	0.118304477	938	16	960
TRANS 18 1 trans octadecenoic acid	g	Enhanced	pre	2.685241667	0.291278261	938	16	960
TRANS 18 1 trans octadecenoic acid	g	Enhanced	early	1.090041667	0.037932088	938	16	960
TRANS 18 1 trans octadecenoic acid	g	Enhanced	mid	1.228575	0.069432742	938	16	960
TRANS 18 1 trans octadecenoic acid	g	Enhanced	late	1.182466667	0.053793676	938	16	960
TRANS 18 2 trans octadecadienoic acid	g	Standard	pre	0.580933333	0.075111623	938	16	960
TRANS 18 2 trans octadecadienoic acid	g	Standard	early	0.283058333	0.018356896	938	16	960
TRANS 18 2 trans octadecadienoic acid	g	Standard	mid	0.306333333	0.019938799	938	16	960
TRANS 18 2 trans octadecadienoic acid	g	Standard	late	0.283991667	0.024393753	938	16	960
TRANS 18 2 trans octadecadienoic acid	g	Enhanced	pre	0.480125	0.052097779	938	16	960
TRANS 18 2 trans octadecadienoic acid	g	Enhanced	early	0.247258333	0.009883076	938	16	960
TRANS 18 2 trans octadecadienoic acid	g	Enhanced	mid	0.278483333	0.01681389	938	16	960
TRANS 18 2 trans octadecadienoic acid	g	Enhanced	late	0.265758333	0.013239151	938	16	960
Tagatose	mg	Standard	pre	3.423758333	1.011995068	938	16	960
Tagatose	mg	Standard	early	3.338758333	0.230410982	938	16	960
Tagatose	mg	Standard	mid	3.053608333	0.277849564	938	16	960
Tagatose	mg	Standard	late	3.634833333	0.368321325	938	16	960
Tagatose	mg	Enhanced	pre	2.131633333	0.547345012	938	16	960
Tagatose	mg	Enhanced	early	6.657091667	0.509501388	938	16	960
Tagatose	mg	Enhanced	mid	6.452208333	0.588305115	938	16	960
Tagatose	mg	Enhanced	late	6.995116667	0.677045607	938	16	960
Thiamin vitamin B1	mg	Standard	pre	2.162791667	0.198178527	938	16	960
Thiamin vitamin B1	mg	Standard	early	1.626458333	0.099850092	938	16	960
Thiamin vitamin B1	mg	Standard	mid	1.592475	0.113369046	938	16	960
Thiamin vitamin B1	mg	Standard	late	1.527516667	0.128907268	938	16	960
Thiamin vitamin B1	mg	Enhanced	pre	2.047758333	0.158712131	938	16	960
Thiamin vitamin B1	mg	Enhanced	early	1.7594	0.087953791	938	16	960
Thiamin vitamin B1	mg	Enhanced	mid	1.807716667	0.087246899	938	16	960
Thiamin vitamin B1	mg	Enhanced	late	1.829566667	0.08731627	938	16	960
Threonine	g	Standard	pre	4.109216667	0.306792622	938	16	960
Threonine	g	Standard	early	4.294866667	0.238506738	938	16	960
Threonine	g	Standard	mid	4.124575	0.27336482	938	16	960
Threonine	g	Standard	late	4.19445	0.256352146	938	16	960
Threonine	g	Enhanced	pre	4.541175	0.354817066	938	16	960
Threonine	g	Enhanced	early	4.300741667	0.171172815	938	16	960
Threonine	g	Enhanced	mid	4.546275	0.230147468	938	16	960
Threonine	g	Enhanced	late	4.497258333	0.249851552	938	16	960
Total Alpha Tocopherol Equivalents	mg	Standard	pre	18.98583333	4.942608758	938	16	960
Total Alpha Tocopherol Equivalents	mg	Standard	early	12.96715	0.739990735	938	16	960
Total Alpha Tocopherol Equivalents	mg	Standard	mid	12.21855833	0.552949739	938	16	960
Total Alpha Tocopherol Equivalents	mg	Standard	late	12.79239167	0.68566188	938	16	960
Total Alpha Tocopherol Equivalents	mg	Enhanced	pre	14.66574167	1.42772283	938	16	960
Total Alpha Tocopherol Equivalents	mg	Enhanced	early	15.23094167	0.363978061	938	16	960
Total Alpha Tocopherol Equivalents	mg	Enhanced	mid	14.70333333	0.767975703	938	16	960
Total Alpha Tocopherol Equivalents	mg	Enhanced	late	15.01550833	0.92697817	938	16	960
Total Carbohydrate	g	Standard	pre	272.3382622	30.77632491	938	16	960
Total Carbohydrate	g	Standard	early	295.3658433	15.16028538	938	16	960
Total Carbohydrate	g	Standard	mid	285.66732	20.5281648	938	16	960
Total Carbohydrate	g	Standard	late	280.38343	23.53517776	938	16	960
Total Carbohydrate	g	Enhanced	pre	274.1194383	18.18697777	938	16	960
Total Carbohydrate	g	Enhanced	early	288.5987917	10.54929147	938	16	960

Total Carbohydrate	g	Enhanced	mid	304.0464817	11.63666879	938	16	960
Total Carbohydrate	g	Enhanced	late	300.3909133	11.86397582	938	16	960
Total Conjugated Linoleic Acid CLA 18 2	g	Standard	pre	0.176283333	0.01955234	938	16	960
Total Conjugated Linoleic Acid CLA 18 2	g	Standard	early	0.048941667	0.002446665	938	16	960
Total Conjugated Linoleic Acid CLA 18 2	g	Standard	mid	0.0521	0.002552803	938	16	960
Total Conjugated Linoleic Acid CLA 18 2	g	Standard	late	0.050358333	0.003260324	938	16	960
Total Conjugated Linoleic Acid CLA 18 2	g	Enhanced	pre	0.216883333	0.037905674	938	16	960
Total Conjugated Linoleic Acid CLA 18 2	g	Enhanced	early	0.0414	0.002442534	938	16	960
Total Conjugated Linoleic Acid CLA 18 2	g	Enhanced	mid	0.038191667	0.002686655	938	16	960
Total Conjugated Linoleic Acid CLA 18 2	g	Enhanced	late	0.0431	0.004265104	938	16	960
Total Dietary Fiber	g	Standard	pre	21.859275	3.127631924	938	16	960
Total Dietary Fiber	g	Standard	early	30.04748333	1.689265851	938	16	960
Total Dietary Fiber	g	Standard	mid	27.9829	2.127964429	938	16	960
Total Dietary Fiber	g	Standard	late	28.22164167	2.172102628	938	16	960
Total Dietary Fiber	g	Enhanced	pre	24.57745	3.179528131	938	16	960
Total Dietary Fiber	g	Enhanced	early	34.06891667	1.107289092	938	16	960
Total Dietary Fiber	g	Enhanced	mid	34.76385	1.586128001	938	16	960
Total Dietary Fiber	g	Enhanced	late	33.74520833	1.537855479	938	16	960
Total Fat	g	Standard	pre	118.7646472	10.46496448	938	16	960
Total Fat	g	Standard	early	80.06212225	5.304947403	938	16	960
Total Fat	g	Standard	mid	77.116365	5.176574564	938	16	960
Total Fat	g	Standard	late	76.40193033	5.391514494	938	16	960
Total Fat	g	Enhanced	pre	122.1148564	13.09537463	938	16	960
Total Fat	g	Enhanced	early	79.4228305	3.769714506	938	16	960
Total Fat	g	Enhanced	mid	79.37515525	4.468405333	938	16	960
Total Fat	g	Enhanced	late	80.70260508	4.772494459	938	16	960
Total Folate	mcg	Standard	pre	487.20312	50.11101064	938	16	960
Total Folate	mcg	Standard	early	486.7265842	14.15749732	938	16	960
Total Folate	mcg	Standard	mid	442.4453442	27.96714209	938	16	960
Total Folate	mcg	Standard	late	469.4934475	29.30148774	938	16	960
Total Folate	mcg	Enhanced	pre	457.8324517	47.58755583	938	16	960
Total Folate	mcg	Enhanced	early	528.87272	14.81465142	938	16	960
Total Folate	mcg	Enhanced	mid	559.9815525	16.52179757	938	16	960
Total Folate	mcg	Enhanced	late	535.8305158	20.00071848	938	16	960
Total Grains ounce	equivalents	Standard	pre	8.7673	1.485144709	938	16	960
Total Grains ounce	equivalents	Standard	early	0.662625	0.036703865	938	16	960
Total Grains ounce	equivalents	Standard	mid	0.398291667	0.033437757	938	16	960
Total Grains ounce	equivalents	Standard	late	0.381058333	0.02565502	938	16	960
Total Grains ounce	equivalents	Enhanced	pre	8.123841667	0.800834031	938	16	960
Total Grains ounce	equivalents	Enhanced	early	1.015258333	0.0748359	938	16	960
Total Grains ounce	equivalents	Enhanced	mid	0.9219	0.075528665	938	16	960
Total Grains ounce	equivalents	Enhanced	late	0.951033333	0.08954508	938	16	960
Total Monounsaturated Fatty Acids MUFA	g	Standard	pre	42.05766667	3.635046298	938	16	960
Total Monounsaturated Fatty Acids MUFA	g	Standard	early	30.43980833	2.059752218	938	16	960
Total Monounsaturated Fatty Acids MUFA	g	Standard	mid	28.36045	1.847476303	938	16	960
Total Monounsaturated Fatty Acids MUFA	g	Standard	late	28.7523	1.951516248	938	16	960
Total Monounsaturated Fatty Acids MUFA	g	Enhanced	pre	45.29570833	4.791909852	938	16	960
Total Monounsaturated Fatty Acids MUFA	g	Enhanced	early	30.15988333	1.538854	938	16	960
Total Monounsaturated Fatty Acids MUFA	g	Enhanced	mid	29.98999167	1.854442392	938	16	960
Total Monounsaturated Fatty Acids MUFA	g	Enhanced	late	30.571675	2.081549935	938	16	960
Total Polyunsaturated Fatty Acids PUFA	g	Standard	pre	26.73740833	3.194880128	938	16	960
Total Polyunsaturated Fatty Acids PUFA	g	Standard	early	13.90755833	0.976491689	938	16	960
Total Polyunsaturated Fatty Acids PUFA	g	Standard	mid	13.874475	0.914067939	938	16	960
Total Polyunsaturated Fatty Acids PUFA	g	Standard	late	13.51811667	0.998417562	938	16	960
Total Polyunsaturated Fatty Acids PUFA	g	Enhanced	pre	24.342725	1.998143163	938	16	960
Total Polyunsaturated Fatty Acids PUFA	g	Enhanced	early	16.32968333	0.66417651	938	16	960
Total Polyunsaturated Fatty Acids PUFA	g	Enhanced	mid	16.07535833	0.983241253	938	16	960
Total Polyunsaturated Fatty Acids PUFA	g	Enhanced	late	16.59298333	0.966442768	938	16	960
Total Protein	g	Standard	pre	108.3793946	7.993392889	938	16	960
Total Protein	g	Standard	early	112.1939257	6.416762694	938	16	960
Total Protein	g	Standard	mid	110.7598186	7.374388938	938	16	960
Total Protein	g	Standard	late	110.8679447	7.285319554	938	16	960
Total Protein	g	Enhanced	pre	116.068308	8.326360883	938	16	960
Total Protein	g	Enhanced	early	113.4586537	4.466025656	938	16	960
Total Protein	g	Enhanced	mid	119.4489638	5.9193401	938	16	960
Total Protein	g	Enhanced	late	118.4077507	6.178487646	938	16	960
Total Saturated Fatty Acids SFA	g	Standard	pre	39.455315	3.423239243	938	16	960
Total Saturated Fatty Acids SFA	g	Standard	early	26.44729782	1.506339009	938	16	960
Total Saturated Fatty Acids SFA	g	Standard	mid	25.97717417	1.657732662	938	16	960
Total Saturated Fatty Acids SFA	g	Standard	late	25.99327333	1.671324677	938	16	960
Total Saturated Fatty Acids SFA	g	Enhanced	pre	41.95000692	6.059952379	938	16	960
Total Saturated Fatty Acids SFA	g	Enhanced	early	24.43824849	1.038674625	938	16	960

Total Saturated Fatty Acids SFA	g	Enhanced	mid	24.60185675	1.251015713	938	16	960
Total Saturated Fatty Acids SFA	g	Enhanced	late	24.82242332	1.465338086	938	16	960
Total Sugars	g	Standard	pre	102.9870083	10.09240712	938	16	960
Total Sugars	g	Standard	early	126.0147333	6.968948188	938	16	960
Total Sugars	g	Standard	mid	119.6140333	8.463427247	938	16	960
Total Sugars	g	Standard	late	122.1669167	10.65612782	938	16	960
Total Sugars	g	Enhanced	pre	112.3364083	10.71235017	938	16	960
Total Sugars	g	Enhanced	early	133.906225	5.397106714	938	16	960
Total Sugars	g	Enhanced	mid	140.508975	5.333872785	938	16	960
Total Sugars	g	Enhanced	late	141.4668083	5.574697735	938	16	960
Total Trans Fatty Acids TRANS	g	Standard	pre	4.298666667	0.589923098	938	16	960
Total Trans Fatty Acids TRANS	g	Standard	early	1.940925	0.118955481	938	16	960
Total Trans Fatty Acids TRANS	g	Standard	mid	2.070683333	0.133233996	938	16	960
Total Trans Fatty Acids TRANS	g	Standard	late	1.872933333	0.148279775	938	16	960
Total Trans Fatty Acids TRANS	g	Enhanced	pre	3.295733333	0.356951108	938	16	960
Total Trans Fatty Acids TRANS	g	Enhanced	early	1.731733333	0.0888789	938	16	960
Total Trans Fatty Acids TRANS	g	Enhanced	mid	1.96005	0.090462535	938	16	960
Total Trans Fatty Acids TRANS	g	Enhanced	late	1.851233333	0.074642435	938	16	960
Total Vitamin A Activity International Units	IU	Standard	pre	8025.650483	1293.5876	938	16	960
Total Vitamin A Activity International Units	IU	Standard	early	8880.332975	639.0310265	938	16	960
Total Vitamin A Activity International Units	IU	Standard	mid	9498.412983	943.7246965	938	16	960
Total Vitamin A Activity International Units	IU	Standard	late	9972.339875	1164.302518	938	16	960
Total Vitamin A Activity International Units	IU	Enhanced	pre	7842.121742	1306.700394	938	16	960
Total Vitamin A Activity International Units	IU	Enhanced	early	12719.90029	417.099143	938	16	960
Total Vitamin A Activity International Units	IU	Enhanced	mid	13392.07359	462.1973416	938	16	960
Total Vitamin A Activity International Units	IU	Enhanced	late	13559.4863	612.3405458	938	16	960
Total Vitamin A Activity Retinol Activity Equivalents	mcg	Standard	pre	1091.821317	168.8341333	938	16	960
Total Vitamin A Activity Retinol Activity Equivalents	mcg	Standard	early	792.6298083	34.46838845	938	16	960
Total Vitamin A Activity Retinol Activity Equivalents	mcg	Standard	mid	841.7604917	59.57737061	938	16	960
Total Vitamin A Activity Retinol Activity Equivalents	mcg	Standard	late	860.336625	70.38886172	938	16	960
Total Vitamin A Activity Retinol Activity Equivalents	mcg	Enhanced	pre	875.580725	94.75204063	938	16	960
Total Vitamin A Activity Retinol Activity Equivalents	mcg	Enhanced	early	1039.374492	40.82436979	938	16	960
Total Vitamin A Activity Retinol Activity Equivalents	mcg	Enhanced	mid	1090.941983	41.0497479	938	16	960
Total Vitamin A Activity Retinol Activity Equivalents	mcg	Enhanced	late	1102.319583	51.15422203	938	16	960
Total Vitamin A Activity Retinol Equivalents	mcg	Standard	pre	1381.247876	197.6844729	938	16	960
Total Vitamin A Activity Retinol Equivalents	mcg	Standard	early	1203.163248	54.37389561	938	16	960
Total Vitamin A Activity Retinol Equivalents	mcg	Standard	mid	1279.547714	96.40556186	938	16	960
Total Vitamin A Activity Retinol Equivalents	mcg	Standard	late	1318.63685	121.3797473	938	16	960
Total Vitamin A Activity Retinol Equivalents	mcg	Enhanced	pre	1171.064166	142.1081276	938	16	960
Total Vitamin A Activity Retinol Equivalents	mcg	Enhanced	early	1630.850486	54.87145911	938	16	960
Total Vitamin A Activity Retinol Equivalents	mcg	Enhanced	mid	1708.302271	59.9955847	938	16	960
Total Vitamin A Activity Retinol Equivalents	mcg	Enhanced	late	1721.30923	76.47032234	938	16	960
Tryptophan	g	Standard	pre	1.260116667	0.098008093	938	16	960
Tryptophan	g	Standard	early	1.23585	0.071053559	938	16	960
Tryptophan	g	Standard	mid	1.191516667	0.079802602	938	16	960
Tryptophan	g	Standard	late	1.212275	0.077154153	938	16	960
Tryptophan	g	Enhanced	pre	1.38385	0.10433195	938	16	960
Tryptophan	g	Enhanced	early	1.257358333	0.054267955	938	16	960
Tryptophan	g	Enhanced	mid	1.332708333	0.070755548	938	16	960
Tryptophan	g	Enhanced	late	1.30155	0.074432892	938	16	960
Tyrosine	g	Standard	pre	3.681233333	0.295906637	938	16	960
Tyrosine	g	Standard	early	3.791375	0.209154043	938	16	960
Tyrosine	g	Standard	mid	3.624816667	0.236076455	938	16	960
Tyrosine	g	Standard	late	3.706333333	0.22750503	938	16	960
Tyrosine	g	Enhanced	pre	3.97335	0.306732321	938	16	960
Tyrosine	g	Enhanced	early	3.767091667	0.14976864	938	16	960
Tyrosine	g	Enhanced	mid	3.965725	0.193783275	938	16	960
Tyrosine	g	Enhanced	late	3.9127	0.215255538	938	16	960
Valine	g	Standard	pre	5.392275	0.406003128	938	16	960
Valine	g	Standard	early	5.535975	0.303351572	938	16	960
Valine	g	Standard	mid	5.306541667	0.344130111	938	16	960
Valine	g	Standard	late	5.404825	0.330625096	938	16	960
Valine	g	Enhanced	pre	5.810083333	0.444926468	938	16	960
Valine	g	Enhanced	early	5.556016667	0.222415632	938	16	960
Valine	g	Enhanced	mid	5.854508333	0.287079515	938	16	960
Valine	g	Enhanced	late	5.788875	0.322756532	938	16	960
Vegetable Protein	g	Standard	pre	31.16015	4.631190047	938	16	960
Vegetable Protein	g	Standard	early	37.15295833	2.337738131	938	16	960
Vegetable Protein	g	Standard	mid	35.30253333	2.700009438	938	16	960
Vegetable Protein	g	Standard	late	35.03999167	3.09719339	938	16	960
Vegetable Protein	g	Enhanced	pre	32.2313	3.206445238	938	16	960
Vegetable Protein	g	Enhanced	early	36.58845833	1.364683382	938	16	960

Vegetable Protein	g	Enhanced	mid	36.98804167	1.715099949	938	16	960
Vegetable Protein	g	Enhanced	late	36.39755833	1.762770821	938	16	960
Vitamin B 12 cobalamin	mcg	Standard	pre	8.957666667	2.668687287	938	16	960
Vitamin B 12 cobalamin	mcg	Standard	early	5.002941667	0.352039281	938	16	960
Vitamin B 12 cobalamin	mcg	Standard	mid	4.6921	0.245293648	938	16	960
Vitamin B 12 cobalamin	mcg	Standard	late	4.870233333	0.24941168	938	16	960
Vitamin B 12 cobalamin	mcg	Enhanced	pre	6.815691667	0.407979962	938	16	960
Vitamin B 12 cobalamin	mcg	Enhanced	early	4.591691667	0.202748401	938	16	960
Vitamin B 12 cobalamin	mcg	Enhanced	mid	4.899133333	0.258742867	938	16	960
Vitamin B 12 cobalamin	mcg	Enhanced	late	4.738441667	0.322313831	938	16	960
Vitamin B 6 pyridoxine pyridoxyl pyridoxamine	mg	Standard	pre	2.544633333	0.320498987	938	16	960
Vitamin B 6 pyridoxine pyridoxyl pyridoxamine	mg	Standard	early	2.536516667	0.094968533	938	16	960
Vitamin B 6 pyridoxine pyridoxyl pyridoxamine	mg	Standard	mid	2.376816667	0.129015907	938	16	960
Vitamin B 6 pyridoxine pyridoxyl pyridoxamine	mg	Standard	late	2.50415	0.117876459	938	16	960
Vitamin B 6 pyridoxine pyridoxyl pyridoxamine	mg	Enhanced	pre	2.57465	0.169657123	938	16	960
Vitamin B 6 pyridoxine pyridoxyl pyridoxamine	mg	Enhanced	early	2.513533333	0.072464079	938	16	960
Vitamin B 6 pyridoxine pyridoxyl pyridoxamine	mg	Enhanced	mid	2.683516667	0.088927056	938	16	960
Vitamin B 6 pyridoxine pyridoxyl pyridoxamine	mg	Enhanced	late	2.539066667	0.109733369	938	16	960
Vitamin C ascorbic acid	mg	Standard	pre	95.07508333	22.35400198	938	16	960
Vitamin C ascorbic acid	mg	Standard	early	106.5781417	8.11277344	938	16	960
Vitamin C ascorbic acid	mg	Standard	mid	98.47759167	11.24150265	938	16	960
Vitamin C ascorbic acid	mg	Standard	late	108.0704667	10.20900529	938	16	960
Vitamin C ascorbic acid	mg	Enhanced	pre	110.88285	27.71109892	938	16	960
Vitamin C ascorbic acid	mg	Enhanced	early	174.8907833	6.290355076	938	16	960
Vitamin C ascorbic acid	mg	Enhanced	mid	181.3092333	4.820602634	938	16	960
Vitamin C ascorbic acid	mg	Enhanced	late	183.9262583	7.329903848	938	16	960
Vitamin D calciferol	mcg	Standard	pre	22.23218333	14.32171832	938	16	960
Vitamin D calciferol	mcg	Standard	early	22.897375	0.536496483	938	16	960
Vitamin D calciferol	mcg	Standard	mid	24.7528	0.285939918	938	16	960
Vitamin D calciferol	mcg	Standard	late	24.1183	0.174979121	938	16	960
Vitamin D calciferol	mcg	Enhanced	pre	8.135658333	1.457298126	938	16	960
Vitamin D calciferol	mcg	Enhanced	early	26.92975	0.361148505	938	16	960
Vitamin D calciferol	mcg	Enhanced	mid	26.46831667	0.332274086	938	16	960
Vitamin D calciferol	mcg	Enhanced	late	26.21965833	0.507142429	938	16	960
Vitamin D2 ergocalciferol	mcg	Standard	pre	0.242425	0.164665549	938	16	960
Vitamin D2 ergocalciferol	mcg	Standard	early	0.175766667	0.155918161	938	16	960
Vitamin D2 ergocalciferol	mcg	Standard	mid	0.008225	0.000567516	938	16	960
Vitamin D2 ergocalciferol	mcg	Standard	late	0.005425	0.000315775	938	16	960
Vitamin D2 ergocalciferol	mcg	Enhanced	pre	0.02815	0.019866865	938	16	960
Vitamin D2 ergocalciferol	mcg	Enhanced	early	1.503725	0.436765803	938	16	960
Vitamin D2 ergocalciferol	mcg	Enhanced	mid	1.003825	0.204102144	938	16	960
Vitamin D2 ergocalciferol	mcg	Enhanced	late	1.337708333	0.408183771	938	16	960
Vitamin D3 cholecalciferol	mcg	Standard	pre	25.53145833	17.2261861	938	16	960
Vitamin D3 cholecalciferol	mcg	Standard	early	20.62195833	0.488092492	938	16	960
Vitamin D3 cholecalciferol	mcg	Standard	mid	21.57033333	0.394764976	938	16	960
Vitamin D3 cholecalciferol	mcg	Standard	late	21.63790833	0.230992273	938	16	960
Vitamin D3 cholecalciferol	mcg	Enhanced	pre	8.107341667	1.46290722	938	16	960
Vitamin D3 cholecalciferol	mcg	Enhanced	early	23.67210833	0.258136813	938	16	960
Vitamin D3 cholecalciferol	mcg	Enhanced	mid	22.94661667	0.161490417	938	16	960
Vitamin D3 cholecalciferol	mcg	Enhanced	late	22.81260833	0.425460548	938	16	960
Vitamin E International Units	IU	Standard	pre	22.691225	5.651037709	938	16	960
Vitamin E International Units	IU	Standard	early	18.75695833	1.019745753	938	16	960
Vitamin E International Units	IU	Standard	mid	17.6106	0.861377202	938	16	960
Vitamin E International Units	IU	Standard	late	18.41901667	0.827005825	938	16	960
Vitamin E International Units	IU	Enhanced	pre	18.40734167	2.061841441	938	16	960
Vitamin E International Units	IU	Enhanced	early	23.04153333	0.501169602	938	16	960
Vitamin E International Units	IU	Enhanced	mid	21.40204167	0.991190495	938	16	960
Vitamin E International Units	IU	Enhanced	late	22.05860833	1.288433544	938	16	960
Vitamin E Total Alpha Tocopherol	mg	Standard	pre	13.313675	2.347127084	938	16	960
Vitamin E Total Alpha Tocopherol	mg	Standard	early	10.9804	0.637722125	938	16	960
Vitamin E Total Alpha Tocopherol	mg	Standard	mid	10.54346667	0.573056522	938	16	960
Vitamin E Total Alpha Tocopherol	mg	Standard	late	10.82796667	0.566316056	938	16	960
Vitamin E Total Alpha Tocopherol	mg	Enhanced	pre	12.08529167	1.418400046	938	16	960
Vitamin E Total Alpha Tocopherol	mg	Enhanced	early	13.24896667	0.365322604	938	16	960
Vitamin E Total Alpha Tocopherol	mg	Enhanced	mid	12.68484167	0.727048996	938	16	960
Vitamin E Total Alpha Tocopherol	mg	Enhanced	late	13.24150833	0.820836771	938	16	960
Vitamin K phylloquinone	mcg	Standard	pre	171.620825	25.17840371	938	16	960
Vitamin K phylloquinone	mcg	Standard	early	101.6754667	5.248046181	938	16	960
Vitamin K phylloquinone	mcg	Standard	mid	96.600025	10.40515141	938	16	960
Vitamin K phylloquinone	mcg	Standard	late	109.2311167	10.8676371	938	16	960
Vitamin K phylloquinone	mcg	Enhanced	pre	224.8470833	65.78959721	938	16	960
Vitamin K phylloquinone	mcg	Enhanced	early	218.0652333	10.44340231	938	16	960

Vitamin K phylloquinone	mcg	Enhanced	mid	220.1163583	6.940222207	938	16	960
Vitamin K phylloquinone	mcg	Enhanced	late	216.0309667	11.05578273	938	16	960
Water	g	Standard	pre	2580.8124	268.1404608	938	16	960
Water	g	Standard	early	2730.922858	199.0303332	938	16	960
Water	g	Standard	mid	2603.173891	247.6234377	938	16	960
Water	g	Standard	late	2962.551783	311.7120603	938	16	960
Water	g	Enhanced	pre	2589.741033	372.9666287	938	16	960
Water	g	Enhanced	early	2396.947708	138.0421872	938	16	960
Water	g	Enhanced	mid	2400.161408	150.7647925	938	16	960
Water	g	Enhanced	late	2323.342489	205.0828433	938	16	960
Whole Grains ounce	equivalents	Standard	pre	2.064608333	0.516455891	938	16	960
Whole Grains ounce	equivalents	Standard	early	-4.44089E-16	#N/A	938	16	960
Whole Grains ounce	equivalents	Standard	mid	-2.77556E-16	#N/A	938	16	960
Whole Grains ounce	equivalents	Standard	late	-1.66533E-16	#N/A	938	16	960
Whole Grains ounce	equivalents	Enhanced	pre	2.448116667	0.673317551	938	16	960
Whole Grains ounce	equivalents	Enhanced	early	0.495466667	0.04344144	938	16	960
Whole Grains ounce	equivalents	Enhanced	mid	0.389208333	0.03724985	938	16	960
Whole Grains ounce	equivalents	Enhanced	late	0.382583333	0.038589513	938	16	960
% Calories from	Alcohol	Standard	pre	2.271383333	0.694075865	938	16	960
% Calories from	Alcohol	Standard	early	0.188158333	0.008126516	938	16	960
% Calories from	Alcohol	Standard	mid	0.157291667	0.00813189	938	16	960
% Calories from	Alcohol	Standard	late	0.164475	0.006890779	938	16	960
% Calories from	Alcohol	Enhanced	pre	3.682908333	1.05220067	938	16	960
% Calories from	Alcohol	Enhanced	early	0.145391667	0.008556718	938	16	960
% Calories from	Alcohol	Enhanced	mid	0.120541667	0.009527894	938	16	960
% Calories from	Alcohol	Enhanced	late	0.136533333	0.015466352	938	16	960
% Calories from	Carbohydrate	Standard	pre	39.24765675	2.196221387	938	16	960
% Calories from	Carbohydrate	Standard	early	51.02765625	0.543981421	938	16	960
% Calories from	Carbohydrate	Standard	mid	50.729198	0.702745651	938	16	960
% Calories from	Carbohydrate	Standard	late	50.30455658	0.988640908	938	16	960
% Calories from	Carbohydrate	Enhanced	pre	39.86773925	1.487527273	938	16	960
% Calories from	Carbohydrate	Enhanced	early	50.33135683	0.447284541	938	16	960
% Calories from	Carbohydrate	Enhanced	mid	51.3013565	0.635856297	938	16	960
% Calories from	Carbohydrate	Enhanced	late	50.90117292	0.660920473	938	16	960
% Calories from	Fat	Standard	pre	40.32674008	1.895957545	938	16	960
% Calories from	Fat	Standard	early	31.05079842	0.504789244	938	16	960
% Calories from	Fat	Standard	mid	30.85753975	0.426441971	938	16	960
% Calories from	Fat	Standard	late	30.94899025	0.501379149	938	16	960
% Calories from	Fat	Enhanced	pre	38.843381	1.498646817	938	16	960
% Calories from	Fat	Enhanced	early	31.06518183	0.590263859	938	16	960
% Calories from	Fat	Enhanced	mid	29.87838183	0.529803814	938	16	960
% Calories from	Fat	Enhanced	late	30.44083975	0.630504691	938	16	960
% Calories from	MUFA	Standard	pre	14.37135	0.812838938	938	16	960
% Calories from	MUFA	Standard	early	11.84409167	0.347670558	938	16	960
% Calories from	MUFA	Standard	mid	11.35876667	0.279395233	938	16	960
% Calories from	MUFA	Standard	late	11.72006667	0.341199553	938	16	960
% Calories from	MUFA	Enhanced	pre	14.325125	0.633991617	938	16	960
% Calories from	MUFA	Enhanced	early	11.77800833	0.315076997	938	16	960
% Calories from	MUFA	Enhanced	mid	11.239	0.270412164	938	16	960
% Calories from	MUFA	Enhanced	late	11.45458333	0.401799694	938	16	960
% Calories from	PUFA	Standard	pre	8.713975	0.389394678	938	16	960
% Calories from	PUFA	Standard	early	5.415925	0.126142177	938	16	960
% Calories from	PUFA	Standard	mid	5.540858333	0.102954628	938	16	960
% Calories from	PUFA	Standard	late	5.452983333	0.16037926	938	16	960
% Calories from	PUFA	Enhanced	pre	7.88035	0.410718385	938	16	960
% Calories from	PUFA	Enhanced	early	6.384275	0.079124644	938	16	960
% Calories from	PUFA	Enhanced	mid	6.009916667	0.114656361	938	16	960
% Calories from	PUFA	Enhanced	late	6.253941667	0.121849398	938	16	960
% Calories from	Protein	Standard	pre	18.12304041	1.768519162	938	16	960
% Calories from	Protein	Standard	early	19.51232367	0.269931453	938	16	960
% Calories from	Protein	Standard	mid	19.88321483	0.402306509	938	16	960
% Calories from	Protein	Standard	late	20.27854858	0.520290921	938	16	960
% Calories from	Protein	Enhanced	pre	17.64684016	0.872352959	938	16	960
% Calories from	Protein	Enhanced	early	20.03289833	0.140618492	938	16	960
% Calories from	Protein	Enhanced	mid	20.3334485	0.261429811	938	16	960
% Calories from	Protein	Enhanced	late	20.18185725	0.336658745	938	16	960
% Calories from	SFA	Standard	pre	13.54360833	0.930920981	938	16	960
% Calories from	SFA	Standard	early	10.29923333	0.141364299	938	16	960
% Calories from	SFA	Standard	mid	10.460375	0.108885449	938	16	960
% Calories from	SFA	Standard	late	10.59435	0.231976279	938	16	960
% Calories from	SFA	Enhanced	pre	13.27753333	0.967035042	938	16	960
% Calories from	SFA	Enhanced	early	9.583941667	0.16016199	938	16	960

% Calories from	SFA	Enhanced	mid	9.326483333	0.132535472	938	16	960
% Calories from	SFA	Enhanced	late	9.410908333	0.257367522	938	16	960
X3 Methylhistidine	mg	Standard	pre	20.44254167	2.824322212	938	16	960
X3 Methylhistidine	mg	Standard	early	28.48045	1.600167764	938	16	960
X3 Methylhistidine	mg	Standard	mid	27.21495833	2.05464261	938	16	960
X3 Methylhistidine	mg	Standard	late	27.44755833	1.550180381	938	16	960
X3 Methylhistidine	mg	Enhanced	pre	28.79786667	2.768645294	938	16	960
X3 Methylhistidine	mg	Enhanced	early	22.47875833	0.862527979	938	16	960
X3 Methylhistidine	mg	Enhanced	mid	25.1537	1.500069	938	16	960
X3 Methylhistidine	mg	Enhanced	late	24.78751667	1.425372435	938	16	960
Xylitol	g	Standard	pre	0.017308333	0.002447559	938	16	960
Xylitol	g	Standard	early	0.017075	0.000624548	938	16	960
Xylitol	g	Standard	mid	0.019908333	0.001523836	938	16	960
Xylitol	g	Standard	late	0.020158333	0.001646416	938	16	960
Xylitol	g	Enhanced	pre	0.025316667	0.007409845	938	16	960
Xylitol	g	Enhanced	early	0.027325	0.001947878	938	16	960
Xylitol	g	Enhanced	mid	0.030158333	0.002060001	938	16	960
Xylitol	g	Enhanced	late	0.029116667	0.001918975	938	16	960
Zinc	mg	Standard	pre	15.024675	1.65173339	938	16	960
Zinc	mg	Standard	early	14.68155	0.639648793	938	16	960
Zinc	mg	Standard	mid	13.92453333	0.7748503	938	16	960
Zinc	mg	Standard	late	14.21301667	0.815741685	938	16	960
Zinc	mg	Enhanced	pre	14.45355833	1.263221018	938	16	960
Zinc	mg	Enhanced	early	13.722875	0.452321858	938	16	960
Zinc	mg	Enhanced	mid	14.12413333	0.545332694	938	16	960
Zinc	mg	Enhanced	late	14.183775	0.685200413	938	16	960

Table S6: Average Individual Serum Markers

Measurement	RangeUnits	Menu	Time	Estimate	StdErr	DF	Nsubjects	Nobs
1,25-dihydroxyvitamin D	48-190 pmol/L	Enhanced	pre	131.5875	9.02161428	58	16	80
1,25-dihydroxyvitamin D	48-190 pmol/L	Enhanced	early	100.52	7.997899724	58	16	80
1,25-dihydroxyvitamin D	48-190 pmol/L	Enhanced	mid	102.75	9.452036156	58	16	80
1,25-dihydroxyvitamin D	48-190 pmol/L	Enhanced	late	108.3	7.74165357	58	16	80
1,25-dihydroxyvitamin D	48-190 pmol/L	Standard	pre	120.8125	5.398884072	58	16	80
1,25-dihydroxyvitamin D	48-190 pmol/L	Standard	early	101.125	5.82139776	58	16	80
1,25-dihydroxyvitamin D	48-190 pmol/L	Standard	mid	111.25	7.381882382	58	16	80
1,25-dihydroxyvitamin D	48-190 pmol/L	Standard	late	112.125	9.080262765	58	16	80
2-aminoadipic acid	0.3- 1.8 umol/L	Enhanced	pre	0.98375	0.075132695	26	8	40
2-aminoadipic acid	0.3- 1.8 umol/L	Enhanced	early	0.79	0.070975348	26	8	40
2-aminoadipic acid	0.3- 1.8 umol/L	Enhanced	mid	0.8825	0.090372493	26	8	40
2-aminoadipic acid	0.3- 1.8 umol/L	Enhanced	late	0.865	0.047762433	26	8	40
2-aminoadipic acid	0.3- 1.8 umol/L	Standard	pre	1.01	0.098821177	26	8	40
2-aminoadipic acid	0.3- 1.8 umol/L	Standard	early	0.78	0.093541435	26	8	40
2-aminoadipic acid	0.3- 1.8 umol/L	Standard	mid	0.8975	0.040983991	26	8	40
2-aminoadipic acid	0.3- 1.8 umol/L	Standard	late	0.785	0.031124749	26	8	40
2-hydroxybutyrate	10-60 umol/L	Enhanced	pre	39.81166667	2.940621785	42	12	60
2-hydroxybutyrate	10-60 umol/L	Enhanced	early	41.195	4.164914798	42	12	60
2-hydroxybutyrate	10-60 umol/L	Enhanced	mid	45.05166667	3.982459748	42	12	60
2-hydroxybutyrate	10-60 umol/L	Enhanced	late	38.79	3.475252189	42	12	60
2-hydroxybutyrate	10-60 umol/L	Standard	pre	33.01833333	1.951198943	42	12	60
2-hydroxybutyrate	10-60 umol/L	Standard	early	40.87	3.615867039	42	12	60
2-hydroxybutyrate	10-60 umol/L	Standard	mid	36.94666667	4.271627758	42	12	60
2-hydroxybutyrate	10-60 umol/L	Standard	late	36.90333333	2.766602074	42	12	60
25-hydroxyvitamin D3	32.4-98.5 nmol/L	Enhanced	pre	79.8425	15.35131499	58	16	80
25-hydroxyvitamin D3	32.4-98.5 nmol/L	Enhanced	early	79.27	10.84543568	58	16	80
25-hydroxyvitamin D3	32.4-98.5 nmol/L	Enhanced	mid	78.0075	5.248152577	58	16	80
25-hydroxyvitamin D3	32.4-98.5 nmol/L	Enhanced	late	79.995	4.363191206	58	16	80
25-hydroxyvitamin D3	32.4-98.5 nmol/L	Standard	pre	82.69	6.344155467	58	16	80
25-hydroxyvitamin D3	32.4-98.5 nmol/L	Standard	early	78.945	4.519507371	58	16	80
25-hydroxyvitamin D3	32.4-98.5 nmol/L	Standard	mid	77.3875	4.556368397	58	16	80
25-hydroxyvitamin D3	32.4-98.5 nmol/L	Standard	late	76.4875	5.133960992	58	16	80
3-hydroxyanthranilic acid	7-50 nmol/L	Enhanced	pre	44.475	5.561439506	58	16	80
3-hydroxyanthranilic acid	7-50 nmol/L	Enhanced	early	32.625	2.657286562	58	16	80
3-hydroxyanthranilic acid	7-50 nmol/L	Enhanced	mid	36.1125	4.679424294	58	16	80
3-hydroxyanthranilic acid	7-50 nmol/L	Enhanced	late	31.075	3.473324686	58	16	80
3-hydroxyanthranilic acid	7-50 nmol/L	Standard	pre	53.41875	3.602982028	58	16	80
3-hydroxyanthranilic acid	7-50 nmol/L	Standard	early	34.875	2.702125899	58	16	80
3-hydroxyanthranilic acid	7-50 nmol/L	Standard	mid	42.975	2.845267452	58	16	80
3-hydroxyanthranilic acid	7-50 nmol/L	Standard	late	37.55	2.70075221	58	16	80
3-hydroxybutyrate	20-700 umol/L	Enhanced	pre	64.1825	23.63817045	42	12	60
3-hydroxybutyrate	20-700 umol/L	Enhanced	early	103.7966667	26.48532861	42	12	60
3-hydroxybutyrate	20-700 umol/L	Enhanced	mid	92.38833333	21.72726889	42	12	60
3-hydroxybutyrate	20-700 umol/L	Enhanced	late	57.60166667	8.420525655	42	12	60
3-hydroxybutyrate	20-700 umol/L	Standard	pre	27.48416667	1.562134587	42	12	60
3-hydroxybutyrate	20-700 umol/L	Standard	early	45.57333333	4.758989544	42	12	60
3-hydroxybutyrate	20-700 umol/L	Standard	mid	34.69333333	2.564203002	42	12	60
3-hydroxybutyrate	20-700 umol/L	Standard	late	33.705	5.644373674	42	12	60
3-hydroxyisobutyrate	15-25 umol/L	Enhanced	pre	18.723125	0.881069546	58	16	80
3-hydroxyisobutyrate	15-25 umol/L	Enhanced	early	18.96625	1.497084602	58	16	80
3-hydroxyisobutyrate	15-25 umol/L	Enhanced	mid	21.75625	1.6702198	58	16	80
3-hydroxyisobutyrate	15-25 umol/L	Enhanced	late	18.74375	0.479683937	58	16	80
3-hydroxyisobutyrate	15-25 umol/L	Standard	pre	19.12	1.600312714	58	16	80
3-hydroxyisobutyrate	15-25 umol/L	Standard	early	18.48375	1.050438803	58	16	80
3-hydroxyisobutyrate	15-25 umol/L	Standard	mid	25.08	2.358558882	58	16	80
3-hydroxyisobutyrate	15-25 umol/L	Standard	late	20.22375	1.513520252	58	16	80
3-hydroxykynurenine	10-65 nmol/L	Enhanced	pre	43.675	3.049269892	58	16	80
3-hydroxykynurenine	10-65 nmol/L	Enhanced	early	37.6	2.342941527	58	16	80
3-hydroxykynurenine	10-65 nmol/L	Enhanced	mid	39.7625	2.971475722	58	16	80
3-hydroxykynurenine	10-65 nmol/L	Enhanced	late	38.2375	2.843684752	58	16	80
3-hydroxykynurenine	10-65 nmol/L	Standard	pre	55.13125	3.848639548	58	16	80
3-hydroxykynurenine	10-65 nmol/L	Standard	early	41.4	2.140531476	58	16	80
3-hydroxykynurenine	10-65 nmol/L	Standard	mid	50.675	3.083360079	58	16	80
3-hydroxykynurenine	10-65 nmol/L	Standard	late	43.3375	1.878574717	58	16	80
4-pyridoxic acid	11-88 nmol/L	Enhanced	pre	32.25	4.268014029	58	16	80
4-pyridoxic acid	11-88 nmol/L	Enhanced	early	25.55	2.008429113	58	16	80
4-pyridoxic acid	11-88 nmol/L	Enhanced	mid	20.66125	1.810696031	58	16	80
4-pyridoxic acid	11-88 nmol/L	Enhanced	late	19.32375	2.645784346	58	16	80
4-pyridoxic acid	11-88 nmol/L	Standard	pre	25.7	4.005894875	58	16	80
4-pyridoxic acid	11-88 nmol/L	Standard	early	19.3875	1.518588726	58	16	80
4-pyridoxic acid	11-88 nmol/L	Standard	mid	17.15	1.460535005	58	16	80
4-pyridoxic acid	11-88 nmol/L	Standard	late	18	1.661674607	58	16	80
Acetoacetate	10-200 umol/L	Enhanced	pre	33.24083333	8.525604933	42	12	60
Acetoacetate	10-200 umol/L	Enhanced	early	51.74166667	8.384028274	42	12	60
Acetoacetate	10-200 umol/L	Enhanced	mid	51.71	7.950783679	42	12	60
Acetoacetate	10-200 umol/L	Enhanced	late	36.745	3.297895583	42	12	60
Acetoacetate	10-200 umol/L	Standard	pre	24.35916667	1.95428426	42	12	60
Acetoacetate	10-200 umol/L	Standard	early	32.32666667	3.240161747	42	12	60
Acetoacetate	10-200 umol/L	Standard	mid	28.62666667	2.01854825	42	12	60
Acetoacetate	10-200 umol/L	Standard	late	26.185	3.586403917	42	12	60
Alanine	230-510 umol/L	Enhanced	pre	397	19.79445156	58	16	80

Alanine	230-510 umol/L	Enhanced	early	318.5	21.31240836	58	16	80
Alanine	230-510 umol/L	Enhanced	mid	328.125	16.37589454	58	16	80
Alanine	230-510 umol/L	Enhanced	late	352.125	20.31504792	58	16	80
Alanine	230-510 umol/L	Standard	pre	429.5	25.36622385	58	16	80
Alanine	230-510 umol/L	Standard	early	371	25.17873607	58	16	80
Alanine	230-510 umol/L	Standard	mid	393.375	20.12766869	58	16	80
Alanine	230-510 umol/L	Standard	late	379.75	21.82941679	58	16	80
Alanine Aminotransferase (ALT)	Males 19-74 U/L; Females 13-44 U/L	Enhanced	pre	26.12035937	3.201672789	43	13	62
Alanine Aminotransferase (ALT)	Males 19-74 U/L; Females 13-44 U/L	Enhanced	early	26.91360436	1.668949607	43	13	62
Alanine Aminotransferase (ALT)	Males 19-74 U/L; Females 13-44 U/L	Enhanced	mid	23.71428571	2.116160228	43	13	62
Alanine Aminotransferase (ALT)	Males 19-74 U/L; Females 13-44 U/L	Enhanced	late	23.18409808	2.176705181	43	13	62
Alanine Aminotransferase (ALT)	Males 19-74 U/L; Females 13-44 U/L	Standard	pre	24.25	2.23529143	43	13	62
Alanine Aminotransferase (ALT)	Males 19-74 U/L; Females 13-44 U/L	Standard	early	25.16666667	1.516880327	43	13	62
Alanine Aminotransferase (ALT)	Males 19-74 U/L; Females 13-44 U/L	Standard	mid	24.16666667	1.16468085	43	13	62
Alanine Aminotransferase (ALT)	Males 19-74 U/L; Females 13-44 U/L	Standard	late	23.83333333	1.673596703	43	13	62
Albumin	3.8-5.0 g/dL	Enhanced	pre	4.74375	0.055507249	58	16	80
Albumin	3.8-5.0 g/dL	Enhanced	early	4.65	0.10155048	58	16	80
Albumin	3.8-5.0 g/dL	Enhanced	mid	4.4125	0.103738704	58	16	80
Albumin	3.8-5.0 g/dL	Enhanced	late	4.4125	0.081848755	58	16	80
Albumin	3.8-5.0 g/dL	Standard	pre	4.68125	0.075486702	58	16	80
Albumin	3.8-5.0 g/dL	Standard	early	4.5625	0.099902296	58	16	80
Albumin	3.8-5.0 g/dL	Standard	mid	4.4625	0.084663857	58	16	80
Albumin	3.8-5.0 g/dL	Standard	late	4.5125	0.092597077	58	16	80
Aldosterone	35-300 pg/mL	Enhanced	pre	175.0733645	21.88463922	57	16	79
Aldosterone	35-300 pg/mL	Enhanced	early	267.7625	43.91170807	57	16	79
Aldosterone	35-300 pg/mL	Enhanced	mid	193.6125	22.29883992	57	16	79
Aldosterone	35-300 pg/mL	Enhanced	late	148.625	17.65144957	57	16	79
Aldosterone	35-300 pg/mL	Standard	pre	181.2625	21.79242405	57	16	79
Aldosterone	35-300 pg/mL	Standard	early	219.65	20.09380346	57	16	79
Aldosterone	35-300 pg/mL	Standard	mid	223.6125	22.64954772	57	16	79
Aldosterone	35-300 pg/mL	Standard	late	194.2375	17.23023267	57	16	79
Alkaline Phosphatase (ALP)	43-99 U/L	Enhanced	pre	69.0625	4.051849019	58	16	80
Alkaline Phosphatase (ALP)	43-99 U/L	Enhanced	early	67	4.565495592	58	16	80
Alkaline Phosphatase (ALP)	43-99 U/L	Enhanced	mid	65.625	3.618258818	58	16	80
Alkaline Phosphatase (ALP)	43-99 U/L	Enhanced	late	64.625	4.007561213	58	16	80
Alkaline Phosphatase (ALP)	43-99 U/L	Standard	pre	65.5625	5.982681712	58	16	80
Alkaline Phosphatase (ALP)	43-99 U/L	Standard	early	62.5	6.222439232	58	16	80
Alkaline Phosphatase (ALP)	43-99 U/L	Standard	mid	59.625	4.971599026	58	16	80
Alkaline Phosphatase (ALP)	43-99 U/L	Standard	late	61.125	5.702076102	58	16	80
All-trans retinol	1.46-2.84 umol/L	Enhanced	pre	2.22875	0.137300281	58	16	80
All-trans retinol	1.46-2.84 umol/L	Enhanced	early	2.0325	0.11625336	58	16	80
All-trans retinol	1.46-2.84 umol/L	Enhanced	mid	1.98375	0.139943487	58	16	80
All-trans retinol	1.46-2.84 umol/L	Enhanced	late	1.94875	0.147271407	58	16	80
All-trans retinol	1.46-2.84 umol/L	Standard	pre	2.401875	0.155683818	58	16	80
All-trans retinol	1.46-2.84 umol/L	Standard	early	2.1525	0.142552074	58	16	80
All-trans retinol	1.46-2.84 umol/L	Standard	mid	2.025	0.131303561	58	16	80
All-trans retinol	1.46-2.84 umol/L	Standard	late	2.08	0.141133802	58	16	80
Alpha-tocopherol	18.9-38.8 umol/L	Enhanced	pre	30.59625	1.937229165	58	16	80
Alpha-tocopherol	18.9-38.8 umol/L	Enhanced	early	31.3525	2.000755131	58	16	80
Alpha-tocopherol	18.9-38.8 umol/L	Enhanced	mid	28.92375	1.181296874	58	16	80
Alpha-tocopherol	18.9-38.8 umol/L	Enhanced	late	27.1475	1.801246747	58	16	80
Alpha-tocopherol	18.9-38.8 umol/L	Standard	pre	29.61	1.769896713	58	16	80
Alpha-tocopherol	18.9-38.8 umol/L	Standard	early	29.995	2.136509273	58	16	80
Alpha-tocopherol	18.9-38.8 umol/L	Standard	mid	28.35125	2.432865105	58	16	80
Alpha-tocopherol	18.9-38.8 umol/L	Standard	late	28.24125	2.446865621	58	16	80
Angiotensin II	10-60 pg/mL	Enhanced	pre	16.0625	2.959937579	58	16	80
Angiotensin II	10-60 pg/mL	Enhanced	early	15.5	2.270737766	58	16	80
Angiotensin II	10-60 pg/mL	Enhanced	mid	13.375	1.935987313	58	16	80
Angiotensin II	10-60 pg/mL	Enhanced	late	14.875	3.93477088	58	16	80
Angiotensin II	10-60 pg/mL	Standard	pre	13.6875	2.609983088	58	16	80
Angiotensin II	10-60 pg/mL	Standard	early	14.375	3.086955924	58	16	80
Angiotensin II	10-60 pg/mL	Standard	mid	13.875	3.124687484	58	16	80
Angiotensin II	10-60 pg/mL	Standard	late	13	2.899353376	58	16	80
Anthranilic acid	7-30 nmol/L	Enhanced	pre	20.0875	1.391849882	58	16	80
Anthranilic acid	7-30 nmol/L	Enhanced	early	19.6375	1.904266386	58	16	80
Anthranilic acid	7-30 nmol/L	Enhanced	mid	19.2375	1.299511627	58	16	80
Anthranilic acid	7-30 nmol/L	Enhanced	late	16.775	1.956060294	58	16	80
Anthranilic acid	7-30 nmol/L	Standard	pre	20.01875	0.760033793	58	16	80
Anthranilic acid	7-30 nmol/L	Standard	early	18.2625	0.565115447	58	16	80
Anthranilic acid	7-30 nmol/L	Standard	mid	19.8125	0.745494278	58	16	80
Anthranilic acid	7-30 nmol/L	Standard	late	20.9	1.605751382	58	16	80
Apigenin, plasma	ng/mL	Enhanced	pre	3.505217153	1.196692863	17	13	36
Apigenin, plasma	ng/mL	Enhanced	early	4.208571429	1.155770665	17	13	36
Apigenin, plasma	ng/mL	Enhanced	mid	2.791883819	0.674637169	17	13	36
Apigenin, plasma	ng/mL	Enhanced	late	3.485692213	1.498357153	17	13	36
Apigenin, plasma	ng/mL	Standard	pre	4.552053862	1.92500569	17	13	36
Apigenin, plasma	ng/mL	Standard	early	2.75819074	0.763237232	17	13	36
Apigenin, plasma	ng/mL	Standard	mid	2.864857061	0.888150104	17	13	36
Apigenin, plasma	ng/mL	Standard	late	1.937145444	0.629834143	17	13	36
Asparagine	20-130 umol/L	Enhanced	pre	63.528125	2.547739644	58	16	80
Asparagine	20-130 umol/L	Enhanced	early	63.92125	2.571035442	58	16	80
Asparagine	20-130 umol/L	Enhanced	mid	62.7525	2.888468858	58	16	80
Asparagine	20-130 umol/L	Enhanced	late	68.3025	2.546264267	58	16	80

Asparagine	20-130 umol/L	Standard	pre	60.795625	1.964072308	58	16	80
Asparagine	20-130 umol/L	Standard	early	60.69625	2.575794742	58	16	80
Asparagine	20-130 umol/L	Standard	mid	61.60375	2.79576909	58	16	80
Asparagine	20-130 umol/L	Standard	late	60.57875	1.978785484	58	16	80
Aspartate Amino-transferase (AST)	Males 20-52 U/L; Females 17-38 U/L	Enhanced	pre	27.625	2.415364232	58	16	80
Aspartate Amino-transferase (AST)	Males 20-52 U/L; Females 17-38 U/L	Enhanced	early	30	3.917109904	58	16	80
Aspartate Amino-transferase (AST)	Males 20-52 U/L; Females 17-38 U/L	Enhanced	mid	23.125	1.374289589	58	16	80
Aspartate Amino-transferase (AST)	Males 20-52 U/L; Females 17-38 U/L	Enhanced	late	22.625	1.223947252	58	16	80
Aspartate Amino-transferase (AST)	Males 20-52 U/L; Females 17-38 U/L	Standard	pre	24.75	2.260392665	58	16	80
Aspartate Amino-transferase (AST)	Males 20-52 U/L; Females 17-38 U/L	Standard	early	23.5	1.075290658	58	16	80
Aspartate Amino-transferase (AST)	Males 20-52 U/L; Females 17-38 U/L	Standard	mid	22.625	1.131060067	58	16	80
Aspartate Amino-transferase (AST)	Males 20-52 U/L; Females 17-38 U/L	Standard	late	21.375	0.808886194	58	16	80
Aspartic Acid	0-26 umol/L	Enhanced	pre	22.26	1.187394403	58	16	80
Aspartic Acid	0-26 umol/L	Enhanced	early	21.9875	1.496688793	58	16	80
Aspartic Acid	0-26 umol/L	Enhanced	mid	18.39375	1.205178848	58	16	80
Aspartic Acid	0-26 umol/L	Enhanced	late	18.14125	1.337005092	58	16	80
Aspartic Acid	0-26 umol/L	Standard	pre	23.66625	1.524528552	58	16	80
Aspartic Acid	0-26 umol/L	Standard	early	23.34	1.006707194	58	16	80
Aspartic Acid	0-26 umol/L	Standard	mid	21.7675	1.336769368	58	16	80
Aspartic Acid	0-26 umol/L	Standard	late	21.745	1.054831148	58	16	80
Atrial Natriuretic Peptide (ANP)	9-68 pg/mL	Enhanced	pre	29.70610178	1.517243798	56	16	78
Atrial Natriuretic Peptide (ANP)	9-68 pg/mL	Enhanced	early	38.45	4.047221269	56	16	78
Atrial Natriuretic Peptide (ANP)	9-68 pg/mL	Enhanced	mid	41.53144735	4.915903222	56	16	78
Atrial Natriuretic Peptide (ANP)	9-68 pg/mL	Enhanced	late	41.9625	5.388469678	56	16	78
Atrial Natriuretic Peptide (ANP)	9-68 pg/mL	Standard	pre	34.29375	3.087201877	56	16	78
Atrial Natriuretic Peptide (ANP)	9-68 pg/mL	Standard	early	39.2375	4.206911631	56	16	78
Atrial Natriuretic Peptide (ANP)	9-68 pg/mL	Standard	mid	38.4375	3.960505709	56	16	78
Atrial Natriuretic Peptide (ANP)	9-68 pg/mL	Standard	late	33.35	2.998958152	56	16	78
Bilirubin	0.2-1.5 mg/dL	Enhanced	pre	0.675	0.057960116	57	16	79
Bilirubin	0.2-1.5 mg/dL	Enhanced	early	0.5875	0.062343554	57	16	79
Bilirubin	0.2-1.5 mg/dL	Enhanced	mid	0.475	0.052291252	57	16	79
Bilirubin	0.2-1.5 mg/dL	Enhanced	late	0.5625	0.055726733	57	16	79
Bilirubin	0.2-1.5 mg/dL	Standard	pre	0.499781631	0.059701384	57	16	79
Bilirubin	0.2-1.5 mg/dL	Standard	early	0.575	0.070156076	57	16	79
Bilirubin	0.2-1.5 mg/dL	Standard	mid	0.4875	0.041221581	57	16	79
Bilirubin	0.2-1.5 mg/dL	Standard	late	0.55	0.066143783	57	16	79
Bone Specific Alkaline Phosphatase	Males: 15.0-41.3 U/L Females: 11.6-29.6 U/L	Enhanced	pre	27.14375	2.794420187	58	16	80
Bone Specific Alkaline Phosphatase	Males: 15.0-41.3 U/L Females: 11.6-29.6 U/L	Enhanced	early	24.4375	1.849234428	58	16	80
Bone Specific Alkaline Phosphatase	Males: 15.0-41.3 U/L Females: 11.6-29.6 U/L	Enhanced	mid	24.425	1.782620087	58	16	80
Bone Specific Alkaline Phosphatase	Males: 15.0-41.3 U/L Females: 11.6-29.6 U/L	Enhanced	late	26.175	2.161723126	58	16	80
Bone Specific Alkaline Phosphatase	Males: 15.0-41.3 U/L Females: 11.6-29.6 U/L	Standard	pre	21.65	1.536686777	58	16	80
Bone Specific Alkaline Phosphatase	Males: 15.0-41.3 U/L Females: 11.6-29.6 U/L	Standard	early	21.125	1.443601183	58	16	80
Bone Specific Alkaline Phosphatase	Males: 15.0-41.3 U/L Females: 11.6-29.6 U/L	Standard	mid	20.8375	1.595397433	58	16	80
Bone Specific Alkaline Phosphatase	Males: 15.0-41.3 U/L Females: 11.6-29.6 U/L	Standard	late	22.05	1.743201078	58	16	80
C-terminal telopeptides of type I collagen (CTX beta)	Males: 0.115-0.748 Females: 0.112-0.738 ng/mL	Enhanced	pre	0.5846875	0.071826991	58	16	80
C-terminal telopeptides of type I collagen (CTX beta)	Males: 0.115-0.748 Females: 0.112-0.738 ng/mL	Enhanced	early	0.658	0.112649761	58	16	80
C-terminal telopeptides of type I collagen (CTX beta)	Males: 0.115-0.748 Females: 0.112-0.738 ng/mL	Enhanced	mid	0.838625	0.143376791	58	16	80
C-terminal telopeptides of type I collagen (CTX beta)	Males: 0.115-0.748 Females: 0.112-0.738 ng/mL	Enhanced	late	0.819375	0.138207378	58	16	80
C-terminal telopeptides of type I collagen (CTX beta)	Males: 0.115-0.748 Females: 0.112-0.738 ng/mL	Standard	pre	0.5339375	0.066032984	58	16	80
C-terminal telopeptides of type I collagen (CTX beta)	Males: 0.115-0.748 Females: 0.112-0.738 ng/mL	Standard	early	0.612625	0.060218077	58	16	80
C-terminal telopeptides of type I collagen (CTX beta)	Males: 0.115-0.748 Females: 0.112-0.738 ng/mL	Standard	mid	0.74425	0.088516727	58	16	80
C-terminal telopeptides of type I collagen (CTX beta)	Males: 0.115-0.748 Females: 0.112-0.738 ng/mL	Standard	late	0.73	0.079510023	58	16	80
Calcium	2.15- 2.50 mmol/L	Enhanced	pre	2.456875	0.017609867	58	16	80
Calcium	2.15- 2.50 mmol/L	Enhanced	early	2.42125	0.024137464	58	16	80
Calcium	2.15- 2.50 mmol/L	Enhanced	mid	2.425	0.02298097	58	16	80
Calcium	2.15- 2.50 mmol/L	Enhanced	late	2.46625	0.016577234	58	16	80
Calcium	2.15- 2.50 mmol/L	Standard	pre	2.414375	0.023375961	58	16	80
Calcium	2.15- 2.50 mmol/L	Standard	early	2.3975	0.036986062	58	16	80
Calcium	2.15- 2.50 mmol/L	Standard	mid	2.41625	0.031071364	58	16	80
Calcium	2.15- 2.50 mmol/L	Standard	late	2.38625	0.031271867	58	16	80
Calcium, Ionized	1.12-1.32 mmol/L	Enhanced	pre	1.259519142	0.017993646	38	14	58
Calcium, Ionized	1.12-1.32 mmol/L	Enhanced	early	1.23009789	0.029174229	38	14	58
Calcium, Ionized	1.12-1.32 mmol/L	Enhanced	mid	1.263800004	0.012877644	38	14	58
Calcium, Ionized	1.12-1.32 mmol/L	Enhanced	late	1.261300004	0.022606404	38	14	58
Calcium, Ionized	1.12-1.32 mmol/L	Standard	pre	1.26375	0.008466386	38	14	58
Calcium, Ionized	1.12-1.32 mmol/L	Standard	early	1.255	0.006373774	38	14	58
Calcium, Ionized	1.12-1.32 mmol/L	Standard	mid	1.263557474	0.006622916	38	14	58
Calcium, Ionized	1.12-1.32 mmol/L	Standard	late	1.268170086	0.007159322	38	14	58
Carboxyethyllysine (advanced glycation end product)	0.05- 0.40 umol/L	Enhanced	pre	0.1	0.0121192	26	8	40
Carboxyethyllysine (advanced glycation end product)	0.05- 0.40 umol/L	Enhanced	early	0.1975	0.030695073	26	8	40
Carboxyethyllysine (advanced glycation end product)	0.05- 0.40 umol/L	Enhanced	mid	0.15	0.012747549	26	8	40
Carboxyethyllysine (advanced glycation end product)	0.05- 0.40 umol/L	Enhanced	late	0.24	0.015411035	26	8	40
Carboxyethyllysine (advanced glycation end product)	0.05- 0.40 umol/L	Standard	pre	0.135	0.034820971	26	8	40
Carboxyethyllysine (advanced glycation end product)	0.05- 0.40 umol/L	Standard	early	0.205	0.025124689	26	8	40
Carboxyethyllysine (advanced glycation end product)	0.05- 0.40 umol/L	Standard	mid	0.2725	0.029448047	26	8	40
Carboxyethyllysine (advanced glycation end product)	0.05- 0.40 umol/L	Standard	late	0.1725	0.016345871	26	8	40
Carboxymethyllysine (advanced glycation end product)	0.05- 0.30 umol/L	Enhanced	pre	0.08875	0.005694021	26	8	40
Carboxymethyllysine (advanced glycation end product)	0.05- 0.30 umol/L	Enhanced	early	0.11	0.012747549	26	8	40
Carboxymethyllysine (advanced glycation end product)	0.05- 0.30 umol/L	Enhanced	mid	0.11	0.010606602	26	8	40
Carboxymethyllysine (advanced glycation end product)	0.05- 0.30 umol/L	Enhanced	late	0.16	0.01118034	26	8	40
Carboxymethyllysine (advanced glycation end product)	0.05- 0.30 umol/L	Standard	pre	0.12625	0.022456555	26	8	40
Carboxymethyllysine (advanced glycation end product)	0.05- 0.30 umol/L	Standard	early	0.1275	0.012437343	26	8	40
Carboxymethyllysine (advanced glycation end product)	0.05- 0.30 umol/L	Standard	mid	0.1925	0.014737283	26	8	40

Carboxymethyllysine (advanced glycation end product)	0.05- 0.30 umol/L	Standard	late	0.1025	0.0073951	26	8	40
Catechin, plasma	ng/mL	Enhanced	pre	7.736850394	0.758831364	1	4	6
Catechin, plasma	ng/mL	Enhanced	early	3.11	0.898025612	1	4	6
Catechin, plasma	ng/mL	Standard	pre	1.95	#N/A	1	4	6
Catechin, plasma	ng/mL	Standard	late	1.31	#N/A	1	4	6
Ceruloplasmin	20-60 mg/dL	Enhanced	pre	21.875	0.91322745	58	16	80
Ceruloplasmin	20-60 mg/dL	Enhanced	early	21.75	0.605959982	58	16	80
Ceruloplasmin	20-60 mg/dL	Enhanced	mid	20.875	0.942693946	58	16	80
Ceruloplasmin	20-60 mg/dL	Enhanced	late	20.75	1.14222918	58	16	80
Ceruloplasmin	20-60 mg/dL	Standard	pre	23.0625	0.961921498	58	16	80
Ceruloplasmin	20-60 mg/dL	Standard	early	22.25	0.65550553	58	16	80
Ceruloplasmin	20-60 mg/dL	Standard	mid	22.5	0.968245837	58	16	80
Ceruloplasmin	20-60 mg/dL	Standard	late	22.375	1.05973906	58	16	80
Chloride	99-108 mmol/L	Enhanced	pre	106.5	1.411448724	58	16	80
Chloride	99-108 mmol/L	Enhanced	early	106.625	1.223947252	58	16	80
Chloride	99-108 mmol/L	Enhanced	mid	106	0.883883476	58	16	80
Chloride	99-108 mmol/L	Enhanced	late	106.75	1.169267933	58	16	80
Chloride	99-108 mmol/L	Standard	pre	108.6875	1.06410724	58	16	80
Chloride	99-108 mmol/L	Standard	early	106.625	1.117160183	58	16	80
Chloride	99-108 mmol/L	Standard	mid	106.5	1.237436867	58	16	80
Chloride	99-108 mmol/L	Standard	late	105.875	1.191604748	58	16	80
Cholesterol	125-199 mg/dL	Enhanced	pre	192.6875	10.10025429	58	16	80
Cholesterol	125-199 mg/dL	Enhanced	early	177.5	11.26665434	58	16	80
Cholesterol	125-199 mg/dL	Enhanced	mid	156.25	8.266555359	58	16	80
Cholesterol	125-199 mg/dL	Enhanced	late	156.125	10.07850048	58	16	80
Cholesterol	125-199 mg/dL	Standard	pre	181.5625	12.3194579	58	16	80
Cholesterol	125-199 mg/dL	Standard	early	175	13.55083023	58	16	80
Cholesterol	125-199 mg/dL	Standard	mid	166.625	13.07662215	58	16	80
Cholesterol	125-199 mg/dL	Standard	late	164.625	14.12880203	58	16	80
Copper	Males 700-1400; Females 800-1550 ng/mL	Enhanced	pre	880.25	42.91333927	58	16	80
Copper	Males 700-1400; Females 800-1550 ng/mL	Enhanced	early	853.25	46.55096334	58	16	80
Copper	Males 700-1400; Females 800-1550 ng/mL	Enhanced	mid	789.625	49.72202024	58	16	80
Copper	Males 700-1400; Females 800-1550 ng/mL	Enhanced	late	785.5	46.23276706	58	16	80
Copper	Males 700-1400; Females 800-1550 ng/mL	Standard	pre	916.625	32.91392064	58	16	80
Copper	Males 700-1400; Females 800-1550 ng/mL	Standard	early	898	27.65298356	58	16	80
Copper	Males 700-1400; Females 800-1550 ng/mL	Standard	mid	874.875	37.78162479	58	16	80
Copper	Males 700-1400; Females 800-1550 ng/mL	Standard	late	879.625	33.34710328	58	16	80
Cortisol, Total	5-21 ug/dL	Enhanced	pre	10.01875	0.677134213	58	16	80
Cortisol, Total	5-21 ug/dL	Enhanced	early	16.8375	0.856398254	58	16	80
Cortisol, Total	5-21 ug/dL	Enhanced	mid	15.6625	0.410768145	58	16	80
Cortisol, Total	5-21 ug/dL	Enhanced	late	14.45	0.783421981	58	16	80
Cortisol, Total	5-21 ug/dL	Standard	pre	9.425	0.808934484	58	16	80
Cortisol, Total	5-21 ug/dL	Standard	early	17.6625	1.126795442	58	16	80
Cortisol, Total	5-21 ug/dL	Standard	mid	17.3625	0.926677111	58	16	80
Cortisol, Total	5-21 ug/dL	Standard	late	16.8125	0.524236796	58	16	80
Cotinine	0-85 nmol/L	Enhanced	pre	0.271875	0.254315776	58	16	80
Cotinine	0-85 nmol/L	Enhanced	early	4.93667E-16	#N/A	#N/A	16	80
Cotinine	0-85 nmol/L	Enhanced	mid	3.1417E-15	#N/A	#N/A	16	80
Cotinine	0-85 nmol/L	Enhanced	late	4.93667E-16	#N/A	#N/A	16	80
Cotinine	0-85 nmol/L	Standard	pre	33.15375	28.15964638	58	16	80
Cotinine	0-85 nmol/L	Standard	early	2.325	2.174838356	58	16	80
Cotinine	0-85 nmol/L	Standard	mid	1.02327E-15	#N/A	58	16	80
Cotinine	0-85 nmol/L	Standard	late	-1.92342E-14	#N/A	58	16	80
Creatinine, Serum	Males: 0.64-1.42 Females: 0.53-1.08 mg/dL	Enhanced	pre	1	0.063121906	58	16	80
Creatinine, Serum	Males: 0.64-1.42 Females: 0.53-1.08 mg/dL	Enhanced	early	0.9875	0.05978477	58	16	80
Creatinine, Serum	Males: 0.64-1.42 Females: 0.53-1.08 mg/dL	Enhanced	mid	0.975	0.065550553	58	16	80
Creatinine, Serum	Males: 0.64-1.42 Females: 0.53-1.08 mg/dL	Enhanced	late	1.0375	0.058463397	58	16	80
Creatinine, Serum	Males: 0.64-1.42 Females: 0.53-1.08 mg/dL	Standard	pre	0.88125	0.044138897	58	16	80
Creatinine, Serum	Males: 0.64-1.42 Females: 0.53-1.08 mg/dL	Standard	early	0.85	0.035355339	58	16	80
Creatinine, Serum	Males: 0.64-1.42 Females: 0.53-1.08 mg/dL	Standard	mid	0.9375	0.046561451	58	16	80
Creatinine, Serum	Males: 0.64-1.42 Females: 0.53-1.08 mg/dL	Standard	late	0.9	0.04330127	58	16	80
Cystathionine	0.07-0.55 umol/L	Enhanced	pre	0.153125	0.011002042	58	16	80
Cystathionine	0.07-0.55 umol/L	Enhanced	early	0.13625	0.014570679	58	16	80
Cystathionine	0.07-0.55 umol/L	Enhanced	mid	0.27	0.031770663	58	16	80
Cystathionine	0.07-0.55 umol/L	Enhanced	late	0.21375	0.023844385	58	16	80
Cystathionine	0.07-0.55 umol/L	Standard	pre	0.465625	0.17012504	58	16	80
Cystathionine	0.07-0.55 umol/L	Standard	early	0.265	0.038283482	58	16	80
Cystathionine	0.07-0.55 umol/L	Standard	mid	0.3375	0.036475591	58	16	80
Cystathionine	0.07-0.55 umol/L	Standard	late	0.23	0.017589059	58	16	80
Cysteine, Total	150-350 umol/L	Enhanced	pre	295.5625	7.541260204	58	16	80
Cysteine, Total	150-350 umol/L	Enhanced	early	280	6.997767501	58	16	80
Cysteine, Total	150-350 umol/L	Enhanced	mid	272.75	7.063882608	58	16	80
Cysteine, Total	150-350 umol/L	Enhanced	late	269.5	6.11350963	58	16	80
Cysteine, Total	150-350 umol/L	Standard	pre	291.375	7.881817486	58	16	80
Cysteine, Total	150-350 umol/L	Standard	early	291.125	9.93799134	58	16	80
Cysteine, Total	150-350 umol/L	Standard	mid	275.25	9.197061351	58	16	80
Cysteine, Total	150-350 umol/L	Standard	late	280.75	10.11303305	58	16	80
DHEA	Males: 0.52-5.18 Females: 0.36-7.82 ug/L	Enhanced	pre	3.265	1.125530431	26	8	40
DHEA	Males: 0.52-5.18 Females: 0.36-7.82 ug/L	Enhanced	early	4.4375	0.667714151	26	8	40
DHEA	Males: 0.52-5.18 Females: 0.36-7.82 ug/L	Enhanced	mid	5.765	1.345908151	26	8	40
DHEA	Males: 0.52-5.18 Females: 0.36-7.82 ug/L	Enhanced	late	4.89	1.470446361	26	8	40
DHEA	Males: 0.52-5.18 Females: 0.36-7.82 ug/L	Standard	pre	3.39875	0.748576514	26	8	40
DHEA	Males: 0.52-5.18 Females: 0.36-7.82 ug/L	Standard	early	7.355	1.517602962	26	8	40

DHEA	Males: 0.52-5.18 Females: 0.36-7.82 ug/L	Standard	mid	7.08	1.713895417	26	8	40
DHEA	Males: 0.52-5.18 Females: 0.36-7.82 ug/L	Standard	late	6.245	1.425282867	26	8	40
DHEA-S	Males: 0.73-3.81 Females: 0.37-2.71 ug/mL	Enhanced	pre	1.545625	0.138725892	58	16	80
DHEA-S	Males: 0.73-3.81 Females: 0.37-2.71 ug/mL	Enhanced	early	1.67375	0.193188198	58	16	80
DHEA-S	Males: 0.73-3.81 Females: 0.37-2.71 ug/mL	Enhanced	mid	1.7775	0.202837296	58	16	80
DHEA-S	Males: 0.73-3.81 Females: 0.37-2.71 ug/mL	Enhanced	late	1.8125	0.219955606	58	16	80
DHEA-S	Males: 0.73-3.81 Females: 0.37-2.71 ug/mL	Standard	pre	1.665	0.242665858	58	16	80
DHEA-S	Males: 0.73-3.81 Females: 0.37-2.71 ug/mL	Standard	early	1.67125	0.233983423	58	16	80
DHEA-S	Males: 0.73-3.81 Females: 0.37-2.71 ug/mL	Standard	mid	1.8775	0.311226154	58	16	80
DHEA-S	Males: 0.73-3.81 Females: 0.37-2.71 ug/mL	Standard	late	1.78125	0.288045339	58	16	80
Daidzein, plasma	ng/mL	Enhanced	pre	11.7	5.444824331	7	9	21
Daidzein, plasma	ng/mL	Enhanced	mid	3.68	1.192839749	7	9	21
Daidzein, plasma	ng/mL	Enhanced	late	1.18	#N/A	7	9	21
Daidzein, plasma	ng/mL	Standard	pre	4.741666667	1.553362194	7	9	21
Daidzein, plasma	ng/mL	Standard	early	1.07	0	7	9	21
Daidzein, plasma	ng/mL	Standard	mid	2.695	0.088388348	7	9	21
Daidzein, plasma	ng/mL	Standard	late	2.663333333	1.097323343	7	9	21
Epicatechin, plasma	ng/mL	Enhanced	pre	1.62	#N/A	1	5	6
Epicatechin, plasma	ng/mL	Enhanced	early	1.36	0.22627417	1	5	6
Epicatechin, plasma	ng/mL	Enhanced	mid	1.2	0	1	5	6
Epicatechin, plasma	ng/mL	Standard	pre	3.68	#N/A	1	5	6
Ferritin	Males: 18-464 Females: 20-137 ng/mL	Enhanced	pre	65.0625	16.50862405	58	16	80
Ferritin	Males: 18-464 Females: 20-137 ng/mL	Enhanced	early	66.5	13.44897766	58	16	80
Ferritin	Males: 18-464 Females: 20-137 ng/mL	Enhanced	mid	40.75	8.351178809	58	16	80
Ferritin	Males: 18-464 Females: 20-137 ng/mL	Enhanced	late	36.375	7.348336334	58	16	80
Ferritin	Males: 18-464 Females: 20-137 ng/mL	Standard	pre	76.5625	18.0380322	58	16	80
Ferritin	Males: 18-464 Females: 20-137 ng/mL	Standard	early	76	17.04314378	58	16	80
Ferritin	Males: 18-464 Females: 20-137 ng/mL	Standard	mid	58.625	14.26965476	58	16	80
Ferritin	Males: 18-464 Females: 20-137 ng/mL	Standard	late	54.375	14.78590704	58	16	80
Flavin mononucleotide	5-29 nmol/L	Enhanced	pre	11.003125	1.816057487	58	16	80
Flavin mononucleotide	5-29 nmol/L	Enhanced	early	12.605	2.088980014	58	16	80
Flavin mononucleotide	5-29 nmol/L	Enhanced	mid	11.45125	1.867122148	58	16	80
Flavin mononucleotide	5-29 nmol/L	Enhanced	late	10.12625	1.997771366	58	16	80
Flavin mononucleotide	5-29 nmol/L	Standard	pre	11.223125	2.572482832	58	16	80
Flavin mononucleotide	5-29 nmol/L	Standard	early	13.805	3.132508978	58	16	80
Flavin mononucleotide	5-29 nmol/L	Standard	mid	12.28375	2.902814872	58	16	80
Flavin mononucleotide	5-29 nmol/L	Standard	late	11.4375	2.521269986	58	16	80
Folate, RBC	288-867 ng/mL	Enhanced	pre	532.6475	53.75102056	31	14	51
Folate, RBC	288-867 ng/mL	Enhanced	early	452.1391741	25.38387668	31	14	51
Folate, RBC	288-867 ng/mL	Enhanced	mid	487.1766741	34.35692488	31	14	51
Folate, RBC	288-867 ng/mL	Enhanced	late	515.9741741	36.03228006	31	14	51
Folate, RBC	288-867 ng/mL	Standard	pre	697.3853861	144.1711583	31	14	51
Folate, RBC	288-867 ng/mL	Standard	early	662.2990528	176.0668761	31	14	51
Folate, RBC	288-867 ng/mL	Standard	mid	654.1312601	153.9777443	31	14	51
Folate, RBC	288-867 ng/mL	Standard	late	581.4144924	149.9524629	31	14	51
Folate, serum	21-64 nmol/L	Enhanced	pre	25.27125	4.187597643	58	16	80
Folate, serum	21-64 nmol/L	Enhanced	early	35.9525	4.638656443	58	16	80
Folate, serum	21-64 nmol/L	Enhanced	mid	24.16625	2.852999484	58	16	80
Folate, serum	21-64 nmol/L	Enhanced	late	23.1025	2.229194607	58	16	80
Folate, serum	21-64 nmol/L	Standard	pre	27.816875	2.868001392	58	16	80
Folate, serum	21-64 nmol/L	Standard	early	33.58625	2.719359379	58	16	80
Folate, serum	21-64 nmol/L	Standard	mid	24.14875	1.877702584	58	16	80
Folate, serum	21-64 nmol/L	Standard	late	21.02	1.507452321	58	16	80
Gamma-tocopherol	2-11 umol/L	Enhanced	pre	3.2425	0.152553782	58	16	80
Gamma-tocopherol	2-11 umol/L	Enhanced	early	2.44125	0.116825691	58	16	80
Gamma-tocopherol	2-11 umol/L	Enhanced	mid	2.16625	0.073525878	58	16	80
Gamma-tocopherol	2-11 umol/L	Enhanced	late	1.8575	0.060097577	58	16	80
Gamma-tocopherol	2-11 umol/L	Standard	pre	4.006875	0.364790112	58	16	80
Gamma-tocopherol	2-11 umol/L	Standard	early	2.6375	0.294786734	58	16	80
Gamma-tocopherol	2-11 umol/L	Standard	mid	2.3125	0.285075374	58	16	80
Gamma-tocopherol	2-11 umol/L	Standard	late	2.25375	0.266639606	58	16	80
Genistein, plasma	ng/mL	Enhanced	pre	17.248	6.741392901	12	11	29
Genistein, plasma	ng/mL	Enhanced	early	4.32	#N/A	12	11	29
Genistein, plasma	ng/mL	Enhanced	mid	9.945	3.975942498	12	11	29
Genistein, plasma	ng/mL	Enhanced	late	3.11	#N/A	12	11	29
Genistein, plasma	ng/mL	Standard	pre	17.05714286	6.327067318	12	11	29
Genistein, plasma	ng/mL	Standard	early	2.71	0	12	11	29
Genistein, plasma	ng/mL	Standard	mid	7.15	2.171855326	12	11	29
Genistein, plasma	ng/mL	Standard	late	3.525	0.73096255	12	11	29
Glutamic Acid	20-140 umol/L	Enhanced	pre	52.386875	4.087662261	58	16	80
Glutamic Acid	20-140 umol/L	Enhanced	early	53.31	3.967977287	58	16	80
Glutamic Acid	20-140 umol/L	Enhanced	mid	45.11875	3.056719768	58	16	80
Glutamic Acid	20-140 umol/L	Enhanced	late	45.2375	4.344152949	58	16	80
Glutamic Acid	20-140 umol/L	Standard	pre	56.72625	5.594761972	58	16	80
Glutamic Acid	20-140 umol/L	Standard	early	56.9975	3.633166985	58	16	80
Glutamic Acid	20-140 umol/L	Standard	mid	53.19875	3.182086303	58	16	80
Glutamic Acid	20-140 umol/L	Standard	late	49.02625	4.429269458	58	16	80
Glutamine	390-700 umol/L	Enhanced	pre	537.6875	8.751757636	58	16	80
Glutamine	390-700 umol/L	Enhanced	early	545.75	25.2498453	58	16	80
Glutamine	390-700 umol/L	Enhanced	mid	557.625	15.02595411	58	16	80
Glutamine	390-700 umol/L	Enhanced	late	564.75	13.50781024	58	16	80
Glutamine	390-700 umol/L	Standard	pre	557.4375	12.2813494	58	16	80
Glutamine	390-700 umol/L	Standard	early	573.625	13.52074506	58	16	80

Glutamine		390-700 umol/L	Standard	mid	591.125	15.53065265	58	16	80
Glutamine		390-700 umol/L	Standard	late	584	15.59146401	58	16	80
Glutathione Peroxidase		27.5-73.6 U/G Hgb	Enhanced	pre	42.35416667	1.961247625	37	14	57
Glutathione Peroxidase		27.5-73.6 U/G Hgb	Enhanced	early	50.98382738	6.314702027	37	14	57
Glutathione Peroxidase		27.5-73.6 U/G Hgb	Enhanced	mid	49.16632738	2.820590719	37	14	57
Glutathione Peroxidase		27.5-73.6 U/G Hgb	Enhanced	late	50.24382738	2.357992338	37	14	57
Glutathione Peroxidase		27.5-73.6 U/G Hgb	Standard	pre	59.445	3.134507039	37	14	57
Glutathione Peroxidase		27.5-73.6 U/G Hgb	Standard	early	63.27784106	3.639911967	37	14	57
Glutathione Peroxidase		27.5-73.6 U/G Hgb	Standard	mid	66.98529255	4.287544495	37	14	57
Glutathione Peroxidase		27.5-73.6 U/G Hgb	Standard	late	71.92820095	8.156352103	37	14	57
Glutathione, Reduced		639-1146 umol/L	Enhanced	pre	759.6563718	61.93693229	25	8	39
Glutathione, Reduced		639-1146 umol/L	Enhanced	early	725.5	51.00306363	25	8	39
Glutathione, Reduced		639-1146 umol/L	Enhanced	mid	731.5	58.50694403	25	8	39
Glutathione, Reduced		639-1146 umol/L	Enhanced	late	733.25	48.73829988	25	8	39
Glutathione, Reduced		639-1146 umol/L	Standard	pre	928.75	72.96156951	25	8	39
Glutathione, Reduced		639-1146 umol/L	Standard	early	840.75	80.62751934	25	8	39
Glutathione, Reduced		639-1146 umol/L	Standard	mid	807	52.52975347	25	8	39
Glutathione, Reduced		639-1146 umol/L	Standard	late	807.25	69.85017448	25	8	39
Glutathione, Oxidized	Calculated	umol/L	Enhanced	pre	245.6734467	20.96606574	25	8	39
Glutathione, Oxidized	Calculated	umol/L	Enhanced	early	216.25	22.37360331	25	8	39
Glutathione, Oxidized	Calculated	umol/L	Enhanced	mid	203.75	15.08362108	25	8	39
Glutathione, Oxidized	Calculated	umol/L	Enhanced	late	186.875	12.72960992	25	8	39
Glutathione, Oxidized	Calculated	umol/L	Standard	pre	241.6875	42.33832991	25	8	39
Glutathione, Oxidized	Calculated	umol/L	Standard	early	239.5	40.46410446	25	8	39
Glutathione, Oxidized	Calculated	umol/L	Standard	mid	192.875	31.73689444	25	8	39
Glutathione, Oxidized	Calculated	umol/L	Standard	late	200	33.72916023	25	8	39
Glutathione, Total		783-1346 umol/L	Enhanced	pre	1250.896797	87.21451794	25	8	39
Glutathione, Total		783-1346 umol/L	Enhanced	early	1158	71.80268101	25	8	39
Glutathione, Total		783-1346 umol/L	Enhanced	mid	1139	82.70202537	25	8	39
Glutathione, Total		783-1346 umol/L	Enhanced	late	1107	59.02647711	25	8	39
Glutathione, Total		783-1346 umol/L	Standard	pre	1412.125	135.0028212	25	8	39
Glutathione, Total		783-1346 umol/L	Standard	early	1319.75	130.1640191	25	8	39
Glutathione, Total		783-1346 umol/L	Standard	mid	1192.75	94.88636296	25	8	39
Glutathione, Total		783-1346 umol/L	Standard	late	1207.25	74.78082558	25	8	39
Glutathione, Total Ratio	Calculated		Enhanced	pre	3.152651703	0.314632047	25	8	39
Glutathione, Total Ratio	Calculated		Enhanced	early	3.485	0.443008183	25	8	39
Glutathione, Total Ratio	Calculated		Enhanced	mid	3.6025	0.222749158	25	8	39
Glutathione, Total Ratio	Calculated		Enhanced	late	3.9825	0.364475222	25	8	39
Glutathione, Total Ratio	Calculated		Standard	pre	4.7725	1.073588463	25	8	39
Glutathione, Total Ratio	Calculated		Standard	early	3.875	0.663310825	25	8	39
Glutathione, Total Ratio	Calculated		Standard	mid	4.59	0.716266361	25	8	39
Glutathione, Total Ratio	Calculated		Standard	late	4.6475	1.009154194	25	8	39
Glycine		150-300 umol/L	Enhanced	pre	324.8125	24.92017529	58	16	80
Glycine		150-300 umol/L	Enhanced	early	295	24.4316649	58	16	80
Glycine		150-300 umol/L	Enhanced	mid	299.5	21.7492816	58	16	80
Glycine		150-300 umol/L	Enhanced	late	306.875	24.27990263	58	16	80
Glycine		150-300 umol/L	Standard	pre	312.4375	22.20702266	58	16	80
Glycine		150-300 umol/L	Standard	early	308.375	18.59598201	58	16	80
Glycine		150-300 umol/L	Standard	mid	304.125	19.17716538	58	16	80
Glycine		150-300 umol/L	Standard	late	299.625	21.14971742	58	16	80
HDL Cholesterol		Males: 40-70 Females: 40-80 mg/dL	Enhanced	pre	62.5625	3.783638192	58	16	80
HDL Cholesterol		Males: 40-70 Females: 40-80 mg/dL	Enhanced	early	54.375	4.160594534	58	16	80
HDL Cholesterol		Males: 40-70 Females: 40-80 mg/dL	Enhanced	mid	49.875	2.775098534	58	16	80
HDL Cholesterol		Males: 40-70 Females: 40-80 mg/dL	Enhanced	late	48.875	2.400764852	58	16	80
HDL Cholesterol		Males: 40-70 Females: 40-80 mg/dL	Standard	pre	65.1875	2.366658872	58	16	80
HDL Cholesterol		Males: 40-70 Females: 40-80 mg/dL	Standard	early	61.25	2.013625462	58	16	80
HDL Cholesterol		Males: 40-70 Females: 40-80 mg/dL	Standard	mid	53.625	2.888216902	58	16	80
HDL Cholesterol		Males: 40-70 Females: 40-80 mg/dL	Standard	late	54.375	3.472829808	58	16	80
Hematocrit		Males: 38.6-49.1 Females: 34.5-43.6 %	Enhanced	pre	41.64166667	1.865007323	37	14	57
Hematocrit		Males: 38.6-49.1 Females: 34.5-43.6 %	Enhanced	early	38.95130307	1.513243924	37	14	57
Hematocrit		Males: 38.6-49.1 Females: 34.5-43.6 %	Enhanced	mid	39.20130307	1.437816878	37	14	57
Hematocrit		Males: 38.6-49.1 Females: 34.5-43.6 %	Enhanced	late	38.25130307	1.392660814	37	14	57
Hematocrit		Males: 38.6-49.1 Females: 34.5-43.6 %	Standard	pre	42.525	1.535517014	37	14	57
Hematocrit		Males: 38.6-49.1 Females: 34.5-43.6 %	Standard	early	40.26601885	1.461128338	37	14	57
Hematocrit		Males: 38.6-49.1 Females: 34.5-43.6 %	Standard	mid	37.85806538	1.571589838	37	14	57
Hematocrit		Males: 38.6-49.1 Females: 34.5-43.6 %	Standard	late	39.25705952	1.284873658	37	14	57
Heme		umol/L	Enhanced	pre	28.225	1.787827767	58	16	80
Heme		umol/L	Enhanced	early	28.475	1.823436831	58	16	80
Heme		umol/L	Enhanced	mid	23.75	1.019803903	58	16	80
Heme		umol/L	Enhanced	late	28.9125	2.912121086	58	16	80
Heme		umol/L	Standard	pre	31.73125	2.028333242	58	16	80
Heme		umol/L	Standard	early	26.2	1.870494587	58	16	80
Heme		umol/L	Standard	mid	25.175	2.126745607	58	16	80
Heme		umol/L	Standard	late	23.375	1.200488182	58	16	80
Hemoglobin		Males: 12.9-16.7 Females: 11.4-14.6 g/dL	Enhanced	pre	14.20833333	0.640357611	37	14	57
Hemoglobin		Males: 12.9-16.7 Females: 11.4-14.6 g/dL	Enhanced	early	13.35821133	0.49891816	37	14	57
Hemoglobin		Males: 12.9-16.7 Females: 11.4-14.6 g/dL	Enhanced	mid	13.43321133	0.456347525	37	14	57
Hemoglobin		Males: 12.9-16.7 Females: 11.4-14.6 g/dL	Enhanced	late	13.15821133	0.491503777	37	14	57
Hemoglobin		Males: 12.9-16.7 Females: 11.4-14.6 g/dL	Standard	pre	14.6	0.56256944	37	14	57
Hemoglobin		Males: 12.9-16.7 Females: 11.4-14.6 g/dL	Standard	early	13.98275533	0.548237237	37	14	57
Hemoglobin		Males: 12.9-16.7 Females: 11.4-14.6 g/dL	Standard	mid	13.15921558	0.573030167	37	14	57
Hemoglobin		Males: 12.9-16.7 Females: 11.4-14.6 g/dL	Standard	late	13.43512752	0.477876679	37	14	57
Hepcidin-25 Bioactive		1.0-39.3 ng/mL	Enhanced	pre	15.43125	3.007905437	58	16	80

Hepcidin-25 Bioactive	1.0-39.3 ng/mL	Enhanced	early	12.1875	2.454233428	58	16	80
Hepcidin-25 Bioactive	1.0-39.3 ng/mL	Enhanced	mid	7.975	1.722983423	58	16	80
Hepcidin-25 Bioactive	1.0-39.3 ng/mL	Enhanced	late	7.1625	1.367015168	58	16	80
Hepcidin-25 Bioactive	1.0-39.3 ng/mL	Standard	pre	14.56875	3.099885803	58	16	80
Hepcidin-25 Bioactive	1.0-39.3 ng/mL	Standard	early	17.0875	3.916608765	58	16	80
Hepcidin-25 Bioactive	1.0-39.3 ng/mL	Standard	mid	11.675	2.020887027	58	16	80
Hepcidin-25 Bioactive	1.0-39.3 ng/mL	Standard	late	11.1875	2.534723697	58	16	80
Hesperetin, plasma	ng/mL	Enhanced	pre	86.3	0	2	11	19
Hesperetin, plasma	ng/mL	Enhanced	early	4.142	1.160969939	2	11	19
Hesperetin, plasma	ng/mL	Enhanced	mid	5.53	#N/A	2	11	19
Hesperetin, plasma	ng/mL	Enhanced	late	3.28	#N/A	2	11	19
Hesperetin, plasma	ng/mL	Standard	pre	2.3225	0.387949336	2	11	19
Hesperetin, plasma	ng/mL	Standard	early	5.483333333	1.509468879	2	11	19
Hesperetin, plasma	ng/mL	Standard	mid	2.16	0	2	11	19
Hesperetin, plasma	ng/mL	Standard	late	1.806666667	0.340500721	2	11	19
High Sensitivity C-Reactive Protein (hsCRP)	0.0-3.0 mg/L	Enhanced	pre	0.541921183	0.113518661	41	15	62
High Sensitivity C-Reactive Protein (hsCRP)	0.0-3.0 mg/L	Enhanced	early	0.633584913	0.157506173	41	15	62
High Sensitivity C-Reactive Protein (hsCRP)	0.0-3.0 mg/L	Enhanced	mid	0.653798978	0.233406531	41	15	62
High Sensitivity C-Reactive Protein (hsCRP)	0.0-3.0 mg/L	Enhanced	late	0.413798978	0.104560759	41	15	62
High Sensitivity C-Reactive Protein (hsCRP)	0.0-3.0 mg/L	Standard	pre	0.572362225	0.090045012	41	15	62
High Sensitivity C-Reactive Protein (hsCRP)	0.0-3.0 mg/L	Standard	early	0.483451487	0.114451613	41	15	62
High Sensitivity C-Reactive Protein (hsCRP)	0.0-3.0 mg/L	Standard	mid	0.33678482	0.057820461	41	15	62
High Sensitivity C-Reactive Protein (hsCRP)	0.0-3.0 mg/L	Standard	late	0.38178482	0.079554779	41	15	62
Histidine	40-200 umol/L	Enhanced	pre	88.859375	3.190305069	58	16	80
Histidine	40-200 umol/L	Enhanced	early	86.68128698	3.418305502	58	16	80
Histidine	40-200 umol/L	Enhanced	mid	90.13602653	3.839179987	58	16	80
Histidine	40-200 umol/L	Enhanced	late	93.52707024	4.1930048	58	16	80
Histidine	40-200 umol/L	Standard	pre	89.025625	2.656808535	58	16	80
Histidine	40-200 umol/L	Standard	early	90.245	2.764606098	58	16	80
Histidine	40-200 umol/L	Standard	mid	91.6125	3.280438114	58	16	80
Histidine	40-200 umol/L	Standard	late	91.53375	2.607650965	58	16	80
Homocysteine, Total	5-15 umol/L	Enhanced	pre	9.5125	0.507426873	58	16	80
Homocysteine, Total	5-15 umol/L	Enhanced	early	9.0125	0.474413289	58	16	80
Homocysteine, Total	5-15 umol/L	Enhanced	mid	9.135	0.56892717	58	16	80
Homocysteine, Total	5-15 umol/L	Enhanced	late	8.92875	0.548653003	58	16	80
Homocysteine, Total	5-15 umol/L	Standard	pre	8.745625	0.832686464	58	16	80
Homocysteine, Total	5-15 umol/L	Standard	early	8.47375	0.733018028	58	16	80
Homocysteine, Total	5-15 umol/L	Standard	mid	8.25875	0.715774138	58	16	80
Homocysteine, Total	5-15 umol/L	Standard	late	8.4125	0.677084167	58	16	80
IgF-1	ng/mL	Enhanced	pre	196.64375	17.8889291	58	16	80
IgF-1	ng/mL	Enhanced	early	179.125	17.50171197	58	16	80
IgF-1	ng/mL	Enhanced	mid	195.075	15.36463828	58	16	80
IgF-1	ng/mL	Enhanced	late	200.6125	13.34101333	58	16	80
IgF-1	ng/mL	Standard	pre	239.58125	23.82370789	58	16	80
IgF-1	ng/mL	Standard	early	255.4	26.0949588	58	16	80
IgF-1	ng/mL	Standard	mid	252.25	23.48316551	58	16	80
IgF-1	ng/mL	Standard	late	254.8625	24.22238723	58	16	80
Insulin	4.3-19.9 uIU/mL	Enhanced	pre	10.46875	0.758129766	58	16	80
Insulin	4.3-19.9 uIU/mL	Enhanced	early	10.3625	0.526467443	58	16	80
Insulin	4.3-19.9 uIU/mL	Enhanced	mid	11.0875	1.650325489	58	16	80
Insulin	4.3-19.9 uIU/mL	Enhanced	late	10.3375	1.325228459	58	16	80
Insulin	4.3-19.9 uIU/mL	Standard	pre	12.14375	0.690303234	58	16	80
Insulin	4.3-19.9 uIU/mL	Standard	early	12.55	1.306115424	58	16	80
Insulin	4.3-19.9 uIU/mL	Standard	mid	13.725	0.962540584	58	16	80
Insulin	4.3-19.9 uIU/mL	Standard	late	11.8875	0.622102659	58	16	80
Iodine	4.0-9.2 ug/dL	Enhanced	pre	6.4375	0.336241868	58	16	80
Iodine	4.0-9.2 ug/dL	Enhanced	early	6.14	0.205045727	58	16	80
Iodine	4.0-9.2 ug/dL	Enhanced	mid	5.9275	0.250797167	58	16	80
Iodine	4.0-9.2 ug/dL	Enhanced	late	5.92625	0.273592635	58	16	80
Iodine	4.0-9.2 ug/dL	Standard	pre	7.085625	0.483289055	58	16	80
Iodine	4.0-9.2 ug/dL	Standard	early	6.37125	0.415626175	58	16	80
Iodine	4.0-9.2 ug/dL	Standard	mid	5.50625	0.416108976	58	16	80
Iodine	4.0-9.2 ug/dL	Standard	late	6.01375	0.454171421	58	16	80
Iron	Males: 650-1750 Females: 500-1700 ng/mL	Enhanced	pre	1561.75	131.6405796	58	16	80
Iron	Males: 650-1750 Females: 500-1700 ng/mL	Enhanced	early	1130.625	57.63867449	58	16	80
Iron	Males: 650-1750 Females: 500-1700 ng/mL	Enhanced	mid	1002.5	80.58186831	58	16	80
Iron	Males: 650-1750 Females: 500-1700 ng/mL	Enhanced	late	1171.875	90.3902023	58	16	80
Iron	Males: 650-1750 Females: 500-1700 ng/mL	Standard	pre	1252.1875	82.43297171	58	16	80
Iron	Males: 650-1750 Females: 500-1700 ng/mL	Standard	early	1177.5	109.0707912	58	16	80
Iron	Males: 650-1750 Females: 500-1700 ng/mL	Standard	mid	1082.5	113.892356	58	16	80
Iron	Males: 650-1750 Females: 500-1700 ng/mL	Standard	late	1201.25	129.9071483	58	16	80
Isoleucine	40-140 umol/L	Enhanced	pre	69.66	3.02385128	58	16	80
Isoleucine	40-140 umol/L	Enhanced	early	70.8025	4.270782551	58	16	80
Isoleucine	40-140 umol/L	Enhanced	mid	92.07875	7.317404005	58	16	80
Isoleucine	40-140 umol/L	Enhanced	late	90.03125	7.691685332	58	16	80
Isoleucine	40-140 umol/L	Standard	pre	78.953125	3.6918979	58	16	80
Isoleucine	40-140 umol/L	Standard	early	68.13875	3.973731102	58	16	80
Isoleucine	40-140 umol/L	Standard	mid	82.9825	3.563221309	58	16	80
Isoleucine	40-140 umol/L	Standard	late	80.80625	3.246918702	58	16	80
Isorhamnetin, plasma	ng/mL	Enhanced	pre	3.10853963	1.02329237	28	14	48
Isorhamnetin, plasma	ng/mL	Enhanced	early	4.457142857	1.591121687	28	14	48
Isorhamnetin, plasma	ng/mL	Enhanced	mid	2.818808699	0.941770311	28	14	48
Isorhamnetin, plasma	ng/mL	Enhanced	late	3.872857143	1.018994531	28	14	48

Isorhamnetin, plasma	ng/mL	Standard	pre	3.985115071	0.564519315	28	14	48
Isorhamnetin, plasma	ng/mL	Standard	early	3.470687918	0.541535548	28	14	48
Isorhamnetin, plasma	ng/mL	Standard	mid	2.955572138	0.553183071	28	14	48
Isorhamnetin, plasma	ng/mL	Standard	late	3.737772025	0.854334168	28	14	48
Kaempferol, plasma	ng/mL	Enhanced	pre	5.292362236	1.371577313	21	15	42
Kaempferol, plasma	ng/mL	Enhanced	early	7.060218805	2.563382982	21	15	42
Kaempferol, plasma	ng/mL	Enhanced	mid	4.701146256	2.045461026	21	15	42
Kaempferol, plasma	ng/mL	Enhanced	late	4.297141397	0.744612173	21	15	42
Kaempferol, plasma	ng/mL	Standard	pre	4.489986232	1.189682988	21	15	42
Kaempferol, plasma	ng/mL	Standard	early	6.114105657	1.477385509	21	15	42
Kaempferol, plasma	ng/mL	Standard	mid	4.02743899	1.527300241	21	15	42
Kaempferol, plasma	ng/mL	Standard	late	4.359445642	1.295551437	21	15	42
Kynurenic acid	20-100 nmol/L	Enhanced	pre	61.29375	4.63602097	58	16	80
Kynurenic acid	20-100 nmol/L	Enhanced	early	44.4875	3.961038275	58	16	80
Kynurenic acid	20-100 nmol/L	Enhanced	mid	47.0125	4.776421173	58	16	80
Kynurenic acid	20-100 nmol/L	Enhanced	late	40.325	3.190647955	58	16	80
Kynurenic acid	20-100 nmol/L	Standard	pre	65.3375	5.32621575	58	16	80
Kynurenic acid	20-100 nmol/L	Standard	early	47.425	3.985158011	58	16	80
Kynurenic acid	20-100 nmol/L	Standard	mid	62.325	5.097847279	58	16	80
Kynurenic acid	20-100 nmol/L	Standard	late	45	3.789459064	58	16	80
LDL Cholesterol	60-99 mg/dL	Enhanced	pre	115.6875	10.49886062	58	16	80
LDL Cholesterol	60-99 mg/dL	Enhanced	early	108	10.95587742	58	16	80
LDL Cholesterol	60-99 mg/dL	Enhanced	mid	90.875	8.155591449	58	16	80
LDL Cholesterol	60-99 mg/dL	Enhanced	late	92.125	9.311346405	58	16	80
LDL Cholesterol	60-99 mg/dL	Standard	pre	97.8125	11.31161512	58	16	80
LDL Cholesterol	60-99 mg/dL	Standard	early	95.75	11.75498564	58	16	80
LDL Cholesterol	60-99 mg/dL	Standard	mid	92	11.38666984	58	16	80
LDL Cholesterol	60-99 mg/dL	Standard	late	90.625	10.93723214	58	16	80
LDL Oxidized	26-117 U/L	Enhanced	pre	52.8625	6.432338647	58	16	80
LDL Oxidized	26-117 U/L	Enhanced	early	49.7125	6.188558937	58	16	80
LDL Oxidized	26-117 U/L	Enhanced	mid	43.6375	4.450313806	58	16	80
LDL Oxidized	26-117 U/L	Enhanced	late	43.7375	5.1042855	58	16	80
LDL Oxidized	26-117 U/L	Standard	pre	49.93125	6.221296197	58	16	80
LDL Oxidized	26-117 U/L	Standard	early	47.675	5.771907993	58	16	80
LDL Oxidized	26-117 U/L	Standard	mid	47.3625	6.661853569	58	16	80
LDL Oxidized	26-117 U/L	Standard	late	47.3125	6.16576834	58	16	80
Leptin	Males: 1.2-9.5 Females: 4.1-25.0 ng/mL	Enhanced	pre	9.175	1.218830126	58	16	80
Leptin	Males: 1.2-9.5 Females: 4.1-25.0 ng/mL	Enhanced	early	7.7625	1.134947342	58	16	80
Leptin	Males: 1.2-9.5 Females: 4.1-25.0 ng/mL	Enhanced	mid	8.3375	1.720005223	58	16	80
Leptin	Males: 1.2-9.5 Females: 4.1-25.0 ng/mL	Enhanced	late	6.9375	1.15419581	58	16	80
Leptin	Males: 1.2-9.5 Females: 4.1-25.0 ng/mL	Standard	pre	27.35625	7.61533423	58	16	80
Leptin	Males: 1.2-9.5 Females: 4.1-25.0 ng/mL	Standard	early	26.3125	8.068930333	58	16	80
Leptin	Males: 1.2-9.5 Females: 4.1-25.0 ng/mL	Standard	mid	24.4875	6.379347476	58	16	80
Leptin	Males: 1.2-9.5 Females: 4.1-25.0 ng/mL	Standard	late	22.2875	5.723920136	58	16	80
Leucine	70-170 umol/L	Enhanced	pre	143.8125	5.471472537	58	16	80
Leucine	70-170 umol/L	Enhanced	early	138.875	6.879402	58	16	80
Leucine	70-170 umol/L	Enhanced	mid	170.125	11.38589794	58	16	80
Leucine	70-170 umol/L	Enhanced	late	173.25	12.06848945	58	16	80
Leucine	70-170 umol/L	Standard	pre	147.75	6.284280985	58	16	80
Leucine	70-170 umol/L	Standard	early	136.25	7.383998747	58	16	80
Leucine	70-170 umol/L	Standard	mid	156.125	4.803766426	58	16	80
Leucine	70-170 umol/L	Standard	late	152	6.637959024	58	16	80
Luteolin, plasma	ng/mL	Enhanced	pre	5.07	0	0	5	6
Luteolin, plasma	ng/mL	Enhanced	early	1.39	#N/A	0	5	6
Luteolin, plasma	ng/mL	Standard	pre	1.93	0.335062183	0	5	6
Luteolin, plasma	ng/mL	Standard	late	0.944778103	0.112459085	0	5	6
Lysine	120-290 umol/L	Enhanced	pre	205.3125	12.88135083	58	16	80
Lysine	120-290 umol/L	Enhanced	early	175.5	11.32751076	58	16	80
Lysine	120-290 umol/L	Enhanced	mid	201	17.61391495	58	16	80
Lysine	120-290 umol/L	Enhanced	late	211	19.29216162	58	16	80
Lysine	120-290 umol/L	Standard	pre	196.5	7.614234203	58	16	80
Lysine	120-290 umol/L	Standard	early	179.625	7.485271997	58	16	80
Lysine	120-290 umol/L	Standard	mid	195.75	7.426199398	58	16	80
Lysine	120-290 umol/L	Standard	late	191.625	11.37834772	58	16	80
Magnesium	0.70-0.91 mmol/L	Enhanced	pre	0.836875	0.022860609	58	16	80
Magnesium	0.70-0.91 mmol/L	Enhanced	early	0.8625	0.027880885	58	16	80
Magnesium	0.70-0.91 mmol/L	Enhanced	mid	0.8575	0.019425338	58	16	80
Magnesium	0.70-0.91 mmol/L	Enhanced	late	0.83625	0.016764686	58	16	80
Magnesium	0.70-0.91 mmol/L	Standard	pre	0.85	0.017566837	58	16	80
Magnesium	0.70-0.91 mmol/L	Standard	early	0.85875	0.01915704	58	16	80
Magnesium	0.70-0.91 mmol/L	Standard	mid	0.85375	0.019680058	58	16	80
Magnesium	0.70-0.91 mmol/L	Standard	late	0.875	0.018456029	58	16	80
Mean corpuscular hemoglobin (MCH)	Males: 27.5-32.5 Females: 26.9-32.7 pg	Enhanced	pre	29.975	0.188469715	37	14	57
Mean corpuscular hemoglobin (MCH)	Males: 27.5-32.5 Females: 26.9-32.7 pg	Enhanced	early	29.96121712	0.177207051	37	14	57
Mean corpuscular hemoglobin (MCH)	Males: 27.5-32.5 Females: 26.9-32.7 pg	Enhanced	mid	30.36121712	0.179100082	37	14	57
Mean corpuscular hemoglobin (MCH)	Males: 27.5-32.5 Females: 26.9-32.7 pg	Enhanced	late	30.36121712	0.197941722	37	14	57
Mean corpuscular hemoglobin (MCH)	Males: 27.5-32.5 Females: 26.9-32.7 pg	Standard	pre	30.3625	0.549982244	37	14	57
Mean corpuscular hemoglobin (MCH)	Males: 27.5-32.5 Females: 26.9-32.7 pg	Standard	early	30.48527718	0.555698022	37	14	57
Mean corpuscular hemoglobin (MCH)	Males: 27.5-32.5 Females: 26.9-32.7 pg	Standard	mid	30.88568269	0.66398219	37	14	57
Mean corpuscular hemoglobin (MCH)	Males: 27.5-32.5 Females: 26.9-32.7 pg	Standard	late	30.59140385	0.59752677	37	14	57
Mean corpuscular hemoglobin concentration (MCHC)	Males: 32.6-35.2 Females: 31.8-34.5 g/dL	Enhanced	pre	34.125	0.169455828	37	14	57
Mean corpuscular hemoglobin concentration (MCHC)	Males: 32.6-35.2 Females: 31.8-34.5 g/dL	Enhanced	early	34.29727396	0.192690521	37	14	57
Mean corpuscular hemoglobin concentration (MCHC)	Males: 32.6-35.2 Females: 31.8-34.5 g/dL	Enhanced	mid	34.29727396	0.305714156	37	14	57

Mean corpuscular hemoglobin concentration (MCHC)	Males: 32.6-35.2 Females: 31.8-34.5 g/dL	Enhanced	late	34.42227396	0.180735549	37	14	57
Mean corpuscular hemoglobin concentration (MCHC)	Males: 32.6-35.2 Females: 31.8-34.5 g/dL	Standard	pre	34.31875	0.344707473	37	14	57
Mean corpuscular hemoglobin concentration (MCHC)	Males: 32.6-35.2 Females: 31.8-34.5 g/dL	Standard	early	34.69425682	0.230752784	37	14	57
Mean corpuscular hemoglobin concentration (MCHC)	Males: 32.6-35.2 Females: 31.8-34.5 g/dL	Standard	mid	34.74094403	0.15580338	37	14	57
Mean corpuscular hemoglobin concentration (MCHC)	Males: 32.6-35.2 Females: 31.8-34.5 g/dL	Standard	late	34.20874698	0.193161652	37	14	57
Mean corpuscular volume (MCV)	Males: 81.8-95.8 Females: 82.7-96.2 fL	Enhanced	pre	87.95833333	0.806089383	37	14	57
Mean corpuscular volume (MCV)	Males: 81.8-95.8 Females: 82.7-96.2 fL	Enhanced	early	87.34766	0.793042144	37	14	57
Mean corpuscular volume (MCV)	Males: 81.8-95.8 Females: 82.7-96.2 fL	Enhanced	mid	88.49766	0.579313769	37	14	57
Mean corpuscular volume (MCV)	Males: 81.8-95.8 Females: 82.7-96.2 fL	Enhanced	late	88.22266	0.832037621	37	14	57
Mean corpuscular volume (MCV)	Males: 81.8-95.8 Females: 82.7-96.2 fL	Standard	pre	88.5	1.657228485	37	14	57
Mean corpuscular volume (MCV)	Males: 81.8-95.8 Females: 82.7-96.2 fL	Standard	early	87.8016434	1.535470548	37	14	57
Mean corpuscular volume (MCV)	Males: 81.8-95.8 Females: 82.7-96.2 fL	Standard	mid	88.91677841	1.72778957	37	14	57
Mean corpuscular volume (MCV)	Males: 81.8-95.8 Females: 82.7-96.2 fL	Standard	late	89.44284446	1.686462106	37	14	57
Methionine	20-50 umol/L	Enhanced	pre	32.906875	1.424572561	58	16	80
Methionine	20-50 umol/L	Enhanced	early	28.58375	1.664519392	58	16	80
Methionine	20-50 umol/L	Enhanced	mid	33.1525	2.573543733	58	16	80
Methionine	20-50 umol/L	Enhanced	late	36.82	2.911978494	58	16	80
Methionine	20-50 umol/L	Standard	pre	33.246875	1.255576604	58	16	80
Methionine	20-50 umol/L	Standard	early	27.97875	0.872362879	58	16	80
Methionine	20-50 umol/L	Standard	mid	34.16125	1.41795786	58	16	80
Methionine	20-50 umol/L	Standard	late	33.355	0.739602934	58	16	80
Methylmalonic acid (MMA)	0-0.26 umol/L	Enhanced	pre	0.201875	0.018263212	58	16	80
Methylmalonic acid (MMA)	0-0.26 umol/L	Enhanced	early	0.1775	0.012960276	58	16	80
Methylmalonic acid (MMA)	0-0.26 umol/L	Enhanced	mid	0.19125	0.019719716	58	16	80
Methylmalonic acid (MMA)	0-0.26 umol/L	Enhanced	late	0.18875	0.020724193	58	16	80
Methylmalonic acid (MMA)	0-0.26 umol/L	Standard	pre	0.180625	0.018986786	58	16	80
Methylmalonic acid (MMA)	0-0.26 umol/L	Standard	early	0.17125	0.017178975	58	16	80
Methylmalonic acid (MMA)	0-0.26 umol/L	Standard	mid	0.1875	0.019182512	58	16	80
Methylmalonic acid (MMA)	0-0.26 umol/L	Standard	late	0.17125	0.016147513	58	16	80
Myricetin, plasma	ng/mL	Enhanced	pre	7.878333333	0.285368508	11	6	22
Myricetin, plasma	ng/mL	Enhanced	early	7.596666667	0.239505664	11	6	22
Myricetin, plasma	ng/mL	Enhanced	mid	7.31	0.086409876	11	6	22
Myricetin, plasma	ng/mL	Enhanced	late	8.375	0.222738636	11	6	22
Myricetin, plasma	ng/mL	Standard	pre	6.888333333	1.040921956	11	6	22
Myricetin, plasma	ng/mL	Standard	early	8.35	#N/A	11	6	22
Myricetin, plasma	ng/mL	Standard	mid	2.01	#N/A	11	6	22
N1-Methylnicotinamide	27-221 nmol/L	Enhanced	pre	144.875	17.97475443	58	16	80
N1-Methylnicotinamide	27-221 nmol/L	Enhanced	early	186	28.5	58	16	80
N1-Methylnicotinamide	27-221 nmol/L	Enhanced	mid	185.375	14.17848006	58	16	80
N1-Methylnicotinamide	27-221 nmol/L	Enhanced	late	153.625	17.12266179	58	16	80
N1-Methylnicotinamide	27-221 nmol/L	Standard	pre	122.5625	11.84022457	58	16	80
N1-Methylnicotinamide	27-221 nmol/L	Standard	early	187.125	17.82329717	58	16	80
N1-Methylnicotinamide	27-221 nmol/L	Standard	mid	142	12.0701077	58	16	80
N1-Methylnicotinamide	27-221 nmol/L	Standard	late	118.625	10.09631353	58	16	80
Naringenin, plasma	ng/mL	Enhanced	pre	-10.36401646	2.233249515	11	15	32
Naringenin, plasma	ng/mL	Enhanced	early	14.1125	5.307869968	11	15	32
Naringenin, plasma	ng/mL	Enhanced	mid	-13.19401646	2.233249515	11	15	32
Naringenin, plasma	ng/mL	Enhanced	late	-3.274280696	2.449428984	11	15	32
Naringenin, plasma	ng/mL	Standard	pre	6.251061928	1.779372289	11	15	32
Naringenin, plasma	ng/mL	Standard	early	3.110058465	0.692103142	11	15	32
Naringenin, plasma	ng/mL	Standard	mid	3.11666737	0.734337854	11	15	32
Naringenin, plasma	ng/mL	Standard	late	1.550882739	0.451597116	11	15	32
Neopterin	0-20 nmol/L	Enhanced	pre	17.28125	1.500154614	58	16	80
Neopterin	0-20 nmol/L	Enhanced	early	15.325	1.910803987	58	16	80
Neopterin	0-20 nmol/L	Enhanced	mid	16.1625	1.527859767	58	16	80
Neopterin	0-20 nmol/L	Enhanced	late	14.675	1.507248113	58	16	80
Neopterin	0-20 nmol/L	Standard	pre	15.9375	1.18696534	58	16	80
Neopterin	0-20 nmol/L	Standard	early	14.875	1.438070539	58	16	80
Neopterin	0-20 nmol/L	Standard	mid	14.9375	1.155413549	58	16	80
Neopterin	0-20 nmol/L	Standard	late	15.275	1.399190614	58	16	80
Nicotinamide	92-388 nmol/L	Enhanced	pre	254.75	19.52942491	58	16	80
Nicotinamide	92-388 nmol/L	Enhanced	early	241.625	21.51811555	58	16	80
Nicotinamide	92-388 nmol/L	Enhanced	mid	232.375	16.19793341	58	16	80
Nicotinamide	92-388 nmol/L	Enhanced	late	266.25	45.63980924	58	16	80
Nicotinamide	92-388 nmol/L	Standard	pre	243.9375	26.57190091	58	16	80
Nicotinamide	92-388 nmol/L	Standard	early	231.125	27.10883015	58	16	80
Nicotinamide	92-388 nmol/L	Standard	mid	200.875	17.65860051	58	16	80
Nicotinamide	92-388 nmol/L	Standard	late	226	18.81488772	58	16	80
Nicotinic acid nmol/L	0-20	Enhanced	pre	-8.88178E-16	#N/A	58	16	80
Nicotinic acid nmol/L	0-20	Enhanced	early	-2.66454E-15	#N/A	58	16	80
Nicotinic acid nmol/L	0-20	Enhanced	mid	-1.77636E-15	#N/A	58	16	80
Nicotinic acid nmol/L	0-20	Enhanced	late	-1.77636E-15	#N/A	#N/A	16	80
Nicotinic acid nmol/L	0-20	Standard	pre	-8.88178E-16	#N/A	58	16	80
Nicotinic acid nmol/L	0-20	Standard	early	-1.33227E-15	#N/A	58	16	80
Nicotinic acid nmol/L	0-20	Standard	mid	-8.88178E-16	#N/A	58	16	80
Nicotinic acid nmol/L	0-20	Standard	late	2.525	2.361921225	58	16	80
Ornithine	30-90 umol/L	Enhanced	pre	80.34875	6.274682745	58	16	80
Ornithine	30-90 umol/L	Enhanced	early	73.53	5.766898538	58	16	80
Ornithine	30-90 umol/L	Enhanced	mid	78.98875	6.113217094	58	16	80
Ornithine	30-90 umol/L	Enhanced	late	76.6225	5.906808241	58	16	80
Ornithine	30-90 umol/L	Standard	pre	80.581875	4.796582193	58	16	80
Ornithine	30-90 umol/L	Standard	early	78.395	4.302978983	58	16	80
Ornithine	30-90 umol/L	Standard	mid	81.175	7.048132953	58	16	80

Ornithine	30-90 umol/L	Standard	late	72.5075	6.3232707	58	16	80
Osteocalcin	Males: 5.2-34.5 Females: 7.7-36.0 ng/mL	Enhanced	pre	22.725	2.174856317	58	16	80
Osteocalcin	Males: 5.2-34.5 Females: 7.7-36.0 ng/mL	Enhanced	early	21.1875	1.735334757	58	16	80
Osteocalcin	Males: 5.2-34.5 Females: 7.7-36.0 ng/mL	Enhanced	mid	22.9	2.4568781	58	16	80
Osteocalcin	Males: 5.2-34.5 Females: 7.7-36.0 ng/mL	Enhanced	late	22.0625	2.183026676	58	16	80
Osteocalcin	Males: 5.2-34.5 Females: 7.7-36.0 ng/mL	Standard	pre	25.7	2.145325587	58	16	80
Osteocalcin	Males: 5.2-34.5 Females: 7.7-36.0 ng/mL	Standard	early	25.325	2.195503217	58	16	80
Osteocalcin	Males: 5.2-34.5 Females: 7.7-36.0 ng/mL	Standard	mid	27.05	2.386027033	58	16	80
Osteocalcin	Males: 5.2-34.5 Females: 7.7-36.0 ng/mL	Standard	late	27.4125	2.39546691	58	16	80
Osteoprotegerin (OPG)	Males: 2.7-5.5 Females: 2.6-5.6 pmol/L	Enhanced	pre	4.1951875	0.379132263	58	16	80
Osteoprotegerin (OPG)	Males: 2.7-5.5 Females: 2.6-5.6 pmol/L	Enhanced	early	3.76425	0.521306374	58	16	80
Osteoprotegerin (OPG)	Males: 2.7-5.5 Females: 2.6-5.6 pmol/L	Enhanced	mid	3.485125	0.425760402	58	16	80
Osteoprotegerin (OPG)	Males: 2.7-5.5 Females: 2.6-5.6 pmol/L	Enhanced	late	3.90875	0.412568205	58	16	80
Osteoprotegerin (OPG)	Males: 2.7-5.5 Females: 2.6-5.6 pmol/L	Standard	pre	3.37725	0.162270367	58	16	80
Osteoprotegerin (OPG)	Males: 2.7-5.5 Females: 2.6-5.6 pmol/L	Standard	early	3.4245	0.268367402	58	16	80
Osteoprotegerin (OPG)	Males: 2.7-5.5 Females: 2.6-5.6 pmol/L	Standard	mid	3.63225	0.159905514	58	16	80
Osteoprotegerin (OPG)	Males: 2.7-5.5 Females: 2.6-5.6 pmol/L	Standard	late	3.217875	0.141342938	58	16	80
Parathyroid Hormone, Intact (iPTH)	10.0-57.0 pg/mL	Enhanced	pre	23.93125	2.567631105	58	16	80
Parathyroid Hormone, Intact (iPTH)	10.0-57.0 pg/mL	Enhanced	early	21.2375	2.745561048	58	16	80
Parathyroid Hormone, Intact (iPTH)	10.0-57.0 pg/mL	Enhanced	mid	24.6125	3.224536667	58	16	80
Parathyroid Hormone, Intact (iPTH)	10.0-57.0 pg/mL	Enhanced	late	19.775	2.372745641	58	16	80
Parathyroid Hormone, Intact (iPTH)	10.0-57.0 pg/mL	Standard	pre	28.5375	2.90613239	58	16	80
Parathyroid Hormone, Intact (iPTH)	10.0-57.0 pg/mL	Standard	early	29.0125	2.601528247	58	16	80
Parathyroid Hormone, Intact (iPTH)	10.0-57.0 pg/mL	Standard	mid	33.3375	2.18738839	58	16	80
Parathyroid Hormone, Intact (iPTH)	10.0-57.0 pg/mL	Standard	late	28.4375	1.575886407	58	16	80
Phenylalanine	26-85 umol/L	Enhanced	pre	72.3825	2.642230947	58	16	80
Phenylalanine	26-85 umol/L	Enhanced	early	67.85	2.966601591	58	16	80
Phenylalanine	26-85 umol/L	Enhanced	mid	71.20125	3.505062605	58	16	80
Phenylalanine	26-85 umol/L	Enhanced	late	74.1275	3.614330311	58	16	80
Phenylalanine	26-85 umol/L	Standard	pre	73.891875	2.291184351	58	16	80
Phenylalanine	26-85 umol/L	Standard	early	68.94625	1.801416055	58	16	80
Phenylalanine	26-85 umol/L	Standard	mid	72.09875	2.757559177	58	16	80
Phenylalanine	26-85 umol/L	Standard	late	73.655	1.070372307	58	16	80
Phosphorus	0.87-1.45 mmol/L	Enhanced	pre	1.328125	0.038516813	58	16	80
Phosphorus	0.87-1.45 mmol/L	Enhanced	early	1.45875	0.04549854	58	16	80
Phosphorus	0.87-1.45 mmol/L	Enhanced	mid	1.455	0.037374791	58	16	80
Phosphorus	0.87-1.45 mmol/L	Enhanced	late	1.45375	0.041228688	58	16	80
Phosphorus	0.87-1.45 mmol/L	Standard	pre	1.37875	0.047760388	58	16	80
Phosphorus	0.87-1.45 mmol/L	Standard	early	1.465	0.068190908	58	16	80
Phosphorus	0.87-1.45 mmol/L	Standard	mid	1.45625	0.06326871	58	16	80
Phosphorus	0.87-1.45 mmol/L	Standard	late	1.3925	0.047128481	58	16	80
Phylloquinone	0.1-8.7 nmol/L	Enhanced	pre	1.775625	0.198972826	58	16	80
Phylloquinone	0.1-8.7 nmol/L	Enhanced	early	4.82875	0.848508687	58	16	80
Phylloquinone	0.1-8.7 nmol/L	Enhanced	mid	1.77	0.124837394	58	16	80
Phylloquinone	0.1-8.7 nmol/L	Enhanced	late	3.72	0.649975961	58	16	80
Phylloquinone	0.1-8.7 nmol/L	Standard	pre	2.024375	0.307835738	58	16	80
Phylloquinone	0.1-8.7 nmol/L	Standard	early	3.5625	0.622326155	58	16	80
Phylloquinone	0.1-8.7 nmol/L	Standard	mid	1.6675	0.2314408	58	16	80
Phylloquinone	0.1-8.7 nmol/L	Standard	late	1.99875	0.234237043	58	16	80
Picolinic acid	20-60 nmol/L	Enhanced	pre	50.1875	3.499416804	58	16	80
Picolinic acid	20-60 nmol/L	Enhanced	early	57.2375	14.11314699	58	16	80
Picolinic acid	20-60 nmol/L	Enhanced	mid	60.7125	13.69686823	58	16	80
Picolinic acid	20-60 nmol/L	Enhanced	late	46.5375	10.48761081	58	16	80
Picolinic acid	20-60 nmol/L	Standard	pre	54.8375	6.720942956	58	16	80
Picolinic acid	20-60 nmol/L	Standard	early	41.7125	2.740462866	58	16	80
Picolinic acid	20-60 nmol/L	Standard	mid	50.6875	4.332956464	58	16	80
Picolinic acid	20-60 nmol/L	Standard	late	41.8875	5.117355198	58	16	80
Platelet count	Males: 145-323 Females: 165-359 10^3/mm3	Enhanced	pre	236.4166667	19.61554679	36	14	56
Platelet count	Males: 145-323 Females: 165-359 10^3/mm3	Enhanced	early	228.4116385	11.99636041	36	14	56
Platelet count	Males: 145-323 Females: 165-359 10^3/mm3	Enhanced	mid	199.9116385	14.75179427	36	14	56
Platelet count	Males: 145-323 Females: 165-359 10^3/mm3	Enhanced	late	203.6616385	15.48781232	36	14	56
Platelet count	Males: 145-323 Females: 165-359 10^3/mm3	Standard	pre	230.8125	12.91708459	36	14	56
Platelet count	Males: 145-323 Females: 165-359 10^3/mm3	Standard	early	247.1279739	18.27257992	36	14	56
Platelet count	Males: 145-323 Females: 165-359 10^3/mm3	Standard	mid	236.6435562	12.13174954	36	14	56
Platelet count	Males: 145-323 Females: 165-359 10^3/mm3	Standard	late	244.1082673	17.86919589	36	14	56
Potassium	3.5-5.5 mmol/L	Enhanced	pre	4.74375	0.085438894	58	16	80
Potassium	3.5-5.5 mmol/L	Enhanced	early	4.45	0.06846532	58	16	80
Potassium	3.5-5.5 mmol/L	Enhanced	mid	4.375	0.094785943	58	16	80
Potassium	3.5-5.5 mmol/L	Enhanced	late	4.475	0.105697091	58	16	80
Potassium	3.5-5.5 mmol/L	Standard	pre	4.93125	0.130485122	58	16	80
Potassium	3.5-5.5 mmol/L	Standard	early	4.4375	0.082797758	58	16	80
Potassium	3.5-5.5 mmol/L	Standard	mid	4.55	0.119895788	58	16	80
Potassium	3.5-5.5 mmol/L	Standard	late	4.5625	0.076928335	58	16	80
Procollagen type 1 N-terminal propeptide (P1NP)	Males: 22-87 Females: 19-83 ug/L	Enhanced	pre	68.06875	7.23884345	58	16	80
Procollagen type 1 N-terminal propeptide (P1NP)	Males: 22-87 Females: 19-83 ug/L	Enhanced	early	63.1	5.579202452	58	16	80
Procollagen type 1 N-terminal propeptide (P1NP)	Males: 22-87 Females: 19-83 ug/L	Enhanced	mid	63.6	7.506351477	58	16	80
Procollagen type 1 N-terminal propeptide (P1NP)	Males: 22-87 Females: 19-83 ug/L	Enhanced	late	61.9	6.565082825	58	16	80
Procollagen type 1 N-terminal propeptide (P1NP)	Males: 22-87 Females: 19-83 ug/L	Standard	pre	62.625	6.58894671	58	16	80
Procollagen type 1 N-terminal propeptide (P1NP)	Males: 22-87 Females: 19-83 ug/L	Standard	early	61.9125	5.98151939	58	16	80
Procollagen type 1 N-terminal propeptide (P1NP)	Males: 22-87 Females: 19-83 ug/L	Standard	mid	61.5375	5.758985846	58	16	80
Procollagen type 1 N-terminal propeptide (P1NP)	Males: 22-87 Females: 19-83 ug/L	Standard	late	62.35	5.478024279	58	16	80
Proline	110-360 umol/L	Enhanced	pre	160.875	9.050962483	58	16	80
Proline	110-360 umol/L	Enhanced	early	145.125	5.702076102	58	16	80

Proline	110-360 umol/L	Enhanced	mid	156.375	8.651115354	58	16	80
Proline	110-360 umol/L	Enhanced	late	168.625	8.558507281	58	16	80
Proline	110-360 umol/L	Standard	pre	183.125	11.518243	58	16	80
Proline	110-360 umol/L	Standard	early	162.875	12.53300331	58	16	80
Proline	110-360 umol/L	Standard	mid	193.25	12.92617258	58	16	80
Proline	110-360 umol/L	Standard	late	163.25	11.56604675	58	16	80
Protein, Total	6.6-8.0 g/dL	Enhanced	pre	7.64375	0.096597384	58	16	80
Protein, Total	6.6-8.0 g/dL	Enhanced	early	7.375	0.155874549	58	16	80
Protein, Total	6.6-8.0 g/dL	Enhanced	mid	7.0375	0.168576299	58	16	80
Protein, Total	6.6-8.0 g/dL	Enhanced	late	7.025	0.143341812	58	16	80
Protein, Total	6.6-8.0 g/dL	Standard	pre	7.60625	0.174650879	58	16	80
Protein, Total	6.6-8.0 g/dL	Standard	early	7.3875	0.172696898	58	16	80
Protein, Total	6.6-8.0 g/dL	Standard	mid	7.2	0.161051234	58	16	80
Protein, Total	6.6-8.0 g/dL	Standard	late	7.275	0.232345379	58	16	80
Pyridoxal	6-28 nmol/L	Enhanced	pre	25.23125	3.301632379	58	16	80
Pyridoxal	6-28 nmol/L	Enhanced	early	20.925	1.994739175	58	16	80
Pyridoxal	6-28 nmol/L	Enhanced	mid	16.5	1.597361105	58	16	80
Pyridoxal	6-28 nmol/L	Enhanced	late	15.1625	1.386648106	58	16	80
Pyridoxal	6-28 nmol/L	Standard	pre	20.225	2.48877166	58	16	80
Pyridoxal	6-28 nmol/L	Standard	early	13.2625	0.683953923	58	16	80
Pyridoxal	6-28 nmol/L	Standard	mid	13.025	1.275091908	58	16	80
Pyridoxal	6-28 nmol/L	Standard	late	13.9125	1.13322514	58	16	80
Pyridoxal 5-phosphate	17-102 nmol/L	Enhanced	pre	95.875	17.39433277	58	16	80
Pyridoxal 5-phosphate	17-102 nmol/L	Enhanced	early	72.4	8.201219422	58	16	80
Pyridoxal 5-phosphate	17-102 nmol/L	Enhanced	mid	57.7875	6.042154973	58	16	80
Pyridoxal 5-phosphate	17-102 nmol/L	Enhanced	late	56.1875	6.475879224	58	16	80
Pyridoxal 5-phosphate	17-102 nmol/L	Standard	pre	69.8625	8.416189523	58	16	80
Pyridoxal 5-phosphate	17-102 nmol/L	Standard	early	47.3	3.034798181	58	16	80
Pyridoxal 5-phosphate	17-102 nmol/L	Standard	mid	50.6625	4.815078449	58	16	80
Pyridoxal 5-phosphate	17-102 nmol/L	Standard	late	50.075	4.49929682	58	16	80
Quercetin, plasma	ng/mL	Enhanced	pre	10.55590741	4.041579218	30	15	51
Quercetin, plasma	ng/mL	Enhanced	early	21.19	7.567549347	30	15	51
Quercetin, plasma	ng/mL	Enhanced	mid	7.584561042	3.354037538	30	15	51
Quercetin, plasma	ng/mL	Enhanced	late	9.346561042	3.321180528	30	15	51
Quercetin, plasma	ng/mL	Standard	pre	18.13305397	5.789775694	30	15	51
Quercetin, plasma	ng/mL	Standard	early	14.15714286	6.349614391	30	15	51
Quercetin, plasma	ng/mL	Standard	mid	13.93736564	4.026225498	30	15	51
Quercetin, plasma	ng/mL	Standard	late	27.39305397	7.795405468	30	15	51
Quinolinic acid	134-384 nmol/L	Enhanced	pre	370	21.18242933	58	16	80
Quinolinic acid	134-384 nmol/L	Enhanced	early	339.5	31.0327448	58	16	80
Quinolinic acid	134-384 nmol/L	Enhanced	mid	333.875	21.47195664	58	16	80
Quinolinic acid	134-384 nmol/L	Enhanced	late	324.375	18.16670848	58	16	80
Quinolinic acid	134-384 nmol/L	Standard	pre	356.75	24.27093169	58	16	80
Quinolinic acid	134-384 nmol/L	Standard	early	329.375	24.15052892	58	16	80
Quinolinic acid	134-384 nmol/L	Standard	mid	354.625	19.58789286	58	16	80
Quinolinic acid	134-384 nmol/L	Standard	late	359.375	22.14154572	58	16	80
RBC count	Males: 4.3-5.6 Females: 3.8-4.9 10^6/mm3	Enhanced	pre	4.74	0.222289226	37	14	57
RBC count	Males: 4.3-5.6 Females: 3.8-4.9 10^6/mm3	Enhanced	early	4.456499777	0.172503123	37	14	57
RBC count	Males: 4.3-5.6 Females: 3.8-4.9 10^6/mm3	Enhanced	mid	4.428999777	0.160330995	37	14	57
RBC count	Males: 4.3-5.6 Females: 3.8-4.9 10^6/mm3	Enhanced	late	4.336499777	0.169041422	37	14	57
RBC count	Males: 4.3-5.6 Females: 3.8-4.9 10^6/mm3	Standard	pre	4.81	0.164988162	37	14	57
RBC count	Males: 4.3-5.6 Females: 3.8-4.9 10^6/mm3	Standard	early	4.592907199	0.178004818	37	14	57
RBC count	Males: 4.3-5.6 Females: 3.8-4.9 10^6/mm3	Standard	mid	4.276192379	0.203665457	37	14	57
RBC count	Males: 4.3-5.6 Females: 3.8-4.9 10^6/mm3	Standard	late	4.396582832	0.150386796	37	14	57
RDW	Males: 11.2-13.9 Females: 11.3-14.0 %	Enhanced	pre	12.4	0.25	37	14	57
RDW	Males: 11.2-13.9 Females: 11.3-14.0 %	Enhanced	early	12.22205066	0.129856033	37	14	57
RDW	Males: 11.2-13.9 Females: 11.3-14.0 %	Enhanced	mid	12.44705066	0.227023286	37	14	57
RDW	Males: 11.2-13.9 Females: 11.3-14.0 %	Enhanced	late	12.09705066	0.181775251	37	14	57
RDW	Males: 11.2-13.9 Females: 11.3-14.0 %	Standard	pre	12.61875	0.213405699	37	14	57
RDW	Males: 11.2-13.9 Females: 11.3-14.0 %	Standard	early	12.45298573	0.168930195	37	14	57
RDW	Males: 11.2-13.9 Females: 11.3-14.0 %	Standard	mid	12.38493645	0.227438923	37	14	57
RDW	Males: 11.2-13.9 Females: 11.3-14.0 %	Standard	late	12.18821189	0.162089488	37	14	57
Renin	1.8-59.4 pg/mL	Enhanced	pre	14.4375	1.588944962	58	16	80
Renin	1.8-59.4 pg/mL	Enhanced	early	17.2875	2.7317827	58	16	80
Renin	1.8-59.4 pg/mL	Enhanced	mid	13.55	2.317797985	58	16	80
Renin	1.8-59.4 pg/mL	Enhanced	late	10.6	1.682446136	58	16	80
Renin	1.8-59.4 pg/mL	Standard	pre	11.45	2.027313493	58	16	80
Renin	1.8-59.4 pg/mL	Standard	early	11.7	2.718397966	58	16	80
Renin	1.8-59.4 pg/mL	Standard	mid	10.6875	1.991971189	58	16	80
Renin	1.8-59.4 pg/mL	Standard	late	11.3375	2.796478673	58	16	80
Retinol Binding Protein	3.0-6.0 mg/dL	Enhanced	pre	4.65625	0.253635673	58	16	80
Retinol Binding Protein	3.0-6.0 mg/dL	Enhanced	early	4.075	0.22132414	58	16	80
Retinol Binding Protein	3.0-6.0 mg/dL	Enhanced	mid	4.0625	0.2920496	58	16	80
Retinol Binding Protein	3.0-6.0 mg/dL	Enhanced	late	3.9625	0.273825617	58	16	80
Retinol Binding Protein	3.0-6.0 mg/dL	Standard	pre	4.94375	0.305731385	58	16	80
Retinol Binding Protein	3.0-6.0 mg/dL	Standard	early	4.5875	0.316443864	58	16	80
Retinol Binding Protein	3.0-6.0 mg/dL	Standard	mid	4.35	0.310241841	58	16	80
Retinol Binding Protein	3.0-6.0 mg/dL	Standard	late	4.3125	0.293916176	58	16	80
Riboflavin	5-38 nmol/L	Enhanced	pre	26.85	7.387949436	58	16	80
Riboflavin	5-38 nmol/L	Enhanced	early	32.5125	8.070866541	58	16	80
Riboflavin	5-38 nmol/L	Enhanced	mid	25.525	8.140211722	58	16	80
Riboflavin	5-38 nmol/L	Enhanced	late	24.93625	8.011005699	58	16	80
Riboflavin	5-38 nmol/L	Standard	pre	17.944375	2.126090793	58	16	80

Riboflavin	5-38 nmol/L	Standard	early	22.225	1.932594597	58	16	80
Riboflavin	5-38 nmol/L	Standard	mid	17.9875	1.785395396	58	16	80
Riboflavin	5-38 nmol/L	Standard	late	16.25	1.39317533	58	16	80
Sarcosine	0.7-2.3 umol/L	Enhanced	pre	1.564375	0.072631548	58	16	80
Sarcosine	0.7-2.3 umol/L	Enhanced	early	1.4175	0.132626708	58	16	80
Sarcosine	0.7-2.3 umol/L	Enhanced	mid	1.68	0.119895788	58	16	80
Sarcosine	0.7-2.3 umol/L	Enhanced	late	1.59375	0.150706021	58	16	80
Sarcosine	0.7-2.3 umol/L	Standard	pre	1.5775	0.099964838	58	16	80
Sarcosine	0.7-2.3 umol/L	Standard	early	1.31875	0.076166707	58	16	80
Sarcosine	0.7-2.3 umol/L	Standard	mid	1.64875	0.094809637	58	16	80
Sarcosine	0.7-2.3 umol/L	Standard	late	1.5325	0.109754698	58	16	80
Sclerostin	Males: 26.8-46.2; Females: 15.8-36.1 pmol/L	Enhanced	pre	27.575	2.273883655	58	16	80
Sclerostin	Males: 26.8-46.2; Females: 15.8-36.1 pmol/L	Enhanced	early	25.15	1.587942852	58	16	80
Sclerostin	Males: 26.8-46.2; Females: 15.8-36.1 pmol/L	Enhanced	mid	26.275	2.083397916	58	16	80
Sclerostin	Males: 26.8-46.2; Females: 15.8-36.1 pmol/L	Enhanced	late	26.7625	2.049995236	58	16	80
Sclerostin	Males: 26.8-46.2; Females: 15.8-36.1 pmol/L	Standard	pre	24.8125	3.080809426	58	16	80
Sclerostin	Males: 26.8-46.2; Females: 15.8-36.1 pmol/L	Standard	early	26.825	3.0542363	58	16	80
Sclerostin	Males: 26.8-46.2; Females: 15.8-36.1 pmol/L	Standard	mid	25.6	1.912867612	58	16	80
Sclerostin	Males: 26.8-46.2; Females: 15.8-36.1 pmol/L	Standard	late	26.775	2.838119426	58	16	80
Selenium	23-190 ng/mL	Enhanced	pre	134.5625	4.545290746	58	16	80
Selenium	23-190 ng/mL	Enhanced	early	127	5.852349955	58	16	80
Selenium	23-190 ng/mL	Enhanced	mid	118.5	4.035003098	58	16	80
Selenium	23-190 ng/mL	Enhanced	late	117	4.503470884	58	16	80
Selenium	23-190 ng/mL	Standard	pre	134.75	4.613160522	58	16	80
Selenium	23-190 ng/mL	Standard	early	124.375	4.753082881	58	16	80
Selenium	23-190 ng/mL	Standard	mid	123.625	4.660128418	58	16	80
Selenium	23-190 ng/mL	Standard	late	122.625	5.584402105	58	16	80
Serine	95-200 umol/L	Enhanced	pre	134.625	7.572436654	58	16	80
Serine	95-200 umol/L	Enhanced	early	129.5	6.602556323	58	16	80
Serine	95-200 umol/L	Enhanced	mid	126.625	7.05101389	58	16	80
Serine	95-200 umol/L	Enhanced	late	133.125	8.191911979	58	16	80
Serine	95-200 umol/L	Standard	pre	123.4375	5.71759722	58	16	80
Serine	95-200 umol/L	Standard	early	124.125	6.228556966	58	16	80
Serine	95-200 umol/L	Standard	mid	125.5	8.780304664	58	16	80
Serine	95-200 umol/L	Standard	late	120.625	4.412119318	58	16	80
Sex Hormone Binding Globulin (SHBG), calculated	Males: 10.71-75 nmol/L	Enhanced	pre	53.725	7.547156013	34	10	50
Sex Hormone Binding Globulin (SHBG), calculated	Males: 10.71-75 nmol/L	Enhanced	early	48.52	6.776626906	34	10	50
Sex Hormone Binding Globulin (SHBG), calculated	Males: 10.71-75 nmol/L	Enhanced	mid	51.82333333	5.439120469	34	10	50
Sex Hormone Binding Globulin (SHBG), calculated	Males: 10.71-75 nmol/L	Enhanced	late	45.755	7.990206071	34	10	50
Sex Hormone Binding Globulin (SHBG), calculated	Males: 10.71-75 nmol/L	Standard	pre	32.835	4.490396001	34	10	50
Sex Hormone Binding Globulin (SHBG), calculated	Males: 10.71-75 nmol/L	Standard	early	35.665	6.18823329	34	10	50
Sex Hormone Binding Globulin (SHBG), calculated	Males: 10.71-75 nmol/L	Standard	mid	41.5425	9.090003008	34	10	50
Sex Hormone Binding Globulin (SHBG), calculated	Males: 10.71-75 nmol/L	Standard	late	42.375	8.588598911	34	10	50
Sodium	137-146 mmol/L	Enhanced	pre	144.875	1.120651317	58	16	80
Sodium	137-146 mmol/L	Enhanced	early	144.25	1.182555496	58	16	80
Sodium	137-146 mmol/L	Enhanced	mid	143.5	0.984250984	58	16	80
Sodium	137-146 mmol/L	Enhanced	late	143.625	0.683225347	58	16	80
Sodium	137-146 mmol/L	Standard	pre	145.125	1.246087627	58	16	80
Sodium	137-146 mmol/L	Standard	early	143.375	1.117160183	58	16	80
Sodium	137-146 mmol/L	Standard	mid	144	1.334634782	58	16	80
Sodium	137-146 mmol/L	Standard	late	144	1.118033989	58	16	80
Superoxide Dismutase (SOD)	1226-1840 U/g Hgb	Enhanced	pre	1676.703333	73.45335082	37	14	57
Superoxide Dismutase (SOD)	1226-1840 U/g Hgb	Enhanced	early	1669.733165	45.62090498	37	14	57
Superoxide Dismutase (SOD)	1226-1840 U/g Hgb	Enhanced	mid	1706.060665	74.49551818	37	14	57
Superoxide Dismutase (SOD)	1226-1840 U/g Hgb	Enhanced	late	1646.445665	98.12827208	37	14	57
Superoxide Dismutase (SOD)	1226-1840 U/g Hgb	Standard	pre	1565.473125	29.96165044	37	14	57
Superoxide Dismutase (SOD)	1226-1840 U/g Hgb	Standard	early	1739.658974	98.17319155	37	14	57
Superoxide Dismutase (SOD)	1226-1840 U/g Hgb	Standard	mid	1793.655862	142.4719925	37	14	57
Superoxide Dismutase (SOD)	1226-1840 U/g Hgb	Standard	late	1701.663963	131.8129061	37	14	57
Testosterone	Males: 7.45-35.19 nmol/L	Enhanced	pre	18.32083333	2.637943595	34	10	50
Testosterone	Males: 7.45-35.19 nmol/L	Enhanced	early	18.12333333	2.776116059	34	10	50
Testosterone	Males: 7.45-35.19 nmol/L	Enhanced	mid	19.92666667	1.913794466	34	10	50
Testosterone	Males: 7.45-35.19 nmol/L	Enhanced	late	17.775	1.434020669	34	10	50
Testosterone	Males: 7.45-35.19 nmol/L	Standard	pre	19.4075	0.515286631	34	10	50
Testosterone	Males: 7.45-35.19 nmol/L	Standard	early	15.72	0.826869095	34	10	50
Testosterone	Males: 7.45-35.19 nmol/L	Standard	mid	19.355	1.518076497	34	10	50
Testosterone	Males: 7.45-35.19 nmol/L	Standard	late	17.5225	1.203683799	34	10	50
Testosterone, Bioavailable	Males: 2.08-12.48 nmol/L	Enhanced	pre	3.103333333	0.464551257	34	10	50
Testosterone, Bioavailable	Males: 2.08-12.48 nmol/L	Enhanced	early	3.053333333	0.415810231	34	10	50
Testosterone, Bioavailable	Males: 2.08-12.48 nmol/L	Enhanced	mid	3.43	0.550615817	34	10	50
Testosterone, Bioavailable	Males: 2.08-12.48 nmol/L	Enhanced	late	3.588333333	0.496137395	34	10	50
Testosterone, Bioavailable	Males: 2.08-12.48 nmol/L	Standard	pre	5.41375	0.637890505	34	10	50
Testosterone, Bioavailable	Males: 2.08-12.48 nmol/L	Standard	early	3.8175	0.639251271	34	10	50
Testosterone, Bioavailable	Males: 2.08-12.48 nmol/L	Standard	mid	4.515	0.85622354	34	10	50
Testosterone, Bioavailable	Males: 2.08-12.48 nmol/L	Standard	late	3.7675	0.739394812	34	10	50
Testosterone, Free	Males: 83-624 pmol/L	Enhanced	pre	190.76	28.52504501	34	10	50
Testosterone, Free	Males: 83-624 pmol/L	Enhanced	early	187.8466667	25.55929779	34	10	50
Testosterone, Free	Males: 83-624 pmol/L	Enhanced	mid	210.8216667	33.86444804	34	10	50
Testosterone, Free	Males: 83-624 pmol/L	Enhanced	late	220.5616667	30.54730907	34	10	50
Testosterone, Free	Males: 83-624 pmol/L	Standard	pre	332.89125	39.21615668	34	10	50
Testosterone, Free	Males: 83-624 pmol/L	Standard	early	234.805	39.4188528	34	10	50
Testosterone, Free	Males: 83-624 pmol/L	Standard	mid	277.805	52.7145641	34	10	50
Testosterone, Free	Males: 83-624 pmol/L	Standard	late	231.54	45.469295	34	10	50

Thiamine	7-16 nmol/L	Enhanced	pre	7.711875	0.598886295	58	16	80
Thiamine	7-16 nmol/L	Enhanced	early	8.0375	0.64374818	58	16	80
Thiamine	7-16 nmol/L	Enhanced	mid	7.09875	0.482287639	58	16	80
Thiamine	7-16 nmol/L	Enhanced	late	7.1475	0.552940294	58	16	80
Thiamine	7-16 nmol/L	Standard	pre	8.188125	0.54131274	58	16	80
Thiamine	7-16 nmol/L	Standard	early	7.68	0.38107988	58	16	80
Thiamine	7-16 nmol/L	Standard	mid	6.8275	0.359564624	58	16	80
Thiamine	7-16 nmol/L	Standard	late	6.7725	0.435343794	58	16	80
Thiamine mono-phosphate	1-3 nmol/L	Enhanced	pre	3.595625	0.439784604	58	16	80
Thiamine mono-phosphate	1-3 nmol/L	Enhanced	early	4.49	0.503223981	58	16	80
Thiamine mono-phosphate	1-3 nmol/L	Enhanced	mid	4.19125	0.400984872	58	16	80
Thiamine mono-phosphate	1-3 nmol/L	Enhanced	late	4.0925	0.406800588	58	16	80
Thiamine mono-phosphate	1-3 nmol/L	Standard	pre	4.63375	0.459574999	58	16	80
Thiamine mono-phosphate	1-3 nmol/L	Standard	early	4.8575	0.581753142	58	16	80
Thiamine mono-phosphate	1-3 nmol/L	Standard	mid	4.43125	0.460619276	58	16	80
Thiamine mono-phosphate	1-3 nmol/L	Standard	late	4.0975	0.373659061	58	16	80
Threonine	70-240 umol/L	Enhanced	pre	147.875	5.608830928	58	16	80
Threonine	70-240 umol/L	Enhanced	early	133	9.390819985	58	16	80
Threonine	70-240 umol/L	Enhanced	mid	148.125	7.888752872	58	16	80
Threonine	70-240 umol/L	Enhanced	late	157.75	7.070515363	58	16	80
Threonine	70-240 umol/L	Standard	pre	154.625	7.222334067	58	16	80
Threonine	70-240 umol/L	Standard	early	148.125	5.890770058	58	16	80
Threonine	70-240 umol/L	Standard	mid	179	8.582321947	58	16	80
Threonine	70-240 umol/L	Standard	late	164.5	8.970437559	58	16	80
Thyriod Stimulation Hormone (TSH), 3rd Generation	0.30-4.00 mU/L	Enhanced	pre	2.933125	0.408870335	58	16	80
Thyriod Stimulation Hormone (TSH), 3rd Generation	0.30-4.00 mU/L	Enhanced	early	3.3375	0.454256776	58	16	80
Thyriod Stimulation Hormone (TSH), 3rd Generation	0.30-4.00 mU/L	Enhanced	mid	3.84375	0.511049586	58	16	80
Thyriod Stimulation Hormone (TSH), 3rd Generation	0.30-4.00 mU/L	Enhanced	late	3.725	0.514808824	58	16	80
Thyriod Stimulation Hormone (TSH), 3rd Generation	0.30-4.00 mU/L	Standard	pre	2.68125	0.326005356	58	16	80
Thyriod Stimulation Hormone (TSH), 3rd Generation	0.30-4.00 mU/L	Standard	early	2.815	0.349941959	58	16	80
Thyriod Stimulation Hormone (TSH), 3rd Generation	0.30-4.00 mU/L	Standard	mid	3.06	0.299290828	58	16	80
Thyriod Stimulation Hormone (TSH), 3rd Generation	0.30-4.00 mU/L	Standard	late	2.90125	0.366731499	58	16	80
Thyroxine, Free (Free T4)	0.8-1.7 ng/dL	Enhanced	pre	1.36875	0.060555695	58	16	80
Thyroxine, Free (Free T4)	0.8-1.7 ng/dL	Enhanced	early	1.3875	0.048210152	58	16	80
Thyroxine, Free (Free T4)	0.8-1.7 ng/dL	Enhanced	mid	1.3125	0.073818824	58	16	80
Thyroxine, Free (Free T4)	0.8-1.7 ng/dL	Enhanced	late	1.3125	0.067169329	58	16	80
Thyroxine, Free (Free T4)	0.8-1.7 ng/dL	Standard	pre	1.31875	0.034161267	58	16	80
Thyroxine, Free (Free T4)	0.8-1.7 ng/dL	Standard	early	1.325	0.045927933	58	16	80
Thyroxine, Free (Free T4)	0.8-1.7 ng/dL	Standard	mid	1.2625	0.039280641	58	16	80
Thyroxine, Free (Free T4)	0.8-1.7 ng/dL	Standard	late	1.275	0.038527588	58	16	80
Total Antioxidant Capacity	1.31-2.23 mmol/L	Enhanced	pre	1.39375	0.020415604	26	8	40
Total Antioxidant Capacity	1.31-2.23 mmol/L	Enhanced	early	1.39	0.012747549	26	8	40
Total Antioxidant Capacity	1.31-2.23 mmol/L	Enhanced	mid	1.36	0.010606602	26	8	40
Total Antioxidant Capacity	1.31-2.23 mmol/L	Enhanced	late	1.3875	0.017809759	26	8	40
Total Antioxidant Capacity	1.31-2.23 mmol/L	Standard	pre	1.5	0.053297514	26	8	40
Total Antioxidant Capacity	1.31-2.23 mmol/L	Standard	early	1.545	0.048669806	26	8	40
Total Antioxidant Capacity	1.31-2.23 mmol/L	Standard	mid	1.5225	0.037312029	26	8	40
Total Antioxidant Capacity	1.31-2.23 mmol/L	Standard	late	1.5325	0.059094733	26	8	40
Total Lipid Peroxides	0-2.00 umol/L	Enhanced	pre	0.77	0.046937458	58	16	80
Total Lipid Peroxides	0-2.00 umol/L	Enhanced	early	0.6925	0.050054658	58	16	80
Total Lipid Peroxides	0-2.00 umol/L	Enhanced	mid	0.69125	0.089711996	58	16	80
Total Lipid Peroxides	0-2.00 umol/L	Enhanced	late	0.635	0.058816452	58	16	80
Total Lipid Peroxides	0-2.00 umol/L	Standard	pre	0.645	0.068030324	58	16	80
Total Lipid Peroxides	0-2.00 umol/L	Standard	early	0.6775	0.060279298	58	16	80
Total Lipid Peroxides	0-2.00 umol/L	Standard	mid	0.62375	0.063070831	58	16	80
Total Lipid Peroxides	0-2.00 umol/L	Standard	late	0.68125	0.064673157	58	16	80
Trans-3-hydroxycotinine	nmol/L	Enhanced	pre	-1.06475E-15	#N/A	58	16	80
Trans-3-hydroxycotinine	nmol/L	Enhanced	early	-1.1113E-15	#N/A	58	16	80
Trans-3-hydroxycotinine	nmol/L	Enhanced	mid	-1.13199E-15	#N/A	58	16	80
Trans-3-hydroxycotinine	nmol/L	Enhanced	late	-1.13199E-15	#N/A	58	16	80
Trans-3-hydroxycotinine	nmol/L	Standard	pre	6.948125	6.116608023	58	16	80
Trans-3-hydroxycotinine	nmol/L	Standard	early	0.355	0.332072093	58	16	80
Trans-3-hydroxycotinine	nmol/L	Standard	mid	5.39586E-16	#N/A	#N/A	16	80
Trans-3-hydroxycotinine	nmol/L	Standard	late	1.58225E-15	#N/A	58	16	80
Transferrin	200-360 mg/dL	Enhanced	pre	268.75	17.62610812	58	16	80
Transferrin	200-360 mg/dL	Enhanced	early	257.375	17.53384005	58	16	80
Transferrin	200-360 mg/dL	Enhanced	mid	247.25	15.40469693	58	16	80
Transferrin	200-360 mg/dL	Enhanced	late	256.75	15.87524606	58	16	80
Transferrin	200-360 mg/dL	Standard	pre	260.375	11.59059088	58	16	80
Transferrin	200-360 mg/dL	Standard	early	260.5	13.85753405	58	16	80
Transferrin	200-360 mg/dL	Standard	mid	250	10.94160637	58	16	80
Transferrin	200-360 mg/dL	Standard	late	247.625	8.306506298	58	16	80
Transferrin Receptors	2.9-8.3 ug/mL	Enhanced	pre	4.175	0.543714079	58	16	80
Transferrin Receptors	2.9-8.3 ug/mL	Enhanced	early	3.95	0.508981827	58	16	80
Transferrin Receptors	2.9-8.3 ug/mL	Enhanced	mid	4.2375	0.641516148	58	16	80
Transferrin Receptors	2.9-8.3 ug/mL	Enhanced	late	4.55	0.656220237	58	16	80
Transferrin Receptors	2.9-8.3 ug/mL	Standard	pre	5.55625	1.453644061	58	16	80
Transferrin Receptors	2.9-8.3 ug/mL	Standard	early	5.4375	1.463507933	58	16	80
Transferrin Receptors	2.9-8.3 ug/mL	Standard	mid	6.375	1.840495008	58	16	80
Transferrin Receptors	2.9-8.3 ug/mL	Standard	late	6.15	1.845602883	58	16	80
Transthyretin	20-40 mg/dL	Enhanced	pre	30	1.386317063	58	16	80
Transthyretin	20-40 mg/dL	Enhanced	early	25.75	0.947859431	58	16	80
Transthyretin	20-40 mg/dL	Enhanced	mid	25.375	1.198143095	58	16	80

Transthyretin	20-40 mg/dL	Enhanced	late	25.125	1.362872655	58	16	80
Transthyretin	20-40 mg/dL	Standard	pre	30.6875	1.83485673	58	16	80
Transthyretin	20-40 mg/dL	Standard	early	29	1.629800601	58	16	80
Transthyretin	20-40 mg/dL	Standard	mid	27.75	1.518119725	58	16	80
Transthyretin	20-40 mg/dL	Standard	late	28	1.31101106	58	16	80
Triglyceride	Males: 40-149; Females: 35-149 mg/dL	Enhanced	pre	71.75	6.136468854	58	16	80
Triglyceride	Males: 40-149; Females: 35-149 mg/dL	Enhanced	early	75.625	6.60004143	58	16	80
Triglyceride	Males: 40-149; Females: 35-149 mg/dL	Enhanced	mid	77.125	8.02814872	58	16	80
Triglyceride	Males: 40-149; Females: 35-149 mg/dL	Enhanced	late	76.375	10.45217905	58	16	80
Triglyceride	Males: 40-149; Females: 35-149 mg/dL	Standard	pre	92.8125	8.135059878	58	16	80
Triglyceride	Males: 40-149; Females: 35-149 mg/dL	Standard	early	90	9.632302425	58	16	80
Triglyceride	Males: 40-149; Females: 35-149 mg/dL	Standard	mid	105	15.55936213	58	16	80
Triglyceride	Males: 40-149; Females: 35-149 mg/dL	Standard	late	97.375	13.63696436	58	16	80
Trigonelline	umol/L	Enhanced	pre	0.5765	0.107343818	58	16	80
Trigonelline	umol/L	Enhanced	early	0.5735	0.02456242	58	16	80
Trigonelline	umol/L	Enhanced	mid	0.41075	0.043807373	58	16	80
Trigonelline	umol/L	Enhanced	late	0.478	0.054250288	58	16	80
Trigonelline	umol/L	Standard	pre	0.945125	0.184750481	58	16	80
Trigonelline	umol/L	Standard	early	0.43925	0.05410255	58	16	80
Trigonelline	umol/L	Standard	mid	0.635	0.052048355	58	16	80
Trigonelline	umol/L	Standard	late	0.63975	0.149238772	58	16	80
Triiodo-thyronine, Free (Free T3)	2.4-4.2 pg/mL	Enhanced	pre	3.34375	0.10805001	58	16	80
Triiodo-thyronine, Free (Free T3)	2.4-4.2 pg/mL	Enhanced	early	3.2125	0.048210152	58	16	80
Triiodo-thyronine, Free (Free T3)	2.4-4.2 pg/mL	Enhanced	mid	3.0125	0.087388322	58	16	80
Triiodo-thyronine, Free (Free T3)	2.4-4.2 pg/mL	Enhanced	late	2.9125	0.05978477	58	16	80
Triiodo-thyronine, Free (Free T3)	2.4-4.2 pg/mL	Standard	pre	3.35625	0.138190737	58	16	80
Triiodo-thyronine, Free (Free T3)	2.4-4.2 pg/mL	Standard	early	3.325	0.128391491	58	16	80
Triiodo-thyronine, Free (Free T3)	2.4-4.2 pg/mL	Standard	mid	3.0125	0.105234114	58	16	80
Triiodo-thyronine, Free (Free T3)	2.4-4.2 pg/mL	Standard	late	3.05	0.113192314	58	16	80
Trypthophan	43-89 umol/L	Enhanced	pre	80.29625	3.150852036	58	16	80
Trypthophan	43-89 umol/L	Enhanced	early	72.48	3.461979203	58	16	80
Trypthophan	43-89 umol/L	Enhanced	mid	79.5025	4.361347179	58	16	80
Trypthophan	43-89 umol/L	Enhanced	late	82.23	3.590219353	58	16	80
Trypthophan	43-89 umol/L	Standard	pre	85.206875	3.988671158	58	16	80
Trypthophan	43-89 umol/L	Standard	early	73.7125	2.860412312	58	16	80
Trypthophan	43-89 umol/L	Standard	mid	80.34875	2.9983899	58	16	80
Trypthophan	43-89 umol/L	Standard	late	83.575	2.163021613	58	16	80
Tyrosine	40-110 umol/L	Enhanced	pre	64.81125	3.088397105	58	16	80
Tyrosine	40-110 umol/L	Enhanced	early	59.765	4.872726072	58	16	80
Tyrosine	40-110 umol/L	Enhanced	mid	62.37875	4.761548277	58	16	80
Tyrosine	40-110 umol/L	Enhanced	late	65.0775	4.680876237	58	16	80
Tyrosine	40-110 umol/L	Standard	pre	74.974375	2.764697858	58	16	80
Tyrosine	40-110 umol/L	Standard	early	65.88	1.683233495	58	16	80
Tyrosine	40-110 umol/L	Standard	mid	71.24	2.701153689	58	16	80
Tyrosine	40-110 umol/L	Standard	late	68.50625	1.634211333	58	16	80
Under-carboxylated Osteocalcin	24-50 %	Enhanced	pre	20.64375	3.168853641	58	16	80
Under-carboxylated Osteocalcin	24-50 %	Enhanced	early	23.15375	3.348070311	58	16	80
Under-carboxylated Osteocalcin	24-50 %	Enhanced	mid	22.51	1.969088463	58	16	80
Under-carboxylated Osteocalcin	24-50 %	Enhanced	late	20.7425	2.124781423	58	16	80
Under-carboxylated Osteocalcin	24-50 %	Standard	pre	20.49875	2.040393029	58	16	80
Under-carboxylated Osteocalcin	24-50 %	Standard	early	26.25125	2.88667815	58	16	80
Under-carboxylated Osteocalcin	24-50 %	Standard	mid	28.815	2.043472076	58	16	80
Under-carboxylated Osteocalcin	24-50 %	Standard	late	31.26875	3.45394993	58	16	80
Valine	150-350 umol/L	Enhanced	pre	278.125	13.55219943	58	16	80
Valine	150-350 umol/L	Enhanced	early	250.75	12.60425275	58	16	80
Valine	150-350 umol/L	Enhanced	mid	313.75	17.81129382	58	16	80
Valine	150-350 umol/L	Enhanced	late	302.125	14.35970654	58	16	80
Valine	150-350 umol/L	Standard	pre	281.0625	8.094383421	58	16	80
Valine	150-350 umol/L	Standard	early	250.625	9.197379892	58	16	80
Valine	150-350 umol/L	Standard	mid	307.5	6.284902545	58	16	80
Valine	150-350 umol/L	Standard	late	290.125	9.095736467	58	16	80
Vitamin B12	118-716 pmol/L	Enhanced	pre	328.130625	37.12833421	58	16	80
Vitamin B12	118-716 pmol/L	Enhanced	early	345.9975	40.81650374	58	16	80
Vitamin B12	118-716 pmol/L	Enhanced	mid	287.42875	27.6397566	58	16	80
Vitamin B12	118-716 pmol/L	Enhanced	late	290.945	22.22379946	58	16	80
Vitamin B12	118-716 pmol/L	Standard	pre	342.33875	52.01734973	58	16	80
Vitamin B12	118-716 pmol/L	Standard	early	324.41125	45.82450687	58	16	80
Vitamin B12	118-716 pmol/L	Standard	mid	313.90625	40.47003193	58	16	80
Vitamin B12	118-716 pmol/L	Standard	late	306.8625	42.80933838	58	16	80
Vitamin C	4-20 ug/mL	Enhanced	pre	10.70125	0.668184194	58	16	80
Vitamin C	4-20 ug/mL	Enhanced	early	13.265	0.453662182	58	16	80
Vitamin C	4-20 ug/mL	Enhanced	mid	11.675	0.531680708	58	16	80
Vitamin C	4-20 ug/mL	Enhanced	late	12.1475	0.440893092	58	16	80
Vitamin C	4-20 ug/mL	Standard	pre	8.5075	0.471688291	58	16	80
Vitamin C	4-20 ug/mL	Standard	early	8.3	0.516054745	58	16	80
Vitamin C	4-20 ug/mL	Standard	mid	8.70875	0.548815308	58	16	80
Vitamin C	4-20 ug/mL	Standard	late	10.17875	0.52108192	58	16	80
Vitamin E: Total Lipids Ratio	0.8-4.7 mg/g	Enhanced	pre	2.775	0.082443162	58	16	80
Vitamin E: Total Lipids Ratio	0.8-4.7 mg/g	Enhanced	early	3.0375	0.096723672	58	16	80
Vitamin E: Total Lipids Ratio	0.8-4.7 mg/g	Enhanced	mid	3.0125	0.083736006	58	16	80
Vitamin E: Total Lipids Ratio	0.8-4.7 mg/g	Enhanced	late	2.825	0.065550553	58	16	80
Vitamin E: Total Lipids Ratio	0.8-4.7 mg/g	Standard	pre	2.63125	0.072315228	58	16	80
Vitamin E: Total Lipids Ratio	0.8-4.7 mg/g	Standard	early	2.7875	0.119160475	58	16	80

Vitamin E: Total Lipids Ratio	0.8-4.7 mg/g	Standard	mid	2.6125	0.143000765	58	16	80
Vitamin E: Total Lipids Ratio	0.8-4.7 mg/g	Standard	late	2.7625	0.141352286	58	16	80
WBC count	Males: 3.4-8.5 Females: 3.6-9.2 10^3/mm3	Enhanced	pre	5.25	0.311581985	37	14	57
WBC count	Males: 3.4-8.5 Females: 3.6-9.2 10^3/mm3	Enhanced	early	5.856742962	0.257348611	37	14	57
WBC count	Males: 3.4-8.5 Females: 3.6-9.2 10^3/mm3	Enhanced	mid	5.481742962	0.345574695	37	14	57
WBC count	Males: 3.4-8.5 Females: 3.6-9.2 10^3/mm3	Enhanced	late	5.556742962	0.24998457	37	14	57
WBC count	Males: 3.4-8.5 Females: 3.6-9.2 10^3/mm3	Standard	pre	5.5375	0.432629135	37	14	57
WBC count	Males: 3.4-8.5 Females: 3.6-9.2 10^3/mm3	Standard	early	5.487298387	0.384113998	37	14	57
WBC count	Males: 3.4-8.5 Females: 3.6-9.2 10^3/mm3	Standard	mid	5.54479096	0.514843389	37	14	57
WBC count	Males: 3.4-8.5 Females: 3.6-9.2 10^3/mm3	Standard	late	5.948917617	0.424821867	37	14	57
Xanthurenic acid	5-35 nmol/L	Enhanced	pre	23.09375	2.138969478	58	16	80
Xanthurenic acid	5-35 nmol/L	Enhanced	early	14.39875	1.975389548	58	16	80
Xanthurenic acid	5-35 nmol/L	Enhanced	mid	15.335	1.779486408	58	16	80
Xanthurenic acid	5-35 nmol/L	Enhanced	late	12.845	1.158369004	58	16	80
Xanthurenic acid	5-35 nmol/L	Standard	pre	24.7225	2.271392573	58	16	80
Xanthurenic acid	5-35 nmol/L	Standard	early	14.395	1.008041108	58	16	80
Xanthurenic acid	5-35 nmol/L	Standard	mid	21.15	1.960787469	58	16	80
Xanthurenic acid	5-35 nmol/L	Standard	late	14.6075	1.35775061	58	16	80
Zinc	Males: 11.5-44.5 Females: 9.95-39.2 umol/L	Enhanced	pre	15.64375	0.898281236	58	16	80
Zinc	Males: 11.5-44.5 Females: 9.95-39.2 umol/L	Enhanced	early	14.7375	0.800183085	58	16	80
Zinc	Males: 11.5-44.5 Females: 9.95-39.2 umol/L	Enhanced	mid	13.175	0.789333184	58	16	80
Zinc	Males: 11.5-44.5 Females: 9.95-39.2 umol/L	Enhanced	late	13.775	0.70771419	58	16	80
Zinc	Males: 11.5-44.5 Females: 9.95-39.2 umol/L	Standard	pre	15.35	0.618781565	58	16	80
Zinc	Males: 11.5-44.5 Females: 9.95-39.2 umol/L	Standard	early	13.325	0.492997845	58	16	80
Zinc	Males: 11.5-44.5 Females: 9.95-39.2 umol/L	Standard	mid	13.85	0.594243216	58	16	80
Zinc	Males: 11.5-44.5 Females: 9.95-39.2 umol/L	Standard	late	14.6875	0.536900101	58	16	80
α-Ketoglutaric acid	20-50 umol/L	Enhanced	pre	7.611875	0.372926494	58	16	80
α-Ketoglutaric acid	20-50 umol/L	Enhanced	early	7.65	0.291874331	58	16	80
α-Ketoglutaric acid	20-50 umol/L	Enhanced	mid	7.10625	0.211282819	58	16	80
α-Ketoglutaric acid	20-50 umol/L	Enhanced	late	7.09125	0.17205956	58	16	80
α-Ketoglutaric acid	20-50 umol/L	Standard	pre	7.30125	0.229856868	58	16	80
α-Ketoglutaric acid	20-50 umol/L	Standard	early	7.4575	0.130321118	58	16	80
α-Ketoglutaric acid	20-50 umol/L	Standard	mid	7.2375	0.172642604	58	16	80
α-Ketoglutaric acid	20-50 umol/L	Standard	late	7.2125	0.208339792	58	16	80
sRANKL, Total	75.1-603.5 pmol/L	Enhanced	pre	435.541875	78.52423421	55	16	77
sRANKL, Total	75.1-603.5 pmol/L	Enhanced	early	394.42875	69.25544711	55	16	77
sRANKL, Total	75.1-603.5 pmol/L	Enhanced	mid	389.905	78.97458867	55	16	77
sRANKL, Total	75.1-603.5 pmol/L	Enhanced	late	381.22125	77.73123147	55	16	77
sRANKL, Total	75.1-603.5 pmol/L	Standard	pre	237.350625	52.03327036	55	16	77
sRANKL, Total	75.1-603.5 pmol/L	Standard	early	222.4347232	58.25200655	55	16	77
sRANKL, Total	75.1-603.5 pmol/L	Standard	mid	247.1990089	67.46518911	55	16	77
sRANKL, Total	75.1-603.5 pmol/L	Standard	late	230.4432947	57.07682119	55	16	77

Table S7: Average Individual Urine Markers per 24-hour excretion

Measurement	RangeUnits	Menu	Time	Estimate	StdErr	DF	Nsubjects	Nobs
3-methyl-histidine	64-320 umol/day	Enhanced	pre	284.34375	20.62329242	139	16	161
3-methyl-histidine	64-320 umol/day	Enhanced	early	259.5625	24.88244824	139	16	161
3-methyl-histidine	64-320 umol/day	Enhanced	mid	215.0625	12.93717919	139	16	161
3-methyl-histidine	64-320 umol/day	Enhanced	late	234.125	12.4824095	139	16	161
3-methyl-histidine	64-320 umol/day	Standard	pre	232.5744898	33.01538235	139	16	161
3-methyl-histidine	64-320 umol/day	Standard	early	212.375	26.55312335	139	16	161
3-methyl-histidine	64-320 umol/day	Standard	mid	240.375	26.45068377	139	16	161
3-methyl-histidine	64-320 umol/day	Standard	late	283.0625	28.71389547	139	16	161
8-OH-dG	0.9-10.5 ug/g Cr	Enhanced	pre	3.8321875	0.856048498	139	16	161
8-OH-dG	0.9-10.5 ug/g Cr	Enhanced	early	3.513125	0.756146138	139	16	161
8-OH-dG	0.9-10.5 ug/g Cr	Enhanced	mid	3.406875	0.810134989	139	16	161
8-OH-dG	0.9-10.5 ug/g Cr	Enhanced	late	2.679375	0.298670802	139	16	161
8-OH-dG	0.9-10.5 ug/g Cr	Standard	pre	3.300620967	0.284950109	139	16	161
8-OH-dG	0.9-10.5 ug/g Cr	Standard	early	3.256875	0.397407554	139	16	161
8-OH-dG	0.9-10.5 ug/g Cr	Standard	mid	3.204375	0.43609277	139	16	161
8-OH-dG	0.9-10.5 ug/g Cr	Standard	late	3.20625	0.267950482	139	16	161
Apigenin, urine	ug/day	Enhanced	pre	14.58272441	5.497544427	127	16	149
Apigenin, urine	ug/day	Enhanced	early	52.82125	8.153058847	127	16	149
Apigenin, urine	ug/day	Enhanced	mid	60.264375	10.09312279	127	16	149
Apigenin, urine	ug/day	Enhanced	late	79.205	14.76754952	127	16	149
Apigenin, urine	ug/day	Standard	pre	76.27240397	36.91834132	127	16	149
Apigenin, urine	ug/day	Standard	early	90.54125	41.34306373	127	16	149
Apigenin, urine	ug/day	Standard	mid	49.264375	16.51613062	127	16	149
Apigenin, urine	ug/day	Standard	late	131.1429984	68.55772141	127	16	149
Brushite Super-saturation	0-2.00	Enhanced	pre	1.341875	0.313488359	139	16	161
Brushite Super-saturation	0-2.00	Enhanced	early	1.30125	0.370001848	139	16	161
Brushite Super-saturation	0-2.00	Enhanced	mid	1.198125	0.280367975	139	16	161
Brushite Super-saturation	0-2.00	Enhanced	late	1.630625	0.445519743	139	16	161
Brushite Super-saturation	0-2.00	Standard	pre	1.757462905	0.266492176	139	16	161
Brushite Super-saturation	0-2.00	Standard	early	1.005625	0.246898634	139	16	161
Brushite Super-saturation	0-2.00	Standard	mid	0.933125	0.143939922	139	16	161
Brushite Super-saturation	0-2.00	Standard	late	1.035625	0.127262148	139	16	161
C-terminal telopeptides of Type I collagen (CTX-1)	Males 54-559; Females 67-544 ug/mmol creatinine	Enhanced	pre	174.34375	28.37689068	138	16	160
C-terminal telopeptides of Type I collagen (CTX-1)	Males 54-559; Females 67-544 ug/mmol creatinine	Enhanced	early	181.125	27.90255785	138	16	160
C-terminal telopeptides of Type I collagen (CTX-1)	Males 54-559; Females 67-544 ug/mmol creatinine	Enhanced	mid	218.9375	38.06136467	138	16	160
C-terminal telopeptides of Type I collagen (CTX-1)	Males 54-559; Females 67-544 ug/mmol creatinine	Enhanced	late	172.75	20.50590616	138	16	160
C-terminal telopeptides of Type I collagen (CTX-1)	Males 54-559; Females 67-544 ug/mmol creatinine	Standard	pre	130.6674563	18.99619927	138	16	160
C-terminal telopeptides of Type I collagen (CTX-1)	Males 54-559; Females 67-544 ug/mmol creatinine	Standard	early	151.6649296	21.53727561	138	16	160
C-terminal telopeptides of Type I collagen (CTX-1)	Males 54-559; Females 67-544 ug/mmol creatinine	Standard	mid	158.875	17.05545871	138	16	160
C-terminal telopeptides of Type I collagen (CTX-1)	Males 54-559; Females 67-544 ug/mmol creatinine	Standard	late	173.25	22.8992358	138	16	160
Calcium	2.5- 7.5 mmol/day	Enhanced	pre	4.82875	0.645907943	138	16	160
Calcium	2.5- 7.5 mmol/day	Enhanced	early	4.829375	0.951052639	138	16	160
Calcium	2.5- 7.5 mmol/day	Enhanced	mid	4.56125	0.584134476	138	16	160
Calcium	2.5- 7.5 mmol/day	Enhanced	late	4.87625	0.78412359	138	16	160
Calcium	2.5- 7.5 mmol/day	Standard	pre	5.253213019	0.532215621	138	16	160
Calcium	2.5- 7.5 mmol/day	Standard	early	4.35125	0.3414454	138	16	160
Calcium	2.5- 7.5 mmol/day	Standard	mid	5.292352338	0.66590245	138	16	160
Calcium	2.5- 7.5 mmol/day	Standard	late	4.903125	0.514825469	138	16	160
Calcium Oxalate Super-saturation	0-2.00	Enhanced	pre	1.174375	0.190142704	139	16	161
Calcium Oxalate Super-saturation	0-2.00	Enhanced	early	1.338125	0.2471145	139	16	161
Calcium Oxalate Super-saturation	0-2.00	Enhanced	mid	1.573125	0.328276676	139	16	161
Calcium Oxalate Super-saturation	0-2.00	Enhanced	late	1.431875	0.289844971	139	16	161
Calcium Oxalate Super-saturation	0-2.00	Standard	pre	1.237963361	0.136300535	139	16	161
Calcium Oxalate Super-saturation	0-2.00	Standard	early	1.35125	0.279264869	139	16	161
Calcium Oxalate Super-saturation	0-2.00	Standard	mid	1.1775	0.148568692	139	16	161
Calcium Oxalate Super-saturation	0-2.00	Standard	late	1.093125	0.133513042	139	16	161
Catechin, urine	ug/day	Enhanced	pre	109.1746394	40.82805209	127	16	149
Catechin, urine	ug/day	Enhanced	early	67.403125	27.66993104	127	16	149
Catechin, urine	ug/day	Enhanced	mid	69.67625	20.78090715	127	16	149
Catechin, urine	ug/day	Enhanced	late	75.77295386	24.13330918	127	16	149
Catechin, urine	ug/day	Standard	pre	89.22712571	30.78503281	127	16	149
Catechin, urine	ug/day	Standard	early	37.00835758	9.744845457	127	16	149
Catechin, urine	ug/day	Standard	mid	22.88562296	9.620766957	127	16	149
Catechin, urine	ug/day	Standard	late	63.45011154	15.37981878	127	16	149
Chrysin, urine	ug/day	Enhanced	early	1.58	0	0	4	4
Chrysin, urine	ug/day	Standard	pre	69.25	54.62368005	0	4	4
Citrate	>320 mg/day	Enhanced	pre	628.7209375	84.26837784	139	16	161
Citrate	>320 mg/day	Enhanced	early	656.075625	80.56614432	139	16	161
Citrate	>320 mg/day	Enhanced	mid	735.595	60.65472597	139	16	161
Citrate	>320 mg/day	Enhanced	late	654.685625	51.22962288	139	16	161
Citrate	>320 mg/day	Standard	pre	619.081544	83.29329337	139	16	161
Citrate	>320 mg/day	Standard	early	517.273125	59.37758277	139	16	161
Citrate	>320 mg/day	Standard	mid	633.67	54.03596198	139	16	161

Citrate	>320 mg/day	Standard	late	683.421875	61.8221383	139	16	161
Copper	0.05-0.79 umol/day	Enhanced	pre	0.3478125	0.044994439	139	16	161
Copper	0.05-0.79 umol/day	Enhanced	early	0.36875	0.060497611	139	16	161
Copper	0.05-0.79 umol/day	Enhanced	mid	0.68375	0.162429672	139	16	161
Copper	0.05-0.79 umol/day	Enhanced	late	0.281875	0.074879786	139	16	161
Copper	0.05-0.79 umol/day	Standard	pre	0.308761944	0.067568024	139	16	161
Copper	0.05-0.79 umol/day	Standard	early	0.4075	0.095361156	139	16	161
Copper	0.05-0.79 umol/day	Standard	mid	0.374375	0.05896528	139	16	161
Copper	0.05-0.79 umol/day	Standard	late	0.349375	0.054988902	139	16	161
Cortisol, Free	Males 4.2-60.0; Females 3.0-43.0 ug/day	Enhanced	pre	29.720625	3.943296546	139	16	161
Cortisol, Free	Males 4.2-60.0; Females 3.0-43.0 ug/day	Enhanced	early	30.918125	4.87582148	139	16	161
Cortisol, Free	Males 4.2-60.0; Females 3.0-43.0 ug/day	Enhanced	mid	28.82375	2.420807386	139	16	161
Cortisol, Free	Males 4.2-60.0; Females 3.0-43.0 ug/day	Enhanced	late	25.94125	3.122041443	139	16	161
Cortisol, Free	Males 4.2-60.0; Females 3.0-43.0 ug/day	Standard	pre	28.7989764	3.642698111	139	16	161
Cortisol, Free	Males 4.2-60.0; Females 3.0-43.0 ug/day	Standard	early	30.5775	5.388660403	139	16	161
Cortisol, Free	Males 4.2-60.0; Females 3.0-43.0 ug/day	Standard	mid	36.48125	6.619803195	139	16	161
Cortisol, Free	Males 4.2-60.0; Females 3.0-43.0 ug/day	Standard	late	39.544375	10.95396562	139	16	161
Creatinine	800-1800 mg/day	Enhanced	pre	1775.196875	134.9641183	139	16	161
Creatinine	800-1800 mg/day	Enhanced	early	1872.065625	131.9946395	139	16	161
Creatinine	800-1800 mg/day	Enhanced	mid	1733.071875	103.7334702	139	16	161
Creatinine	800-1800 mg/day	Enhanced	late	1820.671875	97.37440762	139	16	161
Creatinine	800-1800 mg/day	Standard	pre	1569.483843	174.181348	139	16	161
Creatinine	800-1800 mg/day	Standard	early	1590.43	186.8793005	139	16	161
Creatinine	800-1800 mg/day	Standard	mid	1656.535625	186.1349513	139	16	161
Creatinine	800-1800 mg/day	Standard	late	1677.921875	156.9272156	139	16	161
Daidzein, urine	ug/day	Enhanced	pre	281.905	82.63774697	135	16	157
Daidzein, urine	ug/day	Enhanced	early	291.661875	78.62677705	135	16	157
Daidzein, urine	ug/day	Enhanced	mid	452.905	132.5984311	135	16	157
Daidzein, urine	ug/day	Enhanced	late	853.6891418	219.5469146	135	16	157
Daidzein, urine	ug/day	Standard	pre	1470.991069	617.7569904	135	16	157
Daidzein, urine	ug/day	Standard	early	267.04375	46.83305625	135	16	157
Daidzein, urine	ug/day	Standard	mid	272.344375	81.58412936	135	16	157
Daidzein, urine	ug/day	Standard	late	250.7936786	52.99916876	135	16	157
Deoxypyridinoline (DPD)	Males 2.3-5.4; Females 3.0-7.4 nM/mM creatinine	Enhanced	pre	2.5403125	0.287235761	139	16	161
Deoxypyridinoline (DPD)	Males 2.3-5.4; Females 3.0-7.4 nM/mM creatinine	Enhanced	early	2.068125	0.261426282	139	16	161
Deoxypyridinoline (DPD)	Males 2.3-5.4; Females 3.0-7.4 nM/mM creatinine	Enhanced	mid	2.468125	0.162900798	139	16	161
Deoxypyridinoline (DPD)	Males 2.3-5.4; Females 3.0-7.4 nM/mM creatinine	Enhanced	late	2.268125	0.242069427	139	16	161
Deoxypyridinoline (DPD)	Males 2.3-5.4; Females 3.0-7.4 nM/mM creatinine	Standard	pre	3.533733158	0.415613815	139	16	161
Deoxypyridinoline (DPD)	Males 2.3-5.4; Females 3.0-7.4 nM/mM creatinine	Standard	early	2.915	0.306620949	139	16	161
Deoxypyridinoline (DPD)	Males 2.3-5.4; Females 3.0-7.4 nM/mM creatinine	Standard	mid	3.05	0.399606252	139	16	161
Deoxypyridinoline (DPD)	Males 2.3-5.4; Females 3.0-7.4 nM/mM creatinine	Standard	late	3.2225	0.408759556	139	16	161
Epicatechin, urine	ug/day	Enhanced	pre	180.914495	70.23925265	131	16	153
Epicatechin, urine	ug/day	Enhanced	early	90.51047124	36.5805343	131	16	153
Epicatechin, urine	ug/day	Enhanced	mid	175.79	110.4777218	131	16	153
Epicatechin, urine	ug/day	Enhanced	late	39.54353433	11.25448487	131	16	153
Epicatechin, urine	ug/day	Standard	pre	153.7914678	22.38515888	131	16	153
Epicatechin, urine	ug/day	Standard	early	85.7025	22.27959028	131	16	153
Epicatechin, urine	ug/day	Standard	mid	80.70777553	19.08425867	131	16	153
Epicatechin, urine	ug/day	Standard	late	65.27438168	10.45562571	131	16	153
Gamma-Carboxy-glutamate (GLA)	1.03-4.59 umol/mm creatinine	Enhanced	pre	3.5459375	0.410271189	139	16	161
Gamma-Carboxy-glutamate (GLA)	1.03-4.59 umol/mm creatinine	Enhanced	early	2.949375	0.338285472	139	16	161
Gamma-Carboxy-glutamate (GLA)	1.03-4.59 umol/mm creatinine	Enhanced	mid	2.925	0.345643648	139	16	161
Gamma-Carboxy-glutamate (GLA)	1.03-4.59 umol/mm creatinine	Enhanced	late	2.448125	0.34277924	139	16	161
Gamma-Carboxy-glutamate (GLA)	1.03-4.59 umol/mm creatinine	Standard	pre	3.367237482	0.138913361	139	16	161
Gamma-Carboxy-glutamate (GLA)	1.03-4.59 umol/mm creatinine	Standard	early	2.931875	0.241104129	139	16	161
Gamma-Carboxy-glutamate (GLA)	1.03-4.59 umol/mm creatinine	Standard	mid	2.915625	0.282323559	139	16	161
Gamma-Carboxy-glutamate (GLA)	1.03-4.59 umol/mm creatinine	Standard	late	2.841875	0.146188388	139	16	161
Genistein, urine	ug/day	Enhanced	pre	84.64112322	34.42689521	121	16	143
Genistein, urine	ug/day	Enhanced	early	60.636875	12.80400697	121	16	143
Genistein, urine	ug/day	Enhanced	mid	102.34125	26.16984331	121	16	143
Genistein, urine	ug/day	Enhanced	late	260.967236	51.56606203	121	16	143
Genistein, urine	ug/day	Standard	pre	570.3030548	276.219601	121	16	143
Genistein, urine	ug/day	Standard	early	85.32625	16.82372144	121	16	143
Genistein, urine	ug/day	Standard	mid	114.1273922	40.60627881	121	16	143
Genistein, urine	ug/day	Standard	late	74.4672741	18.87271914	121	16	143
Hesperetin, urine	ug/day	Enhanced	pre	437.3016706	182.4289258	132	16	154
Hesperetin, urine	ug/day	Enhanced	early	797.25125	362.7331834	132	16	154
Hesperetin, urine	ug/day	Enhanced	mid	742.685625	201.9122511	132	16	154
Hesperetin, urine	ug/day	Enhanced	late	906.47	428.5860688	132	16	154
Hesperetin, urine	ug/day	Standard	pre	306.3627214	123.0745782	132	16	154
Hesperetin, urine	ug/day	Standard	early	47.554375	12.20140311	132	16	154
Hesperetin, urine	ug/day	Standard	mid	93.38875	49.45125393	132	16	154
Hesperetin, urine	ug/day	Standard	late	37.74470002	9.708774904	132	16	154
Iodine	0.79-3.62 umol/day	Enhanced	pre	1.6815625	0.205988915	139	16	161
Iodine	0.79-3.62 umol/day	Enhanced	early	1.17625	0.090077007	139	16	161

Iodine	0.79-3.62 umol/day	Enhanced	mid	1.49375	0.117748242	139	16	161
Iodine	0.79-3.62 umol/day	Enhanced	late	1.525625	0.188037641	139	16	161
Iodine	0.79-3.62 umol/day	Standard	pre	2.14012554	0.220421574	139	16	161
Iodine	0.79-3.62 umol/day	Standard	early	0.9875	0.039921799	139	16	161
Iodine	0.79-3.62 umol/day	Standard	mid	1.094375	0.064265304	139	16	161
Iodine	0.79-3.62 umol/day	Standard	late	1.30125	0.10704026	139	16	161
Isorhamnetin, urine	ug/day	Enhanced	pre	16.47725264	3.182752102	59	11	76
Isorhamnetin, urine	ug/day	Enhanced	early	22.98173429	11.38351148	59	11	76
Isorhamnetin, urine	ug/day	Enhanced	mid	7.749726136	1.632623097	59	11	76
Isorhamnetin, urine	ug/day	Enhanced	late	15.33331693	3.529710229	59	11	76
Isorhamnetin, urine	ug/day	Standard	pre	21.10125114	6.763599483	59	11	76
Isorhamnetin, urine	ug/day	Standard	early	31.02813977	9.886526709	59	11	76
Isorhamnetin, urine	ug/day	Standard	mid	5.642160386	2.375486073	59	11	76
Isorhamnetin, urine	ug/day	Standard	late	18.4851242	6.556320247	59	11	76
Kaempferol, urine	ug/day	Enhanced	pre	33.17139554	16.72790228	70	15	91
Kaempferol, urine	ug/day	Enhanced	early	25.84160316	8.242058726	70	15	91
Kaempferol, urine	ug/day	Enhanced	mid	20.56975038	4.474228796	70	15	91
Kaempferol, urine	ug/day	Enhanced	late	27.21201604	12.96136953	70	15	91
Kaempferol, urine	ug/day	Standard	pre	41.07575177	18.8463218	70	15	91
Kaempferol, urine	ug/day	Standard	early	18.65252388	6.034923225	70	15	91
Kaempferol, urine	ug/day	Standard	mid	8.629367385	5.959409012	70	15	91
Kaempferol, urine	ug/day	Standard	late	48.23967025	15.70413213	70	15	91
Luteolin, urine	ug/day	Enhanced	pre	6.939354548	0.844227891	118	16	140
Luteolin, urine	ug/day	Enhanced	early	16.361875	2.4949394	118	16	140
Luteolin, urine	ug/day	Enhanced	mid	14.083125	1.827016424	118	16	140
Luteolin, urine	ug/day	Enhanced	late	21.221875	2.309689329	118	16	140
Luteolin, urine	ug/day	Standard	pre	16.7464806	4.096244083	118	16	140
Luteolin, urine	ug/day	Standard	early	11.52051257	2.87285679	118	16	140
Luteolin, urine	ug/day	Standard	mid	10.38758468	1.947680424	118	16	140
Luteolin, urine	ug/day	Standard	late	14.33996859	2.647990519	118	16	140
Magnesium	72-120 mg/day	Enhanced	pre	107.349375	12.16044396	139	16	161
Magnesium	72-120 mg/day	Enhanced	early	116.8675	13.89924649	139	16	161
Magnesium	72-120 mg/day	Enhanced	mid	95.594375	14.45625071	139	16	161
Magnesium	72-120 mg/day	Enhanced	late	100.000625	12.75328023	139	16	161
Magnesium	72-120 mg/day	Standard	pre	87.1847452	12.1121647	139	16	161
Magnesium	72-120 mg/day	Standard	early	75.24125	7.970302533	139	16	161
Magnesium	72-120 mg/day	Standard	mid	70.8625	12.20136474	139	16	161
Magnesium	72-120 mg/day	Standard	late	78.1125	9.303222728	139	16	161
Myricetin, urine	ug/day	Enhanced	pre	7.188979851	2.556258503	70	15	91
Myricetin, urine	ug/day	Enhanced	early	10.32127197	2.939395013	70	15	91
Myricetin, urine	ug/day	Enhanced	mid	8.493266354	1.834024215	70	15	91
Myricetin, urine	ug/day	Enhanced	late	11.5893366	3.071006452	70	15	91
Myricetin, urine	ug/day	Standard	pre	6.530096125	0.921225336	70	15	91
Myricetin, urine	ug/day	Standard	early	5.018351008	0.849566054	70	15	91
Myricetin, urine	ug/day	Standard	mid	5.380180004	0.737930636	70	15	91
Myricetin, urine	ug/day	Standard	late	6.830809504	0.834524541	70	15	91
N-telopeptides (NTX)	Males 5-87; Females 5-79 nM/mM creatinine	Enhanced	pre	33.5703125	4.516760939	138	16	160
N-telopeptides (NTX)	Males 5-87; Females 5-79 nM/mM creatinine	Enhanced	early	28.6225	4.951796233	138	16	160
N-telopeptides (NTX)	Males 5-87; Females 5-79 nM/mM creatinine	Enhanced	mid	35.7525	4.994018766	138	16	160
N-telopeptides (NTX)	Males 5-87; Females 5-79 nM/mM creatinine	Enhanced	late	29.561875	4.693717122	138	16	160
N-telopeptides (NTX)	Males 5-87; Females 5-79 nM/mM creatinine	Standard	pre	30.70495848	3.88797178	138	16	160
N-telopeptides (NTX)	Males 5-87; Females 5-79 nM/mM creatinine	Standard	early	27.48842468	4.063046748	138	16	160
N-telopeptides (NTX)	Males 5-87; Females 5-79 nM/mM creatinine	Standard	mid	26.26875	3.831321446	138	16	160
N-telopeptides (NTX)	Males 5-87; Females 5-79 nM/mM creatinine	Standard	late	28.279375	4.410135471	138	16	160
Naringenin, urine	ug/day	Enhanced	pre	380.4840625	85.41924091	138	16	160
Naringenin, urine	ug/day	Enhanced	early	809.23375	114.9932079	138	16	160
Naringenin, urine	ug/day	Enhanced	mid	1006.440625	173.9368677	138	16	160
Naringenin, urine	ug/day	Enhanced	late	639.385	128.5073867	138	16	160
Naringenin, urine	ug/day	Standard	pre	558.6005849	143.6467452	138	16	160
Naringenin, urine	ug/day	Standard	early	437.920625	72.90241293	138	16	160
Naringenin, urine	ug/day	Standard	mid	301.54875	103.1598623	138	16	160
Naringenin, urine	ug/day	Standard	late	473.4373569	141.5481322	138	16	160
Nitrogen, Total	4-20 g/day	Enhanced	pre	14.905625	1.189167066	139	16	161
Nitrogen, Total	4-20 g/day	Enhanced	early	13.393125	0.941530892	139	16	161
Nitrogen, Total	4-20 g/day	Enhanced	mid	13.140625	0.767125538	139	16	161
Nitrogen, Total	4-20 g/day	Enhanced	late	12.844375	0.878057354	139	16	161
Nitrogen, Total	4-20 g/day	Standard	pre	14.09770649	1.162278653	139	16	161
Nitrogen, Total	4-20 g/day	Standard	early	11.4375	1.032433034	139	16	161
Nitrogen, Total	4-20 g/day	Standard	mid	12.625	0.818487553	139	16	161
Nitrogen, Total	4-20 g/day	Standard	late	14.375	1.330971215	139	16	161
Oxalate	0-45 mg/day	Enhanced	pre	32.9621875	3.012775125	139	16	161
Oxalate	0-45 mg/day	Enhanced	early	30.47375	2.55378346	139	16	161
Oxalate	0-45 mg/day	Enhanced	mid	41.47125	3.380273398	139	16	161
Oxalate	0-45 mg/day	Enhanced	late	38.6375	2.973412392	139	16	161
Oxalate	0-45 mg/day	Standard	pre	32.68910118	4.289851349	139	16	161

Oxalate	0-45 mg/day	Standard	early	27.00875	3.599959608	139	16	161
Oxalate	0-45 mg/day	Standard	mid	29.181875	3.396830432	139	16	161
Oxalate	0-45 mg/day	Standard	late	33.360625	3.301298329	139	16	161
PGF2-alpha	0.9-4.9 ng/mg creatinine	Enhanced	pre	2.179375	0.27292103	139	16	161
PGF2-alpha	0.9-4.9 ng/mg creatinine	Enhanced	early	2.130625	0.324171344	139	16	161
PGF2-alpha	0.9-4.9 ng/mg creatinine	Enhanced	mid	2.51	0.4596636	139	16	161
PGF2-alpha	0.9-4.9 ng/mg creatinine	Enhanced	late	1.604375	0.136666329	139	16	161
PGF2-alpha	0.9-4.9 ng/mg creatinine	Standard	pre	1.982871974	0.151986532	139	16	161
PGF2-alpha	0.9-4.9 ng/mg creatinine	Standard	early	2.00875	0.138591548	139	16	161
PGF2-alpha	0.9-4.9 ng/mg creatinine	Standard	mid	1.7475	0.159096571	139	16	161
PGF2-alpha	0.9-4.9 ng/mg creatinine	Standard	late	1.84375	0.167366435	139	16	161
Phosphorus	400-1300 mg/day	Enhanced	pre	1139.67125	89.28965128	139	16	161
Phosphorus	400-1300 mg/day	Enhanced	early	1021.05	79.37825414	139	16	161
Phosphorus	400-1300 mg/day	Enhanced	mid	1048.641875	63.98142826	139	16	161
Phosphorus	400-1300 mg/day	Enhanced	late	1034.12	74.1886425	139	16	161
Phosphorus	400-1300 mg/day	Standard	pre	1233.197417	107.2388766	139	16	161
Phosphorus	400-1300 mg/day	Standard	early	963.5725	81.99630255	139	16	161
Phosphorus	400-1300 mg/day	Standard	mid	1007.068125	76.26697845	139	16	161
Phosphorus	400-1300 mg/day	Standard	late	1095.44	109.2987045	139	16	161
Potassium	25-125 mmol/day	Enhanced	pre	77.73625	5.303144409	139	16	161
Potassium	25-125 mmol/day	Enhanced	early	69.834375	7.109166037	139	16	161
Potassium	25-125 mmol/day	Enhanced	mid	78.195625	4.503099231	139	16	161
Potassium	25-125 mmol/day	Enhanced	late	80.351875	3.856297805	139	16	161
Potassium	25-125 mmol/day	Standard	pre	78.75502194	8.562072332	139	16	161
Potassium	25-125 mmol/day	Standard	early	46.436875	3.49899985	139	16	161
Potassium	25-125 mmol/day	Standard	mid	58.31	4.68173337	139	16	161
Potassium	25-125 mmol/day	Standard	late	69.215625	7.212143267	139	16	161
Quercetin, urine	ug/day	Enhanced	pre	42.52980236	15.75295753	61	11	78
Quercetin, urine	ug/day	Enhanced	early	278.5960038	201.4735643	61	11	78
Quercetin, urine	ug/day	Enhanced	mid	363.1933357	262.3129166	61	11	78
Quercetin, urine	ug/day	Enhanced	late	346.7817181	263.7273653	61	11	78
Quercetin, urine	ug/day	Standard	pre	60.13343381	11.70084906	61	11	78
Quercetin, urine	ug/day	Standard	early	48.54871001	17.33288585	61	11	78
Quercetin, urine	ug/day	Standard	mid	19.00709752	4.658984252	61	11	78
Quercetin, urine	ug/day	Standard	late	60.84437001	25.7476716	61	11	78
Selenium	0.00-2.54 umol/day	Enhanced	pre	1.368125	0.108034418	139	16	161
Selenium	0.00-2.54 umol/day	Enhanced	early	1.130625	0.085249805	139	16	161
Selenium	0.00-2.54 umol/day	Enhanced	mid	1.106875	0.069230917	139	16	161
Selenium	0.00-2.54 umol/day	Enhanced	late	0.885	0.066526545	139	16	161
Selenium	0.00-2.54 umol/day	Standard	pre	1.305136918	0.092554832	139	16	161
Selenium	0.00-2.54 umol/day	Standard	early	0.983125	0.088881669	139	16	161
Selenium	0.00-2.54 umol/day	Standard	mid	0.91375	0.064352241	139	16	161
Selenium	0.00-2.54 umol/day	Standard	late	1.049375	0.079811484	139	16	161
Sodium	40-200 mmol/day	Enhanced	pre	182.57375	9.476660722	139	16	161
Sodium	40-200 mmol/day	Enhanced	early	86.40625	5.651559226	139	16	161
Sodium	40-200 mmol/day	Enhanced	mid	98.000625	9.605804334	139	16	161
Sodium	40-200 mmol/day	Enhanced	late	110.626875	7.272938762	139	16	161
Sodium	40-200 mmol/day	Standard	pre	195.2462215	24.91790386	139	16	161
Sodium	40-200 mmol/day	Standard	early	86.975625	5.81871888	139	16	161
Sodium	40-200 mmol/day	Standard	mid	104.25625	12.38651963	139	16	161
Sodium	40-200 mmol/day	Standard	late	118.268125	15.38965115	139	16	161
Sodium Urate Supersaturation	0-2	Enhanced	pre	2.5365625	0.504474111	139	16	161
Sodium Urate Supersaturation	0-2	Enhanced	early	1.558125	0.371883863	139	16	161
Sodium Urate Supersaturation	0-2	Enhanced	mid	1.224375	0.29558131	139	16	161
Sodium Urate Supersaturation	0-2	Enhanced	late	1.491875	0.385337654	139	16	161
Sodium Urate Supersaturation	0-2	Standard	pre	2.39111161	0.42272379	139	16	161
Sodium Urate Supersaturation	0-2	Standard	early	1.2975	0.315204547	139	16	161
Sodium Urate Supersaturation	0-2	Standard	mid	1.035625	0.182443136	139	16	161
Sodium Urate Supersaturation	0-2	Standard	late	1.2725	0.263235577	139	16	161
Specific Gravity	1.009-1.031	Enhanced	pre	1.01421875	0.001540113	139	16	161
Specific Gravity	1.009-1.031	Enhanced	early	1.012875	0.001475715	139	16	161
Specific Gravity	1.009-1.031	Enhanced	mid	1.01175	0.001583607	139	16	161
Specific Gravity	1.009-1.031	Enhanced	late	1.0116875	0.001249805	139	16	161
Specific Gravity	1.009-1.031	Standard	pre	1.012308136	0.000627134	139	16	161
Specific Gravity	1.009-1.031	Standard	early	1.0105	0.001308028	139	16	161
Specific Gravity	1.009-1.031	Standard	mid	1.009625	0.000688919	139	16	161
Specific Gravity	1.009-1.031	Standard	late	1.01	0.000984251	139	16	161
Struvite Super-saturation	0-75	Enhanced	pre	2.99125	2.071668212	139	16	161
Struvite Super-saturation	0-75	Enhanced	early	0.94625	0.321596056	139	16	161
Struvite Super-saturation	0-75	Enhanced	mid	1.196875	0.561201235	139	16	161
Struvite Super-saturation	0-75	Enhanced	late	1.861875	0.644034487	139	16	161
Struvite Super-saturation	0-75	Standard	pre	1.362794328	0.300340838	139	16	161
Struvite Super-saturation	0-75	Standard	early	0.560625	0.287265444	139	16	161
Struvite Super-saturation	0-75	Standard	mid	0.29	0.123050422	139	16	161
Struvite Super-saturation	0-75	Standard	late	0.395625	0.073805263	139	16	161

Sulfate	0-30 mmol/day	Enhanced	pre	32.5821875	2.625127543	139	16	161
Sulfate	0-30 mmol/day	Enhanced	early	33.3725	2.677946245	139	16	161
Sulfate	0-30 mmol/day	Enhanced	mid	30.09375	1.979236506	139	16	161
Sulfate	0-30 mmol/day	Enhanced	late	29.959375	1.835591789	139	16	161
Sulfate	0-30 mmol/day	Standard	pre	28.79596177	2.333758835	139	16	161
Sulfate	0-30 mmol/day	Standard	early	25.445625	1.852607419	139	16	161
Sulfate	0-30 mmol/day	Standard	mid	29.045625	2.484003264	139	16	161
Sulfate	0-30 mmol/day	Standard	late	28.891875	2.79423387	139	16	161
Testosterone	Males: 45.1-611.6; Females: 2.8-47.8 nmol/day	Enhanced	pre	44.86625	13.02985009	139	16	161
Testosterone	Males: 45.1-611.6; Females: 2.8-47.8 nmol/day	Enhanced	early	39.1675	12.37512118	139	16	161
Testosterone	Males: 45.1-611.6; Females: 2.8-47.8 nmol/day	Enhanced	mid	44.44125	12.71590775	139	16	161
Testosterone	Males: 45.1-611.6; Females: 2.8-47.8 nmol/day	Enhanced	late	36.913125	10.13181514	139	16	161
Testosterone	Males: 45.1-611.6; Females: 2.8-47.8 nmol/day	Standard	pre	37.69288206	12.57600856	139	16	161
Testosterone	Males: 45.1-611.6; Females: 2.8-47.8 nmol/day	Standard	early	23.933125	5.893792148	139	16	161
Testosterone	Males: 45.1-611.6; Females: 2.8-47.8 nmol/day	Standard	mid	29.845	8.510312448	139	16	161
Testosterone	Males: 45.1-611.6; Females: 2.8-47.8 nmol/day	Standard	late	28.231875	7.980505884	139	16	161
Uric Acid + NaOH	250-750 mg/day	Enhanced	pre	678.295	44.56884921	139	16	161
Uric Acid + NaOH	250-750 mg/day	Enhanced	early	692.356875	46.36308406	139	16	161
Uric Acid + NaOH	250-750 mg/day	Enhanced	mid	635.8875	19.99064445	139	16	161
Uric Acid + NaOH	250-750 mg/day	Enhanced	late	650.564375	45.51315018	139	16	161
Uric Acid + NaOH	250-750 mg/day	Standard	pre	638.896598	67.84496929	139	16	161
Uric Acid + NaOH	250-750 mg/day	Standard	early	598.061875	43.66062658	139	16	161
Uric Acid + NaOH	250-750 mg/day	Standard	mid	631.118125	44.80427991	139	16	161
Uric Acid + NaOH	250-750 mg/day	Standard	late	772.178125	64.62180266	139	16	161
Uric Acid Super-saturation	0-2	Enhanced	pre	1.503125	0.227620911	139	16	161
Uric Acid Super-saturation	0-2	Enhanced	early	2.091875	0.354540787	139	16	161
Uric Acid Super-saturation	0-2	Enhanced	mid	1.31875	0.22970897	139	16	161
Uric Acid Super-saturation	0-2	Enhanced	late	0.983125	0.184310238	139	16	161
Uric Acid Super-saturation	0-2	Standard	pre	1.12490991	0.146833149	139	16	161
Uric Acid Super-saturation	0-2	Standard	early	2.1125	0.441597774	139	16	161
Uric Acid Super-saturation	0-2	Standard	mid	1.421875	0.28091221	139	16	161
Uric Acid Super-saturation	0-2	Standard	late	1.104375	0.188199606	139	16	161
Urine Volume, 24h pool	mL	Enhanced	pre	2279.96875	320.7448467	139	16	161
Urine Volume, 24h pool	mL	Enhanced	early	1888.5	251.1999484	139	16	161
Urine Volume, 24h pool	mL	Enhanced	mid	2290.6875	334.0580032	139	16	161
Urine Volume, 24h pool	mL	Enhanced	late	2270.4375	316.3711038	139	16	161
Urine Volume, 24h pool	mL	Standard	pre	2101.071109	168.7659381	139	16	161
Urine Volume, 24h pool	mL	Standard	early	1990.625	289.8459417	139	16	161
Urine Volume, 24h pool	mL	Standard	mid	2292.1875	214.6441266	139	16	161
Urine Volume, 24h pool	mL	Standard	late	2556.1875	301.9637543	139	16	161
Zinc	2.3-18.4 umol/day	Enhanced	pre	7.0375	0.807012721	139	16	161
Zinc	2.3-18.4 umol/day	Enhanced	early	5.0625	0.761102716	139	16	161
Zinc	2.3-18.4 umol/day	Enhanced	mid	5.74375	0.71809413	139	16	161
Zinc	2.3-18.4 umol/day	Enhanced	late	5.59375	0.701919105	139	16	161
Zinc	2.3-18.4 umol/day	Standard	pre	10.08102372	1.261445374	139	16	161
Zinc	2.3-18.4 umol/day	Standard	early	8.68125	1.244360324	139	16	161
Zinc	2.3-18.4 umol/day	Standard	mid	8.98125	0.92229069	139	16	161
Zinc	2.3-18.4 umol/day	Standard	late	8.95625	0.996495323	139	16	161
pH	#N/A	Enhanced	pre	6.0709375	0.073577576	139	16	161
pH	#N/A	Enhanced	early	5.928125	0.064855232	139	16	161
pH	#N/A	Enhanced	mid	6.101875	0.055120163	139	16	161
pH	#N/A	Enhanced	late	6.22125	0.076284572	139	16	161
pH	#N/A	Standard	pre	6.171635524	0.08093288	139	16	161
pH	#N/A	Standard	early	5.835625	0.075794941	139	16	161
pH	#N/A	Standard	mid	5.9775	0.073771183	139	16	161
pH	#N/A	Standard	late	6.1475	0.041954216	139	16	161

Table S8: Average Individual Serum Fatty Acids

Measurement	Menu	Time	Estimate	StdErr	DF	Nsubjects	Nobs
FA16:1 (Palmitoleic), serum uM	Standard	pre	127.875	18.3261745	58	16	80
FA16:1 (Palmitoleic), serum uM	Standard	early	367.2875	61.03472996	58	16	80
FA16:1 (Palmitoleic), serum uM	Standard	mid	264.8625	35.27986739	58	16	80
FA16:1 (Palmitoleic), serum uM	Standard	late	215.625	47.05036115	58	16	80
FA16:1 (Palmitoleic), serum uM	Enhanced	pre	172.8875	33.22338544	58	16	80
FA16:1 (Palmitoleic), serum uM	Enhanced	early	204.4625	26.7019366	58	16	80
FA16:1 (Palmitoleic), serum uM	Enhanced	mid	177.275	36.31895489	58	16	80
FA16:1 (Palmitoleic), serum uM	Enhanced	late	184.0875	32.27678847	58	16	80
FA18:0 (Stearic), serum uM	Standard	pre	602.475	41.68569711	58	16	80
FA18:0 (Stearic), serum uM	Standard	early	838.375	74.90902138	58	16	80
FA18:0 (Stearic), serum uM	Standard	mid	749.5375	46.88666501	58	16	80
FA18:0 (Stearic), serum uM	Standard	late	645.2125	88.51484593	58	16	80
FA18:0 (Stearic), serum uM	Enhanced	pre	783.7	77.99524725	58	16	80
FA18:0 (Stearic), serum uM	Enhanced	early	827.75	82.11145056	58	16	80
FA18:0 (Stearic), serum uM	Enhanced	mid	769.2125	101.3349311	58	16	80
FA18:0 (Stearic), serum uM	Enhanced	late	712.1625	64.68940199	58	16	80
FA18:1 (Oleic), serum uM	Standard	pre	1266.125	134.8844891	58	16	80
FA18:1 (Oleic), serum uM	Standard	early	2679.525	338.5285442	58	16	80
FA18:1 (Oleic), serum uM	Standard	mid	2185.0375	239.6974838	58	16	80
FA18:1 (Oleic), serum uM	Standard	late	1734.75	320.3819756	58	16	80
FA18:1 (Oleic), serum uM	Enhanced	pre	1748.73125	244.6129594	58	16	80
FA18:1 (Oleic), serum uM	Enhanced	early	2259.8625	265.946006	58	16	80
FA18:1 (Oleic), serum uM	Enhanced	mid	1877.8875	282.8965333	58	16	80
FA18:1 (Oleic), serum uM	Enhanced	late	1831.7375	223.8701248	58	16	80
FA18:2 Omega-6 (LA), serum uM	Standard	pre	2274.9375	296.5927376	58	16	80
FA18:2 Omega-6 (LA), serum uM	Standard	early	3668.75	459.2116274	58	16	80
FA18:2 Omega-6 (LA), serum uM	Standard	mid	3064.6375	361.2796934	58	16	80
FA18:2 Omega-6 (LA), serum uM	Standard	late	2513.6125	432.2686767	58	16	80
FA18:2 Omega-6 (LA), serum uM	Enhanced	pre	3002.26875	350.0649545	58	16	80
FA18:2 Omega-6 (LA), serum uM	Enhanced	early	3391.875	361.3315556	58	16	80
FA18:2 Omega-6 (LA), serum uM	Enhanced	mid	2772.4875	444.891652	58	16	80
FA18:2 Omega-6 (LA), serum uM	Enhanced	late	2696.75	350.8333548	58	16	80
FA18:3 Omega-3 (ALA), serum uM	Standard	pre	58.525	8.150565548	58	16	80
FA18:3 Omega-3 (ALA), serum uM	Standard	early	85.9875	11.04860621	58	16	80
FA18:3 Omega-3 (ALA), serum uM	Standard	mid	66.7375	7.902727723	58	16	80
FA18:3 Omega-3 (ALA), serum uM	Standard	late	53.4625	9.020998723	58	16	80
FA18:3 Omega-3 (ALA), serum uM	Enhanced	pre	72.0375	9.876165873	58	16	80
FA18:3 Omega-3 (ALA), serum uM	Enhanced	early	76.8375	8.668674522	58	16	80
FA18:3 Omega-3 (ALA), serum uM	Enhanced	mid	59.275	11.72840769	58	16	80
FA18:3 Omega-3 (ALA), serum uM	Enhanced	late	67.875	7.840674198	58	16	80
FA20:4 Omega-6 (AA), serum uM	Standard	pre	613.15625	60.23549711	58	16	80
FA20:4 Omega-6 (AA), serum uM	Standard	early	888.6625	90.13757876	58	16	80
FA20:4 Omega-6 (AA), serum uM	Standard	mid	834.1375	71.78852828	58	16	80
FA20:4 Omega-6 (AA), serum uM	Standard	late	770.1125	88.18567492	58	16	80
FA20:4 Omega-6 (AA), serum uM	Enhanced	pre	797.725	96.61019251	58	16	80
FA20:4 Omega-6 (AA), serum uM	Enhanced	early	822.7625	105.7177314	58	16	80
FA20:4 Omega-6 (AA), serum uM	Enhanced	mid	694.125	88.16494972	58	16	80
FA20:4 Omega-6 (AA), serum uM	Enhanced	late	645.0125	64.84962519	58	16	80
FA20:5 Omega-6 (EPA), serum uM	Standard	pre	32.9	3.769169753	58	16	80
FA20:5 Omega-6 (EPA), serum uM	Standard	early	38.5875	4.183988733	58	16	80
FA20:5 Omega-6 (EPA), serum uM	Standard	mid	34.9875	5.521334686	58	16	80

FA20:5 Omega-6 (EPA), serum uM	Standard	late	31.15	3.716264926	58	16	80
FA20:5 Omega-6 (EPA), serum uM	Enhanced	pre	38.125	3.762655728	58	16	80
FA20:5 Omega-6 (EPA), serum uM	Enhanced	early	38.2625	3.818087606	58	16	80
FA20:5 Omega-6 (EPA), serum uM	Enhanced	mid	40.275	4.740970035	58	16	80
FA20:5 Omega-6 (EPA), serum uM	Enhanced	late	31.4125	2.107014883	58	16	80
FA22:5 Omega-3 (DPA), serum uM	Standard	pre	37.5125	3.982557183	58	16	80
FA22:5 Omega-3 (DPA), serum uM	Standard	early	50.1125	4.769841372	58	16	80
FA22:5 Omega-3 (DPA), serum uM	Standard	mid	45.575	5.759577621	58	16	80
FA22:5 Omega-3 (DPA), serum uM	Standard	late	41.175	6.049709446	58	16	80
FA22:5 Omega-3 (DPA), serum uM	Enhanced	pre	50.5125	6.011380808	58	16	80
FA22:5 Omega-3 (DPA), serum uM	Enhanced	early	51.0375	5.602508855	58	16	80
FA22:5 Omega-3 (DPA), serum uM	Enhanced	mid	44.9125	6.186538751	58	16	80
FA22:5 Omega-3 (DPA), serum uM	Enhanced	late	37.175	3.30036693	58	16	80
FA22:5 Omega-6 (Osbond), serum uM	Standard	pre	17.80625	1.498213454	58	16	80
FA22:5 Omega-6 (Osbond), serum uM	Standard	early	25	2.709474119	58	16	80
FA22:5 Omega-6 (Osbond), serum uM	Standard	mid	22.9	1.8482255	58	16	80
FA22:5 Omega-6 (Osbond), serum uM	Standard	late	21.9375	2.663288469	58	16	80
FA22:5 Omega-6 (Osbond), serum uM	Enhanced	pre	22.025	2.402033644	58	16	80
FA22:5 Omega-6 (Osbond), serum uM	Enhanced	early	19.875	2.298827826	58	16	80
FA22:5 Omega-6 (Osbond), serum uM	Enhanced	mid	19.3375	2.474680377	58	16	80
FA22:5 Omega-6 (Osbond), serum uM	Enhanced	late	18.0625	2.049080396	58	16	80
FA22:6 Omega-3 (DHA), serum uM	Standard	pre	133.7625	19.04226334	58	16	80
FA22:6 Omega-3 (DHA), serum uM	Standard	early	159.0625	16.19991259	58	16	80
FA22:6 Omega-3 (DHA), serum uM	Standard	mid	168.8875	26.3456557	58	16	80
FA22:6 Omega-3 (DHA), serum uM	Standard	late	158.975	15.95860612	58	16	80
FA22:6 Omega-3 (DHA), serum uM	Enhanced	pre	221.45	26.25118747	58	16	80
FA22:6 Omega-3 (DHA), serum uM	Enhanced	early	205.725	21.47590765	58	16	80
FA22:6 Omega-3 (DHA), serum uM	Enhanced	mid	240.8125	29.27272855	58	16	80
FA22:6 Omega-3 (DHA), serum uM	Enhanced	late	199.75	15.50774	58	16	80

Table S9: Average Individual RedBlood Cell Fatty Acids

Measurement	Menu	Time	Estimate	StdErr	DF	Nsubjects	Nobs
FA16:0 (Palmitic), RBC uM	Standard	pre	663.8822622	22.38066819	53	16	75
FA16:0 (Palmitic), RBC uM	Standard	early	714.2125	20.3004344	53	16	75
FA16:0 (Palmitic), RBC uM	Standard	mid	685.9625	20.36725265	53	16	75
FA16:0 (Palmitic), RBC uM	Standard	late	666.5809285	24.91513788	53	16	75
FA16:0 (Palmitic), RBC uM	Enhanced	pre	664.29375	16.90844241	53	16	75
FA16:0 (Palmitic), RBC uM	Enhanced	early	671.0375	18.12159397	53	16	75
FA16:0 (Palmitic), RBC uM	Enhanced	mid	685.175	18.06387754	53	16	75
FA16:0 (Palmitic), RBC uM	Enhanced	late	654.6322284	16.32549163	53	16	75
FA16:1 (Palmitoleic), RBC uM	Standard	pre	2.15500177	0.255279283	51	16	73
FA16:1 (Palmitoleic), RBC uM	Standard	early	3.225	0.782773514	51	16	73
FA16:1 (Palmitoleic), RBC uM	Standard	mid	2.5	0.214330352	51	16	73
FA16:1 (Palmitoleic), RBC uM	Standard	late	3.012359046	0.460729273	51	16	73
FA16:1 (Palmitoleic), RBC uM	Enhanced	pre	3.204166715	0.575504491	51	16	73
FA16:1 (Palmitoleic), RBC uM	Enhanced	early	2.216349405	0.354922329	51	16	73
FA16:1 (Palmitoleic), RBC uM	Enhanced	mid	2.275	0.270849359	51	16	73
FA16:1 (Palmitoleic), RBC uM	Enhanced	late	1.93466372	0.190274125	51	16	73
FA18:0 (Steari), RBC uM	Standard	pre	305.575	14.07593525	57	16	79
FA18:0 (Steari), RBC uM	Standard	early	338.1625	14.4668904	57	16	79
FA18:0 (Steari), RBC uM	Standard	mid	310.05	11.71652145	57	16	79
FA18:0 (Steari), RBC uM	Standard	late	290.85	9.369881936	57	16	79
FA18:0 (Steari), RBC uM	Enhanced	pre	301.83125	10.22264619	57	16	79
FA18:0 (Steari), RBC uM	Enhanced	early	315.25	12.63873016	57	16	79
FA18:0 (Steari), RBC uM	Enhanced	mid	320.025	10.45794779	57	16	79
FA18:0 (Steari), RBC uM	Enhanced	late	293.9026712	9.119399604	57	16	79
FA18:1 (Oleic), RBC uM	Standard	pre	27.95625	3.24209902	58	16	80
FA18:1 (Oleic), RBC uM	Standard	early	32.3375	4.165105697	58	16	80
FA18:1 (Oleic), RBC uM	Standard	mid	28.5375	4.211440427	58	16	80
FA18:1 (Oleic), RBC uM	Standard	late	32.4875	5.257388774	58	16	80
FA18:1 (Oleic), RBC uM	Enhanced	pre	38.0375	5.251843054	58	16	80
FA18:1 (Oleic), RBC uM	Enhanced	early	26.6125	4.489760486	58	16	80
FA18:1 (Oleic), RBC uM	Enhanced	mid	30.0875	3.415289039	58	16	80
FA18:1 (Oleic), RBC uM	Enhanced	late	25.15	2.889961072	58	16	80
FA18:2 Omega-6 (LA), RBC uM	Standard	pre	56.025	17.31979615	37	15	58
FA18:2 Omega-6 (LA), RBC uM	Standard	early	46.16666667	9.257479459	37	15	58
FA18:2 Omega-6 (LA), RBC uM	Standard	mid	30.55714286	3.968388206	37	15	58
FA18:2 Omega-6 (LA), RBC uM	Standard	late	43.95	4.96161656	37	15	58
FA18:2 Omega-6 (LA), RBC uM	Enhanced	pre	59.84285714	7.299045427	37	15	58
FA18:2 Omega-6 (LA), RBC uM	Enhanced	early	50.4	12.73857331	37	15	58
FA18:2 Omega-6 (LA), RBC uM	Enhanced	mid	37.58	5.993423062	37	15	58
FA18:2 Omega-6 (LA), RBC uM	Enhanced	late	34.775	3.201049633	37	15	58
FA18:3 Omega-3 (ALA), RBC uM	Standard	pre	0.543125	0.202308817	53	16	75
FA18:3 Omega-3 (ALA), RBC uM	Standard	early	0.3675	0.060975149	53	16	75
FA18:3 Omega-3 (ALA), RBC uM	Standard	mid	0.35125	0.083206023	53	16	75
FA18:3 Omega-3 (ALA), RBC uM	Standard	late	0.43375	0.084130433	53	16	75
FA18:3 Omega-3 (ALA), RBC uM	Enhanced	pre	0.76463032	0.134228438	53	16	75
FA18:3 Omega-3 (ALA), RBC uM	Enhanced	early	0.386402996	0.095581623	53	16	75
FA18:3 Omega-3 (ALA), RBC uM	Enhanced	mid	0.280911054	0.031797552	53	16	75
FA18:3 Omega-3 (ALA), RBC uM	Enhanced	late	0.365213062	0.064654314	53	16	75
FA20:4 Omega-6 (AA), RBC uM	Standard	pre	74.64375	15.47458208	58	16	80
FA20:4 Omega-6 (AA), RBC uM	Standard	early	105.7	37.35673467	58	16	80

FA20:4 Omega-6 (AA), RBC uM	Standard	mid	80.1375	18.48629507	58	16	80
FA20:4 Omega-6 (AA), RBC uM	Standard	late	95.3125	19.68515809	58	16	80
FA20:4 Omega-6 (AA), RBC uM	Enhanced	pre	70.6625	5.835664527	58	16	80
FA20:4 Omega-6 (AA), RBC uM	Enhanced	early	60.4	15.04879563	58	16	80
FA20:4 Omega-6 (AA), RBC uM	Enhanced	mid	56.5625	11.71462677	58	16	80
FA20:4 Omega-6 (AA), RBC uM	Enhanced	late	37.5	6.749398121	58	16	80
FA20:5 Omega-6 (EPA), RBC uM	Standard	pre	9.439155653	0.569457715	48	16	70
FA20:5 Omega-6 (EPA), RBC uM	Standard	early	9.465860989	0.578318455	48	16	70
FA20:5 Omega-6 (EPA), RBC uM	Standard	mid	8.603543746	0.431346741	48	16	70
FA20:5 Omega-6 (EPA), RBC uM	Standard	late	9.25	0.363145976	48	16	70
FA20:5 Omega-6 (EPA), RBC uM	Enhanced	pre	10.5125	0.339778191	48	16	70
FA20:5 Omega-6 (EPA), RBC uM	Enhanced	early	8.6625	0.355289275	48	16	70
FA20:5 Omega-6 (EPA), RBC uM	Enhanced	mid	9.383742662	0.512436584	48	16	70
FA20:5 Omega-6 (EPA), RBC uM	Enhanced	late	9.554678842	0.758269161	48	16	70
FA22:5 Omega-3 (DPA), RBC uM	Standard	pre	4.988787738	0.420625512	5	14	24
FA22:5 Omega-3 (DPA), RBC uM	Standard	early	5.945276587	1.048137121	5	14	24
FA22:5 Omega-3 (DPA), RBC uM	Standard	mid	4.606365133	0.216367207	5	14	24
FA22:5 Omega-3 (DPA), RBC uM	Enhanced	pre	5.491595711	0.35851211	5	14	24
FA22:5 Omega-3 (DPA), RBC uM	Enhanced	early	3.951817238	0.117325759	5	14	24
FA22:5 Omega-3 (DPA), RBC uM	Enhanced	mid	4.151817238	0.06011529	5	14	24
FA22:5 Omega-3 (DPA), RBC uM	Enhanced	late	5.943990045	0.110640782	5	14	24
FA22:6 Omega-3 (DHA) RBC uM	Standard	pre	7.258838681	0.660983728	54	16	76
FA22:6 Omega-3 (DHA) RBC uM	Standard	early	7.25125	1.386394196	54	16	76
FA22:6 Omega-3 (DHA) RBC uM	Standard	mid	8.0075	1.134494312	54	16	76
FA22:6 Omega-3 (DHA) RBC uM	Standard	late	7.3175	0.730681937	54	16	76
FA22:6 Omega-3 (DHA) RBC uM	Enhanced	pre	7.961440423	0.720797151	54	16	76
FA22:6 Omega-3 (DHA) RBC uM	Enhanced	early	6.90409225	0.918354609	54	16	76
FA22:6 Omega-3 (DHA) RBC uM	Enhanced	mid	7.339136052	0.978199274	54	16	76
FA22:6 Omega-3 (DHA) RBC uM	Enhanced	late	6.0275	0.703073054	54	16	76

Table S10: Average Individual Salivary Cortisol. All values are reported in μg/dL.

Measurement	Menu	Time	Estimate	StdErr	DF	Nsubjects	Nobs
Cortisol	Enhanced	pre	0.329550588	0.040433041	291	16	313
Cortisol	Enhanced	early	0.5434475	0.084437852	291	16	313
Cortisol	Enhanced	mid	0.4457375	0.040194982	291	16	313
Cortisol	Enhanced	late	0.422766744	0.029991294	291	16	313
Cortisol	Standard	pre	0.358788061	0.03522457	291	16	313
Cortisol	Standard	early	0.5844425	0.063308002	291	16	313
Cortisol	Standard	mid	0.5085925	0.041570031	291	16	313
Cortisol	Standard	late	0.461442321	0.039107402	291	16	313

Table S11: Average Individual Leukocyte Subsets. All values are reported in relative percentages.

Subset	Population	SURFACE_MARKERS	Measurement	Menu	Time	Estimate	StdErr	DF	Nsubjects	Nobs
Leukocyte Subsets	Granulocytes	CD45+ CD14- (scatter)	Granulocytes	Enhanced	pre	56.3625	3.397997457	56	16	78
Leukocyte Subsets	Granulocytes	CD45+ CD14- (scatter)	Granulocytes	Enhanced	early	41.425	5.785103445	56	16	78
Leukocyte Subsets	Granulocytes	CD45+ CD14- (scatter)	Granulocytes	Enhanced	mid	45.35	2.942416099	56	16	78
Leukocyte Subsets	Granulocytes	CD45+ CD14- (scatter)	Granulocytes	Enhanced	late	41.8875	3.09199761	56	16	78
Leukocyte Subsets	Granulocytes	CD45+ CD14- (scatter)	Granulocytes	Standard	pre	50.78691848	2.120506156	56	16	78
Leukocyte Subsets	Granulocytes	CD45+ CD14- (scatter)	Granulocytes	Standard	early	44.9125	4.274248381	56	16	78
Leukocyte Subsets	Granulocytes	CD45+ CD14- (scatter)	Granulocytes	Standard	mid	44.75768245	5.196018534	56	16	78
Leukocyte Subsets	Granulocytes	CD45+ CD14- (scatter)	Granulocytes	Standard	late	51.8375	2.709470514	56	16	78
Leukocyte Subsets	Monocytes	CD45+ CD14+	Monocytes	Enhanced	pre	6.125	0.605444052	58	16	80
Leukocyte Subsets	Monocytes	CD45+ CD14+	Monocytes	Enhanced	early	5.075	0.565616367	58	16	80
Leukocyte Subsets	Monocytes	CD45+ CD14+	Monocytes	Enhanced	mid	5.35	0.378731435	58	16	80
Leukocyte Subsets	Monocytes	CD45+ CD14+	Monocytes	Enhanced	late	5.0375	0.461971827	58	16	80
Leukocyte Subsets	Monocytes	CD45+ CD14+	Monocytes	Standard	pre	5.86875	0.361414052	58	16	80
Leukocyte Subsets	Monocytes	CD45+ CD14+	Monocytes	Standard	early	6.6625	0.704436277	58	16	80
Leukocyte Subsets	Monocytes	CD45+ CD14+	Monocytes	Standard	mid	5.3	0.901041065	58	16	80
Leukocyte Subsets	Monocytes	CD45+ CD14+	Monocytes	Standard	late	7.425	0.427839777	58	16	80
Leukocyte Subsets	Lymphocytes	CD45+ CD14- (scatter)	Lymphocytes	Enhanced	pre	35.66875	3.026225701	56	16	78
Leukocyte Subsets	Lymphocytes	CD45+ CD14- (scatter)	Lymphocytes	Enhanced	early	52.575	6.407094457	56	16	78
Leukocyte Subsets	Lymphocytes	CD45+ CD14- (scatter)	Lymphocytes	Enhanced	mid	48.5125	3.136002506	56	16	78
Leukocyte Subsets	Lymphocytes	CD45+ CD14- (scatter)	Lymphocytes	Enhanced	late	52.0875	3.456218919	56	16	78
Leukocyte Subsets	Lymphocytes	CD45+ CD14- (scatter)	Lymphocytes	Standard	pre	42.29716373	2.314611353	56	16	78
Leukocyte Subsets	Lymphocytes	CD45+ CD14- (scatter)	Lymphocytes	Standard	early	47.725	4.898174086	56	16	78
Leukocyte Subsets	Lymphocytes	CD45+ CD14- (scatter)	Lymphocytes	Standard	mid	49.19392228	5.917869108	56	16	78
Leukocyte Subsets	Lymphocytes	CD45+ CD14- (scatter)	Lymphocytes	Standard	late	39.5	3.006815176	56	16	78
Lymphocyte Subsets	T Cells	CD3+	T Cells	Enhanced	pre	67.18376559	2.170429411	54	16	76
Lymphocyte Subsets	T Cells	CD3+	T Cells	Enhanced	early	70	1.719193125	54	16	76
Lymphocyte Subsets	T Cells	CD3+	T Cells	Enhanced	mid	72	2.137609646	54	16	76
Lymphocyte Subsets	T Cells	CD3+	T Cells	Enhanced	late	72.1875	2.673915522	54	16	76
Lymphocyte Subsets	T Cells	CD3+	T Cells	Standard	pre	73.475	1.535466134	54	16	76
Lymphocyte Subsets	T Cells	CD3+	T Cells	Standard	early	70.4125	2.472058701	54	16	76
Lymphocyte Subsets	T Cells	CD3+	T Cells	Standard	mid	67.85	1.298677212	54	16	76
Lymphocyte Subsets	T Cells	CD3+	T Cells	Standard	late	70.925	1.421789146	54	16	76
Lymphocyte Subsets	B Cells	CD19+	B Cells	Enhanced	pre	11.45	1.286832351	58	16	80
Lymphocyte Subsets	B Cells	CD19+	B Cells	Enhanced	early	13.6125	1.4504781	58	16	80
Lymphocyte Subsets	B Cells	CD19+	B Cells	Enhanced	mid	13.075	1.452772135	58	16	80
Lymphocyte Subsets	B Cells	CD19+	B Cells	Enhanced	late	13.6625	1.481546648	58	16	80
Lymphocyte Subsets	B Cells	CD19+	B Cells	Standard	pre	12.3875	1.53092471	58	16	80
Lymphocyte Subsets	B Cells	CD19+	B Cells	Standard	early	13.2375	1.81400261	58	16	80
Lymphocyte Subsets	B Cells	CD19+	B Cells	Standard	mid	12.1	1.466394388	58	16	80
Lymphocyte Subsets	B Cells	CD19+	B Cells	Standard	late	12.6625	1.565241345	58	16	80
Lymphocyte Subsets	NK Cells	CD16/56+	NK Cells	Enhanced	pre	17.21875	0.786156468	58	16	80
Lymphocyte Subsets	NK Cells	CD16/56+	NK Cells	Enhanced	early	15.7	1.063455453	58	16	80
Lymphocyte Subsets	NK Cells	CD16/56+	NK Cells	Enhanced	mid	14.0125	1.616655257	58	16	80
Lymphocyte Subsets	NK Cells	CD16/56+	NK Cells	Enhanced	late	12.6875	1.721140383	58	16	80
Lymphocyte Subsets	NK Cells	CD16/56+	NK Cells	Standard	pre	15.06875	1.051373265	58	16	80
Lymphocyte Subsets	NK Cells	CD16/56+	NK Cells	Standard	early	13.2125	0.764329261	58	16	80
Lymphocyte Subsets	NK Cells	CD16/56+	NK Cells	Standard	mid	14.3375	0.868176519	58	16	80
Lymphocyte Subsets	NK Cells	CD16/56+	NK Cells	Standard	late	12.975	1.230567704	58	16	80
T Cell Subsets	Helper T Cells	CD3+ CD4+	CD4+	Enhanced	pre	64.7125	2.422062493	58	16	80
T Cell Subsets	Helper T Cells	CD3+ CD4+	CD4+	Enhanced	early	66.3625	2.590182034	58	16	80
T Cell Subsets	Helper T Cells	CD3+ CD4+	CD4+	Enhanced	mid	66.475	3.140747821	58	16	80
T Cell Subsets	Helper T Cells	CD3+ CD4+	CD4+	Enhanced	late	67.325	2.817953047	58	16	80
T Cell Subsets	Helper T Cells	CD3+ CD4+	CD4+	Standard	pre	61.4125	2.488328223	58	16	80
T Cell Subsets	Helper T Cells	CD3+ CD4+	CD4+	Standard	early	63.0875	2.33635918	58	16	80
T Cell Subsets	Helper T Cells	CD3+ CD4+	CD4+	Standard	mid	63.325	2.455399026	58	16	80
T Cell Subsets	Helper T Cells	CD3+ CD4+	CD4+	Standard	late	63.4875	2.453341949	58	16	80
T Cell Subsets	Cytotoxic T Cells	CD3+ CD8+	CD8+	Enhanced	pre	29.225	2.160927897	58	16	80
T Cell Subsets	Cytotoxic T Cells	CD3+ CD8+	CD8+	Enhanced	early	28.1625	2.367681982	58	16	80
T Cell Subsets	Cytotoxic T Cells	CD3+ CD8+	CD8+	Enhanced	mid	27.6875	2.603929573	58	16	80
T Cell Subsets	Cytotoxic T Cells	CD3+ CD8+	CD8+	Enhanced	late	27.7625	2.54999617	58	16	80
T Cell Subsets	Cytotoxic T Cells	CD3+ CD8+	CD8+	Standard	pre	31.05625	1.587732205	58	16	80
T Cell Subsets	Cytotoxic T Cells	CD3+ CD8+	CD8+	Standard	early	29.725	1.398632144	58	16	80
T Cell Subsets	Cytotoxic T Cells	CD3+ CD8+	CD8+	Standard	mid	30.0875	1.705820688	58	16	80
T Cell Subsets	Cytotoxic T Cells	CD3+ CD8+	CD8+	Standard	late	28.475	1.57279111	58	16	80
Memory CD4	Bulk Naive	CD4+ CD45RA+	CD4+ CD45RA Bulk Naive	Enhanced	pre	50.00625	4.3517367	55	16	77
Memory CD4	Bulk Naive	CD4+ CD45RA+	CD4+ CD45RA Bulk Naive	Enhanced	early	51.2	4.165633205	55	16	77
Memory CD4	Bulk Naive	CD4+ CD45RA+	CD4+ CD45RA Bulk Naive	Enhanced	mid	51.4125	3.944435856	55	16	77
Memory CD4	Bulk Naive	CD4+ CD45RA+	CD4+ CD45RA Bulk Naive	Enhanced	late	47.53020499	5.002026554	55	16	77
Memory CD4	Bulk Naive	CD4+ CD45RA+	CD4+ CD45RA Bulk Naive	Standard	pre	40.46071073	4.660794797	55	16	77
Memory CD4	Bulk Naive	CD4+ CD45RA+	CD4+ CD45RA Bulk Naive	Standard	early	40.9375	4.562754702	55	16	77
Memory CD4	Bulk Naive	CD4+ CD45RA+	CD4+ CD45RA Bulk Naive	Standard	mid	43.2125	5.62970463	55	16	77
Memory CD4	Bulk Naive	CD4+ CD45RA+	CD4+ CD45RA Bulk Naive	Standard	late	41.675	4.850346315	55	16	77
Memory CD4	Bulk Memory	CD4+ CD45RO+	CD4+ CD45RO Bulk Memory	Enhanced	pre	49.98125	4.354235882	54	16	76
Memory CD4	Bulk Memory	CD4+ CD45RO+	CD4+ CD45RO Bulk Memory	Enhanced	early	48.775	4.174017474	54	16	76
Memory CD4	Bulk Memory	CD4+ CD45RO+	CD4+ CD45RO Bulk Memory	Enhanced	mid	48.5875	3.942652878	54	16	76
Memory CD4	Bulk Memory	CD4+ CD45RO+	CD4+ CD45RO Bulk Memory	Enhanced	late	52.43796722	5.005384242	54	16	76
Memory CD4	Bulk Memory	CD4+ CD45RO+	CD4+ CD45RO Bulk Memory	Standard	pre	59.39315131	4.677695444	54	16	76
Memory CD4	Bulk Memory	CD4+ CD45RO+	CD4+ CD45RO Bulk Memory	Standard	early	59.0875	4.561162047	54	16	76
Memory CD4	Bulk Memory	CD4+ CD45RO+	CD4+ CD45RO Bulk Memory	Standard	mid	55.55	4.839938016	54	16	76
Memory CD4	Bulk Memory	CD4+ CD45RO+	CD4+ CD45RO Bulk Memory	Standard	late	57.22623924	4.929268044	54	16	76
Memory CD8	Bulk Naive	CD8+ CD45RA+	CD8+ CD45RA Bulk Naive	Enhanced	pre	57.5875	3.665961994	55	16	77
Memory CD8	Bulk Naive	CD8+ CD45RA+	CD8+ CD45RA Bulk Naive	Enhanced	early	58.05	4.353303343	55	16	77
Memory CD8	Bulk Naive	CD8+ CD45RA+	CD8+ CD45RA Bulk Naive	Enhanced	mid	57.875	4.469121488	55	16	77
Memory CD8	Bulk Naive	CD8+ CD45RA+	CD8+ CD45RA Bulk Naive	Enhanced	late	54.9375	5.107345736	55	16	77
Memory CD8	Bulk Naive	CD8+ CD45RA+	CD8+ CD45RA Bulk Naive	Standard	pre	51.46130821	6.332719643	55	16	77
Memory CD8	Bulk Naive	CD8+ CD45RA+	CD8+ CD45RA Bulk Naive	Standard	early	52.8625	6.303915884	55	16	77
Memory CD8	Bulk Naive	CD8+ CD45RA+	CD8+ CD45RA Bulk Naive	Standard	mid	55.6	6.188522643	55	16	77
Memory CD8	Bulk Naive	CD8+ CD45RA+	CD8+ CD45RA Bulk Naive	Standard	late	53.7207466	5.682793549	55	16	77
Memory CD8	Bulk Memory	CD8+ CD45RO+	CD8+ CD45RO Bulk Memory	Enhanced	pre	42.4875	3.65842083	55	16	77
Memory CD8	Bulk Memory	CD8+ CD45RO+	CD8+ CD45RO Bulk Memory	Enhanced	early	41.9375	4.355956321	55	16	77
Memory CD8	Bulk Memory	CD8+ CD45RO+	CD8+ CD45RO Bulk Memory	Enhanced	mid	42.125	4.469121488	55	16	77
Memory CD8	Bulk Memory	CD8+ CD45RO+	CD8+ CD45RO Bulk Memory	Enhanced	late	45.05	5.107531204	55	16	77

Memory CD8	Bulk Memory	CD8+ CD45RO+	CD8+ CD45RO Bulk Memory	Standard	pre	48.50947059	6.34591221	55	16	77
Memory CD8	Bulk Memory	CD8+ CD45RO+	CD8+ CD45RO Bulk Memory	Standard	early	47.1125	6.309428399	55	16	77
Memory CD8	Bulk Memory	CD8+ CD45RO+	CD8+ CD45RO Bulk Memory	Standard	mid	44.3125	6.235945134	55	16	77
Memory CD8	Bulk Memory	CD8+ CD45RO+	CD8+ CD45RO Bulk Memory	Standard	late	46.12456147	5.784580553	55	16	77
Central Memory CD4	True Naïve	CD4+/CD45RA+/CD62L+	CD4+/CD45RA+/CD62L+ TRUE NAÏVE	Enhanced	pre	35.23125	7.220462121	51	16	73
Central Memory CD4	True Naïve	CD4+/CD45RA+/CD62L+	CD4+/CD45RA+/CD62L+ TRUE NAÏVE	Enhanced	early	46.66128442	6.088350845	51	16	73
Central Memory CD4	True Naïve	CD4+/CD45RA+/CD62L+	CD4+/CD45RA+/CD62L+ TRUE NAÏVE	Enhanced	mid	47.10414157	6.192745925	51	16	73
Central Memory CD4	True Naïve	CD4+/CD45RA+/CD62L+	CD4+/CD45RA+/CD62L+ TRUE NAÏVE	Enhanced	late	45.40414157	6.211546983	51	16	73
Central Memory CD4	True Naïve	CD4+/CD45RA+/CD62L+	CD4+/CD45RA+/CD62L+ TRUE NAÏVE	Standard	pre	31.47279705	4.352355684	51	16	73
Central Memory CD4	True Naïve	CD4+/CD45RA+/CD62L+	CD4+/CD45RA+/CD62L+ TRUE NAÏVE	Standard	early	31.15	4.201376263	51	16	73
Central Memory CD4	True Naïve	CD4+/CD45RA+/CD62L+	CD4+/CD45RA+/CD62L+ TRUE NAÏVE	Standard	mid	30.75	5.700411169	51	16	73
Central Memory CD4	True Naïve	CD4+/CD45RA+/CD62L+	CD4+/CD45RA+/CD62L+ TRUE NAÏVE	Standard	late	33.0375	4.254884601	51	16	73
Central Memory CD4	Central Memory	CD4+/CD45RA-/CD62L+	CD4+/CD45RA-/CD62L+ CENTRAL MEMORY	Enhanced	pre	44.2375	6.130856575	51	16	73
Central Memory CD4	Central Memory	CD4+/CD45RA-/CD62L+	CD4+/CD45RA-/CD62L+ CENTRAL MEMORY	Enhanced	early	35.11629027	5.233838726	51	16	73
Central Memory CD4	Central Memory	CD4+/CD45RA-/CD62L+	CD4+/CD45RA-/CD62L+ CENTRAL MEMORY	Enhanced	mid	36.1448617	5.186455941	51	16	73
Central Memory CD4	Central Memory	CD4+/CD45RA-/CD62L+	CD4+/CD45RA-/CD62L+ CENTRAL MEMORY	Enhanced	late	36.81629027	5.595024014	51	16	73
Central Memory CD4	Central Memory	CD4+/CD45RA-/CD62L+	CD4+/CD45RA-/CD62L+ CENTRAL MEMORY	Standard	pre	51.82967753	3.784541783	51	16	73
Central Memory CD4	Central Memory	CD4+/CD45RA-/CD62L+	CD4+/CD45RA-/CD62L+ CENTRAL MEMORY	Standard	early	52.6	4.052699717	51	16	73
Central Memory CD4	Central Memory	CD4+/CD45RA-/CD62L+	CD4+/CD45RA-/CD62L+ CENTRAL MEMORY	Standard	mid	53.5	5.294041462	51	16	73
Central Memory CD4	Central Memory	CD4+/CD45RA-/CD62L+	CD4+/CD45RA-/CD62L+ CENTRAL MEMORY	Standard	late	52.6125	3.95527486	51	16	73
Central Memory CD4	Effector Memory	CD4+/CD45RA-/CD62L-	CD4+/CD45RA-/CD62L- EFFECTOR MEMORY	Enhanced	pre	17.725	2.335159256	50	16	72
Central Memory CD4	Effector Memory	CD4+/CD45RA-/CD62L-	CD4+/CD45RA-/CD62L- EFFECTOR MEMORY	Enhanced	early	14.63464512	2.285930912	50	16	72
Central Memory CD4	Effector Memory	CD4+/CD45RA-/CD62L-	CD4+/CD45RA-/CD62L- EFFECTOR MEMORY	Enhanced	mid	14.30607369	2.305070111	50	16	72
Central Memory CD4	Effector Memory	CD4+/CD45RA-/CD62L-	CD4+/CD45RA-/CD62L- EFFECTOR MEMORY	Enhanced	late	15.06321655	2.481412509	50	16	72
Central Memory CD4	Effector Memory	CD4+/CD45RA-/CD62L-	CD4+/CD45RA-/CD62L- EFFECTOR MEMORY	Standard	pre	15.94326029	1.725015134	50	16	72
Central Memory CD4	Effector Memory	CD4+/CD45RA-/CD62L-	CD4+/CD45RA-/CD62L- EFFECTOR MEMORY	Standard	early	15.6	1.592070193	50	16	72
Central Memory CD4	Effector Memory	CD4+/CD45RA-/CD62L-	CD4+/CD45RA-/CD62L- EFFECTOR MEMORY	Standard	mid	15.175	1.612524225	50	16	72
Central Memory CD4	Effector Memory	CD4+/CD45RA-/CD62L-	CD4+/CD45RA-/CD62L- EFFECTOR MEMORY	Standard	late	13.64289851	1.532952931	50	16	72
Central Memory CD4	Terminally Differentiated	CD4+/CD45RA+/CD62L-	CD4+/CD45RA+/CD62L- TERM. DIFF.	Enhanced	pre	2.7875	1.023693909	51	16	73
Central Memory CD4	Terminally Differentiated	CD4+/CD45RA+/CD62L-	CD4+/CD45RA+/CD62L- TERM. DIFF.	Enhanced	early	3.535880586	1.274719665	51	16	73
Central Memory CD4	Terminally Differentiated	CD4+/CD45RA+/CD62L-	CD4+/CD45RA+/CD62L- TERM. DIFF.	Enhanced	mid	2.378737729	0.95264971	51	16	73
Central Memory CD4	Terminally Differentiated	CD4+/CD45RA+/CD62L-	CD4+/CD45RA+/CD62L- TERM. DIFF.	Enhanced	late	2.707309158	0.9753685	51	16	73
Central Memory CD4	Terminally Differentiated	CD4+/CD45RA+/CD62L-	CD4+/CD45RA+/CD62L- TERM. DIFF.	Standard	pre	0.769499442	0.185655419	51	16	73
Central Memory CD4	Terminally Differentiated	CD4+/CD45RA+/CD62L-	CD4+/CD45RA+/CD62L- TERM. DIFF.	Standard	early	0.6625	0.176721444	51	16	73
Central Memory CD4	Terminally Differentiated	CD4+/CD45RA+/CD62L-	CD4+/CD45RA+/CD62L- TERM. DIFF.	Standard	mid	0.575	0.135496771	51	16	73
Central Memory CD4	Terminally Differentiated	CD4+/CD45RA+/CD62L-	CD4+/CD45RA+/CD62L- TERM. DIFF.	Standard	late	0.675	0.241576851	51	16	73
Central Memory CD8	True Naïve	CD8+/CD45RA+/CD62L+	CD8+/CD45RA+/CD62L+ TRUE NAÏVE	Enhanced	pre	36.7375	3.058156997	51	16	73
Central Memory CD8	True Naïve	CD8+/CD45RA+/CD62L+	CD8+/CD45RA+/CD62L+ TRUE NAÏVE	Enhanced	early	42.1829937	4.027731488	51	16	73
Central Memory CD8	True Naïve	CD8+/CD45RA+/CD62L+	CD8+/CD45RA+/CD62L+ TRUE NAÏVE	Enhanced	mid	45.02585085	4.270331045	51	16	73
Central Memory CD8	True Naïve	CD8+/CD45RA+/CD62L+	CD8+/CD45RA+/CD62L+ TRUE NAÏVE	Enhanced	late	43.64013656	3.814675907	51	16	73
Central Memory CD8	True Naïve	CD8+/CD45RA+/CD62L+	CD8+/CD45RA+/CD62L+ TRUE NAÏVE	Standard	pre	43.76247959	4.862856277	51	16	73
Central Memory CD8	True Naïve	CD8+/CD45RA+/CD62L+	CD8+/CD45RA+/CD62L+ TRUE NAÏVE	Standard	early	47.55	5.457391776	51	16	73
Central Memory CD8	True Naïve	CD8+/CD45RA+/CD62L+	CD8+/CD45RA+/CD62L+ TRUE NAÏVE	Standard	mid	48.125	5.771258258	51	16	73
Central Memory CD8	True Naïve	CD8+/CD45RA+/CD62L+	CD8+/CD45RA+/CD62L+ TRUE NAÏVE	Standard	late	46.925	5.911687312	51	16	73
Central Memory CD8	Central Memory	CD8+/CD45RA-/CD62L+	CD8+/CD45RA-/CD62L+ CENTRAL MEMORY	Enhanced	pre	17.425	3.939374554	51	16	73
Central Memory CD8	Central Memory	CD8+/CD45RA-/CD62L+	CD8+/CD45RA-/CD62L+ CENTRAL MEMORY	Enhanced	early	13.56307882	3.26219753	51	16	73
Central Memory CD8	Central Memory	CD8+/CD45RA-/CD62L+	CD8+/CD45RA-/CD62L+ CENTRAL MEMORY	Enhanced	mid	14.54879311	3.904110385	51	16	73
Central Memory CD8	Central Memory	CD8+/CD45RA-/CD62L+	CD8+/CD45RA-/CD62L+ CENTRAL MEMORY	Enhanced	late	15.02022168	3.817832909	51	16	73
Central Memory CD8	Central Memory	CD8+/CD45RA-/CD62L+	CD8+/CD45RA-/CD62L+ CENTRAL MEMORY	Standard	pre	18.14466467	5.124301637	51	16	73
Central Memory CD8	Central Memory	CD8+/CD45RA-/CD62L+	CD8+/CD45RA-/CD62L+ CENTRAL MEMORY	Standard	early	13.4	1.459559009	51	16	73
Central Memory CD8	Central Memory	CD8+/CD45RA-/CD62L+	CD8+/CD45RA-/CD62L+ CENTRAL MEMORY	Standard	mid	12.875	1.326267083	51	16	73
Central Memory CD8	Central Memory	CD8+/CD45RA-/CD62L+	CD8+/CD45RA-/CD62L+ CENTRAL MEMORY	Standard	late	14.275	1.709874813	51	16	73
Central Memory CD8	Effector Memory	CD8+/CD45RA-/CD62L-	CD8+/CD45RA-/CD62L- EFFECTOR MEMORY	Enhanced	pre	28.20625	4.482469652	51	16	73
Central Memory CD8	Effector Memory	CD8+/CD45RA-/CD62L-	CD8+/CD45RA-/CD62L- EFFECTOR MEMORY	Enhanced	early	23.34808285	4.284236701	51	16	73
Central Memory CD8	Effector Memory	CD8+/CD45RA-/CD62L-	CD8+/CD45RA-/CD62L- EFFECTOR MEMORY	Enhanced	mid	24.11951142	5.318942127	51	16	73
Central Memory CD8	Effector Memory	CD8+/CD45RA-/CD62L-	CD8+/CD45RA-/CD62L- EFFECTOR MEMORY	Enhanced	late	24.67665428	4.758083599	51	16	73
Central Memory CD8	Effector Memory	CD8+/CD45RA-/CD62L-	CD8+/CD45RA-/CD62L- EFFECTOR MEMORY	Standard	pre	28.54394897	2.942437838	51	16	73
Central Memory CD8	Effector Memory	CD8+/CD45RA-/CD62L-	CD8+/CD45RA-/CD62L- EFFECTOR MEMORY	Standard	early	31.05	5.064552053	51	16	73
Central Memory CD8	Effector Memory	CD8+/CD45RA-/CD62L-	CD8+/CD45RA-/CD62L- EFFECTOR MEMORY	Standard	mid	29.8875	5.578430489	51	16	73
Central Memory CD8	Effector Memory	CD8+/CD45RA-/CD62L-	CD8+/CD45RA-/CD62L- EFFECTOR MEMORY	Standard	late	31.15	5.187002988	51	16	73
Central Memory CD8	Terminally Differentiated	CD8+/CD45RA+/CD62L-	CD8+/CD45RA+/CD62L- TERM. DIFF.	Enhanced	pre	17.63125	4.704576707	51	16	73
Central Memory CD8	Terminally Differentiated	CD8+/CD45RA+/CD62L-	CD8+/CD45RA+/CD62L- TERM. DIFF.	Enhanced	early	21.91266497	4.424590539	51	16	73
Central Memory CD8	Terminally Differentiated	CD8+/CD45RA+/CD62L-	CD8+/CD45RA+/CD62L- TERM. DIFF.	Enhanced	mid	17.31266497	4.749492683	51	16	73
Central Memory CD8	Terminally Differentiated	CD8+/CD45RA+/CD62L-	CD8+/CD45RA+/CD62L- TERM. DIFF.	Enhanced	late	17.6412364	4.37165621	51	16	73
Central Memory CD8	Terminally Differentiated	CD8+/CD45RA+/CD62L-	CD8+/CD45RA+/CD62L- TERM. DIFF.	Standard	pre	9.37191013	2.107640284	51	16	73
Central Memory CD8	Terminally Differentiated	CD8+/CD45RA+/CD62L-	CD8+/CD45RA+/CD62L- TERM. DIFF.	Standard	early	8	1.41178079	51	16	73
Central Memory CD8	Terminally Differentiated	CD8+/CD45RA+/CD62L-	CD8+/CD45RA+/CD62L- TERM. DIFF.	Standard	mid	9.1375	1.779829477	51	16	73
Central Memory CD8	Terminally Differentiated	CD8+/CD45RA+/CD62L-	CD8+/CD45RA+/CD62L- TERM. DIFF.	Standard	late	7.65	1.256358826	51	16	73
Activated T Cells	Activated CD4 T cells	CD4+ HLA-DR+	CD4+ HLA-DR	Enhanced	pre	3.919183201	0.740775161	50	16	72
Activated T Cells	Activated CD4 T cells	CD4+ HLA-DR+	CD4+ HLA-DR	Enhanced	early	3.0375	0.51597526	50	16	72
Activated T Cells	Activated CD4 T cells	CD4+ HLA-DR+	CD4+ HLA-DR	Enhanced	mid	3.2375	0.547989935	50	16	72
Activated T Cells	Activated CD4 T cells	CD4+ HLA-DR+	CD4+ HLA-DR	Enhanced	late	3.241351955	0.533267257	50	16	72
Activated T Cells	Activated CD4 T cells	CD4+ HLA-DR+	CD4+ HLA-DR	Standard	pre	5.323547519	0.568393386	50	16	72
Activated T Cells	Activated CD4 T cells	CD4+ HLA-DR+	CD4+ HLA-DR	Standard	early	4.0375	0.511718154	50	16	72
Activated T Cells	Activated CD4 T cells	CD4+ HLA-DR+	CD4+ HLA-DR	Standard	mid	3.375	0.491092532	50	16	72
Activated T Cells	Activated CD4 T cells	CD4+ HLA-DR+	CD4+ HLA-DR	Standard	late	3.667303241	0.416825615	50	16	72
Activated T Cells	Activated CD8 T cells	CD8+ HLA-DR+	CD8+ HLA-DR	Enhanced	pre	8.603237788	2.483131437	49	16	71
Activated T Cells	Activated CD8 T cells	CD8+ HLA-DR+	CD8+ HLA-DR	Enhanced	early	8.7875	2.681967416	49	16	71
Activated T Cells	Activated CD8 T cells	CD8+ HLA-DR+	CD8+ HLA-DR	Enhanced	mid	9.058625152	2.7104897	49	16	71
Activated T Cells	Activated CD8 T cells	CD8+ HLA-DR+	CD8+ HLA-DR	Enhanced	late	9.509406998	2.689145119	49	16	71
Activated T Cells	Activated CD8 T cells	CD8+ HLA-DR+	CD8+ HLA-DR	Standard	pre	8.008625169	1.764868386	49	16	71
Activated T Cells	Activated CD8 T cells	CD8+ HLA-DR+	CD8+ HLA-DR	Standard	early	5.275	1.504861912	49	16	71
Activated T Cells	Activated CD8 T cells	CD8+ HLA-DR+	CD8+ HLA-DR	Standard	mid	5.4625	1.334393296	49	16	71
Activated T Cells	Activated CD8 T cells	CD8+ HLA-DR+	CD8+ HLA-DR	Standard	late	5.571344849	1.3684388	49	16	71
Regulatory T Cells	Regulatory T cells	CD4+ CD25+ CD127-	TReg	Enhanced	pre	8.04375	0.243419247	58	16	80
Regulatory T Cells	Regulatory T cells	CD4+ CD25+ CD127-	TReg	Enhanced	early	7.0125	0.661068619	58	16	80
Regulatory T Cells	Regulatory T cells	CD4+ CD25+ CD127-	TReg	Enhanced	mid	6.9	0.719374728	58	16	80
Regulatory T Cells	Regulatory T cells	CD4+ CD25+ CD127-	TReg	Enhanced	late	7.3625	0.760743366	58	16	80
Regulatory T Cells	Regulatory T cells	CD4+ CD25+ CD127-	TReg	Standard	pre	7.0625	0.293916176	58	16	80
Regulatory T Cells	Regulatory T cells	CD4+ CD25+ CD127-	TReg	Standard	early	8.35	0.845761491	58	16	80
Regulatory T Cells	Regulatory T cells	CD4+ CD25+ CD127-	TReg	Standard	mid	8.3375	0.806213662	58	16	80
Regulatory T Cells	Regulatory T cells	CD4+ CD25+ CD127-	TReg	Standard	late	7.025	0.792296898	58	16	80
B Cell Subsets	Naïve B Cells	CD19+ CD27- IgD+	CD19+ IgD+	Enhanced	pre	75.30625	2.330762977	58	16	80
B Cell Subsets	Naïve B Cells	CD19+ CD27- IgD+	CD19+ IgD+	Enhanced	early	75.5	2.851151083	58	16	80

B Cell Subsets	Naïve B Cells	CD19+ CD27- IgD+	CD19+ IgD+	Enhanced	mid	74.0375	2.869720016	58	16	80
B Cell Subsets	Naïve B Cells	CD19+ CD27- IgD+	CD19+ IgD+	Enhanced	late	73.4125	3.153095577	58	16	80
B Cell Subsets	Naïve B Cells	CD19+ CD27- IgD+	CD19+ IgD+	Standard	pre	76.63125	2.903647188	58	16	80
B Cell Subsets	Naïve B Cells	CD19+ CD27- IgD+	CD19+ IgD+	Standard	early	77.1125	3.014518903	58	16	80
B Cell Subsets	Naïve B Cells	CD19+ CD27- IgD+	CD19+ IgD+	Standard	mid	77.3375	3.356753337	58	16	80
B Cell Subsets	Naïve B Cells	CD19+ CD27- IgD+	CD19+ IgD+	Standard	late	74.6375	3.326688364	58	16	80
B Cell Subsets	Unswitched Memory B Cells	CD19+ CD27+ IgD+	CD19+ CD27+ IgD+	Enhanced	pre	7.8625	1.601921454	58	16	80
B Cell Subsets	Unswitched Memory B Cells	CD19+ CD27+ IgD+	CD19+ CD27+ IgD+	Enhanced	early	7.8875	1.940476544	58	16	80
B Cell Subsets	Unswitched Memory B Cells	CD19+ CD27+ IgD+	CD19+ CD27+ IgD+	Enhanced	mid	8.4875	1.631854534	58	16	80
B Cell Subsets	Unswitched Memory B Cells	CD19+ CD27+ IgD+	CD19+ CD27+ IgD+	Enhanced	late	8.9625	2.018116193	58	16	80
B Cell Subsets	Unswitched Memory B Cells	CD19+ CD27+ IgD+	CD19+ CD27+ IgD+	Standard	pre	4.44375	0.711371777	58	16	80
B Cell Subsets	Unswitched Memory B Cells	CD19+ CD27+ IgD+	CD19+ CD27+ IgD+	Standard	early	4	0.752911017	58	16	80
B Cell Subsets	Unswitched Memory B Cells	CD19+ CD27+ IgD+	CD19+ CD27+ IgD+	Standard	mid	4.125	0.612818795	58	16	80
B Cell Subsets	Unswitched Memory B Cells	CD19+ CD27+ IgD+	CD19+ CD27+ IgD+	Standard	late	9.125	2.655992635	58	16	80
B Cell Subsets	Switched Memory	CD19+ CD27+ IgD-	CD19+ CD27+	Enhanced	pre	10.125	0.729136904	58	16	80
B Cell Subsets	Switched Memory	CD19+ CD27+ IgD-	CD19+ CD27+	Enhanced	early	10.25	1.042233179	58	16	80
B Cell Subsets	Switched Memory	CD19+ CD27+ IgD-	CD19+ CD27+	Enhanced	mid	10.8875	0.939820046	58	16	80
B Cell Subsets	Switched Memory	CD19+ CD27+ IgD-	CD19+ CD27+	Enhanced	late	11.5375	1.287188979	58	16	80
B Cell Subsets	Switched Memory	CD19+ CD27+ IgD-	CD19+ CD27+	Standard	pre	10.18125	2.021832737	58	16	80
B Cell Subsets	Switched Memory	CD19+ CD27+ IgD-	CD19+ CD27+	Standard	early	9.05	1.764847727	58	16	80
B Cell Subsets	Switched Memory	CD19+ CD27+ IgD-	CD19+ CD27+	Standard	mid	9.225	1.878808632	58	16	80
B Cell Subsets	Switched Memory	CD19+ CD27+ IgD-	CD19+ CD27+	Standard	late	8.4375	1.318609293	58	16	80

Table S12: Average Individual Plasma Cytokines. All values are reported in pg/ml.

Measurement	Menu	Time	Estimate	StdErr	DF	Nsubjects	Nobs
EGF	Enhanced	pre	19.184375	4.229019024	58	16	80
EGF	Enhanced	early	13.75375	3.53261807	58	16	80
EGF	Enhanced	mid	18.84625	5.168288201	58	16	80
EGF	Enhanced	late	17.6675	4.280916137	58	16	80
EGF	Standard	pre	44.194375	14.83485682	58	16	80
EGF	Standard	early	31.4825	9.482442616	58	16	80
EGF	Standard	mid	45.41125	16.4100427	58	16	80
EGF	Standard	late	55.90125	13.98467782	58	16	80
Eotaxin	Enhanced	pre	81.4875	5.664708041	58	16	80
Eotaxin	Enhanced	early	80.49375	5.11651277	58	16	80
Eotaxin	Enhanced	mid	79.1325	5.599993792	58	16	80
Eotaxin	Enhanced	late	78.00625	6.005142051	58	16	80
Eotaxin	Standard	pre	86.050625	10.42765892	58	16	80
Eotaxin	Standard	early	82.9775	9.186100531	58	16	80
Eotaxin	Standard	mid	83.84625	8.519663091	58	16	80
Eotaxin	Standard	late	76.5775	8.422067136	58	16	80
G-CSF	Enhanced	pre	27.16375	6.4405546	58	16	80
G-CSF	Enhanced	early	20.4775	7.627368229	58	16	80
G-CSF	Enhanced	mid	31.0925	7.818588777	58	16	80
G-CSF	Enhanced	late	29.87375	8.888568139	58	16	80
G-CSF	Standard	pre	53.49125	29.10879729	58	16	80
G-CSF	Standard	early	47.07	29.02258442	58	16	80
G-CSF	Standard	mid	50.44125	27.2690012	58	16	80
G-CSF	Standard	late	56.6525	31.85894717	58	16	80
GM-CSF	Enhanced	pre	15.890625	3.550733797	58	16	80
GM-CSF	Enhanced	early	12.3975	2.973599854	58	16	80
GM-CSF	Enhanced	mid	13.84	3.194224769	58	16	80
GM-CSF	Enhanced	late	14.765	3.523115188	58	16	80
GM-CSF	Standard	pre	34.486875	18.36677901	58	16	80
GM-CSF	Standard	early	29.58	16.00927046	58	16	80
GM-CSF	Standard	mid	30.56375	16.39717648	58	16	80
GM-CSF	Standard	late	30.14875	15.83649459	58	16	80
IFNγ	Enhanced	pre	43.50375	9.95004557	58	16	80
IFNγ	Enhanced	early	40.59125	8.519955332	58	16	80
IFNγ	Enhanced	mid	43.26125	11.39891503	58	16	80
IFNγ	Enhanced	late	42.88875	10.65128109	58	16	80
IFNγ	Standard	pre	218.33875	101.2569201	58	16	80
IFNγ	Standard	early	210.415	96.57544729	58	16	80
IFNγ	Standard	mid	198.365	86.19509773	58	16	80
IFNγ	Standard	late	185.03	93.2503995	58	16	80
IFNa2	Enhanced	pre	64.845625	8.206748863	58	16	80
IFNa2	Enhanced	early	52.505	5.64748921	58	16	80
IFNa2	Enhanced	mid	55.8875	7.302667481	58	16	80
IFNa2	Enhanced	late	55.43125	9.498944586	58	16	80
IFNa2	Standard	pre	95.939375	31.29683042	58	16	80
IFNa2	Standard	early	81.37625	28.42823909	58	16	80
IFNa2	Standard	mid	84.0875	30.59020721	58	16	80
IFNa2	Standard	late	84.50625	28.49802257	58	16	80
IL-10	Enhanced	pre	16.565	4.307441236	58	16	80
IL-10	Enhanced	early	15.45875	5.019112981	58	16	80

IL-10	Enhanced	mid	15.58375	4.152824693	58	16	80
IL-10	Enhanced	late	14.84875	4.411722112	58	16	80
IL-10	Standard	pre	66.336875	48.22215272	58	16	80
IL-10	Standard	early	62.355	45.54279543	58	16	80
IL-10	Standard	mid	58.82	42.57557174	58	16	80
IL-10	Standard	late	56.93	41.41128767	58	16	80
IL-12 p40	Enhanced	pre	50.52	12.05600317	58	16	80
IL-12 p40	Enhanced	early	33.95	11.06253545	58	16	80
IL-12 p40	Enhanced	mid	37.6125	10.991865	58	16	80
IL-12 p40	Enhanced	late	33.3775	11.72362942	58	16	80
IL-12 p40	Standard	pre	118.439375	70.49189819	58	16	80
IL-12 p40	Standard	early	111.96625	68.49296038	58	16	80
IL-12 p40	Standard	mid	99.66	59.40815648	58	16	80
IL-12 p40	Standard	late	106.61625	64.76498092	58	16	80
IL-12 p70	Enhanced	pre	9.028125	1.566476923	58	16	80
IL-12 p70	Enhanced	early	7.66625	1.203564936	58	16	80
IL-12 p70	Enhanced	mid	7.93375	1.417991468	58	16	80
IL-12 p70	Enhanced	late	8.43125	1.631608138	58	16	80
IL-12 p70	Standard	pre	33.740625	24.20103761	58	16	80
IL-12 p70	Standard	early	34.01875	25.48605767	58	16	80
IL-12 p70	Standard	mid	30.245	20.07778444	58	16	80
IL-12 p70	Standard	late	28.84625	20.50382388	58	16	80
IL-13	Enhanced	pre	33.92625	24.45075514	58	16	80
IL-13	Enhanced	early	34.9075	24.78533121	58	16	80
IL-13	Enhanced	mid	35.54125	24.67554929	58	16	80
IL-13	Enhanced	late	35.4575	24.57446119	58	16	80
IL-13	Standard	pre	73.413125	37.75750203	58	16	80
IL-13	Standard	early	75.6125	38.05863243	58	16	80
IL-13	Standard	mid	69.26875	34.01225457	58	16	80
IL-13	Standard	late	73.48	37.86520278	58	16	80
IL-15	Enhanced	pre	7.825	2.602064385	58	16	80
IL-15	Enhanced	early	6.5525	2.263906849	58	16	80
IL-15	Enhanced	mid	6.7775	2.226514056	58	16	80
IL-15	Enhanced	late	6.84125	2.419532779	58	16	80
IL-15	Standard	pre	16.981875	9.601210655	58	16	80
IL-15	Standard	early	16.0075	8.904393184	58	16	80
IL-15	Standard	mid	15.335	7.988989885	58	16	80
IL-15	Standard	late	14.9075	8.490274537	58	16	80
IL-17a	Enhanced	pre	7.280625	1.498202162	58	16	80
IL-17a	Enhanced	early	6.8275	1.306751399	58	16	80
IL-17a	Enhanced	mid	7.02	1.309370078	58	16	80
IL-17a	Enhanced	late	7.28125	1.204140562	58	16	80
IL-17a	Standard	pre	21.431875	7.287380034	58	16	80
IL-17a	Standard	early	21.9375	7.492828811	58	16	80
IL-17a	Standard	mid	21.46125	7.345295569	58	16	80
IL-17a	Standard	late	16.58125	6.170972735	58	16	80
IL-1A	Enhanced	pre	72.006875	50.24457517	58	16	80
IL-1A	Enhanced	early	73.81375	52.42898131	58	16	80
IL-1A	Enhanced	mid	72.955	51.89569593	58	16	80
IL-1A	Enhanced	late	73.7825	52.132239	58	16	80
IL-1A	Standard	pre	137.51	78.78106563	58	16	80
IL-1A	Standard	early	140.63625	80.54319055	58	16	80

IL-1A	Standard	mid	132.93	76.18589395	58	16	80
IL-1A	Standard	late	128.29375	73.90781251	58	16	80
IL-1B	Enhanced	pre	4.2225	0.547918647	58	16	80
IL-1B	Enhanced	early	3.53	0.479107243	58	16	80
IL-1B	Enhanced	mid	3.78375	0.52538955	58	16	80
IL-1B	Enhanced	late	3.74625	0.559002844	58	16	80
IL-1B	Standard	pre	8.96	4.674271919	58	16	80
IL-1B	Standard	early	8.34625	4.377279741	58	16	80
IL-1B	Standard	mid	7.91375	3.838210821	58	16	80
IL-1B	Standard	late	8.03125	4.083215812	58	16	80
IL-1RA	Enhanced	pre	32.784375	7.283061654	58	16	80
IL-1RA	Enhanced	early	28.37625	7.105809229	58	16	80
IL-1RA	Enhanced	mid	30.6	9.065938141	58	16	80
IL-1RA	Enhanced	late	29.35625	8.931851876	58	16	80
IL-1RA	Standard	pre	62.493125	15.90792879	58	16	80
IL-1RA	Standard	early	57.88	14.60572961	58	16	80
IL-1RA	Standard	mid	60.45	14.28354145	58	16	80
IL-1RA	Standard	late	60.58	15.04820888	58	16	80
IL-2	Enhanced	pre	3.31125	0.579518085	58	16	80
IL-2	Enhanced	early	2.86125	0.462014737	58	16	80
IL-2	Enhanced	mid	3.2	0.529994104	58	16	80
IL-2	Enhanced	late	3.34125	0.667477615	58	16	80
IL-2	Standard	pre	10.6	7.424367345	58	16	80
IL-2	Standard	early	10.10625	6.674345927	58	16	80
IL-2	Standard	mid	9.66125	6.328251002	58	16	80
IL-2	Standard	late	9.50125	6.457857965	58	16	80
IL-3	Enhanced	pre	5.864375	0.886051387	58	16	80
IL-3	Enhanced	early	4.2725	0.431692708	58	16	80
IL-3	Enhanced	mid	4.91375	0.570106727	58	16	80
IL-3	Enhanced	late	5.17125	0.582906075	58	16	80
IL-3	Standard	pre	8.080625	2.599528418	58	16	80
IL-3	Standard	early	6.33625	1.967552364	58	16	80
IL-3	Standard	mid	6.5875	2.159514377	58	16	80
IL-3	Standard	late	6.81625	2.020436167	58	16	80
IL-4	Enhanced	pre	52.68875	39.13834598	58	16	80
IL-4	Enhanced	early	51.55625	36.52171379	58	16	80
IL-4	Enhanced	mid	51.2575	39.20279417	58	16	80
IL-4	Enhanced	late	54.00625	41.44789379	58	16	80
IL-4	Standard	pre	112.886875	71.77264394	58	16	80
IL-4	Standard	early	112.585	71.95269082	58	16	80
IL-4	Standard	mid	104.47375	68.08307558	58	16	80
IL-4	Standard	late	108.83	70.11984551	58	16	80
IL-5	Enhanced	pre	6.751875	3.207485013	58	16	80
IL-5	Enhanced	early	6.50125	3.197329177	58	16	80
IL-5	Enhanced	mid	6.9975	3.090053979	58	16	80
IL-5	Enhanced	late	6.9425	3.299838893	58	16	80
IL-5	Standard	pre	22.571875	16.82100194	58	16	80
IL-5	Standard	early	20.685	14.98222968	58	16	80
IL-5	Standard	mid	18.71375	13.06555597	58	16	80
IL-5	Standard	late	20.18125	14.71247934	58	16	80
IL-6	Enhanced	pre	12.29	7.689694453	58	16	80
IL-6	Enhanced	early	12.4475	7.581049101	58	16	80

IL-6	Enhanced	mid	12.3125	7.338379792	58	16	80
IL-6	Enhanced	late	12.60375	7.452911121	58	16	80
IL-6	Standard	pre	16.871875	7.838633936	58	16	80
IL-6	Standard	early	16.985	7.899580883	58	16	80
IL-6	Standard	mid	15.90375	7.104071002	58	16	80
IL-6	Standard	late	16.51875	7.632508074	58	16	80
IL-7	Enhanced	pre	4.796875	0.680547193	58	16	80
IL-7	Enhanced	early	3.88375	0.487403636	58	16	80
IL-7	Enhanced	mid	4.69	0.80089013	58	16	80
IL-7	Enhanced	late	4.2025	0.706633193	58	16	80
IL-7	Standard	pre	10.38625	5.687092757	58	16	80
IL-7	Standard	early	9.1	5.188383961	58	16	80
IL-7	Standard	mid	8.78375	4.816604398	58	16	80
IL-7	Standard	late	9.62	5.438934581	58	16	80
IL-8	Enhanced	pre	10.968125	5.801279556	58	16	80
IL-8	Enhanced	early	11.17875	5.8953627	58	16	80
IL-8	Enhanced	mid	11.01625	5.886766923	58	16	80
IL-8	Enhanced	late	11.28625	5.800516663	58	16	80
IL-8	Standard	pre	17.765625	8.147154049	58	16	80
IL-8	Standard	early	17.82	7.626105658	58	16	80
IL-8	Standard	mid	17.10375	7.481980006	58	16	80
IL-8	Standard	late	16.51625	7.528422382	58	16	80
IP-10	Enhanced	pre	363.84625	51.76367853	58	16	80
IP-10	Enhanced	early	455.22	48.86301774	58	16	80
IP-10	Enhanced	mid	340.50625	36.87698384	58	16	80
IP-10	Enhanced	late	325.845	38.02944082	58	16	80
IP-10	Standard	pre	321.04875	39.59510599	58	16	80
IP-10	Standard	early	459.665	57.37146661	58	16	80
IP-10	Standard	mid	351.76375	38.11279427	58	16	80
IP-10	Standard	late	397.3125	45.98946788	58	16	80
MCP-1	Enhanced	pre	246.1125	23.66315649	58	16	80
MCP-1	Enhanced	early	265.79875	22.54190538	58	16	80
MCP-1	Enhanced	mid	253.245	28.25112315	58	16	80
MCP-1	Enhanced	late	258.755	30.19858208	58	16	80
MCP-1	Standard	pre	253.19875	22.77288239	58	16	80
MCP-1	Standard	early	267.225	24.57371137	58	16	80
MCP-1	Standard	mid	245.3725	20.73130459	58	16	80
MCP-1	Standard	late	241.25375	20.56282545	58	16	80
MIP-1a	Enhanced	pre	10.264375	3.431358882	58	16	80
MIP-1a	Enhanced	early	9.81	3.554388573	58	16	80
MIP-1a	Enhanced	mid	10.38625	3.447456008	58	16	80
MIP-1a	Enhanced	late	10.66125	3.469036447	58	16	80
MIP-1a	Standard	pre	65.814375	52.68129337	58	16	80
MIP-1a	Standard	early	28.73625	17.84933725	58	16	80
MIP-1a	Standard	mid	52.35	40.16167331	58	16	80
MIP-1a	Standard	late	36.22125	25.25520599	58	16	80
MIP-1b	Enhanced	pre	21.551875	3.150323264	58	16	80
MIP-1b	Enhanced	early	19.5875	2.532754028	58	16	80
MIP-1b	Enhanced	mid	20.9925	2.716148324	58	16	80
MIP-1b	Enhanced	late	20.67625	2.944234269	58	16	80
MIP-1b	Standard	pre	28.105	5.847381292	58	16	80
MIP-1b	Standard	early	25.68375	4.679758253	58	16	80

MIP-1b	Standard	mid	23.70125	4.397251783	58	16	80
MIP-1b	Standard	late	26.1625	5.135013921	58	16	80
RANTES	Enhanced	pre	2543	269.316612	58	16	80
RANTES	Enhanced	early	2495.625	227.1021203	58	16	80
RANTES	Enhanced	mid	2803.375	198.9267247	58	16	80
RANTES	Enhanced	late	2657.375	170.9383969	58	16	80
RANTES	Standard	pre	1811.88875	119.1854321	58	16	80
RANTES	Standard	early	1836.75	183.2406844	58	16	80
RANTES	Standard	mid	1820.375	186.1194121	58	16	80
RANTES	Standard	late	2021.25	292.3119454	58	16	80
TNFa	Enhanced	pre	14.491875	1.814389734	58	16	80
TNFa	Enhanced	early	14.08875	1.456962205	58	16	80
TNFa	Enhanced	mid	15.16	1.673869021	58	16	80
TNFa	Enhanced	late	14.62125	1.572218216	58	16	80
TNFa	Standard	pre	34.621875	18.8511996	58	16	80
TNFa	Standard	early	32.2825	16.60182028	58	16	80
TNFa	Standard	mid	31.0675	16.25213839	58	16	80
TNFa	Standard	late	31.5725	16.14643941	58	16	80
TNFb	Enhanced	pre	109.68	82.6675872	58	16	80
TNFb	Enhanced	early	113.6025	87.00476116	58	16	80
TNFb	Enhanced	mid	109.00125	82.1249771	58	16	80
TNFb	Enhanced	late	110.76625	82.81197936	58	16	80
TNFb	Standard	pre	226.314375	121.9193731	58	16	80
TNFb	Standard	early	228.33125	125.2029097	58	16	80
TNFb	Standard	mid	206.46375	110.1488465	58	16	80
TNFb	Standard	late	209.87875	111.1235798	58	16	80
VEGF	Enhanced	pre	48.710625	14.10371373	58	16	80
VEGF	Enhanced	early	39.99875	12.74972125	58	16	80
VEGF	Enhanced	mid	48.0725	15.09301916	58	16	80
VEGF	Enhanced	late	46.43875	14.36472714	58	16	80
VEGF	Standard	pre	84.205	18.38211448	58	16	80
VEGF	Standard	early	69.26125	18.63414175	58	16	80
VEGF	Standard	mid	73.43875	17.90148147	58	16	80
VEGF	Standard	late	72.11625	16.34481186	58	16	80

Table S13: Average Individual T cell function. All values are reported in pg/ml.

Mitogen	Population	Measurement	Menu	Time	Estimate	StdErr	DF	Nsubjects	Nobs
(SEA+SEB, 24hr)	CD4+CD69+Early Activated	3-28 24H-CD4+CD69+ Early Activated	Enhanced	pre	13.4375	3.586233988	57	16	79
(SEA+SEB, 24hr)	CD4+CD69+Early Activated	3-28 24H-CD4+CD69+ Early Activated	Enhanced	early	13.90254193	4.570327484	57	16	79
(SEA+SEB, 24hr)	CD4+CD69+Early Activated	3-28 24H-CD4+CD69+ Early Activated	Enhanced	mid	21.3875	7.006413078	57	16	79
(SEA+SEB, 24hr)	CD4+CD69+Early Activated	3-28 24H-CD4+CD69+ Early Activated	Enhanced	late	16.0875	4.498452425	57	16	79
(SEA+SEB, 24hr)	CD4+CD69+Early Activated	3-28 24H-CD4+CD69+ Early Activated	Standard	pre	17.34375	3.478442961	57	16	79
(SEA+SEB, 24hr)	CD4+CD69+Early Activated	3-28 24H-CD4+CD69+ Early Activated	Standard	early	14.8875	2.908953715	57	16	79
(SEA+SEB, 24hr)	CD4+CD69+Early Activated	3-28 24H-CD4+CD69+ Early Activated	Standard	mid	21.4	5.584521018	57	16	79
(SEA+SEB, 24hr)	CD4+CD69+Early Activated	3-28 24H-CD4+CD69+ Early Activated	Standard	late	14.2	3.327301835	57	16	79
(SEA+SEB, 24hr)	CD4+CD69+CD25+ Fully Activated	3-28 24H-CD4+CD69+CD25+ Fully Activated	Enhanced	pre	25.925	8.688713944	57	16	79
(SEA+SEB, 24hr)	CD4+CD69+CD25+ Fully Activated	3-28 24H-CD4+CD69+CD25+ Fully Activated	Enhanced	early	21.49883241	8.925105862	57	16	79
(SEA+SEB, 24hr)	CD4+CD69+CD25+ Fully Activated	3-28 24H-CD4+CD69+CD25+ Fully Activated	Enhanced	mid	18.9125	7.307924584	57	16	79
(SEA+SEB, 24hr)	CD4+CD69+CD25+ Fully Activated	3-28 24H-CD4+CD69+CD25+ Fully Activated	Enhanced	late	22.5875	8.9693826	57	16	79
(SEA+SEB, 24hr)	CD4+CD69+CD25+ Fully Activated	3-28 24H-CD4+CD69+CD25+ Fully Activated	Standard	pre	32.3125	8.7912943	57	16	79
(SEA+SEB, 24hr)	CD4+CD69+CD25+ Fully Activated	3-28 24H-CD4+CD69+CD25+ Fully Activated	Standard	early	38.2125	12.54678043	57	16	79
(SEA+SEB, 24hr)	CD4+CD69+CD25+ Fully Activated	3-28 24H-CD4+CD69+CD25+ Fully Activated	Standard	mid	27.4375	9.396407849	57	16	79
(SEA+SEB, 24hr)	CD4+CD69+CD25+ Fully Activated	3-28 24H-CD4+CD69+CD25+ Fully Activated	Standard	late	48.15	9.653108308	57	16	79
(SEA+SEB, 24hr)	CD8+CD69+Early Activated	3-28 24H-CD8+CD69+ Early Activated	Enhanced	pre	24.94277292	3.506682678	54	16	76
(SEA+SEB, 24hr)	CD8+CD69+Early Activated	3-28 24H-CD8+CD69+ Early Activated	Enhanced	early	24.53130874	6.676376423	54	16	76
(SEA+SEB, 24hr)	CD8+CD69+Early Activated	3-28 24H-CD8+CD69+ Early Activated	Enhanced	mid	29.125	7.016693265	54	16	76
(SEA+SEB, 24hr)	CD8+CD69+Early Activated	3-28 24H-CD8+CD69+ Early Activated	Enhanced	late	25.6125	6.201371761	54	16	76
(SEA+SEB, 24hr)	CD8+CD69+Early Activated	3-28 24H-CD8+CD69+ Early Activated	Standard	pre	29.3	3.594853092	54	16	76
(SEA+SEB, 24hr)	CD8+CD69+Early Activated	3-28 24H-CD8+CD69+ Early Activated	Standard	early	24.3875	3.967935448	54	16	76
(SEA+SEB, 24hr)	CD8+CD69+Early Activated	3-28 24H-CD8+CD69+ Early Activated	Standard	mid	27.8	3.814487187	54	16	76
(SEA+SEB, 24hr)	CD8+CD69+Early Activated	3-28 24H-CD8+CD69+ Early Activated	Standard	late	23.225	3.2001831	54	16	76
(SEA+SEB, 24hr)	CD8+CD69+CD25+ Fully Activated	3-28 24H-CD8+CD69+CD25+ Fully Activated	Enhanced	pre	23.4052721	6.858963083	54	16	76
(SEA+SEB, 24hr)	CD8+CD69+CD25+ Fully Activated	3-28 24H-CD8+CD69+CD25+ Fully Activated	Enhanced	early	11.32684333	5.53159343	54	16	76
(SEA+SEB, 24hr)	CD8+CD69+CD25+ Fully Activated	3-28 24H-CD8+CD69+CD25+ Fully Activated	Enhanced	mid	11.65	5.100030637	54	16	76
(SEA+SEB, 24hr)	CD8+CD69+CD25+ Fully Activated	3-28 24H-CD8+CD69+CD25+ Fully Activated	Enhanced	late	15.1	5.201952758	54	16	76
(SEA+SEB, 24hr)	CD8+CD69+CD25+ Fully Activated	3-28 24H-CD8+CD69+CD25+ Fully Activated	Standard	pre	23.69375	4.939504637	54	16	76
(SEA+SEB, 24hr)	CD8+CD69+CD25+ Fully Activated	3-28 24H-CD8+CD69+CD25+ Fully Activated	Standard	early	31.6	7.359156711	54	16	76
(SEA+SEB, 24hr)	CD8+CD69+CD25+ Fully Activated	3-28 24H-CD8+CD69+CD25+ Fully Activated	Standard	mid	30.1875	7.205064137	54	16	76
(SEA+SEB, 24hr)	CD8+CD69+CD25+ Fully Activated	3-28 24H-CD8+CD69+CD25+ Fully Activated	Standard	late	35.85	8.271241594	54	16	76
(anti-CD3/CD28, 24hr)	CD4+CD69+ Early Activated	SEA+SEB 24H-CD4+CD69+Early Activated	Enhanced	pre	29.45	3.642929796	58	16	80
(anti-CD3/CD28, 24hr)	CD4+CD69+ Early Activated	SEA+SEB 24H-CD4+CD69+Early Activated	Enhanced	early	25.4125	3.473220655	58	16	80
(anti-CD3/CD28, 24hr)	CD4+CD69+ Early Activated	SEA+SEB 24H-CD4+CD69+Early Activated	Enhanced	mid	10.0625	1.634007487	58	16	80
(anti-CD3/CD28, 24hr)	CD4+CD69+ Early Activated	SEA+SEB 24H-CD4+CD69+Early Activated	Enhanced	late	9.1625	1.196079207	58	16	80
(anti-CD3/CD28, 24hr)	CD4+CD69+ Early Activated	SEA+SEB 24H-CD4+CD69+Early Activated	Standard	pre	11.0875	0.948137566	58	16	80
(anti-CD3/CD28, 24hr)	CD4+CD69+ Early Activated	SEA+SEB 24H-CD4+CD69+Early Activated	Standard	early	13.35	2.014090986	58	16	80
(anti-CD3/CD28, 24hr)	CD4+CD69+ Early Activated	SEA+SEB 24H-CD4+CD69+Early Activated	Standard	mid	11.0375	2.123818605	58	16	80
(anti-CD3/CD28, 24hr)	CD4+CD69+ Early Activated	SEA+SEB 24H-CD4+CD69+Early Activated	Standard	late	15.1375	1.991853526	58	16	80
(anti-CD3/CD28, 24hr)	CD4+CD69+CD25+ Fully Activated	SEA+SEB 24H-CD4+CD69+CD25+ Fully Activated	Enhanced	pre	31.18125	5.817275844	58	16	80
(anti-CD3/CD28, 24hr)	CD4+CD69+CD25+ Fully Activated	SEA+SEB 24H-CD4+CD69+CD25+ Fully Activated	Enhanced	early	30.5375	6.800412154	58	16	80
(anti-CD3/CD28, 24hr)	CD4+CD69+CD25+ Fully Activated	SEA+SEB 24H-CD4+CD69+CD25+ Fully Activated	Enhanced	mid	3.9875	1.436623374	58	16	80
(anti-CD3/CD28, 24hr)	CD4+CD69+CD25+ Fully Activated	SEA+SEB 24H-CD4+CD69+CD25+ Fully Activated	Enhanced	late	6.6875	1.862746418	58	16	80
(anti-CD3/CD28, 24hr)	CD4+CD69+CD25+ Fully Activated	SEA+SEB 24H-CD4+CD69+CD25+ Fully Activated	Standard	pre	10.06875	1.989718445	58	16	80
(anti-CD3/CD28, 24hr)	CD4+CD69+CD25+ Fully Activated	SEA+SEB 24H-CD4+CD69+CD25+ Fully Activated	Standard	early	12.875	2.506974646	58	16	80
(anti-CD3/CD28, 24hr)	CD4+CD69+CD25+ Fully Activated	SEA+SEB 24H-CD4+CD69+CD25+ Fully Activated	Standard	mid	12.0625	2.886116416	58	16	80
(anti-CD3/CD28, 24hr)	CD4+CD69+CD25+ Fully Activated	SEA+SEB 24H-CD4+CD69+CD25+ Fully Activated	Standard	late	14.8375	1.728252143	58	16	80
(anti-CD3/CD28, 24hr)	CD8+CD69+ Early Activated	SEA+SEB 24H-CD8+CD69+Early Activated	Enhanced	pre	41.96401457	6.630144669	54	16	76
(anti-CD3/CD28, 24hr)	CD8+CD69+ Early Activated	SEA+SEB 24H-CD8+CD69+Early Activated	Enhanced	early	37.0625	5.102509723	54	16	76
(anti-CD3/CD28, 24hr)	CD8+CD69+ Early Activated	SEA+SEB 24H-CD8+CD69+Early Activated	Enhanced	mid	24.525	3.340834009	54	16	76
(anti-CD3/CD28, 24hr)	CD8+CD69+ Early Activated	SEA+SEB 24H-CD8+CD69+Early Activated	Enhanced	late	26.1	2.612051397	54	16	76
(anti-CD3/CD28, 24hr)	CD8+CD69+ Early Activated	SEA+SEB 24H-CD8+CD69+Early Activated	Standard	pre	27.85	3.881717967	54	16	76
(anti-CD3/CD28, 24hr)	CD8+CD69+ Early Activated	SEA+SEB 24H-CD8+CD69+Early Activated	Standard	early	26.7875	4.024939343	54	16	76
(anti-CD3/CD28, 24hr)	CD8+CD69+ Early Activated	SEA+SEB 24H-CD8+CD69+Early Activated	Standard	mid	27.55	5.485349807	54	16	76
(anti-CD3/CD28, 24hr)	CD8+CD69+ Early Activated	SEA+SEB 24H-CD8+CD69+Early Activated	Standard	late	24.0125	2.249335839	54	16	76
(anti-CD3/CD28, 24hr)	CD8+CD69+CD25+ Fully Activated	SEA+SEB 24H-CD8+CD69+CD25+ Fully Activated	Enhanced	pre	16.675	2.659489038	54	16	76
(anti-CD3/CD28, 24hr)	CD8+CD69+CD25+ Fully Activated	SEA+SEB 24H-CD8+CD69+CD25+ Fully Activated	Enhanced	early	22.2875	2.348698942	54	16	76
(anti-CD3/CD28, 24hr)	CD8+CD69+CD25+ Fully Activated	SEA+SEB 24H-CD8+CD69+CD25+ Fully Activated	Enhanced	mid	10.3125	4.33987462	54	16	76
(anti-CD3/CD28, 24hr)	CD8+CD69+CD25+ Fully Activated	SEA+SEB 24H-CD8+CD69+CD25+ Fully Activated	Enhanced	late	7.9125	0.882899609	54	16	76
(anti-CD3/CD28, 24hr)	CD8+CD69+CD25+ Fully Activated	SEA+SEB 24H-CD8+CD69+CD25+ Fully Activated	Standard	pre	7.025	0.649278446	54	16	76
(anti-CD3/CD28, 24hr)	CD8+CD69+CD25+ Fully Activated	SEA+SEB 24H-CD8+CD69+CD25+ Fully Activated	Standard	early	9.65	0.767707627	54	16	76
(anti-CD3/CD28, 24hr)	CD8+CD69+CD25+ Fully Activated	SEA+SEB 24H-CD8+CD69+CD25+ Fully Activated	Standard	mid	8.95	1.820027472	54	16	76
(anti-CD3/CD28, 24hr)	CD8+CD69+CD25+ Fully Activated	SEA+SEB 24H-CD8+CD69+CD25+ Fully Activated	Standard	late	6.5875	1.500462168	54	16	76

Table S14: Average Individual Mitogen Stimulated Cytokines. All values are reported in pg/ml.

Mitogen	Measurement	Menu	Time	Estimate	StdErr	DF	Nsubjects	Nobs
anti-CD3+CD28 48 Hr	GM-CSF	Enhanced	pre	448.56875	146.3099825	34	10	50
anti-CD3+CD28 48 Hr	GM-CSF	Enhanced	early	514.8125	187.5565658	34	10	50
anti-CD3+CD28 48 Hr	GM-CSF	Enhanced	mid	132.575	64.9226265	34	10	50
anti-CD3+CD28 48 Hr	GM-CSF	Enhanced	late	634.27	235.9459502	34	10	50
anti-CD3+CD28 48 Hr	GM-CSF	Standard	pre	643.4566667	218.483621	34	10	50
anti-CD3+CD28 48 Hr	GM-CSF	Standard	early	725.805	371.8847102	34	10	50
anti-CD3+CD28 48 Hr	GM-CSF	Standard	mid	414.79	94.17397022	34	10	50
anti-CD3+CD28 48 Hr	GM-CSF	Standard	late	816.91	346.2292864	34	10	50
anti-CD3+CD28 48 Hr	IFNγ	Enhanced	pre	9506.75625	3062.783496	34	10	50
anti-CD3+CD28 48 Hr	IFNγ	Enhanced	early	10873.3525	4449.121684	34	10	50
anti-CD3+CD28 48 Hr	IFNγ	Enhanced	mid	3340.155	2108.10008	34	10	50
anti-CD3+CD28 48 Hr	IFNγ	Enhanced	late	11957.545	4093.868765	34	10	50
anti-CD3+CD28 48 Hr	IFNγ	Standard	pre	16370.41667	5413.536354	34	10	50
anti-CD3+CD28 48 Hr	IFNγ	Standard	early	14358	5790.677604	34	10	50
anti-CD3+CD28 48 Hr	IFNγ	Standard	mid	13364	3418.426839	34	10	50
anti-CD3+CD28 48 Hr	IFNγ	Standard	late	18336.66667	7072.699067	34	10	50
anti-CD3+CD28 48 Hr	IL-10	Enhanced	pre	6059.375	2086.03487	34	10	50
anti-CD3+CD28 48 Hr	IL-10	Enhanced	early	9410	3337.082015	34	10	50
anti-CD3+CD28 48 Hr	IL-10	Enhanced	mid	3119.23	745.5526203	34	10	50
anti-CD3+CD28 48 Hr	IL-10	Enhanced	late	10989.25	3408.27976	34	10	50
anti-CD3+CD28 48 Hr	IL-10	Standard	pre	6726.666667	1972.467704	34	10	50
anti-CD3+CD28 48 Hr	IL-10	Standard	early	6785.11	2475.165344	34	10	50
anti-CD3+CD28 48 Hr	IL-10	Standard	mid	7971.058333	2800.083811	34	10	50
anti-CD3+CD28 48 Hr	IL-10	Standard	late	7499.245	2493.979237	34	10	50
anti-CD3+CD28 48 Hr	IL-12p70	Enhanced	pre	18.8025	6.644198245	34	10	50
anti-CD3+CD28 48 Hr	IL-12p70	Enhanced	early	9.655	3.294151067	34	10	50
anti-CD3+CD28 48 Hr	IL-12p70	Enhanced	mid	13.745	4.916228865	34	10	50
anti-CD3+CD28 48 Hr	IL-12p70	Enhanced	late	11.9525	3.352557559	34	10	50
anti-CD3+CD28 48 Hr	IL-12p70	Standard	pre	12.49416667	3.08237855	34	10	50
anti-CD3+CD28 48 Hr	IL-12p70	Standard	early	9.005	2.575427419	34	10	50
anti-CD3+CD28 48 Hr	IL-12p70	Standard	mid	12.61666667	3.488561202	34	10	50
anti-CD3+CD28 48 Hr	IL-12p70	Standard	late	12.825	4.180906833	34	10	50
anti-CD3+CD28 48 Hr	IL-13	Enhanced	pre	374.08125	112.0245621	34	10	50
anti-CD3+CD28 48 Hr	IL-13	Enhanced	early	455.3975	173.9205564	34	10	50
anti-CD3+CD28 48 Hr	IL-13	Enhanced	mid	153.36	59.39528485	34	10	50
anti-CD3+CD28 48 Hr	IL-13	Enhanced	late	554.97	207.2690158	34	10	50
anti-CD3+CD28 48 Hr	IL-13	Standard	pre	481.85	58.51411668	34	10	50
anti-CD3+CD28 48 Hr	IL-13	Standard	early	305.2916667	53.44203066	34	10	50
anti-CD3+CD28 48 Hr	IL-13	Standard	mid	390.825	44.36057226	34	10	50
anti-CD3+CD28 48 Hr	IL-13	Standard	late	421.2383333	60.45359609	34	10	50
anti-CD3+CD28 48 Hr	IL-1B	Enhanced	pre	16.09	3.261913261	34	10	50
anti-CD3+CD28 48 Hr	IL-1B	Enhanced	early	14.6725	3.341839641	34	10	50
anti-CD3+CD28 48 Hr	IL-1B	Enhanced	mid	21.23	12.06999327	34	10	50
anti-CD3+CD28 48 Hr	IL-1B	Enhanced	late	25.3	6.648177382	34	10	50
anti-CD3+CD28 48 Hr	IL-1B	Standard	pre	115.2133333	40.9648895	34	10	50
anti-CD3+CD28 48 Hr	IL-1B	Standard	early	25.27333333	8.021286841	34	10	50
anti-CD3+CD28 48 Hr	IL-1B	Standard	mid	51.43666667	15.81668856	34	10	50
anti-CD3+CD28 48 Hr	IL-1B	Standard	late	22.19833333	5.564944053	34	10	50
anti-CD3+CD28 48 Hr	IL-2	Enhanced	pre	996.52625	647.4465346	34	10	50
anti-CD3+CD28 48 Hr	IL-2	Enhanced	early	795.82	456.1221503	34	10	50
anti-CD3+CD28 48 Hr	IL-2	Enhanced	mid	361.94	138.4995237	34	10	50
anti-CD3+CD28 48 Hr	IL-2	Enhanced	late	842.695	511.3623963	34	10	50
anti-CD3+CD28 48 Hr	IL-2	Standard	pre	917.1	258.591634	34	10	50

anti-CD3+CD28 48 Hr	IL-2	Standard	early	843.33	391.5290092	34	10	50
anti-CD3+CD28 48 Hr	IL-2	Standard	mid	825.8933333	306.4762973	34	10	50
anti-CD3+CD28 48 Hr	IL-2	Standard	late	914.6383333	386.1811644	34	10	50
anti-CD3+CD28 48 Hr	IL-4	Enhanced	pre	980.485	332.8342353	34	10	50
anti-CD3+CD28 48 Hr	IL-4	Enhanced	early	1127.625	455.6596955	34	10	50
anti-CD3+CD28 48 Hr	IL-4	Enhanced	mid	488.985	52.52160657	34	10	50
anti-CD3+CD28 48 Hr	IL-4	Enhanced	late	1021.9325	332.3833766	34	10	50
anti-CD3+CD28 48 Hr	IL-4	Standard	pre	695.1433333	113.5942925	34	10	50
anti-CD3+CD28 48 Hr	IL-4	Standard	early	391.825	92.8096074	34	10	50
anti-CD3+CD28 48 Hr	IL-4	Standard	mid	616.9266667	113.2001608	34	10	50
anti-CD3+CD28 48 Hr	IL-4	Standard	late	568.4383333	144.7480785	34	10	50
anti-CD3+CD28 48 Hr	IL-5	Enhanced	pre	219.1675	30.80850438	34	10	50
anti-CD3+CD28 48 Hr	IL-5	Enhanced	early	232.7625	59.43635381	34	10	50
anti-CD3+CD28 48 Hr	IL-5	Enhanced	mid	93.43	44.42252708	34	10	50
anti-CD3+CD28 48 Hr	IL-5	Enhanced	late	229.6375	50.0507378	34	10	50
anti-CD3+CD28 48 Hr	IL-5	Standard	pre	159.4691667	23.7434294	34	10	50
anti-CD3+CD28 48 Hr	IL-5	Standard	early	104.7666667	11.49676976	34	10	50
anti-CD3+CD28 48 Hr	IL-5	Standard	mid	130.1083333	17.02148763	34	10	50
anti-CD3+CD28 48 Hr	IL-5	Standard	late	156.595	18.77714372	34	10	50
anti-CD3+CD28 48 Hr	IL-6	Enhanced	pre	362.28375	103.7814937	34	10	50
anti-CD3+CD28 48 Hr	IL-6	Enhanced	early	490.09	191.2787453	34	10	50
anti-CD3+CD28 48 Hr	IL-6	Enhanced	mid	200.32	87.8612475	34	10	50
anti-CD3+CD28 48 Hr	IL-6	Enhanced	late	260.155	79.11999072	34	10	50
anti-CD3+CD28 48 Hr	IL-6	Standard	pre	1154.014167	529.6614954	34	10	50
anti-CD3+CD28 48 Hr	IL-6	Standard	early	171.28	57.33980622	34	10	50
anti-CD3+CD28 48 Hr	IL-6	Standard	mid	274.71	45.72101924	34	10	50
anti-CD3+CD28 48 Hr	IL-6	Standard	late	149.64	44.63884737	34	10	50
anti-CD3+CD28 48 Hr	IL-7	Enhanced	pre	18.015	5.435991745	34	10	50
anti-CD3+CD28 48 Hr	IL-7	Enhanced	early	15.8475	5.397906278	34	10	50
anti-CD3+CD28 48 Hr	IL-7	Enhanced	mid	13.8525	8.80987115	34	10	50
anti-CD3+CD28 48 Hr	IL-7	Enhanced	late	17.3675	6.586233156	34	10	50
anti-CD3+CD28 48 Hr	IL-7	Standard	pre	25.90083333	6.685758435	34	10	50
anti-CD3+CD28 48 Hr	IL-7	Standard	early	22.18166667	6.432755767	34	10	50
anti-CD3+CD28 48 Hr	IL-7	Standard	mid	22.66833333	6.897658386	34	10	50
anti-CD3+CD28 48 Hr	IL-7	Standard	late	22.365	6.610956709	34	10	50
anti-CD3+CD28 48 Hr	IL-8	Enhanced	pre	23362	5145.890329	34	10	50
anti-CD3+CD28 48 Hr	IL-8	Enhanced	early	25422.75	9643.615235	34	10	50
anti-CD3+CD28 48 Hr	IL-8	Enhanced	mid	5404	2404.833804	34	10	50
anti-CD3+CD28 48 Hr	IL-8	Enhanced	late	14541.75	2707.127153	34	10	50
anti-CD3+CD28 48 Hr	IL-8	Standard	pre	18638.75	2304.294655	34	10	50
anti-CD3+CD28 48 Hr	IL-8	Standard	early	9220.5	1967.350937	34	10	50
anti-CD3+CD28 48 Hr	IL-8	Standard	mid	14828.5	2257.602006	34	10	50
anti-CD3+CD28 48 Hr	IL-8	Standard	late	12717.66667	2680.132435	34	10	50
anti-CD3+CD28 48 Hr	TNFa	Enhanced	pre	854.82	259.5463895	34	10	50
anti-CD3+CD28 48 Hr	TNFa	Enhanced	early	959.8675	298.4519751	34	10	50
anti-CD3+CD28 48 Hr	TNFa	Enhanced	mid	441.4	106.8636522	34	10	50
anti-CD3+CD28 48 Hr	TNFa	Enhanced	late	1167.0375	386.8104392	34	10	50
anti-CD3+CD28 48 Hr	TNFa	Standard	pre	1081.448333	261.0710736	34	10	50
anti-CD3+CD28 48 Hr	TNFa	Standard	early	1180.67	423.028023	34	10	50
anti-CD3+CD28 48 Hr	TNFa	Standard	mid	970.1966667	228.1636327	34	10	50
anti-CD3+CD28 48 Hr	TNFa	Standard	late	1385.151667	443.5316818	34	10	50
PMA+ionomycin 48 Hr	GM-CSF	Enhanced	pre	3164.25	351.791753	58	16	80
PMA+ionomycin 48 Hr	GM-CSF	Enhanced	early	3256.875	286.2814086	58	16	80
PMA+ionomycin 48 Hr	GM-CSF	Enhanced	mid	2649.425	487.9665519	58	16	80
PMA+ionomycin 48 Hr	GM-CSF	Enhanced	late	3999.875	548.3664502	58	16	80
PMA+ionomycin 48 Hr	GM-CSF	Standard	pre	4668.283125	704.2288699	58	16	80

PMA+ionomycin 48 Hr	GM-CSF	Standard	early	3653.625	473.1508724	58	16	80
PMA+ionomycin 48 Hr	GM-CSF	Standard	mid	6051.125	1032.661858	58	16	80
PMA+ionomycin 48 Hr	GM-CSF	Standard	late	4062.5	676.7340458	58	16	80
PMA+ionomycin 48 Hr	IFNγ	Enhanced	pre	28787.125	1722.833043	58	16	80
PMA+ionomycin 48 Hr	IFNγ	Enhanced	early	31491.125	2289.646564	58	16	80
PMA+ionomycin 48 Hr	IFNγ	Enhanced	mid	28029	3212.23157	58	16	80
PMA+ionomycin 48 Hr	IFNγ	Enhanced	late	30024.25	2589.092564	58	16	80
PMA+ionomycin 48 Hr	IFNγ	Standard	pre	30824.625	3306.154634	58	16	80
PMA+ionomycin 48 Hr	IFNγ	Standard	early	29259.25	2694.090476	58	16	80
PMA+ionomycin 48 Hr	IFNγ	Standard	mid	35177.125	3541.303796	58	16	80
PMA+ionomycin 48 Hr	IFNγ	Standard	late	27653.375	1846.101754	58	16	80
PMA+ionomycin 48 Hr	IL-10	Enhanced	pre	1709.446875	380.3347909	58	16	80
PMA+ionomycin 48 Hr	IL-10	Enhanced	early	2756.37875	749.3522489	58	16	80
PMA+ionomycin 48 Hr	IL-10	Enhanced	mid	2554.10875	670.56667	58	16	80
PMA+ionomycin 48 Hr	IL-10	Enhanced	late	3354.21375	987.1359095	58	16	80
PMA+ionomycin 48 Hr	IL-10	Standard	pre	2213.3325	529.5723347	58	16	80
PMA+ionomycin 48 Hr	IL-10	Standard	early	3322.0975	918.1278592	58	16	80
PMA+ionomycin 48 Hr	IL-10	Standard	mid	5043.845	1551.158622	58	16	80
PMA+ionomycin 48 Hr	IL-10	Standard	late	3110.51125	847.316093	58	16	80
PMA+ionomycin 48 Hr	IL-12p70	Enhanced	pre	14.82125	2.171958856	58	16	80
PMA+ionomycin 48 Hr	IL-12p70	Enhanced	early	16.3275	2.388241213	58	16	80
PMA+ionomycin 48 Hr	IL-12p70	Enhanced	mid	16.9	2.413280314	58	16	80
PMA+ionomycin 48 Hr	IL-12p70	Enhanced	late	26.06	7.185862857	58	16	80
PMA+ionomycin 48 Hr	IL-12p70	Standard	pre	22.819375	3.318471674	58	16	80
PMA+ionomycin 48 Hr	IL-12p70	Standard	early	22.47	3.610235882	58	16	80
PMA+ionomycin 48 Hr	IL-12p70	Standard	mid	34.875	5.345790985	58	16	80
PMA+ionomycin 48 Hr	IL-12p70	Standard	late	21.005	3.449263961	58	16	80
PMA+ionomycin 48 Hr	IL-13	Enhanced	pre	1394.64875	452.7480135	58	16	80
PMA+ionomycin 48 Hr	IL-13	Enhanced	early	1797.2225	458.4820125	58	16	80
PMA+ionomycin 48 Hr	IL-13	Enhanced	mid	2139.215	879.795756	58	16	80
PMA+ionomycin 48 Hr	IL-13	Enhanced	late	1939.58875	315.2710796	58	16	80
PMA+ionomycin 48 Hr	IL-13	Standard	pre	1460.524375	182.4810316	58	16	80
PMA+ionomycin 48 Hr	IL-13	Standard	early	1128.37375	246.6962634	58	16	80
PMA+ionomycin 48 Hr	IL-13	Standard	mid	1532.62	232.5131684	58	16	80
PMA+ionomycin 48 Hr	IL-13	Standard	late	1359.865	415.9982551	58	16	80
PMA+ionomycin 48 Hr	IL-1B	Enhanced	pre	38.328125	9.34739427	58	16	80
PMA+ionomycin 48 Hr	IL-1B	Enhanced	early	39.5875	15.03976664	58	16	80
PMA+ionomycin 48 Hr	IL-1B	Enhanced	mid	88.23875	58.70264968	58	16	80
PMA+ionomycin 48 Hr	IL-1B	Enhanced	late	41.60875	8.048960422	58	16	80
PMA+ionomycin 48 Hr	IL-1B	Standard	pre	78.126875	20.31230088	58	16	80
PMA+ionomycin 48 Hr	IL-1B	Standard	early	46.72125	10.63575815	58	16	80
PMA+ionomycin 48 Hr	IL-1B	Standard	mid	93.285	21.914569	58	16	80
PMA+ionomycin 48 Hr	IL-1B	Standard	late	61.29375	21.21950812	58	16	80
PMA+ionomycin 48 Hr	IL-2	Enhanced	pre	30705.75	3990.337959	58	16	80
PMA+ionomycin 48 Hr	IL-2	Enhanced	early	37297	5639.099197	58	16	80
PMA+ionomycin 48 Hr	IL-2	Enhanced	mid	30297.875	5368.865684	58	16	80
PMA+ionomycin 48 Hr	IL-2	Enhanced	late	42344.25	6220.073027	58	16	80
PMA+ionomycin 48 Hr	IL-2	Standard	pre	37289.89813	6624.46459	58	16	80
PMA+ionomycin 48 Hr	IL-2	Standard	early	41451.625	6601.936849	58	16	80
PMA+ionomycin 48 Hr	IL-2	Standard	mid	48995.25	6214.276765	58	16	80
PMA+ionomycin 48 Hr	IL-2	Standard	late	39578.875	6872.976067	58	16	80
PMA+ionomycin 48 Hr	IL-4	Enhanced	pre	3864.445625	1783.236805	58	16	80
PMA+ionomycin 48 Hr	IL-4	Enhanced	early	5204.89375	2244.322786	58	16	80
PMA+ionomycin 48 Hr	IL-4	Enhanced	mid	6627.32625	3516.116838	58	16	80
PMA+ionomycin 48 Hr	IL-4	Enhanced	late	4454.28625	1637.720089	58	16	80

PMA+ionomycin 48 Hr	IL-4	Standard	pre	2722.280625	749.0207032	58	16	80
PMA+ionomycin 48 Hr	IL-4	Standard	early	2593.94625	751.7441305	58	16	80
PMA+ionomycin 48 Hr	IL-4	Standard	mid	4033.105	1145.848054	58	16	80
PMA+ionomycin 48 Hr	IL-4	Standard	late	2761.7775	893.4122462	58	16	80
PMA+ionomycin 48 Hr	IL-5	Enhanced	pre	372.1725	103.1567484	58	16	80
PMA+ionomycin 48 Hr	IL-5	Enhanced	early	410.43125	98.85342226	58	16	80
PMA+ionomycin 48 Hr	IL-5	Enhanced	mid	490.73875	186.6770863	58	16	80
PMA+ionomycin 48 Hr	IL-5	Enhanced	late	409.275	78.51775747	58	16	80
PMA+ionomycin 48 Hr	IL-5	Standard	pre	357.364375	63.00867933	58	16	80
PMA+ionomycin 48 Hr	IL-5	Standard	early	270.61125	62.50894062	58	16	80
PMA+ionomycin 48 Hr	IL-5	Standard	mid	401.88375	86.63907753	58	16	80
PMA+ionomycin 48 Hr	IL-5	Standard	late	282.9975	78.71996933	58	16	80
PMA+ionomycin 48 Hr	IL-6	Enhanced	pre	1386.879375	197.9414391	58	16	80
PMA+ionomycin 48 Hr	IL-6	Enhanced	early	1893.255	343.5185606	58	16	80
PMA+ionomycin 48 Hr	IL-6	Enhanced	mid	1462.54	389.1175951	58	16	80
PMA+ionomycin 48 Hr	IL-6	Enhanced	late	2713.75	571.0287249	58	16	80
PMA+ionomycin 48 Hr	IL-6	Standard	pre	1854.11	279.259729	58	16	80
PMA+ionomycin 48 Hr	IL-6	Standard	early	1997.19125	497.5923574	58	16	80
PMA+ionomycin 48 Hr	IL-6	Standard	mid	3031.125	657.7913669	58	16	80
PMA+ionomycin 48 Hr	IL-6	Standard	late	2299.78	743.3754639	58	16	80
PMA+ionomycin 48 Hr	IL-7	Enhanced	pre	39.46875	9.201147082	58	16	80
PMA+ionomycin 48 Hr	IL-7	Enhanced	early	42.5525	10.70925764	58	16	80
PMA+ionomycin 48 Hr	IL-7	Enhanced	mid	35.385	11.2812901	58	16	80
PMA+ionomycin 48 Hr	IL-7	Enhanced	late	42.74125	10.71573003	58	16	80
PMA+ionomycin 48 Hr	IL-7	Standard	pre	42.594375	10.05834928	58	16	80
PMA+ionomycin 48 Hr	IL-7	Standard	early	35.94875	7.682473852	58	16	80
PMA+ionomycin 48 Hr	IL-7	Standard	mid	41.6475	10.87483775	58	16	80
PMA+ionomycin 48 Hr	IL-7	Standard	late	37.07375	9.266474339	58	16	80
PMA+ionomycin 48 Hr	IL-8	Enhanced	pre	12872.75	795.9167817	58	16	80
PMA+ionomycin 48 Hr	IL-8	Enhanced	early	13184.625	1356.587584	58	16	80
PMA+ionomycin 48 Hr	IL-8	Enhanced	mid	13119.375	2389.993965	58	16	80
PMA+ionomycin 48 Hr	IL-8	Enhanced	late	14877.25	1254.348284	58	16	80
PMA+ionomycin 48 Hr	IL-8	Standard	pre	16080.75	998.7298926	58	16	80
PMA+ionomycin 48 Hr	IL-8	Standard	early	12024.75	1073.391234	58	16	80
PMA+ionomycin 48 Hr	IL-8	Standard	mid	18788.5	1573.798202	58	16	80
PMA+ionomycin 48 Hr	IL-8	Standard	late	12888.5	1718.365957	58	16	80
PMA+ionomycin 48 Hr	TNFa	Enhanced	pre	2397.3675	213.5286955	58	16	80
PMA+ionomycin 48 Hr	TNFa	Enhanced	early	2623.875	188.4716853	58	16	80
PMA+ionomycin 48 Hr	TNFa	Enhanced	mid	2384.72375	367.7932285	58	16	80
PMA+ionomycin 48 Hr	TNFa	Enhanced	late	3700.75	505.5831642	58	16	80
PMA+ionomycin 48 Hr	TNFa	Standard	pre	4116.20125	580.3215632	58	16	80
PMA+ionomycin 48 Hr	TNFa	Standard	early	3798.5	795.6516001	58	16	80
PMA+ionomycin 48 Hr	TNFa	Standard	mid	7381.5	1695.470058	58	16	80
PMA+ionomycin 48 Hr	TNFa	Standard	late	4046.875	782.9198841	58	16	80
LPS 48 Hr	GM-CSF	Enhanced	pre	13.448125	1.616842974	58	16	80
LPS 48 Hr	GM-CSF	Enhanced	early	12.45375	1.867051433	58	16	80
LPS 48 Hr	GM-CSF	Enhanced	mid	9.23	0.874832127	58	16	80
LPS 48 Hr	GM-CSF	Enhanced	late	12.83	1.252828051	58	16	80
LPS 48 Hr	GM-CSF	Standard	pre	31.389375	6.172240049	58	16	80
LPS 48 Hr	GM-CSF	Standard	early	22.47625	4.651583715	58	16	80
LPS 48 Hr	GM-CSF	Standard	mid	17.8075	5.516803521	58	16	80
LPS 48 Hr	GM-CSF	Standard	late	19.50625	3.503425528	58	16	80
LPS 48 Hr	IFNγ	Enhanced	pre	2942.279375	1247.413246	58	16	80
LPS 48 Hr	IFNγ	Enhanced	early	2156.94375	736.8797586	58	16	80
LPS 48 Hr	IFNγ	Enhanced	mid	1319.49875	648.5399984	58	16	80

LPS 48 Hr	IFNγ	Enhanced	late	2344.84	706.3616174	58	16	80
LPS 48 Hr	IFNγ	Standard	pre	3427.93	971.6203787	58	16	80
LPS 48 Hr	IFNγ	Standard	early	2253.045	782.0713556	58	16	80
LPS 48 Hr	IFNγ	Standard	mid	2862.47875	1028.604958	58	16	80
LPS 48 Hr	IFNγ	Standard	late	1785.25	663.5576712	58	16	80
LPS 48 Hr	IL-10	Enhanced	pre	8865.625	2560.197033	58	16	80
LPS 48 Hr	IL-10	Enhanced	early	9683.5	2600.929744	58	16	80
LPS 48 Hr	IL-10	Enhanced	mid	6828.0875	2321.862326	58	16	80
LPS 48 Hr	IL-10	Enhanced	late	10346.125	2898.685383	58	16	80
LPS 48 Hr	IL-10	Standard	pre	11204.31188	3006.645234	58	16	80
LPS 48 Hr	IL-10	Standard	early	11140.875	3020.264069	58	16	80
LPS 48 Hr	IL-10	Standard	mid	10916.625	3087.454842	58	16	80
LPS 48 Hr	IL-10	Standard	late	11176.375	3019.257938	58	16	80
LPS 48 Hr	IL-12p70	Enhanced	pre	26.714375	9.564611876	58	16	80
LPS 48 Hr	IL-12p70	Enhanced	early	25.8225	7.496137912	58	16	80
LPS 48 Hr	IL-12p70	Enhanced	mid	22.455	6.124791833	58	16	80
LPS 48 Hr	IL-12p70	Enhanced	late	22.82375	5.784733393	58	16	80
LPS 48 Hr	IL-12p70	Standard	pre	23.093125	7.862283082	58	16	80
LPS 48 Hr	IL-12p70	Standard	early	22.30625	8.883490294	58	16	80
LPS 48 Hr	IL-12p70	Standard	mid	21.7525	8.486215247	58	16	80
LPS 48 Hr	IL-12p70	Standard	late	19.7425	7.283021297	58	16	80
LPS 48 Hr	IL-13	Enhanced	pre	9.95	1.3097832	58	16	80
LPS 48 Hr	IL-13	Enhanced	early	8.49375	1.325067142	58	16	80
LPS 48 Hr	IL-13	Enhanced	mid	5.38375	0.866665552	58	16	80
LPS 48 Hr	IL-13	Enhanced	late	10.09875	1.369018167	58	16	80
LPS 48 Hr	IL-13	Standard	pre	12.499375	1.546302656	58	16	80
LPS 48 Hr	IL-13	Standard	early	11.1	1.869738451	58	16	80
LPS 48 Hr	IL-13	Standard	mid	11.76125	2.824251249	58	16	80
LPS 48 Hr	IL-13	Standard	late	12.1425	2.789587083	58	16	80
LPS 48 Hr	IL-1B	Enhanced	pre	1872.216875	384.9604196	58	16	80
LPS 48 Hr	IL-1B	Enhanced	early	2311.6725	342.8016793	58	16	80
LPS 48 Hr	IL-1B	Enhanced	mid	1574.14375	266.9539881	58	16	80
LPS 48 Hr	IL-1B	Enhanced	late	2157.16125	292.5288265	58	16	80
LPS 48 Hr	IL-1B	Standard	pre	2171.2975	523.6430428	58	16	80
LPS 48 Hr	IL-1B	Standard	early	1824.36375	370.9920516	58	16	80
LPS 48 Hr	IL-1B	Standard	mid	2267.57625	434.4954149	58	16	80
LPS 48 Hr	IL-1B	Standard	late	1555.02375	351.3560445	58	16	80
LPS 48 Hr	IL-2	Enhanced	pre	2.988125	0.824331613	58	16	80
LPS 48 Hr	IL-2	Enhanced	early	2.43125	0.593033614	58	16	80
LPS 48 Hr	IL-2	Enhanced	mid	2.765	0.727478522	58	16	80
LPS 48 Hr	IL-2	Enhanced	late	4.90375	1.737553001	58	16	80
LPS 48 Hr	IL-2	Standard	pre	4.796875	1.692217697	58	16	80
LPS 48 Hr	IL-2	Standard	early	6.50875	2.468420192	58	16	80
LPS 48 Hr	IL-2	Standard	mid	6.975	3.487274634	58	16	80
LPS 48 Hr	IL-2	Standard	late	5.70375	2.546121399	58	16	80
LPS 48 Hr	IL-4	Enhanced	pre	29.39375	3.749438369	58	16	80
LPS 48 Hr	IL-4	Enhanced	early	23.95625	3.39284999	58	16	80
LPS 48 Hr	IL-4	Enhanced	mid	18.93125	3.039095999	58	16	80
LPS 48 Hr	IL-4	Enhanced	late	20.5425	3.128880965	58	16	80
LPS 48 Hr	IL-4	Standard	pre	24.635	2.696024562	58	16	80
LPS 48 Hr	IL-4	Standard	early	17.80875	2.003152545	58	16	80
LPS 48 Hr	IL-4	Standard	mid	19.1625	1.989145349	58	16	80
LPS 48 Hr	IL-4	Standard	late	16.9025	2.373605347	58	16	80
LPS 48 Hr	IL-5	Enhanced	pre	5.135	0.601741613	58	16	80
LPS 48 Hr	IL-5	Enhanced	early	5.7825	0.520662889	58	16	80

LPS 48 Hr	IL-5	Enhanced	mid	4.32625	0.900990666	58	16	80
LPS 48 Hr	IL-5	Enhanced	late	5.36125	0.74438825	58	16	80
LPS 48 Hr	IL-5	Standard	pre	5.35625	0.866742172	58	16	80
LPS 48 Hr	IL-5	Standard	early	4.41625	0.662523437	58	16	80
LPS 48 Hr	IL-5	Standard	mid	5.49125	0.503368148	58	16	80
LPS 48 Hr	IL-5	Standard	late	3.74875	0.452839781	58	16	80
LPS 48 Hr	IL-6	Enhanced	pre	7691.9375	1072.729741	58	16	80
LPS 48 Hr	IL-6	Enhanced	early	10656.875	1867.169569	58	16	80
LPS 48 Hr	IL-6	Enhanced	mid	8421.5	1435.162894	58	16	80
LPS 48 Hr	IL-6	Enhanced	late	11461.625	2018.526698	58	16	80
LPS 48 Hr	IL-6	Standard	pre	10130.3125	1918.93174	58	16	80
LPS 48 Hr	IL-6	Standard	early	9366.25	1897.510011	58	16	80
LPS 48 Hr	IL-6	Standard	mid	11463	2183.217959	58	16	80
LPS 48 Hr	IL-6	Standard	late	8875.375	2154.635257	58	16	80
LPS 48 Hr	IL-7	Enhanced	pre	36.245625	8.96253092	58	16	80
LPS 48 Hr	IL-7	Enhanced	early	40.345	9.592629983	58	16	80
LPS 48 Hr	IL-7	Enhanced	mid	33.105	8.289840544	58	16	80
LPS 48 Hr	IL-7	Enhanced	late	34.48125	7.86683653	58	16	80
LPS 48 Hr	IL-7	Standard	pre	38.32625	8.521722095	58	16	80
LPS 48 Hr	IL-7	Standard	early	35.37625	8.043994642	58	16	80
LPS 48 Hr	IL-7	Standard	mid	36.4	8.048445114	58	16	80
LPS 48 Hr	IL-7	Standard	late	33.05375	7.318004112	58	16	80
LPS 48 Hr	IL-8	Enhanced	pre	23978.125	1393.305021	58	16	80
LPS 48 Hr	IL-8	Enhanced	early	27172.25	2826.607395	58	16	80
LPS 48 Hr	IL-8	Enhanced	mid	13578.75	3517.096076	58	16	80
LPS 48 Hr	IL-8	Enhanced	late	21260.75	2834.160941	58	16	80
LPS 48 Hr	IL-8	Standard	pre	28427.625	1796.938785	58	16	80
LPS 48 Hr	IL-8	Standard	early	15666.75	1191.568343	58	16	80
LPS 48 Hr	IL-8	Standard	mid	29709.75	1765.930666	58	16	80
LPS 48 Hr	IL-8	Standard	late	23520.125	3432.175533	58	16	80
LPS 48 Hr	TNFa	Enhanced	pre	1345.480625	194.5948487	58	16	80
LPS 48 Hr	TNFa	Enhanced	early	1665.125	215.6527473	58	16	80
LPS 48 Hr	TNFa	Enhanced	mid	1502.0675	158.5673997	58	16	80
LPS 48 Hr	TNFa	Enhanced	late	1487.125	189.002599	58	16	80
LPS 48 Hr	TNFa	Standard	pre	1648.625	220.3612402	58	16	80
LPS 48 Hr	TNFa	Standard	early	1736.125	184.3487216	58	16	80
LPS 48 Hr	TNFa	Standard	mid	1802.625	172.3170169	58	16	80
LPS 48 Hr	TNFa	Standard	late	1588.18875	235.7323116	58	16	80

Table S15: Epstein Bar Virus (EBV0 and Herpes Simplex Virus 1 (HSV1) shedding measured from saliva. Dark colored boxes indicate timepoints at which >20 copies/µl, and these are considered relevant positives. Light colored boxes indicate minor shedding, at less than <20 copies/µl.

		EBV				HSV1			
Menu	Subject	Pre	Early	Mid	Late	Pre	Early	Mid	Late
Enhanced	1								
Enhanced	2								
Enhanced	3								
Enhanced	4								
Enhanced	5								
Enhanced	6								
Enhanced	7								
Enhanced	8								
Standard	9								
Standard	10								
Standard	11								
Standard	12								
Standard	13								
Standard	14								
Standard	15								
Standard	16								

Table S16: Shannon Alpha Diversity of the Gastrointestinal Microbiome

Measurement	Menu	Time	Estimate	StdErr	DF	Nsubjects	Nobs
Diversity	Enhanced	pre	2.58651257	0.163611725	26	8	40
Diversity	Enhanced	early	2.755593825	0.354849145	26	8	40
Diversity	Enhanced	mid	2.690782551	0.275806917	26	8	40
Diversity	Enhanced	late	2.603774203	0.356881909	26	8	40
Diversity	Standard	pre	2.810737597	0.044790377	26	8	40
Diversity	Standard	early	2.028379075	0.337577163	26	8	40
Diversity	Standard	mid	2.174672592	0.391046046	26	8	40
Diversity	Standard	late	2.348143394	0.399621056	26	8	40
Evenness	Enhanced	pre	0.511098554	0.032204346	26	8	40
Evenness	Enhanced	early	0.530047447	0.054838781	26	8	40
Evenness	Enhanced	mid	0.526577456	0.054930197	26	8	40
Evenness	Enhanced	late	0.510385197	0.066453177	26	8	40
Evenness	Standard	pre	0.55950048	0.005620713	26	8	40
Evenness	Standard	early	0.437231956	0.072482943	26	8	40
Evenness	Standard	mid	0.44999121	0.079397006	26	8	40
Evenness	Standard	late	0.481914416	0.078433332	26	8	40
Richness	Enhanced	pre	142.5960499	12.58194463	26	8	40
Richness	Enhanced	early	157.7886307	27.62118299	26	8	40
Richness	Enhanced	mid	145.5518058	3.353079926	26	8	40
Richness	Enhanced	late	148.532886	14.45062829	26	8	40
Richness	Standard	pre	130.4324658	8.392078482	26	8	40
Richness	Standard	early	87.82145707	5.968387977	26	8	40
Richness	Standard	mid	98.06725982	6.698991902	26	8	40
Richness	Standard	late	105.1245058	11.13191624	26	8	40

Table S17: Enzymatic Functions (EC_DGE), Enhanced Diet, Early in Mission

	Description	baseMean	log2FoldChange	lfcSE	stat	pvalue	padj
4.2.1.22	cystathionine beta-synthase	0.640036229	-20.57248121	4.118113293	-4.995608364	5.87E-07	0.001163627
4.4.1.1	cystathionine gamma-lyase	1.197801709	-19.35740603	4.105199005	-4.715339258	2.41E-06	0.002393779
3.4.11.23	PepB aminopeptidase	1.373859969	-16.29374604	4.129614631	-3.945585121	7.96E-05	0.052645668
3.4.22.16	cathepsin H	0.484790941	-15.80600657	4.129688215	-3.827409178	0.000129499	0.064231575
5.4.99.60	precorrin-8X/cobalt-precorrin-8 methylmutase	99.4358351	1.009069369	0.297893641	3.387347798	0.000705719	0.20002082
5.4.3.8	glutamate-1-semialdehyde 2,1-aminomutase	116.1926709	0.816975008	0.234932293	3.477491307	0.000506129	0.20002082
5.4.99.61	precorrin-8X/cobalt-precorrin-8 methylmutase	99.4358351	1.009069369	0.297893641	3.387347798	0.000705719	0.20002082
4.2.1.7	altronate hydrolase	427.3465533	1.201149066	0.370264906	3.244026227	0.001178529	0.292275177
1.1.1.100	3-oxoacyl-[acyl-carrier protein] reductase	2297.248751	1.623464179	0.506367168	3.206100795	0.001345468	0.296601056
5.4.99.23	23S rRNA pseudouridine1911/1915/1917 synthase	11541.25338	-3.710173754	1.181957581	-3.139007535	0.001695211	0.33632984
2.5.1.26	alkyldihydroxyacetonephosphate synthase	21.86515454	2.334437541	0.797648931	2.926647866	0.003426366	0.617991803
2.4.2.19	nicotinate-nucleotide pyrophosphorylase (carboxylating)	205.1715531	-0.666935129	0.2479912	-2.689349984	0.007159132	0.748728524
2.7.8.12	CDP-glycerol glycerophosphotransferase	49.11916204	-1.43699532	0.532457406	-2.698798636	0.006959027	0.748728524
2.4.1.208	1,2-diacylglycerol-3-alpha-glucose alpha-1,2-glucosyltransferase	21.76042831	-1.699466039	0.618071526	-2.749626813	0.005966317	0.748728524
3.6.3.3	#N/A	686.2195542	1.130465389	0.402162888	2.81096397	0.004939332	0.748728524
3.6.3.30	iron(III) transport system ATP-binding protein	109.2747114	1.104867003	0.410909885	2.688830429	0.007170283	0.748728524
2.3.1.57	diamine N-acetyltransferase	200.7568856	1.30220694	0.478762405	2.719944017	0.006529297	0.748728524
3.1.31.1	micrococcal nuclease	51.00902493	2.471063197	0.898975798	2.748753861	0.005982229	0.748728524
3.6.3.5	Cd2+/Zn2+-exporting ATPase	686.2195542	1.130465389	0.402162888	2.81096397	0.004939332	0.748728524
3.1.12.1	CRISPR-associated exonuclease Cas4	89.17767433	0.907973622	0.350693439	2.589080725	0.009623253	0.95462672
5.3.1.29	ribose 1,5-bisphosphate isomerase	0.633492245	-1.7637841	3.214975826	-0.548615043	0.58326966	0.999978546
4.4.1.25	L-cysteate sulfo-lyase	0.961780025	2.572503907	3.419506681	0.752302641	0.45186908	0.999978546
3.2.1.99	arabinan endo-1,5-alpha-L-arabinosidase	192.2445618	-0.444804019	0.36335921	-1.224144063	0.220897886	0.999978546
2.8.1.7	cysteine desulfurase	1018.936717	0.123827976	0.180563198	0.685787457	0.492847147	0.999978546
3.4.22.-	#N/A	1.269924312	1.331699326	2.751799274	0.483937669	0.628430106	0.999978546
2.5.1.9	#N/A	198.0755275	-0.633591828	0.595965465	-1.063135141	0.28772067	0.999978546
3.2.1.22	alpha-galactosidase	849.0917731	-0.154591951	0.188754904	-0.819008924	0.412781322	0.999978546
5.3.1.17	4-deoxy-L-threo-5-hexosulose-uronate ketol-isomerase	320.2434146	0.229141374	0.394881362	0.58027903	0.561726466	0.999978546
3.2.1.132	chitosanase	0.19424192	-1.043494191	4.132385508	-0.252516177	0.800642112	0.999978546
6.3.2.26	N-(5-amino-5-carboxypentanoyl)-L-cysteinyl-D-valine synthase	0.04941368	0.09441837	4.143731151	0.022785834	0.981821108	0.999978546
3.1.3.18	phosphoglycolate phosphatase	615.2274367	-0.6947175	0.41706546	-1.665727727	0.095767657	0.999978546
1.3.8.6	glutaryl-CoA dehydrogenase	0.043850487	0.094417027	4.143731151	0.02278551	0.981821367	0.999978546
1.1.1.44	6-phosphogluconate dehydrogenase	235.2879129	0.338223569	0.402522354	0.840260338	0.400762434	0.999978546
6.1.1.14	#N/A	786.3440721	0.077483559	0.326428035	0.237367966	0.812371339	0.999978546
2.5.1.47	cysteine synthase	467.5769601	-0.479859005	0.341892906	-1.403536008	0.160457062	0.999978546
6.3.2.7	UDP-N-acetylmuramoyl-L-alanyl-D-glutamate-L-lysine ligase	3.344305032	-1.510174579	1.675465748	-0.901346137	0.36740431	0.999978546
3.5.2.10	creatinine amidohydrolase	54.4423082	-0.635275762	0.331535043	-1.916164749	0.055344129	0.999978546
4.2.2.20	chondroitin-sulfate-ABC endolyase/exolyase	72.26463302	-0.606250728	0.533159844	-1.137090003	0.255500663	0.999978546
1.2.3.3	pyruvate oxidase	1.435492674	-0.391283186	3.065516787	-0.127640203	0.898433714	0.999978546
1.1.1.6	glycerol dehydrogenase	111.0345153	0.095488706	0.418274432	0.228291999	0.819419244	0.999978546
2.7.7.70	D-beta-D-heptose 7-phosphate kinase / D-beta-D-heptose 1-ph	16.49374106	-0.704306644	0.679337258	-1.036755508	0.299849816	0.999978546
6.1.1.11	seryl-tRNA synthetase	603.6102613	0.020116799	0.152886326	0.131580104	0.895316423	0.999978546
3.1.1.41	cephalosporin-C deacetylase	11.56161355	0.649501231	1.003089737	0.647500624	0.517307991	0.999978546
2.5.1.39	4-hydroxybenzoate polyprenyltransferase	8.366356724	-1.376399845	0.893957721	-1.539669957	0.123640824	0.999978546
2.6.1.37	2-aminoethylphosphonate-pyruvate transaminase	24.00016452	-0.894544965	0.611007963	-1.464047966	0.143180843	0.999978546
1.14.19.3	linoleoyl-CoA desaturase	0.048153551	-0.266241886	4.143731151	-0.064251728	0.94876979	0.999978546
2.1.1.-	#N/A	2085.570337	-0.832165565	0.492227748	-1.690610835	0.090911154	0.999978546
1.5.1.43	carboxynorspermidine synthase	0.02470684	0.094418984	4.143731151	0.022785982	0.98182099	0.999978546
2.3.3.13	2-isopropylmalate synthase	404.3772946	-0.070662667	0.192287774	-0.367483931	0.713258076	0.999978546
3.5.1.28	N-acetylmuramoyl-L-alanine amidase	701.2008379	-0.133324026	0.159069505	-0.8381495	0.401946745	0.999978546
2.4.2.3	uridine phosphorylase	208.4116149	-0.641007538	0.365412028	-1.75420481	0.079395419	0.999978546
3.4.19.5	beta-aspartyl-peptidase (threonine type)	13.69378794	-0.090612553	0.451860933	-0.200531949	0.841064574	0.999978546
1.15.1.2	superoxide reductase	676.2410151	-0.1576551	0.606805445	-0.25981161	0.795009095	0.999978546
2.4.1.20	cellobiose phosphorylase	381.5764817	-0.289482082	0.411327853	-0.703774568	0.481573174	0.999978546
3.1.11.6	#N/A	653.3635538	0.349712168	0.195398523	1.78973803	0.073496036	0.999978546
1.3.1.106	precorrin-6A/cobalt-precorrin-6A reductase	61.18763666	0.54881533	0.333083527	1.647680792	0.099418192	0.999978546
2.8.4.5	threonylcarbamoyladenosine tRNA methylthiotransferase CDK/	414.9569557	0.129950319	0.365203394	0.355829986	0.721967891	0.999978546
2.7.7.23	UDP-N-acetylglucosamine/UDP-N-acetylgalactosamine diphosp	454.2087906	-0.265505969	0.35673129	-0.744274406	0.456710482	0.999978546
4.1.1.23	orotidine-5'-phosphate decarboxylase	397.5941849	-0.216624315	0.140428754	-1.542592304	0.122929726	0.999978546
2.7.4.2	phosphomevalonate kinase	2.382155935	-1.288897093	1.904562098	-0.676741963	0.498569687	0.999978546
3.5.1.54	allophanate hydrolase	0.281440112	1.225768115	4.117206353	0.297718407	0.765918094	0.999978546
4.2.1.49	urocanate hydratase	239.7870597	0.04670733	0.292432718	0.159719918	0.87310171	0.999978546
2.4.1.44	UDP-D-galactose:(glucosyl)LPS alpha-1,3-D-galactosyltransferas	0.029793698	0.965488865	4.130968313	0.233719746	0.815202557	0.999978546
4.2.1.42	galactarate dehydratase	7.014879709	1.047450753	0.873288301	1.199432938	0.230359646	0.999978546
2.7.7.24	glucose-1-phosphate thymidylyltransferase	374.5950283	0.117095232	0.178609341	0.655594114	0.512085314	0.999978546
2.1.1.187	23S rRNA (guanine745-N1)-methyltransferase	118.7001715	0.31561169	0.335276524	0.941347418	0.34652685	0.999978546
3.4.11.18	methionyl aminopeptidase	1322.739451	0.396101141	0.387199509	1.022989782	0.306312677	0.999978546
2.1.1.204	tRNA (cytosine38-C5)-methyltransferase	0.015832449	0.094415661	4.143731151	0.02278518	0.981821629	0.999978546
1.2.1.43	#N/A	5.547997314	-0.476765951	0.916458312	-0.520226555	0.602905679	0.999978546
2.7.1.66	undecaprenol kinase	2.560303601	-0.116336523	2.075615585	-0.056049166	0.95530264	0.999978546
2.6.1.1	aspartate aminotransferase, chloroplastic	444.3986427	-0.071169352	0.252627917	-0.281716101	0.778161208	0.999978546
4.3.1.19	threonine dehydratase	237.8157219	-0.214558535	0.248427194	-0.863667669	0.387770479	0.999978546
3.5.1.10	formyltetrahydrofolate deformylase	26.97695206	-0.451298345	0.762275046	-0.592041347	0.553822902	0.999978546
3.7.1.12	cobalt-precorrin 5A hydrolase	120.4272526	0.158875015	0.364449813	0.435931119	0.662886706	0.999978546
1.1.1.62	17beta-estradiol 17-dehydrogenase	0.374948568	0.094405584	4.143731151	0.022782748	0.981823569	0.999978546
1.3.3.4	#N/A	5.486609838	-0.294793178	0.940340799	-0.313496105	0.75390378	0.999978546
3.5.4.16	GTP cyclohydrolase IA	118.9073355	0.075820591	0.355486456	0.213286863	0.83110322	0.999978546
2.1.1.200	tRNA (cytidine32/uridine32-2'-O)-methyltransferase	1.277044293	-1.712613237	4.070768509	-0.420710053	0.673966819	0.999978546
5.3.1.6	#N/A	448.1376431	0.07692739	0.217274422	0.354056356	0.723296649	0.999978546
1.1.1.335	UDP-N-acetyl-2-amino-2-deoxyglucuronate dehydrogenase	1.283507249	-1.377157395	2.361352593	-0.583207014	0.55975395	0.999978546
1.1.1.350	ureidoglycolate dehydrogenase (NAD+)	0.461004988	-1.73016098	4.07175159	-0.424918108	0.670896374	0.999978546
2.3.1.181	lipoyl(octanoyl) transferase	68.98595382	-0.109287765	0.423663985	-0.257958592	0.796438865	0.999978546
1.2.1.18	Oxidoreductases	18.30084634	0.430946664	0.485504764	0.887626025	0.37474195	0.999978546
2.1.1.104	caffeoyl-CoA O-methyltransferase	0.214256324	0.094408575	4.143731151	0.02278347	0.981822993	0.999978546
5.4.2.8	phosphomannomutase	164.6440248	0.058696252	0.268115988	0.218921117	0.826711496	0.999978546

1.2.99.5	#N/A	122.8406003	1.625383884	1.128103054	1.440811527	0.149637936	0.999978546
2.7.1.191	PTS system, mannose-specific IIA component	129.4912911	-0.20828213	0.463718604	-0.449156294	0.653318914	0.999978546
2.5.1.63	adenosyl-fluoride synthase	1.48293681	0.824254828	1.69082856	0.487485749	0.625914139	0.999978546
2.8.3.-	#N/A	28.61882816	0.126370213	0.734962809	0.171940962	0.863483943	0.999978546
3.6.4.12	DNA replication licensing factor MCM5	5705.400828	-0.275090897	0.254371154	-1.081454764	0.279494872	0.999978546
1.3.99.32	glutaryl-CoA dehydrogenase (non-decarboxylating)	1.134042721	1.938493032	4.086254919	0.474393564	0.635219282	0.999978546
1.12.98.1	#N/A	212.01706	1.947062102	1.29366859	1.505070245	0.132306018	0.999978546
5.4.99.26	tRNA pseudouridine65 synthase	2.537247887	-1.051929005	1.968689471	-0.534329573	0.593113535	0.999978546
1.4.1.2	glutamate dehydrogenase	10.5705985	-1.53577138	1.087498232	-1.412205864	0.157889354	0.999978546
3.1.21.1	deoxyribonuclease I	2.200241027	-0.945117841	1.772542929	-0.533198845	0.593895941	0.999978546
2.7.7.83	UDP-N-acetylglucosamine/UDP-N-acetylgalactosamine diphosp	64.34672785	0.101083779	0.559030144	0.180819908	0.856508936	0.999978546
2.6.1.62	adenosylmethionine---8-amino-7-oxononanoate aminotransfer	70.13286159	-0.512605839	0.565460626	-0.906527909	0.3646565	0.999978546
2.4.1.58	UDP-glucose:(galactosyl)LPS alpha-1,2-glucosyltransferase	0.173954458	-0.86982831	4.1324545	-0.210487087	0.833287529	0.999978546
1.8.3.2	thiol oxidase	0.321384486	0.094406417	4.143731151	0.022782949	0.981823409	0.999978546
1.1.1.310	(S)-sulfolactate dehydrogenase	0.517880281	4.321106361	4.038967228	1.069854276	0.284684907	0.999978546
2.6.1.9	histidinol-phosphate aminotransferase	276.1067268	-0.035884169	0.286671178	-0.125175364	0.900384721	0.999978546
3.1.4.53	3',5'-cyclic-AMP phosphodiesterase	4.758026176	0.08501773	1.583535369	0.053688558	0.957183299	0.999978546
3.2.1.26	beta-fructofuranosidase	664.1409514	0.164746249	0.274102255	0.601039379	0.547813758	0.999978546
1.4.3.5	pyridoxamine 5'-phosphate oxidase	35.59689684	0.396834003	0.414494029	0.957393774	0.33836854	0.999978546
4.2.1.3	#N/A	719.8495603	0.079026442	0.241944176	0.326630894	0.743947074	0.999978546
5.1.3.23	UDP-GlcNAc3NAcA epimerase	1.280800804	-0.169910603	2.200293336	-0.077221796	0.938447103	0.999978546
4.2.3.4	3-dehydroquinate synthase	250.9900161	-0.229216918	0.244169727	-0.938760596	0.347853674	0.999978546
2.4.2.30	poly [ADP-ribose] polymerase 16	7.933816272	1.438085856	1.045083495	1.376048768	0.168806535	0.999978546
6.3.2.32	gamma-F420-2:alpha-L-glutamate ligase	2.771140777	0.325973329	2.411496164	0.135174724	0.892473731	0.999978546
1.3.98.3	oxygen-independent coproporphyrinogen III oxidase	588.784638	0.050025686	0.249513133	0.2004932	0.841094876	0.999978546
2.1.1.207	tRNA (cytidine/uridine-2'-O-)-methyltransferase	189.2348351	-0.266237785	0.384611919	-0.692224478	0.488796369	0.999978546
1.1.1.9	D-xylulose reductase	0.480652276	-1.548459598	4.132978662	-0.374659471	0.707913738	0.999978546
3.5.3.9	allantoate deiminase	16.8615293	-0.601497199	0.445899011	-1.348953877	0.177351781	0.999978546
1.4.1.13	glutamate synthase (NADPH) large chain	1092.906852	-0.085542023	0.127190976	-0.672547891	0.501234962	0.999978546
4.2.1.76	UDP-glucose 4,6-dehydratase	0.160692243	0.094410072	4.143731151	0.022783831	0.981822705	0.999978546
1.11.1.9	glutathione peroxidase	52.20331418	-0.244271049	0.455621753	-0.536126837	0.591870894	0.999978546
4.1.3.-	#N/A	212.8288924	-0.008507259	0.289872892	-0.02934824	0.976586854	0.999978546
3.4.23.36	signal peptidase II	258.7444701	-0.11385217	0.268091113	-0.424677151	0.671072042	0.999978546
1.14.13.89	#N/A	0.349229033	1.622061629	4.109247088	0.394734508	0.693038833	0.999978546
2.4.1.211	1,3-beta-galactosyl-N-acetylhexosamine phosphorylase	341.5823143	0.472273804	0.458670218	1.029658751	0.303170226	0.999978546
5.5.1.4	myo-inositol-1-phosphate synthase	93.41411235	0.155039472	0.432305174	0.358634319	0.71986867	0.999978546
4.1.1.96	carboxynorspermidine decarboxylase	183.4516111	-0.303256203	0.3163424	-0.958632808	0.337743759	0.999978546
2.5.1.32	15-cis-phytoene synthase	6.327521558	-0.471992407	0.851551828	-0.554273259	0.579391848	0.999978546
3.5.1.1	L-asparaginase	324.8676599	-0.172651705	0.238246761	-0.72467598	0.468650834	0.999978546
5.3.1.-	#N/A	0.692871563	0.025615955	2.809148157	0.009118762	0.992724381	0.999978546
3.4.17.-	#N/A	48.75156414	0.167599996	0.506267765	0.331050103	0.740606641	0.999978546
3.4.24.37	saccharolysin	0.321384486	0.094406417	4.143731151	0.022782949	0.981823409	0.999978546
6.2.1.-	#N/A	91.10223053	-0.117313544	0.312977696	-0.374830367	0.707786629	0.999978546
3.5.1.42	nicotinamide-nucleotide amidase	360.2838083	-0.136914978	0.205761892	-0.665404933	0.50579153	0.999978546
6.2.1.4	#N/A	4.11168199	-0.019266562	2.180318771	-0.00883658	0.992949521	0.999978546
1.17.3.2	xanthine dehydrogenase/oxidase	0.783456853	-0.626248044	4.102067892	-0.152666426	0.878661339	0.999978546
1.17.7.1	(E)-4-hydroxy-3-methylbut-2-enyl-diphosphate synthase	384.5079502	0.029406116	0.172756758	0.170216876	0.86483958	0.999978546
4.2.1.51	cyclohexadienyl dehydratase	227.5628859	0.188117195	0.342837333	0.548706856	0.58320664	0.999978546
1.3.1.28	2,3-dihydro-2,3-dihydroxybenzoate dehydrogenase	0.228525281	-0.837375406	4.136218082	-0.20244953	0.839565311	0.999978546
2.4.1.180	UDP-N-acetyl-D-mannosaminouronate:lipid I N-acetyl-D-mannc	6.876760302	0.043014557	0.836310251	0.051433731	0.958979907	0.999978546
3.2.1.18	sialidase-1	148.4124568	-0.380145156	0.296985818	-1.280011144	0.200541217	0.999978546
6.3.5.5	carbamoyl-phosphate synthase	1476.450991	-0.01942487	0.115445843	-0.168259588	0.866379065	0.999978546
2.1.1.222	2-polyprenyl-6-hydroxyphenyl methylase / 3-demethylubiquinc	0.275721832	-1.704528853	4.123016056	-0.413417952	0.679300424	0.999978546
3.4.25.2	ATP-dependent HslUV protease, peptidase subunit HslV	19.83543425	-1.524201537	0.974277472	-1.564442966	0.117713577	0.999978546
2.7.1.20	adenosine kinase	7.663806899	-0.699459179	1.057961376	-0.661138672	0.508523387	0.999978546
1.12.7.2	ferredoxin hydrogenase	28.97808088	0.545702251	0.377683468	1.444866661	0.148495342	0.999978546
1.12.1.2	NAD-reducing hydrogenase large subunit	0.096307101	-0.778791352	4.138114321	-0.188199574	0.850720206	0.999978546
1.2.1.-	#N/A	0.350393969	-1.172826888	4.132997315	-0.28377151	0.776585489	0.999978546
3.4.14.5	dipeptidyl-peptidase 4	245.7884548	-0.198686873	0.227061888	-0.875034006	0.381555403	0.999978546
5.4.3.-	#N/A	0.228801007	1.506396232	4.10628083	0.366851731	0.713729618	0.999978546
3.1.3.26	4-phytase / acid phosphatase	0.687972502	2.766230034	2.83933162	0.974253946	0.329930465	0.999978546
1.14.11.47	50S ribosomal protein L16 3-hydroxylase	1.752367224	-0.383001065	1.786866548	-0.214342289	0.830280143	0.999978546
2.7.7.64	UDP-sugar pyrophosphorylase	1.215621488	2.521702973	4.071904523	0.619293247	0.535723192	0.999978546
2.1.1.77	protein-L-isoaspartate(D-aspartate) O-methyltransferase	4.997462947	-0.76745713	1.342633505	-0.571605823	0.567589049	0.999978546
4.1.1.36	phosphopantothenoylcysteine decarboxylase	282.4352402	-0.078513266	0.149047959	-0.52676512	0.598356708	0.999978546
1.2.1.2	#N/A	428.0325481	0.700345517	0.749038696	0.934992439	0.349792196	0.999978546
1.1.1.67	mannitol 2-dehydrogenase	18.00843058	-0.417372404	1.347748932	-0.309681124	0.756803459	0.999978546
3.2.1.185	non-reducing end beta-L-arabinofuranosidase	0.132242126	1.217987059	4.121195104	0.295542198	0.767579722	0.999978546
2.7.8.7	holo-[acyl-carrier protein] synthase	46.9670023	0.181135755	0.360155679	0.502937385	0.615008296	0.999978546
2.3.1.201	UDP-2-acetamido-3-amino-2,3-dideoxy-glucuronate N-acetyltra	31.47835353	-1.025699299	0.629657424	-1.628979917	0.10331727	0.999978546
5.99.1.3	#N/A	3140.163008	0.271410535	0.194861117	1.392840902	0.163667907	0.999978546
6.3.2.1	pantoate--beta-alanine ligase	113.077425	-0.536211621	0.302421113	-1.773062788	0.076218303	0.999978546
4.3.1.12	ornithine cyclodeaminase	44.99803754	-0.707845165	0.523978464	-1.350905071	0.176725844	0.999978546
2.1.1.14	5-methyltetrahydropteroyltriglutamate--homocysteine methylt	71.03558529	-0.373263661	0.376093738	-0.992475077	0.320965831	0.999978546
2.1.3.3	ornithine carbamoyltransferase	414.3448983	0.090889627	0.219018357	0.414986343	0.678151901	0.999978546
1.1.1.53	3alpha(or 20beta)-hydroxysteroid dehydrogenase	0.015832449	0.094415661	4.143731151	0.02278518	0.981821629	0.999978546
2.1.1.246	[methyl-Co(III) methanol-specific corrinoid protein]:coenzyme M	2.314203398	0.670543488	1.989479726	0.337044645	0.736083245	0.999978546
2.6.1.81	succinylornithine aminotransferase	0.107128162	0.094412122	4.143731151	0.022784326	0.981822311	0.999978546
5.4.99.5	chorismate mutase	340.3622117	0.15406764	0.349216616	0.441180725	0.659082166	0.999978546
2.7.7.12	UDPglucose--hexose-1-phosphate uridylyltransferase	361.433388	-0.145481258	0.194288366	-0.748790373	0.453983563	0.999978546
5.2.1.13	prolycopene isomerase	5.090923286	0.473589084	2.057023102	0.230230318	0.817912804	0.999978546
6.3.4.15	biotin---protein ligase	385.5616898	-0.095476263	0.20505978	-0.465602092	0.641500346	0.999978546
3.1.3.-	#N/A	259.2790445	-0.174627397	0.257389386	-0.678456093	0.497482554	0.999978546
2.5.1.72	quinolinate synthase	172.5065621	-0.302032015	0.227853741	-1.325552144	0.184988087	0.999978546
1.8.4.8	phosphoadenosine phosphosulfate reductase	26.55279816	-0.959411738	0.683660503	-1.403345278	0.160513903	0.999978546
2.3.1.20	diacylglycerol O-acyltransferase / wax synthase	0.015832449	0.094415661	4.143731151	0.02278518	0.981821629	0.999978546
1.5.1.28	opine dehydrogenase	16.88640933	0.218069149	0.751454729	0.29019599	0.771666304	0.999978546

2.7.4.16	thiamine-monophosphate kinase	124.9748864	-0.155801599	0.288139759	-0.540715379	0.588703777	0.999978546
1.1.1.125	#N/A	27.46965	0.146408107	0.518808226	0.282200821	0.777789529	0.999978546
2.6.1.85	para-aminobenzoate synthetase component II	58.14321162	-0.732449992	0.455821763	-1.606878066	0.108081119	0.999978546
4.1.99.1	tryptophanase	214.4032856	-0.567764623	0.448288693	-1.2665156	0.205328548	0.999978546
4.4.1.16	selenocysteine lyase	211.520852	0.027880756	0.262900865	0.106050454	0.915542322	0.999978546
2.7.1.45	2-dehydro-3-deoxygluconokinase	656.6700001	0.337923901	0.31461821	1.074076102	0.282788574	0.999978546
2.7.1.92	5-dehydro-2-deoxygluconokinase	8.500724103	-0.244971315	0.825450051	-0.296773032	0.766639795	0.999978546
5.3.3.-	#N/A	0.107481033	-0.255046689	4.141265221	-0.061586659	0.950892001	0.999978546
3.4.22.47	gingipain K	144.844054	0.680919183	2.751374696	0.247483261	0.804534244	0.999978546
3.1.27.6	ribonuclease I (enterobacter ribonuclease)	0.096307101	-0.778791352	4.138114321	-0.188199574	0.850720206	0.999978546
4.3.3.7	4-hydroxy-tetrahydrodipicolinate synthase	498.9117405	0.145593735	0.17497681	0.832074465	0.405366914	0.999978546
1.1.1.367	UDP-2-acetamido-2,6-beta-L-arabino-hexul-4-ose reductase	104.7367404	-0.025449934	0.460062647	-0.055318411	0.955884795	0.999978546
2.4.1.161	oligosaccharide 4-alpha-D-glucosyltransferase	0.654538727	0.588097185	4.089404042	0.143809997	0.885650508	0.999978546
1.14.12.19	3-phenylpropionate/trans-cinnamate dioxygenase subunit alph	0.192614202	-1.143334171	4.13091893	-0.276774778	0.781953049	0.999978546
2.7.7.42	[glutamine synthetase] adenylyltransferase / [glutamine synthe	8.353306205	-0.14483586	0.989410073	-0.146386078	0.883616617	0.999978546
5.1.3.11	cellobiose epimerase	175.8677582	-0.291080509	0.576520934	-0.504891481	0.613635058	0.999978546
1.6.3.3	NADH oxidase (H2O2-forming)	4.970299014	1.985961611	1.497525449	1.326162178	0.184785984	0.999978546
2.6.1.19	4-aminobutyrate aminotransferase	25.77244632	0.960563822	0.563638555	1.70421951	0.088340086	0.999978546
2.1.2.11	3-methyl-2-oxobutanoate hydroxymethyltransferase	154.993389	-0.391559559	0.23189401	-1.688528125	0.091309895	0.999978546
5.4.3.5	D-ornithine 4,5-aminomutase subunit beta	40.98653382	1.067584569	0.846708486	1.260864379	0.207357714	0.999978546
2.1.1.71	phosphatidyl-N-methylethanolamine N-methyltransferase	0.04941368	0.09441837	4.143731151	0.022785834	0.981821108	0.999978546
2.6.1.21	D-alanine transaminase	35.0129599	0.180511529	0.390317222	0.462473903	0.643741512	0.999978546
2.7.8.6	undecaprenyl-phosphate galactose phosphotransferase	3.522097742	0.012740551	1.846469619	0.006899952	0.994494679	0.999978546
5.4.99.22	23S rRNA pseudouridine2605 synthase	323.9159727	0.005095767	0.271146545	0.018793407	0.985005913	0.999978546
1.1.1.130	3-dehydro-L-gulonate 2-dehydrogenase	15.95022918	0.404308265	0.554890264	0.728627427	0.466229597	0.999978546
2.7.4.26	isopentenyl phosphate kinase	4.547246367	1.59966924	2.211157545	0.723453308	0.469401431	0.999978546
1.3.5.3	menaquinone-dependent protoporphyrinogen oxidase	8.639531851	0.443225149	0.766416995	0.578308091	0.563056133	0.999978546
1.1.1.90	aryl-alcohol dehydrogenase	0.138139806	-0.614846187	4.142596876	-0.148420473	0.882010944	0.999978546
3.1.3.78	phosphatidylinositol-4,5-bisphosphate 4-phosphatase	0.330216866	-0.544028853	4.105165977	-0.132522986	0.894570643	0.999978546
3.4.11.21	aspartyl aminopeptidase	198.5834177	-0.292007747	0.240339123	-1.214982158	0.224372896	0.999978546
3.4.21.-	#N/A	471.7887788	0.034226872	0.201019178	0.1702667	0.864800398	0.999978546
1.5.1.21	delta1-piperideine-2-carboxylate reductase	0.021925243	0.094418168	4.143731151	0.022785785	0.981821147	0.999978546
3.2.1.15	polygalacturonase	0.107128162	0.094412122	4.143731151	0.022784326	0.981822311	0.999978546
2.4.1.144	beta-1,4-mannosyl-glycoprotein beta-1,4-N-acetylglucosaminyl	2.052878989	-1.196752063	1.968553352	-0.607934787	0.543230726	0.999978546
1.1.1.83	tartrate dehydrogenase/decarboxylase / D-malate dehydrogen	0.660399619	0.688830431	4.077962478	0.168915343	0.865863231	0.999978546
2.1.1.173	23S rRNA (guanine2445-N2)-methyltransferase / 23S rRNA (gua	1.561324307	-1.183420817	1.776978591	-0.665973593	0.505427979	0.999978546
1.1.1.287	D-arabinitol dehydrogenase (NADP+)	0.353113201	0.710477296	4.127907925	0.172115587	0.863346659	0.999978546
6.1.1.-	#N/A	4.095223106	1.210442672	1.506215283	0.803631915	0.421609586	0.999978546
6.4.1.3	acetyl-CoA/propionyl-CoA carboxylase	161.1962201	0.404952126	0.308650211	1.31200988	0.189516801	0.999978546
1.7.2.2	nitrite reductase (cytochrome c-552)	50.95058939	-0.808438158	0.521351682	-1.550658002	0.120983664	0.999978546
5.4.4.2	salicylate biosynthesis isochorismate synthase	53.1414597	-0.022006074	0.359783828	-0.061164712	0.951228033	0.999978546
3.1.4.14	acyl carrier protein phosphodiesterase	0.144460652	-1.00194317	4.133461154	-0.24239811	0.808471699	0.999978546
2.3.1.8	phosphate acetyltransferase	784.4992269	1.018307724	0.506864705	2.009032617	0.044533675	0.999978546
3.1.13.3	#N/A	459.8598424	0.246767007	0.313728516	0.786562247	0.431538167	0.999978546
2.1.1.182	16S rRNA (adenine1518-N6/adenine1519-N6)-dimethyltransfer	406.2000725	-0.349218741	0.43741189	-0.798375054	0.424652875	0.999978546
4.2.2.2	pectate lyase	6.801543122	0.40809976	1.254356635	0.325345877	0.744919313	0.999978546
2.7.3.9	#N/A	939.61253	-0.123322056	0.306627056	-0.402189087	0.687544873	0.999978546
4.3.1.3	histidine ammonia-lyase	192.3498098	-0.221266155	0.276365644	-0.800628296	0.423346865	0.999978546
3.1.13.1	exoribonuclease II	8.613747685	-0.967153548	1.044796091	-0.925686416	0.354608969	0.999978546
2.1.1.297	release factor glutamine methyltransferase	376.6235411	0.290963089	0.217559068	1.337398122	0.181092717	0.999978546
3.6.3.10	H+/K+-exchanging ATPase	0.264436359	0.045186449	4.14168021	0.010910173	0.991295114	0.999978546
3.5.1.87	beta-ureidopropionase / N-carbamoyl-L-amino-acid hydrolase	61.22171734	0.016439138	0.464429838	0.035396387	0.971763666	0.999978546
3.4.21.62	subtilisin	0.654451996	-2.4985976	4.114580796	-0.607254475	0.543682046	0.999978546
2.7.1.166	3-deoxy-D-manno-octulosonic acid kinase	0.202265241	1.087836243	4.116916993	0.264235651	0.791598341	0.999978546
3.1.1.29	peptidyl-tRNA hydrolase, PTH1 family	247.2072187	0.303892061	0.323062634	0.940659886	0.346879182	0.999978546
6.4.1.4	#N/A	0.160692243	0.094410072	4.143731151	0.022783831	0.981822705	0.999978546
2.3.1.42	glyceronephosphate O-acyltransferase	0.214256324	0.094408575	4.143731151	0.02278347	0.981822993	0.999978546
1.18.1.2	ferredoxin/flavodoxin---NADP+ reductase	452.6425692	-0.396838955	0.27315823	-1.452780517	0.146284703	0.999978546
1.8.4.12	peptide-methionine (R)-S-oxide reductase	97.44710833	-0.221670146	0.296631142	-0.747292225	0.454887185	0.999978546
3.5.2.17	5-hydroxyisourate hydrolase	0.098827359	0.094416485	4.143731151	0.022785379	0.981821471	0.999978546
5.1.3.7	UDP-N-acetylglucosamine 4-epimerase	49.44551409	-0.008977958	0.473580077	-0.018957634	0.984874902	0.999978546
3.4.24.28	bacillolysin	0.63373079	-1.532443003	4.108757074	-0.37296997	0.709170794	0.999978546
1.1.2.4	D-lactate dehydrogenase (cytochrome)	3.268088468	-0.220745983	1.341565843	-0.164543532	0.86930328	0.999978546
2.7.11.11	protein kinase A	0.642768973	0.094402618	4.143731151	0.022782033	0.98182414	0.999978546
5.1.3.26	GlcNAc-P-P-Und epimerase	22.84782111	-0.602251476	0.59022964	-1.020368065	0.307553933	0.999978546
1.3.1.98	UDP-N-acetylmuramate dehydrogenase	353.7006	-0.086752212	0.170278941	-0.509471174	0.610421998	0.999978546
5.1.3.30	D-psicose/D-tagatose/L-ribulose 3-epimerase	6.991926601	-1.091871458	1.002069612	-1.089616376	0.275882166	0.999978546
4.2.1.93	ATP-dependent NAD(P)H-hydrate dehydratase	0.015832449	0.094415661	4.143731151	0.02278518	0.981821629	0.999978546
4.2.3.153	(5-formylfuran-3-yl)methyl phosphate synthase	5.230391143	3.467460143	2.265853003	1.530311163	0.125939727	0.999978546
2.4.1.5	dextransucrase	2.832586369	-2.299674075	2.434314578	-0.94469059	0.344816863	0.999978546
4.1.1.7	benzoylformate decarboxylase	0.102122587	1.526602962	4.11330392	0.371137896	0.710534827	0.999978546
2.3.1.75	diacylglycerol O-acyltransferase / wax synthase	0.015832449	0.094415661	4.143731151	0.02278518	0.981821629	0.999978546
3.4.23.40	phytepsin	0.116409678	1.169478482	4.119476897	0.283890045	0.776494646	0.999978546
3.2.1.123	endoglycosylceramidase	0.69313487	-1.110637924	4.131041207	-0.268851814	0.788043719	0.999978546
2.7.1.25	adenylylsulfate kinase	63.6182501	-0.05506749	0.280494368	-0.196322977	0.844357378	0.999978546
2.9.1.-	#N/A	0.431659391	-1.960786489	4.107379977	-0.477381323	0.633090611	0.999978546
4.2.1.28	propanediol dehydratase large subunit	328.6225478	0.724426463	0.929562762	0.779319582	0.435791482	0.999978546
5.3.3.18	2-(1,2-epoxy-1,2-dihydrophenyl)acetyl-CoA isomerase	18.26828696	0.302553943	0.989252091	0.305841095	0.759725637	0.999978546
3.1.2.1	acetyl-CoA hydrolase	1.659607312	1.857053154	4.052018557	0.45830322	0.646734611	0.999978546
1.13.11.3	#N/A	0.337017706	-1.685381526	4.124854684	-0.408591733	0.682839298	0.999978546
2.1.1.213	tRNA (guanine10-N2)-dimethyltransferase	2.980175938	2.628487716	2.511380629	1.046630561	0.295270001	0.999978546
1.1.1.178	3-hydroxyacyl-CoA dehydrogenase / 3-hydroxy-2-methylbutyry	0.642768973	0.094402618	4.143731151	0.022782033	0.98182414	0.999978546
2.7.1.56	1-phosphofructokinase	215.1212971	0.136686529	0.208146451	0.656684405	0.511383864	0.999978546
1.1.1.159	7-alpha-hydroxysteroid dehydrogenase	12.0655719	-0.477980234	0.7377525	-0.647886973	0.517058057	0.999978546
2.7.1.211	PTS system, sucrose-specific IIA component	72.37468344	0.050051883	0.408158199	0.122628636	0.902401177	0.999978546
3.2.1.4	endoglucanase	878.084613	-0.288775928	0.466920664	-0.618468938	0.536266267	0.999978546
2.7.7.33	glucose-1-phosphate cytidylyltransferase	68.99084955	0.004063727	0.391532152	0.010379038	0.991718875	0.999978546

2.4.1.41	polypeptide N-acetylgalactosaminyltransferase	0.290251399	-0.227952829	4.141590138	-0.055039929	0.956106653	0.999978546
4.2.1.130	D-lactate dehydratase / protein deglycase	0.096307101	-0.778791352	4.138114321	-0.188199574	0.850720206	0.999978546
2.8.1.11	adenylyltransferase and sulfurtransferase	19.1465002	0.370191641	0.53007793	0.698372108	0.484944515	0.999978546
2.2.1.7	1-deoxy-D-xylulose-5-phosphate synthase	707.1842448	-0.022694025	0.14033945	-0.161708096	0.871535729	0.999978546
3.4.13.22	zinc D-Ala-D-Ala dipeptidase	58.536724	0.007173594	0.396837797	0.018076892	0.985577512	0.999978546
4.4.1.8	#N/A	368.3176599	0.05914998	0.234782104	0.251935643	0.801090809	0.999978546
1.3.1.101	digeranylgeranylglycerophospholipid reductase	9.718970903	0.519816564	1.424842256	0.364823939	0.715242834	0.999978546
3.1.3.82	D-glycero-D-manno-heptose 1,7-bisphosphate phosphatase	19.00358607	-0.041827755	0.473573283	-0.088323722	0.929619385	0.999978546
2.7.1.144	tagatose 6-phosphate kinase	13.17025696	-0.267908845	0.840085994	-0.318906454	0.74979745	0.999978546
1.5.1.24	N5-(carboxyethyl)ornithine synthase	0.256368638	2.597887729	4.087268065	0.635604929	0.525033956	0.999978546
2.7.10.2	protein-tyrosine kinase	0.471880068	-1.387469179	4.117889987	-0.336936922	0.736164451	0.999978546
2.1.1.289	cobalt-precorrin-7 (C5)-methyltransferase	2.079831392	-0.548244706	2.014070813	-0.272207264	0.785462656	0.999978546
1.10.3.9	photosystem II P680 reaction center D1 protein	1.966809294	-1.061705029	3.986915372	-0.266297358	0.790010208	0.999978546
2.5.1.3	thiamine-phosphate pyrophosphorylase	291.6609735	0.05186369	0.155339948	0.333872197	0.738476001	0.999978546
1.16.1.3	aquacobalamin reductase / NAD(P)H-flavin reductase	1.180331694	-2.341226808	4.114238676	-0.569054689	0.569319027	0.999978546
2.1.1.195	cobalt-precorrin-5B (C1)-methyltransferase	169.9058599	-0.010770069	0.336827185	-0.031975059	0.974491941	0.999978546
5.3.1.9	glucose-6-phosphate isomerase	902.8368241	0.099170951	0.28051274	0.353534571	0.723687716	0.999978546
2.7.2.3	phosphoglycerate kinase	1431.260499	-0.215834046	0.499561527	-0.432046974	0.665707273	0.999978546
5.3.3.2	isopentenyl-diphosphate Delta-isomerase	13.07900694	0.385239197	0.785269425	0.490582194	0.623721983	0.999978546
3.4.18.1	cathepsin X	4.756805142	-0.234519817	4.098481533	-0.057221148	0.954369032	0.999978546
3.5.1.5	urease	257.9239362	-0.151882609	0.714601311	-0.212541745	0.831684414	0.999978546
4.2.1.99	aconitate hydratase 2 / 2-methylisocitrate dehydratase	3.37656264	-5.440032155	3.208214283	-1.695657358	0.089950789	0.999978546
2.3.1.241	Kdo2-lipid IVA lauroyltransferase	95.41321668	-0.388726881	0.292364309	-1.329597592	0.183650892	0.999978546
1.2.1.19	aminobutyraldehyde dehydrogenase	0.383706937	-1.324504817	4.13126725	-0.320604971	0.74850977	0.999978546
6.3.5.9	cobyrinic acid a,c-diamide synthase	160.4431359	0.274826239	0.334565291	0.82144277	0.41139411	0.999978546
1.1.1.169	2-dehydropantoate 2-reductase	326.602218	0.06913519	0.250328013	0.2761784	0.782411047	0.999978546
3.5.2.2	dihydropyrimidinase	90.86795266	-0.435327567	0.327665527	-1.328572984	0.18398889	0.999978546
1.18.6.1	nitrogenase delta subunit	63.88890217	0.307843592	0.365213063	0.842915062	0.399275949	0.999978546
2.7.7.80	adenylyltransferase and sulfurtransferase	19.1465002	0.370191641	0.53007793	0.698372108	0.484944515	0.999978546
5.3.1.25	L-fucose/D-arabinose isomerase	329.4791065	-0.485672569	0.383032063	-1.267968445	0.20480922	0.999978546
3.6.3.14	#N/A	3022.576656	0.179934676	0.262596657	0.685213124	0.493209443	0.999978546
2.1.1.181	23S rRNA (adenine1618-N6)-methyltransferase	4.035600293	-0.699343146	0.991634423	-0.70524291	0.480659082	0.999978546
2.3.1.129	UDP-N-acetylglucosamine acyltransferase	179.9466588	-0.369045769	0.334383327	-1.103660798	0.269740307	0.999978546
1.16.1.10	ferric-chelate reductase [NAD(P)H]	0.174502634	-0.586352982	4.140180137	-0.141624993	0.887376225	0.999978546
4.2.1.104	cyanate lyase	0.715825094	1.130592278	3.693705661	0.306086186	0.759539025	0.999978546
3.5.99.7	1-aminocyclopropane-1-carboxylate deaminase	0.33950778	-1.066659778	4.134355493	-0.257999047	0.796407643	0.999978546
1.3.99.4	3-oxosteroid 1-dehydrogenase	1.106189635	0.741588436	3.249046017	0.228248056	0.819453404	0.999978546
1.2.1.22	lactaldehyde dehydrogenase / glycolaldehyde dehydrogenase	36.22550726	0.351430307	0.404010139	0.869855166	0.384379559	0.999978546
2.4.1.318	demethyllactenocin mycarosyltransferase	0.022430994	-0.266241886	4.143731151	-0.064251728	0.94876979	0.999978546
3.6.1.6	ectonucleoside triphosphate diphosphohydrolase 5/6	0.749897135	0.094401748	4.143731151	0.022781823	0.981824308	0.999978546
3.1.27.1	ribonuclease T2	0.308633526	1.236305445	4.112977	0.30058652	0.763729812	0.999978546
1.1.1.40	malate dehydrogenase (oxaloacetate-decarboxylating)(NADP+)	110.9790257	-0.254040852	0.355228859	-0.715146998	0.474518215	0.999978546
3.2.1.51	alpha-L-fucosidase	1023.186115	-0.157112191	0.174456114	-0.900582885	0.367810138	0.999978546
5.4.99.9	UDP-galactopyranose mutase	320.18863	0.016082175	0.254187645	0.063268908	0.949552374	0.999978546
4.1.99.17	phosphomethylpyrimidine synthase	279.9666111	0.158157664	0.243097364	0.650593911	0.515308662	0.999978546
5.1.3.9	N-acylglucosamine-6-phosphate 2-epimerase	169.6607425	-0.148097938	0.457633188	-0.323617128	0.746227919	0.999978546
1.3.1.10	enoyl-acyl-carrier protein] reductase I	82.40735219	-0.194070282	0.288870673	-0.671824106	0.50169568	0.999978546
2.7.6.5	GTP pyrophosphokinase	1266.617419	0.014720185	0.146090615	0.100760647	0.919740467	0.999978546
1.2.1.72	D-erythrose 4-phosphate dehydrogenase	1.431547706	-1.773584781	4.117358794	-0.430757889	0.666644422	0.999978546
5.1.3.25	dTDP-L-rhamnose 4-epimerase	1.791576241	0.878860531	1.78891376	0.491281665	0.623227246	0.999978546
4.2.1.32	#N/A	69.34122182	-0.688490554	0.548728977	-1.254700558	0.209587483	0.999978546
4.2.1.149	crotonobetainyl-CoA hydratase	3.918396303	0.168910765	1.972543533	0.085630944	0.931759799	0.999978546
2.3.1.88	#N/A	0.364127506	-0.67749285	4.136053234	-0.163801772	0.869887196	0.999978546
5.5.1.1	muconate cycloisomerase	0.138446491	0.034232959	4.127762859	0.008293344	0.993382944	0.999978546
3.6.1.55	8-oxo-dGTP diphosphatase	335.946494	-0.137069743	0.24947925	-0.549423421	0.582714905	0.999978546
2.7.1.177	L-threonine kinase	0.594267165	-1.391212398	4.126814367	-0.337115333	0.73602996	0.999978546
3.7.1.17	4,5:9,10-diseco-3-hydroxy-5,9,17-trioxoandrosta-1(10),2-diene-	0.231939278	-0.893763178	4.12272929	-0.216789198	0.82837263	0.999978546
5.4.2.9	phosphoenolpyruvate phosphomutase	16.50042611	-0.723057725	0.769228364	-0.939977981	0.347228855	0.999978546
6.3.1.5	NAD+ synthase	39.38747508	-0.546437718	0.636633808	-0.858323437	0.390713891	0.999978546
1.18.1.-	#N/A	0.305626466	-1.906214562	4.123314889	-0.462301477	0.643865141	0.999978546
4.2.1.113	O-succinylbenzoate synthase	4.188888329	-1.245111877	1.421268278	-0.876056896	0.380999104	0.999978546
2.3.1.-	#N/A	274.7832898	-0.215805931	0.264689115	-0.815318495	0.414890025	0.999978546
1.16.3.2	ferritin heavy chain	526.0547936	0.409254397	0.454571498	0.90030809	0.367956317	0.999978546
1.11.1.21	catalase-peroxidase	8.342629727	-5.182295181	3.06714548	-1.689615056	0.091101624	0.999978546
5.1.3.-	#N/A	3.808216094	-0.445884845	1.286272345	-0.346648862	0.728855133	0.999978546
1.2.7.5	aldehyde:ferredoxin oxidoreductase	41.45335069	-0.182324684	0.678287049	-0.268801659	0.788082316	0.999978546
4.2.1.46	dTDP-glucose 4,6-dehydratase	442.2462817	0.086616474	0.170997488	0.506536529	0.612480053	0.999978546
3.5.5.1	nitrilase	12.224291	-0.043145327	0.739577386	-0.058337813	0.953479548	0.999978546
1.3.5.6	zeta-carotene desaturase	0.089381093	1.708948656	4.106035225	0.41620409	0.677260667	0.999978546
1.14.14.9	4-hydroxyphenylacetate 3-monooxygenase	3.60579661	-1.36152789	1.741369606	-0.781871858	0.434289885	0.999978546
6.3.2.35	D-alanine---D-serine ligase	27.59615683	-0.732005876	0.523715701	-1.397716117	0.162198332	0.999978546
2.7.7.1	nicotinamide-nucleotide adenylyltransferase	6.6903392	0.173954578	1.274427972	0.136496202	0.891429027	0.999978546
2.3.1.157	bifunctional UDP-N-acetylglucosamine pyrophosphorylase / Glu	389.7431354	-0.310180006	0.378632302	-0.819211684	0.41266565	0.999978546
3.1.3.6	2',3'-cyclic-nucleotide 2'-phosphodiesterase / 3'-nucleotidase	234.785924	0.09408522	0.316916785	0.296876732	0.766560621	0.999978546
3.5.2.5	allantoinase	3.997005751	0.474866262	1.303223438	0.364378239	0.715575583	0.999978546
4.3.1.29	D-glucosaminate-6-phosphate ammonia-lyase	0.089381093	1.708948656	4.106035225	0.41620409	0.677260667	0.999978546
3.1.3.27	phosphatidylglycerophosphatase GEP4	30.96879914	-0.39719669	0.516267113	-0.769362757	0.441677991	0.999978546
5.4.2.11	2,3-bisphosphoglycerate-dependent phosphoglycerate mutase	221.9404766	0.137387218	0.348837423	0.393843116	0.69369687	0.999978546
2.7.4.6	nucleoside-diphosphate kinase	76.50444022	0.234741924	0.575375395	0.40798047	0.683288013	0.999978546
3.1.26.3	#N/A	253.7334934	0.092427724	0.277711942	0.332818687	0.739271151	0.999978546
2.1.1.132	precorrin-6Y C5,15-methyltransferase (decarboxylating)	187.4625788	0.128634091	0.321121753	0.40057732	0.688731346	0.999978546
2.7.7.27	glucose-1-phosphate adenylyltransferase	2821.889123	0.185508386	0.447209213	0.414813428	0.678278489	0.999978546
1.12.1.3	NADP-reducing hydrogenase subunit HndB	339.9647259	0.129209822	0.379799601	0.34020526	0.733701957	0.999978546
3.4.24.13	IgA-specific metalloendopeptidase	0.02470684	0.094418984	4.143731151	0.022785982	0.98182099	0.999978546
1.11.1.6	#N/A	118.6950932	-0.091170081	0.418792624	-0.217697437	0.827664861	0.999978546
3.6.5.5	dynamin GTPase	0.770998033	-1.113437754	4.129171775	-0.269651595	0.787428303	0.999978546
2.7.2.8	acetylglutamate kinase	349.4705199	0.235554208	0.307704951	0.76551972	0.443962129	0.999978546

5.4.99.2	methylmalonyl-CoA mutase	438.421622	-0.054636751	0.293239306	-0.186321375	0.852192746	0.999978546
1.1.1.284	S-(hydroxymethyl)glutathione dehydrogenase / alcohol dehydr	1.229341188	0.277229961	3.409943656	0.081300452	0.935203015	0.999978546
4.1.1.82	phosphonopyruvate decarboxylase	16.79511523	0.184250245	0.51968875	0.354539607	0.722934527	0.999978546
1.1.1.197	carbonyl reductase 1	0.067578231	-0.344699102	4.140710721	-0.083246362	0.933655649	0.999978546
6.2.1.20	long-chain-fatty-acid--[acyl-carrier-protein] ligase	12.26279611	-0.882384765	0.893709585	-0.987328299	0.323481722	0.999978546
4.3.1.15	diaminopropionate ammonia-lyase	67.37423002	0.140598741	0.620669655	0.226527493	0.820791179	0.999978546
3.4.13.19	membrane dipeptidase	152.4527255	0.014861235	0.273774209	0.054282815	0.956709841	0.999978546
4.6.1.15	triose/dihydroxyacetone kinase / FAD-AMP lyase (cyclizing)	5.825853036	3.040228769	4.021328188	0.756026026	0.449633592	0.999978546
6.2.1.3	long-chain acyl-CoA synthetase	782.0385652	-0.1136	0.155265261	-0.731651103	0.464381548	0.999978546
2.7.1.196	PTS system, cellobiose-specific IIA component	67.85843679	-0.116143475	0.576732298	-0.201381951	0.840399927	0.999978546
1.1.1.38	malate dehydrogenase (oxaloacetate-decarboxylating)	427.7254982	-0.162681867	0.40688768	-0.399820086	0.689289035	0.999978546
1.14.18.6	4-hydroxysphinganine ceramide fatty acyl 2-hydroxylase	0.100382114	-0.637008364	4.143261082	-0.153745649	0.87781029	0.999978546
6.3.4.21	nicotinate phosphoribosyltransferase	581.9678307	-0.461325806	0.44237017	-1.042850168	0.297017692	0.999978546
3.6.1.25	triphosphatase	0.063329795	0.094410257	4.143731151	0.022783876	0.98182267	0.999978546
1.5.98.1	methylenetetrahydromethanopterin dehydrogenase	30.1788584	2.837153568	1.193293595	2.377582165	0.01742656	0.999978546
4.2.1.17	enoyl-CoA hydratase	1168.14638	-0.04869596	0.6272733	-0.077631169	0.938121448	0.999978546
1.1.1.252	tetrahydroxynaphthalene reductase	0.063329795	0.094410257	4.143731151	0.022783876	0.98182267	0.999978546
4.4.1.21	S-ribosylhomocysteine lyase	148.3246946	-0.079708654	0.287389408	-0.277354183	0.781508159	0.999978546
2.1.1.72	site-specific DNA-methyltransferase (adenine-specific)	1204.253236	-0.081187107	0.272571867	-0.297855784	0.765813236	0.999978546
5.4.1.4	D-galactarolactone isomerase	0.015832449	0.094415661	4.143731151	0.02278518	0.981821629	0.999978546
3.1.13.-	#N/A	1.076630519	-0.648310939	4.11852624	-0.157413332	0.874919107	0.999978546
1.3.99.28	phytoene desaturase	1.632348624	0.91736795	4.096641466	0.223931715	0.822810437	0.999978546
5.3.1.3	L-fucose/D-arabinose isomerase	329.4791065	-0.485672569	0.383032063	-1.267968445	0.20480922	0.999978546
4.2.1.11	enolase	996.8971278	0.116826072	0.40775122	0.286513114	0.774485151	0.999978546
5.1.3.13	dTDP-4-dehydrorhamnose 3,5-epimerase	207.8342078	-0.060891458	0.203431665	-0.299321434	0.764694802	0.999978546
2.4.2.7	adenine phosphoribosyltransferase	209.6202566	0.311510241	0.202100991	1.541359297	0.123229364	0.999978546
3.4.11.5	proline iminopeptidase	13.02301904	-0.79859869	0.576960481	-1.38414799	0.166313145	0.999978546
1.1.1.23	histidinol dehydrogenase	291.6884958	0.094629463	0.214952715	0.440233856	0.659767741	0.999978546
1.5.1.34	dihydropteridine reductase	6.296940433	-0.21887557	0.847657829	-0.258212173	0.796243164	0.999978546
1.8.4.11	peptide-methionine (S)-S-oxide reductase	112.1664182	-0.100264476	0.286100516	-0.350451923	0.725999565	0.999978546
1.1.99.3	#N/A	1.815348364	-2.029131848	2.667008002	-0.760827057	0.446760371	0.999978546
5.3.2.5	2,3-diketo-5-methylthiopentyl-1-phosphate enolase	8.254332185	1.498495146	1.001295693	1.496556069	0.134508808	0.999978546
3.2.1.196	glycogen debranching enzyme	167.3467347	-0.23461619	0.300952657	-0.779578395	0.435639077	0.999978546
3.1.21.5	type III restriction enzyme	189.1393977	0.058443445	0.320775702	0.182194116	0.855430391	0.999978546
3.11.1.2	phosphonoacetate hydrolase	0.50996675	-0.112426778	4.108969575	-0.027361307	0.978171559	0.999978546
1.1.1.184	carbonyl reductase 1	0.067578231	-0.344699102	4.140710721	-0.083246362	0.933655649	0.999978546
3.5.4.27	methenyltetrahydromethanopterin cyclohydrolase	3.406741929	1.300504978	1.846056278	0.704477428	0.481135502	0.999978546
2.4.1.321	cellobionic acid phosphorylase	0.076526885	-0.425564079	4.140217409	-0.102787858	0.918131342	0.999978546
1.1.1.93	tartrate dehydrogenase	0.660399619	0.688830431	4.077962478	0.168915343	0.865863231	0.999978546
2.7.7.75	molybdopterin adenylyltransferase	3.318616763	-0.149965387	1.631342138	-0.091927612	0.926755553	0.999978546
2.7.7.87	L-threonylcarbamoyladenylate synthase	333.119198	0.082225643	0.154378932	0.532622183	0.594295143	0.999978546
3.6.3.21	#N/A	586.7150326	0.080782573	0.385812653	0.209382903	0.834149339	0.999978546
2.7.12.1	#N/A	0.589204892	0.094403102	4.143731151	0.022782149	0.981824047	0.999978546
2.4.1.109	dolichyl-phosphate-mannose-protein mannosyltransferase	0.048153551	-0.266241886	4.143731151	-0.064251728	0.94876979	0.999978546
6.1.1.19	arginyl-tRNA synthetase	902.3901318	0.563778387	0.346699116	1.626131598	0.103921664	0.999978546
1.3.1.34	2,4-dienoyl-CoA reductase (NADPH2)	11.37066263	-0.473906698	0.943128971	-0.502483448	0.615327492	0.999978546
1.3.1.54	precorrin-6A/cobalt-precorrin-6A reductase	61.18763666	0.54881533	0.333083527	1.647680792	0.099418192	0.999978546
1.2.4.2	2-oxoglutarate dehydrogenase E1 component	17.17650179	-0.819995961	1.164241729	-0.704317618	0.481234997	0.999978546
2.7.1.194	PTS system, ascorbate-specific IIA component	24.17034772	0.098699658	0.641604187	0.153832628	0.877741706	0.999978546
3.2.1.183	UDP-N-acetylglucosamine 2-epimerase (hydrolysing)	12.086231	-0.682237867	0.886905046	-0.769234396	0.441754175	0.999978546
2.6.1.82	putrescine aminotransferase	66.44150576	0.539884335	0.851951269	0.633703305	0.526274468	0.999978546
1.11.1.10	non-heme chloroperoxidase	0.399388983	0.885895491	4.078145331	0.217229995	0.82802911	0.999978546
2.4.2.29	queuine tRNA-ribosyltransferase	354.7608664	0.135952109	0.218977028	0.620851009	0.534697658	0.999978546
3.4.21.102	carboxyl-terminal processing protease	593.5158334	0.122572586	0.151847991	0.80720585	0.419547896	0.999978546
2.1.1.45	thymidylate synthase	208.9073855	-0.142177798	0.310654667	-0.457671533	0.647188443	0.999978546
2.7.1.6	galactokinase	521.2465307	-0.005848474	0.138100926	-0.042349272	0.966220267	0.999978546
2.6.1.66	valine--pyruvate aminotransferase	66.13941554	-0.744477459	0.466703619	-1.595182528	0.110671425	0.999978546
2.7.1.16	L-ribulokinase	36.69650726	0.459263891	0.385776305	1.190492741	0.233852781	0.999978546
6.3.2.30	cyanophycin synthetase	4.102299911	2.004700827	3.731008814	0.537307985	0.591054891	0.999978546
4.1.1.3	#N/A	1317.677733	0.138347002	0.203613892	0.679457578	0.496847978	0.999978546
3.2.1.11	dextranase	30.10552948	-0.824163203	0.537669916	-1.532842322	0.12531471	0.999978546
1.8.1.8	thioredoxin 2	161.0699188	-0.167329072	0.253744009	-0.659440485	0.509612952	0.999978546
3.1.4.-	#N/A	256.2826558	-0.155314406	0.261437146	-0.594079336	0.552459051	0.999978546
2.3.1.46	homoserine O-acetyltransferase/O-succinyltransferase	851.3442398	-1.382849379	0.823627188	-1.678974904	0.093156935	0.999978546
6.2.1.7	solute carrier family 27 (fatty acid transporter), member 5	4.984233103	0.735623677	4.041515575	0.182016786	0.855569552	0.999978546
2.4.1.280	N,N'-diacetylchitobiose phosphorylase	8.469517485	-0.426851371	1.205797268	-0.353999285	0.723339419	0.999978546
3.4.21.96	lactocepin	296.0567306	-0.605183468	0.94233214	-0.642218856	0.520731092	0.999978546
4.1.1.-	#N/A	0.100382114	-0.637008364	4.143261082	-0.153745649	0.87781029	0.999978546
4.1.1.49	phosphoenolpyruvate carboxykinase (ATP)	1039.813312	0.063476242	0.386862716	0.164079503	0.869668557	0.999978546
2.3.1.193	tRNA(Met) cytidine acetyltransferase	0.475511433	0.78673487	4.110418739	0.191400176	0.848212082	0.999978546
1.1.1.77	lactaldehyde reductase	536.8571836	-0.032460157	0.40446348	-0.080254853	0.936034565	0.999978546
1.3.1.85	crotonyl-CoA carboxylase/reductase	0.225617469	-0.530375736	4.135469462	-0.12825043	0.897950792	0.999978546
1.6.1.1	NAD(P) transhydrogenase	1.736111865	-2.65930397	3.218736686	-0.826194942	0.408693517	0.999978546
3.2.2.16	5'-methylthioadenosine nucleosidase	0.06610909	0.087442216	4.143404008	0.021103956	0.983162729	0.999978546
1.5.1.38	FMN reductase	7.661298639	0.638704397	1.098177224	0.581604119	0.560833368	0.999978546
1.1.1.137	ribulose-5-phosphate 2-dehydrogenase	33.26585746	-0.14466666	0.751401672	-0.192529063	0.847327805	0.999978546
2.1.1.34	tRNA (guanosine-2'-O-)-methyltransferase	13.99952319	-1.245520608	0.908476573	-1.370999149	0.170375221	0.999978546
3.2.2.26	futalosine hydrolase	2.601413997	-1.670727372	2.195561215	-0.760956862	0.446682834	0.999978546
3.4.23.49	omptin	0.144460652	-1.00194317	4.133461154	-0.24239811	0.808471699	0.999978546
2.7.11.23	CTD kinase subunit alpha	0.071041121	-0.235309633	4.14207572	-0.056809592	0.954696873	0.999978546
6.1.1.4	leucyl-tRNA synthetase	730.0393366	0.052808513	0.134210668	0.393474783	0.693968845	0.999978546
4.1.2.21	2-dehydro-3-deoxyphosphogalactonate aldolase	0.272063502	-1.725855267	4.124242076	-0.418466044	0.675606408	0.999978546
3.1.1.53	sialate O-acetylesterase	284.4241936	-0.144111878	0.27608282	-0.521987852	0.601678788	0.999978546
2.6.1.77	taurine-pyruvate aminotransferase	27.34860526	0.130889605	0.973795361	0.134411818	0.893076937	0.999978546
2.1.1.217	tRNA (adenine22-N1)-methyltransferase	279.7718674	-0.01465466	0.46207342	-0.031715003	0.97469933	0.999978546
2.1.1.242	16S rRNA (guanine1516-N2)-methyltransferase	13.47765524	-0.125099317	0.621383123	-0.201323969	0.840445262	0.999978546
3.5.4.3	guanine deaminase	103.5861723	-0.072252956	0.319889281	-0.225868639	0.82130359	0.999978546

1.13.11.53	1,2-dihydroxy-3-keto-5-methylthiopentene dioxygenase	0.050191057	-0.266239077	4.143731151	-0.06425105	0.948770329	0.999978546
4.1.3.1	isocitrate lyase	1.710554568	-2.248800869	3.342572121	-0.672775571	0.501090081	0.999978546
3.4.24.75	lysostaphin	0.258895683	-1.12398086	4.133566128	-0.271915538	0.785686963	0.999978546
2.7.1.-	#N/A	1291.856995	0.101261192	0.354450899	0.285684681	0.775119639	0.999978546
2.2.1.10	fructose-bisphosphate aldolase / 2-amino-3,7-dideoxy-D-threo-	4.406406866	2.641785911	2.018978724	1.30847635	0.190711791	0.999978546
1.17.99.6	epoxyqueuosine reductase	21.31922042	-0.921043709	0.705978472	-1.304634271	0.192017413	0.999978546
2.4.99.14	3-deoxy-D-manno-octulosonic-acid transferase	102.8745696	-0.135612171	0.327661726	-0.413878582	0.67896303	0.999978546
4.2.1.24	porphobilinogen synthase	65.10279835	0.184484593	0.307276135	0.600386987	0.548248358	0.999978546
2.4.1.260	alpha-1,6-mannosyltransferase	0.214256324	0.094408575	4.143731151	0.02278347	0.981822993	0.999978546
6.3.5.4	asparagine synthase (glutamine-hydrolysing)	377.4072799	0.021288003	0.347668431	0.061230763	0.951175431	0.999978546
6.1.1.22	asparaginyl-tRNA synthetase	736.9327609	0.140006264	0.485802168	0.288196046	0.773196676	0.999978546
2.7.1.33	type I pantothenate kinase	321.0567096	-0.23945734	0.241319002	-0.992285475	0.321058287	0.999978546
6.5.1.1	bifunctional non-homologous end joining protein LigD	28.88893739	0.13144007	0.56041073	0.234542387	0.814563931	0.999978546
2.4.1.10	levansucrase	3.347830754	0.083887036	2.172651164	0.038610448	0.969200972	0.999978546
3.2.2.8	ribosylpyrimidine nucleosidase	9.911351524	-1.715800482	0.918478658	-1.868089657	0.061749575	0.999978546
4.2.1.35	3-isopropylmalate/(R)-2-methylmalate dehydratase large subur	629.01279	3.03E-05	0.236511554	0.000128048	0.999897833	0.999978546
1.1.1.343	6-phosphogluconate dehydrogenase	235.2879129	0.338223569	0.402522354	0.840260338	0.400762434	0.999978546
2.7.1.59	N-acetylglucosamine kinase	0.694599604	-0.318291685	4.094091977	-0.077744146	0.938031577	0.999978546
2.5.1.114	tRNA wybutosine-synthesizing protein 2	2.024429067	1.402991895	4.04939222	0.346469746	0.728989717	0.999978546
2.4.1.336	1,2-diacylglycerol 3-beta-glucosyltransferase	0.462092179	1.698894334	4.095415895	0.41482828	0.678267615	0.999978546
1.3.1.-	#N/A	0.739864946	-1.146900493	4.1281521	-0.277824185	0.781147325	0.999978546
3.4.21.83	oligopeptidase B	1.186270295	-1.935331327	3.812185885	-0.507669716	0.61168499	0.999978546
2.7.3.-	#N/A	31.79458853	-0.469414307	0.499176261	-0.940377867	0.347023771	0.999978546
3.5.1.49	formamidase	2.54817764	-2.658533115	2.116868441	-1.255880178	0.209159416	0.999978546
2.7.7.53	sulfate adenylyltransferase (ADP) / ATP adenylyltransferase	5.267729909	-0.603141577	1.809729188	-0.333277256	0.738925006	0.999978546
1.2.1.76	malonyl-CoA/succinyl-CoA reductase (NADPH)	26.9509768	0.57689918	0.487682085	1.182941094	0.236832494	0.999978546
4.3.2.3	ureidoglycolate lyase	16.18073341	0.790885871	0.69390581	1.13975969	0.254386432	0.999978546
3.1.1.5	lysophospholipase	19.07257356	-1.172745886	0.63141728	-1.857323077	0.063265203	0.999978546
1.1.1.304	meso-butanediol dehydrogenase / (S,S)-butanediol dehydroger	3.716547359	1.23633971	1.308645378	0.944747699	0.344787699	0.999978546
2.3.1.81	aminoglycoside 3-N-acetyltransferase	36.3516502	-0.438332392	0.505477458	-0.867165064	0.385851575	0.999978546
5.3.1.12	glucuronate isomerase	483.7936617	0.330146476	0.31199708	1.058171686	0.289977177	0.999978546
2.1.1.61	tRNA 5-methylaminomethyl-2-thiouridine biosynthesis bifuncti	0.725194853	1.418482947	4.103553939	0.345671817	0.729589367	0.999978546
3.6.1.63	alpha-D-ribose 1-methylphosphonate 5-triphosphate diphospha	2.508459474	-0.956807416	2.506422525	-0.381742267	0.702652546	0.999978546
2.7.11.2	pyruvate dehydrogenase kinase 2/3/4	0.160692243	0.094410072	4.143731151	0.022783831	0.981822705	0.999978546
3.1.11.-	#N/A	0.288921303	-1.341663909	4.128085902	-0.325008719	0.745174473	0.999978546
2.1.1.249	dimethylamine---corrinoid protein Co-methyltransferase	0.443021872	2.760692145	4.073880158	0.67765669	0.497989393	0.999978546
2.3.-.-	#N/A	4.39435923	-0.638426783	4.085732706	-0.156257599	0.875829977	0.999978546
2.5.1.18	glutathione S-transferase	2.922263646	0.069273335	1.922739913	0.036028448	0.971259675	0.999978546
1.3.1.22	steroid 5-alpha-reductase	34.49108498	0.060861343	0.498048464	0.122199641	0.902740911	0.999978546
3.1.2.-	#N/A	237.5533798	-0.38856019	0.198535432	-1.957132724	0.050331866	0.999978546
4.2.1.8	mannonate dehydratase	345.6022606	0.497912767	0.271872472	1.83142031	0.067037832	0.999978546
4.1.1.94	ethylmalonyl-CoA/methylmalonyl-CoA decarboxylase	0.267820405	0.094407394	4.143731151	0.022783185	0.981823221	0.999978546
2.4.1.141	beta-1,4-N-acetylglucosaminyltransferase	0.053564081	0.094415333	4.143731151	0.022785101	0.981821693	0.999978546
3.4.11.2	aminopeptidase N	57.71238724	-0.919979963	0.431260997	-2.133232475	0.03290566	0.999978546
3.5.3.26	(S)-ureidoglycine aminohydrolase	1.594595982	-0.630997956	1.896581697	-0.332702755	0.739358669	0.999978546
4.2.2.6	oligogalacturonide lyase	13.30351774	-0.34183378	0.698519982	-0.489368649	0.624580727	0.999978546
2.7.1.162	N-acetylhexosamine 1-kinase	5.363247739	0.409447298	1.289328397	0.317566338	0.750813911	0.999978546
1.1.1.3	homoserine dehydrogenase	423.4702407	0.024439559	0.167909695	0.145551801	0.88427522	0.999978546
1.13.11.-	#N/A	1.263813578	-2.667251155	4.089110865	-0.652281448	0.514219626	0.999978546
3.4.14.12	Xaa-Xaa-Pro tripeptidyl-peptidase	0.42378319	0.433671025	4.109541689	0.105527832	0.915956987	0.999978546
3.6.3.1	#N/A	0.057984819	-0.266239077	4.143731151	-0.06425105	0.948770329	0.999978546
2.6.1.-	#N/A	415.5908721	0.29225585	0.182500786	1.601395022	0.109289455	0.999978546
5.1.3.31	D-psicose/D-tagatose/L-ribulose 3-epimerase	6.991926601	-1.091871458	1.002069612	-1.089616376	0.275882166	0.999978546
3.2.1.141	maltooligosyltrehalose trehalohydrolase	0.197327189	0.102018647	4.143010963	0.024624276	0.980354655	0.999978546
1.2.7.8	indolepyruvate ferredoxin oxidoreductase, alpha subunit	497.2965975	0.04148388	0.276007185	0.150299999	0.880527934	0.999978546
5.3.3.14	3-hydroxyacyl-[acyl-carrier protein] dehydratase / trans-2-dece	1.695466807	-1.671845444	2.427266445	-0.688777059	0.490963574	0.999978546
3.1.3.70	mannosyl-3-phosphoglycerate phosphatase	0.043850487	0.094417027	4.143731151	0.02278551	0.981821367	0.999978546
2.4.1.292	GalNAc-alpha-(1->4)-GalNAc-alpha-(1->3)-diNAcBac-PP-undeca	0.092883485	-0.253805442	4.12245166	-0.061566627	0.950907955	0.999978546
2.7.4.8	guanylate kinase	389.5186938	0.312725635	0.292152601	1.070418796	0.284430843	0.999978546
3.5.1.108	UDP-3-O-[3-hydroxymyristoyl] N-acetylglucosamine deacetylas	302.2410722	-1.14521001	0.76370966	-1.499535844	0.133734677	0.999978546
1.97.1.9	Tat-targeted selenate reductase subunit YnfE	151.6737258	-0.221121226	0.317361209	-0.696749381	0.485959649	0.999978546
2.7.1.202	PTS system, fructose-specific IIA component	331.5898364	-0.021299047	0.232104148	-0.091765042	0.926884719	0.999978546
2.7.7.7	#N/A	5149.647638	0.337458602	0.188027295	1.794731992	0.072696411	0.999978546
2.3.1.101	formylmethanofuran--tetrahydromethanopterin N-formyltrans	8.959349101	1.788391046	1.639257493	1.090976283	0.275283319	0.999978546
2.5.1.16	spermidine synthase	117.6111361	-0.220502246	0.384771364	-0.573073431	0.566594976	0.999978546
3.6.3.31	polyamine-transporting ATPase	253.4407985	-0.201928518	0.415040479	-0.486527287	0.626593364	0.999978546
2.7.1.215	erythritol kinase (D-erythritol 1-phosphate-forming)	0.067292982	-0.682221722	4.139384262	-0.164812368	0.869091669	0.999978546
1.1.5.2	quinoprotein glucose dehydrogenase	1.663608877	-3.858354872	3.789925603	-1.018055571	0.308651548	0.999978546
2.5.1.76	cysteate synthase	7.56528018	-0.271521039	0.980426249	-0.27694183	0.781824773	0.999978546
3.2.1.78	mannan endo-1,4-beta-mannosidase	336.8419907	-0.961224681	0.396711122	-2.422983949	0.015393607	0.999978546
3.8.1.2	2-haloacid dehalogenase	83.07066021	0.262712837	0.42119853	0.623726862	0.532806979	0.999978546
2.1.1.186	23S rRNA (cytidine2498-2'-O)-methyltransferase	2.278291717	0.98369338	1.788013455	0.550159943	0.582209676	0.999978546
3.6.3.33	vitamin B12 transport system ATP-binding protein	0.098344608	-0.648021468	4.142762687	-0.156422541	0.87569997	0.999978546
1.3.1.104	enoyl-[acyl-carrier protein] reductase III	0.334667623	-1.493796544	4.128881564	-0.361792055	0.71750743	0.999978546
1.1.1.140	sorbitol-6-phosphate 2-dehydrogenase	20.25945935	0.091426401	0.74950172	0.12198291	0.902912553	0.999978546
3.4.21.19	glutamyl endopeptidase	25.77571237	0.004435145	0.654587552	0.006775481	0.99459399	0.999978546
5.4.99.17	squalene-hopene/tetraprenyl-beta-curcumene cyclase	6.587769184	-0.91600028	1.466517989	-0.624608963	0.532227736	0.999978546
4.1.1.51	3-hydroxy-2-methylpyridine-4,5-dicarboxylate 4-decarboxylase	0.02470684	0.094418984	4.143731151	0.022785982	0.98182099	0.999978546
2.8.1.8	lipoyl synthase	68.84685767	0.057458108	0.355868526	0.161458808	0.871732052	0.999978546
2.7.1.29	triose/dihydroxyacetone kinase / FAD-AMP lyase (cyclizing)	5.825853036	3.040228769	4.021328188	0.756026026	0.449633592	0.999978546
3.1.2.23	4-hydroxybenzoyl-CoA thioesterase	2.101663812	-0.210964205	2.148497831	-0.098191491	0.921780239	0.999978546
3.2.1.180	unsaturated chondroitin disaccharide hydrolase	78.8314747	-0.074825587	0.463334599	-0.161493632	0.871704626	0.999978546
3.4.21.66	thermitase	0.052431119	-0.554254637	4.139270063	-0.13390154	0.893480432	0.999978546
2.7.1.74	deoxycitidine kinase	0.485024588	0.81742177	4.118343895	0.198483126	0.842667086	0.999978546
2.3.1.180	3-oxoacyl-[acyl-carrier-protein] synthase III	375.8443716	-0.050964153	0.199869651	-0.254986949	0.798733179	0.999978546
6.3.5.10	adenosylcobyric acid synthase	242.6327014	0.322277249	0.262450479	1.227954511	0.219464044	0.999978546
1.3.1.76	uroporphyrin-III C-methyltransferase / precorrin-2 dehydrogena	35.78655682	0.22537422	0.543505952	0.414667437	0.678385374	0.999978546

3.1.3.46	6-phosphofructo-2-kinase / fructose-2,6-biphosphatase 3	0.160692243	0.094410072	4.143731151	0.022783831	0.981822705	0.999978546
4.1.1.8	oxalyl-CoA decarboxylase	5.420868125	0.819849319	1.155735137	0.709374746	0.478091955	0.999978546
2.1.1.198	16S rRNA (cytidine1402-2'-O)-methyltransferase	509.3553814	-0.04097607	0.169294055	-0.242040809	0.808748542	0.999978546
3.2.1.17	lysozyme	43.33186982	-0.849187428	0.760220457	-1.117027857	0.263982415	0.999978546
3.5.3.3	creatinase	0.047497346	0.094411622	4.143731151	0.022784205	0.981822407	0.999978546
2.4.1.60	abequosyltransferase	0.322690315	0.991173427	4.119947382	0.240579147	0.809881314	0.999978546
2.8.3.12	#N/A	116.8754764	0.059175029	0.560828887	0.105513518	0.915968344	0.999978546
4.2.1.75	uroporphyrinogen-III synthase	152.2832938	-0.175494725	0.233029083	-0.753102241	0.451388478	0.999978546
2.4.2.43	4-amino-4-deoxy-L-arabinose transferase	0.449927408	-1.047241096	4.124370808	-0.253915359	0.799560948	0.999978546
3.4.24.70	oligopeptidase A	13.63582718	0.871050375	0.584888677	1.489258399	0.136419338	0.999978546
3.6.4.-	#N/A	1559.202111	0.056519613	0.198121433	0.285277632	0.775431448	0.999978546
1.18.1.1	rubredoxin---NAD+ reductase	52.66113307	-0.327250113	0.776232236	-0.421587893	0.673325845	0.999978546
1.3.1.38	mitochondrial trans-2-enoyl-CoA reductase	0.868660033	-0.085321444	4.109350655	-0.020762756	0.983434908	0.999978546
4.2.1.151	chorismate dehydratase	11.30106601	-0.483241112	0.867598208	-0.556987217	0.577536163	0.999978546
2.4.1.259	alpha-1,2-mannosyltransferase	0.214256324	0.094408575	4.143731151	0.02278347	0.981822993	0.999978546
2.5.1.78	6,7-dimethyl-8-ribityllumazine synthase	142.8426069	0.075632783	0.288441865	0.262211531	0.793158364	0.999978546
1.1.1.94	glycerol-3-phosphate dehydrogenase (NAD(P)+)	425.844855	-0.20171909	0.194347787	-1.037928411	0.299303385	0.999978546
3.5.3.8	formiminoglutamase	2.774074758	-1.249087169	1.580879246	-0.790121809	0.429456635	0.999978546
3.5.99.10	2-iminobutanoate/2-iminopropanoate deaminase	205.6363897	0.282729712	0.344825823	0.819920356	0.412261511	0.999978546
2.7.1.161	riboflavin kinase, archaea type	1.322181795	-0.006036126	4.140081342	-0.001457973	0.998836706	0.999978546
1.1.1.27	L-lactate dehydrogenase	263.1265027	0.011273458	0.294752026	0.038247263	0.969490538	0.999978546
3.1.21.2	deoxyribonuclease IV	300.1767179	0.150902364	0.254881945	0.592048072	0.553818398	0.999978546
2.8.3.5	3-oxoacid CoA-transferase	7.962912308	-0.436159203	0.954251723	-0.457069338	0.647621208	0.999978546
2.7.8.14	CDP-ribitol ribitolphosphotransferase / teichoic acid ribitol-pho	29.46695896	-0.43408744	0.521234235	-0.832806848	0.404953672	0.999978546
6.3.1.19	proteasome accessory factor A	0.403407769	-0.523786669	4.106602967	-0.127547434	0.898507133	0.999978546
3.4.17.13	muramoyltetrapeptide carboxypeptidase	30.21567958	-0.304577457	0.597575962	-0.509688268	0.610269873	0.999978546
3.4.22.70	sortase A	838.1445998	-0.021175807	0.182783925	-0.115851583	0.907770169	0.999978546
3.5.1.105	chitin disaccharide deacetylase	8.422222645	-0.916271852	1.102390184	-0.83116837	0.405878521	0.999978546
4.1.1.85	3-dehydro-L-gulonate-6-phosphate decarboxylase	0.805198269	-1.767486614	4.123574323	-0.428629746	0.668192695	0.999978546
5.1.3.24	N-acetylneuraminate epimerase	0.240767753	-1.252599762	4.129265175	-0.303346893	0.761625513	0.999978546
2.5.1.31	undecaprenyl diphosphate synthase	402.9684909	-0.09624845	0.155398551	-0.61936517	0.535675821	0.999978546
1.5.1.7	saccharopine dehydrogenase (NAD+, L-lysine forming)	270.5033177	-0.074937826	0.222256064	-0.33716887	0.735989603	0.999978546
3.4.19.1	acylaminoacyl-peptidase	1.683032235	1.189990617	1.433783516	0.829965336	0.406558383	0.999978546
3.1.3.73	alpha-ribazole phosphatase	202.4302529	0.416826156	0.301716333	1.381516713	0.167120143	0.999978546
2.1.1.166	23S rRNA (uridine2552-2'-O)-methyltransferase	12.94395647	-1.14825842	0.995217277	-1.153776614	0.248591764	0.999978546
1.6.5.2	NAD(P)H dehydrogenase (quinone)	9.363692982	-1.04878521	1.011318844	-1.037047036	0.299713937	0.999978546
3.4.21.110	C5a peptidase	0.866408451	-0.334256949	4.115896379	-0.081211216	0.93527398	0.999978546
4.7.1.1	alpha-D-ribose 1-methylphosphonate 5-phosphate C-P lyase	2.28894411	-0.833444879	2.07276296	-0.402093677	0.687615086	0.999978546
5.1.1.3	glutamate racemase	455.6094706	0.022086096	0.480005911	0.046012133	0.963300579	0.999978546
6.3.1.12	D-aspartate ligase	24.50587962	-0.442873044	0.412646941	-1.073249307	0.283159273	0.999978546
1.8.1.2	#N/A	12.775374	-0.521884501	1.126852063	-0.463134885	0.643267686	0.999978546
4.1.99.12	3,4-dihydroxy 2-butanone 4-phosphate synthase	318.5032203	0.141685918	0.225461251	0.628426915	0.52972431	0.999978546
4.1.2.14	2-dehydro-3-deoxyphosphogluconate aldolase / (4S)-4-hydroxy	365.4288842	0.171885431	0.280554607	0.612663013	0.540099183	0.999978546
5.4.99.27	tRNA pseudouridine13 synthase	2.224630463	3.307335915	2.709349501	1.220712172	0.222195017	0.999978546
1.12.99.6	#N/A	125.2879068	-0.255935251	0.509760082	-0.502070013	0.615618273	0.999978546
2.7.1.192	PTS system, N-acetylmuramic acid-specific IIB component	0.096307101	-0.778791352	4.138114321	-0.188199574	0.850720206	0.999978546
1.3.99.33	urocanate reductase	28.26826934	-1.525250039	0.923739531	-1.651168958	0.09870408	0.999978546
3.6.1.41	bis(5'-nucleosyl)-tetraphosphatase (symmetrical)	3.179764336	-0.878115858	1.83062224	-0.479681629	0.631453793	0.999978546
6.3.5.2	GMP synthase (glutamine-hydrolysing)	699.2410668	-0.091307674	0.177765055	-0.513642427	0.607502011	0.999978546
3.6.3.-	#N/A	1836.451269	0.026760462	0.238920833	0.112005562	0.910818996	0.999978546
2.3.1.76	diacylglycerol O-acyltransferase 1	0.015832449	0.094415661	4.143731151	0.02278518	0.981821629	0.999978546
3.1.6.8	arylsulfatase A	1.997616455	-4.202598719	3.496258567	-1.202027436	0.229352899	0.999978546
1.14.13.59	lysine N6-hydroxylase	0.353595073	-1.475840651	4.123325751	-0.357924826	0.720399571	0.999978546
2.7.1.170	anhydro-N-acetylmuramic acid kinase	29.26470967	-0.290628061	0.510777323	-0.568991706	0.569361769	0.999978546
2.6.1.57	aromatic-amino-acid transaminase	12.44296424	-0.931321339	0.696317547	-1.337495146	0.181061066	0.999978546
1.3.99.-	#N/A	55.60821594	-0.027784598	0.38554906	-0.072065013	0.942550169	0.999978546
2.7.8.36	undecaprenyl phosphate N,N'-diacetylbacillosamine 1-phospha	4.593317519	-1.132709343	1.679685382	-0.674358041	0.500083712	0.999978546
5.3.1.16	phosphoribosylformimino-5-aminoimidazole carboxamide ribot	177.1832862	-0.092589666	0.263288891	-0.351665677	0.725089004	0.999978546
3.1.2.6	hydroxyacylglutathione hydrolase	1.655433542	-2.358626989	2.110642136	-1.11749261	0.263783758	0.999978546
1.15.1.1	nickel superoxide dismutase	641.8686433	-0.006870235	0.543814739	-0.012633411	0.989920265	0.999978546
3.5.4.43	hydroxydechloroatrazine ethylaminohydrolase	0.97091914	-1.427011517	3.402305513	-0.419424861	0.674905658	0.999978546
2.4.99.17	S-adenosylmethionine:tRNA ribosyltransferase-isomerase	404.1357236	0.220356194	0.305176799	0.722060768	0.470257117	0.999978546
4.2.2.3	poly(beta-D-mannuronate) lyase	3.76957877	-2.072318341	1.542849758	-1.343175725	0.17921508	0.999978546
6.3.2.8	UDP-N-acetylmuramate--alanine ligase	425.8810352	-0.099396543	0.13418471	-0.740744178	0.458848567	0.999978546
2.7.1.184	sulfofructose kinase	16.85072223	1.356308827	0.852257271	1.591431219	0.111512563	0.999978546
3.5.99.6	#N/A	840.5561841	0.025251963	0.359602249	0.070221926	0.944017023	0.999978546
3.2.1.10	oligo-1,6-glucosidase	163.2185741	-0.491483803	0.279037666	-1.761352906	0.078178691	0.999978546
2.3.1.39	acyl-carrier-protein] S-malonyltransferase	252.4509801	-0.008845468	0.237663308	-0.037218485	0.9703108	0.999978546
2.4.99.12	3-deoxy-D-manno-octulosonic-acid transferase	102.8745696	-0.135612171	0.327661726	-0.413878582	0.67896303	0.999978546
3.2.1.113	mannosyl-oligosaccharide alpha-1,2-mannosidase	1.197753478	0.728155219	4.140439617	0.175864229	0.860400609	0.999978546
3.1.3.36	inositol polyphosphate 5-phosphatase INPP5B/F	0.685511993	-0.663739579	4.135195034	-0.160509861	0.872479455	0.999978546
1.13.12.-	#N/A	1.243420728	-2.043284263	3.897637045	-0.524236669	0.600113939	0.999978546
2.7.7.47	streptomycin 3''-adenylyltransferase	10.73053726	0.372799126	1.00967378	0.369227302	0.711958307	0.999978546
1.1.3.21	alpha-glycerophosphate oxidase	0.145244102	-1.284212244	4.136173644	-0.310483155	0.756193567	0.999978546
1.3.1.12	prephenate dehydrogenase	171.7607912	-0.098596935	0.29263821	-0.336924336	0.736173939	0.999978546
5.3.99.11	2-keto-myo-inositol isomerase	6.726854188	-0.477272054	0.65260738	-0.731331071	0.464576957	0.999978546
3.2.1.21	beta-glucosidase	1951.494518	-0.101117835	0.175690637	-0.575544815	0.564922891	0.999978546
1.5.5.2	RHH-type transcriptional regulator, proline utilization regulon re	58.38325883	-0.125894942	0.458954447	-0.274308143	0.783847827	0.999978546
2.1.2.9	methionyl-tRNA formyltransferase	378.080312	0.249591218	0.180949711	1.379340244	0.167789874	0.999978546
2.4.2.2	pyrimidine-nucleoside phosphorylase	232.276121	0.215721394	0.299944757	0.719203751	0.472015389	0.999978546
2.7.1.17	xylulokinase	428.2689433	-0.043760542	0.234736105	-0.186424421	0.852111943	0.999978546
2.7.7.13	mannose-1-phosphate guanylyltransferase	1345.206609	-1.2823188	0.908451135	-1.411544056	0.158084255	0.999978546
4.2.2.8	heparin/heparan-sulfate lyase	21.98860056	-1.292975782	0.789822121	-1.637046808	0.101620689	0.999978546
1.11.1.15	peroxiredoxin (alkyl hydroperoxide reductase subunit C)	879.9144678	0.406037058	0.511127504	0.794394852	0.4269656	0.999978546
3.5.4.12	dCMP deaminase	242.5971025	-0.211266464	0.204383615	-1.033676127	0.301287597	0.999978546
3.6.3.40	teichoic acid transport system ATP-binding protein	81.29592209	-0.21024431	0.371929447	-0.565280087	0.571883271	0.999978546
2.1.-.-	#N/A	0.192614202	-1.143334171	4.13091893	-0.276774778	0.781953049	0.999978546

3.1.11.5	exodeoxyribonuclease V	497.8412324	0.106894825	0.256383432	0.416933434	0.676727098	0.999978546
1.1.1.336	UDP-N-acetyl-D-mannosaminuronic acid dehydrogenase	33.03340853	-0.097327042	0.429200839	-0.226763402	0.820607724	0.999978546
1.14.13.-	#N/A	1.712504284	-1.784131318	4.11879754	-0.433168006	0.66489272	0.999978546
3.6.1.65	(d)CTP diphosphatase	0.422052821	-1.730112712	4.125225394	-0.419398347	0.674925032	0.999978546
1.2.1.81	sulfoacetaldehyde dehydrogenase	12.88492287	-1.392277483	1.254422444	-1.109895227	0.267044177	0.999978546
4.1.2.17	L-fuculose-phosphate aldolase	179.7216875	-0.091632198	0.417195747	-0.21963838	0.826152798	0.999978546
2.5.1.41	phosphoglycerol geranylgeranyltransferase	3.384201131	2.751466043	2.242037074	1.227217014	0.219741036	0.999978546
1.3.7.9	4-hydroxybenzoyl-CoA reductase subunit gamma	0.297488658	-0.617061014	4.123152101	-0.149657592	0.881034768	0.999978546
5.1.99.3	allantoin racemase	7.808605122	-0.248674341	0.692333823	-0.359182713	0.719458408	0.999978546
2.7.8.23	carboxyvinyl-carboxyphosphonate phosphorylmutase	0.047497346	0.094411622	4.143731151	0.022784205	0.981822407	0.999978546
1.1.1.205	IMP dehydrogenase	738.73706	-0.435806859	0.4291757	-1.01545092	0.309890931	0.999978546
2.4.1.325	dTDP-N-acetylfucosamine:lipid II N-acetylfucosaminyltransferas	0.215045196	-1.198643306	4.130051195	-0.290224806	0.77164426	0.999978546
2.1.3.11	N-succinyl-L-ornithine transcarbamylase	68.30817693	0.000396441	0.404295428	0.000980572	0.999217617	0.999978546
3.5.4.9	methylenetetrahydrofolate dehydrogenase (NADP+) / metheny	432.3435048	-0.07651126	0.235471467	-0.324927945	0.745235607	0.999978546
1.1.1.384	dTDP-3,4-didehydro-2,6-dideoxy-alpha-D-glucose 3-reductase	3.667088234	-0.830413456	1.218999088	-0.681225658	0.495728715	0.999978546
2.1.1.203	tRNA (cytosine34-C5)-methyltransferase	0.433524036	-0.659239989	4.134848861	-0.159435087	0.873326097	0.999978546
2.3.2.10	serine/alanine adding enzyme	1.782642193	-1.487718012	2.297288526	-0.647597372	0.517245397	0.999978546
4.1.2.50	6-pyruvoyltetrahydropterin/6-carboxytetrahydropterin synthas	76.58751425	-0.18972892	0.376555652	-0.503853598	0.61436426	0.999978546
2.7.1.49	hydroxymethylpyrimidine/phosphomethylpyrimidine kinase / t	174.8667027	-0.409461757	0.277564773	-1.475193527	0.140160521	0.999978546
3.6.-.-	#N/A	322.2767022	-0.069199346	0.242259523	-0.285641386	0.775152802	0.999978546
6.3.1.1	aspartate--ammonia ligase	184.1027838	-0.037956372	0.295327687	-0.128522904	0.897735174	0.999978546
2.1.1.298	ribosomal protein L3 glutamine methyltransferase	3.233325143	1.904057093	1.226035576	1.553019448	0.120418488	0.999978546
1.7.5.1	nitrate reductase / nitrite oxidoreductase, alpha subunit	5.245192299	-1.701784379	0.923779473	-1.842197656	0.065446243	0.999978546
1.1.1.14	L-iditol 2-dehydrogenase	109.28552	-0.058890684	0.345409015	-0.170495503	0.86462047	0.999978546
1.3.1.89	tRNA-dihydrouridine synthase 3	0.06939653	0.094411243	4.143731151	0.022784114	0.98182248	0.999978546
1.13.11.2	catechol 2,3-dioxygenase	0.859051352	-1.833751789	4.110177238	-0.446149079	0.655489563	0.999978546
4.3.1.2	methylaspartate ammonia-lyase	48.64895908	0.550075097	0.578443349	0.950957596	0.341625902	0.999978546
3.4.11.7	glutamyl aminopeptidase	1.448565954	0.122976827	3.095389948	0.039729026	0.968309161	0.999978546
3.5.1.98	histone deacetylase 1/2	0.803461216	0.094401337	4.143731151	0.022781724	0.981824387	0.999978546
5.1.3.6	UDP-glucuronate 4-epimerase	31.56312131	-0.820090367	0.631301731	-1.299046599	0.193927938	0.999978546
3.1.26.12	ribonuclease E	25.32080082	-1.59382942	0.672648442	-2.369483551	0.017812947	0.999978546
5.4.99.58	methylornithine synthase	0.369327113	-1.745413126	4.125902502	-0.423037899	0.67226761	0.999978546
2.7.1.26	riboflavin kinase	322.5539954	0.194157323	0.219959813	0.882694528	0.37740134	0.999978546
3.2.1.33	glycogen debranching enzyme	0.160692243	0.094410072	4.143731151	0.022783831	0.981822705	0.999978546
3.2.1.14	chitinase	63.36207878	0.553834347	0.337142327	1.642731576	0.100438483	0.999978546
1.1.3.-	#N/A	0.215045196	-1.198643306	4.130051195	-0.290224806	0.77164426	0.999978546
3.5.1.91	N-substituted formamide deformylase	117.1158786	0.176872028	0.438330133	0.403513275	0.686570671	0.999978546
3.1.1.3	triacylglycerol lipase	15.45567411	0.180880287	0.727444012	0.248651833	0.80363011	0.999978546
3.5.1.32	hippurate hydrolase	17.82442116	-0.045469084	0.68293101	-0.066579322	0.946916607	0.999978546
3.5.1.9	arylformamidase	22.52921188	-1.164296649	0.811067157	-1.435512015	0.151141247	0.999978546
3.1.13.4	poly(A)-specific ribonuclease	0.107128162	0.094412122	4.143731151	0.022784326	0.981822311	0.999978546
1.3.99.29	phytoene desaturase	1.632348624	0.91736795	4.096641466	0.223931715	0.822810437	0.999978546
3.5.2.18	enamidase	9.33551453	-0.848711719	1.305519265	-0.650095132	0.515630773	0.999978546
2.7.1.55	allose kinase	2.123922768	-0.529748826	1.816212073	-0.291677846	0.770532949	0.999978546
4.1.1.19	arginine decarboxylase	193.6903894	-0.384670773	0.347996252	-1.10538769	0.268991636	0.999978546
4.1.2.40	tagatose 1,6-diphosphate aldolase	44.01710737	-0.392390206	0.451998448	-0.868122907	0.385327052	0.999978546
2.1.1.64	2-polyprenyl-6-hydroxyphenyl methylase / 3-demethylubiquinc	0.275721832	-1.704528853	4.123016056	-0.413417952	0.679300424	0.999978546
2.3.1.61	2-oxoglutarate dehydrogenase E2 component (dihydrolipoamid	48.87361501	-0.243367084	0.517198605	-0.470548608	0.637963114	0.999978546
6.1.1.5	isoleucyl-tRNA synthetase	902.9590698	0.006579139	0.108402632	0.060691693	0.951604748	0.999978546
3.1.1.17	gluconolactonase	1.652406123	0.678192404	3.437383911	0.197298999	0.843593563	0.999978546
4.1.2.43	3-hexulose-6-phosphate synthase	16.96607242	-0.098293944	0.783754613	-0.125414182	0.900195661	0.999978546
4.2.1.80	2-keto-4-pentenoate hydratase	0.627092025	-2.785093788	4.106895443	-0.678150644	0.497676183	0.999978546
2.1.1.245	acetyl-CoA decarbonylase/synthase complex subunit delta	937.0964197	-0.144591115	0.668178734	-0.216395865	0.828679189	0.999978546
3.5.3.11	agmatinase	88.61984129	0.061054929	0.327683971	0.186322599	0.852191787	0.999978546
6.6.1.2	cobaltochelatase CobN	201.6340406	0.586708703	0.42197406	1.39039045	0.164410345	0.999978546
3.5.-.-	#N/A	18.06841165	1.105337818	0.662608293	1.668161764	0.095283619	0.999978546
3.7.1.20	fumarylpyruvate hydrolase	3.078512959	-2.069785043	1.928950412	-1.073011017	0.283266173	0.999978546
2.7.8.28	LPPG:FO 2-phospho-L-lactate transferase	4.388340055	4.642665447	4.004503359	1.159361107	0.246309023	0.999978546
1.1.1.85	3-isopropylmalate dehydrogenase	273.3671789	-0.07728982	0.195505828	-0.395332564	0.69259747	0.999978546
3.2.1.122	maltose-6'-phosphate glucosidase	25.24441003	0.173089523	0.624990999	0.276947225	0.78182063	0.999978546
2.1.2.3	phosphoribosylaminoimidazolecarboxamide formyltransferase	631.120063	-0.090957186	0.214561584	-0.423921113	0.671623346	0.999978546
2.7.-.-	#N/A	135.2697332	-0.663773334	0.4497843	-1.475759233	0.140008538	0.999978546
3.2.1.89	arabinogalactan endo-1,4-beta-galactosidase	114.6560584	-0.56618869	0.429799209	-1.31733302	0.187727022	0.999978546
2.8.3.8	#N/A	156.8950941	0.149232828	0.591487939	0.252300711	0.800808638	0.999978546
2.1.1.271	precorrin-4/cobalt-precorrin-4 C11-methyltransferase	110.7485461	0.037708156	0.349953931	0.107751772	0.9141926	0.999978546
1.8.4.-	#N/A	1.874139009	-1.757759855	2.195660392	-0.800560898	0.423385895	0.999978546
2.4.2.9	uracil phosphoribosyltransferase	463.3200667	0.13553584	0.229102912	0.591593702	0.554122692	0.999978546
3.4.-.-	#N/A	2017.633239	0.08128893	0.122292472	0.664709194	0.506236512	0.999978546
4.1.1.44	4-carboxymuconolactone decarboxylase	3240.638862	-1.611389304	1.470319857	-1.095944733	0.273102961	0.999978546
3.1.1.73	feruloyl esterase	0.400658548	-2.00703752	4.094971117	-0.49012251	0.624047211	0.999978546
1.14.11.17	taurine dioxygenase	0.127261092	-0.32570528	4.117646039	-0.079099873	0.936953184	0.999978546
2.1.1.130	#N/A	67.37692647	0.322052295	0.516037335	0.624087199	0.53257032	0.999978546
4.1.1.12	aspartate 4-decarboxylase	35.20555678	-0.749432005	0.522048188	-1.435560975	0.151127306	0.999978546
1.14.17.3	peptidylglycine monooxygenase	0.104862237	-0.808519133	4.137490949	-0.195412907	0.845069713	0.999978546
1.7.2.3	trimethylamine-N-oxide reductase (cytochrome c)	14.90176483	0.368395905	0.995349439	0.370117158	0.711295199	0.999978546
2.10.1.1	molybdopterin molybdotransferase	124.0538972	0.011287203	0.42634809	0.026474149	0.978879152	0.999978546
1.4.3.-	#N/A	1.495574844	1.11701135	3.441440534	0.324576682	0.745501478	0.999978546
2.5.1.19	3-phosphoshikimate 1-carboxyvinyltransferase	330.9966198	-0.112678805	0.276711573	-0.407206696	0.683856184	0.999978546
2.1.3.2	aspartate carbamoyltransferase	339.3596569	0.085418838	0.205238111	0.416193844	0.677268164	0.999978546
3.6.5.-	#N/A	484.9955167	-0.058574552	0.192969737	-0.303542684	0.761476323	0.999978546
3.5.1.78	glutathionylspermidine amidase/synthetase	1.535270693	-0.151221836	3.067037398	-0.049305508	0.96067583	0.999978546
3.6.3.28	#N/A	16.58175273	-1.017060246	0.832969088	-1.22100599	0.222083752	0.999978546
2.1.3.1	methylmalonyl-CoA carboxyltransferase 5S subunit	0.363147851	2.899034752	4.116157834	0.704306022	0.481242217	0.999978546
3.5.1.4	amidase	13.39788218	-0.59827694	0.863098662	-0.693173291	0.488200808	0.999978546
4.3.1.24	phenylalanine ammonia-lyase	0.052431119	-0.554254637	4.139270063	-0.13390154	0.893480432	0.999978546
6.3.5.1	NAD+ synthase (glutamine-hydrolysing)	1103.455858	-1.113513079	0.625736566	-1.779523748	0.075153935	0.999978546
6.2.1.13	acetate---CoA ligase (ADP-forming) subunit alpha	38.89400474	0.510933232	0.869068096	0.587909318	0.556593162	0.999978546

1.2.1.27	malonate-semialdehyde dehydrogenase (acetylating) / methylr	18.30084634	0.430946664	0.485504764	0.887626025	0.37474195	0.999978546
2.3.3.9	malate synthase	4.623532929	-3.125549285	2.06596397	-1.512876957	0.130310916	0.999978546
1.5.99.15	dihydromethanopterin reductase (acceptor)	3.714982287	2.311647029	2.584555773	0.894407872	0.371103702	0.999978546
3.1.3.12	trehalose 6-phosphate phosphatase	28.07036042	-0.337148681	0.460906185	-0.731490902	0.46447936	0.999978546
5.3.1.30	5-deoxy-glucuronate isomerase	37.58263163	0.869018565	0.513531284	1.692240752	0.090600079	0.999978546
2.1.1.178	16S rRNA (cytosine1407-C5)-methyltransferase	0.518083772	0.430153546	4.110797566	0.104639924	0.916661534	0.999978546
2.7.2.7	butyrate kinase	172.3403853	0.032808161	0.347279474	0.094471926	0.924734282	0.999978546
3.2.1.133	cyclomaltodextrinase / maltogenic alpha-amylase / neopullulan	0.261732976	-0.331874779	4.114293375	-0.080663859	0.93570928	0.999978546
1.5.3.22	coenzyme F420H2 oxidase	43.21632078	2.370428862	1.018222733	2.328006226	0.019911773	0.999978546
1.17.4.2	ribonucleoside-triphosphate reductase (thioredoxin)	1317.312582	0.227671139	0.179625957	1.267473488	0.204986038	0.999978546
2.7.2.11	glutamate 5-kinase	251.4337639	0.004845045	0.223092262	0.021717674	0.982673165	0.999978546
2.7.8.8	CDP-diacylglycerol---serine O-phosphatidyltransferase	109.1044487	-0.465610877	0.416985368	-1.116612025	0.264160248	0.999978546
2.7.1.138	ceramide kinase	0.022430994	-0.266241886	4.143731151	-0.064251728	0.94876979	0.999978546
6.1.1.7	alanyl-tRNA synthetase	1078.886596	0.015990963	0.2003766	0.079804543	0.936392711	0.999978546
2.6.1.52	phosphoserine aminotransferase	504.7947067	0.01291242	0.231206311	0.055848044	0.955462861	0.999978546
4.99.1.3	sirohydrochlorin cobaltochelatase	136.2095742	0.194825633	0.322159216	0.604749524	0.545345437	0.999978546
3.1.1.61	two-component system, chemotaxis family, protein-glutamate	143.0684675	-0.085709119	0.531740412	-0.161186017	0.871946894	0.999978546
2.5.1.90	octaprenyl-diphosphate synthase	81.05412723	-0.788429225	0.438054784	-1.799841601	0.071885653	0.999978546
3.5.1.16	acetylornithine deacetylase	66.04934801	-0.400045946	0.283369183	-1.411748242	0.158024103	0.999978546
1.1.1.202	1,3-propanediol dehydrogenase	0.419812109	-1.084384315	4.133539291	-0.262337972	0.793060889	0.999978546
1.20.1.1	phosphonate dehydrogenase	0.021925243	0.094418168	4.143731151	0.022785785	0.981821147	0.999978546
2.3.1.234	N6-L-threonylcarbamoyladenine synthase	411.5437197	0.06711404	0.19844945	0.338192118	0.735218415	0.999978546
4.1.99.22	GTP 3',8-cyclase	176.3824341	1.103853511	0.469454799	2.351352066	0.018705326	0.999978546
1.4.1.3	glutamate dehydrogenase (NAD(P)+)	8.230057854	-1.220672292	1.027390167	-1.188129234	0.234782498	0.999978546
2.6.1.97	archaeosine synthase	3.338260327	2.546500518	2.550419594	0.99846336	0.318054723	0.999978546
2.1.1.193	16S rRNA (uracil1498-N3)-methyltransferase	240.5805697	0.080211066	0.237808995	0.337291976	0.735896808	0.999978546
1.8.7.1	sulfite reductase (ferredoxin)	0.015832449	0.094415661	4.143731151	0.02278518	0.981821629	0.999978546
3.4.22.1	cathepsin B	8.888996236	-0.598314527	3.61563276	-0.165479894	0.868566274	0.999978546
4.2.1.20	tryptophan synthase	461.3372891	-0.2127815	0.277711314	-0.766196727	0.443559256	0.999978546
1.1.1.396	bacteriochlorophyllide a dehydrogenase	0.057984819	-0.266239077	4.143731151	-0.06425105	0.948770329	0.999978546
1.1.2.3	L-lactate dehydrogenase (cytochrome)	0.301644209	-1.170562263	4.131488152	-0.283327029	0.77692616	0.999978546
2.7.11.17	calcium/calmodulin-dependent protein kinase kinase 1	0.589204892	0.094403102	4.143731151	0.022782149	0.981824047	0.999978546
2.3.1.210	dTDP-4-amino-4,6-dideoxy-D-galactose acyltransferase	0.095650897	-0.36177611	4.140215162	-0.087380993	0.930368677	0.999978546
2.1.1.221	tRNA (guanine9-N1)-methyltransferase	0.160692243	0.094410072	4.143731151	0.022783831	0.981822705	0.999978546
2.3.1.202	UDP-4-amino-4,6-dideoxy-N-acetyl-beta-L-altrosamine N-acety	0.222361558	0.094413407	4.143731151	0.022784636	0.981822063	0.999978546
3.4.13.-	#N/A	532.3021501	-0.172295525	0.181573766	-0.948900981	0.342670975	0.999978546
3.4.24.64	mitochondrial-processing peptidase subunit alpha	0.167740794	2.062895766	4.106887283	0.50230153	0.615455433	0.999978546
2.7.7.73	sulfur carrier protein ThiS adenylyltransferase	10.45724072	-0.401646965	0.941837231	-0.426450508	0.669779605	0.999978546
2.8.1.1	#N/A	6.634840969	-1.15874106	0.929406181	-1.246754201	0.212487638	0.999978546
3.4.21.107	serine protease Do	563.0586176	-0.120932885	0.410588494	-0.294535494	0.768348746	0.999978546
3.6.3.6	#N/A	2.40170059	-3.65429363	2.783066642	-1.313045679	0.189167556	0.999978546
3.4.17.19	carboxypeptidase Taq	88.78305047	0.307715769	0.372047907	0.827086412	0.408188085	0.999978546
3.1.26.4	ribonuclease HI	787.2753457	-0.199709817	0.504752742	-0.395658707	0.692356822	0.999978546
2.1.1.13	5-methyltetrahydrofolate--homocysteine methyltransferase	505.0696423	-0.118993932	0.244959895	-0.485769033	0.627130932	0.999978546
1.1.1.103	threonine 3-dehydrogenase	187.2651899	0.518166713	0.675845358	0.766694195	0.443263356	0.999978546
1.8.4.2	protein-disulfide reductase (glutathione)	1.392666108	0.086477484	4.143360556	0.020871339	0.983348289	0.999978546
2.1.1.79	cyclopropane-fatty-acyl-phospholipid synthase	13.91181454	-0.410209172	0.727945747	-0.563516132	0.573083478	0.999978546
2.5.1.131	(4-(4-[2-(gamma-L-glutamylamino)ethyl]phenoxymethyl)furan-:	2.497929715	2.395823495	2.324219574	1.030807726	0.302630994	0.999978546
1.2.7.1	#N/A	6286.64664	0.213613701	0.507696311	0.420750942	0.673936958	0.999978546
2.1.1.248	methylamine---corrinoid protein Co-methyltransferase	3.097301404	-0.684766945	2.149926986	-0.31850707	0.750100333	0.999978546
2.7.13.3	two-component system, sensor histidine kinase PdtaS	2106.00677	0.025711843	0.127698763	0.201347628	0.840426763	0.999978546
2.8.3.19	CoA:oxalate CoA-transferase	4.085827508	0.222623379	1.157007526	0.192413078	0.847418649	0.999978546
2.7.1.207	PTS system, lactose-specific IIA component	3.660562079	0.377220534	1.410789333	0.26738261	0.789174587	0.999978546
2.7.1.2	glucokinase	766.2669656	-0.063016529	0.227455695	-0.277049686	0.781741955	0.999978546
2.7.7.58	#N/A	1.219326878	-2.26146237	3.185072443	-0.710019132	0.477692272	0.999978546
6.3.2.2	glutamate--cysteine ligase	16.25644899	-1.306819637	0.633783069	-2.061935229	0.039213903	0.999978546
1.4.1.11	L-erythro-3,5-diaminohexanoate dehydrogenase	75.26061903	-0.255637436	0.542321897	-0.471375834	0.63737237	0.999978546
6.3.4.5	argininosuccinate synthase	353.5266071	0.267427765	0.231597509	1.154709161	0.248209547	0.999978546
3.5.1.118	gamma-glutamyl hercynylcysteine S-oxide hydrolase	0.788341192	1.413269987	3.588280671	0.393857147	0.69368651	0.999978546
1.5.-.-	#N/A	9.289395333	0.454868385	0.642439894	0.708032595	0.478925016	0.999978546
3.4.19.13	gamma-glutamyltranspeptidase / glutathione hydrolase	29.91949374	1.046816726	0.553318418	1.891888452	0.058505845	0.999978546
2.7.7.43	N-acylneuraminate cytidylyltransferase	24.55902711	0.027639887	0.539864661	0.051197808	0.959167898	0.999978546
2.7.1.67	phosphatidylinositol 4-kinase A	0.208380513	0.419068997	4.142174626	0.101171253	0.919414516	0.999978546
1.1.1.35	3-hydroxyacyl-CoA dehydrogenase	2.996316731	-0.903727667	1.313394734	-0.688085344	0.49139904	0.999978546
2.8.4.4	ribosomal protein S12 methylthiotransferase	617.9371104	0.309499862	0.200847676	1.540968105	0.123324549	0.999978546
1.1.1.58	tagaturonate reductase	366.6022532	0.673596699	0.42988309	1.566929975	0.11713106	0.999978546
2.2.1.1	transketolase	1674.932167	0.100242413	0.276261552	0.36285329	0.716714481	0.999978546
2.6.1.76	diaminobutyrate-2-oxoglutarate transaminase	23.46744053	0.619748927	1.044018707	0.593618603	0.552767235	0.999978546
3.4.24.40	serralysin	0.216806498	0.094405595	4.143731151	0.022782751	0.981823567	0.999978546
2.8.3.16	formyl-CoA transferase	5.93634884	0.651524491	1.107527219	0.588269507	0.556351409	0.999978546
3.1.3.97	3',5'-nucleoside bisphosphate phosphatase	375.6107677	0.279238223	0.183682718	1.520220448	0.12845558	0.999978546
4.2.1.90	L-rhamnonate dehydratase	0.858938804	-0.922597066	4.100206109	-0.225012363	0.821969656	0.999978546
3.6.1.13	ADP-ribose pyrophosphatase	194.8902596	-0.353726786	0.251535257	-1.40627119	0.159643612	0.999978546
1.1.-.-	#N/A	83.0482385	-0.300163864	0.287918623	-1.042530216	0.297165924	0.999978546
3.1.1.11	pectinesterase	182.2115839	0.64695768	0.399591063	1.619049426	0.105436629	0.999978546
4.1.1.81	threonine-phosphate decarboxylase	126.0137083	0.142920378	0.295014772	0.484451599	0.628065406	0.999978546
3.6.3.54	copper-transporting P-type ATPase V	207.7805605	0.361433337	0.343622837	1.051831539	0.292876846	0.999978546
3.5.4.28	5-methylthioadenosine/S-adenosylhomocysteine deaminase	210.6530955	0.109314853	0.482982683	0.226332861	0.820942542	0.999978546
1.6.5.8	#N/A	454.6626916	-0.150523931	0.310834866	-0.484256907	0.628203554	0.999978546
5.3.2.6	4-oxalocrotonate tautomerase	11.37995625	-0.597087703	0.850142585	-0.702338306	0.48246821	0.999978546
1.2.4.1	pyruvate dehydrogenase (acetyl-transferring)	62.46924544	-0.729538699	0.689772736	-1.057650818	0.290214665	0.999978546
2.7.1.86	NADH kinase	0.160692243	0.094410072	4.143731151	0.022783831	0.981822705	0.999978546
5.1.99.8	7,8-dihydroneopterin aldolase/epimerase/oxygenase	48.1069637	-0.916774564	0.405133503	-2.262894965	0.023642165	0.999978546
1.1.1.37	malate dehydrogenase	317.6601585	-0.508510802	0.397664283	-1.278743965	0.200987232	0.999978546
1.13.99.1	inositol oxygenase	0.504441937	1.765208871	4.104990576	0.430015328	0.667184491	0.999978546
2.7.1.197	PTS system, mannitol-specific IIA component	45.44114152	-0.080627539	0.469569621	-0.171705186	0.863669309	0.999978546
3.6.1.7	acylphosphatase	87.39297953	-0.176294831	0.334064302	-0.527727237	0.597688666	0.999978546

1.5.1.33	pteridine reductase	0.26805721	-1.835323617	4.123459543	-0.445093155	0.656252435	0.999978546
2.1.1.220	tRNA (adenine57-N1/adenine58-N1)-methyltransferase catalyti	4.213074941	1.914644368	1.706178205	1.122183112	0.261784592	0.999978546
3.4.22.48	staphopain A	0.269151628	0.028259706	4.141099152	0.006824204	0.994555115	0.999978546
2.7.4.22	uridylate kinase	553.4700535	-0.173186455	0.296048052	-0.584994407	0.558551477	0.999978546
1.1.1.22	UDPglucose 6-dehydrogenase	338.3428138	-5.81E-05	0.210288032	-0.000276342	0.999779511	0.999978546
4.2.1.10	#N/A	224.1164232	0.060147602	0.250927377	0.239701233	0.810561879	0.999978546
3.4.19.-	#N/A	67.95115075	0.125393538	0.316488009	0.396203124	0.691955187	0.999978546
3.2.1.184	GDP/UDP-N,N'-diacetylbacillosamine 2-epimerase (hydrolysing)	11.03001939	-0.021871946	0.668654849	-0.032710368	0.973905556	0.999978546
3.4.22.37	gingipain R	506.8117507	3.313746611	1.336876145	2.478723721	0.013185339	0.999978546
6.3.5.6	aspartyl-tRNA(Asn)/glutamyl-tRNA(Gln) amidotransferase subu	502.939812	-0.083215886	0.284036897	-0.292975619	0.769540788	0.999978546
4.1.1.4	acetoacetate decarboxylase	0.721647054	-1.462301301	4.114317683	-0.355417694	0.722276696	0.999978546
4.6.1.13	1-phosphatidylinositol phosphodiesterase	8.638312496	-0.389097264	0.81838451	-0.475445538	0.634469443	0.999978546
3.4.11.6	aminopeptidase B	5.455731727	1.449920208	1.306631937	1.10889698	0.267474627	0.999978546
3.2.2.21	DNA-3-methyladenine glycosylase II	48.22412736	-0.283310423	0.550318627	-0.514811619	0.606684666	0.999978546
2.5.1.61	hydroxymethylbilane synthase	69.60118872	0.379465205	0.338063408	1.12246755	0.261663697	0.999978546
6.1.1.13	D-alanine--poly(phosphoribitol) ligase subunit 1	8.573527878	-0.352132264	0.943065509	-0.373391096	0.708857385	0.999978546
4.1.1.20	diaminopimelate decarboxylase	367.9980612	-0.002747237	0.188784684	-0.014552222	0.988389417	0.999978546
6.3.4.4	adenylosuccinate synthase	669.1225565	-0.019525121	0.179258701	-0.108921467	0.91326478	0.999978546
3.6.5.4	signal recognition particle subunit SRP54	540.0164761	-0.007469512	0.25605346	-0.029171689	0.976727661	0.999978546
1.1.1.132	GDP-mannose 6-dehydrogenase	7.106379203	0.065178082	1.351746683	0.048217675	0.961542764	0.999978546
5.3.1.5	xylose isomerase	118.9131173	0.076273794	0.466668138	0.16344333	0.870169389	0.999978546
4.2.2.23	rhamnogalacturonan endolyase	106.7907997	-0.338785789	0.369773265	-0.916198711	0.359562682	0.999978546
4.1.1.79	sulfopyruvate decarboxylase subunit alpha	1.162573941	2.528234747	4.070057022	0.621179196	0.534481726	0.999978546
1.17.7.4	4-hydroxy-3-methylbut-2-en-1-yl diphosphate reductase	354.784172	0.019308092	0.419124759	0.046067648	0.963256332	0.999978546
4.1.1.74	indolepyruvate decarboxylase	0.392755038	0.460515369	4.11922074	0.11179672	0.910984588	0.999978546
4.2.2.7	heparin lyase	8.270872475	-0.498613022	1.004823978	-0.496219271	0.619739716	0.999978546
4.2.1.19	imidazoleglycerol-phosphate dehydratase / histidinol-phosphat	197.1721382	-0.029838987	0.245521322	-0.121533179	0.903268737	0.999978546
6.3.-.-	#N/A	0.169214877	0.057211062	4.134457515	0.013837622	0.988959527	0.999978546
2.7.12.2	mitogen-activated protein kinase kinase 1	0.114087933	1.632877952	4.106512764	0.397631286	0.690901995	0.999978546
2.7.1.107	diacylglycerol kinase (ATP)	64.69343955	-0.352208504	0.458411696	-0.768323556	0.442294983	0.999978546
5.1.3.14	UDP-N-acetylglucosamine 2-epimerase (non-hydrolysing)	254.2481963	0.018557004	0.228804402	0.081104225	0.935359066	0.999978546
3.4.11.-	#N/A	191.0081273	-0.277179995	0.344591555	-0.80437257	0.421181838	0.999978546
3.1.3.89	5'-deoxynucleotidase	102.4893594	0.139214021	0.509183398	0.273406441	0.784540802	0.999978546
4.1.1.41	#N/A	0.335113387	-0.54827045	4.138197543	-0.132490159	0.894596607	0.999978546
3.1.3.57	inositol polyphosphate 1-phosphatase	0.160692243	0.094410072	4.143731151	0.022783831	0.981822705	0.999978546
2.4.1.18	1,4-alpha-glucan branching enzyme	1270.326465	0.023101231	0.25365752	0.091072525	0.927434964	0.999978546
4.1.1.37	uroporphyrinogen decarboxylase	71.82733174	0.331208405	0.350559756	0.944798708	0.344761652	0.999978546
3.6.1.61	diadenosine hexaphosphate hydrolase (ATP-forming)	0.213748207	-0.818392885	4.133650812	-0.197983072	0.843058308	0.999978546
2.7.1.195	PTS system, 2-O-A-mannosyl-D-glycerate-specific IIA componer	0.096307101	-0.778791352	4.138114321	-0.188199574	0.850720206	0.999978546
1.11.1.5	cytochrome c peroxidase	29.69912474	-0.415780134	0.513717337	-0.809355854	0.418310488	0.999978546
5.1.1.20	L-Ala-D/L-Glu epimerase	82.99796834	0.284225184	0.369883508	0.768418103	0.442238829	0.999978546
2.6.1.109	8-amino-3,8-dideoxy-alpha-D-manno-octulosonate transaminas	0.169128288	-1.008596429	4.140109862	-0.243615861	0.807528343	0.999978546
3.5.99.2	hydroxymethylpyrimidine/phosphomethylpyrimidine kinase / t	34.47460786	-0.342351245	0.626651949	-0.54631801	0.584847359	0.999978546
3.2.1.86	#N/A	335.5730526	-0.29049218	0.278092122	-1.044589749	0.29621262	0.999978546
5.4.99.62	D-ribose pyranase	8.727499142	-1.320307366	0.74784857	-1.765474212	0.077484101	0.999978546
6.3.2.4	D-alanine-D-alanine ligase	563.867796	-0.106519324	0.443632864	-0.240106927	0.810247364	0.999978546
1.8.1.7	glutathione reductase (NADPH)	3.926453027	-2.674581544	1.883384822	-1.420092969	0.155580617	0.999978546
6.3.1.-	#N/A	1.210030324	1.336341163	3.177568227	0.420554672	0.674080299	0.999978546
5.1.3.8	N-acylglucosamine 2-epimerase	101.6805329	-0.225205538	0.353974616	-0.636219456	0.524633394	0.999978546
2.7.4.3	adenylate kinase	907.9785391	0.413060154	0.398984714	1.035278145	0.300539033	0.999978546
2.4.1.247	beta-D-galactosyl-(1->4)-L-rhamnose phosphorylase	6.615225414	-0.343050344	1.082690946	-0.31684974	0.75135762	0.999978546
6.1.1.18	glutaminyl-tRNA synthetase	464.0232608	-0.079435533	0.142153193	-0.558802311	0.576296645	0.999978546
1.1.5.3	glycerol-3-phosphate dehydrogenase	521.1265768	0.128454746	0.349072044	0.367989209	0.71288128	0.999978546
2.3.1.247	3-keto-5-aminohexanoate cleavage enzyme	56.37761532	0.055392637	0.45379417	0.122065555	0.902847101	0.999978546
1.1.1.380	L-gulonate 5-dehydrogenase	19.37042004	-0.314136826	0.749628113	-0.41905689	0.675174556	0.999978546
1.1.1.262	4-hydroxythreonine-4-phosphate dehydrogenase	221.6681368	0.035013332	0.251196728	0.139386098	0.889145058	0.999978546
2.1.2.2	phosphoribosylglycinamide formyltransferase	478.4970592	-0.071888958	0.214079942	-0.335804267	0.737018472	0.999978546
2.7.7.68	2-phospho-L-lactate guanylyltransferase	2.105625982	-7.85E-05	2.917893757	-2.69E-05	0.999978546	0.999978546
2.1.1.131	precorrin-3B C17-methyltransferase	112.1616963	0.34443727	0.365772934	0.941669646	0.3463618	0.999978546
2.3.1.35	glutamate N-acetyltransferase / amino-acid N-acetyltransferase	232.9759184	-0.096171688	0.304716984	-0.31560987	0.752298645	0.999978546
2.7.1.151	#N/A	0.160692243	0.094410072	4.143731151	0.022783831	0.981822705	0.999978546
2.3.1.12	pyruvate dehydrogenase E2 component (dihydrolipoamide acet	60.27244184	-0.170166707	0.598502173	-0.28432095	0.776164432	0.999978546
4.1.2.13	fructose 1,6-bisphosphate aldolase/phosphatase	1537.630637	0.154308107	0.434219565	0.355368848	0.722313283	0.999978546
2.4.1.186	glycogenin	0.213123362	-0.558144012	4.139315718	-0.134839681	0.892738631	0.999978546
3.1.3.81	phosphatidylglycerophosphatase B	0.21899488	1.112815071	4.121162036	0.270024586	0.787141339	0.999978546
5.3.3.3	4-hydroxybutyryl-CoA dehydratase / vinylacetyl-CoA-Delta-ison	61.11807847	0.746491611	0.403211029	1.851367043	0.064116767	0.999978546
1.2.1.21	lactaldehyde dehydrogenase / glycolaldehyde dehydrogenase	34.86369268	0.237449263	0.40292331	0.589316271	0.555649132	0.999978546
3.6.3.41	heme exporter protein A	3.354275479	-0.552735458	1.019675507	-0.542069956	0.587770315	0.999978546
2.7.10.-	#N/A	4.805320692	-0.484814349	1.430713009	-0.338862054	0.734713651	0.999978546
1.1.1.385	dihydroanticapsin dehydrogenase	0.063329795	0.094410257	4.143731151	0.022783876	0.98182267	0.999978546
5.3.1.24	phosphoribosylanthranilate isomerase	94.20396798	-0.361449526	0.415479076	-0.869958434	0.38432312	0.999978546
5.4.99.18	5-(carboxyamino)imidazole ribonucleotide mutase	311.3499985	-0.103738146	0.197972974	-0.524001556	0.600277458	0.999978546
2.6.1.36	L-lysine 6-transaminase	1.472039473	2.188304398	4.047438573	0.540664017	0.588739184	0.999978546
2.5.1.-	#N/A	211.8230542	-0.913475918	0.645269218	-1.415650852	0.156877755	0.999978546
2.7.7.2	FAD synthetase	322.5539954	0.194157323	0.219959813	0.882694528	0.37740134	0.999978546
2.5.1.21	farnesyl-diphosphate farnesyltransferase	2.623950859	-0.183357615	1.272127039	-0.144134673	0.885394126	0.999978546
4.2.1.47	GDPmannose 4,6-dehydratase	174.6487184	0.113685719	0.245428757	0.463212706	0.64321191	0.999978546
2.3.1.19	phosphate butyryltransferase	49.06941609	-0.000257938	0.382068914	-0.000675108	0.999461342	0.999978546
1.7.1.13	7-cyano-7-deazaguanine reductase	52.99814833	0.316095089	0.412019744	0.767184325	0.442971931	0.999978546
6.1.1.1	tyrosyl-tRNA synthetase	382.8689634	0.105349992	0.230554232	0.456942348	0.647712484	0.999978546
4.2.1.33	3-isopropylmalate dehydratase	629.01279	3.03E-05	0.236511554	0.000128048	0.999897833	0.999978546
4.1.1.68	#N/A	0.107481033	-0.255046689	4.141265221	-0.061586659	0.950892001	0.999978546
3.1.3.1	alkaline phosphatase	161.6018233	0.109578726	0.391143548	0.280149645	0.779362697	0.999978546
2.7.8.26	adenosylcobinamide-GDP ribazoletransferase	150.8851714	0.707810462	0.354789111	1.995017437	0.046040978	0.999978546
1.1.3.15	glycolate oxidase	192.7034939	0.278066386	0.403450359	0.689220816	0.490684319	0.999978546
6.3.2.36	4-phosphopantoate---beta-alanine ligase	4.726773866	0.251377112	2.246145082	0.111914904	0.910890879	0.999978546
3.1.3.16	protein phosphatase	400.4206521	0.221889573	0.268141057	0.827510622	0.407947704	0.999978546

1.4.1.16	diaminopimelate dehydrogenase	159.4748883	-0.089866943	0.210870485	-0.426171275	0.669983047	0.999978546
3.2.1.67	galacturan 1,4-alpha-galacturonidase	0.121388873	-0.679822118	4.137055041	-0.164325132	0.869475198	0.999978546
2.4.1.337	1,2-diacylglycerol 3-alpha-glucosyltransferase	63.56108641	-0.716228435	0.488188709	-1.46711389	0.142345076	0.999978546
3.1.3.5	5'-nucleotidase	331.4545819	-0.10669611	0.322172127	-0.331177346	0.740510531	0.999978546
2.7.9.3	selenide, water dikinase	145.2147763	0.089836856	0.262955979	0.341642186	0.732620185	0.999978546
3.2.1.52	beta-N-acetylhexosaminidase	1287.687515	-0.434529842	0.277311934	-1.566935239	0.11712983	0.999978546
3.1.1.45	carboxymethylenebutenolidase	0.264436359	0.045186449	4.14168021	0.010910173	0.991295114	0.999978546
3.6.3.27	#N/A	156.9113029	-0.551057282	0.523708439	-1.052221504	0.292697936	0.999978546
5.4.2.10	phosphoglucosamine mutase	560.1447399	0.183711572	0.242731802	0.756850034	0.449139713	0.999978546
3.8.1.5	haloalkane dehalogenase	0.20978502	-0.530666537	4.135714859	-0.128313134	0.897901171	0.999978546
6.5.1.4	RNA 3'-terminal phosphate cyclase (ATP)	0.104522907	-0.977299903	4.136864693	-0.236241689	0.813245132	0.999978546
3.4.21.89	signal peptidase I	631.9471178	0.176796059	0.21891848	0.807585552	0.419327479	0.999978546
2.7.11.24	mitogen-activated protein kinase 1/3	0.48207673	0.09440421	4.143731151	0.022782417	0.981823834	0.999978546
3.6.1.66	XTP/dITP diphosphohydrolase	461.2721418	0.279713063	0.279878102	0.999410318	0.317595964	0.999978546
4.1.3.36	naphthoate synthase	46.53090059	-0.367017386	0.362515802	-1.012417621	0.311338417	0.999978546
2.1.1.172	16S rRNA (guanine1207-N2)-methyltransferase	12.66763079	-0.06624737	1.507964162	-0.043931661	0.964958878	0.999978546
4.1.2.29	6-phospho-5-dehydro-2-deoxy-D-gluconate aldolase	0.107128162	0.094412122	4.143731151	0.022784326	0.981822311	0.999978546
2.7.11.1	checkpoint serine/threonine-protein kinase	1213.893284	-0.103320584	0.126642197	-0.815846428	0.414587975	0.999978546
2.4.1.289	N-acetylglucosaminyl-diphospho-decaprenol L-rhamnosyltransf	0.047497346	0.094411622	4.143731151	0.022784205	0.981822407	0.999978546
3.6.3.34	iron complex transport system ATP-binding protein	275.8948902	0.005826789	0.326297476	0.01785729	0.985752702	0.999978546
2.5.1.6	S-adenosylmethionine synthetase	730.5001727	0.293737742	0.244716608	1.200317966	0.230015875	0.999978546
2.5.1.54	3-deoxy-7-phosphoheptulonate synthase	876.9998298	-1.243001597	0.82688036	-1.503242376	0.132776554	0.999978546
2.7.7.8	polyribonucleotide nucleotidyltransferase	1148.232146	-0.047534089	0.250123205	-0.190042698	0.849275672	0.999978546
3.5.4.5	cytidine deaminase	265.2143101	0.250401713	0.292890481	0.854932915	0.392588287	0.999978546
5.5.1.27	D-galactarolactone cycloisomerase	0.264306213	-0.975637338	4.134419772	-0.235979265	0.81344876	0.999978546
3.2.1.25	beta-mannosidase	250.3589538	-0.176901405	0.215183592	-0.822095231	0.411022699	0.999978546
2.1.1.148	thymidylate synthase (FAD)	154.5213535	0.249597997	0.227318312	1.098010954	0.272199706	0.999978546
4.2.1.140	gluconate/galactonate dehydratase	0.079162244	0.090751619	4.143557913	0.021901858	0.982526242	0.999978546
3.6.3.2	Mg2+-importing ATPase	72.70340602	-0.6928285	0.538524718	-1.286530547	0.198257965	0.999978546
4.1.1.70	glutaconyl-CoA decarboxylase	95.93604748	-0.113758887	0.537855741	-0.211504458	0.832493654	0.999978546
2.7.4.9	dTMP kinase	187.7621884	0.136229439	0.268869355	0.506675216	0.612382723	0.999978546
2.7.1.176	UDP-N-acetylglucosamine kinase	4.654161857	0.192843389	1.141201386	0.168982786	0.865810182	0.999978546
2.4.1.291	N-acetylgalactosamine-N,N'-diacetylbacillosaminyl-diphospho-u	0.158324487	0.052535341	4.141950813	0.012683719	0.989880128	0.999978546
2.7.1.30	glycerol kinase	777.9550507	0.172116006	0.317572108	0.541974566	0.587836027	0.999978546
2.9.1.1	L-seryl-tRNA(Ser) seleniumtransferase	26.83744539	0.061074089	0.549466689	0.111151577	0.911496149	0.999978546
1.4.3.16	L-aspartate oxidase	256.2428411	-0.467493646	0.188350454	-2.482041508	0.013063206	0.999978546
3.5.1.102	2-amino-5-formylamino-6-ribosylaminopyrimidin-4(3H)-one 5'-r	3.366320964	1.494147917	2.159063823	0.692035085	0.488915296	0.999978546
3.2.1.8	endo-1,4-beta-xylanase	117.6752233	-0.389040335	0.437494036	-0.889247172	0.373870256	0.999978546
1.1.1.117	D-arabinose 1-dehydrogenase	0.919026554	-0.952895973	4.099326509	-0.232451836	0.816187092	0.999978546
2.7.4.31	5-(aminomethyl)-3-furanmethanol phosphate kinase	6.817399672	1.779682191	3.201759021	0.55584514	0.578316724	0.999978546
2.1.1.10	homocysteine S-methyltransferase	3.904802519	-1.61310046	1.406604319	-1.146804712	0.251462341	0.999978546
3.6.3.32	glycine betaine/proline transport system ATP-binding protein	53.71368105	0.506661737	0.461087736	1.098840192	0.271837778	0.999978546
2.5.1.89	tritrans,polycis-undecaprenyl-diphosphate synthase [geranylge	2.984712843	2.733456025	2.185734677	1.250589129	0.211084419	0.999978546
1.17.1.8	4-hydroxy-tetrahydrodipicolinate reductase	407.8052854	0.166686156	0.247393245	0.673770036	0.500457527	0.999978546
3.1.1.83	epsilon-lactone hydrolase	14.81851815	0.055656647	0.747591881	0.074447901	0.940653995	0.999978546
2.1.1.80	chemotaxis protein methyltransferase CheR	123.6007911	-0.601911947	0.588779046	-1.022305313	0.306636419	0.999978546
2.3.1.15	glycerol-3-phosphate O-acyltransferase 1/2	587.5392468	-0.186426009	0.212990742	-0.875277522	0.381422922	0.999978546
1.17.1.1	CDP-4-dehydro-6-deoxyglucose reductase, E3	83.02591322	0.137638051	0.403867253	0.340800225	0.733253981	0.999978546
1.4.4.2	glycine dehydrogenase	368.0048341	-0.093992857	0.302577166	-0.310640946	0.756073596	0.999978546
4.1.1.32	phosphoenolpyruvate carboxykinase (GTP)	297.5921169	0.184268757	0.567684969	0.324596858	0.745486205	0.999978546
2.3.1.30	serine O-acetyltransferase	286.1181777	-0.047061607	0.246766516	-0.190713097	0.848750377	0.999978546
3.2.1.-	#N/A	103.1252457	0.229393204	0.347681608	0.659779517	0.50939533	0.999978546
5.4.99.15	(1->4)-alpha-D-glucan 1-alpha-D-glucosylmutase	0.155156823	1.387702443	4.109302711	0.33769779	0.735590941	0.999978546
4.2.99.20	2-succinyl-6-hydroxy-2,4-cyclohexadiene-1-carboxylate synthas	3.361004476	-1.015512124	1.554792795	-0.653149492	0.513659908	0.999978546
4.2.1.9	dihydroxy-acid dehydratase	540.2886476	0.09943158	0.233203555	0.426372491	0.669836443	0.999978546
6.3.4.2	CTP synthase	512.1476844	0.004028863	0.179603038	0.022432044	0.98210332	0.999978546
1.12.99.-	#N/A	251.4678543	1.547556957	1.240180069	1.247848595	0.212086509	0.999978546
1.1.1.363	glucose-6-phosphate 1-dehydrogenase	223.5508127	0.310436066	0.471113507	0.658941129	0.509933574	0.999978546
2.4.1.129	monofunctional glycosyltransferase	66.90475561	-0.160543008	0.385349944	-0.416616143	0.676959199	0.999978546
4.3.1.4	formiminotetrahydrofolate cyclodeaminase	55.490593	-0.611515365	0.483211561	-1.265523044	0.205683893	0.999978546
2.1.1.268	tRNAThr (cytosine32-N3)-methyltransferase	0.031664897	0.094413212	4.143731151	0.022784589	0.981822101	0.999978546
2.7.1.21	thymidine kinase	124.3847308	-0.246382081	0.352828429	-0.698305636	0.484986075	0.999978546
1.14.99.46	pyrimidine oxygenase	0.04941368	0.09441837	4.143731151	0.022785834	0.981821108	0.999978546
3.4.23.5	cathepsin D	0.214256324	0.094408575	4.143731151	0.02278347	0.981822993	0.999978546
2.4.1.281	4-O-beta-D-mannosyl-D-glucose phosphorylase	191.910689	-0.527657759	0.437485431	-1.206115042	0.227773136	0.999978546
3.1.3.32	bifunctional polynucleotide phosphatase/kinase	0.228120967	0.831630387	4.140581219	0.200848708	0.840816876	0.999978546
5.1.1.7	diaminopimelate epimerase	227.37739	-0.179514065	0.192292459	-0.933547087	0.350537571	0.999978546
2.3.2.8	arginyl-tRNA---protein transferase	4.178957052	-1.14804563	2.460607786	-0.466569941	0.640807597	0.999978546
2.1.1.314	diphthine methyl ester synthase	0.868720118	1.610855926	4.107781822	0.392147391	0.694949313	0.999978546
2.1.1.206	tRNA (cytidine56-2'-O)-methyltransferase	2.423959494	2.301651719	2.112400965	1.089590356	0.275893633	0.999978546
1.1.1.291	2-hydroxymethylglutarate dehydrogenase	1.511684876	1.647605959	3.391501167	0.485804332	0.627105903	0.999978546
2.5.1.48	cystathionine gamma-synthase	43.32150649	-0.647255784	0.449875887	-1.438743003	0.150223351	0.999978546
6.3.3.1	phosphoribosylformylglycinamidine cyclo-ligase	467.9122205	-0.113696934	0.224922527	-0.505493761	0.61321208	0.999978546
6.3.2.12	#N/A	404.1838337	-0.065488632	0.221167087	-0.29610478	0.767150063	0.999978546
2.8.1.12	molybdopterin synthase catalytic subunit	7.150256944	0.496746343	1.1453745	0.43369775	0.664507941	0.999978546
3.6.4.6	vesicle-fusing ATPase	0.82589221	-0.216942041	4.140668999	-0.052392993	0.958215557	0.999978546
1.21.4.1	#N/A	190.0109388	-1.129072042	0.748818999	-1.507803679	0.13160478	0.999978546
2.7.1.130	tetraacyldisaccharide 4'-kinase	112.9010033	-0.092706126	0.310849519	-0.298234743	0.765524007	0.999978546
1.2.1.8	betaine-aldehyde dehydrogenase	13.49926807	1.294344736	1.110499002	1.165552362	0.243795478	0.999978546
1.1.1.31	3-hydroxyisobutyrate dehydrogenase	24.6938277	0.156019719	0.498199707	0.313167023	0.754153771	0.999978546
2.4.1.320	beta-1,4-mannooligosaccharide/beta-1,4-mannosyl-N-acetylglu	286.1595224	-0.295106564	0.426679348	-0.691635454	0.489166291	0.999978546
2.7.6.1	ribose-phosphate pyrophosphokinase	499.4320736	0.059781708	0.282670361	0.211489127	0.832505616	0.999978546
4.1.2.20	2-dehydro-3-deoxyglucarate aldolase	2.948779531	0.047436874	1.341376355	0.035364329	0.971789228	0.999978546
1.1.1.378	L-rhamnose 1-dehydrogenase	0.031664897	0.094413212	4.143731151	0.022784589	0.981822101	0.999978546
1.1.1.86	ketol-acid reductoisomerase	462.1428873	0.104812664	0.296798166	0.353144582	0.723980052	0.999978546
5.1.3.3	aldose 1-epimerase	653.8019181	-0.072279851	0.336588054	-0.21474277	0.829967875	0.999978546
5.3.1.28	D-sedoheptulose 7-phosphate isomerase	39.20850805	0.000951967	0.343046451	0.002775039	0.997785842	0.999978546

2.3.1.47	8-amino-7-oxononanoate synthase	68.37815135	-0.312298648	0.528956911	-0.590404703	0.554919359	0.999978546
2.4.99.13	3-deoxy-D-manno-octulosonic-acid transferase	102.8745696	-0.135612171	0.327661726	-0.413878582	0.67896303	0.999978546
3.1.3.23	sugar-phosphatase	1.477961753	1.828902556	3.593765486	0.508909822	0.610815435	0.999978546
3.2.1.170	mannosylglycerate hydrolase	11.41345483	-0.164719408	0.906518779	-0.181705456	0.855813884	0.999978546
3.4.13.9	Xaa-Pro dipeptidase	41.36870323	-0.426997395	0.610793588	-0.699086244	0.484498134	0.999978546
2.4.2.48	7-cyano-7-deazaguanine tRNA-ribosyltransferase	3.042578273	1.91235339	2.421526534	0.789730512	0.429685168	0.999978546
3.6.1.26	CDP-diacylglycerol pyrophosphatase	0.18037173	-0.323516828	4.141397008	-0.078117801	0.937734347	0.999978546
2.1.1.290	tRNA wybutosine-synthesizing protein 4	0.053564081	0.094415333	4.143731151	0.022785101	0.981821693	0.999978546
2.1.1.294	O-antigen chain-terminating methyltransferase	0.321384486	0.094406417	4.143731151	0.022782949	0.981823409	0.999978546
1.1.1.41	isocitrate dehydrogenase (NAD+)	33.21155859	0.557111778	0.403999002	1.378992956	0.167896926	0.999978546
2.3.1.24	Acyl-CoA-dependent ceramide synthase	0.053564081	0.094415333	4.143731151	0.022785101	0.981821693	0.999978546
2.7.1.5	rhamnulokinase	365.1696497	-0.449315614	0.536765071	-0.837080574	0.402547279	0.999978546
2.1.1.179	16S rRNA (guanine(1405)-N(7))-methyltransferase	0.01747704	-0.22961245	4.141706174	-0.055439097	0.955788649	0.999978546
2.7.1.189	autoinducer-2 kinase	10.84554191	0.324035146	1.112741709	0.291204278	0.770895091	0.999978546
2.4.-.-	#N/A	73.37059448	-0.615751853	0.330111343	-1.865285354	0.062141417	0.999978546
3.2.2.9	adenosylhomocysteine nucleosidase	244.216832	0.034369061	0.206145521	0.16672233	0.867588535	0.999978546
2.8.3.1	propionate CoA-transferase	63.7064274	-0.127546452	0.526069719	-0.242451614	0.808430245	0.999978546
4.1.1.48	indole-3-glycerol phosphate synthase	101.6641702	-0.185445342	0.408629774	-0.453822394	0.649956664	0.999978546
3.5.3.18	dimethylargininase	1.952238584	-2.555444182	1.779378642	-1.436144125	0.150961334	0.999978546
2.8.1.2	thiosulfate/3-mercaptopyruvate sulfurtransferase	4.40773592	-0.698253259	1.129355953	-0.618275626	0.536393666	0.999978546
2.1.3.-	#N/A	5.301016546	-1.609143921	1.060122657	-1.517884661	0.129043477	0.999978546
2.8.1.-	#N/A	84.54624326	-0.092067027	0.800715276	-0.11498098	0.908460198	0.999978546
3.6.3.7	#N/A	18.59393918	0.22647793	0.48596277	0.466039673	0.641187103	0.999978546
2.7.1.52	fucokinase	36.04461727	-0.014456639	0.473833314	-0.030509966	0.975660345	0.999978546
3.1.26.8	ribonuclease M5	69.66098261	0.216009185	0.363248248	0.594659948	0.552070799	0.999978546
4.1.3.30	methylisocitrate lyase	1.214680599	-0.698106109	2.316381155	-0.301377909	0.763126338	0.999978546
3.5.1.24	choloylglycine hydrolase	386.5802979	0.405584438	0.189539385	2.139842531	0.032367495	0.999978546
2.1.1.35	tRNA (uracil-5-)-methyltransferase	1.234391661	-0.415246295	2.570875445	-0.161519414	0.871684322	0.999978546
5.4.2.6	beta-phosphoglucomutase	18.10806377	-0.85563892	0.776806627	-1.101482519	0.270686709	0.999978546
2.5.1.29	geranylgeranyl diphosphate synthase, type III	398.5314581	0.430868996	0.206850637	2.082995742	0.037251612	0.999978546
2.6.1.45	#N/A	1.573124213	0.289161523	2.186243187	0.132264116	0.894775389	0.999978546
1.3.1.93	very-long-chain enoyl-CoA reductase	0.160692243	0.094410072	4.143731151	0.022783831	0.981822705	0.999978546
1.1.1.69	gluconate 5-dehydrogenase	430.6355441	0.337424765	0.408478735	0.826052218	0.408774471	0.999978546
1.1.1.-	#N/A	1599.197698	-0.207798522	0.818947019	-0.253738664	0.799697462	0.999978546
2.7.1.146	ADP-dependent phosphofructokinase/glucokinase	7.609187489	0.245888078	1.004475699	0.244792461	0.806617132	0.999978546
1.1.99.1	choline dehydrogenase	0.4007054	-1.856143693	4.11904242	-0.450625049	0.65225981	0.999978546
3.6.3.17	simple sugar transport system ATP-binding protein	2516.512826	0.10173022	0.371118066	0.274118211	0.78399378	0.999978546
1.4.1.24	3-dehydroquinate synthase II	6.211384313	1.642604101	1.494666723	1.098976833	0.271778171	0.999978546
4.2.2.17	inulin fructotransferase (DFA-I-forming)	0.203184014	1.577249374	4.124371835	0.382421721	0.702148582	0.999978546
3.6.3.29	molybdate transport system ATP-binding protein	73.99404027	-0.286286916	0.524142579	-0.546200455	0.584928153	0.999978546
1.1.1.377	L-rhamnose 1-dehydrogenase	0.031664897	0.094413212	4.143731151	0.022784589	0.981822101	0.999978546
3.1.3.64	myotubularin	0.240510691	-0.396287166	4.138517643	-0.095755824	0.923714504	0.999978546
3.1.3.3	phosphoserine phosphatase	172.8292548	-0.465435097	0.288930601	-1.610888896	0.107203941	0.999978546
2.7.8.37	alpha-D-ribose 1-methylphosphonate 5-triphosphate synthase	6.201350423	-0.063298977	1.56458554	-0.040457345	0.967728513	0.999978546
2.1.1.215	tRNA (guanine26-N2/guanine27-N2)-dimethyltransferase	0.68372251	1.884001795	4.103983205	0.459066644	0.646186309	0.999978546
2.6.1.51	alanine-glyoxylate transaminase / serine-glyoxylate transamina	1.573124213	0.289161523	2.186243187	0.132264116	0.894775389	0.999978546
1.2.4.4	3-methyl-2-oxobutanoate dehydrogenase (2-methylpropanoyl-	91.63417995	0.787477915	0.354928908	2.218691963	0.026507688	0.999978546
3.4.22.15	cathepsin L	6.825057858	0.58563963	3.531585674	0.165829088	0.868291454	0.999978546
3.1.2.28	1,4-dihydroxy-2-naphthoyl-CoA hydrolase	0.144460652	-1.00194317	4.133461154	-0.24239811	0.808471699	0.999978546
2.7.1.203	PTS system, D-glucosaminate-specific IIA component	2.990922439	-1.2637824	1.64754627	-0.767069443	0.443040229	0.999978546
3.2.1.187	beta-L-arabinobiosidase	0.420346293	-0.183745437	4.092468091	-0.044898441	0.96418826	0.999978546
3.2.1.80	fructan beta-fructosidase	131.8740289	-0.248967572	0.306375244	-0.812623006	0.416434237	0.999978546
5.1.3.32	L-rhamnose mutarotase	138.9551777	0.040661127	0.383948197	0.105902639	0.9156596	0.999978546
2.3.1.199	elongation of very long chain fatty acids protein 6	0.160692243	0.094410072	4.143731151	0.022783831	0.981822705	0.999978546
2.1.1.184	23S rRNA (adenine-N6)-dimethyltransferase	9.19044385	-0.182151198	0.808897584	-0.2251845	0.821835747	0.999978546
5.4.3.3	beta-lysine 5,6-aminomutase alpha subunit	110.9655405	-0.138458398	0.573049923	-0.241616642	0.809077226	0.999978546
2.7.1.39	homoserine kinase	172.7008888	0.174019601	0.263927709	0.659345703	0.509673801	0.999978546
1.3.1.87	2,3-dihydroxy-2,3-dihydrophenylpropionate dehydrogenase	0.198327269	0.054033837	4.142007235	0.013045326	0.989591631	0.999978546
2.1.1.282	tRNA wybutosine-synthesizing protein 3	0.428512649	0.094404856	4.143731151	0.022782573	0.981823709	0.999978546
4.-.-.-	#N/A	0.528273679	0.637291315	4.092532966	0.155720509	0.876253331	0.999978546
4.1.99.3	deoxyribodipyrimidine photo-lyase	1.11007286	-0.340757073	2.804466192	-0.121505146	0.903290939	0.999978546
4.1.2.9	xylulose-5-phosphate/fructose-6-phosphate phosphoketolase	141.7183535	-0.01635918	0.327530131	-0.049947099	0.960164545	0.999978546
2.7.1.43	glucuronokinase	0.428512649	0.094404856	4.143731151	0.022782573	0.981823709	0.999978546
1.1.1.157	3-hydroxybutyryl-CoA dehydrogenase	1460.350109	-0.086348026	0.647648716	-0.133325402	0.893936039	0.999978546
1.1.1.369	myo-inositol 2-dehydrogenase / D-chiro-inositol 1-dehydrogena	80.77035126	0.309015038	0.432697218	0.714159982	0.475128259	0.999978546
1.1.1.34	hydroxymethylglutaryl-CoA reductase (NADPH)	8.97296793	1.765368473	1.736999863	1.016331959	0.309471335	0.999978546
3.4.22.34	legumain	28.63541982	-0.136853852	1.919193788	-0.07130799	0.943152636	0.999978546
2.1.1.86	#N/A	346.4779204	2.77272279	1.447099071	1.916055954	0.055357974	0.999978546
1.14.12.17	nitric oxide dioxygenase	16.24167399	-1.34552839	1.408553272	-0.955255593	0.339448459	0.999978546
2.7.8.-	#N/A	921.7247401	0.10877928	0.221832124	0.490367573	0.623873818	0.999978546
3.1.5.1	dGTPase	347.7338709	-0.034906504	0.211105309	-0.165351142	0.868667607	0.999978546
3.4.24.-	#N/A	3286.291944	0.102051551	0.147230209	0.693142741	0.488219977	0.999978546
2.1.2.13	UDP-4-amino-4-deoxy-L-arabinose formyltransferase / UDP-glu	2.389353235	0.14330415	1.387919669	0.10325104	0.917763732	0.999978546
3.1.3.24	sucrose-6-phosphatase	863.7598049	-0.014253344	0.155819745	-0.091473288	0.92711653	0.999978546
1.1.1.244	methanol dehydrogenase	0.059587395	1.425432941	4.11370627	0.346508197	0.728960825	0.999978546
3.2.1.93	trehalose-6-phosphate hydrolase	11.81364766	-0.87384765	0.784991694	-1.113193499	0.265625334	0.999978546
2.5.1.56	N-acetylneuraminate synthase	25.85352984	-0.632614116	0.480623223	-1.316237098	0.188094476	0.999978546
3.4.17.14	zinc D-Ala-D-Ala carboxypeptidase	0.153183881	1.800672354	4.103419624	0.438822377	0.660790245	0.999978546
2.7.1.15	ribokinase	117.0899583	0.230506294	0.361886147	0.636958048	0.52415217	0.999978546
3.6.3.24	nickel transport system ATP-binding protein	12.08036817	-0.81544829	1.050150469	-0.776506143	0.437450195	0.999978546
2.3.1.117	2,3,4,5-tetrahydropyridine-2,6-dicarboxylate N-succinyltransfer	33.00507093	-0.305679191	0.680392123	-0.44926915	0.65323751	0.999978546
2.3.2.-	#N/A	10.17339722	0.373951519	1.057729825	0.353541623	0.72368243	0.999978546
2.4.1.46	1,2-diacylglycerol 3-beta-galactosyltransferase	0.053564081	0.094415333	4.143731151	0.022785101	0.981821693	0.999978546
2.6.1.87	UDP-4-amino-4-deoxy-L-arabinose-oxoglutarate aminotransfera	0.58308361	-1.052793503	4.131656834	-0.254811458	0.798868725	0.999978546
5.99.1.-	#N/A	358.5001704	-0.15045537	0.256427671	-0.586736095	0.557380962	0.999978546
3.6.1.67	dihydroneopterin triphosphate diphosphatase	2.498223838	-2.80534646	1.912265183	-1.467027944	0.142368454	0.999978546
2.2.1.2	transaldolase	347.3412832	0.029488517	0.386811544	0.076234842	0.939232264	0.999978546

6.1.1.6	#N/A	577.45988	0.100742272	0.143265972	0.703183526	0.481941385	0.999978546
4.3.1.7	#N/A	29.98753477	-0.025118643	0.591943738	-0.042434172	0.966152587	0.999978546
6.1.1.24	nondiscriminating glutamyl-tRNA synthetase	6.713044222	-1.290964214	1.234952224	-1.045355593	0.295858652	0.999978546
2.4.1.25	4-alpha-glucanotransferase	826.5909988	0.611347685	0.272453257	2.243862639	0.024841241	0.999978546
3.4.15.6	cyanophycinase	0.722055264	2.07170776	4.087880988	0.506792582	0.612300362	0.999978546
2.4.1.182	lipid-A-disaccharide synthase	97.65809609	-0.620988806	0.260816602	-2.380940484	0.017268502	0.999978546
3.1.1.24	3-oxoadipate enol-lactonase	0.043850487	0.094417027	4.143731151	0.02278551	0.981821367	0.999978546
2.2.1.9	2-succinyl-5-enolpyruvyl-6-hydroxy-3-cyclohexene-1-carboxylat	86.31340977	-0.19011027	0.367386222	-0.517467065	0.604830154	0.999978546
3.2.1.165	exo-1,4-beta-D-glucosaminidase	3.174521815	0.301182584	1.559325298	0.193149296	0.846842047	0.999978546
1.1.1.18	myo-inositol 2-dehydrogenase / D-chiro-inositol 1-dehydrogena	80.77035126	0.309015038	0.432697218	0.714159982	0.475128259	0.999978546
2.4.1.7	sucrose phosphorylase	40.05453102	0.1369203	0.580434404	0.235892805	0.813515852	0.999978546
3.5.4.2	adenine deaminase	221.7394094	0.427748091	0.366544494	1.166974536	0.243220653	0.999978546
5.2.1.8	peptidylprolyl isomerase	1947.119805	-0.414255122	0.365448597	-1.133552366	0.256982363	0.999978546
5.1.1.18	serine racemase	34.51337451	-0.569992626	0.471782201	-1.208168992	0.226982266	0.999978546
3.5.1.97	acyl-homoserine-lactone acylase	0.654836639	1.171132699	4.094008018	0.286060187	0.774832024	0.999978546
2.6.1.13	ornithine--oxo-acid transaminase	47.38238285	-0.129662169	0.497642997	-0.260552585	0.794437559	0.999978546
1.10.3.10	#N/A	6.011939212	-4.325686678	2.401814673	-1.801007683	0.071701669	0.999978546
2.7.7.6	DNA-directed RNA polymerase	6413.048508	0.283240128	0.350603569	0.807864359	0.419168671	0.999978546
4.1.3.39	4-hydroxy 2-oxovalerate aldolase	107.0039189	-0.406667583	0.377839761	-1.076296423	0.281794708	0.999978546
1.21.4.2	glycine/sarcosine/betaine reductase complex component A	1374.313735	0.177801988	0.894766433	0.198713297	0.842487022	0.999978546
6.4.1.1	pyruvate carboxylase	459.749004	-0.030159367	0.334137172	-0.090260436	0.928080259	0.999978546
2.7.2.4	aspartate kinase	707.3786645	0.026518129	0.34148546	0.07765522	0.938102316	0.999978546
2.3.1.23	lysophosphatidylcholine acyltransferase / lyso-PAF acetyltransf	0.053564081	0.094415333	4.143731151	0.022785101	0.981821693	0.999978546
4.3.1.1	aspartate ammonia-lyase	270.3826272	-0.027451487	0.394073832	-0.069660772	0.944463665	0.999978546
2.3.2.23	ubiquitin-conjugating enzyme E2 R	1.849160922	0.136761847	4.125196337	0.033152809	0.97355273	0.999978546
2.7.1.1	hexokinase	14.71173957	0.196574776	0.583060595	0.337142963	0.736009132	0.999978546
5.1.3.1	ribulose-phosphate 3-epimerase	417.9879526	0.579467162	0.315462384	1.836881963	0.066227323	0.999978546
1.1.1.302	2,5-diamino-6-(ribosylamino)-4(3H)-pyrimidinone 5'-phosphate	2.485401298	2.419985867	2.508190403	0.964833397	0.334628265	0.999978546
3.1.1.-	#N/A	496.6853923	-0.0900286	0.168879074	-0.533095056	0.593967781	0.999978546
1.6.99.3	NADH dehydrogenase	65.55285561	0.124522232	0.364840644	0.341305811	0.732873373	0.999978546
1.7.99.4	#N/A	30.33693764	-0.691197264	0.65488839	-1.055442843	0.291222838	0.999978546
3.5.4.31	5-methylthioadenosine/S-adenosylhomocysteine deaminase	210.6530955	0.109314853	0.482982683	0.226332861	0.820942542	0.999978546
3.1.3.90	maltose 6'-phosphate phosphatase	12.88136179	-2.14912722	0.896174372	-2.398112786	0.01647979	0.999978546
4.1.1.15	glutamate decarboxylase	99.45348073	0.234210238	0.392715535	0.596386486	0.550917066	0.999978546
4.1.1.5	acetolactate decarboxylase	2.903166624	-2.232929565	2.621090091	-0.851908743	0.394264744	0.999978546
4.6.1.17	cyclic pyranopterin monophosphate synthase	64.49444168	-0.349127781	0.358771494	-0.973120181	0.330493575	0.999978546
2.3.1.29	glycine C-acetyltransferase	381.709002	0.09902834	0.471383373	0.210080257	0.833605034	0.999978546
2.1.1.190	23S rRNA (uracil1939-C5)-methyltransferase	488.7838662	-0.104459665	0.189851493	-0.55021777	0.582170016	0.999978546
1.4.7.1	glutamate synthase (ferredoxin)	105.2440678	-0.088467599	0.46187354	-0.191540738	0.848101967	0.999978546
3.1.26.-	#N/A	413.5075683	0.238946213	0.256914783	0.930060192	0.35233992	0.999978546
3.2.1.156	oligosaccharide reducing-end xylanase	66.937681	-0.303202427	0.410194958	-0.739166634	0.459805819	0.999978546
2.8.3.6	#N/A	0.04941368	0.09441837	4.143731151	0.022785834	0.981821108	0.999978546
3.6.3.16	arsenite/tail-anchored protein-transporting ATPase	7.85424895	0.585036088	0.623509377	0.938295571	0.348092535	0.999978546
6.3.5.11	cobyrinic acid a,c-diamide synthase	160.4431359	0.274826239	0.334565291	0.82144277	0.41139411	0.999978546
2.7.11.5	isocitrate dehydrogenase kinase/phosphatase	0.433381955	-1.53921697	4.125912054	-0.373061023	0.709103027	0.999978546
2.1.2.1	glycine hydroxymethyltransferase	453.8568204	0.037930633	0.2303165	0.164689169	0.869188642	0.999978546
2.6.1.44	alanine-glyoxylate transaminase / (R)-3-amino-2-methylpropior	1.573124213	0.289161523	2.186243187	0.132264116	0.894775389	0.999978546
4.2.3.12	6-pyruvoyltetrahydropterin/6-carboxytetrahydropterin synthas	76.58751425	-0.18972892	0.376555652	-0.503853598	0.61436426	0.999978546
3.6.4.13	ATP-dependent helicase HrpA	966.7343647	0.092201972	0.268586068	0.343286503	0.731382934	0.999978546
1.1.1.29	glycerate dehydrogenase	185.3650309	-0.277450706	0.281672299	-0.985012396	0.324617982	0.999978546
4.2.1.83	4-oxalmesaconate hydratase	0.163338744	-0.667057198	4.14053197	-0.161104226	0.872011312	0.999978546
2.3.2.27	protein neuralized	0.3651991	-0.005017798	4.130362361	-0.001214857	0.999030685	0.999978546
2.7.7.72	tRNA nucleotidyltransferase (CCA-adding enzyme)	259.1690687	-0.133095926	0.250062181	-0.532251322	0.594551941	0.999978546
4.4.1.5	lactoylglutathione lyase	653.2741697	0.679321704	0.8708585	0.780059796	0.435355679	0.999978546
3.2.2.1	purine nucleosidase	22.68749659	-0.039221448	0.443188952	-0.088498252	0.929480673	0.999978546
2.1.1.192	23S rRNA (adenine2503-C2)-methyltransferase	414.0283495	0.082144057	0.142127697	0.577959528	0.563291444	0.999978546
1.4.1.4	glutamate dehydrogenase (NADP+)	1144.214867	-0.06611109	0.32770615	-0.201738937	0.840120821	0.999978546
2.7.1.58	2-dehydro-3-deoxygalactonokinase	3.687731951	-1.476204178	1.244012273	-1.1866476	0.235366652	0.999978546
1.1.1.275	(+)-trans-carveol dehydrogenase	0.063329795	0.094410257	4.143731151	0.022783876	0.98182267	0.999978546
1.5.3.1	sarcosine oxidase	20.45793132	0.985081082	0.461957847	2.132404695	0.032973592	0.999978546
2.5.1.77	#N/A	6.782457674	2.11560054	1.919219424	1.102323431	0.270321086	0.999978546
5.3.1.31	sulfoquinovose isomerase	0.144460652	-1.00194317	4.133461154	-0.24239811	0.808471699	0.999978546
2.7.4.24	inositol-hexakisphosphate/diphosphoinositol-pentakisphospha	0.247565813	-0.276629316	4.141286367	-0.06679792	0.946742579	0.999978546
4.2.99.18	DNA-(apurinic or apyrimidinic site) lyase	344.0121466	-0.098314804	0.337634821	-0.291186804	0.770908455	0.999978546
1.16.1.9	ferric-chelate reductase (NADPH)	0.354697033	-1.252004955	4.13215156	-0.302991054	0.76189668	0.999978546
3.2.1.172	unsaturated rhamnogalacturonyl hydrolase	260.0740849	0.69013513	0.498427841	1.384623958	0.166167482	0.999978546
2.4.2.8	hypoxanthine phosphoribosyltransferase	315.6346228	0.457610898	0.271254307	1.687017999	0.091599891	0.999978546
3.2.1.37	xylan 1,4-beta-xylosidase	254.56278	-0.277989225	0.316540522	-0.878210549	0.379829468	0.999978546
1.1.1.307	D-xylose reductase	0.089723976	-0.795848752	4.137904308	-0.192331357	0.847482657	0.999978546
1.1.1.219	dihydroflavonol-4-reductase	13.80100873	-0.157724506	0.622479765	-0.253380936	0.799973858	0.999978546
3.6.3.35	manganese/zinc transport system ATP-binding protein	1.137234124	-0.627384538	4.098890894	-0.153062024	0.878349363	0.999978546
1.7.2.4	nitrous-oxide reductase	0.044861988	-0.525974697	4.141284466	-0.127007623	0.898934365	0.999978546
1.7.7.1	ferredoxin-nitrite reductase	22.63867316	-0.543038899	0.678650928	-0.800174105	0.423609931	0.999978546
2.4.1.227	UDP-N-acetylglucosamine--N-acetylmuramyl-(pentapeptide) py	385.4145432	-0.046801263	0.15361195	-0.304672022	0.760615961	0.999978546
2.4.2.21	nicotinate-nucleotide--dimethylbenzimidazole phosphoribosylt	150.8245576	0.003128052	0.312456613	0.010011158	0.992012385	0.999978546
4.2.1.147	5,6,7,8-tetrahydromethanopterin hydro-lyase	11.36477334	0.608862045	1.081149092	0.563161963	0.573324601	0.999978546
2.3.1.1	amino-acid N-acetyltransferase	239.3172202	-0.077397755	0.287518545	-0.26919222	0.787781767	0.999978546
2.1.1.223	tRNA1Val (adenine37-N6)-methyltransferase	45.39681127	-0.084969676	0.534119974	-0.159083502	0.873603086	0.999978546
4.1.1.39	#N/A	1.506786979	-2.098833151	2.01905032	-1.039515029	0.298565272	0.999978546
2.5.1.105	7,8-dihydropterin-6-yl-methyl-4-(beta-D-ribofuranosyl)aminobe	51.1828228	-0.053568979	0.333982236	-0.160394695	0.872570168	0.999978546
1.1.1.189	carbonyl reductase 1	0.067578231	-0.344699102	4.140710721	-0.083246362	0.933655649	0.999978546
1.17.4.1	ribonucleotide reductase, class II	558.9808335	0.314123013	0.418583035	0.750443728	0.452987503	0.999978546
2.3.1.183	phosphinothricin acetyltransferase	388.6004679	-1.035359786	0.934695288	-1.107697662	0.267992409	0.999978546
6.3.2.9	UDP-N-acetylmuramoylalanine--D-glutamate ligase	386.6429284	-0.094852293	0.182072905	-0.520957759	0.602396198	0.999978546
6.3.2.17	folylpolyglutamate synthase	404.2567415	-0.064251767	0.221156746	-0.290525918	0.771413925	0.999978546
2.7.8.20	phosphoglycerol transferase	16.37875243	-0.258028254	0.761792215	-0.338712118	0.734826611	0.999978546
2.3.3.10	hydroxymethylglutaryl-CoA synthase	12.96787222	1.413374587	1.147040396	1.232192512	0.21787718	0.999978546

2.3.3.15	sulfoacetaldehyde acetyltransferase	10.93170915	-2.553562134	1.768273484	-1.444099093	0.148711103	0.999978546
3.2.1.28	alpha,alpha-trehalase	5.065846339	-0.786103631	0.952278338	-0.825497756	0.409089056	0.999978546
2.7.2.1	acetate kinase	846.1385794	-0.092317724	0.311510866	-0.296354748	0.76695918	0.999978546
3.5.1.44	chemotaxis protein CheD	77.03386573	-0.111458714	0.514083499	-0.216810527	0.828356008	0.999978546
5.1.99.6	NAD(P)H-hydrate epimerase	0.573429732	-2.323165966	4.118164768	-0.564126522	0.57266803	0.999978546
2.1.1.199	16S rRNA (cytosine1402-N4)-methyltransferase	1239.013886	-0.211127876	0.366675907	-0.575788789	0.564757952	0.999978546
3.7.1.14	2-hydroxy-6-oxonona-2,4-dienedioate hydrolase	0.417225619	-1.742109877	4.110073954	-0.42386339	0.671665445	0.999978546
2.7.2.-	#N/A	3.340273351	1.304920632	2.13806768	0.610327093	0.541645153	0.999978546
2.7.7.93	phosphonoformate cytidylyltransferase	0.291631102	2.765714695	4.077526781	0.678282411	0.497592649	0.999978546
1.13.11.49	chlorite dismutase	0.06577573	0.094416003	4.143731151	0.022785263	0.981821564	0.999978546
3.6.4.3	#N/A	0.214256324	0.094408575	4.143731151	0.02278347	0.981822993	0.999978546
3.4.24.71	endothelin-converting enzyme	0.134585963	-0.98543456	4.133791656	-0.238385154	0.811582381	0.999978546
2.6.1.98	UDP-2-acetamido-2-deoxy-ribo-hexuluronate aminotransferase	0.172699552	-1.383436073	4.120455997	-0.335748294	0.737060684	0.999978546
1.1.1.4	(R,R)-butanediol dehydrogenase / meso-butanediol dehydroger	5.627014574	0.210655915	1.677819523	0.125553382	0.900085467	0.999978546
1.8.1.4	dihydrolipoamide dehydrogenase	347.1311495	0.046202055	0.361077662	0.127956005	0.89818379	0.999978546
3.1.13.5	ribonuclease D	8.99875701	-2.75026712	1.07962013	-2.547439644	0.010851662	0.999978546
1.1.1.305	UDP-4-amino-4-deoxy-L-arabinose formyltransferase / UDP-glu	2.389353235	0.14330415	1.387919669	0.10325104	0.917763732	0.999978546
5.4.99.19	16S rRNA pseudouridine516 synthase	278.6387261	-0.576023825	0.241158471	-2.388569733	0.016914098	0.999978546
2.7.1.68	1-phosphatidylinositol-4-phosphate 5-kinase	0.256999344	-0.689455486	4.136675885	-0.166668965	0.867630527	0.999978546
1.1.1.60	2-hydroxy-3-oxopropionate reductase	30.15284766	0.398066322	0.730603305	0.544846046	0.585859407	0.999978546
2.7.1.198	PTS system, glucitol/sorbitol-specific IIA component	25.02906499	-0.55987845	1.012518356	-0.552956346	0.580293303	0.999978546
3.4.21.116	stage IV sporulation protein B	182.2910614	0.547702909	0.459678575	1.191491053	0.233460867	0.999978546
1.2.1.89	D-glyceraldehyde dehydrogenase (NADP+)	0.057984819	-0.266239077	4.143731151	-0.06425105	0.948770329	0.999978546
2.5.1.42	geranylgeranylglycerol-phosphate geranylgeranyltransferase	0.483729329	1.33691851	4.106016559	0.325599883	0.744727101	0.999978546
1.1.1.136	UDP-N-acetyl-D-glucosamine dehydrogenase	10.54149	0.610024302	0.675011164	0.90372476	0.366141367	0.999978546
2.5.1.128	N4-bis(aminopropyl)spermidine synthase	2.284826551	-0.914619115	2.634981342	-0.347106486	0.728511321	0.999978546
2.8.4.3	tRNA-2-methylthio-N6-dimethylallyladenosine synthase	451.3763314	0.132853797	0.152157853	0.873131388	0.382591467	0.999978546
3.2.1.40	alpha-L-rhamnosidase	406.0979955	-0.300096635	0.338984407	-0.885281532	0.376004808	0.999978546
1.1.1.381	3-hydroxy acid dehydrogenase / malonic semialdehyde reducta	0.590372679	0.396432575	4.081473448	0.097129769	0.92262334	0.999978546
3.5.2.7	imidazolonepropionase	167.9124087	-0.063895378	0.390942887	-0.163439163	0.87017267	0.999978546
5.1.99.4	alpha-methylacyl-CoA racemase	0.26814328	2.551125983	4.088751177	0.623937695	0.532668504	0.999978546
6.3.4.20	7-cyano-7-deazaguanine synthase	59.4077229	0.052721133	0.421835059	0.124980445	0.900539031	0.999978546
6.3.2.14	bifunctional isochorismate lyase / aryl carrier protein	1.81108835	-1.078225802	2.496819713	-0.43183967	0.665857945	0.999978546
1.4.3.19	glycine oxidase	0.087385198	-0.73664429	4.138042439	-0.178017577	0.858709172	0.999978546
3.1.3.8	3-phytase	0.021925243	0.094418168	4.143731151	0.022785785	0.981821147	0.999978546
2.4.1.34	1,3-beta-glucan synthase	0.304865092	-2.029895031	4.118839178	-0.492831826	0.622131421	0.999978546
3.1.11.2	exodeoxyribonuclease III	196.406447	-0.220758036	0.250526449	-0.881176566	0.378222261	0.999978546
2.1.1.33	tRNA (guanine-N7-)-methyltransferase	269.7709139	0.441501938	0.275087347	1.60495182	0.108504408	0.999978546
3.5.1.53	N-carbamoylputrescine amidase	98.58245507	-0.077362882	0.387451861	-0.199670951	0.841737934	0.999978546
1.9.3.1	#N/A	2.510583364	-0.437419659	2.531398051	-0.17279766	0.862810477	0.999978546
3.2.1.24	alpha-mannosidase	220.3015912	0.071620282	0.266123352	0.26912438	0.78783397	0.999978546
4.2.1.40	glucarate dehydratase	5.876213181	0.307432552	1.084703246	0.283425492	0.776850689	0.999978546
2.4.2.17	ATP phosphoribosyltransferase	161.4871201	0.127769807	0.243379721	0.524981317	0.599596179	0.999978546
1.2.7.3	#N/A	1651.051343	0.287544578	0.369901573	0.777354301	0.436949767	0.999978546
2.7.8.38	archaetidylserine synthase	1.483140351	3.082316894	4.036978998	0.763520666	0.445152942	0.999978546
2.4.2.1	#N/A	482.41952	0.004317648	0.226372698	0.019073182	0.984782725	0.999978546
1.3.99.26	phytoene desaturase	1.632348624	0.91736795	4.096641466	0.223931715	0.822810437	0.999978546
2.4.1.173	sterol 3beta-glucosyltransferase	0.102122587	1.526602962	4.11330392	0.371137896	0.710534827	0.999978546
2.7.1.73	inosine kinase	0.288921303	-1.341663909	4.128085902	-0.325008719	0.745174473	0.999978546
4.1.1.25	tyrosine decarboxylase	5.813486058	4.040136788	3.975781431	1.016186845	0.30954042	0.999978546
3.2.1.54	cyclomaltodextrinase / maltogenic alpha-amylase / neopullulan	0.261732976	-0.331874779	4.114293375	-0.080663859	0.93570928	0.999978546
2.3.1.242	KDO2-lipid IV(A) palmitoleoyltransferase	3.639820526	-5.194047515	2.858239193	-1.817219331	0.069183536	0.999978546
2.6.1.106	dTDP-3-amino-3,4,6-trideoxy-alpha-D-glucose transaminase	0.02470684	0.094418984	4.143731151	0.022785982	0.98182099	0.999978546
6.3.1.2	glutamine synthetase	942.2618412	-0.144115576	0.166164815	-0.867305009	0.385774913	0.999978546
6.3.2.3	glutathione synthase	0.92520644	-0.784858913	2.621120734	-0.299436383	0.764607105	0.999978546
6.3.2.39	aerobactin synthase	0.288921303	-1.341663909	4.128085902	-0.325008719	0.745174473	0.999978546
3.5.1.82	N-acyl-D-glutamate deacylase	0.031664897	0.094413212	4.143731151	0.022784589	0.981822101	0.999978546
5.1.1.4	proline racemase	4.45986566	0.092690755	0.985964251	0.094010259	0.925101007	0.999978546
2.7.1.60	N-acylmannosamine kinase	0.427661618	-0.111597643	4.107831108	-0.027167047	0.978326498	0.999978546
1.8.5.4	sulfide:quinone oxidoreductase	10.70375631	0.517603289	0.80202569	0.645369961	0.518687465	0.999978546
2.1.1.224	23S rRNA (adenine-C8)-methyltransferase	2.006073184	1.677342836	3.70727784	0.452445948	0.650947752	0.999978546
6.2.1.30	phenylacetate-CoA ligase	364.9902406	-0.130928443	0.31098492	-0.421012192	0.673746179	0.999978546
3.4.21.88	repressor LexA	513.2102101	-0.058117658	0.305570196	-0.190194131	0.84915701	0.999978546
2.7.1.89	thiamine kinase	0.342787921	-1.059741922	4.131987579	-0.256472678	0.797585874	0.999978546
2.4.1.64	alpha,alpha-trehalose phosphorylase	6.474905408	0.726637383	1.050324621	0.691821717	0.489049297	0.999978546
3.6.1.23	dUTP pyrophosphatase	305.483228	0.01634235	0.237429581	0.068830302	0.945124698	0.999978546
1.1.1.26	glyoxylate reductase	1.612855192	2.729529523	2.528545949	1.07948583	0.280371207	0.999978546
3.5.1.19	nicotinamidase	4.871855705	-0.397407227	1.393344813	-0.285218148	0.775477017	0.999978546
2.4.1.12	cellulose synthase (UDP-forming)	7.854470084	-0.741807332	0.984228059	-0.753694558	0.45103265	0.999978546
3.1.21.3	type I restriction enzyme, R subunit	1475.812021	0.060140453	0.255284323	0.235582243	0.813756853	0.999978546
2.1.1.189	23S rRNA (uracil747-C5)-methyltransferase	0.723122727	0.702593675	4.097880054	0.171452963	0.863867613	0.999978546
4.2.1.68	L-fuconate dehydratase	0.164310313	0.468290606	4.129568591	0.113399401	0.909713914	0.999978546
4.6.1.12	2-C-methyl-D-erythritol 2,4-cyclodiphosphate synthase	260.3833251	0.08836163	0.206731693	0.427421787	0.669072141	0.999978546
1.3.8.4	isovaleryl-CoA dehydrogenase	0.107128162	0.094412122	4.143731151	0.022784326	0.981822311	0.999978546
6.3.5.3	phosphoribosylformylglycinamidine synthase	1280.080832	-0.040279001	0.233108794	-0.172790568	0.862816051	0.999978546
3.2.1.45	glucosylceramidase	92.54275142	-0.403254499	0.558085405	-0.722567721	0.469945506	0.999978546
2.1.1.163	demethylmenaquinone methyltransferase / 2-methoxy-6-polyp	59.19781542	-0.255657624	0.38291209	-0.667666626	0.504346418	0.999978546
3.4.23.-	#N/A	81.85829158	-0.373126171	0.451652314	-0.826135855	0.408727031	0.999978546
1.1.1.339	dTDP-6-deoxy-L-talose 4-dehydrogenase (NAD+)	9.784419663	-0.45822196	0.590524045	-0.775958174	0.437773682	0.999978546
6.5.1.-	#N/A	57.77620169	0.899965038	0.852521842	1.055650417	0.291127958	0.999978546
1.5.5.-	#N/A	2.954894685	-2.16694979	1.656283595	-1.308320506	0.190764623	0.999978546
1.1.1.298	malonyl-CoA reductase / 3-hydroxypropionate dehydrogenase	0.051061294	0.971902104	4.129959009	0.235329722	0.813952828	0.999978546
2.7.1.72	streptomycin 6-kinase	0.107128162	0.094412122	4.143731151	0.022784326	0.981822311	0.999978546
5.4.99.28	tRNA pseudouridine32 synthase / 23S rRNA pseudouridine746 s	73.12648985	-0.301682926	0.37260146	-0.809666517	0.418131867	0.999978546
2.7.1.156	adenosylcobinamide kinase / adenosylcobinamide-phosphate g	169.949037	0.480292342	0.295313298	1.626382373	0.103868338	0.999978546
2.3.1.18	galactoside O-acetyltransferase	10.30660446	0.213655977	0.603517523	0.354017852	0.723325505	0.999978546
4.99.1.4	uroporphyrin-III C-methyltransferase / precorrin-2 dehydrogena	35.78655682	0.22537422	0.543505952	0.414667437	0.678385374	0.999978546

3.2.1.97	endo-alpha-N-acetylgalactosaminidase	151.0616393	0.522248303	0.624506687	0.836257344	0.403010145	0.999978546
4.1.1.87	malonate decarboxylase beta subunit	4.427733717	1.773367934	1.573608786	1.126943335	0.25976644	0.999978546
3.1.3.95	myotubularin	0.240510691	-0.396287166	4.138517643	-0.095755824	0.923714504	0.999978546
4.2.1.5	D-arabinonate dehydratase	2.260122362	0.171523326	2.440037409	0.070295367	0.94395857	0.999978546
1.2.99.7	aldehyde oxidoreductase	248.9810997	0.005055938	0.611558015	0.008267308	0.993403718	0.999978546
2.4.2.53	undecaprenyl-phosphate 4-deoxy-4-formamido-L-arabinose tra	32.80543789	0.425569099	0.507630521	0.838344192	0.401837423	0.999978546
1.1.1.173	L-rhamnose 1-dehydrogenase	0.031664897	0.094413212	4.143731151	0.022784589	0.981822101	0.999978546
1.3.7.11	digeranylgeranylglycerophospholipid reductase	9.718970903	0.519816564	1.424842256	0.364823939	0.715242834	0.999978546
2.7.1.169	pantoate kinase	2.104766928	3.827109884	2.777297798	1.377997666	0.168204009	0.999978546
3.6.1.40	exopolyphosphatase / guanosine-5'-triphosphate,3'-diphosphat	219.8727794	0.445123654	0.43060295	1.033721793	0.301266242	0.999978546
6.1.1.12	aspartyl-tRNA synthetase	693.8225422	0.051578802	0.168472787	0.306155096	0.75948656	0.999978546
3.5.1.3	omega-amidase	0.286407904	-1.300552973	4.134365097	-0.314571389	0.753087105	0.999978546
1.5.98.2	5,10-methylenetetrahydromethanopterin reductase	262.9153422	2.63101819	1.153006192	2.281876895	0.022496609	0.999978546
2.1.1.264	23S rRNA (guanine2445-N2)-methyltransferase / 23S rRNA (gua	1.561324307	-1.183420817	1.776978591	-0.665973593	0.505427979	0.999978546
1.1.1.282	quinate/shikimate dehydrogenase	0.314357693	0.09040591	4.123138511	0.021926479	0.982506603	0.999978546
3.1.3.37	fructose-1,6-bisphosphatase I / sedoheptulose-1,7-bisphosphat	0.301146343	-0.9114082	4.115642195	-0.221449814	0.824742209	0.999978546
2.4.2.10	orotate phosphoribosyltransferase	602.0547244	-0.058400366	0.266035422	-0.219521014	0.826244212	0.999978546
3.1.7.2	GTP diphosphokinase / guanosine-3',5'-bis(diphosphate) 3'-diph	2.493487671	-2.604127922	2.269571173	-1.147409675	0.251212345	0.999978546
3.5.4.10	phosphoribosylaminoimidazolecarboxamide formyltransferase	631.4865603	-0.060787903	0.213945413	-0.284128095	0.776312217	0.999978546
2.4.1.319	beta-1,4-mannooligosaccharide/beta-1,4-mannosyl-N-acetylglu	286.1595224	-0.295106564	0.426679348	-0.691635454	0.489166291	0.999978546
3.6.3.38	capsular polysaccharide transport system ATP-binding protein	2.063563325	-0.552757853	2.738183429	-0.201870279	0.840018138	0.999978546
2.3.1.169	#N/A	571.4431582	-0.47086992	0.584266196	-0.80591676	0.420290847	0.999978546
5.4.2.2	phosphoglucomutase	1276.024885	-0.083517624	0.275524813	-0.303121969	0.761796912	0.999978546
5.2.1.1	maleate isomerase	0.513448466	-0.229338269	4.120305671	-0.055660499	0.955612268	0.999978546
2.7.7.62	adenosylcobinamide kinase / adenosylcobinamide-phosphate g	172.204231	0.516823924	0.295340454	1.749925947	0.080131093	0.999978546
2.1.1.265	tellurite methyltransferase	1.446509712	-1.712999606	2.251205898	-0.760925337	0.446701664	0.999978546
3.5.1.25	N-acetylglucosamine-6-phosphate deacetylase	770.3948878	-0.016189787	0.400826511	-0.040391008	0.967781399	0.999978546
3.4.24.55	protease III	0.192614202	-1.143334171	4.13091893	-0.276774778	0.781953049	0.999978546
2.1.1.219	tRNA (adenine57-N1/adenine58-N1)-methyltransferase catalyti	4.213074941	1.914644368	1.706178205	1.122183112	0.261784592	0.999978546
4.1.3.4	hydroxymethylglutaryl-CoA lyase	5.591552884	-0.053185286	1.243762138	-0.042761622	0.965891557	0.999978546
1.1.5.4	malate dehydrogenase (quinone)	0.675139586	-1.977349745	4.124664277	-0.479396531	0.631656561	0.999978546
1.7.2.1	nitrite reductase (NO-forming)	0.06577573	0.094416003	4.143731151	0.022785263	0.981821564	0.999978546
4.1.1.47	tartronate-semialdehyde synthase	3.024359811	2.685887636	1.399241452	1.919531209	0.054917141	0.999978546
2.1.2.10	aminomethyltransferase	205.0394189	-0.008709273	0.309907014	-0.028102859	0.977580113	0.999978546
2.7.8.1	ethanolaminephosphotransferase	0.129559156	-0.240628205	4.142394674	-0.058089155	0.953677613	0.999978546
2.7.1.40	pyruvate kinase	620.5141539	-0.032587588	0.191281047	-0.170364959	0.864723128	0.999978546
2.7.1.11	6-phosphofructokinase 1	1549.328	0.085478821	0.251709584	0.339593034	0.734163024	0.999978546
6.1.1.21	#N/A	382.5298565	0.166704418	0.176356532	0.945269311	0.344521402	0.999978546
1.97.1.4	#N/A	3571.408187	1.194516482	0.524685621	2.276632775	0.022808164	0.999978546
6.3.2.13	UDP-N-acetylmuramoyl-L-alanyl-D-glutamate--2,6-diaminopime	592.4151967	0.058922522	0.14894293	0.395604691	0.692396676	0.999978546
3.2.2.24	ADP-ribosyl-[dinitrogen reductase] hydrolase	1.660127455	0.190542438	2.128010716	0.089540168	0.928652632	0.999978546
5.-.-.-	#N/A	209.3950617	-0.396301039	0.771097917	-0.513943859	0.607291242	0.999978546
3.2.2.28	double-stranded uracil-DNA glycosylase	0.132218179	0.066291295	4.142488731	0.01600277	0.987232182	0.999978546
2.7.1.4	fructokinase	447.9267559	-0.152749516	0.266339426	-0.573514473	0.566296403	0.999978546
2.7.1.14	sedoheptulokinase	8.305635679	-0.247786856	0.873562077	-0.283651114	0.776677762	0.999978546
1.11.1.-	#N/A	2.773257521	1.665077695	2.092562048	0.79571246	0.426199183	0.999978546
2.3.1.4	glucosamine-phosphate N-acetyltransferase	0.549114054	-0.244853893	4.10700397	-0.059618616	0.952459391	0.999978546
4.1.1.11	aspartate 1-decarboxylase	77.14565317	-0.339717766	0.298584842	-1.137759586	0.255220885	0.999978546
3.4.21.81	streptogrisin B	0.59175187	-1.417555539	4.131973232	-0.343069875	0.731545894	0.999978546
2.7.1.165	glycerate 2-kinase	139.7135454	-0.111671165	0.316986791	-0.352289648	0.724621051	0.999978546
1.2.1.87	propionaldehyde dehydrogenase	211.2777077	0.15136595	0.559046158	0.270757518	0.786577535	0.999978546
2.8.3.10	#N/A	110.095947	0.483131381	0.387017443	1.248345235	0.211904655	0.999978546
4.4.1.14	1-aminocyclopropane-1-carboxylate synthase	0.438278362	0.012788957	4.140615125	0.003088661	0.997535609	0.999978546
1.3.1.2	dihydropyrimidine dehydrogenase (NADP+)	0.358716976	-0.856987566	4.134033304	-0.207300595	0.835775109	0.999978546
3.10.1.1	N-sulfoglucosamine sulfohydrolase	0.192614202	-1.143334171	4.13091893	-0.276774778	0.781953049	0.999978546
2.7.1.51	L-fuculokinase	5.794631649	-0.84773574	1.265165265	-0.670059291	0.502819995	0.999978546
4.1.3.44	tRNA wybutosine-synthesizing protein 1	3.612380128	3.393318152	3.048771225	1.113011735	0.265703389	0.999978546
1.1.1.30	3-hydroxybutyrate dehydrogenase	0.609117398	-1.481463375	4.132370315	-0.358502085	0.719967608	0.999978546
5.4.99.20	23S rRNA pseudouridine2457 synthase	1.635705765	-0.062709818	1.680568897	-0.037314637	0.970234135	0.999978546
2.4.99.16	starch synthase (maltosyl-transferring)	2.015113309	-1.287724931	2.465916906	-0.522209377	0.601524558	0.999978546
2.7.4.28	[pyruvate, water dikinase]-phosphate phosphotransferase / [py	11.94070215	-0.948567941	0.778185263	-1.218948733	0.222863651	0.999978546
3.4.23.51	hydrogenase 3 maturation protease	3.847375802	-0.125965103	1.443427646	-0.087268041	0.930458457	0.999978546
2.7.1.208	PTS system, alpha-glucoside-specific IIB component	45.94357866	-0.361425877	0.552566761	-0.654085446	0.513056757	0.999978546
1.6.2.4	NADPH-ferrihemoprotein reductase	0.267820405	0.094407394	4.143731151	0.022783185	0.981823221	0.999978546
6.6.1.1	magnesium chelatase subunit H	18.92594321	0.254197005	0.844000863	0.301180977	0.763276495	0.999978546
1.1.1.102	3-dehydrosphinganine reductase	0.428512649	0.094404856	4.143731151	0.022782573	0.981823709	0.999978546
3.6.3.8	Ca2+-transporting ATPase	547.2942701	-0.04639194	0.204217437	-0.227169338	0.820292069	0.999978546
1.1.1.36	acetoacetyl-CoA reductase	0.635026121	-1.178855154	4.132220567	-0.285283696	0.775426803	0.999978546
2.4.1.290	N,N'-diacetylbacillosaminyl-diphospho-undecaprenol alpha-1,3-	0.063329795	0.094410257	4.143731151	0.022783876	0.98182267	0.999978546
3.1.3.11	fructose-1,6-bisphosphatase I / sedoheptulose-1,7-bisphosphat	417.2305742	-0.037532174	0.214405004	-0.175052697	0.861038227	0.999978546
6.1.1.2	tryptophanyl-tRNA synthetase	437.2335089	0.003125973	0.207193673	0.015087205	0.987962609	0.999978546
1.1.1.79	glyoxylate/hydroxypyruvate reductase	2.265448947	-0.257708347	3.067907083	-0.084001353	0.933055356	0.999978546
6.3.2.34	coenzyme F420-0:L-glutamate ligase / coenzyme F420-1:gamm	4.270530574	1.997952806	1.769189265	1.129304165	0.258769544	0.999978546
1.1.1.374	UDP-N-acetylglucosamine 3-dehydrogenase	15.70625512	2.740502687	2.539176033	1.079288183	0.280459279	0.999978546
2.4.1.21	starch synthase	1036.64266	0.04759841	0.375832158	0.12664805	0.899218965	0.999978546
2.3.2.3	phosphatidylglycerol lysyltransferase	8.777805559	-2.750751316	1.241175921	-2.216246117	0.026674651	0.999978546
3.2.1.58	glucan 1,3-beta-glucosidase	2.050744339	-2.024358028	2.287726431	-0.884877667	0.376222614	0.999978546
1.1.1.81	glyoxylate/hydroxypyruvate reductase	77.79447013	0.5363528	0.378766665	1.416050698	0.156760661	0.999978546
2.7.11.25	mitogen-activated protein kinase kinase kinase 8	0.2108833	-0.266249222	4.143731151	-0.064253498	0.94876838	0.999978546
1.8.99.5	dissimilatory sulfite reductase alpha subunit	158.2360549	0.221464961	0.786409788	0.281615214	0.778238573	0.999978546
2.1.1.63	methylated-DNA-protein]-cysteine S-methyltransferase	145.0292668	-0.304899832	0.396900964	-0.768201287	0.442367609	0.999978546
4.2.99.21	salicylate synthetase	0.795163648	-1.625407729	4.116955723	-0.394808164	0.69298447	0.999978546
2.4.1.288	galactofuranosylgalactofuranosylrhamnosyl-N-acetylglucosamir	0.791497509	-0.951159579	4.116669662	-0.231050742	0.817275382	0.999978546
2.4.2.14	amidophosphoribosyltransferase	674.2738087	-0.184446041	0.196989741	-0.936323078	0.349106869	0.999978546
2.7.7.39	glycerol-3-phosphate cytidylyltransferase	6.785358283	-1.261635771	1.201972868	-1.049637479	0.293884819	0.999978546
4.2.2.10	pectin lyase	22.34609549	-2.072344419	2.176682336	-0.952065621	0.341063703	0.999978546
1.3.5.1	#N/A	497.6955786	-0.196300863	0.234105134	-0.838515839	0.401741056	0.999978546

4.2.1.6	galactonate dehydratase	27.36780082	0.173573446	0.46453007	0.373653843	0.70866187	0.999978546
4.3.2.1	argininosuccinate lyase	310.1718153	0.204264098	0.203610924	1.003207954	0.315760536	0.999978546
6.1.2.1	D-alanine---(R)-lactate ligase	0.157319219	-0.266247166	4.143731151	-0.064253002	0.948768775	0.999978546
2.2.1.6	#N/A	1048.382309	0.013978456	0.214912054	0.065042679	0.948140019	0.999978546
2.3.1.102	acetyl CoA:N6-hydroxylysine acetyl transferase	0.192614202	-1.143334171	4.13091893	-0.276774778	0.781953049	0.999978546
2.6.1.17	acetylornithine/N-succinyldiaminopimelate aminotransferase	610.7636172	-0.303814694	0.546052689	-0.556383478	0.577948731	0.999978546
3.4.24.15	thimet oligopeptidase	1.127149497	0.442471551	2.678901525	0.165169024	0.868810946	0.999978546
3.1.1.31	6-phosphogluconolactonase	234.1506305	0.22824066	0.320883834	0.711287499	0.476906097	0.999978546
3.6.1.31	phosphoribosyl-ATP pyrophosphohydrolase	255.6035093	-0.087932771	0.238707507	-0.368370363	0.712597093	0.999978546
1.20.4.1	arsenate reductase (glutaredoxin)	97.46378776	-0.580830263	0.605437603	-0.959356108	0.337379378	0.999978546
2.7.7.60	2-C-methyl-D-erythritol 4-phosphate cytidylyltransferase	364.2618499	0.109711824	0.15750156	0.696576111	0.48606811	0.999978546
2.7.7.3	pantetheine-phosphate adenylyltransferase	482.6370214	-0.301507111	0.401377642	-0.751180633	0.452543954	0.999978546
2.1.3.6	putrescine carbamoyltransferase	14.52936115	-0.297248218	0.747897498	-0.397445129	0.691039242	0.999978546
3.6.1.1	inorganic pyrophosphatase	704.5610825	-0.091387319	0.20230363	-0.45173346	0.651461011	0.999978546
2.1.1.107	uroporphyrin-III C-methyltransferase	91.90494531	-0.025375323	0.305133233	-0.083161452	0.933723164	0.999978546
1.1.1.337	L-2-hydroxycarboxylate dehydrogenase (NAD+)	6.338592059	1.353742129	1.604337157	0.843801518	0.398780327	0.999978546
3.2.1.143	poly(ADP-ribose) glycohydrolase	0.214256324	0.094408575	4.143731151	0.02278347	0.981822993	0.999978546
1.1.1.42	isocitrate dehydrogenase	416.2986	0.240586191	0.252291055	0.953605709	0.340283265	0.999978546
4.2.1.106	bile-acid 7alpha-dehydratase	6.338790237	0.665738628	1.103419892	0.603341151	0.546281763	0.999978546
3.1.11.3	exodeoxyribonuclease (lambda-induced)	0.5133427	-0.065720222	4.132135831	-0.015904662	0.987310451	0.999978546
2.7.7.18	nicotinate-nucleotide adenylyltransferase	260.0595274	-0.006088813	0.186687106	-0.032615071	0.973981551	0.999978546
2.8.3.9	acetate CoA/acetoacetate CoA-transferase alpha subunit	155.1818872	0.179899883	0.595082685	0.302310734	0.762415197	0.999978546
4.1.1.1	pyruvate decarboxylase	3.712525794	-3.704550973	2.925253796	-1.26640327	0.205368741	0.999978546
3.6.4.7	peroxisome-assembly ATPase	0.267820405	0.094407394	4.143731151	0.022783185	0.981823221	0.999978546
1.14.-.-	#N/A	13.38369178	0.248858277	0.61716992	0.403224897	0.686782786	0.999978546
2.7.1.35	pyridoxine kinase	246.3550928	0.265651888	0.29846795	0.89005164	0.373438159	0.999978546
1.1.1.289	sorbose reductase	0.33823785	0.962178147	4.090711131	0.235210484	0.814045369	0.999978546
3.2.1.135	cyclomaltodextrinase / maltogenic alpha-amylase / neopullulan	0.261732976	-0.331874779	4.114293375	-0.080663859	0.93570928	0.999978546
1.1.1.370	scyllo-inositol 2-dehydrogenase (NAD+)	0.193874331	-0.951258034	4.135954034	-0.229997245	0.81809391	0.999978546
3.2.1.1	alpha-amylase	516.5780335	0.476243402	0.433397754	1.098859876	0.271829191	0.999978546
5.1.3.20	ADP-L-glycero-D-manno-heptose 6-epimerase	13.58974453	-0.247156361	0.530395744	-0.465984811	0.641226373	0.999978546
2.-.-.-	#N/A	311.2391915	0.115277776	0.270032464	0.426903397	0.669449691	0.999978546
3.2.1.96	mannosyl-glycoprotein endo-beta-N-acetylglucosaminidase	29.7488792	-0.061635753	1.009410521	-0.061061136	0.95131052	0.999978546
5.1.3.5	UDP-arabinose 4-epimerase	0.840902598	-0.797807258	4.096437672	-0.194756352	0.845583694	0.999978546
2.7.1.200	PTS system, galactitol-specific IIA component	14.49931809	-0.865737265	0.797325919	-1.08580098	0.277567053	0.999978546
3.6.1.-	#N/A	427.5886094	-0.320674617	0.380006747	-0.843865587	0.39874452	0.999978546
2.7.1.90	diphosphate-dependent phosphofructokinase	118.196786	-0.164795698	0.49670471	-0.331778006	0.740056893	0.999978546
5.3.1.27	6-phospho-3-hexuloisomerase	17.12641049	-0.114643654	0.865657391	-0.132435367	0.894639942	0.999978546
3.2.1.82	exo-poly-alpha-galacturonosidase	14.97042183	-0.155762411	1.285994253	-0.121122167	0.903594272	0.999978546
5.3.1.4	L-arabinose isomerase	323.6098938	-0.010836155	0.296851776	-0.03650359	0.970880816	0.999978546
2.3.1.40	acyl-[acyl-carrier-protein]-phospholipid O-acyltransferase / long	12.07765451	-0.880231869	0.887657579	-0.991634487	0.32137586	0.999978546
2.4.1.282	3-O-alpha-D-glucosyl-L-rhamnose phosphorylase	0.89434512	-0.931962467	4.026902334	-0.231434087	0.816977586	0.999978546
2.1.1.113	site-specific DNA-methyltransferase (cytosine-N4-specific)	0.436862061	0.219071476	4.109383762	0.053310055	0.957484869	0.999978546
2.7.7.9	UTP--glucose-1-phosphate uridylyltransferase	181.7226964	0.39111865	0.350658141	1.115384486	0.264685695	0.999978546
3.1.2.21	medium-chain acyl-[acyl-carrier-protein] hydrolase	1.936026993	-0.928911822	1.720980423	-0.539757344	0.589364388	0.999978546
0.0.0.0	#N/A	2720696.047	-0.305061943	0.838364487	-0.363877464	0.715949514	0.999978546
2.7.7.89	[glutamine synthetase] adenylyltransferase / [glutamine synthe	8.353306205	-0.14483586	0.989410073	-0.146386078	0.883616617	0.999978546
4.2.1.129	squalene-hopene/tetraprenyl-beta-curcumene cyclase	6.587769184	-0.91600028	1.466517989	-0.624608963	0.532227736	0.999978546
4.1.1.18	lysine decarboxylase	7.01613773	0.704671989	1.13142444	0.622818426	0.533403848	0.999978546
5.1.3.2	UDP-glucose 4-epimerase	831.185721	-0.386842928	0.283145984	-1.366231378	0.171866352	0.999978546
3.1.26.5	ribonuclease P/MRP protein subunit POP1	320.5620884	0.265277173	0.340986298	0.777970183	0.436586591	0.999978546
6.3.2.45	UDP-N-acetylmuramate: L-alanyl-gamma-D-glutamyl-meso-diar	13.23918897	-0.58481978	0.733180869	-0.797647353	0.425075163	0.999978546
2.1.1.171	16S rRNA (guanine966-N2)-methyltransferase	16.15378857	-0.63313895	0.787775187	-0.803705118	0.421567297	0.999978546
2.5.1.7	UDP-N-acetylglucosamine 1-carboxyvinyltransferase	756.9220733	0.008656951	0.138033648	0.062716239	0.949992466	0.999978546
2.3.1.31	homoserine O-acetyltransferase/O-succinyltransferase	20.97808603	0.895701944	0.713118754	1.25603476	0.209103368	0.999978546
2.7.1.181	O-antigen chain-terminating methyltransferase	0.321384486	0.094406417	4.143731151	0.022782949	0.981823409	0.999978546
4.1.1.98	4-hydroxy-3-polyprenylbenzoate decarboxylase	42.84468263	-0.219020936	0.429490773	-0.509954928	0.610083039	0.999978546
2.1.1.144	trans-aconitate 2-methyltransferase	4.543822609	-0.73636349	1.10589166	-0.665854999	0.505503787	0.999978546
1.1.1.261	glycerol-1-phosphate dehydrogenase [NAD(P)+]	117.8868652	0.377931099	0.362668621	1.042083811	0.297372823	0.999978546
3.2.2.30	adenosylhomocysteine/aminodeoxyfutalosine nucleosidase	0.072907776	0.991276683	4.130592014	0.239984167	0.810342531	0.999978546
1.21.98.1	cyclic dehypoxanthinyl futalosine synthase	8.600599234	-1.20286717	0.852704367	-1.410649712	0.158347926	0.999978546
1.6.5.3	#N/A	1987.932707	-0.152549669	0.414769954	-0.367793441	0.71302726	0.999978546
1.3.1.78	arogenate dehydrogenase (NADP+)	0.124335172	1.245115371	4.100479329	0.303651176	0.761393658	0.999978546
6.3.5.7	aspartyl-tRNA(Asn)/glutamyl-tRNA(Gln) amidotransferase subu	513.5161994	-0.047956503	0.277023152	-0.173113701	0.862562056	0.999978546
2.6.1.22	4-aminobutyrate aminotransferase / (S)-3-amino-2-methylprop	21.40539401	0.911606109	0.578870782	1.574800695	0.115302456	0.999978546
3.1.1.75	poly(3-hydroxybutyrate) depolymerase	0.153183881	1.800672354	4.103419624	0.438822377	0.660790245	0.999978546
2.1.1.151	precorrin-2/cobalt-factor-2 C20-methyltransferase	67.37692647	0.322052295	0.516037335	0.624087199	0.53257032	0.999978546
6.3.4.23	5-formaminoimidazole-4-carboxamide-1-(beta)-D-ribofuranosy	19.3794777	2.832698138	1.496138718	1.893339236	0.058312771	0.999978546
6.3.4.18	5-(carboxyamino)imidazole ribonucleotide synthase	20.71168242	-0.579178958	0.773912124	-0.748378194	0.454232072	0.999978546
3.2.1.85	6-phospho-beta-galactosidase	2.516302815	-0.367006507	1.405918637	-0.261043916	0.794058641	0.999978546
2.8.1.13	tRNA-uridine 2-sulfurtransferase	432.6097358	0.104016144	0.224797904	0.46270958	0.643572551	0.999978546
4.2.3.3	methylglyoxal synthase	416.339376	-0.156508871	0.270514685	-0.578559612	0.562886363	0.999978546
4.3.1.17	L-serine dehydratase	414.1108953	0.090094705	0.185594389	0.485438732	0.627365163	0.999978546
2.3.1.86	fatty acid synthase subunit alpha, fungi type	0.01747704	-0.22961245	4.141706174	-0.055439097	0.955788649	0.999978546
6.3.3.2	glutamate formiminotransferase / 5-formyltetrahydrofolate cyc	206.2840431	-0.541898507	0.279896069	-1.936070446	0.052859074	0.999978546
5.3.3.8	3-hydroxyacyl-CoA dehydrogenase / enoyl-CoA hydratase / 3-h	0.306876842	-1.264787838	4.129323875	-0.306294172	0.759380676	0.999978546
3.5.3.1	arginase	30.489841	0.321071862	0.455540583	0.704815057	0.480925337	0.999978546
3.4.21.68	tissue plasminogen activator	0.059587395	1.425432941	4.11370627	0.346508197	0.728960825	0.999978546
4.1.3.27	anthranilate synthase / indole-3-glycerol phosphate synthase	257.4550298	-0.164490933	0.406386821	-0.404764439	0.68565067	0.999978546
6.1.1.17	glutamyl-tRNA synthetase	500.0146009	-0.042985654	0.155172644	-0.277018249	0.781766094	0.999978546
6.2.1.17	propionyl-CoA synthetase	0.186031077	-1.133155041	4.131085887	-0.27429956	0.783854423	0.999978546
3.4.23.43	leader peptidase (prepilin peptidase) / N-methyltransferase	211.7759288	-0.149634711	0.43735087	-0.342138821	0.732246424	0.999978546
3.5.1.6	beta-ureidopropionase	61.32383993	0.030738116	0.46153974	0.066599066	0.946900889	0.999978546
2.1.1.197	malonyl-CoA O-methyltransferase	27.782404	-0.634492544	0.617156213	-1.028090668	0.30390718	0.999978546
4.1.99.19	2-iminoacetate synthase	227.5228568	-0.043698129	0.273843866	-0.159573152	0.87321733	0.999978546
3.6.1.27	phosphatidylglycerophosphatase B	477.1536917	-0.016864224	0.230134654	-0.07327981	0.941583457	0.999978546
3.5.3.12	agmatine deiminase	81.72245933	-0.340204272	0.344425236	-0.987744905	0.323277596	0.999978546

2.8.3.17	cinnamoyl-CoA:phenyllactate CoA-transferase	1.036084739	-2.169287454	2.753024941	-0.787965057	0.430717145	0.999978546
1.7.1.15	nitrite reductase (NADH) large subunit	2.283221944	0.136957995	2.077340229	0.065929496	0.947433957	0.999978546
4.1.3.17	4-hydroxy-4-methyl-2-oxoglutarate aldolase	0.768386619	-0.658111941	3.616477402	-0.181975958	0.855601593	0.999978546
2.7.7.67	CDP-2,3-bis-(O-geranylgeranyl)-sn-glycerol synthase	1.61179286	1.455147721	2.489842519	0.584433638	0.558928601	0.999978546
2.5.1.1	farnesyl diphosphate synthase	408.1672705	0.397852517	0.201468949	1.974758484	0.048295562	0.999978546
1.14.13.127	3-(3-hydroxy-phenyl)propionate hydroxylase	0.384912947	-0.127974764	4.103474428	-0.031186928	0.975120464	0.999978546
5.3.1.23	methylthioribose-1-phosphate isomerase	98.34085415	-0.256906021	0.278514955	-0.922413739	0.356312806	0.999978546
6.3.4.24	tyramine---L-glutamate ligase	2.341597874	2.458900814	3.007553815	0.817575001	0.413599907	0.999978546
1.2.1.41	glutamate-5-semialdehyde dehydrogenase	367.9436807	-0.300871962	0.164040054	-1.834137177	0.066633634	0.999978546
4.2.1.44	inosose dehydratase	34.21941793	0.464280682	0.389835679	1.190965084	0.233667292	0.999978546
3.5.4.13	dCTP deaminase	6.58030804	-0.632783337	1.088473378	-0.581349392	0.561004998	0.999978546
2.1.1.228	tRNA (guanine37-N1)-methyltransferase	306.555681	-0.193568197	0.371666014	-0.520812207	0.602497599	0.999978546
1.1.1.17	mannitol-1-phosphate 5-dehydrogenase	23.22468614	-0.472056663	0.591166842	-0.798516814	0.42457064	0.999978546
1.2.1.16	succinate-semialdehyde dehydrogenase / glutarate-semialdehy	8.439430765	0.18247186	1.099333513	0.165984079	0.86816948	0.999978546
3.1.4.46	glycerophosphoryl diester phosphodiesterase	340.9449883	0.178283696	0.235762228	0.75620127	0.449528532	0.999978546
1.1.1.88	hydroxymethylglutaryl-CoA reductase	1.877515391	-0.395394193	2.466209755	-0.160324641	0.872625349	0.999978546
3.5.4.19	phosphoribosyl-AMP cyclohydrolase	246.2107743	-0.11421213	0.232163296	-0.491947398	0.62275653	0.999978546
4.1.1.83	4-hydroxyphenylacetate decarboxylase large subunit	33.23974627	0.871184797	0.935436127	0.931314038	0.351691142	0.999978546
1.2.1.12	glyceraldehyde 3-phosphate dehydrogenase	2889.827608	0.056070649	0.568736077	0.09858817	0.921465264	0.999978546
3.6.1.21	ADP-sugar diphosphatase	0.107128162	0.094412122	4.143731151	0.022784326	0.981822311	0.999978546
6.5.1.3	RNA-editing ligase	36.42033204	-0.716261541	0.627495342	-1.141461128	0.253678079	0.999978546
1.3.8.7	acyl-CoA dehydrogenase	0.861133089	0.505322189	3.078848822	0.16412699	0.869631175	0.999978546
3.4.24.78	spore protease	139.5273193	-0.091541856	0.585619634	-0.156316234	0.87578376	0.999978546
3.1.3.2	acid phosphatase	13.24700897	0.741613798	0.706088	1.050313555	0.293573975	0.999978546
3.2.2.20	DNA-3-methyladenine glycosylase I	32.62903189	-0.845105431	0.519347257	-1.627245392	0.103684991	0.999978546
3.5.4.25	GTP cyclohydrolase II	303.6077484	0.101830679	0.247824113	0.410898993	0.681146601	0.999978546
2.4.1.16	chitin synthase	0.299092876	-0.944431454	4.140575192	-0.22809185	0.819574836	0.999978546
3.4.22.40	bleomycin hydrolase	186.1108036	0.119357347	0.283766745	0.420617811	0.674034186	0.999978546
2.7.8.13	phospho-N-acetylmuramoyl-pentapeptide-transferase	372.9500552	-0.035643657	0.225423775	-0.158118447	0.874363465	0.999978546
1.1.3.48	3-deoxy-alpha-D-manno-octulosonate 8-oxidase	3.333179177	1.092425579	1.461903072	0.74726266	0.454905027	0.999978546
1.1.1.49	glucose-6-phosphate 1-dehydrogenase	223.5508127	0.310436066	0.471113507	0.658941129	0.509933574	0.999978546
5.1.3.15	glucose-6-phosphate 1-epimerase	2.900916458	-0.312256133	1.395249298	-0.223799527	0.822913298	0.999978546
2.7.4.23	ribose 1,5-bisphosphokinase	0.173198025	0.166073128	4.137543803	0.040138095	0.967983031	0.999978546
3.1.3.4	phosphatidate phosphatase	1.343840583	1.077711734	4.127329972	0.261115961	0.794003083	0.999978546
5.2.1.-	#N/A	1.891810036	1.082998929	1.731397313	0.625505723	0.531639193	0.999978546
4.2.1.53	oleate hydratase	635.5203128	-0.305333808	0.555542256	-0.549614012	0.582584146	0.999978546
5.1.1.1	alanine racemase	456.0159821	0.021747821	0.221522176	0.098174464	0.921793759	0.999978546
5.4.99.24	23S rRNA pseudouridine955/2504/2580 synthase	208.4356302	-0.395491774	0.376297776	-1.05100747	0.293255159	0.999978546
1.1.1.95	D-3-phosphoglycerate dehydrogenase / 2-oxoglutarate reducta	733.0911774	0.009358824	0.271164284	0.034513484	0.97246769	0.999978546
3.4.21.26	prolyl oligopeptidase	54.99110323	-0.681340646	0.430117483	-1.584080332	0.113175439	0.999978546
4.2.1.159	dTDP-4-dehydro-6-deoxy-alpha-D-glucopyranose 2,3-dehydrata	6.32428671	-1.401204783	1.321601068	-1.060232787	0.289038708	0.999978546
3.4.24.84	STE24 endopeptidase	4.521324759	0.46846897	1.397198468	0.335291643	0.737405097	0.999978546
2.7.8.41	cardiolipin synthase (CMP-forming)	51.9006002	-0.049046579	0.527424373	-0.092992628	0.925909418	0.999978546
2.7.8.15	UDP-N-acetylglucosamine--dolichyl-phosphate N-acetylglucosa	2.302647437	2.125651016	2.41511739	0.880143973	0.37878132	0.999978546
3.1.3.45	3-deoxy-D-manno-octulosonate 8-phosphate phosphatase (KD(	63.9288138	0.135073572	0.344965995	0.391556195	0.69538616	0.999978546
2.4.1.212	hyaluronan synthase	0.302144354	-0.718301109	4.133161701	-0.173789743	0.862030706	0.999978546
2.4.2.44	5'-methylthioinosine phosphorylase	0.115969639	-0.637008364	4.143261082	-0.153745649	0.87781029	0.999978546
3.1.4.55	phosphoribosyl 1,2-cyclic phosphate phosphodiesterase	94.63300165	-0.400946913	0.34891706	-1.14911811	0.250507282	0.999978546
1.10.3.14	#N/A	311.3418992	-0.166494384	0.263460321	-0.631952409	0.527417976	0.999978546
6.3.4.13	phosphoribosylamine---glycine ligase	540.7811406	-0.10008659	0.21224978	-0.471550971	0.637247329	0.999978546
2.7.1.22	HTH-type transcriptional regulator, transcriptional repressor of	2.053440714	-0.074302525	1.958536172	-0.037937785	0.969737286	0.999978546
1.1.1.1	alcohol dehydrogenase	622.5140963	0.407046412	0.442298096	0.920298812	0.35741663	0.999978546
4.2.1.120	4-hydroxybutyryl-CoA dehydratase / vinylacetyl-CoA-Delta-ison	61.11807847	0.746491611	0.403211029	1.851367043	0.064116767	0.999978546
5.3.1.1	triosephosphate isomerase (TIM)	914.7994511	0.019009464	0.449455816	0.0422944	0.966264009	0.999978546
1.2.1.58	phenylglyoxylate dehydrogenase alpha subunit	1.250377898	2.053824064	2.80336006	0.732629423	0.46378448	0.999978546
2.7.1.78	bifunctional polynucleotide phosphatase/kinase	0.228120967	0.831630387	4.140581219	0.200848708	0.840816876	0.999978546
4.4.1.19	phosphosulfolactate synthase	2.261025615	3.905995829	3.377423666	1.156501587	0.247476051	0.999978546
3.4.19.12	ubiquitin carboxyl-terminal hydrolase MINDY-1/2	2.089260676	-1.554515507	3.440980068	-0.451765333	0.651438046	0.999978546
1.6.5.5	NADPH2:quinone reductase	0.160692243	0.094410072	4.143731151	0.022783831	0.981822705	0.999978546
5.4.99.-	#N/A	11.3310429	2.652560108	2.777763142	0.954926671	0.339614781	0.999978546
6.1.1.10	methionyl-tRNA synthetase	1072.529682	0.604801254	0.375616361	1.610156844	0.10736362	0.999978546
6.2.1.1	acetyl-CoA synthetase	231.4799934	0.028604349	0.372004233	0.076892537	0.938709034	0.999978546
3.2.1.35	hyaluronoglucosaminidase	245.6207223	-0.068081702	0.448759189	-0.151710992	0.879414887	0.999978546
1.3.1.31	2-enoate reductase	51.06122705	0.409883153	0.664282502	0.617031387	0.537214024	0.999978546
2.7.7.85	diadenylate cyclase	239.3174528	0.095607779	0.224740614	0.425413889	0.670534983	0.999978546
2.5.1.55	2-dehydro-3-deoxyphosphooctonate aldolase (KDO 8-P synthas	74.66958636	-0.499992855	0.37027831	-1.350316347	0.176914531	0.999978546
3.6.1.45	UDP-sugar diphosphatase	148.4862257	-0.135044642	0.446875592	-0.3021974	0.762501586	0.999978546
1.5.5.1	electron-transferring-flavoprotein dehydrogenase	0.060694436	-0.452769838	4.140751781	-0.109344839	0.912928983	0.999978546
2.7.4.-	#N/A	277.2844787	-0.029508604	0.170735628	-0.172832142	0.862783371	0.999978546
6.3.2.5	phosphopantothenoylcysteine decarboxylase / phosphopantot	280.6301022	-0.091876747	0.150746243	-0.609479513	0.542206648	0.999978546
4.2.1.1	carbonic anhydrase	85.65558449	0.530482201	0.403022696	1.316258876	0.188087169	0.999978546
4.2.1.119	(3R)-3-hydroxyacyl-CoA dehydrogenase / 3a,7a,12a-trihydroxy-	0.498402437	0.5259576	4.098259219	0.128336831	0.897882419	0.999978546
2.7.11.-	#N/A	276.9316338	-0.033148492	0.16889813	-0.196263225	0.844404144	0.999978546
2.7.7.15	choline-phosphate cytidylyltransferase	6.623524635	0.072708062	0.752596329	0.096609643	0.923036399	0.999978546
4.1.2.53	2-dehydro-3-deoxy-L-rhamnonate aldolase	0.443198042	-2.047212759	4.118926251	-0.497025835	0.619170833	0.999978546
2.3.1.191	UDP-3-O-[3-hydroxymyristoyl] glucosamine N-acyltransferase	125.04161	-0.214909865	0.306456518	-0.701273598	0.483132287	0.999978546
3.4.21.53	ATP-dependent Lon protease	915.8824061	-0.053571311	0.140983357	-0.37998323	0.703957864	0.999978546
2.7.1.63	polyphosphate glucokinase	3.198360875	0.249412645	2.292367241	0.108801348	0.913360055	0.999978546
4.1.1.50	S-adenosylmethionine decarboxylase	23.5955198	-1.082473966	0.6695296	-1.616767901	0.105928391	0.999978546
2.4.1.187	N-acetylglucosaminyldiphosphoundecaprenol N-acetyl-beta-D-(	105.0075222	-0.058803158	0.192882056	-0.304865881	0.760468303	0.999978546
6.3.1.20	lipoate---protein ligase	88.43111534	-0.355269818	0.451119692	-0.787528951	0.430972287	0.999978546
2.7.7.38	3-deoxy-manno-octulosonate cytidylyltransferase (CMP-KDO sy	95.53006735	-0.503067289	0.316851658	-1.587706032	0.112352828	0.999978546
2.4.1.279	nigerose phosphorylase	0.207493739	-0.137238807	4.132120914	-0.033212679	0.973504987	0.999978546
1.3.99.16	isoquinoline 1-oxidoreductase	0.043850487	0.094417027	4.143731151	0.02278551	0.981821367	0.999978546
3.1.1.85	pimeloyl-[acyl-carrier protein] methyl ester esterase	24.44366084	-0.227972993	0.63475803	-0.359149443	0.719483295	0.999978546
4.1.1.33	diphosphomevalonate decarboxylase	2.229319486	-1.946545132	2.003712197	-0.971469423	0.331314572	0.999978546
6.3.2.-	#N/A	11.16411353	0.26545425	0.967778907	0.274292246	0.783860043	0.999978546

2.4.1.-	#N/A	1001.881925	-0.112088895	0.138410104	-0.809831736	0.41803689	0.999978546
4.2.3.5	chorismate synthase	338.384486	0.201071604	0.133105771	1.51061522	0.130886514	0.999978546
1.12.2.1	NiFe] hydrogenase large subunit	22.82601474	-0.559228228	1.007131635	-0.555268258	0.578711187	0.999978546
3.7.1.22	3D-(3,5/4)-trihydroxycyclohexane-1,2-dione acylhydrolase (dec	50.44257656	0.547922858	0.392828652	1.394813885	0.163071971	0.999978546
3.5.4.1	cytosine/creatinine deaminase	15.08932727	0.844757396	0.558329198	1.513009529	0.130277238	0.999978546
4.1.3.38	4-amino-4-deoxychorismate lyase	35.10463143	-0.616790326	0.485971964	-1.269189114	0.204373623	0.999978546
6.4.1.2	acetyl-CoA carboxylase / biotin carboxylase 2	637.2201999	0.195085012	0.213882	0.912115147	0.361708105	0.999978546
6.5.1.2	DNA ligase (NAD+)	524.6015592	-0.115625144	0.147095292	-0.786056051	0.43183465	0.999978546
1.3.3.3	coproporphyrinogen III oxidase	0.900952424	-2.572022007	4.111461265	-0.625573693	0.531594598	0.999978546
5.1.99.1	methylmalonyl-CoA/ethylmalonyl-CoA epimerase	68.30107652	-0.230788487	0.471989762	-0.488969265	0.624863457	0.999978546
1.7.99.1	hydroxylamine reductase	325.2855543	0.833119464	0.460425025	1.809457389	0.070379974	0.999978546
5.3.1.22	hydroxypyruvate isomerase	18.70282022	0.348712697	0.582575727	0.598570591	0.549459272	0.999978546
4.2.1.-	#N/A	0.467580383	-1.123874687	4.131100404	-0.272052136	0.785581932	0.999978546
2.1.1.56	mRNA (guanine-N7-)-methyltransferase	0.053564081	0.094415333	4.143731151	0.022785101	0.981821693	0.999978546
6.2.1.22	[citrate (pro-3S)-lyase] ligase	34.07579315	0.801825177	0.501520153	1.598789544	0.109867373	0.999978546
1.17.2.1	nicotinate dehydrogenase subunit A	0.015832449	0.094415661	4.143731151	0.02278518	0.981821629	0.999978546
5.3.2.1	phenylpyruvate tautomerase	0.107128162	0.094412122	4.143731151	0.022784326	0.981822311	0.999978546
1.1.1.8	glycerol-3-phosphate dehydrogenase (NAD+)	1.167095298	-1.781688271	4.007842593	-0.444550461	0.656644655	0.999978546
2.7.1.53	L-xylulokinase	12.11164162	0.020411589	0.76931611	0.026532123	0.978832912	0.999978546
2.7.7.77	molybdenum cofactor guanylyltransferase	15.50953688	-0.04802103	0.70482942	-0.068131422	0.945681018	0.999978546
1.1.1.87	homoisocitrate dehydrogenase	1.266185865	2.795001745	2.609142909	1.071233674	0.284064374	0.999978546
6.3.2.31	coenzyme F420-0:L-glutamate ligase / coenzyme F420-1:gamm	4.270530574	1.997952806	1.769189265	1.129304165	0.258769544	0.999978546
3.2.1.31	beta-glucuronidase	100.6708401	-0.01772393	0.358254039	-0.049473079	0.960542291	0.999978546
3.1.21.7	deoxyribonuclease V	2.012999821	0.228050727	3.756689073	0.060705244	0.951593955	0.999978546
3.2.-.-	#N/A	42.87598427	0.556725719	0.474578899	1.173094127	0.240758062	0.999978546
2.3.1.22	2-acylglycerol O-acyltransferase 3	0.160692243	0.094410072	4.143731151	0.022783831	0.981822705	0.999978546
2.4.1.8	maltose phosphorylase	30.76090562	-0.097010787	0.392509154	-0.247155477	0.804787901	0.999978546
3.4.11.1	leucyl aminopeptidase	47.78279693	-0.228523615	0.451324383	-0.50634006	0.612617945	0.999978546
2.3.2.26	other hect domain ubiquitin protein ligase E3	0.373766343	0.734067012	4.109706846	0.178617853	0.858237776	0.999978546
2.5.1.74	1,4-dihydroxy-2-naphthoate polyprenyltransferase	209.2981939	-0.919427331	0.649218834	-1.416205573	0.156715325	0.999978546
1.1.1.156	glycerol 2-dehydrogenase (NADP+)	0.034954079	-0.421359027	4.140131853	-0.101774301	0.918935824	0.999978546
3.5.2.14	#N/A	21.35431366	0.625218891	0.837967286	0.746113723	0.45559872	0.999978546
2.3.1.28	chloramphenicol O-acetyltransferase type B	86.94404786	-0.097098238	0.463126649	-0.209658066	0.833934557	0.999978546
2.1.1.174	23S rRNA (guanine1835-N2)-methyltransferase	0.096307101	-0.778791352	4.138114321	-0.188199574	0.850720206	0.999978546
5.1.1.13	aspartate racemase	40.36829995	0.294589489	0.335234269	0.878757087	0.379532999	0.999978546
3.5.2.6	beta-lactamase class C	160.3792134	-0.3199767	0.247833266	-1.291096656	0.196670164	0.999978546
2.7.1.24	dephospho-CoA kinase	224.4460225	0.109859996	0.220578432	0.498054118	0.618445899	0.999978546
3.6.3.55	#N/A	1.408673102	-2.872835304	2.219120203	-1.294583007	0.195464118	0.999978546
3.13.1.1	UDP-sulfoquinovose synthase	0.129569077	-0.801659337	4.137956904	-0.193733129	0.84638485	0.999978546
2.1.1.185	23S rRNA (guanosine2251-2'-O)-methyltransferase	315.2882071	0.106748479	0.161834359	0.659615669	0.509500496	0.999978546
3.1.3.25	myo-inositol-1(or 4)-monophosphatase	79.05547234	-0.57025952	0.294128912	-1.938808111	0.052524708	0.999978546
2.7.7.71	D-glycero-alpha-D-manno-heptose 1-phosphate guanylyltransfe	1.859383022	0.141254198	2.096617561	0.06737242	0.946285225	0.999978546
3.4.25.1	#N/A	17.62314595	1.468044172	1.356328344	1.08236636	0.27908977	0.999978546
3.6.3.9	sodium/potassium-transporting ATPase subunit alpha	0.299485303	0.055454388	4.142061543	0.013388113	0.98931815	0.999978546
1.5.1.20	methylenetetrahydrofolate reductase (NADPH)	397.8327279	-0.267202813	0.248604089	-1.074812624	0.282458628	0.999978546
1.13.11.4	gentisate 1,2-dioxygenase	0.144460652	-1.00194317	4.133461154	-0.24239811	0.808471699	0.999978546
1.2.1.9	glyceraldehyde-3-phosphate dehydrogenase (NADP+)	6.716637653	-1.460486883	1.255571913	-1.163523066	0.244617345	0.999978546
1.3.98.1	dihydroorotate dehydrogenase (fumarate)	120.1794457	0.083667103	0.41421712	0.20198852	0.839925699	0.999978546
3.1.3.100	ribosome biogenesis GTPase / thiamine phosphate phosphatase	416.5264494	-0.331978289	0.384213267	-0.864046918	0.387562117	0.999978546
2.1.1.176	16S rRNA (cytosine967-C5)-methyltransferase	301.1690219	0.330678221	0.20850329	1.585961645	0.11274801	0.999978546
3.5.1.2	glutaminase	86.19117576	0.01296684	0.417886221	0.031029595	0.975245937	0.999978546
5.5.1.2	3-carboxy-cis,cis-muconate cycloisomerase	0.510612935	2.851550263	4.069302118	0.700746757	0.483461071	0.999978546
1.8.1.9	thioredoxin reductase (NADPH)	1244.533356	0.066615074	0.441783455	0.150786712	0.880143969	0.999978546
2.5.1.134	cystathionine beta-synthase (O-acetyl-L-serine)	0.594219345	-1.412920019	4.112708141	-0.34354979	0.73118489	0.999978546
5.1.3.29	L-fucose mutarotase	72.80484909	-0.259057042	0.730479742	-0.354639598	0.722859607	0.999978546
1.1.1.276	serine 3-dehydrogenase (NADP+)	0.034954079	-0.421359027	4.140131853	-0.101774301	0.918935824	0.999978546
3.2.1.41	pullulanase	79.05972656	0.021264616	0.40473668	0.052539383	0.958098915	0.999978546
2.7.1.100	5-methylthioribose kinase	27.51768582	0.292104471	0.441864202	0.661072949	0.508565532	0.999978546
2.7.1.83	pseudouridine kinase	78.72070777	0.219866008	0.448499896	0.490225328	0.62397446	0.999978546
4.2.2.1	hyaluronate lyase	29.93586609	-0.453893273	0.768271123	-0.590798299	0.554655575	0.999978546
2.3.1.187	malonate decarboxylase alpha subunit	10.47559418	1.029975331	0.768474768	1.340285164	0.180152653	0.999978546
2.7.11.22	CTD kinase subunit alpha	0.552718707	-1.127642	4.129899125	-0.273043473	0.784819798	0.999978546
5.3.3.17	trans-2,3-dihydro-3-hydroxyanthranilate isomerase	0.021925243	0.094418168	4.143731151	0.022785785	0.981821147	0.999978546
2.5.1.46	deoxyhypusine synthase	8.030721428	1.407225126	1.138774742	1.235736159	0.216556652	0.999978546
2.8.3.21	L-carnitine CoA-transferase	9.109101761	0.166126014	1.680809998	0.098836879	0.921267787	0.999978546
6.3.2.10	UDP-N-acetylmuramoyl-tripeptide--D-alanyl-D-alanine ligase	403.0847592	-0.0547488	0.154160891	-0.355140656	0.72248422	0.999978546
2.7.1.50	hydroxyethylthiazole kinase	131.5764821	0.466496659	0.38086362	1.224839115	0.220635844	0.999978546
2.1.1.258	5-methyltetrahydrofolate corrinoid/iron sulfur protein methyltr	273.0062463	-0.254422705	0.689633404	-0.368924568	0.712183951	0.999978546
1.7.-.-	#N/A	11.71383892	-0.330145261	0.969103132	-0.340670926	0.733351329	0.999978546
4.2.2.21	chondroitin-sulfate-ABC endolyase/exolyase	72.26463302	-0.606250728	0.533159844	-1.137090003	0.255500663	0.999978546
1.1.1.76	meso-butanediol dehydrogenase / (S,S)-butanediol dehydrogen	3.716547359	1.23633971	1.308645378	0.944747699	0.344787699	0.999978546
2.4.1.83	dolichol-phosphate mannosyltransferase	119.1710587	-0.058972841	0.278153234	-0.212015657	0.832094819	0.999978546
2.7.1.147	ADP-dependent phosphofructokinase/glucokinase	7.609187489	0.245888078	1.004475699	0.244792461	0.806617132	0.999978546
4.2.2.5	chondroitin AC lyase	45.73435572	-0.161032332	0.515775509	-0.312213994	0.754877898	0.999978546
2.1.2.5	glutamate formiminotransferase / 5-formyltetrahydrofolate cyc	154.6551231	0.140837679	0.391595781	0.359650655	0.719108398	0.999978546
1.3.1.44	enoyl-acyl-carrier protein] reductase / trans-2-enoyl-CoA reduc	27.94422685	-0.515834447	0.514718194	-1.00216867	0.316262137	0.999978546
1.5.1.41	aquacobalamin reductase / NAD(P)H-flavin reductase	1.180331694	-2.341226808	4.114238676	-0.569054689	0.569319027	0.999978546
2.7.1.205	PTS system, cellobiose-specific IIA component	67.85843679	-0.116143475	0.576732298	-0.201381951	0.840399927	0.999978546
3.2.1.20	alpha-glucosidase	601.8969372	-0.093630874	0.277849278	-0.336984406	0.736128655	0.999978546
1.13.12.16	nitronate monooxygenase	3.734228943	-0.886871606	1.587455157	-0.55867506	0.576383503	0.999978546
2.4.1.261	alpha-1,2-mannosyltransferase	0.214256324	0.094408575	4.143731151	0.02278347	0.981822993	0.999978546
2.1.1.191	23S rRNA (cytosine1962-C5)-methyltransferase	466.4840486	-0.054697335	0.223119969	-0.245147647	0.806342111	0.999978546
2.4.2.22	xanthine phosphoribosyltransferase	282.3440183	-0.376849325	0.509276258	-0.73997034	0.459317992	0.999978546
4.6.1.-	#N/A	2.805481591	3.144565434	2.478322143	1.268828366	0.204502286	0.999978546
6.1.1.15	prolyl-tRNA synthetase	530.8683727	-0.009688792	0.149307166	-0.064891672	0.948260251	0.999978546
4.4.1.-	#N/A	0.404940627	0.353907227	4.115355083	0.085996766	0.931468988	0.999978546
2.3.2.6	leucyl/phenylalanyl-tRNA---protein transferase	20.29869155	-0.247450721	0.640153928	-0.386548781	0.699090283	0.999978546

1.1.1.47	glucose 1-dehydrogenase	6.850818888	0.404644313	0.854497671	0.47354642	0.635823392	0.999978546
3.1.6.1	arylsulfatase	55.45735393	-0.503305086	0.45201628	-1.113466723	0.265508033	0.999978546
6.2.1.45	ubiquitin-activating enzyme E1	4.37738286	1.363115616	2.032666618	0.670604615	0.502472441	0.999978546
2.7.1.148	4-diphosphocytidyl-2-C-methyl-D-erythritol kinase	307.9305914	-0.087316289	0.279695856	-0.312182993	0.754901457	0.999978546
1.2.1.3	aldehyde dehydrogenase (NAD+)	65.51788066	0.19244284	0.355164413	0.541841562	0.587927657	0.999978546
3.5.1.81	N-acyl-D-amino-acid deacylase	40.77082716	-0.125383524	0.4152139	-0.30197333	0.762672394	0.999978546
5.4.99.12	#N/A	337.9885111	-0.087163298	0.209426579	-0.416199789	0.677263814	0.999978546
2.3.1.16	acetyl-CoA acyltransferase	0.591306247	-1.452547402	4.130419269	-0.351670692	0.725085243	0.999978546
4.2.1.126	N-acetylmuramic acid 6-phosphate etherase	82.6211455	0.17765771	0.355095005	0.50031036	0.61685656	0.999978546
3.5.3.6	arginine deiminase	100.4938897	0.028600968	0.435787709	0.065630507	0.947672	0.999978546
2.7.7.56	ribonuclease PH	33.41871616	0.1003223	0.532537216	0.188385519	0.850574451	0.999978546
2.4.1.166	raffinose-raffinose alpha-galactotransferase	0.509876242	0.377890656	4.084464258	0.092519026	0.926285675	0.999978546
2.7.8.39	archaetidylinositol phosphate synthase	0.621380821	-1.482396584	4.132496161	-0.35871699	0.719806817	0.999978546
3.1.27.-	#N/A	0.616679938	2.90840238	4.064081981	0.715635756	0.474216289	0.999978546
6.3.4.3	methylenetetrahydrofolate dehydrogenase (NADP+) / metheny	919.4527816	0.048432581	0.27372641	0.176937918	0.859557154	0.999978546
5.3.2.8	4-oxalomesaconate tautomerase	0.029793698	0.965488865	4.130968313	0.233719746	0.815202557	0.999978546
1.1.3.9	galactose oxidase	0.092938899	-0.766082851	4.139487465	-0.185067078	0.853176423	0.999978546
2.7.7.59	[protein-PII] uridylyltransferase	6.613776947	-0.262443838	1.102992473	-0.237938014	0.811929171	0.999978546
3.6.1.54	UDP-2,3-diacylglucosamine hydrolase	80.87134064	-0.281805508	0.310002431	-0.909042896	0.363327483	0.999978546
3.4.11.19	D-aminopeptidase	79.30120568	0.256361715	0.364250702	0.703805684	0.481553794	0.999978546
2.7.1.199	PTS system, glucose-specific IIB component	43.92933022	-0.43847387	0.542799068	-0.807801443	0.419204894	0.999978546
2.7.7.41	phosphatidate cytidylyltransferase	329.3149528	-0.07270743	0.15729367	-0.462240027	0.643909202	0.999978546
2.1.1.74	methylenetetrahydrofolate--tRNA-(uracil-5-)-methyltransferase	148.1849758	0.39457511	0.249122796	1.583857902	0.113226059	0.999978546
2.7.6.2	thiamine pyrophosphokinase	166.8556101	-0.035365856	0.355253683	-0.099550991	0.920700804	0.999978546
2.1.1.17	phosphatidylethanolamine/phosphatidyl-N-methylethanolamir	0.04941368	0.09441837	4.143731151	0.022785834	0.981821108	0.999978546
4.3.1.18	D-serine dehydratase	41.51184634	-0.017240087	0.314405233	-0.054833971	0.956270736	0.999978546
2.5.1.108	2-(3-amino-3-carboxypropyl)histidine synthase	2.518104667	3.862370263	3.329218146	1.160143341	0.245990451	0.999978546
2.4.1.1	glycogen phosphorylase	1122.779221	0.123233711	0.118119796	1.043294311	0.296812005	0.999978546
4.2.1.30	#N/A	5.746841115	-1.179989302	1.269889509	-0.929206276	0.352782196	0.999978546
2.7.7.65	two-component system, cell cycle response regulator	4.101623422	0.208348625	1.845258823	0.112910244	0.910101714	0.999978546
1.3.5.4	succinate dehydrogenase / fumarate reductase, flavoprotein su	913.8675227	0.034212574	0.297013339	0.115188678	0.908295573	0.999978546
3.6.3.4	Cu2+-exporting ATPase	642.8453761	-0.20174979	0.330098351	-0.611180848	0.541079861	0.999978546
1.1.5.6	#N/A	0.860990484	-2.214432212	4.115057981	-0.538129043	0.590487963	0.999978546
2.4.1.52	poly(glycerol-phosphate) alpha-glucosyltransferase	0.161648	1.655918303	4.11892868	0.402026457	0.687664555	0.999978546
4.1.1.31	phosphoenolpyruvate carboxylase	18.28372225	-0.947893482	0.852456531	-1.111955212	0.266157408	0.999978546
4.1.2.22	xylulose-5-phosphate/fructose-6-phosphate phosphoketolase	141.7183535	-0.01635918	0.327530131	-0.049947099	0.960164545	0.999978546
3.6.3.25	sulfate transport system ATP-binding protein	43.02532111	-0.22745597	0.530495966	-0.42876098	0.668097178	0.999978546
3.6.1.15	#N/A	5.035962108	-0.977549006	1.573814358	-0.621133618	0.534511712	0.999978546
3.6.3.36	taurine transport system ATP-binding protein	0.734608246	0.025917601	4.069161252	0.006369274	0.994918089	0.999978546
3.2.1.73	licheninase	0.785591429	-2.414466587	3.65831063	-0.659994962	0.509257062	0.999978546
2.1.1.309	18S rRNA (guanine1575-N7)-methyltransferase	0.262409875	-0.346672716	4.140651023	-0.083724205	0.933275712	0.999978546
1.3.8.1	butyryl-CoA dehydrogenase	257.9585902	-0.319306455	0.568955722	-0.561214947	0.574651015	0.999978546
1.8.5.3	anaerobic dimethyl sulfoxide reductase subunit A	18.00441963	-1.142897728	0.823119093	-1.388496195	0.164986003	0.999978546
5.4.3.2	lysine 2,3-aminomutase	170.8248805	0.030518043	0.32961868	0.092585901	0.926232545	0.999978546
2.6.1.2	alanine transaminase	68.37319459	-0.845464271	0.532670326	-1.587218641	0.112463134	0.999978546
1.3.1.74	2-alkenal reductase	1.219066924	-0.090003372	3.789621401	-0.023749964	0.981052052	0.999978546
3.1.3.48	protein-tyrosine phosphatase	512.7382816	-0.213985134	0.266353835	-0.80338672	0.421751248	0.999978546
5.4.99.29	tRNA pseudouridine32 synthase / 23S rRNA pseudouridine746 s	73.12648985	-0.301682926	0.37260146	-0.809666517	0.418131867	0.999978546
3.1.21.4	type II restriction enzyme	23.82095201	0.349882505	0.636521952	0.549678614	0.582539827	0.999978546
2.3.1.97	glycylpeptide N-tetradecanoyltransferase	0.267820405	0.094407394	4.143731151	0.022783185	0.981823221	0.999978546
4.6.1.16	tRNA-intron endonuclease, archaea type	0.575809695	1.639270119	4.104639377	0.399370071	0.689620543	0.999978546
1.18.1.3	3-phenylpropionate/trans-cinnamate dioxygenase ferredoxin re	0.132218179	0.066291295	4.142488731	0.01600277	0.987232182	0.999978546
3.1.6.6	choline-sulfatase	12.21331139	-0.607403619	0.740179819	-0.820616292	0.411864865	0.999978546
1.8.5.2	thiosulfate dehydrogenase [quinone] small subunit	14.79835306	-0.740601051	0.731787898	-1.012043317	0.311517343	0.999978546
6.3.1.8	glutathionylspermidine amidase/synthetase	1.535270693	-0.151221836	3.067037398	-0.049305508	0.96067583	0.999978546
2.3.1.9	acetyl-CoA C-acetyltransferase	972.9845713	0.046314525	0.6269618	0.073871367	0.94111274	0.999978546
1.1.1.28	D-lactate dehydrogenase	178.3211075	0.02970897	0.213504981	0.139148839	0.889332536	0.999978546
2.7.1.167	D-beta-D-heptose 7-phosphate kinase / D-beta-D-heptose 1-ph	16.49374106	-0.704306644	0.679337258	-1.036755508	0.299849816	0.999978546
4.3.2.2	adenylosuccinate lyase	625.3639552	0.053384177	0.21177827	0.2520758	0.800982475	0.999978546
3.2.1.139	alpha-glucuronidase	70.11148679	-0.537009919	0.618185511	-0.868687326	0.385018176	0.999978546
4.2.1.77	trans-L-3-hydroxyproline dehydratase	0.959760061	-1.328968104	4.097446414	-0.32434057	0.745680208	0.999978546
2.3.1.89	tetrahydrodipicolinate N-acetyltransferase	3.970360467	0.080363656	1.136386621	0.070718587	0.943621728	0.999978546
1.5.1.2	pyrroline-5-carboxylate reductase	309.2710635	-0.518068568	0.31538073	-1.64267667	0.100449848	0.999978546
1.7.2.5	nitric oxide reductase subunit B	0.671618783	1.507897378	4.096646413	0.368080919	0.712812898	0.999978546
2.4.1.11	glycogen synthase	62.34508966	0.514230759	0.494387168	1.040137755	0.298275905	0.999978546
2.8.1.6	biotin synthase	192.6670322	-0.154954966	0.293284541	-0.528343448	0.597260981	0.999978546
2.7.1.71	shikimate kinase	321.2596761	-0.26921666	0.257401205	-1.045902873	0.295605878	0.999978546
3.5.1.47	N-acetyldiaminopimelate deacetylase	2.679831447	-1.101205607	2.02538085	-0.543702981	0.586645881	0.999978546
1.2.5.1	pyruvate dehydrogenase (quinone)	24.97110696	-0.110955757	0.491625736	-0.225691514	0.821441358	0.999978546
4.99.1.1	protoporphyrin/coproporphyrin ferrochelatase	9.070073581	-1.914965304	1.066431563	-1.795675757	0.0725461	0.999978546
2.4.2.52	triphosphoribosyl-dephospho-CoA synthase	26.49897562	1.063605872	0.581266948	1.829806212	0.067278922	0.999978546
1.3.4.1	fumarate reductase (CoM/CoB) subunit A	11.21361449	1.812332807	1.374522437	1.318518169	0.187330247	0.999978546
6.2.1.5	succinyl-CoA synthetase alpha subunit	201.7652483	-0.152238101	0.710266293	-0.214339471	0.83028234	0.999978546
4.2.1.136	ADP-dependent NAD(P)H-hydrate dehydratase	296.924103	-0.078375239	0.179767195	-0.435981876	0.662849879	0.999978546
2.7.1.153	phosphatidylinositol-4,5-bisphosphate 3-kinase catalytic subuni	0.107128162	0.094412122	4.143731151	0.022784326	0.981822311	0.999978546
1.5.1.39	FMN reductase [NAD(P)H]	18.48410048	0.243778626	0.900856035	0.270607751	0.786692734	0.999978546
4.1.99.14	spore photoproduct lyase	9.608842198	-1.47315461	0.75771188	-1.944214744	0.05186956	0.999978546
1.1.1.281	GDP-4-dehydro-6-deoxy-D-mannose reductase	14.17185845	0.327831202	0.624189848	0.525210723	0.599436711	0.999978546
6.3.1.10	adenosylcobinamide-phosphate synthase	146.8807414	0.456878851	0.286359883	1.595470869	0.110606979	0.999978546
5.4.2.7	phosphopentomutase	271.5281824	-0.178613032	0.267553408	-0.667578984	0.504402376	0.999978546
2.7.1.190	aminoglycoside 2''-phosphotransferase	1.1071359	0.965569788	3.053119707	0.316256773	0.751807618	0.999978546
1.12.98.2	5,10-methenyltetrahydromethanopterin hydrogenase	33.13934147	2.33795846	1.121069894	2.085470739	0.037026592	0.999978546
2.7.1.36	mevalonate kinase	7.588783217	0.83592548	1.610409507	0.519076344	0.603707504	0.999978546
4.2.1.91	cyclohexadienyl dehydratase	1.859733319	0.443119946	2.04591954	0.216587181	0.828530077	0.999978546
3.5.4.39	GTP cyclohydrolase IV	4.206096909	1.957683113	1.614393782	1.212642872	0.22526639	0.999978546
5.3.1.8	mannose-6-phosphate isomerase	279.7365829	0.049932823	0.272937786	0.182945806	0.854840542	0.999978546
3.1.3.71	2-phosphosulfolactate phosphatase	9.214936184	1.813464548	0.798265512	2.271756104	0.023101245	0.999978546

2.7.11.33	[pyruvate, water dikinase]-phosphate phosphotransferase / [py	11.94070215	-0.948567941	0.778185263	-1.218948733	0.222863651	0.999978546
2.7.1.19	phosphoribulokinase	0.240767753	-1.252599762	4.129265175	-0.303346893	0.761625513	0.999978546
1.2.1.24	succinate-semialdehyde dehydrogenase	0.096307101	-0.778791352	4.138114321	-0.188199574	0.850720206	0.999978546
2.7.1.168	D-glycero-alpha-D-manno-heptose-7-phosphate kinase	31.71658101	-0.373512491	0.554305024	-0.673839266	0.500413508	0.999978546
4.1.3.42	2-dehydro-3-deoxyphosphogluconate aldolase / (4S)-4-hydroxy	365.4288842	0.171885431	0.280554607	0.612663013	0.540099183	0.999978546
1.3.99.31	phytoene desaturase	1.632348624	0.91736795	4.096641466	0.223931715	0.822810437	0.999978546
3.1.3.15	imidazoleglycerol-phosphate dehydratase / histidinol-phosphat	920.6179347	-1.356976559	0.664720383	-2.041424627	0.041208639	0.999978546
4.3.1.14	3-aminobutyryl-CoA ammonia-lyase	70.2832787	-0.06397409	0.671809263	-0.095226568	0.924134868	0.999978546
1.8.2.2	thiosulfate dehydrogenase	0.06577573	0.094416003	4.143731151	0.022785263	0.981821564	0.999978546
2.3.1.54	formate C-acetyltransferase	2143.677149	0.060120501	0.446418643	0.134672917	0.892870487	0.999978546
3.5.4.29	GTP cyclohydrolase IIa	5.074632583	2.942572551	2.222800951	1.32381289	0.185565194	0.999978546
5.3.4.1	protein disulfide-isomerase	8.650518897	0.134546123	1.370128127	0.098199665	0.921773749	0.999978546
2.4.1.117	dolichyl-phosphate beta-glucosyltransferase	0.15199015	-0.437399916	4.140454144	-0.105640565	0.915867538	0.999978546
4.1.3.40	chorismate--pyruvate lyase	0.298604074	-1.014786816	4.133464141	-0.24550517	0.806065306	0.999978546
3.1.11.1	exodeoxyribonuclease I	5.573815682	-0.153361302	1.345111249	-0.114013842	0.90922682	0.999978546
2.5.1.43	nicotianamine synthase	0.050191057	-0.266239077	4.143731151	-0.06425105	0.948770329	0.999978546
3.1.3.87	2-hydroxy-3-keto-5-methylthiopentenyl-1-phosphate phosphat	0.051061294	0.971902104	4.129959009	0.235329722	0.813952828	0.999978546
2.3.1.235	minimal PKS ketosynthase (KS/KS alpha)	0.046441742	-0.12847344	4.133265809	-0.031082792	0.975203513	0.999978546
3.4.21.50	lysyl endopeptidase	0.022430994	-0.266241886	4.143731151	-0.064251728	0.94876979	0.999978546
4.4.1.24	(2R)-sulfolactate sulfo-lyase subunit alpha	10.38634163	0.88391197	1.074515081	0.822614764	0.4107271	0.999978546
3.4.14.4	dipeptidyl-peptidase III	183.6513868	-0.80710623	0.392412899	-2.056778032	0.039707582	0.999978546
3.4.21.105	rhomboid-related protein 1/2/3	57.76563614	-0.792401329	0.412204235	-1.922351257	0.054561574	0.999978546
3.1.6.-	#N/A	5.647174252	-1.172060216	1.00926344	-1.16130256	0.245518879	0.999978546
6.1.1.9	valyl-tRNA synthetase	675.7895904	-0.090798618	0.190975111	-0.475447392	0.634468122	0.999978546
2.7.4.25	CMP/dCMP kinase	386.0131937	-0.098683955	0.259177965	-0.380757505	0.703383195	0.999978546
1.2.1.59	glyceraldehyde-3-phosphate dehydrogenase (NAD(P))	6.878986232	2.200113133	1.731263891	1.270813274	0.203795085	0.999978546
3.5.2.9	5-oxoprolinase (ATP-hydrolysing)	0.116409678	1.169478482	4.119476897	0.283890045	0.776494646	0.999978546
1.1.1.25	shikimate dehydrogenase	261.1601688	0.068984787	0.253104684	0.272554368	0.785195793	0.999978546
4.2.1.59	3-hydroxyacyl-[acyl-carrier protein] dehydratase / trans-2-dece	276.4956797	-0.329529733	0.233819484	-1.409333935	0.158736449	0.999978546
2.3.1.182	(R)-citramalate synthase	90.27086847	0.297497972	0.245823289	1.210210688	0.226198058	0.999978546
4.1.1.65	phosphatidylserine decarboxylase	135.2245112	-0.222058631	0.344861087	-0.643907471	0.519635437	0.999978546
3.4.21.92	ATP-dependent Clp protease, protease subunit	561.0943661	0.09093229	0.234164149	0.388327124	0.697773967	0.999978546
2.7.7.61	holo-ACP synthase	27.06562961	0.613170567	0.698298148	0.878092788	0.379893367	0.999978546
3.3.2.1	bifunctional isochorismate lyase / aryl carrier protein	0.250956274	-0.960699197	4.133964155	-0.232391758	0.81623375	0.999978546
1.2.1.71	succinylglutamic semialdehyde dehydrogenase	0.048153551	-0.266241886	4.143731151	-0.064251728	0.94876979	0.999978546
2.7.9.2	pyruvate, water dikinase	67.74853073	0.080520341	0.514303977	0.156561771	0.875590233	0.999978546
1.2.1.10	acetaldehyde dehydrogenase (acetylating)	400.4918272	-0.129738083	0.466625024	-0.278034988	0.7809855	0.999978546
1.3.1.43	cyclohexadieny/prephenate dehydrogenase	3.773900336	-2.681214758	1.966734676	-1.363282394	0.172793528	0.999978546
4.1.3.3	N-acetylneuraminate lyase	294.7456095	-0.333451406	0.433185673	-0.769765546	0.441438981	0.999978546
5.4.99.1	methylaspartate mutase sigma subunit	67.78198055	0.257631723	0.610061843	0.422304273	0.672802939	0.999978546
2.3.3.14	homocitrate synthase	6.772603954	1.300251217	1.156766161	1.124039811	0.260996137	0.999978546
6.3.4.14	acetyl-CoA carboxylase / biotin carboxylase 2	281.1120721	0.162199642	0.193941068	0.836334685	0.402966646	0.999978546
1.-.-.-	#N/A	90.00714768	-0.304971885	0.408105617	-0.747286665	0.45489054	0.999978546
1.16.1.1	mercuric reductase	0.505665624	-1.562596203	4.120326026	-0.379240913	0.704508973	0.999978546
1.16.3.1	bacterioferritin	74.72166482	-0.37661572	0.495850473	-0.759534862	0.447532668	0.999978546
4.1.2.19	rhamnulose-1-phosphate aldolase	135.5495071	-0.247011753	0.426928664	-0.578578517	0.562873604	0.999978546
2.5.1.129	flavin prenyltransferase	25.08112497	-0.312837952	0.574511136	-0.544528962	0.586077524	0.999978546
1.1.1.271	GDP-L-fucose synthase	181.2243692	0.161806178	0.222296137	0.727885694	0.466683562	0.999978546
5.1.99.7	D-erythro-7,8-dihydroneopterin triphosphate epimerase	0.144460652	-1.00194317	4.133461154	-0.24239811	0.808471699	0.999978546
3.4.17.11	glutamate carboxypeptidase	21.84682526	0.149473024	0.560987512	0.26644626	0.789895542	0.999978546
2.3.1.243	lauroyl-Kdo2-lipid IVA myristoyltransferase	0.273484821	0.233996621	4.09985053	0.057074427	0.954485907	0.999978546
1.8.4.14	L-methionine (R)-S-oxide reductase	1.203925453	5.03320624	4.058700756	1.240102817	0.214937367	0.999978546
4.4.1.15	D-cysteine desulfhydrase	28.02213594	-0.690972276	0.381715769	-1.810174828	0.07026868	0.999978546
2.7.8.42	KDO II ethanolaminephosphotransferase	0.429078891	-1.384087662	4.130720756	-0.335071709	0.737570994	0.999978546
2.6.1.59	dTDP-4-amino-4,6-dideoxygalactose transaminase	72.97848554	0.102898828	0.310583192	0.331308426	0.740411527	0.999978546
1.4.1.14	glutamate synthase (NADH)	1092.906852	-0.085542023	0.127190976	-0.672547891	0.501234962	0.999978546
3.1.3.7	3'(2'), 5'-bisphosphate nucleotidase	529.7510404	0.18869446	0.298275359	0.63261833	0.526982914	0.999978546
2.4.1.315	processive 1,2-diacylglycerol beta-glucosyltransferase	113.4372334	0.202499592	0.36108825	0.560803605	0.574931429	0.999978546
4.2.1.41	5-dehydro-4-deoxyglucarate dehydratase	0.227201514	-1.167578952	4.132521476	-0.282534273	0.77753387	0.999978546
3.2.1.84	#N/A	0.107128162	0.094412122	4.143731151	0.022784326	0.981822311	0.999978546
1.3.7.7	light-independent protochlorophyllide reductase subunit L	0.248906341	-0.029207452	4.139498352	-0.007055795	0.994370337	0.999978546
1.1.1.61	4-hydroxybutyrate dehydrogenase	46.40904916	0.792068119	0.364841667	2.170991395	0.029931823	0.999978546
3.6.3.44	#N/A	1.312530107	0.997109836	2.787249658	0.357739693	0.720538126	0.999978546
2.7.7.82	CMP-N,N'-diacetyllegionaminic acid synthase	8.972758846	-0.895186641	0.570238759	-1.569845311	0.116451103	0.999978546
3.2.1.65	levanase	13.77925283	0.453693381	0.859568694	0.527815152	0.597627639	0.999978546
2.7.4.1	polyphosphate kinase	532.0088662	0.084608912	0.181468763	0.466245045	0.64104011	0.999978546
3.1.-.-	#N/A	7215.830595	0.071786871	0.188378487	0.381077864	0.703145472	0.999978546
1.6.4.-	#N/A	210.9728632	-0.579430465	0.478520916	-1.210878032	0.225942153	0.999978546
3.5.1.18	succinyl-diaminopimelate desuccinylase	238.9037885	0.104468694	0.182561079	0.572239684	0.567159606	0.999978546
2.3.3.-	#N/A	4.148446599	2.337192605	2.033240153	1.149491663	0.250353302	0.999978546
3.1.3.41	4-nitrophenyl phosphatase	2.475652433	1.505573215	1.769756479	0.850723381	0.394923032	0.999978546
2.3.1.48	chromodomain protein Y	4.949563951	-1.367898182	1.457687635	-0.938402816	0.348037439	0.999978546
3.1.3.10	glucose-1-phosphatase	1.077587015	3.49913785	4.034174938	0.86737385	0.385737205	0.999978546
6.3.2.29	cyanophycin synthetase	4.102299911	2.004700827	3.731008814	0.537307985	0.591054891	0.999978546
2.4.2.18	anthranilate phosphoribosyltransferase	118.1962522	-0.208650561	0.43467072	-0.480019819	0.6312133	0.999978546
2.4.1.293	GalNAc5-diNAcBac-PP-undecaprenol beta-1,3-glucosyltransfera	0.450882135	-0.705251823	4.133035977	-0.170637717	0.864508639	0.999978546
3.2.1.23	beta-galactosidase	3131.489228	-0.178592004	0.138441219	-1.290020452	0.197043557	0.999978546
4.2.1.55	3-hydroxybutyryl-CoA dehydratase	14.441369	1.180599286	0.916737202	1.287827399	0.197806052	0.999978546
3.5.1.88	peptide deformylase	403.7885556	0.065403526	0.240917335	0.271477046	0.786024152	0.999978546
2.1.1.177	23S rRNA (pseudouridine1915-N3)-methyltransferase	205.3404314	0.335715833	0.237661459	1.412580039	0.157779242	0.999978546
6.5.1.6	DNA ligase 1	16.24694701	0.644561848	0.880509676	0.732032669	0.464148628	0.999978546
2.6.1.42	branched-chain amino acid aminotransferase	427.0846235	0.46923934	0.278920808	1.682338954	0.092503132	0.999978546
2.6.1.16	glucosamine---fructose-6-phosphate aminotransferase (isomeri	625.9817941	-0.075234217	0.198957105	-0.378142903	0.705324439	0.999978546
5.3.1.13	arabinose 5-phosphate isomerase	81.00631304	-0.471902336	0.307649235	-1.533897315	0.125054917	0.999978546
5.4.99.21	23S rRNA pseudouridine2604 synthase	55.92207997	-0.169489473	0.389580851	-0.435055964	0.663521806	0.999978546
3.4.24.25	vibriolysin	14.58659153	1.190599499	2.645101768	0.450114817	0.652627653	0.999978546
3.1.4.3	phospholipase C	2.840339435	3.277061393	2.541533406	1.289403234	0.197257938	0.999978546

4.1.1.73	tartrate dehydrogenase/decarboxylase / D-malate dehydrogen	0.660399619	0.688830431	4.077962478	0.168915343	0.865863231	0.999978546
2.4.2.-	#N/A	193.4053107	-0.194693394	0.223837124	-0.869799388	0.384410046	0.999978546
3.1.1.32	phospholipase A1/A2	6.442157471	-0.974963702	1.091394475	-0.893319258	0.371686228	0.999978546
1.14.13.1	salicylate hydroxylase	0.048153551	-0.266241886	4.143731151	-0.064251728	0.94876979	0.999978546
1.1.1.303	(R,R)-butanediol dehydrogenase / meso-butanediol dehydroger	5.627014574	0.210655915	1.677819523	0.125553382	0.900085467	0.999978546
3.8.1.3	haloacetate dehalogenase	2.266409738	-1.504099306	1.898425962	-0.792287577	0.428193014	0.999978546
1.1.1.2	alcohol dehydrogenase (NADP+)	2.742843588	-2.059386186	1.712026438	-1.202893916	0.229017375	0.999978546
3.4.14.11	X-Pro dipeptidyl-peptidase	20.93093077	-1.020047797	0.716836955	-1.422984391	0.15474068	0.999978546
1.5.1.3	dihydrofolate reductase	420.2366272	-0.199649243	0.602440843	-0.331400577	0.740341929	0.999978546
2.7.11.26	glycogen synthase kinase 3 beta	0.160692243	0.094410072	4.143731151	0.022783831	0.981822705	0.999978546
6.2.1.42	3-oxocholest-4-en-26-oate---CoA ligase	0.197654718	0.094413872	4.143731151	0.022784748	0.981821974	0.999978546
2.5.1.17	cob(I)alamin adenosyltransferase	297.5379903	-0.044979834	0.281575598	-0.159743366	0.873083238	0.999978546
2.1.1.62	#N/A	0.107128162	0.094412122	4.143731151	0.022784326	0.981822311	0.999978546
2.7.1.48	uridine kinase	1798.770566	-0.475482407	0.609916358	-0.779586251	0.435634451	0.999978546
2.5.1.30	heptaprenyl diphosphate synthase	102.0691133	0.194689744	0.440771358	0.441702349	0.65870461	0.999978546
2.7.7.4	bifunctional enzyme CysN/CysC	320.7491654	-0.197599061	0.252319693	-0.783129762	0.433550916	0.999978546
6.2.1.26	O-succinylbenzoic acid---CoA ligase	43.02822346	-0.278750334	0.360401138	-0.773444655	0.439259271	0.999978546
1.3.1.83	geranylgeranyl diphosphate/geranylgeranyl-bacteriochlorophyl	0.022430994	-0.266241886	4.143731151	-0.064251728	0.94876979	0.999978546
3.2.2.4	AMP nucleosidase	42.98149885	0.070330699	0.538688777	0.130559058	0.896124132	0.999978546
3.4.19.3	pyroglutamyl-peptidase	36.92515586	-0.141598606	0.388769756	-0.364222277	0.715692033	0.999978546
1.1.1.43	phosphogluconate 2-dehydrogenase	0.089723976	-0.795848752	4.137904308	-0.192331357	0.847482657	0.999978546
1.2.7.7	2-oxoisovalerate ferredoxin oxidoreductase, alpha subunit	19.38805552	0.443767072	1.395345149	0.318033909	0.750459216	0.999978546
4.1.3.34	citrate lyase subunit beta / citryl-CoA lyase	88.71502524	0.262277064	0.442819009	0.592289532	0.553656724	0.999978546
5.3.1.26	galactose-6-phosphate isomerase	2.630607547	2.657653426	1.9079405	1.392943557	0.16363686	0.999978546
5.1.3.4	#N/A	185.3708773	-0.287808677	0.2897517	-0.993294178	0.320566614	0.999978546
3.2.1.136	glucuronoarabinoxylan endo-1,4-beta-xylanase	13.2680149	0.043079586	0.587098263	0.073377132	0.941506015	0.999978546
5.1.3.35	2-epi-5-epi-valiolone 7-phosphate 2-epimerase	0.265720381	-0.663197649	4.137994837	-0.160270294	0.872668158	0.999978546
2.7.4.14	UMP-CMP kinase	0.642768973	0.094402618	4.143731151	0.022782033	0.98182414	0.999978546
2.7.8.33	UDP-GlcNAc:undecaprenyl-phosphate/decaprenyl-phosphate G	45.64451026	-0.760183699	0.592723306	-1.282527092	0.199657807	0.999978546
2.7.1.28	triose/dihydroxyacetone kinase / FAD-AMP lyase (cyclizing)	5.825853036	3.040228769	4.021328188	0.756026026	0.449633592	0.999978546
4.1.1.17	ornithine decarboxylase	21.20996489	0.196758533	0.816046968	0.241111775	0.809468486	0.999978546
5.4.2.12	2,3-bisphosphoglycerate-independent phosphoglycerate mutas	1248.632008	-0.279087706	0.343415306	-0.812682783	0.416399955	0.999978546
2.7.4.7	hydroxymethylpyrimidine/phosphomethylpyrimidine kinase / t	174.8667027	-0.409461757	0.277564773	-1.475193527	0.140160521	0.999978546
1.1.1.330	17beta-estradiol 17-dehydrogenase / very-long-chain 3-oxoacyl	0.374948568	0.094405584	4.143731151	0.022782748	0.981823569	0.999978546
2.1.1.43	histone-lysine N-methyltransferase ASH1L	0.160692243	0.094410072	4.143731151	0.022783831	0.981822705	0.999978546
2.7.4.29	Kdo2-lipid A phosphotransferase	0.202890383	0.162822875	4.137797413	0.039350132	0.968611238	0.999978546
2.6.1.83	LL-diaminopimelate aminotransferase	321.5540511	0.065111933	0.210326274	0.309575842	0.75688353	0.999978546
3.1.4.16	2',3'-cyclic-nucleotide 2'-phosphodiesterase / 3'-nucleotidase	234.785924	0.09408522	0.316916785	0.296876732	0.766560621	0.999978546
2.4.1.4	amylosucrase	278.8244437	-0.130663208	0.469660964	-0.278207511	0.78085307	0.999978546
4.6.1.1	adenylate cyclase	23.29535609	-0.185440862	0.577739536	-0.320976582	0.748228138	0.999978546
3.1.21.-	#N/A	822.8588976	-0.472713693	0.498615375	-0.948052782	0.343102585	0.999978546
3.2.1.2	beta-amylase	0.22353784	1.260317611	4.122658445	0.305705075	0.759829209	0.999978546
4.1.1.55	4,5-dihydroxyphthalate decarboxylase	0.043850487	0.094417027	4.143731151	0.02278551	0.981821367	0.999978546
2.5.1.15	dihydropteroate synthase	139.5745534	-0.306781892	0.220991124	-1.388209115	0.165073378	0.999978546
2.7.7.49	RNA-directed DNA polymerase	107.7899205	-0.327431615	0.360939239	-0.907165472	0.3643193	0.999978546
4.2.2.24	rhamnogalacturonan exolyase	3.284691663	-1.031037396	1.627802718	-0.633392109	0.526477617	0.999978546
2.6.1.55	taurine---2-oxoglutarate transaminase	47.87404651	-1.087936536	0.705257014	-1.542610018	0.122925426	0.999978546
1.8.4.10	phosphoadenosine phosphosulfate reductase	26.55279816	-0.959411738	0.683660503	-1.403345278	0.160513903	0.999978546
3.2.1.91	cellulose 1,4-beta-cellobiosidase	0.254639706	-0.893314692	4.108577964	-0.217426735	0.827875798	0.999978546
1.5.1.5	methylenetetrahydrofolate dehydrogenase (NADP+) / metheny	432.3435048	-0.07651126	0.235471467	-0.324927945	0.745235607	0.999978546
3.7.1.8	2,6-dioxo-6-phenylhexa-3-enoate hydrolase	0.247596681	-1.644454991	4.125609698	-0.39859684	0.69019029	0.999978546
2.8.4.1	#N/A	294.1217901	1.712439964	1.176198228	1.455911022	0.145417218	0.999978546
2.8.1.10	thiazole synthase	81.74543984	0.191548055	0.352874134	0.5428226	0.587251951	0.999978546
3.6.3.20	sn-glycerol 3-phosphate transport system ATP-binding protein	11.33154221	1.270751944	0.918419219	1.38362952	0.166471925	0.999978546
2.3.1.41	3-oxoacyl-[acyl-carrier-protein] synthase I	64.89992041	-0.08969223	0.520600993	-0.172285938	0.863212739	0.999978546
2.6.1.11	acetylornithine aminotransferase	668.5746803	-0.262081329	0.508867144	-0.515028985	0.606532766	0.999978546
1.3.5.2	dihydroorotate dehydrogenase	21.83162396	-0.159537576	0.619596957	-0.257486054	0.79680358	0.999978546
2.7.7.19	poly(A) polymerase	28.18380502	-0.24297053	0.633607456	-0.383471702	0.701370048	0.999978546
4.2.1.70	#N/A	46.01611001	-0.278895547	0.658884166	-0.423284641	0.672087599	0.999978546
1.2.1.20	succinate-semialdehyde dehydrogenase / glutarate-semialdehy	8.343123664	0.202987017	1.097048285	0.185030157	0.853205382	0.999978546
4.3.3.6	pyridoxal 5'-phosphate synthase pdxS subunit	136.5195282	-0.328940784	0.310515834	-1.05933659	0.289446517	0.999978546
1.1.1.373	4-hydroxybutyrate dehydrogenase / sulfolactaldehyde 3-reduct	0.167610981	-0.200187485	4.132011979	-0.048447944	0.96135925	0.999978546
2.1.1.226	23S rRNA (cytidine1920-2'-O)/16S rRNA (cytidine1409-2'-O)-me	278.8082903	0.233944061	0.294999439	0.793032221	0.42775905	0.999978546
5.4.99.16	maltose alpha-D-glucosyltransferase / alpha-amylase	86.28108937	0.25584628	0.496450673	0.515350857	0.606307866	0.999978546
3.1.3.21	glycerol-1-phosphatase	0.231939278	-0.893763178	4.12272929	-0.216789198	0.82837263	0.999978546
1.3.1.1	dihydropyrimidine dehydrogenase (NAD+) subunit PreT	55.39741083	-0.257389454	0.440816192	-0.583892922	0.559292356	0.999978546
4.1.2.25	7,8-dihydroneopterin aldolase/epimerase/oxygenase	88.0690056	-0.518018363	0.267928635	-1.933419184	0.05318458	0.999978546
2.3.1.168	2-oxoisovalerate dehydrogenase E2 component (dihydrolipoyl t	0.137221322	-0.666246229	4.136408783	-0.161068759	0.872039246	0.999978546
3.6.3.12	K+-transporting ATPase ATPase A chain	131.978621	-0.777966794	0.583126416	-1.334130597	0.18216106	0.999978546
3.1.1.1	carboxylesterase 1	6.423676539	0.993533317	1.03610491	0.958911889	0.337603135	0.999978546
2.3.1.128	#N/A	202.8758722	-0.545116021	0.434630173	-1.254206573	0.209766931	0.999978546
2.4.1.80	ceramide glucosyltransferase	0.130175812	0.008357316	4.129443689	0.002023836	0.998385214	0.999978546
2.5.1.120	aminodeoxyfutalosine synthase	14.44656408	-0.424943947	0.710340039	-0.598226093	0.549689083	0.999978546
6.3.3.3	dethiobiotin synthetase	42.7523479	0.031277686	0.497842935	0.062826413	0.949904733	0.999978546
3.6.1.11	exopolyphosphatase	219.8727794	0.445123654	0.43060295	1.033721793	0.301266242	0.999978546
2.1.1.37	DNA (cytosine-5)-methyltransferase 1	626.4275744	-0.25920116	0.351499388	-0.73741568	0.460869603	0.999978546
4.3.99.3	7-carboxy-7-deazaguanine synthase	27.63428654	-0.876877502	0.546603011	-1.604231013	0.108663141	0.999978546
3.2.2.23	#N/A	37.572242	-0.203176624	0.656301119	-0.309578361	0.756881614	0.999978546
3.4.23.52	archaeal preflagellin peptidase FlaK	0.111483345	-0.379802755	4.139741955	-0.091745514	0.926900235	0.999978546
3.2.1.64	levanbiose-producing levanase	1.029822388	-1.417735495	4.097864116	-0.345969377	0.729365729	0.999978546
4.1.99.2	tyrosine phenol-lyase	16.61240796	-1.261098529	1.569924245	-0.80328623	0.421809315	0.999978546
3.1.22.4	crossover junction endodeoxyribonuclease RuvC	151.494518	0.060634428	0.284755676	0.212934923	0.831377722	0.999978546
2.7.8.5	CDP-diacylglycerol---glycerol-3-phosphate 3-phosphatidyltransf	283.7050059	0.332844544	0.216908956	1.534489629	0.124909243	0.999978546
1.4.1.21	aspartate dehydrogenase	44.11897346	0.224478042	0.363396013	0.617722907	0.536758009	0.999978546
2.6.1.102	perosamine synthetase	3.986928639	3.046563992	1.587666188	1.918894548	0.054997681	0.999978546
3.2.1.177	alpha-D-xyloside xylohydrolase	556.3891845	-0.200991073	0.220829671	-0.910163348	0.36273637	0.999978546
1.3.1.9	enoyl-acyl-carrier protein] reductase I	337.7412044	-0.15314472	0.167203425	-0.915918556	0.359709613	0.999978546

2.7.1.12	gluconokinase	2.191401162	-0.511760366	1.644024631	-0.311285097	0.755583896	0.999978546
2.1.1.266	23S rRNA (adenine2030-N6)-methyltransferase	8.85099374	-3.209533235	1.449430071	-2.214341553	0.026805292	0.999978546
4.1.2.4	deoxyribose-phosphate aldolase	354.3068753	0.083333367	0.233544009	0.356820828	0.721225942	0.999978546
3.4.13.21	dipeptidase E	57.55391717	-0.075365453	0.438729549	-0.17178112	0.863609609	0.999978546
1.2.1.70	glutamyl-tRNA reductase	52.01114862	-0.2248641	0.363308059	-0.618935073	0.535959132	0.999978546
2.5.1.10	farnesyl diphosphate synthase	408.1672705	0.397852517	0.201468949	1.974758484	0.048295562	0.999978546
2.4.2.28	5'-methylthioadenosine phosphorylase	7.435936035	0.843542912	0.716923162	1.17661551	0.239349004	0.999978546
6.1.1.23	nondiscriminating aspartyl-tRNA synthetase	52.43350952	-0.457816229	0.538259002	-0.850550065	0.395019338	0.999978546
2.7.2.15	propionate kinase	0.163600083	-1.069752695	4.132180033	-0.258883371	0.795725229	0.999978546
2.7.1.3	ketohexokinase	0.044861988	-0.525974697	4.141284466	-0.127007623	0.898934365	0.999978546
6.1.1.20	#N/A	872.735898	0.119359041	0.249927957	0.477573785	0.632953593	0.999978546
1.8.99.2	#N/A	150.0364969	-0.965395016	0.403968066	-2.389780519	0.016858444	0.999978546
2.1.1.170	16S rRNA (guanine527-N7)-methyltransferase	224.0979338	-0.167294688	0.257564561	-0.64952526	0.515998924	0.999978546
2.4.1.230	kojibiose phosphorylase	6.165137258	-0.825977547	1.267240563	-0.651792226	0.514535217	0.999978546
1.2.7.-	#N/A	6148.42736	0.18568194	0.523166941	0.354919101	0.722650198	0.999978546
1.2.7.11	2-oxoglutarate/2-oxoacid ferredoxin oxidoreductase subunit al	1113.830126	0.289125805	0.345980863	0.835669935	0.403340614	0.999978546
4.1.2.48	threonine aldolase	240.4281868	0.100657958	0.22764486	0.442171009	0.658365463	0.999978546
2.1.1.319	type I protein arginine methyltransferase	0.353049384	0.050416647	4.141871734	0.012172431	0.990288045	0.999978546
1.8.2.3	sulfide dehydrogenase [flavocytochrome c] flavoprotein chain	3.046267745	0.198834832	2.410588278	0.082483946	0.934261884	0.999978546
2.3.1.109	arginine N-succinyltransferase	0.096307101	-0.778791352	4.138114321	-0.188199574	0.850720206	0.999978546
2.3.2.2	gamma-glutamyltranspeptidase / glutathione hydrolase	29.91949374	1.046816726	0.553318418	1.891888452	0.058505845	0.999978546
3.1.2.12	S-formylglutathione hydrolase	1.707218919	-2.946056424	2.994608733	-0.98378676	0.32522037	0.999978546
3.4.15.5	peptidyl-dipeptidase Dcp	258.6791263	-0.088921107	0.260693001	-0.341095107	0.733031985	0.999978546
2.7.14.1	protein arginine kinase	100.4784369	0.342041083	0.518047058	0.66025099	0.509092775	0.999978546
2.1.1.196	cobalt-precorrin-6B (C15)-methyltransferase	4.720469386	0.570676073	1.155195403	0.494008261	0.621300345	0.999978546
5.4.2.3	phosphoacetylglucosamine mutase	0.208845794	-0.335395949	4.141001917	-0.080993913	0.935446793	0.999978546
3.5.1.116	ureidoglycolate amidohydrolase	0.579608174	-0.7673027	4.135794128	-0.185527296	0.852815473	0.999978546
2.8.3.25	bile acid CoA-transferase	19.80063959	-0.228588698	0.682279583	-0.335036696	0.737597405	0.999978546
6.5.1.7	DNA ligase 1	16.24694701	0.644561848	0.880509676	0.732032669	0.464148628	0.999978546
3.1.1.4	secretory phospholipase A2	6.442157471	-0.974963702	1.091394475	-0.893319258	0.371686228	0.999978546
2.1.1.227	23S rRNA (cytidine1920-2'-O)/16S rRNA (cytidine1409-2'-O)-me	278.8082903	0.233944061	0.294999439	0.793032221	0.42775905	0.999978546
1.5.3.-	#N/A	0.818610359	-1.721077788	4.114357548	-0.418310214	0.675720323	0.999978546
1.4.5.1	D-amino-acid dehydrogenase	5.40917928	-2.934746527	2.481310822	-1.182740389	0.236912052	0.999978546
1.17.1.4	xanthine dehydrogenase molybdenum-binding subunit	289.3415095	0.180975799	0.39586934	0.457160433	0.647555736	0.999978546
3.4.11.10	bacterial leucyl aminopeptidase	5.455731727	1.448920208	1.306631937	1.10889698	0.267474627	0.999978546
5.3.1.14	L-rhamnose isomerase	112.3655337	0.23556079	0.27913166	0.843905669	0.39872212	0.999978546
3.4.22.41	cathepsin F	2.347625857	-0.302479801	4.099502525	-0.073784514	0.94118185	0.999978546
1.1.1.267	1-deoxy-D-xylulose-5-phosphate reductoisomerase	366.5098855	-0.035992812	0.142926622	-0.251827209	0.801174625	0.999978546
2.3.1.79	maltose O-acetyltransferase	148.2896748	0.038742084	0.251608166	0.153977847	0.877627203	0.999978546
2.3.1.82	aminoglycoside 6'-N-acetyltransferase	5.843599705	1.158352009	1.450419438	0.798632436	0.424503574	0.999978546
3.1.4.52	c-di-GMP phosphodiesterase	50.01167139	-0.84416765	0.819301119	-1.030350906	0.30284531	0.999978546
2.5.1.97	pseudaminic acid synthase	27.63825338	-0.370483457	0.481669537	-0.769165223	0.441795233	0.999978546
2.8.2.22	arylsulfate sulfotransferase	15.39709927	-0.464692413	0.610589161	-0.761055783	0.446623749	0.999978546
3.5.4.26	diaminohydroxyphosphoribosylaminopyrimidine deaminase	165.0856682	-0.296191885	0.32019975	-0.925022224	0.354954348	0.999978546
2.7.6.3	2-amino-4-hydroxy-6-hydroxymethyldihydropteridine diphosph	143.2206681	-0.286621001	0.214664482	-1.335204583	0.181809397	0.999978546
2.3.1.118	N-hydroxyarylamine O-acetyltransferase	9.67675098	-0.667254677	1.324323145	-0.503845817	0.614369728	0.999978546
5.1.3.22	L-ribulose-5-phosphate 3-epimerase	5.033868584	-1.018606829	1.488137355	-0.684484417	0.493669326	0.999978546
2.3.2.16	peptidoglycan pentaglycine glycine transferase (the first glycine	0.052431119	-0.554254637	4.139270063	-0.13390154	0.893480432	0.999978546
5.99.1.2	#N/A	1730.88601	-0.159433806	0.375241971	-0.424882659	0.670922216	0.999978546
2.1.1.90	methanol---5-hydroxybenzimidazolylcobamide Co-methyltransf	10.03781092	1.134570131	1.529421914	0.741829393	0.458190709	0.999978546
3.4.19.11	g-D-glutamyl-meso-diaminopimelate peptidase	66.39931612	-0.214849245	0.486900851	-0.441258717	0.65902571	0.999978546
2.4.99.18	dolichyl-diphosphooligosaccharide---protein glycosyltransferase	15.30748849	1.007225681	1.157379031	0.870264325	0.38415597	0.999978546
2.6.1.110	dTDP-4-dehydro-2,3,6-trideoxy-D-glucose 4-aminotransferase	0.598486539	-1.802306596	4.118950517	-0.437564518	0.661701997	0.999978546
2.7.1.201	PTS system, trehalose-specific IIA component	10.57361883	-1.168633598	0.808032535	-1.446270475	0.148101354	0.999978546
1.1.1.57	fructuronate reductase	269.3151576	0.249583911	0.311576685	0.801035259	0.423111234	0.999978546
3.5.1.-	#N/A	1932.988082	0.442681702	0.895049586	0.494589025	0.620890251	0.999978546
1.7.1.7	GMP reductase	3.492030991	-0.202408011	1.184512848	-0.170878696	0.864319148	0.999978546
2.7.1.23	NAD+ kinase	358.9369047	0.102407415	0.251790011	0.406717542	0.684215455	0.999978546
1.4.1.1	alanine dehydrogenase	179.0955202	-0.285400265	0.325143946	-0.877765889	0.380070779	0.999978546
2.4.1.15	trehalose 6-phosphate synthase	28.28681294	-0.348482767	0.464685011	-0.749933306	0.453294874	0.999978546
2.7.7.-	#N/A	701.1796075	-0.083008935	0.201793149	-0.411356556	0.680811105	0.999978546
2.4.99.15	3-deoxy-D-manno-octulosonic-acid transferase	102.8745696	-0.135612171	0.327661726	-0.413878582	0.67896303	0.999978546
1.17.7.3	(E)-4-hydroxy-3-methylbut-2-enyl-diphosphate synthase	384.5079502	0.029406116	0.172756758	0.170216876	0.86483958	0.999978546
5.4.99.25	tRNA pseudouridine55 synthase	364.7005709	0.124285292	0.23637976	0.525786524	0.599036539	0.999978546
2.7.1.163	hygromycin-B 4-O-kinase	0.732726104	0.035159683	4.110380836	0.008553875	0.993175079	0.999978546
2.7.1.76	deoxyadenosine kinase	0.485024588	0.81742177	4.118343895	0.198483126	0.842667086	0.999978546
3.6.1.9	ectonucleotide pyrophosphatase/phosphodiesterase family me	9.120822582	-1.196058852	1.190984784	-1.004260397	0.315253117	0.999978546
1.13.11.16	2,3-dihydroxyphenylpropionate 1,2-dioxygenase	0.620913234	-3.027568778	4.103607272	-0.737782292	0.460646756	0.999978546
2.5.1.101	N,N'-diacetyllegionaminate synthase	17.20160115	-0.307507719	0.477369567	-0.644171183	0.519464435	0.999978546
2.7.7.76	molybdenum cofactor cytidylyltransferase	2517.858965	1.851511543	1.284421684	1.441513769	0.14943959	0.999978546
3.2.2.27	uracil-DNA glycosylase	169.0721726	-0.064341223	0.231004484	-0.278528027	0.780607053	0.999978546
1.1.1.308	sulfopropanediol 3-dehydrogenase	0.04941368	0.09441837	4.143731151	0.022785834	0.981821108	0.999978546
2.3.3.1	citrate synthase	736.3133749	-0.051977101	0.199362143	-0.260717007	0.79431075	0.999978546
3.1.26.11	ribonuclease Z	239.7056231	0.227058639	0.34379081	0.660455814	0.508961365	0.999978546
1.2.1.38	N-acetyl-gamma-glutamyl-phosphate reductase	221.1370935	-0.259351016	0.316193328	-0.82022925	0.41208543	0.999978546
2.3.1.245	3-hydroxy-5-phosphonooxypentane-2,4-dione thiolase	50.11152848	-0.138083974	0.763854729	-0.180772559	0.856546103	0.999978546
3.1.25.-	#N/A	0.161565365	-0.749127199	4.134214754	-0.181201811	0.856209173	0.999978546
4.1.2.52	4-hydroxy-2-oxoheptanedioate aldolase	10.54245838	0.349030063	0.786853576	0.443576891	0.657348516	0.999978546
3.4.17.4	Gly-Xaa carboxypeptidase	0.224781176	1.406960679	4.099604588	0.34319424	0.731452338	0.999978546
2.7.1.105	6-phosphofructo-2-kinase	0.267820405	0.094407394	4.143731151	0.022783185	0.981823221	0.999978546
5.1.2.3	3-hydroxyacyl-CoA dehydrogenase / enoyl-CoA hydratase / 3-h	0.670887678	-2.544164653	4.112709254	-0.618610384	0.536173059	0.999978546
2.5.1.49	O-acetylhomoserine (thiol)-lyase	620.572906	-0.124717407	0.247096948	-0.504730667	0.613748018	0.999978546
4.2.1.2	#N/A	786.4071845	0.073500222	0.209596577	0.350674726	0.725832389	0.999978546
3.11.1.1	phosphonoacetaldehyde hydrolase	23.83046873	-0.749603855	0.728831141	-1.028501409	0.303714029	0.999978546
1.8.98.1	#N/A	514.6685068	0.7976368	0.494090397	1.614353981	0.106450667	0.999978546
3.4.11.4	tripeptide aminopeptidase	365.2843562	0.260938067	0.177141367	1.473049866	0.14073759	0.999978546
1.5.3.19	4-methylaminobutanoate oxidase (formaldehyde-forming)	0.655665518	-1.80829333	4.122264066	-0.438665088	0.660904227	0.999978546

2.7.1.193	PTS system, N-acetylglucosamine-specific IIA component	629.4791252	-0.038204567	0.488195325	-0.078256725	0.93762384	0.999978546
2.7.1.95	kanamycin kinase	6.400739295	-1.359124042	1.494753638	-0.90926291	0.363211364	0.999978546
4.1.1.22	histidine decarboxylase	0.451163486	0.121130093	4.1145997	0.029439095	0.976514393	0.999978546
4.1.1.35	UDP-glucuronate decarboxylase	71.42773928	-0.080510647	0.302748531	-0.265932413	0.790291262	0.999978546
1.3.8.-	#N/A	0.089381093	1.708948656	4.106035225	0.41620409	0.677260667	0.999978546
4.2.1.12	phosphogluconate dehydratase	0.541914096	-2.757111545	4.106462266	-0.671407982	0.501960661	0.999978546
3.4.11.9	Xaa-Pro aminopeptidase	757.7307781	0.095697497	0.235242329	0.406803899	0.684152023	0.999978546
4.1.2.-	#N/A	1.095079884	1.354223715	2.603845302	0.520086087	0.603003575	0.999978546
3.2.2.-	#N/A	321.9782873	0.300762116	0.429846415	0.699696695	0.484116741	0.999978546
1.1.1.133	dTDP-4-dehydrorhamnose reductase	412.266012	0.016727202	0.187358077	0.089279324	0.928859926	0.999978546
2.1.1.152	precorrin-6A synthase	0.021925243	0.094418168	4.143731151	0.022785785	0.981821147	0.999978546
2.6.99.2	pyridoxine 5-phosphate synthase	45.90984814	-0.144256192	0.471588559	-0.305894172	0.759685223	0.999978546
1.2.1.79	succinate-semialdehyde dehydrogenase / glutarate-semialdehy	8.343123664	0.202987017	1.097048285	0.185030157	0.853205382	0.999978546
1.2.7.4	#N/A	735.1758215	-0.243631627	0.641461245	-0.379807243	0.704088506	0.999978546
1.20.4.3	mycoredoxin	0.089723976	-0.795848752	4.137904308	-0.192331357	0.847482657	0.999978546
2.5.1.75	tRNA dimethylallyltransferase	316.1405562	-0.00320522	0.222631244	-0.01439699	0.988513261	0.999978546
4.2.3.1	threonine synthase	417.3788552	-0.334034221	0.171967242	-1.942429363	0.052085143	0.999978546
2.7.11.21	polo-like kinase 1	0.56438504	-1.96250614	4.111428006	-0.477329565	0.633127462	0.999978546
1.3.1.14	dihydroorotate dehydrogenase (NAD+) catalytic subunit	341.9876951	-0.111764979	0.189237532	-0.590606832	0.554783886	0.999978546
1.1.1.290	erythronate-4-phosphate dehydrogenase	70.05388707	-0.381948391	0.482251885	-0.792010156	0.428354755	0.999978546
1.2.1.31	L-aminoadipate-semialdehyde dehydrogenase	0.034954079	-0.421359027	4.140131853	-0.101774301	0.918935824	0.999978546
4.2.1.79	2-methylcitrate dehydratase	0.582300256	-1.198470239	3.928412648	-0.305077482	0.760307141	0.999978546
2.3.1.51	1-acyl-sn-glycerol-3-phosphate acyltransferase	528.0808745	-0.112190757	0.227053887	-0.494115112	0.621224886	0.999978546
3.5.2.3	dihydroorotase	396.1747044	-0.180073794	0.14012317	-1.285110765	0.19875358	0.999978546
2.1.1.250	trimethylamine---corrinoid protein Co-methyltransferase	2.063895754	0.839541917	2.088322434	0.402017382	0.687671234	0.999978546
2.7.1.180	FAD:protein FMN transferase	253.8645661	0.044703528	0.280041283	0.159631921	0.873171032	0.999978546
6.3.4.22	tRNA(Ile2)-agmatinylcytidine synthase	2.007550506	3.576363863	2.577355233	1.387609988	0.165255838	0.999978546
1.6.1.2	H+-translocating NAD(P) transhydrogenase	18.93975992	-0.108490586	0.724147672	-0.149818318	0.880907957	0.999978546
3.2.1.50	alpha-N-acetylglucosaminidase	95.82601796	-0.215765595	0.353228313	-0.610838902	0.541306237	0.999978546
2.1.1.216	tRNA (guanine26-N2/guanine27-N2)-dimethyltransferase	0.68372251	1.884001795	4.103983205	0.459066644	0.646186309	0.999978546
2.4.2.4	thymidine phosphorylase	11.4572742	-2.650916628	1.194325226	-2.2195936	0.026446367	0.999978546
6.1.1.16	#N/A	514.6442691	0.212981292	0.155566203	1.369071731	0.170976854	0.999978546
6.3.4.6	urea carboxylase	6.97224277	-1.095719547	2.019748533	-0.542502955	0.587472072	0.999978546
1.1.1.193	5-amino-6-(5-phosphoribosylamino)uracil reductase	165.0856682	-0.296191885	0.32019975	-0.925022224	0.354954348	0.999978546
6.3.2.43	[lysine-biosynthesis-protein LysW]---L-2-aminoadipate ligase	0.050191057	-0.266239077	4.143731151	-0.06425105	0.948770329	0.999978546
4.2.1.25	L-arabonate dehydrase	0.031664897	0.094413212	4.143731151	0.022784589	0.981822101	0.999978546
6.3.4.19	tRNA(Ile)-lysidine synthase	465.8400504	0.379301314	0.175554344	2.160592018	0.030726869	0.999978546
3.6.3.15	#N/A	523.0703408	0.137756725	0.282873639	0.486990326	0.626265187	0.999978546
6.3.2.38	N2-citryl-N6-acetyl-N6-hydroxylysine synthase	0.337074854	-1.416842765	4.127192887	-0.343294535	0.731376891	0.999978546
2.7.7.66	phosphoribosyl-dephospho-CoA transferase	0.948413065	1.110312568	2.929707308	0.378984128	0.704699651	0.999978546
2.3.1.251	lipid IVA palmitoyltransferase	12.68719458	1.547012765	1.552854102	0.996238322	0.319134364	0.999978546
2.1.1.201	demethylmenaquinone methyltransferase / 2-methoxy-6-polyp	59.19781542	-0.255657624	0.38291209	-0.667666626	0.504346418	0.999978546
3.5.4.4	adenosine deaminase	49.88921396	-0.342145403	0.437812253	-0.781488871	0.434515019	0.999978546
1.2.1.11	aspartate-semialdehyde dehydrogenase	391.8091547	-0.052509398	0.184066803	-0.285273593	0.775434542	0.999978546
3.2.1.55	alpha-N-arabinofuranosidase	304.048897	-0.076827606	0.292823904	-0.262367944	0.793037784	0.999978546
1.1.1.215	glyoxylate/hydroxypyruvate/2-ketogluconate reductase	1.070230206	-0.528216181	4.105448479	-0.128662236	0.897624918	0.999978546
4.2.1.114	methanogen homoaconitase large subunit	10.62242811	2.19541706	1.675831974	1.310046051	0.190180257	0.999978546
2.7.9.1	pyruvate, orthophosphate dikinase	3366.086176	-0.055767867	0.495767865	-0.112487861	0.910436594	0.999978546
6.2.1.14	6-carboxyhexanoate--CoA ligase	1.997081838	0.507155623	2.186323106	0.231967371	0.816563357	0.999978546
2.3.1.179	3-oxoacyl-[acyl-carrier-protein] synthase II	527.6690916	-0.319625566	0.208131097	-1.535693464	0.124613579	0.999978546
2.3.1.203	UDP-N-acetylbacillosamine N-acetyltransferase	1.434965331	-2.279310843	2.903744144	-0.784955812	0.432479477	0.999978546
2.4.2.12	nicotinamide phosphoribosyltransferase	17.36203353	-0.527658046	1.161023432	-0.454476655	0.649485791	0.999978546
3.6.1.22	NAD+ diphosphatase	138.749748	0.074805268	0.291628284	0.256508961	0.797557862	0.999978546
3.2.1.70	glucan 1,6-alpha-glucosidase	14.70370541	-1.390724528	0.742971743	-1.871840404	0.061228687	0.999978546
2.1.4.1	glycine amidinotransferase	45.52497431	-0.529407827	0.902662481	-0.586495881	0.557542329	0.999978546
3.3.1.1	adenosylhomocysteinase	126.059348	-0.3020248	0.289268458	-1.044098627	0.296439761	0.999978546
2.7.1.140	inositol-polyphosphate multikinase	0.160692243	0.094410072	4.143731151	0.022783831	0.981822705	0.999978546
2.3.1.231	tRNA wybutosine-synthesizing protein 4	0.053564081	0.094415333	4.143731151	0.022785101	0.981821693	0.999978546
2.1.1.133	precorrin-4/cobalt-precorrin-4 C11-methyltransferase	110.7485461	0.037708156	0.349953931	0.107751772	0.9141926	0.999978546
1.1.1.346	2,5-diketo-D-gluconate reductase A	10.87598757	2.029183732	1.148931923	1.766147925	0.077371035	0.999978546
6.1.1.3	threonyl-tRNA synthetase	799.6942148	-0.218709424	0.19956112	-1.09595208	0.273099746	0.999978546
4.4.1.11	methionine-gamma-lyase	55.61284014	-0.697119406	0.524493176	-1.329129601	0.183805216	0.999978546
6.3.2.6	phosphoribosylaminoimidazole carboxylase / phosphoribosylan	329.075709	-0.186528308	0.168482927	-1.107105103	0.26824849	0.999978546
1.2.1.88	1-pyrroline-5-carboxylate dehydrogenase	93.26804562	-0.276160556	0.350469137	-0.787973966	0.430711934	0.999978546
3.4.16.4	D-alanyl-D-alanine carboxypeptidase	1095.067042	-0.209608824	0.274411374	-0.763848894	0.444957295	0.999978546
2.8.3.18	succinyl-CoA:acetate CoA-transferase	17.73499046	0.347478592	0.438858173	0.791778788	0.428489673	0.999978546
3.-.-.-	#N/A	8.523949024	1.197498004	1.344928561	0.89038038	0.373261674	0.999978546
4.2.1.45	CDP-glucose 4,6-dehydratase	79.53278265	0.048813171	0.280101752	0.174269426	0.861653726	0.999978546
3.5.4.33	tRNA(adenine34) deaminase	257.7751313	-0.747860992	0.572496336	-1.306315771	0.191445196	0.999978546
3.1.3.83	D-glycero-D-manno-heptose 1,7-bisphosphate phosphatase	19.00358607	-0.041827755	0.473573283	-0.088323722	0.929619385	0.999978546
1.13.11.54	1,2-dihydroxy-3-keto-5-methylthiopentene dioxygenase	0.050191057	-0.266239077	4.143731151	-0.06425105	0.948770329	0.999978546
2.7.2.2	carbamate kinase	210.8375222	-0.042040378	0.35191875	-0.119460468	0.904910558	0.999978546
2.1.1.22	carnosine N-methyltransferase	0	NA	NA	NA	NA	NA
2.3.1.7	carnitine O-acetyltransferase	0	NA	NA	NA	NA	NA
1.14.11.-	#N/A	0	NA	NA	NA	NA	NA
1.1.1.270	3-keto steroid reductase	0	NA	NA	NA	NA	NA
2.3.1.37	5-aminolevulinate synthase	0	NA	NA	NA	NA	NA
3.1.3.99	IMP and pyridine-specific 5'-nucleotidase	0	NA	NA	NA	NA	NA
2.7.8.11	CDP-diacylglycerol--inositol 3-phosphatidyltransferase	0	NA	NA	NA	NA	NA
3.2.1.106	mannosyl-oligosaccharide glucosidase	0	NA	NA	NA	NA	NA
2.4.1.256	alpha-1,2-glucosyltransferase	0	NA	NA	NA	NA	NA
4.1.1.45	aminocarboxymuconate-semialdehyde decarboxylase	0	NA	NA	NA	NA	NA
3.1.1.56	carboxylesterase 2	0	NA	NA	NA	NA	NA
4.1.1.86	L-2,4-diaminobutyrate decarboxylase	0	NA	NA	NA	NA	NA
2.4.99.-	#N/A	0	NA	NA	NA	NA	NA
4.1.3.41	3-hydroxy-D-aspartate aldolase	0	NA	NA	NA	NA	NA
3.6.1.42	guanosine-diphosphatase	0	NA	NA	NA	NA	NA

3.1.22.-	#N/A	0	NA	NA	NA	NA	NA
2.8.3.22	succinyl-CoA:(S)-malate CoA-transferase subunit A	0	NA	NA	NA	NA	NA
2.7.1.182	phytol kinase	0	NA	NA	NA	NA	NA
2.4.1.131	#N/A	0	NA	NA	NA	NA	NA
2.4.2.6	nucleoside deoxyribosyltransferase	0	NA	NA	NA	NA	NA
2.3.1.222	phosphate propanoyltransferase	0	NA	NA	NA	NA	NA
2.7.7.50	mRNA guanylyltransferase	0	NA	NA	NA	NA	NA
1.3.3.11	pyrroloquinoline-quinone synthase	0	NA	NA	NA	NA	NA
3.5.1.110	ureidoacrylate peracid hydrolase	0	NA	NA	NA	NA	NA
4.1.3.43	4-hydroxy-2-oxovalerate/4-hydroxy-2-oxohexanoate aldolase	0	NA	NA	NA	NA	NA
2.7.1.82	ethanolamine kinase	0	NA	NA	NA	NA	NA
2.5.1.85	all-trans-nonaprenyl-diphosphate synthase	0	NA	NA	NA	NA	NA
2.1.1.225	tRNA:m4X modification enzyme	0	NA	NA	NA	NA	NA
3.1.1.97	diphthine methyl ester acylhydrolase	0	NA	NA	NA	NA	NA
4.2.1.134	very-long-chain (3R)-3-hydroxyacyl-CoA dehydratase	0	NA	NA	NA	NA	NA
2.7.1.150	1-phosphatidylinositol-3-phosphate 5-kinase	0	NA	NA	NA	NA	NA
5.4.99.45	tRNA pseudouridine38/39 synthase	0	NA	NA	NA	NA	NA
3.5.3.17	guanidinopropionase	0	NA	NA	NA	NA	NA
2.5.1.58	protein farnesyltransferase subunit beta	0	NA	NA	NA	NA	NA
2.4.1.198	#N/A	0	NA	NA	NA	NA	NA
2.7.8.17	UDP-N-acetylglucosamine-lysosomal-enzyme	0	NA	NA	NA	NA	NA
2.4.1.267	alpha-1,3-glucosyltransferase	0	NA	NA	NA	NA	NA
2.6.1.108	(5-formylfuran-3-yl)methyl phosphate transaminase	0	NA	NA	NA	NA	NA
2.7.4.21	inositol-hexakisphosphate 5-kinase	0	NA	NA	NA	NA	NA
2.6.1.101	L-glutamine:2-deoxy-scyllo-inosose/3-amino-2,3-dideoxy-scyllo	0	NA	NA	NA	NA	NA
3.1.3.43	pyruvate dehydrogenase phosphatase	0	NA	NA	NA	NA	NA
3.6.1.62	mRNA-decapping enzyme subunit 2	0	NA	NA	NA	NA	NA
1.5.1.-	#N/A	0	NA	NA	NA	NA	NA
3.4.24.56	insulysin	0	NA	NA	NA	NA	NA
6.3.4.11	biotin---protein ligase	0	NA	NA	NA	NA	NA
3.1.4.37	2',3'-cyclic-nucleotide 3'-phosphodiesterase	0	NA	NA	NA	NA	NA
1.3.1.33	protochlorophyllide reductase	0	NA	NA	NA	NA	NA
3.4.17.22	carboxypeptidase D	0	NA	NA	NA	NA	NA
1.1.1.372	L-glyceraldehyde reductase	0	NA	NA	NA	NA	NA
1.3.99.23	all-trans-retinol 13,14-reductase	0	NA	NA	NA	NA	NA
1.1.1.292	1,5-anhydro-D-fructose reductase (1,5-anhydro-D-mannitol-for	0	NA	NA	NA	NA	NA
2.3.1.189	mycothiol synthase	0	NA	NA	NA	NA	NA
2.4.1.132	alpha-1,3/alpha-1,6-mannosyltransferase	0	NA	NA	NA	NA	NA
3.4.11.26	intermediate cleaving peptidase 55	0	NA	NA	NA	NA	NA
2.5.1.92	(2Z,6Z)-farnesyl diphosphate synthase	0	NA	NA	NA	NA	NA
2.5.1.68	short-chain Z-isoprenyl diphosphate synthase	0	NA	NA	NA	NA	NA
3.2.1.49	alpha-N-acetylgalactosaminidase	0	NA	NA	NA	NA	NA
6.3.4.9	biotin---protein ligase	0	NA	NA	NA	NA	NA
5.3.3.1	3beta-hydroxy-Delta5-steroid dehydrogenase / steroid Delta-is	0	NA	NA	NA	NA	NA
1.10.3.11	ubiquinol oxidase	0	NA	NA	NA	NA	NA
1.11.1.1	NADH peroxidase	0	NA	NA	NA	NA	NA
5.3.3.10	5-carboxymethyl-2-hydroxymuconate isomerase	0	NA	NA	NA	NA	NA
3.1.3.96	pseudouridine 5'-phosphatase	0	NA	NA	NA	NA	NA
3.6.1.43	dolichyldiphosphatase	0	NA	NA	NA	NA	NA
3.6.1.29	bis(5'-adenosyl)-triphosphatase	0	NA	NA	NA	NA	NA
2.4.1.101	alpha-1,3-mannosyl-glycoprotein beta-1,2-N-acetylglucosaminy	0	NA	NA	NA	NA	NA
2.3.2.17	peptidoglycan pentaglycine glycine transferase (the second and	0	NA	NA	NA	NA	NA
1.14.11.27	F-box and leucine-rich repeat protein 10/11	0	NA	NA	NA	NA	NA
5.4.3.9	glutamate 2,3-aminomutase	0	NA	NA	NA	NA	NA
2.4.1.250	D-inositol-3-phosphate glycosyltransferase	0	NA	NA	NA	NA	NA
6.2.1.16	acetoacetyl-CoA synthetase	0	NA	NA	NA	NA	NA
3.1.1.89	protein phosphatase methylesterase 1	0	NA	NA	NA	NA	NA
6.4.1.5	geranyl-CoA carboxylase alpha subunit	0	NA	NA	NA	NA	NA
2.1.1.183	18S rRNA (adenine1779-N6/adenine1780-N6)-dimethyltransfer	0	NA	NA	NA	NA	NA
5.1.3.37	mannuronan 5-epimerase	0	NA	NA	NA	NA	NA
1.10.2.-	#N/A	0	NA	NA	NA	NA	NA
2.1.1.214	tRNA (guanine10-N2)-methyltransferase	0	NA	NA	NA	NA	NA
3.1.3.66	inositol polyphosphate-4-phosphatase	0	NA	NA	NA	NA	NA
3.7.1.5	acylpyruvate hydrolase	0	NA	NA	NA	NA	NA
4.1.3.25	malyl-CoA/(S)-citramalyl-CoA lyase	0	NA	NA	NA	NA	NA
2.5.1.44	homospermidine synthase	0	NA	NA	NA	NA	NA
2.3.2.18	peptidoglycan pentaglycine glycine transferase (the fourth and	0	NA	NA	NA	NA	NA
3.5.3.23	succinylarginine dihydrolase	0	NA	NA	NA	NA	NA
1.3.1.88	tRNA-dihydrouridine synthase 1	0	NA	NA	NA	NA	NA
3.1.3.77	enolase-phosphatase E1	0	NA	NA	NA	NA	NA
1.1.99.21	D-sorbitol dehydrogenase (acceptor)	0	NA	NA	NA	NA	NA
2.1.1.320	type II protein arginine methyltransferase	0	NA	NA	NA	NA	NA
2.7.7.79	tRNA(His) guanylyltransferase	0	NA	NA	NA	NA	NA
6.2.1.41	HIP---CoA ligase	0	NA	NA	NA	NA	NA
5.1.1.17	isopenicillin-N epimerase	0	NA	NA	NA	NA	NA
6.2.1.34	feruloyl-CoA synthase	0	NA	NA	NA	NA	NA
2.7.4.12	T-even-induced deoxynucleotide kinase	0	NA	NA	NA	NA	NA
2.4.1.257	alpha-1,3/alpha-1,6-mannosyltransferase	0	NA	NA	NA	NA	NA
1.3.1.91	tRNA-dihydrouridine synthase 2	0	NA	NA	NA	NA	NA
2.8.1.9	molybdenum cofactor sulfurtransferase	0	NA	NA	NA	NA	NA
1.3.99.5	#N/A	0	NA	NA	NA	NA	NA
3.5.1.94	gamma-glutamyl-gamma-aminobutyrate hydrolase	0	NA	NA	NA	NA	NA
1.1.1.371	scyllo-inositol 2-dehydrogenase (NADP+)	0	NA	NA	NA	NA	NA
2.1.1.229	alkylated DNA repair protein alkB homolog 8	0	NA	NA	NA	NA	NA
1.14.14.5	alkanesulfonate monooxygenase	0	NA	NA	NA	NA	NA
1.12.1.4	iron-hydrogenase subunit alpha	0	NA	NA	NA	NA	NA
2.4.1.142	beta-1,4-mannosyltransferase	0	NA	NA	NA	NA	NA

2.4.1.258	alpha-1,3-mannosyltransferase	0	NA	NA	NA	NA	NA
2.4.1.241	digalactosyldiacylglycerol synthase	0	NA	NA	NA	NA	NA
1.13.11.15	3,4-dihydroxyphenylacetate 2,3-dioxygenase	0	NA	NA	NA	NA	NA
4.1.2.57	sulfofructosephosphate aldolase	0	NA	NA	NA	NA	NA
1.3.1.6	NADH-dependent fumarate reductase subunit A	0	NA	NA	NA	NA	NA
5.1.2.2	mandelate racemase	0	NA	NA	NA	NA	NA
1.6.99.1	NADPH2 dehydrogenase	0	NA	NA	NA	NA	NA
2.5.1.87	ditrans,polycis-polyprenyl diphosphate synthase	0	NA	NA	NA	NA	NA
2.1.1.202	multisite-specific tRNA:(cytosine-C5)-methyltransferase	0	NA	NA	NA	NA	NA
2.6.1.100	L-glutamine:2-deoxy-scyllo-inosose/3-amino-2,3-dideoxy-scyllo	0	NA	NA	NA	NA	NA
1.6.2.2	cytochrome-b5 reductase	0	NA	NA	NA	NA	NA
3.5.3.7	guanidinobutyrase	0	NA	NA	NA	NA	NA
1.12.5.1	#N/A	0	NA	NA	NA	NA	NA
1.2.1.60	5-carboxymethyl-2-hydroxymuconic-semialdehyde dehydrogen	0	NA	NA	NA	NA	NA
2.1.1.100	protein-S-isoprenylcysteine O-methyltransferase	0	NA	NA	NA	NA	NA
3.6.1.12	dCTP diphosphatase	0	NA	NA	NA	NA	NA
1.1.1.163	cyclopentanol dehydrogenase	0	NA	NA	NA	NA	NA
1.10.9.1	#N/A	0	NA	NA	NA	NA	NA
6.3.1.11	gamma-glutamylputrescine synthase	0	NA	NA	NA	NA	NA
3.6.1.5	apyrase	0	NA	NA	NA	NA	NA
1.1.1.320	benzil reductase ((S)-benzoin forming)	0	NA	NA	NA	NA	NA
3.4.23.1	pepsin A	0	NA	NA	NA	NA	NA
1.1.1.11	D-arabinitol 4-dehydrogenase	0	NA	NA	NA	NA	NA
3.2.1.46	galactosylceramidase	0	NA	NA	NA	NA	NA
3.2.2.3	uridine nucleosidase	0	NA	NA	NA	NA	NA
3.4.11.24	aminopeptidase S	0	NA	NA	NA	NA	NA
2.1.1.321	type III protein arginine methyltransferase	0	NA	NA	NA	NA	NA
2.4.1.265	alpha-1,3-glucosyltransferase	0	NA	NA	NA	NA	NA
3.5.4.-	#N/A	0	NA	NA	NA	NA	NA
2.3.1.225	palmitoyltransferase ZDHHC9/14/18	0	NA	NA	NA	NA	NA
4.2.1.115	UDP-N-acetylglucosamine 4,6-dehydratase	0	NA	NA	NA	NA	NA
1.13.11.1	catechol 1,2-dioxygenase	0	NA	NA	NA	NA	NA
1.1.1.21	aldehyde reductase	0	NA	NA	NA	NA	NA
3.5.5.7	aliphatic nitrilase	0	NA	NA	NA	NA	NA
3.5.4.34	tRNA-specific adenosine deaminase 1	0	NA	NA	NA	NA	NA
3.4.21.112	membrane-bound transcription factor site-1 protease	0	NA	NA	NA	NA	NA
6.3.1.14	diphthine-ammonia ligase	0	NA	NA	NA	NA	NA
2.1.1.287	ribosomal RNA-processing protein 8	0	NA	NA	NA	NA	NA
2.1.1.260	rRNA small subunit pseudouridine methyltransferase Nep1	0	NA	NA	NA	NA	NA
4.2.1.161	bisanhydrobacterioruberin hydratase	0	NA	NA	NA	NA	NA
3.4.22.49	separase	0	NA	NA	NA	NA	NA
2.7.11.13	classical protein kinase C alpha type	0	NA	NA	NA	NA	NA
2.1.1.205	tRNA (cytidine32/guanosine34-2'-O)-methyltransferase	0	NA	NA	NA	NA	NA
3.2.1.130	glycoprotein endo-alpha-1,2-mannosidase	0	NA	NA	NA	NA	NA
4.4.1.2	cystathionine gamma-lyase / homocysteine desulfhydrase	0	NA	NA	NA	NA	NA
3.2.1.155	xyloglucan-specific exo-beta-1,4-glucanase	0	NA	NA	NA	NA	NA
2.4.1.255	protein O-GlcNAc transferase	0	NA	NA	NA	NA	NA
3.5.1.96	succinylglutamate desuccinylase	0	NA	NA	NA	NA	NA
2.5.1.59	protein farnesyltransferase/geranylgeranyltransferase type-1 s	0	NA	NA	NA	NA	NA
2.5.1.84	all-trans-nonaprenyl-diphosphate synthase	0	NA	NA	NA	NA	NA
3.5.1.38	glutamin-(asparagin-)ase	0	NA	NA	NA	NA	NA
3.7.1.3	kynureninase	0	NA	NA	NA	NA	NA
2.3.1.78	heparan-alpha-glucosaminide N-acetyltransferase	0	NA	NA	NA	NA	NA
2.7.1.91	sphingosine kinase	0	NA	NA	NA	NA	NA
2.1.3.9	N-acetylornithine carbamoyltransferase	0	NA	NA	NA	NA	NA
3.5.4.30	dCTP deaminase (dUMP-forming)	0	NA	NA	NA	NA	NA
6.2.1.31	2-furoate---CoA ligase	0	NA	NA	NA	NA	NA
5.1.3.10	CDP-paratose 2-epimerase	0	NA	NA	NA	NA	NA
1.5.99.6	spermidine dehydrogenase	0	NA	NA	NA	NA	NA
3.5.1.11	penicillin amidase	0	NA	NA	NA	NA	NA
2.7.1.32	choline kinase	0	NA	NA	NA	NA	NA
2.7.1.157	N-acetylgalactosamine kinase	0	NA	NA	NA	NA	NA
1.1.1.51	aldo-keto reductase family 1 member C3	0	NA	NA	NA	NA	NA
1.6.3.4	NADH oxidase (H2O-forming)	0	NA	NA	NA	NA	NA
3.4.24.27	thermolysin	0	NA	NA	NA	NA	NA
1.14.13.81	magnesium-protoporphyrin IX monomethyl ester (oxidative) cy	0	NA	NA	NA	NA	NA
4.1.99.20	3-amino-4-hydroxybenzoic acid synthase	0	NA	NA	NA	NA	NA
2.7.7.52	terminal uridylyltransferase	0	NA	NA	NA	NA	NA
1.1.1.264	L-idonate 5-dehydrogenase	0	NA	NA	NA	NA	NA
2.7.8.29	phosphatidylserine synthase 2	0	NA	NA	NA	NA	NA
2.4.1.87	N-acetyllactosaminide 3-alpha-galactosyltransferase	0	NA	NA	NA	NA	NA
1.8.-.-	#N/A	0	NA	NA	NA	NA	NA
3.1.3.67	phosphatidylinositol-3,4,5-trisphosphate 3-phosphatase and du	0	NA	NA	NA	NA	NA
2.4.1.287	rhamnopyranosyl-N-acetylglucosaminyl-diphospho-decaprenol	0	NA	NA	NA	NA	NA
2.9.1.2	O-phospho-L-seryl-tRNASec:L-selenocysteinyl-tRNA synthase	0	NA	NA	NA	NA	NA
2.8.3.20	succinyl-CoA---D-citramalate CoA-transferase	0	NA	NA	NA	NA	NA
1.1.1.108	carnitine 3-dehydrogenase	0	NA	NA	NA	NA	NA
2.7.1.137	phosphatidylinositol 3-kinase	0	NA	NA	NA	NA	NA
6.3.4.10	biotin---protein ligase	0	NA	NA	NA	NA	NA
3.1.1.84	carboxylesterase 2	0	NA	NA	NA	NA	NA
4.1.1.77	2-oxo-3-hexenedioate decarboxylase	0	NA	NA	NA	NA	NA
2.5.1.60	geranylgeranyl transferase type-2 subunit beta	0	NA	NA	NA	NA	NA
3.4.21.72	IgA-specific serine endopeptidase	0	NA	NA	NA	NA	NA
1.3.5.5	15-cis-phytoene desaturase	0	NA	NA	NA	NA	NA
4.1.3.16	#N/A	0	NA	NA	NA	NA	NA
1.1.1.338	(2R)-3-sulfolactate dehydrogenase (NADP+)	0	NA	NA	NA	NA	NA
3.5.1.89	N-acetylglucosaminylphosphatidylinositol deacetylase	0	NA	NA	NA	NA	NA

1.2.99.8	glyceraldehyde dehydrogenase large subunit	0	NA	NA	NA	NA	NA

Table S17: Enzymatic Functions (EC_DGE), Enhanced Diet, Mid in Mission

	Description	baseMean	log2FoldChange	lfcSE	stat	pvalue	padj
2.7.7.68	2-phospho-L-lactate guanylyltransferase	2.105625982	-18.1202954	2.99685753	-6.046432044	1.48E-09	2.94E-06
1.8.4.14	L-methionine (R)-S-oxide reductase	1.203925453	-16.97676423	4.12965914	-4.110935951	3.94E-05	0.039090604
4.2.2.1	hyaluronate lyase	29.93586609	-2.776806317	0.805843187	-3.445839542	0.000569288	0.376489317
5.1.1.20	L-Ala-D/L-Glu epimerase	82.99796834	1.20386401	0.362703541	3.319140494	0.00090295	0.447863139
2.7.1.198	PTS system, glucitol/sorbitol-specific IIA component	25.02906499	-3.562111616	1.096418104	-3.248862456	0.001158675	0.459762196
1.1.1.271	GDP-L-fucose synthase	181.2243692	0.662575213	0.218325375	3.034806246	0.002406903	0.79588245
5.3.1.29	ribose 1,5-bisphosphate isomerase	0.633492245	-1.816504523	3.186956267	-0.569981001	0.568690584	0.999551235
4.4.1.25	L-cysteate sulfo-lyase	0.961780025	-0.326762298	3.494683911	-0.093502676	0.925504224	0.999551235
3.2.1.99	arabinan endo-1,5-alpha-L-arabinosidase	192.2445618	-0.177212247	0.362014509	-0.489516975	0.62447574	0.999551235
2.8.1.7	cysteine desulfurase	1018.936717	-0.023665408	0.180362139	-0.131210507	0.895608785	0.999551235
3.4.22.-	#N/A	1.269924312	-3.362113359	2.903375117	-1.158001713	0.246863337	0.999551235
2.5.1.9	#N/A	198.0755275	-0.290004257	0.594530586	-0.487786942	0.625700761	0.999551235
3.2.1.22	alpha-galactosidase	849.0917731	-0.116425677	0.188422707	-0.617896212	0.536643756	0.999551235
5.3.1.17	4-deoxy-L-threo-5-hexosulose-uronate ketol-isomerase	320.2434146	-0.390622549	0.394962155	-0.989012603	0.322656976	0.999551235
3.2.1.132	chitosanase	0.19424192	-0.413011202	4.109584917	-0.100499493	0.919947785	0.999551235
6.3.2.26	N-(5-amino-5-carboxypentanoyl)-L-cysteinyl-D-valine synthase	0.04941368	-0.391882467	4.143731151	-0.094572368	0.924654499	0.999551235
3.1.3.18	phosphoglycolate phosphatase	615.2274367	-0.618222282	0.416930813	-1.482793457	0.138129295	0.999551235
1.3.8.6	glutaryl-CoA dehydrogenase	0.043850487	0.733944445	4.133023226	0.177580528	0.859052418	0.999551235
1.1.1.44	6-phosphogluconate dehydrogenase	235.2879129	0.107780306	0.401544174	0.268414568	0.788380227	0.999551235
6.1.1.14	#N/A	786.3440721	-0.111061011	0.326386646	-0.340274373	0.733649914	0.999551235
2.5.1.47	cysteine synthase	467.5769601	-0.273274213	0.341205164	-0.800908785	0.423184454	0.999551235
6.3.2.7	UDP-N-acetylmuramoyl-L-alanyl-D-glutamate-L-lysine ligase	3.344305032	0.920224409	1.549040936	0.594060743	0.552471486	0.999551235
3.5.2.10	creatinine amidohydrolase	54.4423082	-0.151245662	0.322967154	-0.468300446	0.639569749	0.999551235
4.2.2.20	chondroitin-sulfate-ABC endolyase/exolyase	72.26463302	-0.007482466	0.527221552	-0.014192261	0.988676594	0.999551235
1.2.3.3	pyruvate oxidase	1.435492674	-1.156933209	3.08111011	-0.375492328	0.707294351	0.999551235
1.1.1.6	glycerol dehydrogenase	111.0345153	-0.51435376	0.418963274	-1.227682214	0.219566286	0.999551235
2.7.7.70	D-beta-D-heptose 7-phosphate kinase / D-beta-D-heptose 1-ph	16.49374106	-0.272558788	0.666665535	-0.408838876	0.682657908	0.999551235
6.1.1.11	seryl-tRNA synthetase	603.6102613	-0.025146249	0.152373309	-0.165030539	0.868919945	0.999551235
3.1.1.41	cephalosporin-C deacetylase	11.56161355	-0.103993895	1.010646608	-0.102898376	0.918043626	0.999551235
2.5.1.39	4-hydroxybenzoate polyprenyltransferase	8.366356724	0.256100926	0.835105144	0.30666908	0.759095267	0.999551235
2.6.1.37	2-aminoethylphosphonate-pyruvate transaminase	24.00016452	0.640147043	0.584554222	1.095102933	0.273471544	0.999551235
1.14.19.3	linoleoyl-CoA desaturase	0.048153551	-0.752542723	4.143731151	-0.18160993	0.855888856	0.999551235
2.1.1.-	#N/A	2085.570337	-1.048026443	0.492270819	-2.128963167	0.033257308	0.999551235
1.5.1.43	carboxynorspermidine synthase	0.02470684	-0.391881853	4.143731151	-0.09457222	0.924654616	0.999551235
2.3.3.13	2-isopropylmalate synthase	404.3772946	-0.303140158	0.192123322	-1.577841537	0.114602019	0.999551235
3.5.1.28	N-acetylmuramoyl-L-alanine amidase	701.2008379	0.050022931	0.158519678	0.315562911	0.752334293	0.999551235
2.4.2.3	uridine phosphorylase	208.4116149	-0.043999594	0.36309718	-0.12117856	0.903549605	0.999551235
3.4.19.5	beta-aspartyl-peptidase (threonine type)	13.69378794	0.140883237	0.431063493	0.326827114	0.743798651	0.999551235
1.15.1.2	superoxide reductase	676.2410151	-0.76103902	0.606874538	-1.254030236	0.209831016	0.999551235
2.4.1.20	cellobiose phosphorylase	381.5764817	-0.14265473	0.410827392	-0.347237629	0.728412803	0.999551235
3.1.11.6	#N/A	653.3635538	-0.094324542	0.195490129	-0.482502836	0.629448784	0.999551235
1.3.1.106	precorrin-6A/cobalt-precorrin-6A reductase	61.18763666	0.529766801	0.331413302	1.598507958	0.109929976	0.999551235
2.8.4.5	threonylcarbamoyladenosine tRNA methylthiotransferase CDK/	414.9569557	0.893237243	0.364045436	2.453642193	0.014141762	0.999551235
2.7.7.23	UDP-N-acetylglucosamine/UDP-N-acetylgalactosamine diphosp	454.2087906	-0.543989643	0.3565678	-1.525627504	0.127102659	0.999551235
4.1.1.23	orotidine-5'-phosphate decarboxylase	397.5941849	0.010186202	0.138640911	0.073471834	0.941430657	0.999551235
2.7.4.2	phosphomevalonate kinase	2.382155935	-0.57885573	1.819017084	-0.31822446	0.75031468	0.999551235
3.5.1.54	allophanate hydrolase	0.281440112	1.27885412	4.118578375	0.310508628	0.756174199	0.999551235
4.2.1.49	urocanate hydratase	239.7870597	0.142756955	0.290918406	0.490711321	0.623630638	0.999551235
2.4.1.44	UDP-D-galactose:(glucosyl)LPS alpha-1,3-D-galactosyltransferas	0.029793698	-0.391883699	4.143731151	-0.094572665	0.924654262	0.999551235
4.2.1.42	galactarate dehydratase	7.014879709	0.551870763	0.87667302	0.629505815	0.529017963	0.999551235
2.5.1.26	alkyldihydroxyacetonephosphate synthase	21.86515454	0.665222644	0.813548931	0.817679944	0.413539965	0.999551235
2.7.7.24	glucose-1-phosphate thymidylyltransferase	374.5950283	-0.054627803	0.17820614	-0.306542763	0.759191425	0.999551235
5.4.99.60	precorrin-8X/cobalt-precorrin-8 methylmutase	99.4358351	0.541584838	0.298428304	1.814790457	0.069556118	0.999551235
2.4.2.19	nicotinate-nucleotide pyrophosphorylase (carboxylating)	205.1715531	-0.15976333	0.245013978	-0.652058021	0.514363744	0.999551235
2.1.1.187	23S rRNA (guanine745-N1)-methyltransferase	118.7001715	0.055874786	0.334974801	0.166802952	0.867525096	0.999551235
3.4.11.18	methionyl aminopeptidase	1322.739451	-0.202163495	0.3872252	-0.522082485	0.601612901	0.999551235
2.1.1.204	tRNA (cytosine38-C5)-methyltransferase	0.015832449	0.284417857	4.139099958	0.068714904	0.945216554	0.999551235
1.2.1.43	#N/A	5.547997314	-0.719722688	0.910873771	-0.790145365	0.429442879	0.999551235
2.7.1.66	undecaprenol kinase	2.560303601	-3.076585223	2.177416018	-1.412952416	0.157669716	0.999551235
2.6.1.1	aspartate aminotransferase, chloroplastic	444.3986427	0.088154461	0.252137299	0.349628798	0.726617296	0.999551235
4.3.1.19	threonine dehydratase	237.8157219	-0.248300052	0.247922682	-1.001522128	0.316574448	0.999551235
3.5.1.10	formyltetrahydrofolate deformylase	26.97695206	0.399936263	0.744561059	0.537144158	0.59116804	0.999551235
3.7.1.12	cobalt-precorrin 5A hydrolase	120.4272526	-0.137595791	0.364601946	-0.377386332	0.705886522	0.999551235
1.1.1.62	17beta-estradiol 17-dehydrogenase	0.374948568	-0.391895253	4.143731151	-0.094575454	0.924652048	0.999551235
1.3.3.4	#N/A	5.486609838	0.43791321	0.891175397	0.491388353	0.6231518	0.999551235
3.5.4.16	GTP cyclohydrolase IA	118.9073355	0.407423978	0.353329739	1.153098459	0.248869974	0.999551235
2.1.1.200	tRNA (cytidine32/uridine32-2'-O)-methyltransferase	1.277044293	-1.976245379	4.054258685	-0.487449256	0.625939995	0.999551235
5.3.1.6	#N/A	448.1376431	-0.112584996	0.216937228	-0.51897499	0.603778182	0.999551235
1.1.1.335	UDP-N-acetyl-2-amino-2-deoxyglucuronate dehydrogenase	1.283507249	1.374666477	2.203727448	0.623791512	0.532764515	0.999551235
1.1.1.350	ureidoglycolate dehydrogenase (NAD+)	0.461004988	-0.314020192	4.034104742	-0.077841358	0.937954247	0.999551235
2.3.1.181	lipoyl(octanoyl) transferase	68.98595382	0.559794075	0.41491393	1.3491812	0.177278772	0.999551235
1.2.1.18	Oxidoreductases	18.30084634	0.132965987	0.487632871	0.272676423	0.78510196	0.999551235
2.1.1.104	caffeoyl-CoA O-methyltransferase	0.214256324	-0.391892261	4.143731151	-0.094574732	0.924652621	0.999551235
5.4.2.8	phosphomannomutase	164.6440248	0.280930199	0.266080124	1.055810538	0.291054783	0.999551235
1.2.99.5	#N/A	122.8406003	0.5514528	1.130370493	0.487851376	0.625655118	0.999551235
2.7.1.191	PTS system, mannose-specific IIA component	129.4912911	-0.702903161	0.463399612	-1.516840202	0.129307034	0.999551235
2.5.1.63	adenosyl-fluoride synthase	1.48293681	-0.523507691	1.755692625	-0.298177303	0.765567844	0.999551235
2.8.3.-	#N/A	28.61882816	-0.075850053	0.731549785	-0.103684062	0.917420075	0.999551235
3.6.4.12	DNA replication licensing factor MCM5	5705.400828	-0.225202398	0.254345754	-0.885418353	0.375931037	0.999551235
1.3.99.32	glutaryl-CoA dehydrogenase (non-decarboxylating)	1.134042721	-0.608459195	4.137798831	-0.147049003	0.883093342	0.999551235
1.12.98.1	#N/A	212.01706	-2.303592834	1.352377145	-1.703365694	0.088499654	0.999551235
5.4.99.26	tRNA pseudouridine65 synthase	2.537247887	0.867011878	1.898321674	0.45672548	0.647868374	0.999551235
1.4.1.2	glutamate dehydrogenase	10.5705985	-1.297582512	1.057781673	-1.226701639	0.219934752	0.999551235
3.1.21.1	deoxyribonuclease I	2.200241027	1.144224087	1.65555149	0.691143763	0.489475201	0.999551235
2.7.7.83	UDP-N-acetylglucosamine/UDP-N-acetylgalactosamine diphosp	64.34672785	-0.749185042	0.563077217	-1.330519187	0.183347269	0.999551235

2.6.1.62	adenosylmethionine---8-amino-7-oxononanoate aminotransfer	70.13286159	0.147824563	0.559832224	0.264051543	0.791740202	0.999551235
2.4.1.58	UDP-glucose:(galactosyl)LPS alpha-1,2-glucosyltransferase	0.173954458	-1.369305492	4.133490734	-0.331270972	0.740439816	0.999551235
1.8.3.2	thiol oxidase	0.321384486	-0.391894419	4.143731151	-0.094575252	0.924652208	0.999551235
1.1.1.310	(S)-sulfolactate dehydrogenase	0.517880281	0.817634926	4.130672112	0.197942346	0.843090173	0.999551235
2.6.1.9	histidinol-phosphate aminotransferase	276.1067268	-0.091600645	0.286272963	-0.319976584	0.748986081	0.999551235
3.1.4.53	3',5'-cyclic-AMP phosphodiesterase	4.758026176	1.118884912	1.542283814	0.725472771	0.468162044	0.999551235
3.2.1.26	beta-fructofuranosidase	664.1409514	0.058436791	0.273669556	0.213530479	0.830913218	0.999551235
1.4.3.5	pyridoxamine 5'-phosphate oxidase	35.59689684	0.689822094	0.408368189	1.689216037	0.091178037	0.999551235
4.2.1.3	#N/A	719.8495603	0.042085392	0.241647513	0.174160253	0.861739521	0.999551235
5.1.3.23	UDP-GlcNAc3NAcA epimerase	1.280800804	2.50084211	2.070754095	1.207696325	0.227164092	0.999551235
4.2.3.4	3-dehydroquinate synthase	250.9900161	-0.117700026	0.243302193	-0.483760644	0.628555749	0.999551235
2.4.2.30	poly [ADP-ribose] polymerase 16	7.933816272	1.762062571	1.031519563	1.708220215	0.087595489	0.999551235
6.3.2.32	gamma-F420-2:alpha-L-glutamate ligase	2.771140777	-1.730002741	2.443126164	-0.708110275	0.478876779	0.999551235
1.3.98.3	oxygen-independent coproporphyrinogen III oxidase	588.784638	0.01178105	0.249303609	0.047255834	0.962309328	0.999551235
2.1.1.207	tRNA (cytidine/uridine-2'-O-)-methyltransferase	189.2348351	-0.280117262	0.383988263	-0.729494335	0.465699332	0.999551235
1.1.1.9	D-xylulose reductase	0.480652276	1.529795243	4.039402138	0.378718234	0.704897112	0.999551235
3.5.3.9	allantoate deiminase	16.8615293	-0.336444343	0.434509047	-0.774309179	0.438747977	0.999551235
1.4.1.13	glutamate synthase (NADPH) large chain	1092.906852	-0.20990984	0.126932996	-1.653705869	0.09818729	0.999551235
4.2.1.76	UDP-glucose 4,6-dehydratase	0.160692243	-0.391890765	4.143731151	-0.09457437	0.924652908	0.999551235
1.11.1.9	glutathione peroxidase	52.20331418	-0.135665912	0.451919766	-0.300199111	0.764025284	0.999551235
4.1.3.-	#N/A	212.8288924	-0.157907903	0.289579372	-0.545300938	0.585546559	0.999551235
3.4.23.36	signal peptidase II	258.7444701	-0.201707001	0.267519813	-0.753989016	0.450855817	0.999551235
1.14.13.89	#N/A	0.349229033	-0.549523995	4.137841476	-0.132804506	0.89434799	0.999551235
2.4.1.211	1,3-beta-galactosyl-N-acetylhexosamine phosphorylase	341.5823143	-0.953712257	0.459752588	-2.074403239	0.038041877	0.999551235
5.5.1.4	myo-inositol-1-phosphate synthase	93.41411235	0.156137263	0.430686793	0.362530883	0.716955349	0.999551235
4.1.1.96	carboxynorspermidine decarboxylase	183.4516111	0.145783877	0.314182807	0.464009723	0.64264078	0.999551235
2.5.1.32	15-cis-phytoene synthase	6.327521558	-0.21371091	0.812936351	-0.262887629	0.792637192	0.999551235
3.5.1.1	L-asparaginase	324.8676599	0.487140317	0.236132229	2.062998008	0.039112817	0.999551235
5.3.1.-	#N/A	0.692871563	0.053902725	2.787595535	0.019336638	0.984572557	0.999551235
3.4.17.-	#N/A	48.75156414	0.779573517	0.502536559	1.551277222	0.120835263	0.999551235
3.4.24.37	saccharolysin	0.321384486	-0.391894419	4.143731151	-0.094575252	0.924652208	0.999551235
6.2.1.-	#N/A	91.10223053	-0.052898342	0.309467894	-0.170933214	0.86427628	0.999551235
3.5.1.42	nicotinamide-nucleotide amidase	360.2838083	-0.144429567	0.204935557	-0.704756018	0.480962084	0.999551235
6.2.1.4	#N/A	4.11168199	-1.523444467	2.193396082	-0.694559674	0.487331292	0.999551235
1.17.3.2	xanthine dehydrogenase/oxidase	0.783456853	-2.28999774	4.111653593	-0.556952984	0.577559552	0.999551235
1.17.7.1	(E)-4-hydroxy-3-methylbut-2-enyl-diphosphate synthase	384.5079502	0.125856052	0.171851425	0.732353846	0.463952622	0.999551235
4.2.1.51	cyclohexadienyl dehydratase	227.5628859	0.10343083	0.342648477	0.301856969	0.762761101	0.999551235
1.3.1.28	2,3-dihydro-2,3-dihydroxybenzoate dehydrogenase	0.228525281	-1.270315873	4.13145749	-0.307474027	0.758482589	0.999551235
2.4.1.180	UDP-N-acetyl-D-mannosaminouronate:lipid I N-acetyl-D-mannc	6.876760302	0.449589062	0.819357322	0.548709397	0.583204897	0.999551235
3.2.1.18	sialidase-1	148.4124568	-0.184004523	0.294284851	-0.625259924	0.531800478	0.999551235
6.3.5.5	carbamoyl-phosphate synthase	1476.450991	-0.092631057	0.115204186	-0.804059812	0.421362433	0.999551235
2.1.1.222	2-polyprenyl-6-hydroxyphenyl methylase / 3-demethylubiquinc	0.275721832	-1.994544412	4.108451879	-0.485473475	0.627340524	0.999551235
3.4.25.2	ATP-dependent HslUV protease, peptidase subunit HslV	19.83543425	-0.829361166	0.954102052	-0.86925834	0.384705844	0.999551235
2.7.1.20	adenosine kinase	7.663806899	0.375273252	1.001965664	0.374537038	0.708004806	0.999551235
1.12.7.2	ferredoxin hydrogenase	28.97808088	0.497123809	0.374325079	1.328053707	0.184160365	0.999551235
1.12.1.2	NAD-reducing hydrogenase large subunit	0.096307101	-1.192781373	4.131830079	-0.288681129	0.772825405	0.999551235
1.2.1.-	#N/A	0.350393969	0.894026181	4.079549233	0.219148276	0.826534546	0.999551235
3.4.14.5	dipeptidyl-peptidase 4	245.7884548	0.26725324	0.224578702	1.190020413	0.234038369	0.999551235
5.4.3.-	#N/A	0.228801007	1.045247585	4.119044074	0.253759748	0.799681172	0.999551235
3.1.3.26	4-phytase / acid phosphatase	0.687972502	1.723071051	2.855537917	0.603413823	0.546233429	0.999551235
1.14.11.47	50S ribosomal protein L16 3-hydroxylase	1.752367224	0.803845696	1.699576328	0.472968282	0.636235808	0.999551235
2.7.7.64	UDP-sugar pyrophosphorylase	1.215621488	-0.181231587	4.107638185	-0.044120631	0.964808248	0.999551235
2.1.1.77	protein-L-isoaspartate(D-aspartate) O-methyltransferase	4.997462947	1.056291827	1.254355157	0.842099481	0.399732272	0.999551235
4.1.1.36	phosphopantothenoylcysteine decarboxylase	282.4352402	-0.022625362	0.147271428	-0.153630357	0.8779012	0.999551235
1.2.1.2	#N/A	428.0325481	-0.821785364	0.750040941	-1.095654009	0.273230216	0.999551235
1.1.1.67	mannitol 2-dehydrogenase	18.00843058	1.160365781	1.298565179	0.893575309	0.371549162	0.999551235
3.2.1.185	non-reducing end beta-L-arabinofuranosidase	0.132242126	0.30363982	4.141926065	0.073308846	0.941560353	0.999551235
2.7.8.7	holo-[acyl-carrier protein] synthase	46.9670023	0.097237494	0.359731019	0.270306114	0.786924762	0.999551235
2.3.1.201	UDP-2-acetamido-3-amino-2,3-dideoxy-glucuronate N-acetyltra	31.47835353	0.119988409	0.605865953	0.198044483	0.843010261	0.999551235
5.99.1.3	#N/A	3140.163008	-0.131502267	0.194877318	-0.674795143	0.499805927	0.999551235
6.3.2.1	pantoate--beta-alanine ligase	113.077425	-0.182691394	0.298893107	-0.611226523	0.541049627	0.999551235
4.3.1.12	ornithine cyclodeaminase	44.99803754	0.635619102	0.511392085	1.24291932	0.213897566	0.999551235
2.1.1.14	5-methyltetrahydropteroyltriglutamate--homocysteine methylt	71.03558529	0.151571429	0.372210703	0.407219426	0.683846835	0.999551235
2.1.3.3	ornithine carbamoyltransferase	414.3449883	-0.339288458	0.219266958	-1.547376132	0.121772573	0.999551235
1.1.1.53	3alpha(or 20beta)-hydroxysteroid dehydrogenase	0.015832449	0.284417857	4.139099958	0.068714904	0.945216554	0.999551235
2.1.1.246	[methyl-Co(III) methanol-specific corrinoid protein]:coenzyme N	2.314203398	-0.263041891	1.996597135	-0.131745101	0.895185911	0.999551235
2.6.1.81	succinylornithine aminotransferase	0.107128162	-0.391888715	4.143731151	-0.094573876	0.924653301	0.999551235
5.4.99.5	chorismate mutase	340.3622117	0.189986883	0.348858876	0.544595238	0.586031931	0.999551235
2.7.7.12	UDPglucose--hexose-1-phosphate uridylyltransferase	361.433388	-0.400832857	0.194102146	-2.065061446	0.038917187	0.999551235
5.2.1.13	prolycopene isomerase	5.090923286	0.566882334	2.046939378	0.276941437	0.781825074	0.999551235
6.3.4.15	biotin---protein ligase	385.5616898	-0.145363641	0.204364353	-0.711296458	0.476900546	0.999551235
3.1.3.-	#N/A	259.2790445	0.016705863	0.256400928	0.065155235	0.948050403	0.999551235
2.5.1.72	quinolinate synthase	172.5065621	0.10200868	0.224614322	0.454150382	0.649720593	0.999551235
1.8.4.8	phosphoadenosine phosphosulfate reductase	26.55279816	-0.88856255	0.684962026	-1.297243519	0.194547411	0.999551235
2.3.1.20	diacylglycerol O-acyltransferase / wax synthase	0.015832449	0.284417857	4.139099958	0.068714904	0.945216554	0.999551235
1.5.1.28	opine dehydrogenase	16.88640933	0.338111918	0.751583322	0.44986618	0.652806935	0.999551235
2.7.4.16	thiamine-monophosphate kinase	124.9748864	0.355574644	0.284533979	1.24967375	0.21141875	0.999551235
1.1.1.125	#N/A	27.46965	-0.033290511	0.516868903	-0.064408037	0.948645331	0.999551235
2.6.1.85	para-aminobenzoate synthetase component II	58.14321162	0.294976956	0.446172038	0.661128288	0.508530045	0.999551235
4.1.99.1	tryptophanase	214.4032856	-1.024418686	0.448033518	-2.286477784	0.022226322	0.999551235
4.4.1.16	selenocysteine lyase	211.520852	0.22642402	0.261094357	0.867211466	0.385826155	0.999551235
2.7.1.45	2-dehydro-3-deoxygluconokinase	656.6700001	0.193950979	0.314345499	0.616999382	0.537235133	0.999551235
2.7.1.92	5-dehydro-2-deoxygluconokinase	8.500724103	0.080122992	0.814976638	0.098313237	0.921683567	0.999551235
5.3.3.-	#N/A	0.107481033	0.414318669	4.133619734	0.100231443	0.920160584	0.999551235
3.4.22.47	gingipain K	144.844054	-1.20601659	2.785033963	-0.433034787	0.664989498	0.999551235
3.1.27.6	ribonuclease I (enterobacter ribonuclease)	0.096307101	-1.192781373	4.131830079	-0.288681129	0.772825405	0.999551235
4.3.3.7	4-hydroxy-tetrahydrodipicolinate synthase	498.9117405	-0.202101865	0.175055753	-1.154499992	0.248295242	0.999551235

1.1.1.367	UDP-2-acetamido-2,6-beta-L-arabino-hexul-4-ose reductase	104.7367404	0.320666819	0.458430864	0.699487849	0.484247204	0.999551235
2.4.1.161	oligosaccharide 4-alpha-D-glucosyltransferase	0.654538727	-1.398947615	4.117607347	-0.339747697	0.734046539	0.999551235
1.14.12.19	3-phenylpropionate/trans-cinnamate dioxygenase subunit alph	0.192614202	-1.376625876	4.11348949	-0.334661333	0.737880573	0.999551235
2.7.7.42	[glutamine synthetase] adenylyltransferase / [glutamine synthe	8.353306205	0.466818171	0.973861345	0.479347674	0.631691312	0.999551235
5.1.3.11	cellobiose epimerase	175.8677582	-0.540720677	0.576151077	-0.938505018	0.347984939	0.999551235
1.6.3.3	NADH oxidase (H2O2-forming)	4.970299014	-1.025658309	1.573476803	-0.651842028	0.514503086	0.999551235
2.6.1.19	4-aminobutyrate aminotransferase	25.77244632	0.308513103	0.572919658	0.538492787	0.590236883	0.999551235
2.1.2.11	3-methyl-2-oxobutanoate hydroxymethyltransferase	154.993389	0.199834814	0.227102086	0.879933854	0.378895143	0.999551235
5.4.3.5	D-ornithine 4,5-aminomutase subunit beta	40.98653382	0.509343066	0.846483758	0.601716289	0.547363005	0.999551235
2.1.1.71	phosphatidyl-N-methylethanolamine N-methyltransferase	0.04941368	-0.391882467	4.143731151	-0.094572368	0.924654499	0.999551235
2.6.1.21	D-alanine transaminase	35.0129599	0.107413595	0.388692847	0.276345696	0.782282562	0.999551235
2.7.8.6	undecaprenyl-phosphate galactose phosphotransferase	3.522097742	0.561106022	1.812734851	0.309535629	0.756914114	0.999551235
5.4.99.22	23S rRNA pseudouridine2605 synthase	323.9159727	-0.059986659	0.270418374	-0.221829081	0.82444694	0.999551235
1.1.1.130	3-dehydro-L-gulonate 2-dehydrogenase	15.95022918	-0.121060655	0.556972684	-0.217354744	0.827931897	0.999551235
2.7.4.26	isopentenyl phosphate kinase	4.547246367	-1.014106247	2.282687631	-0.444259755	0.656854795	0.999551235
1.3.5.3	menaquinone-dependent protoporphyrinogen oxidase	8.639531851	0.115908754	0.771469987	0.150244022	0.880572095	0.999551235
1.1.1.90	aryl-alcohol dehydrogenase	0.138139806	0.1631836	4.118618639	0.039620954	0.968395322	0.999551235
3.1.3.78	phosphatidylinositol-4,5-bisphosphate 4-phosphatase	0.330216866	-1.412714766	4.123227835	-0.342623503	0.731881718	0.999551235
3.4.11.21	aspartyl aminopeptidase	198.5834177	-0.052971928	0.238480791	-0.22212241	0.824218593	0.999551235
3.4.21.-	#N/A	471.7887788	0.150451745	0.200314743	0.751076745	0.45260647	0.999551235
1.5.1.21	delta1-piperideine-2-carboxylate reductase	0.021925243	0.329456595	4.143731151	0.079507232	0.936629179	0.999551235
3.2.1.15	polygalacturonase	0.107128162	-0.391888715	4.143731151	-0.094573876	0.924653301	0.999551235
2.4.1.144	beta-1,4-mannosyl-glycoprotein beta-1,4-N-acetylglucosaminyl	2.052878989	-0.516608689	1.928979434	-0.267814514	0.788842098	0.999551235
1.1.1.83	tartrate dehydrogenase/decarboxylase / D-malate dehydrogen	0.660399619	0.135768846	4.076623528	0.033304239	0.973431973	0.999551235
2.1.1.173	23S rRNA (guanine2445-N2)-methyltransferase / 23S rRNA (gua	1.561324307	-0.252801989	1.699975064	-0.148709234	0.881783075	0.999551235
1.1.1.287	D-arabinitol dehydrogenase (NADP+)	0.353113201	2.074163384	4.070747973	0.509528813	0.610381607	0.999551235
6.1.1.-	#N/A	4.095223106	1.334180273	1.50324487	0.887533561	0.374791706	0.999551235
6.4.1.3	acetyl-CoA/propionyl-CoA carboxylase	161.1962201	0.18183183	0.308156977	0.590062351	0.555148849	0.999551235
1.7.2.2	nitrite reductase (cytochrome c-552)	50.95058939	0.108003038	0.503799673	0.214376951	0.830253114	0.999551235
5.4.4.2	salicylate biosynthesis isochorismate synthase	53.1414597	0.73989552	0.352211913	2.10071123	0.035666323	0.999551235
3.1.4.14	acyl carrier protein phosphodiesterase	0.144460652	-1.306718627	4.119786615	-0.317181143	0.751106158	0.999551235
2.3.1.8	phosphate acetyltransferase	784.4992269	-0.204858903	0.507110948	-0.403972549	0.686232905	0.999551235
3.1.13.3	#N/A	459.8598424	-0.218830865	0.313836526	-0.697276597	0.485629711	0.999551235
2.1.1.182	16S rRNA (adenine1518-N6/adenine1519-N6)-dimethyltransfer	406.2000725	-0.384516739	0.437276578	-0.879344466	0.379214533	0.999551235
4.2.2.2	pectate lyase	6.801543122	-0.800657239	1.263859143	-0.633501956	0.526405904	0.999551235
2.7.3.9	#N/A	939.61253	-0.322054512	0.306526815	-1.050656896	0.293416199	0.999551235
4.3.1.3	histidine ammonia-lyase	192.3498098	0.183113143	0.273264185	0.670095653	0.502796816	0.999551235
3.1.13.1	exoribonuclease II	8.613747685	0.015556234	1.009736233	0.015406236	0.987708089	0.999551235
2.1.1.297	release factor glutamine methyltransferase	376.6235411	-0.056307553	0.217634694	-0.258725079	0.795847368	0.999551235
3.6.3.10	H+/K+-exchanging ATPase	0.264436359	-0.441114387	4.14168021	-0.106506144	0.915180781	0.999551235
3.5.1.87	beta-ureidopropionase / N-carbamoyl-L-amino-acid hydrolase	61.22171734	0.131672972	0.460666783	0.28583127	0.775007357	0.999551235
3.4.21.62	subtilisin	0.654451996	0.019246407	4.042621873	0.004760872	0.996201388	0.999551235
2.7.1.166	3-deoxy-D-manno-octulosonic acid kinase	0.202265241	0.53794595	4.12474408	0.130419231	0.896234753	0.999551235
3.1.1.29	peptidyl-tRNA hydrolase, PTH1 family	247.2072187	-0.18115958	0.323950724	-0.559219555	0.576011889	0.999551235
6.4.1.4	#N/A	0.160692243	-0.391890765	4.143731151	-0.09457437	0.924652908	0.999551235
2.3.1.42	glyceronephosphate O-acyltransferase	0.214256324	-0.391892261	4.143731151	-0.094574732	0.924652621	0.999551235
1.18.1.2	ferredoxin/flavodoxin---NADP+ reductase	452.6425692	-0.616202182	0.272876169	-2.258175142	0.023934742	0.999551235
1.8.4.12	peptide-methionine (R)-S-oxide reductase	97.44710833	-0.06793482	0.292934907	-0.231910974	0.816607161	0.999551235
3.5.2.17	5-hydroxyisourate hydrolase	0.098827359	-0.391884352	4.143731151	-0.094572823	0.924654137	0.999551235
5.1.3.7	UDP-N-acetylglucosamine 4-epimerase	49.44551409	1.057767662	0.463361115	2.282814908	0.022441273	0.999551235
3.4.24.28	bacillolysin	0.63373079	-0.070992947	4.069013139	-0.017447215	0.986079843	0.999551235
1.1.2.4	D-lactate dehydrogenase (cytochrome)	3.268088468	-0.513020926	1.329664693	-0.385827291	0.699624582	0.999551235
2.7.11.11	protein kinase A	0.642768973	-0.391898218	4.143731151	-0.094576169	0.924651479	0.999551235
5.1.3.26	GlcNAc-P-P-Und epimerase	22.84782111	0.71489581	0.563134293	1.269494362	0.2042648	0.999551235
1.3.1.98	UDP-N-acetylmuramate dehydrogenase	353.7006	-0.059683432	0.169483019	-0.352149923	0.72472583	0.999551235
5.1.3.30	D-psicose/D-tagatose/L-ribulose 3-epimerase	6.991926601	-1.608587475	1.024571419	-1.5700101	0.116412762	0.999551235
4.2.1.93	ATP-dependent NAD(P)H-hydrate dehydratase	0.015832449	0.284417857	4.139099958	0.068714904	0.945216554	0.999551235
4.2.3.153	(5-formylfuran-3-yl)methyl phosphate synthase	5.230391143	-1.226745127	2.380548348	-0.5153204	0.606329146	0.999551235
2.4.1.5	dextransucrase	2.832586369	-2.786863381	2.43458694	-1.144696595	0.252334862	0.999551235
4.1.1.7	benzoylformate decarboxylase	0.102122587	-0.447703369	4.141447253	-0.10810312	0.913913893	0.999551235
2.3.1.75	diacylglycerol O-acyltransferase / wax synthase	0.015832449	0.284417857	4.139099958	0.068714904	0.945216554	0.999551235
3.4.23.40	phytepsin	0.116409678	-0.424740052	4.142298814	-0.10253728	0.918330224	0.999551235
3.2.1.123	endoglycosylceramidase	0.69313487	0.828013313	4.080760613	0.202906613	0.839208026	0.999551235
2.7.1.25	adenylylsulfate kinase	63.6182501	0.127066709	0.275693719	0.460898093	0.64487172	0.999551235
2.9.1.-	#N/A	0.431659391	-2.450542307	4.107640942	-0.59658143	0.550786873	0.999551235
4.2.1.28	propanediol dehydratase large subunit	328.6225478	-0.816403102	0.93112189	-0.87679509	0.380597946	0.999551235
5.3.3.18	2-(1,2-epoxy-1,2-dihydrophenyl)acetyl-CoA isomerase	18.26828696	-1.581724604	1.010066887	-1.565960258	0.117357921	0.999551235
3.1.2.1	acetyl-CoA hydrolase	1.659607312	-1.078226306	4.099061952	-0.263042208	0.792518047	0.999551235
1.13.11.3	#N/A	0.337017706	-2.171682362	4.124854684	-0.526486999	0.598549883	0.999551235
2.1.1.213	tRNA (guanine10-N2)-dimethyltransferase	2.980175938	-1.333422197	2.653216699	-0.502568146	0.61526793	0.999551235
1.1.1.178	3-hydroxyacyl-CoA dehydrogenase / 3-hydroxy-2-methylbutyry	0.642768973	-0.391898218	4.143731151	-0.094576169	0.924651479	0.999551235
2.7.1.56	1-phosphofructokinase	215.1212971	-0.126969302	0.208173937	-0.6099193	0.541915267	0.999551235
1.1.1.159	7-alpha-hydroxysteroid dehydrogenase	12.0655719	-1.118215425	0.744951769	-1.501057481	0.133340695	0.999551235
2.7.1.211	PTS system, sucrose-specific IIA component	72.37468344	-0.139832008	0.407046584	-0.343528269	0.731201078	0.999551235
3.2.1.4	endoglucanase	878.084613	-0.081011022	0.466847557	-0.173527783	0.862236591	0.999551235
2.7.7.33	glucose-1-phosphate cytidylyltransferase	68.99084955	-0.26386425	0.393081567	-0.671270985	0.502047915	0.999551235
2.4.1.41	polypeptide N-acetylgalactosaminyltransferase	0.290251399	-0.64340408	4.135440457	-0.155582963	0.876361755	0.999551235
4.2.1.130	D-lactate dehydratase / protein deglycase	0.096307101	-1.192781373	4.131830079	-0.288681129	0.772825405	0.999551235
2.8.1.11	adenylyltransferase and sulfurtransferase	19.1465002	0.639845129	0.521643423	1.226594835	0.219974911	0.999551235
2.2.1.7	1-deoxy-D-xylulose-5-phosphate synthase	707.1842448	-0.017599668	0.139820915	-0.125872928	0.899832513	0.999551235
3.4.13.22	zinc D-Ala-D-Ala dipeptidase	58.536724	-0.07835788	0.394975506	-0.198386682	0.842742537	0.999551235
4.4.1.8	#N/A	368.3176599	0.240969528	0.233890029	1.030268497	0.302883983	0.999551235
1.3.1.101	digeranylgeranylglycerophospholipid reductase	9.718970903	-1.852973622	1.468692422	-1.261648521	0.207075288	0.999551235
3.1.3.82	D-glycero-D-manno-heptose 1,7-bisphosphate phosphatase	19.00358607	0.492902309	0.460276656	1.070882703	0.284222174	0.999551235
2.7.1.144	tagatose 6-phosphate kinase	13.17025696	-1.162409185	0.849494036	-1.368354734	0.171201066	0.999551235
1.5.1.24	N5-(carboxyethyl)ornithine synthase	0.256368638	-0.391891285	4.143731151	-0.094574496	0.924652808	0.999551235
2.7.10.2	protein-tyrosine kinase	0.471880068	-2.002023975	4.128372761	-0.484942638	0.627717036	0.999551235

2.1.1.289	cobalt-precorrin-7 (C5)-methyltransferase	2.079831392	0.758378861	1.943577866	0.390197313	0.696390649	0.999551235
1.10.3.9	photosystem II P680 reaction center D1 protein	1.966809294	-4.359927587	4.0545016	-1.075330094	0.282226967	0.999551235
2.5.1.3	thiamine-phosphate pyrophosphorylase	291.6609735	0.321821508	0.153597139	2.09523113	0.036150451	0.999551235
1.16.1.3	aquacobalamin reductase / NAD(P)H-flavin reductase	1.180331694	-2.57768995	4.096909634	-0.629179108	0.529231805	0.999551235
2.1.1.195	cobalt-precorrin-5B (C1)-methyltransferase	169.9058599	-0.000266093	0.335861097	-0.000792272	0.999367858	0.999551235
5.3.1.9	glucose-6-phosphate isomerase	902.8368241	-0.239135257	0.280453993	-0.852671966	0.393841241	0.999551235
2.7.2.3	phosphoglycerate kinase	1431.260499	-0.549813968	0.499526017	-1.100671336	0.271039726	0.999551235
5.3.3.2	isopentenyl-diphosphate Delta-isomerase	13.07900694	-0.13307666	0.792084349	-0.168008193	0.866576833	0.999551235
3.4.18.1	cathepsin X	4.756805142	-0.643673141	4.095191624	-0.157177783	0.875104737	0.999551235
3.5.1.5	urease	257.9239362	-0.675258827	0.714606698	-0.944937723	0.344690671	0.999551235
4.2.1.99	aconitate hydratase 2 / 2-methylisocitrate dehydratase	3.37656264	-1.92632558	3.072974357	-0.626860285	0.530750823	0.999551235
2.3.1.241	Kdo2-lipid IVA lauroyltransferase	95.41321668	0.39336276	0.285274735	1.378890984	0.167928368	0.999551235
1.2.1.19	aminobutyraldehyde dehydrogenase	0.383706937	-0.438749586	4.102559857	-0.106945322	0.914832357	0.999551235
6.3.5.9	cobyrinic acid a,c-diamide synthase	160.4431359	0.174681203	0.333845753	0.523239255	0.600807767	0.999551235
1.1.1.169	2-dehydropantoate 2-reductase	326.602218	0.560388122	0.248841217	2.251990764	0.024322856	0.999551235
3.5.2.2	dihydropyrimidinase	90.86795266	-0.747313718	0.327487715	-2.281959548	0.022491729	0.999551235
1.18.6.1	nitrogenase delta subunit	63.88890217	0.016532693	0.365053424	0.045288421	0.963877417	0.999551235
2.7.7.80	adenylyltransferase and sulfurtransferase	19.1465002	0.639845129	0.521643423	1.226594835	0.219974911	0.999551235
5.3.1.25	L-fucose/D-arabinose isomerase	329.4791065	-1.015352085	0.383237061	-2.649409955	0.008063246	0.999551235
3.6.3.14	#N/A	3022.576656	-0.251092897	0.26260288	-0.956169622	0.338986545	0.999551235
2.1.1.181	23S rRNA (adenine1618-N6)-methyltransferase	4.035600293	-0.735329235	0.969220547	-0.758681022	0.448043392	0.999551235
2.3.1.129	UDP-N-acetylglucosamine acyltransferase	179.9466588	0.256187952	0.331388257	0.773074926	0.439478039	0.999551235
1.16.1.10	ferric-chelate reductase [NAD(P)H]	0.174502634	-1.085692314	4.141315025	-0.262161248	0.793197129	0.999551235
4.2.1.104	cyanate lyase	0.715825094	1.182424926	3.686712115	0.320726134	0.74841794	0.999551235
3.5.99.7	1-aminocyclopropane-1-carboxylate deaminase	0.33950778	1.257196701	4.072503393	0.308703659	0.75754696	0.999551235
1.3.99.4	3-oxosteroid 1-dehydrogenase	1.106189635	0.085964167	3.239617748	0.026535281	0.978830393	0.999551235
1.2.1.22	lactaldehyde dehydrogenase / glycolaldehyde dehydrogenase	36.22550726	-0.116589156	0.401431952	-0.290433174	0.771484868	0.999551235
2.4.1.318	demethyllactenocin mycarosyltransferase	0.022430994	-0.752542723	4.143731151	-0.18160993	0.855888856	0.999551235
3.6.1.6	ectonucleoside triphosphate diphosphohydrolase 5/6	0.749897135	-0.389946523	4.143544738	-0.094109403	0.925022251	0.999551235
3.1.27.1	ribonuclease T2	0.308633526	1.551102118	4.108273122	0.377555745	0.705760646	0.999551235
1.1.1.40	malate dehydrogenase (oxaloacetate-decarboxylating)(NADP+)	110.9790257	0.372544903	0.350631689	1.062496388	0.288010397	0.999551235
3.2.1.51	alpha-L-fucosidase	1023.186115	-0.047019457	0.174132169	-0.270021658	0.787143592	0.999551235
5.4.99.9	UDP-galactopyranose mutase	320.18863	0.01218072	0.253452989	0.048059088	0.961669151	0.999551235
4.1.99.17	phosphomethylpyrimidine synthase	279.9666111	0.105401122	0.242477426	0.434684266	0.663791621	0.999551235
5.1.3.9	N-acylglucosamine-6-phosphate 2-epimerase	169.6607425	-0.479576427	0.457290639	-1.048734405	0.294300375	0.999551235
1.3.1.10	enoyl-acyl-carrier protein] reductase I	82.40735219	0.205143472	0.28349655	0.723618937	0.469299712	0.999551235
2.7.6.5	GTP pyrophosphokinase	1266.617419	-0.181819762	0.14599962	-1.245344078	0.2130053	0.999551235
1.2.1.72	D-erythrose 4-phosphate dehydrogenase	1.431547706	-1.718171563	4.095594313	-0.41951703	0.674838311	0.999551235
5.1.3.25	dTDP-L-rhamnose 4-epimerase	1.791576241	0.631725067	1.783479861	0.354209252	0.723182071	0.999551235
4.2.1.32	#N/A	69.34122182	-0.187311806	0.545797001	-0.343189512	0.731455895	0.999551235
4.2.1.149	crotonobetainyl-CoA hydratase	3.918396303	-3.983091141	2.155242328	-1.848094337	0.064588697	0.999551235
2.3.1.88	#N/A	0.364127506	-0.881956101	4.117383434	-0.214203053	0.830388716	0.999551235
5.5.1.1	muconate cycloisomerase	0.138446491	-1.141724353	4.137743456	-0.275929227	0.782602425	0.999551235
3.6.1.55	8-oxo-dGTP diphosphatase	335.946494	0.339427425	0.248168576	1.367729269	0.171396835	0.999551235
2.7.1.177	L-threonine kinase	0.594267165	1.319070066	4.052070877	0.32552986	0.744780087	0.999551235
3.7.1.17	4,5:9,10-diseco-3-hydroxy-5,9,17-trioxoandrosta-1(10),2-diene-	0.231939278	-1.393542934	4.123543997	-0.337947876	0.735402468	0.999551235
5.4.2.9	phosphoenolpyruvate phosphomutase	16.50042611	-0.714704035	0.764760487	-0.934546237	0.350022197	0.999551235
6.3.1.5	NAD+ synthase	39.38747508	-0.241799924	0.633119749	-0.381918152	0.702522076	0.999551235
1.18.1.-	#N/A	0.305626466	-2.392515399	4.123314889	-0.580240768	0.561752265	0.999551235
4.2.1.113	O-succinylbenzoate synthase	4.188888329	-0.51899557	1.351453915	-0.384027575	0.700958009	0.999551235
2.3.1.-	#N/A	274.7832898	0.191527697	0.262882967	0.728566403	0.466266936	0.999551235
1.16.3.2	ferritin heavy chain	526.0547936	0.03693023	0.45417321	0.081313097	0.935192958	0.999551235
1.11.1.21	catalase-peroxidase	8.342629727	0.097721868	2.907947184	0.033605104	0.973192052	0.999551235
5.1.3.-	#N/A	3.808216094	-0.983020877	1.287762902	-0.763355487	0.445251419	0.999551235
1.2.7.5	aldehyde:ferredoxin oxidoreductase	41.45335069	-0.768324081	0.678758278	-1.131955376	0.257653196	0.999551235
4.2.1.46	dTDP-glucose 4,6-dehydratase	442.2462817	-0.213419763	0.170871694	-1.249005951	0.211662897	0.999551235
3.5.5.1	nitrilase	12.224291	-0.107327971	0.741026105	-0.144836963	0.884839598	0.999551235
1.3.5.6	zeta-carotene desaturase	0.089381093	-0.391886445	4.143731151	-0.094573328	0.924653736	0.999551235
1.14.14.9	4-hydroxyphenylacetate 3-monooxygenase	3.60579661	-3.998849567	1.866692424	-2.14221128	0.032176484	0.999551235
6.3.2.35	D-alanine---D-serine ligase	27.59615683	-1.101023516	0.525857428	-2.093768113	0.036280642	0.999551235
2.7.7.1	nicotinamide-nucleotide adenylyltransferase	6.6903392	0.532932464	1.264385153	0.421495351	0.673393405	0.999551235
2.3.1.157	bifunctional UDP-N-acetylglucosamine pyrophosphorylase / Glu	389.7431354	-0.540106667	0.378323111	-1.427633288	0.153397437	0.999551235
3.1.3.6	2',3'-cyclic-nucleotide 2'-phosphodiesterase / 3'-nucleotidase	234.785924	0.519197231	0.315115597	1.647640535	0.099426457	0.999551235
3.5.2.5	allantoinase	3.997005751	-1.316041724	1.412667356	-0.931600577	0.351542985	0.999551235
4.3.1.29	D-glucosaminate-6-phosphate ammonia-lyase	0.089381093	-0.391886445	4.143731151	-0.094573328	0.924653736	0.999551235
3.1.3.27	phosphatidylglycerophosphatase GEP4	30.96879914	0.656396602	0.50153979	1.308762764	0.190614724	0.999551235
5.4.2.11	2,3-bisphosphoglycerate-dependent phosphoglycerate mutase	221.9404766	0.758652708	0.345769128	2.194101924	0.028228083	0.999551235
2.7.4.6	nucleoside-diphosphate kinase	76.50444022	-0.084237574	0.575420852	-0.146392981	0.883611168	0.999551235
3.1.26.3	#N/A	253.7334934	-0.003143046	0.277309741	-0.011334063	0.99095692	0.999551235
2.1.1.132	precorrin-6Y C5,15-methyltransferase (decarboxylating)	187.4625788	0.076080703	0.320604771	0.237303715	0.81242118	0.999551235
2.7.7.27	glucose-1-phosphate adenylyltransferase	2821.889123	-0.69259116	0.447288981	-1.5484199	0.121521233	0.999551235
1.12.1.3	NADP-reducing hydrogenase subunit HndB	339.9647259	-0.219425022	0.379585892	-0.578064219	0.563220763	0.999551235
3.4.24.13	IgA-specific metalloendopeptidase	0.02470684	-0.391881853	4.143731151	-0.09457222	0.924654616	0.999551235
1.11.1.6	#N/A	118.6950932	-0.337025873	0.417748721	-0.806766979	0.419800747	0.999551235
3.6.5.5	dynamin GTPase	0.770998033	-1.062884639	4.103835622	-0.258997859	0.795636893	0.999551235
2.7.2.8	acetylglutamate kinase	349.4705199	0.067898819	0.307600013	0.220737375	0.825296928	0.999551235
5.4.99.2	methylmalonyl-CoA mutase	438.421622	0.155331367	0.291971424	0.532008801	0.5947199	0.999551235
1.1.1.284	S-(hydroxymethyl)glutathione dehydrogenase / alcohol dehydro	1.229341188	-0.803524476	3.405248701	-0.235966458	0.813458698	0.999551235
4.1.1.82	phosphonopyruvate decarboxylase	16.79511523	-0.380232046	0.52446353	-0.724992349	0.468456724	0.999551235
1.1.1.197	carbonyl reductase 1	0.067578231	-0.830999939	4.140710721	-0.20069017	0.840940847	0.999551235
6.2.1.20	long-chain-fatty-acid--[acyl-carrier-protein] ligase	12.26279611	0.271702425	0.856507348	0.317221359	0.751075644	0.999551235
4.3.1.15	diaminopropionate ammonia-lyase	67.37423002	-0.766223469	0.621759042	-1.232347931	0.217819142	0.999551235
3.4.13.19	membrane dipeptidase	152.4527255	-0.050615767	0.272613227	-0.185668787	0.852704507	0.999551235
4.6.1.15	triose/dihydroxyacetone kinase / FAD-AMP lyase (cyclizing)	5.825853036	1.731675002	4.034528823	0.42921369	0.667767721	0.999551235
6.2.1.3	long-chain acyl-CoA synthetase	782.0385652	0.13398347	0.154490003	0.867263041	0.385797902	0.999551235
2.7.1.196	PTS system, cellobiose-specific IIA component	67.85843679	-0.279328013	0.576258579	-0.484726861	0.62787011	0.999551235
1.1.1.38	malate dehydrogenase (oxaloacetate-decarboxylating)	427.7254982	0.758718444	0.405804186	1.869666381	0.061530161	0.999551235

1.14.18.6	4-hydroxysphinganine ceramide fatty acyl 2-hydroxylase	0.100382114	-1.1233092	4.143261082	-0.271117165	0.78630092	0.999551235
6.3.4.21	nicotinate phosphoribosyltransferase	581.9678307	-0.699087247	0.442163195	-1.581061599	0.113863954	0.999551235
3.6.1.25	triphosphatase	0.063329795	1.052988038	4.122789602	0.255406688	0.798409006	0.999551235
1.5.98.1	methylenetetrahydromethanopterin dehydrogenase	30.1788584	-1.528231086	1.252308247	-1.220331408	0.222339268	0.999551235
4.2.1.17	enoyl-CoA hydratase	1168.14638	0.000352704	0.627093151	0.000562443	0.999551235	0.999551235
1.1.1.252	tetrahydroxynaphthalene reductase	0.063329795	1.052988038	4.122789602	0.255406688	0.798409006	0.999551235
4.4.1.21	S-ribosylhomocysteine lyase	148.3246946	0.06425358	0.285820239	0.224804164	0.822131626	0.999551235
2.1.1.72	site-specific DNA-methyltransferase (adenine-specific)	1204.253236	0.325302475	0.272275798	1.194753542	0.232183336	0.999551235
5.4.1.4	D-galactarolactone isomerase	0.015832449	0.284417857	4.139099958	0.068714904	0.945216554	0.999551235
3.1.13.-	#N/A	1.076630519	-1.99658889	4.129606498	-0.483481632	0.628753799	0.999551235
1.3.99.28	phytoene desaturase	1.632348624	-0.923071895	4.11939824	-0.224079305	0.822695595	0.999551235
5.3.1.3	L-fucose/D-arabinose isomerase	329.4791065	-1.015352085	0.383237061	-2.649409955	0.008063246	0.999551235
4.2.1.11	enolase	996.8971278	-0.20204196	0.407720609	-0.495540219	0.620218839	0.999551235
5.1.3.13	dTDP-4-dehydrorhamnose 3,5-epimerase	207.8342078	0.060825772	0.201831062	0.301369727	0.763132576	0.999551235
2.4.2.7	adenine phosphoribosyltransferase	209.6202566	0.21715332	0.201216219	1.079203857	0.280496861	0.999551235
3.4.11.5	proline iminopeptidase	13.02301904	-1.05797902	0.578897802	-1.827574775	0.067613394	0.999551235
1.1.1.23	histidinol dehydrogenase	291.6884958	-0.096645727	0.214730638	-0.450078889	0.652653558	0.999551235
1.5.1.34	dihydropteridine reductase	6.296940433	-0.236186726	0.826615013	-0.285727603	0.775086762	0.999551235
1.8.4.11	peptide-methionine (S)-S-oxide reductase	112.1664182	-0.021390712	0.283338563	-0.075495238	0.939820687	0.999551235
1.1.99.3	#N/A	1.815348364	3.226187466	2.4770524	1.302430044	0.192769418	0.999551235
5.3.2.5	2,3-diketo-5-methylthiopentyl-1-phosphate enolase	8.254332185	0.435759666	1.011184476	0.43093983	0.666512121	0.999551235
3.2.1.196	glycogen debranching enzyme	167.3467347	-0.587933881	0.301415474	-1.950576303	0.051107468	0.999551235
3.1.21.5	type III restriction enzyme	189.1393977	0.278957939	0.320050309	0.871606527	0.383423069	0.999551235
3.11.1.2	phosphonoacetate hydrolase	0.50996675	-0.177766822	4.098903317	-0.043369362	0.9654071	0.999551235
1.1.1.184	carbonyl reductase 1	0.067578321	-0.830999939	4.140710721	-0.20069017	0.840940847	0.999551235
3.5.4.27	methenyltetrahydromethanopterin cyclohydrolase	3.406741929	-1.608489219	1.933401305	-0.831947933	0.405438334	0.999551235
2.4.1.321	cellobionic acid phosphorylase	0.076526885	0.006493138	4.120467203	0.001575826	0.998742674	0.999551235
1.1.1.93	tartrate dehydrogenase	0.660399619	0.135768846	4.076623528	0.033304239	0.973431973	0.999551235
2.7.7.75	molybdopterin adenylyltransferase	3.318616763	0.82291911	1.596730252	0.515377666	0.606289136	0.999551235
2.7.7.87	L-threonylcarbamoyladenylate synthase	333.119198	-0.076092408	0.15385722	-0.494565081	0.620907156	0.999551235
3.6.3.21	#N/A	586.7150326	0.015745697	0.385795896	0.040813543	0.967444543	0.999551235
2.7.12.1	#N/A	0.589204892	-0.391897735	4.143731151	-0.094576052	0.924651572	0.999551235
2.4.1.109	dolichyl-phosphate-mannose-protein mannosyltransferase	0.048153551	-0.752542723	4.143731151	-0.18160993	0.855888856	0.999551235
6.1.1.19	arginyl-tRNA synthetase	902.3901318	0.093597943	0.346812526	0.269880514	0.787252179	0.999551235
1.3.1.34	2,4-dienoyl-CoA reductase (NADPH2)	11.37066263	-0.161925418	0.92618934	-0.174829715	0.861213439	0.999551235
1.3.1.54	precorrin-6A/cobalt-precorrin-6A reductase	61.18763666	0.529766801	0.331413302	1.598507958	0.109929976	0.999551235
1.2.4.2	2-oxoglutarate dehydrogenase E1 component	17.17650179	-0.755092438	1.165427218	-0.64791042	0.517042891	0.999551235
2.7.1.194	PTS system, ascorbate-specific IIA component	24.17034772	-1.347815496	0.65467024	-2.05877007	0.03951627	0.999551235
3.2.1.183	UDP-N-acetylglucosamine 2-epimerase (hydrolysing)	12.086231	-0.602637695	0.876910573	-0.687228223	0.491938922	0.999551235
2.6.1.82	putrescine aminotransferase	66.44150576	-1.086850718	0.858749476	-1.265620241	0.205649076	0.999551235
1.11.1.10	non-heme chloroperoxidase	0.399388983	-2.059158629	4.129237471	-0.498677696	0.61800646	0.999551235
2.4.2.29	queuine tRNA-ribosyltransferase	354.7608664	-0.156172597	0.219023428	-0.713040603	0.475820632	0.999551235
3.4.21.102	carboxyl-terminal processing protease	593.5158334	0.29132233	0.15101034	1.929154852	0.053711641	0.999551235
2.1.1.45	thymidylate synthase	208.9073855	0.271232111	0.30886353	0.878161662	0.379855994	0.999551235
2.7.1.6	galactokinase	521.2465307	-0.032596538	0.137331063	-0.237357357	0.812379569	0.999551235
2.6.1.66	valine--pyruvate aminotransferase	66.13941554	-0.875518285	0.466533858	-1.876644687	0.060566808	0.999551235
2.7.1.16	L-ribulokinase	36.69650726	0.817616076	0.379827265	2.15259975	0.031350148	0.999551235
6.3.2.30	cyanophycin synthetase	4.102299911	-0.34430083	3.756698354	-0.091649847	0.926976246	0.999551235
4.1.1.3	#N/A	1317.677733	-0.105052319	0.203472595	-0.516297138	0.605646895	0.999551235
3.2.1.11	dextranase	30.10552948	0.012282993	0.521228059	0.023565488	0.981199201	0.999551235
1.8.1.8	thioredoxin 2	161.0699188	0.116820556	0.251172103	0.465101637	0.641858674	0.999551235
3.1.4.-	#N/A	256.2826558	0.020457027	0.26049135	0.078532463	0.937404508	0.999551235
2.3.1.46	homoserine O-acetyltransferase/O-succinyltransferase	851.3442398	-1.140201081	0.822990311	-1.385436822	0.165918938	0.999551235
6.2.1.7	solute carrier family 27 (fatty acid transporter), member 5	4.984233103	-2.812380269	4.118134877	-0.68292573	0.494653774	0.999551235
2.4.1.280	N,N'-diacetylchitobiose phosphorylase	8.469517485	0.995758184	1.177171783	0.845890293	0.397613948	0.999551235
3.4.21.96	lactocepin	296.0567306	-1.214379983	0.942748069	-1.288127786	0.197701484	0.999551235
4.1.1.-	#N/A	0.100382114	-1.1233092	4.143261082	-0.271117165	0.78630092	0.999551235
4.1.1.49	phosphoenolpyruvate carboxykinase (ATP)	1039.813312	-0.587300275	0.386928552	-1.517851994	0.129051714	0.999551235
2.3.1.193	tRNA(Met) cytidine acetyltransferase	0.475511433	0.355042723	4.095465024	0.086691675	0.930916594	0.999551235
1.1.1.77	lactaldehyde reductase	536.8571836	-0.358130404	0.404448547	-0.885478279	0.375898729	0.999551235
1.3.1.85	crotonyl-CoA carboxylase/reductase	0.225617469	0.721087588	4.106324412	0.175604145	0.860604946	0.999551235
1.6.1.1	NAD(P) transhydrogenase	1.736111865	-1.032946877	3.146790689	-0.328254078	0.742719562	0.999551235
3.2.2.16	5'-methylthioadenosine nucleosidase	0.06610909	-0.39885862	4.143404008	-0.096263512	0.92331129	0.999551235
1.5.1.38	FMN reductase	7.661298639	-0.512891699	1.11839102	-0.458597834	0.646522992	0.999551235
1.1.1.137	ribulose-5-phosphate 2-dehydrogenase	33.26585746	-0.381263046	0.751053115	-0.507637926	0.611707288	0.999551235
2.1.1.34	tRNA (guanosine-2'-O-)-methyltransferase	13.99952319	0.659947178	0.861983298	0.765614809	0.44390553	0.999551235
3.2.2.26	futalosine hydrolase	2.601413997	-0.822004584	2.147898521	-0.382701778	0.701940896	0.999551235
3.4.23.49	omptin	0.144460652	-1.306718627	4.119786615	-0.317181143	0.751106158	0.999551235
2.7.11.23	CTD kinase subunit alpha	0.071041121	-0.721610469	4.14207572	-0.174214698	0.861696735	0.999551235
6.1.1.4	leucyl-tRNA synthetase	730.0393366	-0.143116681	0.133948993	-1.068441635	0.285321345	0.999551235
4.1.2.21	2-dehydro-3-deoxyphosphogalactonate aldolase	0.272063502	-2.212156104	4.124242076	-0.536378821	0.591696765	0.999551235
3.1.1.53	sialate O-acetylesterase	284.4241936	0.587693145	0.273961178	2.145169431	0.031939303	0.999551235
2.6.1.77	taurine-pyruvate aminotransferase	27.34860526	0.059954317	0.968643581	0.061895127	0.950646348	0.999551235
2.1.1.217	tRNA (adenine22-N1)-methyltransferase	279.7718674	-0.623672725	0.462258777	-1.349185253	0.17727747	0.999551235
2.1.1.242	16S rRNA (guanine1516-N2)-methyltransferase	13.47765524	-0.181567612	0.617074587	-0.294239328	0.768575031	0.999551235
3.5.4.3	guanine deaminase	103.5861723	-0.610102009	0.32084295	-1.901559655	0.057228747	0.999551235
1.13.11.53	1,2-dihydroxy-3-keto-5-methylthiopentene dioxygenase	0.050191057	-0.752539914	4.143731151	-0.181609252	0.855889388	0.999551235
4.1.3.1	isocitrate lyase	1.710554568	0.782764723	3.246975163	0.241075057	0.809496944	0.999551235
3.4.24.75	lysostaphin	0.258895683	0.208172573	4.095212615	0.050833154	0.959458472	0.999551235
2.7.1.-	#N/A	1291.856995	-0.237309458	0.354446666	-0.669520919	0.503163241	0.999551235
2.2.1.10	fructose-bisphosphate aldolase / 2-amino-3,7-dideoxy-D-threo-	4.406406866	-3.026645455	2.234601495	-1.354445283	0.175594359	0.999551235
1.1.1.100	3-oxoacyl-[acyl-carrier protein] reductase	2297.248751	-0.217169367	0.506827643	-0.428487613	0.66829615	0.999551235
1.17.99.6	epoxyqueuosine reductase	21.31922042	-0.273793377	0.69084177	-0.396318504	0.691870078	0.999551235
2.4.99.14	3-deoxy-D-manno-octulosonic-acid transferase	102.8745696	0.408892164	0.322930505	1.26619244	0.205444193	0.999551235
4.2.1.24	porphobilinogen synthase	65.10279835	0.066911519	0.306368397	0.218402159	0.827115783	0.999551235
2.4.1.260	alpha-1,6-mannosyltransferase	0.214256324	-0.391892261	4.143731151	-0.094574732	0.924652621	0.999551235
6.3.5.4	asparagine synthase (glutamine-hydrolysing)	377.4072799	-0.017600362	0.347570214	-0.050638292	0.959613749	0.999551235

6.1.1.22	asparaginyl-tRNA synthetase	736.9327609	1.264869321	0.485110376	2.607384597	0.009123682	0.999551235
2.7.1.33	type I pantothenate kinase	321.0567096	-0.113460358	0.240717863	-0.471341663	0.637396768	0.999551235
6.5.1.1	bifunctional non-homologous end joining protein LigD	28.88893739	-0.268472142	0.565153395	-0.475042961	0.634756352	0.999551235
2.4.1.10	levansucrase	3.347830754	-1.945231236	2.206592836	-0.881554224	0.378017919	0.999551235
3.2.2.8	ribosylpyrimidine nucleosidase	9.911351524	-0.716728201	0.870554542	-0.823300743	0.410336991	0.999551235
4.2.1.35	3-isopropylmalate/(R)-2-methylmalate dehydratase large subur	629.01279	-0.221164652	0.236381554	-0.935625683	0.349465946	0.999551235
1.1.1.343	6-phosphogluconate dehydrogenase	235.2879129	0.107780306	0.401544174	0.268414568	0.788380227	0.999551235
2.7.1.59	N-acetylglucosamine kinase	0.694599604	0.606692924	4.068955439	0.149102868	0.881472463	0.999551235
2.5.1.114	tRNA wybutosine-synthesizing protein 2	2.024429067	-2.046285467	4.125850735	-0.495966916	0.619917753	0.999551235
2.4.1.336	1,2-diacylglycerol 3-beta-glucosyltransferase	0.462092179	1.871567469	4.077613901	0.458985945	0.646244259	0.999551235
1.3.1.-	#N/A	0.739864946	-1.095636576	4.103338664	-0.267011004	0.789460689	0.999551235
3.4.21.83	oligopeptidase B	1.186270295	-0.19760276	3.771691707	-0.052391016	0.958217133	0.999551235
2.7.3.-	#N/A	31.79458853	-0.348457772	0.495188402	-0.703687264	0.481627553	0.999551235
3.5.1.49	formamidase	2.54817764	-1.947469903	2.044819293	-0.952392179	0.340898124	0.999551235
2.7.7.53	sulfate adenylyltransferase (ADP) / ATP adenylyltransferase	5.267729909	0.016716831	1.801795161	0.009277875	0.992597433	0.999551235
1.2.1.76	malonyl-CoA/succinyl-CoA reductase (NADPH)	26.9509768	0.034976399	0.493303195	0.070902438	0.943475403	0.999551235
4.3.2.3	ureidoglycolate lyase	16.18073341	0.998545593	0.67804424	1.472685017	0.140835989	0.999551235
3.1.1.5	lysophospholipase	19.07257356	-0.954164287	0.624433946	-1.52804679	0.126500921	0.999551235
1.1.1.304	meso-butanediol dehydrogenase / (S,S)-butanediol dehydroger	3.716547359	-1.934565453	1.451433783	-1.332865113	0.182576073	0.999551235
2.3.1.81	aminoglycoside 3-N-acetyltransferase	36.3516502	0.163757219	0.496028606	0.330136643	0.741296717	0.999551235
5.3.1.12	glucuronate isomerase	483.7936617	-0.155148793	0.311945478	-0.497358685	0.618936135	0.999551235
2.1.1.61	tRNA 5-methylaminomethyl-2-thiouridine biosynthesis bifuncti	0.725194853	1.805538724	4.082890336	0.442220725	0.65832949	0.999551235
3.6.1.63	alpha-D-ribose 1-methylphosphonate 5-triphosphate diphospha	2.508459474	-0.385433938	2.469090044	-0.156103638	0.875951331	0.999551235
2.7.11.2	pyruvate dehydrogenase kinase 2/3/4	0.160692243	-0.391890765	4.143731151	-0.09457437	0.924652908	0.999551235
3.1.11.-	#N/A	0.288921303	-1.472175659	4.106420589	-0.358505815	0.719964817	0.999551235
2.1.1.249	dimethylamine---corrinoid protein Co-methyltransferase	0.443021872	-0.379913889	4.142602804	-0.091708983	0.92692926	0.999551235
2.3.-.-	#N/A	4.39435923	-0.895794195	4.064509263	-0.220394182	0.825564176	0.999551235
2.5.1.18	glutathione S-transferase	2.922263646	2.628845329	1.794046781	1.465315931	0.142834742	0.999551235
1.3.1.22	steroid 5-alpha-reductase	34.49108498	0.727735264	0.487359119	1.493221807	0.135379136	0.999551235
3.1.2.-	#N/A	237.5533798	0.255639876	0.195008015	1.310919841	0.189884847	0.999551235
4.2.1.8	mannonate dehydratase	345.6022606	0.23956129	0.271521459	0.882292292	0.377618763	0.999551235
4.1.1.94	ethylmalonyl-CoA/methylmalonyl-CoA decarboxylase	0.267820405	-0.391893442	4.143731151	-0.094575017	0.924652395	0.999551235
2.4.1.141	beta-1,4-N-acetylglucosaminyltransferase	0.053564081	-0.391885504	4.143731151	-0.094573101	0.924653917	0.999551235
3.4.11.2	aminopeptidase N	57.71238724	0.181192978	0.419345075	0.432085624	0.665679183	0.999551235
3.5.3.26	(S)-ureidoglycine aminohydrolase	1.594595982	0.233388996	1.84169143	0.126725353	0.899157779	0.999551235
4.2.2.6	oligogalacturonide lyase	13.30351774	0.30400729	0.681320629	0.446202972	0.655450636	0.999551235
2.7.1.162	N-acetylhexosamine 1-kinase	5.363247739	1.042275453	1.263377904	0.824991042	0.409376676	0.999551235
1.1.1.3	homoserine dehydrogenase	423.4702407	-0.188666868	0.167651653	-1.125350478	0.260440547	0.999551235
1.13.11.-	#N/A	1.263813578	-3.420978776	4.107262089	-0.832909783	0.404895612	0.999551235
3.4.14.12	Xaa-Xaa-Pro tripeptidyl-peptidase	0.42378319	0.016527433	4.114336089	0.004017035	0.996794878	0.999551235
3.6.3.1	#N/A	0.057984819	-0.752539914	4.143731151	-0.181609252	0.855889388	0.999551235
2.6.1.-	#N/A	415.5908721	-0.102168281	0.182913491	-0.558560663	0.576461593	0.999551235
5.1.3.31	D-psicose/D-tagatose/L-ribulose 3-epimerase	6.991926601	-1.608587475	1.024571419	-1.5700101	0.116412762	0.999551235
3.2.1.141	maltooligosyltrehalose trehalohydrolase	0.197327189	1.95138594	4.090315724	0.477074649	0.633308966	0.999551235
1.2.7.8	indolepyruvate ferredoxin oxidoreductase, alpha subunit	497.2965975	0.326191938	0.275063296	1.185879555	0.235669868	0.999551235
5.3.3.14	3-hydroxyacyl-[acyl-carrier protein] dehydratase / trans-2-dece	1.695466807	-1.48585128	2.386330313	-0.622651136	0.5335138	0.999551235
3.1.3.70	mannosyl-3-phosphoglycerate phosphatase	0.043850487	0.733944445	4.133023226	0.177580528	0.859052418	0.999551235
2.4.1.292	GalNAc-alpha-(1->4)-GalNAc-alpha-(1->3)-diNAcBac-PP-undeca	0.092883485	-0.952445852	4.137771167	-0.230183307	0.817949333	0.999551235
2.7.4.8	guanylate kinase	389.5186938	-0.398709435	0.292883284	-1.361325336	0.1734109	0.999551235
3.5.1.108	UDP-3-O-[3-hydroxymyristoyl] N-acetylglucosamine deacetylas	302.2410722	-0.476075576	0.762168201	-0.624633218	0.532211813	0.999551235
1.97.1.9	Tat-targeted selenate reductase subunit YnfE	151.6737258	-0.467198737	0.316845619	-1.474531157	0.140338635	0.999551235
2.7.1.202	PTS system, fructose-specific IIA component	331.5898364	-0.222298045	0.231952794	-0.958376234	0.337873075	0.999551235
2.7.7.7	#N/A	5149.647638	-0.041837619	0.188035339	-0.222498705	0.823925681	0.999551235
2.3.1.101	formylmethanofuran--tetrahydromethanopterin N-formyltrans	8.959349101	-2.610549536	1.740500579	-1.499884325	0.133644369	0.999551235
2.5.1.16	spermidine synthase	117.6111361	0.199441408	0.382350256	0.521619652	0.601935177	0.999551235
3.6.3.31	polyamine-transporting ATPase	253.4407985	0.042809796	0.414284341	0.103334333	0.917697628	0.999551235
2.7.1.215	erythritol kinase (D-erythritol 1-phosphate-forming)	0.067292982	-1.141936265	4.136920822	-0.27603532	0.782520938	0.999551235
1.1.5.2	quinoprotein glucose dehydrogenase	1.663608877	-2.330327968	3.74237056	-0.62268766	0.533489793	0.999551235
2.5.1.76	cysteate synthase	7.56528018	1.710002486	0.888335802	1.924950543	0.054235546	0.999551235
3.2.1.78	mannan endo-1,4-beta-mannosidase	336.8419907	-0.293264121	0.394772853	-0.742868003	0.457561599	0.999551235
3.8.1.2	2-haloacid dehalogenase	83.07066021	0.220900574	0.419752328	0.526264083	0.598704735	0.999551235
2.1.1.186	23S rRNA (cytidine2498-2'-O)-methyltransferase	2.278291717	1.695494986	1.748722964	0.9695618	0.332264961	0.999551235
3.6.3.33	vitamin B12 transport system ATP-binding protein	0.098344608	-1.134322304	4.142762687	-0.273808178	0.784232038	0.999551235
1.3.1.104	enoyl-[acyl-carrier protein] reductase III	0.334667623	-1.980097381	4.128881564	-0.479572337	0.63153152	0.999551235
1.1.1.140	sorbitol-6-phosphate 2-dehydrogenase	20.25945935	-0.820270542	0.767393853	-1.068904238	0.285112822	0.999551235
3.4.21.19	glutamyl endopeptidase	25.77571237	1.185633193	0.639282986	1.854629669	0.063649128	0.999551235
5.4.99.17	squalene-hopene/tetraprenyl-beta-curcumene cyclase	6.587769184	0.496204963	1.374422927	0.361027856	0.718078623	0.999551235
4.1.1.51	3-hydroxy-2-methylpyridine-4,5-dicarboxylate 4-decarboxylase	0.02470684	-0.391881853	4.143731151	-0.09457222	0.924654616	0.999551235
2.8.1.8	lipoyl synthase	68.84685767	0.961710134	0.34749256	2.767570433	0.005647584	0.999551235
2.7.1.29	triose/dihydroxyacetone kinase / FAD-AMP lyase (cyclizing)	5.825853036	1.731675002	4.034528823	0.42921369	0.667767721	0.999551235
3.1.2.23	4-hydroxybenzoyl-CoA thioesterase	2.101663812	0.040549084	2.117470692	0.019149774	0.984721625	0.999551235
3.2.1.180	unsaturated chondroitin disaccharide hydrolase	78.8314747	-0.018033437	0.461188899	-0.039102061	0.968809018	0.999551235
3.4.21.66	thermitase	0.052431119	-1.040555473	4.139270063	-0.251386224	0.801515517	0.999551235
2.7.1.74	deoxycitidine kinase	0.485024588	1.168746869	4.091703715	0.285638196	0.775155245	0.999551235
2.3.1.180	3-oxoacyl-[acyl-carrier-protein] synthase III	375.8443716	0.047071888	0.198850357	0.23672016	0.812873894	0.999551235
6.3.5.10	adenosylcobyric acid synthase	242.6327014	0.14450218	0.262118337	0.551286042	0.581437605	0.999551235
1.3.1.76	uroporphyrin-III C-methyltransferase / precorrin-2 dehydrogena	35.78655682	0.483971615	0.540527156	0.895369658	0.370589515	0.999551235
3.1.3.46	6-phosphofructo-2-kinase / fructose-2,6-biphosphatase 3	0.160692243	-0.391890765	4.143731151	-0.09457437	0.924652908	0.999551235
4.1.1.8	oxalyl-CoA decarboxylase	5.420868125	-1.250987009	1.198557351	-1.043743971	0.29660386	0.999551235
2.1.1.198	16S rRNA (cytidine1402-2'-O)-methyltransferase	509.3553814	0.329966842	0.167933947	1.964860868	0.049430343	0.999551235
3.2.1.17	lysozyme	43.33186982	0.473247402	0.750049157	0.630955181	0.52806983	0.999551235
3.5.3.3	creatinase	0.047497346	0.883941495	4.12545845	0.214265034	0.830340384	0.999551235
2.4.1.60	abequosyltransferase	0.322690315	1.769211193	4.101109658	0.431398168	0.666178882	0.999551235
2.8.3.12	#N/A	116.8754764	-0.075613608	0.559951203	-0.135036066	0.892583359	0.999551235
4.2.1.75	uroporphyrinogen-III synthase	152.2832938	0.068136996	0.230434711	0.295688942	0.767467643	0.999551235
2.4.2.43	4-amino-4-deoxy-L-arabinose transferase	0.449927408	-0.063221988	4.099541689	-0.015421721	0.987695735	0.999551235
3.4.24.70	oligopeptidase A	13.63582718	0.456463031	0.590022851	0.77363619	0.439145964	0.999551235

3.6.4.-	#N/A	1559.202111	0.051923428	0.197988581	0.262254662	0.793125114	0.999551235
1.18.1.1	rubredoxin---NAD+ reductase	52.66113307	-0.838906339	0.778405908	-1.0777235	0.281157168	0.999551235
1.3.1.38	mitochondrial trans-2-enoyl-CoA reductase	0.868660033	-0.77776308	4.100752293	-0.189663512	0.849572815	0.999551235
4.2.1.151	chorismate dehydratase	11.30106601	0.488083097	0.843205555	0.578842364	0.562695543	0.999551235
2.4.1.259	alpha-1,2-mannosyltransferase	0.214256324	-0.391892261	4.143731151	-0.094574732	0.924652621	0.999551235
2.5.1.78	6,7-dimethyl-8-ribityllumazine synthase	142.8426069	0.25262368	0.286838465	0.880717586	0.378470696	0.999551235
2.7.8.12	CDP-glycerol glycerophosphotransferase	49.11916204	-0.857629654	0.525243969	-1.632821517	0.10250654	0.999551235
1.1.1.94	glycerol-3-phosphate dehydrogenase (NAD(P)+)	425.844855	0.001645446	0.193163805	0.008518396	0.993203385	0.999551235
3.5.3.8	formiminoglutamase	2.774074758	1.423328443	1.412960912	1.007337451	0.313772638	0.999551235
3.5.99.10	2-iminobutanoate/2-iminopropanoate deaminase	205.6363897	-0.061356021	0.344577573	-0.178061562	0.858674629	0.999551235
2.7.1.161	riboflavin kinase, archaea type	1.322181795	-0.492336963	4.140081342	-0.118919635	0.905339026	0.999551235
1.1.1.27	L-lactate dehydrogenase	263.1265027	-0.389023184	0.29500942	-1.318680549	0.187275932	0.999551235
3.1.21.2	deoxyribonuclease IV	300.1767179	0.348493379	0.253622121	1.374065393	0.169421382	0.999551235
2.8.3.5	3-oxoacid CoA-transferase	7.962912308	0.495075856	0.927572828	0.533732599	0.593526552	0.999551235
2.7.8.14	CDP-ribitol ribitolphosphotransferase / teichoic acid ribitol-phos	29.46695896	-0.164522419	0.515069133	-0.319418129	0.749409466	0.999551235
6.3.1.19	proteasome accessory factor A	0.403407769	1.155978333	4.068192748	0.284150335	0.776295174	0.999551235
3.4.17.13	muramoyltetrapeptide carboxypeptidase	30.21567958	0.329854501	0.589205146	0.559829635	0.575595648	0.999551235
3.4.22.70	sortase A	838.1445998	-0.108021458	0.18263837	-0.591449968	0.554218969	0.999551235
3.5.1.105	chitin disaccharide deacetylase	8.422222645	-1.43590476	1.120986491	-1.280929585	0.2002184	0.999551235
4.1.1.85	3-dehydro-L-gulonate-6-phosphate decarboxylase	0.805198269	-2.253787451	4.123574323	-0.546561617	0.584679945	0.999551235
5.1.3.24	N-acetylneuraminate epimerase	0.240767753	-1.429598065	4.109365218	-0.34788781	0.727924441	0.999551235
2.5.1.31	undecaprenyl diphosphate synthase	402.9684909	-0.055450017	0.154242004	-0.35950011	0.719220995	0.999551235
1.5.1.7	saccharopine dehydrogenase (NAD+, L-lysine forming)	270.5033177	0.16055924	0.220779379	0.727238392	0.467079931	0.999551235
3.4.19.1	acylaminoacyl-peptidase	1.683032235	1.882139382	1.35794311	1.386022263	0.165740106	0.999551235
3.1.3.73	alpha-ribazole phosphatase	202.4302529	0.138560162	0.301591199	0.45943039	0.645925129	0.999551235
2.1.1.166	23S rRNA (uridine2552-2'-O)-methyltransferase	12.94395647	-0.682325827	0.983428215	-0.693823725	0.487792762	0.999551235
1.6.5.2	NAD(P)H dehydrogenase (quinone)	9.363692982	-0.159753505	0.985393758	-0.162121491	0.871210183	0.999551235
3.4.21.110	C5a peptidase	0.866408451	1.654773276	4.069517071	0.406626449	0.684282368	0.999551235
4.7.1.1	alpha-D-ribose 1-methylphosphonate 5-phosphate C-P lyase	2.28894411	-0.642787153	2.077692179	-0.309375546	0.75703587	0.999551235
5.1.1.3	glutamate racemase	455.6094706	0.020136452	0.47967456	0.041979404	0.966515117	0.999551235
6.3.1.12	D-aspartate ligase	24.50587962	-0.140068215	0.402221691	-0.348236355	0.727662687	0.999551235
1.8.1.2	#N/A	12.775374	-0.206606699	1.119813731	-0.184500952	0.85362048	0.999551235
4.1.99.12	3,4-dihydroxy 2-butanone 4-phosphate synthase	318.5032203	0.35523807	0.224217211	1.584347915	0.113114568	0.999551235
4.1.2.14	2-dehydro-3-deoxyphosphogluconate aldolase / (4S)-4-hydroxy	365.4288842	-0.155513458	0.280231996	-0.554945403	0.578932005	0.999551235
5.4.99.27	tRNA pseudouridine13 synthase	2.224630463	-0.40407015	2.814188372	-0.143583192	0.885829614	0.999551235
1.12.99.6	#N/A	125.2879068	-0.128861494	0.507646026	-0.253841235	0.799618215	0.999551235
2.7.1.192	PTS system, N-acetylmuramic acid-specific IIB component	0.096307101	-1.192781373	4.131830079	-0.288681129	0.772825405	0.999551235
1.3.99.33	urocanate reductase	28.26826934	-2.259325026	0.944137774	-2.393003529	0.01671108	0.999551235
3.6.1.41	bis(5'-nucleosyl)-tetraphosphatase (symmetrical)	3.179764336	0.29184141	1.773048621	0.16459865	0.869259894	0.999551235
6.3.5.2	GMP synthase (glutamine-hydrolysing)	699.2410668	-0.245044991	0.177490488	-1.380609145	0.16739917	0.999551235
3.6.3.-	#N/A	1836.451269	0.226173189	0.238761255	0.947277605	0.34349734	0.999551235
2.3.1.76	diacylglycerol O-acyltransferase 1	0.015832449	0.284417857	4.139099958	0.068714904	0.945216554	0.999551235
3.1.6.8	arylsulfatase A	1.997616455	-0.729550137	3.380617253	-0.215803826	0.829140666	0.999551235
1.14.13.59	lysine N6-hydroxylase	0.353595073	-0.689967803	4.096471678	-0.168429775	0.866245186	0.999551235
2.7.1.170	anhydro-N-acetylmuramic acid kinase	29.26470967	0.153695572	0.499635152	0.307615609	0.758374841	0.999551235
2.6.1.57	aromatic-amino-acid transaminase	12.44296424	0.446557539	0.661569332	0.674997339	0.499677456	0.999551235
1.3.99.-	#N/A	55.60821594	-0.246317564	0.382036684	-0.644748461	0.519090206	0.999551235
2.7.8.36	undecaprenyl phosphate N,N'-diacetylbacillosamine 1-phospha	4.593317519	-0.315043782	1.615097139	-0.195061817	0.845344554	0.999551235
5.3.1.16	phosphoribosylformimino-5-aminoimidazole carboxamide ribot	177.1832862	-0.042070984	0.262391094	-0.160336938	0.872615663	0.999551235
3.1.2.6	hydroxyacylglutathione hydrolase	1.655433542	-1.419349665	2.031721355	-0.698594648	0.484805389	0.999551235
1.15.1.1	nickel superoxide dismutase	641.8686433	-0.338452113	0.543579851	-0.6226355	0.533524077	0.999551235
3.5.4.43	hydroxydechloroatrazine ethylaminohydrolase	0.97091914	1.746076638	3.307993564	0.527835561	0.597613473	0.999551235
2.4.99.17	S-adenosylmethionine:tRNA ribosyltransferase-isomerase	404.1357236	0.491819802	0.304441786	1.615480612	0.106206656	0.999551235
4.2.2.3	poly(beta-D-mannuronate) lyase	3.76957877	-1.432949563	1.465280035	-0.977935636	0.328106159	0.999551235
6.3.2.8	UDP-N-acetylmuramate--alanine ligase	425.8810352	-0.088433734	0.133225906	-0.663787819	0.506826123	0.999551235
2.7.1.184	sulfofructose kinase	16.85072223	0.045755047	0.875766729	0.052245702	0.958332918	0.999551235
3.5.99.6	#N/A	840.5561841	-0.366542631	0.359533031	-1.019496403	0.30796736	0.999551235
3.2.1.10	oligo-1,6-glucosidase	163.2185741	-0.386977263	0.277372035	-1.395156011	0.162968799	0.999551235
2.3.1.39	acyl-carrier-protein] S-malonyltransferase	252.4509801	-0.081593894	0.237226806	-0.343948879	0.730884732	0.999551235
2.4.99.12	3-deoxy-D-manno-octulosonic-acid transferase	102.8745696	0.408892164	0.322930505	1.26619244	0.205444193	0.999551235
3.2.1.113	mannosyl-oligosaccharide alpha-1,2-mannosidase	1.197753478	-0.242869232	4.123981459	-0.058891931	0.953038185	0.999551235
3.1.3.36	inositol polyphosphate 5-phosphatase INPP5B/F	0.685511993	-0.815442279	4.114391776	-0.198192667	0.842894325	0.999551235
1.13.12.-	#N/A	1.243420728	-0.97375313	3.865697643	-0.251895834	0.80112158	0.999551235
2.7.7.47	streptomycin 3''-adenylyltransferase	10.73053726	0.860533764	0.993000332	0.866599674	0.386161392	0.999551235
1.1.3.21	alpha-glycerophosphate oxidase	0.145244102	-1.77051308	4.136173644	-0.428055791	0.668610502	0.999551235
1.3.1.12	prephenate dehydrogenase	171.7607912	-0.135411556	0.292078663	-0.463613311	0.642924816	0.999551235
5.3.99.11	2-keto-myo-inositol isomerase	6.726854188	-0.805954792	0.656994208	-1.226730438	0.219923924	0.999551235
3.2.1.21	beta-glucosidase	1951.494518	0.126408879	0.175428935	0.720570294	0.471173936	0.999551235
1.5.5.2	RHH-type transcriptional regulator, proline utilization regulon re	58.38325883	-0.07736125	0.455956375	-0.169668096	0.865271166	0.999551235
2.1.2.9	methionyl-tRNA formyltransferase	378.080312	0.002192782	0.180812925	0.01212735	0.990324012	0.999551235
2.4.2.2	pyrimidine-nucleoside phosphorylase	232.276121	0.005741655	0.299938256	0.01914279	0.984727196	0.999551235
2.7.1.17	xylulokinase	428.2689433	-0.218867187	0.234424351	-0.933636743	0.350491305	0.999551235
2.7.7.13	mannose-1-phosphate guanylyltransferase	1345.206609	-0.969502748	0.907932319	-1.067813898	0.285604468	0.999551235
4.2.2.8	heparin/heparan-sulfate lyase	21.98860056	0.868908515	0.740079523	1.174074526	0.240365176	0.999551235
1.11.1.15	peroxiredoxin (alkyl hydroperoxide reductase subunit C)	879.9144678	-0.196532997	0.511156561	-0.384486891	0.70061761	0.999551235
3.5.4.12	dCMP deaminase	242.5971025	0.017178485	0.202584734	0.084796544	0.932423141	0.999551235
3.6.3.40	teichoic acid transport system ATP-binding protein	81.29592209	-0.481837923	0.371910399	-1.295575288	0.195121847	0.999551235
2.1.-.-	#N/A	0.192614202	-1.376625876	4.11348949	-0.334661333	0.737880573	0.999551235
3.1.11.5	exodeoxyribonuclease V	497.8412324	0.036730729	0.256315609	0.14330274	0.886051092	0.999551235
1.1.1.336	UDP-N-acetyl-D-mannosaminuronic acid dehydrogenase	33.03340853	0.5198694	0.418772251	1.241413201	0.214453145	0.999551235
1.14.13.-	#N/A	1.712504284	-0.773526039	4.092374559	-0.189016432	0.850079938	0.999551235
3.6.1.65	(d)CTP diphosphatase	0.422052821	-2.216413549	4.125225394	-0.537283018	0.591072134	0.999551235
1.2.1.81	sulfoacetaldehyde dehydrogenase	12.88492287	-0.189622756	1.228882159	-0.154305077	0.877369194	0.999551235
4.1.2.17	L-fuculose-phosphate aldolase	179.7216875	-0.733993968	0.417797854	-1.756816033	0.078949171	0.999551235
2.5.1.41	phosphoglycerol geranylgeranyltransferase	3.384201131	-1.47158326	2.370260623	-0.620852933	0.534696392	0.999551235
1.3.7.9	4-hydroxybenzoyl-CoA reductase subunit gamma	0.297488658	-1.116941734	4.123891291	-0.270846551	0.786509055	0.999551235
5.1.99.3	allantoin racemase	7.808605122	0.838321054	0.647875611	1.293953716	0.195681409	0.999551235

2.7.8.23	carboxyvinyl-carboxyphosphonate phosphorylmutase	0.047497346	0.883941495	4.12545845	0.214265034	0.830340384	0.999551235
1.1.1.205	IMP dehydrogenase	738.73706	-0.782672531	0.429142968	-1.823803699	0.068181755	0.999551235
2.4.1.325	dTDP-N-acetylfucosamine:lipid II N-acetylfucosaminyltransferas	0.215045196	-1.403546335	4.111327126	-0.341385225	0.732813597	0.999551235
2.1.3.11	N-succinyl-L-ornithine transcarbamylase	68.30817693	0.309631182	0.400189382	0.773711636	0.439101337	0.999551235
3.5.4.9	methylenetetrahydrofolate dehydrogenase (NADP+) / metheny	432.3435048	-0.179577689	0.235073275	-0.763922182	0.444913618	0.999551235
1.1.1.384	dTDP-3,4-didehydro-2,6-dideoxy-alpha-D-glucose 3-reductase	3.667088234	-0.808657346	1.20858145	-0.669096275	0.503434066	0.999551235
2.1.1.203	tRNA (cytosine34-C5)-methyltransferase	0.433524036	-0.498127949	4.11102072	-0.121168922	0.903557239	0.999551235
2.3.2.10	serine/alanine adding enzyme	1.782642193	0.266759324	2.236430594	0.119279053	0.905054278	0.999551235
4.1.2.50	6-pyruvoyltetrahydropterin/6-carboxytetrahydropterin synthas	76.58751425	0.747376966	0.368678328	2.027179006	0.042644109	0.999551235
2.7.1.49	hydroxymethylpyrimidine/phosphomethylpyrimidine kinase / t	174.8667027	-0.048497553	0.27541998	-0.176085818	0.860226522	0.999551235
3.6.-.-	#N/A	322.2767022	0.145565565	0.241381193	0.603052635	0.546473674	0.999551235
6.3.1.1	aspartate--ammonia ligase	184.1027838	0.074522584	0.294307166	0.253213623	0.80010314	0.999551235
2.1.1.298	ribosomal protein L3 glutamine methyltransferase	3.233325143	1.445623216	1.241299302	1.164604873	0.24417897	0.999551235
1.7.5.1	nitrate reductase / nitrite oxidoreductase, alpha subunit	5.245192299	-0.15028429	0.824675005	-0.182234564	0.855398649	0.999551235
1.1.1.14	L-iditol 2-dehydrogenase	109.28552	0.173843922	0.342835118	0.507077348	0.612100548	0.999551235
1.3.1.89	tRNA-dihydrouridine synthase 3	0.06939653	0.247057487	4.134346507	0.059757325	0.952348915	0.999551235
1.13.11.2	catechol 2,3-dioxygenase	0.859051352	-1.82771937	4.103933271	-0.445357965	0.656061085	0.999551235
4.3.1.2	methylaspartate ammonia-lyase	48.64895908	-0.186071745	0.579250033	-0.321228718	0.748037071	0.999551235
3.4.11.7	glutamyl aminopeptidase	1.448565954	-0.814373376	3.118353531	-0.261154923	0.793973039	0.999551235
3.5.1.98	histone deacetylase 1/2	0.803461216	-0.384919799	4.143069716	-0.092906909	0.925977517	0.999551235
5.1.3.6	UDP-glucuronate 4-epimerase	31.56312131	-0.11532919	0.613352951	-0.188030709	0.850852578	0.999551235
3.1.26.12	ribonuclease E	25.32080082	-0.633511137	0.653000151	-0.970154657	0.331969408	0.999551235
5.4.99.58	methylornithine synthase	0.369327113	0.803209304	4.043487406	0.198642712	0.84254224	0.999551235
2.7.1.26	riboflavin kinase	322.5539954	-0.271953852	0.220250277	-1.234749195	0.216923861	0.999551235
3.2.1.33	glycogen debranching enzyme	0.160692243	-0.391890765	4.143731151	-0.09457437	0.924652908	0.999551235
3.2.1.14	chitinase	63.36207878	0.518667478	0.334551611	1.550336212	0.12106084	0.999551235
1.1.3.-	#N/A	0.215045196	-1.403546335	4.111327126	-0.341385225	0.732813597	0.999551235
3.5.1.91	N-substituted formamide deformylase	117.1158786	0.544792794	0.436358783	1.248497374	0.21184897	0.999551235
3.1.1.3	triacylglycerol lipase	15.45567411	0.845042283	0.715823098	1.180518324	0.237794127	0.999551235
3.5.1.32	hippurate hydrolase	17.82442116	0.076608012	0.681862345	0.11235114	0.910544994	0.999551235
3.5.1.9	arylformamidase	22.52921188	-1.156725394	0.814920141	-1.419434047	0.155772512	0.999551235
3.1.13.4	poly(A)-specific ribonuclease	0.107128162	-0.391888715	4.143731151	-0.094573876	0.924653301	0.999551235
1.3.99.29	phytoene desaturase	1.632348624	-0.923071895	4.11939824	-0.224079305	0.822695595	0.999551235
3.5.2.18	enamidase	9.33551453	-2.063324228	1.349545768	-1.528902744	0.126288556	0.999551235
2.7.1.55	allose kinase	2.123922768	0.847481005	1.739939504	0.487074984	0.626205194	0.999551235
4.1.1.19	arginine decarboxylase	193.6903894	0.302992573	0.344864438	0.878584569	0.379626565	0.999551235
4.1.2.40	tagatose 1,6-diphosphate aldolase	44.01710737	-0.126213586	0.444778582	-0.28376723	0.776588769	0.999551235
2.1.1.64	2-polyprenyl-6-hydroxyphenyl methylase / 3-demethylubiquinc	0.275721832	-1.994544412	4.108451879	-0.485473475	0.627340524	0.999551235
2.3.1.61	2-oxoglutarate dehydrogenase E2 component (dihydrolipoamid	48.87361501	0.485145428	0.509147411	0.95285848	0.340661779	0.999551235
6.1.1.5	isoleucyl-tRNA synthetase	902.9590698	-0.080698407	0.108055637	-0.746822745	0.455170564	0.999551235
3.1.1.17	gluconolactonase	1.652406123	1.830119553	3.390059114	0.539848861	0.589301267	0.999551235
4.1.2.43	3-hexulose-6-phosphate synthase	16.96607242	-1.030557505	0.800515051	-1.287368056	0.197966033	0.999551235
4.2.1.80	2-keto-4-pentenoate hydratase	0.627092025	-2.67599035	4.095174724	-0.653449616	0.513466461	0.999551235
2.1.1.245	acetyl-CoA decarbonylase/synthase complex subunit delta	937.0964197	-1.207204024	0.668677949	-1.805359404	0.07101846	0.999551235
3.5.3.11	agmatinase	88.61984129	0.017189917	0.325721583	0.05277488	0.957911276	0.999551235
6.6.1.2	cobaltochelatase CobN	201.6340406	0.573144785	0.421720279	1.359063849	0.174126357	0.999551235
3.5.-.-	#N/A	18.06841165	-0.81024094	0.692240727	-1.170461241	0.241815403	0.999551235
3.7.1.20	fumarylpyruvate hydrolase	3.078512959	-0.8635773	1.83753892	-0.469964087	0.638380676	0.999551235
2.7.8.28	LPPG:FO 2-phospho-L-lactate transferase	4.388340055	-1.651525931	4.105018001	-0.402318804	0.687449418	0.999551235
1.1.1.85	3-isopropylmalate dehydrogenase	273.3671789	-0.310419469	0.195073108	-1.591298115	0.111542501	0.999551235
3.2.1.122	maltose-6'-phosphate glucosidase	25.24441003	-0.312374647	0.626914493	-0.49827313	0.618291544	0.999551235
2.1.2.3	phosphoribosylaminoimidazolecarboxamide formyltransferase	631.120063	0.140297601	0.213751134	0.656359562	0.511592802	0.999551235
2.7.-.-	#N/A	135.2697332	0.358007579	0.445069923	0.80438502	0.42117465	0.999551235
3.2.1.89	arabinogalactan endo-1,4-beta-galactosidase	114.6560584	0.044591766	0.426010264	0.104672985	0.916635299	0.999551235
2.8.3.8	#N/A	156.8950941	-0.673364861	0.59220575	-1.137045463	0.255519281	0.999551235
2.1.1.271	precorrin-4/cobalt-precorrin-4 C11-methyltransferase	110.7485461	-0.072906027	0.349326266	-0.208704682	0.834678785	0.999551235
1.8.4.-	#N/A	1.874139009	-1.172773872	2.19076908	-0.535325189	0.592425011	0.999551235
2.4.2.9	uracil phosphoribosyltransferase	463.3200667	-0.021994284	0.228787996	-0.096133909	0.92341422	0.999551235
3.4.-.-	#N/A	2017.633239	-0.13901101	0.122217815	-1.137403821	0.255369511	0.999551235
4.1.1.44	4-carboxymuconolactone decarboxylase	3240.638862	-1.437916457	1.468969122	-0.978860914	0.327648707	0.999551235
3.1.1.73	feruloyl esterase	0.400658548	-3.422653986	4.108980865	-0.832969074	0.404862171	0.999551235
1.14.11.17	taurine dioxygenase	0.127261092	-1.075573955	4.135512686	-0.260082373	0.794800234	0.999551235
2.1.1.130	#N/A	67.37692647	0.080800529	0.515384222	0.156777266	0.875420391	0.999551235
4.1.1.12	aspartate 4-decarboxylase	35.20555678	0.294996336	0.505230941	0.583884146	0.559298261	0.999551235
1.14.17.3	peptidylglycine monooxygenase	0.104862237	-1.29481997	4.137490949	-0.312948109	0.754320086	0.999551235
1.7.2.3	trimethylamine-N-oxide reductase (cytochrome c)	14.90176483	0.472659054	1.000090625	0.472616223	0.636487006	0.999551235
2.10.1.1	molybdopterin molybdotransferase	124.0538972	0.14630895	0.424455888	0.344697658	0.730321679	0.999551235
1.4.3.-	#N/A	1.495574844	1.312349628	3.422504931	0.383447111	0.701388278	0.999551235
2.5.1.19	3-phosphoshikimate 1-carboxyvinyltransferase	330.9966198	-0.132767361	0.276345534	-0.480439684	0.630914782	0.999551235
2.1.3.2	aspartate carbamoyltransferase	339.3596569	0.160938682	0.204156496	0.788310366	0.430515186	0.999551235
3.6.5.-	#N/A	484.9955167	-0.136995815	0.192484309	-0.711724585	0.476635341	0.999551235
3.5.1.78	glutathionylspermidine amidase/synthetase	1.535270693	-0.213388864	3.059306132	-0.069750739	0.944392056	0.999551235
3.6.3.28	#N/A	16.58175273	0.510323279	0.798334754	0.639234702	0.52267026	0.999551235
2.1.3.1	methylmalonyl-CoA carboxyltransferase 5S subunit	0.363147851	6.814120453	4.091859335	1.665287073	0.095855496	0.999551235
3.5.1.4	amidase	13.39788218	0.41710264	0.83552651	0.499209343	0.617631914	0.999551235
4.3.1.24	phenylalanine ammonia-lyase	0.052431119	-1.040555473	4.139270063	-0.251386224	0.801515517	0.999551235
6.3.5.1	NAD+ synthase (glutamine-hydrolysing)	1103.455858	-0.891801961	0.625440494	-1.425878193	0.153903505	0.999551235
6.2.1.13	acetate---CoA ligase (ADP-forming) subunit alpha	38.89400474	-1.737133676	0.902246601	-1.925342445	0.054186531	0.999551235
1.2.1.27	malonate-semialdehyde dehydrogenase (acetylating) / methylr	18.30084634	0.132965987	0.487632871	0.272676423	0.78510196	0.999551235
2.3.3.9	malate synthase	4.623532929	-2.922829692	2.013811324	-1.451392023	0.14667073	0.999551235
1.5.99.15	dihydromethanopterin reductase (acceptor)	3.714982287	-2.111130813	2.691322977	-0.784421205	0.432793	0.999551235
3.1.3.12	trehalose 6-phosphate phosphatase	28.07036042	0.405611612	0.446164216	0.909108346	0.363292938	0.999551235
5.3.1.30	5-deoxy-glucuronate isomerase	37.58263163	0.224252981	0.51614463	0.434477019	0.663942079	0.999551235
2.1.1.178	16S rRNA (cytosine1407-C5)-methyltransferase	0.518083772	4.141787838	4.039064389	1.025432486	0.305159166	0.999551235
2.7.2.7	butyrate kinase	172.3403853	0.394542211	0.345246621	1.1427837	0.253128408	0.999551235
3.2.1.133	cyclomaltodextrinase / maltogenic alpha-amylase / neopullular	0.261732976	-1.187214371	4.118287609	-0.288278645	0.773133453	0.999551235
1.5.3.22	coenzyme F420H2 oxidase	43.21632078	-1.875657791	1.092125257	-1.717438342	0.085899111	0.999551235

1.17.4.2	ribonucleoside-triphosphate reductase (thioredoxin)	1317.312582	-0.13659092	0.179614104	-0.76046879	0.446974418	0.999551235
2.7.2.11	glutamate 5-kinase	251.4337639	0.137847086	0.221870607	0.621294941	0.534405582	0.999551235
2.7.8.8	CDP-diacylglycerol---serine O-phosphatidyltransferase	109.1044487	0.058963329	0.414259041	0.142334441	0.886815844	0.999551235
2.4.1.208	1,2-diacylglycerol-3-alpha-glucose alpha-1,2-glucosyltransferase	21.76042831	-1.081079708	0.599027618	-1.804724314	0.071117833	0.999551235
2.7.1.138	ceramide kinase	0.022430994	-0.752542723	4.143731151	-0.18160993	0.855888856	0.999551235
6.1.1.7	alanyl-tRNA synthetase	1078.886596	-0.348274884	0.200399675	-1.73790144	0.082228182	0.999551235
2.6.1.52	phosphoserine aminotransferase	504.7947067	-0.08006356	0.230761716	-0.346953392	0.728626334	0.999551235
4.99.1.3	sirohydrochlorin cobaltochelatase	136.2095742	0.310857306	0.320219247	0.970763966	0.33166583	0.999551235
3.1.1.61	two-component system, chemotaxis family, protein-glutamate	143.0684675	-0.501892352	0.532762207	-0.942056974	0.346163471	0.999551235
2.5.1.90	octaprenyl-diphosphate synthase	81.05412723	0.220949958	0.429414271	0.514537996	0.606875903	0.999551235
3.5.1.16	acetylornithine deacetylase	66.04934801	0.478690437	0.272087943	1.759322489	0.078522749	0.999551235
1.1.1.202	1,3-propanediol dehydrogenase	0.419812109	1.053054815	4.077472133	0.258261683	0.796204956	0.999551235
1.20.1.1	phosphonate dehydrogenase	0.021925243	0.329456595	4.143731151	0.079507232	0.936629179	0.999551235
2.3.1.234	N6-L-threonylcarbamoyladenine synthase	411.5437197	-0.081413821	0.198135943	-0.4108988	0.681146742	0.999551235
4.1.99.22	GTP 3',8-cyclase	176.3824341	-0.435206338	0.472437578	-0.921193314	0.356949509	0.999551235
1.4.1.3	glutamate dehydrogenase (NAD(P)+)	8.230057854	0.030862778	0.989199336	0.031199756	0.975110235	0.999551235
2.6.1.97	archaeosine synthase	3.338260327	-2.972376431	2.70572323	-1.098551544	0.271963723	0.999551235
2.1.1.193	16S rRNA (uracil1498-N3)-methyltransferase	240.5805697	0.084365236	0.236851258	0.356195011	0.721694528	0.999551235
1.8.7.1	sulfite reductase (ferredoxin)	0.015832449	0.284417857	4.139099958	0.068714904	0.945216554	0.999551235
3.4.22.1	cathepsin B	8.888996236	-1.805291261	3.627186381	-0.497711193	0.618687618	0.999551235
4.2.1.20	tryptophan synthase	461.3372891	0.002027302	0.277195887	0.007313606	0.994164639	0.999551235
1.1.1.396	bacteriochlorophyllide a dehydrogenase	0.057984819	-0.752539914	4.143731151	-0.181609252	0.855889388	0.999551235
1.1.2.3	L-lactate dehydrogenase (cytochrome)	0.301644209	0.394435031	4.091049404	0.096414145	0.923191658	0.999551235
2.7.11.17	calcium/calmodulin-dependent protein kinase kinase 1	0.589204892	-0.391897735	4.143731151	-0.094576052	0.924651572	0.999551235
2.3.1.210	dTDP-4-amino-4,6-dideoxy-D-galactose acyltransferase	0.095650897	0.285548631	4.118076954	0.069340285	0.94471876	0.999551235
2.1.1.221	tRNA (guanine9-N1)-methyltransferase	0.160692243	-0.391890765	4.143731151	-0.09457437	0.924652908	0.999551235
2.3.1.202	UDP-4-amino-4,6-dideoxy-N-acetyl-beta-L-altrosamine N-acety	0.222361558	-0.391887429	4.143731151	-0.094573565	0.924653547	0.999551235
3.4.13.-	#N/A	532.3021501	0.046495946	0.180520161	0.2575665	0.796741487	0.999551235
3.4.24.64	mitochondrial-processing peptidase subunit alpha	0.167740794	0.488328207	4.131569344	0.118194363	0.905913656	0.999551235
2.7.7.73	sulfur carrier protein ThiS adenylyltransferase	10.45724072	0.005726607	0.930995208	0.00615106	0.995092195	0.999551235
2.8.1.1	#N/A	6.634840969	-0.649994608	0.892378979	-0.728384042	0.466378529	0.999551235
3.4.21.107	serine protease Do	563.0586176	-0.887788124	0.410920588	-2.160485867	0.030735077	0.999551235
3.6.3.6	#N/A	2.40170059	-2.402897571	2.723444637	-0.882300869	0.377614126	0.999551235
3.4.17.19	carboxypeptidase Taq	88.78305047	0.093951998	0.370862621	0.253333694	0.800010361	0.999551235
3.1.26.4	ribonuclease HI	787.2753457	-0.672788861	0.504692034	-1.33306812	0.18250945	0.999551235
2.1.1.13	5-methyltetrahydrofolate--homocysteine methyltransferase	505.0696423	-0.2702643	0.24473682	-1.104305841	0.26946049	0.999551235
1.1.1.103	threonine 3-dehydrogenase	187.2651899	-0.517664019	0.676454949	-0.765260155	0.444116646	0.999551235
1.8.4.2	protein-disulfide reductase (glutathione)	1.392666108	-0.348728989	4.138814988	-0.084258173	0.932851167	0.999551235
2.1.1.79	cyclopropane-fatty-acyl-phospholipid synthase	13.91181454	-0.381880907	0.722782384	-0.528348387	0.597257553	0.999551235
5.4.99.23	23S rRNA pseudouridine1911/1915/1917 synthase	11541.25338	-1.758342453	1.181809038	-1.487839742	0.136793162	0.999551235
2.5.1.131	(4-(4-[2-(gamma-L-glutamylamino)ethyl]phenoxymethyl)furan-2	2.497929715	-0.646425696	2.407244477	-0.268533463	0.788288721	0.999551235
1.2.7.1	#N/A	6286.64664	-0.302953342	0.507696428	-0.596721436	0.550693378	0.999551235
2.1.1.248	methylamine---corrinoid protein Co-methyltransferase	3.097301404	0.197928413	2.07486658	0.095393321	0.924002421	0.999551235
2.7.13.3	two-component system, sensor histidine kinase PdtaS	2106.00677	0.059455457	0.127520546	0.466242176	0.641042163	0.999551235
2.8.3.19	CoA:oxalate CoA-transferase	4.085827508	-0.458974991	1.187326526	-0.386561726	0.699080697	0.999551235
2.7.1.207	PTS system, lactose-specific IIA component	3.660562079	0.763037272	1.388111656	0.549694449	0.582528965	0.999551235
2.7.1.2	glucokinase	766.2669656	-0.075703529	0.227083029	-0.333373785	0.738852149	0.999551235
2.7.7.58	#N/A	1.219326878	-0.381893925	3.127868672	-0.122093977	0.902824592	0.999551235
6.3.2.2	glutamate--cysteine ligase	16.25644899	-1.262125391	0.63087619	-2.00059126	0.045436456	0.999551235
1.4.1.11	L-erythro-3,5-diaminohexanoate dehydrogenase	75.26061903	-0.448799146	0.539963253	-0.831166091	0.405879808	0.999551235
6.3.4.5	argininosuccinate synthase	353.5266071	0.160855025	0.231208774	0.695713326	0.486608378	0.999551235
3.5.1.118	gamma-glutamyl hercynylcysteine S-oxide hydrolase	0.788341192	0.381330548	3.605601311	0.105760597	0.9157723	0.999551235
1.5.-.-	#N/A	9.289395333	1.414808238	0.604161234	2.341772623	0.019192403	0.999551235
3.4.19.13	gamma-glutamyltranspeptidase / glutathione hydrolase	29.91949374	1.295081215	0.549452001	2.357041585	0.018421184	0.999551235
2.7.7.43	N-acylneuraminate cytidylyltransferase	24.55902711	0.254712079	0.535365327	0.475772462	0.634236491	0.999551235
2.7.1.67	phosphatidylinositol 4-kinase A	0.208380513	-0.788571102	4.142174626	-0.190376112	0.849014415	0.999551235
1.1.1.35	3-hydroxyacyl-CoA dehydrogenase	2.996316731	-0.31127061	1.264055343	-0.246247612	0.805490561	0.999551235
2.8.4.4	ribosomal protein S12 methylthiotransferase	617.9371104	0.028483138	0.200688461	0.141927132	0.887137563	0.999551235
1.1.1.58	tagaturonate reductase	366.6022532	-0.158428981	0.430235732	-0.368237617	0.712696063	0.999551235
2.2.1.1	transketolase	1674.932167	-0.471044667	0.276338117	-1.704595342	0.088269921	0.999551235
2.6.1.76	diaminobutyrate-2-oxoglutarate transaminase	23.46744053	1.426053336	1.033137839	1.380312754	0.16749037	0.999551235
3.4.24.40	serralysin	0.216806498	2.695002497	4.066486669	0.662734866	0.507500376	0.999551235
2.8.3.16	formyl-CoA transferase	5.93634884	1.247130147	1.088400215	1.145837836	0.251862257	0.999551235
3.1.3.97	3',5'-nucleoside bisphosphate phosphatase	375.6107677	-0.038307471	0.183618406	-0.208625443	0.834740647	0.999551235
4.2.1.90	L-rhamnonate dehydratase	0.858938804	-1.423175211	4.10045079	-0.347077744	0.728532913	0.999551235
3.6.1.13	ADP-ribose pyrophosphatase	194.8902596	-0.18254976	0.250235467	-0.729511936	0.465688569	0.999551235
1.1.-.-	#N/A	83.0482385	0.071922041	0.282062032	0.25498661	0.798733441	0.999551235
3.1.1.11	pectinesterase	182.2115839	0.314135017	0.399156622	0.786996883	0.431283691	0.999551235
4.1.1.81	threonine-phosphate decarboxylase	126.0137083	0.126681733	0.293614713	0.431455671	0.666137078	0.999551235
3.6.3.54	copper-transporting P-type ATPase V	207.7805605	0.07290372	0.343446497	0.212270966	0.831895645	0.999551235
3.5.4.28	5-methylthioadenosine/S-adenosylhomocysteine deaminase	210.6530955	-0.209225114	0.48385541	-0.432412473	0.665441655	0.999551235
1.6.5.8	#N/A	454.6626916	0.560589597	0.309424041	1.811719592	0.070029535	0.999551235
5.3.2.6	4-oxalocrotonate tautomerase	11.37995625	-0.01106138	0.837315809	-0.013210523	0.989459834	0.999551235
1.2.4.1	pyruvate dehydrogenase (acetyl-transferring)	62.46924544	-0.22757235	0.686570481	-0.331462474	0.740295182	0.999551235
2.7.1.86	NADH kinase	0.160692243	-0.391890765	4.143731151	-0.09457437	0.924652908	0.999551235
5.1.99.8	7,8-dihydroneopterin aldolase/epimerase/oxygenase	48.1069637	0.483548189	0.386959046	1.249610764	0.211441769	0.999551235
1.1.1.37	malate dehydrogenase	317.6601585	-0.16722106	0.395891272	-0.422391377	0.672739371	0.999551235
1.13.99.1	inositol oxygenase	0.504441937	-0.587534918	4.133760758	-0.142130847	0.886976653	0.999551235
2.7.1.197	PTS system, mannitol-specific IIA component	45.44114152	0.285627651	0.462982337	0.616929909	0.537280958	0.999551235
3.6.1.7	acylphosphatase	87.39297953	-0.279041583	0.332062263	-0.840329099	0.40072389	0.999551235
1.5.1.33	pteridine reductase	0.26805721	-2.321624453	4.123459543	-0.563028309	0.573415607	0.999551235
2.1.1.220	tRNA (adenine57-N1/adenine58-N1)-methyltransferase catalyti	4.213074941	-1.471496539	1.858161044	-0.791910122	0.428413085	0.999551235
3.4.22.48	staphopain A	0.269151628	2.120854907	4.085780813	0.51908191	0.603703623	0.999551235
2.7.4.22	uridylate kinase	553.4700535	-0.302934552	0.295770786	-1.024220669	0.305731058	0.999551235
1.1.1.22	UDPglucose 6-dehydrogenase	338.3428138	0.409982151	0.208720658	1.964262448	0.049499664	0.999551235
4.2.1.10	#N/A	224.1164232	0.190278858	0.249913643	0.761378432	0.446431066	0.999551235
3.4.19.-	#N/A	67.95115075	-0.403205396	0.317000078	-1.271940997	0.203394084	0.999551235

3.2.1.184	GDP/UDP-N,N'-diacetylbacillosamine 2-epimerase (hydrolysing)	11.03001939	0.038008172	0.651495704	0.058339865	0.953477914	0.999551235
3.4.22.37	gingipain R	506.8117507	0.122536872	1.412491731	0.086752276	0.930868423	0.999551235
6.3.5.6	aspartyl-tRNA(Asn)/glutamyl-tRNA(Gln) amidotransferase subu	502.939812	-0.250638537	0.284216089	-0.881859073	0.377853022	0.999551235
4.1.1.4	acetoacetate decarboxylase	0.721647054	-1.068175917	4.095602844	-0.260810425	0.794238705	0.999551235
4.6.1.13	1-phosphatidylinositol phosphodiesterase	8.638312496	0.046166458	0.804880705	0.057358137	0.95425991	0.999551235
3.4.11.6	aminopeptidase B	5.455731727	-0.368913607	1.349692948	-0.273331507	0.784598398	0.999551235
3.2.2.21	DNA-3-methyladenine glycosylase II	48.22412736	0.572875434	0.544532636	1.052049769	0.292776716	0.999551235
2.5.1.61	hydroxymethylbilane synthase	69.60118872	0.286994977	0.337014987	0.85157927	0.394447649	0.999551235
6.1.1.13	D-alanine--poly(phosphoribitol) ligase subunit 1	8.573527878	-0.591592865	0.950493473	-0.622406025	0.53367492	0.999551235
4.1.1.20	diaminopimelate decarboxylase	367.9980612	-0.079997389	0.18832469	-0.424784392	0.670993856	0.999551235
6.3.4.4	adenylosuccinate synthase	669.1225565	0.048529424	0.178662387	0.271626416	0.785909286	0.999551235
3.6.5.4	signal recognition particle subunit SRP54	540.0164761	-0.235351316	0.255857449	-0.919853288	0.357649432	0.999551235
1.1.1.132	GDP-mannose 6-dehydrogenase	7.106379203	0.369627443	1.345542247	0.274705193	0.783542741	0.999551235
5.3.1.5	xylose isomerase	118.9131173	0.226169596	0.464573092	0.486833182	0.626376554	0.999551235
4.2.2.23	rhamnogalacturonan endolyase	106.7907997	-0.101274737	0.367123574	-0.275860076	0.78265554	0.999551235
4.1.1.79	sulfopyruvate decarboxylase subunit alpha	1.162573941	-0.678406551	4.136120325	-0.164020023	0.869715381	0.999551235
1.17.7.4	4-hydroxy-3-methylbut-2-en-1-yl diphosphate reductase	354.784172	-0.558305451	0.419363173	-1.331317308	0.183084625	0.999551235
4.1.1.74	indolepyruvate decarboxylase	0.392755038	1.280227338	4.084444622	0.313439759	0.753946582	0.999551235
4.2.2.7	heparin lyase	8.270872475	0.892589033	0.942761997	0.94678088	0.343750447	0.999551235
4.2.1.19	imidazoleglycerol-phosphate dehydratase / histidinol-phosphat	197.1721382	-0.03880079	0.244577778	-0.158643972	0.873949383	0.999551235
6.3.-.-	#N/A	0.169214877	-0.918918869	4.119123597	-0.223086015	0.823468562	0.999551235
2.7.12.2	mitogen-activated protein kinase kinase 1	0.114087933	-0.391886818	4.143731151	-0.094573418	0.924653665	0.999551235
2.7.1.107	diacylglycerol kinase (ATP)	64.69343955	-0.776276979	0.459362958	-1.689898946	0.091047289	0.999551235
5.1.3.14	UDP-N-acetylglucosamine 2-epimerase (non-hydrolysing)	254.2481963	0.390627895	0.227272837	1.718761906	0.085657735	0.999551235
3.4.11.-	#N/A	191.0081273	0.071057754	0.342644231	0.207380563	0.835712661	0.999551235
3.1.3.89	5'-deoxynucleotidase	102.4893594	0.87530866	0.505855077	1.730354601	0.08356694	0.999551235
4.1.1.41	#N/A	0.335113387	-0.814728893	4.122502239	-0.197629703	0.843334793	0.999551235
3.1.3.57	inositol polyphosphate 1-phosphatase	0.160692243	-0.391890765	4.143731151	-0.09457437	0.924652908	0.999551235
2.4.1.18	1,4-alpha-glucan branching enzyme	1270.326465	-0.303797385	0.253667431	-1.197620775	0.231064685	0.999551235
4.1.1.37	uroporphyrinogen decarboxylase	71.82733174	0.073112332	0.348549712	0.209761563	0.833853774	0.999551235
3.6.1.61	diadenosine hexaphosphate hydrolase (ATP-forming)	0.213748207	-0.027658286	4.105801243	-0.006736392	0.994625178	0.999551235
2.7.1.195	PTS system, 2-O-A-mannosyl-D-glycerate-specific IIA componer	0.096307101	-1.192781373	4.131830079	-0.288681129	0.772825405	0.999551235
1.11.1.5	cytochrome c peroxidase	29.69912474	-0.252101565	0.502800263	-0.501395054	0.61609312	0.999551235
2.6.1.109	8-amino-3,8-dideoxy-alpha-D-manno-octulosonate transaminas	0.169128288	-0.190292387	4.108973709	-0.046311415	0.963062041	0.999551235
3.5.99.2	hydroxymethylpyrimidine/phosphomethylpyrimidine kinase / t	34.47460786	-0.002391234	0.621216152	-0.003849278	0.996928728	0.999551235
3.2.1.86	#N/A	335.5730526	-0.246116064	0.277220617	-0.88779856	0.374649118	0.999551235
5.4.99.62	D-ribose pyranase	8.727499142	-0.815621545	0.721224864	-1.130883843	0.258103985	0.999551235
6.3.2.4	D-alanine-D-alanine ligase	563.867796	-0.176028484	0.443415211	-0.396983413	0.691379692	0.999551235
1.8.1.7	glutathione reductase (NADPH)	3.926453027	-1.39185176	1.78683479	-0.77894821	0.436010223	0.999551235
6.3.1.-	#N/A	1.210030324	1.899641451	3.169615198	0.59932873	0.548953693	0.999551235
5.1.3.8	N-acylglucosamine 2-epimerase	101.6805329	0.569768418	0.346900661	1.642454114	0.100495928	0.999551235
2.7.4.3	adenylate kinase	907.9785391	-0.6816616	0.399259099	-1.707316381	0.087763263	0.999551235
2.4.1.247	beta-D-galactosyl-(1->4)-L-rhamnose phosphorylase	6.615225414	-1.158981809	1.100401231	-1.053235653	0.292233002	0.999551235
6.1.1.18	glutaminyl-tRNA synthetase	464.0232608	-0.085983918	0.141305624	-0.608496079	0.542858506	0.999551235
1.1.5.3	glycerol-3-phosphate dehydrogenase	521.1265768	-0.068759545	0.34905001	-0.196990527	0.843834951	0.999551235
2.3.1.247	3-keto-5-aminohexanoate cleavage enzyme	56.37761532	-0.51662476	0.453513692	-1.139160224	0.254636332	0.999551235
1.1.1.380	L-gulonate 5-dehydrogenase	19.37042004	0.644194839	0.723256495	0.89068656	0.373097347	0.999551235
1.1.1.262	4-hydroxythreonine-4-phosphate dehydrogenase	221.6681368	0.333677148	0.248741106	1.341463636	0.179769968	0.999551235
2.1.2.2	phosphoribosylglycinamide formyltransferase	478.4970592	0.309078462	0.212852695	1.452076811	0.146480249	0.999551235
2.1.1.131	precorrin-3B C17-methyltransferase	112.1616963	0.06440961	0.365921331	0.176020377	0.860277934	0.999551235
2.3.1.35	glutamate N-acetyltransferase / amino-acid N-acetyltransferase	232.9759184	-0.141767803	0.30438966	-0.465744476	0.641398412	0.999551235
2.7.1.151	#N/A	0.160692243	-0.391890765	4.143731151	-0.09457437	0.924652908	0.999551235
2.3.1.12	pyruvate dehydrogenase E2 component (dihydrolipoamide ace	60.27244184	-0.334567691	0.598731877	-0.55879385	0.57630242	0.999551235
4.1.2.13	fructose 1,6-bisphosphate aldolase/phosphatase	1537.630637	-0.282389074	0.434216382	-0.650341824	0.515471447	0.999551235
2.4.1.186	glycogenin	0.213123362	-1.044444849	4.139315718	-0.25232307	0.800791358	0.999551235
3.1.3.81	phosphatidylglycerophosphatase B	0.21899488	1.918812287	4.096997636	0.468345959	0.639537207	0.999551235
5.3.3.3	4-hydroxybutyryl-CoA dehydratase / vinylacetyl-CoA-Delta-ison	61.11807847	0.206319415	0.404311613	0.510298018	0.609842691	0.999551235
1.2.1.21	lactaldehyde dehydrogenase / glycolaldehyde dehydrogenase	34.86369268	-0.097447122	0.398012757	-0.244834167	0.806584837	0.999551235
3.6.3.41	heme exporter protein A	3.354275479	0.763322366	0.932735622	0.81836948	0.413146244	0.999551235
2.7.10.-	#N/A	4.805320692	-1.947880972	1.450693859	-1.342723663	0.17936147	0.999551235
1.1.1.385	dihydroanticapsin dehydrogenase	0.063329795	1.052988038	4.122789602	0.255406688	0.798409006	0.999551235
5.3.1.24	phosphoribosylanthranilate isomerase	94.20396798	-0.038533733	0.413875783	-0.093104585	0.925820475	0.999551235
5.4.99.18	5-(carboxyamino)imidazole ribonucleotide mutase	311.3499985	0.125201995	0.196331349	0.637707606	0.523664033	0.999551235
2.6.1.36	L-lysine 6-transaminase	1.472039473	-0.45872565	4.093216494	-0.112069726	0.910768121	0.999551235
2.5.1.-	#N/A	211.8230542	-0.281570863	0.64383846	-0.437331537	0.661870927	0.999551235
2.7.7.2	FAD synthetase	322.5539954	-0.271953852	0.220250277	-1.234749195	0.216923861	0.999551235
2.5.1.21	farnesyl-diphosphate farnesyltransferase	2.623950859	0.235752928	1.213553869	0.194266554	0.845967173	0.999551235
4.2.1.47	GDPmannose 4,6-dehydratase	174.6487184	0.49922631	0.242904526	2.055236751	0.039856143	0.999551235
2.3.1.19	phosphate butyryltransferase	49.06941609	0.626335833	0.375370516	1.66858026	0.095200594	0.999551235
1.7.1.13	7-cyano-7-deazaguanine reductase	52.99814833	0.612853027	0.409293175	1.497344849	0.134303551	0.999551235
6.1.1.1	tyrosyl-tRNA synthetase	382.8689634	0.032366967	0.230113296	0.140656659	0.888141186	0.999551235
4.2.1.33	3-isopropylmalate dehydratase	629.01279	-0.221164652	0.236381554	-0.935625683	0.349465946	0.999551235
4.1.1.68	#N/A	0.107481033	0.414318669	4.133619734	0.100231443	0.920160584	0.999551235
3.4.11.23	PepB aminopeptidase	1.373859969	4.62585979	4.059876301	1.139409048	0.254532584	0.999551235
3.1.3.1	alkaline phosphatase	161.6018233	0.29662995	0.389251151	0.762052851	0.446028461	0.999551235
2.7.8.26	adenosylcobinamide-GDP ribazoletransferase	150.8851714	0.145111335	0.355765335	0.407884976	0.683358123	0.999551235
1.1.3.15	glycolate oxidase	192.7034939	-0.511444234	0.403762231	-1.266696573	0.205263806	0.999551235
6.3.2.36	4-phosphopantoate---beta-alanine ligase	4.726773866	-1.870386117	2.280848726	-0.82003953	0.412193573	0.999551235
3.1.3.16	protein phosphatase	400.4206521	0.276703923	0.26772576	1.033534926	0.301353635	0.999551235
1.4.1.16	diaminopimelate dehydrogenase	159.4748883	-0.074035184	0.209061485	-0.354131149	0.723240599	0.999551235
3.2.1.67	galacturan 1,4-alpha-galacturonidase	0.121388873	-0.286462008	4.114563762	-0.069621477	0.944494942	0.999551235
2.4.1.337	1,2-diacylglycerol 3-alpha-glucosyltransferase	63.56108641	-0.589172145	0.484352147	-1.216412788	0.223827715	0.999551235
3.1.3.5	5'-nucleotidase	331.4545819	0.262314206	0.32107752	0.816980914	0.413939335	0.999551235
2.7.9.3	selenide, water dikinase	145.2147763	0.084854056	0.261409648	0.324601854	0.745482424	0.999551235
3.2.1.52	beta-N-acetylhexosaminidase	1287.687515	-0.273340495	0.277060355	-0.986573827	0.323851607	0.999551235
3.1.1.45	carboxymethylenebutenolidase	0.264436359	-0.441114387	4.14168021	-0.106506144	0.915180781	0.999551235
3.6.3.27	#N/A	156.9113029	0.344353566	0.521333389	0.660524672	0.508917191	0.999551235

5.4.2.10	phosphoglucosamine mutase	560.1447399	-0.291399612	0.242941242	-1.199465394	0.230347033	0.999551235
3.8.1.5	haloalkane dehalogenase	0.20978502	0.660437679	4.106937729	0.160810249	0.872242853	0.999551235
6.5.1.4	RNA 3'-terminal phosphate cyclase (ATP)	0.104522907	-1.463600739	4.136864693	-0.353794685	0.723492757	0.999551235
3.4.21.89	signal peptidase I	631.9471178	0.044194337	0.218656201	0.202117924	0.839824536	0.999551235
2.7.11.24	mitogen-activated protein kinase 1/3	0.48207673	-0.391896626	4.143731151	-0.094575785	0.924651784	0.999551235
3.6.1.66	XTP/dITP diphosphohydrolase	461.2721418	-0.028280983	0.279931092	-0.101028373	0.919527937	0.999551235
4.1.3.36	naphthoate synthase	46.53090059	0.171800214	0.352145696	0.487866856	0.625644152	0.999551235
2.1.1.172	16S rRNA (guanine1207-N2)-methyltransferase	12.66763079	0.293650294	1.48565314	0.197657371	0.843313144	0.999551235
4.1.2.29	6-phospho-5-dehydro-2-deoxy-D-gluconate aldolase	0.107128162	-0.391888715	4.143731151	-0.094573876	0.924653301	0.999551235
2.7.11.1	checkpoint serine/threonine-protein kinase	1213.893284	-0.130903639	0.126395354	-1.035668125	0.300356998	0.999551235
2.4.1.289	N-acetylglucosaminyl-diphospho-decaprenol L-rhamnosyltransf	0.047497346	0.883941495	4.12545845	0.214265034	0.830340384	0.999551235
3.6.3.34	iron complex transport system ATP-binding protein	275.8948902	0.319414259	0.32544278	0.981475941	0.326358093	0.999551235
2.5.1.6	S-adenosylmethionine synthetase	730.5001727	-0.152046492	0.244843155	-0.620995478	0.534602599	0.999551235
2.5.1.54	3-deoxy-7-phosphoheptulonate synthase	876.9998298	-1.686925651	0.826842307	-2.04020239	0.041330172	0.999551235
2.7.7.8	polyribonucleotide nucleotidyltransferase	1148.232146	-0.329228782	0.250074748	-1.316521496	0.187999068	0.999551235
3.5.4.5	cytidine deaminase	265.2143101	0.284083774	0.291830312	0.973455334	0.330327049	0.999551235
5.5.1.27	D-galactarolactone cycloisomerase	0.264306213	0.325087469	4.095890636	0.079369177	0.936738985	0.999551235
3.2.1.25	beta-mannosidase	250.3589538	-0.115775043	0.213695906	-0.541774735	0.587973698	0.999551235
2.1.1.148	thymidylate synthase (FAD)	154.5213535	0.214206902	0.226352942	0.94634026	0.343975065	0.999551235
4.2.1.140	gluconate/galactonate dehydratase	0.079162244	1.181119126	4.12079077	0.286624387	0.77439994	0.999551235
3.6.3.2	Mg2+-importing ATPase	72.70340602	0.232483852	0.531254331	0.437613096	0.661666776	0.999551235
4.1.1.70	glutaconyl-CoA decarboxylase	95.93604748	-0.232334497	0.536956258	-0.43268794	0.665241493	0.999551235
2.7.4.9	dTMP kinase	187.7621884	0.16568085	0.268032921	0.618136197	0.536485564	0.999551235
2.7.1.176	UDP-N-acetylglucosamine kinase	4.654161857	1.051113767	1.100583758	0.955051135	0.339551839	0.999551235
2.4.1.291	N-acetylgalactosamine-N,N'-diacetylbacillosaminyl-diphospho-u	0.158324487	1.66590149	4.102206385	0.406098897	0.684669936	0.999551235
2.7.1.30	glycerol kinase	777.9550507	-0.362495619	0.317698757	-1.14100421	0.253868171	0.999551235
2.9.1.1	L-seryl-tRNA(Ser) seleniumtransferase	26.83744539	0.01861007	0.545928875	0.034088817	0.972806326	0.999551235
1.4.3.16	L-aspartate oxidase	256.2428411	-0.002448955	0.18533961	-0.013213336	0.98945759	0.999551235
3.5.1.102	2-amino-5-formylamino-6-ribosylaminopyrimidin-4(3H)-one 5'-r	3.366320964	-3.818044461	2.361938785	-1.616487475	0.105988959	0.999551235
3.2.1.8	endo-1,4-beta-xylanase	117.6752233	0.128015744	0.434936456	0.294332062	0.768504175	0.999551235
1.1.1.117	D-arabinose 1-dehydrogenase	0.919026554	-1.453494236	4.099557083	-0.354549091	0.722927421	0.999551235
2.7.4.31	5-(aminomethyl)-3-furanmethanol phosphate kinase	6.817399672	-3.895020158	3.336620629	-1.167354815	0.243067111	0.999551235
2.1.1.10	homocysteine S-methyltransferase	3.904802519	-0.68552952	1.334137748	-0.513837136	0.607365862	0.999551235
3.6.3.32	glycine betaine/proline transport system ATP-binding protein	53.71368105	0.47841268	0.458325326	1.04382772	0.296565104	0.999551235
2.5.1.89	tritrans,polycis-undecaprenyl-diphosphate synthase [geranylge	2.984712843	-1.840984942	2.366348151	-0.777985666	0.436577464	0.999551235
1.17.1.8	4-hydroxy-tetrahydrodipicolinate reductase	407.8052854	-0.272987242	0.247689877	-1.102133219	0.270403759	0.999551235
3.1.1.83	epsilon-lactone hydrolase	14.81851815	0.176912612	0.743525722	0.237937447	0.811929611	0.999551235
2.1.1.80	chemotaxis protein methyltransferase CheR	123.6007911	-0.879285965	0.589895655	-1.490578812	0.13607211	0.999551235
2.3.1.15	glycerol-3-phosphate O-acyltransferase 1/2	587.5392468	-0.046657738	0.212372441	-0.219697703	0.826106593	0.999551235
1.17.1.1	CDP-4-dehydro-6-deoxyglucose reductase, E3	83.02591322	-0.196158339	0.405197231	-0.484105824	0.628310768	0.999551235
1.4.4.2	glycine dehydrogenase	368.0048341	0.103497723	0.301480273	0.343298492	0.731373915	0.999551235
4.1.1.32	phosphoenolpyruvate carboxykinase (GTP)	297.5921169	-0.411769068	0.567945522	-0.72501508	0.468442779	0.999551235
2.3.1.30	serine O-acetyltransferase	286.1181777	0.078294858	0.246117903	0.318119312	0.750394435	0.999551235
3.2.1.-	#N/A	103.1252457	0.105988386	0.346642372	0.305757156	0.759789552	0.999551235
5.4.99.15	(1->4)-alpha-D-glucan 1-alpha-D-glucosylmutase	0.155156823	0.782681909	4.122889507	0.189838197	0.849435923	0.999551235
4.2.99.20	2-succinyl-6-hydroxy-2,4-cyclohexadiene-1-carboxylate synthas	3.361004476	-0.550035065	1.51710077	-0.362556711	0.716936053	0.999551235
4.2.1.9	dihydroxy-acid dehydratase	540.2886476	-0.156113171	0.233086114	-0.66976607	0.503006926	0.999551235
6.3.4.2	CTP synthase	512.1476844	-0.079338897	0.17923251	-0.442659078	0.658012346	0.999551235
1.12.99.-	#N/A	251.4678543	-2.400166165	1.300395003	-1.845720846	0.064932749	0.999551235
1.1.1.363	glucose-6-phosphate 1-dehydrogenase	223.5508127	0.163067637	0.470068099	0.346902156	0.728664827	0.999551235
2.4.1.129	monofunctional glycosyltransferase	66.90475561	-0.13700687	0.383494812	-0.35725873	0.720898123	0.999551235
4.3.1.4	formiminotetrahydrofolate cyclodeaminase	55.490593	-0.138679598	0.478005865	-0.290121123	0.771723577	0.999551235
2.1.1.268	tRNAThr (cytosine32-N3)-methyltransferase	0.031664897	0.649715207	4.129795478	0.157323822	0.874989647	0.999551235
2.7.1.21	thymidine kinase	124.3847308	0.430164803	0.349312103	1.231462635	0.218149883	0.999551235
1.14.99.46	pyrimidine oxygenase	0.04941368	-0.391882467	4.143731151	-0.094572368	0.924654499	0.999551235
3.4.23.5	cathepsin D	0.214256324	-0.391892261	4.143731151	-0.094574732	0.924652621	0.999551235
2.4.1.281	4-O-beta-D-mannosyl-D-glucose phosphorylase	191.910689	-0.486281159	0.436178277	-1.114867898	0.264907035	0.999551235
3.1.3.32	bifunctional polynucleotide phosphatase/kinase	0.228120967	-0.404137747	4.143165283	-0.097543236	0.922295001	0.999551235
5.1.1.7	diaminopimelate epimerase	227.37739	-0.087292135	0.190855209	-0.457373608	0.647402531	0.999551235
2.3.2.8	arginyl-tRNA---protein transferase	4.178957052	-2.093248872	2.474990202	-0.845760468	0.397686383	0.999551235
2.1.1.314	diphthine methyl ester synthase	0.868720118	-0.541565721	4.138928449	-0.130846843	0.895896466	0.999551235
2.1.1.206	tRNA (cytidine56-2'-O)-methyltransferase	2.423959494	0.795377424	2.167200114	0.367006913	0.713613861	0.999551235
1.1.1.291	2-hydroxymethylglutarate dehydrogenase	1.511684876	1.658938309	3.378350465	0.491049797	0.623391228	0.999551235
2.5.1.48	cystathionine gamma-synthase	43.32150649	-0.464314854	0.445473019	-1.042296244	0.297274353	0.999551235
6.3.3.1	phosphoribosylformylglycinamidine cyclo-ligase	467.9122205	0.237302737	0.22374026	1.060617061	0.288863966	0.999551235
6.3.2.12	#N/A	404.1838337	-0.167043095	0.22076224	-0.756665159	0.449250493	0.999551235
2.8.1.12	molybdopterin synthase catalytic subunit	7.150256944	-1.476676319	1.17495828	-1.256790427	0.208829533	0.999551235
3.6.4.6	vesicle-fusing ATPase	0.82589221	-0.556162644	4.129160353	-0.134691462	0.892855824	0.999551235
1.21.4.1	#N/A	190.0109388	-1.775165229	0.750032244	-2.366785219	0.017943342	0.999551235
2.7.1.130	tetraacyldisaccharide 4'-kinase	112.9010033	0.383626627	0.307192149	1.248816507	0.211732195	0.999551235
1.2.1.8	betaine-aldehyde dehydrogenase	13.49926807	0.648574191	1.121034081	0.578549932	0.562892897	0.999551235
1.1.1.31	3-hydroxyisobutyrate dehydrogenase	24.6938277	-0.319656158	0.497664163	-0.642312992	0.52066998	0.999551235
2.4.1.320	beta-1,4-mannooligosaccharide/beta-1,4-mannosyl-N-acetylglu	286.1595224	-0.443110991	0.426162478	-1.039770073	0.298446736	0.999551235
2.7.6.1	ribose-phosphate pyrophosphokinase	499.4320736	-0.102262675	0.282463705	-0.362038283	0.717323423	0.999551235
4.1.2.20	2-dehydro-3-deoxyglucarate aldolase	2.948779531	0.256360404	1.308299686	0.195949298	0.844649851	0.999551235
1.1.1.378	L-rhamnose 1-dehydrogenase	0.031664897	0.649715207	4.129795478	0.157323822	0.874989647	0.999551235
1.1.1.86	ketol-acid reductoisomerase	462.1428873	-0.328384722	0.296864725	-1.106176295	0.268650221	0.999551235
5.1.3.3	aldose 1-epimerase	653.8019181	-0.113698926	0.336353821	-0.338033699	0.735337793	0.999551235
5.3.1.28	D-sedoheptulose 7-phosphate isomerase	39.20850805	0.26873791	0.337595796	0.79603453	0.426011966	0.999551235
2.3.1.47	8-amino-7-oxononanoate synthase	68.37815135	0.269317319	0.522234973	0.515701424	0.60606296	0.999551235
2.4.99.13	3-deoxy-D-manno-octulosonic-acid transferase	102.8745696	0.408892164	0.322930505	1.26619244	0.205444193	0.999551235
3.1.3.23	sugar-phosphatase	1.477961753	-0.98976207	3.637019435	-0.272135491	0.785517841	0.999551235
3.2.1.170	mannosylglycerate hydrolase	11.41345483	-0.245263969	0.905423409	-0.270883176	0.786480885	0.999551235
3.4.13.9	Xaa-Pro dipeptidase	41.36870323	-0.948086381	0.612179849	-1.548705635	0.121452499	0.999551235
2.4.2.48	7-cyano-7-deazaguanine tRNA-ribosyltransferase	3.042578273	-3.119207229	2.601060251	-1.199206065	0.230447829	0.999551235
3.6.1.26	CDP-diacylglycerol pyrophosphatase	0.18037173	-0.809817665	4.141397008	-0.195542148	0.844968545	0.999551235
2.1.1.290	tRNA wybutosine-synthesizing protein 4	0.053564081	-0.391885504	4.143731151	-0.094573101	0.924653917	0.999551235

2.1.1.294	O-antigen chain-terminating methyltransferase	0.321384486	-0.391894419	4.143731151	-0.094575252	0.924652208	0.999551235
1.1.1.41	isocitrate dehydrogenase (NAD+)	33.21155859	-0.038968495	0.408104539	-0.095486552	0.923928371	0.999551235
2.3.1.24	Acyl-CoA-dependent ceramide synthase	0.053564081	-0.391885504	4.143731151	-0.094573101	0.924653917	0.999551235
2.7.1.5	rhamnulokinase	365.1696497	-0.757934507	0.536542646	-1.412626775	0.157765492	0.999551235
2.1.1.179	16S rRNA (guanine(1405)-N(7))-methyltransferase	0.01747704	-0.715913286	4.141706174	-0.172854678	0.862765657	0.999551235
2.7.1.189	autoinducer-2 kinase	10.84554191	-0.586634389	1.122507564	-0.522610633	0.601245239	0.999551235
2.4.-.-	#N/A	73.37059448	-0.17691682	0.325239757	-0.54395816	0.586470265	0.999551235
3.2.2.9	adenosylhomocysteine nucleosidase	244.216832	0.008414902	0.205349939	0.040978351	0.967313155	0.999551235
2.8.3.1	propionate CoA-transferase	63.7064274	-0.220516207	0.525417287	-0.419697282	0.674706611	0.999551235
4.1.1.48	indole-3-glycerol phosphate synthase	101.6641702	-0.024042346	0.407784444	-0.058958468	0.952985188	0.999551235
3.5.3.18	dimethylargininase	1.952238584	-1.372869409	1.66946617	-0.822340358	0.410883213	0.999551235
2.8.1.2	thiosulfate/3-mercaptopyruvate sulfurtransferase	4.40773592	-0.731762354	1.106980169	-0.661043779	0.508584238	0.999551235
2.1.3.-	#N/A	5.301016546	-1.505107014	1.040942002	-1.445908621	0.148202834	0.999551235
2.8.1.-	#N/A	84.54624326	-0.125866974	0.798149	-0.157698592	0.87469431	0.999551235
3.6.3.7	#N/A	18.59393918	-0.193662889	0.487999484	-0.396850602	0.691477633	0.999551235
2.7.1.52	fucokinase	36.04461727	-0.193841514	0.47185536	-0.410807062	0.681214015	0.999551235
3.1.26.8	ribonuclease M5	69.66098261	0.164335232	0.362586629	0.453230259	0.650382946	0.999551235
4.1.3.30	methylisocitrate lyase	1.214680599	0.544899324	2.232083929	0.244121342	0.807136844	0.999551235
3.5.1.24	choloylglycine hydrolase	386.5802979	0.169863192	0.189225646	0.897675318	0.369358672	0.999551235
2.1.1.35	tRNA (uracil-5-)-methyltransferase	1.234391661	0.067280269	2.523493435	0.026661559	0.978729674	0.999551235
5.4.2.6	beta-phosphoglucomutase	18.10806377	-0.790448714	0.773217795	-1.02228469	0.306646177	0.999551235
2.5.1.29	geranylgeranyl diphosphate synthase, type III	398.5314581	0.097108433	0.206703279	0.469796286	0.638500568	0.999551235
2.6.1.45	#N/A	1.573124213	1.31893862	2.119898297	0.622170706	0.533829626	0.999551235
1.3.1.93	very-long-chain enoyl-CoA reductase	0.160692243	-0.391890765	4.143731151	-0.09457437	0.924652908	0.999551235
1.1.1.69	gluconate 5-dehydrogenase	430.6355441	-0.138703301	0.408533847	-0.339514834	0.734221924	0.999551235
1.1.1.-	#N/A	1599.197698	1.646143156	0.818700503	2.010678081	0.044359471	0.999551235
2.7.1.146	ADP-dependent phosphofructokinase/glucokinase	7.609187489	-1.906178209	1.062666774	-1.793768523	0.072850123	0.999551235
5.4.3.8	glutamate-1-semialdehyde 2,1-aminomutase	116.1926709	-0.174042461	0.239723711	-0.726012708	0.46783098	0.999551235
1.1.99.1	choline dehydrogenase	0.4007054	-2.060045213	4.100250673	-0.502419334	0.615372582	0.999551235
3.6.3.17	simple sugar transport system ATP-binding protein	2516.512826	-0.686889378	0.371186058	-1.850525805	0.064237802	0.999551235
1.4.1.24	3-dehydroquinate synthase II	6.211384313	0.608897041	1.499583128	0.406044206	0.68471012	0.999551235
4.2.2.17	inulin fructotransferase (DFA-I-forming)	0.203184014	0.858257445	4.124409388	0.208092205	0.835156973	0.999551235
3.6.3.29	molybdate transport system ATP-binding protein	73.99404027	-0.03471048	0.521915403	-0.06650595	0.946975021	0.999551235
1.1.1.377	L-rhamnose 1-dehydrogenase	0.031664897	0.649715207	4.129795478	0.157323822	0.874989647	0.999551235
3.1.3.64	myotubularin	0.240510691	-0.008563956	4.11557845	-0.002080863	0.998339712	0.999551235
3.1.3.3	phosphoserine phosphatase	172.8292548	-0.117779343	0.286778266	-0.41069829	0.681293781	0.999551235
2.7.8.37	alpha-D-ribose 1-methylphosphonate 5-triphosphate synthase	6.201350423	1.510284748	1.483578995	1.018000897	0.30867753	0.999551235
2.1.1.215	tRNA (guanine26-N2/guanine27-N2)-dimethyltransferase	0.68372251	0.156859503	4.135271637	0.037932092	0.969741826	0.999551235
2.6.1.51	alanine-glyoxylate transaminase / serine-glyoxylate transamina	1.573124213	1.31893862	2.119898297	0.622170706	0.533829626	0.999551235
1.2.4.4	3-methyl-2-oxobutanoate dehydrogenase (2-methylpropanoyl-	91.63417995	0.595437882	0.35408639	1.681617536	0.092643028	0.999551235
3.4.22.15	cathepsin L	6.825057858	0.302003784	3.529466165	0.08556642	0.931811094	0.999551235
3.1.2.28	1,4-dihydroxy-2-naphthoyl-CoA hydrolase	0.144460652	-1.306718627	4.119786615	-0.317181143	0.751106158	0.999551235
2.7.1.203	PTS system, D-glucosaminate-specific IIA component	2.990922439	-0.004081656	1.546161481	-0.002639864	0.997893696	0.999551235
3.2.1.187	beta-L-arabinobiosidase	0.420346293	-2.073399086	4.1276366	-0.502321131	0.615441647	0.999551235
3.2.1.80	fructan beta-fructosidase	131.8740289	0.268573378	0.302754648	0.887099108	0.375025544	0.999551235
5.1.3.32	L-rhamnose mutarotase	138.9551777	0.354399945	0.380659311	0.931016096	0.351845238	0.999551235
2.3.1.199	elongation of very long chain fatty acids protein 6	0.160692243	-0.391890765	4.143731151	-0.09457437	0.924652908	0.999551235
2.1.1.184	23S rRNA (adenine-N6)-dimethyltransferase	9.19044385	0.734223428	0.778967369	0.94255993	0.345906044	0.999551235
5.4.3.3	beta-lysine 5,6-aminomutase alpha subunit	110.9655405	-0.482672815	0.571668379	-0.844323095	0.398488884	0.999551235
2.7.1.39	homoserine kinase	172.7008888	-0.136808205	0.263928506	-0.518353272	0.604211811	0.999551235
1.3.1.87	2,3-dihydroxy-2,3-dihydrophenylpropionate dehydrogenase	0.198327269	-0.432267	4.142007235	-0.10436172	0.9168823	0.999551235
2.1.1.282	tRNA wybutosine-synthesizing protein 3	0.428512649	-0.391895981	4.143731151	-0.094575629	0.924651908	0.999551235
4.-.-.-	#N/A	0.528273679	-0.934767566	4.106902869	-0.227608881	0.819950315	0.999551235
4.1.99.3	deoxyribodipyrimidine photo-lyase	1.11007286	-0.420636668	2.785685031	-0.150999364	0.879976217	0.999551235
4.1.2.9	xylulose-5-phosphate/fructose-6-phosphate phosphoketolase	141.7183535	-0.355809519	0.327749135	-1.085615432	0.27764917	0.999551235
2.7.1.43	glucuronokinase	0.428512649	-0.391895981	4.143731151	-0.094575629	0.924651908	0.999551235
1.1.1.157	3-hydroxybutyryl-CoA dehydrogenase	1460.350109	0.04761975	0.647497111	0.073544344	0.941372959	0.999551235
1.1.1.369	myo-inositol 2-dehydrogenase / D-chiro-inositol 1-dehydrogena	80.77035126	0.044539199	0.432402587	0.103004007	0.917959791	0.999551235
1.1.1.34	hydroxymethylglutaryl-CoA reductase (NADPH)	8.97296793	-1.690656996	1.804403192	-0.936961874	0.34877817	0.999551235
3.4.22.34	legumain	28.63541982	-1.454759484	1.942239019	-0.749011563	0.453850236	0.999551235
2.1.1.86	#N/A	346.4779204	-1.967668363	1.487382458	-1.322906797	0.185866375	0.999551235
1.14.12.17	nitric oxide dioxygenase	16.24167399	-2.778065699	1.433287275	-1.938247655	0.052593015	0.999551235
2.7.8.-	#N/A	921.7247401	-0.086907836	0.221715491	-0.391979087	0.695073666	0.999551235
3.1.5.1	dGTPase	347.7338709	-0.012914798	0.21050525	-0.061351427	0.951079335	0.999551235
3.4.24.-	#N/A	3286.291944	-0.130550841	0.147168944	-0.88708145	0.37503505	0.999551235
2.1.2.13	UDP-4-amino-4-deoxy-L-arabinose formyltransferase / UDP-glu	2.389353235	0.313850735	1.360257641	0.230728889	0.817525428	0.999551235
3.1.3.24	sucrose-6-phosphatase	863.7598049	0.084647499	0.155372281	0.54480438	0.585888066	0.999551235
1.1.1.244	methanol dehydrogenase	0.059587395	-0.391885395	4.143731151	-0.094573075	0.924653937	0.999551235
3.2.1.93	trehalose-6-phosphate hydrolase	11.81364766	-0.241042308	0.768813186	-0.3135252	0.753881678	0.999551235
2.5.1.56	N-acetylneuraminate synthase	25.85352984	0.078998706	0.467889116	0.168840658	0.865921977	0.999551235
3.4.17.14	zinc D-Ala-D-Ala carboxypeptidase	0.153183881	-0.469733734	4.140729394	-0.113442268	0.909679931	0.999551235
2.7.1.15	ribokinase	117.0899583	-0.267021107	0.363213088	-0.735163781	0.462239754	0.999551235
3.6.3.24	nickel transport system ATP-binding protein	12.08036817	0.191101948	1.027059828	0.186067007	0.852392215	0.999551235
2.3.1.117	2,3,4,5-tetrahydropyridine-2,6-dicarboxylate N-succinyltransfer	33.00507093	0.365561824	0.671969922	0.544015158	0.586431042	0.999551235
2.3.2.-	#N/A	10.17339722	-0.327968904	1.071788682	-0.306001462	0.759603533	0.999551235
2.4.1.46	1,2-diacylglycerol 3-beta-galactosyltransferase	0.053564081	-0.391885504	4.143731151	-0.094573101	0.924653917	0.999551235
2.6.1.87	UDP-4-amino-4-deoxy-L-arabinose-oxoglutarate aminotransfera	0.58308361	-0.670362574	4.104080175	-0.163340516	0.870250335	0.999551235
5.99.1.-	#N/A	358.5001704	0.343357982	0.254963385	1.346695263	0.178078398	0.999551235
3.6.1.67	dihydroneopterin triphosphate diphosphatase	2.498223838	-0.14999597	1.73532671	-0.086436732	0.931119248	0.999551235
2.2.1.2	transaldolase	347.3412832	-0.386042178	0.386881802	-0.997829766	0.318361914	0.999551235
6.1.1.6	#N/A	577.45988	-0.114345058	0.142977941	-0.799739157	0.423861941	0.999551235
4.3.1.7	#N/A	29.98753477	-0.839235908	0.596530551	-1.406861572	0.159468441	0.999551235
6.1.1.24	nondiscriminating glutamyl-tRNA synthetase	6.713044222	-0.225600362	1.174293309	-0.192115854	0.847651457	0.999551235
2.4.1.25	4-alpha-glucanotransferase	826.5909988	-0.112148289	0.272639954	-0.411342094	0.680821708	0.999551235
3.4.15.6	cyanophycinase	0.722055264	0.188565871	4.107429952	0.045908481	0.963383194	0.999551235
2.4.1.182	lipid-A-disaccharide synthase	97.65809609	0.300658223	0.251931597	1.193412124	0.232708009	0.999551235
3.1.1.24	3-oxoadipate enol-lactonase	0.043850487	0.733944445	4.133023226	0.177580528	0.859052418	0.999551235

2.2.1.9	2-succinyl-5-enolpyruvyl-6-hydroxy-3-cyclohexene-1-carboxylat	86.31340977	0.144046795	0.363004466	0.396818243	0.691501497	0.999551235
3.2.1.165	exo-1,4-beta-D-glucosaminidase	3.174521815	-0.07645725	1.561254065	-0.04897169	0.960941857	0.999551235
1.1.1.18	myo-inositol 2-dehydrogenase / D-chiro-inositol 1-dehydrogena	80.77035126	0.044539199	0.432402587	0.103004007	0.917959791	0.999551235
2.4.1.7	sucrose phosphorylase	40.05453102	-0.848114158	0.58869754	-1.44066197	0.149680204	0.999551235
3.5.4.2	adenine deaminase	221.7394094	0.270355259	0.366458771	0.737750821	0.460665883	0.999551235
5.2.1.8	peptidylprolyl isomerase	1947.119805	-0.31582357	0.365277387	-0.864612982	0.387251245	0.999551235
5.1.1.18	serine racemase	34.51337451	-0.911549398	0.474539944	-1.920911843	0.054742822	0.999551235
3.5.1.97	acyl-homoserine-lactone acylase	0.654836639	0.821023241	4.106084295	0.199952846	0.84151746	0.999551235
2.6.1.13	ornithine--oxo-acid transaminase	47.38238285	0.486444924	0.487672361	0.99748307	0.318530087	0.999551235
1.10.3.10	#N/A	6.011939212	-0.556737043	2.211670478	-0.251726941	0.801252132	0.999551235
2.7.7.6	DNA-directed RNA polymerase	6413.048508	-0.370966361	0.350617885	-1.058036047	0.290039009	0.999551235
4.1.3.39	4-hydroxy 2-oxovalerate aldolase	107.0039189	0.218110533	0.374503596	0.582399034	0.560297932	0.999551235
1.21.4.2	glycine/sarcosine/betaine reductase complex component A	1374.313735	-1.210283253	0.89491859	-1.352394806	0.17624905	0.999551235
6.4.1.1	pyruvate carboxylase	459.749004	0.100080719	0.333493824	0.300097669	0.764102658	0.999551235
2.7.2.4	aspartate kinase	707.3786645	0.403745481	0.340958882	1.184147127	0.236354828	0.999551235
2.3.1.23	lysophosphatidylcholine acyltransferase / lyso-PAF acetyltransf	0.053564081	-0.391885504	4.143731151	-0.094573101	0.924653917	0.999551235
4.3.1.1	aspartate ammonia-lyase	270.3826272	0.254396532	0.39293741	0.647422531	0.517358518	0.999551235
2.3.2.23	ubiquitin-conjugating enzyme E2 R	1.849160922	-0.738452709	4.111141255	-0.179622315	0.857449085	0.999551235
2.7.1.1	hexokinase	14.71173957	0.26684687	0.573875354	0.464990991	0.641937908	0.999551235
5.1.3.1	ribulose-phosphate 3-epimerase	417.9879526	0.041220006	0.315549647	0.130629225	0.896068622	0.999551235
1.1.1.302	2,5-diamino-6-(ribosylamino)-4(3H)-pyrimidinone 5'-phosphate	2.485401298	-2.45057043	2.678091921	-0.915043435	0.360168828	0.999551235
3.1.1.-	#N/A	496.6853923	-0.127905278	0.168437604	-0.759362964	0.447635462	0.999551235
1.6.99.3	NADH dehydrogenase	65.55285561	0.463113843	0.360501314	1.284638434	0.198918662	0.999551235
1.7.99.4	#N/A	30.33693764	-0.49571387	0.649574992	-0.763135706	0.445382468	0.999551235
3.5.4.31	5-methylthioadenosine/S-adenosylhomocysteine deaminase	210.6530955	-0.209225114	0.48385541	-0.432412473	0.665441655	0.999551235
3.1.3.90	maltose 6'-phosphate phosphatase	12.88136179	-0.925629984	0.843793377	-1.096986547	0.272647273	0.999551235
4.1.1.15	glutamate decarboxylase	99.45348073	0.414580617	0.390743525	1.061004445	0.288687881	0.999551235
4.1.1.5	acetolactate decarboxylase	2.903166624	0.397733843	2.544579673	0.156306304	0.875791588	0.999551235
4.6.1.17	cyclic pyranopterin monophosphate synthase	64.49444168	-0.116157379	0.355176165	-0.327041594	0.743636426	0.999551235
2.3.1.29	glycine C-acetyltransferase	381.709002	0.009618297	0.470849795	0.020427528	0.983702325	0.999551235
2.1.1.190	23S rRNA (uracil1939-C5)-methyltransferase	488.7838662	0.11601879	0.189068619	0.613633242	0.539457711	0.999551235
1.4.7.1	glutamate synthase (ferredoxin)	105.2440678	0.050895387	0.460973973	0.110408373	0.912085513	0.999551235
3.1.26.-	#N/A	413.5075683	0.135070814	0.256575899	0.526436094	0.598585243	0.999551235
3.2.1.156	oligosaccharide reducing-end xylanase	66.937681	0.343195287	0.403743819	0.850032299	0.395307129	0.999551235
2.8.3.6	#N/A	0.04941368	-0.391882467	4.143731151	-0.094572368	0.924654499	0.999551235
3.6.3.16	arsenite/tail-anchored protein-transporting ATPase	7.85424895	-0.033387181	0.630087574	-0.05298816	0.957741341	0.999551235
6.3.5.11	cobyrinic acid a,c-diamide synthase	160.4431359	0.174681203	0.333845753	0.523239255	0.600807767	0.999551235
2.7.11.5	isocitrate dehydrogenase kinase/phosphatase	0.433381955	-1.56494776	4.101020657	-0.38159958	0.702758396	0.999551235
2.1.2.1	glycine hydroxymethyltransferase	453.8568204	0.1116174	0.229781214	0.485755117	0.6271408	0.999551235
2.6.1.44	alanine-glyoxylate transaminase / (R)-3-amino-2-methylpropior	1.573124213	1.31893862	2.119898297	0.622170706	0.533829626	0.999551235
4.2.3.12	6-pyruvoyltetrahydropterin/6-carboxytetrahydropterin synthas	76.58751425	0.747376966	0.368678328	2.027179006	0.042644109	0.999551235
3.6.4.13	ATP-dependent helicase HrpA	966.7343647	0.231445629	0.268198177	0.862964961	0.388156732	0.999551235
1.1.1.29	glycerate dehydrogenase	185.3650309	0.084797272	0.279525212	0.303361803	0.761614152	0.999551235
4.2.1.83	4-oxalmesaconate hydratase	0.163338744	-0.222828029	4.121633622	-0.054063036	0.956884942	0.999551235
2.3.2.27	protein neuralized	0.3651991	-0.531105025	4.113495548	-0.129112824	0.897268375	0.999551235
2.7.7.72	tRNA nucleotidyltransferase (CCA-adding enzyme)	259.1690687	0.022538298	0.249142207	0.090463588	0.927918828	0.999551235
4.4.1.5	lactoylglutathione lyase	653.2741697	-1.116688904	0.87133217	-1.281588059	0.199987191	0.999551235
3.2.2.1	purine nucleosidase	22.68749659	-0.343273453	0.442832366	-0.775176973	0.438235093	0.999551235
2.1.1.192	23S rRNA (adenine2503-C2)-methyltransferase	414.0283495	-0.133519458	0.141920032	-0.940807695	0.346803417	0.999551235
1.4.1.4	glutamate dehydrogenase (NADP+)	1144.214867	-0.118124095	0.327471205	-0.360715975	0.718311781	0.999551235
2.7.1.58	2-dehydro-3-deoxygalactonokinase	3.687731951	-3.122812308	1.346287592	-2.31957297	0.020363989	0.999551235
1.1.1.275	(+)-trans-carveol dehydrogenase	0.063329795	1.052988038	4.122789602	0.255406688	0.798409006	0.999551235
1.5.3.1	sarcosine oxidase	20.45793132	0.959642974	0.461588014	2.079003233	0.037617053	0.999551235
2.5.1.77	#N/A	6.782457674	-3.298129068	2.083320106	-1.583112004	0.113395938	0.999551235
5.3.1.31	sulfoquinovose isomerase	0.144460652	-1.306718627	4.119786615	-0.317181143	0.751106158	0.999551235
2.7.4.24	inositol-hexakisphosphate/diphosphoinositol-pentakisphospha	0.247565813	-0.13659414	4.129772159	-0.033075466	0.973614407	0.999551235
4.2.99.18	DNA-(apurinic or apyrimidinic site) lyase	344.0121466	0.026524406	0.33722074	0.078655915	0.937306311	0.999551235
1.16.1.9	ferric-chelate reductase (NADPH)	0.354697033	0.463994047	4.085996142	0.113557143	0.909588863	0.999551235
3.2.1.172	unsaturated rhamnogalacturonyl hydrolase	260.0740849	0.006078478	0.498493039	0.012193708	0.99027107	0.999551235
2.4.2.8	hypoxanthine phosphoribosyltransferase	315.6346228	-0.009076792	0.271316233	-0.033454659	0.973312022	0.999551235
3.2.1.37	xylan 1,4-beta-xylosidase	254.56278	0.124515727	0.315083479	0.395183295	0.69270762	0.999551235
1.1.1.307	D-xylose reductase	0.089723976	-1.20166047	4.130988035	-0.290889361	0.771135938	0.999551235
1.1.1.219	dihydroflavonol-4-reductase	13.80100873	-0.639273165	0.627874385	-1.018154554	0.308604513	0.999551235
3.6.3.35	manganese/zinc transport system ATP-binding protein	1.137234124	-0.583122638	4.097815253	-0.142300861	0.886842367	0.999551235
1.7.2.4	nitrous-oxide reductase	0.044861988	-1.012275533	4.141284466	-0.244435161	0.806893813	0.999551235
1.7.7.1	ferredoxin-nitrite reductase	22.63867316	-0.077725621	0.666281071	-0.116655905	0.907132735	0.999551235
2.4.1.227	UDP-N-acetylglucosamine--N-acetylmuramyl-(pentapeptide) py	385.4145432	-0.013202338	0.152614386	-0.086507821	0.931062739	0.999551235
2.4.2.21	nicotinate-nucleotide--dimethylbenzimidazole phosphoribosylt	150.8245576	0.163899522	0.310972687	0.527054396	0.598155814	0.999551235
4.2.1.147	5,6,7,8-tetrahydromethanopterin hydro-lyase	11.36477334	-1.081331824	1.124472468	-0.961634771	0.336233099	0.999551235
2.3.1.1	amino-acid N-acetyltransferase	239.3172202	-0.08184623	0.287055086	-0.285123777	0.775549314	0.999551235
2.1.1.223	tRNA1Val (adenine37-N6)-methyltransferase	45.39681127	0.562648568	0.527257104	1.06712373	0.285915967	0.999551235
4.1.1.39	#N/A	1.506786979	1.563561546	1.775914167	0.880426304	0.378628412	0.999551235
2.5.1.105	7,8-dihydropterin-6-yl-methyl-4-(beta-D-ribofuranosyl)aminobe	51.1828228	0.827177424	0.322108909	2.568005417	0.010228556	0.999551235
1.1.1.189	carbonyl reductase 1	0.067578231	-0.830999939	4.140710721	-0.20069017	0.840940847	0.999551235
1.17.4.1	ribonucleotide reductase, class II	558.9808335	0.721153799	0.417847729	1.725877035	0.084369537	0.999551235
2.3.1.183	phosphinothricin acetyltransferase	388.6004679	-0.634593519	0.933542229	-0.679769483	0.496650432	0.999551235
6.3.2.9	UDP-N-acetylmuramoylalanine--D-glutamate ligase	386.6429284	0.010551079	0.181191771	0.058231555	0.953564186	0.999551235
6.3.2.17	folylpolyglutamate synthase	404.2567415	-0.167111433	0.220754728	-0.757000473	0.449049579	0.999551235
2.7.8.20	phosphoglycerol transferase	16.37875243	-0.93597369	0.773701043	-1.209735593	0.226380367	0.999551235
2.3.3.10	hydroxymethylglutaryl-CoA synthase	12.96787222	-1.547007796	1.197291071	-1.29208998	0.196325986	0.999551235
2.3.3.15	sulfoacetaldehyde acetyltransferase	10.93170915	-1.28838548	1.718660192	-0.749645268	0.453468379	0.999551235
3.2.1.28	alpha,alpha-trehalase	5.065846339	-0.284341329	0.903585532	-0.314681144	0.753003763	0.999551235
2.7.2.1	acetate kinase	846.1385794	-0.455021611	0.311403324	-1.461197027	0.143961379	0.999551235
3.5.1.44	chemotaxis protein CheD	77.03386573	-0.377139387	0.515498196	-0.731601759	0.464411674	0.999551235
5.1.99.6	NAD(P)H-hydrate epimerase	0.573429732	-2.809466803	4.118164768	-0.682213306	0.495104082	0.999551235
2.1.1.199	16S rRNA (cytosine1402-N4)-methyltransferase	1239.013886	-0.406092929	0.366583938	-1.107776109	0.26795852	0.999551235
3.7.1.14	2-hydroxy-6-oxonona-2,4-dienedioate hydrolase	0.417225619	-1.20300274	4.089621669	-0.294159909	0.768635714	0.999551235

2.7.2.-	#N/A	3.340273351	-3.391510742	2.31479702	-1.465143904	0.142881661	0.999551235
2.7.7.93	phosphonoformate cytidylyltransferase	0.291631102	-0.379489665	4.142564586	-0.091607423	0.927009954	0.999551235
1.13.11.49	chlorite dismutase	0.06577573	1.018164182	4.122326352	0.246987767	0.804917691	0.999551235
3.6.4.3	#N/A	0.214256324	-0.391892261	4.143731151	-0.094574732	0.924652621	0.999551235
3.4.24.71	endothelin-converting enzyme	0.134585963	-1.298439094	4.12059973	-0.315109251	0.752678705	0.999551235
2.6.1.98	UDP-2-acetamido-2-deoxy-ribo-hexuluronate aminotransferase	0.172699552	-1.938064451	4.12647118	-0.4696663	0.638593448	0.999551235
1.1.1.4	(R,R)-butanediol dehydrogenase / meso-butanediol dehydroger	5.627014574	-0.995365853	1.692248252	-0.588191391	0.556403835	0.999551235
1.8.1.4	dihydrolipoamide dehydrogenase	347.1311495	-0.108614129	0.36065961	-0.301154124	0.763296971	0.999551235
3.1.13.5	ribonuclease D	8.99875701	-1.499770516	1.016978146	-1.474732295	0.14028453	0.999551235
1.1.1.305	UDP-4-amino-4-deoxy-L-arabinose formyltransferase / UDP-glu	2.389353235	0.313850735	1.360257641	0.230728889	0.817525428	0.999551235
5.4.99.19	16S rRNA pseudouridine516 synthase	278.6387261	-0.530030616	0.239507724	-2.213000099	0.026897639	0.999551235
2.7.1.68	1-phosphatidylinositol-4-phosphate 5-kinase	0.256999344	-0.929766121	4.119645027	-0.225690834	0.821441888	0.999551235
1.1.1.60	2-hydroxy-3-oxopropionate reductase	30.15284766	-0.783262279	0.740500242	-1.057747498	0.290170574	0.999551235
3.4.21.116	stage IV sporulation protein B	182.2910614	0.418201948	0.460832448	0.907492407	0.364146463	0.999551235
1.2.1.89	D-glyceraldehyde dehydrogenase (NADP+)	0.057984819	-0.752539914	4.143731151	-0.181609252	0.855889388	0.999551235
2.5.1.42	geranylgeranylglycerol-phosphate geranylgeranyltransferase	0.483729329	-0.56743358	4.138440309	-0.137112907	0.890941551	0.999551235
1.1.1.136	UDP-N-acetyl-D-glucosamine dehydrogenase	10.54149	-0.350088502	0.683955991	-0.511858228	0.608750235	0.999551235
2.5.1.128	N4-bis(aminopropyl)spermidine synthase	2.284826551	0.180977224	2.589853606	0.069879326	0.944289708	0.999551235
2.8.4.3	tRNA-2-methylthio-N6-dimethylallyladenosine synthase	451.3763314	0.167456042	0.151366534	1.106295014	0.268598849	0.999551235
3.2.1.40	alpha-L-rhamnosidase	406.0979955	0.131629895	0.337948393	0.389497028	0.696908509	0.999551235
1.1.1.381	3-hydroxy acid dehydrogenase / malonic semialdehyde reducta	0.590372679	-0.760259965	4.100792444	-0.185393427	0.852920463	0.999551235
3.5.2.7	imidazolonepropionase	167.9124087	0.028354733	0.389169601	0.072859578	0.94191786	0.999551235
5.1.99.4	alpha-methylacyl-CoA racemase	0.26814328	-0.391889726	4.143731151	-0.09457412	0.924653107	0.999551235
6.3.4.20	7-cyano-7-deazaguanine synthase	59.4077229	0.298770442	0.419709438	0.711850664	0.476557256	0.999551235
6.3.2.14	bifunctional isochorismate lyase / aryl carrier protein	1.81108835	-0.877067919	2.480833986	-0.35353753	0.723685498	0.999551235
1.4.3.19	glycine oxidase	0.087385198	-1.222945126	4.138042439	-0.295537115	0.767583604	0.999551235
3.1.3.8	3-phytase	0.021925243	0.329456595	4.143731151	0.079507232	0.936629179	0.999551235
2.4.1.34	1,3-beta-glucan synthase	0.304865092	-2.516195868	4.118839178	-0.610899275	0.541266265	0.999551235
3.1.11.2	exodeoxyribonuclease III	196.406447	-0.121545429	0.249447186	-0.487259169	0.626074679	0.999551235
2.1.1.33	tRNA (guanine-N7-)-methyltransferase	269.7709139	0.284495658	0.274868086	1.035026155	0.300656697	0.999551235
3.5.1.53	N-carbamoylputrescine amidase	98.58245507	0.567015558	0.383856794	1.477153896	0.139634389	0.999551235
1.9.3.1	#N/A	2.510583364	-4.435334449	2.641808419	-1.678900868	0.093171365	0.999551235
3.2.1.24	alpha-mannosidase	220.3015912	-0.054789802	0.265638906	-0.206256694	0.836590404	0.999551235
3.6.3.3	#N/A	686.2195542	0.090186389	0.402488061	0.22407221	0.822701116	0.999551235
4.2.1.40	glucarate dehydratase	5.876213181	0.026817255	1.088133327	0.024645192	0.980337972	0.999551235
2.4.2.17	ATP phosphoribosyltransferase	161.4871201	0.053946086	0.242483031	0.222473654	0.823945181	0.999551235
1.2.7.3	#N/A	1651.051343	0.121090853	0.369852439	0.327403149	0.743362985	0.999551235
2.7.8.38	archaetidylserine synthase	1.483140351	-1.563999898	4.136358588	-0.378110327	0.705348637	0.999551235
2.4.2.1	#N/A	482.41952	0.072362192	0.225696531	0.320617211	0.748500493	0.999551235
1.3.99.26	phytoene desaturase	1.632348624	-0.923071895	4.11939824	-0.224079305	0.822695595	0.999551235
2.4.1.173	sterol 3beta-glucosyltransferase	0.102122587	-0.447703369	4.141447253	-0.10810312	0.913913893	0.999551235
2.7.1.73	inosine kinase	0.288921303	-1.472175659	4.106420589	-0.358505815	0.719964817	0.999551235
4.1.1.25	tyrosine decarboxylase	5.813486058	-2.103869577	4.081645215	-0.51544646	0.606241074	0.999551235
3.2.1.54	cyclomaltodextrinase / maltogenic alpha-amylase / neopullular	0.261732976	-1.187214371	4.118287609	-0.288278645	0.773133453	0.999551235
2.3.1.242	KDO2-lipid IV(A) palmitoleoyltransferase	3.639820526	-5.680351115	2.858239193	-1.987360305	0.046882483	0.999551235
2.6.1.106	dTDP-3-amino-3,4,6-trideoxy-alpha-D-glucose transaminase	0.02470684	-0.391881853	4.143731151	-0.09457222	0.924654616	0.999551235
6.3.1.2	glutamine synthetase	942.2618412	-0.13571654	0.165834381	-0.818386026	0.413136798	0.999551235
3.6.3.30	iron(III) transport system ATP-binding protein	109.2747114	-0.05671536	0.414121494	-0.136953432	0.891067604	0.999551235
6.3.2.3	glutathione synthase	0.92520644	-0.759860671	2.615164634	-0.290559401	0.771388314	0.999551235
6.3.2.39	aerobactin synthase	0.288921303	-1.472175659	4.106420589	-0.358505815	0.719964817	0.999551235
3.5.1.82	N-acyl-D-glutamate deacylase	0.031664897	0.649715207	4.129795478	0.157323822	0.874989647	0.999551235
5.1.1.4	proline racemase	4.45986566	0.39860839	0.961872833	0.414408616	0.67857488	0.999551235
2.7.1.60	N-acylmannosamine kinase	0.427661618	-0.269003523	4.106398236	-0.065508387	0.947769229	0.999551235
1.8.5.4	sulfide:quinone oxidoreductase	10.70375631	0.733091473	0.779301693	0.940703041	0.34685706	0.999551235
2.1.1.224	23S rRNA (adenine-C8)-methyltransferase	2.006073184	-1.05515203	3.740508804	-0.282087835	0.777876161	0.999551235
6.2.1.30	phenylacetate-CoA ligase	364.9902406	0.523860402	0.309547627	1.692341844	0.090580814	0.999551235
3.4.21.88	repressor LexA	513.2102101	-0.388937473	0.305654078	-1.272475982	0.203204053	0.999551235
2.7.1.89	thiamine kinase	0.342787921	-1.382252603	4.119360353	-0.3355503	0.737210008	0.999551235
2.4.1.64	alpha,alpha-trehalose phosphorylase	6.474905408	-0.543034498	1.074751975	-0.505264945	0.613372761	0.999551235
3.6.1.23	dUTP pyrophosphatase	305.483228	0.192524165	0.236236921	0.814962217	0.415093938	0.999551235
1.1.1.26	glyoxylate reductase	1.612855192	-0.374217533	2.621711361	-0.142737885	0.886497196	0.999551235
3.5.1.19	nicotinamidase	4.871855705	-0.654569383	1.403960729	-0.466230551	0.641050484	0.999551235
2.4.1.12	cellulose synthase (UDP-forming)	7.854470084	-0.034330203	0.957242542	-0.035863641	0.971391088	0.999551235
3.1.21.3	type I restriction enzyme, R subunit	1475.812021	0.295354276	0.255063183	1.157965147	0.24687826	0.999551235
2.1.1.189	23S rRNA (uracil747-C5)-methyltransferase	0.723122727	0.716149104	4.080737134	0.175495035	0.860690672	0.999551235
4.2.1.68	L-fuconate dehydratase	0.164310313	0.400036774	4.131899786	0.096816669	0.922871986	0.999551235
4.6.1.12	2-C-methyl-D-erythritol 2,4-cyclodiphosphate synthase	260.3833251	0.12591731	0.20564223	0.612312513	0.540331012	0.999551235
1.3.8.4	isovaleryl-CoA dehydrogenase	0.107128162	-0.391888715	4.143731151	-0.094573876	0.924653301	0.999551235
6.3.5.3	phosphoribosylformylglycinamidine synthase	1280.080832	-0.015248518	0.23285758	-0.065484309	0.947788399	0.999551235
3.2.1.45	glucosylceramidase	92.54275142	1.092202724	0.550666953	1.983417959	0.047320763	0.999551235
2.1.1.163	demethylmenaquinone methyltransferase / 2-methoxy-6-polyp	59.19781542	0.530023609	0.374912485	1.413726218	0.157442304	0.999551235
3.4.23.-	#N/A	81.85829158	0.423333993	0.447682731	0.945611619	0.344346716	0.999551235
1.1.1.339	dTDP-6-deoxy-L-talose 4-dehydrogenase (NAD+)	9.784419663	0.017936194	0.564611365	0.031767327	0.974657602	0.999551235
6.5.1.-	#N/A	57.77620169	-0.671839571	0.860082018	-0.781134307	0.434723506	0.999551235
1.5.5.-	#N/A	2.954894685	-1.341372734	1.568466227	-0.855213017	0.392433231	0.999551235
1.1.1.298	malonyl-CoA reductase / 3-hydroxypropionate dehydrogenase	0.051061294	-0.40886677	4.142956969	-0.098689601	0.921384726	0.999551235
2.7.1.72	streptomycin 6-kinase	0.107128162	-0.391888715	4.143731151	-0.094573876	0.924653301	0.999551235
5.4.99.28	tRNA pseudouridine32 synthase / 23S rRNA pseudouridine746 s	73.12648985	0.279600489	0.366746292	0.762381228	0.445832507	0.999551235
2.7.1.156	adenosylcobinamide kinase / adenosylcobinamide-phosphate g	169.949037	0.191590066	0.29555048	0.648248198	0.516824433	0.999551235
2.3.1.18	galactoside O-acetyltransferase	10.30660446	-0.590034153	0.618811058	-0.95349646	0.340338589	0.999551235
4.99.1.4	uroporphyrin-III C-methyltransferase / precorrin-2 dehydrogena	35.78655682	0.483971615	0.540527156	0.895369658	0.370589515	0.999551235
3.2.1.97	endo-alpha-N-acetylgalactosaminidase	151.0616393	-1.683479359	0.629265557	-2.675308287	0.007466056	0.999551235
4.1.1.87	malonate decarboxylase beta subunit	4.427733717	0.248666136	1.594385416	0.155963629	0.87606169	0.999551235
3.1.3.95	myotubularin	0.240510691	-0.008563956	4.11557845	-0.002080863	0.998339712	0.999551235
4.2.1.5	D-arabinonate dehydratase	2.260122362	-0.410052775	2.440281976	-0.168034997	0.866555747	0.999551235
1.2.99.7	aldehyde oxidoreductase	248.9810997	-0.904307004	0.612105994	-1.477369953	0.139576496	0.999551235
2.4.2.53	undecaprenyl-phosphate 4-deoxy-4-formamido-L-arabinose tra	32.80543789	-0.188029177	0.512201602	-0.36709994	0.713544472	0.999551235

1.1.1.173	L-rhamnose 1-dehydrogenase	0.031664897	0.649715207	4.129795478	0.157323822	0.874989647	0.999551235
1.3.7.11	digeranylgeranylglycerophospholipid reductase	9.718970903	-1.852973622	1.468692422	-1.261648521	0.207075288	0.999551235
2.7.1.169	pantoate kinase	2.104766928	-1.757833213	2.944393432	-0.597010302	0.550500502	0.999551235
3.6.1.40	exopolyphosphatase / guanosine-5'-triphosphate,3'-diphosphat	219.8727794	-0.131887497	0.43127516	-0.305808239	0.759750655	0.999551235
6.1.1.12	aspartyl-tRNA synthetase	693.8225422	-0.08753614	0.168300902	-0.520116881	0.602982113	0.999551235
3.5.1.3	omega-amidase	0.286407904	-0.071010865	4.09620532	-0.017335768	0.986168751	0.999551235
1.5.98.2	5,10-methylenetetrahydromethanopterin reductase	262.9153422	-2.674337217	1.21991397	-2.192234274	0.028362595	0.999551235
2.1.1.264	23S rRNA (guanine2445-N2)-methyltransferase / 23S rRNA (gua	1.561324307	-0.252801989	1.699975064	-0.148709234	0.881783075	0.999551235
1.1.1.282	quinate/shikimate dehydrogenase	0.314357693	0.641768672	4.102921438	0.15641749	0.875703952	0.999551235
3.1.3.37	fructose-1,6-bisphosphatase I / sedoheptulose-1,7-bisphosphat	0.301146343	-1.411421797	4.116287858	-0.342887049	0.731683435	0.999551235
2.4.2.10	orotate phosphoribosyltransferase	602.0547244	-0.300825995	0.265842616	-1.131594322	0.257805028	0.999551235
3.1.7.2	GTP diphosphokinase / guanosine-3',5'-bis(diphosphate) 3'-diph	2.493487671	-0.67417035	2.14139587	-0.314827519	0.752892616	0.999551235
3.5.4.10	phosphoribosylaminoimidazolecarboxamide formyltransferase	631.4865603	0.15061186	0.21316435	0.70655276	0.479844455	0.999551235
2.4.1.319	beta-1,4-mannooligosaccharide/beta-1,4-mannosyl-N-acetylglu	286.1595224	-0.443110991	0.426162478	-1.039770073	0.298446736	0.999551235
3.6.3.38	capsular polysaccharide transport system ATP-binding protein	2.063563325	-0.138431376	2.73482469	-0.050618007	0.959629914	0.999551235
2.3.1.169	#N/A	571.4431582	-1.328420875	0.584836451	-2.271439944	0.023120359	0.999551235
5.4.2.2	phosphoglucomutase	1276.024885	-0.500987547	0.275501997	-1.818453418	0.06899486	0.999551235
5.2.1.1	maleate isomerase	0.513448466	1.167630945	4.092055743	0.285340919	0.775382967	0.999551235
2.7.7.62	adenosylcobinamide kinase / adenosylcobinamide-phosphate g	172.204231	0.194802537	0.295677896	0.658833616	0.510002618	0.999551235
2.1.1.265	tellurite methyltransferase	1.446509712	-1.597669761	2.214438243	-0.721478581	0.470615114	0.999551235
3.5.1.25	N-acetylglucosamine-6-phosphate deacetylase	770.3948878	-0.376318235	0.400788808	-0.938943971	0.347759512	0.999551235
3.4.24.55	protease III	0.192614202	-1.376625876	4.11348949	-0.334661333	0.737880573	0.999551235
2.1.1.219	tRNA (adenine57-N1/adenine58-N1)-methyltransferase catalyti	4.213074941	-1.471496539	1.858161044	-0.791910122	0.428413085	0.999551235
4.1.3.4	hydroxymethylglutaryl-CoA lyase	5.591552884	1.33342747	1.191967501	1.118677706	0.263277659	0.999551235
1.1.5.4	malate dehydrogenase (quinone)	0.675139586	0.708310633	4.054752928	0.174686509	0.861325969	0.999551235
1.7.2.1	nitrite reductase (NO-forming)	0.06577573	1.018164182	4.122326352	0.246987767	0.804917691	0.999551235
4.1.1.47	tartronate-semialdehyde synthase	3.024359811	-1.932255909	1.628455187	-1.186557619	0.235402161	0.999551235
2.1.2.10	aminomethyltransferase	205.0394189	0.120573422	0.307888153	0.391614362	0.695343175	0.999551235
2.7.8.1	ethanolaminephosphotransferase	0.129559156	-0.714640491	4.141236185	-0.172566948	0.862991833	0.999551235
2.7.1.40	pyruvate kinase	620.5141539	-0.205756161	0.19101485	-1.07717364	0.281402699	0.999551235
2.7.1.11	6-phosphofructokinase 1	1549.328	-0.417706124	0.251743874	-1.659250401	0.097065345	0.999551235
6.1.1.21	#N/A	382.5298565	-0.139833646	0.176512833	-0.792201021	0.428243473	0.999551235
1.97.1.4	#N/A	3571.408187	0.394370888	0.524764632	0.751519564	0.452340031	0.999551235
6.3.2.13	UDP-N-acetylmuramoyl-L-alanyl-D-glutamate--2,6-diaminopime	592.4151967	-0.064496446	0.14850983	-0.434290756	0.664077316	0.999551235
3.2.2.24	ADP-ribosyl-[dinitrogen reductase] hydrolase	1.660127455	0.709545653	2.107548653	0.336668694	0.736366665	0.999551235
5.-.-.-	#N/A	209.3950617	-0.059147636	0.769859638	-0.076829117	0.938759487	0.999551235
3.2.2.28	double-stranded uracil-DNA glycosylase	0.132218179	-0.420009541	4.142488731	-0.10139063	0.919240374	0.999551235
2.7.1.4	fructokinase	447.9267559	-0.024489345	0.26569921	-0.092169432	0.926563424	0.999551235
2.7.1.14	sedoheptulokinase	8.305635679	-0.37787399	0.867858814	-0.435409521	0.663265201	0.999551235
1.11.1.-	#N/A	2.773257521	-0.846729172	2.155899141	-0.3927499	0.694504211	0.999551235
2.3.1.4	glucosamine-phosphate N-acetyltransferase	0.549114054	0.234061105	4.103274465	0.057042517	0.954511326	0.999551235
4.1.1.11	aspartate 1-decarboxylase	77.14565317	-0.022135624	0.293914792	-0.075313065	0.939965627	0.999551235
3.4.21.81	streptogrisin B	0.59175187	-0.742169056	4.104933498	-0.180799289	0.856525122	0.999551235
2.7.1.165	glycerate 2-kinase	139.7135454	0.28414242	0.314892706	0.902346784	0.366872681	0.999551235
4.4.1.1	cystathionine gamma-lyase	1.197801709	-3.001221231	4.07412063	-0.736654975	0.461332194	0.999551235
1.2.1.87	propionaldehyde dehydrogenase	211.2777077	-0.95257669	0.560470399	-1.699602139	0.089205788	0.999551235
2.8.3.10	#N/A	110.095947	0.213344784	0.386729983	0.551663417	0.581178978	0.999551235
4.4.1.14	1-aminocyclopropane-1-carboxylate synthase	0.438278362	0.778110002	4.115921805	0.189048782	0.850054584	0.999551235
1.3.1.2	dihydropyrimidine dehydrogenase (NADP+)	0.358716976	-0.995066254	4.11268822	-0.241950326	0.808818654	0.999551235
3.10.1.1	N-sulfoglucosamine sulfohydrolase	0.192614202	-1.376625876	4.11348949	-0.334661333	0.737880573	0.999551235
2.7.1.51	L-fuculokinase	5.794631649	-0.692939103	1.249545504	-0.554552916	0.579200502	0.999551235
4.1.3.44	tRNA wybutosine-synthesizing protein 1	3.612380128	-1.663152374	3.187865754	-0.521713429	0.601869873	0.999551235
1.1.1.30	3-hydroxybutyrate dehydrogenase	0.609117398	1.660266276	4.040415725	0.410914715	0.681135072	0.999551235
5.4.99.20	23S rRNA pseudouridine2457 synthase	1.635705765	-0.438580234	1.675929726	-0.26169369	0.793557606	0.999551235
2.4.99.16	starch synthase (maltosyl-transferring)	2.015113309	-0.157410242	2.397111164	-0.065666642	0.947643231	0.999551235
2.7.4.28	[pyruvate, water dikinase]-phosphate phosphotransferase / [py	11.94070215	-0.84862954	0.771207296	-1.100390965	0.271161813	0.999551235
3.4.23.51	hydrogenase 3 maturation protease	3.847375802	1.113453146	1.388795532	0.801740156	0.422703282	0.999551235
2.7.1.208	PTS system, alpha-glucoside-specific IIB component	45.94357866	-0.5709797	0.551863343	-1.034639656	0.300837227	0.999551235
1.6.2.4	NADPH-ferrihemoprotein reductase	0.267820405	-0.391893442	4.143731151	-0.094575017	0.924652395	0.999551235
6.6.1.1	magnesium chelatase subunit H	18.92594321	1.142027646	0.832678545	1.371510835	0.170215768	0.999551235
1.1.1.102	3-dehydrosphinganine reductase	0.428512649	-0.391895981	4.143731151	-0.094575629	0.924651908	0.999551235
3.6.3.8	Ca2+-transporting ATPase	547.2942701	0.19810697	0.203619402	0.972927769	0.330589202	0.999551235
1.1.1.36	acetoacetyl-CoA reductase	0.635026121	1.548171108	4.052235123	0.382053623	0.702421591	0.999551235
2.4.1.290	N,N'-diacetylbacillosaminyl-diphospho-undecaprenol alpha-1,3-	0.063329795	1.052988038	4.122789602	0.255406688	0.798409006	0.999551235
3.1.3.11	fructose-1,6-bisphosphatase I / sedoheptulose-1,7-bisphosphat	417.2305742	-0.158001394	0.214054773	-0.738135348	0.460432205	0.999551235
6.1.1.2	tryptophanyl-tRNA synthetase	437.2335089	-0.17500548	0.207127597	-0.844916286	0.398157581	0.999551235
1.1.1.79	glyoxylate/hydroxypyruvate reductase	2.265448947	-0.994105335	3.061699676	-0.324690675	0.745415193	0.999551235
6.3.2.34	coenzyme F420-0:L-glutamate ligase / coenzyme F420-1:gamm	4.270530574	-2.918403008	1.940261394	-1.504128782	0.132548211	0.999551235
1.1.1.374	UDP-N-acetylglucosamine 3-dehydrogenase	15.70625512	-3.28940502	2.669678244	-1.232135381	0.217898517	0.999551235
2.4.1.21	starch synthase	1036.64266	-0.371857862	0.375860268	-0.989351343	0.322491272	0.999551235
2.3.2.3	phosphatidylglycerol lysyltransferase	8.777805559	-1.057010358	1.082825068	-0.976159852	0.328985255	0.999551235
3.2.1.58	glucan 1,3-beta-glucosidase	2.050744339	1.114353527	2.151670486	0.517901572	0.604526945	0.999551235
1.1.1.81	glyoxylate/hydroxypyruvate reductase	77.79447013	0.374601368	0.378499132	0.989702055	0.32231977	0.999551235
2.7.11.25	mitogen-activated protein kinase kinase kinase 8	0.2108833	-0.752550059	4.143731151	-0.1816117	0.855887466	0.999551235
1.8.99.5	dissimilatory sulfite reductase alpha subunit	158.2360549	0.18203078	0.784081955	0.232157849	0.816415415	0.999551235
2.1.1.63	methylated-DNA-protein]-cysteine S-methyltransferase	145.0292668	0.390002947	0.39454164	0.988496291	0.32290965	0.999551235
4.2.99.21	salicylate synthetase	0.795163648	-1.567281702	4.097170513	-0.38252782	0.702069898	0.999551235
2.3.1.57	diamine N-acetyltransferase	200.7568856	0.977942101	0.478343645	2.044434186	0.040910673	0.999551235
2.4.1.288	galactofuranosylgalactofuranosylrhamnosyl-N-acetylglucosami	0.791497509	2.074963488	4.039001176	0.513731836	0.607439491	0.999551235
2.4.2.14	amidophosphoribosyltransferase	674.2738087	0.105960987	0.196208161	0.540043729	0.589166876	0.999551235
2.7.7.39	glycerol-3-phosphate cytidylyltransferase	6.785358283	-0.789013809	1.171499683	-0.673507488	0.500624487	0.999551235
4.2.2.10	pectin lyase	22.34609549	2.356262693	2.060503304	1.14353745	0.252815515	0.999551235
1.3.5.1	#N/A	497.6955786	0.147059515	0.232914414	0.631388639	0.527786443	0.999551235
4.2.1.6	galactonate dehydratase	27.36780082	0.539163	0.458554603	1.175788002	0.239679602	0.999551235
4.3.2.1	argininosuccinate lyase	310.1718153	0.125088613	0.203093463	0.61591649	0.537949637	0.999551235
6.1.2.1	D-alanine---(R)-lactate ligase	0.157319219	-0.752548003	4.143731151	-0.181611204	0.855887856	0.999551235
2.2.1.6	#N/A	1048.382309	-0.330031654	0.21495353	-1.535362802	0.124694736	0.999551235

2.3.1.102	acetyl CoA:N6-hydroxylysine acetyl transferase	0.192614202	-1.376625876	4.11348949	-0.334661333	0.737880573	0.999551235
2.6.1.17	acetylornithine/N-succinyldiaminopimelate aminotransferase	610.7636172	-1.133688153	0.546342043	-2.075052005	0.037981715	0.999551235
3.4.24.15	thimet oligopeptidase	1.127149497	0.180938461	2.66319624	0.067940341	0.945833125	0.999551235
3.1.1.31	6-phosphogluconolactonase	234.1506305	0.12997573	0.319986933	0.406190743	0.684602455	0.999551235
3.6.1.31	phosphoribosyl-ATP pyrophosphohydrolase	255.6035093	-0.138893145	0.237864301	-0.583917571	0.559275771	0.999551235
1.20.4.1	arsenate reductase (glutaredoxin)	97.46378776	0.61479532	0.600809656	1.023278028	0.306176409	0.999551235
2.7.7.60	2-C-methyl-D-erythritol 4-phosphate cytidylyltransferase	364.2618499	0.185739225	0.156517622	1.186698487	0.235346572	0.999551235
2.7.7.3	pantetheine-phosphate adenylyltransferase	482.6370214	-0.047887792	0.400622514	-0.119533452	0.904852739	0.999551235
2.1.3.6	putrescine carbamoyltransferase	14.52936115	-0.312133404	0.746903644	-0.417903174	0.676017911	0.999551235
3.6.1.1	inorganic pyrophosphatase	704.5610825	0.007590146	0.201746758	0.037622146	0.96998895	0.999551235
2.1.1.107	uroporphyrin-III C-methyltransferase	91.90494531	-0.006393319	0.303995887	-0.02103094	0.983220975	0.999551235
1.1.1.337	L-2-hydroxycarboxylate dehydrogenase (NAD+)	6.338592059	-1.177646889	1.673103377	-0.70386977	0.481513879	0.999551235
3.2.1.143	poly(ADP-ribose) glycohydrolase	0.214256324	-0.391892261	4.143731151	-0.094574732	0.924652621	0.999551235
1.1.1.42	isocitrate dehydrogenase	416.2986	-0.190546861	0.252380721	-0.754997686	0.45025037	0.999551235
4.2.1.106	bile-acid 7alpha-dehydratase	6.338790237	-0.868364903	1.134037644	-0.765728464	0.443837887	0.999551235
3.1.11.3	exodeoxyribonuclease (lambda-induced)	0.5133427	1.132883037	4.112115261	0.275498853	0.782933007	0.999551235
2.7.7.18	nicotinate-nucleotide adenylyltransferase	260.0595274	-0.053802841	0.18576749	-0.289624635	0.772103418	0.999551235
2.8.3.9	acetate CoA/acetoacetate CoA-transferase alpha subunit	155.1818872	-0.703730672	0.595908888	-1.180936694	0.237627873	0.999551235
4.1.1.1	pyruvate decarboxylase	3.712525794	-5.57327268	2.944388392	-1.892845623	0.058378402	0.999551235
3.6.4.7	peroxisome-assembly ATPase	0.267820405	-0.391893442	4.143731151	-0.094575017	0.924652395	0.999551235
1.14.-.-	#N/A	13.38369178	0.934174026	0.59272851	1.576057184	0.115012625	0.999551235
2.7.1.35	pyridoxine kinase	246.3550928	0.416582371	0.297576599	1.399916433	0.161538344	0.999551235
1.1.1.289	sorbose reductase	0.33823785	-1.356102893	4.129010793	-0.328432877	0.742584388	0.999551235
3.2.1.135	cyclomaltodextrinase / maltogenic alpha-amylase / neopullulan	0.261732976	-1.187214371	4.118287609	-0.288278645	0.773133453	0.999551235
1.1.1.370	scyllo-inositol 2-dehydrogenase (NAD+)	0.193874331	-1.246208472	4.121717821	-0.30235172	0.762383955	0.999551235
3.2.1.1	alpha-amylase	516.5780335	0.349166201	0.433423708	0.805600143	0.420473443	0.999551235
5.1.3.20	ADP-L-glycero-D-manno-heptose 6-epimerase	13.58974453	0.315347427	0.512210908	0.615659335	0.538119381	0.999551235
2.-.-.-	#N/A	311.2391915	-0.457646707	0.270681265	-1.690721766	0.090889955	0.999551235
3.2.1.96	mannosyl-glycoprotein endo-beta-N-acetylglucosaminidase	29.7488792	-0.523101167	1.010954173	-0.517433116	0.604853848	0.999551235
5.1.3.5	UDP-arabinose 4-epimerase	0.840902598	-0.167755821	4.082225136	-0.041094211	0.96722079	0.999551235
2.7.1.200	PTS system, galactitol-specific IIA component	14.49931809	-1.273360405	0.796371652	-1.598952451	0.109831168	0.999551235
3.6.1.-	#N/A	427.5886094	-0.577520379	0.38006525	-1.519529554	0.128629255	0.999551235
2.7.1.90	diphosphate-dependent phosphofructokinase	118.196786	-0.20433394	0.494938038	-0.412847517	0.679718335	0.999551235
5.3.1.27	6-phospho-3-hexuloisomerase	17.12641049	-1.084534107	0.883240355	-1.227903707	0.219483118	0.999551235
3.2.1.82	exo-poly-alpha-galacturonosidase	14.97042183	-0.878801702	1.293692786	-0.679297057	0.496949661	0.999551235
5.3.1.4	L-arabinose isomerase	323.6098938	-0.310149888	0.296717235	-1.045270892	0.295897787	0.999551235
2.3.1.40	acyl-[acyl-carrier-protein]-phospholipid O-acyltransferase / long	12.07765451	0.160967071	0.85104944	0.189139507	0.849983478	0.999551235
2.4.1.282	3-O-alpha-D-glucosyl-L-rhamnose phosphorylase	0.89434512	-1.224431535	4.026104498	-0.304123138	0.76103408	0.999551235
2.1.1.113	site-specific DNA-methyltransferase (cytosine-N4-specific)	0.436862061	6.157130293	4.070071223	1.512781953	0.130335054	0.999551235
2.7.7.9	UTP--glucose-1-phosphate uridylyltransferase	181.7226964	-0.185265339	0.351271341	-0.527413761	0.597906289	0.999551235
3.1.2.21	medium-chain acyl-[acyl-carrier-protein] hydrolase	1.936026993	-0.603311176	1.671027555	-0.361042027	0.71806803	0.999551235
0.0.0.0	#N/A	2720696.047	0.521103272	0.838364384	0.621571338	0.534223772	0.999551235
2.7.7.89	[glutamine synthetase] adenylyltransferase / [glutamine synthe	8.353306205	0.466818171	0.973861345	0.479347674	0.631691312	0.999551235
4.2.1.129	squalene-hopene/tetraprenyl-beta-curcumene cyclase	6.587769184	0.496204963	1.374422927	0.361027856	0.718078623	0.999551235
4.1.1.18	lysine decarboxylase	7.01613773	1.071116152	1.123680484	0.953221282	0.340477966	0.999551235
5.1.3.2	UDP-glucose 4-epimerase	831.185721	-0.104615829	0.282719588	-0.370033891	0.711357238	0.999551235
5.4.99.61	precorrin-8X/cobalt-precorrin-8 methylmutase	99.4358351	0.541584838	0.298428304	1.814790457	0.069556118	0.999551235
3.1.26.5	ribonuclease P/MRP protein subunit POP1	320.5620884	-0.333128679	0.34132125	-0.975997477	0.329065715	0.999551235
6.3.2.45	UDP-N-acetylmuramate: L-alanyl-gamma-D-glutamyl-meso-diar	13.23918897	0.867772015	0.700049118	1.239587327	0.21512807	0.999551235
2.1.1.171	16S rRNA (guanine966-N2)-methyltransferase	16.15378857	-0.684689378	0.784740565	-0.872504122	0.38293342	0.999551235
2.5.1.7	UDP-N-acetylglucosamine 1-carboxyvinyltransferase	756.9220733	-0.185493067	0.137929578	-1.34483893	0.178677255	0.999551235
2.3.1.31	homoserine O-acetyltransferase/O-succinyltransferase	20.97808603	0.144139724	0.723432693	0.199244139	0.842071774	0.999551235
2.7.1.181	O-antigen chain-terminating methyltransferase	0.321384486	-0.391894419	4.143731151	-0.094575252	0.924652208	0.999551235
4.1.1.98	4-hydroxy-3-polyprenylbenzoate decarboxylase	42.84468263	0.20345497	0.421175327	0.483064788	0.629049735	0.999551235
2.1.1.144	trans-aconitate 2-methyltransferase	4.543822609	0.348787978	1.064635617	0.327612539	0.74320464	0.999551235
1.1.1.261	glycerol-1-phosphate dehydrogenase [NAD(P)+]	117.8868652	-0.374957943	0.364127831	-1.029742609	0.303130848	0.999551235
3.2.2.30	adenosylhomocysteine/aminodeoxyfutalosine nucleosidase	0.072907776	-0.391886654	4.143731151	-0.094573378	0.924653696	0.999551235
1.21.98.1	cyclic dehypoxanthinyl futalosine synthase	8.600599234	1.009511598	0.76881107	1.313081507	0.189155485	0.999551235
1.6.5.3	#N/A	1987.932707	-0.357308022	0.414749284	-0.861503649	0.388960712	0.999551235
1.3.1.78	arogenate dehydrogenase (NADP+)	0.124335172	-0.95125443	4.140981039	-0.229717166	0.818311555	0.999551235
6.3.5.7	aspartyl-tRNA(Asn)/glutamyl-tRNA(Gln) amidotransferase subu	513.5161994	-0.271112066	0.277289018	-0.97772378	0.328210957	0.999551235
2.6.1.22	4-aminobutyrate aminotransferase / (S)-3-amino-2-methylprop	21.40539401	0.277701566	0.588117678	0.472187075	0.636793264	0.999551235
3.1.1.75	poly(3-hydroxybutyrate) depolymerase	0.153183881	-0.469733734	4.140729394	-0.113442268	0.909679931	0.999551235
2.1.1.151	precorrin-2/cobalt-factor-2 C20-methyltransferase	67.37692647	0.080800529	0.515384222	0.156777266	0.875420391	0.999551235
6.3.4.23	5-formaminoimidazole-4-carboxamide-1-(beta)-D-ribofuranosy	19.3794077	-1.060210742	1.556236457	-0.681265843	0.495703292	0.999551235
6.3.4.18	5-(carboxyamino)imidazole ribonucleotide synthase	20.71168242	-0.189043391	0.76371735	-0.24753057	0.804497636	0.999551235
3.2.1.85	6-phospho-beta-galactosidase	2.516302815	-0.584853951	1.404952475	-0.416280238	0.677204951	0.999551235
2.8.1.13	tRNA-uridine 2-sulfurtransferase	432.6097358	-0.02684243	0.224498587	-0.119566164	0.904826824	0.999551235
4.2.3.3	methylglyoxal synthase	416.339376	-0.29779157	0.270034007	-1.102792841	0.270117138	0.999551235
4.3.1.17	L-serine dehydratase	414.1108953	-0.016653181	0.185157963	-0.089940397	0.928334579	0.999551235
2.3.1.86	fatty acid synthase subunit alpha, fungi type	0.01747704	-0.715913286	4.141706174	-0.172854678	0.862765657	0.999551235
6.3.3.2	glutamate formiminotransferase / 5-formyltetrahydrofolate cyc	206.2840431	-0.179462349	0.277486506	-0.646742617	0.517798536	0.999551235
5.3.3.8	3-hydroxyacyl-CoA dehydrogenase / enoyl-CoA hydratase / 3-h	0.306876842	-1.446634924	4.109619134	-0.35201192	0.724829323	0.999551235
3.5.3.1	arginase	30.489841	0.559525099	0.447743426	1.249655643	0.211425367	0.999551235
3.4.21.68	tissue plasminogen activator	0.059587395	-0.391885395	4.143731151	-0.094573075	0.924653937	0.999551235
4.1.3.27	anthranilate synthase / indole-3-glycerol phosphate synthase	257.4550298	0.065968355	0.405962411	0.162498679	0.870913169	0.999551235
6.1.1.17	glutamyl-tRNA synthetase	500.0146009	-0.07641541	0.15467988	-0.494022947	0.621289974	0.999551235
6.2.1.17	propionyl-CoA synthetase	0.186031077	-1.371644349	4.113904889	-0.333416641	0.738819803	0.999551235
3.4.23.43	leader peptidase (prepilin peptidase) / N-methyltransferase	211.7759288	-0.248960445	0.438094808	-0.568279834	0.56984497	0.999551235
3.5.1.6	beta-ureidopropionase	61.32383993	0.13311851	0.457850739	0.290746522	0.771245189	0.999551235
2.1.1.197	malonyl-CoA O-methyltransferase	27.782404	0.005212661	0.606301175	0.008597478	0.993140289	0.999551235
4.1.99.19	2-iminoacetate synthase	227.5228568	0.214688664	0.27263666	0.787453397	0.431016499	0.999551235
3.6.1.27	phosphatidylglycerophosphatase B	477.1536917	0.195070003	0.229520889	0.849900868	0.395380203	0.999551235
3.5.3.12	agmatine deiminase	81.72245933	-0.202298801	0.342516469	-0.590625036	0.554771686	0.999551235
2.8.3.17	cinnamoyl-CoA:phenyllactate CoA-transferase	1.036084739	0.251478889	2.65666126	0.094659749	0.92458509	0.999551235
1.7.1.15	nitrite reductase (NADH) large subunit	2.283221944	-0.649972398	2.084366649	-0.311832085	0.755168139	0.999551235
4.1.3.17	4-hydroxy-4-methyl-2-oxoglutarate aldolase	0.768386619	0.272553219	3.574088847	0.076258098	0.939213761	0.999551235

2.7.7.67	CDP-2,3-bis-(O-geranylgeranyl)-sn-glycerol synthase	1.61179286	-1.185438115	2.57478942	-0.460401968	0.645227723	0.999551235
2.5.1.1	farnesyl diphosphate synthase	408.1672705	0.077012632	0.201309254	0.382558827	0.702046903	0.999551235
1.14.13.127	3-(3-hydroxy-phenyl)propionate hydroxylase	0.384912947	-0.111088541	4.107542529	-0.027045013	0.978423832	0.999551235
5.3.1.23	methylthioribose-1-phosphate isomerase	98.34085415	-0.201104626	0.275953185	-0.728763562	0.466146304	0.999551235
6.3.4.24	tyramine---L-glutamate ligase	2.341597874	-0.920079088	3.108887813	-0.295951203	0.767267347	0.999551235
1.2.1.41	glutamate-5-semialdehyde dehydrogenase	367.9436807	-0.198815473	0.163013133	-1.219628566	0.222605713	0.999551235
4.2.1.44	inosose dehydratase	34.21941793	-0.375225705	0.398384809	-0.941867503	0.34626048	0.999551235
3.5.4.13	dCTP deaminase	6.58030804	-1.186252987	1.095895358	-1.082450964	0.279052193	0.999551235
2.1.1.228	tRNA (guanine37-N1)-methyltransferase	306.555681	-0.293448633	0.371377192	-0.790163315	0.429432397	0.999551235
1.1.1.17	mannitol-1-phosphate 5-dehydrogenase	23.22468614	-0.421389622	0.58749266	-0.717267893	0.473208814	0.999551235
1.2.1.16	succinate-semialdehyde dehydrogenase / glutarate-semialdehy	8.439430765	-0.526302603	1.107885556	-0.475051416	0.634750326	0.999551235
3.1.4.46	glycerophosphoryl diester phosphodiesterase	340.9449883	0.44124445	0.234669581	1.880279701	0.060069968	0.999551235
1.1.1.88	hydroxymethylglutaryl-CoA reductase	1.877515391	-0.014858852	2.446250302	-0.006074134	0.995153572	0.999551235
3.5.4.19	phosphoribosyl-AMP cyclohydrolase	246.2107743	-0.131790119	0.231094747	-0.57028609	0.568483674	0.999551235
4.1.1.83	4-hydroxyphenylacetate decarboxylase large subunit	33.23974627	-0.077747592	0.936323699	-0.08303495	0.933823749	0.999551235
1.2.1.12	glyceraldehyde 3-phosphate dehydrogenase	2889.827608	-0.513081747	0.568746293	-0.902127634	0.366989071	0.999551235
3.6.1.21	ADP-sugar diphosphatase	0.107128162	-0.391888715	4.143731151	-0.094573876	0.924653301	0.999551235
6.5.1.3	RNA-editing ligase	36.42033204	-0.003747683	0.615344471	-0.006090382	0.995140608	0.999551235
1.3.8.7	acyl-CoA dehydrogenase	0.861133089	0.596547931	3.057123019	0.195133767	0.845288229	0.999551235
3.4.24.78	spore protease	139.5273193	0.745880705	0.583684409	1.277883551	0.201290489	0.999551235
3.1.3.2	acid phosphatase	13.24700897	0.368112237	0.706537242	0.521008965	0.602360526	0.999551235
3.2.2.20	DNA-3-methyladenine glycosylase I	32.62903189	0.462376956	0.502423961	0.920292406	0.357419976	0.999551235
3.5.4.25	GTP cyclohydrolase II	303.6077484	0.364830943	0.246526916	1.479882799	0.138904527	0.999551235
2.4.1.16	chitin synthase	0.299092876	-1.43073229	4.140575192	-0.345539502	0.729688818	0.999551235
3.4.22.40	bleomycin hydrolase	186.1108036	0.052823995	0.282911866	0.186715375	0.851883801	0.999551235
2.7.8.13	phospho-N-acetylmuramoyl-pentapeptide-transferase	372.9500552	0.066271797	0.224790301	0.29481609	0.768134375	0.999551235
1.1.3.48	3-deoxy-alpha-D-manno-octulosonate 8-oxidase	3.333179177	-0.001633286	1.472544726	-0.001109159	0.999115019	0.999551235
1.1.1.49	glucose-6-phosphate 1-dehydrogenase	223.5508127	0.163067637	0.470068099	0.346902156	0.728664827	0.999551235
5.1.3.15	glucose-6-phosphate 1-epimerase	2.900916458	-0.649963414	1.381539926	-0.470462997	0.638024265	0.999551235
2.7.4.23	ribose 1,5-bisphosphokinase	0.173198025	1.742097071	4.09276188	0.425653171	0.67036059	0.999551235
3.1.3.4	phosphatidate phosphatase	1.343840583	2.129284038	4.091509259	0.520415305	0.602774145	0.999551235
5.2.1.-	#N/A	1.891810036	0.734290658	1.731077751	0.424181212	0.671433662	0.999551235
4.2.1.53	oleate hydratase	635.5203128	-0.331144572	0.555494777	-0.596125446	0.551091426	0.999551235
5.1.1.1	alanine racemase	456.0159821	-0.041712279	0.221190214	-0.188581033	0.850421199	0.999551235
5.4.99.24	23S rRNA pseudouridine955/2504/2580 synthase	208.4356302	-0.255984166	0.375287146	-0.682102142	0.495174366	0.999551235
1.1.1.95	D-3-phosphoglycerate dehydrogenase / 2-oxoglutarate reducta	733.0911774	-0.045888727	0.270799329	-0.169456575	0.865437527	0.999551235
3.4.21.26	prolyl oligopeptidase	54.99110323	0.413522621	0.418655116	0.987740517	0.323279746	0.999551235
4.2.1.159	dTDP-4-dehydro-6-deoxy-alpha-D-glucopyranose 2,3-dehydrata	6.32428671	-1.187056543	1.314815282	-0.902831416	0.366615375	0.999551235
3.4.24.84	STE24 endopeptidase	4.521324759	0.266722328	1.401886888	0.190259521	0.849105772	0.999551235
2.7.8.41	cardiolipin synthase (CMP-forming)	51.9006002	-0.508985529	0.529176416	-0.961844696	0.336127622	0.999551235
2.7.8.15	UDP-N-acetylglucosamine--dolichyl-phosphate N-acetylglucosa	2.302647437	-1.113869077	2.507481164	-0.444218323	0.656884746	0.999551235
3.1.3.45	3-deoxy-D-manno-octulosonate 8-phosphate phosphatase (KD(	63.9288138	0.677823989	0.339087352	1.998965711	0.045612064	0.999551235
2.4.1.212	hyaluronan synthase	0.302144354	0.566738126	4.102896176	0.138131237	0.890136699	0.999551235
2.4.2.44	5'-methylthioinosine phosphorylase	0.115969639	-1.1233092	4.143261082	-0.271117165	0.78630092	0.999551235
3.1.4.55	phosphoribosyl 1,2-cyclic phosphate phosphodiesterase	94.63300165	0.506825955	0.342100506	1.481511855	0.138470228	0.999551235
1.10.3.14	#N/A	311.3418992	0.294360448	0.261759312	1.124546232	0.260781369	0.999551235
6.3.4.13	phosphoribosylamine---glycine ligase	540.7811406	0.043491297	0.211531799	0.205601698	0.837102051	0.999551235
2.7.1.22	HTH-type transcriptional regulator, transcriptional repressor of	2.053440714	2.791020139	1.809601886	1.542339319	0.122991159	0.999551235
1.1.1.1	alcohol dehydrogenase	622.5140963	-0.174338116	0.442382235	-0.394089324	0.693515093	0.999551235
4.2.1.120	4-hydroxybutyryl-CoA dehydratase / vinylacetyl-CoA-Delta-ison	61.11807847	0.206319415	0.404311613	0.510298018	0.609842691	0.999551235
5.3.1.1	triosephosphate isomerase (TIM)	914.7994511	-0.161262585	0.449365581	-0.358867239	0.719694409	0.999551235
1.2.1.58	phenylglyoxylate dehydrogenase alpha subunit	1.250377898	-0.497237465	2.860798363	-0.173810735	0.862014208	0.999551235
2.7.1.78	bifunctional polynucleotide phosphatase/kinase	0.228120967	-0.404137747	4.143165283	-0.097543236	0.922295001	0.999551235
4.4.1.19	phosphosulfolactate synthase	2.261025615	-1.610800397	3.501435662	-0.460039982	0.645487523	0.999551235
3.4.19.12	ubiquitin carboxyl-terminal hydrolase MINDY-1/2	2.089260676	-0.670582917	3.402672317	-0.197075373	0.843768555	0.999551235
1.6.5.5	NADPH2:quinone reductase	0.160692243	-0.391890765	4.143731151	-0.09457437	0.924652908	0.999551235
5.4.99.-	#N/A	11.3310429	-2.429913402	2.88186081	-0.843175143	0.399130498	0.999551235
6.1.1.10	methionyl-tRNA synthetase	1072.529682	-0.365448877	0.375866157	-0.972284602	0.330908982	0.999551235
6.2.1.1	acetyl-CoA synthetase	231.4799934	0.046528787	0.37149647	0.125246915	0.900328077	0.999551235
3.2.1.35	hyaluronoglucosaminidase	245.6207223	-0.72360739	0.449134394	-1.611115513	0.107154548	0.999551235
1.3.1.31	2-enoate reductase	51.06122705	-1.171960183	0.675983227	-1.733711926	0.082969209	0.999551235
2.7.7.85	diadenylate cyclase	239.3174528	0.091987476	0.223979004	0.41069687	0.681294822	0.999551235
2.5.1.55	2-dehydro-3-deoxyphosphooctonate aldolase (KDO 8-P synthas	74.66958636	0.304880069	0.362686321	0.840616397	0.40056287	0.999551235
3.1.12.1	CRISPR-associated exonuclease Cas4	89.17767433	0.274483928	0.352452083	0.778783673	0.436107157	0.999551235
3.6.1.45	UDP-sugar diphosphatase	148.4862257	0.135360798	0.445497897	0.303841609	0.761248565	0.999551235
1.5.5.1	electron-transferring-flavoprotein dehydrogenase	0.060694436	-0.324536578	4.126379248	-0.078649237	0.937311624	0.999551235
2.7.4.-	#N/A	277.2844787	-0.235829041	0.170438164	-1.383663351	0.16646156	0.999551235
6.3.2.5	phosphopantothenoylcysteine decarboxylase / phosphopantot	280.6301022	-0.018945626	0.148901943	-0.127235588	0.898753939	0.999551235
4.2.1.1	carbonic anhydrase	85.65558449	0.362642606	0.402708132	0.900509768	0.36784903	0.999551235
4.2.1.119	(3R)-3-hydroxyacyl-CoA dehydrogenase / 3a,7a,12a-trihydroxy-	0.498402437	-0.181041076	4.103194178	-0.044121986	0.964807168	0.999551235
2.7.11.-	#N/A	276.9316338	-0.239499404	0.16858844	-1.420615811	0.155428479	0.999551235
2.7.7.15	choline-phosphate cytidylyltransferase	6.623524635	0.52518258	0.727968728	0.721435633	0.47064153	0.999551235
4.1.2.53	2-dehydro-3-deoxy-L-rhamnonate aldolase	0.443198042	-1.820362817	4.113571984	-0.442526064	0.658108574	0.999551235
2.3.1.191	UDP-3-O-[3-hydroxymyristoyl] glucosamine N-acyltransferase	125.04161	0.434148271	0.301558418	1.439682148	0.149957346	0.999551235
3.1.31.1	micrococcal nuclease	51.00902493	-2.245488241	1.042640438	-2.15365543	0.031267204	0.999551235
3.4.21.53	ATP-dependent Lon protease	915.8824061	-0.113152056	0.140691932	-0.804254048	0.42125027	0.999551235
2.7.1.63	polyphosphate glucokinase	3.198360875	-0.195650443	2.299434194	-0.085086342	0.932192749	0.999551235
4.1.1.50	S-adenosylmethionine decarboxylase	23.5955198	0.260297956	0.648714681	0.401251834	0.688234721	0.999551235
2.4.1.187	N-acetylglucosaminyldiphosphoundecaprenol N-acetyl-beta-D-(	105.0075222	0.468552869	0.186483721	2.512567135	0.01198563	0.999551235
6.3.1.20	lipoate---protein ligase	88.43111534	0.046402058	0.447215929	0.103757615	0.917361704	0.999551235
2.7.7.38	3-deoxy-manno-octulosonate cytidylyltransferase (CMP-KDO sy	95.53006735	0.237592545	0.310322135	0.765631962	0.443895321	0.999551235
2.4.1.279	nigerose phosphorylase	0.207493739	-0.982230577	4.113109417	-0.238804874	0.811256891	0.999551235
1.3.99.16	isoquinoline 1-oxidoreductase	0.043850487	0.733944445	4.133023226	0.177580528	0.859052418	0.999551235
3.1.1.85	pimeloyl-[acyl-carrier protein] methyl ester esterase	24.44366084	0.37407465	0.620523913	0.602836801	0.546617262	0.999551235
4.1.1.33	diphosphomevalonate decarboxylase	2.229319486	-1.989889623	1.984649888	-1.002640131	0.316034526	0.999551235
6.3.2.-	#N/A	11.16411353	0.406183536	0.966845403	0.420112186	0.674403501	0.999551235
2.4.1.-	#N/A	1001.881925	-0.105678255	0.138029056	-0.765623256	0.443900503	0.999551235

4.2.3.5	chorismate synthase	338.384486	0.160194113	0.132156239	1.212157022	0.225452279	0.999551235
1.12.2.1	NiFe] hydrogenase large subunit	22.82601474	0.387957465	0.985421703	0.393696895	0.693804834	0.999551235
3.7.1.22	3D-(3,5/4)-trihydroxycyclohexane-1,2-dione acylhydrolase (dec	50.44257656	-0.425677306	0.398736536	-1.067565342	0.285716624	0.999551235
3.5.4.1	cytosine/creatinine deaminase	15.08932727	0.474006062	0.561351506	0.844401516	0.398445076	0.999551235
4.1.3.38	4-amino-4-deoxychorismate lyase	35.10463143	0.408831726	0.473094116	0.864165739	0.38749685	0.999551235
6.4.1.2	acetyl-CoA carboxylase / biotin carboxylase 2	637.2201999	0.156323443	0.213617536	0.731791247	0.464295993	0.999551235
6.5.1.2	DNA ligase (NAD+)	524.6015592	-0.002712207	0.146448709	-0.018519841	0.985224149	0.999551235
1.3.3.3	coproporphyrinogen III oxidase	0.900952424	-2.759157949	4.091946103	-0.674289905	0.500127021	0.999551235
5.1.99.1	methylmalonyl-CoA/ethylmalonyl-CoA epimerase	68.30107652	0.204203389	0.464685951	0.439443862	0.660339951	0.999551235
1.7.99.1	hydroxylamine reductase	325.2855543	0.344834253	0.461199919	0.74768932	0.454647575	0.999551235
5.3.1.22	hydroxypyruvate isomerase	18.70282022	0.600938545	0.572413979	1.049832057	0.293795334	0.999551235
4.2.1.-	#N/A	0.467580383	-1.144125379	4.10608336	-0.278641537	0.780519933	0.999551235
2.1.1.56	mRNA (guanine-N7-)-methyltransferase	0.053564081	-0.391885504	4.143731151	-0.094573101	0.924653917	0.999551235
6.2.1.22	[citrate (pro-3S)-lyase] ligase	34.07579315	0.558985022	0.501206694	1.115278445	0.264731119	0.999551235
1.17.2.1	nicotinate dehydrogenase subunit A	0.015832449	0.284417857	4.139099958	0.068714904	0.945216554	0.999551235
5.3.2.1	phenylpyruvate tautomerase	0.107128162	-0.391888715	4.143731151	-0.094573876	0.924653301	0.999551235
1.1.1.8	glycerol-3-phosphate dehydrogenase (NAD+)	1.167095298	0.053230015	3.973934248	0.01339479	0.989312823	0.999551235
2.7.1.53	L-xylulokinase	12.11164162	-1.156089233	0.789070466	-1.465127999	0.142885999	0.999551235
2.7.7.77	molybdenum cofactor guanylyltransferase	15.50953688	-0.193089035	0.704452111	-0.274098171	0.784009179	0.999551235
1.1.1.87	homoisocitrate dehydrogenase	1.266185865	-1.607574086	2.762669694	-0.581891527	0.560639748	0.999551235
6.3.2.31	coenzyme F420-0:L-glutamate ligase / coenzyme F420-1:gamm	4.270530574	-2.918403008	1.940261394	-1.504128782	0.132548211	0.999551235
3.2.1.31	beta-glucuronidase	100.6708401	-0.007596769	0.356877341	-0.021286779	0.98301689	0.999551235
3.1.21.7	deoxyribonuclease V	2.012999821	0.200801692	3.753900904	0.053491474	0.957340324	0.999551235
3.2.-.-	#N/A	42.87598427	0.071326428	0.475123818	0.150121769	0.880668545	0.999551235
2.3.1.22	2-acylglycerol O-acyltransferase 3	0.160692243	-0.391890765	4.143731151	-0.09457437	0.924652908	0.999551235
2.4.1.8	maltose phosphorylase	30.76090562	0.244353196	0.382229891	0.639283326	0.522638634	0.999551235
3.4.11.1	leucyl aminopeptidase	47.78279693	0.072469678	0.443129148	0.163540761	0.870092682	0.999551235
2.3.2.26	other hect domain ubiquitin protein ligase E3	0.373766343	-1.247592759	4.13615447	-0.301631085	0.762933309	0.999551235
2.5.1.74	1,4-dihydroxy-2-naphthoate polyprenyltransferase	209.2981939	-0.268340684	0.647721254	-0.414284203	0.678665981	0.999551235
1.1.1.156	glycerol 2-dehydrogenase (NADP+)	0.034954079	-0.907659863	4.140131853	-0.219234531	0.826467358	0.999551235
3.5.2.14	#N/A	21.35431366	0.233768117	0.834679213	0.280069413	0.779424251	0.999551235
2.3.1.28	chloramphenicol O-acetyltransferase type B	86.94404786	0.502129272	0.459464559	1.092857462	0.274456388	0.999551235
2.1.1.174	23S rRNA (guanine1835-N2)-methyltransferase	0.096307101	-1.192781373	4.131830079	-0.288681129	0.772825405	0.999551235
5.1.1.13	aspartate racemase	40.36829995	0.746338017	0.327272219	2.280480818	0.022579187	0.999551235
3.5.2.6	beta-lactamase class C	160.3792134	0.088439397	0.245035741	0.360924476	0.718155906	0.999551235
2.7.1.24	dephospho-CoA kinase	224.4460225	0.345103021	0.218991123	1.575876759	0.115054208	0.999551235
3.6.3.55	#N/A	1.408673102	0.337309097	2.048909181	0.164628623	0.869236301	0.999551235
3.13.1.1	UDP-sulfoquinovose synthase	0.129569077	-1.287960173	4.137956904	-0.311255096	0.755606701	0.999551235
2.1.1.185	23S rRNA (guanosine2251-2'-O)-methyltransferase	315.2882071	0.03981105	0.160844692	0.247512364	0.804511724	0.999551235
3.1.3.25	myo-inositol-1(or 4)-monophosphatase	79.05547234	0.296478193	0.284048862	1.043757722	0.296597497	0.999551235
2.7.7.71	D-glycero-alpha-D-manno-heptose 1-phosphate guanylyltransfe	1.859383022	2.390160351	2.005271037	1.191938799	0.233285244	0.999551235
3.4.25.1	#N/A	17.62314595	-2.775430501	1.466733638	-1.892252573	0.058457337	0.999551235
3.6.3.9	sodium/potassium-transporting ATPase subunit alpha	0.299485303	0.487834253	4.122400719	0.118337417	0.905800311	0.999551235
1.5.1.20	methylenetetrahydrofolate reductase (NADPH)	397.8327279	-0.166029452	0.247852185	-0.669872861	0.502938842	0.999551235
1.13.11.4	gentisate 1,2-dioxygenase	0.144460652	-1.306718627	4.119786615	-0.317181143	0.751106158	0.999551235
1.2.1.9	glyceraldehyde-3-phosphate dehydrogenase (NADP+)	6.716637653	-0.830778209	1.186617013	-0.700123292	0.483850311	0.999551235
1.3.98.1	dihydroorotate dehydrogenase (fumarate)	120.1794457	0.275999801	0.410250873	0.672758596	0.501100883	0.999551235
3.1.3.100	ribosome biogenesis GTPase / thiamine phosphate phosphatas	416.5264494	-0.567305237	0.38424035	-1.47643327	0.139827617	0.999551235
2.1.1.176	16S rRNA (cytosine967-C5)-methyltransferase	301.1690219	-0.300857249	0.209362601	-1.437015247	0.150713661	0.999551235
3.5.1.2	glutaminase	86.19117576	0.534945562	0.412650685	1.296364167	0.194850051	0.999551235
5.5.1.2	3-carboxy-cis,cis-muconate cycloisomerase	0.510612935	-0.524481104	4.139292472	-0.126707911	0.899171584	0.999551235
1.8.1.9	thioredoxin reductase (NADPH)	1244.533356	-0.444087694	0.441778759	-1.00522645	0.314787821	0.999551235
2.5.1.134	cystathionine beta-synthase (O-acetyl-L-serine)	0.594219345	-2.036232676	4.122000957	-0.493991316	0.621312313	0.999551235
5.1.3.29	L-fucose mutarotase	72.80484909	-1.263675395	0.732664289	-1.724767282	0.08456942	0.999551235
1.1.1.276	serine 3-dehydrogenase (NADP+)	0.034954079	-0.907659863	4.140131853	-0.219234531	0.826467358	0.999551235
3.2.1.41	pullulanase	79.05972656	-0.389557472	0.405771097	-0.960042436	0.337033858	0.999551235
2.7.1.100	5-methylthioribose kinase	27.51768582	-0.563743401	0.448020038	-1.258299525	0.208283453	0.999551235
2.7.1.83	pseudouridine kinase	78.72070777	0.060323685	0.447799826	0.134711274	0.892840159	0.999551235
2.3.1.187	malonate decarboxylase alpha subunit	10.47559418	1.400369611	0.75896543	1.845103288	0.065022515	0.999551235
2.7.11.22	CTD kinase subunit alpha	0.552718707	-0.9586486	4.105445848	-0.233506575	0.815368064	0.999551235
5.3.3.17	trans-2,3-dihydro-3-hydroxyanthranilate isomerase	0.021925243	0.329456595	4.143731151	0.079507232	0.936629179	0.999551235
2.5.1.46	deoxyhypusine synthase	8.030721428	0.259173355	1.158070818	0.223797501	0.822914875	0.999551235
2.8.3.21	L-carnitine CoA-transferase	9.109101761	0.306235343	1.664968454	0.183928616	0.854069455	0.999551235
6.3.2.10	UDP-N-acetylmuramoyl-tripeptide--D-alanyl-D-alanine ligase	403.0847592	-0.115460246	0.153475694	-0.752303135	0.451868783	0.999551235
2.7.1.50	hydroxyethylthiazole kinase	131.5764821	0.067969308	0.381708319	0.17806609	0.858671073	0.999551235
3.4.22.16	cathepsin H	0.484790941	4.813366459	4.080322059	1.179653564	0.23813803	0.999551235
2.1.1.258	5-methyltetrahydrofolate corrinoid/iron sulfur protein methyltr	273.0062463	-0.698945521	0.689787909	-1.013275981	0.310928357	0.999551235
1.7.-.-	#N/A	11.71383892	0.383681306	0.956425403	0.401161769	0.688301026	0.999551235
4.2.2.21	chondroitin-sulfate-ABC endolyase/exolyase	72.26463302	-0.007482466	0.527221552	-0.014192261	0.988676594	0.999551235
1.1.1.76	meso-butanediol dehydrogenase / (S,S)-butanediol dehydroger	3.716547359	-1.934565453	1.451433783	-1.332865113	0.182576073	0.999551235
2.4.1.83	dolichol-phosphate mannosyltransferase	119.1710587	0.203380483	0.275102493	0.739289855	0.459731008	0.999551235
2.7.1.147	ADP-dependent phosphofructokinase/glucokinase	7.609187489	-1.906178209	1.062666774	-1.793768523	0.072850123	0.999551235
4.2.2.5	chondroitin AC lyase	45.73435572	0.601892198	0.507303698	1.186453402	0.235443293	0.999551235
2.1.2.5	glutamate formiminotransferase / 5-formyltetrahydrofolate cyc	154.6551231	0.640772569	0.389138269	1.646644957	0.099631044	0.999551235
1.3.1.44	enoyl-acyl-carrier protein] reductase / trans-2-enoyl-CoA reduc	27.94422685	0.099508061	0.505203316	0.196966366	0.843853858	0.999551235
1.5.1.41	aquacobalamin reductase / NAD(P)H-flavin reductase	1.180331694	-2.57768995	4.096909634	-0.629179108	0.529231805	0.999551235
2.7.1.205	PTS system, cellobiose-specific IIA component	67.85843679	-0.279328013	0.576258579	-0.484726861	0.62787011	0.999551235
3.2.1.20	alpha-glucosidase	601.8969372	0.09119506	0.277364932	0.328790877	0.742313759	0.999551235
1.13.12.16	nitronate monooxygenase	3.734228943	-0.299916462	1.549966033	-0.193498732	0.846568399	0.999551235
2.4.1.261	alpha-1,2-mannosyltransferase	0.214256324	-0.391892261	4.143731151	-0.094574732	0.924652621	0.999551235
2.1.1.191	23S rRNA (cytosine1962-C5)-methyltransferase	466.4840486	-0.00839811	0.222655102	-0.037718021	0.969912508	0.999551235
2.4.2.22	xanthine phosphoribosyltransferase	282.3440183	-0.020134597	0.508160343	-0.039622528	0.968394067	0.999551235
4.6.1.-	#N/A	2.805481591	-2.47540308	2.661917614	-0.929932267	0.352406155	0.999551235
6.1.1.15	prolyl-tRNA synthetase	530.8683727	-0.142739685	0.148890501	-0.958688999	0.337715442	0.999551235
4.4.1.-	#N/A	0.404940627	1.019992221	4.085714888	0.249648409	0.802859259	0.999551235
2.3.2.6	leucyl/phenylalanyl-tRNA---protein transferase	20.29869155	1.039270691	0.62169356	1.671676782	0.094588075	0.999551235
1.1.1.47	glucose 1-dehydrogenase	6.850818888	-0.225108741	0.859992716	-0.261756567	0.793509126	0.999551235

3.1.6.1	arylsulfatase	55.45735393	0.510355759	0.442294581	1.153882008	0.248548546	0.999551235
6.2.1.45	ubiquitin-activating enzyme E1	4.37738286	-1.076011587	2.096688624	-0.5131957	0.607814433	0.999551235
2.7.1.148	4-diphosphocytidyl-2-C-methyl-D-erythritol kinase	307.9305914	-0.124696027	0.279276176	-0.446497185	0.655238146	0.999551235
1.2.1.3	aldehyde dehydrogenase (NAD+)	65.51788066	-0.082620389	0.356255751	-0.231913138	0.81660548	0.999551235
3.5.1.81	N-acyl-D-amino-acid deacylase	40.77082716	0.406299234	0.40899707	0.993403777	0.320513222	0.999551235
5.4.99.12	#N/A	337.9885111	0.003828551	0.208635913	0.018350393	0.985359326	0.999551235
2.3.1.16	acetyl-CoA acyltransferase	0.591306247	-1.869732693	4.124345185	-0.453340496	0.650303577	0.999551235
4.2.1.126	N-acetylmuramic acid 6-phosphate etherase	82.6211455	0.328875425	0.351744894	0.934982798	0.349797164	0.999551235
3.5.3.6	arginine deiminase	100.4938897	-0.329505634	0.43524793	-0.757052731	0.449018272	0.999551235
2.7.7.56	ribonuclease PH	33.41871616	-0.000957436	0.531074432	-0.001802828	0.998561552	0.999551235
2.4.1.166	raffinose-raffinose alpha-galactotransferase	0.509876242	-1.766565542	4.131293699	-0.427605896	0.668938073	0.999551235
2.7.8.39	archaetidylinositol phosphate synthase	0.621380821	-0.6821832	4.106430062	-0.16612561	0.8680581	0.999551235
3.1.27.-	#N/A	0.616679938	0.778235444	4.121803504	0.188809448	0.850242167	0.999551235
6.3.4.3	methylenetetrahydrofolate dehydrogenase (NADP+) / metheny	919.4527816	-0.578320448	0.273885348	-2.11154212	0.034725742	0.999551235
5.3.2.8	4-oxalomesaconate tautomerase	0.029793698	-0.391883699	4.143731151	-0.094572665	0.924654262	0.999551235
1.1.3.9	galactose oxidase	0.092938899	-1.252383687	4.139487465	-0.302545592	0.762236183	0.999551235
2.7.7.59	[protein-PII] uridylyltransferase	6.613776947	0.581413209	1.068418366	0.544181219	0.586316775	0.999551235
3.6.1.54	UDP-2,3-diacylglucosamine hydrolase	80.87134064	0.655881529	0.301547438	2.175052569	0.029626177	0.999551235
3.4.11.19	D-aminopeptidase	79.30120568	0.237747376	0.362125421	0.656533242	0.511481087	0.999551235
2.7.1.199	PTS system, glucose-specific IIB component	43.92933022	-0.563289252	0.541180529	-1.040852768	0.297943885	0.999551235
2.7.7.41	phosphatidate cytidylyltransferase	329.3149528	0.132197969	0.155691405	0.849102549	0.395824232	0.999551235
2.1.1.74	methylenetetrahydrofolate--tRNA-(uracil-5-)-methyltransferase	148.1849758	0.168476427	0.248863126	0.676984293	0.49841592	0.999551235
2.7.6.2	thiamine pyrophosphokinase	166.8556101	-0.044983752	0.354501047	-0.126893143	0.899024974	0.999551235
2.1.1.17	phosphatidylethanolamine/phosphatidyl-N-methylethanolamir	0.04941368	-0.391882467	4.143731151	-0.094572368	0.924654499	0.999551235
4.3.1.18	D-serine dehydratase	41.51184634	-0.101348091	0.310812747	-0.326074434	0.74436804	0.999551235
2.5.1.108	2-(3-amino-3-carboxypropyl)histidine synthase	2.518104667	-1.63693561	3.450964314	-0.474341506	0.635256398	0.999551235
2.4.1.1	glycogen phosphorylase	1122.779221	-0.139741128	0.118046224	-1.183783129	0.236498922	0.999551235
4.2.1.30	#N/A	5.746841115	-4.196277901	1.458784324	-2.876558125	0.004020381	0.999551235
2.7.7.65	two-component system, cell cycle response regulator	4.101623422	-0.432298059	1.879889481	-0.229959295	0.8181234	0.999551235
1.3.5.4	succinate dehydrogenase / fumarate reductase, flavoprotein su	913.8675227	-0.147295778	0.29688364	-0.49613976	0.619795809	0.999551235
3.6.3.4	Cu2+-exporting ATPase	642.8453761	-0.343944952	0.329831768	-1.042789036	0.29704601	0.999551235
1.1.5.6	#N/A	0.860990484	-2.51143974	4.100874586	-0.612415641	0.540262796	0.999551235
2.4.1.52	poly(glycerol-phosphate) alpha-glucosyltransferase	0.161648	0.187943742	4.126562851	0.045544863	0.963673016	0.999551235
4.1.1.31	phosphoenolpyruvate carboxylase	18.28372225	-0.834980984	0.844188736	-0.98909278	0.32261775	0.999551235
4.1.2.22	xylulose-5-phosphate/fructose-6-phosphate phosphoketolase	141.7183535	-0.355809519	0.327749135	-1.085615432	0.27764917	0.999551235
3.6.3.25	sulfate transport system ATP-binding protein	43.02532111	0.576439971	0.522866695	1.102460678	0.270261444	0.999551235
3.6.1.15	#N/A	5.035962108	1.090802747	1.505673723	0.724461568	0.468782413	0.999551235
3.6.3.36	taurine transport system ATP-binding protein	0.734608246	0.116608371	4.070641992	0.028646187	0.977146776	0.999551235
3.2.1.73	licheninase	0.785591429	1.690533945	3.530840601	0.478790786	0.632087473	0.999551235
2.1.1.309	18S rRNA (guanine1575-N7)-methyltransferase	0.262409875	-0.723878206	4.131658692	-0.175202808	0.860920278	0.999551235
1.3.8.1	butyryl-CoA dehydrogenase	257.9585902	-1.113822436	0.569349537	-1.956306914	0.05042901	0.999551235
1.8.5.3	anaerobic dimethyl sulfoxide reductase subunit A	18.00441963	-0.92029699	0.807684692	-1.139426065	0.25452549	0.999551235
5.4.3.2	lysine 2,3-aminomutase	170.8248805	0.028067986	0.327934676	0.085590174	0.93179221	0.999551235
2.6.1.2	alanine transaminase	68.37319459	-0.957672072	0.532903561	-1.797083265	0.072322402	0.999551235
1.3.1.74	2-alkenal reductase	1.219066924	-1.747470644	3.814397072	-0.458124996	0.646862642	0.999551235
3.1.3.48	protein-tyrosine phosphatase	512.7382816	0.047155721	0.265706097	0.177473238	0.859136685	0.999551235
5.4.99.29	tRNA pseudouridine32 synthase / 23S rRNA pseudouridine746 s	73.12648985	0.279600489	0.366746292	0.762381228	0.445832507	0.999551235
3.1.21.4	type II restriction enzyme	23.82095201	0.818484371	0.627990849	1.303338054	0.192459375	0.999551235
2.3.1.97	glycylpeptide N-tetradecanoyltransferase	0.267820405	-0.391893442	4.143731151	-0.094575017	0.924652395	0.999551235
4.6.1.16	tRNA-intron endonuclease, archaea type	0.575809695	-0.542466964	4.138910161	-0.13106517	0.895723754	0.999551235
1.18.1.3	3-phenylpropionate/trans-cinnamate dioxygenase ferredoxin re	0.132218179	-0.420009541	4.142488731	-0.10139063	0.919240374	0.999551235
3.1.6.6	choline-sulfatase	12.21331139	0.3167615	0.712676042	0.444467726	0.656704457	0.999551235
1.8.5.2	thiosulfate dehydrogenase [quinone] small subunit	14.79835306	-0.468952817	0.696256372	-0.673534687	0.500607189	0.999551235
6.3.1.8	glutathionylspermidine amidase/synthetase	1.535270693	-0.213388864	3.059306132	-0.069750739	0.944392056	0.999551235
2.3.1.9	acetyl-CoA C-acetyltransferase	972.9845713	-0.084571797	0.626825701	-0.134920755	0.892674529	0.999551235
1.1.1.28	D-lactate dehydrogenase	178.3211075	0.253507306	0.211175586	1.200457449	0.229961729	0.999551235
2.7.1.167	D-beta-D-heptose 7-phosphate kinase / D-beta-D-heptose 1-ph	16.49374106	-0.272558788	0.666665535	-0.408838876	0.682657908	0.999551235
4.3.2.2	adenylosuccinate lyase	625.3639552	-0.041757196	0.211425863	-0.197502781	0.843434106	0.999551235
3.2.1.139	alpha-glucuronidase	70.11148679	0.126874046	0.61216782	0.207253701	0.83581173	0.999551235
4.2.1.77	trans-L-3-hydroxyproline dehydratase	0.959760061	0.425284253	4.054784076	0.104884562	0.916467409	0.999551235
2.3.1.89	tetrahydrodipicolinate N-acetyltransferase	3.970360467	0.152805043	1.117447974	0.13674466	0.891232628	0.999551235
1.5.1.2	pyrroline-5-carboxylate reductase	309.2710635	-0.43698273	0.314845014	-1.38792965	0.165158468	0.999551235
1.7.2.5	nitric oxide reductase subunit B	0.671618783	1.238117308	4.106719476	0.301485727	0.763044132	0.999551235
2.4.1.11	glycogen synthase	62.34508969	0.666468707	0.489869623	1.360502214	0.173671053	0.999551235
2.8.1.6	biotin synthase	192.6670322	0.174948	0.291565723	0.600029378	0.548486657	0.999551235
2.7.1.71	shikimate kinase	321.2596761	-0.0251006	0.256377987	-0.09790466	0.922008	0.999551235
3.5.1.47	N-acetyldiaminopimelate deacetylase	2.679831447	0.36516417	1.967771194	0.185572475	0.852780041	0.999551235
1.2.5.1	pyruvate dehydrogenase (quinone)	24.97110696	0.021991951	0.484976109	0.045346463	0.963831153	0.999551235
4.99.1.1	protoporphyrin/coproporphyrin ferrochelatase	9.070073581	-0.620935264	0.99350641	-0.624993717	0.531975182	0.999551235
2.4.2.52	triphosphoribosyl-dephospho-CoA synthase	26.49897562	0.311892123	0.583519099	0.534501996	0.592994269	0.999551235
1.3.4.1	fumarate reductase (CoM/CoB) subunit A	11.21361449	-0.860841514	1.418743417	-0.606763354	0.544007971	0.999551235
6.2.1.5	succinyl-CoA synthetase alpha subunit	201.7652483	0.991708215	0.707946988	1.400822705	0.161267097	0.999551235
4.2.1.136	ADP-dependent NAD(P)H-hydrate dehydratase	296.924103	-0.048026821	0.178908754	-0.268443103	0.788358265	0.999551235
2.7.1.153	phosphatidylinositol-4,5-bisphosphate 3-kinase catalytic subuni	0.107128162	-0.391888715	4.143731151	-0.094573876	0.924653301	0.999551235
1.5.1.39	FMN reductase [NAD(P)H]	18.48410048	0.898817191	0.892536596	1.007036792	0.313917094	0.999551235
4.1.99.14	spore photoproduct lyase	9.608842198	0.059281677	0.691456234	0.085734533	0.93167745	0.999551235
1.1.1.281	GDP-4-dehydro-6-deoxy-D-mannose reductase	14.17185845	0.353825903	0.623086405	0.567860092	0.570129972	0.999551235
6.3.1.10	adenosylcobinamide-phosphate synthase	146.8807414	0.187098129	0.286443459	0.653176476	0.513642514	0.999551235
5.4.2.7	phosphopentomutase	271.5281824	-0.239480708	0.266986337	-0.896977393	0.36973098	0.999551235
2.7.1.190	aminoglycoside 2''-phosphotransferase	1.1071359	0.351878972	3.055038321	0.115179888	0.908302539	0.999551235
1.12.98.2	5,10-methenyltetrahydromethanopterin hydrogenase	33.13934147	-2.021564391	1.178091818	-1.715965055	0.086168438	0.999551235
2.7.1.36	mevalonate kinase	7.588783217	-0.512414024	1.627194654	-0.314906408	0.752832716	0.999551235
4.2.1.91	cyclohexadienyl dehydratase	1.859733319	0.087401919	2.038189284	0.042882141	0.965795485	0.999551235
3.5.4.39	GTP cyclohydrolase IV	4.206096909	-1.456893559	1.708606546	-0.852679373	0.393837132	0.999551235
5.3.1.8	mannose-6-phosphate isomerase	279.7365829	0.274157404	0.271673764	1.009141995	0.31290654	0.999551235
3.1.3.71	2-phosphosulfolactate phosphatase	9.214936184	0.184702416	0.82990046	0.222559722	0.823878187	0.999551235
2.7.11.33	[pyruvate, water dikinase]-phosphate phosphotransferase / [py	11.94070215	-0.84862954	0.771207296	-1.100390965	0.271161813	0.999551235

2.7.1.19	phosphoribulokinase	0.240767753	-1.429598065	4.109365218	-0.34788781	0.727924441	0.999551235
1.2.1.24	succinate-semialdehyde dehydrogenase	0.096307101	-1.192781373	4.131830079	-0.288681129	0.772825405	0.999551235
2.7.1.168	D-glycero-alpha-D-manno-heptose-7-phosphate kinase	31.71658101	0.013099819	0.54927085	0.023849471	0.980972679	0.999551235
4.1.3.42	2-dehydro-3-deoxyphosphogluconate aldolase / (4S)-4-hydroxy	365.4288842	-0.155513458	0.280231996	-0.554945403	0.578932005	0.999551235
1.3.99.31	phytoene desaturase	1.632348624	-0.923071895	4.11939824	-0.224079305	0.822695595	0.999551235
3.1.3.15	imidazoleglycerol-phosphate dehydratase / histidinol-phosphat	920.6179347	-1.021392137	0.664252463	-1.53765653	0.124132618	0.999551235
4.3.1.14	3-aminobutyryl-CoA ammonia-lyase	70.2832787	-0.730569496	0.671793022	-1.087491938	0.27681946	0.999551235
1.8.2.2	thiosulfate dehydrogenase	0.06577573	1.018164182	4.122326352	0.246987767	0.804917691	0.999551235
2.3.1.54	formate C-acetyltransferase	2143.677149	-0.272353601	0.446433121	-0.610065849	0.541818188	0.999551235
3.5.4.29	GTP cyclohydrolase IIa	5.074632583	-1.080771062	2.327186516	-0.464411019	0.642353297	0.999551235
5.3.4.1	protein disulfide-isomerase	8.650518897	0.365589883	1.35610993	0.269587203	0.787477847	0.999551235
2.4.1.117	dolichyl-phosphate beta-glucosyltransferase	0.15199015	-0.824927547	4.132191868	-0.199634376	0.84176654	0.999551235
4.1.3.40	chorismate--pyruvate lyase	0.298604074	-0.380772468	4.11055224	-0.092632923	0.926195188	0.999551235
3.1.11.1	exodeoxyribonuclease I	5.573815682	0.33867335	1.326002039	0.255409373	0.798406932	0.999551235
2.5.1.43	nicotianamine synthase	0.050191057	-0.752539914	4.143731151	-0.181609252	0.855889388	0.999551235
3.1.3.87	2-hydroxy-3-keto-5-methylthiopentenyl-1-phosphate phosphat	0.051061294	-0.40886677	4.142956969	-0.098689601	0.921384726	0.999551235
2.3.1.235	minimal PKS ketosynthase (KS/KS alpha)	0.046441742	-0.721762791	4.142085491	-0.174251061	0.861668159	0.999551235
3.4.21.50	lysyl endopeptidase	0.022430994	-0.752542723	4.143731151	-0.18160993	0.855888856	0.999551235
4.4.1.24	(2R)-sulfolactate sulfo-lyase subunit alpha	10.38634163	0.174008341	1.079116556	0.161250738	0.871895921	0.999551235
3.4.14.4	dipeptidyl-peptidase III	183.6513868	-0.096745337	0.38826719	-0.249172063	0.803227688	0.999551235
3.4.21.105	rhomboid-related protein 1/2/3	57.76563614	-0.300995814	0.405741622	-0.741841108	0.458183611	0.999551235
3.1.6.-	#N/A	5.647174252	-1.377606995	1.016137666	-1.355728699	0.175185505	0.999551235
6.1.1.9	valyl-tRNA synthetase	675.7895904	-0.09929122	0.190679471	-0.520723175	0.602559627	0.999551235
2.7.4.25	CMP/dCMP kinase	386.0131937	-0.267884806	0.259001272	-1.034299191	0.300996315	0.999551235
1.2.1.59	glyceraldehyde-3-phosphate dehydrogenase (NAD(P))	6.878986232	-1.487296918	1.817776574	-0.818195668	0.413245468	0.999551235
3.5.2.9	5-oxoprolinase (ATP-hydrolysing)	0.116409678	-0.424740052	4.142298814	-0.10253728	0.918330224	0.999551235
1.1.1.25	shikimate dehydrogenase	261.1601688	0.051003523	0.252533861	0.201967065	0.839942472	0.999551235
4.2.1.59	3-hydroxyacyl-[acyl-carrier protein] dehydratase / trans-2-dece	276.4956797	0.059054153	0.232012226	0.254530351	0.799085859	0.999551235
2.3.1.182	(R)-citramalate synthase	90.27086847	0.109307037	0.244633671	0.446819263	0.655005562	0.999551235
4.1.1.65	phosphatidylserine decarboxylase	135.2245112	-0.073886476	0.343428513	-0.215143684	0.829655296	0.999551235
3.4.21.92	ATP-dependent Clp protease, protease subunit	561.0943661	-0.310870936	0.234169705	-1.327545488	0.184328303	0.999551235
2.7.7.61	holo-ACP synthase	27.06562961	0.262353784	0.697624153	0.376067518	0.706866702	0.999551235
3.3.2.1	bifunctional isochorismate lyase / aryl carrier protein	0.250956274	-1.327537732	4.124210724	-0.321888919	0.747536848	0.999551235
1.2.1.71	succinylglutamic semialdehyde dehydrogenase	0.048153551	-0.752542723	4.143731151	-0.18160993	0.855888856	0.999551235
2.7.9.2	pyruvate, water dikinase	67.74853073	-0.590916441	0.515228776	-1.146901081	0.251422506	0.999551235
1.2.1.10	acetaldehyde dehydrogenase (acetylating)	400.4918272	-0.652559916	0.466920262	-1.397583206	0.162238264	0.999551235
1.3.1.43	cyclohexadieny/prephenate dehydrogenase	3.773900336	-1.547092088	1.869976874	-0.827332204	0.408048796	0.999551235
4.1.3.3	N-acetylneuraminate lyase	294.7456095	-0.455421182	0.432578036	-1.052806994	0.292429459	0.999551235
5.4.99.1	methylaspartate mutase sigma subunit	67.78198055	-0.161449433	0.609674031	-0.264812711	0.791153744	0.999551235
2.3.3.14	homocitrate synthase	6.772603954	0.643706442	1.177753492	0.546554475	0.584684853	0.999551235
6.3.4.14	acetyl-CoA carboxylase / biotin carboxylase 2	281.1120721	0.321786821	0.192578088	1.670942028	0.094733129	0.999551235
1.-.-.-	#N/A	90.00714768	0.040688194	0.405364291	0.100374392	0.920047099	0.999551235
1.16.1.1	mercuric reductase	0.505665624	0.428824235	4.069164456	0.105383855	0.916071227	0.999551235
1.16.3.1	bacterioferritin	74.72166482	0.4637073	0.490501383	0.945374093	0.344467924	0.999551235
4.1.2.19	rhamnulose-1-phosphate aldolase	135.5495071	-0.167687635	0.425156618	-0.394413795	0.693275561	0.999551235
2.5.1.129	flavin prenyltransferase	25.08112497	0.208617805	0.560304062	0.372329632	0.709647439	0.999551235
5.1.99.7	D-erythro-7,8-dihydroneopterin triphosphate epimerase	0.144460652	-1.306718627	4.119786615	-0.317181143	0.751106158	0.999551235
3.4.17.11	glutamate carboxypeptidase	21.84682526	-0.134728597	0.560370852	-0.240427561	0.809998814	0.999551235
2.3.1.243	lauroyl-Kdo2-lipid IVA myristoyltransferase	0.273484821	-1.77475361	4.132321097	-0.429481052	0.66757318	0.999551235
4.4.1.15	D-cysteine desulfhydrase	28.02213594	0.426542615	0.35907038	1.187908107	0.234869615	0.999551235
2.7.8.42	KDO II ethanolaminephosphotransferase	0.429078891	0.112078329	4.090440497	0.027400064	0.978140648	0.999551235
2.6.1.59	dTDP-4-amino-4,6-dideoxygalactose transaminase	72.97848554	-0.54042503	0.313878786	-1.721763477	0.085112373	0.999551235
1.4.1.14	glutamate synthase (NADH)	1092.906852	-0.20990984	0.126932996	-1.653705869	0.09818729	0.999551235
3.1.3.7	3'(2'), 5'-bisphosphate nucleotidase	529.7510404	-0.086992565	0.298156201	-0.291768425	0.770463688	0.999551235
2.4.1.315	processive 1,2-diacylglycerol beta-glucosyltransferase	113.4372334	-0.522899464	0.362418143	-1.442807082	0.149074824	0.999551235
4.2.1.41	5-dehydro-4-deoxyglucarate dehydratase	0.227201514	-1.653879789	4.132521476	-0.400210815	0.689001249	0.999551235
3.2.1.84	#N/A	0.107128162	-0.391888715	4.143731151	-0.094573876	0.924653301	0.999551235
1.3.7.7	light-independent protochlorophyllide reductase subunit L	0.248906341	1.795106047	4.084484733	0.439493881	0.660303715	0.999551235
1.1.1.61	4-hydroxybutyrate dehydrogenase	46.40904916	-0.110316935	0.371568855	-0.296894999	0.766546674	0.999551235
3.6.3.44	#N/A	1.312530107	0.359489935	2.791266566	0.128790972	0.897523049	0.999551235
2.7.7.82	CMP-N,N'-diacetyllegionaminic acid synthase	8.972758846	-0.169535564	0.525682561	-0.322505588	0.747069705	0.999551235
3.2.1.65	levanase	13.77925283	0.202872151	0.857299954	0.236640805	0.812935462	0.999551235
2.7.4.1	polyphosphate kinase	532.0088662	-0.078452954	0.181346802	-0.432612837	0.665296062	0.999551235
3.1.-.-	#N/A	7215.830595	0.007949904	0.188350482	0.042208038	0.966332855	0.999551235
1.6.4.-	#N/A	210.9728632	-0.922068872	0.477395342	-1.93145762	0.053426485	0.999551235
3.5.1.18	succinyl-diaminopimelate desuccinylase	238.9037885	-0.174799257	0.182271722	-0.959003707	0.337556878	0.999551235
2.3.3.-	#N/A	4.148446599	-0.730128491	2.119832038	-0.34442752	0.730524796	0.999551235
3.1.3.41	4-nitrophenyl phosphatase	2.475652433	-0.175942811	1.819289489	-0.096709629	0.922956993	0.999551235
2.3.1.48	chromodomain protein Y	4.949563951	-1.602960354	1.45144717	-1.104387667	0.269425008	0.999551235
3.1.3.10	glucose-1-phosphatase	1.077587015	5.469374257	3.996390742	1.368578452	0.171131083	0.999551235
6.3.2.29	cyanophycin synthetase	4.102299911	-0.34430083	3.756698354	-0.091649847	0.926976246	0.999551235
2.4.2.18	anthranilate phosphoribosyltransferase	118.1962522	-0.137605643	0.43432446	-0.316826832	0.751375003	0.999551235
2.4.1.293	GalNAc5-diNAcBac-PP-undecaprenol beta-1,3-glucosyltransfera	0.450882135	0.76778753	4.102479458	0.187152072	0.851541402	0.999551235
3.2.1.23	beta-galactosidase	3131.489228	-0.193809162	0.138286817	-1.401501364	0.1610642	0.999551235
4.2.1.55	3-hydroxybutyryl-CoA dehydratase	14.441369	0.308419364	0.922969386	0.334159907	0.738258897	0.999551235
4.2.1.7	altronate hydrolase	427.3465533	-0.10626607	0.370960184	-0.286462199	0.774524142	0.999551235
3.5.1.88	peptide deformylase	403.7885556	0.073060751	0.24042938	0.303876136	0.761222259	0.999551235
2.1.1.177	23S rRNA (pseudouridine1915-N3)-methyltransferase	205.3404314	0.328787845	0.2368683	1.38806183	0.165118218	0.999551235
6.5.1.6	DNA ligase 1	16.24694701	-0.623330297	0.896738796	-0.695107984	0.486987631	0.999551235
2.6.1.42	branched-chain amino acid aminotransferase	427.0846235	0.040076293	0.278971013	0.143657552	0.885770892	0.999551235
2.6.1.16	glucosamine---fructose-6-phosphate aminotransferase (isomeri	625.9817941	-0.181841636	0.198790467	-0.914740221	0.360328024	0.999551235
5.3.1.13	arabinose 5-phosphate isomerase	81.00631304	0.290144264	0.300767485	0.964679622	0.334705305	0.999551235
5.4.99.21	23S rRNA pseudouridine2604 synthase	55.92207997	-0.065194	0.387131927	-0.16840254	0.86626661	0.999551235
3.4.24.25	vibriolysin	14.58659153	-2.005714442	2.716296506	-0.738400406	0.460271168	0.999551235
3.1.4.3	phospholipase C	2.840339435	0.527173965	2.631556958	0.200327781	0.841224237	0.999551235
4.1.1.73	tartrate dehydrogenase/decarboxylase / D-malate dehydrogen	0.660399619	0.135768846	4.076623528	0.033304239	0.973431973	0.999551235
2.4.2.-	#N/A	193.4053107	-0.268639449	0.223167865	-1.203755074	0.228684259	0.999551235

3.1.1.32	phospholipase A1/A2	6.442157471	0.306752472	1.03406478	0.296647249	0.766735834	0.999551235
1.14.13.1	salicylate hydroxylase	0.048153551	-0.752542723	4.143731151	-0.18160993	0.855888856	0.999551235
1.1.1.303	(R,R)-butanediol dehydrogenase / meso-butanediol dehydroger	5.627014574	-0.995365853	1.692248252	-0.588191391	0.556403835	0.999551235
3.8.1.3	haloacetate dehalogenase	2.266409738	-0.430744014	1.857765482	-0.231861351	0.816645704	0.999551235
1.1.1.2	alcohol dehydrogenase (NADP+)	2.742843588	-1.128080016	1.615293298	-0.698374727	0.484942878	0.999551235
3.4.14.11	X-Pro dipeptidyl-peptidase	20.93093077	-0.884888486	0.713142055	-1.240830603	0.214668333	0.999551235
1.5.1.3	dihydrofolate reductase	420.2366272	0.356147806	0.601259892	0.592335878	0.553625695	0.999551235
2.7.11.26	glycogen synthase kinase 3 beta	0.160692243	-0.391890765	4.143731151	-0.09457437	0.924652908	0.999551235
6.2.1.42	3-oxocholest-4-en-26-oate---CoA ligase	0.197654718	-0.391886964	4.143731151	-0.094573453	0.924653637	0.999551235
2.5.1.17	cob(I)alamin adenosyltransferase	297.5379903	-0.097540988	0.280932523	-0.347204327	0.72843782	0.999551235
2.1.1.62	#N/A	0.107128162	-0.391888715	4.143731151	-0.094573876	0.924653301	0.999551235
2.7.1.48	uridine kinase	1798.770566	-0.755805003	0.609927244	-1.239172394	0.215281662	0.999551235
2.5.1.30	heptaprenyl diphosphate synthase	102.0691133	0.074374933	0.440404916	0.168878526	0.865892191	0.999551235
2.7.7.4	bifunctional enzyme CysN/CysC	320.7491654	0.156502143	0.250847122	0.623894514	0.532696863	0.999551235
6.2.1.26	O-succinylbenzoic acid---CoA ligase	43.02822346	0.981778818	0.342425034	2.867135048	0.004142062	0.999551235
1.3.1.83	geranylgeranyl diphosphate/geranylgeranyl-bacteriochlorophyl	0.022430994	-0.752542723	4.143731151	-0.18160993	0.855888856	0.999551235
3.2.2.4	AMP nucleosidase	42.98149885	1.242081556	0.525637934	2.36299832	0.018127752	0.999551235
3.4.19.3	pyroglutamyl-peptidase	36.92515586	-0.338613463	0.386801762	-0.875418614	0.381346175	0.999551235
1.1.1.43	phosphogluconate 2-dehydrogenase	0.089723976	-1.20166047	4.130988035	-0.290889361	0.771135938	0.999551235
1.2.7.7	2-oxoisovalerate ferredoxin oxidoreductase, alpha subunit	19.38805552	-2.252238274	1.428689588	-1.576436403	0.114925265	0.999551235
4.1.3.34	citrate lyase subunit beta / citryl-CoA lyase	88.71502524	0.110084462	0.442000529	0.249059571	0.803314702	0.999551235
3.6.3.5	Cd2+/Zn2+-exporting ATPase	686.2195542	0.090186389	0.402488061	0.22407221	0.822701116	0.999551235
5.3.1.26	galactose-6-phosphate isomerase	2.630607547	1.367511321	1.920551627	0.712040906	0.476439446	0.999551235
5.1.3.4	#N/A	185.3708773	-0.62099951	0.289915577	-2.142001188	0.032193386	0.999551235
3.2.1.136	glucuronoarabinoxylan endo-1,4-beta-xylanase	13.2680149	0.206053088	0.57837497	0.356262112	0.721644281	0.999551235
5.1.3.35	2-epi-5-epi-valiolone 7-phosphate 2-epimerase	0.265720381	1.28575995	4.086023383	0.314672685	0.753010185	0.999551235
2.7.4.14	UMP-CMP kinase	0.642768973	-0.391898218	4.143731151	-0.094576169	0.924651479	0.999551235
2.7.8.33	UDP-GlcNAc:undecaprenyl-phosphate/decaprenyl-phosphate G	45.64451026	-0.166645229	0.587332355	-0.283732417	0.77661545	0.999551235
2.7.1.28	triose/dihydroxyacetone kinase / FAD-AMP lyase (cyclizing)	5.825853036	1.731675002	4.034528823	0.42921369	0.667767721	0.999551235
4.1.1.17	ornithine decarboxylase	21.20996489	0.351982892	0.814983825	0.43188942	0.665821785	0.999551235
5.4.2.12	2,3-bisphosphoglycerate-independent phosphoglycerate mutas	1248.632008	-0.268301434	0.343330279	-0.781467441	0.434527619	0.999551235
2.7.4.7	hydroxymethylpyrimidine/phosphomethylpyrimidine kinase / t	174.8667027	-0.048497553	0.27541998	-0.176085818	0.860226522	0.999551235
1.1.1.330	17beta-estradiol 17-dehydrogenase / very-long-chain 3-oxoacyl	0.374948568	-0.391895253	4.143731151	-0.094575454	0.924652048	0.999551235
2.1.1.43	histone-lysine N-methyltransferase ASH1L	0.160692243	-0.391890765	4.143731151	-0.09457437	0.924652908	0.999551235
2.7.4.29	Kdo2-lipid A phosphotransferase	0.202890383	1.517857209	4.107468154	0.369535965	0.711728271	0.999551235
2.6.1.83	LL-diaminopimelate aminotransferase	321.5540511	0.200175515	0.209365259	0.956106647	0.339018357	0.999551235
3.1.4.16	2',3'-cyclic-nucleotide 2'-phosphodiesterase / 3'-nucleotidase	234.785924	0.519197231	0.315115597	1.647640535	0.099426457	0.999551235
2.4.1.4	amylosucrase	278.8244437	0.130145323	0.468341125	0.277885747	0.781100065	0.999551235
4.6.1.1	adenylate cyclase	23.29535609	-0.106584513	0.574983593	-0.18536966	0.852939104	0.999551235
3.1.21.-	#N/A	822.8588976	-0.060735078	0.498121596	-0.121928216	0.902955869	0.999551235
3.2.1.2	beta-amylase	0.22353784	-0.391896335	4.143731151	-0.094575715	0.92465184	0.999551235
4.1.1.55	4,5-dihydroxyphthalate decarboxylase	0.043850487	0.733944445	4.133023226	0.177580528	0.859052418	0.999551235
2.5.1.15	dihydropteroate synthase	139.5745534	-0.058587852	0.217946516	-0.268817566	0.788070075	0.999551235
2.7.7.49	RNA-directed DNA polymerase	107.7899205	0.412453841	0.354845425	1.16234792	0.245094169	0.999551235
4.2.2.24	rhamnogalacturonan exolyase	3.284691663	-3.439220254	1.742589974	-1.973625641	0.048424326	0.999551235
2.6.1.55	taurine---2-oxoglutarate transaminase	47.87404651	-1.212162304	0.703871949	-1.722134695	0.085045121	0.999551235
1.8.4.10	phosphoadenosine phosphosulfate reductase	26.55279816	-0.88856255	0.684962026	-1.297243519	0.194547411	0.999551235
3.2.1.91	cellulose 1,4-beta-cellobiosidase	0.254639706	-1.691570318	4.128600401	-0.409720039	0.682011328	0.999551235
1.5.1.5	methylenetetrahydrofolate dehydrogenase (NADP+) / metheny	432.3435048	-0.179577689	0.235073275	-0.763922182	0.444913618	0.999551235
3.7.1.8	2,6-dioxo-6-phenylhexa-3-enoate hydrolase	0.247596681	-2.130755827	4.125609698	-0.51647053	0.605525817	0.999551235
2.8.4.1	#N/A	294.1217901	-3.193255654	1.237198236	-2.581037993	0.009850374	0.999551235
2.8.1.10	thiazole synthase	81.74543984	0.702199365	0.349185077	2.010966138	0.044329033	0.999551235
3.6.3.20	sn-glycerol 3-phosphate transport system ATP-binding protein	11.33154221	0.626751843	0.917528297	0.683087209	0.494551738	0.999551235
2.3.1.41	3-oxoacyl-[acyl-carrier-protein] synthase I	64.89992041	0.602114857	0.512669889	1.174468933	0.240207249	0.999551235
2.6.1.11	acetylornithine aminotransferase	668.5746803	-0.936873298	0.509047816	-1.840442625	0.06570328	0.999551235
1.3.5.2	dihydroorotate dehydrogenase	21.83162396	0.459803921	0.606980803	0.757526299	0.448734616	0.999551235
2.7.7.19	poly(A) polymerase	28.18380502	-0.073158704	0.625722341	-0.116918798	0.906924401	0.999551235
4.2.1.70	#N/A	46.01611001	-0.204587014	0.658892052	-0.310501566	0.756179569	0.999551235
1.2.1.20	succinate-semialdehyde dehydrogenase / glutarate-semialdehy	8.343123664	-0.488792026	1.105102719	-0.442304609	0.658268796	0.999551235
4.3.3.6	pyridoxal 5'-phosphate synthase pdxS subunit	136.5195282	-0.230772966	0.310067171	-0.744267655	0.456714565	0.999551235
1.1.1.373	4-hydroxybutyrate dehydrogenase / sulfolactaldehyde 3-reduct	0.167610981	0.851717774	4.107890607	0.207337014	0.835746669	0.999551235
2.1.1.226	23S rRNA (cytidine1920-2'-O)/16S rRNA (cytidine1409-2'-O)-me	278.8082903	-0.215824423	0.295255504	-0.730975107	0.464794358	0.999551235
5.4.99.16	maltose alpha-D-glucosyltransferase / alpha-amylase	86.28108937	0.349435328	0.495742102	0.704873213	0.480889142	0.999551235
3.1.3.21	glycerol-1-phosphatase	0.231939278	-1.393542934	4.123543997	-0.337947876	0.735402468	0.999551235
1.3.1.1	dihydropyrimidine dehydrogenase (NAD+) subunit PreT	55.39741083	-0.438317545	0.43769201	-1.001429167	0.316619369	0.999551235
4.1.2.25	7,8-dihydroneopterin aldolase/epimerase/oxygenase	88.0690056	-0.066080061	0.26282696	-0.251420405	0.801489094	0.999551235
2.3.1.168	2-oxoisovalerate dehydrogenase E2 component (dihydrolipoyl t	0.137221322	-0.088835922	4.111799819	-0.021605118	0.982762951	0.999551235
3.6.3.12	K+-transporting ATPase ATPase A chain	131.978621	0.278034066	0.579670761	0.479641349	0.631482439	0.999551235
3.1.1.1	carboxylesterase 1	6.423676539	0.081853438	1.033702757	0.079184696	0.936885717	0.999551235
2.3.1.128	#N/A	202.8758722	0.184915591	0.43253374	0.427517147	0.669002699	0.999551235
2.4.1.80	ceramide glucosyltransferase	0.130175812	-1.045973435	4.13916511	-0.252701549	0.800498851	0.999551235
2.5.1.120	aminodeoxyfutalosine synthase	14.44656408	0.471997737	0.681652707	0.692431397	0.488666454	0.999551235
6.3.3.3	dethiobiotin synthetase	42.7523479	0.494110169	0.492885454	1.002484786	0.316109511	0.999551235
3.6.1.11	exopolyphosphatase	219.8727794	-0.131887497	0.43127516	-0.305808239	0.759750655	0.999551235
2.1.1.37	DNA (cytosine-5)-methyltransferase 1	626.4275744	0.145352264	0.351154036	0.413927362	0.678927304	0.999551235
4.3.99.3	7-carboxy-7-deazaguanine synthase	27.63428654	0.433212024	0.527906085	0.820623283	0.411860881	0.999551235
3.2.2.23	#N/A	37.572242	0.89278485	0.647260771	1.379327915	0.167793674	0.999551235
3.4.23.52	archaeal preflagellin peptidase FlaK	0.111483345	0.413832616	4.115593187	0.100552362	0.919905815	0.999551235
3.2.1.64	levanbiose-producing levanase	1.029822388	-1.717574803	4.097147169	-0.419212377	0.675060928	0.999551235
4.1.99.2	tyrosine phenol-lyase	16.61240796	-0.793785045	1.548376246	-0.512656434	0.608191669	0.999551235
3.1.22.4	crossover junction endodeoxyribonuclease RuvC	151.494518	0.236514935	0.282888196	0.836072125	0.403114329	0.999551235
2.7.8.5	CDP-diacylglycerol---glycerol-3-phosphate 3-phosphatidyltransf	283.7050059	-0.032461922	0.217092613	-0.149530292	0.881135209	0.999551235
1.4.1.21	aspartate dehydrogenase	44.11897346	0.369855606	0.35497023	1.041934154	0.297442208	0.999551235
2.6.1.102	perosamine synthetase	3.986928839	-0.002974294	1.724445138	-0.001724783	0.998623823	0.999551235
3.2.1.177	alpha-D-xyloside xylohydrolase	556.3891845	0.197828679	0.219802299	0.90003007	0.368104249	0.999551235
1.3.1.9	enoyl-acyl-carrier protein] reductase I	337.7412044	-0.049842069	0.165919466	-0.300399167	0.763872699	0.999551235
2.7.1.12	gluconokinase	2.191401162	0.520994319	1.581049935	0.329524266	0.741759456	0.999551235

2.1.1.266	23S rRNA (adenine2030-N6)-methyltransferase	8.85099374	0.551041825	1.229187863	0.448297483	0.653938517	0.999551235
4.1.2.4	deoxyribose-phosphate aldolase	354.3068753	0.041601868	0.232726694	0.178758472	0.858127355	0.999551235
3.4.13.21	dipeptidase E	57.55391717	-0.156591036	0.435620783	-0.359466403	0.719246207	0.999551235
1.2.1.70	glutamyl-tRNA reductase	52.01114862	-0.138843072	0.360800669	-0.384819331	0.700371276	0.999551235
2.5.1.10	farnesyl diphosphate synthase	408.1672705	0.077012632	0.201309254	0.382558827	0.702046903	0.999551235
2.4.2.28	5'-methylthioadenosine phosphorylase	7.435936035	1.031241811	0.70642134	1.459811238	0.144341959	0.999551235
6.1.1.23	nondiscriminating aspartyl-tRNA synthetase	52.43350952	-0.471680496	0.538016846	-0.876702096	0.380648468	0.999551235
2.7.2.15	propionate kinase	0.163600083	-1.340446566	4.116621499	-0.325618123	0.744713299	0.999551235
2.7.1.3	ketohexokinase	0.044861988	-1.012275533	4.141284466	-0.244435161	0.806893813	0.999551235
6.1.1.20	#N/A	872.735898	-0.1280375	0.249894698	-0.512365811	0.608395014	0.999551235
1.8.99.2	#N/A	150.0364969	-0.022501047	0.397332049	-0.056630335	0.954839669	0.999551235
2.1.1.170	16S rRNA (guanine527-N7)-methyltransferase	224.0979338	0.019213407	0.256281251	0.074970006	0.940238577	0.999551235
2.4.1.230	kojibiose phosphorylase	6.165137258	-1.245705238	1.269384572	-0.981345816	0.326422237	0.999551235
1.2.7.-	#N/A	6148.42736	-0.320211986	0.52316628	-0.612065414	0.540494479	0.999551235
1.2.7.11	2-oxoglutarate/2-oxoacid ferredoxin oxidoreductase subunit al	1113.830126	0.093304061	0.345897765	0.26974462	0.787356731	0.999551235
4.1.2.48	threonine aldolase	240.4281868	0.606678893	0.225104972	2.695093263	0.007036893	0.999551235
2.1.1.319	type I protein arginine methyltransferase	0.353049384	0.477478753	4.121897504	0.11583955	0.907779705	0.999551235
1.8.2.3	sulfide dehydrogenase [flavocytochrome c] flavoprotein chain	3.046267745	0.05701913	2.409615979	0.023663161	0.981121291	0.999551235
2.3.1.109	arginine N-succinyltransferase	0.096307101	-1.192781373	4.131830079	-0.288681129	0.772825405	0.999551235
2.3.2.2	gamma-glutamyltranspeptidase / glutathione hydrolase	29.91949374	1.295081215	0.549452001	2.357041585	0.018421184	0.999551235
3.1.2.12	S-formylglutathione hydrolase	1.707218919	0.337728235	2.878386463	0.117332484	0.906596584	0.999551235
3.4.15.5	peptidyl-dipeptidase Dcp	258.6791263	0.355077896	0.258664956	1.372732902	0.169835397	0.999551235
2.7.14.1	protein arginine kinase	100.4784369	-0.07934951	0.518947954	-0.152904562	0.878473538	0.999551235
2.1.1.196	cobalt-precorrin-6B (C15)-methyltransferase	4.720469386	0.917066045	1.138871054	0.805241332	0.420680429	0.999551235
5.4.2.3	phosphoacetylglucosamine mutase	0.208845794	-0.738274385	4.133880041	-0.178591149	0.858258745	0.999551235
3.5.1.116	ureidoglycolate amidohydrolase	0.579608174	1.168826233	4.082661493	0.286290263	0.774655815	0.999551235
2.8.3.25	bile acid CoA-transferase	19.80063959	-1.108080468	0.69276161	-1.599511941	0.109706898	0.999551235
6.5.1.7	DNA ligase 1	16.24694701	-0.623330297	0.896738796	-0.695107984	0.486987631	0.999551235
3.1.1.4	secretory phospholipase A2	6.442157471	0.306752472	1.03406478	0.296647249	0.766735834	0.999551235
2.1.1.227	23S rRNA (cytidine1920-2'-O)/16S rRNA (cytidine1409-2'-O)-me	278.8082903	-0.215824423	0.295255504	-0.730975107	0.464794358	0.999551235
1.5.3.-	#N/A	0.818610359	-1.663045	4.094515257	-0.406164074	0.684622049	0.999551235
1.4.5.1	D-amino-acid dehydrogenase	5.40917928	-3.35510327	2.484705454	-1.350302212	0.176919063	0.999551235
1.17.1.4	xanthine dehydrogenase molybdenum-binding subunit	289.3415095	-0.546378531	0.396173295	-1.379140232	0.167851522	0.999551235
3.4.11.10	bacterial leucyl aminopeptidase	5.455731727	-0.368913607	1.349692948	-0.273331507	0.784598398	0.999551235
5.3.1.14	L-rhamnose isomerase	112.3655337	0.153108038	0.277189751	0.552358223	0.580702948	0.999551235
3.4.22.41	cathepsin F	2.347625857	-0.858037882	4.095754477	-0.209494462	0.834062258	0.999551235
1.1.1.267	1-deoxy-D-xylulose-5-phosphate reductoisomerase	366.5098855	-0.066230642	0.142057124	-0.466225419	0.641054156	0.999551235
2.3.1.79	maltose O-acetyltransferase	148.2896748	0.325123959	0.249702778	1.302043821	0.192901406	0.999551235
2.3.1.82	aminoglycoside 6'-N-acetyltransferase	5.843599705	-0.339303408	1.472480349	-0.230429838	0.817757778	0.999551235
3.1.4.52	c-di-GMP phosphodiesterase	50.01167139	-1.036185382	0.819921413	-1.263761826	0.206315527	0.999551235
2.5.1.97	pseudaminic acid synthase	27.63825338	-0.916283782	0.48802617	-1.877530014	0.060445488	0.999551235
2.8.2.22	arylsulfate sulfotransferase	15.39709927	0.006421261	0.594281882	0.010805076	0.991378964	0.999551235
3.5.4.26	diaminohydroxyphosphoribosylaminopyrimidine deaminase	165.0856682	0.369858284	0.31743605	1.165142663	0.24396125	0.999551235
2.7.6.3	2-amino-4-hydroxy-6-hydroxymethyldihydropteridine diphosph	143.2206681	0.193117978	0.210691716	0.916590274	0.359357383	0.999551235
2.3.1.118	N-hydroxyarylamine O-acetyltransferase	9.67675098	-1.016332509	1.299551852	-0.782063839	0.434177057	0.999551235
5.1.3.22	L-ribulose-5-phosphate 3-epimerase	5.033868584	-0.643905147	1.457382697	-0.441822967	0.658617317	0.999551235
2.3.2.16	peptidoglycan pentaglycine glycine transferase (the first glycine	0.052431119	-1.040555473	4.139270063	-0.251386224	0.801515517	0.999551235
5.99.1.2	#N/A	1730.88601	0.063925998	0.37511908	0.170415214	0.864683608	0.999551235
2.1.1.90	methanol---5-hydroxybenzimidazolylcobamide Co-methyltrans	10.03781092	-3.35721537	1.70776138	-1.965857414	0.049315084	0.999551235
3.4.19.11	g-D-glutamyl-meso-diaminopimelate peptidase	66.39931612	-0.494762247	0.489499814	-1.010750634	0.312135796	0.999551235
4.2.1.22	cystathionine beta-synthase	0.640036229	0.823034948	4.05325792	0.203055163	0.839091916	0.999551235
2.4.99.18	dolichyl-diphosphooligosaccharide---protein glycosyltransferase	15.30748849	-0.237023644	1.182254391	-0.200484469	0.841101704	0.999551235
2.6.1.110	dTDP-4-dehydro-2,3,6-trideoxy-D-glucose 4-aminotransferase	0.598486539	-0.44180224	4.078671943	-0.108320121	0.913741762	0.999551235
2.7.1.201	PTS system, trehalose-specific IIA component	10.57361883	-0.0816029	0.778086229	-0.104876423	0.916473868	0.999551235
1.1.1.57	fructuronate reductase	269.3151576	-0.159721809	0.311362067	-0.512977738	0.607966893	0.999551235
3.5.1.-	#N/A	1932.988082	-1.585286519	0.895275575	-1.770724637	0.076606503	0.999551235
1.7.1.7	GMP reductase	3.492030991	-0.249060307	1.167659785	-0.213298694	0.831093992	0.999551235
2.7.1.23	NAD+ kinase	358.9369047	-0.085445465	0.251593116	-0.339617658	0.734144479	0.999551235
1.4.1.1	alanine dehydrogenase	179.0955202	0.000182724	0.322764968	0.000566122	0.9995483	0.999551235
2.4.1.15	trehalose 6-phosphate synthase	28.28681294	0.392381471	0.450007918	0.871943482	0.383239211	0.999551235
2.7.7.-	#N/A	701.1796075	-0.204349868	0.201522276	-1.014031164	0.310567882	0.999551235
2.4.99.15	3-deoxy-D-manno-octulosonic-acid transferase	102.8745696	0.408892164	0.322930505	1.26619244	0.205444193	0.999551235
1.17.7.3	(E)-4-hydroxy-3-methylbut-2-enyl-diphosphate synthase	384.5079502	0.125856052	0.171851425	0.732353846	0.463952622	0.999551235
5.4.99.25	tRNA pseudouridine55 synthase	364.7005709	-0.218355915	0.236421723	-0.923586515	0.355701637	0.999551235
2.7.1.163	hygromycin-B 4-O-kinase	0.732726104	0.95187975	4.092326053	0.232601151	0.816071134	0.999551235
2.7.1.76	deoxyadenosine kinase	0.485024588	1.168746869	4.091703715	0.285638196	0.775155245	0.999551235
3.6.1.9	ectonucleotide pyrophosphatase/phosphodiesterase family me	9.120822582	0.180145124	1.139453133	0.158097879	0.874379672	0.999551235
1.13.11.16	2,3-dihydroxyphenylpropionate 1,2-dioxygenase	0.620913234	-3.513869614	4.103607272	-0.856287988	0.391838504	0.999551235
2.5.1.101	N,N'-diacetyllegionaminate synthase	17.20160115	0.229258161	0.452759056	0.506357979	0.612605368	0.999551235
2.7.7.76	molybdenum cofactor cytidylyltransferase	2517.858965	2.346327246	1.284303424	1.826925945	0.067710905	0.999551235
3.2.2.27	uracil-DNA glycosylase	169.0721726	0.014249388	0.229704288	0.062033618	0.95053606	0.999551235
1.1.1.308	sulfopropanediol 3-dehydrogenase	0.04941368	-0.391882467	4.143731151	-0.094572368	0.924654499	0.999551235
2.3.3.1	citrate synthase	736.3133749	-0.438966403	0.199341485	-2.202082534	0.027659479	0.999551235
3.1.26.11	ribonuclease Z	239.7056231	-0.009142988	0.343600213	-0.026609378	0.978771293	0.999551235
1.2.1.38	N-acetyl-gamma-glutamyl-phosphate reductase	221.1370935	-0.021886378	0.314943969	-0.069492928	0.944597262	0.999551235
2.3.1.245	3-hydroxy-5-phosphonooxypentane-2,4-dione thiolase	50.11152848	-0.59018224	0.7652485	-0.771229529	0.440570894	0.999551235
3.1.25.-	#N/A	0.161565365	-0.540998316	4.136706255	-0.130779969	0.895949369	0.999551235
4.1.2.52	4-hydroxy-2-oxoheptanedioate aldolase	10.54245838	0.496864964	0.781652139	0.635659955	0.524998082	0.999551235
3.4.17.4	Gly-Xaa carboxypeptidase	0.224781176	-0.131756913	4.139593251	-0.031828468	0.974608844	0.999551235
2.7.1.105	6-phosphofructo-2-kinase	0.267820405	-0.391893442	4.143731151	-0.094575017	0.924652395	0.999551235
5.1.2.3	3-hydroxyacyl-CoA dehydrogenase / enoyl-CoA hydratase / 3-h	0.670887678	-1.883324493	4.088855725	-0.4605994	0.645086042	0.999551235
2.5.1.49	O-acetylhomoserine (thiol)-lyase	620.572906	-0.230132644	0.246945852	-0.931915404	0.351380246	0.999551235
4.2.1.2	#N/A	786.4071845	-0.026469337	0.209366268	-0.126425987	0.899394733	0.999551235
3.11.1.1	phosphonoacetaldehyde hydrolase	23.83046873	0.493428073	0.70963346	0.695328083	0.486849719	0.999551235
1.8.98.1	#N/A	514.6685068	-0.501154174	0.494773222	-1.01289672	0.311109495	0.999551235
3.4.11.4	tripeptide aminopeptidase	365.2843562	0.207663097	0.176269246	1.178101693	0.238756067	0.999551235
1.5.3.19	4-methylaminobutanoate oxidase (formaldehyde-forming)	0.655665518	0.305701577	4.074166998	0.07503413	0.940187556	0.999551235

2.7.1.193	PTS system, N-acetylglucosamine-specific IIA component	629.4791252	-0.547232764	0.488327155	-1.120627345	0.26244652	0.999551235
2.7.1.95	kanamycin kinase	6.400739295	-0.120637742	1.382929172	-0.087233493	0.930485917	0.999551235
4.1.1.22	histidine decarboxylase	0.451163486	0.226180032	4.113238473	0.05498831	0.956147777	0.999551235
4.1.1.35	UDP-glucuronate decarboxylase	71.42773928	0.621336948	0.295285946	2.104187332	0.035362109	0.999551235
1.3.8.-	#N/A	0.089381093	-0.391886445	4.143731151	-0.094573328	0.924653736	0.999551235
4.2.1.12	phosphogluconate dehydratase	0.541914096	-3.243412382	4.106462266	-0.789831288	0.429626304	0.999551235
3.4.11.9	Xaa-Pro aminopeptidase	757.7307781	0.110362025	0.234896368	0.46983283	0.638474457	0.999551235
4.1.2.-	#N/A	1.095079884	-2.398271658	2.760813178	-0.868683067	0.385020506	0.999551235
3.2.2.-	#N/A	321.9782873	0.246730548	0.429704776	0.57418619	0.565841813	0.999551235
1.1.1.133	dTDP-4-dehydrorhamnose reductase	412.266012	-0.052002346	0.186654612	-0.27860199	0.780550286	0.999551235
2.1.1.152	precorrin-6A synthase	0.021925243	0.329456595	4.143731151	0.079507232	0.936629179	0.999551235
2.6.99.2	pyridoxine 5-phosphate synthase	45.90984814	0.186789863	0.464728522	0.401933289	0.687733123	0.999551235
1.2.1.79	succinate-semialdehyde dehydrogenase / glutarate-semialdehy	8.343123664	-0.488792026	1.105102719	-0.442304609	0.658268796	0.999551235
1.2.7.4	#N/A	735.1758215	0.600723222	0.640959345	0.937225156	0.348642752	0.999551235
1.20.4.3	mycoredoxin	0.089723976	-1.20166047	4.130988035	-0.290889361	0.771135938	0.999551235
2.5.1.75	tRNA dimethylallyltransferase	316.1405562	0.148083587	0.221690979	0.667972993	0.504150832	0.999551235
4.2.3.1	threonine synthase	417.3788552	-0.354213839	0.171290322	-2.067915071	0.03864801	0.999551235
2.7.11.21	polo-like kinase 1	0.56438504	-2.526868139	4.118226816	-0.613581585	0.539491855	0.999551235
1.3.1.14	dihydroorotate dehydrogenase (NAD+) catalytic subunit	341.9876951	0.071644897	0.187908231	0.381275991	0.702998467	0.999551235
1.1.1.290	erythronate-4-phosphate dehydrogenase	70.05388707	0.332667567	0.474914767	0.700478464	0.48362855	0.999551235
1.2.1.31	L-aminoadipate-semialdehyde dehydrogenase	0.034954079	-0.907659863	4.140131853	-0.219234531	0.826467358	0.999551235
4.2.1.79	2-methylcitrate dehydratase	0.582300256	-0.797902566	3.902002227	-0.20448542	0.837974184	0.999551235
2.3.1.51	1-acyl-sn-glycerol-3-phosphate acyltransferase	528.0808745	-0.106911916	0.226662886	-0.471678085	0.637156582	0.999551235
3.5.2.3	dihydroorotase	396.1747044	-0.036582349	0.138779638	-0.263600264	0.792087955	0.999551235
2.1.1.250	trimethylamine---corrinoid protein Co-methyltransferase	2.063895754	0.390108253	2.089474031	0.186701652	0.851894561	0.999551235
2.7.1.180	FAD:protein FMN transferase	253.8645661	0.175160073	0.279391145	0.626934949	0.530701877	0.999551235
6.3.4.22	tRNA(Ile2)-agmatinylcytidine synthase	2.007550506	1.412367005	2.64842149	0.533286341	0.593835381	0.999551235
1.6.1.2	H+-translocating NAD(P) transhydrogenase	18.93975992	0.308226332	0.70969639	0.434307312	0.664065295	0.999551235
3.2.1.50	alpha-N-acetylglucosaminidase	95.82601796	0.47739778	0.348511727	1.36981841	0.170743594	0.999551235
2.1.1.216	tRNA (guanine26-N2/guanine27-N2)-dimethyltransferase	0.68372251	0.156859503	4.135271637	0.037932092	0.969741826	0.999551235
2.4.2.4	thymidine phosphorylase	11.4572742	-1.79158883	1.143983233	-1.566097105	0.117325886	0.999551235
6.1.1.16	#N/A	514.6442691	0.168743722	0.154986637	1.088763041	0.276258394	0.999551235
6.3.4.6	urea carboxylase	6.97224277	-0.348540588	2.007689796	-0.173602809	0.862177624	0.999551235
1.1.1.193	5-amino-6-(5-phosphoribosylamino)uracil reductase	165.0856682	0.369858284	0.31743605	1.165142663	0.24396125	0.999551235
6.3.2.43	[lysine-biosynthesis-protein LysW]---L-2-aminoadipate ligase	0.050191057	-0.752539914	4.143731151	-0.181609252	0.855889388	0.999551235
4.2.1.25	L-arabonate dehydrase	0.031664897	0.649715207	4.129795478	0.157323822	0.874989647	0.999551235
6.3.4.19	tRNA(Ile)-lysidine synthase	465.8400504	0.086104902	0.175360963	0.491015223	0.623415681	0.999551235
3.6.3.15	#N/A	523.0703408	-0.272725527	0.282900147	-0.964034589	0.335028585	0.999551235
6.3.2.38	N2-citryl-N6-acetyl-N6-hydroxylysine synthase	0.337074854	-1.507734052	4.104194764	-0.367364158	0.713347403	0.999551235
2.7.7.66	phosphoribosyl-dephospho-CoA transferase	0.948413065	0.393929992	2.946268475	0.133704717	0.893636075	0.999551235
2.3.1.251	lipid IVA palmitoyltransferase	12.68719458	1.024429168	1.574924491	0.650462403	0.51539358	0.999551235
2.1.1.201	demethylmenaquinone methyltransferase / 2-methoxy-6-polyp	59.19781542	0.530023609	0.374912485	1.413726218	0.157442304	0.999551235
3.5.4.4	adenosine deaminase	49.88921396	-0.245726283	0.435513513	-0.564221949	0.572603092	0.999551235
1.2.1.11	aspartate-semialdehyde dehydrogenase	391.8091547	-0.203133003	0.183749066	-1.105491349	0.268946741	0.999551235
3.2.1.55	alpha-N-arabinofuranosidase	304.048897	0.008281928	0.292267405	0.028336817	0.977393517	0.999551235
1.1.1.215	glyoxylate/hydroxypyruvate/2-ketogluconate reductase	1.070230206	0.29984372	4.094694583	0.073227371	0.941625186	0.999551235
4.2.1.114	methanogen homoaconitase large subunit	10.62242811	-3.715645036	1.876590096	-1.979998213	0.04770373	0.999551235
2.7.9.1	pyruvate, orthophosphate dikinase	3366.086176	-0.644127152	0.495798818	-1.299170406	0.193885455	0.999551235
6.2.1.14	6-carboxyhexanoate--CoA ligase	1.997081838	-0.002928938	2.204258626	-0.001328763	0.998939801	0.999551235
2.3.1.179	3-oxoacyl-[acyl-carrier-protein] synthase II	527.6690916	-0.021207692	0.20710545	-0.102400452	0.918438825	0.999551235
2.3.1.203	UDP-N-acetylbacillosamine N-acetyltransferase	1.434965331	-0.210978307	2.792632718	-0.075548175	0.939778569	0.999551235
2.4.2.12	nicotinamide phosphoribosyltransferase	17.36203353	0.673301453	1.123464322	0.599308265	0.548967337	0.999551235
3.6.1.22	NAD+ diphosphatase	138.749748	0.577228605	0.288902434	1.99800534	0.045716081	0.999551235
3.2.1.70	glucan 1,6-alpha-glucosidase	14.70370541	-1.297136709	0.736376839	-1.761512097	0.078151767	0.999551235
2.1.4.1	glycine amidinotransferase	45.52497431	-2.287687952	0.912836132	-2.506132122	0.012206	0.999551235
3.3.1.1	adenosylhomocysteinase	126.059348	0.053159427	0.285324473	0.18631219	0.852199949	0.999551235
2.7.1.140	inositol-polyphosphate multikinase	0.160692243	-0.391890765	4.143731151	-0.09457437	0.924652908	0.999551235
2.3.1.231	tRNA wybutosine-synthesizing protein 4	0.053564081	-0.391885504	4.143731151	-0.094573101	0.924653917	0.999551235
2.1.1.133	precorrin-4/cobalt-precorrin-4 C11-methyltransferase	110.7485461	-0.072906027	0.349326266	-0.208704682	0.834678785	0.999551235
1.1.1.346	2,5-diketo-D-gluconate reductase A	10.87598757	1.757859932	1.161439779	1.513517932	0.13014815	0.999551235
6.1.1.3	threonyl-tRNA synthetase	799.6942148	-0.138035143	0.199110049	-0.693260557	0.488146051	0.999551235
4.4.1.11	methionine-gamma-lyase	55.61284014	0.118000739	0.515808314	0.228768586	0.819048784	0.999551235
6.3.2.6	phosphoribosylaminoimidazole carboxylase / phosphoribosylan	329.075709	-0.06660475	0.16694319	-0.398966557	0.689917847	0.999551235
1.2.1.88	1-pyrroline-5-carboxylate dehydrogenase	93.26804562	-0.030733526	0.346040207	-0.0888149	0.929229016	0.999551235
3.4.16.4	D-alanyl-D-alanine carboxypeptidase	1095.067042	-0.290097693	0.27434223	-1.057429956	0.290315406	0.999551235
2.8.3.18	succinyl-CoA:acetate CoA-transferase	17.73499046	-0.03386079	0.444405106	-0.076193523	0.939265136	0.999551235
3.-.-.-	#N/A	8.523949024	-0.9078717	1.389394528	-0.653429736	0.513479274	0.999551235
4.2.1.45	CDP-glucose 4,6-dehydratase	79.53278265	0.090054087	0.278700253	0.323121665	0.746603111	0.999551235
3.5.4.33	tRNA(adenine34) deaminase	257.7751313	-0.779036245	0.572093236	-1.361729515	0.173283263	0.999551235
3.1.3.83	D-glycero-D-manno-heptose 1,7-bisphosphate phosphatase	19.00358607	0.492902309	0.460276656	1.070882703	0.284222174	0.999551235
1.13.11.54	1,2-dihydroxy-3-keto-5-methylthiopentene dioxygenase	0.050191057	-0.752539914	4.143731151	-0.181609252	0.855889388	0.999551235
2.7.2.2	carbamate kinase	210.8375222	-0.65583343	0.352453573	-1.860765448	0.062777307	0.999551235
2.1.1.22	carnosine N-methyltransferase	0	NA	NA	NA	NA	NA
2.3.1.7	carnitine O-acetyltransferase	0	NA	NA	NA	NA	NA
1.14.11.-	#N/A	0	NA	NA	NA	NA	NA
1.1.1.270	3-keto steroid reductase	0	NA	NA	NA	NA	NA
2.3.1.37	5-aminolevulinate synthase	0	NA	NA	NA	NA	NA
3.1.3.99	IMP and pyridine-specific 5'-nucleotidase	0	NA	NA	NA	NA	NA
2.7.8.11	CDP-diacylglycerol--inositol 3-phosphatidyltransferase	0	NA	NA	NA	NA	NA
3.2.1.106	mannosyl-oligosaccharide glucosidase	0	NA	NA	NA	NA	NA
2.4.1.256	alpha-1,2-glucosyltransferase	0	NA	NA	NA	NA	NA
4.1.1.45	aminocarboxymuconate-semialdehyde decarboxylase	0	NA	NA	NA	NA	NA
3.1.1.56	carboxylesterase 2	0	NA	NA	NA	NA	NA
4.1.1.86	L-2,4-diaminobutyrate decarboxylase	0	NA	NA	NA	NA	NA
2.4.99.-	#N/A	0	NA	NA	NA	NA	NA
4.1.3.41	3-hydroxy-D-aspartate aldolase	0	NA	NA	NA	NA	NA
3.6.1.42	guanosine-diphosphatase	0	NA	NA	NA	NA	NA

3.1.22.-	#N/A	0	NA	NA	NA	NA	NA
2.8.3.22	succinyl-CoA:(S)-malate CoA-transferase subunit A	0	NA	NA	NA	NA	NA
2.7.1.182	phytol kinase	0	NA	NA	NA	NA	NA
2.4.1.131	#N/A	0	NA	NA	NA	NA	NA
2.4.2.6	nucleoside deoxyribosyltransferase	0	NA	NA	NA	NA	NA
2.3.1.222	phosphate propanoyltransferase	0	NA	NA	NA	NA	NA
2.7.7.50	mRNA guanylyltransferase	0	NA	NA	NA	NA	NA
1.3.3.11	pyrroloquinoline-quinone synthase	0	NA	NA	NA	NA	NA
3.5.1.110	ureidoacrylate peracid hydrolase	0	NA	NA	NA	NA	NA
4.1.3.43	4-hydroxy-2-oxovalerate/4-hydroxy-2-oxohexanoate aldolase	0	NA	NA	NA	NA	NA
2.7.1.82	ethanolamine kinase	0	NA	NA	NA	NA	NA
2.5.1.85	all-trans-nonaprenyl-diphosphate synthase	0	NA	NA	NA	NA	NA
2.1.1.225	tRNA:m4X modification enzyme	0	NA	NA	NA	NA	NA
3.1.1.97	diphthine methyl ester acylhydrolase	0	NA	NA	NA	NA	NA
4.2.1.134	very-long-chain (3R)-3-hydroxyacyl-CoA dehydratase	0	NA	NA	NA	NA	NA
2.7.1.150	1-phosphatidylinositol-3-phosphate 5-kinase	0	NA	NA	NA	NA	NA
5.4.99.45	tRNA pseudouridine38/39 synthase	0	NA	NA	NA	NA	NA
3.5.3.17	guanidinopropionase	0	NA	NA	NA	NA	NA
2.5.1.58	protein farnesyltransferase subunit beta	0	NA	NA	NA	NA	NA
2.4.1.198	#N/A	0	NA	NA	NA	NA	NA
2.7.8.17	UDP-N-acetylglucosamine-lysosomal-enzyme	0	NA	NA	NA	NA	NA
2.4.1.267	alpha-1,3-glucosyltransferase	0	NA	NA	NA	NA	NA
2.6.1.108	(5-formylfuran-3-yl)methyl phosphate transaminase	0	NA	NA	NA	NA	NA
2.7.4.21	inositol-hexakisphosphate 5-kinase	0	NA	NA	NA	NA	NA
2.6.1.101	L-glutamine:2-deoxy-scyllo-inosose/3-amino-2,3-dideoxy-scyllo	0	NA	NA	NA	NA	NA
3.1.3.43	pyruvate dehydrogenase phosphatase	0	NA	NA	NA	NA	NA
3.6.1.62	mRNA-decapping enzyme subunit 2	0	NA	NA	NA	NA	NA
1.5.1.-	#N/A	0	NA	NA	NA	NA	NA
3.4.24.56	insulysin	0	NA	NA	NA	NA	NA
6.3.4.11	biotin---protein ligase	0	NA	NA	NA	NA	NA
3.1.4.37	2',3'-cyclic-nucleotide 3'-phosphodiesterase	0	NA	NA	NA	NA	NA
1.3.1.33	protochlorophyllide reductase	0	NA	NA	NA	NA	NA
3.4.17.22	carboxypeptidase D	0	NA	NA	NA	NA	NA
1.1.1.372	L-glyceraldehyde reductase	0	NA	NA	NA	NA	NA
1.3.99.23	all-trans-retinol 13,14-reductase	0	NA	NA	NA	NA	NA
1.1.1.292	1,5-anhydro-D-fructose reductase (1,5-anhydro-D-mannitol-for	0	NA	NA	NA	NA	NA
2.3.1.189	mycothiol synthase	0	NA	NA	NA	NA	NA
2.4.1.132	alpha-1,3/alpha-1,6-mannosyltransferase	0	NA	NA	NA	NA	NA
3.4.11.26	intermediate cleaving peptidase 55	0	NA	NA	NA	NA	NA
2.5.1.92	(2Z,6Z)-farnesyl diphosphate synthase	0	NA	NA	NA	NA	NA
2.5.1.68	short-chain Z-isoprenyl diphosphate synthase	0	NA	NA	NA	NA	NA
3.2.1.49	alpha-N-acetylgalactosaminidase	0	NA	NA	NA	NA	NA
6.3.4.9	biotin---protein ligase	0	NA	NA	NA	NA	NA
5.3.3.1	3beta-hydroxy-Delta5-steroid dehydrogenase / steroid Delta-is	0	NA	NA	NA	NA	NA
1.10.3.11	ubiquinol oxidase	0	NA	NA	NA	NA	NA
1.11.1.1	NADH peroxidase	0	NA	NA	NA	NA	NA
5.3.3.10	5-carboxymethyl-2-hydroxymuconate isomerase	0	NA	NA	NA	NA	NA
3.1.3.96	pseudouridine 5'-phosphatase	0	NA	NA	NA	NA	NA
3.6.1.43	dolichyldiphosphatase	0	NA	NA	NA	NA	NA
3.6.1.29	bis(5'-adenosyl)-triphosphatase	0	NA	NA	NA	NA	NA
2.4.1.101	alpha-1,3-mannosyl-glycoprotein beta-1,2-N-acetylglucosaminy	0	NA	NA	NA	NA	NA
2.3.2.17	peptidoglycan pentaglycine glycine transferase (the second and	0	NA	NA	NA	NA	NA
1.14.11.27	F-box and leucine-rich repeat protein 10/11	0	NA	NA	NA	NA	NA
5.4.3.9	glutamate 2,3-aminomutase	0	NA	NA	NA	NA	NA
2.4.1.250	D-inositol-3-phosphate glycosyltransferase	0	NA	NA	NA	NA	NA
6.2.1.16	acetoacetyl-CoA synthetase	0	NA	NA	NA	NA	NA
3.1.1.89	protein phosphatase methylesterase 1	0	NA	NA	NA	NA	NA
6.4.1.5	geranyl-CoA carboxylase alpha subunit	0	NA	NA	NA	NA	NA
2.1.1.183	18S rRNA (adenine1779-N6/adenine1780-N6)-dimethyltransfer	0	NA	NA	NA	NA	NA
5.1.3.37	mannuronan 5-epimerase	0	NA	NA	NA	NA	NA
1.10.2.-	#N/A	0	NA	NA	NA	NA	NA
2.1.1.214	tRNA (guanine10-N2)-methyltransferase	0	NA	NA	NA	NA	NA
3.1.3.66	inositol polyphosphate-4-phosphatase	0	NA	NA	NA	NA	NA
3.7.1.5	acylpyruvate hydrolase	0	NA	NA	NA	NA	NA
4.1.3.25	malyl-CoA/(S)-citramalyl-CoA lyase	0	NA	NA	NA	NA	NA
2.5.1.44	homospermidine synthase	0	NA	NA	NA	NA	NA
2.3.2.18	peptidoglycan pentaglycine glycine transferase (the fourth and	0	NA	NA	NA	NA	NA
3.5.3.23	succinylarginine dihydrolase	0	NA	NA	NA	NA	NA
1.3.1.88	tRNA-dihydrouridine synthase 1	0	NA	NA	NA	NA	NA
3.1.3.77	enolase-phosphatase E1	0	NA	NA	NA	NA	NA
1.1.99.21	D-sorbitol dehydrogenase (acceptor)	0	NA	NA	NA	NA	NA
2.1.1.320	type II protein arginine methyltransferase	0	NA	NA	NA	NA	NA
2.7.7.79	tRNA(His) guanylyltransferase	0	NA	NA	NA	NA	NA
6.2.1.41	HIP---CoA ligase	0	NA	NA	NA	NA	NA
5.1.1.17	isopenicillin-N epimerase	0	NA	NA	NA	NA	NA
6.2.1.34	feruloyl-CoA synthase	0	NA	NA	NA	NA	NA
2.7.4.12	T-even-induced deoxynucleotide kinase	0	NA	NA	NA	NA	NA
2.4.1.257	alpha-1,3/alpha-1,6-mannosyltransferase	0	NA	NA	NA	NA	NA
1.3.1.91	tRNA-dihydrouridine synthase 2	0	NA	NA	NA	NA	NA
2.8.1.9	molybdenum cofactor sulfurtransferase	0	NA	NA	NA	NA	NA
1.3.99.5	#N/A	0	NA	NA	NA	NA	NA
3.5.1.94	gamma-glutamyl-gamma-aminobutyrate hydrolase	0	NA	NA	NA	NA	NA
1.1.1.371	scyllo-inositol 2-dehydrogenase (NADP+)	0	NA	NA	NA	NA	NA
2.1.1.229	alkylated DNA repair protein alkB homolog 8	0	NA	NA	NA	NA	NA
1.14.14.5	alkanesulfonate monooxygenase	0	NA	NA	NA	NA	NA
1.12.1.4	iron-hydrogenase subunit alpha	0	NA	NA	NA	NA	NA
2.4.1.142	beta-1,4-mannosyltransferase	0	NA	NA	NA	NA	NA

2.4.1.258	alpha-1,3-mannosyltransferase	0	NA	NA	NA	NA	NA
2.4.1.241	digalactosyldiacylglycerol synthase	0	NA	NA	NA	NA	NA
1.13.11.15	3,4-dihydroxyphenylacetate 2,3-dioxygenase	0	NA	NA	NA	NA	NA
4.1.2.57	sulfofructosephosphate aldolase	0	NA	NA	NA	NA	NA
1.3.1.6	NADH-dependent fumarate reductase subunit A	0	NA	NA	NA	NA	NA
5.1.2.2	mandelate racemase	0	NA	NA	NA	NA	NA
1.6.99.1	NADPH2 dehydrogenase	0	NA	NA	NA	NA	NA
2.5.1.87	ditrans,polycis-polyprenyl diphosphate synthase	0	NA	NA	NA	NA	NA
2.1.1.202	multisite-specific tRNA:(cytosine-C5)-methyltransferase	0	NA	NA	NA	NA	NA
2.6.1.100	L-glutamine:2-deoxy-scyllo-inosose/3-amino-2,3-dideoxy-scyllo	0	NA	NA	NA	NA	NA
1.6.2.2	cytochrome-b5 reductase	0	NA	NA	NA	NA	NA
3.5.3.7	guanidinobutyrase	0	NA	NA	NA	NA	NA
1.12.5.1	#N/A	0	NA	NA	NA	NA	NA
1.2.1.60	5-carboxymethyl-2-hydroxymuconic-semialdehyde dehydrogen	0	NA	NA	NA	NA	NA
2.1.1.100	protein-S-isoprenylcysteine O-methyltransferase	0	NA	NA	NA	NA	NA
3.6.1.12	dCTP diphosphatase	0	NA	NA	NA	NA	NA
1.1.1.163	cyclopentanol dehydrogenase	0	NA	NA	NA	NA	NA
1.10.9.1	#N/A	0	NA	NA	NA	NA	NA
6.3.1.11	gamma-glutamylputrescine synthase	0	NA	NA	NA	NA	NA
3.6.1.5	apyrase	0	NA	NA	NA	NA	NA
1.1.1.320	benzil reductase ((S)-benzoin forming)	0	NA	NA	NA	NA	NA
3.4.23.1	pepsin A	0	NA	NA	NA	NA	NA
1.1.1.11	D-arabinitol 4-dehydrogenase	0	NA	NA	NA	NA	NA
3.2.1.46	galactosylceramidase	0	NA	NA	NA	NA	NA
3.2.2.3	uridine nucleosidase	0	NA	NA	NA	NA	NA
3.4.11.24	aminopeptidase S	0	NA	NA	NA	NA	NA
2.1.1.321	type III protein arginine methyltransferase	0	NA	NA	NA	NA	NA
2.4.1.265	alpha-1,3-glucosyltransferase	0	NA	NA	NA	NA	NA
3.5.4.-	#N/A	0	NA	NA	NA	NA	NA
2.3.1.225	palmitoyltransferase ZDHHC9/14/18	0	NA	NA	NA	NA	NA
4.2.1.115	UDP-N-acetylglucosamine 4,6-dehydratase	0	NA	NA	NA	NA	NA
1.13.11.1	catechol 1,2-dioxygenase	0	NA	NA	NA	NA	NA
1.1.1.21	aldehyde reductase	0	NA	NA	NA	NA	NA
3.5.5.7	aliphatic nitrilase	0	NA	NA	NA	NA	NA
3.5.4.34	tRNA-specific adenosine deaminase 1	0	NA	NA	NA	NA	NA
3.4.21.112	membrane-bound transcription factor site-1 protease	0	NA	NA	NA	NA	NA
6.3.1.14	diphthine-ammonia ligase	0	NA	NA	NA	NA	NA
2.1.1.287	ribosomal RNA-processing protein 8	0	NA	NA	NA	NA	NA
2.1.1.260	rRNA small subunit pseudouridine methyltransferase Nep1	0	NA	NA	NA	NA	NA
4.2.1.161	bisanhydrobacterioruberin hydratase	0	NA	NA	NA	NA	NA
3.4.22.49	separase	0	NA	NA	NA	NA	NA
2.7.11.13	classical protein kinase C alpha type	0	NA	NA	NA	NA	NA
2.1.1.205	tRNA (cytidine32/guanosine34-2'-O)-methyltransferase	0	NA	NA	NA	NA	NA
3.2.1.130	glycoprotein endo-alpha-1,2-mannosidase	0	NA	NA	NA	NA	NA
4.4.1.2	cystathionine gamma-lyase / homocysteine desulfhydrase	0	NA	NA	NA	NA	NA
3.2.1.155	xyloglucan-specific exo-beta-1,4-glucanase	0	NA	NA	NA	NA	NA
2.4.1.255	protein O-GlcNAc transferase	0	NA	NA	NA	NA	NA
3.5.1.96	succinylglutamate desuccinylase	0	NA	NA	NA	NA	NA
2.5.1.59	protein farnesyltransferase/geranylgeranyltransferase type-1 s	0	NA	NA	NA	NA	NA
2.5.1.84	all-trans-nonaprenyl-diphosphate synthase	0	NA	NA	NA	NA	NA
3.5.1.38	glutamin-(asparagin-)ase	0	NA	NA	NA	NA	NA
3.7.1.3	kynureninase	0	NA	NA	NA	NA	NA
2.3.1.78	heparan-alpha-glucosaminide N-acetyltransferase	0	NA	NA	NA	NA	NA
2.7.1.91	sphingosine kinase	0	NA	NA	NA	NA	NA
2.1.3.9	N-acetylornithine carbamoyltransferase	0	NA	NA	NA	NA	NA
3.5.4.30	dCTP deaminase (dUMP-forming)	0	NA	NA	NA	NA	NA
6.2.1.31	2-furoate---CoA ligase	0	NA	NA	NA	NA	NA
5.1.3.10	CDP-paratose 2-epimerase	0	NA	NA	NA	NA	NA
1.5.99.6	spermidine dehydrogenase	0	NA	NA	NA	NA	NA
3.5.1.11	penicillin amidase	0	NA	NA	NA	NA	NA
2.7.1.32	choline kinase	0	NA	NA	NA	NA	NA
2.7.1.157	N-acetylgalactosamine kinase	0	NA	NA	NA	NA	NA
1.1.1.51	aldo-keto reductase family 1 member C3	0	NA	NA	NA	NA	NA
1.6.3.4	NADH oxidase (H2O-forming)	0	NA	NA	NA	NA	NA
3.4.24.27	thermolysin	0	NA	NA	NA	NA	NA
1.14.13.81	magnesium-protoporphyrin IX monomethyl ester (oxidative) cy	0	NA	NA	NA	NA	NA
4.1.99.20	3-amino-4-hydroxybenzoic acid synthase	0	NA	NA	NA	NA	NA
2.7.7.52	terminal uridylyltransferase	0	NA	NA	NA	NA	NA
1.1.1.264	L-idonate 5-dehydrogenase	0	NA	NA	NA	NA	NA
2.7.8.29	phosphatidylserine synthase 2	0	NA	NA	NA	NA	NA
2.4.1.87	N-acetyllactosaminide 3-alpha-galactosyltransferase	0	NA	NA	NA	NA	NA
1.8.-.-	#N/A	0	NA	NA	NA	NA	NA
3.1.3.67	phosphatidylinositol-3,4,5-trisphosphate 3-phosphatase and du	0	NA	NA	NA	NA	NA
2.4.1.287	rhamnopyranosyl-N-acetylglucosaminyl-diphospho-decaprenol	0	NA	NA	NA	NA	NA
2.9.1.2	O-phospho-L-seryl-tRNASec:L-selenocysteinyl-tRNA synthase	0	NA	NA	NA	NA	NA
2.8.3.20	succinyl-CoA---D-citramalate CoA-transferase	0	NA	NA	NA	NA	NA
1.1.1.108	carnitine 3-dehydrogenase	0	NA	NA	NA	NA	NA
2.7.1.137	phosphatidylinositol 3-kinase	0	NA	NA	NA	NA	NA
6.3.4.10	biotin---protein ligase	0	NA	NA	NA	NA	NA
3.1.1.84	carboxylesterase 2	0	NA	NA	NA	NA	NA
4.1.1.77	2-oxo-3-hexenedioate decarboxylase	0	NA	NA	NA	NA	NA
2.5.1.60	geranylgeranyl transferase type-2 subunit beta	0	NA	NA	NA	NA	NA
3.4.21.72	IgA-specific serine endopeptidase	0	NA	NA	NA	NA	NA
1.3.5.5	15-cis-phytoene desaturase	0	NA	NA	NA	NA	NA
4.1.3.16	#N/A	0	NA	NA	NA	NA	NA
1.1.1.338	(2R)-3-sulfolactate dehydrogenase (NADP+)	0	NA	NA	NA	NA	NA
3.5.1.89	N-acetylglucosaminylphosphatidylinositol deacetylase	0	NA	NA	NA	NA	NA

1.2.99.8	glyceraldehyde dehydrogenase large subunit	0	NA	NA	NA	NA	NA

Table S17: Enzymatic Functions (EC_DGE), Enhanced Diet, Late in Mission

	Description	baseMean	log2FoldChange	lfcSE	stat	pvalue	padj
3.4.22.16	cathepsin H	0.484790941	-19.1447048	4.129688215	-4.635871719	3.55E-06	0.007051857
3.1.3.10	glucose-1-phosphatase	1.077587015	-17.56525896	4.084057988	-4.300932801	1.70E-05	0.011247999
4.2.1.22	cystathionine beta-synthase	0.640036229	-17.71765255	4.118113293	-4.302371326	1.69E-05	0.011247999
2.1.1.113	site-specific DNA-methyltransferase (cytosine-N4-specific)	0.436862061	-17.47430365	4.132390397	-4.228618784	2.35E-05	0.011662466
3.1.31.1	micrococcal nuclease	51.00902493	3.724798366	0.941172458	3.957615137	7.57E-05	0.030038474
2.1.1.178	16S rRNA (cytosine1407-C5)-methyltransferase	0.518083772	-15.97085644	4.129589284	-3.867420061	1.10E-04	0.036370975
2.1.3.1	methylmalonyl-CoA carboxyltransferase 5S subunit	0.363147851	-15.12679293	4.140519072	-3.65335666	2.59E-04	0.073361038
5.1.1.3	glutamate racemase	455.6094706	1.610938557	0.486377068	3.312118646	9.26E-04	0.229628825
2.2.1.7	1-deoxy-D-xylulose-5-phosphate synthase	707.1842448	0.437665745	0.149497921	2.927570773	3.42E-03	0.753085179
3.5.3.9	allantoate deiminase	16.8615293	-1.472238004	0.524737681	-2.805664731	5.02E-03	0.830186742
6.3.2.4	D-alanine-D-alanine ligase	563.867796	1.282499477	0.449606367	2.85249403	4.34E-03	0.830186742
4.6.1.17	cyclic pyranopterin monophosphate synthase	64.49444168	-1.144349916	0.4067198	-2.813607587	4.90E-03	0.830186742
6.1.1.19	arginyl-tRNA synthetase	902.3901318	0.968046257	0.354072953	2.734030509	6.26E-03	0.886624759
4.6.1.12	2-C-methyl-D-erythritol 2,4-cyclodiphosphate synthase	260.3833251	0.609395333	0.221142976	2.755662168	5.86E-03	0.886624759
6.1.1.14	#N/A	786.3440721	0.8439858	0.331781991	2.543796293	1.10E-02	0.902234781
1.1.1.67	mannitol 2-dehydrogenase	18.00843058	3.459270211	1.323557166	2.613616019	8.96E-03	0.902234781
3.1.13.3	#N/A	459.8598424	0.819934809	0.322310132	2.543931223	1.10E-02	0.902234781
4.3.2.3	ureidoglycolate lyase	16.18073341	1.848077357	0.711039988	2.599118741	9.35E-03	0.902234781
2.7.1.184	sulfofructose kinase	16.85072223	2.365911997	0.876257649	2.700018655	6.93E-03	0.902234781
3.2.1.10	oligo-1,6-glucosidase	163.2185741	-0.7980565	0.305809258	-2.609654484	0.009063371	0.902234781
2.1.1.13	5-methyltetrahydrofolate--homocysteine methyltransferase	505.0696423	-0.687315566	0.261572067	-2.627633649	0.008598106	0.902234781
1.2.4.4	3-methyl-2-oxobutanoate dehydrogenase (2-methylpropanoyl-	91.63417995	1.007204408	0.379367574	2.654956503	0.007931864	0.902234781
3.4.21.116	stage IV sporulation protein B	182.2910614	1.248529921	0.490142213	2.54728095	0.010856599	0.902234781
2.7.1.208	PTS system, alpha-glucoside-specific IIB component	45.94357866	-1.575188156	0.622320869	-2.531151108	0.011368886	0.902234781
1.2.1.41	glutamate-5-semialdehyde dehydrogenase	367.9436807	-0.464365257	0.179841518	-2.582080392	0.009820671	0.902234781
2.1.1.-	#N/A	2085.570337	-1.240224513	0.501388484	-2.473579976	0.013376686	0.947833753
5.4.99.62	D-ribose pyranase	8.727499142	-2.120367439	0.851110993	-2.491293682	0.012727886	0.947833753
2.1.1.86	#N/A	346.4779204	3.68502419	1.48420875	2.482820688	0.013034668	0.947833753
2.5.1.48	cystathionine gamma-synthase	43.32150649	-1.241749917	0.506403417	-2.452096244	0.014202667	0.971658311
5.3.1.29	ribose 1,5-bisphosphate isomerase	0.633492245	-1.401012986	3.206986422	-0.436862775	0.66221087	0.996994191
4.4.1.25	L-cysteate sulfo-lyase	0.961780025	2.120936011	3.462159568	0.612604928	0.540137599	0.996994191
3.2.1.99	arabinan endo-1,5-alpha-L-arabinosidase	192.2445618	-0.304893568	0.384452196	-0.793059765	0.427743003	0.996994191
2.8.1.7	cysteine desulfurase	1018.936717	0.121434838	0.188821409	0.643120072	0.520146191	0.996994191
3.4.22.-	#N/A	1.269924312	-1.541209372	2.903375117	-0.530833706	0.595534021	0.996994191
2.5.1.9	#N/A	198.0755275	-0.908713639	0.614929467	-1.477752633	0.139474001	0.996994191
3.2.1.22	alpha-galactosidase	849.0917731	-0.197870053	0.19941187	-0.992268181	0.321066721	0.996994191
5.3.1.17	4-deoxy-L-threo-5-hexosulose-uronate ketol-isomerase	320.2434146	-0.119528768	0.404475122	-0.295515746	0.767599926	0.996994191
3.2.1.132	chitosanase	0.19424192	0.479413265	4.118331709	0.11640958	0.907327943	0.996994191
6.3.2.26	N-(5-amino-5-carboxypentanoyl)-L-cysteinyl-D-valine synthase	0.04941368	2.308811263	4.121547766	0.560180639	0.575356231	0.996994191
3.1.3.18	phosphoglycolate phosphatase	615.2274367	-0.650848883	0.433034608	-1.502995074	0.132840314	0.996994191
1.3.8.6	glutaryl-CoA dehydrogenase	0.043850487	1.769618963	4.123058137	0.429200585	0.667777258	0.996994191
1.1.1.44	6-phosphogluconate dehydrogenase	235.2879129	0.841704208	0.411023082	2.047827104	0.040576944	0.996994191
2.5.1.47	cysteine synthase	467.5769601	-0.539602908	0.354609497	-1.521682052	0.128088767	0.996994191
6.3.2.7	UDP-N-acetylmuramoyl-L-alanyl-D-glutamate-L-lysine ligase	3.344305032	0.857596851	1.602808205	0.535058935	0.592609105	0.996994191
3.5.2.10	creatinine amidohydrolase	54.4423082	-0.567536595	0.364979133	-1.55498368	0.119949956	0.996994191
4.2.2.20	chondroitin-sulfate-ABC endolyase/exolyase	72.26463302	-1.372847469	0.59268238	-2.316329141	0.020540304	0.996994191
1.2.3.3	pyruvate oxidase	1.435492674	2.651376331	3.023368579	0.876961	0.380507822	0.996994191
1.1.1.6	glycerol dehydrogenase	111.0345153	-0.088128506	0.444217541	-0.198390423	0.84273961	0.996994191
2.7.7.70	D-beta-D-heptose 7-phosphate kinase / D-beta-D-heptose 1-ph	16.49374106	-1.037872963	0.746008152	-1.391235418	0.16415405	0.996994191
6.1.1.11	seryl-tRNA synthetase	603.6102613	0.188127575	0.163233928	1.1525029	0.249114479	0.996994191
3.1.1.41	cephalosporin-C deacetylase	11.56161355	0.443079039	1.060927349	0.417633724	0.676214936	0.996994191
2.5.1.39	4-hydroxybenzoate polyprenyltransferase	8.366356724	-1.222205691	0.916695797	-1.33327293	0.182442254	0.996994191
2.6.1.37	2-aminoethylphosphonate-pyruvate transaminase	24.00016452	-0.898481814	0.664927922	-1.351246932	0.176616345	0.996994191
1.14.19.3	linoleoyl-CoA desaturase	0.048153551	1.099249132	4.140903343	0.265461191	0.790654203	0.996994191
1.5.1.43	carboxynorspermidine synthase	0.02470684	2.040638566	4.125310065	0.494663076	0.62083797	0.996994191
2.3.3.13	2-isopropylmalate synthase	404.3772946	-0.359923524	0.208650026	-1.725010687	0.084525547	0.996994191
3.5.1.28	N-acetylmuramoyl-L-alanine amidase	701.2008379	0.029536006	0.17109752	0.172626732	0.862944839	0.996994191
2.4.2.3	uridine phosphorylase	208.4116149	-0.048511283	0.384061571	-0.126311212	0.899485581	0.996994191
3.4.19.5	beta-aspartyl-peptidase (threonine type)	13.69378794	-0.072817906	0.509180174	-0.143010097	0.886282207	0.996994191
1.15.1.2	superoxide reductase	676.2410151	0.429861113	0.610378795	0.704253027	0.481275214	0.996994191
2.4.1.20	cellobiose phosphorylase	381.5764817	-0.17105593	0.422542911	-0.40482499	0.685606158	0.996994191
3.1.11.6	#N/A	653.3635538	-0.045311005	0.20742933	-0.218440687	0.827085767	0.996994191
1.3.1.106	precorrin-6A/cobalt-precorrin-6A reductase	61.18763666	-0.096806526	0.372013594	-0.260223087	0.794691697	0.996994191
2.8.4.5	threonylcarbamoyladenosine tRNA methylthiotransferase CDK/	414.9569557	0.443586981	0.375455794	1.181462606	0.237419	0.996994191
2.7.7.23	UDP-N-acetylglucosamine/UDP-N-acetylgalactosamine diphosp	454.2087906	-0.127757684	0.36838944	-0.346800615	0.728741115	0.996994191
4.1.1.23	orotidine-5'-phosphate decarboxylase	397.5941849	0.084150171	0.152088838	0.553296168	0.580060624	0.996994191
2.7.4.2	phosphomevalonate kinase	2.382155935	-0.958713202	1.889388737	-0.507419772	0.611860315	0.996994191
3.5.1.54	allophanate hydrolase	0.281440112	2.585438389	4.110439925	0.628993109	0.529353567	0.996994191
2.4.1.44	UDP-D-galactose:(glucosyl)LPS alpha-1,3-D-galactosyltransferas	0.029793698	1.661718259	4.127569099	0.402590052	0.687249829	0.996994191
4.2.1.42	galactarate dehydratase	7.014879709	-0.625343575	0.976348692	-0.640492049	0.521852756	0.996994191
2.5.1.26	alkyldihydroxyacetonephosphate synthase	21.86515454	0.686803195	0.864849148	0.794130625	0.42711939	0.996994191
2.7.7.24	glucose-1-phosphate thymidylyltransferase	374.5950283	-0.133502881	0.193250855	-0.690826857	0.489674356	0.996994191
5.4.99.60	precorrin-8X/cobalt-precorrin-8 methylmutase	99.4358351	-0.043693865	0.33231724	-0.131482392	0.895393715	0.996994191
2.4.2.19	nicotinate-nucleotide pyrophosphorylase (carboxylating)	205.1715531	-0.172297253	0.265521779	-0.648900644	0.516402599	0.996994191
2.1.1.187	23S rRNA (guanine745-N1)-methyltransferase	118.7001715	-0.137619492	0.36425547	-0.377810365	0.705571474	0.996994191
3.4.11.18	methionyl aminopeptidase	1322.739451	0.340051739	0.390268149	0.871328443	0.383574844	0.996994191
2.1.1.204	tRNA (cytosine38-C5)-methyltransferase	0.015832449	1.463162531	4.140620044	0.353367978	0.723812589	0.996994191
1.2.1.43	#N/A	5.547997314	0.095399969	0.910619226	0.104763843	0.916563201	0.996994191
2.7.1.66	undecaprenol kinase	2.560303601	-1.815803675	2.173772581	-0.835323663	0.403535498	0.996994191
4.3.1.19	threonine dehydratase	237.8157219	-0.219139045	0.268686258	-0.815594538	0.414732074	0.996994191
3.7.1.12	cobalt-precorrin 5A hydrolase	120.4272526	-0.550892292	0.399047885	-1.380516757	0.167427594	0.996994191
1.1.1.62	17beta-estradiol 17-dehydrogenase	0.374948568	4.041750821	4.06761596	0.993641204	0.320397576	0.996994191
1.3.3.4	#N/A	5.486609838	1.248865359	0.949540312	1.315231531	0.188432101	0.996994191
3.5.4.16	GTP cyclohydrolase IA	118.9073355	-0.262921842	0.384952781	-0.682997642	0.494608332	0.996994191
2.1.1.200	tRNA (cytidine32/uridine32-2'-O)-methyltransferase	1.277044293	1.868280336	4.015670849	0.465247379	0.641754313	0.996994191

5.3.1.6	#N/A	448.1376431	0.081115777	0.228946256	0.35430052	0.723113678	0.996994191
1.1.1.335	UDP-N-acetyl-2-amino-2-deoxyglucuronate dehydrogenase	1.283507249	1.270110726	2.284474298	0.555975056	0.578227907	0.996994191
1.1.1.350	ureidoglycolate dehydrogenase (NAD+)	0.461004988	1.66405975	3.997350395	0.416290689	0.677197305	0.996994191
2.3.1.181	lipoyl(octanoyl) transferase	68.98595382	0.469035059	0.451143233	1.039658859	0.298498421	0.996994191
1.2.1.18	Oxidoreductases	18.30084634	0.099149572	0.539870965	0.183654203	0.854284738	0.996994191
2.1.1.104	caffeoyl-CoA O-methyltransferase	0.214256324	3.521308529	4.077187401	0.863661192	0.387774039	0.996994191
5.4.2.8	phosphomannomutase	164.6440248	0.159646086	0.289796732	0.550889879	0.581709166	0.996994191
1.2.99.5	#N/A	122.8406003	1.495404181	1.163459662	1.285308146	0.198684625	0.996994191
2.7.1.191	PTS system, mannose-specific IIA component	129.4912911	-0.380610818	0.483923522	-0.786510266	0.431568607	0.996994191
2.5.1.63	adenosyl-fluoride synthase	1.48293681	1.237399245	1.719090436	0.719798807	0.47164888	0.996994191
2.8.3.-	#N/A	28.61882816	0.26190083	0.776601408	0.337239705	0.735936208	0.996994191
3.6.4.12	DNA replication licensing factor MCM5	5705.400828	0.036243396	0.256476051	0.141312983	0.887622694	0.996994191
1.3.99.32	glutaryl-CoA dehydrogenase (non-decarboxylating)	1.134042721	3.877470312	4.063961283	0.954111037	0.34002744	0.996994191
1.12.98.1	#N/A	212.01706	1.243146615	1.348955468	0.921562383	0.356756888	0.996994191
5.4.99.26	tRNA pseudouridine65 synthase	2.537247887	1.166122858	1.925982097	0.60546921	0.544867277	0.996994191
1.4.1.2	glutamate dehydrogenase	10.5705985	0.342035221	1.085775629	0.315014642	0.752750537	0.996994191
3.1.21.1	deoxyribonuclease I	2.200241027	0.823422501	1.683632254	0.489075034	0.624788576	0.996994191
2.7.7.83	UDP-N-acetylglucosamine/UDP-N-acetylgalactosamine diphosp	64.34672785	0.387764088	0.596464139	0.650104613	0.51562465	0.996994191
2.6.1.62	adenosylmethionine---8-amino-7-oxononanoate aminotransfer	70.13286159	-0.925863843	0.611244151	-1.514720167	0.129843285	0.996994191
2.4.1.58	UDP-glucose:(galactosyl)LPS alpha-1,2-glucosyltransferase	0.173954458	0.970247712	4.11193917	0.235958673	0.813464739	0.996994191
1.8.3.2	thiol oxidase	0.321384486	3.896626821	4.069934058	0.957417679	0.338356479	0.996994191
1.1.1.310	(S)-sulfolactate dehydrogenase	0.517880281	1.713158025	4.125240695	0.415286804	0.67793196	0.996994191
2.6.1.9	histidinol-phosphate aminotransferase	276.1067268	-0.164665955	0.306671892	-0.53694505	0.591305571	0.996994191
3.1.4.53	3',5'-cyclic-AMP phosphodiesterase	4.758026176	0.6070131	1.568272218	0.387058505	0.698712896	0.996994191
3.2.1.26	beta-fructofuranosidase	664.1409514	-0.165619587	0.281175134	-0.589026436	0.55584354	0.996994191
1.4.3.5	pyridoxamine 5'-phosphate oxidase	35.59689684	0.294886148	0.455938818	0.646766927	0.517782801	0.996994191
4.2.1.3	#N/A	719.8495603	0.125859303	0.251528234	0.50037843	0.616808638	0.996994191
5.1.3.23	UDP-GlcNAc3NAcA epimerase	1.280800804	2.957843192	2.090372192	1.414983993	0.157073189	0.996994191
4.2.3.4	3-dehydroquinate synthase	250.9900161	-0.204190204	0.265200917	-0.769945316	0.441332331	0.996994191
2.4.2.30	poly [ADP-ribose] polymerase 16	7.933816272	2.319078952	1.035256534	2.240100764	0.025084382	0.996994191
6.3.2.32	gamma-F420-2:alpha-L-glutamate ligase	2.771140777	3.35366593	2.350136118	1.427009229	0.153577234	0.996994191
1.3.98.3	oxygen-independent coproporphyrinogen III oxidase	588.784638	-0.348100134	0.26506913	-1.313242829	0.189101136	0.996994191
2.1.1.207	tRNA (cytidine/uridine-2'-O-)-methyltransferase	189.2348351	-0.318458008	0.41048712	-0.775805116	0.437864063	0.996994191
1.4.1.13	glutamate synthase (NADPH) large chain	1092.906852	-0.188949853	0.136178314	-1.387517929	0.165283888	0.996994191
4.2.1.76	UDP-glucose 4,6-dehydratase	0.160692243	3.261377394	4.083493213	0.798673397	0.424479817	0.996994191
1.11.1.9	glutathione peroxidase	52.20331418	0.303660971	0.485360349	0.625640253	0.53155093	0.996994191
4.1.3.-	#N/A	212.8288924	-0.16681077	0.310944397	-0.536464948	0.591637255	0.996994191
3.4.23.36	signal peptidase II	258.7444701	0.092110829	0.2854999	0.322629986	0.746975482	0.996994191
1.14.13.89	#N/A	0.349229033	1.271369748	4.137841476	0.307254339	0.758649788	0.996994191
2.4.1.211	1,3-beta-galactosyl-N-acetylhexosamine phosphorylase	341.5823143	-0.21886318	0.470692294	-0.464981438	0.641944749	0.996994191
5.5.1.4	myo-inositol-1-phosphate synthase	93.41411235	0.27573319	0.455132439	0.60583067	0.544627201	0.996994191
4.1.1.96	carboxynorspermidine decarboxylase	183.4516111	-0.428902762	0.340524143	-1.259537015	0.207836429	0.996994191
2.5.1.32	15-cis-phytoene synthase	6.327521558	1.08159874	0.805273886	1.34314394	0.17922537	0.996994191
3.5.1.1	L-asparaginase	324.8676599	0.293318255	0.252675709	1.160848646	0.245703456	0.996994191
5.3.1.-	#N/A	0.692871563	0.701120987	2.796901667	0.250677739	0.802063273	0.996994191
3.4.17.-	#N/A	48.75156414	-0.215288294	0.555371356	-0.387647458	0.698276944	0.996994191
3.4.24.37	saccharolysin	0.321384486	3.896626821	4.069934058	0.957417679	0.338356479	0.996994191
6.2.1.-	#N/A	91.10223053	-0.336762089	0.343817661	-0.979478738	0.327343489	0.996994191
3.5.1.42	nicotinamide-nucleotide amidase	360.2838083	-0.049439574	0.219733493	-0.224997896	0.821980911	0.996994191
6.2.1.4	#N/A	4.11168199	3.031141928	2.092607339	1.448500094	0.14747724	0.996994191
1.17.3.2	xanthine dehydrogenase/oxidase	0.783456853	1.656443693	4.046457455	0.409356508	0.682278052	0.996994191
1.17.7.1	(E)-4-hydroxy-3-methylbut-2-enyl-diphosphate synthase	384.5079502	-0.096213522	0.187826966	-0.512245521	0.608479188	0.996994191
4.2.1.51	cyclohexadienyl dehydratase	227.5628859	-0.210101888	0.368448329	-0.570234334	0.568518772	0.996994191
1.3.1.28	2,3-dihydro-2,3-dihydroxybenzoate dehydrogenase	0.228525281	2.299800674	4.072336347	0.564737408	0.572252386	0.996994191
2.4.1.180	UDP-N-acetyl-D-mannosaminouronate:lipid I N-acetyl-D-mannc	6.876760302	0.542497098	0.86867693	0.624509618	0.532292956	0.996994191
3.2.1.18	sialidase-1	148.4124568	-0.482332573	0.321102878	-1.502112271	0.133068116	0.996994191
6.3.5.5	carbamoyl-phosphate synthase	1476.450991	-0.066302551	0.123032265	-0.538903765	0.589953259	0.996994191
3.4.25.2	ATP-dependent HslUV protease, peptidase subunit HslV	19.83543425	-0.291115998	1.004457939	-0.289823981	0.771950901	0.996994191
2.7.1.20	adenosine kinase	7.663806899	-1.420029502	1.181026264	-1.202369114	0.22922055	0.996994191
1.12.7.2	ferredoxin hydrogenase	28.97808088	0.46988322	0.418662514	1.122343666	0.261716347	0.996994191
1.12.1.2	NAD-reducing hydrogenase large subunit	0.096307101	0.646944639	4.130400399	0.156630006	0.875536453	0.996994191
1.2.1.-	#N/A	0.350393969	0.959347063	4.08811997	0.234667052	0.814467162	0.996994191
3.4.14.5	dipeptidyl-peptidase 4	245.7884548	-0.326030273	0.246803222	-1.321013037	0.186497017	0.996994191
5.4.3.-	#N/A	0.228801007	2.128302001	4.115023632	0.517202862	0.605014554	0.996994191
3.1.3.26	4-phytase / acid phosphatase	0.687972502	1.061870705	2.935698561	0.36170972	0.717568963	0.996994191
1.14.11.47	50S ribosomal protein L16 3-hydroxylase	1.752367224	0.916514388	1.735961029	0.527957928	0.597528537	0.996994191
2.7.7.64	UDP-sugar pyrophosphorylase	1.215621488	2.484245587	4.090907042	0.607260337	0.543678156	0.996994191
2.1.1.77	protein-L-isoaspartate(D-aspartate) O-methyltransferase	4.997462947	1.970974131	1.305969127	1.509204231	0.131246599	0.996994191
4.1.1.36	phosphopantothenoylcysteine decarboxylase	282.4352402	-0.02641046	0.163177027	-0.161851583	0.871422731	0.996994191
1.2.1.2	#N/A	428.0325481	0.970958555	0.755782297	1.284706666	0.198894808	0.996994191
3.2.1.185	non-reducing end beta-L-arabinofuranosidase	0.132242126	1.415244138	4.143098164	0.341590781	0.732658876	0.996994191
2.7.8.7	holo-[acyl-carrier protein] synthase	46.9670023	-0.111108546	0.39930242	-0.278256632	0.780815365	0.996994191
2.3.1.201	UDP-2-acetamido-3-amino-2,3-dideoxy-glucuronate N-acetyltra	31.47835353	-0.353726177	0.660956131	-0.535173457	0.592529919	0.996994191
5.99.1.3	#N/A	3140.163008	0.325206021	0.198011071	1.642362818	0.100514836	0.996994191
6.3.2.1	pantoate--beta-alanine ligase	113.077425	-0.327428833	0.327850938	-0.998712508	0.31793398	0.996994191
4.3.1.12	ornithine cyclodeaminase	44.99803754	-0.441711407	0.569480862	-0.775638719	0.437962332	0.996994191
2.1.1.14	5-methyltetrahydropteroyltriglutamate--homocysteine methylt	71.05358529	-0.149385594	0.406923668	-0.367109621	0.713537251	0.996994191
2.1.3.3	ornithine carbamoyltransferase	414.3448983	-0.111955549	0.234311861	-0.477805727	0.632788484	0.996994191
1.1.1.53	3alpha(or 20beta)-hydroxysteroid dehydrogenase	0.015832449	1.463162531	4.140620044	0.353367978	0.723812589	0.996994191
2.1.1.246	[methyl-Co(III) methanol-specific corrinoid protein]:coenzyme M	2.314203398	1.97085929	1.972789718	0.999021473	0.317784289	0.996994191
2.6.1.81	succinylornithine aminotransferase	0.107128162	2.905607862	4.094274383	0.709675901	0.47790514	0.996994191
5.4.99.5	chorismate mutase	340.3622117	-0.19768621	0.368525206	-0.536425207	0.591664714	0.996994191
2.7.7.12	UDPglucose--hexose-1-phosphate uridylyltransferase	361.433388	-0.29439871	0.209561822	-1.404829887	0.16007187	0.996994191
5.2.1.13	prolycopene isomerase	5.090923286	1.151634525	2.05070737	0.561579161	0.574402782	0.996994191
6.3.4.15	biotin---protein ligase	385.5616898	-0.248931435	0.220208477	-1.130435296	0.258292849	0.996994191
3.1.3.-	#N/A	259.2790445	-0.093217712	0.277315882	-0.336142708	0.736763254	0.996994191
2.5.1.72	quinolinate synthase	172.5065621	0.105342178	0.245395136	0.429275738	0.667722571	0.996994191

1.8.4.8	phosphoadenosine phosphosulfate reductase	26.55279816	-1.124557802	0.728764169	-1.543102488	0.122805911	0.996994191
2.3.1.20	diacylglycerol O-acyltransferase / wax synthase	0.015832449	1.463162531	4.140620044	0.353367978	0.723812589	0.996994191
1.5.1.28	opine dehydrogenase	16.88640933	-0.070724432	0.787769082	-0.089778127	0.92846353	0.996994191
2.7.4.16	thiamine-monophosphate kinase	124.9748864	0.224002294	0.31042476	0.721599314	0.470540861	0.996994191
1.1.1.125	#N/A	27.46965	-0.384681402	0.580298079	-0.662903111	0.507392609	0.996994191
2.6.1.85	para-aminobenzoate synthetase component II	58.14321162	-1.175446437	0.509407035	-2.307479789	0.021028088	0.996994191
4.4.1.16	selenocysteine lyase	211.520852	0.331477546	0.279878457	1.184362491	0.236269601	0.996994191
2.7.1.45	2-dehydro-3-deoxygluconokinase	656.6700001	0.313900018	0.320384306	0.979760904	0.327204155	0.996994191
2.7.1.92	5-dehydro-2-deoxygluconokinase	8.500724103	-0.383368302	0.861142711	-0.445185562	0.65618566	0.996994191
5.3.3.-	#N/A	0.107481033	1.349360801	4.124476657	0.327159277	0.74354742	0.996994191
3.4.22.47	gingipain K	144.844054	2.544900255	2.727848361	0.932933184	0.350854468	0.996994191
3.1.27.6	ribonuclease I (enterobacter ribonuclease)	0.096307101	0.646944639	4.130400399	0.156630006	0.875536453	0.996994191
1.1.1.367	UDP-2-acetamido-2,6-beta-L-arabino-hexul-4-ose reductase	104.7367404	0.119942867	0.50134167	0.239243762	0.810916571	0.996994191
2.4.1.161	oligosaccharide 4-alpha-D-glucosyltransferase	0.654538727	0.870983698	4.102411627	0.212310167	0.831865064	0.996994191
1.14.12.19	3-phenylpropionate/trans-cinnamate dioxygenase subunit alph	0.192614202	0.463843892	4.112600639	0.112786028	0.910200195	0.996994191
2.7.7.42	[glutamine synthetase] adenylyltransferase / [glutamine synthe	8.353306205	-0.200371827	1.042546727	-0.192194577	0.847589794	0.996994191
5.1.3.11	cellobiose epimerase	175.8677582	-0.887697352	0.599952402	-1.479612965	0.138976565	0.996994191
1.6.3.3	NADH oxidase (H2O2-forming)	4.970299014	2.509588896	1.521014804	1.649943768	0.098954438	0.996994191
2.6.1.19	4-aminobutyrate aminotransferase	25.77244632	0.376149538	0.61411309	0.612508581	0.540201322	0.996994191
2.1.2.11	3-methyl-2-oxobutanoate hydroxymethyltransferase	154.993389	-0.067339259	0.249871377	-0.269495691	0.787548258	0.996994191
5.4.3.5	D-ornithine 4,5-aminomutase subunit beta	40.98653382	1.163958112	0.903484185	1.288299375	0.197641771	0.996994191
2.1.1.71	phosphatidyl-N-methylethanolamine N-methyltransferase	0.04941368	2.308811263	4.121547766	0.560180639	0.575356231	0.996994191
2.6.1.21	D-alanine transaminase	35.0129599	-0.13271991	0.435054409	-0.305065083	0.760316584	0.996994191
2.7.8.6	undecaprenyl-phosphate galactose phosphotransferase	3.522097742	1.794825355	1.847694292	0.971386534	0.331355831	0.996994191
5.4.99.22	23S rRNA pseudouridine2605 synthase	323.9159727	0.205361072	0.284275259	0.722402197	0.470047237	0.996994191
1.1.1.130	3-dehydro-L-gulonate 2-dehydrogenase	15.95022918	-0.441285147	0.628434775	-0.702197212	0.482556184	0.996994191
2.7.4.26	isopentenyl phosphate kinase	4.547246367	4.291620034	2.209758023	1.942122164	0.052122313	0.996994191
1.3.5.3	menaquinone-dependent protoporphyrinogen oxidase	8.639531851	0.170455196	0.819926132	0.207890917	0.835314141	0.996994191
1.1.1.90	aryl-alcohol dehydrogenase	0.138139806	1.219723085	4.118217636	0.296177423	0.76709459	0.996994191
3.1.3.78	phosphatidylinositol-4,5-bisphosphate 4-phosphatase	0.330216866	0.330633188	4.130022291	0.08005603	0.936192693	0.996994191
3.4.11.21	aspartyl aminopeptidase	198.5834177	-0.242401139	0.26054775	-0.93035207	0.352188826	0.996994191
3.4.21.-	#N/A	471.7887788	0.174032199	0.214101271	0.812849912	0.416304115	0.996994191
1.5.1.21	delta1-piperideine-2-carboxylate reductase	0.021925243	1.675481703	4.126913301	0.405989072	0.68475063	0.996994191
3.2.1.15	polygalacturonase	0.107128162	2.905607862	4.094274383	0.709675901	0.47790514	0.996994191
2.4.1.144	beta-1,4-mannosyl-glycoprotein beta-1,4-N-acetylglucosaminyl	2.052878989	0.314782603	1.921998823	0.16377877	0.869905305	0.996994191
1.1.1.83	tartrate dehydrogenase/decarboxylase / D-malate dehydrogen	0.660399619	-0.297704537	4.125124233	-0.072168623	0.942467715	0.996994191
2.1.1.173	23S rRNA (guanine2445-N2)-methyltransferase / 23S rRNA (gua	1.561324307	-1.792221137	1.855881177	-0.965698213	0.334195214	0.996994191
1.1.1.287	D-arabinitol dehydrogenase (NADP+)	0.353113201	2.095479698	4.106990764	0.510222647	0.609895488	0.996994191
6.1.1.-	#N/A	4.095223106	1.447290026	1.511319597	0.957633335	0.338247683	0.996994191
6.4.1.3	acetyl-CoA/propionyl-CoA carboxylase	161.1962201	0.492862822	0.327748165	1.503785145	0.132636697	0.996994191
1.7.2.2	nitrite reductase (cytochrome c-552)	50.95058939	-0.982460826	0.565302726	-1.737937533	0.082221821	0.996994191
5.4.4.2	salicylate biosynthesis isochorismate synthase	53.1414597	-0.443343782	0.402579258	-1.101258381	0.270784219	0.996994191
3.1.4.14	acyl carrier protein phosphodiesterase	0.144460652	0.533493742	4.118712294	0.129529257	0.896938876	0.996994191
2.3.1.8	phosphate acetyltransferase	784.4992269	-0.1287237	0.51329891	-0.250777271	0.801986315	0.996994191
2.1.1.182	16S rRNA (adenine1518-N6/adenine1519-N6)-dimethyltransfer	406.2000725	-0.050878384	0.450600516	-0.112912396	0.910100009	0.996994191
4.2.2.2	pectate lyase	6.801543122	-0.43416279	1.335952812	-0.324983627	0.745193464	0.996994191
2.7.3.9	#N/A	939.61253	0.289242661	0.31148289	0.928598876	0.353097004	0.996994191
4.3.1.3	histidine ammonia-lyase	192.3498098	-0.468726651	0.299277614	-1.566193491	0.117303326	0.996994191
3.1.13.1	exoribonuclease II	8.613747685	-1.073146354	1.100016532	-0.975572933	0.329276143	0.996994191
2.1.1.297	release factor glutamine methyltransferase	376.6235411	0.407181668	0.231810387	1.756529	0.078998124	0.996994191
3.6.3.10	H+/K+-exchanging ATPase	0.264436359	3.427376895	4.076086	0.840849995	0.400431975	0.996994191
3.5.1.87	beta-ureidopropionase / N-carbamoyl-L-amino-acid hydrolase	61.22171734	0.352066316	0.498446652	0.706326975	0.479984822	0.996994191
3.4.21.62	subtilisin	0.654451996	-0.854272257	4.090666615	-0.208834485	0.834577449	0.996994191
2.7.1.166	3-deoxy-D-manno-octulosonic acid kinase	0.202265241	2.538431052	4.114504451	0.616946969	0.537269705	0.996994191
3.1.1.29	peptidyl-tRNA hydrolase, PTH1 family	247.2072187	-0.356492118	0.351672921	-1.013703632	0.310724191	0.996994191
6.4.1.4	#N/A	0.160692243	3.261377394	4.083493213	0.798673397	0.424479817	0.996994191
2.3.1.42	glyceronephosphate O-acyltransferase	0.214256324	3.521308529	4.077187401	0.863661192	0.387774039	0.996994191
1.18.1.2	ferredoxin/flavodoxin---NADP+ reductase	452.6425692	-0.132032	0.284200085	-0.464574103	0.642236481	0.996994191
1.8.4.12	peptide-methionine (R)-S-oxide reductase	97.44710833	-0.057507926	0.322966191	-0.178061753	0.858674479	0.996994191
3.5.2.17	5-hydroxyisourate hydrolase	0.098827359	2.786904217	4.103455825	0.679160282	0.49703631	0.996994191
5.1.3.7	UDP-N-acetylglucosamine 4-epimerase	49.44551409	0.924974147	0.497973122	1.857478056	0.06324317	0.996994191
3.4.24.28	bacillolysin	0.63373079	1.249301817	4.0686673	0.307054307	0.758802037	0.996994191
1.1.2.4	D-lactate dehydrogenase (cytochrome)	3.268088468	1.420600656	1.302783378	1.090435049	0.27552155	0.996994191
2.7.11.11	protein kinase A	0.642768973	4.558254324	4.061086832	1.122422276	0.261682938	0.996994191
5.1.3.26	GlcNAc-P-P-Und epimerase	22.84782111	-0.528872939	0.647839879	-0.816363666	0.414292171	0.996994191
5.1.3.30	D-psicose/D-tagatose/L-ribulose 3-epimerase	6.991926601	-0.596725765	1.038830331	-0.574420815	0.565683071	0.996994191
4.2.1.93	ATP-dependent NAD(P)H-hydrate dehydratase	0.015832449	1.463162531	4.140620044	0.353367978	0.723812589	0.996994191
4.2.3.153	(5-formylfuran-3-yl)methyl phosphate synthase	5.230391143	4.092425593	2.30086729	1.778644779	0.075298018	0.996994191
2.4.1.5	dextransucrase	2.832586369	1.626264691	2.342755545	0.694167471	0.48757719	0.996994191
4.1.1.7	benzoylformate decarboxylase	0.102122587	1.373190375	4.141447253	0.331572586	0.740212023	0.996994191
2.3.1.75	diacylglycerol O-acyltransferase / wax synthase	0.015832449	1.463162531	4.140620044	0.353367978	0.723812589	0.996994191
3.4.23.40	phytepsin	0.116409678	1.396153691	4.142298814	0.337048039	0.736080687	0.996994191
3.2.1.123	endoglycosylceramidase	0.69313487	2.416100152	4.072003557	0.593344313	0.552950749	0.996994191
2.7.1.25	adenylylsulfate kinase	63.6182501	-0.031404476	0.309553132	-0.101451005	0.919192449	0.996994191
2.9.1.-	#N/A	0.431659391	-0.610667487	4.106310911	-0.148714381	0.881779013	0.996994191
4.2.1.28	propanediol dehydratase large subunit	328.6225478	1.369107972	0.931816776	1.469288821	0.141754468	0.996994191
3.1.2.1	acetyl-CoA hydrolase	1.659607312	2.74959813	4.050766389	0.678784671	0.497274308	0.996994191
2.1.1.213	tRNA (guanine10-N2)-dimethyltransferase	2.980175938	4.875329054	2.512808909	1.94019093	0.05235649	0.996994191
1.1.1.178	3-hydroxyacyl-CoA dehydrogenase / 3-hydroxy-2-methylbutyry	0.642768973	4.558254324	4.061086832	1.122422276	0.261682938	0.996994191
2.7.1.56	1-phosphofructokinase	215.1212971	0.06028904	0.22703074	0.265554525	0.790582312	0.996994191
1.1.1.159	7-alpha-hydroxysteroid dehydrogenase	12.0655719	-0.304113411	0.793935369	-0.383045551	0.70168599	0.996994191
2.7.1.211	PTS system, sucrose-specific IIA component	72.37468344	-0.465812733	0.448291257	-1.039085028	0.298765194	0.996994191
3.2.1.4	endoglucanase	878.084613	0.039178448	0.477373599	0.082070831	0.934590388	0.996994191
2.7.7.33	glucose-1-phosphate cytidylyltransferase	68.99084955	-0.104881056	0.430131263	-0.243834998	0.807358614	0.996994191
2.4.1.41	polypeptide N-acetylgalactosaminyltransferase	0.290251399	3.136707783	4.076552337	0.769451125	0.441625548	0.996994191
4.2.1.130	D-lactate dehydratase / protein deglycase	0.096307101	0.646944639	4.130400399	0.156630006	0.875536453	0.996994191
2.8.1.11	adenylyltransferase and sulfurtransferase	19.1465002	-0.278100633	0.598157011	-0.464929154	0.641982192	0.996994191

3.4.13.22	zinc D-Ala-D-Ala dipeptidase	58.536724	0.440524504	0.426326805	1.033302384	0.301462412	0.996994191
4.4.1.8	#N/A	368.3176599	0.222598294	0.24894799	0.894155822	0.371238526	0.996994191
1.3.1.101	digeranylgeranylglycerophospholipid reductase	9.718970903	1.660175065	1.428689546	1.162026467	0.245224714	0.996994191
3.1.3.82	D-glycero-D-manno-heptose 1,7-bisphosphate phosphatase	19.00358607	0.123732139	0.517201282	0.239234014	0.810924129	0.996994191
2.7.1.144	tagatose 6-phosphate kinase	13.17025696	-0.844545044	0.916479405	-0.921510118	0.356784161	0.996994191
1.5.1.24	N5-(carboxyethyl)ornithine synthase	0.256368638	1.802937799	4.121922383	0.437402171	0.66181971	0.996994191
2.7.10.2	protein-tyrosine kinase	0.471880068	0.248664832	4.104313805	0.060586213	0.951688753	0.996994191
2.1.1.289	cobalt-precorrin-7 (C5)-methyltransferase	2.079831392	2.631224383	1.902687593	1.382898797	0.166695899	0.996994191
1.10.3.9	photosystem II P680 reaction center D1 protein	1.966809294	-2.373635436	4.041525393	-0.587311771	0.556994337	0.996994191
2.5.1.3	thiamine-phosphate pyrophosphorylase	291.6609735	-0.163665091	0.171806742	-0.952611575	0.34078691	0.996994191
1.16.1.3	aquacobalamin reductase / NAD(P)H-flavin reductase	1.180331694	2.472657046	4.028416753	0.613803684	0.539345062	0.996994191
2.1.1.195	cobalt-precorrin-5B (C1)-methyltransferase	169.9058599	-0.580981562	0.365535209	-1.589399728	0.111970176	0.996994191
5.3.1.9	glucose-6-phosphate isomerase	902.8368241	0.259513474	0.285774359	0.908106222	0.363822104	0.996994191
2.7.2.3	phosphoglycerate kinase	1431.260499	0.170180528	0.501079396	0.33962787	0.734136787	0.996994191
5.3.3.2	isopentenyl-diphosphate Delta-isomerase	13.07900694	1.459105664	0.829093334	1.759881069	0.078427974	0.996994191
3.4.18.1	cathepsin X	4.756805142	2.690187053	4.060854118	0.662468283	0.507671155	0.996994191
3.5.1.5	urease	257.9239362	0.249648983	0.722812207	0.345385677	0.729804443	0.996994191
4.2.1.99	aconitate hydratase 2 / 2-methylisocitrate dehydratase	3.37656264	-1.616656886	3.112983495	-0.519327163	0.603532615	0.996994191
2.3.1.241	Kdo2-lipid IVA lauroyltransferase	95.41321668	-0.191703745	0.318539821	-0.601820345	0.547293731	0.996994191
1.2.1.19	aminobutyraldehyde dehydrogenase	0.383706937	1.270481743	4.08252016	0.31120036	0.755648309	0.996994191
6.3.5.9	cobyrinic acid a,c-diamide synthase	160.4431359	-0.373553353	0.361652638	-1.032906478	0.301647667	0.996994191
3.5.2.2	dihydropyrimidinase	90.86795266	-0.351522018	0.355817511	-0.987927819	0.323188	0.996994191
1.18.6.1	nitrogenase delta subunit	63.88890217	-0.127918944	0.403825381	-0.316767966	0.751419673	0.996994191
2.7.7.80	adenylyltransferase and sulfurtransferase	19.1465002	-0.278100633	0.598157011	-0.464929154	0.641982192	0.996994191
5.3.1.25	L-fucose/D-arabinose isomerase	329.4791065	-0.604032942	0.394839344	-1.529819535	0.126061405	0.996994191
2.1.1.181	23S rRNA (adenine1618-N6)-methyltransferase	4.035600293	-1.16143804	1.067310174	-1.08819167	0.276510502	0.996994191
2.3.1.129	UDP-N-acetylglucosamine acyltransferase	179.9466588	0.037925832	0.352701812	0.10752945	0.914368963	0.996994191
1.16.1.10	ferric-chelate reductase [NAD(P)H]	0.174502634	1.715112867	4.115609418	0.416733634	0.67687325	0.996994191
4.2.1.104	cyanate lyase	0.715825094	3.079851272	3.645594044	0.844814654	0.398214331	0.996994191
3.5.99.7	1-aminocyclopropane-1-carboxylate deaminase	0.33950778	1.042306897	4.09000852	0.254842231	0.798844956	0.996994191
1.3.99.4	3-oxosteroid 1-dehydrogenase	1.106189635	1.762958977	3.237603264	0.544525945	0.5860796	0.996994191
1.2.1.22	lactaldehyde dehydrogenase / glycolaldehyde dehydrogenase	36.22550726	0.245123257	0.443635587	0.552532898	0.580583301	0.996994191
2.4.1.318	demethyllactenocin mycarosyltransferase	0.022430994	1.099249132	4.140903343	0.265461191	0.790654203	0.996994191
3.6.1.6	ectonucleoside triphosphate diphosphohydrolase 5/6	0.749897135	4.707473975	4.059612556	1.159587008	0.246216993	0.996994191
3.1.27.1	ribonuclease T2	0.308633526	2.186705211	4.114325926	0.531485655	0.595082279	0.996994191
1.1.1.40	malate dehydrogenase (oxaloacetate-decarboxylating)(NADP+)	110.9790257	-0.394420003	0.38335464	-1.02886456	0.303543324	0.996994191
3.2.1.51	alpha-L-fucosidase	1023.186115	-0.178191249	0.184153921	-0.967621261	0.333233555	0.996994191
5.4.99.9	UDP-galactopyranose mutase	320.18863	-0.062906045	0.2692456	-0.233638154	0.815265905	0.996994191
4.1.99.17	phosphomethylpyrimidine synthase	279.9666111	-0.336918819	0.263431011	-1.278964149	0.200909681	0.996994191
5.1.3.9	N-acylglucosamine-6-phosphate 2-epimerase	169.6607425	-0.291109414	0.474588941	-0.613392748	0.53961668	0.996994191
1.3.1.10	enoyl-acyl-carrier protein] reductase I	82.40735219	-0.35168704	0.318671038	-1.103605281	0.269764399	0.996994191
2.7.6.5	GTP pyrophosphokinase	1266.617419	-0.046820723	0.154248868	-0.303540139	0.761478263	0.996994191
1.2.1.72	D-erythrose 4-phosphate dehydrogenase	1.431547706	3.302170652	4.034909051	0.818400269	0.413128668	0.996994191
5.1.3.25	dTDP-L-rhamnose 4-epimerase	1.791576241	1.029389804	1.791918067	0.57446254	0.565654843	0.996994191
4.2.1.32	#N/A	69.34122182	-1.110868275	0.604121897	-1.838814784	0.065942433	0.996994191
4.2.1.149	crotonobetainyl-CoA hydratase	3.918396303	-0.829130354	2.039009902	-0.406633805	0.684276965	0.996994191
2.3.1.88	#N/A	0.364127506	2.307929596	4.083438509	0.56519269	0.571942708	0.996994191
5.5.1.1	muconate cycloisomerase	0.138446491	0.868541318	4.12365988	0.210623898	0.833180763	0.996994191
3.6.1.55	8-oxo-dGTP diphosphatase	335.946494	-0.095894187	0.26880325	-0.356744894	0.721282793	0.996994191
2.7.1.177	L-threonine kinase	0.594267165	0.302131947	4.103476016	0.073628296	0.941306155	0.996994191
3.7.1.17	4,5:9,10-diseco-3-hydroxy-5,9,17-trioxoandrosta-1(10),2-diene-	0.231939278	0.952858335	4.106808223	0.232019194	0.816523106	0.996994191
5.4.2.9	phosphoenolpyruvate phosphomutase	16.50042611	-1.096001244	0.846586571	-1.29461213	0.195454066	0.996994191
4.2.1.113	O-succinylbenzoate synthase	4.188888329	-0.753969683	1.385789009	-0.544072494	0.586391587	0.996994191
2.3.1.-	#N/A	274.7832898	-0.030850222	0.282125201	-0.109349401	0.912925365	0.996994191
1.16.3.2	ferritin heavy chain	526.0547936	0.553236089	0.459435439	1.204165029	0.2285258	0.996994191
1.11.1.21	catalase-peroxidase	8.342629727	-1.328391969	2.979087406	-0.445905671	0.655665386	0.996994191
5.1.3.-	#N/A	3.808216094	-1.450436793	1.386952033	-1.045772859	0.295665915	0.996994191
1.2.7.5	aldehyde:ferredoxin oxidoreductase	41.45335069	-0.261920682	0.718351072	-0.364613755	0.715399746	0.996994191
4.2.1.46	dTDP-glucose 4,6-dehydratase	442.2462817	0.047819251	0.183484709	0.26061709	0.794387809	0.996994191
3.5.5.1	nitrilase	12.224291	-0.336662566	0.786673854	-0.427956979	0.668682442	0.996994191
1.3.5.6	zeta-carotene desaturase	0.089381093	1.782761126	4.122597094	0.432436419	0.665424253	0.996994191
1.14.14.9	4-hydroxyphenylacetate 3-monooxygenase	3.60579661	-0.791012001	1.753494893	-0.451105962	0.651913181	0.996994191
6.3.2.35	D-alanine---D-serine ligase	27.59615683	0.199692207	0.557227916	0.358367198	0.720068536	0.996994191
2.7.7.1	nicotinamide-nucleotide adenylyltransferase	6.6903392	0.744538529	1.310427977	0.568164403	0.56992334	0.996994191
2.3.1.157	bifunctional UDP-N-acetylglucosamine pyrophosphorylase / Glu	389.7431354	-0.23699961	0.391295854	-0.605678816	0.544728054	0.996994191
3.1.3.6	2',3'-cyclic-nucleotide 2'-phosphodiesterase / 3'-nucleotidase	234.785924	0.565421757	0.329990145	1.713450435	0.086629705	0.996994191
4.3.1.29	D-glucosaminate-6-phosphate ammonia-lyase	0.089381093	1.782761126	4.122597094	0.432436419	0.665424253	0.996994191
3.1.3.27	phosphatidylglycerophosphatase GEP4	30.96879914	-0.091694309	0.563798813	-0.162636098	0.870804963	0.996994191
2.7.4.6	nucleoside-diphosphate kinase	76.50444022	0.093279177	0.609516906	0.153037883	0.878368401	0.996994191
3.1.26.3	#N/A	253.7334934	0.119960462	0.297326003	0.403464417	0.686606607	0.996994191
2.1.1.132	precorrin-6Y C5,15-methyltransferase (decarboxylating)	187.4625788	-0.409366451	0.347435841	-1.178250492	0.238696759	0.996994191
2.7.7.27	glucose-1-phosphate adenylyltransferase	2821.889123	0.108115507	0.44838937	0.241119692	0.809462351	0.996994191
1.12.1.3	NADP-reducing hydrogenase subunit HndB	339.9647259	0.458750915	0.391041561	1.173151299	0.240735138	0.996994191
3.4.24.13	IgA-specific metalloendopeptidase	0.02470684	2.040638566	4.125310065	0.494663076	0.62083797	0.996994191
1.11.1.6	#N/A	118.6950932	-0.15273362	0.44653433	-0.342042279	0.732319075	0.996994191
3.6.5.5	dynamin GTPase	0.770998033	1.958241205	4.080691366	0.479879763	0.631312892	0.996994191
2.7.2.8	acetylglutamate kinase	349.4705199	-0.279174094	0.32691754	-0.853958749	0.393127846	0.996994191
5.4.99.2	methylmalonyl-CoA mutase	438.421622	0.052876379	0.303938868	0.173970441	0.861888692	0.996994191
1.1.1.284	S-(hydroxymethyl)glutathione dehydrogenase / alcohol dehydro	1.229341188	2.762228704	3.354002871	0.823561819	0.410188578	0.996994191
4.1.1.82	phosphonopyruvate decarboxylase	16.79511523	-0.614092135	0.587707955	-1.044893352	0.296072263	0.996994191
1.1.1.197	carbonyl reductase 1	0.067578231	1.009477571	4.138890667	0.243900516	0.807307869	0.996994191
6.2.1.20	long-chain-fatty-acid--[acyl-carrier-protein] ligase	12.26279611	0.257623172	0.865766743	0.297566492	0.766034053	0.996994191
4.3.1.15	diaminopropionate ammonia-lyase	67.37423002	-0.059682517	0.645035946	-0.092525878	0.926280232	0.996994191
3.4.13.19	membrane dipeptidase	152.4527255	-0.131272783	0.296196319	-0.443195187	0.65762456	0.996994191
4.6.1.15	triose/dihydroxyacetone kinase / FAD-AMP lyase (cyclizing)	5.825853036	0.704699383	4.07553071	0.172909845	0.862722293	0.996994191
6.2.1.3	long-chain acyl-CoA synthetase	782.0385652	0.010738825	0.1652522	0.064984463	0.948186371	0.996994191
2.7.1.196	PTS system, cellobiose-specific IIA component	67.85843679	-0.646623341	0.617833935	-1.04659732	0.295285338	0.996994191

1.1.1.38	malate dehydrogenase (oxaloacetate-decarboxylating)	427.7254982	-0.366000439	0.424952358	-0.861274052	0.389087124	0.996994191
1.14.18.6	4-hydroxysphinganine ceramide fatty acyl 2-hydroxylase	0.100382114	1.111420998	4.120235158	0.269746982	0.787354914	0.996994191
6.3.4.21	nicotinate phosphoribosyltransferase	581.9678307	-0.477108385	0.45177426	-1.056076955	0.290933058	0.996994191
3.6.1.25	triphosphatase	0.063329795	1.458576194	4.141019462	0.352226356	0.724668513	0.996994191
1.5.98.1	methylenetetrahydromethanopterin dehydrogenase	30.1788584	2.416527331	1.228868915	1.966464691	0.049244957	0.996994191
4.2.1.17	enoyl-CoA hydratase	1168.14638	-0.078498442	0.62886509	-0.124825568	0.900661645	0.996994191
1.1.1.252	tetrahydroxynaphthalene reductase	0.063329795	1.458576194	4.141019462	0.352226356	0.724668513	0.996994191
4.4.1.21	S-ribosylhomocysteine lyase	148.3246946	-0.513599212	0.315351407	-1.628656795	0.103385694	0.996994191
2.1.1.72	site-specific DNA-methyltransferase (adenine-specific)	1204.253236	0.215887405	0.281009071	0.768257779	0.442334053	0.996994191
5.4.1.4	D-galactarolactone isomerase	0.015832449	1.463162531	4.140620044	0.353367978	0.723812589	0.996994191
3.1.13.-	#N/A	1.076630519	3.515558209	4.041033371	0.869965152	0.384319449	0.996994191
1.3.99.28	phytoene desaturase	1.632348624	3.119400767	4.065906252	0.767209221	0.442957131	0.996994191
5.3.1.3	L-fucose/D-arabinose isomerase	329.4791065	-0.604032942	0.394839344	-1.529819535	0.126061405	0.996994191
4.2.1.11	enolase	996.8971278	0.381041881	0.412180535	0.924453844	0.355250072	0.996994191
2.4.2.7	adenine phosphoribosyltransferase	209.6202566	0.380620214	0.218526446	1.741758129	0.081550776	0.996994191
3.4.11.5	proline iminopeptidase	13.02301904	-0.286895042	0.628763595	-0.456284436	0.648185455	0.996994191
1.1.1.23	histidinol dehydrogenase	291.6884958	0.049866319	0.232071482	0.214874825	0.829864913	0.996994191
1.5.1.34	dihydropteridine reductase	6.296940433	0.338353761	0.829714513	0.407795399	0.683423892	0.996994191
1.8.4.11	peptide-methionine (S)-S-oxide reductase	112.1664182	0.020321496	0.310994463	0.065343593	0.947900434	0.996994191
1.1.99.3	#N/A	1.815348364	1.472631002	2.549045613	0.577718576	0.563454136	0.996994191
5.3.2.5	2,3-diketo-5-methylthiopentyl-1-phosphate enolase	8.254332185	1.576346072	1.064901506	1.480274056	0.138800124	0.996994191
3.2.1.196	glycogen debranching enzyme	167.3467347	-0.64124865	0.327672991	-1.956977433	0.050350122	0.996994191
3.1.21.5	type III restriction enzyme	189.1393977	-0.023292087	0.347190759	-0.067087289	0.946512213	0.996994191
3.11.1.2	phosphonoacetate hydrolase	0.50996675	1.051679834	4.100234302	0.256492619	0.797570479	0.996994191
1.1.1.184	carbonyl reductase 1	0.067578231	1.009477571	4.138890667	0.243900516	0.807307869	0.996994191
3.5.4.27	methenyltetrahydromethanopterin cyclohydrolase	3.406741929	1.915846129	1.861289895	1.029310981	0.303333565	0.996994191
2.4.1.321	cellobionic acid phosphorylase	0.076526885	1.052005601	4.128957442	0.254787223	0.798887444	0.996994191
1.1.1.93	tartrate dehydrogenase	0.660399619	-0.297704537	4.125124233	-0.072168623	0.942467715	0.996994191
2.7.7.75	molybdopterin adenylyltransferase	3.318616763	0.656424019	1.608673507	0.408052981	0.683234779	0.996994191
2.7.7.87	L-threonylcarbamoyladenylate synthase	333.119198	-0.12451682	0.169630252	-0.73404843	0.462919222	0.996994191
3.6.3.21	#N/A	586.7150326	-0.40598621	0.402273597	-1.009229074	0.312864786	0.996994191
2.7.12.1	#N/A	0.589204892	4.473992392	4.061992578	1.101428008	0.270710421	0.996994191
2.4.1.109	dolichyl-phosphate-mannose-protein mannosyltransferase	0.048153551	1.099249132	4.140903343	0.265461191	0.790654203	0.996994191
1.3.1.54	precorrin-6A/cobalt-precorrin-6A reductase	61.18763666	-0.096806526	0.372013594	-0.260223087	0.794691697	0.996994191
1.2.4.2	2-oxoglutarate dehydrogenase E1 component	17.17650179	-0.440419778	1.210651926	-0.363787286	0.716016858	0.996994191
2.7.1.194	PTS system, ascorbate-specific IIA component	24.17034772	-0.851654151	0.712299375	-1.195640739	0.231836786	0.996994191
3.2.1.183	UDP-N-acetylglucosamine 2-epimerase (hydrolysing)	12.086231	-0.149469876	0.879708551	-0.169908404	0.865082172	0.996994191
2.6.1.82	putrescine aminotransferase	66.44150576	0.468731957	0.893750009	0.524455331	0.599961881	0.996994191
1.11.1.10	non-heme chloroperoxidase	0.399388983	0.2536972	4.103945022	0.061817885	0.950707861	0.996994191
2.4.2.29	queuine tRNA-ribosyltransferase	354.7608664	0.019361475	0.235367325	0.082260674	0.934439426	0.996994191
3.4.21.102	carboxyl-terminal processing protease	593.5158334	0.07477826	0.163723462	0.456735152	0.647861421	0.996994191
2.1.1.45	thymidylate synthase	208.9073855	0.319282889	0.331292812	0.963748312	0.335172126	0.996994191
2.7.1.6	galactokinase	521.2465307	-0.01295857	0.149439359	-0.086714572	0.930898393	0.996994191
2.6.1.66	valine--pyruvate aminotransferase	66.13941554	-0.986584683	0.511806403	-1.927652091	0.053898415	0.996994191
2.7.1.16	L-ribulokinase	36.69650726	0.191729543	0.427822776	0.448151791	0.654043652	0.996994191
6.3.2.30	cyanophycin synthetase	4.102299911	2.060207873	3.74717898	0.549802367	0.582454935	0.996994191
4.1.1.3	#N/A	1317.677733	0.232036571	0.208837092	1.111088884	0.266530092	0.996994191
3.2.1.11	dextranase	30.10552948	-0.860304448	0.594532159	-1.447027608	0.147889191	0.996994191
1.8.1.8	thioredoxin 2	161.0699188	-0.045620991	0.275248476	-0.165744754	0.868357825	0.996994191
3.1.4.-	#N/A	256.2826558	-0.086323117	0.281569456	-0.306578414	0.759164286	0.996994191
2.3.1.46	homoserine O-acetyltransferase/O-succinyltransferase	851.3442398	-1.479606083	0.826730533	-1.789707801	0.073500898	0.996994191
6.2.1.7	solute carrier family 27 (fatty acid transporter), member 5	4.984233103	3.352720507	4.021433684	0.833712743	0.404442876	0.996994191
2.4.1.280	N,N'-diacetylchitobiose phosphorylase	8.469517485	-0.693404042	1.24073237	-0.558866729	0.576252677	0.996994191
3.4.21.96	lactocepin	296.0567306	-0.680361235	0.969030953	-0.702104751	0.48261384	0.996994191
4.1.1.-	#N/A	0.100382114	1.111420998	4.120235158	0.269746982	0.787354914	0.996994191
4.1.1.49	phosphoenolpyruvate carboxykinase (ATP)	1039.813312	-0.049601108	0.390438751	-0.127039408	0.898909208	0.996994191
2.3.1.193	tRNA(Met) cytidine acetyltransferase	0.475511433	2.676364791	4.076356556	0.65655807	0.511465117	0.996994191
1.1.1.77	lactaldehyde reductase	536.8571836	0.058069206	0.412583366	0.140745389	0.888071087	0.996994191
1.3.1.85	crotonyl-CoA carboxylase/reductase	0.225617469	1.184904032	4.113083569	0.288081682	0.773284214	0.996994191
1.6.1.1	NAD(P) transhydrogenase	1.736111865	1.700965218	3.117932422	0.545542683	0.585380333	0.996994191
3.2.2.16	5'-methylthioadenosine nucleosidase	0.06610909	2.339491357	4.116341197	0.56834243	0.569802474	0.996994191
1.5.1.38	FMN reductase	7.661298639	0.465412142	1.159379235	0.401432187	0.688101956	0.996994191
1.1.1.137	ribulose-5-phosphate 2-dehydrogenase	33.26585746	0.917810896	0.781234469	1.174821301	0.240066216	0.996994191
2.1.1.34	tRNA (guanosine-2'-O-)-methyltransferase	13.99952319	-0.918118953	0.952803384	-0.963597493	0.335247764	0.996994191
3.2.2.26	futalosine hydrolase	2.601413997	-0.433041555	2.187184756	-0.197990386	0.843052585	0.996994191
3.4.23.49	omptin	0.144460652	0.533493742	4.118712294	0.129529257	0.896938876	0.996994191
2.7.11.23	CTD kinase subunit alpha	0.071041121	2.142783183	4.106048003	0.521860237	0.601767646	0.996994191
6.1.1.4	leucyl-tRNA synthetase	730.0393065	0.019786085	0.144424648	0.136999367	0.891031295	0.996994191
4.1.2.21	2-dehydro-3-deoxyphosphogalactonate aldolase	0.272063502	-0.254581606	4.113270827	-0.061892741	0.950648248	0.996994191
3.1.1.53	sialate O-acetylesterase	284.4241936	-0.136526585	0.294222406	-0.464025113	0.642629753	0.996994191
2.6.1.77	taurine-pyruvate aminotransferase	27.34860526	0.695719502	0.99339692	0.700343929	0.483712544	0.996994191
2.1.1.217	tRNA (adenine22-N1)-methyltransferase	279.7718674	-0.575381665	0.476550942	-1.207387529	0.227282936	0.996994191
2.1.1.242	16S rRNA (guanine1516-N2)-methyltransferase	13.47765524	-1.2116154	0.718358896	-1.686643552	0.091671912	0.996994191
3.5.4.3	guanine deaminase	103.5861723	-0.051833244	0.344288631	-0.150551716	0.880329352	0.996994191
1.13.11.53	1,2-dihydroxy-3-keto-5-methylthiopentene dioxygenase	0.050191057	1.314825092	4.126913272	0.318597704	0.750031595	0.996994191
4.1.3.1	isocitrate lyase	1.710554568	-1.909746508	3.355731696	-0.569099881	0.569288359	0.996994191
3.4.24.75	lysostaphin	0.258895683	0.826295269	4.095343814	0.201764566	0.840100784	0.996994191
2.2.1.10	fructose-bisphosphate aldolase / 2-amino-3,7-dideoxy-D-threo-	4.406406866	3.560146211	2.051042182	1.735774253	0.082603758	0.996994191
1.1.1.100	3-oxoacyl-[acyl-carrier protein] reductase	2297.248751	-0.053473365	0.514703762	-0.103891537	0.917255423	0.996994191
1.17.99.6	epoxyqueuosine reductase	21.31922042	-1.076112667	0.763998096	-1.408527944	0.158974798	0.996994191
2.4.99.14	3-deoxy-D-manno-octulosonic-acid transferase	102.8745696	-0.356249249	0.359208676	-0.99176126	0.321313999	0.996994191
4.2.1.24	porphobilinogen synthase	65.10279835	-0.242293962	0.342441995	-0.707547456	0.479226332	0.996994191
2.4.1.260	alpha-1,6-mannosyltransferase	0.214256324	3.521308529	4.077187401	0.863661192	0.387774039	0.996994191
6.3.5.4	asparagine synthase (glutamine-hydrolysing)	377.4072799	-0.079570201	0.365895225	-0.217467174	0.827844286	0.996994191
6.1.1.22	asparaginyl-tRNA synthetase	736.9327609	0.036988423	0.495222684	0.074690486	0.940460977	0.996994191
2.7.1.33	type I pantothenate kinase	321.0567096	-0.341980591	0.261627109	-1.307129801	0.191168632	0.996994191
6.5.1.1	bifunctional non-homologous end joining protein LigD	28.88893739	0.393267702	0.608432548	0.646362038	0.518044919	0.996994191

2.4.1.10	levansucrase	3.347830754	2.854327146	2.109693564	1.352958171	0.176068994	0.996994191
3.2.2.8	ribosylpyrimidine nucleosidase	9.911351524	-1.285380083	0.968765905	-1.326822173	0.184567514	0.996994191
4.2.1.35	3-isopropylmalate/(R)-2-methylmalate dehydratase large subur	629.01279	-0.144553666	0.24812134	-0.582592638	0.560167562	0.996994191
1.1.1.343	6-phosphogluconate dehydrogenase	235.2879129	0.841704208	0.411023082	2.047827104	0.040576944	0.996994191
2.7.1.59	N-acetylglucosamine kinase	0.694599604	1.077719498	4.070412178	0.264769132	0.791187318	0.996994191
2.5.1.114	tRNA wybutosine-synthesizing protein 2	2.024429067	2.597643973	4.057872607	0.640149217	0.522075594	0.996994191
2.4.1.336	1,2-diacylglycerol 3-beta-glucosyltransferase	0.462092179	1.637025509	4.117064532	0.397619589	0.690910618	0.996994191
1.3.1.-	#N/A	0.739864946	1.825575149	4.082247232	0.447198576	0.654731689	0.996994191
3.4.21.83	oligopeptidase B	1.186270295	-1.078578094	3.839693771	-0.280902113	0.77878548	0.996994191
2.7.3.-	#N/A	31.79458853	-1.028642776	0.555686803	-1.851119679	0.064152338	0.996994191
3.5.1.49	formamidase	2.54817764	-0.351030078	2.023810466	-0.173450075	0.862297667	0.996994191
1.2.1.76	malonyl-CoA/succinyl-CoA reductase (NADPH)	26.9509768	0.619283866	0.532068016	1.16391861	0.244456997	0.996994191
3.1.1.5	lysophospholipase	19.07257356	-1.438933117	0.699889146	-2.055944325	0.039787883	0.996994191
1.1.1.304	meso-butanediol dehydrogenase / (S,S)-butanediol dehydroger	3.716547359	-0.724865535	1.430846039	-0.506599254	0.612436032	0.996994191
2.3.1.81	aminoglycoside 3-N-acetyltransferase	36.3516502	-0.411485271	0.555152463	-0.741211286	0.458565342	0.996994191
5.3.1.12	glucuronate isomerase	483.7936617	-0.077409113	0.320524874	-0.241507349	0.809161921	0.996994191
2.1.1.61	tRNA 5-methylaminomethyl-2-thiouridine biosynthesis bifuncti	0.725194853	2.481318528	4.091035463	0.606525793	0.54416566	0.996994191
3.6.1.63	alpha-D-ribose 1-methylphosphonate 5-triphosphate diphospha	2.508459474	0.356987307	2.485179571	0.143646484	0.885779633	0.996994191
2.7.11.2	pyruvate dehydrogenase kinase 2/3/4	0.160692243	3.261377394	4.083493213	0.798673397	0.424479817	0.996994191
3.1.11.-	#N/A	0.288921303	0.368593529	4.10574585	0.089775047	0.928465977	0.996994191
2.1.1.249	dimethylamine---corrinoid protein Co-methyltransferase	0.443021872	3.290980429	4.080354087	0.806542854	0.419929909	0.996994191
2.3.-.-	#N/A	4.39435923	1.334675306	4.051261719	0.329446824	0.741817981	0.996994191
2.5.1.18	glutathione S-transferase	2.922263646	2.811149271	1.844836716	1.523792998	0.127560429	0.996994191
1.3.1.22	steroid 5-alpha-reductase	34.49108498	-0.327876096	0.551067545	-0.5949835	0.5518545	0.996994191
3.1.2.-	#N/A	237.5533798	-0.201142495	0.215199646	-0.934678557	0.349953981	0.996994191
4.2.1.8	mannonate dehydratase	345.6022606	-0.481061608	0.290402344	-1.656534865	0.097613551	0.996994191
4.1.1.94	ethylmalonyl-CoA/methylmalonyl-CoA decarboxylase	0.267820405	3.726658981	4.072976819	0.914971812	0.360206428	0.996994191
2.4.1.141	beta-1,4-N-acetylglucosaminyltransferase	0.053564081	2.349751186	4.116844279	0.570765136	0.568158859	0.996994191
3.4.11.2	aminopeptidase N	57.71238724	-0.976887329	0.475286636	-2.055364608	0.039843801	0.996994191
3.5.3.26	(S)-ureidoglycine aminohydrolase	1.594595982	-2.1924539	2.052810362	-1.068025543	0.28550899	0.996994191
4.2.2.6	oligogalacturonide lyase	13.30351774	-0.706303665	0.775915703	-0.91028402	0.362672744	0.996994191
2.7.1.162	N-acetylhexosamine 1-kinase	5.363247739	1.148593488	1.329401468	0.863992944	0.387591767	0.996994191
1.1.1.3	homoserine dehydrogenase	423.4702407	-0.274241796	0.182929908	-1.499163249	0.133831286	0.996994191
3.4.14.12	Xaa-Xaa-Pro tripeptidyl-peptidase	0.42378319	2.681820974	4.077791714	0.657665021	0.510753403	0.996994191
3.6.3.1	#N/A	0.057984819	1.314825092	4.126913272	0.318597704	0.750031595	0.996994191
2.6.1.-	#N/A	415.5908721	-0.076927261	0.199066757	-0.386439516	0.699171189	0.996994191
5.1.3.31	D-psicose/D-tagatose/L-ribulose 3-epimerase	6.991926601	-0.596725765	1.038830331	-0.574420815	0.565683071	0.996994191
3.2.1.141	maltooligosyltrehalose trehalohydrolase	0.197327189	1.936168186	4.118324022	0.470134981	0.638258583	0.996994191
1.2.7.8	indolepyruvate ferredoxin oxidoreductase, alpha subunit	497.2965975	0.439550981	0.285219675	1.541096284	0.123293354	0.996994191
5.3.3.14	3-hydroxyacyl-[acyl-carrier protein] dehydratase / trans-2-dece	1.695466807	2.105343094	2.333888992	0.902075078	0.367016987	0.996994191
3.1.3.70	mannosyl-3-phosphoglycerate phosphatase	0.043850487	1.769618963	4.123058137	0.429200585	0.667777258	0.996994191
2.4.1.292	GalNAc-alpha-(1->4)-GalNAc-alpha-(1->3)-diNAcBac-PP-undeca	0.092883485	0.868447891	4.137771167	0.209883016	0.833758978	0.996994191
2.7.4.8	guanylate kinase	389.5186938	-0.283934024	0.309983308	-0.915965529	0.359684975	0.996994191
3.5.1.108	UDP-3-O-[3-hydroxymyristoyl] N-acetylglucosamine deacetylas	302.2410722	1.100902687	0.770050206	1.429650532	0.152817345	0.996994191
1.97.1.9	Tat-targeted selenate reductase subunit YnfE	151.6737258	-0.201107889	0.338357643	-0.594364847	0.552268115	0.996994191
2.7.1.202	PTS system, fructose-specific IIA component	331.5898364	-0.16594235	0.249218617	-0.665850538	0.505506638	0.996994191
2.7.7.7	#N/A	5149.647638	-0.027733153	0.190770928	-0.145374105	0.884415509	0.996994191
2.3.1.101	formylmethanofuran--tetrahydromethanopterin N-formyltrans	8.959349101	2.082066538	1.670440016	1.24641802	0.212610969	0.996994191
2.5.1.16	spermidine synthase	117.6111361	-0.453433859	0.418935826	-1.08234682	0.279098449	0.996994191
3.6.3.31	polyamine-transporting ATPase	253.4407985	-0.424662823	0.439003229	-0.967334167	0.333377009	0.996994191
2.7.1.215	erythritol kinase (D-erythritol 1-phosphate-forming)	0.067292982	0.697548482	4.135308985	0.168681103	0.866047483	0.996994191
1.1.5.2	quinoprotein glucose dehydrogenase	1.663608877	-0.892429063	3.731058664	-0.239189234	0.810958851	0.996994191
2.5.1.76	cysteate synthase	7.56528018	1.698752285	0.96415435	1.761909061	0.078084664	0.996994191
3.2.1.78	mannan endo-1,4-beta-mannosidase	336.8419907	0.063215076	0.405515587	0.155888153	0.876121184	0.996994191
3.8.1.2	2-haloacid dehalogenase	83.07066021	-0.129104951	0.455628897	-0.283355493	0.776904342	0.996994191
2.1.1.186	23S rRNA (cytidine2498-2'-O)-methyltransferase	2.278291717	1.973679859	1.821276391	1.083679483	0.278506935	0.996994191
3.6.3.33	vitamin B12 transport system ATP-binding protein	0.098344608	0.874743224	4.128915508	0.211857865	0.832217923	0.996994191
1.3.1.104	enoyl-[acyl-carrier protein] reductase III	0.334667623	1.0701426	4.080515566	0.262256713	0.793123532	0.996994191
3.4.21.19	glutamyl endopeptidase	25.77571237	-0.427675737	0.724870947	-0.590002591	0.555188913	0.996994191
5.4.99.17	squalene-hopene/tetraprenyl-beta-curcumene cyclase	6.587769184	0.19700705	1.423540392	0.138392314	0.889930371	0.996994191
4.1.1.51	3-hydroxy-2-methylpyridine-4,5-dicarboxylate 4-decarboxylase	0.02470684	2.040638566	4.125310065	0.494663076	0.62083797	0.996994191
2.8.1.8	lipoyl synthase	68.84685767	-0.184360149	0.392968059	-0.469147924	0.638963906	0.996994191
2.7.1.29	triose/dihydroxyacetone kinase / FAD-AMP lyase (cyclizing)	5.825853036	0.704699383	4.07553071	0.172909845	0.862722293	0.996994191
3.1.2.23	4-hydroxybenzoyl-CoA thioesterase	2.101663812	0.319731799	2.129038331	0.150176629	0.880625264	0.996994191
3.2.1.180	unsaturated chondroitin disaccharide hydrolase	78.8314747	0.144350971	0.4934336	0.292543862	0.769870829	0.996994191
3.4.21.66	thermitase	0.052431119	0.78195638	4.139114155	0.188918776	0.850156477	0.996994191
2.7.1.74	deoxycitidine kinase	0.485024588	2.476627872	4.071758115	0.608245333	0.543024772	0.996994191
2.3.1.180	3-oxoacyl-[acyl-carrier-protein] synthase III	375.8443716	0.164358726	0.213572064	0.769570341	0.441554804	0.996994191
6.3.5.10	adenosylcobyric acid synthase	242.6327014	0.027527137	0.283523559	0.097089417	0.922655385	0.996994191
1.3.1.76	uroporphyrin-III C-methyltransferase / precorrin-2 dehydrogena	35.78655682	0.403558293	0.589955972	0.684048153	0.49394476	0.996994191
3.1.3.46	6-phosphofructo-2-kinase / fructose-2,6-biphosphatase 3	0.160692243	3.261377394	4.083493213	0.798673397	0.424479817	0.996994191
4.1.1.8	oxalyl-CoA decarboxylase	5.420868125	0.990509983	1.164275115	0.850752515	0.394906844	0.996994191
2.1.1.198	16S rRNA (cytidine1402-2'-O)-methyltransferase	509.3553814	0.012606566	0.181804986	0.069341146	0.944718075	0.996994191
3.2.1.17	lysozyme	43.33186982	-0.6721438	0.818285539	-0.82140496	0.411415639	0.996994191
3.5.3.3	creatinase	0.047497346	1.429004528	4.143731151	0.344859373	0.730200094	0.996994191
2.4.1.60	abequosyltransferase	0.322690315	2.169920804	4.114514955	0.527381922	0.597928395	0.996994191
2.8.3.12	#N/A	116.8754766	-0.057914831	0.586155689	-0.098804519	0.92129348	0.996994191
4.2.1.75	uroporphyrinogen-III synthase	152.2832938	-0.126496645	0.254751441	-0.496549281	0.619506927	0.996994191
2.4.2.43	4-amino-4-deoxy-L-arabinose transferase	0.449927408	0.849147007	4.102082575	0.207003879	0.836006829	0.996994191
3.4.24.70	oligopeptidase A	13.63582718	0.559900813	0.612189243	0.9145878	0.360408066	0.996994191
1.18.1.1	rubredoxin---NAD+ reductase	52.66113307	0.857761198	0.803624869	1.067365174	0.285806968	0.996994191
1.3.1.38	mitochondrial trans-2-enoyl-CoA reductase	0.868660033	1.982929019	4.08312431	0.485640129	0.627222339	0.996994191
4.2.1.151	chorismate dehydratase	11.30106601	-0.619699017	0.92674435	-0.668683891	0.503697146	0.996994191
2.4.1.259	alpha-1,2-mannosyltransferase	0.214256324	3.521308529	4.077187401	0.863661192	0.387774039	0.996994191
2.5.1.78	6,7-dimethyl-8-ribityllumazine synthase	142.8426069	-0.153039576	0.316143844	-0.484082101	0.628327603	0.996994191
2.7.8.12	CDP-glycerol glycerophosphotransferase	49.11916204	-0.575861324	0.572341943	-1.006149086	0.314343859	0.996994191
1.1.1.94	glycerol-3-phosphate dehydrogenase (NAD(P)+)	425.844855	0.278122141	0.204875829	1.357515633	0.17461743	0.996994191

3.5.3.8	formiminoglutamase	2.774074758	0.876818631	1.47470931	0.594570486	0.552130613	0.996994191
3.5.99.10	2-iminobutanoate/2-iminopropanoate deaminase	205.6363897	-0.205579777	0.364990196	-0.563247395	0.573266433	0.996994191
2.7.1.161	riboflavin kinase, archaea type	1.322181795	4.863009679	4.053697577	1.199647874	0.230276125	0.996994191
1.1.1.27	L-lactate dehydrogenase	263.1265027	0.232001924	0.310262508	0.7477601	0.454604873	0.996994191
3.1.21.2	deoxyribonuclease IV	300.1767179	-0.155905021	0.272613165	-0.571891021	0.567395808	0.996994191
2.8.3.5	3-oxoacid CoA-transferase	7.962912308	0.075170426	0.941032181	0.079880824	0.936332042	0.996994191
2.7.8.14	CDP-ribitol ribitolphosphotransferase / teichoic acid ribitol-phos	29.46695896	0.108028984	0.56245225	0.192067832	0.847689072	0.996994191
6.3.1.19	proteasome accessory factor A	0.403407769	0.364199012	4.131099963	0.0881603	0.92974927	0.996994191
3.4.17.13	muramoyltetrapeptide carboxypeptidase	30.21567958	-0.289291883	0.653105643	-0.442948068	0.657803298	0.996994191
3.4.22.70	sortase A	838.1445998	-0.12242885	0.193299156	-0.633364635	0.526495554	0.996994191
3.5.1.105	chitin disaccharide deacetylase	8.422222645	-0.369079056	1.145789708	-0.322117622	0.747363589	0.996994191
4.1.1.85	3-dehydro-L-gulonate-6-phosphate decarboxylase	0.805198269	2.112579913	4.044288643	0.52236131	0.601418789	0.996994191
5.1.3.24	N-acetylneuraminate epimerase	0.240767753	0.411045137	4.108600605	0.100045046	0.920308563	0.996994191
2.5.1.31	undecaprenyl diphosphate synthase	402.9684909	0.073010078	0.167279479	0.436455675	0.662506152	0.996994191
1.5.1.7	saccharopine dehydrogenase (NAD+, L-lysine forming)	270.5033177	-0.190570844	0.240397639	-0.79273176	0.427934122	0.996994191
3.4.19.1	acylaminoacyl-peptidase	1.683032235	2.499921647	1.402128468	1.782947642	0.074594827	0.996994191
3.1.3.73	alpha-ribazole phosphatase	202.4302529	-0.020639522	0.325513934	-0.063405955	0.949443245	0.996994191
2.1.1.166	23S rRNA (uridine2552-2'-O)-methyltransferase	12.94395647	-0.340366475	1.038724219	-0.327677423	0.743155576	0.996994191
1.6.5.2	NAD(P)H dehydrogenase (quinone)	9.363692982	-0.440223183	1.060512805	-0.415104072	0.678065719	0.996994191
3.4.21.110	C5a peptidase	0.866408451	1.306811926	4.105946382	0.318273013	0.750277853	0.996994191
4.7.1.1	alpha-D-ribose 1-methylphosphonate 5-phosphate C-P lyase	2.28894411	0.217164478	2.091563029	0.1038288	0.917305211	0.996994191
6.3.1.12	D-aspartate ligase	24.50587962	0.210607859	0.442634381	0.475805469	0.634212973	0.996994191
1.8.1.2	#N/A	12.775374	0.139351461	1.13174524	0.123129708	0.902004387	0.996994191
4.1.99.12	3,4-dihydroxy 2-butanone 4-phosphate synthase	318.5032203	0.392105614	0.239436978	1.637615115	0.101502008	0.996994191
4.1.2.14	2-dehydro-3-deoxyphosphogluconate aldolase / (4S)-4-hydroxy	365.4288842	0.448185666	0.288890054	1.551405662	0.120804499	0.996994191
5.4.99.27	tRNA pseudouridine13 synthase	2.224630463	4.243345426	2.732206403	1.553083772	0.120403122	0.996994191
1.12.99.6	#N/A	125.2879068	-0.079098715	0.531088271	-0.148937039	0.881603314	0.996994191
2.7.1.192	PTS system, N-acetylmuramic acid-specific IIB component	0.096307101	0.646944639	4.130400399	0.156630006	0.875536453	0.996994191
1.3.99.33	urocanate reductase	28.26826934	-1.493037043	0.97119632	-1.537317442	0.124215592	0.996994191
6.3.5.2	GMP synthase (glutamine-hydrolysing)	699.2410668	-0.032536801	0.187894238	-0.173165508	0.862521335	0.996994191
3.6.3.-	#N/A	1836.451269	-0.246698517	0.245482173	-1.004954915	0.31491856	0.996994191
2.3.1.76	diacylglycerol O-acyltransferase 1	0.015832449	1.463162531	4.140620044	0.353367978	0.723812589	0.996994191
3.1.6.8	arylsulfatase A	1.997616455	-2.656070318	3.47633087	-0.764044165	0.444840924	0.996994191
2.7.1.170	anhydro-N-acetylmuramic acid kinase	29.26470967	0.334702849	0.548506375	0.610207765	0.541724187	0.996994191
2.6.1.57	aromatic-amino-acid transaminase	12.44296424	-0.590800598	0.746660394	-0.791257449	0.428793774	0.996994191
1.3.99.-	#N/A	55.60821594	-0.57163439	0.425412452	-1.34371805	0.179039578	0.996994191
2.7.8.36	undecaprenyl phosphate N,N'-diacetylbacillosamine 1-phospha	4.593317519	-1.747416515	1.785431079	-0.978708467	0.327724047	0.996994191
5.3.1.16	phosphoribosylformimino-5-aminoimidazole carboxamide ribot	177.1832862	-0.208695223	0.287206314	-0.726638702	0.467447313	0.996994191
3.1.2.6	hydroxyacylglutathione hydrolase	1.655433542	0.644939721	1.990514522	0.324006539	0.745933084	0.996994191
1.15.1.1	nickel superoxide dismutase	641.8686433	0.789205642	0.546305394	1.444623558	0.148563652	0.996994191
3.5.4.43	hydroxydechloroatrazine ethylaminohydrolase	0.97091914	1.945334599	3.331687915	0.583888602	0.559295262	0.996994191
2.4.99.17	S-adenosylmethionine:tRNA ribosyltransferase-isomerase	404.1357236	-0.184875065	0.321904823	-0.574315921	0.565754037	0.996994191
4.2.2.3	poly(beta-D-mannuronate) lyase	3.76957877	-2.099028248	1.590295238	-1.319898468	0.186868919	0.996994191
6.3.2.8	UDP-N-acetylmuramate--alanine ligase	425.8810352	-0.225059424	0.147517351	-1.525647132	0.127097767	0.996994191
3.5.99.6	#N/A	840.5561841	0.122110522	0.364007098	0.335461926	0.737276661	0.996994191
2.3.1.39	acyl-carrier-protein] S-malonyltransferase	252.4509801	-0.376739105	0.259157785	-1.453705529	0.146027965	0.996994191
2.4.99.12	3-deoxy-D-manno-octulosonic-acid transferase	102.8745696	-0.356249249	0.359208676	-0.99176126	0.321313999	0.996994191
3.2.1.113	mannosyl-oligosaccharide alpha-1,2-mannosidase	1.197753478	4.540144879	4.057645831	1.11891108	0.263178076	0.996994191
3.1.3.36	inositol polyphosphate 5-phosphatase INPP5B/F	0.685511993	2.990803126	4.066095173	0.735546759	0.462006574	0.996994191
2.7.7.47	streptomycin 3''-adenylyltransferase	10.73053726	1.325645428	1.038713265	1.276238086	0.201871369	0.996994191
1.1.3.21	alpha-glycerophosphate oxidase	0.145244102	0.362563249	4.116607583	0.088073308	0.929818411	0.996994191
1.3.1.12	prephenate dehydrogenase	171.7607912	-0.070474368	0.317890091	-0.221694133	0.824551998	0.996994191
5.3.99.11	2-keto-myo-inositol isomerase	6.726854188	-0.791399542	0.69984396	-1.130822852	0.25812966	0.996994191
3.2.1.21	beta-glucosidase	1951.494518	-0.306135742	0.182064063	-1.681472652	0.092671144	0.996994191
1.5.5.2	RHH-type transcriptional regulator, proline utilization regulon re	58.38325883	0.101501695	0.492790752	0.205973213	0.836811835	0.996994191
2.1.2.9	methionyl-tRNA formyltransferase	378.080312	0.170364164	0.195506412	0.871399368	0.383536131	0.996994191
2.4.2.2	pyrimidine-nucleoside phosphorylase	232.276121	0.025186929	0.319501517	0.078831954	0.937166287	0.996994191
2.7.1.17	xylulokinase	428.2689433	-0.121647778	0.247119781	-0.492262405	0.622533853	0.996994191
2.7.7.13	mannose-1-phosphate guanylyltransferase	1345.206609	-1.181264109	0.90967677	-1.298553671	0.194097146	0.996994191
4.2.2.8	heparin/heparan-sulfate lyase	21.98860056	-1.311337225	0.839413671	-1.562206181	0.118239425	0.996994191
1.11.1.15	peroxiredoxin (alkyl hydroperoxide reductase subunit C)	879.9144678	0.740849732	0.514253228	1.440632148	0.149688633	0.996994191
3.5.4.12	dCMP deaminase	242.5971025	-0.137896966	0.2222261	-0.620525517	0.53491186	0.996994191
3.6.3.40	teichoic acid transport system ATP-binding protein	81.29592209	-0.655654143	0.41017101	-1.598489719	0.109934032	0.996994191
2.1.-.-	#N/A	0.192614202	0.463843892	4.112600639	0.112786028	0.910200195	0.996994191
3.1.11.5	exodeoxyribonuclease V	497.8412324	-0.090258213	0.273680491	-0.329794107	0.741555541	0.996994191
1.1.1.336	UDP-N-acetyl-D-mannosaminuronic acid dehydrogenase	33.03340853	0.080611977	0.469051244	0.171861769	0.863546203	0.996994191
1.14.13.-	#N/A	1.712504284	3.63364436	4.033361094	0.900897359	0.367642895	0.996994191
3.6.1.65	(d)CTP diphosphatase	0.422052821	0.697243081	4.083515653	0.170745784	0.864423661	0.996994191
1.2.1.81	sulfoacetaldehyde dehydrogenase	12.88492287	-1.69370349	1.326390743	-1.276926501	0.201628197	0.996994191
4.1.2.17	L-fuculose-phosphate aldolase	179.7216875	0.174626489	0.430726798	0.405422856	0.685166713	0.996994191
2.5.1.41	phosphoglycerol geranylgeranyltransferase	3.384201131	4.272306474	2.264799325	1.886395155	0.059241725	0.996994191
1.3.7.9	4-hydroxybenzoyl-CoA reductase subunit gamma	0.297488658	1.703030493	4.107535663	0.414611249	0.678426512	0.996994191
5.1.99.3	allantoin racemase	7.808605122	0.606761834	0.70856182	0.856328716	0.391815982	0.996994191
2.7.8.23	carboxyvinyl-carboxyphosphonate phosphorylmutase	0.047497346	1.429004528	4.143731151	0.344859373	0.730200094	0.996994191
1.1.1.205	IMP dehydrogenase	738.73706	-0.487126651	0.437815404	-1.112630222	0.265867275	0.996994191
2.4.1.325	dTDP-N-acetylfucosamine:lipid II N-acetylfucosaminyltransferas	0.215045196	0.437013907	4.110503157	0.106316402	0.915331318	0.996994191
2.1.3.11	N-succinyl-L-ornithine transcarbamylase	68.30817693	-0.219152696	0.439579749	-0.498550482	0.618096097	0.996994191
3.5.4.9	methylenetetrahydrofolate dehydrogenase (NADP+) / metheny	432.3435048	0.410837804	0.245749412	1.671775329	0.094568634	0.996994191
2.1.1.203	tRNA (cytosine34-C5)-methyltransferase	0.433524036	2.371776455	4.082145067	0.58101229	0.56123217	0.996994191
2.3.2.10	serine/alanine adding enzyme	1.782642193	2.257614947	2.209157115	1.021934987	0.306811672	0.996994191
4.1.2.50	6-pyruvoyltetrahydropterin/6-carboxytetrahydropterin synthas	76.58751425	0.393150996	0.404525038	0.971882971	0.331108772	0.996994191
2.7.1.49	hydroxymethylpyrimidine/phosphomethylpyrimidine kinase / t	174.8667027	-0.403986534	0.30214579	-1.337058291	0.18120361	0.996994191
3.6.-.-	#N/A	322.2767022	-0.089542859	0.26073914	-0.343419324	0.731283024	0.996994191
6.3.1.1	aspartate--ammonia ligase	184.1027838	-0.592418781	0.322806395	-1.835213896	0.066474003	0.996994191
2.1.1.298	ribosomal protein L3 glutamine methyltransferase	3.233325143	1.621114388	1.252898514	1.293891222	0.195702997	0.996994191
1.7.5.1	nitrate reductase / nitrite oxidoreductase, alpha subunit	5.245192299	-0.957182923	0.920757119	-1.039560709	0.298544039	0.996994191
1.1.1.14	L-iditol 2-dehydrogenase	109.28552	-0.248274262	0.373481458	-0.664756595	0.506206189	0.996994191

1.3.1.89	tRNA-dihydrouridine synthase 3	0.06939653	2.268391649	4.115092055	0.551237158	0.581471111	0.996994191
1.13.11.2	catechol 2,3-dioxygenase	0.859051352	2.289239342	4.050592255	0.565161635	0.571963829	0.996994191
4.3.1.2	methylaspartate ammonia-lyase	48.64895908	0.254475326	0.611493463	0.416153797	0.677297466	0.996994191
3.4.11.7	glutamyl aminopeptidase	1.448565954	0.718783201	3.116310719	0.230651968	0.817585191	0.996994191
3.5.1.98	histone deacetylase 1/2	0.803461216	4.772795194	4.059016084	1.175850279	0.239654711	0.996994191
5.1.3.6	UDP-glucuronate 4-epimerase	31.56312131	0.267308195	0.659727948	0.405179431	0.685345623	0.996994191
3.1.26.12	ribonuclease E	25.32080082	-0.592074645	0.713000354	-0.830398809	0.406313339	0.996994191
5.4.99.58	methylornithine synthase	0.369327113	-0.391110649	4.12407987	-0.094835857	0.924445205	0.996994191
3.2.1.33	glycogen debranching enzyme	0.160692243	3.261377394	4.083493213	0.798673397	0.424479817	0.996994191
3.2.1.14	chitinase	63.36207878	0.580905095	0.367454508	1.580889833	0.113903229	0.996994191
1.1.3.-	#N/A	0.215045196	0.437013907	4.110503157	0.106316402	0.915331318	0.996994191
3.5.1.91	N-substituted formamide deformylase	117.1158786	-0.031905708	0.468699753	-0.068072807	0.945727677	0.996994191
3.1.1.3	triacylglycerol lipase	15.45567411	0.499206611	0.74022403	0.674399358	0.500057451	0.996994191
3.5.1.32	hippurate hydrolase	17.82442116	0.166070293	0.724938331	0.229081959	0.818805215	0.996994191
3.5.1.9	arylformamidase	22.52921188	-0.427865429	0.836644947	-0.511406219	0.609066641	0.996994191
3.1.13.4	poly(A)-specific ribonuclease	0.107128162	2.905607862	4.094274383	0.709675901	0.47790514	0.996994191
1.3.99.29	phytoene desaturase	1.632348624	3.119400767	4.065906252	0.767209221	0.442957131	0.996994191
3.5.2.18	enamidase	9.33551453	-0.095386016	1.340701207	-0.071146364	0.943281269	0.996994191
2.7.1.55	allose kinase	2.123922768	0.792159023	1.783902313	0.444059642	0.656999464	0.996994191
4.1.1.19	arginine decarboxylase	193.6903894	0.14917711	0.365187037	0.408495086	0.682910238	0.996994191
2.3.1.61	2-oxoglutarate dehydrogenase E2 component (dihydrolipoamid	48.87361501	0.543823288	0.546045909	0.995929608	0.319284348	0.996994191
6.1.1.5	isoleucyl-tRNA synthetase	902.9590698	0.093784387	0.11737715	0.799000381	0.424290191	0.996994191
3.1.1.17	gluconolactonase	1.652406123	2.360222732	3.392747209	0.695667135	0.486637312	0.996994191
4.1.2.43	3-hexulose-6-phosphate synthase	16.96607242	0.541545958	0.832386838	0.650594091	0.515308546	0.996994191
4.2.1.80	2-keto-4-pentenoate hydratase	0.627092025	-1.351582328	4.098371645	-0.329785204	0.741562268	0.996994191
2.1.1.245	acetyl-CoA decarbonylase/synthase complex subunit delta	937.0964197	0.058815545	0.669682443	0.087826022	0.930014955	0.996994191
3.5.3.11	agmatinase	88.61984129	-0.133945248	0.359700031	-0.372380419	0.70960963	0.996994191
6.6.1.2	cobaltochelatase CobN	201.6340406	0.540931344	0.446062852	1.212679651	0.225252323	0.996994191
3.5.-.-	#N/A	18.06841165	0.444438969	0.723455451	0.61432804	0.538998577	0.996994191
3.7.1.20	fumarylpyruvate hydrolase	3.078512959	0.7800786	1.824291763	0.427606272	0.668937799	0.996994191
2.7.8.28	LPPG:FO 2-phospho-L-lactate transferase	4.388340055	5.264050597	4.0306012	1.306021195	0.19154535	0.996994191
1.1.1.85	3-isopropylmalate dehydrogenase	273.3671789	-0.15517165	0.212330014	-0.730804122	0.464898805	0.996994191
3.2.1.122	maltose-6'-phosphate glucosidase	25.24441003	-1.020141308	0.697576417	-1.462407965	0.143629448	0.996994191
2.1.2.3	phosphoribosylaminoimidazolecarboxamide formyltransferase	631.120063	-0.0863833	0.224245093	-0.385218238	0.700075732	0.996994191
2.7.-.-	#N/A	135.2697332	-0.453970935	0.480500233	-0.944788168	0.344767034	0.996994191
3.2.1.89	arabinogalactan endo-1,4-beta-galactosidase	114.6560584	-1.01038522	0.466281843	-2.166898056	0.030242628	0.996994191
2.8.3.8	#N/A	156.8950941	0.163957116	0.612415776	0.267721902	0.78891339	0.996994191
2.1.1.271	precorrin-4/cobalt-precorrin-4 C11-methyltransferase	110.7485461	-0.51492011	0.384611301	-1.338806502	0.180633674	0.996994191
1.8.4.-	#N/A	1.874139009	0.757509036	2.174578759	0.348347482	0.727579238	0.996994191
2.4.2.9	uracil phosphoribosyltransferase	463.3200667	-0.083194295	0.241713369	-0.344185743	0.730706604	0.996994191
3.4.-.-	#N/A	2017.633239	0.146794801	0.128253316	1.144569243	0.252387639	0.996994191
4.1.1.44	4-carboxymuconolactone decarboxylase	3240.638862	-1.870367388	1.471333142	-1.271205912	0.203655404	0.996994191
3.1.1.73	feruloyl esterase	0.400658548	-2.750526432	4.100705394	-0.670744705	0.502383178	0.996994191
1.14.11.17	taurine dioxygenase	0.127261092	0.745319789	4.135512686	0.180224278	0.856976499	0.996994191
2.1.1.130	#N/A	67.37692647	-0.575953911	0.560182948	-1.028153237	0.303877752	0.996994191
4.1.1.12	aspartate 4-decarboxylase	35.20555678	-0.430296583	0.566435517	-0.759656783	0.447459768	0.996994191
1.14.17.3	peptidylglycine monooxygenase	0.104862237	0.633124411	4.128595819	0.153351028	0.878121462	0.996994191
1.7.2.3	trimethylamine-N-oxide reductase (cytochrome c)	14.90176483	0.864368579	1.013953367	0.852473701	0.393951229	0.996994191
2.10.1.1	molybdopterin molybdotransferase	124.0538972	-0.458453075	0.452570475	-1.012998198	0.311061022	0.996994191
1.4.3.-	#N/A	1.495574844	1.675397617	3.444857775	0.486347398	0.626720879	0.996994191
2.5.1.19	3-phosphoshikimate 1-carboxyvinyltransferase	330.9966198	-0.144486755	0.296487835	-0.48732777	0.626026071	0.996994191
2.1.3.2	aspartate carbamoyltransferase	339.3596569	-0.056615373	0.219895234	-0.25746521	0.796819669	0.996994191
3.6.5.-	#N/A	484.9955167	0.296826112	0.204248218	1.453261699	0.146151107	0.996994191
3.5.1.78	glutathionylspermidine amidase/synthetase	1.535270693	1.883074867	3.043164053	0.618788483	0.536055711	0.996994191
3.6.3.28	#N/A	16.58175273	-0.818185055	0.839708344	-0.974368137	0.329873784	0.996994191
3.5.1.4	amidase	13.39788218	-0.308153213	0.915106628	-0.336740226	0.736312737	0.996994191
4.3.1.24	phenylalanine ammonia-lyase	0.052431119	0.78195638	4.139114155	0.188918776	0.850156477	0.996994191
6.3.5.1	NAD+ synthase (glutamine-hydrolysing)	1103.455858	-0.687406719	0.628809049	-1.093188337	0.274311117	0.996994191
6.2.1.13	acetate---CoA ligase (ADP-forming) subunit alpha	38.89400474	-0.054031584	0.904138392	-0.059760303	0.952346543	0.996994191
1.2.1.27	malonate-semialdehyde dehydrogenase (acetylating) / methylr	18.30084634	0.099149572	0.539870965	0.183654203	0.854284738	0.996994191
2.3.3.9	malate synthase	4.623532929	-0.349547928	1.978365561	-0.176685207	0.859755662	0.996994191
1.5.99.15	dihydromethanopterin reductase (acceptor)	3.714982287	3.381124196	2.609415574	1.295740023	0.195065068	0.996994191
5.3.1.30	5-deoxy-glucuronate isomerase	37.58263163	0.682551254	0.55368348	1.232746287	0.217670437	0.996994191
3.2.1.133	cyclomaltodextrinase / maltogenic alpha-amylase / neopullular	0.261732976	1.164912141	4.105588734	0.283738147	0.776611058	0.996994191
1.5.3.22	coenzyme F420H2 oxidase	43.21632078	2.032189209	1.067300601	1.904045784	0.056904222	0.996994191
1.17.4.2	ribonucleoside-triphosphate reductase (thioredoxin)	1317.312582	0.284996911	0.186290657	1.529850802	0.126053663	0.996994191
2.7.2.11	glutamate 5-kinase	251.4337639	-0.109491453	0.241753416	-0.452905504	0.650616787	0.996994191
2.7.8.8	CDP-diacylglycerol---serine O-phosphatidyltransferase	109.1044487	-0.187717207	0.444539611	-0.422273297	0.672825546	0.996994191
2.4.1.208	1,2-diacylglycerol-3-alpha-glucose alpha-1,2-glucosyltransferase	21.76042831	-1.129612085	0.659376863	-1.713150927	0.086684777	0.996994191
2.7.1.138	ceramide kinase	0.022430994	1.099249132	4.140903343	0.265461191	0.790654203	0.996994191
6.1.1.7	alanyl-tRNA synthetase	1078.886596	0.198946452	0.20673019	0.962348325	0.335874662	0.996994191
2.6.1.52	phosphoserine aminotransferase	504.7947067	0.150272188	0.242518714	0.619631308	0.53550055	0.996994191
4.99.1.3	sirohydrochlorin cobaltochelatase	136.2095742	0.13714654	0.345465276	0.396990811	0.691374237	0.996994191
3.1.1.61	two-component system, chemotaxis family, protein-glutamate	143.0684675	-0.278158613	0.562192101	-0.494775028	0.620758933	0.996994191
3.5.1.16	acetylornithine deacetylase	66.04934801	-0.104397197	0.30884699	-0.338022386	0.735346318	0.996994191
1.1.1.202	1,3-propanediol dehydrogenase	0.419812109	1.010463801	4.089827622	0.247067577	0.804855926	0.996994191
1.20.1.1	phosphonate dehydrogenase	0.021925243	1.675481703	4.126913301	0.405989072	0.68475063	0.996994191
2.3.1.234	N6-L-threonylcarbamoyladenine synthase	411.5437197	-0.136766911	0.213594293	-0.640311635	0.521970018	0.996994191
4.1.99.22	GTP 3',8-cyclase	176.3824341	-0.222814943	0.502761373	-0.443182303	0.657633878	0.996994191
1.4.1.3	glutamate dehydrogenase (NAD(P)+)	8.230057854	-1.242262856	1.088673264	-1.141079603	0.253836798	0.996994191
2.6.1.97	archaeosine synthase	3.338260327	3.282330353	2.579149157	1.272640764	0.203145547	0.996994191
2.1.1.193	16S rRNA (uracil1498-N3)-methyltransferase	240.5805697	0.106336309	0.255768125	0.415752779	0.677590916	0.996994191
1.8.7.1	sulfite reductase (ferredoxin)	0.015832449	1.463162531	4.140620044	0.353367978	0.723812589	0.996994191
3.4.22.1	cathepsin B	8.888996236	2.252807282	3.587780614	0.627911103	0.530062177	0.996994191
4.2.1.20	tryptophan synthase	461.3372891	-0.076882135	0.29338534	-0.262051726	0.793281563	0.996994191
1.1.1.396	bacteriochlorophyllide a dehydrogenase	0.057984819	1.314825092	4.126913272	0.318597704	0.750031595	0.996994191
1.1.2.3	L-lactate dehydrogenase (cytochrome)	0.301644209	0.522005818	4.107735279	0.127078739	0.898878079	0.996994191

2.7.11.17	calcium/calmodulin-dependent protein kinase kinase 1	0.589204892	4.473992392	4.061992578	1.101428008	0.270710421	0.996994191
2.3.1.210	dTDP-4-amino-4,6-dideoxy-D-galactose acyltransferase	0.095650897	1.135554362	4.127736264	0.27510342	0.783236784	0.996994191
2.1.1.221	tRNA (guanine9-N1)-methyltransferase	0.160692243	3.261377394	4.083493213	0.798673397	0.424479817	0.996994191
2.3.1.202	UDP-4-amino-4,6-dideoxy-N-acetyl-beta-L-altrosamine N-acety	0.222361558	3.484635609	4.081802081	0.853700287	0.393271075	0.996994191
3.4.13.-	#N/A	532.3021501	0.193989036	0.192093225	1.009869226	0.312557948	0.996994191
3.4.24.64	mitochondrial-processing peptidase subunit alpha	0.167740794	1.579265648	4.131951497	0.382208177	0.702306957	0.996994191
2.7.7.73	sulfur carrier protein ThiS adenylyltransferase	10.45724072	-0.180033144	1.000603424	-0.179924573	0.857211784	0.996994191
2.8.1.1	#N/A	6.634840969	-0.616597172	0.981033817	-0.628517755	0.529664819	0.996994191
3.4.21.107	serine protease Do	563.0586176	-0.410663499	0.420439714	-0.976747643	0.328694102	0.996994191
3.6.3.6	#N/A	2.40170059	-1.346263121	2.72583373	-0.493890404	0.621383582	0.996994191
3.4.17.19	carboxypeptidase Taq	88.78305047	0.222197976	0.398612923	0.55742793	0.577235087	0.996994191
3.1.26.4	ribonuclease HI	787.2753457	-0.656664685	0.510062594	-1.287419803	0.197948006	0.996994191
1.1.1.103	threonine 3-dehydrogenase	187.2651899	-0.309132025	0.688339473	-0.449098209	0.653360812	0.996994191
1.8.4.2	protein-disulfide reductase (glutathione)	1.392666108	5.282452246	4.05478875	1.302768793	0.192653708	0.996994191
2.1.1.79	cyclopropane-fatty-acyl-phospholipid synthase	13.91181454	-0.913902796	0.796858893	-1.146881592	0.251430562	0.996994191
5.4.99.23	23S rRNA pseudouridine1911/1915/1917 synthase	11541.25338	-1.821179185	1.181989723	-1.540774129	0.123371768	0.996994191
2.5.1.131	(4-(4-[2-(gamma-L-glutamylamino)ethyl]phenoxymethyl)furan-:	2.497929715	3.423737523	2.337191329	1.464893986	0.142949844	0.996994191
1.2.7.1	#N/A	6286.64664	-0.035378134	0.508073487	-0.069631924	0.944486627	0.996994191
2.1.1.248	methylamine---corrinoid protein Co-methyltransferase	3.097301404	0.660409847	2.084925919	0.316754586	0.751429826	0.996994191
2.7.13.3	two-component system, sensor histidine kinase PdtaS	2106.00677	0.093124275	0.13395685	0.695181133	0.486941795	0.996994191
2.8.3.19	CoA:oxalate CoA-transferase	4.085827508	0.638255451	1.172383845	0.544408262	0.586160562	0.996994191
2.7.1.207	PTS system, lactose-specific IIA component	3.660562079	-1.120186189	1.54894296	-0.723193958	0.469560731	0.996994191
2.7.1.2	glucokinase	766.2669656	0.085406095	0.235058878	0.363339159	0.716351544	0.996994191
2.7.7.58	#N/A	1.219326878	0.749716442	3.128098464	0.239671625	0.810584834	0.996994191
6.3.2.2	glutamate--cysteine ligase	16.25644899	-1.342021327	0.683886764	-1.962344349	0.049722407	0.996994191
6.3.4.5	argininosuccinate synthase	353.5266071	0.24295375	0.24657177	0.985326705	0.324463619	0.996994191
3.5.1.118	gamma-glutamyl hercynylcysteine S-oxide hydrolase	0.788341192	1.111586781	3.608381251	0.308056911	0.758039028	0.996994191
1.5.-.-	#N/A	9.289395333	-0.410722171	0.760092637	-0.540358044	0.588950136	0.996994191
3.4.19.13	gamma-glutamyltranspeptidase / glutathione hydrolase	29.91949374	0.651857483	0.607836016	1.072423262	0.283529964	0.996994191
2.7.7.43	N-acylneuraminate cytidylyltransferase	24.55902711	-0.654238568	0.607293235	-1.077302578	0.281345111	0.996994191
2.7.1.67	phosphatidylinositol 4-kinase A	0.208380513	2.859493603	4.079916125	0.700870684	0.483383721	0.996994191
1.1.1.35	3-hydroxyacyl-CoA dehydrogenase	2.996316731	0.797362353	1.265020043	0.630315984	0.528487867	0.996994191
2.8.4.4	ribosomal protein S12 methylthiotransferase	617.9371104	0.107589415	0.211680608	0.508262973	0.611268933	0.996994191
1.1.1.58	tagaturonate reductase	366.6022532	0.030858045	0.439140669	0.070269158	0.94397943	0.996994191
2.2.1.1	transketolase	1674.932167	0.172184207	0.279647539	0.615718659	0.53808022	0.996994191
2.6.1.76	diaminobutyrate-2-oxoglutarate transaminase	23.46744053	2.586012001	1.068902625	2.419314857	0.015549774	0.996994191
3.4.24.40	serralysin	0.216806498	1.752853521	4.123668648	0.42507138	0.670784641	0.996994191
2.8.3.16	formyl-CoA transferase	5.93634884	1.473855064	1.135712546	1.297736006	0.194378066	0.996994191
3.1.3.97	3',5'-nucleoside bisphosphate phosphatase	375.6107677	0.237053052	0.197409343	1.200819821	0.229821101	0.996994191
4.2.1.90	L-rhamnonate dehydratase	0.858938804	0.979207393	4.095538744	0.239091229	0.811034843	0.996994191
3.6.1.13	ADP-ribose pyrophosphatase	194.8902596	0.046865916	0.271147141	0.172843114	0.862774747	0.996994191
1.1.-.-	#N/A	83.0482385	0.08099399	0.310720106	0.260665429	0.794350528	0.996994191
3.1.1.11	pectinesterase	182.2115839	0.143199008	0.41989426	0.341035879	0.733076571	0.996994191
4.1.1.81	threonine-phosphate decarboxylase	126.0137083	-0.162290938	0.322382517	-0.503411101	0.614675269	0.996994191
3.6.3.54	copper-transporting P-type ATPase V	207.7805605	0.096561758	0.367135862	0.26301369	0.792540028	0.996994191
3.5.4.28	5-methylthioadenosine/S-adenosylhomocysteine deaminase	210.6530955	-0.33048386	0.516834951	-0.639437909	0.522538094	0.996994191
1.6.5.8	#N/A	454.6626916	-0.082825221	0.321385888	-0.257712689	0.796628653	0.996994191
5.3.2.6	4-oxalocrotonate tautomerase	11.37995625	-0.142343997	0.894954395	-0.159051676	0.87362816	0.996994191
1.2.4.1	pyruvate dehydrogenase (acetyl-transferring)	62.46924544	0.129610752	0.73661753	0.175953934	0.860330133	0.996994191
2.7.1.86	NADH kinase	0.160692243	3.261377394	4.083493213	0.798673397	0.424479817	0.996994191
5.1.99.8	7,8-dihydroneopterin aldolase/epimerase/oxygenase	48.1069637	-0.081488643	0.432298486	-0.188500876	0.850484029	0.996994191
1.1.1.37	malate dehydrogenase	317.6601585	0.068432935	0.405327507	0.168833681	0.865927465	0.996994191
1.13.99.1	inositol oxygenase	0.504441937	1.20832257	4.136106318	0.292140114	0.770179497	0.996994191
2.7.1.197	PTS system, mannitol-specific IIA component	45.44114152	-0.39869119	0.520040908	-0.766653515	0.443287548	0.996994191
3.6.1.7	acylphosphatase	87.39297953	-0.481635746	0.365795399	-1.316680713	0.187945671	0.996994191
2.1.1.220	tRNA (adenine57-N1/adenine58-N1)-methyltransferase catalyti	4.213074941	2.811992248	1.750885594	1.606039971	0.108265128	0.996994191
3.4.22.48	staphopain A	0.269151628	1.568765174	4.12621369	0.380194845	0.703800786	0.996994191
2.7.4.22	uridylate kinase	553.4700535	0.134182593	0.305018229	0.439916636	0.659997487	0.996994191
1.1.1.22	UDPglucose 6-dehydrogenase	338.3428138	0.427423187	0.225144878	1.898436207	0.057638644	0.996994191
3.4.19.-	#N/A	67.95115075	-0.103410189	0.347869208	-0.297267441	0.766262337	0.996994191
3.2.1.184	GDP/UDP-N,N'-diacetylbacillosamine 2-epimerase (hydrolysing)	11.03001939	0.880222452	0.706996553	1.24501661	0.213125645	0.996994191
3.4.22.37	gingipain R	506.8117507	1.873880805	1.397422447	1.340955134	0.17993502	0.996994191
6.3.5.6	aspartyl-tRNA(Asn)/glutamyl-tRNA(Gln) amidotransferase subu	502.939812	-0.036423133	0.300620643	-0.121159788	0.903564474	0.996994191
4.1.1.4	acetoacetate decarboxylase	0.721647054	0.439993933	4.09632712	0.107411816	0.914462281	0.996994191
4.6.1.13	1-phosphatidylinositol phosphodiesterase	8.638312496	-0.582360718	0.881338703	-0.660768348	0.508760885	0.996994191
3.4.11.6	aminopeptidase B	5.455731727	0.449390871	1.371952424	0.327555725	0.743247603	0.996994191
3.2.2.21	DNA-3-methyladenine glycosylase II	48.22412736	-0.184266062	0.599129397	-0.307556369	0.758419924	0.996994191
2.5.1.61	hydroxymethylbilane synthase	69.60118893	0.676034983	0.366082916	1.846671763	0.064794727	0.996994191
6.1.1.13	D-alanine--poly(phosphoribitol) ligase subunit 1	8.573527878	-1.721374959	1.02873071	-1.673299866	0.094268279	0.996994191
4.1.1.20	diaminopimelate decarboxylase	367.9980612	-0.236274403	0.20483722	-1.153473981	0.248715891	0.996994191
6.3.4.4	adenylosuccinate synthase	669.1225565	-0.069545037	0.189296166	-0.367387457	0.713330027	0.996994191
3.6.5.4	signal recognition particle subunit SRP54	540.0164761	-0.048774831	0.267197335	-0.182542356	0.855157118	0.996994191
1.1.1.132	GDP-mannose 6-dehydrogenase	7.106379203	-0.263375523	1.418597199	-0.185659131	0.85271208	0.996994191
5.3.1.5	xylose isomerase	118.9131173	-0.090607174	0.487833976	-0.185733628	0.852653656	0.996994191
4.2.2.23	rhamnogalacturonan endolyase	106.7907997	0.48194137	0.390459991	1.234291301	0.217094376	0.996994191
4.1.1.79	sulfopyruvate decarboxylase subunit alpha	1.162573941	2.730313376	4.088923433	0.667734043	0.504303375	0.996994191
1.17.7.4	4-hydroxy-3-methylbut-2-en-1-yl diphosphate reductase	354.784172	-0.221135871	0.429306676	-0.515100004	0.60648314	0.996994191
4.1.1.74	indolepyruvate decarboxylase	0.392755038	1.472809431	4.095046262	0.35965636	0.719104131	0.996994191
4.2.2.7	heparin lyase	8.270872475	-0.072783149	0.983413647	-0.074010717	0.941001858	0.996994191
4.2.1.19	imidazoleglycerol-phosphate dehydratase / histidinol-phosphat	197.1721382	0.197898239	0.264086354	0.749369423	0.453634575	0.996994191
6.3.-.-	#N/A	0.169214877	0.921554111	4.118240387	0.223773754	0.822933353	0.996994191
2.7.12.2	mitogen-activated protein kinase kinase 1	0.114087933	2.094818269	4.119178137	0.508552483	0.611065943	0.996994191
2.7.1.107	diacylglycerol kinase (ATP)	64.69343955	-0.491755138	0.494259377	-0.994933351	0.319768681	0.996994191
5.1.3.14	UDP-N-acetylglucosamine 2-epimerase (non-hydrolysing)	254.2481963	0.126695864	0.248843353	0.509139032	0.610654775	0.996994191
3.4.11.-	#N/A	191.0081273	-0.45844784	0.369415389	-1.241009047	0.214602407	0.996994191
3.1.3.89	5'-deoxynucleotidase	102.4893594	1.050403845	0.543224811	1.933644827	0.053156812	0.996994191
4.1.1.41	#N/A	0.335113387	2.541563563	4.081531325	0.622698532	0.533482648	0.996994191

3.1.3.57	inositol polyphosphate 1-phosphatase	0.160692243	3.261377394	4.083493213	0.798673397	0.424479817	0.996994191
2.4.1.18	1,4-alpha-glucan branching enzyme	1270.326465	-0.046049601	0.25943643	-0.177498591	0.859116772	0.996994191
4.1.1.37	uroporphyrinogen decarboxylase	71.82733174	-0.299716423	0.387159416	-0.774142151	0.438846733	0.996994191
3.6.1.61	diadenosine hexaphosphate hydrolase (ATP-forming)	0.213748207	0.924193779	4.110332311	0.224846487	0.822098701	0.996994191
2.7.1.195	PTS system, 2-O-A-mannosyl-D-glycerate-specific IIA componer	0.096307101	0.646944639	4.130400399	0.156630006	0.875536453	0.996994191
1.11.1.5	cytochrome c peroxidase	29.69912474	-0.460347316	0.562654535	-0.818170454	0.413259864	0.996994191
5.1.1.20	L-Ala-D/L-Glu epimerase	82.99796834	0.325788348	0.397707251	0.819166227	0.412691581	0.996994191
2.6.1.109	8-amino-3,8-dideoxy-alpha-D-manno-octulosonate transaminas	0.169128288	0.778055416	4.111898369	0.189220488	0.849920011	0.996994191
3.5.99.2	hydroxymethylpyrimidine/phosphomethylpyrimidine kinase / t	34.47460786	-0.635543424	0.685274712	-0.927428684	0.353704004	0.996994191
3.2.1.86	#N/A	335.5730526	-0.695528518	0.297154805	-2.340626858	0.019251397	0.996994191
6.3.1.-	#N/A	1.210030324	1.273355091	3.195814969	0.398444561	0.690302517	0.996994191
2.7.4.3	adenylate kinase	907.9785391	0.195768607	0.403322292	0.485389999	0.627399725	0.996994191
2.4.1.247	beta-D-galactosyl-(1->4)-L-rhamnose phosphorylase	6.615225414	-0.543351902	1.151658897	-0.471799336	0.637070025	0.996994191
6.1.1.18	glutaminyl-tRNA synthetase	464.0232608	-0.149612355	0.154907821	-0.965815376	0.334136573	0.996994191
1.1.5.3	glycerol-3-phosphate dehydrogenase	521.1265768	0.559049437	0.358245547	1.560520265	0.118636985	0.996994191
2.3.1.247	3-keto-5-aminohexanoate cleavage enzyme	56.37761532	-0.192859337	0.49367143	-0.390663355	0.696046089	0.996994191
1.1.1.380	L-gulonate 5-dehydrogenase	19.37042004	-0.893160445	0.830489922	-1.075462111	0.282167887	0.996994191
1.1.1.262	4-hydroxythreonine-4-phosphate dehydrogenase	221.6681368	0.573775478	0.264989658	2.165274985	0.030366633	0.996994191
2.1.2.2	phosphoribosylglycinamide formyltransferase	478.4970592	0.032916044	0.225128893	0.14620977	0.883755793	0.996994191
2.7.7.68	2-phospho-L-lactate guanylyltransferase	2.105625982	1.805672935	2.874771386	0.628110098	0.529931819	0.996994191
2.1.1.131	precorrin-3B C17-methyltransferase	112.1616963	-0.398049341	0.401633257	-0.991076645	0.321648156	0.996994191
2.3.1.35	glutamate N-acetyltransferase / amino-acid N-acetyltransferase	232.9759184	-0.072292719	0.326812492	-0.221205494	0.824932432	0.996994191
2.7.1.151	#N/A	0.160692243	3.261377394	4.083493213	0.798673397	0.424479817	0.996994191
2.3.1.12	pyruvate dehydrogenase E2 component (dihydrolipoamide acet	60.27244184	0.261233524	0.639621976	0.408418619	0.682966366	0.996994191
4.1.2.13	fructose 1,6-bisphosphate aldolase/phosphatase	1537.630637	0.417529255	0.435997321	0.957641789	0.338243419	0.996994191
2.4.1.186	glycogenin	0.213123362	2.913385076	4.068639398	0.716058807	0.473955038	0.996994191
3.1.3.81	phosphatidylglycerophosphatase B	0.21899488	1.960227515	4.117804827	0.476037014	0.634048008	0.996994191
5.3.3.3	4-hydroxybutyryl-CoA dehydratase / vinylacetyl-CoA-Delta-ison	61.11807847	0.380863283	0.437490546	0.87056346	0.383992555	0.996994191
1.2.1.21	lactaldehyde dehydrogenase / glycolaldehyde dehydrogenase	34.86369268	-0.130883222	0.453037188	-0.288901719	0.772656588	0.996994191
3.6.3.41	heme exporter protein A	3.354275479	0.460513625	1.029327316	0.447392796	0.654591477	0.996994191
2.7.10.-	#N/A	4.805320692	-1.657087418	1.474943367	-1.123492233	0.261228497	0.996994191
1.1.1.385	dihydroanticapsin dehydrogenase	0.063329795	1.458576194	4.141019462	0.352226356	0.724668513	0.996994191
5.3.1.24	phosphoribosylanthranilate isomerase	94.20396798	-0.581970534	0.457702288	-1.271504533	0.203549216	0.996994191
5.4.99.18	5-(carboxyamino)imidazole ribonucleotide mutase	311.3499985	0.091194411	0.212230806	0.429694502	0.667417883	0.996994191
2.6.1.36	L-lysine 6-transaminase	1.472039473	1.257478674	4.087158509	0.307665746	0.758336687	0.996994191
2.5.1.-	#N/A	211.8230542	0.358698323	0.673351502	0.532705908	0.594237176	0.996994191
4.2.1.47	GDPmannose 4,6-dehydratase	174.6487184	0.239770766	0.26516447	0.904234138	0.365871262	0.996994191
1.7.1.13	7-cyano-7-deazaguanine reductase	52.99814833	-0.039669107	0.455850471	-0.087022292	0.930653872	0.996994191
6.1.1.1	tyrosyl-tRNA synthetase	382.8689634	0.419373163	0.244756262	1.713431799	0.08663313	0.996994191
4.2.1.33	3-isopropylmalate dehydratase	629.01279	-0.144553666	0.24812134	-0.582592638	0.560167562	0.996994191
4.1.1.68	#N/A	0.107481033	1.349360801	4.124476657	0.327159277	0.74354742	0.996994191
3.4.11.23	PepB aminopeptidase	1.373859969	7.073712893	4.038799909	1.751439302	0.079870268	0.996994191
3.1.3.1	alkaline phosphatase	161.6018233	0.02653869	0.416901096	0.06365704	0.949243312	0.996994191
2.7.8.26	adenosylcobinamide-GDP ribazoletransferase	150.8851714	0.11320453	0.385085332	0.293972584	0.768778853	0.996994191
1.1.3.15	glycolate oxidase	192.7034939	0.143717779	0.415543503	0.345854954	0.729451724	0.996994191
6.3.2.36	4-phosphopantoate---beta-alanine ligase	4.726773866	2.19109629	2.233404711	0.981056537	0.326564862	0.996994191
3.1.3.16	protein phosphatase	400.4206521	0.086810962	0.284505524	0.305129269	0.7602677	0.996994191
1.4.1.16	diaminopimelate dehydrogenase	159.4748883	-0.03410638	0.229908918	-0.148347354	0.882068646	0.996994191
3.2.1.67	galacturan 1,4-alpha-galacturonidase	0.121388873	0.904802081	4.119846113	0.219620359	0.826166835	0.996994191
2.4.1.337	1,2-diacylglycerol 3-alpha-glucosyltransferase	63.56108641	-0.030758026	0.519339474	-0.05922528	0.952772674	0.996994191
3.1.3.5	5'-nucleotidase	331.4545819	0.582987435	0.335574648	1.737280924	0.082337597	0.996994191
2.7.9.3	selenide, water dikinase	145.2147763	-0.419036645	0.288484105	-1.452546735	0.146349644	0.996994191
3.2.1.52	beta-N-acetylhexosaminidase	1287.687515	-0.265274524	0.282648085	-0.938532891	0.347970622	0.996994191
3.1.1.45	carboxymethylenebutenolidase	0.264436359	3.427376895	4.076086	0.840849995	0.400431975	0.996994191
3.6.3.27	#N/A	156.9113029	-0.67227231	0.56307862	-1.193922636	0.232508232	0.996994191
5.4.2.10	phosphoglucosamine mutase	560.1447399	0.029731493	0.254598417	0.116777996	0.907035981	0.996994191
3.8.1.5	haloalkane dehalogenase	0.20978502	1.170504523	4.113805192	0.284530858	0.776003589	0.996994191
6.5.1.4	RNA 3'-terminal phosphate cyclase (ATP)	0.104522907	1.107844965	4.110295616	0.269529267	0.787522423	0.996994191
3.4.21.89	signal peptidase I	631.9471178	-0.045954686	0.229945556	-0.199850291	0.841597668	0.996994191
2.7.11.24	mitogen-activated protein kinase 1/3	0.48207673	4.280913035	4.064288756	1.053299431	0.29220378	0.996994191
3.6.1.66	XTP/dITP diphosphohydrolase	461.2721418	-0.053934257	0.298272355	-0.180822178	0.856507154	0.996994191
4.1.3.36	naphthoate synthase	46.53090059	0.032918325	0.393348121	0.08368751	0.933304888	0.996994191
2.1.1.172	16S rRNA (guanine1207-N2)-methyltransferase	12.66763079	3.359929881	1.474841219	2.278163803	0.02271682	0.996994191
4.1.2.29	6-phospho-5-dehydro-2-deoxy-D-gluconate aldolase	0.107128162	2.905607862	4.094274383	0.709675901	0.47790514	0.996994191
2.7.11.1	checkpoint serine/threonine-protein kinase	1213.893284	0.083164464	0.134905205	0.616465937	0.537587046	0.996994191
2.4.1.289	N-acetylglucosaminyl-diphospho-decaprenol L-rhamnosyltransf	0.047497346	1.429004528	4.143731151	0.344859373	0.730200094	0.996994191
3.6.3.34	iron complex transport system ATP-binding protein	275.8948902	-0.285897383	0.349994266	-0.816863047	0.414006697	0.996994191
2.5.1.6	S-adenosylmethionine synthetase	730.5001727	0.56815615	0.251951008	2.255026307	0.024131678	0.996994191
2.5.1.54	3-deoxy-7-phosphoheptulonate synthase	876.9998298	-1.86266396	0.831360552	-2.240500774	0.025058431	0.996994191
2.7.7.8	polyribonucleotide nucleotidyltransferase	1148.232146	0.197443305	0.255846391	0.771725972	0.440276745	0.996994191
3.5.4.5	cytidine deaminase	265.2143101	0.036192981	0.308279459	0.117403155	0.906540584	0.996994191
5.5.1.27	D-galactarolactone cycloisomerase	0.264306213	1.359216313	4.089470425	0.332369762	0.739610068	0.996994191
3.2.1.25	beta-mannosidase	250.3589538	-0.217568115	0.232905077	-0.934149301	0.350226884	0.996994191
2.1.1.148	thymidylate synthase (FAD)	154.5213535	0.457331948	0.246794295	1.853089624	0.063869514	0.996994191
4.2.1.140	gluconate/galactonate dehydratase	0.079162244	1.485225644	4.138299125	0.358897605	0.719671691	0.996994191
3.6.3.2	Mg2+-importing ATPase	72.70340602	-0.581083504	0.586092534	-0.991453516	0.321464179	0.996994191
4.1.1.70	glutaconyl-CoA decarboxylase	95.93604748	-0.140024857	0.567388486	-0.24678833	0.805072042	0.996994191
2.7.4.9	dTMP kinase	187.7621884	0.598686951	0.287607038	2.0816144	0.037377705	0.996994191
2.7.1.176	UDP-N-acetylglucosamine kinase	4.654161857	0.532433508	1.126407634	0.472682795	0.636439503	0.996994191
2.4.1.291	N-acetylgalactosamine-N,N'-diacetylbacillosaminyl-diphospho-u	0.158324487	1.536238142	4.130344588	0.371939462	0.709937923	0.996994191
2.7.1.30	glycerol kinase	777.9550507	0.219481904	0.323276897	0.678928516	0.497183157	0.996994191
2.9.1.1	L-seryl-tRNA(Ser) seleniumtransferase	26.83744539	-0.273525457	0.603900579	-0.45293127	0.650598233	0.996994191
1.4.3.16	L-aspartate oxidase	256.2428411	-0.03678138	0.20327668	-0.180942449	0.856412749	0.996994191
3.5.1.102	2-amino-5-formylamino-6-ribosylaminopyrimidin-4(3H)-one 5'-r	3.366320964	1.669479565	2.189522539	0.762485672	0.445770191	0.996994191
3.2.1.8	endo-1,4-beta-xylanase	117.6752233	-0.030242842	0.462501179	-0.065389761	0.947863676	0.996994191
1.1.1.117	D-arabinose 1-dehydrogenase	0.919026554	0.91029319	4.094966274	0.22229565	0.824083738	0.996994191
2.7.4.31	5-(aminomethyl)-3-furanmethanol phosphate kinase	6.817399672	3.247726503	3.22355719	1.007497715	0.313695655	0.996994191

2.1.1.10	homocysteine S-methyltransferase	3.904802519	0.128775643	1.331632272	0.096705108	0.922960583	0.996994191
3.6.3.32	glycine betaine/proline transport system ATP-binding protein	53.71368105	0.308254635	0.499879086	0.616658395	0.537460069	0.996994191
2.5.1.89	tritrans,polycis-undecaprenyl-diphosphate synthase [geranylge	2.984712843	3.30854053	2.216263166	1.492846419	0.135477393	0.996994191
1.17.1.8	4-hydroxy-tetrahydrodipicolinate reductase	407.8052854	0.22955873	0.261382995	0.878246614	0.3798099	0.996994191
3.1.1.83	epsilon-lactone hydrolase	14.81851815	0.073630485	0.799995709	0.092038601	0.926667371	0.996994191
2.1.1.80	chemotaxis protein methyltransferase CheR	123.6007911	-0.510486533	0.625934614	-0.815558881	0.414752475	0.996994191
2.3.1.15	glycerol-3-phosphate O-acyltransferase 1/2	587.5392468	-0.329938091	0.226559961	-1.456294789	0.145311145	0.996994191
1.17.1.1	CDP-4-dehydro-6-deoxyglucose reductase, E3	83.02591322	0.085317272	0.440400649	0.19372649	0.846390049	0.996994191
1.4.4.2	glycine dehydrogenase	368.0048341	-0.171733562	0.317729885	-0.540501758	0.588851049	0.996994191
4.1.1.32	phosphoenolpyruvate carboxykinase (GTP)	297.5921169	0.718572556	0.577695594	1.243860199	0.21355102	0.996994191
2.3.1.30	serine O-acetyltransferase	286.1181777	-0.217234738	0.267592189	-0.8118127	0.416899114	0.996994191
3.2.1.-	#N/A	103.1252457	-0.286298559	0.3779444	-0.757515016	0.448741373	0.996994191
5.4.99.15	(1->4)-alpha-D-glucan 1-alpha-D-glucosylmutase	0.155156823	2.037805974	4.116358126	0.4950507	0.620564332	0.996994191
4.2.99.20	2-succinyl-6-hydroxy-2,4-cyclohexadiene-1-carboxylate synthas	3.361004476	-0.540356025	1.551527251	-0.348273628	0.727634697	0.996994191
6.3.4.2	CTP synthase	512.1476844	-0.028946714	0.192977377	-0.150000558	0.880764175	0.996994191
1.12.99.-	#N/A	251.4678543	1.863098551	1.29710006	1.436356846	0.150900826	0.996994191
1.1.1.363	glucose-6-phosphate 1-dehydrogenase	223.5508127	0.818011922	0.479414797	1.706271745	0.087957496	0.996994191
2.4.1.129	monofunctional glycosyltransferase	66.90475561	-0.081066136	0.421270531	-0.192432487	0.847403447	0.996994191
4.3.1.4	formiminotetrahydrofolate cyclodeaminase	55.490593	-0.461372785	0.527472868	-0.87468534	0.381745139	0.996994191
2.1.1.268	tRNAThr (cytosine32-N3)-methyltransferase	0.031664897	1.436859336	4.142987902	0.346817169	0.728728678	0.996994191
2.7.1.21	thymidine kinase	124.3847308	-0.255012365	0.383898768	-0.66426982	0.506517634	0.996994191
1.14.99.46	pyrimidine oxygenase	0.04941368	2.308811263	4.121547766	0.560180639	0.575356231	0.996994191
3.4.23.5	cathepsin D	0.214256324	3.521308529	4.077187401	0.863661192	0.387774039	0.996994191
2.4.1.281	4-O-beta-D-mannosyl-D-glucose phosphorylase	191.910689	-0.427776618	0.455480154	-0.939177294	0.347639725	0.996994191
3.1.3.32	bifunctional polynucleotide phosphatase/kinase	0.228120967	3.403480411	4.080613193	0.834061022	0.404246598	0.996994191
5.1.1.7	diaminopimelate epimerase	227.37739	-0.219064609	0.210711964	-1.039640112	0.298507134	0.996994191
2.3.2.8	arginyl-tRNA---protein transferase	4.178957052	0.319422242	2.450342995	0.130358175	0.896283055	0.996994191
2.1.1.314	diphthine methyl ester synthase	0.868720118	4.557984901	4.053846547	1.124360493	0.260860125	0.996994191
2.1.1.206	tRNA (cytidine56-2'-O)-methyltransferase	2.423959494	3.649985059	2.110542171	1.729406363	0.083736392	0.996994191
1.1.1.291	2-hydroxymethylglutarate dehydrogenase	1.511684876	2.163982137	3.390435693	0.638260782	0.523303936	0.996994191
6.3.3.1	phosphoribosylformylglycinamidine cyclo-ligase	467.9122205	-0.059118417	0.236383812	-0.250095032	0.802513858	0.996994191
6.3.2.12	#N/A	404.1838337	-0.348575236	0.237449711	-1.467996044	0.142105298	0.996994191
2.8.1.12	molybdopterin synthase catalytic subunit	7.150256944	0.629490398	1.15334314	0.545796282	0.585205979	0.996994191
3.6.4.6	vesicle-fusing ATPase	0.82589221	4.14360902	4.059368692	1.020752076	0.307371915	0.996994191
1.21.4.1	#N/A	190.0109388	-0.463010534	0.75461158	-0.613574647	0.539496441	0.996994191
2.7.1.130	tetraacyldisaccharide 4'-kinase	112.9010033	0.399205125	0.333201927	1.198087686	0.230882882	0.996994191
1.2.1.8	betaine-aldehyde dehydrogenase	13.49926807	1.522848358	1.176149524	1.294774454	0.195398047	0.996994191
1.1.1.31	3-hydroxyisobutyrate dehydrogenase	24.6938277	-1.056843563	0.582874254	-1.813158766	0.069807338	0.996994191
2.4.1.320	beta-1,4-mannooligosaccharide/beta-1,4-mannosyl-N-acetylglu	286.1595224	0.110962813	0.437547003	0.253602041	0.79980302	0.996994191
2.7.6.1	ribose-phosphate pyrophosphokinase	499.4320736	0.074383534	0.295539945	0.251686906	0.801283079	0.996994191
4.1.2.20	2-dehydro-3-deoxyglucarate aldolase	2.948779531	-0.35536144	1.425302262	-0.249323564	0.803110505	0.996994191
1.1.1.378	L-rhamnose 1-dehydrogenase	0.031664897	1.436859336	4.142987902	0.346817169	0.728728678	0.996994191
5.1.3.3	aldose 1-epimerase	653.8019181	-0.43940528	0.347291711	-1.265233998	0.205787459	0.996994191
5.3.1.28	D-sedoheptulose 7-phosphate isomerase	39.20850805	-0.144942749	0.383310567	-0.378133977	0.705331069	0.996994191
2.3.1.47	8-amino-7-oxononanoate synthase	68.37815135	-0.106052547	0.558343308	-0.189941466	0.849354998	0.996994191
2.4.99.13	3-deoxy-D-manno-octulosonic-acid transferase	102.8745696	-0.356249249	0.359208676	-0.99176126	0.321313999	0.996994191
3.1.3.23	sugar-phosphatase	1.477961753	1.883240967	3.636259306	0.517906125	0.604523768	0.996994191
3.2.1.170	mannosylglycerate hydrolase	11.41345483	0.785657464	0.955593219	0.822167265	0.410981706	0.996994191
3.4.13.9	Xaa-Pro dipeptidase	41.36870323	-0.303499796	0.647198748	-0.468943731	0.639109857	0.996994191
2.4.2.48	7-cyano-7-deazaguanine tRNA-ribosyltransferase	3.042578273	3.000687305	2.443572746	1.227991804	0.219450045	0.996994191
3.6.1.26	CDP-diacylglycerol pyrophosphatase	0.18037173	2.603197329	4.084601999	0.637319702	0.52391662	0.996994191
2.1.1.290	tRNA wybutosine-synthesizing protein 4	0.053564081	2.349751186	4.116844279	0.570765136	0.568158859	0.996994191
2.1.1.294	O-antigen chain-terminating methyltransferase	0.321384486	3.896626821	4.069934058	0.957417679	0.338356479	0.996994191
1.1.1.41	isocitrate dehydrogenase (NAD+)	33.21155859	0.161232406	0.44855283	0.359450204	0.719258323	0.996994191
2.3.1.24	Acyl-CoA-dependent ceramide synthase	0.053564081	2.349751186	4.116844279	0.570765136	0.568158859	0.996994191
2.7.1.5	rhamnulokinase	365.1696497	-0.56240147	0.544676769	-1.032541686	0.30181843	0.996994191
2.1.1.179	16S rRNA (guanine(1405)-N(7))-methyltransferase	0.01747704	1.141178067	4.13842089	0.275752056	0.78273851	0.996994191
2.7.1.189	autoinducer-2 kinase	10.84554191	0.357953738	1.175637497	0.304476285	0.760765057	0.996994191
2.4.-.-	#N/A	73.37059448	-0.648589916	0.363952406	-1.782073441	0.074737256	0.996994191
3.2.2.9	adenosylhomocysteine nucleosidase	244.216832	0.168397165	0.223094942	0.754822873	0.450355268	0.996994191
2.8.3.1	propionate CoA-transferase	63.7064274	-0.415592095	0.568307447	-0.731280396	0.464607903	0.996994191
4.1.1.48	indole-3-glycerol phosphate synthase	101.6641702	-0.710959809	0.451557812	-1.574460212	0.115381093	0.996994191
3.5.3.18	dimethylargininase	1.952238584	-0.956560546	1.730383673	-0.552802573	0.580398607	0.996994191
2.8.1.2	thiosulfate/3-mercaptopyruvate sulfurtransferase	4.40773592	-0.667234366	1.203081899	-0.554604276	0.579165363	0.996994191
2.1.3.-	#N/A	5.301016546	-1.154856932	1.054388399	-1.095286076	0.273391326	0.996994191
2.8.1.-	#N/A	84.54624326	0.222857123	0.810751507	0.274877223	0.783410567	0.996994191
3.6.3.7	#N/A	18.59393918	-0.061138854	0.534562001	-0.114371866	0.908943015	0.996994191
2.7.1.52	fucokinase	36.04461727	-0.396383917	0.522728984	-0.758297184	0.448273092	0.996994191
3.1.26.8	ribonuclease M5	69.66098261	-0.167756765	0.402079051	-0.417223342	0.676515053	0.996994191
4.1.3.30	methylisocitrate lyase	1.214680599	1.026687253	2.238760395	0.458596308	0.646524088	0.996994191
3.5.1.24	choloylglycine hydrolase	386.5802979	0.216620631	0.202725915	1.068539416	0.28527726	0.996994191
2.1.1.35	tRNA (uracil-5-)-methyltransferase	1.234391661	-0.552360276	2.618985068	-0.210906233	0.83296044	0.996994191
5.4.2.6	beta-phosphoglucomutase	18.10806377	-0.780848008	0.836254545	-0.933744411	0.350435751	0.996994191
2.6.1.45	#N/A	1.573124213	3.18471117	2.106699737	1.511706256	0.130608607	0.996994191
1.3.1.93	very-long-chain enoyl-CoA reductase	0.160692243	3.261377394	4.083493213	0.798673397	0.424479817	0.996994191
1.1.1.69	gluconate 5-dehydrogenase	430.6355441	0.241234841	0.415470155	0.580630975	0.561489192	0.996994191
1.1.1.-	#N/A	1599.197698	-0.545635478	0.82434869	-0.661898883	0.508036027	0.996994191
2.7.1.146	ADP-dependent phosphofructokinase/glucokinase	7.609187489	-1.190242302	1.120899505	-1.061863527	0.288297645	0.996994191
5.4.3.8	glutamate-1-semialdehyde 2,1-aminomutase	116.1926709	-0.137865773	0.264367974	-0.521491958	0.602024107	0.996994191
3.6.3.17	simple sugar transport system ATP-binding protein	2516.512826	-0.066744349	0.372708984	-0.179078992	0.857875678	0.996994191
1.4.1.24	3-dehydroquinate synthase II	6.211384313	2.670005021	1.515395436	1.761919666	0.078082871	0.996994191
4.2.2.17	inulin fructotransferase (DFA-I-forming)	0.203184014	2.227299348	4.112337613	0.541613933	0.588084492	0.996994191
3.6.3.29	molybdate transport system ATP-binding protein	73.99404027	-0.896790584	0.573107348	-1.5647864	0.117633002	0.996994191
1.1.1.377	L-rhamnose 1-dehydrogenase	0.031664897	1.436859336	4.142987902	0.346817169	0.728728678	0.996994191
3.1.3.64	myotubularin	0.240510691	2.224112472	4.095847329	0.543016449	0.587118477	0.996994191
3.1.3.3	phosphoserine phosphatase	172.8292548	0.193349584	0.307812035	0.62814173	0.529911098	0.996994191
2.7.8.37	alpha-D-ribose 1-methylphosphonate 5-triphosphate synthase	6.201350423	0.582623963	1.554688404	0.374752884	0.707844258	0.996994191

2.1.1.215	tRNA (guanine26-N2/guanine27-N2)-dimethyltransferase	0.68372251	4.043597878	4.058887184	0.996233129	0.319136886	0.996994191
2.6.1.51	alanine-glyoxylate transaminase / serine-glyoxylate transamina	1.573124213	3.18471117	2.106699737	1.511706256	0.130608607	0.996994191
3.4.22.15	cathepsin L	6.825057858	3.277089012	3.520093691	0.930966417	0.351870936	0.996994191
3.1.2.28	1,4-dihydroxy-2-naphthoyl-CoA hydrolase	0.144460652	0.533493742	4.118712294	0.129529257	0.896938876	0.996994191
3.2.1.80	fructan beta-fructosidase	131.8740289	-0.070591606	0.328713668	-0.214751052	0.829961417	0.996994191
5.1.3.32	L-rhamnose mutarotase	138.9551777	0.377978057	0.398575223	0.948323015	0.342965038	0.996994191
2.3.1.199	elongation of very long chain fatty acids protein 6	0.160692243	3.261377394	4.083493213	0.798673397	0.424479817	0.996994191
2.1.1.184	23S rRNA (adenine-N6)-dimethyltransferase	9.19044385	1.178542048	0.840871532	1.401572063	0.161043074	0.996994191
5.4.3.3	beta-lysine 5,6-aminomutase alpha subunit	110.9655405	-0.963905375	0.616041338	-1.564676453	0.117658792	0.996994191
2.7.1.39	homoserine kinase	172.7008888	-0.046632679	0.286286982	-0.16288788	0.870606714	0.996994191
1.3.1.87	2,3-dihydroxy-2,3-dihydrophenylpropionate dehydrogenase	0.198327269	3.184331323	4.082708077	0.779955672	0.435416967	0.996994191
2.1.1.282	tRNA wybutosine-synthesizing protein 3	0.428512649	4.168444403	4.065781869	1.025250379	0.305245062	0.996994191
4.-.-.-	#N/A	0.528273679	1.426108446	4.100303078	0.347805618	0.72798617	0.996994191
4.1.99.3	deoxyribodipyrimidine photo-lyase	1.11007286	2.753652359	2.730851927	1.008349201	0.313286851	0.996994191
4.1.2.9	xylulose-5-phosphate/fructose-6-phosphate phosphoketolase	141.7183535	0.128710734	0.349240213	0.368545	0.712466898	0.996994191
2.7.1.43	glucuronokinase	0.428512649	4.168444403	4.065781869	1.025250379	0.305245062	0.996994191
1.1.1.369	myo-inositol 2-dehydrogenase / D-chiro-inositol 1-dehydrogena	80.77035126	-0.039438016	0.463467613	-0.085093359	0.93218717	0.996994191
1.1.1.34	hydroxymethylglutaryl-CoA reductase (NADPH)	8.97296793	2.205686943	1.764777238	1.249838731	0.211358465	0.996994191
3.4.22.34	legumain	28.63541982	2.441694971	1.897209567	1.286992756	0.198096813	0.996994191
1.14.12.17	nitric oxide dioxygenase	16.24167399	-0.565386582	1.416452994	-0.399156615	0.689777809	0.996994191
2.7.8.-	#N/A	921.7247401	-0.31173637	0.232235683	-1.342327614	0.179489794	0.996994191
3.1.5.1	dGTPase	347.7338709	-0.168181153	0.227555468	-0.739077618	0.459859867	0.996994191
3.4.24.-	#N/A	3286.291944	0.100302241	0.150599958	0.666017724	0.505399772	0.996994191
2.1.2.13	UDP-4-amino-4-deoxy-L-arabinose formyltransferase / UDP-glu	2.389353235	1.234422349	1.353169975	0.912244856	0.361639836	0.996994191
3.1.3.24	sucrose-6-phosphatase	863.7598049	-0.266567327	0.167093666	-1.595316766	0.110641418	0.996994191
1.1.1.244	methanol dehydrogenase	0.059587395	1.736442037	4.124299308	0.421027163	0.673735247	0.996994191
3.2.1.93	trehalose-6-phosphate hydrolase	11.81364766	-1.467873375	0.859152422	-1.70851334	0.087541133	0.996994191
2.5.1.56	N-acetylneuraminate synthase	25.85352984	-0.646927431	0.529039017	-1.222835008	0.22139202	0.996994191
3.4.17.14	zinc D-Ala-D-Ala carboxypeptidase	0.153183881	1.38223821	4.137869078	0.334045902	0.738344921	0.996994191
2.7.1.15	ribokinase	117.0899583	-0.110348051	0.394305577	-0.279854146	0.779589408	0.996994191
3.6.3.24	nickel transport system ATP-binding protein	12.08036817	-1.313257199	1.123276298	-1.169131051	0.242350835	0.996994191
2.3.2.-	#N/A	10.17339722	0.697646872	1.079049799	0.646538161	0.517930892	0.996994191
2.4.1.46	1,2-diacylglycerol 3-beta-galactosyltransferase	0.053564081	2.349751186	4.116844279	0.570765136	0.568158859	0.996994191
2.6.1.87	UDP-4-amino-4-deoxy-L-arabinose-oxoglutarate aminotransfera	0.58308361	2.428961101	4.069279775	0.596901967	0.550572834	0.996994191
5.99.1.-	#N/A	358.5001704	0.073328792	0.272077536	0.269514319	0.787533925	0.996994191
3.6.1.67	dihydroneopterin triphosphate diphosphatase	2.498223838	-1.282541869	1.816755291	-0.705951911	0.480218044	0.996994191
2.2.1.2	transaldolase	347.3412832	0.236395038	0.39550671	0.597701712	0.550038983	0.996994191
6.1.1.6	#N/A	577.45988	0.16124863	0.154223058	1.045554614	0.295766713	0.996994191
4.3.1.7	#N/A	29.98753477	-1.502015563	0.671008774	-2.238443996	0.025192116	0.996994191
6.1.1.24	nondiscriminating glutamyl-tRNA synthetase	6.713044222	-0.890885175	1.280767855	-0.69558677	0.486687654	0.996994191
2.4.1.25	4-alpha-glucanotransferase	826.5909988	-0.080185754	0.279975145	-0.286403118	0.774569387	0.996994191
3.4.15.6	cyanophycinase	0.722055264	2.088553084	4.100943101	0.509286043	0.61055174	0.996994191
2.4.1.182	lipid-A-disaccharide synthase	97.65809609	-0.241377259	0.283339429	-0.851901411	0.394268813	0.996994191
3.1.1.24	3-oxoadipate enol-lactonase	0.043850487	1.769618963	4.123058137	0.429200585	0.667777258	0.996994191
2.2.1.9	2-succinyl-5-enolpyruvyl-6-hydroxy-3-cyclohexene-1-carboxylat	86.31340977	-0.067420537	0.394216331	-0.171024211	0.864204729	0.996994191
3.2.1.165	exo-1,4-beta-D-glucosaminidase	3.174521815	-0.252224377	1.626230358	-0.15509757	0.876744398	0.996994191
1.1.1.18	myo-inositol 2-dehydrogenase / D-chiro-inositol 1-dehydrogena	80.77035126	-0.039438016	0.463467613	-0.085093359	0.93218717	0.996994191
2.4.1.7	sucrose phosphorylase	40.05453102	-0.676999281	0.643650257	-1.051812337	0.292885658	0.996994191
3.5.4.2	adenine deaminase	221.7394094	-0.419356059	0.396348003	-1.058050137	0.290032585	0.996994191
5.2.1.8	peptidylprolyl isomerase	1947.119805	-0.073546442	0.368337425	-0.19967138	0.841737599	0.996994191
5.1.1.18	serine racemase	34.51337451	-0.352391768	0.516001575	-0.682927698	0.494652531	0.996994191
3.5.1.97	acyl-homoserine-lactone acylase	0.654836639	1.583170509	4.10446801	0.385718808	0.699704932	0.996994191
2.6.1.13	ornithine--oxo-acid transaminase	47.38238285	-0.060951767	0.531470136	-0.114685216	0.908694632	0.996994191
1.10.3.10	#N/A	6.011939212	0.976197396	2.238473484	0.436099602	0.662764466	0.996994191
2.7.7.6	DNA-directed RNA polymerase	6413.048508	0.353028739	0.351254195	1.005052023	0.3148718	0.996994191
4.1.3.39	4-hydroxy 2-oxovalerate aldolase	107.0039189	0.229301207	0.403250739	0.568631833	0.569606017	0.996994191
1.21.4.2	glycine/sarcosine/betaine reductase complex component A	1374.313735	0.117535901	0.895575333	0.131240663	0.89558493	0.996994191
6.4.1.1	pyruvate carboxylase	459.749004	0.453303092	0.343351163	1.320231705	0.18675767	0.996994191
2.7.2.4	aspartate kinase	707.3786645	-0.307511011	0.349776445	-0.879164438	0.379312123	0.996994191
2.3.1.23	lysophosphatidylcholine acyltransferase / lyso-PAF acetyltransf	0.053564081	2.349751186	4.116844279	0.570765136	0.568158859	0.996994191
4.3.1.1	aspartate ammonia-lyase	270.3826272	0.463648029	0.404176808	1.147141596	0.251323105	0.996994191
2.3.2.23	ubiquitin-conjugating enzyme E2 R	1.849160922	3.886332137	4.054174647	0.958600079	0.337760253	0.996994191
2.7.1.1	hexokinase	14.71173957	1.247168045	0.612419768	2.036459484	0.041704238	0.996994191
5.1.3.1	ribulose-phosphate 3-epimerase	417.9879526	0.152660619	0.327711368	0.465838643	0.641331002	0.996994191
1.1.1.302	2,5-diamino-6-(ribosylamino)-4(3H)-pyrimidinone 5'-phosphate	2.485401298	3.622895549	2.52788812	1.433170843	0.15180902	0.996994191
1.6.99.3	NADH dehydrogenase	65.55285561	0.794000643	0.390108638	2.035332123	0.041817466	0.996994191
1.7.99.4	#N/A	30.33693764	-0.774598353	0.713604367	-1.085473112	0.277712166	0.996994191
3.5.4.31	5-methylthioadenosine/S-adenosylhomocysteine deaminase	210.6530955	-0.33048386	0.516834951	-0.639437909	0.522538094	0.996994191
3.1.3.90	maltose 6'-phosphate phosphatase	12.88136179	-1.64812642	0.952112481	-1.731020707	0.083448071	0.996994191
4.1.1.15	glutamate decarboxylase	99.45348073	0.131789226	0.426984514	0.308651067	0.75758697	0.996994191
4.1.1.5	acetolactate decarboxylase	2.903166624	1.483959218	2.534254551	0.585560443	0.558170937	0.996994191
2.3.1.29	glycine C-acetyltransferase	381.709002	0.124954567	0.478879191	0.260931294	0.794145491	0.996994191
2.1.1.190	23S rRNA (uracil1939-C5)-methyltransferase	488.7838662	-0.142456056	0.20497159	-0.695003909	0.487052852	0.996994191
1.4.7.1	glutamate synthase (ferredoxin)	105.2440678	0.038107535	0.494580827	0.077050167	0.938583636	0.996994191
3.1.26.-	#N/A	413.5075683	0.312620892	0.271150498	1.15294235	0.248934048	0.996994191
3.2.1.156	oligosaccharide reducing-end xylanase	66.937681	-0.431173042	0.445375766	-0.968110694	0.33298909	0.996994191
2.8.3.6	#N/A	0.04941368	2.308811263	4.121547766	0.560180639	0.575356231	0.996994191
3.6.3.16	arsenite/tail-anchored protein-transporting ATPase	7.85424895	0.747159567	0.680082357	1.098631009	0.271929046	0.996994191
6.3.5.11	cobyrinic acid a,c-diamide synthase	160.4431359	-0.373553353	0.361652638	-1.032906478	0.301647667	0.996994191
2.7.11.5	isocitrate dehydrogenase kinase/phosphatase	0.433381955	0.276056288	4.100512596	0.067322385	0.946325057	0.996994191
2.1.2.1	glycine hydroxymethyltransferase	453.8568204	0.103146905	0.243928539	0.42285706	0.672399553	0.996994191
2.6.1.44	alanine-glyoxylate transaminase / (R)-3-amino-2-methylpropior	1.573124213	3.18471117	2.106699737	1.511706256	0.130608607	0.996994191
4.2.3.12	6-pyruvoyltetrahydropterin/6-carboxytetrahydropterin synthas	76.58751425	0.393150996	0.404525038	0.971882971	0.331108772	0.996994191
3.6.4.13	ATP-dependent helicase HrpA	966.7343647	0.169909251	0.274221837	0.619605106	0.535517804	0.996994191
1.1.1.29	glycerate dehydrogenase	185.3650309	-0.273317952	0.303474657	-0.900628589	0.367785829	0.996994191
4.2.1.83	4-oxalmesaconate hydratase	0.163338744	2.053392563	4.093744639	0.501592733	0.615954032	0.996994191
2.3.2.27	protein neuralized	0.3651991	2.038355429	4.093503845	0.497948825	0.618520113	0.996994191

2.7.7.72	tRNA nucleotidyltransferase (CCA-adding enzyme)	259.1690687	-0.024113172	0.269602234	-0.089439807	0.928732389	0.996994191
4.4.1.5	lactoylglutathione lyase	653.2741697	-0.573469612	0.875464629	-0.65504601	0.512438131	0.996994191
3.2.2.1	purine nucleosidase	22.68749659	-1.105004066	0.514843794	-2.146289961	0.031849852	0.996994191
1.4.1.4	glutamate dehydrogenase (NADP+)	1144.214867	0.221140004	0.33131775	0.667455953	0.504480936	0.996994191
2.7.1.58	2-dehydro-3-deoxygalactonokinase	3.687731951	0.217534833	1.225478869	0.177510064	0.859107761	0.996994191
1.1.1.275	(+)-trans-carveol dehydrogenase	0.063329795	1.458576194	4.141019462	0.352226356	0.724668513	0.996994191
1.5.3.1	sarcosine oxidase	20.45793132	0.513446092	0.502637085	1.021504596	0.307015433	0.996994191
2.5.1.77	#N/A	6.782457674	2.870120541	1.951380272	1.470815598	0.141340993	0.996994191
5.3.1.31	sulfoquinovose isomerase	0.144460652	0.533493742	4.118712294	0.129529257	0.896938876	0.996994191
2.7.4.24	inositol-hexakisphosphate/diphosphoinositol-pentakisphospha	0.247565813	3.054910924	4.073728523	0.749905377	0.453311696	0.996994191
4.2.99.18	DNA-(apurinic or apyrimidinic site) lyase	344.0121466	0.091111544	0.357507405	0.254852187	0.798837267	0.996994191
1.16.1.9	ferric-chelate reductase (NADPH)	0.354697033	0.844792641	4.088979165	0.206602335	0.836320437	0.996994191
3.2.1.172	unsaturated rhamnogalacturonyl hydrolase	260.0740849	-0.378000151	0.513635306	-0.735931014	0.461772682	0.996994191
2.4.2.8	hypoxanthine phosphoribosyltransferase	315.6346228	0.128734047	0.286303198	0.449642366	0.652968335	0.996994191
3.2.1.37	xylan 1,4-beta-xylosidase	254.56278	-0.296744965	0.33556518	-0.884313935	0.376526768	0.996994191
1.1.1.307	D-xylose reductase	0.089723976	0.638106246	4.129588845	0.154520527	0.877199327	0.996994191
1.1.1.219	dihydroflavonol-4-reductase	13.80100873	0.053573715	0.666699456	0.080356621	0.935953627	0.996994191
3.6.3.35	manganese/zinc transport system ATP-binding protein	1.137234124	2.656366172	4.069757789	0.652708665	0.513944115	0.996994191
1.7.2.4	nitrous-oxide reductase	0.044861988	0.80861821	4.141284466	0.195257828	0.84519111	0.996994191
1.7.7.1	ferredoxin-nitrite reductase	22.63867316	-0.113664376	0.727348747	-0.156272182	0.875818482	0.996994191
2.4.1.227	UDP-N-acetylglucosamine--N-acetylmuramyl-(pentapeptide) py	385.4145432	-0.010262443	0.166967732	-0.061463632	0.950989977	0.996994191
2.4.2.21	nicotinate-nucleotide--dimethylbenzimidazole phosphoribosylt	150.8245576	-0.446056763	0.341289269	-1.306975645	0.191220983	0.996994191
4.2.1.147	5,6,7,8-tetrahydromethanopterin hydro-lyase	11.36477334	1.645206097	1.100464846	1.495010135	0.134911797	0.996994191
2.3.1.1	amino-acid N-acetyltransferase	239.3172202	-0.041434216	0.308913884	-0.134128695	0.893300809	0.996994191
2.1.1.223	tRNA1Val (adenine37-N6)-methyltransferase	45.39681127	0.7036221	0.568473021	1.237740532	0.215812285	0.996994191
4.1.1.39	#N/A	1.506786979	0.139435614	1.888439077	0.073836438	0.941140533	0.996994191
2.5.1.105	7,8-dihydropterin-6-yl-methyl-4-(beta-D-ribofuranosyl)aminobe	51.1828228	0.679901448	0.357024897	1.904353039	0.056864221	0.996994191
1.1.1.189	carbonyl reductase 1	0.067578231	1.009477571	4.138890667	0.243900516	0.807307869	0.996994191
1.17.4.1	ribonucleotide reductase, class II	558.9808335	0.339889098	0.428744887	0.792753706	0.427921333	0.996994191
2.3.1.183	phosphinothricin acetyltransferase	388.6004679	-1.355364765	0.940686275	-1.440825491	0.14963399	0.996994191
6.3.2.9	UDP-N-acetylmuramoylalanine--D-glutamate ligase	386.6429284	-0.035751488	0.196632215	-0.18181908	0.85572471	0.996994191
6.3.2.17	folylpolyglutamate synthase	404.2567415	-0.348608093	0.237442986	-1.468176	0.142056422	0.996994191
2.7.8.20	phosphoglycerol transferase	16.37875243	-1.504377142	0.850799884	-1.768191522	0.077028891	0.996994191
2.3.3.10	hydroxymethylglutaryl-CoA synthase	12.96787222	2.391846767	1.161116381	2.059954373	0.039402903	0.996994191
2.3.3.15	sulfoacetaldehyde acetyltransferase	10.93170915	0.105666185	1.713630072	0.061662191	0.95083185	0.996994191
3.2.1.28	alpha,alpha-trehalase	5.065846339	1.224793748	0.887533239	1.379997609	0.167587381	0.996994191
2.7.2.1	acetate kinase	846.1385794	0.060040659	0.31608823	0.18994905	0.849349056	0.996994191
3.5.1.44	chemotaxis protein CheD	77.03386573	-0.505222136	0.561024157	-0.900535439	0.367835375	0.996994191
5.1.99.6	NAD(P)H-hydrate epimerase	0.573429732	-0.513914336	4.085602983	-0.125786656	0.899900805	0.996994191
2.1.1.199	16S rRNA (cytosine1402-N4)-methyltransferase	1239.013886	0.088737552	0.369531488	0.240135292	0.810225375	0.996994191
3.7.1.14	2-hydroxy-6-oxonona-2,4-dienedioate hydrolase	0.417225619	-0.257532947	4.101030139	-0.062797136	0.949928047	0.996994191
2.7.2.-	#N/A	3.340273351	2.734077059	2.152893163	1.269954824	0.204100723	0.996994191
2.7.7.93	phosphonoformate cytidylyltransferase	0.291631102	1.459915428	4.140901481	0.352559807	0.724418474	0.996994191
1.13.11.49	chlorite dismutase	0.06577573	1.818740011	4.121422411	0.441289397	0.659003501	0.996994191
3.6.4.3	#N/A	0.214256324	3.521308529	4.077187401	0.863661192	0.387774039	0.996994191
3.4.24.71	endothelin-converting enzyme	0.134585963	0.541740756	4.11950186	0.131506375	0.895374744	0.996994191
1.1.1.4	(R,R)-butanediol dehydrogenase / meso-butanediol dehydroger	5.627014574	0.715809332	1.680143729	0.426040534	0.67007831	0.996994191
1.8.1.4	dihydrolipoamide dehydrogenase	347.1311495	-0.039308343	0.373961468	-0.105113352	0.916285865	0.996994191
3.1.13.5	ribonuclease D	8.99875701	-1.402413662	1.079768565	-1.298809493	0.194009316	0.996994191
1.1.1.305	UDP-4-amino-4-deoxy-L-arabinose formyltransferase / UDP-glu	2.389353235	1.234422349	1.353169975	0.912244856	0.361639836	0.996994191
5.4.99.19	16S rRNA pseudouridine516 synthase	278.6387261	-0.523406633	0.255566116	-2.048028278	0.040557229	0.996994191
2.7.1.68	1-phosphatidylinositol-4-phosphate 5-kinase	0.256999344	1.936757345	4.093852717	0.473089161	0.63614957	0.996994191
1.1.1.60	2-hydroxy-3-oxopropionate reductase	30.15284766	-0.906394366	0.798296861	-1.135410159	0.256203505	0.996994191
2.7.1.198	PTS system, glucitol/sorbitol-specific IIA component	25.02906499	-1.380027139	1.100489406	-1.254012198	0.209837572	0.996994191
1.2.1.89	D-glyceraldehyde dehydrogenase (NADP+)	0.057984819	1.314825092	4.126913272	0.318597704	0.750031595	0.996994191
2.5.1.42	geranylgeranylglycerol-phosphate geranylgeranyltransferase	0.483729329	2.951938711	4.085030339	0.722623448	0.469911258	0.996994191
1.1.1.136	UDP-N-acetyl-D-glucosamine dehydrogenase	10.54149	0.151498814	0.744773075	0.203416072	0.838809839	0.996994191
2.5.1.128	N4-bis(aminopropyl)spermidine synthase	2.284826551	0.48741404	2.608266472	0.186872793	0.851760371	0.996994191
2.8.4.3	tRNA-2-methylthio-N6-dimethylallyladenosine synthase	451.3763314	0.156627202	0.164927581	0.949672583	0.342278645	0.996994191
3.2.1.40	alpha-L-rhamnosidase	406.0979955	-0.320085997	0.354556298	-0.902779046	0.366643174	0.996994191
1.1.1.381	3-hydroxy acid dehydrogenase / malonic semialdehyde reducta	0.590372679	0.564991774	4.092551402	0.138053678	0.890197995	0.996994191
3.5.2.7	imidazolonepropionase	167.9124087	-0.323385007	0.414885062	-0.779456858	0.435710642	0.996994191
5.1.99.4	alpha-methylacyl-CoA racemase	0.26814328	1.90920835	4.118946746	0.463518581	0.642992699	0.996994191
6.3.2.14	bifunctional isochorismate lyase / aryl carrier protein	1.81108835	1.640894604	2.454478093	0.668530963	0.503794724	0.996994191
1.4.3.19	glycine oxidase	0.087385198	0.676249257	4.131273186	0.163690278	0.869974971	0.996994191
3.1.3.8	3-phytase	0.021925243	1.675481703	4.126913301	0.405989072	0.68475063	0.996994191
2.4.1.34	1,3-beta-glucan synthase	0.304865092	-0.314093314	4.096636158	-0.07667103	0.938885251	0.996994191
3.1.11.2	exodeoxyribonuclease III	196.406404	-0.181842661	0.27237716	-0.66761347	0.504380357	0.996994191
2.1.1.33	tRNA (guanine-N7-)-methyltransferase	269.7709139	0.078419064	0.295369765	0.265494554	0.790628505	0.996994191
3.5.1.53	N-carbamoylputrescine amidase	98.58245507	0.131955244	0.41576437	0.317379875	0.750955376	0.996994191
1.9.3.1	#N/A	2.510583364	-2.127001711	2.614050894	-0.813680298	0.415828124	0.996994191
3.2.1.24	alpha-mannosidase	220.3015912	0.062132357	0.285552905	0.217586148	0.827751578	0.996994191
3.6.3.3	#N/A	686.2195542	0.109065879	0.411120066	0.265289604	0.790786373	0.996994191
4.2.1.40	glucarate dehydratase	5.876213181	-0.652797269	1.166796291	-0.559478355	0.5758353	0.996994191
2.4.2.17	ATP phosphoribosyltransferase	161.4871201	0.209544689	0.263660963	0.794750527	0.426758634	0.996994191
1.2.7.3	#N/A	1651.051343	0.383968835	0.373506673	1.028010644	0.303944821	0.996994191
2.7.8.38	archaetidylserine synthase	1.483140351	3.43121494	4.05444486	0.846284771	0.397393901	0.996994191
2.4.2.1	#N/A	482.41952	0.148878883	0.237461696	0.62695957	0.530685737	0.996994191
1.3.99.26	phytoene desaturase	1.632348624	3.119400767	4.065906252	0.767209221	0.442957131	0.996994191
2.4.1.173	sterol 3beta-glucosyltransferase	0.102122587	1.373190375	4.141447253	0.331572586	0.740212023	0.996994191
2.7.1.73	inosine kinase	0.288921303	0.368593529	4.10574585	0.089775047	0.928465977	0.996994191
4.1.1.25	tyrosine decarboxylase	5.813486058	4.903514607	3.999516489	1.226026851	0.220188571	0.996994191
3.2.1.54	cyclomaltodextrinase / maltogenic alpha-amylase / neopullular	0.261732976	1.164912141	4.105588734	0.283738147	0.776611058	0.996994191
2.6.1.106	dTDP-3-amino-3,4,6-trideoxy-alpha-D-glucose transaminase	0.02470684	2.040638566	4.125310065	0.494663076	0.62083797	0.996994191
6.3.1.2	glutamine synthetase	942.2618412	-0.302463639	0.176992419	-1.708907309	0.08746812	0.996994191
3.6.3.30	iron(III) transport system ATP-binding protein	109.2747114	0.584620894	0.439694876	1.329605884	0.183648158	0.996994191
6.3.2.3	glutathione synthase	0.92520644	1.510964815	2.57885848	0.58590451	0.557939686	0.996994191

6.3.2.39	aerobactin synthase	0.288921303	0.368593529	4.10574585	0.089775047	0.928465977	0.996994191
3.5.1.82	N-acyl-D-glutamate deacylase	0.031664897	1.436859336	4.142987902	0.346817169	0.728728678	0.996994191
5.1.1.4	proline racemase	4.45986566	-1.360300422	1.139661375	-1.193600531	0.232634266	0.996994191
2.7.1.60	N-acylmannosamine kinase	0.427661618	1.780836773	4.101570835	0.434184083	0.66415477	0.996994191
1.8.5.4	sulfide:quinone oxidoreductase	10.70375631	0.084483567	0.901982821	0.093664275	0.92537585	0.996994191
2.1.1.224	23S rRNA (adenine-C8)-methyltransferase	2.006073184	2.655292927	3.697136214	0.718202623	0.472632361	0.996994191
6.2.1.30	phenylacetate-CoA ligase	364.9902406	0.250415179	0.324049975	0.772767159	0.439660193	0.996994191
3.4.21.88	repressor LexA	513.2102101	0.416364577	0.315841139	1.318272149	0.187412561	0.996994191
2.7.1.89	thiamine kinase	0.342787921	2.541781811	4.058437537	0.626295659	0.531121033	0.996994191
2.4.1.64	alpha,alpha-trehalose phosphorylase	6.474905408	0.525882769	1.113111195	0.472444057	0.636609863	0.996994191
3.6.1.23	dUTP pyrophosphatase	305.483228	0.267267381	0.252522208	1.058391589	0.289876953	0.996994191
1.1.1.26	glyoxylate reductase	1.612855192	3.378290581	2.549741344	1.324954231	0.185186332	0.996994191
3.5.1.19	nicotinamidase	4.871855705	-0.093799191	1.419003027	-0.066102178	0.947296477	0.996994191
2.4.1.12	cellulose synthase (UDP-forming)	7.854470084	-0.248820212	0.971128352	-0.256217637	0.797782791	0.996994191
3.1.21.3	type I restriction enzyme, R subunit	1475.812021	0.332114101	0.261549387	1.269794989	0.204157666	0.996994191
2.1.1.189	23S rRNA (uracil747-C5)-methyltransferase	0.723122727	1.382970787	4.092900156	0.337895071	0.735442262	0.996994191
4.2.1.68	L-fuconate dehydratase	0.164310313	1.305603353	4.113808326	0.317370974	0.750962129	0.996994191
1.3.8.4	isovaleryl-CoA dehydrogenase	0.107128162	2.905607862	4.094274383	0.709675901	0.47790514	0.996994191
6.3.5.3	phosphoribosylformylglycinamidine synthase	1280.080832	0.030889711	0.23822825	0.129664348	0.896831991	0.996994191
3.2.1.45	glucosylceramidase	92.54275142	-0.443607163	0.591630759	-0.74980409	0.453372705	0.996994191
2.1.1.163	demethylmenaquinone methyltransferase / 2-methoxy-6-polyp	59.19781542	-0.31834755	0.419451577	-0.758961384	0.447875657	0.996994191
3.4.23.-	#N/A	81.85829158	-0.262319302	0.49228112	-0.532864844	0.594127143	0.996994191
1.1.1.339	dTDP-6-deoxy-L-talose 4-dehydrogenase (NAD+)	9.784419663	0.078900135	0.613630083	0.128579314	0.897690536	0.996994191
6.5.1.-	#N/A	57.77620169	0.919245714	0.897452438	1.024283489	0.305701394	0.996994191
1.5.5.-	#N/A	2.954894685	-1.664802914	1.619022299	-1.028276704	0.303819686	0.996994191
1.1.1.298	malonyl-CoA reductase / 3-hydroxypropionate dehydrogenase	0.051061294	1.428001729	4.141463361	0.344806076	0.730240165	0.996994191
2.7.1.72	streptomycin 6-kinase	0.107128162	2.905607862	4.094274383	0.709675901	0.47790514	0.996994191
5.4.99.28	tRNA pseudouridine32 synthase / 23S rRNA pseudouridine746 s	73.12648985	-0.359555097	0.409966117	-0.877036132	0.380467013	0.996994191
2.7.1.156	adenosylcobinamide kinase / adenosylcobinamide-phosphate g	169.949037	0.359834514	0.319128218	1.127554672	0.259508039	0.996994191
2.3.1.18	galactoside O-acetyltransferase	10.30660446	-0.2315774	0.671700043	-0.344763116	0.730272464	0.996994191
4.99.1.4	uroporphyrin-III C-methyltransferase / precorrin-2 dehydrogena	35.78655682	0.403558293	0.589955972	0.684048153	0.49394476	0.996994191
3.2.1.97	endo-alpha-N-acetylgalactosaminidase	151.0616393	-0.913085923	0.661806807	-1.379686508	0.167683189	0.996994191
4.1.1.87	malonate decarboxylase beta subunit	4.427733717	2.121783013	1.634605743	1.298039617	0.194273722	0.996994191
3.1.3.95	myotubularin	0.240510691	2.224112812	4.095847329	0.543016449	0.587118477	0.996994191
4.2.1.5	D-arabinonate dehydratase	2.260122362	1.294226616	2.438535592	0.530739276	0.595599465	0.996994191
1.2.99.7	aldehyde oxidoreductase	248.9810997	0.268150613	0.61653977	0.434928331	0.663614449	0.996994191
2.4.2.53	undecaprenyl-phosphate 4-deoxy-4-formamido-L-arabinose tra	32.80543789	0.14422819	0.55921137	0.257913551	0.796473627	0.996994191
1.1.1.173	L-rhamnose 1-dehydrogenase	0.031664897	1.436859336	4.142987902	0.346817169	0.728728678	0.996994191
1.3.7.11	digeranylgeranylglycerophospholipid reductase	9.718970903	1.660175065	1.428689546	1.162026467	0.245224714	0.996994191
2.7.1.169	pantoate kinase	2.104766928	3.318810336	2.818432025	1.177537832	0.238980907	0.996994191
3.6.1.40	exopolyphosphatase / guanosine-5'-triphosphate,3'-diphosphat	219.8727794	0.17171314	0.456906632	0.375816694	0.707053176	0.996994191
6.1.1.12	aspartyl-tRNA synthetase	693.8225422	0.071474341	0.179672416	0.397803642	0.690774932	0.996994191
3.5.1.3	omega-amidase	0.286407904	0.742952356	4.106799716	0.180907862	0.856439898	0.996994191
1.5.98.2	5,10-methylenetetrahydromethanopterin reductase	262.9153422	2.467715962	1.208702486	2.041623965	0.041188846	0.996994191
2.1.1.264	23S rRNA (guanine2445-N2)-methyltransferase / 23S rRNA (gua	1.561324307	-1.792221137	1.855881177	-0.965698213	0.334195214	0.996994191
1.1.1.282	quinate/shikimate dehydrogenase	0.314357693	1.313418973	4.106989546	0.319800905	0.749119262	0.996994191
3.1.3.37	fructose-1,6-bisphosphatase I / sedoheptulose-1,7-bisphosphat	0.301146343	0.940107129	4.103146541	0.229118585	0.818776749	0.996994191
3.1.7.2	GTP diphosphokinase / guanosine-3',5'-bis(diphosphate) 3'-dipł	2.493487671	1.570094383	2.120002189	0.740609794	0.458930068	0.996994191
3.5.4.10	phosphoribosylaminoimidazolecarboxamide formyltransferase	631.4865603	-0.057253708	0.223566732	-0.256092253	0.797879604	0.996994191
2.4.1.319	beta-1,4-mannooligosaccharide/beta-1,4-mannosyl-N-acetylglu	286.1595224	0.110962813	0.437547003	0.253602041	0.79980302	0.996994191
2.3.1.169	#N/A	571.4431582	-0.13851125	0.586853229	-0.236023666	0.813414306	0.996994191
5.4.2.2	phosphoglucomutase	1276.024885	0.099673715	0.279571803	0.356522776	0.721449099	0.996994191
5.2.1.1	maleate isomerase	0.513448466	2.482653773	4.094937639	0.60627389	0.544332894	0.996994191
2.7.7.62	adenosylcobinamide kinase / adenosylcobinamide-phosphate g	172.204231	0.397533137	0.318972441	1.246293051	0.212656828	0.996994191
2.1.1.265	tellurite methyltransferase	1.446509712	-0.434125383	2.224169801	-0.19518536	0.84524784	0.996994191
3.5.1.25	N-acetylglucosamine-6-phosphate deacetylase	770.3948878	0.294604341	0.404353754	0.7285807	0.466258188	0.996994191
3.4.24.55	protease III	0.192614202	0.463843892	4.112600639	0.112786028	0.910200195	0.996994191
2.1.1.219	tRNA (adenine57-N1/adenine58-N1)-methyltransferase catalyti	4.213074941	2.811992248	1.750885594	1.606039971	0.108265128	0.996994191
4.1.3.4	hydroxymethylglutaryl-CoA lyase	5.591552884	0.415212852	1.252171253	0.331594301	0.740195623	0.996994191
1.7.2.1	nitrite reductase (NO-forming)	0.06577573	1.818740011	4.121422411	0.441289397	0.659003501	0.996994191
2.1.2.10	aminomethyltransferase	205.0394189	-0.269724778	0.329762662	-0.817936077	0.413393689	0.996994191
2.7.8.1	ethanolaminephosphotransferase	0.129559156	2.351523111	4.100643168	0.573452264	0.566338511	0.996994191
2.7.1.40	pyruvate kinase	620.5141539	0.171578534	0.201142067	0.853021631	0.393647309	0.996994191
2.7.1.11	6-phosphofructokinase 1	1549.328	-0.080532578	0.256152547	-0.31439304	0.753222541	0.996994191
6.1.1.21	#N/A	382.5298565	-0.18274113	0.193005448	-0.946818508	0.343731269	0.996994191
1.97.1.4	#N/A	3571.408187	1.00509899	0.526284195	1.909802726	0.056158618	0.996994191
6.3.2.13	UDP-N-acetylmuramoyl-L-alanyl-D-glutamate--2,6-diaminopime	592.4151967	0.100016385	0.160063494	0.624854442	0.532066596	0.996994191
3.2.2.24	ADP-ribosyl-[dinitrogen reductase] hydrolase	1.660127455	1.023872293	2.11248855	0.484675901	0.627906263	0.996994191
5.-.-.-	#N/A	209.3950617	0.482827688	0.777357405	0.621114154	0.534524517	0.996994191
3.2.2.28	double-stranded uracil-DNA glycosylase	0.132218179	2.851346258	4.093899451	0.696486636	0.486124123	0.996994191
2.7.1.4	fructokinase	447.9267559	-0.483883004	0.282045995	-1.715617357	0.0862321	0.996994191
2.7.1.14	sedoheptulokinase	8.305635679	-1.088116658	0.960899986	-1.13239325	0.257469142	0.996994191
1.11.1.-	#N/A	2.773257521	1.037004793	2.15303701	0.481647453	0.63005641	0.996994191
2.3.1.4	glucosamine-phosphate N-acetyltransferase	0.549114054	1.919574816	4.100533031	0.468128119	0.639692972	0.996994191
4.1.1.11	aspartate 1-decarboxylase	77.14565317	-0.114973603	0.325166208	-0.353584107	0.723650587	0.996994191
3.4.21.81	streptogrisin B	0.59175187	1.974701451	4.075639925	0.484513227	0.62802168	0.996994191
2.7.1.165	glycerate 2-kinase	139.7135454	-0.449511435	0.347869097	-1.292185592	0.19629288	0.996994191
4.4.1.1	cystathionine gamma-lyase	1.197801709	-5.081946517	4.09538931	-1.240894609	0.214644685	0.996994191
1.2.1.87	propionaldehyde dehydrogenase	211.2777077	0.535267267	0.565668355	0.946256339	0.344017857	0.996994191
2.8.3.10	#N/A	110.095947	-0.283344479	0.421709526	-0.671894899	0.501650608	0.996994191
4.4.1.14	1-aminocyclopropane-1-carboxylate synthase	0.438278362	3.424351102	4.075716121	0.840183909	0.400805279	0.996994191
1.3.1.2	dihydropyrimidine dehydrogenase (NADP+)	0.358716976	1.870628671	4.089193287	0.457456652	0.647342853	0.996994191
3.10.1.1	N-sulfoglucosamine sulfohydrolase	0.192614202	0.463843892	4.112600639	0.112786028	0.910200195	0.996994191
2.7.1.51	L-fuculokinase	5.794631649	-0.839466532	1.308353289	-0.641620684	0.5211195	0.996994191
4.1.3.44	tRNA wybutosine-synthesizing protein 1	3.612380128	5.301970085	3.067148149	1.728631885	0.083874999	0.996994191
1.1.1.30	3-hydroxybutyrate dehydrogenase	0.609117398	2.061015594	4.055437883	0.508210372	0.611305818	0.996994191
5.4.99.20	23S rRNA pseudouridine2457 synthase	1.635705765	-0.618080114	1.720798761	-0.359182101	0.719458866	0.996994191

2.4.99.16	starch synthase (maltosyl-transferring)	2.015113309	-1.485063523	2.524497185	-0.588261113	0.556357043	0.996994191
2.7.4.28	[pyruvate, water dikinase]-phosphate phosphotransferase / [py	11.94070215	-1.207680594	0.843931713	-1.43101696	0.152425354	0.996994191
3.4.23.51	hydrogenase 3 maturation protease	3.847375802	1.119394682	1.453090817	0.770354247	0.441089786	0.996994191
1.6.2.4	NADPH-ferrihemoprotein reductase	0.267820405	3.726658981	4.072976819	0.914971812	0.360206428	0.996994191
6.6.1.1	magnesium chelatase subunit H	18.92594321	-0.370588822	0.888961711	-0.416878272	0.676767447	0.996994191
1.1.1.102	3-dehydrosphinganine reductase	0.428512649	4.168444403	4.065781869	1.025250379	0.305245062	0.996994191
3.6.3.8	Ca2+-transporting ATPase	547.2942701	-0.129207801	0.219566083	-0.588468853	0.556217634	0.996994191
1.1.1.36	acetoacetyl-CoA reductase	0.635026121	2.763460581	4.050135168	0.682313174	0.495040945	0.996994191
2.4.1.290	N,N'-diacetylbacillosaminyl-diphospho-undecaprenol alpha-1,3-	0.063329795	1.458576194	4.141019462	0.352226356	0.724668513	0.996994191
3.1.3.11	fructose-1,6-bisphosphatase I / sedoheptulose-1,7-bisphosphat	417.2305742	0.057017204	0.227969589	0.250108817	0.802503198	0.996994191
6.1.1.2	tryptophanyl-tRNA synthetase	437.2335089	-0.221165055	0.223348762	-0.990222885	0.322065189	0.996994191
1.1.1.79	glyoxylate/hydroxypyruvate reductase	2.265448947	2.960891197	3.007359261	0.984548549	0.324845873	0.996994191
6.3.2.34	coenzyme F420-0:L-glutamate ligase / coenzyme F420-1:gamm	4.270530574	2.473856393	1.800995702	1.373604829	0.169564397	0.996994191
1.1.1.374	UDP-N-acetylglucosamine 3-dehydrogenase	15.70625512	3.489334827	2.571433444	1.356960972	0.174793612	0.996994191
2.4.1.21	starch synthase	1036.64266	0.115915078	0.380092883	0.304965138	0.760392704	0.996994191
2.3.2.3	phosphatidylglycerol lysyltransferase	8.777805559	-1.025613859	1.130292537	-0.907387977	0.364201665	0.996994191
3.2.1.58	glucan 1,3-beta-glucosidase	2.050744339	-0.509192295	2.237225106	-0.227599938	0.819957268	0.996994191
1.1.1.81	glyoxylate/hydroxypyruvate reductase	77.79447013	0.31410351	0.414066775	0.758581777	0.448102777	0.996994191
2.7.11.25	mitogen-activated protein kinase kinase kinase 8	0.2108833	3.07477649	4.080544784	0.753521075	0.451136852	0.996994191
1.8.99.5	dissimilatory sulfite reductase alpha subunit	158.2360549	0.577011471	0.790554125	0.729882309	0.465462127	0.996994191
2.1.1.63	methylated-DNA-protein]-cysteine S-methyltransferase	145.0292668	-0.449585274	0.429553005	-1.046635151	0.295267883	0.996994191
4.2.99.21	salicylate synthetase	0.795163648	1.084761972	4.078800294	0.265951234	0.790276766	0.996994191
2.3.1.57	diamine N-acetyltransferase	200.7568856	0.863227882	0.509184582	1.69531426	0.090015821	0.996994191
2.4.1.288	galactofuranosylgalactofuranosylrhamnosyl-N-acetylglucosami	0.791497509	0.366138167	4.117847049	0.088914951	0.929149502	0.996994191
2.4.2.14	amidophosphoribosyltransferase	674.2738087	-0.158318525	0.207788428	-0.761921762	0.446106701	0.996994191
2.7.7.39	glycerol-3-phosphate cytidylyltransferase	6.785358283	0.501871593	1.219943787	0.411389113	0.680787235	0.996994191
4.2.2.10	pectin lyase	22.34609549	1.503822303	2.116304773	0.710588721	0.477339134	0.996994191
1.3.5.1	#N/A	497.6955786	0.191097043	0.243245704	0.785613227	0.432094113	0.996994191
4.2.1.6	galactonate dehydratase	27.36780082	0.159371643	0.511980245	0.311284751	0.755584159	0.996994191
4.3.2.1	argininosuccinate lyase	310.1718153	0.196608547	0.219354395	0.896305483	0.370089632	0.996994191
6.1.2.1	D-alanine---(R)-lactate ligase	0.157319219	2.71703545	4.090558974	0.664221068	0.506548831	0.996994191
2.2.1.6	#N/A	1048.382309	-0.126027493	0.223105781	-0.564877754	0.572156916	0.996994191
2.3.1.102	acetyl CoA:N6-hydroxylysine acetyl transferase	0.192614202	0.463843892	4.112600639	0.112786028	0.910200195	0.996994191
2.6.1.17	acetylornithine/N-succinyldiaminopimelate aminotransferase	610.7636172	-0.505390066	0.553381826	-0.913275503	0.361097663	0.996994191
3.4.24.15	thimet oligopeptidase	1.127149497	1.62491944	2.64187256	0.615063521	0.538512771	0.996994191
3.1.1.31	6-phosphogluconolactonase	234.1506305	0.395824333	0.334427293	1.183588603	0.236575954	0.996994191
3.6.1.31	phosphoribosyl-ATP pyrophosphohydrolase	255.6035093	0.101024178	0.254132431	0.397525723	0.690979822	0.996994191
1.20.4.1	arsenate reductase (glutaredoxin)	97.46378776	0.606572484	0.629729289	0.963227364	0.335433436	0.996994191
2.7.7.60	2-C-methyl-D-erythritol 4-phosphate cytidylyltransferase	364.2618499	0.309906614	0.170891729	1.813467604	0.069759731	0.996994191
2.7.7.3	pantetheine-phosphate adenylyltransferase	482.6370214	-0.268195179	0.409272954	-0.655296609	0.512276803	0.996994191
2.1.3.6	putrescine carbamoyltransferase	14.52936115	-0.357646028	0.796649138	-0.448937947	0.653476421	0.996994191
3.6.1.1	inorganic pyrophosphatase	704.5610825	-0.049851848	0.212385773	-0.234723105	0.814423653	0.996994191
2.1.1.107	uroporphyrin-III C-methyltransferase	91.90494531	-0.321250486	0.337229305	-0.952617347	0.340783985	0.996994191
1.1.1.337	L-2-hydroxycarboxylate dehydrogenase (NAD+)	6.338592059	3.107741622	1.609565115	1.930795836	0.053508306	0.996994191
3.2.1.143	poly(ADP-ribose) glycohydrolase	0.214256324	3.521308529	4.077187401	0.863661192	0.387774039	0.996994191
1.1.1.42	isocitrate dehydrogenase	416.2986	0.095641929	0.26620535	0.359278765	0.719386559	0.996994191
4.2.1.106	bile-acid 7alpha-dehydratase	6.338790237	0.103797743	1.17429378	0.088391632	0.929565412	0.996994191
3.1.11.3	exodeoxyribonuclease (lambda-induced)	0.5133427	2.60127918	4.09311947	0.63552486	0.525086158	0.996994191
2.7.7.18	nicotinate-nucleotide adenylyltransferase	260.0595274	0.109197938	0.202819352	0.538399996	0.590300929	0.996994191
2.8.3.9	acetate CoA/acetoacetate CoA-transferase alpha subunit	155.1818872	0.179527025	0.615941406	0.291467699	0.770693644	0.996994191
4.1.1.1	pyruvate decarboxylase	3.712525794	-3.237706778	2.92101996	-1.10841652	0.267681974	0.996994191
3.6.4.7	peroxisome-assembly ATPase	0.267820405	3.726658981	4.072976819	0.914971812	0.360206428	0.996994191
1.14.-.-	#N/A	13.38369178	0.633864215	0.658932432	0.961956316	0.336071548	0.996994191
2.7.1.35	pyridoxine kinase	246.3550928	-0.181205669	0.322203307	-0.562395435	0.573846628	0.996994191
1.1.1.289	sorbose reductase	0.33823785	0.547811231	4.122773156	0.132874454	0.894292671	0.996994191
3.2.1.135	cyclomaltodextrinase / maltogenic alpha-amylase / neopullular	0.261732976	1.164912141	4.105588734	0.283738147	0.776611058	0.996994191
1.1.1.370	scyllo-inositol 2-dehydrogenase (NAD+)	0.193874331	1.759668163	4.089330528	0.43030715	0.666972227	0.996994191
3.2.1.1	alpha-amylase	516.5780335	0.917349909	0.442497955	2.073116718	0.038161419	0.996994191
5.1.3.20	ADP-L-glycero-D-manno-heptose 6-epimerase	13.58974453	-0.046234392	0.563441211	-0.082057172	0.93460125	0.996994191
2.-.-.-	#N/A	311.2391915	0.156636956	0.287308695	0.545186968	0.585624933	0.996994191
3.2.1.96	mannosyl-glycoprotein endo-beta-N-acetylglucosaminidase	29.7488792	1.547630092	1.050143494	1.473732019	0.140553758	0.996994191
2.7.1.200	PTS system, galactitol-specific IIA component	14.49931809	-1.133842186	0.874422535	-1.296675396	0.194742898	0.996994191
3.6.1.-	#N/A	427.5886094	-0.354884608	0.393787875	-0.901207553	0.367477976	0.996994191
2.7.1.90	diphosphate-dependent phosphofructokinase	118.196786	0.615531606	0.508374537	1.210783707	0.225978311	0.996994191
5.3.1.27	6-phospho-3-hexuloisomerase	17.12641049	-0.854779328	0.9320447	-0.917101216	0.359089604	0.996994191
3.2.1.82	exo-poly-alpha-galacturonosidase	14.97042183	0.127643607	1.345051231	0.094898695	0.924395293	0.996994191
5.3.1.4	L-arabinose isomerase	323.6098938	-0.069080513	0.310279077	-0.222639932	0.823815755	0.996994191
2.3.1.40	acyl-[acyl-carrier-protein]-phospholipid O-acyltransferase / long	12.07765451	0.248845975	0.859669714	0.289466956	0.772224063	0.996994191
2.4.1.282	3-O-alpha-D-glucosyl-L-rhamnose phosphorylase	0.89434512	2.909225069	3.980054214	0.730951116	0.464809012	0.996994191
2.7.7.9	UTP--glucose-1-phosphate uridylyltransferase	181.7226964	0.230874754	0.36986414	0.624215025	0.532486381	0.996994191
3.1.2.21	medium-chain acyl-[acyl-carrier-protein] hydrolase	1.936026993	0.733157122	1.646012549	0.445414054	0.656020558	0.996994191
0.0.0.0	#N/A	2720696.047	0.140983603	0.838365176	0.168164909	0.866453547	0.996994191
2.7.7.89	[glutamine synthetase] adenylyltransferase / [glutamine synthe	8.353306205	-0.200371827	1.042546727	-0.192194577	0.847589794	0.996994191
4.2.1.129	squalene-hopene/tetraprenyl-beta-curcumene cyclase	6.587769184	0.19700705	1.423540392	0.138392314	0.889930371	0.996994191
4.1.1.18	lysine decarboxylase	7.01613773	0.728775598	1.179577789	0.617827501	0.536689053	0.996994191
5.1.3.2	UDP-glucose 4-epimerase	831.185721	-0.294808181	0.293413821	-1.004752194	0.315016189	0.996994191
5.4.99.61	precorrin-8X/cobalt-precorrin-8 methylmutase	99.4358351	-0.043693865	0.33231724	-0.131482392	0.895393715	0.996994191
3.1.26.5	ribonuclease P/MRP protein subunit POP1	320.5620884	0.200967772	0.354544033	0.566834451	0.570826662	0.996994191
6.3.2.45	UDP-N-acetylmuramate: L-alanyl-gamma-D-glutamyl-meso-diar	13.23918897	-0.339092019	0.784918333	-0.432009299	0.665734655	0.996994191
2.1.1.171	16S rRNA (guanine966-N2)-methyltransferase	16.15378857	-0.995209752	0.855701223	-1.163034159	0.244815643	0.996994191
2.5.1.7	UDP-N-acetylglucosamine 1-carboxyvinyltransferase	756.9220733	-0.166172112	0.149589688	-1.110852717	0.266631751	0.996994191
2.3.1.31	homoserine O-acetyltransferase/O-succinyltransferase	20.97808603	0.874403948	0.763951069	1.144581091	0.252382729	0.996994191
2.7.1.181	O-antigen chain-terminating methyltransferase	0.321384486	3.896626821	4.069934058	0.957417679	0.338356479	0.996994191
4.1.1.98	4-hydroxy-3-polyprenylbenzoate decarboxylase	42.84468263	-0.536513581	0.475232551	-1.128949565	0.25891911	0.996994191
2.1.1.144	trans-aconitate 2-methyltransferase	4.543822609	-1.249208792	1.186913082	-1.052485485	0.292576866	0.996994191
1.1.1.261	glycerol-1-phosphate dehydrogenase [NAD(P)+]	117.8868652	0.273840381	0.386916963	0.707749744	0.479100681	0.996994191
3.2.2.30	adenosylhomocysteine/aminodeoxyfutalosine nucleosidase	0.072907776	1.447251421	4.142030529	0.349406266	0.72678433	0.996994191

1.21.98.1	cyclic dehypoxanthinyl futalosine synthase	8.600599234	0.169992995	0.846504081	0.200817691	0.840841129	0.996994191
1.6.5.3	#N/A	1987.932707	0.690065595	0.416857651	1.655398658	0.097843658	0.996994191
1.3.1.78	arogenate dehydrogenase (NADP+)	0.124335172	1.149152654	4.122705915	0.278737479	0.780446298	0.996994191
2.6.1.22	4-aminobutyrate aminotransferase / (S)-3-amino-2-methylprop	21.40539401	0.22007259	0.633345826	0.347476182	0.728233609	0.996994191
3.1.1.75	poly(3-hydroxybutyrate) depolymerase	0.153183881	1.38223821	4.137869078	0.334045902	0.738344921	0.996994191
2.1.1.151	precorrin-2/cobalt-factor-2 C20-methyltransferase	67.37692647	-0.575953911	0.560182948	-1.028153237	0.303877752	0.996994191
6.3.4.23	5-formaminoimidazole-4-carboxamide-1-(beta)-D-ribofuranosy	19.3794077	3.228831612	1.530858472	2.109164022	0.034930426	0.996994191
6.3.4.18	5-(carboxyamino)imidazole ribonucleotide synthase	20.71168242	-0.523753043	0.829040961	-0.631757739	0.527545192	0.996994191
3.2.1.85	6-phospho-beta-galactosidase	2.516302815	-2.84147472	1.610998783	-1.763796938	0.077766173	0.996994191
2.8.1.13	tRNA-uridine 2-sulfurtransferase	432.6097358	-0.102482564	0.240290444	-0.426494546	0.669747521	0.996994191
4.2.3.3	methylglyoxal synthase	416.339376	-0.086345014	0.281781484	-0.306425437	0.759280743	0.996994191
4.3.1.17	L-serine dehydratase	414.1108953	-0.218837603	0.201034756	-1.088556065	0.2763497	0.996994191
2.3.1.86	fatty acid synthase subunit alpha, fungi type	0.01747704	1.141178067	4.13842089	0.275752056	0.78273851	0.996994191
6.3.3.2	glutamate formiminotransferase / 5-formyltetrahydrofolate cyc	206.2840431	-0.662365073	0.301167439	-2.199324983	0.027854823	0.996994191
5.3.3.8	3-hydroxyacyl-CoA dehydrogenase / enoyl-CoA hydratase / 3-h	0.306876842	1.547468842	4.077244725	0.379537886	0.704288476	0.996994191
3.5.3.1	arginase	30.489841	0.186288338	0.500066357	0.372527237	0.709500337	0.996994191
3.4.21.68	tissue plasminogen activator	0.059587395	1.736442037	4.124299308	0.421027163	0.673735247	0.996994191
4.1.3.27	anthranilate synthase / indole-3-glycerol phosphate synthase	257.4550298	-0.479870769	0.435436848	-1.102044467	0.27044234	0.996994191
6.1.1.17	glutamyl-tRNA synthetase	500.0146009	-0.240963874	0.169007965	-1.425754541	0.153939207	0.996994191
6.2.1.17	propionyl-CoA synthetase	0.186031077	0.468808166	4.113003643	0.113981948	0.909252104	0.996994191
3.4.23.43	leader peptidase (prepilin peptidase) / N-methyltransferase	211.7759288	-0.189472672	0.470865673	-0.402392195	0.687395413	0.996994191
3.5.1.6	beta-ureidopropionase	61.32383993	0.35164039	0.49562693	0.709486044	0.478022909	0.996994191
2.1.1.197	malonyl-CoA O-methyltransferase	27.782404	-0.636193294	0.674608074	-0.943056151	0.345652183	0.996994191
4.1.99.19	2-iminoacetate synthase	227.5228568	-0.153870915	0.295484611	-0.520740877	0.602547294	0.996994191
3.6.1.27	phosphatidylglycerophosphatase B	477.1536917	0.059912101	0.245397258	0.244143318	0.807119824	0.996994191
3.5.3.12	agmatine deiminase	81.72245933	-0.818753877	0.383162695	-2.136830876	0.03261175	0.996994191
2.8.3.17	cinnamoyl-CoA:phenyllactate CoA-transferase	1.036084739	0.77829952	2.658671229	0.292740039	0.769720863	0.996994191
1.7.1.15	nitrite reductase (NADH) large subunit	2.283221944	2.633022475	2.027354358	1.298748028	0.194030416	0.996994191
4.1.3.17	4-hydroxy-4-methyl-2-oxoglutarate aldolase	0.768386619	0.875723158	3.574491759	0.244992356	0.80646235	0.996994191
2.7.7.67	CDP-2,3-bis-(O-geranylgeranyl)-sn-glycerol synthase	1.61179286	2.605606641	2.487636518	1.047422572	0.294904726	0.996994191
1.14.13.127	3-(3-hydroxy-phenyl)propionate hydroxylase	0.384912947	0.613637327	4.131248093	0.148535579	0.88192011	0.996994191
5.3.1.23	methylthioribose-1-phosphate isomerase	98.34085415	0.026459946	0.302657591	0.087425349	0.930333421	0.996994191
6.3.4.24	tyramine---L-glutamate ligase	2.341597874	4.761689609	2.986324831	1.594498214	0.110824491	0.996994191
4.2.1.44	inosose dehydratase	34.21941793	-0.203832039	0.441600359	-0.4615758	0.644385552	0.996994191
3.5.4.13	dCTP deaminase	6.58030804	-1.050345392	1.160218535	-0.905299614	0.365306681	0.996994191
2.1.1.228	tRNA (guanine37-N1)-methyltransferase	306.555681	-0.422745038	0.390899366	-1.081467699	0.279489122	0.996994191
1.1.1.17	mannitol-1-phosphate 5-dehydrogenase	23.22468614	-0.120468539	0.634474602	-0.189871333	0.849409957	0.996994191
1.2.1.16	succinate-semialdehyde dehydrogenase / glutarate-semialdehy	8.439430765	-0.073184274	1.164356967	-0.062853812	0.949882915	0.996994191
3.1.4.46	glycerophosphoryl diester phosphodiesterase	340.9449883	0.035632927	0.252337534	0.141211363	0.88770297	0.996994191
1.1.1.88	hydroxymethylglutaryl-CoA reductase	1.877515391	0.230139404	2.456167799	0.093698568	0.925348608	0.996994191
3.5.4.19	phosphoribosyl-AMP cyclohydrolase	246.2107743	0.026415098	0.248150423	0.106447927	0.915226969	0.996994191
4.1.1.83	4-hydroxyphenylacetate decarboxylase large subunit	33.23974627	-0.435444339	1.003992339	-0.43371281	0.664497003	0.996994191
1.2.1.12	glyceraldehyde 3-phosphate dehydrogenase	2889.827608	0.04550521	0.569531665	0.07989935	0.936317306	0.996994191
3.6.1.21	ADP-sugar diphosphatase	0.107128162	2.905607862	4.094274383	0.709675901	0.47790514	0.996994191
6.5.1.3	RNA-editing ligase	36.42033204	-0.562916181	0.677615843	-0.830730548	0.406125865	0.996994191
1.3.8.7	acyl-CoA dehydrogenase	0.861133089	1.825931412	3.04159353	0.600320652	0.548292558	0.996994191
3.4.24.78	spore protease	139.5273193	-0.228987126	0.625269638	-0.366221405	0.714199871	0.996994191
3.1.3.2	acid phosphatase	13.24700897	0.733533466	0.76612611	0.957457861	0.338336206	0.996994191
3.2.2.20	DNA-3-methyladenine glycosylase I	32.62903189	-1.015242697	0.572575335	-1.773116365	0.076209427	0.996994191
3.5.4.25	GTP cyclohydrolase II	303.6077484	0.342177565	0.262375001	1.304154603	0.192180875	0.996994191
2.4.1.16	chitin synthase	0.299092876	2.354111749	4.08015098	0.576966823	0.563961868	0.996994191
3.4.22.40	bleomycin hydrolase	186.1108036	0.319765946	0.300918605	1.062632688	0.287948557	0.996994191
2.7.8.13	phospho-N-acetylmuramoyl-pentapeptide-transferase	372.9500552	-0.233114372	0.242945119	-0.959535112	0.33728924	0.996994191
1.1.3.48	3-deoxy-alpha-D-manno-octulosonate 8-oxidase	3.333179177	1.00529393	1.532114708	0.656147953	0.511728933	0.996994191
1.1.1.49	glucose-6-phosphate 1-dehydrogenase	223.5508127	0.818011922	0.479414797	1.706271745	0.087957496	0.996994191
5.1.3.15	glucose-6-phosphate 1-epimerase	2.900916458	0.248347813	1.375330483	0.18057319	0.856702601	0.996994191
2.7.4.23	ribose 1,5-bisphosphokinase	0.173198025	1.632248895	4.129062177	0.395307415	0.692616028	0.996994191
3.1.3.4	phosphatidate phosphatase	1.343840583	5.622688778	4.053491444	1.3871224	0.165404443	0.996994191
5.2.1.-	#N/A	1.891810036	1.31943956	1.777676933	0.742226855	0.4579499	0.996994191
4.2.1.53	oleate hydratase	635.5203128	-0.352819237	0.564863846	-0.624609344	0.532227485	0.996994191
5.1.1.1	alanine racemase	456.0159821	-0.259013394	0.237639624	-1.089941942	0.275738719	0.996994191
5.4.99.24	23S rRNA pseudouridine955/2504/2580 synthase	208.4356302	-0.495829034	0.3995158	-1.241074905	0.214578079	0.996994191
1.1.1.95	D-3-phosphoglycerate dehydrogenase / 2-oxoglutarate reducta	733.0911774	0.348732949	0.278314951	1.253015507	0.210200063	0.996994191
3.4.21.26	prolyl oligopeptidase	54.99110323	-0.533639794	0.468637355	-1.138705202	0.254826132	0.996994191
4.2.1.159	dTDP-4-dehydro-6-deoxy-alpha-D-glucopyranose 2,3-dehydrata	6.32428671	-0.426684865	1.327636075	-0.321386917	0.747917196	0.996994191
3.4.24.84	STE24 endopeptidase	4.521324759	0.162731637	1.449959328	0.112231863	0.910639565	0.996994191
2.7.8.41	cardiolipin synthase (CMP-forming)	51.9006002	-0.128824849	0.572220298	-0.225131561	0.821876929	0.996994191
2.7.8.15	UDP-N-acetylglucosamine--dolichyl-phosphate N-acetylglucosa	2.302647437	2.442229041	2.440136728	1.000857457	0.316895727	0.996994191
3.1.3.45	3-deoxy-D-manno-octulosonate 8-phosphate phosphatase (KD(	63.9288138	0.342339296	0.374638537	0.913785588	0.360829521	0.996994191
2.4.1.212	hyaluronan synthase	0.302144354	1.094386114	4.10790981	0.266409479	0.789923865	0.996994191
2.4.2.44	5'-methylthioinosine phosphorylase	0.115969639	1.111420998	4.120235158	0.269746982	0.787354914	0.996994191
3.1.4.55	phosphoribosyl 1,2-cyclic phosphate phosphodiesterase	94.63300165	0.574848898	0.371247019	1.548426976	0.121519531	0.996994191
1.10.3.14	#N/A	311.3418992	-0.063718544	0.280605924	-0.227074837	0.82036555	0.996994191
6.3.4.13	phosphoribosylamine---glycine ligase	540.7811406	-0.227200569	0.224252508	-1.013146169	0.310990349	0.996994191
2.7.1.22	HTH-type transcriptional regulator, transcriptional repressor of	2.053440714	2.28204421	1.859891028	1.226977374	0.219831095	0.996994191
1.1.1.1	alcohol dehydrogenase	622.5140963	0.313253368	0.448706845	0.69812478	0.485099162	0.996994191
4.2.1.120	4-hydroxybutyryl-CoA dehydratase / vinylacetyl-CoA-Delta-ison	61.11807847	0.380863283	0.437490546	0.87056346	0.383992555	0.996994191
5.3.1.1	triosephosphate isomerase (TIM)	914.7994511	0.294430728	0.452656343	0.650450905	0.515401004	0.996994191
1.2.1.58	phenylglyoxylate dehydrogenase alpha subunit	1.250377898	1.357509111	2.841302997	0.477776961	0.632808961	0.996994191
2.7.1.78	bifunctional polynucleotide phosphatase/kinase	0.228120967	3.403480411	4.080613193	0.834061022	0.404246598	0.996994191
4.4.1.19	phosphosulfolactate synthase	2.261025615	4.503130447	3.404277338	1.322786013	0.18590655	0.996994191
3.4.19.12	ubiquitin carboxyl-terminal hydrolase MINDY-1/2	2.089260676	3.432075681	3.36643073	1.019499867	0.307965716	0.996994191
1.6.5.5	NADPH2:quinone reductase	0.160692243	3.261377394	4.083493213	0.798673397	0.424479817	0.996994191
5.4.99.-	#N/A	11.3310429	3.959697596	2.806064205	1.411121524	0.158208785	0.996994191
6.1.1.10	methionyl-tRNA synthetase	1072.529682	-0.238322608	0.384137845	-0.620409083	0.534988494	0.996994191
6.2.1.1	acetyl-CoA synthetase	231.4799934	0.827367854	0.387140447	2.137125842	0.032587758	0.996994191
3.2.1.35	hyaluronoglucosaminidase	245.6207223	-0.369389292	0.463638524	-0.796718289	0.42561466	0.996994191

1.3.1.31	2-enoate reductase	51.06122705	-0.405694151	0.724552467	-0.559923773	0.575531433	0.996994191
2.7.7.85	diadenylate cyclase	239.3174528	-0.065473352	0.243814327	-0.268537755	0.788285417	0.996994191
2.5.1.55	2-dehydro-3-deoxyphosphooctonate aldolase (KDO 8-P synthas	74.66958636	-0.466540166	0.402943791	-1.157829397	0.246933664	0.996994191
3.1.12.1	CRISPR-associated exonuclease Cas4	89.17767433	0.593714001	0.381580805	1.555932564	0.119724129	0.996994191
3.6.1.45	UDP-sugar diphosphatase	148.4862257	-0.091463046	0.470602021	-0.194353279	0.84589927	0.996994191
1.5.5.1	electron-transferring-flavoprotein dehydrogenase	0.060694436	0.969200506	4.133329716	0.234484199	0.814609099	0.996994191
2.7.4.-	#N/A	277.2844787	-0.221469075	0.187600213	-1.180537438	0.237786529	0.996994191
6.3.2.5	phosphopantothenoylcysteine decarboxylase / phosphopantot	280.6301022	-0.031846872	0.164940284	-0.193081223	0.846895358	0.996994191
4.2.1.1	carbonic anhydrase	85.65558449	0.483600759	0.433967795	1.114370156	0.265120421	0.996994191
4.2.1.119	(3R)-3-hydroxyacyl-CoA dehydrogenase / 3a,7a,12a-trihydroxy-	0.498402437	1.612463988	4.099880186	0.393295393	0.694101321	0.996994191
2.7.11.-	#N/A	276.9316338	-0.243514662	0.185813377	-1.310533532	0.190015408	0.996994191
2.7.7.15	choline-phosphate cytidylyltransferase	6.623524635	0.823132652	0.793473999	1.03737823	0.29955962	0.996994191
4.1.2.53	2-dehydro-3-deoxy-L-rhamnonate aldolase	0.443198042	-0.454063104	4.101186137	-0.110715068	0.911842297	0.996994191
2.3.1.191	UDP-3-O-[3-hydroxymyristoyl] glucosamine N-acyltransferase	125.04161	0.22148871	0.328225522	0.674806483	0.499798721	0.996994191
3.4.21.53	ATP-dependent Lon protease	915.8824061	-0.015008589	0.15090112	-0.099459757	0.920773239	0.996994191
2.7.1.63	polyphosphate glucokinase	3.198360875	0.252415826	2.358011921	0.107046035	0.914752459	0.996994191
4.1.1.50	S-adenosylmethionine decarboxylase	23.5955198	-0.46441268	0.710585525	-0.653563383	0.513393142	0.996994191
2.4.1.187	N-acetylglucosaminyldiphosphoundecaprenol N-acetyl-beta-D-	105.0075222	0.121044812	0.211532748	0.572227291	0.567168001	0.996994191
6.3.1.20	lipoate---protein ligase	88.43111534	0.14451006	0.476956091	0.30298399	0.761902062	0.996994191
2.7.7.38	3-deoxy-manno-octulosonate cytidylyltransferase (CMP-KDO sy	95.53006735	-0.337997113	0.344370999	-0.981491222	0.326350561	0.996994191
2.4.1.279	nigerose phosphorylase	0.207493739	0.858502183	4.112410442	0.208758876	0.834636476	0.996994191
1.3.99.16	isoquinoline 1-oxidoreductase	0.043850487	1.769618963	4.123058137	0.429200585	0.667777258	0.996994191
3.1.1.85	pimeloyl-[acyl-carrier protein] methyl ester esterase	24.44366084	-0.308440096	0.695336172	-0.443584137	0.657343276	0.996994191
4.1.1.33	diphosphomevalonate decarboxylase	2.229319486	0.177682042	1.950783873	0.091082382	0.927427131	0.996994191
6.3.2.-	#N/A	11.16411353	0.611174601	0.987168999	0.619118512	0.535838289	0.996994191
2.4.1.-	#N/A	1001.881925	-0.035217603	0.147773587	-0.238321364	0.811631853	0.996994191
4.2.3.5	chorismate synthase	338.384486	0.050057259	0.146859768	0.340850731	0.733215957	0.996994191
1.12.2.1	NiFe] hydrogenase large subunit	22.82601474	-0.442386068	1.066954669	-0.414624989	0.678416452	0.996994191
3.7.1.22	3D-(3,5/4)-trihydroxycyclohexane-1,2-dione acylhydrolase (dec	50.44257656	-0.338538599	0.440006447	-0.769394634	0.441659073	0.996994191
3.5.4.1	cytosine/creatinine deaminase	15.08932727	0.470670393	0.582235136	0.808385416	0.418868745	0.996994191
4.1.3.38	4-amino-4-deoxychorismate lyase	35.10463143	-1.146578339	0.5453633	-2.102411985	0.035517202	0.996994191
1.3.3.3	coproporphyrinogen III oxidase	0.900952424	0.670210612	4.054937644	0.165282594	0.868721558	0.996994191
5.1.99.1	methylmalonyl-CoA/ethylmalonyl-CoA epimerase	68.30107652	0.150089259	0.498662481	0.30098366	0.763426955	0.996994191
1.7.99.1	hydroxylamine reductase	325.2855543	1.0409563	0.483407238	2.153373426	0.031289342	0.996994191
5.3.1.22	hydroxypyruvate isomerase	18.70282022	0.309297127	0.63806723	0.484740655	0.627860324	0.996994191
4.2.1.-	#N/A	0.467580383	1.525313361	4.087797071	0.373138229	0.709045567	0.996994191
2.1.1.56	mRNA (guanine-N7-)-methyltransferase	0.053564081	2.349751186	4.116844279	0.570765136	0.568158859	0.996994191
6.2.1.22	[citrate (pro-3S)-lyase] ligase	34.07579315	0.105115163	0.556646782	0.18883638	0.850221058	0.996994191
1.17.2.1	nicotinate dehydrogenase subunit A	0.015832449	1.463162531	4.140620044	0.353367978	0.723812589	0.996994191
5.3.2.1	phenylpyruvate tautomerase	0.107128162	2.905607862	4.094274383	0.709675901	0.47790514	0.996994191
1.1.1.8	glycerol-3-phosphate dehydrogenase (NAD+)	1.167095298	2.025488418	3.951554641	0.512580137	0.608245051	0.996994191
2.7.1.53	L-xylulokinase	12.11164162	-0.168392971	0.829560355	-0.202990621	0.839142363	0.996994191
2.7.7.77	molybdenum cofactor guanylyltransferase	15.50953688	0.39817086	0.758055572	0.525252864	0.59940742	0.996994191
1.1.1.87	homoisocitrate dehydrogenase	1.266185865	2.942311259	2.638983109	1.1149413	0.264875577	0.996994191
6.3.2.31	coenzyme F420-0:L-glutamate ligase / coenzyme F420-1:gamm	4.270530574	2.473856393	1.800995702	1.373604829	0.169564397	0.996994191
3.2.1.31	beta-glucuronidase	100.6708401	-0.933659743	0.399631603	-2.336301076	0.019475556	0.996994191
3.1.21.7	deoxyribonuclease V	2.012999821	1.798141658	3.745428543	0.480089698	0.631163613	0.996994191
3.2.-.-	#N/A	42.87598427	0.284664106	0.516593989	0.551040299	0.58160605	0.996994191
2.3.1.22	2-acylglycerol O-acyltransferase 3	0.160692243	3.261377394	4.083493213	0.798673397	0.424479817	0.996994191
2.4.1.8	maltose phosphorylase	30.76090562	-0.052165788	0.425658498	-0.122553145	0.90246096	0.996994191
3.4.11.1	leucyl aminopeptidase	47.78279693	0.424945828	0.477582594	0.889784999	0.373581343	0.996994191
2.3.2.26	other hect domain ubiquitin protein ligase E3	0.373766343	1.933920236	4.09885594	0.471819519	0.637055618	0.996994191
2.5.1.74	1,4-dihydroxy-2-naphthoate polyprenyltransferase	209.2981939	0.325742626	0.677932109	0.480494465	0.630875838	0.996994191
1.1.1.156	glycerol 2-dehydrogenase (NADP+)	0.034954079	0.921806092	4.139322395	0.222694925	0.823772951	0.996994191
3.5.2.14	#N/A	21.35431364	0.216265441	0.886575044	0.243933598	0.807282247	0.996994191
2.3.1.28	chloramphenicol O-acetyltransferase type B	86.94404786	-0.413560468	0.500756569	-0.825871278	0.408877115	0.996994191
2.1.1.174	23S rRNA (guanine1835-N2)-methyltransferase	0.096307101	0.646944639	4.130400399	0.156630006	0.875536453	0.996994191
5.1.1.13	aspartate racemase	40.36829995	0.470800509	0.365886514	1.28673917	0.198185216	0.996994191
3.5.2.6	beta-lactamase class C	160.3792134	-0.257933418	0.270267102	-0.954364833	0.339899001	0.996994191
2.7.1.24	dephospho-CoA kinase	224.4460225	0.064975256	0.23909448	0.271755568	0.785809971	0.996994191
3.6.3.55	#N/A	1.408673102	1.467659501	2.074422813	0.707502584	0.479254207	0.996994191
3.13.1.1	UDP-sulfoquinovose synthase	0.129569077	1.438524157	4.102003005	0.350688226	0.72582226	0.996994191
2.1.1.185	23S rRNA (guanosine2251-2'-O)-methyltransferase	315.2882071	0.122050853	0.176156925	0.692852994	0.488401811	0.996994191
3.1.3.25	myo-inositol-1(or 4)-monophosphatase	79.05547234	-0.55662689	0.323331364	-1.721536946	0.085153434	0.996994191
2.7.7.71	D-glycero-alpha-D-manno-heptose 1-phosphate guanylyltransfe	1.859383022	1.485460293	2.083502655	0.712962995	0.475868656	0.996994191
3.4.25.1	#N/A	17.62314595	3.068291898	1.375087042	2.231343765	0.025658367	0.996994191
3.6.3.9	sodium/potassium-transporting ATPase subunit alpha	0.299485303	3.319647976	4.079802788	0.81367854	0.415829131	0.996994191
1.5.1.20	methylenetetrahydrofolate reductase (NADPH)	397.8327279	0.045501405	0.260670841	0.174555024	0.861429292	0.996994191
1.13.11.4	gentisate 1,2-dioxygenase	0.144460652	0.533493742	4.118712294	0.129529257	0.896938876	0.996994191
1.2.1.9	glyceraldehyde-3-phosphate dehydrogenase (NADP+)	6.716637653	0.461938267	1.215769816	0.379955368	0.703978546	0.996994191
1.3.98.1	dihydroorotate dehydrogenase (fumarate)	120.1794457	0.906465114	0.428154101	2.117146868	0.034247385	0.996994191
3.1.3.100	ribosome biogenesis GTPase / thiamine phosphate phosphatas	416.5264494	-0.38027004	0.398103735	-0.955203397	0.339474848	0.996994191
2.1.1.176	16S rRNA (cytosine967-C5)-methyltransferase	301.1690219	0.113008996	0.225285432	0.501625848	0.615930734	0.996994191
3.5.1.2	glutaminase	86.19117576	-0.710871831	0.461612802	-1.539974256	0.123566629	0.996994191
5.5.1.2	3-carboxy-cis,cis-muconate cycloisomerase	0.510612935	1.450627994	4.127309163	0.35147064	0.725235295	0.996994191
1.8.1.9	thioredoxin reductase (NADPH)	1244.533356	0.149505477	0.444656516	0.336226888	0.736699778	0.996994191
5.1.3.29	L-fucose mutarotase	72.80484909	-0.422011991	0.753999163	-0.559698222	0.575685295	0.996994191
1.1.1.276	serine 3-dehydrogenase (NADP+)	0.034954079	0.921806092	4.139322395	0.222694925	0.823772951	0.996994191
3.2.1.41	pullulanase	79.05972656	0.685004478	0.430142338	1.592506519	0.111270939	0.996994191
2.7.1.100	5-methylthioribose kinase	27.51768582	-0.501285574	0.500504524	-1.001560525	0.316555895	0.996994191
2.7.1.83	pseudouridine kinase	78.72070777	-0.113267403	0.485520919	-0.23329047	0.815535859	0.996994191
4.2.2.1	hyaluronate lyase	29.93586609	-0.63193276	0.830174688	-0.761204562	0.446534894	0.996994191
2.3.1.187	malonate decarboxylase alpha subunit	10.47559418	1.262937332	0.821924196	1.536561812	0.12440065	0.996994191
2.7.11.22	CTD kinase subunit alpha	0.552718707	2.0674581	4.068407896	0.508173751	0.611331498	0.996994191
5.3.3.17	trans-2,3-dihydro-3-hydroxyanthranilate isomerase	0.021925243	1.675481703	4.126913301	0.405989072	0.68475063	0.996994191
2.5.1.46	deoxyhypusine synthase	8.030721428	2.451144881	1.182249704	2.073288641	0.038145425	0.996994191
2.8.3.21	L-carnitine CoA-transferase	9.109101761	1.588603578	1.694885437	0.9372926	0.348608068	0.996994191

6.3.2.10	UDP-N-acetylmuramoyl-tripeptide--D-alanyl-D-alanine ligase	403.0847592	-0.125288893	0.168139889	-0.745146756	0.456183006	0.996994191
2.7.1.50	hydroxyethylthiazole kinase	131.5764821	-0.185223987	0.415817948	-0.445444907	0.655998266	0.996994191
2.1.1.258	5-methyltetrahydrofolate corrinoid/iron sulfur protein methyltr	273.0062463	-0.103129786	0.694337235	-0.148529822	0.881924653	0.996994191
1.7.-.-	#N/A	11.71383892	-0.086317935	1.023507052	-0.084335457	0.932789722	0.996994191
4.2.2.21	chondroitin-sulfate-ABC endolyase/exolyase	72.26463302	-1.372847469	0.59268238	-2.316329141	0.020540304	0.996994191
1.1.1.76	meso-butanediol dehydrogenase / (S,S)-butanediol dehydrogen	3.716547359	-0.724865535	1.430846039	-0.506599254	0.612436032	0.996994191
2.4.1.83	dolichol-phosphate mannosyltransferase	119.1710587	-0.50439968	0.30827917	-1.636178273	0.101802282	0.996994191
2.7.1.147	ADP-dependent phosphofructokinase/glucokinase	7.609187489	-1.190242302	1.120899505	-1.061863527	0.288297645	0.996994191
4.2.2.5	chondroitin AC lyase	45.73435572	-0.705495626	0.570166716	-1.237349719	0.215957277	0.996994191
2.1.2.5	glutamate formiminotransferase / 5-formyltetrahydrofolate cyc	154.6551231	-0.122315096	0.415949224	-0.294062565	0.768710096	0.996994191
1.3.1.44	enoyl-acyl-carrier protein] reductase / trans-2-enoyl-CoA reduc	27.94422685	0.27217609	0.550731946	0.494207922	0.621159346	0.996994191
1.5.1.41	aquacobalamin reductase / NAD(P)H-flavin reductase	1.180331694	2.472657046	4.028416753	0.613803684	0.539345062	0.996994191
2.7.1.205	PTS system, cellobiose-specific IIA component	67.85843679	-0.646623341	0.617833935	-1.04659732	0.295285338	0.996994191
3.2.1.20	alpha-glucosidase	601.8969372	-0.263574921	0.290072425	-0.908652108	0.363533791	0.996994191
1.13.12.16	nitronate monooxygenase	3.734228943	-0.187768052	1.591142477	-0.118008321	0.906061065	0.996994191
2.4.1.261	alpha-1,2-mannosyltransferase	0.214256324	3.521308529	4.077187401	0.863661192	0.387774039	0.996994191
2.1.1.191	23S rRNA (cytosine1962-C5)-methyltransferase	466.4840486	-0.275843636	0.239004404	-1.154136204	0.248444333	0.996994191
2.4.2.22	xanthine phosphoribosyltransferase	282.3440183	-0.339147506	0.521021116	-0.650928524	0.515092627	0.996994191
4.6.1.-	#N/A	2.805481591	3.374773292	2.51245927	1.343215125	0.179202325	0.996994191
6.1.1.15	prolyl-tRNA synthetase	530.8683727	-0.088976778	0.161809708	-0.549885288	0.582398055	0.996994191
4.4.1.-	#N/A	0.404940627	1.395981795	4.09875324	0.340586933	0.733414568	0.996994191
2.3.2.6	leucyl/phenylalanyl-tRNA---protein transferase	20.29869155	0.217311484	0.685563424	0.316982319	0.751257019	0.996994191
1.1.1.47	glucose 1-dehydrogenase	6.850818888	0.66295985	0.913042939	0.726099312	0.467777891	0.996994191
3.1.6.1	arylsulfatase	55.45735393	-0.920379931	0.501835379	-1.83402759	0.066649899	0.996994191
6.2.1.45	ubiquitin-activating enzyme E1	4.37738286	0.765427272	2.096567766	0.365085872	0.715047306	0.996994191
2.7.1.148	4-diphosphocytidyl-2-C-methyl-D-erythritol kinase	307.9305914	0.523914091	0.294354553	1.779874255	0.075096542	0.996994191
1.2.1.3	aldehyde dehydrogenase (NAD+)	65.51788066	-0.5915929	0.398709405	-1.483769616	0.13787005	0.996994191
3.5.1.81	N-acyl-D-amino-acid deacylase	40.77082716	-0.099323129	0.456244076	-0.217697356	0.827664924	0.996994191
5.4.99.12	#N/A	337.9885111	-0.291171169	0.227917763	-1.277527323	0.20141614	0.996994191
2.3.1.16	acetyl-CoA acyltransferase	0.591306247	2.887563553	4.046030485	0.71367815	0.47542622	0.996994191
4.2.1.126	N-acetylmuramic acid 6-phosphate etherase	82.6211455	0.237403689	0.385411769	0.615974155	0.537911578	0.996994191
3.5.3.6	arginine deiminase	100.4938897	-0.034741274	0.462887127	-0.075053446	0.940172188	0.996994191
2.7.7.56	ribonuclease PH	33.41871616	-0.0432901	0.583638451	-0.074172803	0.940872886	0.996994191
2.4.1.166	raffinose-raffinose alpha-galactotransferase	0.509876242	0.914278871	4.106432832	0.222645519	0.823811406	0.996994191
2.7.8.39	archaetidylinositol phosphate synthase	0.621380821	2.887981084	4.048362474	0.713370184	0.475616718	0.996994191
3.1.27.-	#N/A	0.616679938	3.135142831	4.076768498	0.769026456	0.441877605	0.996994191
5.3.2.8	4-oxalomesaconate tautomerase	0.029793698	1.661718259	4.127569099	0.402590052	0.687249829	0.996994191
1.1.3.9	galactose oxidase	0.092938899	0.738823359	4.12666749	0.179036319	0.857909184	0.996994191
2.7.7.59	[protein-PII] uridylyltransferase	6.613776947	1.088364415	1.075424756	1.012032138	0.311522687	0.996994191
3.4.11.19	D-aminopeptidase	79.30120568	0.289488565	0.394464532	0.733877299	0.463023524	0.996994191
2.7.1.199	PTS system, glucose-specific IIB component	43.92933022	-1.229335619	0.607937586	-2.022141166	0.04316177	0.996994191
2.7.7.41	phosphatidate cytidylyltransferase	329.3149528	-0.184084342	0.172373298	-1.067940008	0.285547574	0.996994191
2.1.1.74	methylenetetrahydrofolate--tRNA-(uracil-5-)-methyltransferase	148.1849758	0.163146199	0.272809664	0.598022068	0.549825208	0.996994191
2.7.6.2	thiamine pyrophosphokinase	166.8556101	-0.315981839	0.380123127	-0.83126181	0.405825744	0.996994191
2.1.1.17	phosphatidylethanolamine/phosphatidyl-N-methylethanolamir	0.04941368	2.308811263	4.121547766	0.560180639	0.575356231	0.996994191
4.3.1.18	D-serine dehydratase	41.51184634	-0.085785592	0.349723571	-0.245295425	0.806227694	0.996994191
2.5.1.108	2-(3-amino-3-carboxypropyl)histidine synthase	2.518104667	4.681093605	3.355585035	1.39501564	0.163011123	0.996994191
2.4.1.1	glycogen phosphorylase	1122.779221	0.067069917	0.126511545	0.53014859	0.596008912	0.996994191
4.2.1.30	#N/A	5.746841115	-2.136757914	1.382499201	-1.545576238	0.122206944	0.996994191
2.7.7.65	two-component system, cell cycle response regulator	4.101623422	2.333000648	1.853062111	1.258997545	0.208031219	0.996994191
1.3.5.4	succinate dehydrogenase / fumarate reductase, flavoprotein su	913.8675227	0.186744185	0.303128941	0.616055283	0.537858034	0.996994191
1.1.5.6	#N/A	0.860990484	1.944791977	4.036455804	0.481806831	0.629943177	0.996994191
2.4.1.52	poly(glycerol-phosphate) alpha-glucosyltransferase	0.161648	1.522170715	4.132343452	0.368355325	0.712608305	0.996994191
4.1.1.31	phosphoenolpyruvate carboxylase	18.28372225	-1.011151355	0.929161857	-1.088240275	0.276489049	0.996994191
4.1.2.22	xylulose-5-phosphate/fructose-6-phosphate phosphoketolase	141.7183535	0.128710734	0.349240213	0.368545	0.712466898	0.996994191
3.6.3.25	sulfate transport system ATP-binding protein	43.02532111	0.279785765	0.57003157	0.490825034	0.623550202	0.996994191
3.6.1.15	#N/A	5.035962108	-0.414446321	1.575051641	-0.263131894	0.792448922	0.996994191
3.6.3.36	taurine transport system ATP-binding protein	0.734608246	0.32201136	4.08113483	0.078902407	0.937110249	0.996994191
3.2.1.73	licheninase	0.785591429	-1.079870526	3.65831063	-0.295182841	0.767854209	0.996994191
2.1.1.309	18S rRNA (guanine1575-N7)-methyltransferase	0.262409875	2.725656987	4.083349181	0.667505243	0.504449462	0.996994191
1.3.8.1	butyryl-CoA dehydrogenase	257.9585902	-0.233706946	0.579884466	-0.40302329	0.686931091	0.996994191
1.8.5.3	anaerobic dimethyl sulfoxide reductase subunit A	18.00441963	-0.464366216	0.867960133	-0.535008692	0.592643847	0.996994191
5.4.3.2	lysine 2,3-aminomutase	170.8248805	-0.52130462	0.355924	-1.464651499	0.143016024	0.996994191
2.6.1.2	alanine transaminase	68.37319459	-0.885482585	0.578003074	-1.531968643	0.125530173	0.996994191
1.3.1.74	2-alkenal reductase	1.219066924	0.45566172	3.790303398	0.120217743	0.904310663	0.996994191
3.1.3.48	protein-tyrosine phosphatase	512.7382816	-0.241828329	0.280694697	-0.861535083	0.388943407	0.996994191
5.4.99.29	tRNA pseudouridine32 synthase / 23S rRNA pseudouridine746 s	73.12648985	-0.359555097	0.409966117	-0.877036132	0.380467013	0.996994191
3.1.21.4	type II restriction enzyme	23.82095201	1.048968125	0.66814164	1.569978672	0.116420073	0.996994191
2.3.1.97	glycylpeptide N-tetradecanoyltransferase	0.267820405	3.726658981	4.072976819	0.914971812	0.360206428	0.996994191
4.6.1.16	tRNA-intron endonuclease, archaea type	0.575809695	3.823674836	4.063130127	0.9410663	0.346670884	0.996994191
1.18.1.3	3-phenylpropionate/trans-cinnamate dioxygenase ferredoxin re	0.132218179	2.851346258	4.093899451	0.696486636	0.486124123	0.996994191
3.1.6.6	choline-sulfatase	12.21331139	-1.032357782	0.813350879	-1.269264974	0.204346574	0.996994191
1.8.5.2	thiosulfate dehydrogenase [quinone] small subunit	14.79835306	-0.856764483	0.804376337	-1.0651289	0.286817603	0.996994191
6.3.1.8	glutathionylspermidine amidase/synthetase	1.535270693	1.883074867	3.043164053	0.618788483	0.536055711	0.996994191
2.3.1.9	acetyl-CoA C-acetyltransferase	972.9845713	-0.228873429	0.629423717	-0.363623776	0.71613897	0.996994191
1.1.1.28	D-lactate dehydrogenase	178.3211075	0.232221082	0.231491276	1.003152629	0.315787225	0.996994191
2.7.1.167	D-beta-D-heptose 7-phosphate kinase / D-beta-D-heptose 1-ph	16.49374106	-1.037872963	0.746008152	-1.391235418	0.16415405	0.996994191
4.3.2.2	adenylosuccinate lyase	625.3639552	0.037489316	0.221577616	0.169192706	0.865645067	0.996994191
3.2.1.139	alpha-glucuronidase	70.11148679	-0.31437777	0.643955535	-0.488197946	0.625409639	0.996994191
4.2.1.77	trans-L-3-hydroxyproline dehydratase	0.959760061	-0.540335658	4.123244091	-0.131046246	0.895738724	0.996994191
2.3.1.89	tetrahydrodipicolinate N-acetyltransferase	3.970360467	0.565138282	1.169230012	0.483342265	0.628852735	0.996994191
1.5.1.2	pyrroline-5-carboxylate reductase	309.2710635	-0.499433175	0.333414142	-1.497936387	0.134149779	0.996994191
1.7.2.5	nitric oxide reductase subunit B	0.671618783	2.540820141	4.104801353	0.618987357	0.535924688	0.996994191
2.4.1.11	glycogen synthase	62.34508966	0.262783261	0.528208975	0.497498668	0.618837442	0.996994191
2.8.1.6	biotin synthase	192.6670322	-0.124305974	0.316218932	-0.393100986	0.694244896	0.996994191
2.7.1.71	shikimate kinase	321.2596761	-0.339715421	0.275186329	-1.234492361	0.217019492	0.996994191
3.5.1.47	N-acetyldiaminopimelate deacetylase	2.679831447	0.660361752	1.993219905	0.331304013	0.740414861	0.996994191

1.2.5.1	pyruvate dehydrogenase (quinone)	24.97110696	0.612525922	0.526610644	1.163147629	0.24476961	0.996994191
2.4.2.52	triphosphoribosyl-dephospho-CoA synthase	26.49897562	0.241574339	0.643276307	0.375537443	0.707260805	0.996994191
1.3.4.1	fumarate reductase (CoM/CoB) subunit A	11.21361449	2.167363243	1.398073321	1.550250055	0.12108151	0.996994191
6.2.1.5	succinyl-CoA synthetase alpha subunit	201.7652483	0.361019729	0.717272811	0.503322757	0.614737369	0.996994191
4.2.1.136	ADP-dependent NAD(P)H-hydrate dehydratase	296.924103	-0.140208937	0.196411233	-0.713853961	0.475317488	0.996994191
2.7.1.153	phosphatidylinositol-4,5-bisphosphate 3-kinase catalytic subuni	0.107128162	2.905607862	4.094274383	0.709675901	0.47790514	0.996994191
1.5.1.39	FMN reductase [NAD(P)H]	18.48410048	0.333452138	0.955309658	0.349051363	0.72705075	0.996994191
1.1.1.281	GDP-4-dehydro-6-deoxy-D-mannose reductase	14.17185845	0.997594382	0.640154137	1.558365908	0.119146534	0.996994191
6.3.1.10	adenosylcobinamide-phosphate synthase	146.8807414	-0.150568391	0.31523524	-0.477638195	0.63290774	0.996994191
5.4.2.7	phosphopentomutase	271.5281824	-0.090428392	0.284125635	-0.318269036	0.750280869	0.996994191
2.7.1.190	aminoglycoside 2''-phosphotransferase	1.1071359	1.869901157	3.048641949	0.613355451	0.539641336	0.996994191
1.12.98.2	5,10-methenyltetrahydromethanopterin hydrogenase	33.13934147	2.717810401	1.149507004	2.36432696	0.018062863	0.996994191
2.7.1.36	mevalonate kinase	7.588783217	2.823592545	1.604021011	1.760321421	0.078353324	0.996994191
4.2.1.91	cyclohexadienyl dehydratase	1.859733319	0.71061703	2.04290814	0.347845807	0.727955986	0.996994191
3.5.4.39	GTP cyclohydrolase IV	4.206096909	2.174818826	1.64320308	1.323524069	0.185661157	0.996994191
5.3.1.8	mannose-6-phosphate isomerase	279.7365829	0.246392637	0.28891238	0.852828242	0.39375456	0.996994191
3.1.3.71	2-phosphosulfolactate phosphatase	9.214936184	0.65741771	0.862804274	0.761954628	0.446087084	0.996994191
2.7.11.33	[pyruvate, water dikinase]-phosphate phosphotransferase / [py	11.94070215	-1.207680594	0.843931713	-1.43101696	0.152425354	0.996994191
2.7.1.19	phosphoribulokinase	0.240767753	0.411045137	4.108600605	0.100045046	0.920308563	0.996994191
1.2.1.24	succinate-semialdehyde dehydrogenase	0.096307101	0.646944639	4.130400399	0.156630006	0.875536453	0.996994191
2.7.1.168	D-glycero-alpha-D-manno-heptose-7-phosphate kinase	31.71658101	-0.080222976	0.602604213	-0.133127141	0.894092831	0.996994191
4.1.3.42	2-dehydro-3-deoxyphosphogluconate aldolase / (4S)-4-hydroxy	365.4288842	0.448185666	0.288890054	1.551405662	0.120804499	0.996994191
1.3.99.31	phytoene desaturase	1.632348624	3.119400767	4.065906252	0.767209221	0.442957131	0.996994191
3.1.3.15	imidazoleglycerol-phosphate dehydratase / histidinol-phosphat	920.6179347	0.09050223	0.665668096	0.135956989	0.891855283	0.996994191
4.3.1.14	3-aminobutyryl-CoA ammonia-lyase	70.2832787	0.15584173	0.699691825	0.2227291	0.823746352	0.996994191
1.8.2.2	thiosulfate dehydrogenase	0.06577573	1.818740011	4.121422411	0.441289397	0.659003501	0.996994191
2.3.1.54	formate C-acetyltransferase	2143.677149	0.84888645	0.447645143	1.896337901	0.057915378	0.996994191
3.5.4.29	GTP cyclohydrolase IIa	5.074632583	4.24151552	2.251197541	1.884115206	0.059549394	0.996994191
5.3.4.1	protein disulfide-isomerase	8.650518897	2.660148526	1.346522813	1.97556885	0.04820363	0.996994191
2.4.1.117	dolichyl-phosphate beta-glucosyltransferase	0.15199015	2.050314349	4.101585174	0.499883402	0.61715718	0.996994191
4.1.3.40	chorismate--pyruvate lyase	0.298604074	2.491959129	4.06203249	0.613475923	0.539561698	0.996994191
3.1.11.1	exodeoxyribonuclease I	5.573815682	0.25932237	1.337609021	0.193870082	0.846277611	0.996994191
2.5.1.43	nicotianamine synthase	0.050191057	1.314825092	4.126913272	0.318597704	0.750031595	0.996994191
3.1.3.87	2-hydroxy-3-keto-5-methylthiopentenyl-1-phosphate phosphat	0.051061294	1.428001729	4.141463361	0.344806076	0.730240165	0.996994191
2.3.1.235	minimal PKS ketosynthase (KS/KS alpha)	0.046441742	1.126749671	4.139547257	0.272191523	0.78547476	0.996994191
3.4.21.50	lysyl endopeptidase	0.022430994	1.099249132	4.140903343	0.265461191	0.790654203	0.996994191
4.4.1.24	(2R)-sulfolactate sulfo-lyase subunit alpha	10.38634163	-0.133440404	1.153161371	-0.115717026	0.907876812	0.996994191
3.4.14.4	dipeptidyl-peptidase III	183.6513868	-0.480778465	0.40946003	-1.174176794	0.240324219	0.996994191
3.4.21.105	rhomboid-related protein 1/2/3	57.76563614	-0.749442568	0.451675901	-1.659248514	0.097065725	0.996994191
3.1.6.-	#N/A	5.647174252	-1.401470623	1.048901665	-1.336131565	0.181506274	0.996994191
6.1.1.9	valyl-tRNA synthetase	675.7895904	-0.297426892	0.204742504	-1.452687581	0.146310517	0.996994191
2.7.4.25	CMP/dCMP kinase	386.0131937	0.266296524	0.27288339	0.975861975	0.329132868	0.996994191
1.2.1.59	glyceraldehyde-3-phosphate dehydrogenase (NAD(P))	6.878986232	2.617894333	1.76205197	1.485707787	0.13735643	0.996994191
3.5.2.9	5-oxoprolinase (ATP-hydrolysing)	0.116409678	1.396153691	4.142298814	0.337048039	0.736080687	0.996994191
1.1.1.25	shikimate dehydrogenase	261.1601688	0.121380234	0.271838166	0.446516527	0.655224177	0.996994191
4.2.1.59	3-hydroxyacyl-[acyl-carrier protein] dehydratase / trans-2-dece	276.4956797	-0.02428191	0.250911627	-0.096774749	0.922905277	0.996994191
2.3.1.182	(R)-citramalate synthase	90.27086847	0.208512749	0.27092509	0.76963248	0.441517932	0.996994191
4.1.1.65	phosphatidylserine decarboxylase	135.2245112	0.046083278	0.368091551	0.125195151	0.900369056	0.996994191
3.4.21.92	ATP-dependent Clp protease, protease subunit	561.0943661	-0.071383029	0.245321341	-0.290977657	0.771068408	0.996994191
2.7.7.61	holo-ACP synthase	27.06562961	-0.11124318	0.763141515	-0.145770054	0.884102917	0.996994191
3.3.2.1	bifunctional isochorismate lyase / aryl carrier protein	0.250956274	2.243253004	4.068610089	0.551356103	0.581389586	0.996994191
1.2.1.71	succinylglutamic semialdehyde dehydrogenase	0.048153551	1.099249132	4.140903343	0.265461191	0.790654203	0.996994191
2.7.9.2	pyruvate, water dikinase	67.74853073	-0.286824046	0.546732301	-0.524615145	0.599850756	0.996994191
1.2.1.10	acetaldehyde dehydrogenase (acetylating)	400.4918272	-0.063618177	0.478915145	-0.132838098	0.894321424	0.996994191
1.3.1.43	cyclohexadieny/prephenate dehydrogenase	3.773900336	-0.694112537	1.867133141	-0.371753102	0.710076684	0.996994191
4.1.3.3	N-acetylneuraminate lyase	294.7456095	-0.184736324	0.443449526	-0.416589293	0.676978842	0.996994191
5.4.99.1	methylaspartate mutase sigma subunit	67.78198055	-0.417676864	0.644928882	-0.647632438	0.517222712	0.996994191
2.3.3.14	homocitrate synthase	6.772603954	2.345807325	1.166228353	2.011447689	0.044278191	0.996994191
6.3.4.14	acetyl-CoA carboxylase / biotin carboxylase 2	281.1120721	0.124072547	0.209710232	0.591638022	0.554093007	0.996994191
1.-.-.-	#N/A	90.00714768	-0.283538139	0.442936918	-0.640132098	0.522086723	0.996994191
1.16.1.1	mercuric reductase	0.505665624	0.737934509	4.076418464	0.18102521	0.856347788	0.996994191
1.16.3.1	bacterioferritin	74.72166482	-0.480018038	0.537468723	-0.893108785	0.371798919	0.996994191
4.1.2.19	rhamnulose-1-phosphate aldolase	135.5495071	-0.636370297	0.456037332	-1.395434656	0.162884807	0.996994191
2.5.1.129	flavin prenyltransferase	25.08112497	-0.712905238	0.633430687	-1.125466848	0.260391258	0.996994191
1.1.1.271	GDP-L-fucose synthase	181.2243692	0.415685323	0.238534155	1.742665838	0.081392002	0.996994191
5.1.99.7	D-erythro-7,8-dihydroneopterin triphosphate epimerase	0.144460652	0.533493742	4.118712294	0.129529257	0.896938876	0.996994191
3.4.17.11	glutamate carboxypeptidase	21.84682526	-0.335362243	0.62106466	-0.539979593	0.589211106	0.996994191
2.3.1.243	lauroyl-Kdo2-lipid IVA myristoyltransferase	0.273484821	0.713493011	4.10837095	0.173668108	0.862126303	0.996994191
1.8.4.14	L-methionine (R)-S-oxide reductase	1.203925453	4.910110791	4.059756419	1.209459456	0.226486378	0.996994191
4.4.1.15	D-cysteine desulfhydrase	28.02213594	-0.563855442	0.423512065	-1.33137988	0.183064046	0.996994191
2.7.8.42	KDO II ethanolaminephosphotransferase	0.429078891	0.719509081	4.088604974	0.175979114	0.860310351	0.996994191
2.6.1.59	dTDP-4-amino-4,6-dideoxygalactose transaminase	72.97848554	-0.193405178	0.344425232	-0.561530224	0.574436132	0.996994191
1.4.1.14	glutamate synthase (NADH)	1092.906852	-0.188949853	0.136178314	-1.387517929	0.165283888	0.996994191
3.1.3.7	3'(2'), 5'-bisphosphate nucleotidase	529.7510404	0.737594729	0.306459992	2.406822254	0.016092	0.996994191
2.4.1.315	processive 1,2-diacylglycerol beta-glucosyltransferase	113.4372334	-0.041609842	0.385534978	-0.10792754	0.914053171	0.996994191
4.2.1.41	5-dehydro-4-deoxyglucarate dehydratase	0.227201514	0.421160751	4.11502619	0.10234704	0.918481219	0.996994191
3.2.1.84	#N/A	0.107128162	2.905607862	4.094274383	0.709675901	0.47790514	0.996994191
1.3.7.7	light-independent protochlorophyllide reductase subunit L	0.248906341	1.43931336	4.128805281	0.348602867	0.727387475	0.996994191
1.1.1.61	4-hydroxybutyrate dehydrogenase	46.40904916	0.232469429	0.408248147	0.569431682	0.569063221	0.996994191
3.6.3.44	#N/A	1.312530107	4.223803246	2.706789666	1.560447529	0.118654161	0.996994191
2.7.7.82	CMP-N,N'-diacetyllegionaminic acid synthase	8.972758846	-0.720175946	0.608788421	-1.182965905	0.23682266	0.996994191
3.2.1.65	levanase	13.77925283	-0.109949903	0.937216546	-0.117315367	0.906610148	0.996994191
2.7.4.1	polyphosphate kinase	532.0088662	-0.330576314	0.196742912	-1.6802451	0.092909638	0.996994191
3.1.-.-	#N/A	7215.830595	0.183219362	0.190143141	0.963586489	0.335253283	0.996994191
1.6.4.-	#N/A	210.9728632	-0.201692095	0.492666038	-0.409389078	0.682254154	0.996994191
3.5.1.18	succinyl-diaminopimelate desuccinylase	238.9037885	-0.038752723	0.199767662	-0.19398897	0.846184519	0.996994191
2.3.3.-	#N/A	4.148446599	3.642401746	2.053582764	1.773681495	0.076115849	0.996994191

3.1.3.41	4-nitrophenyl phosphatase	2.475652433	1.841630334	1.787348835	1.03036984	0.302836425	0.996994191
2.3.1.48	chromodomain protein Y	4.949563951	0.94555973	1.438757072	0.657205965	0.51104849	0.996994191
6.3.2.29	cyanophycin synthetase	4.102299911	2.060207873	3.74717898	0.549802367	0.582454935	0.996994191
2.4.2.18	anthranilate phosphoribosyltransferase	118.1962522	-0.665506136	0.475469074	-1.399683329	0.161608168	0.996994191
2.4.1.293	GalNAc5-diNAcBac-PP-undecaprenol beta-1,3-glucosyltransfera	0.450882135	2.258444539	4.087080304	0.552581396	0.580550084	0.996994191
3.2.1.23	beta-galactosidase	3131.489228	-0.188245618	0.142521512	-1.320822488	0.18656056	0.996994191
4.2.1.55	3-hydroxybutyryl-CoA dehydratase	14.441369	1.309964113	0.972633385	1.346822075	0.178037543	0.996994191
4.2.1.7	altronate hydrolase	427.3465533	-0.105521497	0.384442342	-0.274479384	0.783716245	0.996994191
3.5.1.88	peptide deformylase	403.7885556	0.151327315	0.255084302	0.593244325	0.553017652	0.996994191
2.1.1.177	23S rRNA (pseudouridine1915-N3)-methyltransferase	205.3404314	0.131084625	0.258638221	0.506826194	0.612276776	0.996994191
6.5.1.6	DNA ligase 1	16.24694701	1.603038566	0.877809995	1.826179441	0.067823237	0.996994191
2.6.1.42	branched-chain amino acid aminotransferase	427.0846235	0.232209298	0.292157587	0.79480838	0.426724975	0.996994191
2.6.1.16	glucosamine---fructose-6-phosphate aminotransferase (isomeri	625.9817941	0.204845128	0.21001173	0.975398507	0.329362624	0.996994191
5.3.1.13	arabinose 5-phosphate isomerase	81.00631304	-0.213329952	0.335522924	-0.635813343	0.524898089	0.996994191
5.4.99.21	23S rRNA pseudouridine2604 synthase	55.92207997	-0.536387234	0.432949133	-1.238915134	0.215376928	0.996994191
3.4.24.25	vibriolysin	14.58659153	2.019156782	2.676355694	0.754442612	0.450583492	0.996994191
3.1.4.3	phospholipase C	2.840339435	1.53815105	2.622475623	0.586526348	0.557521861	0.996994191
4.1.1.73	tartrate dehydrogenase/decarboxylase / D-malate dehydrogen	0.660399619	-0.297704537	4.125124233	-0.072168623	0.942467715	0.996994191
2.4.2.-	#N/A	193.4053107	-0.152557924	0.24420058	-0.624723838	0.532152325	0.996994191
1.14.13.1	salicylate hydroxylase	0.048153551	1.099249132	4.140903343	0.265461191	0.790654203	0.996994191
1.1.1.303	(R,R)-butanediol dehydrogenase / meso-butanediol dehydroger	5.627014574	0.715809332	1.680143729	0.426040534	0.67007831	0.996994191
3.8.1.3	haloacetate dehalogenase	2.266409738	-0.656952846	1.928072977	-0.340730281	0.733306641	0.996994191
1.1.1.2	alcohol dehydrogenase (NADP+)	2.742843588	0.545604691	1.596488154	0.341753047	0.732536747	0.996994191
3.4.14.11	X-Pro dipeptidyl-peptidase	20.93093077	-1.614136782	0.786318128	-2.052778289	0.040094085	0.996994191
1.5.1.3	dihydrofolate reductase	420.2366272	1.352835567	0.614240209	2.202453612	0.027633282	0.996994191
2.7.11.26	glycogen synthase kinase 3 beta	0.160692243	3.261377394	4.083493213	0.798673397	0.424479817	0.996994191
6.2.1.42	3-oxocholest-4-en-26-oate---CoA ligase	0.197654718	3.379908499	4.08443805	0.827508817	0.407948727	0.996994191
2.5.1.17	cob(I)alamin adenosyltransferase	297.5379903	-0.4636645	0.299542872	-1.547906973	0.121644696	0.996994191
2.1.1.62	#N/A	0.107128162	2.905607862	4.094274383	0.709675901	0.47790514	0.996994191
2.7.1.48	uridine kinase	1798.770566	-0.045933735	0.611767742	-0.075083617	0.940148183	0.996994191
2.5.1.30	heptaprenyl diphosphate synthase	102.0691133	-0.551895201	0.481522069	-1.146147262	0.251734225	0.996994191
6.2.1.26	O-succinylbenzoic acid---CoA ligase	43.02822346	0.316782315	0.387782848	0.816906465	0.413981883	0.996994191
1.3.1.83	geranylgeranyl diphosphate/geranylgeranyl-bacteriochlorophyl	0.022430994	1.099249132	4.140903343	0.265461191	0.790654203	0.996994191
3.2.2.4	AMP nucleosidase	42.98149885	-0.135043904	0.586901429	-0.230096398	0.818016864	0.996994191
3.4.19.3	pyroglutamyl-peptidase	36.92515586	0.043111294	0.42420943	0.101627382	0.919052444	0.996994191
1.1.1.43	phosphogluconate 2-dehydrogenase	0.089723976	0.638106246	4.129588845	0.154520527	0.877199327	0.996994191
1.2.7.7	2-oxoisovalerate ferredoxin oxidoreductase, alpha subunit	19.38805552	1.867785401	1.401418134	1.332782384	0.182603228	0.996994191
4.1.3.34	citrate lyase subunit beta / citryl-CoA lyase	88.71502524	-0.150032301	0.477159086	-0.31442826	0.753195796	0.996994191
3.6.3.5	Cd2+/Zn2+-exporting ATPase	686.2195542	0.109065879	0.411120066	0.265289604	0.790786373	0.996994191
5.3.1.26	galactose-6-phosphate isomerase	2.630607547	1.22999356	1.993603658	0.616969955	0.537254543	0.996994191
5.1.3.4	#N/A	185.3708773	-0.265410393	0.31023461	-0.8555151	0.392266049	0.996994191
3.2.1.136	glucuronoarabinoxylan endo-1,4-beta-xylanase	13.2680149	-0.087515309	0.647170985	-0.135227491	0.892432012	0.996994191
5.1.3.35	2-epi-5-epi-valiolone 7-phosphate 2-epimerase	0.265720381	1.172861572	4.114713166	0.285040907	0.775612802	0.996994191
2.7.4.14	UMP-CMP kinase	0.642768973	4.558254324	4.061086832	1.122422276	0.261682938	0.996994191
2.7.8.33	UDP-GlcNAc:undecaprenyl-phosphate/decaprenyl-phosphate G	45.64451026	-0.361961389	0.642245986	-0.563586846	0.573035341	0.996994191
2.7.1.28	triose/dihydroxyacetone kinase / FAD-AMP lyase (cyclizing)	5.825853036	0.704699383	4.07553071	0.172909845	0.862722293	0.996994191
4.1.1.17	ornithine decarboxylase	21.20996489	-0.051848943	0.875179306	-0.059243794	0.952757928	0.996994191
5.4.2.12	2,3-bisphosphoglycerate-independent phosphoglycerate mutas	1248.632008	-0.155971198	0.349415985	-0.446376826	0.65532507	0.996994191
2.7.4.7	hydroxymethylpyrimidine/phosphomethylpyrimidine kinase / t	174.8667027	-0.403986534	0.30214579	-1.337058291	0.18120361	0.996994191
1.1.1.330	17beta-estradiol 17-dehydrogenase / very-long-chain 3-oxoacyl	0.374948568	4.041750821	4.06761596	0.993641204	0.320397576	0.996994191
2.1.1.43	histone-lysine N-methyltransferase ASH1L	0.160692243	3.261377394	4.083493213	0.798673397	0.424479817	0.996994191
2.7.4.29	Kdo2-lipid A phosphotransferase	0.202890383	2.316301216	4.115704853	0.562795754	0.573573972	0.996994191
2.6.1.83	LL-diaminopimelate aminotransferase	321.5540511	-0.042336992	0.227197646	-0.186344325	0.85217475	0.996994191
3.1.4.16	2',3'-cyclic-nucleotide 2'-phosphodiesterase / 3'-nucleotidase	234.785924	0.565421757	0.329990145	1.713450435	0.086629705	0.996994191
2.4.1.4	amylosucrase	278.8244437	-0.560868843	0.483319997	-1.160450317	0.245865511	0.996994191
4.6.1.1	adenylate cyclase	23.29535609	-0.260048038	0.635808968	-0.409003413	0.682537156	0.996994191
3.1.21.-	#N/A	822.8588976	0.975476372	0.504587262	1.933216405	0.053209545	0.996994191
3.2.1.2	beta-amylase	0.22353784	2.783192181	4.107731187	0.677549736	0.498057225	0.996994191
4.1.1.55	4,5-dihydroxyphthalate decarboxylase	0.043850487	1.769618963	4.123058137	0.429200585	0.667777258	0.996994191
2.5.1.15	dihydropteroate synthase	139.5745534	-0.139237853	0.241568187	-0.576391514	0.564350579	0.996994191
2.7.7.49	RNA-directed DNA polymerase	107.7899205	-0.063720382	0.383387948	-0.166203403	0.867996882	0.996994191
4.2.2.24	rhamnogalacturonan exolyase	3.284691663	-0.27666351	1.660580696	-0.166606483	0.867679692	0.996994191
2.6.1.55	taurine---2-oxoglutarate transaminase	47.87404651	0.660916699	0.731624193	0.903355446	0.366337278	0.996994191
1.8.4.10	phosphoadenosine phosphosulfate reductase	26.55279816	-1.124557802	0.728764169	-1.543102488	0.122805911	0.996994191
1.5.1.5	methylenetetrahydrofolate dehydrogenase (NADP+) / metheny	432.3435048	0.410837804	0.245749412	1.671775329	0.094568634	0.996994191
2.8.4.1	#N/A	294.1217901	1.004606742	1.2375444	0.811774302	0.41692115	0.996994191
2.8.1.10	thiazole synthase	81.74543984	0.032757524	0.385062619	0.085070641	0.932205231	0.996994191
3.6.3.20	sn-glycerol 3-phosphate transport system ATP-binding protein	11.33154221	0.927660183	0.987074261	0.939807895	0.347316108	0.996994191
2.3.1.41	3-oxoacyl-[acyl-carrier-protein] synthase I	64.89992041	-0.248926241	0.553649002	-0.449610205	0.652991529	0.996994191
2.6.1.11	acetylornithine aminotransferase	668.5746803	-0.482180503	0.516097401	-0.934281984	0.350158455	0.996994191
1.3.5.2	dihydroorotate dehydrogenase	21.83162396	0.186035524	0.654771961	0.284122619	0.776316414	0.996994191
2.7.7.19	poly(A) polymerase	28.18380502	-1.000294982	0.700405166	-1.428166196	0.153244028	0.996994191
4.3.3.6	pyridoxal 5'-phosphate synthase pdxS subunit	136.5195282	-0.124034161	0.338696457	-0.366210389	0.71420809	0.996994191
1.1.1.373	4-hydroxybutyrate dehydrogenase / sulfolactaldehyde 3-reduct	0.167610981	1.016961379	4.141564051	0.245550079	0.806030538	0.996994191
2.1.1.226	23S rRNA (cytidine1920-2'-O)/16S rRNA (cytidine1409-2'-O)-me	278.8082903	0.5029882	0.308932364	1.628149908	0.103493103	0.996994191
5.4.99.16	maltose alpha-D-glucosyltransferase / alpha-amylase	86.28108937	0.151011856	0.535577197	0.281960951	0.777973452	0.996994191
3.1.3.21	glycerol-1-phosphatase	0.231939278	0.952858335	4.106808223	0.232019194	0.816523106	0.996994191
1.3.1.1	dihydropyrimidine dehydrogenase (NAD+) subunit PreT	55.39741083	-0.041505124	0.473923221	-0.087577738	0.930212297	0.996994191
4.1.2.25	7,8-dihydroneopterin aldolase/epimerase/oxygenase	88.0690056	-0.315037211	0.293279287	-1.074188408	0.282738247	0.996994191
2.3.1.168	2-oxoisovalerate dehydrogenase E2 component (dihydrolipoyl t	0.137221322	0.954716522	4.117558757	0.231864699	0.816643103	0.996994191
3.6.3.12	K+-transporting ATPase ATPase A chain	131.978621	-1.400303166	0.638630155	-2.192666844	0.028331391	0.996994191
3.1.1.1	carboxylesterase 1	6.423676539	1.89901486	1.022193262	1.85778456	0.063199615	0.996994191
2.3.1.128	#N/A	202.8758722	-0.540227427	0.463249297	-1.166169988	0.243545724	0.996994191
2.4.1.80	ceramide glucosyltransferase	0.130175812	1.292301483	4.116808575	0.313908568	0.753590483	0.996994191
2.5.1.120	aminodeoxyfutalosine synthase	14.44656408	-0.555423071	0.772917109	-0.718606257	0.472383557	0.996994191
6.3.3.3	dethiobiotin synthetase	42.7523479	-0.825921004	0.557355685	-1.481856249	0.138378549	0.996994191
3.6.1.11	exopolyphosphatase	219.8727794	0.17171314	0.456906632	0.375816694	0.707053176	0.996994191

2.1.1.37	DNA (cytosine-5)-methyltransferase 1	626.4275744	-0.121683448	0.369308533	-0.329489943	0.741785394	0.996994191
4.3.99.3	7-carboxy-7-deazaguanine synthase	27.63428654	-0.633820066	0.591866896	-1.070882778	0.28422214	0.996994191
3.2.2.23	#N/A	37.572242	0.283939172	0.701863501	0.404550417	0.685808011	0.996994191
3.4.23.52	archaeal preflagellin peptidase FlaK	0.111483345	1.158955833	4.124909527	0.280965152	0.778737128	0.996994191
3.2.1.64	levanbiose-producing levanase	1.029822388	1.991276228	4.058001107	0.490703717	0.623636017	0.996994191
3.1.22.4	crossover junction endodeoxyribonuclease RuvC	151.494518	0.316499984	0.30642254	1.032887411	0.301656591	0.996994191
2.7.8.5	CDP-diacylglycerol---glycerol-3-phosphate 3-phosphatidyltransf	283.7050059	0.29408979	0.233169391	1.261270997	0.207211227	0.996994191
1.4.1.21	aspartate dehydrogenase	44.11897346	0.085591718	0.400753251	0.213577103	0.830876857	0.996994191
2.6.1.102	perosamine synthetase	3.986928839	1.225285885	1.698408077	0.72143197	0.470643783	0.996994191
3.2.1.177	alpha-D-xyloside xylohydrolase	556.3891845	-0.161761854	0.232221741	-0.696583592	0.486063427	0.996994191
1.3.1.9	enoyl-acyl-carrier protein] reductase I	337.7412044	-0.259594293	0.182824288	-1.419911411	0.155633473	0.996994191
2.7.1.12	gluconokinase	2.191401162	-0.528171347	1.695253875	-0.31155885	0.755375812	0.996994191
2.1.1.266	23S rRNA (adenine2030-N6)-methyltransferase	8.85099374	0.97930921	1.27645075	0.767212687	0.442955071	0.996994191
4.1.2.4	deoxyribose-phosphate aldolase	354.3068753	0.250436472	0.246928795	1.014205218	0.310484839	0.996994191
3.4.13.21	dipeptidase E	57.55391717	0.418932013	0.46280676	0.905198562	0.365360204	0.996994191
1.2.1.70	glutamyl-tRNA reductase	52.01114862	-0.1937312	0.398585243	-0.486047096	0.626933775	0.996994191
2.4.2.28	5'-methylthioadenosine phosphorylase	7.435936035	1.02262708	0.764568887	1.337521179	0.181052574	0.996994191
6.1.1.23	nondiscriminating aspartyl-tRNA synthetase	52.43350952	-0.689995756	0.592312306	-1.164918825	0.244051853	0.996994191
2.7.2.15	propionate kinase	0.163600083	0.499894041	4.115639736	0.12146205	0.903325072	0.996994191
2.7.1.3	ketohexokinase	0.044861988	0.80861821	4.141284466	0.195257828	0.84519111	0.996994191
6.1.1.20	#N/A	872.735898	-0.150179152	0.260066936	-0.577463457	0.563626418	0.996994191
1.8.99.2	#N/A	150.0364969	-0.280132062	0.41714377	-0.671547995	0.501871495	0.996994191
2.1.1.170	16S rRNA (guanine527-N7)-methyltransferase	224.0979338	0.024787365	0.277607157	0.089289358	0.928851951	0.996994191
2.4.1.230	kojibiose phosphorylase	6.165137258	0.314017513	1.305071666	0.240613233	0.809854893	0.996994191
1.2.7.-	#N/A	6148.42736	-0.061183172	0.523536905	-0.11686506	0.906966986	0.996994191
1.2.7.11	2-oxoglutarate/2-oxoacid ferredoxin oxidoreductase subunit al	1113.830126	0.347105175	0.351154205	0.98846937	0.322922828	0.996994191
4.1.2.48	threonine aldolase	240.4281868	0.102373945	0.244889764	0.418040931	0.675917191	0.996994191
2.1.1.319	type I protein arginine methyltransferase	0.353049384	3.474037201	4.075919419	0.85233216	0.394029761	0.996994191
1.8.2.3	sulfide dehydrogenase [flavocytochrome c] flavoprotein chain	3.046267745	2.02831395	2.402692861	0.844183617	0.398566808	0.996994191
2.3.1.109	arginine N-succinyltransferase	0.096307101	0.646944639	4.130400399	0.156630006	0.875536453	0.996994191
2.3.2.2	gamma-glutamyltranspeptidase / glutathione hydrolase	29.91949374	0.651857483	0.607836016	1.072423262	0.283529964	0.996994191
3.1.2.12	S-formylglutathione hydrolase	1.707218919	2.449231659	2.869070367	0.853667337	0.393289337	0.996994191
3.4.15.5	peptidyl-dipeptidase Dcp	258.6791263	-0.307703986	0.281331664	-1.093741038	0.27406857	0.996994191
2.7.14.1	protein arginine kinase	100.4784369	0.577047617	0.551137107	1.04701282	0.295093666	0.996994191
2.1.1.196	cobalt-precorrin-6B (C15)-methyltransferase	4.720469386	1.768392723	1.174574671	1.505560069	0.132180146	0.996994191
5.4.2.3	phosphoacetylglucosamine mutase	0.208845794	2.509094237	4.090524616	0.613391795	0.53961731	0.996994191
3.5.1.116	ureidoglycolate amidohydrolase	0.579608174	3.018275643	4.068100237	0.741937383	0.458125275	0.996994191
2.8.3.25	bile acid CoA-transferase	19.80063959	-0.623282358	0.745002126	-0.836618226	0.402807197	0.996994191
6.5.1.7	DNA ligase 1	16.24694701	1.603038566	0.877809995	1.826179441	0.067823237	0.996994191
2.1.1.227	23S rRNA (cytidine1920-2'-O)/16S rRNA (cytidine1409-2'-O)-me	278.8082903	0.5029882	0.308932364	1.628149908	0.103493103	0.996994191
1.4.5.1	D-amino-acid dehydrogenase	5.40917928	-1.261867855	2.457979465	-0.513376077	0.607688277	0.996994191
1.17.1.4	xanthine dehydrogenase molybdenum-binding subunit	289.3415095	0.258341014	0.404900553	0.638035716	0.52345043	0.996994191
3.4.11.10	bacterial leucyl aminopeptidase	5.455731727	0.449390871	1.371952424	0.327555725	0.743247603	0.996994191
5.3.1.14	L-rhamnose isomerase	112.3655337	0.269233047	0.301941565	0.89167269	0.372568396	0.996994191
3.4.22.41	cathepsin F	2.347625857	1.896956113	4.079778783	0.464965434	0.64195621	0.996994191
1.1.1.267	1-deoxy-D-xylulose-5-phosphate reductoisomerase	366.5098855	-0.188393524	0.157186603	-1.198534226	0.230709107	0.996994191
2.3.1.79	maltose O-acetyltransferase	148.2896748	0.085525399	0.275011659	0.310988268	0.755809541	0.996994191
2.3.1.82	aminoglycoside 6'-N-acetyltransferase	5.843599705	1.969174631	1.454999651	1.353384951	0.175932683	0.996994191
3.1.4.52	c-di-GMP phosphodiesterase	50.01167139	-1.271295387	0.88716607	-1.43298468	0.151862215	0.996994191
2.5.1.97	pseudaminic acid synthase	27.63825338	-0.578754803	0.533030771	-1.085781223	0.277575796	0.996994191
2.8.2.22	arylsulfate sulfotransferase	15.39709927	-1.076318036	0.69197299	-1.555433596	0.119842838	0.996994191
3.5.4.26	diaminohydroxyphosphoribosylaminopyrimidine deaminase	165.0856682	-0.46899328	0.349018266	-1.343749957	0.179029257	0.996994191
2.7.6.3	2-amino-4-hydroxy-6-hydroxymethyldihydropteridine diphosph	143.2206681	-0.255237309	0.235958204	-1.08170559	0.279383368	0.996994191
2.3.1.118	N-hydroxyarylamine O-acetyltransferase	9.67675098	-1.566855881	1.433457871	-1.093060293	0.274367328	0.996994191
5.1.3.22	L-ribulose-5-phosphate 3-epimerase	5.033868584	0.092933688	1.515253268	0.061332115	0.951094715	0.996994191
2.3.2.16	peptidoglycan pentaglycine glycine transferase (the first glycine	0.052431119	0.78195638	4.139114155	0.188918776	0.850156477	0.996994191
5.99.1.2	#N/A	1730.88601	0.08978316	0.379827164	0.236378986	0.813138601	0.996994191
2.1.1.90	methanol---5-hydroxybenzimidazolylcobamide Co-methyltransf	10.03781092	1.471873895	1.576045041	0.933903446	0.350353701	0.996994191
3.4.19.11	g-D-glutamyl-meso-diaminopimelate peptidase	66.39931612	-0.263452392	0.532340729	-0.494894299	0.620674735	0.996994191
2.4.99.18	dolichyl-diphosphooligosaccharide---protein glycosyltransferase	15.30748849	2.280124612	1.204387836	1.893181368	0.058333754	0.996994191
2.7.1.201	PTS system, trehalose-specific IIA component	10.57361883	-1.347363498	0.850551117	-1.584106434	0.1131695	0.996994191
1.1.1.57	fructuronate reductase	269.3151576	-0.605284282	0.331518464	-1.825793574	0.067881362	0.996994191
3.5.1.-	#N/A	1932.988082	-0.615790407	0.896470018	-0.686905747	0.492142125	0.996994191
2.7.1.23	NAD+ kinase	358.9369047	0.334570003	0.265597813	1.259686587	0.207782446	0.996994191
1.4.1.1	alanine dehydrogenase	179.0955202	0.110078921	0.340221347	0.323550892	0.746278072	0.996994191
2.4.1.15	trehalose 6-phosphate synthase	28.28681294	0.034998947	0.505740049	0.069203432	0.944827691	0.996994191
2.7.7.-	#N/A	701.1796075	-0.225951147	0.21259859	-1.062806422	0.287869746	0.996994191
2.4.99.15	3-deoxy-D-manno-octulosonic-acid transferase	102.8745696	-0.356249249	0.359208676	-0.99176126	0.321313999	0.996994191
1.17.7.3	(E)-4-hydroxy-3-methylbut-2-enyl-diphosphate synthase	384.5079502	-0.096213522	0.187826966	-0.512245521	0.608479188	0.996994191
5.4.99.25	tRNA pseudouridine55 synthase	364.7005709	-0.017581351	0.251620902	-0.069872378	0.944295239	0.996994191
2.7.1.163	hygromycin-B 4-O-kinase	0.732726104	3.163118751	4.055551203	0.779947926	0.435421527	0.996994191
2.7.1.76	deoxyadenosine kinase	0.485024588	2.476627872	4.071758115	0.608245333	0.543024772	0.996994191
3.6.1.9	ectonucleotide pyrophosphatase/phosphodiesterase family me	9.120822582	-0.079494382	1.223646554	-0.06496515	0.948201748	0.996994191
1.13.11.16	2,3-dihydroxyphenylpropionate 1,2-dioxygenase	0.620913234	-1.447017004	4.086659869	-0.354083053	0.723276642	0.996994191
2.5.1.101	N,N'-diacetyllegionaminate synthase	17.20160115	0.134599179	0.516542478	0.260577173	0.794418595	0.996994191
2.7.7.76	molybdenum cofactor cytidylyltransferase	2517.858965	1.580031693	1.284905268	1.229687303	0.218814223	0.996994191
3.2.2.27	uracil-DNA glycosylase	169.0721726	0.125793778	0.251203577	0.500764281	0.616537026	0.996994191
1.1.1.308	sulfopropanediol 3-dehydrogenase	0.04941368	2.308811263	4.121547766	0.560180639	0.575356231	0.996994191
2.3.3.1	citrate synthase	736.3133749	0.104265235	0.207945849	0.501405705	0.616085626	0.996994191
3.1.26.11	ribonuclease Z	239.7056231	-0.237635122	0.363490117	-0.653759513	0.513266755	0.996994191
1.2.1.38	N-acetyl-gamma-glutamyl-phosphate reductase	221.1370935	-0.050964308	0.33656989	-0.151422661	0.879642315	0.996994191
2.3.1.245	3-hydroxy-5-phosphonooxypentane-2,4-dione thiolase	50.11152848	0.139330985	0.800952786	0.173956552	0.861899608	0.996994191
3.1.25.-	#N/A	0.161565365	0.558556164	4.136706255	0.135024372	0.892592605	0.996994191
4.1.2.52	4-hydroxy-2-oxoheptanedioate aldolase	10.54245838	0.734547918	0.835772901	0.878884583	0.379463859	0.996994191
3.4.17.4	Gly-Xaa carboxypeptidase	0.224781176	1.040030242	4.133370578	0.251617953	0.801336381	0.996994191
2.7.1.105	6-phosphofructo-2-kinase	0.267820405	3.726658981	4.072976819	0.914971812	0.360206428	0.996994191
2.5.1.49	O-acetylhomoserine (thiol)-lyase	620.572906	-0.197390556	0.259758274	-0.759900936	0.447313802	0.996994191

4.2.1.2	#N/A	786.4071845	0.11714027	0.218325085	0.536540589	0.591584992	0.996994191
3.11.1.1	phosphonoacetaldehyde hydrolase	23.83046873	-0.662424375	0.785527807	-0.843285711	0.399068672	0.996994191
1.8.98.1	#N/A	514.6685068	1.095345258	0.499190359	2.194243617	0.0282179	0.996994191
3.4.11.4	tripeptide aminopeptidase	365.2843562	-0.029000556	0.192180812	-0.150902455	0.880052664	0.996994191
2.7.1.193	PTS system, N-acetylglucosamine-specific IIA component	629.4791252	-0.251344045	0.492282357	-0.510568866	0.609652982	0.996994191
2.7.1.95	kanamycin kinase	6.400739295	-0.129841889	1.417587464	-0.091593565	0.927020964	0.996994191
4.1.1.22	histidine decarboxylase	0.451163486	2.01003889	4.093083524	0.491081816	0.623368582	0.996994191
4.1.1.35	UDP-glucuronate decarboxylase	71.42773928	0.161981095	0.32953806	0.491539869	0.623044661	0.996994191
1.3.8.-	#N/A	0.089381093	1.782761126	4.122597094	0.432436419	0.665424253	0.996994191
4.2.1.12	phosphogluconate dehydratase	0.541914096	-0.969279698	4.077450558	-0.237717094	0.812100524	0.996994191
3.4.11.9	Xaa-Pro aminopeptidase	757.7307781	-0.084846456	0.245510397	-0.345592111	0.729649275	0.996994191
4.1.2.-	#N/A	1.095079884	0.327107024	2.666824956	0.122657853	0.90237804	0.996994191
3.2.2.-	#N/A	321.9782873	0.160995902	0.446825105	0.360310781	0.718614738	0.996994191
1.1.1.133	dTDP-4-dehydrorhamnose reductase	412.266012	-0.198518757	0.201469848	-0.985352197	0.324451102	0.996994191
2.1.1.152	precorrin-6A synthase	0.021925243	1.675481703	4.126913301	0.405989072	0.68475063	0.996994191
2.6.99.2	pyridoxine 5-phosphate synthase	45.90984814	-0.305591924	0.517246186	-0.590805563	0.554650707	0.996994191
1.2.7.4	#N/A	735.1758215	0.071422845	0.644396017	0.110836882	0.911745698	0.996994191
1.20.4.3	mycoredoxin	0.089723976	0.638106246	4.129588845	0.154520527	0.877199327	0.996994191
2.5.1.75	tRNA dimethylallyltransferase	316.1405562	0.084606652	0.238830915	0.35425335	0.723149025	0.996994191
4.2.3.1	threonine synthase	417.3788552	-0.2187916	0.185536028	-1.179240508	0.23830242	0.996994191
2.7.11.21	polo-like kinase 1	0.56438504	0.980722719	4.068143719	0.241073764	0.809497946	0.996994191
1.3.1.14	dihydroorotate dehydrogenase (NAD+) catalytic subunit	341.9876951	-0.120135621	0.204039395	-0.588786402	0.556004569	0.996994191
1.1.1.290	erythronate-4-phosphate dehydrogenase	70.05388707	-0.27150149	0.514855283	-0.527335543	0.597960596	0.996994191
1.2.1.31	L-aminoadipate-semialdehyde dehydrogenase	0.034954079	0.921806092	4.139322395	0.222694925	0.823772951	0.996994191
4.2.1.79	2-methylcitrate dehydratase	0.582300256	-0.758140442	3.944870631	-0.192183854	0.847598193	0.996994191
2.3.1.51	1-acyl-sn-glycerol-3-phosphate acyltransferase	528.0808745	0.194438653	0.238901737	0.813885473	0.415710564	0.996994191
3.5.2.3	dihydroorotase	396.1747044	-0.339830475	0.154280006	-2.202686421	0.027616858	0.996994191
2.1.1.250	trimethylamine---corrinoid protein Co-methyltransferase	2.063895754	-2.219800569	2.284437508	-0.971705534	0.331197063	0.996994191
2.7.1.180	FAD:protein FMN transferase	253.8645661	-0.092173817	0.303248793	-0.303954439	0.761162603	0.996994191
6.3.4.22	tRNA(Ile2)-agmatinylcytidine synthase	2.007550506	4.785396998	2.593887447	1.844874574	0.065055786	0.996994191
1.6.1.2	H+-translocating NAD(P) transhydrogenase	18.93975992	0.600466086	0.759493334	0.79061403	0.429169257	0.996994191
3.2.1.50	alpha-N-acetylglucosaminidase	95.82601796	-0.470916039	0.38728306	-1.215947938	0.224004755	0.996994191
2.1.1.216	tRNA (guanine26-N2/guanine27-N2)-dimethyltransferase	0.68372251	4.043597878	4.058887184	0.996233129	0.319136886	0.996994191
2.4.2.4	thymidine phosphorylase	11.4572742	1.156554793	1.120726837	1.0319685	0.302086874	0.996994191
6.1.1.16	#N/A	514.6442691	0.210233381	0.167729681	1.253405957	0.210058005	0.996994191
6.3.4.6	urea carboxylase	6.97224277	3.23388695	1.969529313	1.641959288	0.100598442	0.996994191
1.1.1.193	5-amino-6-(5-phosphoribosylamino)uracil reductase	165.0856682	-0.46899328	0.349018266	-1.343749957	0.179029257	0.996994191
6.3.2.43	[lysine-biosynthesis-protein LysW]---L-2-aminoadipate ligase	0.050191057	1.314825092	4.126913272	0.318597704	0.750031595	0.996994191
4.2.1.25	L-arabonate dehydrase	0.031664897	1.436859336	4.142987902	0.346817169	0.728728678	0.996994191
6.3.4.19	tRNA(Ile)-lysidine synthase	465.8400504	0.075249787	0.188907422	0.398342142	0.690378001	0.996994191
3.6.3.15	#N/A	523.0703408	-0.029000509	0.294073692	-0.098616467	0.921442795	0.996994191
6.3.2.38	N2-citryl-N6-acetyl-N6-hydroxylysine synthase	0.337074854	0.333131425	4.103588499	0.081180514	0.935298396	0.996994191
2.7.7.66	phosphoribosyl-dephospho-CoA transferase	0.948413065	3.13453172	2.924192945	1.071930539	0.283751232	0.996994191
2.3.1.251	lipid IVA palmitoyltransferase	12.68719458	-0.168842358	1.621021932	-0.104157973	0.917043986	0.996994191
2.1.1.201	demethylmenaquinone methyltransferase / 2-methoxy-6-polyp	59.19781542	-0.31834755	0.419451577	-0.758961384	0.447875657	0.996994191
3.5.4.4	adenosine deaminase	49.88921396	-0.421849747	0.48021716	-0.87845621	0.379696192	0.996994191
3.2.1.55	alpha-N-arabinofuranosidase	304.048897	-0.302593291	0.311676814	-0.970855953	0.331620015	0.996994191
1.1.1.215	glyoxylate/hydroxypyruvate/2-ketogluconate reductase	1.070230206	3.4430193	4.045846055	0.851001065	0.394768761	0.996994191
4.2.1.114	methanogen homoaconitase large subunit	10.62242811	2.891871069	1.710485093	1.690673062	0.090899262	0.996994191
2.7.9.1	pyruvate, orthophosphate dikinase	3366.086176	0.23218953	0.496572396	0.467584449	0.640081788	0.996994191
2.3.1.203	UDP-N-acetylbacillosamine N-acetyltransferase	1.434965331	-2.452931814	2.944269387	-0.833120714	0.404776652	0.996994191
2.4.2.12	nicotinamide phosphoribosyltransferase	17.36203353	1.49236108	1.130203289	1.320435973	0.1866895	0.996994191
3.6.1.22	NAD+ diphosphatase	138.749748	0.060101239	0.317824866	0.189101751	0.850013069	0.996994191
3.2.1.70	glucan 1,6-alpha-glucosidase	14.70370541	-0.954825051	0.793733361	-1.202954415	0.228993961	0.996994191
2.1.4.1	glycine amidinotransferase	45.52497431	-0.317716359	0.930472156	-0.341457138	0.732759467	0.996994191
3.3.1.1	adenosylhomocysteinase	126.059348	-0.177762703	0.313230801	-0.567513483	0.570365369	0.996994191
2.7.1.140	inositol-polyphosphate multikinase	0.160692243	3.261377394	4.083493213	0.798673397	0.424479817	0.996994191
2.3.1.231	tRNA wybutosine-synthesizing protein 4	0.053564081	2.349751186	4.116844279	0.570765136	0.568158859	0.996994191
2.1.1.133	precorrin-4/cobalt-precorrin-4 C11-methyltransferase	110.7485461	-0.51492011	0.384611301	-1.338806502	0.180633674	0.996994191
1.1.1.346	2,5-diketo-D-gluconate reductase A	10.87598757	2.281599154	1.168714229	1.952230149	0.050910886	0.996994191
6.1.1.3	threonyl-tRNA synthetase	799.6942148	-0.243668916	0.20992252	-1.160756437	0.245740963	0.996994191
4.4.1.11	methionine-gamma-lyase	55.61284014	-0.392488489	0.563643428	-0.696341817	0.486214791	0.996994191
1.2.1.88	1-pyrroline-5-carboxylate dehydrogenase	93.26804562	-0.201370549	0.377467694	-0.533477573	0.593703032	0.996994191
3.4.16.4	D-alanyl-D-alanine carboxypeptidase	1095.067042	-0.256379885	0.283925386	-0.90298331	0.366534753	0.996994191
2.8.3.18	succinyl-CoA:acetate CoA-transferase	17.73499046	-0.513963166	0.492739571	-1.043072642	0.29691465	0.996994191
3.-.-.-	#N/A	8.523949024	1.64957874	1.397796256	1.180128172	0.237949241	0.996994191
4.2.1.45	CDP-glucose 4,6-dehydratase	79.53278265	0.019128262	0.308236799	0.062057034	0.950517413	0.996994191
3.5.4.33	tRNA(adenine34) deaminase	257.7751313	-0.771626026	0.594670019	-1.297570082	0.194435108	0.996994191
3.1.3.83	D-glycero-D-manno-heptose 1,7-bisphosphate phosphatase	19.00358607	0.123732139	0.517201282	0.239234014	0.810924129	0.996994191
1.13.11.54	1,2-dihydroxy-3-keto-5-methylthiopentene dioxygenase	0.050191057	1.314825092	4.126913272	0.318597704	0.750031595	0.996994191
2.7.2.2	carbamate kinase	210.8375222	-0.407236882	0.370737496	-1.098450752	0.272007711	0.996994191
3.1.1.-	#N/A	496.6853923	0.010223066	0.181663884	0.056274622	0.955123036	0.998234556
2.5.1.134	cystathionine beta-synthase (O-acetyl-L-serine)	0.594219345	0.226445745	4.101481853	0.055210715	0.955970593	0.998234556
3.6.3.4	Cu2+-exporting ATPase	642.8453761	-0.018652225	0.337002642	-0.055347416	0.955861688	0.998234556
4.99.1.1	protoporphyrin/coproporphyrin ferrochelatase	9.070073581	-0.056030505	1.0104867	-0.055449028	0.955780737	0.998234556
4.2.1.49	urocanate hydratase	239.7870597	0.013618255	0.30910884	0.044056503	0.964859364	0.998489161
2.6.1.1	aspartate aminotransferase, chloroplastic	444.3986427	0.013144807	0.267954118	0.049056185	0.960874521	0.998489161
4.3.3.7	4-hydroxy-tetrahydrodipicolinate synthase	498.9117405	-0.009508692	0.187927237	-0.050597731	0.959646071	0.998489161
1.3.1.98	UDP-N-acetylmuramate dehydrogenase	353.7006	0.008481698	0.184930991	0.045864126	0.963418548	0.998489161
2.7.1.-	#N/A	1291.856995	-0.015838984	0.358170102	-0.044221959	0.964727478	0.998489161
1.13.11.-	#N/A	1.263813578	-0.206660951	4.075861101	-0.050703629	0.959561684	0.998489161
1.14.13.59	lysine N6-hydroxylase	0.353595073	0.198294495	4.102272395	0.04833772	0.961447093	0.998489161
2.3.1.19	phosphate butyryltransferase	49.06941609	-0.02199679	0.419688212	-0.052412218	0.958200239	0.998489161
2.5.1.29	geranylgeranyl diphosphate synthase, type III	398.5314581	0.010452977	0.221733079	0.047142163	0.962399924	0.998489161
1.1.99.1	choline dehydrogenase	0.4007054	-0.219481883	4.09942782	-0.053539638	0.957301949	0.998489161
1.1.1.157	3-hydroxybutyryl-CoA dehydrogenase	1460.350109	0.029705957	0.648832179	0.04578373	0.963482627	0.998489161
2.4.2.10	orotate phosphoribosyltransferase	602.0547244	-0.01245702	0.275460459	-0.045222533	0.963929934	0.998489161

3.6.3.38	capsular polysaccharide transport system ATP-binding protein	2.063563325	0.129781745	2.769112751	0.046867627	0.962618729	0.998489161
6.4.1.2	acetyl-CoA carboxylase / biotin carboxylase 2	637.2201999	-0.010426231	0.226288978	-0.046074851	0.96325059	0.998489161
3.7.1.8	2,6-dioxo-6-phenylhexa-3-enoate hydrolase	0.247596681	-0.203060655	4.11668904	-0.049326207	0.960659335	0.998489161
1.5.3.-	#N/A	0.818610359	0.178250652	4.094212532	0.043537225	0.965273291	0.998489161
1.7.1.7	GMP reductase	3.492030991	-0.058012577	1.187604863	-0.048848383	0.961040124	0.998489161
2.3.1.179	3-oxoacyl-[acyl-carrier-protein] synthase II	527.6690916	-0.009997483	0.219786557	-0.045487234	0.96371895	0.998489161
6.3.2.6	phosphoribosylaminoimidazole carboxylase / phosphoribosylan	329.075709	0.007787494	0.182103059	0.042764211	0.965889494	0.998605917
3.5.1.10	formyltetrahydrofolate deformylase	26.97695206	0.01973101	0.80003262	0.024662757	0.980323961	0.998608707
1.1.1.9	D-xylulose reductase	0.480652276	0.157473892	4.110281326	0.038312193	0.969438769	0.998608707
2.1.1.222	2-polyprenyl-6-hydroxyphenyl methylase / 3-demethylubiquinc	0.275721832	-0.154275226	4.107414861	-0.037560176	0.97003836	0.998608707
1.13.11.3	#N/A	0.337017706	-0.107201702	4.107830431	-0.026096915	0.979180038	0.998608707
3.6.3.14	#N/A	3022.576656	0.008950897	0.264938828	0.033784768	0.973048782	0.998608707
1.18.1.-	#N/A	0.305626466	-0.159120944	4.09938434	-0.038815815	0.969037236	0.998608707
3.5.2.5	allantoinase	3.997005751	0.056392366	1.37027547	0.041154036	0.967173097	0.998608707
2.7.7.53	sulfate adenylyltransferase (ADP) / ATP adenylyltransferase	5.267729909	-0.055754742	1.868332548	-0.02984198	0.976193079	0.998608707
1.1.1.140	sorbitol-6-phosphate 2-dehydrogenase	20.25945935	-0.024441119	0.801702993	-0.030486501	0.975679059	0.998608707
3.6.4.-	#N/A	1559.202111	-0.008110603	0.205433635	-0.039480403	0.968507378	0.998608707
3.6.1.41	bis(5'-nucleosyl)-tetraphosphatase (symmetrical)	3.179764336	-0.051482552	1.813797633	-0.028383846	0.977356008	0.998608707
1.1.1.384	dTDP-3,4-didehydro-2,6-dideoxy-alpha-D-glucose 3-reductase	3.667088234	0.03724924	1.206460001	0.030874824	0.975369368	0.998608707
2.1.1.64	2-polyprenyl-6-hydroxyphenyl methylase / 3-demethylubiquinc	0.275721832	-0.154275226	4.107414861	-0.037560176	0.97003836	0.998608707
3.1.3.12	trehalose 6-phosphate phosphatase	28.07036042	-0.019640403	0.50283704	-0.03905918	0.968843206	0.998608707
4.2.1.10	#N/A	224.1164232	-0.006371274	0.271640787	-0.023454776	0.981287512	0.998608707
2.5.1.21	farnesyl-diphosphate farnesyltransferase	2.623950859	0.041279833	1.250248939	0.033017291	0.973660799	0.998608707
4.2.1.9	dihydroxy-acid dehydratase	540.2886476	-0.006323048	0.245334322	-0.025773188	0.979438248	0.998608707
1.1.1.86	ketol-acid reductoisomerase	462.1428873	-0.008925736	0.307525147	-0.029024409	0.976845123	0.998608707
2.6.1.98	UDP-2-acetamido-2-deoxy-ribo-hexuluronate aminotransferase	0.172699552	-0.117170708	4.12647118	-0.028394893	0.977347197	0.998608707
6.3.4.20	7-cyano-7-deazaguanine synthase	59.4077229	0.014474056	0.462265808	0.031311112	0.975021429	0.998608707
2.3.1.242	KDO2-lipid IV(A) palmitoleoyltransferase	3.639820526	0.069399627	2.725351818	0.025464465	0.979684492	0.998608707
5.1.3.5	UDP-arabinose 4-epimerase	0.840902598	-0.145380163	4.125699335	-0.035237702	0.971890199	0.998608707
6.3.5.7	aspartyl-tRNA(Asn)/glutamyl-tRNA(Gln) amidotransferase subu	513.5161994	-0.007144813	0.293511607	-0.024342522	0.980579396	0.998608707
2.5.1.1	farnesyl diphosphate synthase	408.1672705	-0.005015497	0.216240287	-0.02319409	0.981495453	0.998608707
3.1.1.32	phospholipase A1/A2	6.442157471	0.026116054	1.11867845	0.023345452	0.981374716	0.998608707
2.7.7.4	bifunctional enzyme CysN/CysC	320.7491654	-0.00812855	0.266635291	-0.030485649	0.975679738	0.998608707
3.2.1.91	cellulose 1,4-beta-cellobiosidase	0.254639706	0.129323425	4.128600401	0.031323793	0.975011315	0.998608707
1.2.1.20	succinate-semialdehyde dehydrogenase / glutarate-semialdehy	8.343123664	-0.03390252	1.161877208	-0.02917909	0.976721758	0.998608707
2.5.1.10	farnesyl diphosphate synthase	408.1672705	-0.005015497	0.216240287	-0.02319409	0.981495453	0.998608707
3.1.1.4	secretory phospholipase A2	6.442157471	0.026116054	1.11867845	0.023345452	0.981374716	0.998608707
1.2.1.79	succinate-semialdehyde dehydrogenase / glutarate-semialdehy	8.343123664	-0.03390252	1.161877208	-0.02917909	0.976721758	0.998608707
4.1.99.1	tryptophanase	214.4032856	-0.005588405	0.459514629	-0.012161539	0.990296735	0.999224525
5.3.3.18	2-(1,2-epoxy-1,2-dihydrophenyl)acetyl-CoA isomerase	18.26828696	-0.018100227	1.056037499	-0.017139758	0.986325121	0.999224525
1.1.1.169	2-dehydropantoate 2-reductase	326.602218	0.002971148	0.266343098	0.011155342	0.991099509	0.999224525
6.3.1.5	NAD+ synthase	39.38747508	-0.007095487	0.67961723	-0.010440416	0.991669904	0.999224525
1.13.12.-	#N/A	1.243420728	0.042088491	3.87001479	0.010875538	0.991322748	0.999224525
4.1.2.40	tagatose 1,6-diphosphate aldolase	44.01710737	-0.009812779	0.484556359	-0.020251058	0.983843098	0.999224525
2.7.2.7	butyrate kinase	172.3403853	0.006379934	0.372153334	0.017143295	0.9863223	0.999224525
2.5.1.90	octaprenyl-diphosphate synthase	81.05412723	0.007687629	0.46528642	0.016522358	0.986817666	0.999224525
1.5.1.33	pteridine reductase	0.26805721	-0.088409857	4.100301344	-0.021561795	0.98279751	0.999224525
2.7.1.203	PTS system, D-glucosaminate-specific IIA component	2.990922439	-0.021818951	1.633201226	-0.013359622	0.989340881	0.999224525
3.2.1.187	beta-L-arabinobiosidase	0.420346293	-0.065143936	4.113902869	-0.015835069	0.987365971	0.999224525
2.3.1.117	2,3,4,5-tetrahydropyridine-2,6-dicarboxylate N-succinyltransfer	33.00507093	-0.011652685	0.73313746	-0.01589427	0.987318742	0.999224525
4.1.1.47	tartronate-semialdehyde synthase	3.024359811	0.024425398	1.573041308	0.015527499	0.987611346	0.999224525
6.5.1.2	DNA ligase (NAD+)	524.6015592	-0.002118364	0.159981819	-0.013241277	0.989435298	0.999224525
6.3.4.3	methylenetetrahydrofolate dehydrogenase (NADP+) / metheny	919.4527816	-0.003476102	0.279249675	-0.012448006	0.990068185	0.999224525
3.6.1.54	UDP-2,3-diacylglucosamine hydrolase	80.87134064	0.006471442	0.336880981	0.019209877	0.984673679	0.999224525
4.1.99.14	spore photoproduct lyase	9.608842198	0.008188227	0.748868506	0.010934132	0.991275999	0.999224525
1.5.3.19	4-methylaminobutanoate oxidase (formaldehyde-forming)	0.655665518	0.06078743	4.090316048	0.014861304	0.988142831	0.999224525
1.2.1.11	aspartate-semialdehyde dehydrogenase	391.8091547	-0.002302001	0.198322162	-0.011607379	0.990738859	0.999224525
1.1.5.4	malate dehydrogenase (quinone)	0.675139586	-0.038718249	4.088577504	-0.009469858	0.992444259	0.999497163
1.3.1.34	2,4-dienoyl-CoA reductase (NADPH2)	11.37066263	0.007480059	0.987344087	0.007575939	0.993955333	0.999611245
1.8.1.7	glutathione reductase (NADPH)	3.926453027	-0.01087861	1.768766075	-0.006150395	0.995092726	0.999611245
2.1.1.192	23S rRNA (adenine2503-C2)-methyltransferase	414.0283495	-0.001131471	0.155516564	-0.007275568	0.994194988	0.999611245
2.6.1.110	dTDP-4-dehydro-2,3,6-trideoxy-D-glucose 4-aminotransferase	0.598486539	0.022763667	4.093339591	0.005561148	0.995562869	0.999611245
5.1.2.3	3-hydroxyacyl-CoA dehydrogenase / enoyl-CoA hydratase / 3-h	0.670887678	0.031277218	4.066430671	0.007691566	0.993863079	0.999611245
6.2.1.14	6-carboxyhexanoate--CoA ligase	1.997081838	0.012378092	2.234723431	0.005538982	0.995580555	0.999611245
5.1.3.13	dTDP-4-dehydrorhamnose 3,5-epimerase	207.8342078	0.000982119	0.221379596	0.004436357	0.99646031	0.999721457
2.7.1.26	riboflavin kinase	322.5539954	-0.000827901	0.236085498	-0.003506783	0.997201998	0.999721457
2.7.7.2	FAD synthetase	322.5539954	-0.000827901	0.236085498	-0.003506783	0.997201998	0.999721457
5.4.2.11	2,3-bisphosphoglycerate-dependent phosphoglycerate mutase	221.9404766	-0.000388308	0.362805249	-0.001070293	0.99914603	0.999939653
1.4.1.11	L-erythro-3,5-diaminohexanoate dehydrogenase	75.26061903	-4.32E-05	0.571154018	-7.56E-05	0.999939653	0.999939653
5.1.3.8	N-acylglucosamine 2-epimerase	101.6805329	0.000415378	0.375533502	0.0011061	0.99911746	0.999939653
4.2.1.70	#N/A	46.01611001	-0.001492876	0.708981798	-0.002105662	0.998319926	0.999939653
4.1.99.2	tyrosine phenol-lyase	16.61240796	0.00025803	1.571217178	0.000164223	0.999868969	0.999939653
2.1.1.22	carnosine N-methyltransferase	0	NA	NA	NA	NA	NA
2.3.1.7	carnitine O-acetyltransferase	0	NA	NA	NA	NA	NA
1.14.11.-	#N/A	0	NA	NA	NA	NA	NA
1.1.1.270	3-keto steroid reductase	0	NA	NA	NA	NA	NA
2.3.1.37	5-aminolevulinate synthase	0	NA	NA	NA	NA	NA
3.1.3.99	IMP and pyridine-specific 5'-nucleotidase	0	NA	NA	NA	NA	NA
2.7.8.11	CDP-diacylglycerol--inositol 3-phosphatidyltransferase	0	NA	NA	NA	NA	NA
3.2.1.106	mannosyl-oligosaccharide glucosidase	0	NA	NA	NA	NA	NA
2.4.1.256	alpha-1,2-glucosyltransferase	0	NA	NA	NA	NA	NA
4.1.1.45	aminocarboxymuconate-semialdehyde decarboxylase	0	NA	NA	NA	NA	NA
3.1.1.56	carboxylesterase 2	0	NA	NA	NA	NA	NA
4.1.1.86	L-2,4-diaminobutyrate decarboxylase	0	NA	NA	NA	NA	NA
2.4.99.-	#N/A	0	NA	NA	NA	NA	NA
4.1.3.41	3-hydroxy-D-aspartate aldolase	0	NA	NA	NA	NA	NA
3.6.1.42	guanosine-diphosphatase	0	NA	NA	NA	NA	NA

3.1.22.-	#N/A	0	NA	NA	NA	NA	NA
2.8.3.22	succinyl-CoA:(S)-malate CoA-transferase subunit A	0	NA	NA	NA	NA	NA
2.7.1.182	phytol kinase	0	NA	NA	NA	NA	NA
2.4.1.131	#N/A	0	NA	NA	NA	NA	NA
2.4.2.6	nucleoside deoxyribosyltransferase	0	NA	NA	NA	NA	NA
2.3.1.222	phosphate propanoyltransferase	0	NA	NA	NA	NA	NA
2.7.7.50	mRNA guanylyltransferase	0	NA	NA	NA	NA	NA
1.3.3.11	pyrroloquinoline-quinone synthase	0	NA	NA	NA	NA	NA
3.5.1.110	ureidoacrylate peracid hydrolase	0	NA	NA	NA	NA	NA
4.1.3.43	4-hydroxy-2-oxovalerate/4-hydroxy-2-oxohexanoate aldolase	0	NA	NA	NA	NA	NA
2.7.1.82	ethanolamine kinase	0	NA	NA	NA	NA	NA
2.5.1.85	all-trans-nonaprenyl-diphosphate synthase	0	NA	NA	NA	NA	NA
2.1.1.225	tRNA:m4X modification enzyme	0	NA	NA	NA	NA	NA
3.1.1.97	diphthine methyl ester acylhydrolase	0	NA	NA	NA	NA	NA
4.2.1.134	very-long-chain (3R)-3-hydroxyacyl-CoA dehydratase	0	NA	NA	NA	NA	NA
2.7.1.150	1-phosphatidylinositol-3-phosphate 5-kinase	0	NA	NA	NA	NA	NA
5.4.99.45	tRNA pseudouridine38/39 synthase	0	NA	NA	NA	NA	NA
3.5.3.17	guanidinopropionase	0	NA	NA	NA	NA	NA
2.5.1.58	protein farnesyltransferase subunit beta	0	NA	NA	NA	NA	NA
2.4.1.198	#N/A	0	NA	NA	NA	NA	NA
2.7.8.17	UDP-N-acetylglucosamine-lysosomal-enzyme	0	NA	NA	NA	NA	NA
2.4.1.267	alpha-1,3-glucosyltransferase	0	NA	NA	NA	NA	NA
2.6.1.108	(5-formylfuran-3-yl)methyl phosphate transaminase	0	NA	NA	NA	NA	NA
2.7.4.21	inositol-hexakisphosphate 5-kinase	0	NA	NA	NA	NA	NA
2.6.1.101	L-glutamine:2-deoxy-scyllo-inosose/3-amino-2,3-dideoxy-scyllo	0	NA	NA	NA	NA	NA
3.1.3.43	pyruvate dehydrogenase phosphatase	0	NA	NA	NA	NA	NA
3.6.1.62	mRNA-decapping enzyme subunit 2	0	NA	NA	NA	NA	NA
1.5.1.-	#N/A	0	NA	NA	NA	NA	NA
3.4.24.56	insulysin	0	NA	NA	NA	NA	NA
6.3.4.11	biotin---protein ligase	0	NA	NA	NA	NA	NA
3.1.4.37	2',3'-cyclic-nucleotide 3'-phosphodiesterase	0	NA	NA	NA	NA	NA
1.3.1.33	protochlorophyllide reductase	0	NA	NA	NA	NA	NA
3.4.17.22	carboxypeptidase D	0	NA	NA	NA	NA	NA
1.1.1.372	L-glyceraldehyde reductase	0	NA	NA	NA	NA	NA
1.3.99.23	all-trans-retinol 13,14-reductase	0	NA	NA	NA	NA	NA
1.1.1.292	1,5-anhydro-D-fructose reductase (1,5-anhydro-D-mannitol-for	0	NA	NA	NA	NA	NA
2.3.1.189	mycothiol synthase	0	NA	NA	NA	NA	NA
2.4.1.132	alpha-1,3/alpha-1,6-mannosyltransferase	0	NA	NA	NA	NA	NA
3.4.11.26	intermediate cleaving peptidase 55	0	NA	NA	NA	NA	NA
2.5.1.92	(2Z,6Z)-farnesyl diphosphate synthase	0	NA	NA	NA	NA	NA
2.5.1.68	short-chain Z-isoprenyl diphosphate synthase	0	NA	NA	NA	NA	NA
3.2.1.49	alpha-N-acetylgalactosaminidase	0	NA	NA	NA	NA	NA
6.3.4.9	biotin---protein ligase	0	NA	NA	NA	NA	NA
5.3.3.1	3beta-hydroxy-Delta5-steroid dehydrogenase / steroid Delta-is	0	NA	NA	NA	NA	NA
1.10.3.11	ubiquinol oxidase	0	NA	NA	NA	NA	NA
1.11.1.1	NADH peroxidase	0	NA	NA	NA	NA	NA
5.3.3.10	5-carboxymethyl-2-hydroxymuconate isomerase	0	NA	NA	NA	NA	NA
3.1.3.96	pseudouridine 5'-phosphatase	0	NA	NA	NA	NA	NA
3.6.1.43	dolichyldiphosphatase	0	NA	NA	NA	NA	NA
3.6.1.29	bis(5'-adenosyl)-triphosphatase	0	NA	NA	NA	NA	NA
2.4.1.101	alpha-1,3-mannosyl-glycoprotein beta-1,2-N-acetylglucosaminy	0	NA	NA	NA	NA	NA
2.3.2.17	peptidoglycan pentaglycine glycine transferase (the second and	0	NA	NA	NA	NA	NA
1.14.11.27	F-box and leucine-rich repeat protein 10/11	0	NA	NA	NA	NA	NA
5.4.3.9	glutamate 2,3-aminomutase	0	NA	NA	NA	NA	NA
2.4.1.250	D-inositol-3-phosphate glycosyltransferase	0	NA	NA	NA	NA	NA
6.2.1.16	acetoacetyl-CoA synthetase	0	NA	NA	NA	NA	NA
3.1.1.89	protein phosphatase methylesterase 1	0	NA	NA	NA	NA	NA
6.4.1.5	geranyl-CoA carboxylase alpha subunit	0	NA	NA	NA	NA	NA
2.1.1.183	18S rRNA (adenine1779-N6/adenine1780-N6)-dimethyltransfer	0	NA	NA	NA	NA	NA
5.1.3.37	mannuronan 5-epimerase	0	NA	NA	NA	NA	NA
1.10.2.-	#N/A	0	NA	NA	NA	NA	NA
2.1.1.214	tRNA (guanine10-N2)-methyltransferase	0	NA	NA	NA	NA	NA
3.1.3.66	inositol polyphosphate-4-phosphatase	0	NA	NA	NA	NA	NA
3.7.1.5	acylpyruvate hydrolase	0	NA	NA	NA	NA	NA
4.1.3.25	malyl-CoA/(S)-citramalyl-CoA lyase	0	NA	NA	NA	NA	NA
2.5.1.44	homospermidine synthase	0	NA	NA	NA	NA	NA
2.3.2.18	peptidoglycan pentaglycine glycine transferase (the fourth and	0	NA	NA	NA	NA	NA
3.5.3.23	succinylarginine dihydrolase	0	NA	NA	NA	NA	NA
1.3.1.88	tRNA-dihydrouridine synthase 1	0	NA	NA	NA	NA	NA
3.1.3.77	enolase-phosphatase E1	0	NA	NA	NA	NA	NA
1.1.99.21	D-sorbitol dehydrogenase (acceptor)	0	NA	NA	NA	NA	NA
2.1.1.320	type II protein arginine methyltransferase	0	NA	NA	NA	NA	NA
2.7.7.79	tRNA(His) guanylyltransferase	0	NA	NA	NA	NA	NA
6.2.1.41	HIP---CoA ligase	0	NA	NA	NA	NA	NA
5.1.1.17	isopenicillin-N epimerase	0	NA	NA	NA	NA	NA
6.2.1.34	feruloyl-CoA synthase	0	NA	NA	NA	NA	NA
2.7.4.12	T-even-induced deoxynucleotide kinase	0	NA	NA	NA	NA	NA
2.4.1.257	alpha-1,3/alpha-1,6-mannosyltransferase	0	NA	NA	NA	NA	NA
1.3.1.91	tRNA-dihydrouridine synthase 2	0	NA	NA	NA	NA	NA
2.8.1.9	molybdenum cofactor sulfurtransferase	0	NA	NA	NA	NA	NA
1.3.99.5	#N/A	0	NA	NA	NA	NA	NA
3.5.1.94	gamma-glutamyl-gamma-aminobutyrate hydrolase	0	NA	NA	NA	NA	NA
1.1.1.371	scyllo-inositol 2-dehydrogenase (NADP+)	0	NA	NA	NA	NA	NA
2.1.1.229	alkylated DNA repair protein alkB homolog 8	0	NA	NA	NA	NA	NA
1.14.14.5	alkanesulfonate monooxygenase	0	NA	NA	NA	NA	NA
1.12.1.4	iron-hydrogenase subunit alpha	0	NA	NA	NA	NA	NA
2.4.1.142	beta-1,4-mannosyltransferase	0	NA	NA	NA	NA	NA

2.4.1.258	alpha-1,3-mannosyltransferase	0	NA	NA	NA	NA	NA
2.4.1.241	digalactosyldiacylglycerol synthase	0	NA	NA	NA	NA	NA
1.13.11.15	3,4-dihydroxyphenylacetate 2,3-dioxygenase	0	NA	NA	NA	NA	NA
4.1.2.57	sulfofructosephosphate aldolase	0	NA	NA	NA	NA	NA
1.3.1.6	NADH-dependent fumarate reductase subunit A	0	NA	NA	NA	NA	NA
5.1.2.2	mandelate racemase	0	NA	NA	NA	NA	NA
1.6.99.1	NADPH2 dehydrogenase	0	NA	NA	NA	NA	NA
2.5.1.87	ditrans,polycis-polyprenyl diphosphate synthase	0	NA	NA	NA	NA	NA
2.1.1.202	multisite-specific tRNA:(cytosine-C5)-methyltransferase	0	NA	NA	NA	NA	NA
2.6.1.100	L-glutamine:2-deoxy-scyllo-inosose/3-amino-2,3-dideoxy-scyllo	0	NA	NA	NA	NA	NA
1.6.2.2	cytochrome-b5 reductase	0	NA	NA	NA	NA	NA
3.5.3.7	guanidinobutyrase	0	NA	NA	NA	NA	NA
1.12.5.1	#N/A	0	NA	NA	NA	NA	NA
1.2.1.60	5-carboxymethyl-2-hydroxymuconic-semialdehyde dehydrogen	0	NA	NA	NA	NA	NA
2.1.1.100	protein-S-isoprenylcysteine O-methyltransferase	0	NA	NA	NA	NA	NA
3.6.1.12	dCTP diphosphatase	0	NA	NA	NA	NA	NA
1.1.1.163	cyclopentanol dehydrogenase	0	NA	NA	NA	NA	NA
1.10.9.1	#N/A	0	NA	NA	NA	NA	NA
6.3.1.11	gamma-glutamylputrescine synthase	0	NA	NA	NA	NA	NA
3.6.1.5	apyrase	0	NA	NA	NA	NA	NA
1.1.1.320	benzil reductase ((S)-benzoin forming)	0	NA	NA	NA	NA	NA
3.4.23.1	pepsin A	0	NA	NA	NA	NA	NA
1.1.1.11	D-arabinitol 4-dehydrogenase	0	NA	NA	NA	NA	NA
3.2.1.46	galactosylceramidase	0	NA	NA	NA	NA	NA
3.2.2.3	uridine nucleosidase	0	NA	NA	NA	NA	NA
3.4.11.24	aminopeptidase S	0	NA	NA	NA	NA	NA
2.1.1.321	type III protein arginine methyltransferase	0	NA	NA	NA	NA	NA
2.4.1.265	alpha-1,3-glucosyltransferase	0	NA	NA	NA	NA	NA
3.5.4.-	#N/A	0	NA	NA	NA	NA	NA
2.3.1.225	palmitoyltransferase ZDHHC9/14/18	0	NA	NA	NA	NA	NA
4.2.1.115	UDP-N-acetylglucosamine 4,6-dehydratase	0	NA	NA	NA	NA	NA
1.13.11.1	catechol 1,2-dioxygenase	0	NA	NA	NA	NA	NA
1.1.1.21	aldehyde reductase	0	NA	NA	NA	NA	NA
3.5.5.7	aliphatic nitrilase	0	NA	NA	NA	NA	NA
3.5.4.34	tRNA-specific adenosine deaminase 1	0	NA	NA	NA	NA	NA
3.4.21.112	membrane-bound transcription factor site-1 protease	0	NA	NA	NA	NA	NA
6.3.1.14	diphthine-ammonia ligase	0	NA	NA	NA	NA	NA
2.1.1.287	ribosomal RNA-processing protein 8	0	NA	NA	NA	NA	NA
2.1.1.260	rRNA small subunit pseudouridine methyltransferase Nep1	0	NA	NA	NA	NA	NA
4.2.1.161	bisanhydrobacterioruberin hydratase	0	NA	NA	NA	NA	NA
3.4.22.49	separase	0	NA	NA	NA	NA	NA
2.7.11.13	classical protein kinase C alpha type	0	NA	NA	NA	NA	NA
2.1.1.205	tRNA (cytidine32/guanosine34-2'-O)-methyltransferase	0	NA	NA	NA	NA	NA
3.2.1.130	glycoprotein endo-alpha-1,2-mannosidase	0	NA	NA	NA	NA	NA
4.4.1.2	cystathionine gamma-lyase / homocysteine desulfhydrase	0	NA	NA	NA	NA	NA
3.2.1.155	xyloglucan-specific exo-beta-1,4-glucanase	0	NA	NA	NA	NA	NA
2.4.1.255	protein O-GlcNAc transferase	0	NA	NA	NA	NA	NA
3.5.1.96	succinylglutamate desuccinylase	0	NA	NA	NA	NA	NA
2.5.1.59	protein farnesyltransferase/geranylgeranyltransferase type-1 s	0	NA	NA	NA	NA	NA
2.5.1.84	all-trans-nonaprenyl-diphosphate synthase	0	NA	NA	NA	NA	NA
3.5.1.38	glutamin-(asparagin-)ase	0	NA	NA	NA	NA	NA
3.7.1.3	kynureninase	0	NA	NA	NA	NA	NA
2.3.1.78	heparan-alpha-glucosaminide N-acetyltransferase	0	NA	NA	NA	NA	NA
2.7.1.91	sphingosine kinase	0	NA	NA	NA	NA	NA
2.1.3.9	N-acetylornithine carbamoyltransferase	0	NA	NA	NA	NA	NA
3.5.4.30	dCTP deaminase (dUMP-forming)	0	NA	NA	NA	NA	NA
6.2.1.31	2-furoate---CoA ligase	0	NA	NA	NA	NA	NA
5.1.3.10	CDP-paratose 2-epimerase	0	NA	NA	NA	NA	NA
1.5.99.6	spermidine dehydrogenase	0	NA	NA	NA	NA	NA
3.5.1.11	penicillin amidase	0	NA	NA	NA	NA	NA
2.7.1.32	choline kinase	0	NA	NA	NA	NA	NA
2.7.1.157	N-acetylgalactosamine kinase	0	NA	NA	NA	NA	NA
1.1.1.51	aldo-keto reductase family 1 member C3	0	NA	NA	NA	NA	NA
1.6.3.4	NADH oxidase (H2O-forming)	0	NA	NA	NA	NA	NA
3.4.24.27	thermolysin	0	NA	NA	NA	NA	NA
1.14.13.81	magnesium-protoporphyrin IX monomethyl ester (oxidative) cy	0	NA	NA	NA	NA	NA
4.1.99.20	3-amino-4-hydroxybenzoic acid synthase	0	NA	NA	NA	NA	NA
2.7.7.52	terminal uridylyltransferase	0	NA	NA	NA	NA	NA
1.1.1.264	L-idonate 5-dehydrogenase	0	NA	NA	NA	NA	NA
2.7.8.29	phosphatidylserine synthase 2	0	NA	NA	NA	NA	NA
2.4.1.87	N-acetyllactosaminide 3-alpha-galactosyltransferase	0	NA	NA	NA	NA	NA
1.8.-.-	#N/A	0	NA	NA	NA	NA	NA
3.1.3.67	phosphatidylinositol-3,4,5-trisphosphate 3-phosphatase and du	0	NA	NA	NA	NA	NA
2.4.1.287	rhamnopyranosyl-N-acetylglucosaminyl-diphospho-decaprenol	0	NA	NA	NA	NA	NA
2.9.1.2	O-phospho-L-seryl-tRNASec:L-selenocysteinyl-tRNA synthase	0	NA	NA	NA	NA	NA
2.8.3.20	succinyl-CoA---D-citramalate CoA-transferase	0	NA	NA	NA	NA	NA
1.1.1.108	carnitine 3-dehydrogenase	0	NA	NA	NA	NA	NA
2.7.1.137	phosphatidylinositol 3-kinase	0	NA	NA	NA	NA	NA
6.3.4.10	biotin---protein ligase	0	NA	NA	NA	NA	NA
3.1.1.84	carboxylesterase 2	0	NA	NA	NA	NA	NA
4.1.1.77	2-oxo-3-hexenedioate decarboxylase	0	NA	NA	NA	NA	NA
2.5.1.60	geranylgeranyl transferase type-2 subunit beta	0	NA	NA	NA	NA	NA
3.4.21.72	IgA-specific serine endopeptidase	0	NA	NA	NA	NA	NA
1.3.5.5	15-cis-phytoene desaturase	0	NA	NA	NA	NA	NA
4.1.3.16	#N/A	0	NA	NA	NA	NA	NA
1.1.1.338	(2R)-3-sulfolactate dehydrogenase (NADP+)	0	NA	NA	NA	NA	NA
3.5.1.89	N-acetylglucosaminylphosphatidylinositol deacetylase	0	NA	NA	NA	NA	NA

1.2.99.8	glyceraldehyde dehydrogenase large subunit	0	NA	NA	NA	NA	NA

Table S17: Enzymatic Functions (EC_DGE), Standard Diet, Early in Mission

	Description	baseMean	log2FoldChange	lfcSE	stat	pvalue	padj
3.4.22.37	gingipain R	0.847080334	-23.65895932	4.131468992	-5.726524722	1.03E-08	2.04E-05
3.2.1.49	alpha-N-acetylgalactosaminidase	2.267948236	19.45136299	4.112306415	4.730037363	2.24E-06	0.002235806
1.1.1.284	S-(hydroxymethyl)glutathione dehydrogenase / alcohol dehydro	2.230351518	-18.99010446	4.115889893	-4.613851429	3.95E-06	0.002624626
3.2.1.8	endo-1,4-beta-xylanase	983.5067301	3.636606011	0.800852568	4.540918211	5.60E-06	0.002789286
4.1.1.-	#N/A	0.314148663	18.28197841	4.114795172	4.442986259	8.87E-06	0.003534558
1.3.1.74	2-alkenal reductase	0.667842043	18.06468928	4.112363227	4.392775706	1.12E-05	0.003715497
1.14.12.19	3-phenylpropionate/trans-cinnamate dioxygenase subunit alph	1.118856663	-17.86902245	4.128566662	-4.328141923	1.50E-05	0.004279174
3.6.1.42	guanosine-diphosphatase	0.312227018	17.401894	4.116251797	4.227606779	2.36E-05	0.004864684
3.4.11.26	intermediate cleaving peptidase 55	0.312227018	17.401894	4.116251797	4.227606779	2.36E-05	0.004864684
2.3.1.199	elongation of very long chain fatty acids protein 6	0.416302691	17.33685271	4.114800598	4.213291092	2.52E-05	0.004864684
4.2.1.22	cystathionine beta-synthase	0.792135483	-17.26961118	4.11323436	-4.198547826	2.69E-05	0.004864684
1.3.99.4	3-oxosteroid 1-dehydrogenase	0.726380301	-16.25815654	4.115967225	-3.950020894	7.81E-05	0.012524662
2.3.1.79	maltose O-acetyltransferase	191.1526115	-1.449739604	0.368024415	-3.939248442	8.17E-05	0.012524662
3.1.3.23	sugar-phosphatase	0.6779552	-15.73648517	4.131559242	-3.808848972	0.000139615	0.017123399
1.1.1.26	glyoxylate reductase	0.566106049	-15.71770007	4.125910311	-3.809510843	0.000139242	0.017123399
3.2.2.30	adenosylhomocysteine/aminodeoxyfutalosine nucleosidase	0.689389753	-15.70749826	4.136219167	-3.797549797	0.000146133	0.017123399
2.6.1.102	perosamine synthetase	0.34586761	-15.71390747	4.128860523	-3.805870259	0.000141306	0.017123399
5.3.1.27	6-phospho-3-hexuloisomerase	13.80075987	-4.246284852	1.171929164	-3.623328937	0.000290836	0.032143698
1.8.99.2	adenylyl-sulfate reductase;	49.97111568	-2.972058473	0.82336107	-3.609666016	0.000306592	0.032143698
6.3.1.-	#N/A	0.197426127	-14.70891536	4.141443943	-3.55163937	0.000382839	0.038130783
1.16.1.9	ferric-chelate reductase (NADPH)	0.390839791	-14.62027156	4.135815758	-3.535039376	0.000407714	0.038674623
3.2.1.17	lysozyme	68.4832737	-3.31040768	0.988314834	-3.349547701	0.000809436	0.070104211
4.2.1.5	D-arabinonate dehydratase	0.322417442	-13.87087894	4.138974616	-3.351283886	0.000804378	0.070104211
3.5.1.9	arylformamidase	136.0042455	2.686721732	0.819056578	3.280263908	0.0010371	0.086079321
1.5.1.-	#N/A	0.239309938	-13.53172221	4.141839411	-3.267080365	0.001086628	0.086582542
2.4.2.17	ATP phosphoribosyltransferase	157.6760459	-0.63906556	0.196684022	-3.249199161	0.001157304	0.088667306
3.4.21.105	rhomboid-related protein 1/2/3	65.52118998	-1.373459934	0.42808812	-3.208357972	0.001334952	0.098489811
2.5.1.39	4-hydroxybenzoate polyprenyltransferase	7.596073966	-4.69417498	1.483098249	-3.165113965	0.001550222	0.110287236
3.1.21.2	deoxyribonuclease IV	367.0371785	-1.045336454	0.338831628	-3.085120652	0.002034696	0.139762564
2.3.1.29	glycine C-acetyltransferase	448.8884273	1.216658087	0.405256192	3.002194932	0.002680405	0.177978877
4.3.1.12	ornithine cyclodeaminase	24.93366618	-1.762732764	0.597249302	-2.95141871	0.003163178	0.180030014
6.3.1.12	D-aspartate ligase	24.89074862	-1.593278197	0.533914999	-2.984142047	0.002843747	0.180030014
1.4.7.1	glutamate synthase (ferredoxin)	49.96845184	-1.998992466	0.675788952	-2.958012942	0.003096291	0.180030014
3.2.1.64	levanbiose-producing levanase	0.405616982	-12.33685711	4.143731151	-2.97723396	0.00290862	0.180030014
3.4.22.41	cathepsin F	0.390452259	-12.22908675	4.139372617	-2.954333393	0.003133453	0.180030014
2.2.1.7	1-deoxy-D-xylulose-5-phosphate synthase	675.3423551	0.538118218	0.185486935	2.901111172	0.003718419	0.200191657
1.1.1.94	glycerol-3-phosphate dehydrogenase (NAD(P)+)	453.6570729	0.732928195	0.252306383	2.904913411	0.003673547	0.200191657
2.7.1.165	glycerate 2-kinase	141.9706858	-1.21773243	0.429430715	-2.83569011	0.004572679	0.239704652
3.6.1.25	triphosphatase	0.176081768	-11.71357621	4.143731151	-2.82681858	0.004701295	0.240127705
2.7.8.37	alpha-D-ribose 1-methylphosphonate 5-triphosphate synthase	11.54475179	-4.37938308	1.573152048	-2.783826958	0.005372169	0.267533999
6.3.5.1	NAD+ synthase (glutamine-hydrolysing)	5370.660459	-3.193106203	1.175593933	-2.716164242	0.006604315	0.313233206
5.4.99.1	methylaspartate mutase sigma subunit	88.22815367	1.598088343	0.586657513	2.724056725	0.006448543	0.313233206
3.5.1.1	L-asparaginase	366.752951	0.915929255	0.340309676	2.69145816	0.007114042	0.329562138
5.1.3.30	D-psicose/D-tagatose/L-ribulose 3-epimerase	12.77438443	-2.010985877	0.758018143	-2.652952169	0.007979118	0.353208942
5.1.3.31	D-psicose/D-tagatose/L-ribulose 3-epimerase	12.77438443	-2.010985877	0.758018143	-2.652952169	0.007979118	0.353208942
1.3.1.104	enoyl-[acyl-carrier protein] reductase III	0.779460713	-10.95685678	4.143731151	-2.644200693	0.008188409	0.354593709
4.1.99.14	spore photoproduct lyase	6.75289143	-3.827225732	1.458230676	-2.624568112	0.008675894	0.362375218
1.3.1.1	dihydropyrimidine dehydrogenase (NAD+) subunit PreT	44.09819686	1.373735634	0.524002304	2.621621363	0.00875126	0.362375218
2.7.6.3	2-amino-4-hydroxy-6-hydroxymethyldihydropteridine diphosph	195.0628172	-1.134038485	0.433610225	-2.615340732	0.008913848	0.362375218
6.2.1.3	long-chain acyl-CoA synthetase	787.0709517	0.624646085	0.240043998	2.602214964	0.009262377	0.369013118
6.3.5.3	phosphoribosylformylglycinamidine synthase	1099.359206	0.571582188	0.221349759	2.582258008	0.009815617	0.369125582
4.1.1.98	4-hydroxy-3-polyprenylbenzoate decarboxylase	25.44402293	-1.844873378	0.711737373	-2.592070402	0.009540025	0.369125582
2.6.1.59	dTDP-4-amino-4,6-dideoxygalactose transaminase	60.17477876	-0.856443134	0.332520827	-2.575607497	0.010006416	0.369125582
2.5.1.30	heptaprenyl diphosphate synthase	94.25819124	-0.943619453	0.365737698	-2.580044272	0.009878765	0.369125582
3.1.2.-	#N/A	298.9955864	-0.761513654	0.302270307	-2.519313466	0.011758392	0.37178917
4.2.2.8	heparin/heparan-sulfate lyase	26.41907383	-2.897196649	1.138672706	-2.544362953	0.010947726	0.37178917
1.1.1.103	threonine 3-dehydrogenase	279.8371802	1.496703724	0.586143695	2.553475771	0.01066537	0.37178917
2.5.1.-	#N/A	278.6566298	1.74922941	0.688669671	2.540012262	0.011084858	0.37178917
3.4.22.15	cathepsin L	89.02316587	3.955136745	1.55713258	2.540012838	0.01108484	0.37178917
1.21.4.2	glycine/sarcosine/betaine reductase complex component A	2335.584865	1.355317697	0.537199693	2.522930887	0.011638127	0.37178917
4.4.1.5	lactoylglutathione lyase	1030.235915	1.290953902	0.512378838	2.519530091	0.01175116	0.37178917
2.5.1.74	1,4-dihydroxy-2-naphthoate polyprenyltransferase	275.5790845	1.819595291	0.719929788	2.527462152	0.011489021	0.37178917
2.4.1.83	dolichol-phosphate mannosyltransferase	101.4455041	-0.893551808	0.35116107	-2.544563977	0.010941427	0.37178917
6.1.1.23	nondiscriminating aspartyl-tRNA synthetase	40.42760406	-1.993029253	0.794204925	-2.509464736	0.012091429	0.376345721
1.14.11.47	50S ribosomal protein L16 3-hydroxylase	3.642950035	-3.708734393	1.483497268	-2.499994083	0.012419538	0.380611075
2.8.1.10	thiazole synthase	168.3003271	-1.574503226	0.631443624	-2.493497705	0.01264914	0.38177405
4.2.1.24	porphobilinogen synthase	56.76727574	-1.371528405	0.551285806	-2.48787179	0.012851005	0.382077647
3.1.1.5	lysophospholipase	20.15479229	-1.688870677	0.681053397	-2.479791871	0.013145909	0.385097807
6.3.2.35	D-alanine---D-serine ligase	29.18882695	-1.619044197	0.655263666	-2.470828587	0.013480042	0.387234818
2.6.1.77	taurine-pyruvate aminotransferase	15.705862	1.956357185	0.794500241	2.462374563	0.013802044	0.387234818
2.1.1.79	cyclopropane-fatty-acyl-phospholipid synthase	18.11968335	-1.702856492	0.690488586	-2.466161682	0.013656967	0.387234818
4.4.1.11	methionine-gamma-lyase	52.03597116	1.039585024	0.423770477	2.453179444	0.014159968	0.39175912
3.4.25.2	ATP-dependent HslUV protease, peptidase subunit HslV	11.0559166	-2.988683188	1.226922612	-2.435918256	0.014854041	0.399854722
3.4.21.19	glutamyl endopeptidase	47.63602797	1.37932256	0.566072984	2.436651454	0.01482396	0.399854722
4.2.1.42	galactarate dehydratase	4.67297282	-3.838479963	1.586343952	-2.419702208	0.015533222	0.401846467
3.2.1.4	endoglucanase	673.5239206	1.055395692	0.436001421	2.420624433	0.015493876	0.401846467
2.4.1.187	N-acetylglucosaminyldiphosphoundecaprenol N-acetyl-beta-D-r	145.0502687	-0.920183773	0.380132614	-2.420691464	0.015491019	0.401846467
4.2.1.40	glucarate dehydratase	7.189205811	-3.083335108	1.282111418	-2.404888581	0.016177397	0.413145837
3.5.1.10	formyltetrahydrofolate deformylase	24.4019335	-2.446713535	1.023703773	-2.390060094	0.016845616	0.418201659
1.18.6.1	nitrogenase delta subunit	64.43291247	-1.839653158	0.772734673	-2.380704817	0.017279552	0.418201659
3.4.22.40	bleomycin hydrolase	151.4023736	0.701499453	0.294414202	2.382695698	0.017186394	0.418201659
3.6.3.12	K+-transporting ATPase ATPase A chain	176.7916052	-1.811285296	0.758934514	-2.386616057	0.017004239	0.418201659
2.8.2.22	arylsulfate sulfotransferase	22.28707327	-1.953640832	0.821681343	-2.377613719	0.017425069	0.418201659
2.6.1.62	adenosylmethionine---8-amino-7-oxononanoate aminotransfer	73.02597664	-1.779476566	0.750064317	-2.372431971	0.017671417	0.419065032
1.3.1.98	UDP-N-acetylmuramate dehydrogenase	388.1332859	0.603810492	0.255373667	2.364419553	0.018058349	0.423202717

5.1.3.2	UDP-glucose 4-epimerase	1242.539518	1.024289113	0.43554863	2.351721578	0.018686756	0.432639031
2.7.6.2	thiamine pyrophosphokinase	207.1046171	-0.462039205	0.196814386	-2.347588586	0.018895379	0.432639031
4.1.3.34	citrate lyase subunit beta / cityl-CoA lyase	77.48266292	0.887684383	0.381318821	2.327932256	0.019915701	0.450819053
3.6.3.54	copper-transporting P-type ATPase V	180.24383	0.704007573	0.303807077	2.317284971	0.020488213	0.453472458
2.5.1.17	cob(I)alamin adenosyltransferase	391.6330143	0.566197993	0.244066204	2.319854136	0.020348769	0.453472458
3.6.3.14	#N/A	2909.060585	0.636040856	0.276237066	2.302518141	0.02130597	0.461981623
5.1.1.18	serine racemase	30.0924605	-1.963968861	0.853166359	-2.301976444	0.021336501	0.461981623
2.5.1.129	flavin prenyltransferase	18.81128005	-2.417170942	1.053588436	-2.29422691	0.021777468	0.466459323
2.5.1.48	cystathionine gamma-synthase	39.17001989	-1.622935462	0.709380595	-2.287820494	0.022147977	0.469348616
1.2.1.3	aldehyde dehydrogenase (NAD+)	54.50178897	-1.185370539	0.519986413	-2.279618291	0.022630337	0.474522438
4.2.1.44	inosose dehydratase	44.65464953	-1.288528571	0.568335549	-2.267196856	0.023378203	0.485097708
3.5.5.1	nitrilase	6.75173712	-3.570019686	1.583753374	-2.254151275	0.024186653	0.48538814
3.2.1.80	fructan beta-fructosidase	126.3111575	-0.965195363	0.428729005	-2.25129476	0.024366874	0.48538814
2.4.1.11	glycogen synthase	55.29743903	1.464869772	0.649634647	2.254913249	0.024138775	0.48538814
2.1.1.37	DNA (cytosine-5)-methyltransferase 1	565.5999211	-0.951563822	0.422330752	-2.253124636	0.024251292	0.48538814
1.5.3.1	sarcosine oxidase	12.21788258	-2.203472545	0.981709945	-2.244525032	0.02479864	0.489097933
1.1.1.367	UDP-2-acetamido-2,6-beta-L-arabino-hexul-4-ose reductase	89.12835807	-0.967170107	0.434519	-2.225840774	0.026024849	0.503401203
2.3.1.118	N-hydroxyarylamine O-acetyltransferase	6.247718801	-2.530716862	1.137004976	-2.225774658	0.026029279	0.503401203
3.5.4.2	adenine deaminase	259.7447852	1.023887862	0.460818858	2.22188794	0.02629088	0.503571464
6.3.3.1	phosphoribosylformylglycinamidine cyclo-ligase	403.1993717	0.377842889	0.171161763	2.207519263	0.027277803	0.513565589
2.4.1.21	starch synthase	1092.909783	0.819451941	0.371331019	2.206796362	0.027328289	0.513565589
3.5.4.3	guanine deaminase	138.5600482	1.070273274	0.490772313	2.180793916	0.029198663	0.528761238
2.4.1.208	1,2-diacylglycerol-3-alpha-glucose alpha-1,2-glucosyltransferase	10.86455472	-1.565161954	0.717164713	-2.182430235	0.029077795	0.528761238
2.8.1.6	biotin synthase	211.0746319	-0.729055693	0.333945451	-2.18315803	0.029024174	0.528761238
2.7.9.1	pyruvate, orthophosphate dikinase	3849.975539	1.213362725	0.55544147	2.184501502	0.028925417	0.528761238
1.4.1.2	glutamate dehydrogenase	48.68472367	1.785702602	0.822726086	2.170470383	0.02997123	0.53248714
4.3.1.2	methylaspartate ammonia-lyase	56.28752504	1.584831975	0.735865292	2.1536985	0.031263824	0.53248714
5.4.99.18	5-(carboxyamino)imidazole ribonucleotide mutase	295.3770587	0.510573065	0.237462817	2.150117946	0.031545886	0.53248714
3.6.3.27	#N/A	174.0743809	-1.156969436	0.536790823	-2.155345039	0.031134844	0.53248714
5.4.2.2	phosphoglucomutase	10668.07044	1.582382512	0.737093319	2.146787214	0.031810226	0.53248714
4.1.3.4	hydroxymethylglutaryl-CoA lyase	4.714175398	-3.264632841	1.518907826	-2.149329133	0.031608319	0.53248714
3.2.1.136	glucuronoarabinoxylan endo-1,4-beta-xylanase	28.86644239	1.670276391	0.770219194	2.168572797	0.030115132	0.53248714
5.99.1.2	#N/A	1392.585077	-0.695885193	0.323547512	-2.150797541	0.031492183	0.53248714
1.4.1.1	alanine dehydrogenase	202.9207115	0.746188561	0.345851127	2.15754266	0.030963407	0.53248714
2.7.1.15	ribokinase	109.9336484	-0.67726543	0.317312017	-2.134383173	0.032811428	0.5401683
6.5.1.3	RNA-editing ligase	69.07652995	1.649220419	0.77246203	2.135018105	0.032759531	0.5401683
6.3.2.8	UDP-N-acetylmuramate--alanine ligase	411.158067	0.470957854	0.222270067	2.118854148	0.034102795	0.543462141
4.6.1.13	1-phosphatidylinositol phosphodiesterase	17.26687161	-2.105494419	0.990883588	-2.124865569	0.033597832	0.543462141
2.4.1.25	4-alpha-glucanotransferase	679.9918035	0.620131626	0.292028173	2.123533561	0.033709166	0.543462141
3.5.1.47	N-acetyldiaminopimelate deacetylase	5.108520293	2.940750996	1.387472822	2.119501693	0.034048091	0.543462141
3.6.1.11	exopolyphosphatase	149.2278941	-0.785068579	0.374104855	-2.098525502	0.03585875	0.566909755
3.1.1.1	carboxylesterase 1	4.915211358	-2.422985651	1.156863214	-2.094444375	0.036220413	0.568118608
2.7.7.19	poly(A) polymerase	26.75217356	-1.192520877	0.571158934	-2.08789674	0.03680715	0.572811277
2.7.7.33	glucose-1-phosphate cytidylyltransferase	43.70030962	-0.912529062	0.441600791	-2.066411746	0.038789618	0.576905617
1.6.5.2	NAD(P)H dehydrogenase (quinone)	14.05382573	1.352095195	0.654262806	2.066593398	0.038772483	0.576905617
1.1.5.3	glycerol-3-phosphate dehydrogenase	393.6201507	0.687135414	0.332350748	2.067500728	0.038686996	0.576905617
2.7.8.20	phosphoglycerol transferase	36.26566766	-0.990708454	0.477846027	-2.07327967	0.03814626	0.576905617
5.1.1.4	proline racemase	6.577327919	-2.773050875	1.340189225	-2.069148761	0.038532129	0.576905617
2.3.1.28	chloramphenicol O-acetyltransferase type B	85.27593719	-1.061953099	0.514508681	-2.064013959	0.039016393	0.576905617
6.2.1.26	O-succinylbenzoic acid---CoA ligase	92.79033664	-1.181671294	0.57274852	-2.063159051	0.039097519	0.576905617
6.1.1.11	seryl-tRNA synthetase	599.7939619	0.438018051	0.214178723	2.045105342	0.040844474	0.584068903
1.15.1.2	superoxide reductase	1449.052551	1.264282617	0.620150371	2.038671065	0.041482867	0.584068903
3.4.13.19	membrane dipeptidase	203.4932454	0.640081143	0.312467535	2.048472469	0.040513727	0.584068903
3.5.1.105	chitin disaccharide deacetylase	3.814956233	-3.198331497	1.569230657	-2.03815257	0.041534677	0.584068903
1.3.99.-	#N/A	44.4194708	-1.17705175	0.57383286	-2.051210085	0.040246492	0.584068903
2.7.1.48	uridine kinase	2305.548305	1.442732324	0.704680085	2.047357878	0.040622959	0.584068903
4.1.2.25	7,8-dihydroneopterin aldolase/epimerase/oxygenase	127.4383164	-0.931373488	0.457195302	-2.037145795	0.041635434	0.584068903
3.5.4.16	GTP cyclohydrolase IA	161.7547469	-0.938930362	0.462255848	-2.031191959	0.042235526	0.588343828
2.6.1.9	histidinol-phosphate aminotransferase	241.1110682	-0.405537859	0.200124388	-2.026428977	0.042720844	0.589793548
3.6.1.40	exopolyphosphatase / guanosine-5'-triphosphate,3'-diphosphat	148.2287987	-0.765420865	0.378102632	-2.024373277	0.042931759	0.589793548
2.7.-.-	#N/A	187.9179305	0.644582004	0.320175417	2.013215163	0.044091999	0.59345447
3.6.3.8	Ca2+-transporting ATPase	569.4036309	0.670109714	0.332443523	2.015709939	0.043830316	0.59345447
4.3.1.14	3-aminobutyryl-CoA ammonia-lyase	95.00817285	1.03327094	0.5123618	2.016682236	0.043728685	0.59345447
2.7.7.73	sulfur carrier protein ThiS adenylyltransferase	11.94926618	-2.329656036	1.167994821	-1.994577368	0.046088994	0.612061841
3.6.3.15	#N/A	673.0695012	0.613350821	0.307354971	1.995578012	0.045979875	0.612061841
2.5.1.61	hydroxymethylbilane synthase	68.00050909	-0.768720836	0.386218379	-1.990378702	0.046549234	0.614079964
2.7.8.41	cardiolipin synthase (CMP-forming)	61.66824824	-1.175093427	0.591867703	-1.985398801	0.047100119	0.617259458
3.1.1.41	cephalosporin-C deacetylase	12.9623897	1.429526297	0.723336509	1.976294959	0.04812138	0.626521499
2.7.1.92	5-dehydro-2-deoxygluconokinase	11.52794472	-2.093782979	1.062176909	-1.971218693	0.048698867	0.629923003
2.7.1.16	L-ribulokinase	41.05216264	-0.83869451	0.428673499	-1.956487893	0.050407707	0.630817589
5.1.1.7	diaminopimelate epimerase	534.3515027	-1.168132788	0.599885517	-1.947259528	0.051503628	0.630817589
3.6.3.24	nickel transport system ATP-binding protein	10.48207584	-2.885047705	1.482320404	-1.946305062	0.051618106	0.630817589
2.4.1.64	alpha,alpha-trehalose phosphorylase	4.662255611	-3.324812271	1.698181935	-1.957865764	0.050245766	0.630817589
2.4.2.53	undecaprenyl-phosphate 4-deoxy-4-formamido-L-arabinose tra	25.70573289	-1.121590659	0.575258095	-1.949717299	0.051209823	0.630817589
3.1.3.45	3-deoxy-D-manno-octulosonate 8-phosphate phosphatase (KD(	69.92550246	-0.985548253	0.501230899	-1.966255981	0.049269049	0.630817589
2.7.1.53	L-xylulokinase	7.910272842	1.559645406	0.795025007	1.961756412	0.049790851	0.630817589
4.1.2.19	rhamnulose-1-phosphate aldolase	129.9339125	-0.848611076	0.435164656	-1.950091913	0.051165165	0.630817589
2.3.1.203	UDP-N-acetylbacillosamine N-acetyltransferase	3.040640095	-3.678803661	1.889873427	-1.946587326	0.051584229	0.630817589
4.2.1.32	#N/A	57.89837913	-1.336015678	0.68889029	-1.939373653	0.052455856	0.635694274
5.3.1.5	xylose isomerase	119.6721669	0.879736429	0.454733993	1.934617697	0.053037227	0.635694274
2.7.1.5	rhamnulokinase	286.9725374	-0.713169982	0.370019822	-1.927383184	0.053931894	0.635694274
3.1.13.5	ribonuclease D	6.041358097	-2.373147245	1.231082374	-1.927691676	0.053893488	0.635694274
0.0.0.0	#N/A	4559514.544	1.217244027	0.629192211	1.934613947	0.053037687	0.635694274
4.2.2.5	chondroitin AC lyase	46.22719144	-1.72688361	0.894002013	-1.931632797	0.053404845	0.635694274
3.2.1.183	UDP-N-acetylglucosamine 2-epimerase (hydrolysing)	12.14589756	-2.75573473	1.4334064	-1.922507622	0.054541915	0.6372455
2.3.1.81	aminoglycoside 3-N-acetyltransferase	34.85777649	-1.811703032	0.944215364	-1.918739199	0.055017348	0.6372455
1.7.1.15	nitrite reductase (NADH) large subunit	3.939076588	2.720570405	1.417929024	1.918692938	0.055023206	0.6372455

1.5.1.28	opine dehydrogenase	8.554545849	-1.778646225	0.935167879	-1.901953933	0.057177177	0.658363799
2.7.7.1	nicotinamide-nucleotide adenylyltransferase	3.591754005	-2.81834958	1.485631319	-1.897072001	0.057818436	0.658969227
1.1.1.69	gluconate 5-dehydrogenase	308.5135377	0.589194357	0.31067138	1.896519584	0.057891373	0.658969227
3.4.11.6	aminopeptidase B	12.58645714	-2.895334716	1.53125264	-1.890827575	0.058647365	0.659074439
2.4.1.7	sucrose phosphorylase	40.52910576	-1.496180021	0.792052999	-1.888989782	0.058893198	0.659074439
3.4.11.10	bacterial leucyl aminopeptidase	12.58645714	-2.895334716	1.53125264	-1.890827575	0.058647365	0.659074439
2.1.1.130	#N/A	73.91478762	-0.854207939	0.453661394	-1.882919618	0.059711262	0.659213956
2.1.1.151	precorrin-2/cobalt-factor-2 C20-methyltransferase	73.91478762	-0.854207939	0.453661394	-1.882919618	0.059711262	0.659213956
3.4.17.11	glutamate carboxypeptidase	31.27839536	-1.05302305	0.559660105	-1.881540313	0.059898457	0.659213956
2.8.4.5	threonylcarbamoyladenosine tRNA methylthiotransferase CDK/	336.5404218	-0.595795393	0.318800001	-1.868868856	0.061641062	0.6695032
5.4.99.62	D-ribose pyranase	11.97500773	-2.199194156	1.179176125	-1.86502602	0.062177757	0.6695032
3.5.2.7	imidazolonepropionase	168.0587454	0.784795758	0.419514766	1.870722607	0.06138354	0.6695032
2.7.7.18	nicotinate-nucleotide adenylyltransferase	289.546131	-0.436786986	0.233961332	-1.866919559	0.061912822	0.6695032
2.6.1.37	2-aminoethylphosphonate-pyruvate transaminase	28.43514803	-1.696175944	0.921809632	-1.840050141	0.065760876	0.673981583
2.8.1.11	adenylyltransferase and sulfurtransferase	38.27308209	-1.253374769	0.680353964	-1.842239238	0.065440163	0.673981583
2.7.7.80	adenylyltransferase and sulfurtransferase	38.27308209	-1.253374769	0.680353964	-1.842239238	0.065440163	0.673981583
1.1.1.23	histidinol dehydrogenase	231.2619341	-0.304498812	0.165961094	-1.834760214	0.066541226	0.673981583
3.2.1.78	mannan endo-1,4-beta-mannosidase	244.4033901	0.989211461	0.537660134	1.839845281	0.065790955	0.673981583
3.2.1.10	oligo-1,6-glucosidase	174.9034232	-0.86584017	0.477113119	-1.814748193	0.069562615	0.673981583
4.1.1.44	4-carboxymuconolactone decarboxylase	73.14122184	-0.978492451	0.539089027	-1.815085082	0.069510835	0.673981583
5.1.3.14	UDP-N-acetylglucosamine 2-epimerase (non-hydrolysing)	267.9615043	-0.430323048	0.232475876	-1.851043882	0.06416324	0.673981583
4.1.1.70	glutaconyl-CoA decarboxylase	137.1694256	1.015847228	0.560763929	1.811541677	0.070057044	0.673981583
3.6.3.17	simple sugar transport system ATP-binding protein	2360.199292	0.733107156	0.399131413	1.836756347	0.066245873	0.673981583
2.1.1.199	16S rRNA (cytosine1402-N4)-methyltransferase	1164.169383	0.707925814	0.390266304	1.81395577	0.069684536	0.673981583
2.1.1.107	uroporphyrin-III C-methyltransferase	93.24422569	-0.659360919	0.35892232	-1.837057444	0.066201416	0.673981583
1.2.1.12	glyceraldehyde 3-phosphate dehydrogenase	3426.713094	1.022636299	0.56515303	1.809485653	0.070375587	0.673981583
3.2.2.20	DNA-3-methyladenine glycosylase I	67.37949366	-1.248197358	0.689086572	-1.811379599	0.070082112	0.673981583
3.4.11.1	leucyl aminopeptidase	44.43899661	0.756127065	0.414745499	1.823110958	0.068286587	0.673981583
6.3.4.3	methylenetetrahydrofolate dehydrogenase (NADP+) / metheny	810.1039202	0.532428498	0.289451363	1.839440288	0.065850453	0.673981583
3.4.11.19	D-aminopeptidase	57.32240294	0.834505071	0.45662173	1.827563201	0.067615133	0.673981583
1.8.5.2	thiosulfate dehydrogenase [quinone] small subunit	17.43969102	-2.325369531	1.277931142	-1.819636016	0.068814453	0.673981583
3.1.-.-	#N/A	7256.639758	0.392630721	0.21558952	1.821195769	0.068577103	0.673981583
1.17.1.4	xanthine dehydrogenase molybdenum-binding subunit	365.9000832	1.002060354	0.546930322	1.832153591	0.066928541	0.673981583
3.1.4.52	c-di-GMP phosphodiesterase	11.05301254	-1.659493504	0.910982711	-1.82165203	0.068507801	0.673981583
4.2.1.2	#N/A	775.0896236	0.4321231	0.233725253	1.848850713	0.064479372	0.673981583
1.6.1.2	H+-translocating NAD(P) transhydrogenase	7.406383389	-3.029591617	1.666550552	-1.81788162	0.069082228	0.673981583
4.2.1.20	tryptophan synthase	461.3621495	-0.480319941	0.266273107	-1.803862003	0.071252943	0.675885058
5.1.3.22	L-ribulose-5-phosphate 3-epimerase	8.998605211	-1.793869756	0.993865003	-1.804943076	0.07108359	0.675885058
1.97.1.4	#N/A	2043.548934	0.926437897	0.516397511	1.794040207	0.072806752	0.687350944
2.8.1.-	#N/A	58.92031517	1.242522872	0.694579248	1.788885681	0.073633228	0.691874485
3.6.3.-	#N/A	1695.617713	-0.330101225	0.185639394	-1.778185211	0.075373441	0.692747356
1.2.1.81	sulfoacetaldehyde dehydrogenase	9.761748302	1.336189849	0.749549517	1.782657208	0.074642121	0.692747356
2.3.1.1	amino-acid N-acetyltransferase	187.3742114	-0.428532126	0.240432588	-1.78233795	0.074694138	0.692747356
2.1.1.228	tRNA (guanine37-N1)-methyltransferase	241.8350834	-0.437927263	0.246647872	-1.775516081	0.075812713	0.692747356
4.1.3.38	4-amino-4-deoxychorismate lyase	50.77012324	-1.453286125	0.817746221	-1.777184765	0.075537845	0.692747356
2.6.99.2	pyridoxine 5-phosphate synthase	56.3305921	-0.938000007	0.527467621	-1.778308222	0.075353247	0.692747356
2.8.3.12	#N/A	134.3823044	0.893465755	0.505461716	1.767623	0.07712395	0.695162485
4.1.1.87	malonate decarboxylase beta subunit	7.252252901	-2.273181663	1.285389464	-1.76847697	0.076981199	0.695162485
3.1.6.6	choline-sulfatase	24.56530346	-1.661666959	0.939231806	-1.769176627	0.076864403	0.695162485
5.1.3.6	UDP-glucuronate 4-epimerase	20.60700147	-1.783903993	1.016825737	-1.754385169	0.079364531	0.705911617
1.16.3.1	bacterioferritin	121.0308506	0.864969577	0.492469284	1.756392949	0.079021336	0.705911617
2.6.1.83	LL-diaminopimelate aminotransferase	298.573527	-0.476192406	0.271443427	-1.754297058	0.07937962	0.705911617
2.7.7.23	UDP-N-acetylglucosamine/UDP-N-acetylgalactosamine diphosp	466.8862771	0.75041773	0.43079242	1.741947385	0.081517651	0.706192206
5.3.1.9	glucose-6-phosphate isomerase	996.9255714	0.639992986	0.365879975	1.749188342	0.080258469	0.706192206
2.4.2.29	queuine tRNA-ribosyltransferase	301.7581135	-0.368998867	0.211154957	-1.747526429	0.080546065	0.706192206
2.4.2.2	pyrimidine-nucleoside phosphorylase	168.5824076	0.587636832	0.337367567	1.741829651	0.081538257	0.706192206
6.3.2.4	D-alanine-D-alanine ligase	331.7670026	-0.33822708	0.193916777	-1.744186781	0.081126526	0.706192206
5.4.99.21	23S rRNA pseudouridine2604 synthase	51.97311598	-0.977878785	0.559080619	-1.749083677	0.080276556	0.706192206
1.1.1.193	5-amino-6-(5-phosphoribosylamino)uracil reductase	148.3103672	-0.648666889	0.373408117	-1.737152622	0.082360235	0.710223327
3.5.4.26	diaminohydroxyphosphoribosylaminopyrimidine deaminase	147.8638147	-0.645111483	0.372459731	-1.732030148	0.083268195	0.71188946
2.7.1.163	hygromycin-B 4-O-kinase	4.223768401	3.208967334	1.850659106	1.733959173	0.082925327	0.71188946
1.12.2.1	NiFe] hydrogenase large subunit	10.41753835	-2.556424212	1.479537129	-1.727854044	0.084014395	0.715199468
3.4.11.-	#N/A	205.27062	0.647502446	0.376518024	1.719711686	0.085484863	0.718608074
1.1.1.29	glycerate dehydrogenase	213.4454083	0.348109125	0.202430842	1.719644704	0.085497045	0.718608074
6.3.3.3	dethiobiotin synthetase	100.4835213	-1.221530004	0.709672874	-1.721257848	0.085204045	0.718608074
2.1.1.144	trans-aconitate 2-methyltransferase	2.013320454	-2.794256308	1.627317381	-1.717093629	0.085962066	0.719480822
5.4.2.8	phosphomannomutase	156.1336159	0.55408984	0.323625673	1.712131905	0.086872363	0.722564595
3.4.11.5	proline iminopeptidase	13.29992319	-1.361826532	0.797027599	-1.708631588	0.087519214	0.722564595
3.5.1.4	amidase	10.62089373	-1.8034662	1.056376888	-1.707218532	0.087781442	0.722564595
3.2.1.28	alpha,alpha-trehalase	2.844993824	-2.50072495	1.462753605	-1.709600948	0.08733969	0.722564595
2.4.1.211	1,3-beta-galactosyl-N-acetylhexosamine phosphorylase	886.5408123	-1.08106238	0.637247201	-1.69645685	0.089799395	0.728032065
2.3.1.157	bifunctional UDP-N-acetylglucosamine pyrophosphorylase / Glu	425.7922078	0.781497039	0.46175058	1.692465744	0.090557206	0.728032065
1.3.1.34	2,4-dienoyl-CoA reductase (NADPH2)	4.426268906	-2.503963352	1.480201692	-1.691636597	0.090715284	0.728032065
3.4.17.19	carboxypeptidase Taq	71.80582444	0.640857625	0.377231008	1.698846626	0.089348086	0.728032065
4.1.1.81	threonine-phosphate decarboxylase	111.5133339	-0.561177091	0.332032856	-1.690125182	0.091004008	0.728032065
1.3.5.4	succinate dehydrogenase / fumarate reductase, flavoprotein su	851.5573064	0.392831758	0.232047906	1.692890766	0.090476261	0.728032065
3.1.3.41	4-nitrophenyl phosphatase	2.491093023	3.467416617	2.039507368	1.700124585	0.089107494	0.728032065
4.3.1.1	aspartate ammonia-lyase	327.7532297	0.781207338	0.464496134	1.681838191	0.092600221	0.737838559
2.5.1.7	UDP-N-acetylglucosamine 1-carboxyvinyltransferase	705.9287071	0.286116564	0.170828408	1.674876951	0.093958377	0.745677636
5.4.2.6	beta-phosphoglucomutase	14.7895933	-1.360967081	0.814050457	-1.6718461	0.094554674	0.747432183
3.4.22.34	legumain	232.7625314	2.434001936	1.460986453	1.665998977	0.095713618	0.747692265
3.2.1.31	beta-glucuronidase	65.88671643	-0.895500811	0.537363659	-1.666470734	0.095619693	0.747692265
1.8.1.9	thioredoxin reductase (NADPH)	2323.064914	0.797904115	0.477924126	1.669520477	0.095014276	0.747692265
3.1.3.26	4-phytase / acid phosphatase	3.711875381	2.133655802	1.284987126	1.660449166	0.096824126	0.749426841
2.4.-.-	#N/A	68.04439895	-0.887027607	0.533463805	-1.662769991	0.096358486	0.749426841
6.3.2.5	phosphopantothenoylcysteine decarboxylase / phosphopantot	273.46446	0.264419013	0.159360035	1.659255492	0.09706432	0.749426841
2.4.2.14	amidophosphoribosyltransferase	632.8867892	0.265787483	0.160419923	1.656823401	0.097555185	0.750308601

4.2.2.2	pectate lyase	2.166420747	3.952869043	2.399616149	1.647292232	0.099497994	0.759386987
1.17.99.6	epoxyqueuosine reductase	17.31413246	-1.462780493	0.887843359	-1.647565956	0.099441771	0.759386987
3.6.1.45	UDP-sugar diphosphatase	164.3997029	0.586754777	0.357671084	1.640487038	0.100903941	0.76489858
2.7.7.65	two-component system, cell cycle response regulator	2.26188146	-3.882948829	2.369110381	-1.638990256	0.101215289	0.76489858
6.3.4.19	tRNA(Ile)-lysidine synthase	409.2291933	0.270228005	0.164950417	1.638237782	0.101372101	0.76489858
4.2.2.3	poly(beta-D-mannuronate) lyase	4.039561784	-3.960961953	2.429000982	-1.630695905	0.102954501	0.773907043
3.4.22.1	cathepsin B	63.68857474	3.011590778	1.850256931	1.627660855	0.103596818	0.774376334
4.2.1.10	#N/A	252.2617019	-0.454753469	0.280247568	-1.6226848	0.104656802	0.774376334
2.4.1.18	1,4-alpha-glucan branching enzyme	1083.344365	0.466099631	0.287491189	1.621265794	0.104960648	0.774376334
2.7.7.9	UTP--glucose-1-phosphate uridylyltransferase	171.0660117	0.525996793	0.324127768	1.622806945	0.104630681	0.774376334
3.2.2.4	AMP nucleosidase	32.96225727	-1.129365008	0.695957048	-1.622751017	0.104642641	0.774376334
4.2.2.7	heparin lyase	6.977243599	-2.212683463	1.366686637	-1.619013022	0.105444461	0.775075155
4.2.1.49	urocanate hydratase	221.4685891	0.694289801	0.430712672	1.611955826	0.106971553	0.778061147
5.4.99.22	23S rRNA pseudouridine2605 synthase	322.0947105	0.478854061	0.297613801	1.608978007	0.107621151	0.778061147
5.3.1.16	phosphoribosylformimino-5-aminoimidazole carboxamide ribot	198.1663491	-0.465307784	0.289665459	-1.606362679	0.108194246	0.778061147
2.2.1.1	transketolase	1381.337546	0.351845113	0.218309846	1.611677711	0.10703209	0.778061147
5.3.1.23	methylthioribose-1-phosphate isomerase	68.25774551	-0.61756252	0.382995374	-1.612454253	0.106863127	0.778061147
2.3.1.51	1-acyl-sn-glycerol-3-phosphate acyltransferase	441.3538744	0.510878347	0.317908152	1.606999831	0.108054405	0.778061147
3.5.1.32	hippurate hydrolase	22.867801	-1.154089438	0.719484185	-1.604051154	0.108702777	0.778906232
1.11.1.9	glutathione peroxidase	87.79499751	0.799277908	0.499840666	1.599065388	0.109806074	0.780112351
2.4.1.180	UDP-N-acetyl-D-mannosaminouronate:lipid I N-acetyl-D-mannc	4.766590627	-3.459242324	2.167210407	-1.59617281	0.110450216	0.780112351
4.1.1.36	phosphopantothenoylcysteine decarboxylase	273.1904406	0.264278995	0.165259301	1.599177739	0.109781115	0.780112351
4.2.1.113	O-succinylbenzoate synthase	6.192673817	-2.616626364	1.641405039	-1.594138133	0.1109051	0.780112351
1.1.1.14	L-iditol 2-dehydrogenase	103.1114767	-0.754296153	0.473072229	-1.594462974	0.110832377	0.780112351
2.3.1.57	diamine N-acetyltransferase	81.34146399	-0.829907744	0.521059998	-1.592729721	0.111220837	0.780112351
2.3.1.201	UDP-2-acetamido-3-amino-2,3-dideoxy-glucuronate N-acetyltra	22.62710242	1.239223908	0.780820334	1.587079452	0.112494651	0.781073699
2.7.1.197	PTS system, mannitol-specific IIA component	35.32517345	-0.888534183	0.55980099	-1.587232247	0.112460054	0.781073699
2.7.1.180	FAD:protein FMN transferase	243.1625185	-0.562442999	0.354427693	-1.586904778	0.112534213	0.781073699
1.3.1.106	precorrin-6A/cobalt-precorrin-6A reductase	80.75062147	0.955920198	0.605557877	1.578577761	0.114432939	0.782921367
1.3.1.54	precorrin-6A/cobalt-precorrin-6A reductase	80.75062147	0.955920198	0.605557877	1.578577761	0.114432939	0.782921367
4.2.1.75	uroporphyrinogen-III synthase	157.1676558	-0.412662757	0.260634034	-1.583303417	0.113352324	0.782921367
3.6.5.-	#N/A	429.493709	0.319604257	0.20264926	1.577130147	0.114765582	0.782921367
3.4.22.16	cathepsin H	6.140464691	6.360715223	4.020693077	1.581994721	0.113650776	0.782921367
2.3.1.169	#N/A	421.7701187	0.884112915	0.562412196	1.572001676	0.115950163	0.788302812
5.4.99.12	#N/A	317.454115	-0.38149962	0.243519329	-1.566609192	0.117206068	0.794130909
6.3.2.45	UDP-N-acetylmuramate: L-alanyl-gamma-D-glutamyl-meso-diar	13.36009396	-1.521013064	0.972221857	-1.564471167	0.117706959	0.794821229
3.4.22.-	#N/A	6.960518191	2.914440153	1.885329417	1.545851949	0.122140328	0.795205554
2.7.7.42	[glutamine synthetase] adenylyltransferase / [glutamine synthe	10.5009941	1.504706888	0.974391605	1.544252721	0.122527121	0.795205554
2.1.1.45	thymidylate synthase	183.6399445	-0.552507345	0.357808956	-1.544140622	0.12255427	0.795205554
4.1.2.14	2-dehydro-3-deoxyphosphogluconate aldolase / (4S)-4-hydroxy	296.8325156	0.454136435	0.29384956	1.545472574	0.122231998	0.795205554
2.1.2.2	phosphoribosylglycinamide formyltransferase	412.4568963	0.186486598	0.119444031	1.561288548	0.118455684	0.795205554
3.2.1.-	#N/A	106.9534303	0.647254919	0.417390514	1.550717849	0.120969315	0.795205554
1.8.99.5	dissimilatory sulfite reductase alpha subunit	53.43867049	0.97911665	0.63162133	1.550164004	0.121102157	0.795205554
2.7.7.89	[glutamine synthetase] adenylyltransferase / [glutamine synthe	10.5009941	1.504706888	0.974391605	1.544252721	0.122527121	0.795205554
1.7.99.1	hydroxylamine reductase	187.9977427	-0.745563319	0.480170721	-1.552704666	0.120493707	0.795205554
3.2.1.20	alpha-glucosidase	623.9134192	-0.583674141	0.375294548	-1.555242791	0.119888257	0.795205554
4.1.3.42	2-dehydro-3-deoxyphosphogluconate aldolase / (4S)-4-hydroxy	296.8325156	0.454136435	0.29384956	1.545472574	0.122231998	0.795205554
1.2.1.38	N-acetyl-gamma-glutamyl-phosphate reductase	189.9986926	-0.519189	0.333977713	-1.554561816	0.120050464	0.795205554
2.7.1.30	glycerol kinase	776.031683	0.549772411	0.35689602	1.540427407	0.123456205	0.798457015
3.4.21.83	oligopeptidase B	1.520068509	-3.173638702	2.06710328	-1.535307274	0.124708368	0.803945209
3.1.3.27	phosphatidylglycerophosphatase GEP4	37.35501302	-1.376460426	0.899964732	-1.529460408	0.126150347	0.804503529
4.3.1.17	L-serine dehydratase	492.0753982	0.429549402	0.280508685	1.531323005	0.125689583	0.804503529
4.2.1.159	dTDP-4-dehydro-6-deoxy-alpha-D-glucopyranose 2,3-dehydrata	3.686345816	3.6800985	2.407212379	1.528780149	0.126318955	0.804503529
1.13.12.16	nitronate monooxygenase	2.660689283	-2.882586512	1.886001797	-1.528411328	0.126410444	0.804503529
3.5.4.25	GTP cyclohydrolase II	294.5812985	0.462693659	0.303387953	1.525089094	0.127236877	0.807184265
6.3.5.10	adenosylcobyric acid synthase	172.5070828	-0.475282877	0.312502854	-1.520891317	0.128287113	0.811263269
1.2.1.70	glutamyl-tRNA reductase	46.88750121	-1.019501781	0.673286127	-1.514217717	0.12997063	0.819308526
2.3.1.15	glycerol-3-phosphate O-acyltransferase 1/2	513.1341149	-0.352781321	0.234582501	-1.503868873	0.132615133	0.828117069
2.8.4.3	tRNA-2-methylthio-N6-dimethylallyladenosine synthase	406.9485174	0.277723474	0.184401171	1.506083027	0.132045861	0.828117069
4.1.99.19	2-iminoacetate synthase	230.7809752	-0.536461507	0.356571528	-1.504499001	0.13245293	0.828117069
4.1.99.12	3,4-dihydroxy 2-butanone 4-phosphate synthase	303.3060112	0.450181911	0.300248319	1.499365299	0.133778891	0.832773595
2.7.1.193	PTS system, N-acetylglucosamine-specific IIA component	552.7318893	0.721929044	0.482386532	1.496577943	0.134503113	0.834673524
2.8.3.-	#N/A	14.81102674	-0.969456355	0.651118226	-1.488909872	0.136511104	0.836708056
6.3.5.9	cobyrinic acid a,c-diamide synthase	147.2244454	-0.475554834	0.319309342	-1.489323274	0.136402262	0.836708056
6.3.5.11	cobyrinic acid a,c-diamide synthase	147.2244454	-0.475554834	0.319309342	-1.489323274	0.136402262	0.836708056
2.3.1.183	phosphinothricin acetyltransferase	219.4034355	0.970903738	0.650219211	1.493194483	0.135386286	0.836708056
2.5.1.16	spermidine synthase	74.31269831	-0.683047137	0.459821795	-1.485460552	0.137421866	0.839706615
2.5.1.9	#N/A	543.7887389	-1.186887396	0.803088938	-1.477902807	0.139433795	0.849321184
2.4.1.129	monofunctional glycosyltransferase	61.93255458	0.541892353	0.367046951	1.47635705	0.139848066	0.849321184
2.6.1.1	aspartate aminotransferase, chloroplastic	520.0808933	0.384013413	0.260404447	1.474680702	0.140298407	0.849466341
2.7.1.208	PTS system, alpha-glucoside-specific IIB component	59.53385477	-0.928718039	0.63069457	-1.472532162	0.140877228	0.850386179
1.2.7.5	aldehyde:ferredoxin oxidoreductase	32.16256784	0.934118037	0.640644741	1.458090542	0.144815588	0.853469383
2.1.1.245	acetyl-CoA decarbonylase/synthase complex subunit delta	736.5094664	0.92182554	0.629639453	1.464053015	0.143179463	0.853469383
3.1.1.83	epsilon-lactone hydrolase	8.852331657	-1.515956827	1.039507753	-1.458341049	0.144746561	0.853469383
2.7.7.6	DNA-directed RNA polymerase	5724.757651	0.505204276	0.344421199	1.466821082	0.142424733	0.853469383
2.7.1.14	sedoheptulokinase	8.938681165	-2.002581744	1.368571221	-1.463264544	0.143395006	0.853469383
6.3.4.13	phosphoribosylamine---glycine ligase	488.5378408	0.221758201	0.151065594	1.467959676	0.142115176	0.853469383
3.1.22.4	crossover junction endodeoxyribonuclease RuvC	139.4664006	-0.405786012	0.278183521	-1.458698956	0.144647984	0.853469383
2.5.1.49	O-acetylhomoserine (thiol)-lyase	646.0339653	-0.32933515	0.225432299	-1.460904901	0.144041541	0.853469383
5.4.99.5	chorismate mutase	299.5030678	-0.403217697	0.278165883	-1.449558417	0.147181699	0.859782828
2.5.1.72	quinolinate synthase	156.0969679	-0.311681699	0.214870283	-1.450557496	0.146903118	0.859782828
2.3.1.39	acyl-carrier-protein] S-malonyltransferase	274.8139792	-0.488336949	0.336710068	-1.450318822	0.146969633	0.859782828
5.4.2.10	phosphoglucosamine mutase	492.1079234	0.392891205	0.271766576	1.445693617	0.148263155	0.861050162
6.1.1.15	prolyl-tRNA synthetase	485.2734754	-0.224616554	0.155286759	-1.446463013	0.148047379	0.861050162
2.7.7.38	3-deoxy-manno-octulosonate cytidylyltransferase (CMP-KDO sy	88.70939335	-0.622081205	0.430921041	-1.44360833	0.148849181	0.861940604
6.1.1.14	#N/A	630.4145281	0.330413027	0.234678032	1.407941866	0.159148285	0.862494941
4.2.2.20	chondroitin-sulfate-ABC endolyase/exolyase	92.73215075	-0.925756967	0.645780394	-1.43354765	0.151701392	0.862494941

5.4.99.60	precorrin-8X/cobalt-precorrin-8 methylmutase	102.094744	0.6945493	0.507373316	1.368911763	0.171026858	0.862494941
2.1.1.187	23S rRNA (guanine745-N1)-methyltransferase	129.5080327	0.507859344	0.363816826	1.39592044	0.162738455	0.862494941
3.4.11.18	methionyl aminopeptidase	1044.301231	0.440085299	0.318159326	1.383223004	0.166596498	0.862494941
6.3.2.1	pantoate--beta-alanine ligase	150.1648978	-0.508972141	0.369810334	-1.376305893	0.168726949	0.862494941
3.1.3.-	#N/A	200.6898365	-0.389383343	0.273054256	-1.426029201	0.153859913	0.862494941
2.7.1.45	2-dehydro-3-deoxygluconokinase	519.2465247	0.345475743	0.248041797	1.392812611	0.163676464	0.862494941
1.18.1.2	ferredoxin/flavodoxin---NADP+ reductase	421.7852528	0.574023164	0.401546116	1.429532354	0.152851283	0.862494941
3.4.18.1	cathepsin X	33.57074902	1.779928815	1.257287016	1.415690126	0.15686625	0.862494941
2.7.4.6	nucleoside-diphosphate kinase	77.16799679	-0.58452747	0.422765532	-1.382628017	0.166778954	0.862494941
5.4.99.2	methylmalonyl-CoA mutase	436.038468	0.439275098	0.307887107	1.4267408	0.153654621	0.862494941
2.7.1.196	PTS system, cellobiose-specific IIA component	42.12668325	-0.714603854	0.511290777	-1.397646674	0.162219195	0.862494941
2.4.2.7	adenine phosphoribosyltransferase	220.4972688	0.471775443	0.343311521	1.374190536	0.169382538	0.862494941
5.3.2.5	2,3-diketo-5-methylthiopentyl-1-phosphate enolase	7.481498469	1.356423465	0.949068889	1.429214971	0.152942456	0.862494941
2.4.1.280	N,N'-diacetylchitobiose phosphorylase	5.176582286	-2.228131741	1.546115221	-1.441116232	0.149551848	0.862494941
2.7.7.7	#N/A	4748.086639	0.300797769	0.218220714	1.378410709	0.168076519	0.862494941
3.2.1.14	chitinase	74.35089469	-0.94598889	0.683705071	-1.383621287	0.166474447	0.862494941
3.6.3.28	#N/A	9.816075023	-1.606840943	1.154429792	-1.391891437	0.163955275	0.862494941
6.1.1.18	glutaminyl-tRNA synthetase	439.2984639	-0.257762412	0.186746663	-1.380278543	0.167500899	0.862494941
2.3.1.35	glutamate N-acetyltransferase / amino-acid N-acetyltransferase	176.3579632	-0.353320681	0.254930739	-1.385947737	0.165762863	0.862494941
2.7.10.-	#N/A	1.425729739	-2.529278225	1.758139311	-1.438610813	0.150260821	0.862494941
2.7.8.26	adenosylcobinamide-GDP ribazoletransferase	117.5949016	-0.565330813	0.401806043	-1.406974392	0.159434983	0.862494941
2.5.1.54	3-deoxy-7-phosphoheptulonate synthase	4039.606101	1.427907355	1.020761842	1.398864354	0.161853664	0.862494941
2.1.1.10	homocysteine S-methyltransferase	5.268345962	-2.031543923	1.441730491	-1.409101033	0.158805295	0.862494941
2.3.1.30	serine O-acetyltransferase	298.4087406	-0.4029703	0.284665003	-1.415594804	0.156894173	0.862494941
4.2.99.20	2-succinyl-6-hydroxy-2,4-cyclohexadiene-1-carboxylate synthas	4.655924674	-2.298826537	1.6741061	-1.373166573	0.169700569	0.862494941
1.1.1.31	3-hydroxyisobutyrate dehydrogenase	19.43101592	0.580627616	0.412224946	1.408521299	0.158976765	0.862494941
4.1.1.48	indole-3-glycerol phosphate synthase	96.2007013	-0.663960166	0.481733708	-1.378272177	0.16811927	0.862494941
6.1.1.6	#N/A	607.4222191	0.396887598	0.289079826	1.372934266	0.169772783	0.862494941
6.1.1.24	nondiscriminating glutamyl-tRNA synthetase	13.45465817	0.84060604	0.603971668	1.391797141	0.163983836	0.862494941
3.6.3.16	arsenite/tail-anchored protein-transporting ATPase	8.08124422	-1.667629236	1.169051104	-1.426481041	0.153729536	0.862494941
4.6.1.12	2-C-methyl-D-erythritol 2,4-cyclodiphosphate synthase	226.1923799	-0.303131224	0.219842388	-1.378857035	0.167938837	0.862494941
3.2.1.45	glucosylceramidase	63.37924565	-1.115409056	0.793572654	-1.405553796	0.159856664	0.862494941
3.1.3.11	fructose-1,6-bisphosphatase I / sedoheptulose-1,7-bisphosphat	458.6616046	0.331339902	0.233734967	1.417588074	0.156311064	0.862494941
5.4.99.61	precorrin-8X/cobalt-precorrin-8 methylmutase	102.094744	0.6945493	0.507373316	1.368911763	0.171026858	0.862494941
3.5.3.12	agmatine deiminase	62.47918888	-0.683593516	0.483696213	-1.413270347	0.157576249	0.862494941
2.5.1.55	2-dehydro-3-deoxyphosphooctonate aldolase (KDO 8-P synthas	101.1365769	-0.55649007	0.403760216	-1.378268706	0.168120341	0.862494941
4.2.3.5	chorismate synthase	374.3081163	0.362156425	0.264248999	1.370512006	0.17052713	0.862494941
3.5.1.2	glutaminase	188.4444747	0.663755554	0.471366049	1.408153079	0.159085747	0.862494941
2.7.1.50	hydroxyethylthiazole kinase	118.3186211	-0.53707008	0.376845862	-1.42517176	0.154107557	0.862494941
4.2.2.21	chondroitin-sulfate-ABC endolyase/exolyase	92.73215075	-0.925756967	0.645780394	-1.43354765	0.151701392	0.862494941
2.7.1.205	PTS system, cellobiose-specific IIA component	42.12668325	-0.714603854	0.511290777	-1.397646674	0.162219195	0.862494941
3.1.6.1	arylsulfatase	68.00014758	-1.249972083	0.875289532	-1.428066985	0.153272579	0.862494941
2.7.1.148	4-diphosphocytidyl-2-C-methyl-D-erythritol kinase	306.3553243	0.371801253	0.266305931	1.396143344	0.162671334	0.862494941
6.3.1.10	adenosylcobinamide-phosphate synthase	125.3070087	-0.548924649	0.387319842	-1.417238647	0.156413166	0.862494941
3.1.1.32	phospholipase A1/A2	2.533868293	-2.779833752	2.030229325	-1.369221554	0.17093003	0.862494941
5.3.1.26	galactose-6-phosphate isomerase	8.787182483	2.143349174	1.542624887	1.389416955	0.164705997	0.862494941
2.3.1.128	#N/A	187.3750258	0.685200952	0.494350732	1.38606238	0.165727856	0.862494941
2.1.1.170	16S rRNA (guanine527-N7)-methyltransferase	266.2469813	-0.490677291	0.345631665	-1.419653757	0.155708507	0.862494941
3.1.1.4	secretory phospholipase A2	2.533868293	-2.779833752	2.030229325	-1.369221554	0.17093003	0.862494941
4.4.1.8	#N/A	447.9802593	0.485090612	0.356258212	1.361626472	0.173315797	0.86686351
6.1.1.5	isoleucyl-tRNA synthetase	783.2345106	0.317781497	0.233323919	1.36197565	0.17320557	0.86686351
2.7.7.8	polyribonucleotide nucleotidyltransferase	1007.659678	0.340863815	0.251027854	1.357872482	0.17450415	0.86686351
3.6.1.15	#N/A	4.552541307	-2.394437854	1.762557296	-1.358502137	0.174304405	0.86686351
1.5.1.3	dihydrofolate reductase	135.387892	-0.467942974	0.3433113	-1.363028175	0.172873631	0.86686351
2.4.1.4	amylosucrase	262.5900682	0.604361506	0.444897168	1.358429653	0.17432739	0.86686351
5.1.3.29	L-fucose mutarotase	71.42123421	-0.74680846	0.550582357	-1.356397368	0.17497277	0.86702925
3.2.1.93	trehalose-6-phosphate hydrolase	14.12340887	-1.022643888	0.756247177	-1.352261428	0.176291699	0.871397181
6.1.1.10	methionyl-tRNA synthetase	823.9019929	-0.473105955	0.350360975	-1.350338618	0.17690739	0.872276044
4.1.2.9	xylulose-5-phosphate/fructose-6-phosphate phosphoketolase	113.3229537	0.412752843	0.306764028	1.345506006	0.178461883	0.87345472
2.4.2.10	orotate phosphoribosyltransferase	556.3995842	0.375548282	0.278564834	1.348153951	0.177608872	0.87345472
4.1.2.22	xylulose-5-phosphate/fructose-6-phosphate phosphoketolase	113.3229537	0.412752843	0.306764028	1.345506006	0.178461883	0.87345472
3.1.4.-	#N/A	196.6449542	-0.3780239	0.283044461	-1.335563672	0.181691931	0.880609065
1.5.98.2	5,10-methylenetetrahydromethanopterin reductase	40.82137094	1.800810152	1.345532901	1.338362035	0.180778448	0.880609065
2.1.1.258	5-methyltetrahydrofolate corrinoid/iron sulfur protein methyltr	251.1608261	0.845927522	0.632668454	1.337078713	0.181196945	0.880609065
3.6.3.25	sulfate transport system ATP-binding protein	37.65838914	-1.066787028	0.798090512	-1.33667424	0.181328994	0.880609065
1.1.1.125	#N/A	21.0930698	-0.889977967	0.667652803	-1.332995177	0.182533386	0.882540063
4.3.1.3	histidine ammonia-lyase	217.1796459	0.496046818	0.373023675	1.329799826	0.183584233	0.883332832
2.7.1.201	PTS system, trehalose-specific IIA component	10.53537854	-1.423870326	1.07014639	-1.330537896	0.183341109	0.883332832
3.5.1.42	nicotinamide-nucleotide amidase	507.4585151	0.504524368	0.38064804	1.325435351	0.185026799	0.885875573
2.7.7.72	tRNA nucleotidyltransferase (CCA-adding enzyme)	197.4056266	-0.369542344	0.279293189	-1.323134104	0.185790785	0.885875573
2.4.1.315	processive 1,2-diacylglycerol beta-glucosyltransferase	64.5026741	-0.426349402	0.322300716	-1.322831074	0.185891561	0.885875573
1.2.7.-	#N/A	4868.982823	0.680700236	0.514158998	1.323909994	0.185532939	0.885875573
3.1.3.82	D-glycero-D-manno-heptose 1,7-bisphosphate phosphatase	27.20891321	-0.752268299	0.570536714	-1.318527416	0.187327154	0.888465929
3.1.3.83	D-glycero-D-manno-heptose 1,7-bisphosphate phosphatase	27.20891321	-0.752268299	0.570536714	-1.318527416	0.187327154	0.888465929
1.2.7.1	#N/A	4974.621445	0.66920131	0.508303249	1.316539508	0.187993027	0.889506199
2.1.1.297	release factor glutamine methyltransferase	340.1934048	0.232492723	0.178321438	1.303784477	0.192307077	0.899260399
2.5.1.31	undecaprenyl diphosphate synthase	411.3346321	0.311769754	0.239260547	1.303055425	0.19255584	0.899260399
1.1.1.262	4-hydroxythreonine-4-phosphate dehydrogenase	161.576444	0.333263828	0.254830879	1.307784318	0.190946475	0.899260399
2.1.1.171	16S rRNA (guanine966-N2)-methyltransferase	10.12371931	-1.207264061	0.926053397	-1.303665712	0.192347585	0.899260399
2.6.1.22	4-aminobutyrate aminotransferase / (S)-3-amino-2-methylprop	25.41220286	0.861832498	0.661701839	1.302448396	0.192763148	0.899260399
3.1.4.55	phosphoribosyl 1,2-cyclic phosphate phosphodiesterase	141.2270294	0.485129882	0.37210783	1.303734678	0.192324061	0.899260399
3.2.1.96	mannosyl-glycoprotein endo-beta-N-acetylglucosaminidase	18.59201307	-1.289993063	0.992491455	-1.299752311	0.193685875	0.901453885
1.1.1.77	lactaldehyde reductase	361.3221681	0.354830399	0.273850254	1.295709582	0.195075559	0.905805392
3.5.1.44	chemotaxis protein CheD	22.51654174	-0.809055682	0.625817163	-1.292798808	0.196080653	0.906247472
3.1.12.1	CRISPR-associated exonuclease Cas4	70.35374821	-0.515237717	0.398163295	-1.2940362	0.195652917	0.906247472
5.3.1.17	4-deoxy-L-threo-5-hexosulose-uronate ketol-isomerase	242.425249	0.436148193	0.342320176	1.274094323	0.20262999	0.909940399
2.7.3.-	#N/A	31.26877507	-0.87661949	0.692045457	-1.266707961	0.205259732	0.909940399

3.4.11.2	aminopeptidase N	47.09178138	-0.702391796	0.551610639	-1.273347081	0.202894907	0.909940399
2.7.1.49	hydroxymethylpyrimidine/phosphomethylpyrimidine kinase / t	196.5194831	-0.348196063	0.274919966	-1.266536106	0.205321211	0.909940399
4.1.1.12	aspartate 4-decarboxylase	60.2823711	-0.883939215	0.689081157	-1.282779547	0.199569322	0.909940399
4.99.1.3	sirohydrochlorin cobaltochelatase	153.816797	0.455196163	0.355923031	1.278917415	0.200926139	0.909940399
1.4.1.11	L-erythro-3,5-diaminohexanoate dehydrogenase	90.5850665	0.623111281	0.487530146	1.278097953	0.201214891	0.909940399
2.7.4.3	adenylate kinase	755.136847	0.4518775	0.35226069	1.282792865	0.199564655	0.909940399
3.6.3.41	heme exporter protein A	3.666482455	1.874272864	1.470408355	1.274661462	0.202429093	0.909940399
2.3.1.19	phosphate butyryltransferase	55.23559176	-0.61887211	0.485863433	-1.27375733	0.202749432	0.909940399
2.7.6.1	ribose-phosphate pyrophosphokinase	590.4496245	0.429719566	0.339335142	1.266357393	0.205385158	0.909940399
3.6.3.29	molybdate transport system ATP-binding protein	78.3810439	-0.692227841	0.545709129	-1.268492324	0.20462219	0.909940399
2.8.3.10	#N/A	92.4380191	0.597809738	0.469191378	1.274127715	0.202618158	0.909940399
6.3.3.2	glutamate formiminotransferase / 5-formyltetrahydrofolate cyc	201.089566	-0.289617907	0.225489432	-1.284396808	0.199003149	0.909940399
3.5.4.13	dCTP deaminase	6.87186702	-1.011055324	0.801118664	-1.262054385	0.206929217	0.909940399
2.7.7.85	diadenylate cyclase	249.0442064	0.369337092	0.292453752	1.262890592	0.206628501	0.909940399
6.5.1.2	DNA ligase (NAD+)	464.5080408	0.285358792	0.225730195	1.26415871	0.206173068	0.909940399
5.1.99.1	methylmalonyl-CoA/ethylmalonyl-CoA epimerase	69.18936228	-0.484705519	0.376109592	-1.288734798	0.197490301	0.909940399
2.7.4.7	hydroxymethylpyrimidine/phosphomethylpyrimidine kinase / t	196.5194831	-0.348196063	0.274919966	-1.266536106	0.205321211	0.909940399
2.7.14.1	protein arginine kinase	137.5777148	0.820704296	0.649391407	1.26380529	0.206299922	0.909940399
3.5.1.-	#N/A	3277.707675	-1.328252346	1.051892295	-1.262726567	0.206687463	0.909940399
2.4.2.12	nicotinamide phosphoribosyltransferase	8.025752757	1.360966065	1.06898561	1.273137872	0.202969122	0.909940399
3.6.1.1	inorganic pyrophosphatase	703.8118417	0.265898286	0.211168341	1.259176852	0.20796646	0.912487201
3.2.1.21	beta-glucosidase	2657.934981	-0.725949084	0.578194592	-1.255544576	0.209281137	0.916237417
5.3.1.12	glucuronate isomerase	529.9970512	0.583430318	0.465534384	1.253248607	0.210115246	0.917871862
2.1.2.5	glutamate formiminotransferase / 5-formyltetrahydrofolate cyc	152.7191343	0.594840192	0.476383981	1.248656999	0.211790555	0.923165833
2.1.1.181	23S rRNA (adenine1618-N6)-methyltransferase	5.132909253	-1.651264015	1.324382111	-1.246818423	0.212464084	0.924079595
2.8.1.7	cysteine desulfurase	1139.077994	0.299366575	0.240499195	1.244771627	0.213215708	0.925328303
1.1.2.4	D-lactate dehydrogenase (cytochrome)	4.173147047	2.02036257	1.625089175	1.243231819	0.213782421	0.92577083
6.1.1.-	#N/A	6.459165869	-1.592424081	1.288985414	-1.235408922	0.216678354	0.936276097
2.1.1.207	tRNA (cytidine/uridine-2'-O-)-methyltransferase	169.8966336	-0.497836593	0.40501259	-1.229187943	0.219001348	0.940195444
4.1.3.39	4-hydroxy 2-oxovalerate aldolase	57.95554725	-0.555708389	0.451980198	-1.229497203	0.218885446	0.940195444
2.7.7.71	D-glycero-alpha-D-manno-heptose 1-phosphate guanylyltransfe	3.00025743	-3.516670592	2.860548451	-1.229369351	0.218933356	0.940195444
2.10.1.1	molybdopterin molybdotransferase	116.2328554	0.575270287	0.468677607	1.227432842	0.219659949	0.940994877
3.1.1.29	peptidyl-tRNA hydrolase, PTH1 family	279.5423892	-0.410536156	0.338261171	-1.213666217	0.22487521	0.943055618
2.7.7.27	glucose-1-phosphate adenylyltransferase	2589.01273	0.594664659	0.488197394	1.218082412	0.223192657	0.943055618
3.4.19.13	gamma-glutamyltranspeptidase / glutathione hydrolase	38.94457833	-0.93782477	0.771884634	-1.214980489	0.224373533	0.943055618
2.4.1.337	1,2-diacylglycerol 3-alpha-glucosyltransferase	134.8407682	0.645857528	0.528257841	1.222617966	0.221474024	0.943055618
4.3.1.4	formiminotetrahydrofolate cyclodeaminase	55.2180708	0.813184026	0.665565932	1.221793345	0.221785786	0.943055618
4.6.1.17	cyclic pyranopterin monophosphate synthase	67.32316178	-0.72091907	0.59380942	-1.214057988	0.224725581	0.943055618
4.1.1.11	aspartate 1-decarboxylase	122.8047799	-0.497807772	0.408311363	-1.219186673	0.222773349	0.943055618
2.3.2.3	phosphatidylglycerol lysyltransferase	2.629663203	-2.305650558	1.8883891	-1.22096159	0.222100563	0.943055618
2.3.2.2	gamma-glutamyltranspeptidase / glutathione hydrolase	38.94457833	-0.93782477	0.771884634	-1.214980489	0.224373533	0.943055618
3.2.1.50	alpha-N-acetylglucosaminidase	117.1078347	-0.589308641	0.482429241	-1.221544201	0.221880041	0.943055618
2.3.1.187	malonate decarboxylase alpha subunit	9.455022523	-1.601252628	1.32439425	-1.209045289	0.226645447	0.94667051
2.7.7.49	RNA-directed DNA polymerase	100.8236658	-0.658169886	0.544978228	-1.207699414	0.227162904	0.94667051
2.7.1.95	kanamycin kinase	7.098706671	-1.597129859	1.322319909	-1.207824104	0.227114928	0.94667051
1.4.1.13	glutamate synthase (NADPH) large chain	1037.619229	0.207819604	0.172497909	1.204765935	0.228293674	0.947418746
1.4.1.14	glutamate synthase (NADH)	1037.619229	0.207819604	0.172497909	1.204765935	0.228293674	0.947418746
3.2.1.11	dextranase	27.01727601	-1.050210722	0.874421769	-1.201034511	0.229737815	0.949455864
2.7.1.199	PTS system, glucose-specific IIB component	59.39654169	-0.726839099	0.604587277	-1.202207069	0.229283311	0.949455864
3.5.1.28	N-acetylmuramoyl-L-alanine amidase	773.0604699	-0.228105899	0.190721474	-1.196015813	0.231690388	0.949644555
3.1.26.3	#N/A	233.5547131	-0.392129916	0.327235285	-1.19831184	0.230795639	0.949644555
2.3.1.46	homoserine O-acetyltransferase/O-succinyltransferase	218.3870639	-0.388393292	0.324109731	-1.198338877	0.230785117	0.949644555
4.7.1.1	alpha-D-ribose 1-methylphosphonate 5-phosphate C-P lyase	3.093319832	-2.547010404	2.12912804	-1.19626925	0.231591504	0.949644555
6.1.1.2	tryptophanyl-tRNA synthetase	420.3540249	0.348335145	0.291948973	1.193137077	0.232815693	0.951534175
4.2.3.3	methylglyoxal synthase	510.2834238	0.348600731	0.292358857	1.192372737	0.233115125	0.951534175
1.1.1.346	2,5-diketo-D-gluconate reductase A	4.277529261	-1.769823375	1.485778058	-1.191176142	0.233584444	0.951534175
5.1.3.32	L-rhamnose mutarotase	119.1287929	-0.446449077	0.375956319	-1.18750252	0.235029463	0.951582704
2.7.9.2	pyruvate, water dikinase	53.46043133	0.519061659	0.436749484	1.188465418	0.234650097	0.951582704
1.1.1.57	fructuronate reductase	266.1380159	0.559586769	0.47120915	1.187554972	0.235008787	0.951582704
1.2.1.8	betaine-aldehyde dehydrogenase	10.0171133	1.32119993	1.114219603	1.185762597	0.235716066	0.952426784
2.7.4.16	thiamine-monophosphate kinase	118.4753888	0.448922031	0.37972746	1.182221666	0.237117758	0.953212473
3.5.1.91	N-substituted formamide deformylase	129.8540039	0.327272647	0.277547397	1.179159488	0.238334674	0.953212473
3.6.3.32	glycine betaine/proline transport system ATP-binding protein	73.99996966	-0.749966349	0.636623469	-1.178037545	0.238781639	0.953212473
2.-.-.-	#N/A	276.2509304	-0.333967017	0.282515491	-1.182119309	0.237158364	0.953212473
2.7.8.5	CDP-diacylglycerol---glycerol-3-phosphate 3-phosphatidyltransf	307.8633605	-0.432736619	0.367220382	-1.178411222	0.238632706	0.953212473
3.11.1.1	phosphonoacetaldehyde hydrolase	29.24177546	-0.871881602	0.73931879	-1.179303994	0.238277148	0.953212473
4.2.1.51	cyclohexadienyl dehydratase	217.7655411	-0.368490304	0.315461703	-1.168098381	0.242767083	0.965253552
1.1.1.339	dTDP-6-deoxy-L-talose 4-dehydrogenase (NAD+)	18.79373412	-0.635081812	0.543235883	-1.169071912	0.24237466	0.965253552
2.6.1.82	putrescine aminotransferase	53.88562689	0.647539974	0.556767943	1.163033868	0.244815762	0.967639542
2.7.1.107	diacylglycerol kinase (ATP)	109.2477564	-0.485890935	0.41778654	-1.163012422	0.244824463	0.967639542
3.2.1.187	beta-L-arabinobiosidase	5.256782339	-1.990602806	1.709592959	-1.164372371	0.244273139	0.967639542
1.2.1.76	malonyl-CoA/succinyl-CoA reductase (NADPH)	28.49464127	0.76141894	0.656831897	1.159229543	0.246362632	0.968102157
2.8.1.1	#N/A	9.932706052	-1.056672332	0.912104356	-1.158499381	0.246660304	0.968102157
2.4.1.182	lipid-A-disaccharide synthase	104.0503707	-0.670878373	0.579368592	-1.157947431	0.24688549	0.968102157
3.5.4.33	tRNA(adenine34) deaminase	428.6671125	-0.664324831	0.572736417	-1.159913725	0.246083934	0.968102157
1.4.3.5	pyridoxamine 5'-phosphate oxidase	196.6921172	-0.973434135	0.841548041	-1.156718438	0.247387414	0.968164498
5.3.3.14	3-hydroxyacyl-[acyl-carrier protein] dehydratase / trans-2-dece	2.895859573	2.005714269	1.736533266	1.155010565	0.2480861	0.96899512
3.4.11.23	PepB aminopeptidase	3.030118032	-3.282372845	2.853195217	-1.150420001	0.249970926	0.972592008
2.7.1.11	6-phosphofructokinase 1	1517.868815	0.350139162	0.304365752	1.150389488	0.249983488	0.972592008
3.6.3.7	#N/A	11.08064655	-1.085431249	0.956623814	-1.13464795	0.256522854	0.992155209
6.1.1.17	glutamyl-tRNA synthetase	495.3645692	0.295231254	0.260732498	1.132314753	0.25750213	0.992155209
1.1.1.47	glucose 1-dehydrogenase	18.79187857	1.305022643	1.151644647	1.133181704	0.257137956	0.992155209
2.4.2.28	5'-methylthioadenosine phosphorylase	9.012879735	-0.956337738	0.842668774	-1.134891629	0.256420728	0.992155209
1.1.1.290	erythronate-4-phosphate dehydrogenase	61.10219299	-0.650622181	0.572252006	-1.13695046	0.255558996	0.992155209
2.7.7.56	ribonuclease PH	38.61928976	-0.715523114	0.633304167	-1.129825368	0.258549817	0.992565709
6.2.1.14	6-carboxyhexanoate--CoA ligase	3.108126578	-2.16460592	1.916099478	-1.129693914	0.258605222	0.992565709
3.1.3.25	myo-inositol-1(or 4)-monophosphatase	93.69475451	-0.462844177	0.410539407	-1.127404994	0.259571289	0.994357706

2.1.1.22	carnosine N-methyltransferase	0.929084248	0.980312744	4.102595223	0.238949419	0.811144805	0.996944933
5.3.1.29	ribose 1,5-bisphosphate isomerase	1.243333821	0.411853442	3.891425581	0.105836135	0.915712366	0.996944933
4.4.1.25	L-cysteate sulfo-lyase	2.615552796	-0.108328545	2.126527037	-0.050941532	0.959372111	0.996944933
3.2.1.99	arabinan endo-1,5-alpha-L-arabinosidase	139.1340885	-0.346073525	0.565754621	-0.611702516	0.540734597	0.996944933
2.3.1.7	carnitine O-acetyltransferase	0.017504292	0.778777863	4.141980666	0.188020642	0.85086047	0.996944933
1.14.11.-	#N/A	0.589631369	-0.971414717	4.120194796	-0.235769124	0.813611828	0.996944933
3.2.1.22	alpha-galactosidase	682.0919527	-0.340083395	0.338234685	-1.00546576	0.314672629	0.996944933
3.2.1.132	chitosanase	0.184986723	-0.485884797	4.127511293	-0.117718587	0.906290639	0.996944933
3.1.3.18	phosphoglycolate phosphatase	780.8428987	0.147274005	0.460423473	0.319866413	0.7490696	0.996944933
1.3.8.6	glutaryl-CoA dehydrogenase	0.112273496	0.244702483	4.138007264	0.059135344	0.952844308	0.996944933
1.1.1.44	6-phosphogluconate dehydrogenase	386.0194263	0.319842192	0.652107503	0.490474639	0.623798072	0.996944933
2.5.1.47	cysteine synthase	480.1082915	-0.22220569	0.379347169	-0.585758134	0.558038061	0.996944933
6.3.2.7	UDP-N-acetylmuramoyl-L-alanyl-D-glutamate-L-lysine ligase	4.22944368	0.652552689	1.747972323	0.373319806	0.708910437	0.996944933
3.5.2.10	creatinine amidohydrolase	77.38836357	0.035816252	0.517896734	0.069157131	0.944864546	0.996944933
1.2.3.3	pyruvate oxidase	5.87582389	0.187372501	1.834869049	0.102117642	0.918663298	0.996944933
1.1.1.6	glycerol dehydrogenase	131.5645382	-0.231233034	0.483615338	-0.478134202	0.63255469	0.996944933
2.7.7.70	D-beta-D-heptose 7-phosphate kinase / D-beta-D-heptose 1-ph	30.61416296	0.395005903	0.693359096	0.569698884	0.568881947	0.996944933
2.1.1.-	#N/A	1033.626585	0.040758906	0.235908818	0.172773983	0.862829088	0.996944933
1.5.1.43	carboxynorspermidine synthase	0.131959613	0.721404871	4.142513233	0.174146667	0.861750199	0.996944933
2.3.3.13	2-isopropylmalate synthase	379.9594429	-0.036532345	0.161154277	-0.226691751	0.820663442	0.996944933
2.4.2.3	uridine phosphorylase	240.7680276	0.206078393	0.26730047	0.770961583	0.440729703	0.996944933
3.4.19.5	beta-aspartyl-peptidase (threonine type)	19.4807214	-0.287397636	0.553465357	-0.519269421	0.603572874	0.996944933
2.4.1.20	cellobiose phosphorylase	219.5275666	0.362145452	0.361993345	1.000420195	0.317107201	0.996944933
3.1.11.6	#N/A	611.926668	0.130513316	0.181049236	0.720871952	0.470988301	0.996944933
4.1.1.23	orotidine-5'-phosphate decarboxylase	361.1107121	-0.05023551	0.222802151	-0.225471389	0.821612581	0.996944933
1.1.1.270	3-keto steroid reductase	0.499547738	-0.30383844	4.118602018	-0.073772226	0.941191627	0.996944933
2.7.4.2	phosphomevalonate kinase	4.874322445	0.762913504	1.429958421	0.53352146	0.59367266	0.996944933
3.5.1.54	allophanate hydrolase	0.548905896	-0.983971642	4.109149793	-0.239458694	0.810749922	0.996944933
2.4.1.44	UDP-D-galactose:(glucosyl)LPS alpha-1,3-D-galactosyltransferas	0.907193448	-1.654481819	4.113327055	-0.402224719	0.687518652	0.996944933
2.5.1.26	alkyldihydroxyacetonephosphate synthase	19.14698164	-0.652249948	0.801511114	-0.813775301	0.415773687	0.996944933
2.7.7.24	glucose-1-phosphate thymidylyltransferase	376.3120803	0.136170073	0.23892419	0.56993004	0.568725149	0.996944933
2.4.2.19	nicotinate-nucleotide pyrophosphorylase (carboxylating)	246.5480813	0.189757002	0.311491217	0.609188931	0.542399217	0.996944933
2.1.1.204	tRNA (cytosine38-C5)-methyltransferase	0.144519491	0.262611323	4.137682566	0.063468214	0.94939367	0.996944933
1.2.1.43	#N/A	5.915036706	-0.163888518	1.159296752	-0.14136891	0.887578514	0.996944933
2.7.1.66	undecaprenol kinase	2.161074354	-0.627898077	1.87155787	-0.335494877	0.737251809	0.996944933
4.3.1.19	threonine dehydratase	227.9571154	0.046247221	0.29793861	0.155223993	0.876644734	0.996944933
3.7.1.12	cobalt-precorrin 5A hydrolase	99.95703726	0.021957856	0.438414441	0.0500847	0.960054892	0.996944933
1.1.1.62	17beta-estradiol 17-dehydrogenase	0.771316995	1.274787368	4.10502725	0.310542973	0.756148086	0.996944933
1.3.3.4	#N/A	12.23533618	-0.06875954	1.168709937	-0.058833709	0.953084559	0.996944933
2.1.1.200	tRNA (cytidine32/uridine32-2'-O)-methyltransferase	3.219890731	-1.611695832	2.95701733	-0.545041051	0.585725285	0.996944933
5.3.1.6	#N/A	479.29167	0.190153832	0.223645617	0.850246182	0.395188231	0.996944933
1.1.1.335	UDP-N-acetyl-2-amino-2-deoxyglucuronate dehydrogenase	3.835544045	0.253286528	1.839549446	0.137689437	0.890485868	0.996944933
1.1.1.350	ureidoglycolate dehydrogenase (NAD+)	2.906536393	-1.155140189	1.920467612	-0.601489024	0.54751432	0.996944933
2.3.1.181	lipoyl(octanoyl) transferase	98.92787013	0.011916724	0.702318948	0.016967681	0.986462399	0.996944933
1.2.1.18	Oxidoreductases	18.35639239	0.371870783	0.583881065	0.636894747	0.524193404	0.996944933
2.1.1.104	caffeoyl-CoA O-methyltransferase	0.263418337	1.989278778	4.13089506	0.481561199	0.630117695	0.996944933
1.2.99.5	#N/A	30.58832785	0.035406531	0.847449492	0.041780108	0.966673993	0.996944933
2.7.1.191	PTS system, mannose-specific IIA component	117.6263563	-0.041167633	0.455004433	-0.090477433	0.927907826	0.996944933
2.5.1.63	adenosyl-fluoride synthase	4.321299614	0.629106342	1.781938992	0.353045949	0.724053994	0.996944933
3.6.4.12	DNA replication licensing factor MCM5	12313.76222	0.034146249	0.62393314	0.054727417	0.956355627	0.996944933
2.7.8.11	CDP-diacylglycerol--inositol 3-phosphatidyltransferase	0.582805694	-0.313605345	4.114963576	-0.076210965	0.939251259	0.996944933
1.12.98.1	#N/A	28.04594534	-0.372518065	1.224363872	-0.304254375	0.760934102	0.996944933
3.2.1.106	mannosyl-oligosaccharide glucosidase	0.284782452	-0.220840253	4.133436238	-0.053427763	0.957391085	0.996944933
5.4.99.26	tRNA pseudouridine65 synthase	1.975607773	-0.020452196	2.097472593	-0.009750876	0.992220049	0.996944933
3.1.21.1	deoxyribonuclease I	1.14797295	-1.5989781	4.104692913	-0.389548776	0.696870236	0.996944933
2.7.7.83	UDP-N-acetylglucosamine/UDP-N-acetylgalactosamine diphosp	41.03166629	-0.631864867	0.862731441	-0.732400418	0.463924204	0.996944933
1.8.3.2	thiol oxidase	3.056885843	0.839318323	3.720390621	0.225599516	0.821512917	0.996944933
1.1.1.310	(S)-sulfolactate dehydrogenase	0.458766862	-0.608160212	4.13040249	-0.147239939	0.882942637	0.996944933
3.1.4.53	3',5'-cyclic-AMP phosphodiesterase	0.527495585	-0.860008475	4.11047154	-0.2092238	0.834273534	0.996944933
3.2.1.26	beta-fructofuranosidase	668.236276	0.334027367	0.432598959	0.772140942	0.440030956	0.996944933
4.2.1.3	#N/A	1254.902064	-0.262243977	0.567130487	-0.462405008	0.643790909	0.996944933
5.1.3.23	UDP-GlcNAc3NAcA epimerase	1.804103656	-0.253800331	2.087944981	-0.121555086	0.903251386	0.996944933
4.2.3.4	3-dehydroquinate synthase	263.3059402	-0.328026047	0.310839971	-1.055289146	0.291293104	0.996944933
2.4.2.30	poly [ADP-ribose] polymerase 16	1.661425333	-0.46057857	2.32390696	-0.198191485	0.84289525	0.996944933
6.3.2.32	gamma-F420-2:alpha-L-glutamate ligase	0.812016276	0.321505519	4.104823787	0.07832383	0.937570462	0.996944933
1.3.98.3	oxygen-independent coproporphyrinogen III oxidase	570.8711187	-0.0674955	0.26170708	-0.257904754	0.796480416	0.996944933
2.4.1.256	alpha-1,2-glucosyltransferase	0.333031825	-0.271430703	4.130787787	-0.065709186	0.947609359	0.996944933
1.1.1.9	D-xylulose reductase	0.801105635	0.131449473	4.1038948	0.03203042	0.974447792	0.996944933
3.5.3.9	allantoate deiminase	14.3761206	-0.054756564	0.659981856	-0.082966772	0.933877961	0.996944933
4.2.1.76	UDP-glucose 4,6-dehydratase	1.498656123	0.519192655	4.099365492	0.126651955	0.899215874	0.996944933
3.1.1.56	carboxylesterase 2	0.167196626	-3.226634141	4.143731151	-0.778678448	0.436169155	0.996944933
4.1.3.-	#N/A	225.5498462	-0.09008841	0.252354379	-0.356991666	0.721098044	0.996944933
3.4.23.36	signal peptidase II	249.2065007	-0.058280244	0.24614131	-0.236775551	0.81283092	0.996944933
4.1.1.86	L-2,4-diaminobutyrate decarboxylase	0.110533983	0.351083501	4.142162324	0.084758509	0.93245338	0.996944933
2.4.99.-	#N/A	0.084668419	1.060899532	4.141794518	0.256144898	0.797838955	0.996944933
5.5.1.4	myo-inositol-1-phosphate synthase	93.74847179	0.014545509	0.373347887	0.038959665	0.968922547	0.996944933
4.1.1.96	carboxynorspermidine decarboxylase	158.8615104	-0.486996262	0.443423793	-1.098263713	0.272089352	0.996944933
2.5.1.32	15-cis-phytoene synthase	4.713411545	-1.198302019	1.747789136	-0.685610177	0.492958962	0.996944933
5.3.1.-	#N/A	0.594915199	-1.485661061	4.097872418	-0.362544489	0.716945184	0.996944933
3.4.17.-	#N/A	57.62759611	0.365517848	0.547380722	0.667757985	0.50428809	0.996944933
3.4.24.37	saccharolysin	0.04233421	1.096522945	4.143245313	0.264653155	0.791276668	0.996944933
6.2.1.-	#N/A	80.6595248	-0.287993551	0.431377006	-0.667614517	0.504379689	0.996944933
6.2.1.4	#N/A	20.93996699	1.017679251	1.47499851	0.689952732	0.490223913	0.996944933
1.17.3.2	xanthine dehydrogenase/oxidase	0.020495196	0.766777793	4.142686886	0.185091902	0.853156953	0.996944933
1.17.7.1	(E)-4-hydroxy-3-methylbut-2-enyl-diphosphate synthase	363.237913	-0.177782312	0.168058057	-1.057862475	0.290118145	0.996944933
4.1.3.41	3-hydroxy-D-aspartate aldolase	0.311717752	0.366218427	4.120398967	0.088879361	0.929177786	0.996944933
1.3.1.28	2,3-dihydro-2,3-dihydroxybenzoate dehydrogenase	0.630791762	-0.711595525	4.123008533	-0.17259133	0.862972668	0.996944933

3.2.1.18	sialidase-1	722.2496707	-0.418799601	0.63453622	-0.660008977	0.509248069	0.996944933
6.3.5.5	carbamoyl-phosphate synthase	1356.851513	-0.039223806	0.178020646	-0.220332905	0.825611895	0.996944933
2.1.1.222	2-polyprenyl-6-hydroxyphenyl methylase / 3-demethylubiquinc	0.340645926	0.699344457	4.12584154	0.16950347	0.865400643	0.996944933
3.1.22.-	#N/A	0.333031825	-0.271430703	4.130787787	-0.065709186	0.947609359	0.996944933
2.7.1.20	adenosine kinase	2.502983657	-0.464944563	1.775167654	-0.26191586	0.793386312	0.996944933
1.12.7.2	ferredoxin hydrogenase	22.43350305	-0.189948448	0.532516816	-0.356699437	0.721316827	0.996944933
2.8.3.22	succinyl-CoA:(S)-malate CoA-transferase subunit A	0.128757426	0.100390562	4.134677189	0.024280145	0.98062915	0.996944933
1.2.1.-	#N/A	0.816141609	-2.511128336	3.863086005	-0.650031693	0.51567175	0.996944933
3.4.14.5	dipeptidyl-peptidase 4	252.4844495	-0.348168124	0.521795817	-0.667249741	0.504612624	0.996944933
2.7.7.64	UDP-sugar pyrophosphorylase	2.795318261	0.980002062	3.687008157	0.265798723	0.790394226	0.996944933
2.1.1.77	protein-L-isoaspartate(D-aspartate) O-methyltransferase	1.597836612	-2.215185045	2.689720677	-0.823574382	0.410181437	0.996944933
1.2.1.2	#N/A	245.2872466	0.333170557	0.521667446	0.63866465	0.523041114	0.996944933
1.1.1.67	mannitol 2-dehydrogenase	4.516882265	-0.484952696	1.746555922	-0.277662278	0.78127162	0.996944933
3.2.1.185	non-reducing end beta-L-arabinofuranosidase	0.714364175	-0.925863145	4.115848591	-0.22495073	0.822017604	0.996944933
2.7.8.7	holo-[acyl-carrier protein] synthase	35.60714793	-0.403237436	0.586453303	-0.687586607	0.491713145	0.996944933
5.99.1.3	#N/A	3660.059785	-0.412187623	0.536541003	-0.768231357	0.442349747	0.996944933
2.1.1.14	5-methyltetrahydropteroyltriglutamate--homocysteine methylt	94.21501856	-0.326297529	0.536206545	-0.608529552	0.542836312	0.996944933
2.4.1.131	#N/A	1.764497646	1.097573856	3.530616723	0.310873126	0.755897076	0.996944933
2.1.3.3	ornithine carbamoyltransferase	321.7840829	-0.069103958	0.233405633	-0.296068082	0.767178089	0.996944933
2.1.1.246	[methyl-Co(III) methanol-specific corrinoid protein]:coenzyme M	5.57705486	0.341624887	1.188823755	0.287363779	0.773833792	0.996944933
2.6.1.81	succinylornithine aminotransferase	0.203337428	-0.039944826	4.132401932	-0.009666249	0.992287569	0.996944933
2.7.7.12	UDPglucose--hexose-1-phosphate uridylyltransferase	348.5210504	-0.122528348	0.290995722	-0.421065805	0.673707031	0.996944933
5.2.1.13	prolycopene isomerase	1.503143303	-0.538799606	3.134078292	-0.171916447	0.863503216	0.996944933
6.3.4.15	biotin---protein ligase	359.9504088	-0.114814466	0.228255194	-0.503009214	0.614957794	0.996944933
1.8.4.8	phosphoadenosine phosphosulfate reductase	38.80354416	0.363311676	1.088555616	0.333755731	0.738563891	0.996944933
2.3.1.20	diacylglycerol O-acyltransferase / wax synthase	1.228064538	0.467007348	4.100876508	0.113879886	0.909333011	0.996944933
2.6.1.85	para-aminobenzoate synthetase component II	61.58760241	-0.666340958	0.625632351	-1.065067939	0.286845186	0.996944933
4.1.99.1	tryptophanase	227.0207991	0.270067057	0.456480626	0.591628737	0.554099226	0.996944933
4.4.1.16	selenocysteine lyase	255.398513	0.329664986	0.339011826	0.972429161	0.330837091	0.996944933
2.4.2.6	nucleoside deoxyribosyltransferase	0.102241287	0.681417775	4.141140062	0.164548353	0.869299486	0.996944933
5.3.3.-	#N/A	0.157074332	2.252406163	4.133738514	0.544883561	0.585833603	0.996944933
3.4.22.47	gingipain K	1.00454257	1.106960842	4.143731151	0.267141086	0.789360534	0.996944933
3.1.27.6	ribonuclease I (enterobacter ribonuclease)	0.207448764	-3.379298924	4.143731151	-0.815520795	0.414774266	0.996944933
4.3.3.7	4-hydroxy-tetrahydrodipicolinate synthase	488.6728682	0.110315471	0.216584539	0.509341393	0.610512949	0.996944933
5.1.3.11	cellobiose epimerase	95.78211526	-0.286172428	0.604970314	-0.473035488	0.636187861	0.996944933
1.6.3.3	NADH oxidase (H2O2-forming)	0.663509303	-1.430519408	4.117631862	-0.347413138	0.728280964	0.996944933
2.6.1.19	4-aminobutyrate aminotransferase	27.32058772	0.621544401	0.57177605	1.087041685	0.277018388	0.996944933
2.1.2.11	3-methyl-2-oxobutanoate hydroxymethyltransferase	194.9990628	-0.087526325	0.331104932	-0.264346182	0.791513178	0.996944933
5.4.3.5	D-ornithine 4,5-aminomutase subunit beta	35.04208565	0.7320163	0.930965832	0.786297708	0.431693095	0.996944933
2.6.1.21	D-alanine transaminase	38.44577382	0.203993966	0.446318559	0.457059116	0.647628556	0.996944933
2.7.8.6	undecaprenyl-phosphate galactose phosphotransferase	5.98517338	0.300000243	0.944648554	0.317578682	0.750804547	0.996944933
1.1.1.130	3-dehydro-L-gulonate 2-dehydrogenase	10.5048034	0.12146162	0.725800681	0.167348452	0.867095883	0.996944933
2.7.4.26	isopentenyl phosphate kinase	0.374667258	1.133678139	4.117687681	0.275319118	0.783071078	0.996944933
1.3.5.3	menaquinone-dependent protoporphyrinogen oxidase	14.33543221	-0.411705167	0.866707624	-0.475021975	0.634771311	0.996944933
1.1.1.90	aryl-alcohol dehydrogenase	0.095768651	0.034807213	4.136129673	0.008415407	0.993285556	0.996944933
3.4.11.21	aspartyl aminopeptidase	189.1715901	-0.087495586	0.327137856	-0.267457844	0.789116667	0.996944933
3.4.21.-	#N/A	392.414596	0.033157984	0.268667853	0.12341627	0.901777474	0.996944933
2.3.1.222	phosphate propanoyltransferase	0.103005941	0.222138118	4.137063428	0.053694637	0.957178456	0.996944933
2.4.1.144	beta-1,4-mannosyl-glycoprotein beta-1,4-N-acetylglucosaminyl	0.830970326	-2.640229446	4.095811001	-0.644617011	0.519175408	0.996944933
1.1.1.83	tartrate dehydrogenase/decarboxylase / D-malate dehydrogen	5.410002282	1.072966527	0.965341534	1.111489031	0.266357909	0.996944933
2.1.1.173	23S rRNA (guanine2445-N2)-methyltransferase / 23S rRNA (gua	1.313961173	-2.335315099	2.501144283	-0.933698673	0.35045935	0.996944933
1.1.1.287	D-arabinitol dehydrogenase (NADP+)	0.799170637	-0.196214354	4.106856561	-0.04777726	0.96189376	0.996944933
6.4.1.3	acetyl-CoA/propionyl-CoA carboxylase	273.9201141	-0.015470656	0.459111993	-0.033696912	0.973118842	0.996944933
1.7.2.2	nitrite reductase (cytochrome c-552)	49.12275699	0.055484282	0.451141935	0.12298631	0.902117939	0.996944933
5.4.4.2	salicylate biosynthesis isochorismate synthase	61.24446731	-0.294539346	0.443730707	-0.663779498	0.506831449	0.996944933
2.7.7.50	mRNA guanylyltransferase	1.392230011	0.728306511	4.09962537	0.177651967	0.85899631	0.996944933
3.1.4.14	acyl carrier protein phosphodiesterase	0.051862191	1.106985684	4.143731151	0.267147082	0.789355919	0.996944933
2.3.1.8	phosphate acetyltransferase	571.8730186	0.252550762	0.397698895	0.635030081	0.525408797	0.996944933
3.1.13.3	#N/A	386.1764493	0.200177129	0.238728179	0.838514877	0.401741596	0.996944933
2.1.1.182	16S rRNA (adenine1518-N6/adenine1519-N6)-dimethyltransfer	820.9449571	-0.11926724	0.642903395	-0.18551347	0.852826316	0.996944933
2.7.3.9	#N/A	954.3616646	0.362909333	0.448883267	0.808471509	0.418819201	0.996944933
3.1.13.1	exoribonuclease II	7.538701321	-0.87554433	1.166747251	-0.750414736	0.453004959	0.996944933
1.3.3.11	pyrroloquinoline-quinone synthase	0.110533983	0.351083501	4.142162324	0.084758509	0.93245338	0.996944933
3.6.3.10	H+/K+-exchanging ATPase	3.674943414	1.854074776	3.851979829	0.481330344	0.630281734	0.996944933
3.5.1.87	beta-ureidopropionase / N-carbamoyl-L-amino-acid hydrolase	41.46682057	0.184709821	0.488701703	0.377960257	0.705460118	0.996944933
3.4.21.62	subtilisin	0.102172711	0.801041013	4.139877907	0.193493874	0.846572204	0.996944933
6.4.1.4	#N/A	2.745395653	0.811118021	3.730361644	0.217436833	0.827867929	0.996944933
3.5.1.110	ureidoacrylate peracid hydrolase	0.074204671	1.828321644	4.143731151	0.441225933	0.659049441	0.996944933
2.3.1.42	glyceronephosphate O-acyltransferase	0.906836526	0.850759278	4.102716975	0.207364847	0.835724934	0.996944933
1.8.4.12	peptide-methionine (R)-S-oxide reductase	160.8333044	0.16583881	0.437625843	0.378951135	0.704724151	0.996944933
3.5.2.17	5-hydroxyisourate hydrolase	0.196024097	-0.131968967	4.130704016	-0.031948299	0.974513281	0.996944933
5.1.3.7	UDP-N-acetylglucosamine 4-epimerase	42.11554931	-0.569999742	0.672555003	-0.847513942	0.396708719	0.996944933
3.4.24.28	bacillolysin	0.938853782	-1.025452846	4.106267026	-0.249728729	0.802797139	0.996944933
2.7.11.11	protein kinase A	4.556984542	0.676649224	2.816972991	0.240204371	0.810171826	0.996944933
5.1.3.26	GlcNAc-P-P-Und epimerase	55.79927746	-0.47189436	0.697257792	-0.676786068	0.498541699	0.996944933
4.1.3.43	4-hydroxy-2-oxovalerate/4-hydroxy-2-oxohexanoate aldolase	0.165800975	0.061971976	4.136604711	0.014981363	0.988047049	0.996944933
4.2.1.93	ATP-dependent NAD(P)H-hydrate dehydratase	0.404452483	0.210253567	4.118778732	0.051047551	0.95928763	0.996944933
4.2.3.153	(5-formylfuran-3-yl)methyl phosphate synthase	1.166323075	0.327095575	4.101515437	0.079749932	0.936436146	0.996944933
2.7.1.82	ethanolamine kinase	1.883458461	0.623144072	4.097795136	0.152068137	0.879133195	0.996944933
2.4.1.5	dextransucrase	0.839572008	-2.213696742	4.101501618	-0.53972836	0.589384379	0.996944933
2.3.1.75	diacylglycerol O-acyltransferase / wax synthase	1.228064538	0.467007348	4.100876508	0.113879886	0.909333011	0.996944933
3.2.1.123	endoglycosylceramidase	0.937054039	-0.855448952	3.751972386	-0.227999799	0.819646396	0.996944933
2.7.1.25	adenylylsulfate kinase	65.7994572	-0.218657702	0.557450424	-0.392246005	0.694876455	0.996944933
2.9.1.-	#N/A	0.512348345	-0.669531569	4.110118884	-0.162898346	0.870598473	0.996944933
4.2.1.28	propanediol dehydratase large subunit	272.216343	0.302473518	0.924035595	0.327339683	0.743410982	0.996944933
5.3.3.18	2-(1,2-epoxy-1,2-dihydrophenyl)acetyl-CoA isomerase	12.98823612	-0.10331412	0.715969664	-0.144299578	0.885263911	0.996944933
3.1.2.1	acetyl-CoA hydrolase	33.55176493	0.22233548	2.134683642	0.104153831	0.917047272	0.996944933

2.1.1.213	tRNA (guanine10-N2)-dimethyltransferase	0.249773869	-0.154751873	4.134519116	-0.037429232	0.970142766	0.996944933
1.1.1.178	3-hydroxyacyl-CoA dehydrogenase / 3-hydroxy-2-methylbutyry	0.416289782	-0.290558718	4.123574593	-0.070462826	0.943825288	0.996944933
2.7.1.56	1-phosphofructokinase	313.4501232	0.389031314	0.380355646	1.022809357	0.306397993	0.996944933
1.1.1.159	7-alpha-hydroxysteroid dehydrogenase	14.93492507	0.087641159	1.061065432	0.082597318	0.934171734	0.996944933
2.7.1.211	PTS system, sucrose-specific IIA component	94.53090177	0.399194705	0.402610357	0.991516234	0.32143357	0.996944933
2.4.1.41	polypeptide N-acetylgalactosaminyltransferase	3.059020224	0.659491489	2.968611195	0.222154889	0.82419331	0.996944933
4.2.1.130	D-lactate dehydratase / protein deglycase	1.123723061	0.521934955	3.244491459	0.160868032	0.872197341	0.996944933
2.1.1.225	tRNA:m4X modification enzyme	1.426288518	0.865482372	4.099423544	0.211122945	0.832791336	0.996944933
3.4.13.22	zinc D-Ala-D-Ala dipeptidase	69.29723343	-0.255668934	0.564582622	-0.452845915	0.650659698	0.996944933
1.3.1.101	digeranylgeranylglycerophospholipid reductase	2.870100187	0.901968969	3.909691541	0.230700801	0.817547251	0.996944933
2.7.1.144	tagatose 6-phosphate kinase	19.2680341	-0.191127429	0.675085498	-0.283115886	0.777088004	0.996944933
1.5.1.24	N5-(carboxyethyl)ornithine synthase	0.365589093	-1.075436114	4.115987246	-0.261282664	0.793874534	0.996944933
2.1.1.289	cobalt-precorrin-7 (C5)-methyltransferase	1.380155497	0.783163652	4.100583291	0.190988354	0.848534718	0.996944933
3.1.1.97	diphthine methyl ester acylhydrolase	0.368040408	-0.28077643	4.127255755	-0.068029811	0.945761903	0.996944933
4.2.1.134	very-long-chain (3R)-3-hydroxyacyl-CoA dehydratase	0.052512875	0.445935252	4.139416356	0.107729016	0.914210652	0.996944933
1.10.3.9	photosystem II P680 reaction center D1 protein	0.896335188	-0.797561558	4.102141401	-0.194425662	0.845842598	0.996944933
2.5.1.3	thiamine-phosphate pyrophosphorylase	546.7049117	0.558154451	0.538105084	1.037259204	0.299615073	0.996944933
1.16.1.3	aquacobalamin reductase / NAD(P)H-flavin reductase	1.401765292	-0.840238213	3.966604917	-0.211828057	0.832241179	0.996944933
2.1.1.195	cobalt-precorrin-5B (C1)-methyltransferase	157.4125858	-0.208116624	0.319583921	-0.651211186	0.514910171	0.996944933
2.7.2.3	phosphoglycerate kinase	1220.852491	0.160381471	0.471941438	0.339833417	0.733981981	0.996944933
5.3.3.2	isopentenyl-diphosphate Delta-isomerase	23.14189555	-0.205334676	0.746202555	-0.27517284	0.783183452	0.996944933
2.7.1.150	1-phosphatidylinositol-3-phosphate 5-kinase	0.457193986	0.083880702	4.116687298	0.020375777	0.983743607	0.996944933
3.5.1.5	urease	390.454859	0.21297793	0.65034053	0.327486785	0.743299736	0.996944933
4.2.1.99	aconitate hydratase 2 / 2-methylisocitrate dehydratase	5.473792319	0.097444448	2.001154532	0.048694114	0.961163066	0.996944933
2.3.1.241	Kdo2-lipid IVA lauroyltransferase	89.05169472	-0.320836926	0.442722124	-0.724691424	0.468641358	0.996944933
1.2.1.19	aminobutyraldehyde dehydrogenase	0.098065194	1.081349995	4.142590869	0.261032293	0.794067604	0.996944933
1.1.1.169	2-dehydropantoate 2-reductase	287.7816628	0.039917694	0.309454709	0.128993656	0.897362669	0.996944933
3.5.2.2	dihydropyrimidinase	110.1995053	0.036217256	0.318892778	0.113571892	0.90957717	0.996944933
5.4.99.45	tRNA pseudouridine38/39 synthase	0.201524496	-0.057771136	4.136321218	-0.013966791	0.988856475	0.996944933
5.3.1.25	L-fucose/D-arabinose isomerase	314.2306221	0.022587146	0.399188916	0.056582597	0.954877698	0.996944933
2.3.1.129	UDP-N-acetylglucosamine acyltransferase	195.8513799	0.217270825	0.315977532	0.687614792	0.491695391	0.996944933
2.5.1.58	protein farnesyltransferase subunit beta	0.766825899	-0.328133271	4.109580535	-0.079845928	0.936359796	0.996944933
4.2.1.104	cyanate lyase	0.603088763	1.810263302	4.118778232	0.439514633	0.660288682	0.996944933
3.5.99.7	1-aminocyclopropane-1-carboxylate deaminase	1.103206331	-0.797949699	2.406590389	-0.331568556	0.740215067	0.996944933
2.4.1.198	#N/A	0.270591585	0.772288216	4.126433014	0.187156368	0.851538034	0.996944933
1.2.1.22	lactaldehyde dehydrogenase / glycolaldehyde dehydrogenase	20.55117114	-0.474670902	0.57564543	-0.824589022	0.409604956	0.996944933
3.6.1.6	ectonucleoside triphosphate diphosphohydrolase 5/6	3.61936385	0.745603616	2.860820083	0.260625833	0.794381066	0.996944933
1.1.1.40	malate dehydrogenase (oxaloacetate-decarboxylating)(NADP+)	103.4381533	-0.146667225	0.430340794	-0.340816458	0.733241759	0.996944933
2.7.8.17	UDP-N-acetylglucosamine-lysosomal-enzyme	2.981701137	0.379115374	3.701165373	0.102431352	0.918414299	0.996944933
3.2.1.51	alpha-L-fucosidase	1731.162235	-0.046879045	0.427076467	-0.109767334	0.912593898	0.996944933
5.4.99.9	UDP-galactopyranose mutase	302.3987479	0.13547601	0.198879593	0.681196133	0.495747394	0.996944933
4.1.99.17	phosphomethylpyrimidine synthase	285.8239162	-0.160199333	0.277205124	-0.577908991	0.563325565	0.996944933
5.1.3.9	N-acylglucosamine-6-phosphate 2-epimerase	224.6239802	-0.048536426	0.581471502	-0.08347172	0.933476463	0.996944933
1.3.1.10	enoyl-acyl-carrier protein] reductase I	101.9054622	-0.072001583	0.401797691	-0.1791986	0.857781763	0.996944933
2.7.6.5	GTP pyrophosphokinase	1176.233156	0.095271846	0.140410905	0.678521701	0.497440969	0.996944933
1.2.1.72	D-erythrose 4-phosphate dehydrogenase	1.604490321	-0.433068141	3.144102272	-0.137739839	0.890446032	0.996944933
5.1.3.25	dTDP-L-rhamnose 4-epimerase	7.819350156	-0.936409538	1.151104663	-0.813487746	0.415938469	0.996944933
4.2.1.149	crotonobetainyl-CoA hydratase	2.640428685	-0.830415552	1.850057936	-0.448859215	0.653533219	0.996944933
2.3.1.88	#N/A	6.11169659	0.967302051	2.451401165	0.394591495	0.693144391	0.996944933
5.5.1.1	muconate cycloisomerase	0.112351376	0.403045323	4.13830458	0.097393828	0.922413646	0.996944933
3.6.1.55	8-oxo-dGTP diphosphatase	263.2355375	-0.099336734	0.291848799	-0.340370541	0.733577501	0.996944933
5.4.2.9	phosphoenolpyruvate phosphomutase	19.55533939	-0.765365001	0.910037063	-0.841026186	0.400333264	0.996944933
6.3.1.5	NAD+ synthase	24.1008445	0.597111629	0.595534394	1.002648437	0.316030517	0.996944933
1.18.1.-	#N/A	0.842428939	1.631337517	4.098421762	0.398040419	0.690600392	0.996944933
2.3.1.-	#N/A	294.6393979	-0.20897043	0.327497116	-0.638083268	0.523419478	0.996944933
1.16.3.2	ferritin heavy chain	754.4409733	0.040610565	0.504506339	0.080495648	0.935843057	0.996944933
1.11.1.21	catalase-peroxidase	7.96020901	-0.046866161	2.037524826	-0.023001517	0.981649063	0.996944933
5.1.3.-	#N/A	101.2987499	0.084226489	0.876437554	0.096100959	0.92344039	0.996944933
4.2.1.46	dTDP-glucose 4,6-dehydratase	412.7164931	0.104505766	0.224866473	0.464745876	0.642113451	0.996944933
1.14.14.9	4-hydroxyphenylacetate 3-monooxygenase	2.25640993	-1.042452757	2.48852381	-0.418904072	0.675286241	0.996944933
3.1.3.6	2',3'-cyclic-nucleotide 2'-phosphodiesterase / 3'-nucleotidase	285.4621956	0.260852048	0.445578603	0.585423192	0.558263198	0.996944933
3.5.2.5	allantoinase	7.295496758	-0.777706144	1.007042208	-0.772267675	0.439955907	0.996944933
4.3.1.29	D-glucosaminate-6-phosphate ammonia-lyase	0.040990391	0.558766837	4.140771598	0.134942685	0.892657191	0.996944933
5.4.2.11	2,3-bisphosphoglycerate-dependent phosphoglycerate mutase	320.0771963	0.225321833	0.537054363	0.419551258	0.674813302	0.996944933
2.1.1.132	precorrin-6Y C5,15-methyltransferase (decarboxylating)	165.8060595	-0.233540408	0.249933984	-0.934408378	0.350093278	0.996944933
2.4.1.267	alpha-1,3-glucosyltransferase	0.129855667	0.32631698	4.137954808	0.078859484	0.93714439	0.996944933
1.12.1.3	NADP-reducing hydrogenase subunit HndB	567.3491264	0.045665085	0.515973875	0.088502707	0.929477133	0.996944933
3.4.24.13	IgA-specific metalloendopeptidase	8.078081151	-0.84193482	2.107340833	-0.39952475	0.689506591	0.996944933
1.11.1.6	#N/A	161.5587878	0.394991432	0.556215181	0.710141408	0.477616451	0.996944933
3.6.5.5	dynamin GTPase	7.357062005	0.485747787	2.001276612	0.242718964	0.808223115	0.996944933
2.7.2.8	acetylglutamate kinase	350.0145196	0.3253099	0.396810971	0.819810751	0.412324001	0.996944933
2.7.4.21	inositol-hexakisphosphate 5-kinase	0.249773869	-0.154751873	4.134519116	-0.037429232	0.970142766	0.996944933
4.1.1.82	phosphonopyruvate decarboxylase	15.5295624	-0.893708744	0.841048183	-1.062613013	0.287957483	0.996944933
6.2.1.20	long-chain-fatty-acid--[acyl-carrier-protein] ligase	6.062451873	-1.485840248	1.555996839	-0.954912125	0.339622138	0.996944933
2.6.1.101	L-glutamine:2-deoxy-scyllo-inosose/3-amino-2,3-dideoxy-scyllo	0.025751485	0.746325135	4.143731151	0.180109449	0.857066645	0.996944933
4.3.1.15	diaminopropionate ammonia-lyase	83.25829541	0.18030921	0.4397728	0.41000537	0.681802008	0.996944933
4.6.1.15	triose/dihydroxyacetone kinase / FAD-AMP lyase (cyclizing)	1.318253244	-2.300378781	2.810666682	-0.818445956	0.413102589	0.996944933
3.1.3.43	pyruvate dehydrogenase phosphatase	1.498669032	1.123482367	4.099217185	0.274072418	0.78402897	0.996944933
1.1.1.38	malate dehydrogenase (oxaloacetate-decarboxylating)	352.0587986	0.210151559	0.269164025	0.780756489	0.434945729	0.996944933
6.3.4.21	nicotinate phosphoribosyltransferase	719.7523161	0.13717962	0.581182937	0.236035182	0.81340537	0.996944933
1.5.98.1	methylenetetrahydromethanopterin dehydrogenase	7.559254633	1.171402325	1.95763936	0.598374935	0.549589786	0.996944933
4.2.1.17	enoyl-CoA hydratase	991.304438	0.562474043	0.597419139	0.941506567	0.346445326	0.996944933
4.4.1.21	S-ribosylhomocysteine lyase	157.4335133	-0.272122652	0.363673761	-0.748260341	0.454303141	0.996944933
3.6.1.62	mRNA-decapping enzyme subunit 2	0.250485555	2.149835927	4.130031866	0.52053737	0.602689088	0.996944933
2.1.1.72	site-specific DNA-methyltransferase (adenine-specific)	1002.074774	-0.281322532	0.262279564	-1.07260561	0.283448106	0.996944933
5.4.1.4	D-galactarolactone isomerase	0.052512875	0.445935252	4.139416356	0.107729016	0.914210652	0.996944933
3.1.13.-	#N/A	15.34080406	0.034517238	1.70327844	0.020265176	0.983831835	0.996944933

5.3.1.3	L-fucose/D-arabinose isomerase	314.2306221	0.022587146	0.399188916	0.056582597	0.954877698	0.996944933
4.2.1.11	enolase	1347.16273	0.330393112	0.424291202	0.778694234	0.436159854	0.996944933
5.1.3.13	dTDP-4-dehydrorhamnose 3,5-epimerase	198.2590378	0.0360237	0.305383699	0.117962092	0.906097694	0.996944933
1.5.1.34	dihydropteridine reductase	16.32083196	-0.563949974	1.043125361	-0.540634899	0.588759258	0.996944933
1.8.4.11	peptide-methionine (S)-S-oxide reductase	173.9237147	0.152065138	0.414570484	0.36680165	0.713766977	0.996944933
3.4.24.56	insulysin	0.63958207	-0.319213142	4.112881882	-0.07761301	0.938135893	0.996944933
1.1.99.3	#N/A	2.344093746	-1.866538801	2.196066835	-0.849946264	0.395354963	0.996944933
6.3.4.11	biotin---protein ligase	1.456275519	-0.021285983	4.100745809	-0.005190759	0.995858392	0.996944933
3.2.1.196	glycogen debranching enzyme	169.2043627	-0.12789054	0.464317507	-0.275437688	0.782979993	0.996944933
3.1.21.5	type III restriction enzyme	190.996469	0.14906739	0.255572697	0.583268057	0.559712863	0.996944933
3.11.1.2	phosphonoacetate hydrolase	0.557746465	0.213877603	4.115925445	0.05196343	0.958557833	0.996944933
3.5.4.27	methenyltetrahydromethanopterin cyclohydrolase	1.31203418	0.812143515	4.099898641	0.198088681	0.842975681	0.996944933
2.4.1.321	cellobionic acid phosphorylase	0.183277685	0.531893383	4.136647432	0.128580787	0.89768937	0.996944933
1.1.1.93	tartrate dehydrogenase	5.410002282	1.072966527	0.965341534	1.111489031	0.266357909	0.996944933
2.7.7.75	molybdopterin adenylyltransferase	1.658651571	-0.982962107	2.473053442	-0.397469012	0.691021634	0.996944933
2.7.7.87	L-threonylcarbamoyladenylate synthase	305.4179164	0.076179035	0.157365892	0.484088605	0.628322988	0.996944933
3.6.3.21	#N/A	551.7754522	0.148140897	0.334657534	0.442664162	0.658008668	0.996944933
2.7.12.1	#N/A	7.925832161	0.80364381	4.094029938	0.196296515	0.844378089	0.996944933
6.1.1.19	arginyl-tRNA synthetase	514.4061134	-0.255903317	0.276041014	-0.927048168	0.353901526	0.996944933
1.2.4.2	2-oxoglutarate dehydrogenase E1 component	25.41993019	-0.296001563	0.790900789	-0.374258778	0.708211798	0.996944933
2.7.1.194	PTS system, ascorbate-specific IIA component	22.57368151	-0.587212045	0.808489489	-0.726307582	0.467650232	0.996944933
1.11.1.10	non-heme chloroperoxidase	2.224202965	-1.154465581	4.078115046	-0.283088036	0.777109352	0.996944933
3.4.21.102	carboxyl-terminal processing protease	540.2492765	0.155617762	0.21350167	0.728883113	0.466073165	0.996944933
2.7.1.6	galactokinase	445.280387	0.122156844	0.201307069	0.606818453	0.543971401	0.996944933
2.6.1.66	valine--pyruvate aminotransferase	70.05961202	-0.420212306	0.433600408	-0.969123409	0.332483619	0.996944933
6.3.2.30	cyanophycin synthetase	0.53521663	1.34159318	4.107130854	0.326649729	0.743932827	0.996944933
4.1.1.3	#N/A	2726.451397	0.384977296	0.582516902	0.660886052	0.508685391	0.996944933
1.8.1.8	thioredoxin 2	156.4033575	0.304772379	0.322466454	0.945128944	0.34459305	0.996944933
6.2.1.7	solute carrier family 27 (fatty acid transporter), member 5	0.48997058	2.255013193	4.121005594	0.547199741	0.584241514	0.996944933
3.4.21.96	lactocepin	235.7046641	-0.353153371	0.74022125	-0.477091641	0.633296867	0.996944933
4.1.1.49	phosphoenolpyruvate carboxykinase (ATP)	1207.42963	0.453973674	0.457896595	0.991432735	0.321474322	0.996944933
2.3.1.193	tRNA(Met) cytidine acetyltransferase	0.767393725	-0.961673492	4.109016237	-0.234039837	0.814954052	0.996944933
1.6.1.1	NAD(P) transhydrogenase	5.221596558	-0.718589739	2.065470904	-0.347906009	0.727910772	0.996944933
1.1.1.372	L-glyceraldehyde reductase	4.412671685	-0.369767644	4.094641164	-0.090305262	0.928044639	0.996944933
1.5.1.38	FMN reductase	13.56279913	-0.019106095	1.096878547	-0.017418606	0.986102666	0.996944933
1.1.1.137	ribulose-5-phosphate 2-dehydrogenase	24.26923239	-0.23311515	0.613732768	-0.379831683	0.704070362	0.996944933
2.1.1.34	tRNA (guanosine-2'-O-)-methyltransferase	7.652998207	-0.343465349	1.468256694	-0.233927317	0.815041406	0.996944933
3.2.2.26	futalosine hydrolase	1.673267527	-2.605624748	3.75282573	-0.694310084	0.487487769	0.996944933
3.4.23.49	omptin	0.074204671	1.828321644	4.143731151	0.441225933	0.659049441	0.996944933
2.7.11.23	CTD kinase subunit alpha	1.84467463	-0.21528234	4.099157003	-0.052518686	0.958115407	0.996944933
6.1.1.4	leucyl-tRNA synthetase	687.997849	0.15971486	0.18760552	0.851333478	0.394584133	0.996944933
4.1.2.21	2-dehydro-3-deoxyphosphogalactonate aldolase	0.046509078	0.746325135	4.143731151	0.180109449	0.857066645	0.996944933
3.1.1.53	sialate O-acetylesterase	282.7241622	-0.052643883	0.450908461	-0.11675071	0.907057604	0.996944933
2.3.1.189	mycothiol synthase	0.446767538	0.85677218	4.134023058	0.207249009	0.835815395	0.996944933
2.1.1.217	tRNA (adenine22-N1)-methyltransferase	250.9195669	-0.15536736	0.456856572	-0.340079074	0.73379698	0.996944933
2.1.1.242	16S rRNA (guanine1516-N2)-methyltransferase	12.35996513	-0.676405946	0.806292612	-0.838908773	0.401520504	0.996944933
4.1.3.1	isocitrate lyase	1.190703912	-0.608833471	3.54881379	-0.171559712	0.863783683	0.996944933
2.7.1.-	#N/A	1909.117588	0.366477154	0.504349515	0.726633302	0.467450622	0.996944933
2.2.1.10	fructose-bisphosphate aldolase / 2-amino-3,7-dideoxy-D-threo-	2.56819499	-0.396878526	2.012929451	-0.197164648	0.843698695	0.996944933
1.1.1.100	3-oxoacyl-[acyl-carrier protein] reductase	830.0443141	-0.189721813	0.336320977	-0.564109365	0.572679705	0.996944933
2.4.99.14	3-deoxy-D-manno-octulosonic-acid transferase	104.6062867	-0.408074653	0.481327791	-0.847810288	0.396543632	0.996944933
2.4.1.260	alpha-1,6-mannosyltransferase	1.51946093	-0.05833743	4.099931676	-0.014228879	0.988647381	0.996944933
6.3.5.4	asparagine synthase (glutamine-hydrolysing)	377.2433377	-0.221170271	0.227408997	-0.972566056	0.33076902	0.996944933
6.1.1.22	asparaginyl-tRNA synthetase	569.499554	0.194629444	0.350574348	0.555173091	0.578776273	0.996944933
2.7.1.33	type I pantothenate kinase	366.0985092	0.371544883	0.388802695	0.955612931	0.339267826	0.996944933
6.5.1.1	bifunctional non-homologous end joining protein LigD	24.55321329	-0.059640085	0.715412658	-0.083364593	0.933561641	0.996944933
2.4.1.10	levansucrase	0.401328364	-1.184132079	4.114505788	-0.287794486	0.773504058	0.996944933
3.2.2.8	ribosylpyrimidine nucleosidase	7.613796596	-0.466032282	1.041003907	-0.447675824	0.654387172	0.996944933
4.2.1.35	3-isopropylmalate/(R)-2-methylmalate dehydratase large subur	590.1564115	-0.077070162	0.157230958	-0.490171671	0.624012425	0.996944933
1.1.1.343	6-phosphogluconate dehydrogenase	386.0194263	0.319842192	0.652107503	0.490474639	0.623798072	0.996944933
2.7.1.59	N-acetylglucosamine kinase	0.905041437	-0.094069526	3.709347549	-0.025360127	0.979767715	0.996944933
2.5.1.114	tRNA wybutosine-synthesizing protein 2	0.249773869	-0.154751873	4.134519116	-0.037429232	0.970142766	0.996944933
2.4.1.336	1,2-diacylglycerol 3-beta-glucosyltransferase	0.52830923	0.104330823	4.107650673	0.025399147	0.979736591	0.996944933
3.5.1.49	formamidase	0.917025487	-1.276668766	2.81443722	-0.453614228	0.650106511	0.996944933
2.7.7.53	sulfate adenylyltransferase (ADP) / ATP adenylyltransferase	1.402946654	2.331139309	2.855590325	0.816342348	0.41430436	0.996944933
4.3.2.3	ureidoglycolate lyase	78.66999186	0.838695221	1.897913082	0.441903915	0.658558738	0.996944933
2.4.1.132	alpha-1,3/alpha-1,6-mannosyltransferase	1.814404281	1.028185899	4.097896762	0.250905759	0.801886971	0.996944933
1.1.1.304	meso-butanediol dehydrogenase / (S,S)-butanediol dehydroger	2.544822965	0.309823523	1.935104418	0.160106876	0.872796885	0.996944933
2.1.1.61	tRNA 5-methylaminomethyl-2-thiouridine biosynthesis bifuncti	3.684858882	0.727364855	1.600583007	0.454437447	0.649514005	0.996944933
3.6.1.63	alpha-D-ribose 1-methylphosphonate 5-triphosphate diphospha	2.990499697	-2.255596084	2.211888338	-1.019760376	0.30784212	0.996944933
2.7.11.2	pyruvate dehydrogenase kinase 2/3/4	3.406061021	1.000889903	3.544423455	0.282384404	0.777648773	0.996944933
3.1.11.-	#N/A	0.311872348	0.950820642	4.127430861	0.230366219	0.817807209	0.996944933
2.1.1.249	dimethylamine---corrinoid protein Co-methyltransferase	1.264396432	-0.044998189	3.044508516	-0.014780116	0.988207603	0.996944933
2.3.-.-	#N/A	2.137499889	-1.515464154	2.511577474	-0.603391363	0.546248367	0.996944933
2.5.1.18	glutathione S-transferase	5.747224439	0.54114984	1.748237597	0.309540214	0.756910627	0.996944933
1.3.1.22	steroid 5-alpha-reductase	119.5639343	-0.817807676	0.879095973	-0.930282586	0.352224791	0.996944933
4.2.1.8	mannonate dehydratase	319.1697185	-0.023557501	0.206913141	-0.113852128	0.909355015	0.996944933
4.1.1.94	ethylmalonyl-CoA/methylmalonyl-CoA decarboxylase	2.58784862	0.627454046	3.147030159	0.199379737	0.84196571	0.996944933
2.4.1.141	beta-1,4-N-acetylglucosaminyltransferase	0.388858125	0.510286714	4.117292203	0.123937454	0.901364797	0.996944933
3.5.3.26	(S)-ureidoglycine aminohydrolase	8.571204104	0.501619907	0.829342621	0.604840381	0.545285059	0.996944933
4.2.2.6	oligogalacturonide lyase	18.28317034	-0.077594157	0.67012771	-0.115790104	0.907818894	0.996944933
2.7.1.162	N-acetylhexosamine 1-kinase	7.442655289	0.725391269	1.759470899	0.412278072	0.680135619	0.996944933
1.1.1.3	homoserine dehydrogenase	435.1502177	-0.23249932	0.216650345	-1.073154627	0.283201745	0.996944933
1.13.11.-	#N/A	0.969176871	-0.52323628	4.099791101	-0.127625108	0.898445661	0.996944933
3.6.3.1	#N/A	7.365742814	-0.033127529	2.043673722	-0.016209793	0.987067022	0.996944933
2.6.1.-	#N/A	379.879431	-0.161811493	0.302786458	-0.534407958	0.593059314	0.996944933
3.2.1.141	maltooligosyltrehalose trehalohydrolase	0.465749231	0.290095906	4.108901613	0.070601814	0.943714667	0.996944933

1.2.7.8	indolepyruvate ferredoxin oxidoreductase, alpha subunit	571.351912	-0.021142024	0.316009738	-0.066903076	0.946658864	0.996944933
3.1.3.70	mannosyl-3-phosphoglycerate phosphatase	0.122002457	0.22210673	4.140415365	0.053643586	0.95721913	0.996944933
2.7.4.8	guanylate kinase	418.9878264	0.204242969	0.311655554	0.655348401	0.512243465	0.996944933
3.5.1.108	UDP-3-O-[3-hydroxymyristoyl] N-acetylglucosamine deacetylas	116.3137628	-0.05248061	0.297862559	-0.17619069	0.860144135	0.996944933
1.97.1.9	Tat-targeted selenate reductase subunit YnfE	165.715164	0.270215005	0.349653049	0.772808947	0.439635457	0.996944933
2.7.1.202	PTS system, fructose-specific IIA component	408.9532809	0.108161054	0.377113265	0.286813177	0.774255373	0.996944933
2.3.1.101	formylmethanofuran--tetrahydromethanopterin N-formyltrans	2.080730589	0.142079773	4.098024516	0.034670308	0.972342637	0.996944933
3.6.3.31	polyamine-transporting ATPase	242.7358801	-0.492172862	0.457316266	-1.076219892	0.281828926	0.996944933
6.3.4.9	biotin---protein ligase	1.456275519	-0.021285983	4.100745809	-0.005190759	0.995858392	0.996944933
1.1.5.2	quinoprotein glucose dehydrogenase	1.838329898	0.699748846	4.100281118	0.170658749	0.864492101	0.996944933
2.5.1.76	cysteate synthase	1.65237058	-1.66999433	2.843148769	-0.587374937	0.556951922	0.996944933
3.8.1.2	2-haloacid dehalogenase	90.88721424	-0.02324592	0.373986675	-0.062157082	0.95043774	0.996944933
2.1.1.186	23S rRNA (cytidine2498-2'-O)-methyltransferase	1.410587932	-2.118676086	2.351669758	-0.900924153	0.367628648	0.996944933
3.6.3.33	vitamin B12 transport system ATP-binding protein	0.081334971	0.404882397	4.142921482	0.097728716	0.922147713	0.996944933
1.1.1.140	sorbitol-6-phosphate 2-dehydrogenase	9.218496711	-0.783247088	1.136239349	-0.689332831	0.490613842	0.996944933
5.4.99.17	squalene-hopene/tetraprenyl-beta-curcumene cyclase	7.194674308	-0.813199679	1.46142114	-0.556444448	0.577907061	0.996944933
5.3.3.1	3beta-hydroxy-Delta5-steroid dehydrogenase / steroid Delta-is	0.017504292	0.778777863	4.141980666	0.188020642	0.85086047	0.996944933
2.8.1.8	lipoyl synthase	74.59543311	-0.523554176	0.50304907	-1.040761641	0.297986187	0.996944933
2.7.1.29	triose/dihydroxyacetone kinase / FAD-AMP lyase (cyclizing)	1.318253244	-2.300378781	2.810666682	-0.818445956	0.413102589	0.996944933
3.1.2.23	4-hydroxybenzoyl-CoA thioesterase	0.325679685	0.409469693	4.126454233	0.099230397	0.92095534	0.996944933
3.2.1.180	unsaturated chondroitin disaccharide hydrolase	100.6903551	-0.469340662	0.503823346	-0.931557987	0.351565004	0.996944933
3.4.21.66	thermitase	0.051502971	0.522475046	4.141439557	0.126157834	0.899606988	0.996944933
2.3.1.180	3-oxoacyl-[acyl-carrier-protein] synthase III	380.2113221	0.220143898	0.27318125	0.805852883	0.420327682	0.996944933
1.3.1.76	uroporphyrin-III C-methyltransferase / precorrin-2 dehydrogen	30.31722373	-0.707982086	0.853762266	-0.82924968	0.406963138	0.996944933
3.1.3.46	6-phosphofructo-2-kinase / fructose-2,6-biphosphatase 3	3.811408119	0.291617157	2.831195018	0.103001437	0.91796183	0.996944933
4.1.1.8	oxalyl-CoA decarboxylase	0.835501983	-0.245673898	3.777569086	-0.065034919	0.948146198	0.996944933
2.1.1.198	16S rRNA (cytidine1402-2'-O)-methyltransferase	604.2734402	0.127493976	0.396563904	0.321496673	0.747834033	0.996944933
3.5.3.3	creatinase	0.347232792	0.028298595	4.124594227	0.00686094	0.994525805	0.996944933
2.4.2.43	4-amino-4-deoxy-L-arabinose transferase	0.111464417	1.106978919	4.143731151	0.267145449	0.789357176	0.996944933
3.4.24.70	oligopeptidase A	23.81967512	-0.479086149	0.960642506	-0.498714293	0.617980674	0.996944933
3.6.4.-	#N/A	1316.288446	-0.09152004	0.197269907	-0.463933101	0.642695676	0.996944933
1.11.1.1	NADH peroxidase	1.37195059	-2.800293883	3.384047206	-0.827498469	0.407954589	0.996944933
1.18.1.1	rubredoxin---NAD+ reductase	51.1723042	-0.297672547	0.630774828	-0.471915704	0.636986958	0.996944933
1.3.1.38	mitochondrial trans-2-enoyl-CoA reductase	12.07210064	0.736399504	1.989110089	0.370215559	0.711221884	0.996944933
4.2.1.151	chorismate dehydratase	8.95134946	-0.814896966	0.951442803	-0.856485501	0.39172929	0.996944933
2.4.1.259	alpha-1,2-mannosyltransferase	1.191163093	0.624609261	4.100709476	0.152317365	0.87893663	0.996944933
2.5.1.78	6,7-dimethyl-8-ribityllumazine synthase	125.3847375	-0.434562122	0.392715593	-1.106556832	0.26848558	0.996944933
2.7.8.12	CDP-glycerol glycerophosphotransferase	32.67163653	-0.15378432	0.710170889	-0.216545513	0.828562552	0.996944933
3.5.3.8	formiminoglutamase	2.637396381	0.844432593	1.942060373	0.434812741	0.663698356	0.996944933
3.5.99.10	2-iminobutanoate/2-iminopropanoate deaminase	243.9802341	0.102635861	0.298768608	0.343529602	0.731200075	0.996944933
1.1.1.27	L-lactate dehydrogenase	346.4267477	0.063934704	0.513748446	0.124447489	0.900960975	0.996944933
2.8.3.5	3-oxoacid CoA-transferase	11.57711064	-0.950837344	1.10900151	-0.857381469	0.391234097	0.996944933
2.7.8.14	CDP-ribitol ribitolphosphotransferase / teichoic acid ribitol-pho	27.28268986	0.56343701	0.967059821	0.582628911	0.560143139	0.996944933
6.3.1.19	proteasome accessory factor A	1.381132656	-2.269288092	4.101228294	-0.553319135	0.580044901	0.996944933
3.4.17.13	muramoyltetrapeptide carboxypeptidase	75.16794398	-0.702040405	0.865171181	-0.811446821	0.41710912	0.996944933
3.4.22.70	sortase A	1125.394995	0.372092044	0.342719176	1.085705353	0.277609372	0.996944933
4.1.1.85	3-dehydro-L-gulonate-6-phosphate decarboxylase	0.249683108	-0.022040033	4.135339665	-0.005329679	0.995747552	0.996944933
1.5.1.7	saccharopine dehydrogenase (NAD+, L-lysine forming)	197.2445999	-0.209841329	0.320854671	-0.6540074	0.513107038	0.996944933
3.4.19.1	acylaminoacyl-peptidase	1.868496682	1.415593315	1.853420276	0.763773513	0.445002224	0.996944933
3.1.3.73	alpha-ribazole phosphatase	197.9568009	-0.019664998	0.258383951	-0.076107662	0.939333444	0.996944933
2.1.1.166	23S rRNA (uridine2552-2'-O)-methyltransferase	15.58275964	-1.070457494	1.16636771	-0.917770172	0.358739201	0.996944933
3.4.21.110	C5a peptidase	1.02995056	0.994735621	4.115252358	0.241719228	0.80899773	0.996944933
5.1.1.3	glutamate racemase	294.3667194	0.007494827	0.220032919	0.034062298	0.972827473	0.996944933
1.8.1.2	#N/A	13.3789185	-1.211072772	1.196952412	-1.011796927	0.31163516	0.996944933
3.1.3.96	pseudouridine 5'-phosphatase	0.416289782	-0.290558718	4.123574593	-0.070462826	0.943825288	0.996944933
5.4.99.27	tRNA pseudouridine13 synthase	2.123783118	-0.310078071	4.098156304	-0.075662822	0.939687355	0.996944933
1.12.99.6	#N/A	96.42638783	0.446186824	0.569157576	0.783942519	0.433073839	0.996944933
3.6.1.43	dolichyldiphosphatase	0.416289782	-0.290558718	4.123574593	-0.070462826	0.943825288	0.996944933
2.7.1.192	PTS system, N-acetylmuramic acid-specific IIB component	0.136601963	0.163689037	4.139022345	0.039547754	0.968453681	0.996944933
1.3.99.33	urocanate reductase	24.00152255	0.151332058	0.675553798	0.224011852	0.822748081	0.996944933
3.6.1.41	bis(5'-nucleosyl)-tetraphosphatase (symmetrical)	3.187241846	0.274571302	1.279580522	0.214579151	0.83009545	0.996944933
6.3.5.2	GMP synthase (glutamine-hydrolysing)	746.5195763	0.063709322	0.248014252	0.256877666	0.797273212	0.996944933
3.6.1.29	bis(5'-adenosyl)-triphosphatase	0.035008583	0.581845947	4.14032189	0.140531573	0.888240008	0.996944933
2.3.1.76	diacylglycerol O-acyltransferase 1	1.228064538	0.467007348	4.100876508	0.113879886	0.909333011	0.996944933
3.1.6.8	arylsulfatase A	1.683971084	2.135170131	3.132640591	0.681587967	0.495499526	0.996944933
1.14.13.59	lysine N6-hydroxylase	1.497268624	0.900309152	4.09926454	0.219626995	0.826161666	0.996944933
2.7.1.170	anhydro-N-acetylmuramic acid kinase	25.33708748	0.044475034	0.656069884	0.067790086	0.945952736	0.996944933
2.6.1.57	aromatic-amino-acid transaminase	22.44406187	0.556912333	0.709038563	0.78544717	0.432191434	0.996944933
2.7.8.36	undecaprenyl phosphate N,N'-diacetylbacillosamine 1-phospha	2.256262368	-0.740835382	2.451606637	-0.302183626	0.762512086	0.996944933
3.1.2.6	hydroxyacylglutathione hydrolase	0.647579986	-1.740079966	4.111242941	-0.423249122	0.67211351	0.996944933
1.15.1.1	nickel superoxide dismutase	1042.834107	0.414616624	0.63316997	0.654826734	0.512579316	0.996944933
2.4.1.101	alpha-1,3-mannosyl-glycoprotein beta-1,2-N-acetylglucosaminy	4.740996338	0.523439416	3.292692448	0.15897003	0.873692486	0.996944933
3.5.4.43	hydroxydechloroatrazine ethylaminohydrolase	1.738495381	-1.471882284	2.066138046	-0.712383322	0.476227441	0.996944933
2.3.2.17	peptidoglycan pentaglycine glycine transferase (the second and	0.581404477	1.431579331	4.096841987	0.349434842	0.72676288	0.996944933
2.4.99.17	S-adenosylmethionine:tRNA ribosyltransferase-isomerase	312.4137439	-0.247143887	0.294356629	-0.839607003	0.401128772	0.996944933
2.7.1.184	sulfofructose kinase	3.910939087	-1.427051943	1.412518036	-1.01028936	0.312356677	0.996944933
3.5.99.6	#N/A	813.8476878	0.314721792	0.323952473	0.971506065	0.331296334	0.996944933
2.4.99.12	3-deoxy-D-manno-octulosonic-acid transferase	104.6062867	-0.408074653	0.481327791	-0.847810288	0.396543632	0.996944933
3.2.1.113	mannosyl-oligosaccharide alpha-1,2-mannosidase	10.79752778	0.437789449	4.093783545	0.106940058	0.914836534	0.996944933
3.1.3.36	inositol polyphosphate 5-phosphatase INPP5B/F	8.344447898	0.821420225	4.093970444	0.200641464	0.840978934	0.996944933
1.13.12.-	#N/A	1.914163853	0.378587275	4.083544837	0.092710449	0.926133596	0.996944933
2.7.7.47	streptomycin 3''-adenylyltransferase	12.33183838	-0.334218783	1.073551638	-0.31132064	0.755556878	0.996944933
1.14.11.27	F-box and leucine-rich repeat protein 10/11	0.245483854	0.945385589	4.138694701	0.228426027	0.819315057	0.996944933
1.1.3.21	alpha-glycerophosphate oxidase	4.838986957	1.683910687	2.355174327	0.714983459	0.474619264	0.996944933
1.3.1.12	prephenate dehydrogenase	153.5917507	-0.266940266	0.294773088	-0.905578825	0.365158821	0.996944933
5.3.99.11	2-keto-myo-inositol isomerase	4.341769719	-1.471583013	1.32925443	-1.107073995	0.268261938	0.996944933
1.5.5.2	RHH-type transcriptional regulator, proline utilization regulon re	63.59410052	0.266833227	0.484502665	0.550736344	0.581814427	0.996944933

2.1.2.9	methionyl-tRNA formyltransferase	346.3261987	-0.077293092	0.15423234	-0.501147112	0.616267593	0.996944933
2.7.1.17	xylulokinase	345.6234778	-0.011851986	0.307627786	-0.038527033	0.969267479	0.996944933
2.7.7.13	mannose-1-phosphate guanylyltransferase	358.2910064	0.152763792	0.283261303	0.539303429	0.589677504	0.996944933
5.4.3.9	glutamate 2,3-aminomutase	0.081980782	0.293232697	4.138295101	0.070858334	0.943510505	0.996944933
1.11.1.15	peroxiredoxin (alkyl hydroperoxide reductase subunit C)	1347.271603	0.386043421	0.576660975	0.669446066	0.503210974	0.996944933
3.5.4.12	dCMP deaminase	245.9939325	0.042948628	0.270278383	0.158905155	0.873743599	0.996944933
3.6.3.40	teichoic acid transport system ATP-binding protein	74.48854514	-0.312377202	0.550788301	-0.567145675	0.570615213	0.996944933
2.4.1.250	D-inositol-3-phosphate glycosyltransferase	0.122971174	0.118293499	4.136874465	0.028594897	0.977187682	0.996944933
2.1.-.-	#N/A	0.255199619	0.240083024	4.122870721	0.058232004	0.953563828	0.996944933
3.1.11.5	exodeoxyribonuclease V	537.3560927	-0.027464726	0.393027704	-0.069879873	0.944289273	0.996944933
1.1.1.336	UDP-N-acetyl-D-mannosaminuronic acid dehydrogenase	25.22143025	-0.770386415	0.830595305	-0.92751116	0.3536612	0.996944933
1.14.13.-	#N/A	2.723150752	-2.338859591	3.089236728	-0.757099503	0.448990252	0.996944933
3.6.1.65	(d)CTP diphosphatase	3.073861829	1.059368696	2.778004533	0.381341601	0.702949789	0.996944933
4.1.2.17	L-fuculose-phosphate aldolase	163.6600843	-0.136810363	0.445051631	-0.307403353	0.758536376	0.996944933
2.5.1.41	phosphoglycerol geranylgeranyltransferase	0.624441127	0.64883422	4.107926839	0.157946878	0.874498658	0.996944933
5.1.99.3	allantoin racemase	5.521276769	-0.317579739	1.217960767	-0.2607471	0.794287541	0.996944933
1.1.1.205	IMP dehydrogenase	601.6865534	0.060901473	0.278093144	0.21899667	0.826652642	0.996944933
2.4.1.325	dTDP-N-acetylfucosamine:lipid II N-acetylfucosaminyltransferas	0.162992848	-0.25620097	4.132822836	-0.061991762	0.950569392	0.996944933
2.1.3.11	N-succinyl-L-ornithine transcarbamylase	88.30516408	0.187094717	0.400168989	0.46753927	0.640114103	0.996944933
3.5.4.9	methylenetetrahydrofolate dehydrogenase (NADP+) / metheny	460.8469926	0.286821667	0.32574259	0.880516321	0.378579668	0.996944933
1.1.1.384	dTDP-3,4-didehydro-2,6-dideoxy-alpha-D-glucose 3-reductase	3.094463476	1.538353708	2.292490265	0.671040454	0.502194759	0.996944933
2.1.1.203	tRNA (cytosine34-C5)-methyltransferase	2.749594251	0.169928906	3.03828906	0.055929144	0.955398254	0.996944933
2.3.2.10	serine/alanine adding enzyme	5.042106869	0.227550081	1.812935371	0.125514723	0.90011607	0.996944933
4.1.2.50	6-pyruvoyltetrahydropterin/6-carboxytetrahydropterin synthas	143.680634	0.303294197	0.407197221	0.744833661	0.456372283	0.996944933
3.6.-.-	#N/A	345.8259585	0.066570747	0.224078817	0.297086303	0.766400621	0.996944933
6.3.1.1	aspartate--ammonia ligase	200.3070772	-0.264363864	0.286368376	-0.923160121	0.355923767	0.996944933
3.1.1.89	protein phosphatase methylesterase 1	1.451571512	0.462223117	4.099464378	0.112752076	0.910227114	0.996944933
2.1.1.298	ribosomal protein L3 glutamine methyltransferase	2.881866914	0.475262984	1.147938295	0.414014399	0.678863561	0.996944933
1.7.5.1	nitrate reductase / nitrite oxidoreductase, alpha subunit	30.68379277	0.206387158	0.926613956	0.222732624	0.823743609	0.996944933
1.3.1.89	tRNA-dihydrouridine synthase 3	2.528684853	0.40067396	3.932969152	0.101875694	0.918855343	0.996944933
1.13.11.2	catechol 2,3-dioxygenase	2.031466026	2.889334775	3.423102875	0.844068928	0.39863089	0.996944933
6.4.1.5	geranyl-CoA carboxylase alpha subunit	0.683567942	-0.322482505	4.111670109	-0.078431026	0.937485194	0.996944933
3.4.11.7	glutamyl aminopeptidase	3.896020177	1.371556682	2.308576945	0.59411348	0.552436216	0.996944933
3.5.1.98	histone deacetylase 1/2	6.724734731	0.880943731	4.094231535	0.215167052	0.829637078	0.996944933
3.1.26.12	ribonuclease E	21.05414725	0.196126455	0.910295602	0.21545359	0.829413692	0.996944933
5.4.99.58	methylornithine synthase	1.195008066	-0.618495311	2.742908416	-0.225488867	0.821598985	0.996944933
2.7.1.26	riboflavin kinase	334.3661825	-0.18997835	0.232337296	-0.817683399	0.413537992	0.996944933
3.2.1.33	glycogen debranching enzyme	2.01001148	1.907519589	4.016132679	0.474964286	0.634812429	0.996944933
1.1.3.-	#N/A	0.196207128	1.002864067	4.129916598	0.242829133	0.808137766	0.996944933
3.1.1.3	triacylglycerol lipase	4.439500809	-0.603117469	1.519279066	-0.396976094	0.69138509	0.996944933
3.1.13.4	poly(A)-specific ribonuclease	2.749647617	0.449714955	4.096427956	0.10978222	0.912582092	0.996944933
3.5.2.18	enamidase	3.748924151	0.077003125	1.90076968	0.040511549	0.967685299	0.996944933
2.7.1.55	allose kinase	14.51981234	0.037454665	0.757195312	0.049464998	0.96054873	0.996944933
4.1.1.19	arginine decarboxylase	178.3389114	-0.104198356	0.468742725	-0.222293276	0.824085586	0.996944933
4.1.2.40	tagatose 1,6-diphosphate aldolase	239.5719266	-0.093049033	0.672281671	-0.138407809	0.889918126	0.996944933
2.1.1.64	2-polyprenyl-6-hydroxyphenyl methylase / 3-demethylubiquinc	0.340645926	0.699344457	4.12584154	0.16950347	0.865400643	0.996944933
2.3.1.61	2-oxoglutarate dehydrogenase E2 component (dihydrolipoamid	116.1652786	0.437123089	0.679407217	0.64338894	0.519971758	0.996944933
3.1.1.17	gluconolactonase	0.591879764	-0.229224005	4.10632942	-0.055822118	0.955483515	0.996944933
4.1.2.43	3-hexulose-6-phosphate synthase	9.761425783	-0.803000718	0.952006575	-0.843482323	0.398958748	0.996944933
4.2.1.80	2-keto-4-pentenoate hydratase	0.950669968	-0.251366033	4.112056364	-0.061129034	0.951256447	0.996944933
3.5.3.11	agmatinase	76.90483775	0.052209946	0.376612553	0.138630391	0.889742227	0.996944933
6.6.1.2	cobaltochelatase CobN	121.2009386	-0.198535095	0.591152953	-0.335843869	0.736988607	0.996944933
2.1.1.183	18S rRNA (adenine1779-N6/adenine1780-N6)-dimethyltransfer	1.791028411	1.049026415	4.098140775	0.255976179	0.797969231	0.996944933
3.5.-.-	#N/A	13.91699383	-0.179469091	0.737197705	-0.243447707	0.80765859	0.996944933
3.7.1.20	fumarylpyruvate hydrolase	5.87380492	1.132448491	1.29035795	0.877623524	0.380148059	0.996944933
2.7.8.28	LPPG:FO 2-phospho-L-lactate transferase	0.874214996	0.432778868	4.104167584	0.105448635	0.916019826	0.996944933
1.1.1.85	3-isopropylmalate dehydrogenase	228.1220397	-0.105856226	0.239344396	-0.442275768	0.658289664	0.996944933
3.2.1.122	maltose-6'-phosphate glucosidase	26.0661655	0.01955522	0.655728988	0.029822107	0.976208928	0.996944933
2.1.2.3	phosphoribosylaminoimidazolecarboxamide formyltransferase	547.7326221	0.200427597	0.18265647	1.097292626	0.272513493	0.996944933
3.2.1.89	arabinogalactan endo-1,4-beta-galactosidase	105.1190644	-0.58915587	0.579824745	-1.016093009	0.309585098	0.996944933
2.8.3.8	#N/A	183.3888661	0.488793027	0.529573227	0.922994218	0.356010218	0.996944933
2.1.1.271	precorrin-4/cobalt-precorrin-4 C11-methyltransferase	98.82307915	-0.392261386	0.371773178	-1.05510943	0.29137528	0.996944933
1.8.4.-	#N/A	2.424680651	0.585167097	2.854808734	0.204975938	0.837590925	0.996944933
2.4.2.9	uracil phosphoribosyltransferase	517.0070671	0.253330326	0.296937774	0.853142808	0.393580114	0.996944933
3.4.-.-	#N/A	1952.852084	-0.235063433	0.294526527	-0.798106152	0.424808892	0.996944933
3.1.1.73	feruloyl esterase	0.78497298	-0.736948649	4.094427532	-0.179988202	0.857161831	0.996944933
1.7.2.3	trimethylamine-N-oxide reductase (cytochrome c)	15.37738031	0.330306481	0.697950206	0.473252215	0.636033249	0.996944933
1.4.3.-	#N/A	0.039493742	1.096522945	4.143245313	0.264653155	0.791276668	0.996944933
2.5.1.19	3-phosphoshikimate 1-carboxyvinyltransferase	298.9208236	-0.328129034	0.345546177	-0.949595323	0.342317916	0.996944933
2.1.3.2	aspartate carbamoyltransferase	349.324848	-0.018880994	0.284550818	-0.066353681	0.947096246	0.996944933
3.5.1.78	glutathionylspermidine amidase/synthetase	1.249834218	-1.200825655	3.40618042	-0.352543174	0.724430946	0.996944933
2.1.3.1	methylmalonyl-CoA carboxyltransferase 5S subunit	0.495699816	0.148125937	4.124846347	0.035910656	0.971353599	0.996944933
2.1.1.214	tRNA (guanine10-N2)-methyltransferase	2.397965246	0.949052788	4.096792469	0.231657521	0.816804027	0.996944933
6.2.1.13	acetate---CoA ligase (ADP-forming) subunit alpha	21.3186108	0.38108903	0.812974328	0.468758996	0.639241912	0.996944933
1.2.1.27	malonate-semialdehyde dehydrogenase (acetylating) / methylr	18.35639239	0.371870783	0.583881065	0.636894747	0.524193404	0.996944933
2.3.3.9	malate synthase	7.740445911	1.253356686	2.368379069	0.529204426	0.596663647	0.996944933
1.5.99.15	dihydromethanopterin reductase (acceptor)	1.787205415	0.771339966	4.097940143	0.188226265	0.850699284	0.996944933
3.1.3.12	trehalose 6-phosphate phosphatase	40.02501221	-0.622321453	0.609539764	-1.02096941	0.307268932	0.996944933
5.3.1.30	5-deoxy-glucuronate isomerase	31.66558741	-0.268479304	0.470586111	-0.570521096	0.568324318	0.996944933
2.1.1.178	16S rRNA (cytosine1407-C5)-methyltransferase	0.283636172	-0.155304167	4.132534895	-0.037580848	0.970021878	0.996944933
2.7.2.7	butyrate kinase	160.9491394	-0.372799436	0.384181977	-0.970372006	0.331861097	0.996944933
3.2.1.133	cyclomaltodextrinase / maltogenic alpha-amylase / neopullulan	0.30127684	-0.681748901	4.12669228	-0.165204686	0.868782877	0.996944933
1.5.3.22	coenzyme F420H2 oxidase	4.822589886	0.34043754	2.160135658	0.157600074	0.874771945	0.996944933
1.17.4.2	ribonucleoside-triphosphate reductase (thioredoxin)	1225.795642	0.035217544	0.295817567	0.119051565	0.905234503	0.996944933
2.7.2.11	glutamate 5-kinase	246.2464691	-0.202357823	0.195971694	-1.032586999	0.301797215	0.996944933
2.7.8.8	CDP-diacylglycerol---serine O-phosphatidyltransferase	124.5052398	-0.319960681	0.503218052	-0.635829098	0.524887819	0.996944933
6.1.1.7	alanyl-tRNA synthetase	1204.488849	0.196400172	0.303708869	0.646672494	0.517843929	0.996944933

2.6.1.52	phosphoserine aminotransferase	469.6711707	-0.048868192	0.390044524	-0.125288753	0.900294956	0.996944933
3.1.1.61	two-component system, chemotaxis family, protein-glutamate	50.27352804	-0.09780818	0.650477424	-0.150363682	0.880477693	0.996944933
2.5.1.90	octaprenyl-diphosphate synthase	86.87419176	-0.269194559	0.477988915	-0.563181594	0.573311235	0.996944933
3.5.1.16	acetylornithine deacetylase	73.83330864	-0.080952424	0.422446425	-0.19162767	0.848033867	0.996944933
1.1.1.202	1,3-propanediol dehydrogenase	2.374458127	-2.243513034	4.087149497	-0.54891876	0.583061203	0.996944933
1.20.1.1	phosphonate dehydrogenase	0.332585466	0.070611165	4.115196288	0.017158638	0.986310059	0.996944933
2.3.1.234	N6-L-threonylcarbamoyladenine synthase	531.8124809	0.251043843	0.316096891	0.794199025	0.427079576	0.996944933
4.1.99.22	GTP 3',8-cyclase	105.6089693	-0.127522679	0.356441198	-0.357766386	0.720518148	0.996944933
1.4.1.3	glutamate dehydrogenase (NAD(P)+)	18.29244421	0.017027595	0.620533148	0.027440267	0.978108582	0.996944933
2.6.1.97	archaeosine synthase	0.7285168	0.850480625	4.105603063	0.207151206	0.835891773	0.996944933
2.1.1.193	16S rRNA (uracil1498-N3)-methyltransferase	289.4001831	-0.107583646	0.437874345	-0.245695249	0.80591815	0.996944933
1.1.2.3	L-lactate dehydrogenase (cytochrome)	0.529669772	-0.646149798	4.118129659	-0.156903704	0.875320741	0.996944933
3.1.3.66	inositol polyphosphate-4-phosphatase	1.375871973	-0.173820388	4.10151829	-0.042379523	0.966196152	0.996944933
2.7.11.17	calcium/calmodulin-dependent protein kinase kinase 1	11.32903906	0.54042396	4.093727954	0.132012671	0.89497427	0.996944933
2.3.1.210	dTDP-4-amino-4,6-dideoxy-D-galactose acyltransferase	0.061162681	0.413195434	4.141488419	0.099769791	0.920527092	0.996944933
2.1.1.221	tRNA (guanine9-N1)-methyltransferase	3.682511275	0.716828776	2.893749406	0.247716258	0.804353951	0.996944933
2.3.1.202	UDP-4-amino-4,6-dideoxy-N-acetyl-beta-L-altrosamine N-acety	0.206011882	-0.153343021	4.12913762	-0.037136815	0.970375918	0.996944933
3.4.13.-	#N/A	542.4797353	0.295970857	0.277357864	1.067108225	0.285922968	0.996944933
3.4.24.64	mitochondrial-processing peptidase subunit alpha	2.288629601	0.745750652	3.221750893	0.231473716	0.816946802	0.996944933
3.4.21.107	serine protease Do	631.2321672	0.159435933	0.386761709	0.412232983	0.680168664	0.996944933
3.1.26.4	ribonuclease HI	1467.073592	-0.689182259	0.643744025	-1.07058432	0.284356377	0.996944933
3.7.1.5	acylpyruvate hydrolase	0.424682647	1.733082104	4.113349588	0.421331099	0.673513324	0.996944933
2.1.1.13	5-methyltetrahydrofolate--homocysteine methyltransferase	580.1326798	-0.012089585	0.409146268	-0.029548321	0.976427281	0.996944933
1.8.4.2	protein-disulfide reductase (glutathione)	11.94444886	0.779620802	4.093648825	0.190446429	0.848959318	0.996944933
5.4.99.23	23S rRNA pseudouridine1911/1915/1917 synthase	11838.22793	-1.195908758	1.110934329	-1.076489156	0.281708548	0.996944933
2.1.1.248	methylamine---corrinoid protein Co-methyltransferase	0.303919997	1.240510147	4.125759372	0.300674382	0.763662806	0.996944933
2.7.13.3	two-component system, sensor histidine kinase PdtaS	2275.191186	-0.197969628	0.274708885	-0.720652437	0.471123383	0.996944933
2.8.3.19	CoA:oxalate CoA-transferase	7.009045476	0.093003868	0.978327416	0.095064154	0.92426387	0.996944933
2.7.1.207	PTS system, lactose-specific IIA component	9.523751013	-0.09301042	0.990418598	-0.093910212	0.925180482	0.996944933
2.7.1.2	glucokinase	1290.074803	0.417951868	0.510562663	0.818610327	0.413008773	0.996944933
2.7.7.58	#N/A	3.116189468	-0.237141253	2.62868872	-0.090212756	0.928118148	0.996944933
6.3.2.2	glutamate--cysteine ligase	19.66428531	0.585700933	0.575130157	1.018379798	0.308497499	0.996944933
6.3.4.5	argininosuccinate synthase	292.8803441	-0.086725432	0.193456206	-0.448294906	0.653940376	0.996944933
3.5.1.118	gamma-glutamyl hercynylcysteine S-oxide hydrolase	0.812531649	0.277618453	3.226690757	0.086038135	0.931436103	0.996944933
1.5.-.-	#N/A	6.74317746	-0.920545795	1.064226277	-0.864990666	0.387043912	0.996944933
2.7.7.43	N-acylneuraminate cytidylyltransferase	11.83068856	-0.876697331	1.089700728	-0.804530371	0.421090737	0.996944933
2.7.1.67	phosphatidylinositol 4-kinase A	1.710812699	0.646257754	3.549795487	0.182054926	0.855539622	0.996944933
1.1.1.35	3-hydroxyacyl-CoA dehydrogenase	4.746715694	-0.659153252	1.363461019	-0.483441215	0.62878249	0.996944933
2.8.4.4	ribosomal protein S12 methylthiotransferase	623.2588148	0.29173027	0.27502405	1.060744576	0.288805996	0.996944933
1.1.1.58	tagaturonate reductase	243.3490786	0.163105383	0.345400684	0.472220787	0.636769204	0.996944933
2.6.1.76	diaminobutyrate-2-oxoglutarate transaminase	5.447023162	-1.5300915	1.525230663	-1.003186952	0.315770667	0.996944933
3.4.24.40	serralysin	0.231763367	-0.19515031	4.126197015	-0.047295442	0.962277761	0.996944933
2.8.3.16	formyl-CoA transferase	2.442937505	-1.131759461	1.947597911	-0.581105296	0.561169489	0.996944933
3.1.3.97	3',5'-nucleoside bisphosphate phosphatase	1311.671976	0.64493086	0.724396039	0.890301472	0.373304031	0.996944933
4.2.1.90	L-rhamnonate dehydratase	2.379476149	0.266854802	1.832091177	0.145655852	0.884193074	0.996944933
3.6.1.13	ADP-ribose pyrophosphatase	291.3683185	-0.392823093	0.442832024	-0.887070201	0.375041106	0.996944933
1.1.-.-	#N/A	107.6860614	0.233866285	0.491477575	0.475843246	0.634186058	0.996944933
3.1.1.11	pectinesterase	172.979135	0.374365615	0.49212793	0.760707922	0.446831542	0.996944933
2.3.2.18	peptidoglycan pentaglycine glycine transferase (the fourth and	0.020495196	0.766777793	4.142686886	0.185091902	0.853156953	0.996944933
3.5.4.28	5-methylthioadenosine/S-adenosylhomocysteine deaminase	646.4515798	-0.179328032	0.774803878	-0.23144958	0.816965551	0.996944933
1.6.5.8	#N/A	428.1980446	-0.032779271	0.4386088	-0.074734641	0.940425845	0.996944933
5.3.2.6	4-oxalocrotonate tautomerase	9.196708722	-0.384492692	1.301852286	-0.295342795	0.767732028	0.996944933
1.2.4.1	pyruvate dehydrogenase (acetyl-transferring)	104.4906236	-0.458293285	0.817489716	-0.56061046	0.57506312	0.996944933
3.5.3.23	succinylarginine dihydrolase	0.099032361	1.476806516	4.143282072	0.356433979	0.721515586	0.996944933
5.1.99.8	7,8-dihydroneopterin aldolase/epimerase/oxygenase	59.65369116	-0.164462877	0.606861495	-0.271005621	0.786386709	0.996944933
1.1.1.37	malate dehydrogenase	310.0391925	0.400207238	0.404850482	0.988530966	0.322892677	0.996944933
3.6.1.7	acylphosphatase	266.5174633	0.571299448	0.653491823	0.874225855	0.381995268	0.996944933
2.1.1.220	tRNA (adenine57-N1/adenine58-N1)-methyltransferase catalyt	3.95831283	0.017198175	1.726325169	0.009962304	0.992051363	0.996944933
3.4.22.48	staphopain A	0.245162986	-3.155835298	4.143731151	-0.761592676	0.446303147	0.996944933
2.7.4.22	uridylate kinase	517.0140077	0.261300291	0.265974634	0.982425603	0.325890218	0.996944933
1.3.1.88	tRNA-dihydrouridine synthase 1	0.249773869	-0.154751873	4.134519116	-0.037429232	0.970142766	0.996944933
1.1.1.22	UDPglucose 6-dehydrogenase	273.6119241	-0.35931439	0.349913502	-1.026866322	0.304483414	0.996944933
3.4.19.-	#N/A	72.96599458	0.194805425	0.4283386	0.454793065	0.64925812	0.996944933
3.2.1.184	GDP/UDP-N,N'-diacetylbacillosamine 2-epimerase (hydrolysing)	9.274406239	0.766730667	0.7986686	0.960011033	0.337049662	0.996944933
6.3.5.6	aspartyl-tRNA(Asn)/glutamyl-tRNA(Gln) amidotransferase subu	500.7375315	-0.131477958	0.458592295	-0.286699012	0.774342794	0.996944933
2.1.1.320	type II protein arginine methyltransferase	0.416289782	-0.290558718	4.123574593	-0.070462826	0.943825288	0.996944933
4.1.1.4	acetoacetate decarboxylase	0.197769159	1.060505765	4.141780262	0.256050707	0.797911683	0.996944933
3.2.2.21	DNA-3-methyladenine glycosylase II	50.9815646	-0.137362337	0.41385896	-0.331906156	0.739960122	0.996944933
6.1.1.13	D-alanine--poly(phosphoribitol) ligase subunit 1	16.40246966	0.315817395	0.653346134	0.483384502	0.628822751	0.996944933
4.1.1.20	diaminopimelate decarboxylase	541.1671212	-0.226100579	0.430717571	-0.524939297	0.599625391	0.996944933
6.3.4.4	adenylosuccinate synthase	684.2271798	0.298483676	0.29731824	1.00391983	0.315417258	0.996944933
3.6.5.4	signal recognition particle subunit SRP54	622.0072358	0.120422524	0.415774612	0.289634144	0.772096143	0.996944933
1.1.1.132	GDP-mannose 6-dehydrogenase	5.365709567	-0.679444274	2.021345159	-0.336134712	0.736769283	0.996944933
4.2.2.23	rhamnogalacturonan endolyase	83.38770096	-0.213636337	0.567796857	-0.376254878	0.706727421	0.996944933
1.17.7.4	4-hydroxy-3-methylbut-2-en-1-yl diphosphate reductase	340.093813	0.086774137	0.418414256	0.207388099	0.835706776	0.996944933
4.1.1.74	indolepyruvate decarboxylase	0.623578467	-1.55909264	4.113038938	-0.379060997	0.704642569	0.996944933
4.2.1.19	imidazoleglycerol-phosphate dehydratase / histidinol-phosphat	179.9053962	-0.152186825	0.212763815	-0.715285281	0.474432782	0.996944933
6.3.-.-	#N/A	0.185059353	0.545169479	4.138654554	0.131726258	0.895200815	0.996944933
2.7.12.2	mitogen-activated protein kinase kinase 1	0.552060613	-0.31048877	4.116122808	-0.075432339	0.93987073	0.996944933
3.1.3.89	5'-deoxynucleotidase	496.8947972	-1.040264698	1.079875697	-0.963318927	0.335387498	0.996944933
4.1.1.41	#N/A	2.58784862	0.627454046	3.147030159	0.199379737	0.84196571	0.996944933
3.1.3.57	inositol polyphosphate 1-phosphatase	0.363965079	0.911530758	4.138110668	0.220277037	0.825655403	0.996944933
4.1.1.37	uroporphyrinogen decarboxylase	83.4923204	0.085411624	0.563611837	0.151543346	0.87954712	0.996944933
2.7.1.195	PTS system, 2-O-A-mannosyl-D-glycerate-specific IIA componer	0.248796225	-0.588898762	4.12644119	-0.142713475	0.886516474	0.996944933
1.11.1.5	cytochrome c peroxidase	35.45100758	0.120621137	0.599720528	0.201128912	0.840597776	0.996944933
5.1.1.20	L-Ala-D/L-Glu epimerase	206.6439338	-0.053707817	0.649757306	-0.082658274	0.934123264	0.996944933
2.6.1.109	8-amino-3,8-dideoxy-alpha-D-manno-octulosonate transaminas	0.052512875	0.445935252	4.139416356	0.107729016	0.914210652	0.996944933

3.5.99.2	hydroxymethylpyrimidine/phosphomethylpyrimidine kinase / t	29.66472934	0.269558009	0.737247179	0.365627726	0.714642883	0.996944933
3.2.1.86	#N/A	1540.806296	0.551765331	0.555305041	0.993625647	0.320405153	0.996944933
1.8.1.7	glutathione reductase (NADPH)	5.508827407	0.879887497	1.239545068	0.70984712	0.477798945	0.996944933
2.7.7.79	tRNA(His) guanylyltransferase	1.231597149	1.019483963	4.100397702	0.248630508	0.803646608	0.996944933
5.1.3.8	N-acylglucosamine 2-epimerase	139.7091188	-0.299284441	0.438349665	-0.682752755	0.494763088	0.996944933
2.4.1.247	beta-D-galactosyl-(1->4)-L-rhamnose phosphorylase	2.458672492	-1.421496058	1.912740049	-0.743172633	0.45737717	0.996944933
2.3.1.247	3-keto-5-aminohexanoate cleavage enzyme	82.61576769	0.407606266	0.483002323	0.843901253	0.398724588	0.996944933
1.1.1.380	L-gulonate 5-dehydrogenase	17.29678145	0.059885183	0.771258822	0.077646026	0.938109629	0.996944933
2.1.1.131	precorrin-3B C17-methyltransferase	100.1396827	-0.05041927	0.361970912	-0.139290944	0.889220246	0.996944933
2.7.1.151	#N/A	2.104401683	0.730453733	4.097269769	0.178278164	0.858504527	0.996944933
2.3.1.12	pyruvate dehydrogenase E2 component (dihydrolipoamide ace	87.93707027	-0.303921664	0.855678555	-0.355182051	0.722453211	0.996944933
4.1.2.13	fructose 1,6-bisphosphate aldolase/phosphatase	1639.603458	0.483850541	0.467438814	1.035109894	0.300617592	0.996944933
2.4.1.186	glycogenin	0.70344152	2.615356955	4.113878426	0.635739972	0.524945919	0.996944933
3.1.3.81	phosphatidylglycerophosphatase B	0.236562323	-0.038273702	4.135351276	-0.009255248	0.992615486	0.996944933
5.3.3.3	4-hydroxybutyryl-CoA dehydratase / vinylacetyl-CoA-Delta-ison	56.64405634	0.260952547	0.420449098	0.620651936	0.53482866	0.996944933
1.2.1.21	lactaldehyde dehydrogenase / glycolaldehyde dehydrogenase	20.27192498	-0.451444399	0.575426098	-0.784539319	0.43272372	0.996944933
5.3.1.24	phosphoribosylanthranilate isomerase	103.2707371	-0.548855302	0.496650659	-1.105113409	0.269110451	0.996944933
2.6.1.36	L-lysine 6-transaminase	2.906010432	2.466922764	3.819377908	0.645896485	0.518346396	0.996944933
2.7.7.2	FAD synthetase	334.3661825	-0.18997835	0.232337296	-0.817683399	0.413537992	0.996944933
2.5.1.21	farnesyl-diphosphate farnesyltransferase	3.986138423	-0.888583331	1.798625444	-0.494034672	0.621281693	0.996944933
4.2.1.47	GDPmannose 4,6-dehydratase	191.2217744	-0.189529014	0.434266079	-0.436435226	0.662520986	0.996944933
1.7.1.13	7-cyano-7-deazaguanine reductase	60.68859191	0.079786141	0.560540703	0.142337819	0.886813176	0.996944933
6.1.1.1	tyrosyl-tRNA synthetase	369.2065589	-0.182987627	0.222636524	-0.821911984	0.411126992	0.996944933
4.2.1.33	3-isopropylmalate dehydratase	590.1564115	-0.077070162	0.157230958	-0.490171671	0.624012425	0.996944933
4.1.1.68	#N/A	0.157074332	2.252406163	4.133738514	0.544883561	0.585833603	0.996944933
3.1.3.1	alkaline phosphatase	149.18058	-0.10196467	0.42038825	-0.24254881	0.808354941	0.996944933
1.1.3.15	glycolate oxidase	168.3993579	0.034685485	0.416870092	0.083204541	0.933688902	0.996944933
6.3.2.36	4-phosphopantoate---beta-alanine ligase	2.229993848	0.703417954	4.097029595	0.171689742	0.863681451	0.996944933
3.1.3.16	protein phosphatase	407.6783791	0.25943328	0.285634017	0.908271651	0.363734716	0.996944933
1.4.1.16	diaminopimelate dehydrogenase	191.3264728	-0.221761573	0.346966939	-0.639143239	0.522729753	0.996944933
3.1.3.5	5'-nucleotidase	334.6478068	0.063370637	0.324750072	0.195136637	0.845285982	0.996944933
2.7.9.3	selenide, water dikinase	162.5069829	0.058723477	0.34479603	0.170313668	0.864763463	0.996944933
3.2.1.52	beta-N-acetylhexosaminidase	1969.843787	-0.241772466	0.405218976	-0.596646455	0.550743449	0.996944933
3.1.1.45	carboxymethylenebutenolidase	0.306481125	1.367423321	4.133089968	0.330847703	0.740759526	0.996944933
3.8.1.5	haloalkane dehalogenase	0.039493742	1.096522945	4.143245313	0.264653155	0.791276668	0.996944933
6.5.1.4	RNA 3'-terminal phosphate cyclase (ATP)	0.559597965	-0.945060743	4.12182809	-0.229281941	0.818649789	0.996944933
3.4.21.89	signal peptidase I	629.1568785	0.191194313	0.220038367	0.868913523	0.384894433	0.996944933
2.7.11.24	mitogen-activated protein kinase 1/3	19.47956978	0.178138081	4.093200154	0.043520491	0.96528663	0.996944933
3.6.1.66	XTP/dITP diphosphohydrolase	350.6006094	0.269185148	0.30279501	0.889001269	0.374002398	0.996944933
4.1.3.36	naphthoate synthase	60.70473352	0.042781317	0.471399785	0.090753791	0.927688228	0.996944933
2.1.1.172	16S rRNA (guanine1207-N2)-methyltransferase	6.81937072	0.31876008	1.206285596	0.264249263	0.791587853	0.996944933
2.7.11.1	checkpoint serine/threonine-protein kinase	1323.478779	-0.196323467	0.248436509	-0.790235977	0.429389969	0.996944933
3.6.3.34	iron complex transport system ATP-binding protein	291.8109359	-0.211964175	0.381704466	-0.555309656	0.578682875	0.996944933
2.5.1.6	S-adenosylmethionine synthetase	664.9956883	0.114297342	0.406876598	0.280914024	0.778776344	0.996944933
3.5.4.5	cytidine deaminase	296.4043363	0.247105938	0.276855876	0.892543592	0.372101638	0.996944933
5.5.1.27	D-galactarolactone cycloisomerase	0.422036594	-1.043772104	4.120233486	-0.253328387	0.800014462	0.996944933
3.2.1.25	beta-mannosidase	235.1877339	-0.113977903	0.287549827	-0.396376184	0.691827532	0.996944933
2.1.1.148	thymidylate synthase (FAD)	157.2032252	-0.314774446	0.32305968	-0.974353857	0.329880872	0.996944933
3.6.3.2	Mg2+-importing ATPase	52.92754109	-0.133854107	0.592888477	-0.225766079	0.821383361	0.996944933
2.7.4.9	dTMP kinase	164.5735539	0.312493802	0.290824997	1.074508056	0.282595036	0.996944933
6.2.1.34	feruloyl-CoA synthase	0.103724382	1.1069846	4.143731151	0.26714682	0.78935612	0.996944933
2.7.1.176	UDP-N-acetylglucosamine kinase	2.748491948	-0.91007704	1.521140106	-0.598286139	0.549649024	0.996944933
2.4.1.291	N-acetylgalactosamine-N,N'-diacetylbacillosaminyl-diphospho-u	1.395090128	-0.257671373	4.099665866	-0.062851798	0.949884518	0.996944933
2.9.1.1	L-seryl-tRNA(Ser) seleniumtransferase	31.33378398	-0.507444119	0.694098029	-0.731084224	0.46472771	0.996944933
1.4.3.16	L-aspartate oxidase	271.3226124	-0.192686907	0.27999578	-0.688177898	0.49134076	0.996944933
3.5.1.102	2-amino-5-formylamino-6-ribosylaminopyrimidin-4(3H)-one 5'-r	0.581356147	0.21556072	4.111806703	0.052424818	0.958190199	0.996944933
2.4.1.257	alpha-1,3/alpha-1,6-mannosyltransferase	1.814404281	1.028185899	4.097896762	0.250905759	0.801886971	0.996944933
2.7.4.31	5-(aminomethyl)-3-furanmethanol phosphate kinase	2.223575051	1.052386897	4.023858567	0.261536751	0.793678612	0.996944933
2.5.1.89	tritrans,polycis-undecaprenyl-diphosphate synthase [geranylge	0.762496501	1.084187859	4.101378819	0.264347164	0.791512421	0.996944933
1.17.1.8	4-hydroxy-tetrahydrodipicolinate reductase	419.5572778	0.086530509	0.280077404	0.308952124	0.757357946	0.996944933
2.1.1.80	chemotaxis protein methyltransferase CheR	51.42789199	0.36576977	0.634460036	0.576505611	0.564273479	0.996944933
1.3.1.91	tRNA-dihydrouridine synthase 2	0.173834568	0.558494693	4.137918399	0.134969963	0.892635623	0.996944933
1.17.1.1	CDP-4-dehydro-6-deoxyglucose reductase, E3	51.88480358	-0.007682718	0.458194694	-0.016767365	0.986622205	0.996944933
1.4.4.2	glycine dehydrogenase	313.8763903	0.327512079	0.420778098	0.778348683	0.436363483	0.996944933
2.8.1.9	molybdenum cofactor sulfurtransferase	0.582805694	-0.313605345	4.114963576	-0.076210965	0.939251259	0.996944933
4.1.1.32	phosphoenolpyruvate carboxykinase (GTP)	256.1271171	0.082654986	0.59264617	0.139467679	0.889080595	0.996944933
1.3.99.5	#N/A	0.280447894	1.415065436	4.115354492	0.343850193	0.730958951	0.996944933
5.4.99.15	(1->4)-alpha-D-glucan 1-alpha-D-glucosylmutase	0.103724382	1.1069846	4.143731151	0.26714682	0.78935612	0.996944933
4.2.1.9	dihydroxy-acid dehydratase	494.2953836	-0.131687022	0.253843996	-0.518771465	0.603920119	0.996944933
6.3.4.2	CTP synthase	447.5705848	0.112571216	0.231277027	0.486737561	0.626444324	0.996944933
1.12.99.-	#N/A	35.47611908	0.886196084	1.414692486	0.626423122	0.531037447	0.996944933
3.5.1.94	gamma-glutamyl-gamma-aminobutyrate hydrolase	0.020495196	0.766777793	4.142686886	0.185091902	0.853156953	0.996944933
1.1.1.363	glucose-6-phosphate 1-dehydrogenase	351.7397334	-0.072406616	0.638901053	-0.113329937	0.909768984	0.996944933
2.1.1.268	tRNAThr (cytosine32-N3)-methyltransferase	0.833542549	0.732019579	4.104061911	0.178364653	0.858436607	0.996944933
2.7.1.21	thymidine kinase	135.8458384	0.25031982	0.392347171	0.638005927	0.523469822	0.996944933
3.4.23.5	cathepsin D	0.897866547	2.587045612	4.111535163	0.629216463	0.529207352	0.996944933
2.4.1.281	4-O-beta-D-mannosyl-D-glucose phosphorylase	101.0610914	-0.019704694	0.539498126	-0.03652412	0.970864447	0.996944933
3.1.3.32	bifunctional polynucleotide phosphatase/kinase	0.714858879	-0.764313713	3.823640452	-0.199891627	0.841565339	0.996944933
2.1.1.314	diphthine methyl ester synthase	1.408052998	0.648342336	3.771959295	0.171884765	0.863528124	0.996944933
2.1.1.206	tRNA (cytidine56-2'-O)-methyltransferase	0.478742931	1.360792032	4.113298126	0.330827475	0.740774806	0.996944933
1.1.1.291	2-hydroxymethylglutarate dehydrogenase	1.165624298	0.694626561	4.100960969	0.169381412	0.865496643	0.996944933
6.3.2.12	#N/A	407.8113641	0.023311186	0.198946143	0.117173347	0.906722687	0.996944933
2.8.1.12	molybdopterin synthase catalytic subunit	4.69347111	0.057444622	1.549665299	0.037069051	0.970429949	0.996944933
3.6.4.6	vesicle-fusing ATPase	4.693647225	0.589922229	2.053332082	0.287299962	0.773882652	0.996944933
1.21.4.1	#N/A	273.8860975	0.296927968	0.665259446	0.446334088	0.655355937	0.996944933
2.7.1.130	tetraacyldisaccharide 4'-kinase	131.049192	-0.480894581	0.494522387	-0.972442489	0.330830463	0.996944933
2.4.1.320	beta-1,4-mannooligosaccharide/beta-1,4-mannosyl-N-acetylglu	163.8803014	0.227289327	0.352917593	0.644029461	0.51955633	0.996944933

4.1.2.20	2-dehydro-3-deoxyglucarate aldolase	2.922842527	-0.74206413	1.455353823	-0.509885719	0.610131528	0.996944933
1.1.1.86	ketol-acid reductoisomerase	435.8011911	0.087152785	0.316229403	0.275599879	0.782855403	0.996944933
5.1.3.3	aldose 1-epimerase	759.9440507	-0.093129624	0.37329702	-0.249478617	0.802990579	0.996944933
5.3.1.28	D-sedoheptulose 7-phosphate isomerase	48.71117419	-0.29539671	0.447567236	-0.660005214	0.509250483	0.996944933
2.3.1.47	8-amino-7-oxononanoate synthase	86.46061388	-0.298924046	0.663882526	-0.450266477	0.652518308	0.996944933
2.4.99.13	3-deoxy-D-manno-octulosonic-acid transferase	104.6062867	-0.408074653	0.481327791	-0.847810288	0.396543632	0.996944933
3.2.1.170	mannosylglycerate hydrolase	9.078498944	0.471543211	1.493078563	0.315819424	0.752139574	0.996944933
3.4.13.9	Xaa-Pro dipeptidase	53.83327966	-0.475782733	0.612628744	-0.776624894	0.43738011	0.996944933
2.4.2.48	7-cyano-7-deazaguanine tRNA-ribosyltransferase	0.499547738	-0.30383844	4.118602018	-0.073772226	0.941191627	0.996944933
3.6.1.26	CDP-diacylglycerol pyrophosphatase	0.081334971	0.404882397	4.142921482	0.097728716	0.922147713	0.996944933
2.1.1.290	tRNA wybutosine-synthesizing protein 4	0.052512875	0.445935252	4.139416356	0.107729016	0.914210652	0.996944933
2.1.1.229	alkylated DNA repair protein alkB homolog 8	0.035008583	0.581845947	4.14032189	0.140531573	0.888240008	0.996944933
2.1.1.294	O-antigen chain-terminating methyltransferase	2.5617666	0.987094195	3.783248293	0.260911819	0.79416051	0.996944933
1.14.14.5	alkanesulfonate monooxygenase	0.151201469	0.110255777	4.137728292	0.026646452	0.978741723	0.996944933
1.1.1.41	isocitrate dehydrogenase (NAD+)	37.24360312	-0.43076269	0.417581882	-1.031564609	0.302276127	0.996944933
2.3.1.24	Acyl-CoA-dependent ceramide synthase	1.915657591	0.648118161	4.097793812	0.158162707	0.874328589	0.996944933
2.7.1.189	autoinducer-2 kinase	13.76266685	-0.864324714	0.922300311	-0.937140217	0.348686437	0.996944933
3.2.2.9	adenosylhomocysteine nucleosidase	243.1991387	-0.177186754	0.305956918	-0.579123213	0.562506038	0.996944933
2.8.3.1	propionate CoA-transferase	89.25752976	0.036913984	0.527992522	0.069913839	0.944262238	0.996944933
3.5.3.18	dimethylargininase	1.226516595	1.375834234	2.711468159	0.507413015	0.611865055	0.996944933
2.8.1.2	thiosulfate/3-mercaptopyruvate sulfurtransferase	7.144411477	-0.497714552	1.041825434	-0.477733155	0.632840143	0.996944933
2.1.3.-	#N/A	2.822043806	-1.634130877	2.587101387	-0.631645472	0.527618566	0.996944933
2.7.1.52	fucokinase	57.1794349	-0.140226829	0.655007548	-0.214084294	0.830481323	0.996944933
3.1.26.8	ribonuclease M5	72.41566023	0.286968102	0.357191172	0.803402	0.42174242	0.996944933
4.1.3.30	methylisocitrate lyase	0.651710156	-1.043688619	4.110637614	-0.253899448	0.799573242	0.996944933
3.5.1.24	choloylglycine hydrolase	410.6257429	-0.031802974	0.400228186	-0.079462104	0.936665073	0.996944933
2.1.1.35	tRNA (uracil-5-)-methyltransferase	0.644054713	-1.568630205	4.108412173	-0.381809356	0.702602779	0.996944933
2.5.1.29	geranylgeranyl diphosphate synthase, type III	364.885785	0.191996462	0.192452331	0.997631263	0.318458196	0.996944933
2.6.1.45	#N/A	1.130264311	1.265102956	3.58445464	0.352941544	0.724132265	0.996944933
1.3.1.93	very-long-chain enoyl-CoA reductase	0.999108385	0.822758958	4.10220278	0.20056516	0.841038603	0.996944933
1.1.1.-	#N/A	3841.379493	-0.048948388	0.786569603	-0.062230205	0.950379509	0.996944933
2.7.1.146	ADP-dependent phosphofructokinase/glucokinase	1.596352927	-0.341707144	2.972685745	-0.114948963	0.908485575	0.996944933
5.4.3.8	glutamate-1-semialdehyde 2,1-aminomutase	119.9576647	-0.333053169	0.425342751	-0.783023028	0.433613589	0.996944933
1.1.99.1	choline dehydrogenase	0.295593336	0.363585188	4.119804861	0.088253012	0.929675584	0.996944933
1.4.1.24	3-dehydroquinate synthase II	3.146725448	1.364607158	1.760869294	0.774962209	0.438361991	0.996944933
4.2.2.17	inulin fructotransferase (DFA-I-forming)	0.177059089	0.685352543	4.141271084	0.165493282	0.868555737	0.996944933
3.1.3.64	myotubularin	3.613934552	1.097599575	2.802727017	0.391618437	0.695340163	0.996944933
3.1.3.3	phosphoserine phosphatase	138.4424214	-0.433629175	0.402777333	-1.076597762	0.281660005	0.996944933
2.1.1.215	tRNA (guanine26-N2/guanine27-N2)-dimethyltransferase	2.22570527	1.137883436	3.267802974	0.348210539	0.727682073	0.996944933
2.6.1.51	alanine-glyoxylate transaminase / serine-glyoxylate transamina	1.130264311	1.265102956	3.58445464	0.352941544	0.724132265	0.996944933
1.2.4.4	3-methyl-2-oxobutanoate dehydrogenase (2-methylpropanoyl-	106.8812829	-0.045891101	0.544045587	-0.084351573	0.932776909	0.996944933
3.1.2.28	1,4-dihydroxy-2-naphthoyl-CoA hydrolase	0.020495196	0.766777793	4.142686886	0.185091902	0.853156953	0.996944933
2.7.1.203	PTS system, D-glucosaminate-specific IIA component	3.533453409	-0.360116365	1.284156965	-0.280430177	0.779147488	0.996944933
1.12.1.4	iron-hydrogenase subunit alpha	0.249773869	-0.154751873	4.134519116	-0.037429232	0.970142766	0.996944933
2.1.1.184	23S rRNA (adenine-N6)-dimethyltransferase	38.39874982	0.243138949	0.44665126	0.544359706	0.586193968	0.996944933
5.4.3.3	beta-lysine 5,6-aminomutase alpha subunit	144.6425607	0.523617455	0.484025789	1.081796605	0.279342915	0.996944933
2.7.1.39	homoserine kinase	148.0990502	0.251184045	0.29649025	0.847191584	0.396888344	0.996944933
1.3.1.87	2,3-dihydroxy-2,3-dihydrophenylpropionate dehydrogenase	0.111464417	1.106978919	4.143731151	0.267145449	0.789357176	0.996944933
2.4.1.142	beta-1,4-mannosyltransferase	2.114580348	0.706130844	4.091108479	0.172601349	0.862964791	0.996944933
2.4.1.258	alpha-1,3-mannosyltransferase	0.723054713	-0.055078595	4.109010393	-0.013404345	0.9893052	0.996944933
2.1.1.282	tRNA wybutosine-synthesizing protein 3	0.510438089	0.806539946	4.11088144	0.196196353	0.844456482	0.996944933
4.-.-.-	#N/A	0.043561417	1.106985704	4.143731151	0.267147086	0.789355915	0.996944933
4.1.99.3	deoxyribodipyrimidine photo-lyase	0.334769309	-0.308690841	4.117593106	-0.074968758	0.940239569	0.996944933
2.7.1.43	glucuronokinase	1.764226465	0.498485962	4.098222264	0.121634682	0.903188345	0.996944933
1.1.1.157	3-hydroxybutyryl-CoA dehydrogenase	1028.321371	0.517810005	0.558275178	0.927517514	0.353657903	0.996944933
1.1.1.369	myo-inositol 2-dehydrogenase / D-chiro-inositol 1-dehydrogena	75.32692191	-0.366770634	0.488048301	-0.751504786	0.452348922	0.996944933
1.1.1.34	hydroxymethylglutaryl-CoA reductase (NADPH)	5.653749803	0.865914109	2.571988317	0.33667109	0.736364859	0.996944933
2.1.1.86	#N/A	31.96680807	1.114394635	1.680182655	0.663258028	0.507165313	0.996944933
1.14.12.17	nitric oxide dioxygenase	9.04465669	0.8788886	1.057471361	0.83112284	0.405904238	0.996944933
2.7.8.-	#N/A	803.4885897	-0.049486676	0.26095894	-0.189633956	0.849595977	0.996944933
3.1.5.1	dGTPase	378.9543563	-0.258048288	0.246567032	-1.04656444	0.29530051	0.996944933
3.4.24.-	#N/A	2958.235347	0.164540768	0.175765251	0.93613935	0.349201445	0.996944933
2.1.2.13	UDP-4-amino-4-deoxy-L-arabinose formyltransferase / UDP-glu	1.285501156	-1.630532084	2.077069673	-0.785015595	0.432444425	0.996944933
3.1.3.24	sucrose-6-phosphatase	973.9245196	0.056362131	0.334388996	0.168552588	0.866148576	0.996944933
2.4.1.241	digalactosyldiacylglycerol synthase	0.104075673	1.817862207	4.143245313	0.438753216	0.660840362	0.996944933
2.5.1.56	N-acetylneuraminate synthase	25.93261642	0.456906175	0.661241048	0.690982776	0.489576365	0.996944933
2.3.1.117	2,3,4,5-tetrahydropyridine-2,6-dicarboxylate N-succinyltransfer	34.45067098	0.17696894	0.576059587	0.307205962	0.758686608	0.996944933
2.3.2.-	#N/A	18.67247501	0.740701523	1.076096327	0.688322694	0.491249594	0.996944933
2.4.1.46	1,2-diacylglycerol 3-beta-galactosyltransferase	5.249527659	-0.032941129	4.020220917	-0.008193861	0.993462318	0.996944933
1.13.11.15	3,4-dihydroxyphenylacetate 2,3-dioxygenase	0.110533983	0.351083501	4.142162324	0.084758509	0.93245338	0.996944933
2.6.1.87	UDP-4-amino-4-deoxy-L-arabinose-oxoglutarate aminotransfera	0.040990391	0.558766837	4.140771598	0.134942685	0.892657191	0.996944933
4.1.2.57	sulfofructosephosphate aldolase	0.103724382	1.1069846	4.143731151	0.26714682	0.78935612	0.996944933
5.99.1.-	#N/A	287.1172249	0.157338056	0.345720977	0.455101271	0.649036384	0.996944933
3.6.1.67	dihydroneopterin triphosphate diphosphatase	2.254603037	0.494380618	1.574475164	0.313997089	0.75352325	0.996944933
2.2.1.2	transaldolase	460.0355829	0.020853573	0.604733669	0.034483896	0.972491284	0.996944933
4.3.1.7	#N/A	47.02469326	-0.386328831	0.482414188	-0.800823942	0.423233577	0.996944933
2.2.1.9	2-succinyl-5-enolpyruvyl-6-hydroxy-3-cyclohexene-1-carboxylat	95.19445505	-0.099470118	0.358993825	-0.277080304	0.781718445	0.996944933
3.2.1.165	exo-1,4-beta-D-glucosaminidase	2.50673581	-0.55020712	2.205993261	-0.249414688	0.803040025	0.996944933
1.1.1.18	myo-inositol 2-dehydrogenase / D-chiro-inositol 1-dehydrogena	75.32692191	-0.366770634	0.488048301	-0.751504786	0.452348922	0.996944933
5.2.1.8	peptidylprolyl isomerase	2610.435006	0.406071691	0.412296806	0.984901375	0.324672518	0.996944933
2.6.1.13	ornithine--oxo-acid transaminase	35.59584511	0.234686686	0.355959402	0.659307452	0.509698358	0.996944933
1.10.3.10	#N/A	9.229660608	-1.371877916	1.730507177	-0.792760605	0.427917313	0.996944933
6.4.1.1	pyruvate carboxylase	480.0100183	0.211322036	0.37038899	0.570540814	0.568310948	0.996944933
2.7.2.4	aspartate kinase	755.1354989	0.088000569	0.370166635	0.23773231	0.812088722	0.996944933
2.3.1.23	lysophosphatidylcholine acyltransferase / lyso-PAF acetyltransf	1.237997841	1.080145526	4.100271928	0.263432657	0.792217123	0.996944933
2.3.2.23	ubiquitin-conjugating enzyme E2 R	9.282151261	0.744706207	4.093881017	0.181907145	0.855655597	0.996944933
2.7.1.1	hexokinase	23.12651405	0.058301285	0.791720276	0.073638741	0.941297844	0.996944933

5.1.3.1	ribulose-phosphate 3-epimerase	405.4522483	0.201417633	0.332288874	0.606152202	0.544413689	0.996944933
1.1.1.302	2,5-diamino-6-(ribosylamino)-4(3H)-pyrimidinone 5'-phosphate	0.582805694	-0.313605345	4.114963576	-0.076210965	0.939251259	0.996944933
3.1.1.-	#N/A	541.0085476	0.035933792	0.304175638	0.11813501	0.905960684	0.996944933
1.6.99.3	NADH dehydrogenase	195.4290965	-0.396835603	0.680906537	-0.582804807	0.560024708	0.996944933
1.7.99.4	#N/A	62.74469958	0.622653204	0.582478909	1.068971243	0.285082627	0.996944933
3.5.4.31	5-methylthioadenosine/S-adenosylhomocysteine deaminase	646.4515798	-0.179328032	0.774803878	-0.23144958	0.816965551	0.996944933
3.1.3.90	maltose 6'-phosphate phosphatase	7.623733685	0.272472224	0.721799071	0.377490405	0.705809194	0.996944933
4.1.1.15	glutamate decarboxylase	198.5891402	-0.192532815	0.671913858	-0.286543896	0.774461578	0.996944933
4.1.1.5	acetolactate decarboxylase	2.950417394	-1.268897796	2.041301007	-0.621612291	0.534196837	0.996944933
2.1.1.190	23S rRNA (uracil1939-C5)-methyltransferase	470.4162853	0.128468505	0.294602484	0.436074072	0.662782988	0.996944933
3.1.26.-	#N/A	361.3170482	-0.017169105	0.215264527	-0.079758169	0.936429595	0.996944933
3.2.1.156	oligosaccharide reducing-end xylanase	47.24343399	0.404462048	0.699150248	0.578505192	0.562923093	0.996944933
2.7.11.5	isocitrate dehydrogenase kinase/phosphatase	0.941316252	-1.00209026	4.098582479	-0.24449679	0.806846088	0.996944933
2.1.2.1	glycine hydroxymethyltransferase	400.2794024	0.231029404	0.232660667	0.992988661	0.32071548	0.996944933
2.6.1.44	alanine-glyoxylate transaminase / (R)-3-amino-2-methylpropior	1.130264311	1.265102956	3.58445464	0.352941544	0.724132265	0.996944933
4.2.3.12	6-pyruvoyltetrahydropterin/6-carboxytetrahydropterin synthas	143.680634	0.303294197	0.407197221	0.744833661	0.456372283	0.996944933
3.6.4.13	ATP-dependent helicase HrpA	1266.701025	0.280887122	0.431186572	0.651428268	0.514770068	0.996944933
4.2.1.83	4-oxalmesaconate hydratase	0.709423283	-1.941209051	3.864105194	-0.502369618	0.615407546	0.996944933
2.3.2.27	protein neuralized	11.57495195	0.480934749	4.093710844	0.117481368	0.906478608	0.996944933
1.6.99.1	NADPH2 dehydrogenase	0.530483847	-1.101706156	4.118769147	-0.267484318	0.789096287	0.996944933
2.5.1.87	ditrans,polycis-polyprenyl diphosphate synthase	0.052512875	0.445935252	4.139416356	0.107729016	0.914210652	0.996944933
3.2.2.1	purine nucleosidase	27.4703256	-0.259028665	0.606091756	-0.427375331	0.669105973	0.996944933
2.1.1.192	23S rRNA (adenine2503-C2)-methyltransferase	509.0918047	-0.056079527	0.205245141	-0.273231937	0.784674932	0.996944933
1.4.1.4	glutamate dehydrogenase (NADP+)	1096.26824	0.329195365	0.310100504	1.061576362	0.28842805	0.996944933
2.7.1.58	2-dehydro-3-deoxygalactonokinase	5.245567172	-0.513640298	1.398051373	-0.367397298	0.713322686	0.996944933
2.5.1.77	#N/A	1.536021083	0.853024263	4.098810969	0.208115053	0.835139134	0.996944933
5.3.1.31	sulfoquinovose isomerase	0.13101338	0.376614129	4.138534189	0.091001816	0.927491148	0.996944933
2.7.4.24	inositol-hexakisphosphate/diphosphoinositol-pentakisphospha	4.421491168	1.465776713	2.741763836	0.534610856	0.592918975	0.996944933
4.2.99.18	DNA-(apurinic or apyrimidinic site) lyase	479.2168979	-0.417345923	0.437309936	-0.954348139	0.339907448	0.996944933
3.2.1.172	unsaturated rhamnogalacturonyl hydrolase	155.0754149	0.026176054	0.465304808	0.056255714	0.955138098	0.996944933
2.4.2.8	hypoxanthine phosphoribosyltransferase	321.3459054	-0.096293814	0.256530005	-0.375370568	0.707384891	0.996944933
3.2.1.37	xylan 1,4-beta-xylosidase	187.3640125	0.098693636	0.574324651	0.171842939	0.863561007	0.996944933
1.1.1.307	D-xylose reductase	0.179515503	0.542400451	4.13816919	0.131072565	0.895717904	0.996944933
1.1.1.219	dihydroflavonol-4-reductase	15.39927764	0.794414824	0.914298807	0.868878771	0.384913442	0.996944933
3.6.3.35	manganese/zinc transport system ATP-binding protein	2.734835095	1.782360443	1.968176068	0.905589938	0.365152937	0.996944933
1.7.2.4	nitrous-oxide reductase	0.408715984	0.202999106	4.118314365	0.049291795	0.960686759	0.996944933
2.1.1.202	multisite-specific tRNA:(cytosine-C5)-methyltransferase	0.206248383	1.476565847	4.134230176	0.357156178	0.72097489	0.996944933
1.7.7.1	ferredoxin-nitrite reductase	24.30469765	0.289944163	0.575457841	0.50384953	0.614367119	0.996944933
2.4.1.227	UDP-N-acetylglucosamine--N-acetylmuramyl-(pentapeptide) py	364.4604836	0.023519391	0.154453579	0.152274818	0.878970185	0.996944933
2.6.1.100	L-glutamine:2-deoxy-scyllo-inosose/3-amino-2,3-dideoxy-scyllo	0.025751485	0.746325135	4.143731151	0.180109449	0.857066645	0.996944933
2.4.2.21	nicotinate-nucleotide--dimethylbenzimidazole phosphoribosylt	143.4673169	-0.198671621	0.298808817	-0.66487871	0.506128074	0.996944933
4.2.1.147	5,6,7,8-tetrahydromethanopterin hydro-lyase	3.430205491	1.290729048	3.054234091	0.422603183	0.672584804	0.996944933
2.1.1.223	tRNA1Val (adenine37-N6)-methyltransferase	87.6555404	0.236603601	0.569966628	0.41511834	0.678055274	0.996944933
4.1.1.39	#N/A	0.674912036	-0.372258074	4.105618648	-0.090670397	0.927754493	0.996944933
2.5.1.105	7,8-dihydropterin-6-yl-methyl-4-(beta-D-ribofuranosyl)aminobe	39.00221093	-0.133242533	0.46273627	-0.287944866	0.773388942	0.996944933
1.17.4.1	ribonucleotide reductase, class II	441.4559732	-0.02209805	0.399304825	-0.055341304	0.955866557	0.996944933
6.3.2.9	UDP-N-acetylmuramoylalanine--D-glutamate ligase	401.0932987	-0.221671325	0.278627352	-0.795583502	0.426274159	0.996944933
6.3.2.17	folylpolyglutamate synthase	408.1969088	0.020335466	0.198017732	0.102695177	0.918204901	0.996944933
2.3.3.10	hydroxymethylglutaryl-CoA synthase	5.711122078	0.423021869	1.369084256	0.308981618	0.75733551	0.996944933
2.3.3.15	sulfoacetaldehyde acetyltransferase	1.166067547	0.305999952	4.136349042	0.073978271	0.941027675	0.996944933
2.7.2.1	acetate kinase	2424.083986	-0.606667582	0.785153083	-0.772674266	0.43971518	0.996944933
2.7.2.-	#N/A	0.770139323	0.167355899	4.106472412	0.040754176	0.967491871	0.996944933
1.13.11.49	chlorite dismutase	0.051862191	1.106985684	4.143731151	0.267147082	0.789355919	0.996944933
3.6.4.3	#N/A	2.836180328	-0.186600946	3.091521614	-0.060358933	0.951869766	0.996944933
2.6.1.98	UDP-2-acetamido-2-deoxy-ribo-hexuluronate aminotransferase	0.251634167	0.273238824	4.135429191	0.066072664	0.947319974	0.996944933
1.1.1.4	(R,R)-butanediol dehydrogenase / meso-butanediol dehydroger	2.80919775	0.883083891	1.538629634	0.573941819	0.566007173	0.996944933
1.8.1.4	dihydrolipoamide dehydrogenase	317.743301	0.167072059	0.440169143	0.379563316	0.704269596	0.996944933
1.1.1.305	UDP-4-amino-4-deoxy-L-arabinose formyltransferase / UDP-glu	1.285501156	-1.630532084	2.077069673	-0.785015595	0.432444425	0.996944933
5.4.99.19	16S rRNA pseudouridine516 synthase	318.1196796	0.009596146	0.498744351	0.019240612	0.98464916	0.996944933
2.7.1.68	1-phosphatidylinositol-4-phosphate 5-kinase	1.131986754	0.079856992	4.102712808	0.019464436	0.984470607	0.996944933
1.1.1.60	2-hydroxy-3-oxopropionate reductase	9.559379802	-0.140906083	0.75257854	-0.187231067	0.851479467	0.996944933
2.7.1.198	PTS system, glucitol/sorbitol-specific IIA component	10.56111943	-0.668770206	0.749485669	-0.892305528	0.372229192	0.996944933
3.4.21.116	stage IV sporulation protein B	186.7204116	-0.529447177	0.664502052	-0.796757777	0.425591721	0.996944933
1.6.2.2	cytochrome-b5 reductase	0.078987483	1.060294701	4.14177237	0.256000235	0.797950656	0.996944933
1.2.1.89	D-glyceraldehyde dehydrogenase (NADP+)	0.103005941	0.222138118	4.137063428	0.053694637	0.957178456	0.996944933
1.1.1.136	UDP-N-acetyl-D-glucosamine dehydrogenase	13.44204196	0.590623432	0.681191642	0.867044449	0.385917656	0.996944933
2.5.1.128	N4-bis(aminopropyl)spermidine synthase	4.506886474	1.174090566	3.23910143	0.362474159	0.716997731	0.996944933
3.2.1.40	alpha-L-rhamnosidase	377.6323579	-0.46543004	0.482296089	-0.96502968	0.334529946	0.996944933
1.1.1.381	3-hydroxy acid dehydrogenase / malonic semialdehyde reducta	0.577711424	-1.193643837	4.113596979	-0.290170341	0.771685925	0.996944933
5.1.99.4	alpha-methylacyl-CoA racemase	1.348104808	0.759008781	3.512529221	0.216086111	0.828920625	0.996944933
3.5.3.7	guanidinobutyrase	0.574681685	1.820299608	4.117017004	0.442140415	0.6583876	0.996944933
6.3.4.20	7-cyano-7-deazaguanine synthase	94.67236829	-0.089218166	0.551393582	-0.161804869	0.871459519	0.996944933
6.3.2.14	bifunctional isochorismate lyase / aryl carrier protein	4.385493731	0.460262907	2.101493513	0.219017049	0.826636767	0.996944933
1.4.3.19	glycine oxidase	0.103005941	0.222138118	4.137063428	0.053694637	0.957178456	0.996944933
3.1.11.2	exodeoxyribonuclease III	258.3334323	-0.223987156	0.303964721	-0.736885368	0.461192063	0.996944933
2.1.1.33	tRNA (guanine-N7-)-methyltransferase	245.3849363	0.174550234	0.30171124	0.57853408	0.562903595	0.996944933
1.12.5.1	#N/A	0.276334958	-0.096838687	4.123302027	-0.023485713	0.981262835	0.996944933
3.5.1.53	N-carbamoylputrescine amidase	105.5660389	-0.306499575	0.38258627	-0.801125392	0.423059058	0.996944933
1.9.3.1	#N/A	0.209529612	1.106972556	4.143731151	0.267143914	0.789358358	0.996944933
3.2.1.24	alpha-mannosidase	269.881228	-0.45802382	0.447485908	-1.02354915	0.306048274	0.996944933
3.6.3.3	#N/A	575.5346922	0.045928947	0.278533887	0.164895365	0.869026341	0.996944933
2.1.1.100	protein-S-isoprenylcysteine O-methyltransferase	0.166515913	0.190402508	4.138090197	0.04601217	0.96330055	0.996944933
1.2.7.3	#N/A	1254.655337	0.267402097	0.302837777	0.882987914	0.377242802	0.996944933
2.7.8.38	archaetidylserine synthase	0.293518541	0.194678154	4.132957774	0.047103833	0.962430472	0.996944933
2.4.2.1	#N/A	957.9806977	0.299366166	0.603499253	0.496050599	0.619858712	0.996944933
2.7.1.73	inosine kinase	0.730107326	-1.921800872	4.105034615	-0.468157044	0.639672288	0.996944933
4.1.1.25	tyrosine decarboxylase	0.874214996	0.432778868	4.104167584	0.105448635	0.916019826	0.996944933

3.2.1.54	cyclomaltodextrinase / maltogenic alpha-amylase / neopullulan	0.30127684	-0.681748901	4.12669228	-0.165204686	0.868782877	0.996944933
2.3.1.242	KDO2-lipid IV(A) palmitoleoyltransferase	3.363987485	-0.600409685	2.031566564	-0.295540247	0.767581212	0.996944933
6.3.1.2	glutamine synthetase	935.7833556	-0.158219867	0.220326564	-0.718115256	0.472686224	0.996944933
3.6.3.30	iron(III) transport system ATP-binding protein	97.39821977	0.161363719	0.397007025	0.406450538	0.684411593	0.996944933
6.3.2.3	glutathione synthase	1.801797141	-1.664561499	1.928968462	-0.862928312	0.388176883	0.996944933
6.3.2.39	aerobactin synthase	2.405771588	0.483926296	4.096819821	0.118122426	0.905970655	0.996944933
3.5.1.82	N-acyl-D-glutamate deacylase	0.439714584	1.118772904	4.123577649	0.271311225	0.786151674	0.996944933
2.7.1.60	N-acylmannosamine kinase	0.061485587	0.410222495	4.139390501	0.099102149	0.921057166	0.996944933
1.8.5.4	sulfide:quinone oxidoreductase	3.822115518	-2.017406389	2.064298599	-0.977284192	0.328428477	0.996944933
2.1.1.224	23S rRNA (adenine-C8)-methyltransferase	4.139167645	0.298107858	3.305462228	0.090186436	0.928139063	0.996944933
6.2.1.30	phenylacetate-CoA ligase	732.7612545	0.658513552	0.590141122	1.115857763	0.264483024	0.996944933
1.1.1.163	cyclopentanol dehydrogenase	0.213055426	1.090984828	4.142999974	0.263332087	0.792294631	0.996944933
3.4.21.88	repressor LexA	498.3446664	0.162805169	0.37261059	0.436931138	0.662161289	0.996944933
2.7.1.89	thiamine kinase	0.567411961	-0.842721551	4.118730056	-0.204607134	0.837879082	0.996944933
3.6.1.23	dUTP pyrophosphatase	266.0926212	-0.033510544	0.276382732	-0.121246882	0.903495492	0.996944933
3.5.1.19	nicotinamidase	5.650251342	0.200216798	1.280242155	0.156389787	0.875725787	0.996944933
2.4.1.12	cellulose synthase (UDP-forming)	8.384802002	0.931801871	1.042441709	0.893864725	0.371394273	0.996944933
3.1.21.3	type I restriction enzyme, R subunit	1199.129253	-0.140598437	0.253651484	-0.554297711	0.579375117	0.996944933
2.1.1.189	23S rRNA (uracil747-C5)-methyltransferase	0.50752299	-1.009229728	4.119077604	-0.245013526	0.806445958	0.996944933
4.2.1.68	L-fuconate dehydratase	0.087521458	0.256056607	4.138412209	0.061873152	0.950663848	0.996944933
1.10.9.1	#N/A	0.084668419	1.060899532	4.141794518	0.256144898	0.797838955	0.996944933
1.3.8.4	isovaleryl-CoA dehydrogenase	0.545047208	-1.281115259	4.117602288	-0.311131374	0.755700751	0.996944933
2.1.1.163	demethylmenaquinone methyltransferase / 2-methoxy-6-polyp	61.69477923	-0.473710915	0.474794648	-0.997717469	0.31841638	0.996944933
3.4.23.-	#N/A	121.6829844	-0.374762496	0.5490726	-0.682537238	0.494899304	0.996944933
6.5.1.-	#N/A	117.6895471	0.678598027	0.796391744	0.852090735	0.394163734	0.996944933
6.3.1.11	gamma-glutamylputrescine synthase	0.102475978	0.198319299	4.137494487	0.047932221	0.96177026	0.996944933
1.5.5.-	#N/A	3.832395075	-1.194585797	1.747682807	-0.683525518	0.494274831	0.996944933
1.1.1.298	malonyl-CoA reductase / 3-hydroxypropionate dehydrogenase	0.201546003	-0.483000626	4.130495086	-0.116935286	0.906911335	0.996944933
2.7.1.72	streptomycin 6-kinase	0.20799384	1.010168611	4.139828872	0.244012166	0.807221397	0.996944933
5.4.99.28	tRNA pseudouridine32 synthase / 23S rRNA pseudouridine746 s	66.23259907	-0.443322393	0.581020672	-0.763006231	0.44545968	0.996944933
2.7.1.156	adenosylcobinamide kinase / adenosylcobinamide-phosphate g	139.991378	-0.044366751	0.41359757	-0.107270338	0.914574516	0.996944933
2.3.1.18	galactoside O-acetyltransferase	43.53976159	-0.627675945	0.82065567	-0.7648469	0.444362717	0.996944933
4.99.1.4	uroporphyrin-III C-methyltransferase / precorrin-2 dehydrogena	30.31722373	-0.707982086	0.853762266	-0.82924968	0.406963138	0.996944933
3.2.1.97	endo-alpha-N-acetylgalactosaminidase	605.1706156	-0.066416293	0.750020719	-0.088552612	0.929437471	0.996944933
3.1.3.95	myotubularin	3.613934552	1.097599575	2.802727017	0.391618437	0.695340163	0.996944933
3.6.1.5	apyrase	0.249773869	-0.154751873	4.134519116	-0.037429232	0.970142766	0.996944933
1.2.99.7	aldehyde oxidoreductase	192.6359077	0.586252141	0.561629082	1.043842209	0.2965584	0.996944933
1.3.7.11	digeranylgeranylglycerophospholipid reductase	2.870100187	0.901968969	3.909691541	0.230700801	0.817547251	0.996944933
2.7.1.169	pantoate kinase	0.104075673	1.817862207	4.143245313	0.438753216	0.660840362	0.996944933
6.1.1.12	aspartyl-tRNA synthetase	692.5248136	-0.172145153	0.300919431	-0.572063934	0.567278662	0.996944933
3.5.1.3	omega-amidase	2.642522728	0.716465025	3.821712303	0.187472255	0.851290375	0.996944933
2.1.1.264	23S rRNA (guanine2445-N2)-methyltransferase / 23S rRNA (gua	1.313961173	-2.335315099	2.501144283	-0.933698673	0.35045935	0.996944933
1.1.1.282	quinate/shikimate dehydrogenase	0.322402874	-0.070379043	4.127234919	-0.017052347	0.986394855	0.996944933
3.1.7.2	GTP diphosphokinase / guanosine-3',5'-bis(diphosphate) 3'-diph	5.410257227	-0.65461572	1.581293658	-0.413974796	0.678892565	0.996944933
3.5.4.10	phosphoribosylaminoimidazolecarboxamide formyltransferase	546.3313652	0.19028938	0.184751179	1.029976537	0.30302102	0.996944933
2.4.1.319	beta-1,4-mannooligosaccharide/beta-1,4-mannosyl-N-acetylglu	163.8803014	0.227289327	0.352917593	0.644029461	0.51955633	0.996944933
3.6.3.38	capsular polysaccharide transport system ATP-binding protein	0.528533578	-0.305606755	4.117970203	-0.074212959	0.940840934	0.996944933
5.2.1.1	maleate isomerase	0.8515885	-0.650936375	4.099837302	-0.15877127	0.873849085	0.996944933
2.7.7.62	adenosylcobinamide kinase / adenosylcobinamide-phosphate g	139.991378	-0.044366751	0.41359757	-0.107270338	0.914574516	0.996944933
1.1.1.320	benzil reductase ((S)-benzoin forming)	0.13068425	1.106984261	4.143731151	0.267146738	0.789356183	0.996944933
2.1.1.265	tellurite methyltransferase	1.655407417	0.090621028	1.962726513	0.046170991	0.963173964	0.996944933
3.5.1.25	N-acetylglucosamine-6-phosphate deacetylase	970.9548437	0.277833298	0.47965942	0.579230358	0.562433749	0.996944933
3.4.24.55	protease III	0.658608599	-0.316241461	4.117790086	-0.07679883	0.938783582	0.996944933
2.1.1.219	tRNA (adenine57-N1/adenine58-N1)-methyltransferase catalyti	3.95831283	0.017198175	1.726325169	0.009962304	0.992051363	0.996944933
1.1.5.4	malate dehydrogenase (quinone)	1.565747164	-2.018211076	2.613791065	-0.772139404	0.440031866	0.996944933
1.7.2.1	nitrite reductase (NO-forming)	0.040667486	0.746328438	4.143731151	0.180110246	0.857066019	0.996944933
4.1.1.47	tartronate-semialdehyde synthase	1.982359021	1.632040559	2.183464162	0.747454704	0.454789135	0.996944933
2.1.2.10	aminomethyltransferase	191.58773	0.174685607	0.345353089	0.505817415	0.612984832	0.996944933
2.7.8.1	ethanolaminephosphotransferase	1.503869701	0.354412402	4.099608443	0.086450305	0.931108459	0.996944933
2.7.1.40	pyruvate kinase	528.5119295	0.101976952	0.18968605	0.53760913	0.590846926	0.996944933
3.4.23.1	pepsin A	0.078987483	1.060294701	4.14177237	0.256000235	0.797950656	0.996944933
6.1.1.21	#N/A	372.2009374	0.151644929	0.181995253	0.833235631	0.404711851	0.996944933
1.1.1.11	D-arabinitol 4-dehydrogenase	0.221067967	-0.057988387	4.130271841	-0.014039848	0.98879819	0.996944933
6.3.2.13	UDP-N-acetylmuramoyl-L-alanyl-D-glutamate--2,6-diaminopime	496.0040111	0.160100297	0.184987791	0.865464127	0.386784097	0.996944933
3.2.2.24	ADP-ribosyl-[dinitrogen reductase] hydrolase	2.896195258	1.212985283	3.115460135	0.389343863	0.697021793	0.996944933
5.-.-.-	#N/A	559.3196393	0.45590119	0.739346681	0.616627086	0.537480725	0.996944933
3.2.2.28	double-stranded uracil-DNA glycosylase	1.308970377	0.367891837	4.074434575	0.090292734	0.928054594	0.996944933
2.7.1.4	fructokinase	457.5016156	0.254332603	0.287725783	0.883940953	0.376728088	0.996944933
1.11.1.-	#N/A	3.659730758	0.033135974	2.162983845	0.015319566	0.987777233	0.996944933
3.4.21.81	streptogrisin B	8.877711127	2.330427714	2.203718264	1.057498026	0.290284355	0.996944933
4.4.1.1	cystathionine gamma-lyase	3.136372628	-2.352483698	2.504636806	-0.939251428	0.34760167	0.996944933
1.2.1.87	propionaldehyde dehydrogenase	223.0956313	-0.033547713	0.647046522	-0.051847452	0.958650245	0.996944933
4.4.1.14	1-aminocyclopropane-1-carboxylate synthase	3.109366266	0.559362336	2.998889621	0.186523149	0.852034527	0.996944933
1.3.1.2	dihydropyrimidine dehydrogenase (NADP+)	0.077254456	0.362469253	4.139694903	0.087559412	0.930226863	0.996944933
3.10.1.1	N-sulfoglucosamine sulfohydrolase	0.500048027	-0.363476341	4.108939735	-0.088459886	0.929511165	0.996944933
2.7.1.51	L-fuculokinase	17.52662205	-1.055618206	1.072119088	-0.984609096	0.32481612	0.996944933
4.1.3.44	tRNA wybutosine-synthesizing protein 1	3.850197146	0.104056436	2.908505699	0.035776597	0.971460494	0.996944933
1.1.1.30	3-hydroxybutyrate dehydrogenase	0.712468766	-2.279168624	3.837729809	-0.593884598	0.552589301	0.996944933
5.4.99.20	23S rRNA pseudouridine2457 synthase	2.784565633	-1.163852227	1.903154292	-0.611538556	0.540843101	0.996944933
2.4.99.16	starch synthase (maltosyl-transferring)	1.915180048	-1.714640459	3.633791611	-0.471859876	0.637026809	0.996944933
2.7.4.28	[pyruvate, water dikinase]-phosphate phosphotransferase / [py	9.875085199	-0.386454662	0.700210118	-0.551912422	0.581008357	0.996944933
3.4.23.51	hydrogenase 3 maturation protease	1.085645249	-1.032278461	3.538756452	-0.291706557	0.770510995	0.996944933
3.2.2.3	uridine nucleosidase	0.052512875	0.445935252	4.139416356	0.107729016	0.914210652	0.996944933
1.6.2.4	NADPH-ferrihemoprotein reductase	1.311335403	1.123984874	4.100114104	0.274135023	0.78398086	0.996944933
6.6.1.1	magnesium chelatase subunit H	18.6825575	0.176625569	0.702378094	0.251467935	0.80145235	0.996944933
1.1.1.102	3-dehydrosphinganine reductase	1.670128019	1.557528291	4.099141324	0.379964526	0.703971748	0.996944933
1.1.1.36	acetoacetyl-CoA reductase	0.578478959	-1.795978392	4.0971542	-0.438347766	0.661134205	0.996944933

1.1.1.79	glyoxylate/hydroxypyruvate reductase	10.11112133	-0.126168055	1.898306938	-0.066463464	0.947008845	0.996944933
6.3.2.34	coenzyme F420-0:L-glutamate ligase / coenzyme F420-1:gamm	1.257810662	0.298530367	4.080429156	0.073161512	0.941677593	0.996944933
1.1.1.374	UDP-N-acetylglucosamine 3-dehydrogenase	1.956313881	0.946853393	4.095904567	0.231170765	0.817182141	0.996944933
3.2.1.58	glucan 1,3-beta-glucosidase	1.646498268	-2.566793198	2.422093112	-1.059741752	0.289262103	0.996944933
1.1.1.81	glyoxylate/hydroxypyruvate reductase	52.56367318	0.242868209	0.436108802	0.556898206	0.577596981	0.996944933
2.7.11.25	mitogen-activated protein kinase kinase kinase 8	0.495257723	0.289560935	4.114933348	0.070368317	0.943900508	0.996944933
2.1.1.63	methylated-DNA-protein]-cysteine S-methyltransferase	165.7113079	-0.37285837	0.345636452	-1.078758817	0.280695258	0.996944933
4.2.99.21	salicylate synthetase	0.575016627	-0.757945068	4.120846178	-0.183929473	0.854068783	0.996944933
2.4.1.288	galactofuranosylgalactofuranosylrhamnosyl-N-acetylglucosami	0.870667307	-1.330651387	4.100803568	-0.324485522	0.745570482	0.996944933
2.7.7.39	glycerol-3-phosphate cytidylyltransferase	10.10101612	-0.96202085	1.129005444	-0.852095847	0.394160897	0.996944933
4.2.2.10	pectin lyase	10.9423628	0.138033362	1.195345157	0.115475736	0.908068051	0.996944933
1.3.5.1	#N/A	476.3325581	0.056871105	0.335245118	0.169640367	0.865292975	0.996944933
4.2.1.6	galactonate dehydratase	37.59083693	0.496140491	0.459354278	1.080082444	0.280105469	0.996944933
4.3.2.1	argininosuccinate lyase	276.1844737	-0.10622698	0.197942163	-0.536656661	0.591504797	0.996944933
6.1.2.1	D-alanine---(R)-lactate ligase	0.017504292	0.778777863	4.141980666	0.188020642	0.85086047	0.996944933
2.2.1.6	#N/A	949.9615364	-0.136519012	0.209419791	-0.651891647	0.514471074	0.996944933
2.3.1.102	acetyl CoA:N6-hydroxylysine acetyl transferase	1.45851818	0.617063429	4.100508504	0.150484612	0.880382291	0.996944933
2.6.1.17	acetylornithine/N-succinyldiaminopimelate aminotransferase	710.8284773	0.217786828	0.466951407	0.466401482	0.640928151	0.996944933
2.1.1.321	type III protein arginine methyltransferase	0.500958201	-0.047602025	4.116246476	-0.011564425	0.990773129	0.996944933
3.4.24.15	thimet oligopeptidase	1.404001712	1.044317682	3.676970638	0.284015779	0.776398288	0.996944933
3.1.1.31	6-phosphogluconolactonase	371.1096164	0.180905374	0.54927539	0.329352775	0.741889059	0.996944933
3.6.1.31	phosphoribosyl-ATP pyrophosphohydrolase	275.8587485	0.071565007	0.22257179	0.321536737	0.747803677	0.996944933
1.20.4.1	arsenate reductase (glutaredoxin)	92.4590067	-0.367287964	0.459988535	-0.798471997	0.424596637	0.996944933
2.7.7.60	2-C-methyl-D-erythritol 4-phosphate cytidylyltransferase	341.3139113	-0.166229088	0.232340094	-0.715455887	0.474327389	0.996944933
2.7.7.3	pantetheine-phosphate adenylyltransferase	410.527713	0.008883883	0.35474272	0.025043171	0.980020529	0.996944933
2.1.3.6	putrescine carbamoyltransferase	5.844709524	0.050294687	1.280192221	0.039286824	0.968661711	0.996944933
2.4.1.265	alpha-1,3-glucosyltransferase	0.122530041	0.115004769	4.137516692	0.027795602	0.977825173	0.996944933
3.5.4.-	#N/A	0.035008583	0.581845947	4.14032189	0.140531573	0.888240008	0.996944933
1.1.1.337	L-2-hydroxycarboxylate dehydrogenase (NAD+)	2.293813537	0.569759009	3.263803185	0.174569046	0.861418273	0.996944933
3.2.1.143	poly(ADP-ribose) glycohydrolase	0.208151345	2.317132913	4.135630105	0.560285338	0.575284826	0.996944933
1.1.1.42	isocitrate dehydrogenase	498.3051866	0.290582418	0.396855298	0.73221252	0.464038864	0.996944933
4.2.1.106	bile-acid 7alpha-dehydratase	5.369375598	0.028482317	1.1709784	0.02432352	0.980594552	0.996944933
2.8.3.9	acetate CoA/acetoacetate CoA-transferase alpha subunit	181.9968591	0.505438358	0.541589068	0.933250665	0.350690561	0.996944933
2.3.1.225	palmitoyltransferase ZDHHC9/14/18	2.026598504	0.585080657	4.097623235	0.142785372	0.88645969	0.996944933
4.1.1.1	pyruvate decarboxylase	0.143466369	0.046079892	4.136114884	0.011140864	0.991111061	0.996944933
3.6.4.7	peroxisome-assembly ATPase	9.240900872	0.423282309	4.093937872	0.10339246	0.917651496	0.996944933
1.14.-.-	#N/A	8.314588655	0.352137125	1.035084655	0.340201281	0.733704954	0.996944933
2.7.1.35	pyridoxine kinase	248.946753	0.059669684	0.265845052	0.224452865	0.822404939	0.996944933
1.1.1.289	sorbose reductase	0.110533983	0.351083501	4.142162324	0.084758509	0.93245338	0.996944933
3.2.1.135	cyclomaltodextrinase / maltogenic alpha-amylase / neopullular	0.30127684	-0.681748901	4.12669228	-0.165204686	0.868782877	0.996944933
1.1.1.370	scyllo-inositol 2-dehydrogenase (NAD+)	0.128757426	0.100390562	4.134677189	0.024280145	0.98062915	0.996944933
3.2.1.1	alpha-amylase	2005.81684	-0.393869081	0.993785201	-0.396332206	0.691859972	0.996944933
5.1.3.20	ADP-L-glycero-D-manno-heptose 6-epimerase	19.91059957	0.342875229	0.676454191	0.506871321	0.612245109	0.996944933
4.2.1.115	UDP-N-acetylglucosamine 4,6-dehydratase	0.138985024	1.106983469	4.143731151	0.267146547	0.78935633	0.996944933
2.7.1.200	PTS system, galactitol-specific IIA component	13.58689989	-0.282056652	0.923186614	-0.305525066	0.759966282	0.996944933
3.6.1.-	#N/A	368.9897887	0.087516478	0.227278471	0.38506277	0.70019091	0.996944933
2.7.1.90	diphosphate-dependent phosphofructokinase	109.7540539	-0.314300008	0.458802206	-0.685044674	0.493315731	0.996944933
3.2.1.82	exo-poly-alpha-galacturonosidase	1.206697936	-0.102600554	2.717414491	-0.037756682	0.969881683	0.996944933
5.3.1.4	L-arabinose isomerase	262.4860847	-0.158248804	0.292107195	-0.54174908	0.587991374	0.996944933
2.3.1.40	acyl-[acyl-carrier-protein]-phospholipid O-acyltransferase / long	5.957426123	-1.429725831	1.560418761	-0.916244964	0.359538427	0.996944933
2.4.1.282	3-O-alpha-D-glucosyl-L-rhamnose phosphorylase	0.087521458	0.256056607	4.138412209	0.061873152	0.950663848	0.996944933
2.1.1.113	site-specific DNA-methyltransferase (cytosine-N4-specific)	0.549311404	0.20270594	4.107695754	0.049347847	0.96064209	0.996944933
1.13.11.1	catechol 1,2-dioxygenase	0.055266992	0.746328438	4.143731151	0.180110246	0.857066019	0.996944933
3.1.2.21	medium-chain acyl-[acyl-carrier-protein] hydrolase	2.090803846	1.718806798	1.982584347	0.866952672	0.385967942	0.996944933
4.2.1.129	squalene-hopene/tetraprenyl-beta-curcumene cyclase	7.194674084	-0.813199679	1.46142114	-0.556444448	0.577907061	0.996944933
4.1.1.18	lysine decarboxylase	3.388512186	-1.121175849	1.595164162	-0.702859226	0.482143483	0.996944933
3.1.26.5	ribonuclease P/MRP protein subunit POP1	316.2440482	0.00234876	0.291259041	0.008064161	0.9935658	0.996944933
2.3.1.31	homoserine O-acetyltransferase/O-succinyltransferase	18.80023827	0.729748091	0.902950909	0.808181357	0.418986188	0.996944933
2.7.1.181	O-antigen chain-terminating methyltransferase	2.5617666	0.987094195	3.783248293	0.260911819	0.79416051	0.996944933
3.5.5.7	aliphatic nitrilase	0.040667486	0.746328438	4.143731151	0.180110246	0.857066019	0.996944933
1.1.1.261	glycerol-1-phosphate dehydrogenase [NAD(P)+]	72.65407799	-0.203803311	0.357915545	-0.569417322	0.569072964	0.996944933
3.4.21.112	membrane-bound transcription factor site-1 protease	0.333031825	-0.271430703	4.130787787	-0.065709186	0.947609359	0.996944933
1.21.98.1	cyclic dehypoxanthinyl futalosine synthase	7.265508363	0.038234891	1.270281145	0.03009955	0.975987659	0.996944933
1.6.5.3	#N/A	1547.45876	0.060273102	0.225295004	0.267529688	0.789061359	0.996944933
1.3.1.78	arogenate dehydrogenase (NADP+)	0.051502971	0.522475046	4.141439557	0.126157834	0.899606988	0.996944933
6.3.5.7	aspartyl-tRNA(Asn)/glutamyl-tRNA(Gln) amidotransferase subu	503.111813	-0.137904289	0.459303614	-0.300246471	0.76398916	0.996944933
6.3.4.23	5-formaminoimidazole-4-carboxamide-1-(beta)-D-ribofuranosy	4.800089674	0.452640016	3.024152407	0.149675001	0.881021032	0.996944933
6.3.4.18	5-(carboxyamino)imidazole ribonucleotide synthase	16.67334866	-0.529397797	0.823155247	-0.643132384	0.520138203	0.996944933
3.2.1.85	6-phospho-beta-galactosidase	5.569374415	1.017366686	1.553393905	0.654931555	0.512511823	0.996944933
6.3.1.14	diphthine-ammonia ligase	0.663713212	0.601831114	4.107082188	0.146534957	0.883499096	0.996944933
2.1.1.287	ribosomal RNA-processing protein 8	0.26066422	1.632576199	4.126116739	0.395668931	0.692349278	0.996944933
2.8.1.13	tRNA-uridine 2-sulfurtransferase	511.8885342	0.19527314	0.290469683	0.672266854	0.501413827	0.996944933
5.3.3.8	3-hydroxyacyl-CoA dehydrogenase / enoyl-CoA hydratase / 3-h	0.732925299	1.55765825	4.104438413	0.379505816	0.704312286	0.996944933
3.5.3.1	arginase	24.36303767	-0.552809129	0.710259016	-0.778320468	0.436380112	0.996944933
4.1.3.27	anthranilate synthase / indole-3-glycerol phosphate synthase	268.9554381	-0.256902839	0.429963026	-0.59749984	0.550173714	0.996944933
3.4.23.43	leader peptidase (prepilin peptidase) / N-methyltransferase	105.4913545	-0.28258926	0.668878152	-0.42248242	0.67267293	0.996944933
3.5.1.6	beta-ureidopropionase	41.72853179	0.158396348	0.495015875	0.31998236	0.748981703	0.996944933
2.1.1.197	malonyl-CoA O-methyltransferase	55.86749342	-0.487868595	0.920099978	-0.53023433	0.595949472	0.996944933
3.6.1.27	phosphatidylglycerophosphatase B	463.6147504	0.295128887	0.345164269	0.85503893	0.392529596	0.996944933
2.8.3.17	cinnamoyl-CoA:phenyllactate CoA-transferase	0.78183725	1.163353338	4.138937811	0.281075336	0.778652618	0.996944933
2.1.1.260	rRNA small subunit pseudouridine methyltransferase Nep1	2.083596875	1.065242157	4.09731749	0.259985261	0.794875143	0.996944933
4.1.3.17	4-hydroxy-4-methyl-2-oxoglutarate aldolase	0.138732965	0.562377445	4.140702604	0.135816913	0.89196602	0.996944933
2.7.7.67	CDP-2,3-bis-(O-geranylgeranyl)-sn-glycerol synthase	0.353849542	0.573249688	4.119408356	0.139158257	0.889325093	0.996944933
2.5.1.1	farnesyl diphosphate synthase	376.183266	0.213124625	0.19828122	1.074860365	0.282437249	0.996944933
1.14.13.127	3-(3-hydroxy-phenyl)propionate hydroxylase	0.111464417	1.106978919	4.143731151	0.267145449	0.789357176	0.996944933
1.2.1.41	glutamate-5-semialdehyde dehydrogenase	370.2096274	-0.068661291	0.198131103	-0.346544738	0.728933369	0.996944933
1.1.1.17	mannitol-1-phosphate 5-dehydrogenase	25.46882439	-0.054592739	0.59567143	-0.091649081	0.926976854	0.996944933

1.2.1.16	succinate-semialdehyde dehydrogenase / glutarate-semialdehy	8.506514607	-0.252542798	1.001065802	-0.252273924	0.800829342	0.996944933
3.1.4.46	glycerophosphoryl diester phosphodiesterase	320.093814	-0.152003153	0.445016253	-0.341567644	0.732676291	0.996944933
1.1.1.88	hydroxymethylglutaryl-CoA reductase	4.580753034	1.207698493	1.360467521	0.887708435	0.374697608	0.996944933
3.5.4.19	phosphoribosyl-AMP cyclohydrolase	258.1677959	0.058980043	0.214733498	0.274666244	0.783572667	0.996944933
4.1.1.83	4-hydroxyphenylacetate decarboxylase large subunit	66.87725712	-0.187362869	0.614302362	-0.305001056	0.760365348	0.996944933
3.6.1.21	ADP-sugar diphosphatase	1.525601218	0.977816038	3.594594466	0.27202402	0.78560355	0.996944933
1.3.8.7	acyl-CoA dehydrogenase	0.07857	0.564649092	4.140659147	0.136366958	0.891531193	0.996944933
3.4.24.78	spore protease	147.416855	-0.458927446	0.601651452	-0.762779586	0.445594858	0.996944933
3.1.3.2	acid phosphatase	20.82235118	0.319501189	0.828143402	0.385804184	0.699641697	0.996944933
2.4.1.16	chitin synthase	0.059838501	0.796648382	4.140558968	0.192401168	0.847427977	0.996944933
2.7.8.13	phospho-N-acetylmuramoyl-pentapeptide-transferase	347.4877158	-0.00901206	0.176317691	-0.051112627	0.959235775	0.996944933
1.1.3.48	3-deoxy-alpha-D-manno-octulosonate 8-oxidase	3.68938889	0.220419101	1.405653186	0.156809022	0.875395363	0.996944933
1.1.1.49	glucose-6-phosphate 1-dehydrogenase	351.7397334	-0.072406616	0.638901053	-0.113329937	0.909768984	0.996944933
5.1.3.15	glucose-6-phosphate 1-epimerase	7.796606696	-0.461775756	1.50581546	-0.306661585	0.759100973	0.996944933
2.7.4.23	ribose 1,5-bisphosphokinase	0.110533983	0.351083501	4.142162324	0.084758509	0.93245338	0.996944933
3.1.3.4	phosphatidate phosphatase	1.586426012	-0.062158426	2.823181116	-0.022017158	0.982434268	0.996944933
5.2.1.-	#N/A	2.589767527	-1.423816909	2.064492522	-0.689669201	0.490402238	0.996944933
4.2.1.53	oleate hydratase	647.2225734	0.106547703	0.477500751	0.2231362	0.823429505	0.996944933
5.1.1.1	alanine racemase	418.4676232	-0.0374711	0.208639196	-0.179597603	0.857468487	0.996944933
5.4.99.24	23S rRNA pseudouridine955/2504/2580 synthase	151.4035972	-0.362658512	0.324517495	-1.117531469	0.263767152	0.996944933
1.1.1.95	D-3-phosphoglycerate dehydrogenase / 2-oxoglutarate reducta	655.6787153	-0.167491563	0.344870876	-0.485664563	0.627205012	0.996944933
3.4.21.26	prolyl oligopeptidase	48.70671163	0.196732525	0.677174597	0.290519647	0.771418723	0.996944933
3.4.24.84	STE24 endopeptidase	7.536420039	-1.020820922	1.062534114	-0.960741785	0.336682016	0.996944933
2.7.8.15	UDP-N-acetylglucosamine--dolichyl-phosphate N-acetylglucosa	1.224751113	0.257952509	4.101207081	0.062896729	0.94984874	0.996944933
2.4.1.212	hyaluronan synthase	0.179400451	0.248595871	4.138953896	0.060062488	0.952105866	0.996944933
2.4.2.44	5'-methylthioinosine phosphorylase	0.035008583	0.581845947	4.14032189	0.140531573	0.888240008	0.996944933
1.10.3.14	#N/A	364.9435136	0.230929622	0.421757949	0.547540651	0.58400735	0.996944933
2.7.1.22	HTH-type transcriptional regulator, transcriptional repressor of	0.749927376	-0.730171922	3.795272333	-0.192389862	0.847436833	0.996944933
1.1.1.1	alcohol dehydrogenase	554.2128909	0.305572778	0.43119324	0.708667829	0.478530633	0.996944933
2.7.11.13	classical protein kinase C alpha type	1.08659773	1.00028358	4.101557079	0.243878986	0.807324545	0.996944933
4.2.1.120	4-hydroxybutyryl-CoA dehydratase / vinylacetyl-CoA-Delta-ison	56.64405634	0.260952547	0.420449098	0.620651936	0.53482866	0.996944933
5.3.1.1	triosephosphate isomerase (TIM)	980.3903779	0.108456379	0.457056056	0.237293387	0.812429192	0.996944933
1.2.1.58	phenylglyoxylate dehydrogenase alpha subunit	0.14203695	-3.087831228	4.143731151	-0.745181363	0.456162088	0.996944933
2.7.1.78	bifunctional polynucleotide phosphatase/kinase	2.130257046	-0.235565006	2.548380275	-0.092437149	0.926350725	0.996944933
3.4.19.12	ubiquitin carboxyl-terminal hydrolase MINDY-1/2	14.16158211	0.52903349	1.889578025	0.279974408	0.779497139	0.996944933
1.6.5.5	NADPH2:quinone reductase	5.194413046	0.020928774	3.395656116	0.006163396	0.995082352	0.996944933
5.4.99.-	#N/A	5.046551887	0.74197187	2.526032754	0.293730106	0.768964147	0.996944933
6.2.1.1	acetyl-CoA synthetase	128.7675066	0.086928512	0.298151104	0.291558579	0.770624149	0.996944933
3.2.1.35	hyaluronoglucosaminidase	600.3728921	0.380595087	0.598803728	0.63559238	0.525042137	0.996944933
1.3.1.31	2-enoate reductase	39.40553896	-0.391543644	0.722724754	-0.541760388	0.587983583	0.996944933
1.5.5.1	electron-transferring-flavoprotein dehydrogenase	0.962125619	0.404354779	4.10103648	0.098598191	0.921457306	0.996944933
2.7.4.-	#N/A	274.03306	-0.036569825	0.232989791	-0.156958917	0.875277226	0.996944933
4.2.1.1	carbonic anhydrase	88.34783082	0.058724337	0.319124575	0.184016969	0.854000143	0.996944933
4.2.1.119	(3R)-3-hydroxyacyl-CoA dehydrogenase / 3a,7a,12a-trihydroxy-	0.33160195	-0.114340067	4.118136566	-0.027765001	0.97784958	0.996944933
2.7.11.-	#N/A	267.8154515	-0.017414967	0.223505199	-0.077917505	0.937893675	0.996944933
2.7.7.15	choline-phosphate cytidylyltransferase	8.165197538	-1.185651475	1.210075118	-0.979816424	0.327176743	0.996944933
4.1.2.53	2-dehydro-3-deoxy-L-rhamnonate aldolase	0.020495196	0.766777793	4.142686886	0.185091902	0.853156953	0.996944933
2.3.1.191	UDP-3-O-[3-hydroxymyristoyl] glucosamine N-acyltransferase	114.3876791	-0.355037332	0.318933145	-1.113202992	0.265621258	0.996944933
3.1.31.1	micrococcal nuclease	28.70376923	0.7010351	1.557456421	0.450115387	0.652627242	0.996944933
3.4.21.53	ATP-dependent Lon protease	752.9861725	0.166678614	0.153790802	1.083800925	0.278453075	0.996944933
2.7.1.63	polyphosphate glucokinase	1.077532702	-0.261517982	4.088099802	-0.063970547	0.948993679	0.996944933
2.1.1.205	tRNA (cytidine32/guanosine34-2'-O)-methyltransferase	2.365052993	0.439270519	3.983968811	0.110259528	0.912203554	0.996944933
4.1.1.50	S-adenosylmethionine decarboxylase	17.97606195	-0.026273295	0.667332885	-0.039370598	0.968594921	0.996944933
6.3.1.20	lipoate---protein ligase	123.1964858	-0.23946135	0.608947044	-0.393238381	0.694143424	0.996944933
2.4.1.279	nigerose phosphorylase	0.936136296	-1.781670811	4.102608344	-0.434277577	0.664086885	0.996944933
3.1.1.85	pimeloyl-[acyl-carrier protein] methyl ester esterase	49.33588044	0.290973293	0.866967283	0.335621999	0.737155932	0.996944933
3.2.1.130	glycoprotein endo-alpha-1,2-mannosidase	1.452736613	1.097516332	3.615332125	0.303572755	0.761453411	0.996944933
4.1.1.33	diphosphomevalonate decarboxylase	3.592499223	1.422979814	1.564707596	0.909422193	0.363127311	0.996944933
6.3.2.-	#N/A	3.844159933	-1.859829245	1.748740676	-1.063524896	0.287543981	0.996944933
2.4.1.-	#N/A	1222.267335	-0.242864096	0.396056517	-0.613205656	0.539740366	0.996944933
3.7.1.22	3D-(3,5/4)-trihydroxycyclohexane-1,2-dione acylhydrolase (dec	50.10551438	-0.229994641	0.411097376	-0.559465116	0.575844332	0.996944933
3.5.4.1	cytosine/creatinine deaminase	18.19951039	0.439072445	0.740434036	0.592993331	0.553185616	0.996944933
6.4.1.2	acetyl-CoA carboxylase / biotin carboxylase 2	597.5241611	0.165851111	0.349669335	0.474308424	0.635279986	0.996944933
1.3.3.3	coproporphyrinogen III oxidase	1.057957104	0.198618161	4.09800953	0.048466984	0.961344077	0.996944933
5.3.1.22	hydroxypyruvate isomerase	4.146248837	-1.445574009	1.351226676	-1.069823468	0.284698777	0.996944933
3.2.1.155	xyloglucan-specific exo-beta-1,4-glucanase	0.222928835	-3.007304203	4.143731151	-0.725747905	0.467993328	0.996944933
4.2.1.-	#N/A	0.500212525	-0.299932827	4.119751545	-0.07280362	0.94196239	0.996944933
2.1.1.56	mRNA (guanine-N7-)-methyltransferase	1.79241483	1.017437023	4.097956041	0.248279145	0.803918434	0.996944933
6.2.1.22	[citrate (pro-3S)-lyase] ligase	21.13495036	-0.500967439	0.558599617	-0.896827395	0.369811027	0.996944933
1.17.2.1	nicotinate dehydrogenase subunit A	0.166515913	0.190402508	4.138090197	0.04601217	0.96330055	0.996944933
5.3.2.1	phenylpyruvate tautomerase	0.940931647	1.116974742	4.102892698	0.272240788	0.785436881	0.996944933
1.1.1.8	glycerol-3-phosphate dehydrogenase (NAD+)	2.798147256	0.889406084	3.001083747	0.296361634	0.766953921	0.996944933
2.7.7.77	molybdenum cofactor guanylyltransferase	16.25494552	-0.866983024	0.911624652	-0.951030692	0.341588796	0.996944933
1.1.1.87	homoisocitrate dehydrogenase	0.166515913	0.190402508	4.138090197	0.04601217	0.96330055	0.996944933
6.3.2.31	coenzyme F420-0:L-glutamate ligase / coenzyme F420-1:gamm	1.257810662	0.298530367	4.080429156	0.073161512	0.941677593	0.996944933
2.4.1.255	protein O-GlcNAc transferase	1.352957927	0.752647513	4.099813335	0.183580922	0.854342231	0.996944933
3.2.-.-	#N/A	49.66509319	0.235297634	0.674768624	0.348708617	0.727308074	0.996944933
2.3.1.22	2-acylglycerol O-acyltransferase 3	2.917573992	0.44519429	4.096217474	0.108684242	0.913452941	0.996944933
2.4.1.8	maltose phosphorylase	23.28563407	-0.400795422	0.744878217	-0.538068388	0.590529836	0.996944933
3.5.1.96	succinylglutamate desuccinylase	0.040667486	0.746328438	4.143731151	0.180110246	0.857066019	0.996944933
2.3.2.26	other hect domain ubiquitin protein ligase E3	3.299592357	0.780486062	3.030543553	0.257539959	0.796761972	0.996944933
1.1.1.156	glycerol 2-dehydrogenase (NADP+)	0.687316943	1.705655672	4.1163585	0.414360331	0.678610236	0.996944933
3.5.2.14	#N/A	21.13792353	-0.423136589	1.10812662	-0.381848591	0.702573674	0.996944933
2.1.1.174	23S rRNA (guanine1835-N2)-methyltransferase	0.193014423	-0.065959317	4.131788599	-0.015963865	0.987263219	0.996944933
5.1.1.13	aspartate racemase	62.58129594	0.091452023	0.53656209	0.17044071	0.864663559	0.996944933
3.5.2.6	beta-lactamase class C	211.9174155	0.156437506	0.276663079	0.565444099	0.571771737	0.996944933
2.7.1.24	dephospho-CoA kinase	234.9531747	0.224055743	0.250645966	0.893913221	0.371368324	0.996944933

3.6.3.55	#N/A	1.048181113	0.35022697	4.075505983	0.085934598	0.931518408	0.996944933
3.13.1.1	UDP-sulfoquinovose synthase	0.039493742	1.096522945	4.143245313	0.264653155	0.791276668	0.996944933
2.1.1.185	23S rRNA (guanosine2251-2'-O)-methyltransferase	292.4598568	-0.104443515	0.183178168	-0.570174468	0.568559371	0.996944933
3.4.25.1	#N/A	26.70507566	0.151510187	2.823363827	0.053663005	0.957203658	0.996944933
3.6.3.9	sodium/potassium-transporting ATPase subunit alpha	1.356910129	1.083295053	3.687585098	0.29376815	0.768935074	0.996944933
1.5.1.20	methylenetetrahydrofolate reductase (NADPH)	318.0389004	-0.200479998	0.184285879	-1.087874987	0.2766503	0.996944933
1.13.11.4	gentisate 1,2-dioxygenase	0.110533983	0.351083501	4.142162324	0.084758509	0.93245338	0.996944933
1.2.1.9	glyceraldehyde-3-phosphate dehydrogenase (NADP+)	5.223169037	0.252333651	1.63267903	0.154551903	0.87717459	0.996944933
1.3.98.1	dihydroorotate dehydrogenase (fumarate)	176.8433461	0.224844501	0.596205026	0.377126142	0.706079865	0.996944933
3.1.3.100	ribosome biogenesis GTPase / thiamine phosphate phosphatas	362.0250623	0.109801771	0.227288486	0.483094296	0.629028784	0.996944933
2.1.1.176	16S rRNA (cytosine967-C5)-methyltransferase	285.324976	-0.033819279	0.210667245	-0.16053411	0.872460354	0.996944933
5.5.1.2	3-carboxy-cis,cis-muconate cycloisomerase	0.801064419	0.514072085	4.088315192	0.125741794	0.899936318	0.996944933
2.5.1.134	cystathionine beta-synthase (O-acetyl-L-serine)	0.078537166	1.828324946	4.143731151	0.44122673	0.659048864	0.996944933
1.1.1.276	serine 3-dehydrogenase (NADP+)	0.122131417	0.563412579	4.140682777	0.136067555	0.891767877	0.996944933
3.2.1.41	pullulanase	58.97450458	0.076479576	0.380231899	0.201139296	0.840589656	0.996944933
2.7.1.100	5-methylthioribose kinase	21.01730638	-0.249106763	0.683346364	-0.36453953	0.715455161	0.996944933
2.7.1.83	pseudouridine kinase	71.962011	-0.084227303	0.392460612	-0.214613393	0.830068751	0.996944933
4.2.2.1	hyaluronate lyase	44.64325423	-0.053068581	0.639899185	-0.082932721	0.933905036	0.996944933
2.7.11.22	CTD kinase subunit alpha	8.228143643	0.53658889	4.094044564	0.131065718	0.89572332	0.996944933
5.3.3.17	trans-2,3-dihydro-3-hydroxyanthranilate isomerase	0.035008583	0.581845947	4.14032189	0.140531573	0.888240008	0.996944933
2.5.1.46	deoxyhypusine synthase	3.308839742	1.672159497	2.389555609	0.69977844	0.484065681	0.996944933
2.8.3.21	L-carnitine CoA-transferase	1.461921331	0.64001179	3.172248391	0.201753366	0.840109541	0.996944933
6.3.2.10	UDP-N-acetylmuramoyl-tripeptide--D-alanyl-D-alanine ligase	353.4964146	-0.117106078	0.170921188	-0.685146644	0.493251389	0.996944933
1.7.-.-	#N/A	19.90229137	-0.593718144	0.998529262	-0.594592634	0.552115805	0.996944933
1.1.1.76	meso-butanediol dehydrogenase / (S,S)-butanediol dehydroger	2.544822965	0.309823523	1.935104418	0.160106876	0.872796885	0.996944933
2.7.1.147	ADP-dependent phosphofructokinase/glucokinase	1.596352927	-0.341707144	2.972685745	-0.114948963	0.908485575	0.996944933
1.3.1.44	enoyl-acyl-carrier protein] reductase / trans-2-enoyl-CoA reduc	11.55160526	0.16666163	0.875483598	0.190365223	0.849022947	0.996944933
1.5.1.41	aquacobalamin reductase / NAD(P)H-flavin reductase	1.401765292	-0.840238213	3.966604917	-0.211828057	0.832241179	0.996944933
3.5.1.38	glutamin-(asparagin-)ase	0.486306948	-0.30233635	4.119163264	-0.073397516	0.941489794	0.996944933
2.4.1.261	alpha-1,2-mannosyltransferase	1.191163093	0.624609261	4.100709476	0.152317365	0.87893663	0.996944933
2.1.1.191	23S rRNA (cytosine1962-C5)-methyltransferase	406.1474614	-0.168047893	0.198867968	-0.845022429	0.398098317	0.996944933
3.7.1.3	kynureninase	0.600135268	0.614017296	4.114544007	0.149230946	0.881371402	0.996944933
2.4.2.22	xanthine phosphoribosyltransferase	289.7354219	-0.040051206	0.454794032	-0.088064494	0.929825417	0.996944933
4.6.1.-	#N/A	0.249773869	-0.154751873	4.134519116	-0.037429232	0.970142766	0.996944933
4.4.1.-	#N/A	0.783122018	0.211579433	4.106647817	0.051521202	0.958910207	0.996944933
2.3.2.6	leucyl/phenylalanyl-tRNA---protein transferase	29.52000004	-0.703915512	0.688730905	-1.022047228	0.306758548	0.996944933
2.3.1.78	heparan-alpha-glucosaminide N-acetyltransferase	0.457925214	0.910728755	4.112943525	0.22142992	0.824757698	0.996944933
6.2.1.45	ubiquitin-activating enzyme E1	6.168451976	-0.43328877	2.112145867	-0.205141499	0.837461574	0.996944933
3.5.1.81	N-acyl-D-amino-acid deacylase	49.05676068	0.168006323	0.557121682	0.301561273	0.762986534	0.996944933
2.3.1.16	acetyl-CoA acyltransferase	1.009606782	-1.493951366	3.558237941	-0.419857073	0.674589869	0.996944933
4.2.1.126	N-acetylmuramic acid 6-phosphate etherase	72.37809151	-0.098281561	0.422378307	-0.2326861	0.816005163	0.996944933
3.5.3.6	arginine deiminase	75.43333123	0.170458444	0.47392414	0.359674535	0.719090538	0.996944933
2.4.1.166	raffinose-raffinose alpha-galactotransferase	0.473040041	0.158599271	4.135342215	0.038352151	0.96940691	0.996944933
2.7.8.39	archaetidylinositol phosphate synthase	0.782416094	0.698458459	4.104664869	0.170162116	0.864882644	0.996944933
3.1.27.-	#N/A	2.565835127	0.555148439	3.187930588	0.174140692	0.861754894	0.996944933
5.3.2.8	4-oxalomesaconate tautomerase	0.020495196	0.766777793	4.142686886	0.185091902	0.853156953	0.996944933
1.1.3.9	galactose oxidase	0.296485225	0.407401765	4.122040915	0.098834964	0.921269307	0.996944933
2.7.7.59	[protein-PII] uridylyltransferase	10.91023372	-0.221038281	1.205176581	-0.183407382	0.854478384	0.996944933
3.6.1.54	UDP-2,3-diacylglucosamine hydrolase	93.92320793	-0.212465166	0.517125246	-0.410858236	0.681176488	0.996944933
2.7.7.41	phosphatidate cytidylyltransferase	426.9388583	0.143783886	0.233437765	0.615940981	0.537933473	0.996944933
2.1.1.74	methylenetetrahydrofolate--tRNA-(uracil-5-)-methyltransferase	123.201116	-0.287097334	0.36624254	-0.783899472	0.4330991	0.996944933
4.3.1.18	D-serine dehydratase	35.25449199	0.431815079	0.457636655	0.943576251	0.345386233	0.996944933
2.5.1.108	2-(3-amino-3-carboxypropyl)histidine synthase	0.899296214	0.673514176	4.103329017	0.164138477	0.869622132	0.996944933
2.4.1.1	glycogen phosphorylase	967.5698812	0.096999576	0.195150325	0.497050551	0.619153404	0.996944933
4.2.1.30	#N/A	13.82081441	-0.24202433	1.068113728	-0.226590412	0.820742248	0.996944933
2.7.1.91	sphingosine kinase	0.070017166	0.341363118	4.138829611	0.082478176	0.934266472	0.996944933
3.6.3.4	Cu2+-exporting ATPase	720.9885904	0.058450074	0.452655995	0.129126919	0.897257222	0.996944933
2.1.3.9	N-acetylornithine carbamoyltransferase	0.471782961	0.978350162	4.115370317	0.237730772	0.812089915	0.996944933
1.1.5.6	#N/A	0.834744568	1.40304942	4.094998838	0.342625108	0.73188051	0.996944933
2.4.1.52	poly(glycerol-phosphate) alpha-glucosyltransferase	0.626809568	2.631637867	4.116249928	0.63932898	0.52260894	0.996944933
4.1.1.31	phosphoenolpyruvate carboxylase	14.51864403	0.201898835	0.72487293	0.278529969	0.780605563	0.996944933
3.6.3.36	taurine transport system ATP-binding protein	1.346509197	0.248644003	2.686445	0.092555032	0.92625707	0.996944933
3.2.1.73	licheninase	7.463162102	-1.721646677	1.706555144	-1.008843273	0.313049804	0.996944933
2.1.1.309	18S rRNA (guanine1575-N7)-methyltransferase	2.903351773	0.084003188	3.78108237	0.022216704	0.982275093	0.996944933
1.3.8.1	butyryl-CoA dehydrogenase	257.9828952	0.099832675	0.52203402	0.191237872	0.848339233	0.996944933
1.8.5.3	anaerobic dimethyl sulfoxide reductase subunit A	14.50986278	-0.704717842	0.735022819	-0.958770019	0.337674616	0.996944933
5.4.3.2	lysine 2,3-aminomutase	201.7077419	0.059157909	0.398304539	0.148524315	0.881928999	0.996944933
2.6.1.2	alanine transaminase	96.41482852	-0.349800411	0.519147327	-0.673797962	0.50043977	0.996944933
3.1.3.48	protein-tyrosine phosphatase	496.7287062	0.204804618	0.299221412	0.684458431	0.49368573	0.996944933
5.4.99.29	tRNA pseudouridine32 synthase / 23S rRNA pseudouridine746 s	66.23259907	-0.443322393	0.581020672	-0.763006231	0.44545968	0.996944933
3.1.21.4	type II restriction enzyme	19.39455655	-0.609929228	0.727741057	-0.838112982	0.401967252	0.996944933
2.3.1.97	glycylpeptide N-tetradecanoyltransferase	2.404544264	0.613374353	3.867086546	0.158614074	0.873972939	0.996944933
4.6.1.16	tRNA-intron endonuclease, archaea type	0.578535221	-0.075159113	4.113092732	-0.018273138	0.985420956	0.996944933
1.18.1.3	3-phenylpropionate/trans-cinnamate dioxygenase ferredoxin re	0.36309423	-1.024783374	4.119081808	-0.248789274	0.803523788	0.996944933
6.3.1.8	glutathionylspermidine amidase/synthetase	1.249834218	-1.200825655	3.40618042	-0.352543174	0.724430946	0.996944933
2.3.1.9	acetyl-CoA C-acetyltransferase	806.2977231	0.394934277	0.572151547	0.690261661	0.490029653	0.996944933
1.1.1.28	D-lactate dehydrogenase	327.873437	0.05728923	0.465008469	0.123200401	0.901948408	0.996944933
2.7.1.167	D-beta-D-heptose 7-phosphate kinase / D-beta-D-heptose 1-ph	30.61416296	0.395005903	0.693359096	0.569698884	0.568881947	0.996944933
4.3.2.2	adenylosuccinate lyase	603.0231759	0.22109809	0.230984219	0.957199978	0.338466328	0.996944933
6.2.1.31	2-furoate---CoA ligase	0.078987483	1.060294701	4.14177237	0.256000235	0.797950656	0.996944933
3.2.1.139	alpha-glucuronidase	61.56210552	0.057144781	0.673526454	0.084844152	0.932385292	0.996944933
4.2.1.77	trans-L-3-hydroxyproline dehydratase	0.556212935	0.524507248	4.129556555	0.127012971	0.898930132	0.996944933
2.3.1.89	tetrahydrodipicolinate N-acetyltransferase	3.32986813	-0.724934611	1.215607674	-0.596355738	0.550937603	0.996944933
1.5.1.2	pyrroline-5-carboxylate reductase	402.521796	0.075842809	0.448662832	0.16904188	0.8657637	0.996944933
1.7.2.5	nitric oxide reductase subunit B	0.040990391	0.558766837	4.140771598	0.134942685	0.892657191	0.996944933
2.7.1.71	shikimate kinase	453.6115401	-0.041610688	0.388151174	-0.107202273	0.914628512	0.996944933
1.2.5.1	pyruvate dehydrogenase (quinone)	71.21907842	0.462847099	0.652215833	0.70965327	0.477919177	0.996944933

4.99.1.1	protoporphyrin/coproporphyrin ferrochelatase	7.519813609	0.359357122	0.79449252	0.452310265	0.651045482	0.996944933
2.4.2.52	triphosphoribosyl-dephospho-CoA synthase	19.79538825	-0.487856931	0.622326771	-0.783924063	0.433084669	0.996944933
1.3.4.1	fumarate reductase (CoM/CoB) subunit A	2.34115809	-0.106553048	4.092630213	-0.026035347	0.979229145	0.996944933
6.2.1.5	succinyl-CoA synthetase alpha subunit	166.7141587	0.04798201	0.641870459	0.074753416	0.940410906	0.996944933
4.2.1.136	ADP-dependent NAD(P)H-hydrate dehydratase	352.2405352	0.146925704	0.348136296	0.422035008	0.672999465	0.996944933
5.1.3.10	CDP-paratose 2-epimerase	0.695290102	-0.387959989	4.103307873	-0.094548106	0.92467377	0.996944933
2.7.1.153	phosphatidylinositol-4,5-bisphosphate 3-kinase catalytic subuni	1.323599265	0.723277286	4.099766492	0.176419142	0.859964668	0.996944933
1.5.1.39	FMN reductase [NAD(P)H]	10.96471843	-0.055912039	0.819982156	-0.068186897	0.945636857	0.996944933
1.1.1.281	GDP-4-dehydro-6-deoxy-D-mannose reductase	10.70714889	-0.047686634	1.196950196	-0.039840115	0.968220594	0.996944933
5.4.2.7	phosphopentomutase	326.2463797	0.262436863	0.444745639	0.590083049	0.555134973	0.996944933
2.7.1.190	aminoglycoside 2''-phosphotransferase	3.45828159	1.952644608	2.183460229	0.894289065	0.371167249	0.996944933
1.12.98.2	5,10-methenyltetrahydromethanopterin hydrogenase	10.46252972	0.290962215	1.779639792	0.163495004	0.870128706	0.996944933
2.7.1.36	mevalonate kinase	4.067718738	1.99190705	1.830023245	1.088459972	0.276392098	0.996944933
4.2.1.91	cyclohexadienyl dehydratase	4.49090516	1.035280745	1.428434066	0.724766211	0.468595468	0.996944933
3.5.4.39	GTP cyclohydrolase IV	1.827417476	0.451909439	4.098177231	0.110270838	0.912194584	0.996944933
5.3.1.8	mannose-6-phosphate isomerase	421.1889985	0.221126813	0.671592522	0.329257408	0.741961135	0.996944933
3.1.3.71	2-phosphosulfolactate phosphatase	2.504384397	-0.924053852	1.642714013	-0.56251657	0.573764117	0.996944933
2.7.11.33	[pyruvate, water dikinase]-phosphate phosphotransferase / [py	9.875085199	-0.386454662	0.700210118	-0.551912422	0.581008357	0.996944933
2.7.1.19	phosphoribulokinase	0.688126876	-1.368215824	4.113557288	-0.332611345	0.739427678	0.996944933
1.2.1.24	succinate-semialdehyde dehydrogenase	0.649086519	-0.234951972	4.104896781	-0.057236999	0.954356406	0.996944933
2.7.1.168	D-glycero-alpha-D-manno-heptose-7-phosphate kinase	31.41129843	-0.675501402	0.746063085	-0.905421291	0.365242241	0.996944933
3.1.3.15	imidazoleglycerol-phosphate dehydratase / histidinol-phosphat	717.5286595	-0.035979392	0.457788798	-0.078593867	0.937355666	0.996944933
1.8.2.2	thiosulfate dehydrogenase	0.118481225	1.037825341	4.141001742	0.250621807	0.80210652	0.996944933
2.3.1.54	formate C-acetyltransferase	1844.276914	0.207695821	0.521310375	0.398411065	0.690327203	0.996944933
3.5.4.29	GTP cyclohydrolase IIa	1.229475	0.538017497	4.100579972	0.13120522	0.895612967	0.996944933
1.5.99.6	spermidine dehydrogenase	0.131528836	0.192458565	4.137447435	0.046516256	0.962898777	0.996944933
5.3.4.1	protein disulfide-isomerase	30.55821104	-0.395366887	1.05368734	-0.375222205	0.707495217	0.996944933
2.4.1.117	dolichyl-phosphate beta-glucosyltransferase	0.969773766	0.940437346	4.102248292	0.22924925	0.818675195	0.996944933
4.1.3.40	chorismate--pyruvate lyase	0.460350927	-0.211894893	4.11072583	-0.051546832	0.958889785	0.996944933
3.1.11.1	exodeoxyribonuclease I	5.432575923	-0.057578446	0.846760633	-0.067998491	0.945786835	0.996944933
2.3.1.235	minimal PKS ketosynthase (KS/KS alpha)	0.207448764	-3.379298924	4.143731151	-0.815520795	0.414774266	0.996944933
4.4.1.24	(2R)-sulfolactate sulfo-lyase subunit alpha	6.567064541	-0.100519204	1.10414279	-0.091038229	0.927462215	0.996944933
3.5.1.11	penicillin amidase	0.017504292	0.778777863	4.141980666	0.188020642	0.85086047	0.996944933
3.4.14.4	dipeptidyl-peptidase III	511.5424619	0.774565716	0.689000271	1.124187826	0.260933353	0.996944933
3.1.6.-	#N/A	21.90800047	-0.118541447	0.644894638	-0.183815215	0.854158419	0.996944933
6.1.1.9	valyl-tRNA synthetase	560.2418703	-0.074600749	0.237972165	-0.313485188	0.753912072	0.996944933
2.7.4.25	CMP/dCMP kinase	352.665934	0.161922709	0.239996253	0.674688486	0.499873701	0.996944933
1.2.1.59	glyceraldehyde-3-phosphate dehydrogenase (NAD(P))	5.22268142	-0.902407136	1.694507906	-0.532548201	0.594346367	0.996944933
1.1.1.25	shikimate dehydrogenase	278.5865007	-0.1525801	0.292299492	-0.521999197	0.60167089	0.996944933
4.2.1.59	3-hydroxyacyl-[acyl-carrier protein] dehydratase / trans-2-dece	228.3164338	-0.056583661	0.282124758	-0.200562551	0.841040643	0.996944933
2.3.1.182	(R)-citramalate synthase	88.16405049	0.00237772	0.314654063	0.007556617	0.99397075	0.996944933
2.7.1.32	choline kinase	2.466264155	0.448981942	4.096787432	0.109593663	0.912731636	0.996944933
4.1.1.65	phosphatidylserine decarboxylase	131.5107749	-0.322702293	0.385148001	-0.837865684	0.402106144	0.996944933
3.4.21.92	ATP-dependent Clp protease, protease subunit	546.6920958	0.148804346	0.28743057	0.517705356	0.604663861	0.996944933
2.7.7.61	holo-ACP synthase	18.79323321	-0.231985748	0.553663456	-0.419001372	0.67521513	0.996944933
2.7.1.157	N-acetylgalactosamine kinase	0.82830909	-0.144448462	4.107217578	-0.03516942	0.971944646	0.996944933
3.3.2.1	bifunctional isochorismate lyase / aryl carrier protein	0.147099389	-0.042647419	4.136777274	-0.010309334	0.991774487	0.996944933
1.2.1.10	acetaldehyde dehydrogenase (acetylating)	462.7407636	0.380729646	0.526539123	0.7230795	0.469631043	0.996944933
1.3.1.43	cyclohexadieny/prephenate dehydrogenase	0.078537166	1.828324946	4.143731151	0.44122673	0.659048864	0.996944933
4.1.3.3	N-acetylneuraminate lyase	310.9855885	-0.087084818	0.408013976	-0.21343587	0.830987005	0.996944933
2.3.3.14	homocitrate synthase	1.443527753	-0.691562168	3.489605474	-0.198177752	0.842905994	0.996944933
6.3.4.14	acetyl-CoA carboxylase / biotin carboxylase 2	325.4663148	0.108222889	0.361582315	0.299303601	0.764708407	0.996944933
1.-.-.-	#N/A	74.03478962	-0.084701284	0.404918063	-0.209181292	0.834306716	0.996944933
1.16.1.1	mercuric reductase	0.246942505	1.106978947	4.143731151	0.267145456	0.78935717	0.996944933
1.1.1.271	GDP-L-fucose synthase	230.4977144	-0.13036295	0.390825541	-0.333557908	0.738713185	0.996944933
2.3.1.243	lauroyl-Kdo2-lipid IVA myristoyltransferase	0.925009175	-0.526660507	4.099560942	-0.128467539	0.897778985	0.996944933
1.8.4.14	L-methionine (R)-S-oxide reductase	1.882391337	2.291303985	3.181691331	0.72015282	0.471430909	0.996944933
1.6.3.4	NADH oxidase (H2O-forming)	0.020495196	0.766777793	4.142686886	0.185091902	0.853156953	0.996944933
4.4.1.15	D-cysteine desulfhydrase	23.97297618	0.223421498	0.54545216	0.409607871	0.682093622	0.996944933
2.7.8.42	KDO II ethanolaminephosphotransferase	0.213509619	-0.098577901	4.129115425	-0.023873855	0.980953229	0.996944933
3.1.3.7	3'(2'), 5'-bisphosphate nucleotidase	473.5000343	0.080128098	0.209763008	0.381993464	0.702466213	0.996944933
3.2.1.84	#N/A	2.28393639	0.158504623	4.097221042	0.038685885	0.969140827	0.996944933
1.1.1.61	4-hydroxybutyrate dehydrogenase	27.54769835	0.236853411	0.515265469	0.459672587	0.645751249	0.996944933
3.6.3.44	#N/A	6.055494739	-1.35125203	2.004914226	-0.673969995	0.50033039	0.996944933
2.7.7.82	CMP-N,N'-diacetyllegionaminic acid synthase	10.71142702	-0.621665284	0.672699368	-0.924135377	0.355415836	0.996944933
3.2.1.65	levanase	13.36105166	0.545847592	0.921798573	0.592154954	0.553746831	0.996944933
2.7.4.1	polyphosphate kinase	535.6898625	-0.02776471	0.19599582	-0.141659707	0.887348803	0.996944933
1.6.4.-	#N/A	210.9950788	-0.299102197	0.603956319	-0.495238128	0.62043204	0.996944933
3.5.1.18	succinyl-diaminopimelate desuccinylase	253.8850799	0.251538859	0.408557816	0.615675063	0.538108999	0.996944933
2.3.3.-	#N/A	0.541183171	0.760170294	4.109992663	0.184956606	0.853263072	0.996944933
1.14.13.81	magnesium-protoporphyrin IX monomethyl ester (oxidative) cy	0.020495196	0.766777793	4.142686886	0.185091902	0.853156953	0.996944933
2.3.1.48	chromodomain protein Y	6.701815243	-1.660409721	1.763215264	-0.941694275	0.346349187	0.996944933
3.1.3.10	glucose-1-phosphatase	1.299394767	0.930822663	3.476986409	0.267709606	0.788922855	0.996944933
6.3.2.29	cyanophycin synthetase	0.53521663	1.34159318	4.107130854	0.326649729	0.743932827	0.996944933
2.4.2.18	anthranilate phosphoribosyltransferase	118.0786532	-0.419552396	0.532051385	-0.788556157	0.430371464	0.996944933
2.4.1.293	GalNAc5-diNAcBac-PP-undecaprenol beta-1,3-glucosyltransfera	1.168164939	1.421793308	4.103069955	0.346519392	0.728952413	0.996944933
3.2.1.23	beta-galactosidase	3081.654415	0.059725768	0.154012898	0.387797182	0.698166131	0.996944933
4.2.1.55	3-hydroxybutyryl-CoA dehydratase	11.55891586	0.540423437	1.033011517	0.523153352	0.600867541	0.996944933
4.2.1.7	altronate hydrolase	265.8229694	0.235826646	0.261642376	0.901331999	0.367411825	0.996944933
3.5.1.88	peptide deformylase	337.0782091	-0.069718103	0.17154417	-0.406414879	0.684437789	0.996944933
2.1.1.177	23S rRNA (pseudouridine1915-N3)-methyltransferase	198.3826389	-0.389595732	0.371853426	-1.047713172	0.294770777	0.996944933
6.5.1.6	DNA ligase 1	6.069130183	-1.676785889	1.575809235	-1.064079238	0.287292805	0.996944933
2.6.1.42	branched-chain amino acid aminotransferase	374.7399444	-0.051849551	0.207663061	-0.249681145	0.802833941	0.996944933
2.6.1.16	glucosamine---fructose-6-phosphate aminotransferase (isomeri	553.618185	-0.045336161	0.226192093	-0.200432121	0.841142641	0.996944933
4.1.99.20	3-amino-4-hydroxybenzoic acid synthase	0.49136452	-1.329415546	4.113207215	-0.323206558	0.746538815	0.996944933
2.7.7.52	terminal uridylyltransferase	0.442154223	0.448225972	4.115295735	0.108917075	0.913268264	0.996944933
5.3.1.13	arabinose 5-phosphate isomerase	85.30070013	-0.373669227	0.443735974	-0.842098115	0.399733036	0.996944933

3.1.4.3	phospholipase C	6.386108775	-0.764151339	1.767512881	-0.432331412	0.66550056	0.996944933
4.1.1.73	tartrate dehydrogenase/decarboxylase / D-malate dehydrogen	5.410002282	1.072966527	0.965341534	1.111489031	0.266357909	0.996944933
2.4.2.-	#N/A	195.2219842	-0.275125961	0.266539846	-1.032213253	0.301972228	0.996944933
1.1.1.303	(R,R)-butanediol dehydrogenase / meso-butanediol dehydroger	2.80919775	0.883083891	1.538629634	0.573941819	0.566007173	0.996944933
3.8.1.3	haloacetate dehalogenase	2.59668313	-0.22597827	2.400779036	-0.094127059	0.925008226	0.996944933
1.1.1.2	alcohol dehydrogenase (NADP+)	4.141518092	-0.103813809	2.383076096	-0.043562943	0.96525279	0.996944933
3.4.14.11	X-Pro dipeptidyl-peptidase	18.79872223	0.356388938	0.874729186	0.407427743	0.683693854	0.996944933
2.7.11.26	glycogen synthase kinase 3 beta	2.032021168	0.53379524	4.097563507	0.130271377	0.896351725	0.996944933
2.1.1.62	#N/A	0.71527601	0.864920417	4.105796456	0.210658377	0.833153856	0.996944933
2.7.7.4	bifunctional enzyme CysN/CysC	241.1413969	-0.215048514	0.420828644	-0.511012066	0.609342608	0.996944933
3.4.19.3	pyroglutamyl-peptidase	46.39155512	0.34286681	0.475232022	0.721472449	0.470618886	0.996944933
1.2.7.7	2-oxoisovalerate ferredoxin oxidoreductase, alpha subunit	4.383532921	-0.373501734	2.645895023	-0.141162718	0.887741398	0.996944933
3.6.3.5	Cd2+/Zn2+-exporting ATPase	575.5346922	0.045928947	0.278533887	0.164895365	0.869026341	0.996944933
5.1.3.4	#N/A	158.5584007	-0.106884325	0.31018142	-0.344586484	0.730405269	0.996944933
1.1.1.264	L-idonate 5-dehydrogenase	0.035008583	0.581845947	4.14032189	0.140531573	0.888240008	0.996944933
5.1.3.35	2-epi-5-epi-valiolone 7-phosphate 2-epimerase	0.538064334	-1.465165655	4.117049735	-0.35587757	0.721932254	0.996944933
2.7.4.14	UMP-CMP kinase	8.48881237	0.428399747	2.109882486	0.203044364	0.839100357	0.996944933
2.7.8.33	UDP-GlcNAc:undecaprenyl-phosphate/decaprenyl-phosphate G	45.98542719	-0.492783581	0.503272964	-0.979157666	0.327502082	0.996944933
2.7.1.28	triose/dihydroxyacetone kinase / FAD-AMP lyase (cyclizing)	1.318253244	-2.300378781	2.810666682	-0.818445956	0.413102589	0.996944933
4.1.1.17	ornithine decarboxylase	76.59343413	-0.139372629	0.779881801	-0.178709938	0.858165465	0.996944933
5.4.2.12	2,3-bisphosphoglycerate-independent phosphoglycerate mutas	1222.827048	-0.076861049	0.339823038	-0.226179629	0.821061714	0.996944933
1.1.1.330	17beta-estradiol 17-dehydrogenase / very-long-chain 3-oxoacyl	0.771316995	1.274787368	4.10502725	0.310542973	0.756148086	0.996944933
2.1.1.43	histone-lysine N-methyltransferase ASH1L	3.621512174	0.566002859	3.536962047	0.160025144	0.872861268	0.996944933
2.7.4.29	Kdo2-lipid A phosphotransferase	0.671500197	-0.234591086	4.111133718	-0.057062383	0.954495502	0.996944933
3.1.4.16	2',3'-cyclic-nucleotide 2'-phosphodiesterase / 3'-nucleotidase	285.4621956	0.260852048	0.445578603	0.585423192	0.558263198	0.996944933
4.6.1.1	adenylate cyclase	15.07032412	-0.497723589	0.796963246	-0.624525148	0.53228276	0.996944933
2.7.8.29	phosphatidylserine synthase 2	0.372518596	0.103720685	4.122777405	0.025157964	0.979928966	0.996944933
3.1.21.-	#N/A	430.4301869	-0.279475807	0.311502734	-0.897185729	0.369619819	0.996944933
4.1.1.55	4,5-dihydroxyphthalate decarboxylase	0.035008583	0.581845947	4.14032189	0.140531573	0.888240008	0.996944933
2.5.1.15	dihydropteroate synthase	181.9806537	-0.478822948	0.450682988	-1.062438478	0.288036674	0.996944933
4.2.2.24	rhamnogalacturonan exolyase	2.020556691	1.645646743	2.529676606	0.650536412	0.515345789	0.996944933
2.6.1.55	taurine---2-oxoglutarate transaminase	19.30889591	-0.518609421	0.671929557	-0.771821116	0.440220383	0.996944933
1.8.4.10	phosphoadenosine phosphosulfate reductase	38.80354416	0.363311676	1.088555616	0.333755731	0.738563891	0.996944933
3.2.1.91	cellulose 1,4-beta-cellobiosidase	0.566914952	-1.570128129	4.111995012	-0.381840962	0.702579334	0.996944933
1.5.1.5	methylenetetrahydrofolate dehydrogenase (NADP+) / metheny	460.8469926	0.286821667	0.32574259	0.880516321	0.378579668	0.996944933
2.8.4.1	#N/A	74.49309531	1.008887325	1.550214248	0.650805092	0.515172313	0.996944933
3.6.3.20	sn-glycerol 3-phosphate transport system ATP-binding protein	3.684835706	-1.749180508	1.772677755	-0.986744772	0.323767775	0.996944933
2.3.1.41	3-oxoacyl-[acyl-carrier-protein] synthase I	47.54071951	-0.460254542	0.794868156	-0.579032558	0.562567205	0.996944933
2.6.1.11	acetylornithine aminotransferase	772.0904338	0.188693424	0.402580921	0.468709306	0.639277434	0.996944933
1.3.5.2	dihydroorotate dehydrogenase	19.86044219	0.094938718	0.724060596	0.131119852	0.895680497	0.996944933
4.2.1.70	#N/A	44.63206652	0.334741042	0.554431593	0.603755353	0.546006307	0.996944933
1.2.1.20	succinate-semialdehyde dehydrogenase / glutarate-semialdehy	7.836932892	-0.046483632	1.013699557	-0.045855433	0.963425476	0.996944933
4.3.3.6	pyridoxal 5'-phosphate synthase pdxS subunit	93.34197574	-0.174309039	0.538732449	-0.323554	0.746275719	0.996944933
1.1.1.373	4-hydroxybutyrate dehydrogenase / sulfolactaldehyde 3-reduct	0.225362151	-0.026551246	4.134065803	-0.00642255	0.994875581	0.996944933
2.1.1.226	23S rRNA (cytidine1920-2'-O)/16S rRNA (cytidine1409-2'-O)-me	253.4357891	0.041248535	0.288847976	0.142803614	0.886445283	0.996944933
5.4.99.16	maltose alpha-D-glucosyltransferase / alpha-amylase	62.92338092	-0.270600473	0.444934828	-0.608180021	0.543068084	0.996944933
3.1.3.67	phosphatidylinositol-3,4,5-trisphosphate 3-phosphatase and du	1.263287807	0.549131325	4.100286777	0.133925102	0.893461801	0.996944933
2.4.1.80	ceramide glucosyltransferase	1.085351351	1.508719585	4.104732369	0.36755614	0.713204224	0.996944933
2.5.1.120	aminodeoxyfutalosine synthase	9.285788668	1.189313397	1.240965836	0.958377227	0.337872574	0.996944933
2.4.1.287	rhamnopyranosyl-N-acetylglucosaminyl-diphospho-decaprenol	0.231763367	-0.19515031	4.126197015	-0.047295442	0.962277761	0.996944933
4.3.99.3	7-carboxy-7-deazaguanine synthase	14.00860713	-0.90136975	0.851244862	-1.058884218	0.289652512	0.996944933
3.2.2.23	#N/A	78.49287026	-0.069852469	0.713797588	-0.097860332	0.9220432	0.996944933
4.1.99.2	tyrosine phenol-lyase	3.669322862	-1.808153473	2.341882362	-0.772094065	0.440058717	0.996944933
1.4.1.21	aspartate dehydrogenase	40.35370295	-0.250223716	0.457082778	-0.547436326	0.584079004	0.996944933
2.9.1.2	O-phospho-L-seryl-tRNASec:L-selenocysteinyl-tRNA synthase	1.325513361	0.221479003	4.100589869	0.054011498	0.956926003	0.996944933
3.2.1.177	alpha-D-xyloside xylohydrolase	417.1300843	0.018905973	0.370500203	0.051028239	0.959303018	0.996944933
1.3.1.9	enoyl-acyl-carrier protein] reductase I	380.3676926	0.0791423	0.325807439	0.242911273	0.808074133	0.996944933
2.7.1.12	gluconokinase	3.957617601	-1.524851892	1.568136949	-0.972397145	0.330853012	0.996944933
2.1.1.266	23S rRNA (adenine2030-N6)-methyltransferase	6.928663293	-0.604442053	1.2445467	-0.485672456	0.627199415	0.996944933
4.1.2.4	deoxyribose-phosphate aldolase	536.7825912	0.265141349	0.455187912	0.582487676	0.560238241	0.996944933
3.4.13.21	dipeptidase E	32.5585104	0.275405058	0.38792802	0.709938554	0.477742241	0.996944933
2.5.1.10	farnesyl diphosphate synthase	376.183266	0.213124625	0.19828122	1.074860365	0.282437249	0.996944933
2.8.3.20	succinyl-CoA---D-citramalate CoA-transferase	0.051502971	0.522475046	4.141439557	0.126157834	0.899606988	0.996944933
2.7.2.15	propionate kinase	0.232008508	-0.522325017	4.130245682	-0.126463425	0.899365099	0.996944933
6.1.1.20	#N/A	740.7444539	0.188098548	0.194527543	0.966950722	0.333568668	0.996944933
2.4.1.230	kojibiose phosphorylase	16.45124371	0.215940155	0.99397084	0.21724999	0.828013528	0.996944933
1.2.7.11	2-oxoglutarate/2-oxoacid ferredoxin oxidoreductase subunit al	859.9599729	0.236636296	0.288077375	0.821433116	0.411399607	0.996944933
4.1.2.48	threonine aldolase	275.6256696	-0.258063157	0.268637619	-0.960636704	0.336734867	0.996944933
2.1.1.319	type I protein arginine methyltransferase	5.72190343	0.950272955	2.588408981	0.367126278	0.713524827	0.996944933
3.1.2.12	S-formylglutathione hydrolase	0.959060744	-1.152079664	3.667346602	-0.314145291	0.753410692	0.996944933
3.4.15.5	peptidyl-dipeptidase Dcp	237.1508587	-0.084560944	0.421341909	-0.200694358	0.840937572	0.996944933
2.1.1.196	cobalt-precorrin-6B (C15)-methyltransferase	2.601572733	-0.770319546	1.774133771	-0.434194737	0.664147035	0.996944933
5.4.2.3	phosphoacetylglucosamine mutase	1.577647769	0.020841063	4.099759639	0.005083484	0.995943984	0.996944933
3.5.1.116	ureidoglycolate amidohydrolase	0.244004913	-0.085027182	4.126821941	-0.02060355	0.983561909	0.996944933
2.8.3.25	bile acid CoA-transferase	12.81360026	-0.050365535	0.89628714	-0.056193527	0.955187638	0.996944933
6.5.1.7	DNA ligase 1	6.069130183	-1.676785889	1.575809235	-1.064079238	0.287292805	0.996944933
2.1.1.227	23S rRNA (cytidine1920-2'-O)/16S rRNA (cytidine1409-2'-O)-me	253.4357891	0.041248535	0.288847976	0.142803614	0.886445283	0.996944933
1.5.3.-	#N/A	0.327782895	-0.13017286	4.134426062	-0.03148511	0.974882667	0.996944933
1.4.5.1	D-amino-acid dehydrogenase	4.746604651	-1.297769516	1.727323938	-0.751317971	0.452461316	0.996944933
1.1.1.108	carnitine 3-dehydrogenase	0.055503779	0.282472617	4.137674506	0.068268448	0.94557194	0.996944933
5.3.1.14	L-rhamnose isomerase	106.3100671	-0.187318024	0.314086122	-0.596390641	0.550914291	0.996944933
1.1.1.267	1-deoxy-D-xylulose-5-phosphate reductoisomerase	352.3064475	0.134382016	0.162425464	0.82734574	0.408041125	0.996944933
2.3.1.82	aminoglycoside 6'-N-acetyltransferase	9.056722013	0.099529815	0.653474351	0.152308679	0.87894348	0.996944933
2.5.1.97	pseudaminic acid synthase	14.79377256	-1.002967923	0.967234462	-1.036943949	0.29976198	0.996944933
2.1.1.90	methanol---5-hydroxybenzimidazolylcobamide Co-methyltransf	3.288051399	-0.187847297	2.561814888	-0.073325867	0.941546808	0.996944933
3.4.19.11	g-D-glutamyl-meso-diaminopimelate peptidase	119.8936844	-0.933345766	0.837887183	-1.113927728	0.265310193	0.996944933
2.4.99.18	dolichyl-diphosphooligosaccharide---protein glycosyltransferase	10.80229488	-0.017990487	1.594856553	-0.011280317	0.9909998	0.996944933

2.6.1.110	dTDP-4-dehydro-2,3,6-trideoxy-D-glucose 4-aminotransferase	5.438875754	1.30568261	2.35884478	0.553526294	0.579903081	0.996944933
1.7.1.7	GMP reductase	8.098065539	0.320064902	1.204523772	0.265719041	0.790455598	0.996944933
2.7.1.23	NAD+ kinase	351.0343927	0.098219934	0.215327722	0.456141613	0.648288148	0.996944933
2.7.1.137	phosphatidylinositol 3-kinase	3.121209164	0.500870123	3.005338256	0.16666015	0.867637463	0.996944933
2.4.1.15	trehalose 6-phosphate synthase	39.99829842	-0.622342478	0.606217882	-1.02659868	0.304609476	0.996944933
2.7.7.-	#N/A	737.2154561	0.023505575	0.253460292	0.09273869	0.926111159	0.996944933
2.4.99.15	3-deoxy-D-manno-octulosonic-acid transferase	104.6062867	-0.408074653	0.481327791	-0.847810288	0.396543632	0.996944933
1.17.7.3	(E)-4-hydroxy-3-methylbut-2-enyl-diphosphate synthase	363.237913	-0.177782312	0.168058057	-1.057862475	0.290118145	0.996944933
5.4.99.25	tRNA pseudouridine55 synthase	354.9260802	0.121422523	0.203271058	0.5973429	0.550278468	0.996944933
3.6.1.9	ectonucleotide pyrophosphatase/phosphodiesterase family me	8.082410966	0.007334002	0.98546749	0.007442156	0.994062074	0.996944933
2.5.1.101	N,N'-diacetyllegionaminate synthase	17.87356951	0.080362276	0.713054374	0.112701469	0.910267236	0.996944933
2.7.7.76	molybdenum cofactor cytidylyltransferase	3243.651555	0.198826773	1.04427274	0.19039736	0.848997766	0.996944933
3.2.2.27	uracil-DNA glycosylase	212.7511443	0.33337354	0.587834277	0.567121642	0.57063154	0.996944933
1.1.1.308	sulfopropanediol 3-dehydrogenase	0.443584731	-1.354021127	4.113106867	-0.32919668	0.742007032	0.996944933
2.3.3.1	citrate synthase	968.5318437	0.272641426	0.310735359	0.87740715	0.380265531	0.996944933
3.1.26.11	ribonuclease Z	244.95852	-0.26833129	0.304041922	-0.882547013	0.377481068	0.996944933
2.3.1.245	3-hydroxy-5-phosphonooxypentane-2,4-dione thiolase	47.6858419	0.429337877	0.6903333	0.621928388	0.533988957	0.996944933
3.1.25.-	#N/A	0.802665179	-1.099254003	4.114779958	-0.2671477	0.789355442	0.996944933
6.3.4.10	biotin---protein ligase	1.456275519	-0.021285983	4.100745809	-0.005190759	0.995858392	0.996944933
4.1.2.52	4-hydroxy-2-oxoheptanedioate aldolase	7.602420736	-0.675770768	1.155007089	-0.585079325	0.55849438	0.996944933
3.4.17.4	Gly-Xaa carboxypeptidase	0.887846555	-0.99790294	4.110056374	-0.242795439	0.808163869	0.996944933
3.1.1.84	carboxylesterase 2	0.167196626	-3.226634141	4.143731151	-0.778678448	0.436169155	0.996944933
2.7.1.105	6-phosphofructo-2-kinase	6.021524969	0.795353791	2.465070101	0.322649563	0.746960653	0.996944933
5.1.2.3	3-hydroxyacyl-CoA dehydrogenase / enoyl-CoA hydratase / 3-h	1.265356629	0.786831885	4.086458994	0.192546135	0.847314433	0.996944933
1.8.98.1	#N/A	299.754454	0.116440296	0.481616372	0.241769804	0.808958538	0.996944933
3.4.11.4	tripeptide aminopeptidase	303.3330781	0.00975234	0.241925007	0.040311417	0.967844852	0.996944933
1.5.3.19	4-methylaminobutanoate oxidase (formaldehyde-forming)	0.213055426	1.090984828	4.142999974	0.263332087	0.792294631	0.996944933
2.5.1.60	geranylgeranyl transferase type-2 subunit beta	0.326417885	1.323833439	4.121741788	0.321183011	0.748071706	0.996944933
3.4.21.72	IgA-specific serine endopeptidase	1.260296949	1.914592882	4.103752484	0.466546871	0.640824106	0.996944933
4.1.1.22	histidine decarboxylase	0.995281257	0.671385154	4.077281096	0.164664917	0.869207732	0.996944933
4.1.1.35	UDP-glucuronate decarboxylase	85.12043626	0.040250141	0.433083853	0.092938447	0.925952462	0.996944933
4.2.1.12	phosphogluconate dehydratase	0.449882367	-0.505523507	4.1129981	-0.122908763	0.902179347	0.996944933
3.4.11.9	Xaa-Pro aminopeptidase	736.9519013	0.201957677	0.211523242	0.95477771	0.339690122	0.996944933
4.1.2.-	#N/A	5.460202902	-0.2649724	1.354708529	-0.19559366	0.844928223	0.996944933
3.2.2.-	#N/A	1975.209521	-0.905774625	1.056929057	-0.856987154	0.391451984	0.996944933
1.1.1.133	dTDP-4-dehydrorhamnose reductase	378.8196254	0.080741245	0.218027155	0.370326551	0.711139193	0.996944933
1.2.1.79	succinate-semialdehyde dehydrogenase / glutarate-semialdehy	7.836932892	-0.046483632	1.013699557	-0.045855433	0.963425476	0.996944933
1.2.7.4	#N/A	586.3369208	0.460681447	0.495353678	0.9300051	0.352368444	0.996944933
2.5.1.75	tRNA dimethylallyltransferase	650.812732	-0.701615913	0.700472547	-1.001632278	0.316521225	0.996944933
4.2.3.1	threonine synthase	413.1252022	-0.130939174	0.191619409	-0.683329392	0.494398726	0.996944933
2.7.11.21	polo-like kinase 1	1.054442185	0.942737796	4.101579064	0.229847525	0.818210254	0.996944933
1.3.1.14	dihydroorotate dehydrogenase (NAD+) catalytic subunit	331.264359	0.034390873	0.22738577	0.151244614	0.879782758	0.996944933
4.2.1.79	2-methylcitrate dehydratase	0.764996219	2.074672836	4.096529483	0.506446455	0.612543269	0.996944933
3.5.2.3	dihydroorotase	378.9687693	0.03539206	0.170975353	0.207000946	0.83600912	0.996944933
2.1.1.250	trimethylamine---corrinoid protein Co-methyltransferase	3.364886548	0.575814786	1.743808292	0.330205326	0.741244823	0.996944933
2.1.1.216	tRNA (guanine26-N2/guanine27-N2)-dimethyltransferase	2.22570527	1.137883436	3.267802974	0.348210539	0.727682073	0.996944933
2.4.2.4	thymidine phosphorylase	3.167265103	0.335488387	1.98192669	0.169273863	0.865581234	0.996944933
6.1.1.16	#N/A	481.2742586	-0.011320138	0.136039427	-0.083212182	0.933682826	0.996944933
6.3.4.6	urea carboxylase	2.097824574	-0.249883574	3.010076209	-0.083015697	0.933839058	0.996944933
6.3.2.43	[lysine-biosynthesis-protein LysW]---L-2-aminoadipate ligase	0.208936523	2.156739115	4.130121372	0.522197515	0.601532816	0.996944933
6.3.2.38	N2-citryl-N6-acetyl-N6-hydroxylysine synthase	1.063733801	0.379462942	4.100003023	0.092551869	0.926259583	0.996944933
2.7.7.66	phosphoribosyl-dephospho-CoA transferase	1.683890448	-0.249855922	2.822166512	-0.088533374	0.92945276	0.996944933
2.3.1.251	lipid IVA palmitoyltransferase	1.352951646	-1.538326575	3.005508545	-0.511835702	0.608766002	0.996944933
2.1.1.201	demethylmenaquinone methyltransferase / 2-methoxy-6-polyp	61.69477923	-0.473710915	0.474794648	-0.997717469	0.31841638	0.996944933
3.5.4.4	adenosine deaminase	45.49932187	-0.34212749	0.535226232	-0.639220332	0.522679607	0.996944933
1.2.1.11	aspartate-semialdehyde dehydrogenase	340.0143125	-0.153594734	0.184954219	-0.830447313	0.406285925	0.996944933
4.1.3.16	#N/A	1.127391998	2.34485611	4.077235863	0.575109262	0.565217404	0.996944933
3.2.1.55	alpha-N-arabinofuranosidase	371.5198309	-0.422847372	0.519513048	-0.813930226	0.415684924	0.996944933
1.1.1.215	glyoxylate/hydroxypyruvate/2-ketogluconate reductase	0.185410644	1.081639064	4.137156614	0.261445037	0.79374933	0.996944933
4.2.1.114	methanogen homoaconitase large subunit	2.400786458	0.37605225	4.09408997	0.091852464	0.92681526	0.996944933
2.3.1.179	3-oxoacyl-[acyl-carrier-protein] synthase II	483.3914302	0.105203379	0.307591112	0.342023469	0.732333231	0.996944933
3.6.1.22	NAD+ diphosphatase	128.9123178	0.009131384	0.445092657	0.020515692	0.983631995	0.996944933
3.2.1.70	glucan 1,6-alpha-glucosidase	19.62187726	-0.509281786	0.676287923	-0.753054681	0.451417056	0.996944933
2.1.4.1	glycine amidinotransferase	23.72075067	-0.405602983	0.713720555	-0.568293823	0.569835473	0.996944933
3.3.1.1	adenosylhomocysteinase	99.61581088	-0.115881009	0.299102292	-0.387429359	0.698438372	0.996944933
2.7.1.140	inositol-polyphosphate multikinase	2.104401683	0.730453733	4.097269769	0.178278164	0.858504527	0.996944933
2.3.1.231	tRNA wybutosine-synthesizing protein 4	0.052512875	0.445935252	4.139416356	0.107729016	0.914210652	0.996944933
2.1.1.133	precorrin-4/cobalt-precorrin-4 C11-methyltransferase	98.82307915	-0.392261386	0.371773178	-1.05510943	0.29137528	0.996944933
1.1.1.338	(2R)-3-sulfolactate dehydrogenase (NADP+)	0.413530944	1.106970167	4.143731151	0.267143337	0.789358802	0.996944933
6.1.1.3	threonyl-tRNA synthetase	879.1230179	-0.13588392	0.353173138	-0.384751571	0.700421483	0.996944933
6.3.2.6	phosphoribosylaminoimidazole carboxylase / phosphoribosylan	355.7877861	-0.011036435	0.229587143	-0.048070787	0.961659828	0.996944933
1.2.1.88	1-pyrroline-5-carboxylate dehydrogenase	87.99903725	0.22014761	0.399307684	0.551323249	0.581412104	0.996944933
3.4.16.4	D-alanyl-D-alanine carboxypeptidase	6876.188992	0.309213588	0.757041097	0.408450201	0.682943184	0.996944933
2.8.3.18	succinyl-CoA:acetate CoA-transferase	16.37592351	-0.471454358	0.853764513	-0.552206551	0.580806846	0.996944933
3.-.-.-	#N/A	7.254996096	0.285778861	1.159252095	0.24652003	0.805279701	0.996944933
3.5.1.89	N-acetylglucosaminylphosphatidylinositol deacetylase	0.083257956	0.679950179	4.141091794	0.164195872	0.86957695	0.996944933
4.2.1.45	CDP-glucose 4,6-dehydratase	62.0299926	-0.194938586	0.4325101	-0.450714528	0.65219531	0.996944933
2.7.2.2	carbamate kinase	196.6123808	-0.34524611	0.351810814	-0.981340243	0.326424984	0.996944933
2.7.1.177	L-threonine kinase	0.753503802	0.000494157	4.102472686	0.000120453	0.999903892	0.999903892
3.1.3.8	3-phytase	0.154508912	-0.002296378	4.13282253	-0.000555644	0.99955666	0.999903892
6.3.2.26	N-(5-amino-5-carboxypentanoyl)-L-cysteinyl-D-valine synthase	0	NA	NA	NA	NA	NA
1.14.19.3	linoleoyl-CoA desaturase	0	NA	NA	NA	NA	NA
2.3.1.37	5-aminolevulinate synthase	0	NA	NA	NA	NA	NA
3.1.3.99	IMP and pyridine-specific 5'-nucleotidase	0	NA	NA	NA	NA	NA
1.3.99.32	glutaryl-CoA dehydrogenase (non-decarboxylating)	0	NA	NA	NA	NA	NA
2.4.1.58	UDP-glucose:(galactosyl)LPS alpha-1,2-glucosyltransferase	0	NA	NA	NA	NA	NA
4.1.1.45	aminocarboxymuconate-semialdehyde decarboxylase	0	NA	NA	NA	NA	NA

1.14.13.89	#N/A	0	NA	NA	NA	NA	NA
1.12.1.2	NAD-reducing hydrogenase large subunit	0	NA	NA	NA	NA	NA
5.4.3.-	#N/A	0	NA	NA	NA	NA	NA
2.7.1.182	phytol kinase	0	NA	NA	NA	NA	NA
1.1.1.53	3alpha(or 20beta)-hydroxysteroid dehydrogenase	0	NA	NA	NA	NA	NA
2.4.1.161	oligosaccharide 4-alpha-D-glucosyltransferase	0	NA	NA	NA	NA	NA
2.1.1.71	phosphatidyl-N-methylethanolamine N-methyltransferase	0	NA	NA	NA	NA	NA
3.1.3.78	phosphatidylinositol-4,5-bisphosphate 4-phosphatase	0	NA	NA	NA	NA	NA
1.5.1.21	delta1-piperideine-2-carboxylate reductase	0	NA	NA	NA	NA	NA
3.2.1.15	polygalacturonase	0	NA	NA	NA	NA	NA
2.7.1.166	3-deoxy-D-manno-octulosonic acid kinase	0	NA	NA	NA	NA	NA
4.1.1.7	benzoylformate decarboxylase	0	NA	NA	NA	NA	NA
3.4.23.40	phytepsin	0	NA	NA	NA	NA	NA
1.13.11.3	#N/A	0	NA	NA	NA	NA	NA
2.5.1.85	all-trans-nonaprenyl-diphosphate synthase	0	NA	NA	NA	NA	NA
2.7.10.2	protein-tyrosine kinase	0	NA	NA	NA	NA	NA
3.5.3.17	guanidinopropionase	0	NA	NA	NA	NA	NA
1.16.1.10	ferric-chelate reductase [NAD(P)H]	0	NA	NA	NA	NA	NA
2.4.1.318	demethyllactenocin mycarosyltransferase	0	NA	NA	NA	NA	NA
3.1.27.1	ribonuclease T2	0	NA	NA	NA	NA	NA
3.7.1.17	4,5:9,10-diseco-3-hydroxy-5,9,17-trioxoandrosta-1(10),2-diene-	0	NA	NA	NA	NA	NA
1.3.5.6	zeta-carotene desaturase	0	NA	NA	NA	NA	NA
2.6.1.108	(5-formylfuran-3-yl)methyl phosphate transaminase	0	NA	NA	NA	NA	NA
1.1.1.197	carbonyl reductase 1	0	NA	NA	NA	NA	NA
1.14.18.6	4-hydroxysphinganine ceramide fatty acyl 2-hydroxylase	0	NA	NA	NA	NA	NA
1.1.1.252	tetrahydroxynaphthalene reductase	0	NA	NA	NA	NA	NA
1.3.99.28	phytoene desaturase	0	NA	NA	NA	NA	NA
3.1.4.37	2',3'-cyclic-nucleotide 3'-phosphodiesterase	0	NA	NA	NA	NA	NA
1.1.1.184	carbonyl reductase 1	0	NA	NA	NA	NA	NA
2.4.1.109	dolichyl-phosphate-mannose-protein mannosyltransferase	0	NA	NA	NA	NA	NA
1.3.1.33	protochlorophyllide reductase	0	NA	NA	NA	NA	NA
3.4.17.22	carboxypeptidase D	0	NA	NA	NA	NA	NA
1.3.1.85	crotonyl-CoA carboxylase/reductase	0	NA	NA	NA	NA	NA
3.2.2.16	5'-methylthioadenosine nucleosidase	0	NA	NA	NA	NA	NA
1.3.99.23	all-trans-retinol 13,14-reductase	0	NA	NA	NA	NA	NA
1.1.1.292	1,5-anhydro-D-fructose reductase (1,5-anhydro-D-mannitol-for	0	NA	NA	NA	NA	NA
1.13.11.53	1,2-dihydroxy-3-keto-5-methylthiopentene dioxygenase	0	NA	NA	NA	NA	NA
3.4.24.75	lysostaphin	0	NA	NA	NA	NA	NA
1.3.1.-	#N/A	0	NA	NA	NA	NA	NA
2.5.1.92	(2Z,6Z)-farnesyl diphosphate synthase	0	NA	NA	NA	NA	NA
2.5.1.68	short-chain Z-isoprenyl diphosphate synthase	0	NA	NA	NA	NA	NA
3.4.14.12	Xaa-Xaa-Pro tripeptidyl-peptidase	0	NA	NA	NA	NA	NA
2.4.1.292	GalNAc-alpha-(1->4)-GalNAc-alpha-(1->3)-diNAcBac-PP-undeca	0	NA	NA	NA	NA	NA
2.7.1.215	erythritol kinase (D-erythritol 1-phosphate-forming)	0	NA	NA	NA	NA	NA
4.1.1.51	3-hydroxy-2-methylpyridine-4,5-dicarboxylate 4-decarboxylase	0	NA	NA	NA	NA	NA
2.7.1.74	deoxycitidine kinase	0	NA	NA	NA	NA	NA
1.10.3.11	ubiquinol oxidase	0	NA	NA	NA	NA	NA
2.4.1.60	abequosyltransferase	0	NA	NA	NA	NA	NA
5.3.3.10	5-carboxymethyl-2-hydroxymuconate isomerase	0	NA	NA	NA	NA	NA
2.7.1.161	riboflavin kinase, archaea type	0	NA	NA	NA	NA	NA
5.1.3.24	N-acetylneuraminate epimerase	0	NA	NA	NA	NA	NA
1.3.7.9	4-hydroxybenzoyl-CoA reductase subunit gamma	0	NA	NA	NA	NA	NA
2.7.8.23	carboxyvinyl-carboxyphosphonate phosphorylmutase	0	NA	NA	NA	NA	NA
6.2.1.16	acetoacetyl-CoA synthetase	0	NA	NA	NA	NA	NA
1.3.99.29	phytoene desaturase	0	NA	NA	NA	NA	NA
5.1.3.37	mannuronan 5-epimerase	0	NA	NA	NA	NA	NA
1.14.11.17	taurine dioxygenase	0	NA	NA	NA	NA	NA
1.10.2.-	#N/A	0	NA	NA	NA	NA	NA
1.14.17.3	peptidylglycine monooxygenase	0	NA	NA	NA	NA	NA
4.3.1.24	phenylalanine ammonia-lyase	0	NA	NA	NA	NA	NA
2.7.1.138	ceramide kinase	0	NA	NA	NA	NA	NA
1.8.7.1	sulfite reductase (ferredoxin)	0	NA	NA	NA	NA	NA
1.1.1.396	bacteriochlorophyllide a dehydrogenase	0	NA	NA	NA	NA	NA
3.6.3.6	#N/A	0	NA	NA	NA	NA	NA
2.5.1.131	(4-(4-[2-(gamma-L-glutamylamino)ethyl]phenoxymethyl)furan-:	0	NA	NA	NA	NA	NA
4.1.3.25	malyl-CoA/(S)-citramalyl-CoA lyase	0	NA	NA	NA	NA	NA
2.5.1.44	homospermidine synthase	0	NA	NA	NA	NA	NA
2.7.1.86	NADH kinase	0	NA	NA	NA	NA	NA
1.13.99.1	inositol oxygenase	0	NA	NA	NA	NA	NA
1.5.1.33	pteridine reductase	0	NA	NA	NA	NA	NA
3.1.3.77	enolase-phosphatase E1	0	NA	NA	NA	NA	NA
1.1.99.21	D-sorbitol dehydrogenase (acceptor)	0	NA	NA	NA	NA	NA
4.1.1.79	sulfopyruvate decarboxylase subunit alpha	0	NA	NA	NA	NA	NA
3.6.1.61	diadenosine hexaphosphate hydrolase (ATP-forming)	0	NA	NA	NA	NA	NA
2.7.7.68	2-phospho-L-lactate guanylyltransferase	0	NA	NA	NA	NA	NA
1.1.1.385	dihydroanticapsin dehydrogenase	0	NA	NA	NA	NA	NA
3.2.1.67	galacturan 1,4-alpha-galacturonidase	0	NA	NA	NA	NA	NA
6.2.1.41	HIP---CoA ligase	0	NA	NA	NA	NA	NA
5.1.1.17	isopenicillin-N epimerase	0	NA	NA	NA	NA	NA
4.1.2.29	6-phospho-5-dehydro-2-deoxy-D-gluconate aldolase	0	NA	NA	NA	NA	NA
2.4.1.289	N-acetylglucosaminyl-diphospho-decaprenol L-rhamnosyltransf	0	NA	NA	NA	NA	NA
4.2.1.140	gluconate/galactonate dehydratase	0	NA	NA	NA	NA	NA
2.7.4.12	T-even-induced deoxynucleotide kinase	0	NA	NA	NA	NA	NA
1.1.1.117	D-arabinose 1-dehydrogenase	0	NA	NA	NA	NA	NA
1.14.99.46	pyrimidine oxygenase	0	NA	NA	NA	NA	NA
2.3.2.8	arginyl-tRNA---protein transferase	0	NA	NA	NA	NA	NA
1.1.1.371	scyllo-inositol 2-dehydrogenase (NADP+)	0	NA	NA	NA	NA	NA

1.1.1.378	L-rhamnose 1-dehydrogenase	0	NA	NA	NA	NA	NA
2.1.1.179	16S rRNA (guanine(1405)-N(7))-methyltransferase	0	NA	NA	NA	NA	NA
1.1.1.377	L-rhamnose 1-dehydrogenase	0	NA	NA	NA	NA	NA
1.1.1.244	methanol dehydrogenase	0	NA	NA	NA	NA	NA
3.4.17.14	zinc D-Ala-D-Ala carboxypeptidase	0	NA	NA	NA	NA	NA
3.4.15.6	cyanophycinase	0	NA	NA	NA	NA	NA
3.1.1.24	3-oxoadipate enol-lactonase	0	NA	NA	NA	NA	NA
3.5.1.97	acyl-homoserine-lactone acylase	0	NA	NA	NA	NA	NA
1.3.1.6	NADH-dependent fumarate reductase subunit A	0	NA	NA	NA	NA	NA
2.8.3.6	#N/A	0	NA	NA	NA	NA	NA
5.1.2.2	mandelate racemase	0	NA	NA	NA	NA	NA
1.1.1.275	(+)-trans-carveol dehydrogenase	0	NA	NA	NA	NA	NA
1.1.1.189	carbonyl reductase 1	0	NA	NA	NA	NA	NA
5.1.99.6	NAD(P)H-hydrate epimerase	0	NA	NA	NA	NA	NA
3.7.1.14	2-hydroxy-6-oxonona-2,4-dienedioate hydrolase	0	NA	NA	NA	NA	NA
2.7.7.93	phosphonoformate cytidylyltransferase	0	NA	NA	NA	NA	NA
3.4.24.71	endothelin-converting enzyme	0	NA	NA	NA	NA	NA
2.5.1.42	geranylgeranylglycerol-phosphate geranylgeranyltransferase	0	NA	NA	NA	NA	NA
2.4.1.34	1,3-beta-glucan synthase	0	NA	NA	NA	NA	NA
1.2.1.60	5-carboxymethyl-2-hydroxymuconic-semialdehyde dehydrogen	0	NA	NA	NA	NA	NA
1.3.99.26	phytoene desaturase	0	NA	NA	NA	NA	NA
3.6.1.12	dCTP diphosphatase	0	NA	NA	NA	NA	NA
2.4.1.173	sterol 3beta-glucosyltransferase	0	NA	NA	NA	NA	NA
2.6.1.106	dTDP-3-amino-3,4,6-trideoxy-alpha-D-glucose transaminase	0	NA	NA	NA	NA	NA
1.1.1.173	L-rhamnose 1-dehydrogenase	0	NA	NA	NA	NA	NA
3.1.3.37	fructose-1,6-bisphosphatase I / sedoheptulose-1,7-bisphosphat	0	NA	NA	NA	NA	NA
2.3.1.4	glucosamine-phosphate N-acetyltransferase	0	NA	NA	NA	NA	NA
3.2.1.46	galactosylceramidase	0	NA	NA	NA	NA	NA
3.4.11.24	aminopeptidase S	0	NA	NA	NA	NA	NA
2.4.1.290	N,N'-diacetylbacillosaminyl-diphospho-undecaprenol alpha-1,3-	0	NA	NA	NA	NA	NA
3.1.11.3	exodeoxyribonuclease (lambda-induced)	0	NA	NA	NA	NA	NA
5.1.3.5	UDP-arabinose 4-epimerase	0	NA	NA	NA	NA	NA
1.1.1.21	aldehyde reductase	0	NA	NA	NA	NA	NA
3.5.4.34	tRNA-specific adenosine deaminase 1	0	NA	NA	NA	NA	NA
3.1.1.75	poly(3-hydroxybutyrate) depolymerase	0	NA	NA	NA	NA	NA
2.3.1.86	fatty acid synthase subunit alpha, fungi type	0	NA	NA	NA	NA	NA
3.4.21.68	tissue plasminogen activator	0	NA	NA	NA	NA	NA
6.2.1.17	propionyl-CoA synthetase	0	NA	NA	NA	NA	NA
6.3.4.24	tyramine---L-glutamate ligase	0	NA	NA	NA	NA	NA
4.2.1.161	bisanhydrobacterioruberin hydratase	0	NA	NA	NA	NA	NA
3.4.22.49	separase	0	NA	NA	NA	NA	NA
4.4.1.19	phosphosulfolactate synthase	0	NA	NA	NA	NA	NA
1.3.99.16	isoquinoline 1-oxidoreductase	0	NA	NA	NA	NA	NA
4.4.1.2	cystathionine gamma-lyase / homocysteine desulfhydrase	0	NA	NA	NA	NA	NA
3.1.21.7	deoxyribonuclease V	0	NA	NA	NA	NA	NA
2.5.1.59	protein farnesyltransferase/geranylgeranyltransferase type-1 s	0	NA	NA	NA	NA	NA
2.5.1.84	all-trans-nonaprenyl-diphosphate synthase	0	NA	NA	NA	NA	NA
2.1.1.17	phosphatidylethanolamine/phosphatidyl-N-methylethanolamir	0	NA	NA	NA	NA	NA
3.5.4.30	dCTP deaminase (dUMP-forming)	0	NA	NA	NA	NA	NA
1.3.99.31	phytoene desaturase	0	NA	NA	NA	NA	NA
2.5.1.43	nicotianamine synthase	0	NA	NA	NA	NA	NA
3.1.3.87	2-hydroxy-3-keto-5-methylthiopentenyl-1-phosphate phosphat	0	NA	NA	NA	NA	NA
3.4.21.50	lysyl endopeptidase	0	NA	NA	NA	NA	NA
3.5.2.9	5-oxoprolinase (ATP-hydrolysing)	0	NA	NA	NA	NA	NA
1.1.1.51	aldo-keto reductase family 1 member C3	0	NA	NA	NA	NA	NA
1.2.1.71	succinylglutamic semialdehyde dehydrogenase	0	NA	NA	NA	NA	NA
5.1.99.7	D-erythro-7,8-dihydroneopterin triphosphate epimerase	0	NA	NA	NA	NA	NA
3.4.24.27	thermolysin	0	NA	NA	NA	NA	NA
4.2.1.41	5-dehydro-4-deoxyglucarate dehydratase	0	NA	NA	NA	NA	NA
1.3.7.7	light-independent protochlorophyllide reductase subunit L	0	NA	NA	NA	NA	NA
3.4.24.25	vibriolysin	0	NA	NA	NA	NA	NA
1.14.13.1	salicylate hydroxylase	0	NA	NA	NA	NA	NA
6.2.1.42	3-oxocholest-4-en-26-oate---CoA ligase	0	NA	NA	NA	NA	NA
1.3.1.83	geranylgeranyl diphosphate/geranylgeranyl-bacteriochlorophyl	0	NA	NA	NA	NA	NA
1.1.1.43	phosphogluconate 2-dehydrogenase	0	NA	NA	NA	NA	NA
3.2.1.2	beta-amylase	0	NA	NA	NA	NA	NA
2.4.1.87	N-acetyllactosaminide 3-alpha-galactosyltransferase	0	NA	NA	NA	NA	NA
3.7.1.8	2,6-dioxo-6-phenylhexa-3-enoate hydrolase	0	NA	NA	NA	NA	NA
1.8.-.-	#N/A	0	NA	NA	NA	NA	NA
3.1.3.21	glycerol-1-phosphatase	0	NA	NA	NA	NA	NA
2.3.1.168	2-oxoisovalerate dehydrogenase E2 component (dihydrolipoyl t	0	NA	NA	NA	NA	NA
3.4.23.52	archaeal preflagellin peptidase FlaK	0	NA	NA	NA	NA	NA
2.7.1.3	ketohexokinase	0	NA	NA	NA	NA	NA
1.8.2.3	sulfide dehydrogenase [flavocytochrome c] flavoprotein chain	0	NA	NA	NA	NA	NA
2.3.1.109	arginine N-succinyltransferase	0	NA	NA	NA	NA	NA
2.3.2.16	peptidoglycan pentaglycine glycine transferase (the first glycine	0	NA	NA	NA	NA	NA
2.7.1.76	deoxyadenosine kinase	0	NA	NA	NA	NA	NA
1.13.11.16	2,3-dihydroxyphenylpropionate 1,2-dioxygenase	0	NA	NA	NA	NA	NA
4.1.1.77	2-oxo-3-hexenedioate decarboxylase	0	NA	NA	NA	NA	NA
1.3.8.-	#N/A	0	NA	NA	NA	NA	NA
2.1.1.152	precorrin-6A synthase	0	NA	NA	NA	NA	NA
1.20.4.3	mycoredoxin	0	NA	NA	NA	NA	NA
1.2.1.31	L-aminoadipate-semialdehyde dehydrogenase	0	NA	NA	NA	NA	NA
6.3.4.22	tRNA(Ile2)-agmatinylcytidine synthase	0	NA	NA	NA	NA	NA
1.3.5.5	15-cis-phytoene desaturase	0	NA	NA	NA	NA	NA
4.2.1.25	L-arabonate dehydrase	0	NA	NA	NA	NA	NA
1.2.99.8	glyceraldehyde dehydrogenase large subunit	0	NA	NA	NA	NA	NA

1.13.11.54	1,2-dihydroxy-3-keto-5-methylthiopentene dioxygenase	0	NA	NA	NA	NA	NA

Table S17: Enzymatic Functions (EC_DGE), Standard Diet, Mid in Mission

	Description	baseMean	log2FoldChange	lfcSE	stat	pvalue	padj
1.14.12.19	3-phenylpropionate/trans-cinnamate dioxygenase subunit alph	1.118856663	-17.75912223	4.128566662	-4.301522461	1.70E-05	0.014989063
1.3.99.4	3-oxosteroid 1-dehydrogenase	0.726380301	-17.09349099	4.115967225	-4.152970627	3.28E-05	0.014989063
1.3.1.104	enoyl-[acyl-carrier protein] reductase III	0.779460713	16.90612469	4.101824056	4.121611375	3.76E-05	0.014989063
6.5.1.4	RNA 3'-terminal phosphate cyclase (ATP)	0.559597965	-17.36135882	4.125852642	-4.207944472	2.58E-05	0.014989063
3.2.1.64	levanbiose-producing levanase	0.405616982	17.39254106	4.099047632	4.243068786	2.20E-05	0.014989063
1.2.1.58	phenylglyoxylate dehydrogenase alpha subunit	0.14203695	16.4655732	4.119130275	3.997342181	6.41E-05	0.021267147
3.1.3.23	sugar-phosphatase	0.6779552	-15.65434085	4.131559242	-3.788966814	1.51E-04	0.043048577
3.6.4.12	DNA replication licensing factor MCM5	12313.76222	2.291699347	0.623801704	3.673762565	2.39E-04	0.059512205
1.5.1.-	#N/A	0.239309938	-14.72122068	4.141839411	-3.554271235	3.79E-04	0.082505764
1.16.1.9	ferric-chelate reductase (NADPH)	0.390839791	-14.60305341	4.135815758	-3.530876195	4.14E-04	0.082505764
3.4.22.41	cathepsin F	0.390452259	-14.15400667	4.139372617	-3.419360368	6.28E-04	0.113668116
4.2.1.5	D-arabinonate dehydratase	0.322417442	-13.72790313	4.138974616	-3.316740111	9.11E-04	0.139553838
1.8.1.9	thioredoxin reductase (NADPH)	2323.064914	1.593643632	0.477645288	3.336458395	8.49E-04	0.139553838
1.18.1.1	rubredoxin---NAD+ reductase	51.1723042	-2.02955949	0.654248036	-3.102125462	1.92E-03	0.273382804
4.1.1.3	#N/A	2726.451397	1.763325896	0.582158852	3.028942854	2.45E-03	0.32590594
1.8.1.7	glutathione reductase (NADPH)	5.508827407	-4.680693341	1.571034515	-2.979370151	2.89E-03	0.35960782
2.2.1.7	1-deoxy-D-xylulose-5-phosphate synthase	675.3423551	0.543069368	0.184424772	2.94466607	3.23E-03	0.378835501
3.6.1.25	triphosphatase	0.176081768	-11.81318145	4.143731151	-2.850856153	4.36E-03	0.482525366
2.7.1.92	5-dehydro-2-deoxygluconokinase	11.52794472	-3.024302447	1.07996226	-2.800377903	5.10E-03	0.519061265
4.4.1.11	methionine-gamma-lyase	52.03597116	1.164236512	0.416741771	2.79366407	5.21E-03	0.519061265
1.2.1.43	#N/A	5.915036706	2.746447727	1.025006419	2.679444417	7.37E-03	0.671762505
5.1.3.30	D-psicose/D-tagatose/L-ribulose 3-epimerase	12.77438443	-1.859379148	0.698358787	-2.662498393	7.76E-03	0.671762505
5.1.3.31	D-psicose/D-tagatose/L-ribulose 3-epimerase	12.77438443	-1.859379148	0.698358787	-2.662498393	0.007756294	0.671762505
3.4.24.13	IgA-specific metalloendopeptidase	8.078081151	-6.048172299	2.30971771	-2.618576406	0.008829752	0.684013219
3.2.1.49	alpha-N-acetylgalactosaminidase	2.267948236	-10.83504221	4.143731151	-2.614803379	8.93E-03	0.684013219
2.3.1.118	N-hydroxyarylamine O-acetyltransferase	6.247718801	-2.87979286	1.099095698	-2.620147513	0.008789174	0.684013219
1.12.2.1	NiFe] hydrogenase large subunit	10.41753835	2.900845811	1.12908543	2.56919958	0.010193373	0.752044424
1.2.3.3	pyruvate oxidase	5.87582389	-5.282083922	2.072188482	-2.549036425	0.010802102	0.768492379
6.3.1.12	D-aspartate ligase	24.89074862	-1.231463366	0.490687147	-2.509671127	0.012084365	0.830070847
2.3.1.81	aminoglycoside 3-N-acetyltransferase	34.85777649	2.139983218	0.862716096	2.480518478	0.013119146	0.871111321
2.1.1.13	5-methyltetrahydrofolate--homocysteine methyltransferase	580.1326798	0.996848928	0.407454019	2.446531099	0.014423835	0.882113649
1.1.1.103	threonine 3-dehydrogenase	279.8371802	1.42845553	0.584995639	2.441822527	0.014613329	0.882113649
4.4.1.5	lactoylglutathione lyase	1030.235915	1.25961549	0.511741112	2.461431105	0.013838397	0.882113649
3.2.1.4	endoglucanase	673.5239206	1.055524809	0.435362721	2.424472189	1.53E-02	0.890277575
4.1.3.34	citrate lyase subunit beta / cityl-CoA lyase	77.48266292	0.90929006	0.376182205	2.417153308	0.015642427	0.890277575
3.5.1.91	N-substituted formamide deformylase	129.8540039	0.645563606	0.271767459	2.375426434	0.017528686	0.969920634
2.1.1.22	carnosine N-methyltransferase	0.929084248	-0.224101715	4.102188328	-0.054629797	0.9564334	0.999677092
5.3.1.29	ribose 1,5-bisphosphate isomerase	1.243333821	-1.325856295	3.899987068	-0.33996428	0.733883428	0.999677092
4.4.1.25	L-cysteate sulfo-lyase	2.615552796	1.433781784	2.032610906	0.705389202	0.480568062	0.999677092
3.2.1.99	arabinan endo-1,5-alpha-L-arabinosidase	139.1340885	-0.073225882	0.562501573	-0.130178982	0.896424823	0.999677092
2.8.1.7	cysteine desulfurase	1139.077994	0.006492229	0.240121592	0.027037257	0.978430018	0.999677092
3.4.22.-	#N/A	6.960518191	-1.625160063	2.007200198	-0.809665156	0.418132649	0.999677092
2.3.1.7	carnitine O-acetyltransferase	0.017504292	0.120319428	4.141980666	0.029048766	0.976825697	0.999677092
1.14.11.-	#N/A	0.589631369	-1.435137827	4.106982896	-0.349438472	0.726760155	0.999677092
2.5.1.9	#N/A	543.7887389	1.192456539	0.797597885	1.495059806	0.134898835	0.999677092
3.2.1.22	alpha-galactosidase	682.0919527	0.035509334	0.337079513	0.105344089	0.91610278	0.999677092
5.3.1.17	4-deoxy-L-threo-5-hexosulose-uronate ketol-isomerase	242.425249	0.127263891	0.341532085	0.372626457	0.709426479	0.999677092
3.2.1.132	chitosanase	0.184986723	-1.144343231	4.127511293	-0.277247753	0.781589875	0.999677092
3.1.3.18	phosphoglycolate phosphatase	780.8428987	-0.498646508	0.460410219	-1.083048306	0.278786982	0.999677092
1.3.8.6	glutaryl-CoA dehydrogenase	0.112273496	-0.413755952	4.138007264	-0.09998918	0.920352916	0.999677092
1.1.1.44	6-phosphogluconate dehydrogenase	386.0194263	-0.440004684	0.652013012	-0.674840343	0.499777206	0.999677092
6.1.1.14	#N/A	630.4145281	0.198159748	0.233874013	0.847292718	0.396831985	0.999677092
2.5.1.47	cysteine synthase	480.1082915	0.505551033	0.377369872	1.339669833	0.180352709	0.999677092
6.3.2.7	UDP-N-acetylmuramoyl-L-alanyl-D-glutamate-L-lysine ligase	4.22944368	-0.37946988	1.762686123	-0.215279326	0.829549547	0.999677092
3.5.2.10	creatinine amidohydrolase	77.38836357	0.048598876	0.513760819	0.094594361	0.924637029	0.999677092
4.2.2.20	chondroitin-sulfate-ABC endolyase/exolyase	92.73215075	0.350250927	0.633520791	0.552864139	0.580356445	0.999677092
1.1.1.6	glycerol dehydrogenase	131.5645382	-0.485377446	0.482127612	-1.00674061	0.314059442	0.999677092
2.7.7.70	D-beta-D-heptose 7-phosphate kinase / D-beta-D-heptose 1-ph	30.61416296	-0.374467891	0.695175583	-0.538666634	0.590116901	0.999677092
6.1.1.11	seryl-tRNA synthetase	599.7939619	0.304664727	0.213323103	1.428184395	0.153238791	0.999677092
3.1.1.41	cephalosporin-C deacetylase	12.9623897	-1.042286944	0.776159671	-1.342876966	0.179311816	0.999677092
2.5.1.39	4-hydroxybenzoate polyprenyltransferase	7.596073966	0.330680037	1.105231764	0.299195198	0.764791113	0.999677092
2.6.1.37	2-aminoethylphosphonate-pyruvate transaminase	28.43514803	-0.745700019	0.893295933	-0.834773776	0.403845094	0.999677092
2.1.1.-	#N/A	1033.626585	0.097203892	0.235245588	0.413201766	0.679458794	0.999677092
1.5.1.43	carboxynorspermidine synthase	0.131959613	0.062946437	4.142513233	0.015195229	0.987876428	0.999677092
2.3.3.13	2-isopropylmalate synthase	379.9594429	-0.003218899	0.158459804	-0.02031366	0.983793159	0.999677092
3.5.1.28	N-acetylmuramoyl-L-alanine amidase	773.0604699	-0.206956566	0.189505855	-1.092085336	0.274795594	0.999677092
2.4.2.3	uridine phosphorylase	240.7680276	-0.248758455	0.266474628	-0.933516472	0.35055337	0.999677092
3.4.19.5	beta-aspartyl-peptidase (threonine type)	19.4807214	-0.168009264	0.534278122	-0.314460311	0.753171456	0.999677092
1.15.1.2	superoxide reductase	1449.052551	-0.053644708	0.620322297	-0.086478768	0.931085833	0.999677092
2.4.1.20	cellobiose phosphorylase	219.5275666	0.366962764	0.360257646	1.018612008	0.308387201	0.999677092
3.1.11.6	#N/A	611.926668	-0.014636196	0.179857657	-0.081376555	0.935142494	0.999677092
1.3.1.106	precorrin-6A/cobalt-precorrin-6A reductase	80.75062147	0.078017744	0.606384038	0.128660617	0.897626199	0.999677092
2.8.4.5	threonylcarbamoyladenosine tRNA methylthiotransferase CDK/	336.5404218	0.450097761	0.314332116	1.431917828	0.152167339	0.999677092
2.7.7.23	UDP-N-acetylglucosamine/UDP-N-acetylgalactosamine diphosp	466.8862771	0.668208972	0.430256512	1.553047896	0.120411692	0.999677092
4.1.1.23	orotidine-5'-phosphate decarboxylase	361.1107121	-0.094833753	0.220922199	-0.429263125	0.667731749	0.999677092
1.1.1.270	3-keto steroid reductase	0.499547738	-0.762859882	4.108896089	-0.185660544	0.852710972	0.999677092
2.7.4.2	phosphomevalonate kinase	4.874322445	-1.618332423	1.513767952	-1.069075627	0.285035593	0.999677092
3.5.1.54	allophanate hydrolase	0.548905896	-1.584069943	4.105134529	-0.385875282	0.699589038	0.999677092
4.2.1.49	urocanate hydratase	221.4685891	0.589432378	0.429445265	1.372543664	0.169894256	0.999677092
2.4.1.44	UDP-D-galactose:(glucosyl)LPS alpha-1,3-D-galactosyltransferas	0.907193448	-2.135535526	4.099797545	-0.520888045	0.602444764	0.999677092
4.2.1.42	galactarate dehydratase	4.67297282	-1.233683833	1.290454145	-0.956007493	0.339068449	0.999677092
2.5.1.26	alkyldihydroxyacetonephosphate synthase	19.14698164	0.149613295	0.769877501	0.194333897	0.845914446	0.999677092
2.7.7.24	glucose-1-phosphate thymidylyltransferase	376.3120803	0.164421127	0.237303608	0.692872429	0.488389613	0.999677092
5.4.99.60	precorrin-8X/cobalt-precorrin-8 methylmutase	102.094744	-0.078547974	0.50764316	-0.154730686	0.877033637	0.999677092
2.4.2.19	nicotinate-nucleotide pyrophosphorylase (carboxylating)	246.5480813	-0.24797447	0.310855938	-0.797715084	0.425035849	0.999677092

2.1.1.187	23S rRNA (guanine745-N1)-methyltransferase	129.5080327	0.625340562	0.359259465	1.740637683	0.081747108	0.999677092
3.4.11.18	methionyl aminopeptidase	1044.301231	0.121005945	0.317900388	0.380641074	0.703469599	0.999677092
2.1.1.204	tRNA (cytosine38-C5)-methyltransferase	0.144519491	-0.326039106	4.131567667	-0.078914139	0.937100917	0.999677092
2.7.1.66	undecaprenol kinase	2.161074354	-1.305750182	1.896896942	-0.688361161	0.491225375	0.999677092
2.6.1.1	aspartate aminotransferase, chloroplastic	520.0808933	-0.015841115	0.260159071	-0.060890114	0.951446723	0.999677092
4.3.1.19	threonine dehydratase	227.9571154	-0.091487991	0.296210493	-0.308861412	0.757426951	0.999677092
3.5.1.10	formyltetrahydrofolate deformylase	24.4019335	-0.92712077	0.966342796	-0.959411891	0.337351287	0.999677092
3.7.1.12	cobalt-precorrin 5A hydrolase	99.95703726	0.089792987	0.434234619	0.206784496	0.836178164	0.999677092
1.1.1.62	17beta-estradiol 17-dehydrogenase	0.771316995	-0.055954859	4.105202021	-0.013630233	0.989124985	0.999677092
1.3.3.4	#N/A	12.23533618	0.925446949	1.111289246	0.832768743	0.404975166	0.999677092
3.5.4.16	GTP cyclohydrolase IA	161.7547469	-0.555602154	0.457590879	-1.214189746	0.224675275	0.999677092
2.1.1.200	tRNA (cytidine32/uridine32-2'-O)-methyltransferase	3.219890731	-1.333767977	2.949845029	-0.45214849	0.651162013	0.999677092
5.3.1.6	#N/A	479.29167	-0.061264483	0.222687175	-0.275114556	0.783228229	0.999677092
1.1.1.335	UDP-N-acetyl-2-amino-2-deoxyglucuronate dehydrogenase	3.835544045	1.246139029	1.760478809	0.70784097	0.47904402	0.999677092
1.1.1.350	ureidoglycolate dehydrogenase (NAD+)	2.906536393	-3.083345011	2.026385637	-1.521598335	0.128109755	0.999677092
2.3.1.181	lipoyl(octanoyl) transferase	98.92787013	-0.298861339	0.700344341	-0.426734852	0.669572463	0.999677092
1.2.1.18	Oxidoreductases	18.35639239	0.338433957	0.569460393	0.594306401	0.552307198	0.999677092
2.1.1.104	caffeoyl-CoA O-methyltransferase	0.263418337	-0.008225558	4.140199955	-0.001986754	0.998414801	0.999677092
5.4.2.8	phosphomannomutase	156.1336159	-0.016687515	0.323558842	-0.051574901	0.958867419	0.999677092
1.2.99.5	#N/A	30.58832785	0.382312134	0.827819008	0.461830582	0.644202818	0.999677092
2.7.1.191	PTS system, mannose-specific IIA component	117.6263563	-1.021496347	0.456657263	-2.236899374	0.025292917	0.999677092
2.5.1.63	adenosyl-fluoride synthase	4.321299614	-1.388500456	1.824492701	-0.761033714	0.446636931	0.999677092
2.8.3.-	#N/A	14.81102674	-1.206449463	0.633162056	-1.905435505	0.056723484	0.999677092
2.7.8.11	CDP-diacylglycerol--inositol 3-phosphatidyltransferase	0.582805694	-0.770921528	4.106500735	-0.187731983	0.851086757	0.999677092
1.12.98.1	#N/A	28.04594534	1.136225607	1.17536706	0.966698529	0.333694763	0.999677092
3.2.1.106	mannosyl-oligosaccharide glucosidase	0.284782452	-0.710913002	4.120484219	-0.172531422	0.86301976	0.999677092
5.4.99.26	tRNA pseudouridine65 synthase	1.975607773	1.992555674	1.969183768	1.01186883	0.311600775	0.999677092
1.4.1.2	glutamate dehydrogenase	48.68472367	-0.531034705	0.844951038	-0.628479854	0.52968964	0.999677092
3.1.21.1	deoxyribonuclease I	1.14797295	0.026791388	4.056019617	0.00660534	0.99472974	0.999677092
2.7.7.83	UDP-N-acetylglucosamine/UDP-N-acetylgalactosamine diphosp	41.03166629	0.35734747	0.844336037	0.423228968	0.672128213	0.999677092
2.6.1.62	adenosylmethionine---8-amino-7-oxononanoate aminotransfer	73.02597664	-0.651564166	0.734725392	-0.886813187	0.375179487	0.999677092
1.8.3.2	thiol oxidase	3.056885843	-0.045391919	3.719812242	-0.012202745	0.99026386	0.999677092
1.1.1.310	(S)-sulfolactate dehydrogenase	0.458766862	-1.266618647	4.13040249	-0.306657438	0.759104129	0.999677092
2.6.1.9	histidinol-phosphate aminotransferase	241.1110682	-0.077171495	0.19468372	-0.396394189	0.691814252	0.999677092
3.1.4.53	3',5'-cyclic-AMP phosphodiesterase	0.527495585	-1.630578817	4.119399541	-0.395829247	0.692231	0.999677092
3.2.1.26	beta-fructofuranosidase	668.236276	-0.179603468	0.43256569	-0.415205071	0.677991787	0.999677092
1.4.3.5	pyridoxamine 5'-phosphate oxidase	196.6921172	-1.6392988	0.838072751	-1.956034005	0.050461148	0.999677092
4.2.1.3	#N/A	1254.902064	0.882938835	0.56630071	1.559134252	0.118964608	0.999677092
5.1.3.23	UDP-GlcNAc3NAcA epimerase	1.804103656	0.144984327	2.01598235	0.071917459	0.942667596	0.999677092
4.2.3.4	3-dehydroquinate synthase	263.3059402	0.588550448	0.305797198	1.924643037	0.054274031	0.999677092
2.4.2.30	poly [ADP-ribose] polymerase 16	1.661425333	-0.078471758	2.297845573	-0.034150144	0.972757423	0.999677092
6.3.2.32	gamma-F420-2:alpha-L-glutamate ligase	0.812016276	-0.233456375	4.101177527	-0.056924231	0.954605552	0.999677092
1.3.98.3	oxygen-independent coproporphyrinogen III oxidase	570.8711187	0.595811676	0.259624335	2.294899195	0.021738902	0.999677092
2.4.1.256	alpha-1,2-glucosyltransferase	0.333031825	-0.735914733	4.11703374	-0.178748774	0.85813497	0.999677092
2.1.1.207	tRNA (cytidine/uridine-2'-O-)-methyltransferase	169.8966336	-0.67170236	0.402495759	-1.668843323	0.095148434	0.999677092
1.1.1.9	D-xylulose reductase	0.801105635	0.971718799	4.091637408	0.237488981	0.812277467	0.999677092
3.5.3.9	allantoate deiminase	14.3761206	-0.231223548	0.646320947	-0.357753449	0.72052783	0.999677092
1.4.1.13	glutamate synthase (NADPH) large chain	1037.619229	-0.044246315	0.172034442	-0.257194516	0.797028618	0.999677092
4.2.1.76	UDP-glucose 4,6-dehydratase	1.498656123	-0.482014449	4.098478777	-0.117608136	0.906378158	0.999677092
1.11.1.9	glutathione peroxidase	87.79499751	0.114255481	0.499612942	0.228687993	0.819111428	0.999677092
3.1.1.56	carboxylesterase 2	0.167196626	-3.703195448	4.143731151	-0.893686225	0.371489798	0.999677092
4.1.3.-	#N/A	225.5498462	-0.19602226	0.249981322	-0.784147625	0.432953493	0.999677092
3.4.23.36	signal peptidase II	249.2065007	-0.098518642	0.243677784	-0.404298826	0.685992988	0.999677092
4.1.1.86	L-2,4-diaminobutyrate decarboxylase	0.110533983	-0.307374933	4.142162324	-0.074206395	0.940846158	0.999677092
2.4.99.-	#N/A	0.084668419	1.508835902	4.130322632	0.365307032	0.71488223	0.999677092
2.4.1.211	1,3-beta-galactosyl-N-acetylhexosamine phosphorylase	886.5408123	-0.302006929	0.634727673	-0.475805518	0.634212938	0.999677092
5.5.1.4	myo-inositol-1-phosphate synthase	93.74847179	-0.456346877	0.372933765	-1.223667364	0.221077736	0.999677092
4.1.1.96	carboxynorspermidine decarboxylase	158.8615104	0.258250112	0.438080205	0.589504181	0.555523108	0.999677092
2.5.1.32	15-cis-phytoene synthase	4.713411545	-0.060390091	1.670026605	-0.036161155	0.971153859	0.999677092
3.5.1.1	L-asparaginase	366.752951	0.609647938	0.339874765	1.793742873	0.072854219	0.999677092
5.3.1.-	#N/A	0.594915199	0.033313346	4.048382251	0.008228804	0.993434438	0.999677092
3.4.17.-	#N/A	57.62759611	0.199698362	0.543815951	0.367216816	0.713457297	0.999677092
3.4.24.37	saccharolysin	0.04233421	1.159403773	4.143245313	0.279829864	0.779608038	0.999677092
6.2.1.-	#N/A	80.6595248	-0.323047421	0.42660489	-0.757252035	0.448898881	0.999677092
3.5.1.42	nicotinamide-nucleotide amidase	507.4585151	0.339851167	0.379930849	0.894507957	0.371050174	0.999677092
6.2.1.4	#N/A	20.93996699	-1.18996765	1.513779681	-0.786090383	0.431814538	0.999677092
1.17.3.2	xanthine dehydrogenase/oxidase	0.020495196	0.108319359	4.142686886	0.026147127	0.979139988	0.999677092
1.17.7.1	(E)-4-hydroxy-3-methylbut-2-enyl-diphosphate synthase	363.237913	-0.050042854	0.164822796	-0.303616095	0.761420388	0.999677092
4.1.3.41	3-hydroxy-D-aspartate aldolase	0.311717752	-0.430594502	4.130682839	-0.104242935	0.916976562	0.999677092
4.2.1.51	cyclohexadienyl dehydratase	217.7655411	0.000321842	0.311356494	0.001033676	0.999175246	0.999677092
1.3.1.28	2,3-dihydro-2,3-dihydroxybenzoate dehydrogenase	0.630791762	-1.354049101	4.121489598	-0.328533912	0.742508008	0.999677092
2.4.1.180	UDP-N-acetyl-D-mannosaminouronate:lipid I N-acetyl-D-manno	4.766590627	-0.301298815	2.006979263	-0.150125525	0.880665583	0.999677092
3.2.1.18	sialidase-1	722.2496707	-1.041438209	0.634193546	-1.642145708	0.100559811	0.999677092
3.6.1.42	guanosine-diphosphatase	0.312227018	-3.793965404	4.143731151	-0.915591593	0.359881143	0.999677092
6.3.5.5	carbamoyl-phosphate synthase	1356.851513	-0.140109271	0.177448615	-0.78957658	0.42977509	0.999677092
2.1.1.222	2-polyprenyl-6-hydroxyphenyl methylase / 3-demethylubiquino	0.340645926	0.100068475	4.122469961	0.024273912	0.980634122	0.999677092
3.4.25.2	ATP-dependent HslUV protease, peptidase subunit HslV	11.0559166	-0.019697545	0.980644689	-0.020086322	0.983974511	0.999677092
3.1.22.-	#N/A	0.333031825	-0.735914733	4.11703374	-0.178748774	0.85813497	0.999677092
2.7.1.20	adenosine kinase	2.502983657	1.395679871	1.647226512	0.847290801	0.396833053	0.999677092
1.12.7.2	ferredoxin hydrogenase	22.43350305	-0.040641247	0.516130791	-0.078742148	0.93723772	0.999677092
2.8.3.22	succinyl-CoA:(S)-malate CoA-transferase subunit A	0.128757426	-0.558067872	4.134677189	-0.134972538	0.892633587	0.999677092
1.2.1.-	#N/A	0.816141609	-0.010141037	3.849363502	-0.002634471	0.997897999	0.999677092
3.4.14.5	dipeptidyl-peptidase 4	252.4844495	-0.07772015	0.519928229	-0.149482458	0.88117295	0.999677092
3.1.3.26	4-phytase / acid phosphatase	3.711875381	2.179692172	1.253059111	1.739496687	0.081947434	0.999677092
1.14.11.47	50S ribosomal protein L16 3-hydroxylase	3.642950035	-1.559142921	1.306432982	-1.193435058	0.232699032	0.999677092
2.7.7.64	UDP-sugar pyrophosphorylase	2.795318261	0.052761249	3.686434338	0.014312271	0.988580849	0.999677092
2.1.1.77	protein-L-isoaspartate(D-aspartate) O-methyltransferase	1.597836612	0.265731667	2.561793649	0.103728755	0.917384606	0.999677092

4.1.1.36	phosphopantothenoylcysteine decarboxylase	273.1904406	0.044558947	0.163070143	0.273250187	0.784660904	0.999677092
1.2.1.2	#N/A	245.2872466	-0.107526909	0.521554	-0.206166398	0.836660935	0.999677092
1.1.1.67	mannitol 2-dehydrogenase	4.516882265	-0.192704722	1.698095959	-0.113482822	0.909647781	0.999677092
3.2.1.185	non-reducing end beta-L-arabinofuranosidase	0.714364175	0.415529033	4.07214669	0.102041764	0.918723525	0.999677092
2.7.8.7	holo-[acyl-carrier protein] synthase	35.60714793	0.03138502	0.572405867	0.054830011	0.956273891	0.999677092
2.3.1.201	UDP-2-acetamido-3-amino-2,3-dideoxy-glucuronate N-acetyltra	22.62710242	1.196339088	0.774754065	1.544153354	0.122551186	0.999677092
5.99.1.3	#N/A	3660.059785	-0.30125408	0.536412263	-0.561609234	0.574382288	0.999677092
6.3.2.1	pantoate--beta-alanine ligase	150.1648978	-0.138563385	0.364222944	-0.380435628	0.703622072	0.999677092
4.3.1.12	ornithine cyclodeaminase	24.93366618	-0.373693265	0.524485267	-0.712495257	0.476158147	0.999677092
2.1.1.14	5-methyltetrahydropteroyltriglutamate--homocysteine methylt	94.21501856	-0.764307264	0.53465233	-1.429540696	0.152848887	0.999677092
2.4.1.131	#N/A	1.764497646	0.159371268	3.529714533	0.045151319	0.963986696	0.999677092
2.1.3.3	ornithine carbamoyltransferase	321.7840829	-0.124682309	0.23148587	-0.538617362	0.590150905	0.999677092
2.1.1.246	[methyl-Co(III) methanol-specific corrinoid protein]:coenzyme M	5.57705486	-0.058909491	1.165654302	-0.050537703	0.959693905	0.999677092
2.6.1.81	succinylornithine aminotransferase	0.203337428	-0.641180367	4.127546313	-0.155341774	0.876551884	0.999677092
5.4.99.5	chorismate mutase	299.5030678	-0.072672861	0.275057934	-0.26420929	0.791618653	0.999677092
2.7.7.12	UDPglucose--hexose-1-phosphate uridylyltransferase	348.5210504	-0.182854269	0.289390818	-0.631859264	0.527478844	0.999677092
5.2.1.13	prolycopene isomerase	1.503143303	1.273667643	3.084381282	0.412941049	0.679649805	0.999677092
6.3.4.15	biotin---protein ligase	359.9504088	-0.117773246	0.226308508	-0.520410158	0.602777732	0.999677092
3.1.3.-	#N/A	200.6898365	0.133675395	0.267262725	0.500164752	0.616959075	0.999677092
2.5.1.72	quinolinate synthase	156.0969679	-0.441120274	0.210786666	-2.092733297	0.036372969	0.999677092
1.8.4.8	phosphoadenosine phosphosulfate reductase	38.80354416	-0.535091701	1.100209927	-0.486354184	0.626716069	0.999677092
2.3.1.20	diacylglycerol O-acyltransferase / wax synthase	1.228064538	-0.497384623	4.099653058	-0.121323589	0.903434737	0.999677092
1.5.1.28	opine dehydrogenase	8.554545849	-0.727284073	0.825928319	-0.880565609	0.37855298	0.999677092
2.7.4.16	thiamine-monophosphate kinase	118.4753888	0.127213835	0.378533382	0.33607032	0.736817839	0.999677092
1.1.1.125	#N/A	21.0930698	-0.487677844	0.643261945	-0.758132589	0.448371612	0.999677092
2.6.1.85	para-aminobenzoate synthetase component II	61.58760241	-0.204357923	0.616795544	-0.331321983	0.740401288	0.999677092
4.1.99.1	tryptophanase	227.0207991	0.144266117	0.455217035	0.316917218	0.751306417	0.999677092
4.4.1.16	selenocysteine lyase	255.398513	-0.060953903	0.338198083	-0.180231369	0.856970933	0.999677092
2.7.1.45	2-dehydro-3-deoxygluconokinase	519.2465247	0.084436185	0.247352103	0.341360288	0.732832367	0.999677092
2.4.2.6	nucleoside deoxyribosyltransferase	0.102241287	0.022959341	4.141140062	0.005544208	0.995576385	0.999677092
5.3.3.-	#N/A	0.157074332	0.448526278	4.143731151	0.108242128	0.913803628	0.999677092
3.4.22.47	gingipain K	1.00454257	4.771412883	4.03377309	1.182865961	0.236862274	0.999677092
3.1.27.6	ribonuclease I (enterobacter ribonuclease)	0.207448764	-4.061570657	4.143731151	-0.98017234	0.327001056	0.999677092
4.3.3.7	4-hydroxy-tetrahydrodipicolinate synthase	488.6728682	-0.262155909	0.215896695	-1.214265507	0.224646353	0.999677092
1.1.1.367	UDP-2-acetamido-2,6-beta-L-arabino-hexul-4-ose reductase	89.12835807	0.098170491	0.41820676	0.234741521	0.814409359	0.999677092
2.7.7.42	[glutamine synthetase] adenylyltransferase / [glutamine synthe	10.5009941	1.114375934	0.970245138	1.148550907	0.25074121	0.999677092
5.1.3.11	cellobiose epimerase	95.78211526	0.19620468	0.599618324	0.327215951	0.743504557	0.999677092
1.6.3.3	NADH oxidase (H2O2-forming)	0.663509303	-2.012913076	4.110975705	-0.489643632	0.624386096	0.999677092
2.6.1.19	4-aminobutyrate aminotransferase	27.32058772	-0.107284956	0.574001319	-0.18690716	0.851733425	0.999677092
2.1.2.11	3-methyl-2-oxobutanoate hydroxymethyltransferase	194.9990628	-0.112693906	0.328499104	-0.343056965	0.731555605	0.999677092
5.4.3.5	D-ornithine 4,5-aminomutase subunit beta	35.04208565	0.825870564	0.924554901	0.89326287	0.371716417	0.999677092
2.6.1.21	D-alanine transaminase	38.44577383	0.186057055	0.435193962	0.427526738	0.668995714	0.999677092
2.7.8.6	undecaprenyl-phosphate galactose phosphotransferase	5.98517338	0.881949406	0.893198031	0.987406349	0.323443473	0.999677092
5.4.99.22	23S rRNA pseudouridine2605 synthase	322.0947105	-0.094912114	0.297406127	-0.319133016	0.749625648	0.999677092
1.1.1.130	3-dehydro-L-gulonate 2-dehydrogenase	10.5048034	-0.632526396	0.726255252	-0.870942268	0.383785676	0.999677092
2.7.4.26	isopentenyl phosphate kinase	0.374667258	-0.245353706	4.118597902	-0.059572144	0.952496405	0.999677092
1.3.5.3	menaquinone-dependent protoporphyrinogen oxidase	14.33543221	-0.872026425	0.862237185	-1.011353303	0.311847363	0.999677092
1.1.1.90	aryl-alcohol dehydrogenase	0.095768651	-0.623651221	4.136129673	-0.150781351	0.880148198	0.999677092
3.4.11.21	aspartyl aminopeptidase	189.1715901	0.191395848	0.323229139	0.592136738	0.553759027	0.999677092
3.4.21.-	#N/A	392.414596	-0.004125131	0.267359224	-0.01542917	0.987689792	0.999677092
2.3.1.222	phosphate propanoyltransferase	0.103005941	-0.436320317	4.137063428	-0.10546619	0.916005897	0.999677092
2.4.1.144	beta-1,4-mannosyl-glycoprotein beta-1,4-N-acetylglucosaminyl	0.830970326	-3.29868788	4.095811001	-0.805380883	0.42059992	0.999677092
1.1.1.83	tartrate dehydrogenase/decarboxylase / D-malate dehydrogena	5.410002282	0.263459365	0.972147822	0.271007514	0.786385253	0.999677092
2.1.1.173	23S rRNA (guanine2445-N2)-methyltransferase / 23S rRNA (gua	1.313961173	-2.947557179	2.492975776	-1.182344894	0.237068879	0.999677092
1.1.1.287	D-arabinitol dehydrogenase (NADP+)	0.799170637	0.391978567	4.093568084	0.095754745	0.923715361	0.999677092
6.1.1.-	#N/A	6.459165869	0.646545751	1.131560661	0.571375246	0.567745305	0.999677092
6.4.1.3	acetyl-CoA/propionyl-CoA carboxylase	273.9201141	-0.739849632	0.459575498	-1.609854386	0.107429649	0.999677092
1.7.2.2	nitrite reductase (cytochrome c-552)	49.12275699	-0.301608398	0.44710548	-0.674579963	0.499942667	0.999677092
5.4.4.2	salicylate biosynthesis isochorismate synthase	61.24446731	-0.419188524	0.438930889	-0.9550217	0.339566723	0.999677092
2.7.7.50	mRNA guanylyltransferase	1.392230011	-0.411377067	4.099130428	-0.100357155	0.920060783	0.999677092
3.1.4.14	acyl carrier protein phosphodiesterase	0.051862191	0.448527249	4.143731151	0.108242362	0.913803442	0.999677092
2.3.1.8	phosphate acetyltransferase	571.8730186	-0.119869311	0.397397937	-0.301635464	0.76292997	0.999677092
3.1.13.3	#N/A	386.1764493	0.257147061	0.236848723	1.085701701	0.277610988	0.999677092
2.1.1.182	16S rRNA (adenine1518-N6/adenine1519-N6)-dimethyltransfer	820.9449571	0.096196588	0.641845643	0.149874957	0.88086327	0.999677092
4.2.2.2	pectate lyase	2.166420747	2.807660973	2.418418918	1.160948979	0.245662649	0.999677092
2.7.3.9	#N/A	954.3616646	-0.348848382	0.448904744	-0.777110038	0.437093852	0.999677092
4.3.1.3	histidine ammonia-lyase	217.1796459	0.280697567	0.371676672	0.755219762	0.450117133	0.999677092
3.1.13.1	exoribonuclease II	7.538701321	-0.390867062	1.119656784	-0.349095426	0.72701767	0.999677092
2.1.1.297	release factor glutamine methyltransferase	340.1934048	0.188863427	0.176002841	1.07307033	0.283239562	0.999677092
1.3.3.11	pyrroloquinoline-quinone synthase	0.110533983	-0.307374933	4.142162324	-0.074206395	0.940846158	0.999677092
3.6.3.10	H+/K+-exchanging ATPase	3.674943414	-1.205772659	3.880218703	-0.310748633	0.755991722	0.999677092
3.5.1.87	beta-ureidopropionase / N-carbamoyl-L-amino-acid hydrolase	41.46682057	0.105298839	0.481796007	0.218554819	0.826996851	0.999677092
3.4.21.62	subtilisin	0.102172711	1.178640217	4.125609384	0.285688757	0.775116517	0.999677092
3.1.1.29	peptidyl-tRNA hydrolase, PTH1 family	279.5423892	-0.405253145	0.336266187	-1.205155797	0.228143163	0.999677092
6.4.1.4	#N/A	2.745395653	-0.319016475	3.73007486	-0.085525489	0.931843633	0.999677092
3.5.1.110	ureidoacrylate peracid hydrolase	0.074204671	0.448523946	4.143731151	0.108241565	0.913804074	0.999677092
2.3.1.42	glyceronephosphate O-acyltransferase	0.906836526	-0.039507386	4.101049891	-0.009633481	0.992313713	0.999677092
1.18.1.2	ferredoxin/flavodoxin---NADP+ reductase	421.7852528	-0.147805039	0.401589457	-0.368050099	0.712835878	0.999677092
1.8.4.12	peptide-methionine (R)-S-oxide reductase	160.8333044	0.079621583	0.435035839	0.183023043	0.854779938	0.999677092
3.5.2.17	5-hydroxyisourate hydrolase	0.196024097	-0.790638352	4.13072455	-0.191404278	0.848208868	0.999677092
5.1.3.7	UDP-N-acetylglucosamine 4-epimerase	42.11554931	-0.000865191	0.659584187	-0.001311722	0.998953398	0.999677092
3.4.24.28	bacillolysin	0.938853782	-0.330549289	4.082667399	-0.08096405	0.935470542	0.999677092
1.1.2.4	D-lactate dehydrogenase (cytochrome)	4.173147047	0.538468852	1.653967647	0.325561901	0.744755841	0.999677092
2.7.11.11	protein kinase A	4.556984542	-0.438011191	2.816675651	-0.155506436	0.876422081	0.999677092
5.1.3.26	GlcNAc-P-P-Und epimerase	55.79927746	-0.27379798	0.689951152	-0.396836761	0.691487841	0.999677092
1.3.1.98	UDP-N-acetylmuramate dehydrogenase	388.1332859	0.175647079	0.254971062	0.688890251	0.490892335	0.999677092
4.1.3.43	4-hydroxy-2-oxovalerate/4-hydroxy-2-oxohexanoate aldolase	0.165800975	-0.568154282	4.133977711	-0.137435255	0.890686766	0.999677092

4.2.1.93	ATP-dependent NAD(P)H-hydrate dehydratase	0.404452483	0.227238214	4.106904164	0.055330781	0.95587494	0.999677092
4.2.3.153	(5-formylfuran-3-yl)methyl phosphate synthase	1.166323075	-0.375542123	4.099298318	-0.091611318	0.927006859	0.999677092
2.7.1.82	ethanolamine kinase	1.883458461	-0.338974278	4.097057294	-0.082736036	0.934061431	0.999677092
2.4.1.5	dextransucrase	0.839572008	-2.872155176	4.101501618	-0.70026918	0.483759215	0.999677092
2.3.1.75	diacylglycerol O-acyltransferase / wax synthase	1.228064538	-0.497384623	4.099653058	-0.121323589	0.903434737	0.999677092
3.2.1.123	endoglycosylceramidase	0.937054039	0.152579252	3.712488992	0.04109891	0.967217043	0.999677092
2.7.1.25	adenylylsulfate kinase	65.7994572	-0.758777829	0.557143528	-1.361907283	0.173227148	0.999677092
2.9.1.-	#N/A	0.512348345	-1.441814019	4.11775024	-0.350146059	0.726229086	0.999677092
4.2.1.28	propanediol dehydratase large subunit	272.216343	-1.320812749	0.925295628	-1.427449465	0.153450382	0.999677092
5.3.3.18	2-(1,2-epoxy-1,2-dihydrophenyl)acetyl-CoA isomerase	12.98823612	0.601930322	0.674634349	0.892231951	0.37226862	0.999677092
2.1.1.213	tRNA (guanine10-N2)-dimethyltransferase	0.249773869	-0.680453166	4.123822835	-0.165005431	0.868939707	0.999677092
1.1.1.178	3-hydroxyacyl-CoA dehydrogenase / 3-hydroxy-2-methylbutyry	0.416289782	-0.751854895	4.112195485	-0.182835397	0.854927174	0.999677092
2.7.1.56	1-phosphofructokinase	313.4501232	-0.618234514	0.381306868	-1.621356878	0.104941123	0.999677092
1.1.1.159	7-alpha-hydroxysteroid dehydrogenase	14.93492507	1.269735214	1.035986816	1.225628738	0.220338418	0.999677092
2.7.1.211	PTS system, sucrose-specific IIA component	94.53090177	0.128187986	0.400113109	0.320379372	0.748680761	0.999677092
2.7.7.33	glucose-1-phosphate cytidylyltransferase	43.70030962	-0.226838428	0.418920363	-0.541483413	0.588174427	0.999677092
2.4.1.41	polypeptide N-acetylgalactosaminyltransferase	3.059020224	-0.292357988	2.967893033	-0.098506915	0.921529781	0.999677092
4.2.1.130	D-lactate dehydratase / protein deglycase	1.123723061	0.500667286	3.241430311	0.154458754	0.877248031	0.999677092
2.8.1.11	adenylyltransferase and sulfurtransferase	38.27308209	-1.157347136	0.667386891	-1.734147241	0.082891961	0.999677092
2.1.1.225	tRNA:m4X modification enzyme	1.426288518	-0.365590639	4.099220384	-0.089185407	0.928934563	0.999677092
3.4.13.22	zinc D-Ala-D-Ala dipeptidase	69.29723343	-0.395743617	0.56058124	-0.705952302	0.4802178	0.999677092
4.4.1.8	#N/A	447.9802593	0.466136753	0.355403945	1.311568877	0.18966564	0.999677092
1.3.1.101	digeranylgeranylglycerophospholipid reductase	2.870100187	0.632201588	3.908480759	0.161751235	0.871501756	0.999677092
3.1.3.82	D-glycero-D-manno-heptose 1,7-bisphosphate phosphatase	27.20891321	-0.354945311	0.546383902	-0.649626224	0.515933689	0.999677092
2.7.1.144	tagatose 6-phosphate kinase	19.2680341	-0.673191247	0.67011645	-1.004588451	0.31509506	0.999677092
1.5.1.24	N5-(carboxyethyl)ornithine synthase	0.365589093	-1.733894548	4.115987246	-0.421258484	0.673566342	0.999677092
2.1.1.289	cobalt-precorrin-7 (C5)-methyltransferase	1.380155497	0.565434163	4.100351275	0.137898957	0.890320275	0.999677092
3.1.1.97	diphthine methyl ester acylhydrolase	0.368040408	-0.743715803	4.114656962	-0.180747948	0.856565421	0.999677092
4.2.1.134	very-long-chain (3R)-3-hydroxyacyl-CoA dehydratase	0.052512875	-0.212523182	4.139416356	-0.05134134	0.959053527	0.999677092
1.10.3.9	photosystem II P680 reaction center D1 protein	0.896335188	-0.021690701	4.076573026	-0.005320818	0.995754622	0.999677092
2.5.1.3	thiamine-phosphate pyrophosphorylase	546.7049117	0.765423942	0.537453217	1.424168502	0.154397701	0.999677092
1.16.1.3	aquacobalamin reductase / NAD(P)H-flavin reductase	1.401765292	-1.311531389	3.964718721	-0.330800614	0.740795097	0.999677092
2.1.1.195	cobalt-precorrin-5B (C1)-methyltransferase	157.4125858	-0.013856496	0.315158137	-0.043966803	0.964930865	0.999677092
5.3.1.9	glucose-6-phosphate isomerase	996.9255714	0.046657283	0.365846717	0.127532326	0.898519089	0.999677092
2.7.2.3	phosphoglycerate kinase	1220.852491	-0.622116984	0.471973379	-1.318118799	0.187463882	0.999677092
5.3.3.2	isopentenyl-diphosphate Delta-isomerase	23.14189555	-1.287641981	0.760228702	-1.693756072	0.090311645	0.999677092
3.4.18.1	cathepsin X	33.57074902	-1.088084895	1.357438979	-0.801571865	0.422800658	0.999677092
2.7.1.150	1-phosphatidylinositol-3-phosphate 5-kinase	0.457193986	0.077749749	4.105677923	0.018937128	0.984891261	0.999677092
3.5.1.5	urease	390.454859	0.048127711	0.649745642	0.074071618	0.940953399	0.999677092
4.2.1.99	aconitate hydratase 2 / 2-methylisocitrate dehydratase	5.473792319	-1.033336726	2.016424299	-0.512459965	0.608329133	0.999677092
2.3.1.241	Kdo2-lipid IVA lauroyltransferase	89.05169472	0.210718137	0.434939497	0.484476895	0.628047458	0.999677092
1.2.1.19	aminobutyraldehyde dehydrogenase	0.098065194	0.42289156	4.142590869	0.102083834	0.918690132	0.999677092
6.3.5.9	cobyrinic acid a,c-diamide synthase	147.2244454	-0.268935886	0.314339714	-0.855558093	0.392242258	0.999677092
1.1.1.169	2-dehydropantoate 2-reductase	287.7816628	0.086653685	0.307444409	0.281851555	0.778057338	0.999677092
3.5.2.2	dihydropyrimidinase	110.1995053	-0.23460543	0.315698552	-0.743131156	0.457402279	0.999677092
1.18.6.1	nitrogenase delta subunit	64.43291247	-0.630440496	0.751525481	-0.838881065	0.401536055	0.999677092
5.4.99.45	tRNA pseudouridine38/39 synthase	0.201524496	-0.634878647	4.129303936	-0.153749556	0.877807209	0.999677092
2.7.7.80	adenylyltransferase and sulfurtransferase	38.27308209	-1.157347136	0.667386891	-1.734147241	0.082891961	0.999677092
5.3.1.25	L-fucose/D-arabinose isomerase	314.2306221	-0.470650303	0.398809217	-1.18013898	0.237944944	0.999677092
3.6.3.14	#N/A	2909.060585	0.030041924	0.276217363	0.108761896	0.913391347	0.999677092
2.1.1.181	23S rRNA (adenine1618-N6)-methyltransferase	5.132909253	-1.686429488	1.283873572	-1.313547942	0.188998377	0.999677092
2.3.1.129	UDP-N-acetylglucosamine acyltransferase	195.8513799	0.043924805	0.314153371	0.139819619	0.888802512	0.999677092
2.5.1.58	protein farnesyltransferase subunit beta	0.766825899	-0.782861853	4.10298632	-0.19080294	0.848679985	0.999677092
4.2.1.104	cyanate lyase	0.603088763	0.495326977	4.11895307	0.120255552	0.904280713	0.999677092
3.5.99.7	1-aminocyclopropane-1-carboxylate deaminase	1.103206331	1.024255548	2.278356685	0.44955891	0.653028522	0.999677092
2.4.1.198	#N/A	0.270591585	-0.356159389	4.123910861	-0.086364473	0.931176688	0.999677092
1.2.1.22	lactaldehyde dehydrogenase / glycolaldehyde dehydrogenase	20.55117114	-0.890801212	0.570535718	-1.561341707	0.118443148	0.999677092
3.6.1.6	ectonucleoside triphosphate diphosphohydrolase 5/6	3.61936385	-0.13040026	2.86007092	-0.045593366	0.963634357	0.999677092
1.1.1.40	malate dehydrogenase (oxaloacetate-decarboxylating)(NADP+)	103.4381533	0.206027323	0.42468016	0.485135268	0.627580396	0.999677092
2.7.8.17	UDP-N-acetylglucosamine-lysosomal-enzyme	2.981701137	-0.516033433	3.700462864	-0.13945105	0.889093734	0.999677092
3.2.1.51	alpha-L-fucosidase	1731.162235	-0.377297873	0.426914442	-0.883778659	0.376815709	0.999677092
5.4.99.9	UDP-galactopyranose mutase	302.3987479	-0.007125301	0.196968167	-0.036174885	0.971142912	0.999677092
4.1.99.17	phosphomethylpyrimidine synthase	285.8239162	0.218584289	0.273680625	0.798683827	0.424473767	0.999677092
5.1.3.9	N-acylglucosamine-6-phosphate 2-epimerase	224.6239802	-0.832994964	0.581650963	-1.432121695	0.152108996	0.999677092
1.3.1.10	enoyl-acyl-carrier protein] reductase I	101.9054622	-0.005148513	0.397622774	-0.012948234	0.989669093	0.999677092
2.7.6.5	GTP pyrophosphokinase	1176.233156	0.177660259	0.139346402	1.274954051	0.202325506	0.999677092
1.2.1.72	D-erythrose 4-phosphate dehydrogenase	1.604490321	-0.468529662	3.137582877	-0.149328219	0.88129465	0.999677092
5.1.3.25	dTDP-L-rhamnose 4-epimerase	7.819350156	-0.343107225	1.077213626	-0.318513633	0.750095355	0.999677092
4.2.1.32	#N/A	57.89837913	-0.487054755	0.675347712	-0.721191094	0.470791951	0.999677092
4.2.1.149	crotonobetainyl-CoA hydratase	2.640428685	-1.343161784	1.847052593	-0.727191954	0.467108375	0.999677092
2.3.1.88	#N/A	6.11169659	-0.062779909	2.451035937	-0.025613622	0.97956552	0.999677092
5.5.1.1	muconate cycloisomerase	0.112351376	0.369713326	4.126459759	0.089595767	0.928608449	0.999677092
3.6.1.55	8-oxo-dGTP diphosphatase	263.2355375	0.067132704	0.289292564	0.232058174	0.816492831	0.999677092
2.7.1.177	L-threonine kinase	0.753503802	-0.12008867	4.101559248	-0.029278785	0.976642247	0.999677092
5.4.2.9	phosphoenolpyruvate phosphomutase	19.55533939	0.494745432	0.867836787	0.570090412	0.568616378	0.999677092
6.3.1.5	NAD+ synthase	24.1008445	0.566993658	0.584881443	0.969416392	0.332337477	0.999677092
1.18.1.-	#N/A	0.842428939	-0.637023638	4.115761795	-0.154776605	0.876997435	0.999677092
4.2.1.113	O-succinylbenzoate synthase	6.192673817	-3.06588813	1.621140978	-1.891191557	0.058598778	0.999677092
2.3.1.-	#N/A	294.6393979	0.257016719	0.324645597	0.791683983	0.428544964	0.999677092
1.16.3.2	ferritin heavy chain	754.4409733	-0.083835455	0.503821831	-0.166399013	0.867842951	0.999677092
1.11.1.21	catalase-peroxidase	7.96020901	-3.452410981	2.147068913	-1.607964682	0.107842914	0.999677092
5.1.3.-	#N/A	101.2987499	-1.486099793	0.886689134	-1.676009928	0.093736248	0.999677092
1.2.7.5	aldehyde:ferredoxin oxidoreductase	32.16256784	0.611119388	0.637730549	0.958272092	0.337925573	0.999677092
4.2.1.46	dTDP-glucose 4,6-dehydratase	412.7164931	0.250700103	0.222995122	1.1242403	0.260911097	0.999677092
3.5.5.1	nitrilase	6.75173712	0.263151452	1.25341127	0.20994821	0.833708095	0.999677092
1.14.14.9	4-hydroxyphenylacetate 3-monooxygenase	2.25640993	0.512866634	2.399034972	0.213780391	0.830718317	0.999677092
6.3.2.35	D-alanine---D-serine ligase	29.18882695	-0.202141196	0.587199659	-0.344246106	0.730661211	0.999677092

2.7.7.1	nicotinamide-nucleotide adenylyltransferase	3.591754005	-1.384768447	1.261215518	-1.097963375	0.272220483	0.999677092
2.3.1.157	bifunctional UDP-N-acetylglucosamine pyrophosphorylase / Glu	425.7922078	0.65177983	0.461251547	1.413068063	0.157635713	0.999677092
3.1.3.6	2',3'-cyclic-nucleotide 2'-phosphodiesterase / 3'-nucleotidase	285.4621956	-0.168462923	0.44495182	-0.378609358	0.704977972	0.999677092
3.5.2.5	allantoinase	7.295496758	0.353265254	0.93057807	0.379619148	0.704228145	0.999677092
4.3.1.29	D-glucosaminate-6-phosphate ammonia-lyase	0.040990391	-0.099691597	4.140771598	-0.024075609	0.980792299	0.999677092
3.1.3.27	phosphatidylglycerophosphatase GEP4	37.35501302	-0.273046683	0.879956148	-0.310295784	0.756336037	0.999677092
5.4.2.11	2,3-bisphosphoglycerate-dependent phosphoglycerate mutase	320.0771963	-0.371896039	0.536979391	-0.692570414	0.488579183	0.999677092
2.7.4.6	nucleoside-diphosphate kinase	77.16799679	-0.719634508	0.418139658	-1.721038637	0.085243813	0.999677092
3.1.26.3	#N/A	233.5547131	-0.21145347	0.324234619	-0.652161916	0.514296725	0.999677092
2.1.1.132	precorrin-6Y C5,15-methyltransferase (decarboxylating)	165.8060595	0.027503207	0.244332755	0.112564553	0.910375789	0.999677092
2.7.7.27	glucose-1-phosphate adenylyltransferase	2589.01273	-0.201701303	0.488224771	-0.413132055	0.679509865	0.999677092
2.4.1.267	alpha-1,3-glucosyltransferase	0.129855667	0.286509305	4.124679205	0.069462203	0.944621717	0.999677092
1.12.1.3	NADP-reducing hydrogenase subunit HndB	567.3491264	-0.755365865	0.516147825	-1.463468076	0.143339343	0.999677092
1.11.1.6	#N/A	161.5587878	0.19531974	0.554708844	0.35211218	0.724754134	0.999677092
3.6.5.5	dynamin GTPase	7.357062005	-0.340875016	2.000428948	-0.170400961	0.864694816	0.999677092
2.7.2.8	acetylglutamate kinase	350.0145196	0.057227539	0.396234751	0.144428369	0.885162216	0.999677092
5.4.99.2	methylmalonyl-CoA mutase	436.038468	-0.096316252	0.307863722	-0.312853529	0.754391945	0.999677092
1.1.1.284	S-(hydroxymethyl)glutathione dehydrogenase / alcohol dehydro	2.230351518	-2.377861492	4.096433378	-0.580471174	0.56159692	0.999677092
2.7.4.21	inositol-hexakisphosphate 5-kinase	0.249773869	-0.680453166	4.123822835	-0.165005431	0.868939707	0.999677092
4.1.1.82	phosphonopyruvate decarboxylase	15.5295624	0.137616628	0.795985321	0.172888399	0.862739151	0.999677092
6.2.1.20	long-chain-fatty-acid--[acyl-carrier-protein] ligase	6.062451873	-2.017258275	1.542162104	-1.308071487	0.190849064	0.999677092
2.6.1.101	L-glutamine:2-deoxy-scyllo-inosose/3-amino-2,3-dideoxy-scyllo	0.025751485	0.087866701	4.143731151	0.02120473	0.983082341	0.999677092
4.3.1.15	diaminopropionate ammonia-lyase	83.25829541	0.173311501	0.435025864	0.398393557	0.690340106	0.999677092
3.4.13.19	membrane dipeptidase	203.4932454	0.673649184	0.309731364	2.174946623	0.029634117	0.999677092
4.6.1.15	triose/dihydroxyacetone kinase / FAD-AMP lyase (cyclizing)	1.318253244	-1.117790696	2.748521871	-0.406687939	0.6842372	0.999677092
3.1.3.43	pyruvate dehydrogenase phosphatase	1.498669032	-0.279291456	4.099527669	-0.068127716	0.945683968	0.999677092
6.2.1.3	long-chain acyl-CoA synthetase	787.0709517	0.203519503	0.239881215	0.848417845	0.396205308	0.999677092
2.7.1.196	PTS system, cellobiose-specific IIA component	42.12668325	-0.81491506	0.501512593	-1.62491445	0.104180789	0.999677092
1.1.1.38	malate dehydrogenase (oxaloacetate-decarboxylating)	352.0587986	0.305923085	0.26719703	1.14493445	0.252236312	0.999677092
6.3.4.21	nicotinate phosphoribosyltransferase	719.7523161	0.572489106	0.580573672	0.986074866	0.324096376	0.999677092
1.5.98.1	methylenetetrahydromethanopterin dehydrogenase	7.559254633	2.049749795	1.878638644	1.091082525	0.275236571	0.999677092
4.2.1.17	enoyl-CoA hydratase	991.304438	-0.23344202	0.597484301	-0.390708207	0.696012932	0.999677092
4.4.1.21	S-ribosylhomocysteine lyase	157.4335133	0.075074359	0.35884076	0.209213577	0.834281514	0.999677092
3.6.1.62	mRNA-decapping enzyme subunit 2	0.250485555	0.965168427	4.12830692	0.233792798	0.815145841	0.999677092
2.1.1.72	site-specific DNA-methyltransferase (adenine-specific)	1002.074774	0.121967109	0.26126173	0.466838785	0.640615224	0.999677092
5.4.1.4	D-galactarolactone isomerase	0.052512875	-0.212523182	4.139416356	-0.05134134	0.959053527	0.999677092
3.1.13.-	#N/A	15.34080406	-1.096300846	1.708542681	-0.641658449	0.521094974	0.999677092
5.3.1.3	L-fucose/D-arabinose isomerase	314.2306221	-0.470650303	0.398809217	-1.18013898	0.237944944	0.999677092
4.2.1.11	enolase	1347.16273	-0.588198321	0.424369815	-1.38605127	0.165731249	0.999677092
5.1.3.13	dTDP-4-dehydrorhamnose 3,5-epimerase	198.2590378	0.102243989	0.302824745	0.337634194	0.735638871	0.999677092
2.4.2.7	adenine phosphoribosyltransferase	220.4972688	0.011955078	0.342564898	0.034898723	0.972160499	0.999677092
3.4.11.5	proline iminopeptidase	13.29992319	-0.514268941	0.72968458	-0.704782526	0.480945585	0.999677092
1.1.1.23	histidinol dehydrogenase	231.2619341	-0.073396976	0.159878842	-0.459078732	0.646177629	0.999677092
1.5.1.34	dihydropteridine reductase	16.32083196	-0.56508636	1.030126804	-0.548560001	0.583307442	0.999677092
1.8.4.11	peptide-methionine (S)-S-oxide reductase	173.9237147	0.158967531	0.411896389	0.385940578	0.699540677	0.999677092
3.4.24.56	insulysin	0.63958207	-0.775537911	4.105137471	-0.188918865	0.850156408	0.999677092
1.1.99.3	#N/A	2.344093746	0.529876881	2.073919066	0.255495448	0.798340459	0.999677092
5.3.2.5	2,3-diketo-5-methylthiopentyl-1-phosphate enolase	7.481498469	-1.726702542	1.058191441	-1.631748732	0.102732429	0.999677092
6.3.4.11	biotin---protein ligase	1.456275519	-0.290724248	4.097533312	-0.07095104	0.943436722	0.999677092
3.2.1.196	glycogen debranching enzyme	169.2043627	-0.497362491	0.463506267	-1.073043726	0.283251498	0.999677092
3.1.21.5	type III restriction enzyme	190.996469	0.001328214	0.253212877	0.005245442	0.995814762	0.999677092
3.11.1.2	phosphonoacetate hydrolase	0.557746465	0.956290475	4.101471418	0.233157903	0.815638794	0.999677092
3.5.4.27	methenyltetrahydromethanopterin cyclohydrolase	1.31203418	-0.225347337	4.099092905	-0.054974928	0.956158438	0.999677092
2.4.1.321	cellobionic acid phosphorylase	0.183277685	0.91378405	4.125321317	0.221506152	0.824698347	0.999677092
1.1.1.93	tartrate dehydrogenase	5.410002282	0.263459365	0.972147822	0.271007514	0.786385253	0.999677092
2.7.7.75	molybdopterin adenylyltransferase	1.658651571	1.788316886	2.32543599	0.769024344	0.441878859	0.999677092
2.7.7.87	L-threonylcarbamoyladenylate synthase	305.4179164	-0.107579713	0.154951887	-0.694278173	0.487507777	0.999677092
3.6.3.21	#N/A	551.7754522	0.650465456	0.333223535	1.952039359	0.050933532	0.999677092
2.7.12.1	#N/A	7.925832161	-0.162324418	4.09386524	-0.03965065	0.968371646	0.999677092
6.1.1.19	arginyl-tRNA synthetase	514.4061134	-0.419396402	0.275022336	-1.524953965	0.12727058	0.999677092
1.3.1.34	2,4-dienoyl-CoA reductase (NADPH2)	4.426268906	-0.33775973	1.235023327	-0.273484494	0.78448081	0.999677092
1.3.1.54	precorrin-6A/cobalt-precorrin-6A reductase	80.75062147	0.078017744	0.606384038	0.128660617	0.897626199	0.999677092
1.2.4.2	2-oxoglutarate dehydrogenase E1 component	25.41993019	-0.112901387	0.770721367	-0.146487942	0.883536209	0.999677092
2.7.1.194	PTS system, ascorbate-specific IIA component	22.57368151	-0.952371563	0.798375006	-1.192887498	0.232913436	0.999677092
3.2.1.183	UDP-N-acetylglucosamine 2-epimerase (hydrolysing)	12.14589756	2.415672196	1.078250061	2.240363607	0.025067327	0.999677092
2.6.1.82	putrescine aminotransferase	53.88562689	-0.230864424	0.559389038	-0.412708167	0.679820441	0.999677092
1.11.1.10	non-heme chloroperoxidase	2.224202965	-1.750415267	4.077111516	-0.429327297	0.667685054	0.999677092
2.4.2.29	queuine tRNA-ribosyltransferase	301.7581135	-0.098717614	0.20717915	-0.476484308	0.633729384	0.999677092
3.4.21.102	carboxyl-terminal processing protease	540.2492765	-0.023249925	0.212563763	-0.109378591	0.912902214	0.999677092
2.1.1.45	thymidylate synthase	183.6399445	-0.430229406	0.353948285	-1.215514876	0.224169779	0.999677092
2.7.1.6	galactokinase	445.280387	0.152648764	0.199581081	0.764845866	0.444363332	0.999677092
2.6.1.66	valine--pyruvate aminotransferase	70.05961202	-0.289853179	0.426312526	-0.679907724	0.496562891	0.999677092
2.7.1.16	L-ribulokinase	41.05216264	-0.332439339	0.407198391	-0.816406317	0.414267785	0.999677092
6.3.2.30	cyanophycin synthetase	0.53521663	-0.174159011	4.111715597	-0.042356775	0.966214286	0.999677092
3.2.1.11	dextranase	27.01727601	-0.55841772	0.851869306	-0.655520413	0.512132748	0.999677092
1.8.1.8	thioredoxin 2	156.4033575	-0.18217197	0.322114866	-0.565549711	0.571699922	0.999677092
3.1.4.-	#N/A	196.6449542	0.180704629	0.277184289	0.651929553	0.514446619	0.999677092
2.3.1.46	homoserine O-acetyltransferase/O-succinyltransferase	218.3870639	-0.255955273	0.321213452	-0.796838585	0.425544783	0.999677092
6.2.1.7	solute carrier family 27 (fatty acid transporter), member 5	0.48997058	2.512508529	4.092617946	0.613912308	0.539273276	0.999677092
2.4.1.280	N,N'-diacetylchitobiose phosphorylase	5.176582286	-2.208490707	1.481273671	-1.490940365	0.135977152	0.999677092
3.4.21.96	lactocepin	235.7046641	0.2218764	0.736787901	0.301140124	0.763307646	0.999677092
4.1.1.-	#N/A	0.314148663	-3.926413577	4.143731151	-0.947555098	0.343355994	0.999677092
4.1.1.49	phosphoenolpyruvate carboxykinase (ATP)	1207.42963	-0.351090017	0.457934747	-0.766681323	0.443271011	0.999677092
2.3.1.193	tRNA(Met) cytidine acetyltransferase	0.767393725	-1.561512235	4.105194149	-0.380374759	0.703667249	0.999677092
1.1.1.77	lactaldehyde reductase	361.3221681	0.07699203	0.272855201	0.282171752	0.777811817	0.999677092
1.6.1.1	NAD(P) transhydrogenase	5.221596558	-1.249683212	2.059458211	-0.606801928	0.543982369	0.999677092
1.1.1.372	L-glyceraldehyde reductase	4.412671685	-0.816911324	4.093409534	-0.199567455	0.841818883	0.999677092

1.5.1.38	FMN reductase	13.56279913	-0.420136157	1.090469412	-0.385280093	0.700029909	0.999677092
1.1.1.137	ribulose-5-phosphate 2-dehydrogenase	24.26923239	-0.770375088	0.612823406	-1.25709149	0.208720508	0.999677092
2.1.1.34	tRNA (guanosine-2'-O-)-methyltransferase	7.652998207	0.701726342	1.421739189	0.493568967	0.621610622	0.999677092
3.2.2.26	futalosine hydrolase	1.673267527	-1.080919236	3.697229498	-0.292359248	0.770011963	0.999677092
3.4.23.49	omptin	0.074204671	0.448523946	4.143731151	0.108241565	0.913804074	0.999677092
2.7.11.23	CTD kinase subunit alpha	1.84467463	-0.42731935	4.096109922	-0.104323213	0.916912857	0.999677092
6.1.1.4	leucyl-tRNA synthetase	687.997849	0.182413234	0.18644169	0.97839294	0.327880019	0.999677092
4.1.2.21	2-dehydro-3-deoxyphosphogalactonate aldolase	0.046509078	0.087866701	4.143731151	0.02120473	0.983082341	0.999677092
3.1.1.53	sialate O-acetylesterase	282.7241622	0.126914389	0.449271649	0.282489201	0.777568426	0.999677092
2.6.1.77	taurine-pyruvate aminotransferase	15.705862	1.597023511	0.789562202	2.022669661	0.043107217	0.999677092
2.3.1.189	mycothiol synthase	0.446767538	2.253331964	4.096363474	0.550081061	0.582263776	0.999677092
2.1.1.217	tRNA (adenine22-N1)-methyltransferase	250.9195669	-0.018539967	0.455095305	-0.040738648	0.967504251	0.999677092
2.1.1.242	16S rRNA (guanine1516-N2)-methyltransferase	12.35996513	-0.103923698	0.771353517	-0.134729013	0.892826134	0.999677092
3.5.4.3	guanine deaminase	138.5600482	-0.261553498	0.493005677	-0.53052837	0.595745644	0.999677092
4.1.3.1	isocitrate lyase	1.190703912	-2.309580436	3.563732825	-0.648079009	0.51693385	0.999677092
2.7.1.-	#N/A	1909.117588	-0.258329832	0.504302065	-0.512252179	0.608474529	0.999677092
2.2.1.10	fructose-bisphosphate aldolase / 2-amino-3,7-dideoxy-D-threo-	2.56819499	-1.215286796	2.014315011	-0.603325095	0.546292442	0.999677092
1.1.1.100	3-oxoacyl-[acyl-carrier protein] reductase	830.0443141	-0.498521258	0.335897449	-1.484147201	0.137769873	0.999677092
1.17.99.6	epoxyqueuosine reductase	17.31413246	-0.383676624	0.842871444	-0.45520183	0.648964044	0.999677092
2.4.99.14	3-deoxy-D-manno-octulosonic-acid transferase	104.6062867	0.109755847	0.47516922	0.230982653	0.817328279	0.999677092
4.2.1.24	porphobilinogen synthase	56.76727574	-0.126610765	0.525728639	-0.240829118	0.809687561	0.999677092
2.4.1.260	alpha-1,6-mannosyltransferase	1.51946093	-0.692371469	4.097576455	-0.16897097	0.865819475	0.999677092
6.3.5.4	asparagine synthase (glutamine-hydrolysing)	377.2433377	-0.036193174	0.224643609	-0.16111375	0.872003811	0.999677092
6.1.1.22	asparaginyl-tRNA synthetase	569.499554	0.010121244	0.349974615	0.028919936	0.976928445	0.999677092
2.7.1.33	type I pantothenate kinase	366.0985092	0.002360139	0.3882712	0.006078585	0.995150021	0.999677092
6.5.1.1	bifunctional non-homologous end joining protein LigD	24.55321329	-0.052333981	0.704108022	-0.074326636	0.940750483	0.999677092
2.4.1.10	levansucrase	0.401328364	-1.842590513	4.114505788	-0.447827906	0.654277402	0.999677092
3.2.2.8	ribosylpyrimidine nucleosidase	7.613796596	-0.802075455	1.029767203	-0.778890076	0.436044471	0.999677092
4.2.1.35	3-isopropylmalate/(R)-2-methylmalate dehydratase large subur	590.1564115	-0.25963912	0.156032278	-1.664009031	0.096110625	0.999677092
1.1.1.343	6-phosphogluconate dehydrogenase	386.0194263	-0.440004684	0.652013012	-0.674840343	0.499777206	0.999677092
2.7.1.59	N-acetylglucosamine kinase	0.905041437	1.959284112	3.646962364	0.537237272	0.591103729	0.999677092
2.5.1.114	tRNA wybutosine-synthesizing protein 2	0.249773869	-0.680453166	4.123822835	-0.165005431	0.868939707	0.999677092
2.4.1.336	1,2-diacylglycerol 3-beta-glucosyltransferase	0.52830923	-0.020254175	4.107017095	-0.004931602	0.996065167	0.999677092
3.4.21.83	oligopeptidase B	1.520068509	-2.659099387	2.021981467	-1.315095826	0.188477699	0.999677092
2.7.3.-	#N/A	31.26877507	-0.828646967	0.678682889	-1.220963398	0.222099878	0.999677092
3.5.1.49	formamidase	0.917025487	-1.878057345	2.809153108	-0.668549301	0.503783023	0.999677092
2.7.7.53	sulfate adenylyltransferase (ADP) / ATP adenylyltransferase	1.402946654	1.262228885	2.861168113	0.441158588	0.659098191	0.999677092
1.2.1.76	malonyl-CoA/succinyl-CoA reductase (NADPH)	28.49464127	-0.459589999	0.666352109	-0.689710429	0.490376306	0.999677092
4.3.2.3	ureidoglycolate lyase	78.66999186	-1.85461627	1.943325834	-0.954351678	0.339905658	0.999677092
3.1.1.5	lysophospholipase	20.15479229	-0.920638732	0.635860183	-1.447863471	0.147655237	0.999677092
2.4.1.132	alpha-1,3/alpha-1,6-mannosyltransferase	1.814404281	-0.192372527	4.09772309	-0.0469462	0.962556107	0.999677092
1.1.1.304	meso-butanediol dehydrogenase / (S,S)-butanediol dehydroger	2.544822965	-3.712303495	2.122457999	-1.749058637	0.080280884	0.999677092
5.3.1.12	glucuronate isomerase	529.9970512	0.155685745	0.465375079	0.334538208	0.737973464	0.999677092
2.1.1.61	tRNA 5-methylaminomethyl-2-thiouridine biosynthesis bifuncti	3.684858882	1.409211111	1.563945677	0.901061419	0.367555664	0.999677092
3.6.1.63	alpha-D-ribose 1-methylphosphonate 5-triphosphate diphosph	2.990499697	-0.561399929	2.145916544	-0.261613123	0.793619725	0.999677092
3.4.11.26	intermediate cleaving peptidase 55	0.312227018	-3.793965404	4.143731151	-0.915591593	0.359881143	0.999677092
2.7.11.2	pyruvate dehydrogenase kinase 2/3/4	3.406061021	-0.216418104	3.544303493	-0.061060827	0.951310766	0.999677092
3.1.11.-	#N/A	0.311872348	1.792554763	4.094527096	0.437792869	0.661536441	0.999677092
2.1.1.249	dimethylamine---corrinoid protein Co-methyltransferase	1.264396432	-0.270596492	3.032532832	-0.089231183	0.928898184	0.999677092
2.3.-.-	#N/A	2.137499889	-1.07675645	2.460751329	-0.437572231	0.661696405	0.999677092
2.5.1.18	glutathione S-transferase	5.747224439	-2.296541632	1.837909525	-1.249540089	0.2114676	0.999677092
1.3.1.22	steroid 5-alpha-reductase	119.5639343	-1.577052867	0.878538704	-1.795086385	0.072639938	0.999677092
3.1.2.-	#N/A	298.9955864	-0.66304896	0.299633427	-2.212867126	0.026906808	0.999677092
4.2.1.8	mannonate dehydratase	319.1697185	0.107677039	0.20409811	0.527574896	0.597794421	0.999677092
4.1.1.94	ethylmalonyl-CoA/methylmalonyl-CoA decarboxylase	2.58784862	-0.107461367	3.145836808	-0.034159867	0.972749669	0.999677092
2.4.1.141	beta-1,4-N-acetylglucosaminyltransferase	0.388858125	-0.45946073	4.113706345	-0.111690211	0.911069041	0.999677092
3.4.11.2	aminopeptidase N	47.09178138	-0.357154909	0.539790332	-0.661654882	0.508192426	0.999677092
3.5.3.26	(S)-ureidoglycine aminohydrolase	8.571204104	0.594366509	0.805502015	0.73788333	0.46058535	0.999677092
4.2.2.6	oligogalacturonide lyase	18.28317034	-0.377625312	0.663732652	-0.568941894	0.569395574	0.999677092
2.7.1.162	N-acetylhexosamine 1-kinase	7.442655289	-0.020785694	1.753725141	-0.01185231	0.990543446	0.999677092
1.1.1.3	homoserine dehydrogenase	435.1502177	-0.356692721	0.215136677	-1.657981919	0.097321116	0.999677092
1.13.11.-	#N/A	0.969176871	-1.120442793	4.097892946	-0.273419244	0.784530961	0.999677092
3.6.3.1	#N/A	7.365742814	0.37058264	2.040876524	0.181580138	0.855912238	0.999677092
2.6.1.-	#N/A	379.879431	-0.412917612	0.301813593	-1.368121324	0.171274103	0.999677092
3.2.1.141	maltooligosyltrehalose trehalohydrolase	0.465749231	-0.107242216	4.110690004	-0.026088617	0.979186656	0.999677092
1.2.7.8	indolepyruvate ferredoxin oxidoreductase, alpha subunit	571.351912	0.626091117	0.31428811	1.992092917	0.046360864	0.999677092
5.3.3.14	3-hydroxyacyl-[acyl-carrier protein] dehydratase / trans-2-dece	2.895859573	-0.962409278	1.816191616	-0.529905143	0.5961777	0.999677092
3.1.3.70	mannosyl-3-phosphoglycerate phosphatase	0.122002457	-0.436351705	4.140415365	-0.105388389	0.916067629	0.999677092
2.7.4.8	guanylate kinase	418.9878264	-0.137700847	0.310940238	-0.442853097	0.657871995	0.999677092
3.5.1.108	UDP-3-O-[3-hydroxymyristoyl] N-acetylglucosamine deacetylas	116.3137628	-0.111779163	0.293415548	-0.380958556	0.703234002	0.999677092
1.97.1.9	Tat-targeted selenate reductase subunit YnfE	165.715164	0.154442342	0.34734016	0.444642918	0.656577827	0.999677092
2.7.1.202	PTS system, fructose-specific IIA component	408.9532809	-0.676418302	0.37743171	-1.792160766	0.073107217	0.999677092
2.7.7.7	#N/A	4748.086639	0.177252264	0.21810605	0.812688434	0.416396714	0.999677092
2.3.1.101	formylmethanofuran--tetrahydromethanopterin N-formyltrans	2.080730589	-0.400595487	4.096374836	-0.097792683	0.922096918	0.999677092
2.5.1.16	spermidine synthase	74.31269831	-0.096916974	0.448729602	-0.21598079	0.829002722	0.999677092
3.6.3.31	polyamine-transporting ATPase	242.7358801	-0.00047708	0.454316826	-0.001050103	0.999162139	0.999677092
6.3.4.9	biotin---protein ligase	1.456275519	-0.290724248	4.097533312	-0.07095104	0.943436722	0.999677092
1.1.5.2	quinoprotein glucose dehydrogenase	1.838329898	-0.194587732	4.100440479	-0.047455324	0.962150336	0.999677092
2.5.1.76	cysteate synthase	1.65237058	0.556419726	2.758116492	0.201739023	0.840120754	0.999677092
3.2.1.78	mannan endo-1,4-beta-mannosidase	244.4033901	0.42793673	0.537664581	0.795917651	0.426079901	0.999677092
3.8.1.2	2-haloacid dehalogenase	90.88721424	0.057892195	0.368517226	0.157094948	0.875170019	0.999677092
2.1.1.186	23S rRNA (cytidine2498-2'-O)-methyltransferase	1.410587932	-0.569606657	2.275552163	-0.250315799	0.80234314	0.999677092
3.6.3.33	vitamin B12 transport system ATP-binding protein	0.081334971	-0.253576038	4.142921482	-0.061207059	0.951194308	0.999677092
1.1.1.140	sorbitol-6-phosphate 2-dehydrogenase	9.218496711	-0.242677777	1.072764956	-0.226217099	0.821032572	0.999677092
3.4.21.19	glutamyl endopeptidase	47.63602797	0.940818401	0.558309956	1.685118437	0.091965723	0.999677092
5.4.99.17	squalene-hopene/tetraprenyl-beta-curcumene cyclase	7.194674308	0.00789086	1.41156842	0.005590137	0.99553974	0.999677092
5.3.3.1	3beta-hydroxy-Delta5-steroid dehydrogenase / steroid Delta-is	0.017504292	0.120319428	4.141980666	0.029048766	0.976825697	0.999677092

2.8.1.8	lipoyl synthase	74.59543311	-0.366458133	0.496581975	-0.737961005	0.460538146	0.999677092
2.7.1.29	triose/dihydroxyacetone kinase / FAD-AMP lyase (cyclizing)	1.318253244	-1.117790696	2.748521871	-0.406687939	0.6842372	0.999677092
3.1.2.23	4-hydroxybenzoyl-CoA thioesterase	0.325679685	0.51370685	4.111010962	0.124958764	0.900556196	0.999677092
3.2.1.180	unsaturated chondroitin disaccharide hydrolase	100.6903551	-0.609741046	0.500393763	-1.218522475	0.223025489	0.999677092
3.4.21.66	thermitase	0.051502971	-0.135983389	4.141439557	-0.032834812	0.973806317	0.999677092
2.3.1.180	3-oxoacyl-[acyl-carrier-protein] synthase III	380.2113221	-0.131845862	0.27251446	-0.483812351	0.628519049	0.999677092
6.3.5.10	adenosylcobyric acid synthase	172.5070828	-0.027661186	0.30683672	-0.090149531	0.928168389	0.999677092
1.3.1.76	uroporphyrin-III C-methyltransferase / precorrin-2 dehydrogena	30.31722373	0.024199596	0.835822525	0.028953032	0.97690205	0.999677092
3.1.3.46	6-phosphofructo-2-kinase / fructose-2,6-biphosphatase 3	3.811408119	-0.341104232	2.829932469	-0.120534407	0.904059825	0.999677092
4.1.1.8	oxalyl-CoA decarboxylase	0.835501983	-2.274222963	3.807248931	-0.597340233	0.550280248	0.999677092
2.1.1.198	16S rRNA (cytidine1402-2'-O)-methyltransferase	604.2734402	-0.314772521	0.396270766	-0.794336974	0.426999285	0.999677092
3.2.1.17	lysozyme	68.4832737	-0.544062094	0.94198955	-0.577567017	0.563556481	0.999677092
3.5.3.3	creatinase	0.347232792	0.13150169	4.109143287	0.032002216	0.974470284	0.999677092
2.8.3.12	#N/A	134.3823044	0.255781025	0.505430816	0.506065355	0.61281077	0.999677092
4.2.1.75	uroporphyrinogen-III synthase	157.1676558	-0.14399745	0.254394187	-0.566040647	0.571366149	0.999677092
2.4.2.43	4-amino-4-deoxy-L-arabinose transferase	0.111464417	0.448520484	4.143731151	0.10824073	0.913804737	0.999677092
3.4.24.70	oligopeptidase A	23.81967512	-0.376148759	0.945301158	-0.397914205	0.690693429	0.999677092
3.6.4.-	#N/A	1316.288446	0.080571884	0.196489867	0.410056176	0.681764739	0.999677092
1.11.1.1	NADH peroxidase	1.37195059	-3.458754652	3.384047206	-1.02207636	0.306744761	0.999677092
1.3.1.38	mitochondrial trans-2-enoyl-CoA reductase	12.07210064	0.373885845	1.988249386	0.188047761	0.850839211	0.999677092
4.2.1.151	chorismate dehydratase	8.95134946	0.479468632	0.874794829	0.548092668	0.583628275	0.999677092
2.4.1.259	alpha-1,2-mannosyltransferase	1.191163093	0.076473384	4.098417029	0.018659249	0.985112937	0.999677092
2.5.1.78	6,7-dimethyl-8-ribityllumazine synthase	125.3847375	-0.317471089	0.388423245	-0.817332879	0.413738222	0.999677092
2.7.8.12	CDP-glycerol glycerophosphotransferase	32.67163653	-0.410273427	0.705324284	-0.581680563	0.560781866	0.999677092
1.1.1.94	glycerol-3-phosphate dehydrogenase (NAD(P)+)	453.6570729	0.364553015	0.251756631	1.448037388	0.147606595	0.999677092
3.5.3.8	formiminoglutamase	2.637396381	-1.24108403	2.020475132	-0.614253554	0.53904779	0.999677092
3.5.99.10	2-iminobutanoate/2-iminopropanoate deaminase	243.9802341	-0.331101229	0.297780387	-1.11189737	0.266182279	0.999677092
1.1.1.27	L-lactate dehydrogenase	346.4267477	-0.601563375	0.513541691	-1.171401243	0.241437533	0.999677092
3.1.21.2	deoxyribonuclease IV	367.0371785	-0.259733474	0.334928776	-0.775488678	0.438050953	0.999677092
2.8.3.5	3-oxoacid CoA-transferase	11.57711064	1.290173699	1.013476995	1.273017251	0.20301192	0.999677092
2.7.8.14	CDP-ribitol ribitolphosphotransferase / teichoic acid ribitol-phos	27.28268986	0.847817088	0.956402132	0.886465075	0.375366966	0.999677092
6.3.1.19	proteasome accessory factor A	1.381132656	-0.204673074	4.034880397	-0.050725933	0.959543912	0.999677092
3.4.17.13	muramoyltetrapeptide carboxypeptidase	75.16794398	-0.99733519	0.860199017	-1.159423772	0.246283491	0.999677092
3.4.22.70	sortase A	1125.394995	0.289756113	0.342297279	0.846504282	0.397271486	0.999677092
3.5.1.105	chitin disaccharide deacetylase	3.814956233	-1.131881204	1.391774509	-0.81326479	0.41606626	0.999677092
4.1.1.85	3-dehydro-L-gulonate-6-phosphate decarboxylase	0.249683108	0.218205905	4.122011293	0.052936756	0.957782299	0.999677092
2.5.1.31	undecaprenyl diphosphate synthase	411.3346321	-0.094691141	0.238638674	-0.396797129	0.691517068	0.999677092
1.5.1.7	saccharopine dehydrogenase (NAD+, L-lysine forming)	197.2445999	-0.05525848	0.317707893	-0.17392857	0.861921599	0.999677092
3.4.19.1	acylaminoacyl-peptidase	1.868496682	1.404857715	1.818017162	0.772741724	0.439675249	0.999677092
3.1.3.73	alpha-ribazole phosphatase	197.9568009	0.113900997	0.254593699	0.44738341	0.654598252	0.999677092
2.1.1.166	23S rRNA (uridine2552-2'-O)-methyltransferase	15.58275964	-0.194515611	1.095970152	-0.177482581	0.859129347	0.999677092
1.6.5.2	NAD(P)H dehydrogenase (quinone)	14.05382573	0.415345622	0.663146526	0.626325564	0.531101422	0.999677092
3.4.21.110	C5a peptidase	1.02995056	1.947948982	4.103418861	0.474713659	0.634991081	0.999677092
4.7.1.1	alpha-D-ribose 1-methylphosphonate 5-phosphate C-P lyase	3.093319832	-0.324694036	2.011399354	-0.161426937	0.871757153	0.999677092
5.1.1.3	glutamate racemase	294.3667194	-0.022015977	0.217635144	-0.101160028	0.919423427	0.999677092
1.8.1.2	#N/A	13.3789185	-0.427232724	1.142140582	-0.374063167	0.708357321	0.999677092
4.1.99.12	3,4-dihydroxy 2-butanone 4-phosphate synthase	303.3060112	-0.106305457	0.30013926	-0.354187111	0.723198662	0.999677092
3.1.3.96	pseudouridine 5'-phosphatase	0.416289782	-0.751854895	4.112195485	-0.182835397	0.854927174	0.999677092
4.1.2.14	2-dehydro-3-deoxyphosphogluconate aldolase / (4S)-4-hydroxy	296.8325156	0.102901204	0.293069656	0.351115177	0.725501942	0.999677092
5.4.99.27	tRNA pseudouridine13 synthase	2.123783118	-0.676440895	4.095583938	-0.16516348	0.868815309	0.999677092
1.12.99.6	#N/A	96.42638783	0.511138363	0.564046874	0.906198379	0.364830861	0.999677092
3.6.1.43	dolichyldiphosphatase	0.416289782	-0.751854895	4.112195485	-0.182835397	0.854927174	0.999677092
2.7.1.192	PTS system, N-acetylmuramic acid-specific IIB component	0.136601963	-0.494769398	4.139022345	-0.119537745	0.904849338	0.999677092
1.3.99.33	urocanate reductase	24.00152255	0.906365305	0.647425554	1.399952937	0.161527412	0.999677092
3.6.1.41	bis(5'-nucleosyl)-tetraphosphatase (symmetrical)	3.187241846	0.603937224	1.20815457	0.499884071	0.617156709	0.999677092
6.3.5.2	GMP synthase (glutamine-hydrolysing)	746.5195763	0.031007294	0.247182648	0.125442842	0.900172973	0.999677092
3.6.3.-	#N/A	1695.617713	-0.059583707	0.184859659	-0.322318605	0.74721134	0.999677092
3.6.1.29	bis(5'-adenosyl)-triphosphatase	0.035008583	-0.076612487	4.14032189	-0.018503993	0.985236792	0.999677092
2.3.1.76	diacylglycerol O-acyltransferase 1	1.228064538	-0.497384623	4.099653058	-0.121323589	0.903434737	0.999677092
3.1.6.8	arylsulfatase A	1.683971084	1.383574013	3.135854058	0.441211226	0.659060087	0.999677092
1.14.13.59	lysine N6-hydroxylase	1.497268624	0.071327947	4.099205417	0.017400432	0.986117164	0.999677092
2.7.1.170	anhydro-N-acetylmuramic acid kinase	25.33708748	-0.03421575	0.640147724	-0.053449773	0.957373549	0.999677092
2.6.1.57	aromatic-amino-acid transaminase	22.44406187	0.749749083	0.694684078	1.079266255	0.280469052	0.999677092
1.3.99.-	#N/A	44.4194708	-0.867153471	0.560322199	-1.547597921	0.121719132	0.999677092
2.7.8.36	undecaprenyl phosphate N,N'-diacetylbacillosamine 1-phospha	2.256262368	-1.737986989	2.453498717	-0.708370857	0.478714985	0.999677092
5.3.1.16	phosphoribosylformimino-5-aminoimidazole carboxamide ribot	198.1663491	0.006838067	0.284161026	0.024064055	0.980801515	0.999677092
3.1.2.6	hydroxyacylglutathione hydrolase	0.647579986	-2.398538401	4.111242941	-0.583409552	0.559617629	0.999677092
1.15.1.1	nickel superoxide dismutase	1042.834107	-0.240092281	0.633024026	-0.379278307	0.704481207	0.999677092
2.4.1.101	alpha-1,3-mannosyl-glycoprotein beta-1,2-N-acetylglucosaminy	4.740996338	-0.275892122	3.292106536	-0.083804129	0.933212165	0.999677092
3.5.4.43	hydroxydechloroatrazine ethylaminohydrolase	1.738495381	1.3871512	1.896454929	0.731444327	0.464507798	0.999677092
2.3.2.17	peptidoglycan pentaglycine glycine transferase (the second and	0.581404477	-0.138310665	4.110098579	-0.033651423	0.973155116	0.999677092
2.4.99.17	S-adenosylmethionine:tRNA ribosyltransferase-isomerase	312.4137439	-0.213769441	0.292440487	-0.730984425	0.464788666	0.999677092
4.2.2.3	poly(beta-D-mannuronate) lyase	4.039561784	-1.003301248	2.275209884	-0.440970855	0.659234097	0.999677092
6.3.2.8	UDP-N-acetylmuramate--alanine ligase	411.158067	0.197432404	0.221349663	0.891948066	0.37242077	0.999677092
2.7.1.184	sulfofructose kinase	3.910939087	-0.339174403	1.229772401	-0.275802582	0.782699701	0.999677092
3.5.99.6	#N/A	813.8476878	0.386582251	0.323336946	1.195601848	0.23185197	0.999677092
3.2.1.10	oligo-1,6-glucosidase	174.9034232	-0.858557074	0.47453967	-1.809241943	0.070413424	0.999677092
2.3.1.39	acyl-carrier-protein] S-malonyltransferase	274.8139792	-0.262158732	0.333967416	-0.784982963	0.432463557	0.999677092
2.4.99.12	3-deoxy-D-manno-octulosonic-acid transferase	104.6062867	0.109755847	0.47516922	0.230982653	0.817328279	0.999677092
3.2.1.113	mannosyl-oligosaccharide alpha-1,2-mannosidase	10.79752778	-0.303914549	4.09356808	-0.074241968	0.940817853	0.999677092
3.1.3.36	inositol polyphosphate 5-phosphatase INPP5B/F	8.344447898	0.063842962	4.093733939	0.015595289	0.987557264	0.999677092
1.13.12.-	#N/A	1.914163853	-1.556397039	4.093530259	-0.380208998	0.70379028	0.999677092
2.7.7.47	streptomycin 3''-adenylyltransferase	12.33183838	-0.057558931	1.031477981	-0.055802385	0.955499235	0.999677092
1.14.11.27	F-box and leucine-rich repeat protein 10/11	0.245483854	1.439411087	4.117034092	0.34962331	0.726621415	0.999677092
1.1.3.21	alpha-glycerophosphate oxidase	4.838986957	-2.297355372	2.466157597	-0.931552539	0.35156782	0.999677092
1.3.1.12	prephenate dehydrogenase	153.5917507	-0.052847658	0.289560681	-0.182509786	0.855182675	0.999677092
5.3.99.11	2-keto-myo-inositol isomerase	4.341769719	-0.87781122	1.225365648	-0.716366761	0.473764914	0.999677092

3.2.1.21	beta-glucosidase	2657.934981	-0.263154355	0.577932699	-0.455337369	0.648866546	0.999677092
1.5.5.2	RHH-type transcriptional regulator, proline utilization regulon re	63.59410052	0.110762322	0.481420208	0.230074101	0.818034189	0.999677092
2.1.2.9	methionyl-tRNA formyltransferase	346.3261987	0.148398309	0.149913004	0.98989617	0.322224872	0.999677092
2.4.2.2	pyrimidine-nucleoside phosphorylase	168.5824076	0.360314083	0.335763015	1.073120227	0.283217177	0.999677092
2.7.1.17	xylulokinase	345.6234778	-0.053143752	0.306301035	-0.173501706	0.862257087	0.999677092
2.7.7.13	mannose-1-phosphate guanylyltransferase	358.2910064	0.065854729	0.282143293	0.233408806	0.815443976	0.999677092
4.2.2.8	heparin/heparan-sulfate lyase	26.41907383	-1.559648944	1.089219553	-1.431895838	0.152173634	0.999677092
5.4.3.9	glutamate 2,3-aminomutase	0.081980782	-0.365225737	4.138295101	-0.088255122	0.929673907	0.999677092
1.11.1.15	peroxiredoxin (alkyl hydroperoxide reductase subunit C)	1347.271603	0.172093198	0.576470753	0.298528932	0.765299497	0.999677092
3.5.4.12	dCMP deaminase	245.9939325	0.005876306	0.267977728	0.021928336	0.982505121	0.999677092
3.6.3.40	teichoic acid transport system ATP-binding protein	74.48854514	0.008464616	0.544689656	0.015540254	0.98760117	0.999677092
2.4.1.250	D-inositol-3-phosphate glycosyltransferase	0.122971174	-0.540164935	4.136874465	-0.1305732	0.896112945	0.999677092
2.1.-.-	#N/A	0.255199619	-0.359224048	4.11945823	-0.08720177	0.930511133	0.999677092
3.1.11.5	exodeoxyribonuclease V	537.3560927	0.18069266	0.39185342	0.461123091	0.644710296	0.999677092
1.1.1.336	UDP-N-acetyl-D-mannosaminuronic acid dehydrogenase	25.22143025	-0.584532194	0.817807011	-0.714755665	0.474760035	0.999677092
1.14.13.-	#N/A	2.723150752	-2.941477743	3.084867067	-0.953518475	0.34032744	0.999677092
3.6.1.65	(d)CTP diphosphatase	3.073861829	-2.288834887	2.876324372	-0.795749919	0.426177406	0.999677092
1.2.1.81	sulfoacetaldehyde dehydrogenase	9.761748302	0.716964814	0.748014761	0.958490195	0.337815633	0.999677092
4.1.2.17	L-fuculose-phosphate aldolase	163.6600843	-0.848749352	0.445386756	-1.90564569	0.05669619	0.999677092
2.5.1.41	phosphoglycerol geranylgeranyltransferase	0.624441127	-0.425688192	4.106392131	-0.103664769	0.917435386	0.999677092
5.1.99.3	allantoin racemase	5.521276769	-0.530432164	1.188580496	-0.446273656	0.655399583	0.999677092
1.1.1.205	IMP dehydrogenase	601.6865534	-0.489195833	0.277960955	-1.759944423	0.07841723	0.999677092
2.4.1.325	dTDP-N-acetylfucosamine:lipid II N-acetylfucosaminyltransferas	0.162992848	-0.914659404	4.132822836	-0.2213159	0.824846471	0.999677092
2.1.3.11	N-succinyl-L-ornithine transcarbamylase	88.30516408	0.339199665	0.395335734	0.858004059	0.390890222	0.999677092
3.5.4.9	methylenetetrahydrofolate dehydrogenase (NADP+) / metheny	460.8469926	-0.196276476	0.325606746	-0.602802241	0.546640256	0.999677092
1.1.1.384	dTDP-3,4-didehydro-2,6-dideoxy-alpha-D-glucose 3-reductase	3.094463476	0.588723685	2.296507712	0.256356067	0.797675908	0.999677092
2.1.1.203	tRNA (cytosine34-C5)-methyltransferase	2.749594251	-0.175230033	3.036116865	-0.057715181	0.953975501	0.999677092
2.3.2.10	serine/alanine adding enzyme	5.042106869	-2.348171753	1.891149429	-1.241663782	0.214360638	0.999677092
4.1.2.50	6-pyruvoyltetrahydropterin/6-carboxytetrahydropterin synthas	143.680634	0.828945086	0.403172058	2.056057879	0.039776938	0.999677092
2.7.1.49	hydroxymethylpyrimidine/phosphomethylpyrimidine kinase / t	196.5194831	-0.161383665	0.270883478	-0.59576784	0.551330331	0.999677092
3.6.-.-	#N/A	345.8259585	-0.104707436	0.222621161	-0.470339099	0.638112767	0.999677092
6.3.1.1	aspartate--ammonia ligase	200.3070772	-0.328413628	0.283697091	-1.157620709	0.247018855	0.999677092
3.1.1.89	protein phosphatase methylesterase 1	1.451571512	-0.411374224	4.09821726	-0.100378823	0.920043582	0.999677092
2.1.1.298	ribosomal protein L3 glutamine methyltransferase	2.881866914	0.529205336	1.088782603	0.486052344	0.626930054	0.999677092
1.7.5.1	nitrate reductase / nitrite oxidoreductase, alpha subunit	30.68379277	0.62466292	0.903535837	0.691353784	0.48934324	0.999677092
1.1.1.14	L-iditol 2-dehydrogenase	103.1114767	0.06777142	0.463806219	0.146120119	0.883826564	0.999677092
1.3.1.89	tRNA-dihydrouridine synthase 3	2.528684853	-0.223764952	3.931838729	-0.05691102	0.954616076	0.999677092
1.13.11.2	catechol 2,3-dioxygenase	2.031466026	-0.237481891	3.462569392	-0.068585453	0.945319598	0.999677092
6.4.1.5	geranyl-CoA carboxylase alpha subunit	0.683567942	-0.778224796	4.104346334	-0.189609924	0.84961481	0.999677092
4.3.1.2	methylaspartate ammonia-lyase	56.28752504	1.33448575	0.732256407	1.822429599	0.068389827	0.999677092
3.4.11.7	glutamyl aminopeptidase	3.896020177	-1.309550698	2.360710475	-0.554727364	0.579081157	0.999677092
3.5.1.98	histone deacetylase 1/2	6.724734731	-0.193679613	4.094098592	-0.047307022	0.962268531	0.999677092
5.1.3.6	UDP-glucuronate 4-epimerase	20.60700147	-0.502243995	0.950385863	-0.528463243	0.597177853	0.999677092
3.1.26.12	ribonuclease E	21.05414725	0.550731915	0.891937362	0.61745582	0.536934113	0.999677092
5.4.99.58	methylornithine synthase	1.195008066	0.863710616	2.649222296	0.326024214	0.744406035	0.999677092
2.7.1.26	riboflavin kinase	334.3661825	0.38971942	0.228212465	1.707704352	0.087691214	0.999677092
3.2.1.33	glycogen debranching enzyme	2.01001148	0.936116283	4.015489571	0.233126314	0.815663322	0.999677092
3.2.1.14	chitinase	74.35089469	-0.307769562	0.673901096	-0.456698414	0.647887831	0.999677092
1.1.3.-	#N/A	0.196207128	-0.488169202	4.139032005	-0.117942843	0.906112946	0.999677092
3.1.1.3	triacylglycerol lipase	4.439500809	0.284205839	1.448796808	0.196166803	0.84447961	0.999677092
3.5.1.32	hippurate hydrolase	22.867801	0.293475653	0.669271134	0.438500389	0.661023588	0.999677092
3.5.1.9	arylformamidase	136.0042455	0.257613424	0.828149178	0.31107128	0.755746434	0.999677092
3.1.13.4	poly(A)-specific ribonuclease	2.749647617	-0.299662592	4.095589783	-0.07316714	0.941673114	0.999677092
3.5.2.18	enamidase	3.748924151	1.524714177	1.851364363	0.823562453	0.410188217	0.999677092
2.7.1.55	allose kinase	14.51981234	-0.632656062	0.756869494	-0.83588527	0.40321945	0.999677092
4.1.1.19	arginine decarboxylase	178.3389114	0.099195436	0.466081717	0.21282842	0.831460795	0.999677092
4.1.2.40	tagatose 1,6-diphosphate aldolase	239.5719266	-0.367166532	0.671060298	-0.547143876	0.584279891	0.999677092
2.1.1.64	2-polyprenyl-6-hydroxyphenyl methylase / 3-demethylubiquinc	0.340645926	0.100068475	4.122469961	0.024273912	0.980634122	0.999677092
2.3.1.61	2-oxoglutarate dehydrogenase E2 component (dihydrolipoamid	116.1652786	-0.297751574	0.678270088	-0.438986738	0.660671145	0.999677092
6.1.1.5	isoleucyl-tRNA synthetase	783.2345106	0.185814267	0.232722785	0.798436076	0.424617475	0.999677092
3.1.1.17	gluconolactonase	0.591879764	-1.004930209	4.111422929	-0.244423944	0.806902499	0.999677092
4.1.2.43	3-hexulose-6-phosphate synthase	9.761425783	-1.386749021	0.957589059	-1.448167152	0.147570309	0.999677092
4.2.1.80	2-keto-4-pentenoate hydratase	0.950669968	-1.049147513	4.121630484	-0.254546718	0.799073216	0.999677092
2.1.1.245	acetyl-CoA decarbonylase/synthase complex subunit delta	736.5094664	0.107275839	0.629755655	0.170345178	0.864738683	0.999677092
3.5.3.11	agmatinase	76.90483775	0.05148481	0.371214854	0.138692753	0.889692945	0.999677092
6.6.1.2	cobaltochelatase CobN	121.2009386	0.275676938	0.586981015	0.469652221	0.638603508	0.999677092
2.1.1.183	18S rRNA (adenine1779-N6/adenine1780-N6)-dimethyltransfer	1.791028411	-0.306077304	4.098281819	-0.074684299	0.940465901	0.999677092
3.5.-.-	#N/A	13.91699383	-0.195343773	0.720070692	-0.271284161	0.786172488	0.999677092
3.7.1.20	fumarylpyruvate hydrolase	5.87380492	2.481614223	1.242248615	1.997679203	0.04575145	0.999677092
2.7.8.28	LPPG:FO 2-phospho-L-lactate transferase	0.874214996	-0.504261795	4.102311327	-0.122921386	0.90216935	0.999677092
1.1.1.85	3-isopropylmalate dehydrogenase	228.1220397	-0.144830256	0.236644459	-0.612016257	0.540527002	0.999677092
3.2.1.122	maltose-6'-phosphate glucosidase	26.0661655	-0.428148384	0.653238586	-0.655424209	0.512194669	0.999677092
2.1.2.3	phosphoribosylaminoimidazolecarboxamide formyltransferase	547.7326221	0.042142907	0.1815458	0.232133745	0.816434136	0.999677092
2.7.-.-	#N/A	187.9179305	0.126506498	0.320126224	0.39517693	0.692712318	0.999677092
3.2.1.89	arabinogalactan endo-1,4-beta-galactosidase	105.1190644	0.086713993	0.573164008	0.151290018	0.879746943	0.999677092
2.8.3.8	#N/A	183.3888661	0.042682421	0.528926858	0.080696263	0.935683509	0.999677092
2.1.1.271	precorrin-4/cobalt-precorrin-4 C11-methyltransferase	98.82307915	-0.216409953	0.365395589	-0.592262085	0.553675101	0.999677092
1.8.4.-	#N/A	2.424680651	-1.528738993	2.877926008	-0.531194683	0.595283877	0.999677092
2.4.2.9	uracil phosphoribosyltransferase	517.0070671	-0.02574024	0.296340946	-0.086860222	0.930782618	0.999677092
3.4.-.-	#N/A	1952.852084	-0.214403026	0.294241365	-0.728663781	0.466207353	0.999677092
4.1.1.44	4-carboxymuconolactone decarboxylase	73.14122184	-0.053908934	0.526343758	-0.102421531	0.918422094	0.999677092
3.1.1.73	feruloyl esterase	0.78497298	-2.340581312	4.113013259	-0.569067291	0.569310475	0.999677092
2.1.1.130	#N/A	73.91478762	-0.275373779	0.440914952	-0.624550785	0.532265929	0.999677092
4.1.1.12	aspartate 4-decarboxylase	60.2823711	-0.499418076	0.679737879	-0.734721562	0.462509086	0.999677092
1.7.2.3	trimethylamine-N-oxide reductase (cytochrome c)	15.37738031	0.631577629	0.671873948	0.940023989	0.347205256	0.999677092
2.10.1.1	molybdopterin molybdotransferase	116.2328554	0.396944812	0.467029528	0.84993515	0.395361142	0.999677092
1.4.3.-	#N/A	0.039493742	0.43806451	4.143245313	0.105729803	0.915796734	0.999677092

2.5.1.19	3-phosphoshikimate 1-carboxyvinyltransferase	298.9208236	0.64950858	0.341712334	1.900746667	0.057335203	0.999677092
2.1.3.2	aspartate carbamoyltransferase	349.324848	-0.544417856	0.28431605	-1.91483336	0.055513759	0.999677092
3.6.5.-	#N/A	429.493709	-0.072355812	0.201910764	-0.358355396	0.720077367	0.999677092
3.5.1.78	glutathionylspermidine amidase/synthetase	1.249834218	-1.802092233	3.40217836	-0.529687759	0.596328436	0.999677092
3.6.3.28	#N/A	9.816075023	0.801519238	1.033056935	0.77587131	0.437824974	0.999677092
2.1.3.1	methylmalonyl-CoA carboxyltransferase 5S subunit	0.495699816	1.450831751	4.086950734	0.35499125	0.722596146	0.999677092
2.1.1.214	tRNA (guanine10-N2)-methyltransferase	2.397965246	-0.341805076	4.096779873	-0.083432619	0.933507552	0.999677092
3.5.1.4	amidase	10.62089373	-0.903745722	0.973804961	-0.92805619	0.353378423	0.999677092
6.3.5.1	NAD+ synthase (glutamine-hydrolysing)	5370.660459	-0.827999534	1.17487206	-0.704757192	0.480961353	0.999677092
6.2.1.13	acetate---CoA ligase (ADP-forming) subunit alpha	21.3186108	0.171893418	0.805261452	0.213462867	0.83096595	0.999677092
1.2.1.27	malonate-semialdehyde dehydrogenase (acetylating) / methylr	18.35639239	0.338433957	0.569460393	0.594306401	0.552307198	0.999677092
2.3.3.9	malate synthase	7.740445911	-1.135176582	2.398601188	-0.47326608	0.636023359	0.999677092
1.5.99.15	dihydromethanopterin reductase (acceptor)	1.787205415	-0.13651257	4.097097947	-0.033319333	0.973419937	0.999677092
3.1.3.12	trehalose 6-phosphate phosphatase	40.02501221	-0.389955637	0.598836353	-0.651188985	0.5149245	0.999677092
5.3.1.30	5-deoxy-glucuronate isomerase	31.66558741	-0.372607241	0.4592044	-0.811419143	0.41712501	0.999677092
2.1.1.178	16S rRNA (cytosine1407-C5)-methyltransferase	0.283636172	0.171628884	4.116049642	0.041697477	0.966739866	0.999677092
2.7.2.7	butyrate kinase	160.9491394	0.022250206	0.379111775	0.058690359	0.953198738	0.999677092
3.2.1.133	cyclomaltodextrinase / maltogenic alpha-amylase / neopullular	0.30127684	-1.332711405	4.125972188	-0.323005426	0.746691133	0.999677092
1.5.3.22	coenzyme F420H2 oxidase	4.822589886	1.822853153	2.08790176	0.873055039	0.382633079	0.999677092
1.17.4.2	ribonucleoside-triphosphate reductase (thioredoxin)	1225.795642	0.411641723	0.295172806	1.394578749	0.163142907	0.999677092
2.7.2.11	glutamate 5-kinase	246.2464691	0.047450493	0.191028896	0.24839432	0.803829328	0.999677092
2.7.8.8	CDP-diacylglycerol---serine O-phosphatidyltransferase	124.5052398	-0.091681866	0.499508295	-0.183544231	0.854371017	0.999677092
2.4.1.208	1,2-diacylglycerol-3-alpha-glucose alpha-1,2-glucosyltransferase	10.86455472	-1.012699114	0.6448328	-1.570483254	0.116302727	0.999677092
6.1.1.7	alanyl-tRNA synthetase	1204.488849	0.159178191	0.303269913	0.524873006	0.599671476	0.999677092
2.6.1.52	phosphoserine aminotransferase	469.6711707	-0.470497866	0.389620252	-1.207580623	0.227208616	0.999677092
4.99.1.3	sirohydrochlorin cobaltochelatase	153.816797	-0.181559929	0.355930115	-0.510099937	0.609981449	0.999677092
3.1.1.61	two-component system, chemotaxis family, protein-glutamate	50.27352804	0.04434146	0.643263862	0.068931993	0.945043752	0.999677092
2.5.1.90	octaprenyl-diphosphate synthase	86.87419176	0.837484962	0.467475417	1.791505889	0.07321215	0.999677092
3.5.1.16	acetylornithine deacetylase	73.83330864	-0.013219011	0.416526979	-0.031736266	0.974682373	0.999677092
1.1.1.202	1,3-propanediol dehydrogenase	2.374458127	-1.176115749	4.063152307	-0.289458937	0.772230199	0.999677092
1.20.1.1	phosphonate dehydrogenase	0.332585466	-0.70260957	4.122109387	-0.170449036	0.864657011	0.999677092
2.3.1.234	N6-L-threonylcarbamoyladenine synthase	531.8124809	0.301994598	0.31498981	0.958744023	0.337687715	0.999677092
4.1.99.22	GTP 3',8-cyclase	105.6089693	-0.818347768	0.357090543	-2.291709439	0.021922415	0.999677092
1.4.1.3	glutamate dehydrogenase (NAD(P)+)	18.29244421	0.354118442	0.590230263	0.599966597	0.548528497	0.999677092
2.6.1.97	archaeosine synthase	0.7285168	-0.360909198	4.105096796	-0.087917342	0.929942373	0.999677092
2.1.1.193	16S rRNA (uracil1498-N3)-methyltransferase	289.4001831	0.920514615	0.434504501	2.118538732	0.034129468	0.999677092
3.4.22.1	cathepsin B	63.68857474	-0.703141688	1.95470275	-0.359717961	0.719058059	0.999677092
4.2.1.20	tryptophan synthase	461.3621495	-0.020274165	0.263693988	-0.076885201	0.938714871	0.999677092
1.1.2.3	L-lactate dehydrogenase (cytochrome)	0.529669772	-1.246635973	4.113823531	-0.303035841	0.761862548	0.999677092
3.1.3.66	inositol polyphosphate-4-phosphatase	1.375871973	-0.327829834	4.097424987	-0.080008746	0.9362303	0.999677092
2.7.11.17	calcium/calmodulin-dependent protein kinase kinase 1	11.32903906	-0.295305651	4.093557899	-0.072139117	0.942491197	0.999677092
2.3.1.210	dTDP-4-amino-4,6-dideoxy-D-galactose acyltransferase	0.061162681	-0.245263001	4.141488419	-0.05922098	0.9527761	0.999677092
2.1.1.221	tRNA (guanine9-N1)-methyltransferase	3.682511275	-0.311751616	2.893260442	-0.107750969	0.914193237	0.999677092
2.3.1.202	UDP-4-amino-4,6-dideoxy-N-acetyl-beta-L-altrosamine N-acety	0.206011882	-0.823172586	4.130228034	-0.199304392	0.842024644	0.999677092
3.4.13.-	#N/A	542.4797353	-0.165905445	0.277156013	-0.598599477	0.549440005	0.999677092
3.4.24.64	mitochondrial-processing peptidase subunit alpha	2.288629601	-0.110220532	3.220735657	-0.03422216	0.972699996	0.999677092
2.7.7.73	sulfur carrier protein ThiS adenylyltransferase	11.94926618	0.144561988	1.037642776	0.139317684	0.889199117	0.999677092
2.8.1.1	#N/A	9.932706052	-1.338017836	0.899505102	-1.487504444	0.13688163	0.999677092
3.4.21.107	serine protease Do	631.2321672	-0.020235551	0.386290913	-0.052384228	0.958222541	0.999677092
3.4.17.19	carboxypeptidase Taq	71.80582444	0.451859765	0.372793684	1.212090719	0.225477656	0.999677092
3.1.26.4	ribonuclease HI	1467.073592	-0.235646825	0.642922284	-0.366524587	0.713973669	0.999677092
3.7.1.5	acylpyruvate hydrolase	0.424682647	-0.027816767	4.121950132	-0.006748448	0.994615558	0.999677092
1.8.4.2	protein-disulfide reductase (glutathione)	11.94444886	-0.125166919	4.0935208	-0.030576837	0.975607015	0.999677092
2.1.1.79	cyclopropane-fatty-acyl-phospholipid synthase	18.11968335	-0.135223535	0.601507379	-0.224807774	0.822128818	0.999677092
5.4.99.23	23S rRNA pseudouridine1911/1915/1917 synthase	11838.22793	1.178622916	1.110637564	1.061212905	0.288593156	0.999677092
1.2.7.1	#N/A	4974.621445	0.024810485	0.508300536	0.04881066	0.961070187	0.999677092
2.1.1.248	methylamine---corrinoid protein Co-methyltransferase	0.303919997	-0.489651489	4.133201008	-0.118467863	0.905696958	0.999677092
2.7.13.3	two-component system, sensor histidine kinase PdtaS	2275.191186	-0.269616345	0.274469944	-0.982316465	0.325943966	0.999677092
2.8.3.19	CoA:oxalate CoA-transferase	7.009045476	-0.235921455	0.958120191	-0.246233674	0.80550135	0.999677092
2.7.1.207	PTS system, lactose-specific IIA component	9.523751013	-1.539189333	1.009782078	-1.524278719	0.127439101	0.999677092
2.7.1.2	glucokinase	1290.074803	-0.501291782	0.510677669	-0.981620721	0.326286735	0.999677092
2.7.7.58	#N/A	3.116189468	-2.600899967	2.668789258	-0.974561764	0.329777688	0.999677092
6.3.2.2	glutamate--cysteine ligase	19.66428531	-0.916000083	0.602735618	-1.519737768	0.128576895	0.999677092
1.4.1.11	L-erythro-3,5-diaminohexanoate dehydrogenase	90.5850665	0.268412172	0.48550194	0.552854994	0.580362708	0.999677092
6.3.4.5	argininosuccinate synthase	292.8803441	-0.01656436	0.190388767	-0.087002823	0.930669268	0.999677092
3.5.1.118	gamma-glutamyl hercynylcysteine S-oxide hydrolase	0.812531649	-0.526777175	3.218096575	-0.163692159	0.869973491	0.999677092
1.5.-.-	#N/A	6.74317746	0.857678454	0.96601334	0.887853633	0.374619489	0.999677092
3.4.19.13	gamma-glutamyltranspeptidase / glutathione hydrolase	38.94457833	-0.468495095	0.757639381	-0.618361593	0.536337009	0.999677092
2.7.7.43	N-acylneuraminate cytidylyltransferase	11.83068856	-1.120147202	1.081706593	-1.035537002	0.300418195	0.999677092
2.7.1.67	phosphatidylinositol 4-kinase A	1.710812699	-0.289447854	3.548758558	-0.08156313	0.934994122	0.999677092
1.1.1.35	3-hydroxyacyl-CoA dehydrogenase	4.746715694	-2.94211855	1.476616121	-1.99247354	0.046319126	0.999677092
2.8.4.4	ribosomal protein S12 methylthiotransferase	623.2588148	0.283373604	0.274196714	1.033468272	0.301384811	0.999677092
1.1.1.58	tagaturonate reductase	243.3490786	0.35360766	0.343289684	1.030056179	0.302983634	0.999677092
2.2.1.1	transketolase	1381.337546	-0.031902017	0.218079141	-0.146286419	0.883695287	0.999677092
2.6.1.76	diaminobutyrate-2-oxoglutarate transaminase	5.447023162	0.734346967	1.419235799	0.517424213	0.604860061	0.999677092
3.4.24.40	serralysin	0.231763367	-0.887011995	4.129302346	-0.214809166	0.829916106	0.999677092
2.8.3.16	formyl-CoA transferase	2.442937505	-0.913090117	1.884884276	-0.48442768	0.628082378	0.999677092
3.1.3.97	3',5'-nucleoside bisphosphate phosphatase	1311.671976	-0.531624388	0.724627645	-0.733651816	0.463160972	0.999677092
4.2.1.90	L-rhamnonate dehydratase	2.379476149	-0.906198546	1.871271763	-0.484268808	0.62819511	0.999677092
3.6.1.13	ADP-ribose pyrophosphatase	291.3683185	-0.406278441	0.440736913	-0.921816234	0.356624438	0.999677092
1.1.-.-	#N/A	107.6860614	-0.732542944	0.493287839	-1.485021293	0.137538184	0.999677092
3.1.1.11	pectinesterase	172.979135	0.916533868	0.489030643	1.874184946	0.060904938	0.999677092
4.1.1.81	threonine-phosphate decarboxylase	111.5133339	-0.030308428	0.323598407	-0.093660623	0.925378751	0.999677092
3.6.3.54	copper-transporting P-type ATPase V	180.24383	0.444712521	0.302186422	1.47164958	0.14111553	0.999677092
2.3.2.18	peptidoglycan pentaglycine glycine transferase (the fourth and	0.020495196	0.108319359	4.142686886	0.026147127	0.979139988	0.999677092
3.5.4.28	5-methylthioadenosine/S-adenosylhomocysteine deaminase	646.4515798	-0.927056953	0.774640676	-1.19675739	0.231401131	0.999677092
1.6.5.8	#N/A	428.1980446	-0.435411445	0.43848736	-0.992985169	0.320717182	0.999677092

5.3.2.6	4-oxalocrotonate tautomerase	9.196708722	-0.427655774	1.287078768	-0.332268533	0.739686498	0.999677092
1.2.4.1	pyruvate dehydrogenase (acetyl-transferring)	104.4906236	-0.507691071	0.815344901	-0.622670321	0.53350119	0.999677092
3.5.3.23	succinylarginine dihydrolase	0.099032361	0.097008819	4.143282072	0.02341352	0.98132042	0.999677092
5.1.99.8	7,8-dihydroneopterin aldolase/epimerase/oxygenase	59.65369116	-0.143080167	0.601960295	-0.237690372	0.812121251	0.999677092
1.1.1.37	malate dehydrogenase	310.0391925	-0.449712434	0.405330647	-1.109495265	0.267216586	0.999677092
2.7.1.197	PTS system, mannitol-specific IIA component	35.32517345	-0.173060855	0.536061982	-0.322837397	0.746818389	0.999677092
3.6.1.7	acylphosphatase	266.5174633	-0.76507725	0.654405901	-1.169117284	0.242356381	0.999677092
2.1.1.220	tRNA (adenine57-N1/adenine58-N1)-methyltransferase catalyti	3.95831283	-0.195393474	1.71238583	-0.114105986	0.909153777	0.999677092
3.4.22.48	staphopain A	0.245162986	-3.718312875	4.143731151	-0.897334489	0.369540458	0.999677092
2.7.4.22	uridylate kinase	517.0140077	-0.039304074	0.265327653	-0.148134102	0.882236937	0.999677092
1.3.1.88	tRNA-dihydrouridine synthase 1	0.249773869	-0.680453166	4.123822835	-0.165005431	0.868939707	0.999677092
1.1.1.22	UDPglucose 6-dehydrogenase	273.6119241	-0.046326862	0.347212464	-0.133425112	0.893857186	0.999677092
4.2.1.10	#N/A	252.2617019	0.516423821	0.274213551	1.883290666	0.059660988	0.999677092
3.4.19.-	#N/A	72.96599458	0.24478463	0.423235447	0.578365144	0.563017621	0.999677092
3.2.1.184	GDP/UDP-N,N'-diacetylbacillosamine 2-epimerase (hydrolysing)	9.274406239	-0.872128063	0.834395206	-1.045221805	0.295920468	0.999677092
3.4.22.37	gingipain R	0.847080334	1.987884878	4.088705099	0.486189351	0.626832921	0.999677092
6.3.5.6	aspartyl-tRNA(Asn)/glutamyl-tRNA(Gln) amidotransferase subu	500.7375315	0.039953057	0.457481588	0.087332601	0.930407141	0.999677092
2.1.1.320	type II protein arginine methyltransferase	0.416289782	-0.751854895	4.112195485	-0.182835397	0.854927174	0.999677092
4.1.1.4	acetoacetate decarboxylase	0.197769159	1.563293498	4.132320925	0.378308831	0.705201186	0.999677092
4.6.1.13	1-phosphatidylinositol phosphodiesterase	17.26687161	-0.433070617	0.907014435	-0.477468274	0.633028707	0.999677092
3.4.11.6	aminopeptidase B	12.58645714	-1.106351009	1.403234013	-0.788429441	0.430445555	0.999677092
3.2.2.21	DNA-3-methyladenine glycosylase II	50.9815646	0.15953003	0.402148397	0.396694433	0.691592806	0.999677092
2.5.1.61	hydroxymethylbilane synthase	68.00050909	-0.073295236	0.369720206	-0.198245146	0.842853267	0.999677092
6.1.1.13	D-alanine--poly(phosphoribitol) ligase subunit 1	16.40246966	-0.444050892	0.6533418	-0.679660924	0.496719184	0.999677092
4.1.1.20	diaminopimelate decarboxylase	541.1671212	-0.11188671	0.429717128	-0.260372936	0.794576117	0.999677092
6.3.4.4	adenylosuccinate synthase	684.2271798	-0.144198858	0.297020549	-0.485484452	0.627332739	0.999677092
3.6.5.4	signal recognition particle subunit SRP54	622.0072358	-0.425574395	0.415650622	-1.023875274	0.30589419	0.999677092
1.1.1.132	GDP-mannose 6-dehydrogenase	5.365709567	-0.663976104	2.001752241	-0.331697445	0.74011773	0.999677092
5.3.1.5	xylose isomerase	119.6721669	0.329595569	0.454386015	0.725364686	0.468228332	0.999677092
4.2.2.23	rhamnogalacturonan endolyase	83.38770096	-0.22659616	0.56451099	-0.401402567	0.68812376	0.999677092
1.17.7.4	4-hydroxy-3-methylbut-2-en-1-yl diphosphate reductase	340.093813	-0.405018677	0.418120025	-0.968666059	0.332711832	0.999677092
4.1.1.74	indolepyruvate decarboxylase	0.623578467	-2.217551075	4.113038938	-0.539151491	0.589782329	0.999677092
4.2.2.7	heparin lyase	6.977243599	-1.275319434	1.271460336	-1.00303517	0.315843892	0.999677092
4.2.1.19	imidazoleglycerol-phosphate dehydratase / histidinol-phosphat	179.9053962	-0.159611833	0.208631896	-0.765040419	0.444247477	0.999677092
6.3.-.-	#N/A	0.185059353	-0.055940128	4.1339311	-0.013531945	0.9892034	0.999677092
2.7.12.2	mitogen-activated protein kinase kinase 1	0.552060613	-0.768352046	4.107262175	-0.187071585	0.851604507	0.999677092
2.7.1.107	diacylglycerol kinase (ATP)	109.2477564	-0.826348455	0.415219043	-1.990150667	0.046574341	0.999677092
5.1.3.14	UDP-N-acetylglucosamine 2-epimerase (non-hydrolysing)	267.9615043	-0.122351273	0.227890401	-0.53688647	0.591346038	0.999677092
3.4.11.-	#N/A	205.27062	-0.009949589	0.376324661	-0.026438844	0.978907312	0.999677092
3.1.3.89	5'-deoxynucleotidase	496.8947972	-0.409745952	1.075758999	-0.3808901	0.703284799	0.999677092
4.1.1.41	#N/A	2.58784862	-0.107461367	3.145836808	-0.034159867	0.972749669	0.999677092
3.1.3.57	inositol polyphosphate 1-phosphatase	0.363965079	1.355361956	4.114918841	0.329377567	0.741870322	0.999677092
2.4.1.18	1,4-alpha-glucan branching enzyme	1083.344365	0.073381039	0.287333079	0.255386673	0.798424463	0.999677092
4.1.1.37	uroporphyrinogen decarboxylase	83.4923204	0.239115578	0.558713081	0.427975622	0.668668869	0.999677092
2.7.1.195	PTS system, 2-O-A-mannosyl-D-glycerate-specific IIA componer	0.248796225	-1.247357197	4.12644119	-0.302284012	0.762435565	0.999677092
1.11.1.5	cytochrome c peroxidase	35.45100758	0.339706678	0.587445279	0.578277995	0.563076449	0.999677092
5.1.1.20	L-Ala-D/L-Glu epimerase	206.6439338	-0.230334011	0.646577856	-0.356235538	0.72166418	0.999677092
2.6.1.109	8-amino-3,8-dideoxy-alpha-D-manno-octulosonate transaminas	0.052512875	-0.212523182	4.139416356	-0.05134134	0.959053527	0.999677092
3.5.99.2	hydroxymethylpyrimidine/phosphomethylpyrimidine kinase / t	29.66472934	0.454048209	0.727895337	0.623782274	0.532770582	0.999677092
3.2.1.86	#N/A	1540.806296	-0.488416914	0.555814109	-0.878741482	0.379541461	0.999677092
5.4.99.62	D-ribose pyranase	11.97500773	-0.456279333	1.026657363	-0.444431949	0.656730319	0.999677092
6.3.2.4	D-alanine-D-alanine ligase	331.7670026	-0.234583215	0.190770638	-1.229661007	0.218824074	0.999677092
2.7.7.79	tRNA(His) guanylyltransferase	1.231597149	0.325260929	4.09868614	0.079357364	0.93674838	0.999677092
6.3.1.-	#N/A	0.197426127	1.266475772	4.120831992	0.307334969	0.758588421	0.999677092
5.1.3.8	N-acylglucosamine 2-epimerase	139.7091188	-0.89884662	0.438253943	-2.050972125	0.040269661	0.999677092
2.7.4.3	adenylate kinase	755.136847	0.052922175	0.351987348	0.150352493	0.88048652	0.999677092
2.4.1.247	beta-D-galactosyl-(1->4)-L-rhamnose phosphorylase	2.458672492	-1.468160726	1.874021096	-0.783428068	0.433375779	0.999677092
6.1.1.18	glutaminyl-tRNA synthetase	439.2984639	-0.331424379	0.184831309	-1.793118169	0.072954031	0.999677092
1.1.5.3	glycerol-3-phosphate dehydrogenase	393.6201507	0.208040149	0.332084891	0.626466769	0.531008827	0.999677092
2.3.1.247	3-keto-5-aminohexanoate cleavage enzyme	82.61576769	0.2446007	0.47943181	0.510188717	0.609919256	0.999677092
1.1.1.380	L-gulonate 5-dehydrogenase	17.29678145	-0.106717542	0.76113169	-0.140209038	0.888494831	0.999677092
1.1.1.262	4-hydroxythreonine-4-phosphate dehydrogenase	161.576444	-0.001377547	0.253504591	-0.005434011	0.995664308	0.999677092
2.1.2.2	phosphoribosylglycinamide formyltransferase	412.4568963	-0.258374989	0.118449562	-2.181308105	0.029160635	0.999677092
2.1.1.131	precorrin-3B C17-methyltransferase	100.1396827	-0.115027705	0.358043078	-0.32126778	0.748007471	0.999677092
2.3.1.35	glutamate N-acetyltransferase / amino-acid N-acetyltransferase	176.3579632	-0.089948847	0.249185911	-0.360970837	0.718121248	0.999677092
2.7.1.151	#N/A	2.104401683	-0.145249648	4.096480433	-0.035457181	0.97171519	0.999677092
2.3.1.12	pyruvate dehydrogenase E2 component (dihydrolipoamide ace	87.93707027	0.207808903	0.851145095	0.244152147	0.807112987	0.999677092
4.1.2.13	fructose 1,6-bisphosphate aldolase/phosphatase	1639.603458	-0.24773821	0.46744711	-0.529981263	0.596124922	0.999677092
2.4.1.186	glycogenin	0.70344152	0.468269451	4.121473076	0.113617011	0.909541402	0.999677092
3.1.3.81	phosphatidylglycerophosphatase B	0.236562323	-0.696732136	4.135351276	-0.168481972	0.866204126	0.999677092
5.3.3.3	4-hydroxybutyryl-CoA dehydratase / vinylacetyl-CoA-Delta-ison	56.64405634	-0.547322816	0.422429928	-1.295653505	0.195094886	0.999677092
1.2.1.21	lactaldehyde dehydrogenase / glycolaldehyde dehydrogenase	20.27192498	-0.873084179	0.570369491	-1.530734362	0.125835058	0.999677092
3.6.3.41	heme exporter protein A	3.666482455	1.909081666	1.450009947	1.316599013	0.18797307	0.999677092
2.7.10.-	#N/A	1.425729739	-0.215971302	1.561812985	-0.138282435	0.890017207	0.999677092
5.3.1.24	phosphoribosylanthranilate isomerase	103.2707371	-0.490702485	0.492492648	-0.996365098	0.319072785	0.999677092
5.4.99.18	5-(carboxyamino)imidazole ribonucleotide mutase	295.3770587	-0.123560004	0.237428201	-0.520409974	0.60277786	0.999677092
2.6.1.36	L-lysine 6-transaminase	2.906010432	0.184967516	3.833718294	0.04824755	0.961518955	0.999677092
2.5.1.-	#N/A	278.6566298	-0.039716897	0.691643517	-0.057423941	0.954207492	0.999677092
2.7.7.2	FAD synthetase	334.3661825	0.38971942	0.228212465	1.707704352	0.087691214	0.999677092
2.5.1.21	farnesyl-diphosphate farnesyltransferase	3.986138423	0.424024291	1.692698931	0.250501896	0.802199239	0.999677092
4.2.1.47	GDPmannose 4,6-dehydratase	191.2217744	-0.413631239	0.433114119	-0.955016751	0.339569226	0.999677092
2.3.1.19	phosphate butyryltransferase	55.23559176	-0.452706058	0.476691531	-0.949683451	0.342273121	0.999677092
1.7.1.13	7-cyano-7-deazaguanine reductase	60.68859191	-0.005230176	0.556546288	-0.009397558	0.992501944	0.999677092
6.1.1.1	tyrosyl-tRNA synthetase	369.2065589	0.262508554	0.219358093	1.196712416	0.231418666	0.999677092
4.2.1.33	3-isopropylmalate dehydratase	590.1564115	-0.25963912	0.156032278	-1.664009031	0.096110625	0.999677092
4.1.1.68	#N/A	0.157074332	0.448526278	4.143731151	0.108242128	0.913803628	0.999677092
3.4.11.23	PepB aminopeptidase	3.030118032	-2.115578764	2.808327656	-0.753323338	0.451255638	0.999677092

3.1.3.1	alkaline phosphatase	149.18058	-0.268564888	0.418381894	-0.641913266	0.5209295	0.999677092
2.7.8.26	adenosylcobinamide-GDP ribazoletransferase	117.5949016	-0.110823604	0.39496877	-0.28058827	0.779026214	0.999677092
1.1.3.15	glycolate oxidase	168.3993579	-0.117900592	0.414900512	-0.284165937	0.776283218	0.999677092
6.3.2.36	4-phosphopantoate---beta-alanine ligase	2.229993848	-0.099472791	4.096170556	-0.024284338	0.980625806	0.999677092
3.1.3.16	protein phosphatase	407.6783791	0.209103258	0.284390793	0.735267326	0.462176703	0.999677092
1.4.1.16	diaminopimelate dehydrogenase	191.3264728	-0.502636376	0.345510771	-1.454763261	0.145734812	0.999677092
2.4.1.337	1,2-diacylglycerol 3-alpha-glucosyltransferase	134.8407682	0.083207736	0.528577988	0.157418087	0.87491536	0.999677092
3.1.3.5	5'-nucleotidase	334.6478068	0.189228657	0.322887135	0.58605202	0.557840558	0.999677092
2.7.9.3	selenide, water dikinase	162.5069829	-0.154152917	0.342857095	-0.449612736	0.652989703	0.999677092
3.2.1.52	beta-N-acetylhexosaminidase	1969.843787	-0.715395724	0.405128721	-1.765847955	0.077421361	0.999677092
3.1.1.45	carboxymethylenebutenolidase	0.306481125	0.102588948	4.140453896	0.024777223	0.980232659	0.999677092
3.6.3.27	#N/A	174.0743809	-0.807340077	0.532274539	-1.516773803	0.129323803	0.999677092
5.4.2.10	phosphoglucosamine mutase	492.1079234	0.19924882	0.270944831	0.735385206	0.462104929	0.999677092
3.8.1.5	haloalkane dehalogenase	0.039493742	0.43806451	4.143245313	0.105729803	0.915796734	0.999677092
3.4.21.89	signal peptidase I	629.1568785	-0.287245504	0.219775459	-1.306995355	0.191214289	0.999677092
2.7.11.24	mitogen-activated protein kinase 1/3	19.47956978	-0.522858392	4.093053207	-0.127742877	0.898352457	0.999677092
3.6.1.66	XTP/dITP diphosphohydrolase	350.6006094	-0.010658612	0.301971302	-0.035296772	0.971843097	0.999677092
4.1.3.36	naphthoate synthase	60.70473352	-0.16913903	0.468195589	-0.361257206	0.717907181	0.999677092
2.1.1.172	16S rRNA (guanine1207-N2)-methyltransferase	6.81937072	-0.961075036	1.227347027	-0.783050771	0.433597298	0.999677092
2.7.11.1	checkpoint serine/threonine-protein kinase	1323.478779	-0.242503695	0.247957054	-0.978006839	0.328070941	0.999677092
3.6.3.34	iron complex transport system ATP-binding protein	291.8109359	-0.098739321	0.379987256	-0.259849034	0.794980226	0.999677092
2.5.1.6	S-adenosylmethionine synthetase	664.9956883	-0.06198472	0.406470308	-0.152495076	0.878796474	0.999677092
2.5.1.54	3-deoxy-7-phosphoheptulonate synthase	4039.606101	-0.555990092	1.021215314	-0.544439634	0.586138978	0.999677092
2.7.7.8	polyribonucleotide nucleotidyltransferase	1007.659678	0.406678302	0.250377784	1.624258732	0.104320601	0.999677092
3.5.4.5	cytidine deaminase	296.4043363	0.153476421	0.275249079	0.557591043	0.577123674	0.999677092
5.5.1.27	D-galactarolactone cycloisomerase	0.422036594	-1.029401108	4.115796027	-0.250109845	0.802502402	0.999677092
3.2.1.25	beta-mannosidase	235.1877339	-0.293693455	0.28569527	-1.027995511	0.303951939	0.999677092
2.1.1.148	thymidylate synthase (FAD)	157.2032252	-0.043275914	0.318033248	-0.136073553	0.891763135	0.999677092
3.6.3.2	Mg2+-importing ATPase	52.92754109	-0.184607188	0.588447731	-0.313718922	0.753734528	0.999677092
4.1.1.70	glutaconyl-CoA decarboxylase	137.1694256	0.060270709	0.561795192	0.107282351	0.914564986	0.999677092
2.7.4.9	dTMP kinase	164.5735539	0.375376369	0.287191839	1.307057926	0.191193039	0.999677092
6.2.1.34	feruloyl-CoA synthase	0.103724382	0.448526166	4.143731151	0.108242101	0.913803649	0.999677092
2.7.1.176	UDP-N-acetylglucosamine kinase	2.748491948	1.963721075	1.269285962	1.547106904	0.12183747	0.999677092
2.4.1.291	N-acetylgalactosamine-N,N'-diacetylbacillosaminyl-diphospho-u	1.395090128	2.210634369	4.045169902	0.546487397	0.584730949	0.999677092
2.7.1.30	glycerol kinase	776.031683	0.392567614	0.356385019	1.1015267	0.27066749	0.999677092
2.9.1.1	L-seryl-tRNA(Ser) seleniumtransferase	31.33378398	1.273194797	0.658752968	1.932734819	0.053268874	0.999677092
1.4.3.16	L-aspartate oxidase	271.3226124	0.14111729	0.276749281	0.509910231	0.610114354	0.999677092
3.5.1.102	2-amino-5-formylamino-6-ribosylaminopyrimidin-4(3H)-one 5'-r	0.581356147	0.384484019	4.102497305	0.093719505	0.925331975	0.999677092
3.2.1.8	endo-1,4-beta-xylanase	983.5067301	1.161498187	0.803088823	1.446288571	0.14809628	0.999677092
2.4.1.257	alpha-1,3/alpha-1,6-mannosyltransferase	1.814404281	-0.192372527	4.09772309	-0.0469462	0.962556107	0.999677092
2.7.4.31	5-(aminomethyl)-3-furanmethanol phosphate kinase	2.223575051	0.281041859	4.023012746	0.069858555	0.94430624	0.999677092
2.1.1.10	homocysteine S-methyltransferase	5.268345962	-1.16644957	1.327606233	-0.878611098	0.379612176	0.999677092
3.6.3.32	glycine betaine/proline transport system ATP-binding protein	73.99996966	-0.636650988	0.628807381	-1.012473784	0.311311575	0.999677092
2.5.1.89	tritrans,polycis-undecaprenyl-diphosphate synthase [geranylge	0.762496501	-0.142212867	4.101048561	-0.034677196	0.972337145	0.999677092
1.17.1.8	4-hydroxy-tetrahydrodipicolinate reductase	419.5572778	-0.034957589	0.278974686	-0.125307388	0.900280204	0.999677092
3.1.1.83	epsilon-lactone hydrolase	8.852331657	-0.054118935	0.923992319	-0.058570763	0.953293999	0.999677092
2.1.1.80	chemotaxis protein methyltransferase CheR	51.42789199	0.140382968	0.631641141	0.222251146	0.824118381	0.999677092
1.3.1.91	tRNA-dihydrouridine synthase 2	0.173834568	0.510690127	4.121551259	0.123907261	0.901388704	0.999677092
2.3.1.15	glycerol-3-phosphate O-acyltransferase 1/2	513.1341149	-0.444564344	0.233038988	-1.907682264	0.056432295	0.999677092
1.17.1.1	CDP-4-dehydro-6-deoxyglucose reductase, E3	51.88480358	0.582525971	0.445277675	1.308230804	0.190795037	0.999677092
1.4.4.2	glycine dehydrogenase	313.8763903	0.261213832	0.419797633	0.622237505	0.533785708	0.999677092
2.8.1.9	molybdenum cofactor sulfurtransferase	0.582805694	-0.770921528	4.106500735	-0.187731983	0.851086757	0.999677092
4.1.1.32	phosphoenolpyruvate carboxykinase (GTP)	256.1271171	0.208212582	0.591123886	0.352231717	0.724664492	0.999677092
1.3.99.5	#N/A	0.280447894	-0.407384225	4.135627531	-0.098506024	0.921530489	0.999677092
2.3.1.30	serine O-acetyltransferase	298.4087406	-0.226673911	0.282046959	-0.803674367	0.421585062	0.999677092
3.2.1.-	#N/A	106.9534303	0.040598956	0.416923798	0.097377402	0.92242669	0.999677092
5.4.99.15	(1->4)-alpha-D-glucan 1-alpha-D-glucosylmutase	0.103724382	0.448526166	4.143731151	0.108242101	0.913803649	0.999677092
4.2.99.20	2-succinyl-6-hydroxy-2,4-cyclohexadiene-1-carboxylate synthas	4.655924674	-1.514946629	1.539263262	-0.984202421	0.325015996	0.999677092
4.2.1.9	dihydroxy-acid dehydratase	494.2953836	0.223467808	0.251951443	0.886947918	0.375106941	0.999677092
6.3.4.2	CTP synthase	447.5705848	0.057169737	0.229976343	0.248589645	0.803678219	0.999677092
1.12.99.-	#N/A	35.47611908	1.605413738	1.399108224	1.147455008	0.251193618	0.999677092
3.5.1.94	gamma-glutamyl-gamma-aminobutyrate hydrolase	0.020495196	0.108319359	4.142686886	0.026147127	0.979139988	0.999677092
1.1.1.363	glucose-6-phosphate 1-dehydrogenase	351.7397334	-0.146360707	0.637673798	-0.229522849	0.818462564	0.999677092
2.4.1.129	monofunctional glycosyltransferase	61.93255458	-0.204218562	0.368270826	-0.554533641	0.579213689	0.999677092
4.3.1.4	formiminotetrahydrofolate cyclodeaminase	55.2180708	0.270423629	0.665733088	0.406204278	0.684592511	0.999677092
2.1.1.268	tRNAThr (cytosine32-N3)-methyltransferase	0.833542549	-0.403332288	4.103224937	-0.098296412	0.921696927	0.999677092
2.7.1.21	thymidine kinase	135.8458384	0.261040298	0.389626135	0.669976357	0.502872862	0.999677092
3.4.23.5	cathepsin D	0.897866547	1.384074848	4.111035313	0.336673062	0.736363372	0.999677092
2.4.1.281	4-O-beta-D-mannosyl-D-glucose phosphorylase	101.0610914	0.071285082	0.536319867	0.132915237	0.894260417	0.999677092
3.1.3.32	bifunctional polynucleotide phosphatase/kinase	0.714858879	0.768939045	3.765560273	0.204203091	0.838194796	0.999677092
5.1.1.7	diaminopimelate epimerase	534.3515027	-0.94814744	0.598045208	-1.58541098	0.112872988	0.999677092
2.1.1.314	diphthine methyl ester synthase	1.408052998	-0.155350545	3.770446205	-0.041202165	0.967134728	0.999677092
2.1.1.206	tRNA (cytidine56-2'-O)-methyltransferase	0.478742931	-0.175170553	4.115501016	-0.042563603	0.96604941	0.999677092
1.1.1.291	2-hydroxymethylglutarate dehydrogenase	1.165624298	-0.42064818	4.10027854	-0.102590147	0.918288263	0.999677092
2.5.1.48	cystathionine gamma-synthase	39.17001989	-0.932076414	0.686231008	-1.358254588	0.174382915	0.999677092
6.3.3.1	phosphoribosylformylglycinamidine cyclo-ligase	403.1993717	-0.21224028	0.170956855	-1.241484467	0.214426833	0.999677092
6.3.2.12	#N/A	407.8113641	-0.127838191	0.197562493	-0.647077234	0.517581959	0.999677092
2.8.1.12	molybdopterin synthase catalytic subunit	4.69347111	1.9437593	1.452556122	1.338164681	0.18084276	0.999677092
3.6.4.6	vesicle-fusing ATPase	4.693647225	-0.437784606	2.052501633	-0.213293183	0.831098291	0.999677092
1.21.4.1	#N/A	273.8860975	-0.957547711	0.666138484	-1.43746043	0.150587209	0.999677092
2.7.1.130	tetraacyldisaccharide 4'-kinase	131.049192	-0.372279647	0.49120695	-0.757887582	0.448518285	0.999677092
1.2.1.8	betaine-aldehyde dehydrogenase	10.0171133	-0.060858143	1.153065982	-0.052779411	0.957907666	0.999677092
1.1.1.31	3-hydroxyisobutyrate dehydrogenase	19.43101592	0.336136845	0.399019651	0.842406743	0.39956032	0.999677092
2.4.1.320	beta-1,4-mannooligosaccharide/beta-1,4-mannosyl-N-acetylglu	163.8803014	0.123291893	0.350898711	0.351360349	0.725318026	0.999677092
2.7.6.1	ribose-phosphate pyrophosphokinase	590.4496245	0.015079986	0.339137225	0.044465734	0.964533165	0.999677092
4.1.2.20	2-dehydro-3-deoxyglucarate aldolase	2.922842527	-0.881995427	1.415532683	-0.62308376	0.533229481	0.999677092
1.1.1.86	ketol-acid reductoisomerase	435.8011911	0.005697889	0.315284375	0.018072221	0.985581239	0.999677092

5.1.3.3	aldose 1-epimerase	759.9440507	-0.071602758	0.372753123	-0.19209164	0.847670424	0.999677092
5.3.1.28	D-sedoheptulose 7-phosphate isomerase	48.71117419	0.333193076	0.432201854	0.770920053	0.44075432	0.999677092
2.3.1.47	8-amino-7-oxononanoate synthase	86.46061388	-0.982694361	0.664309089	-1.479272791	0.139067422	0.999677092
2.4.99.13	3-deoxy-D-manno-octulosonic-acid transferase	104.6062867	0.109755847	0.47516922	0.230982653	0.817328279	0.999677092
3.2.1.170	mannosylglycerate hydrolase	9.078498944	-0.159366898	1.498208658	-0.10637163	0.915287501	0.999677092
3.4.13.9	Xaa-Pro dipeptidase	53.83327966	-1.074855955	0.613524629	-1.751936115	0.079784794	0.999677092
2.4.2.48	7-cyano-7-deazaguanine tRNA-ribosyltransferase	0.499547738	-0.762859882	4.108896089	-0.185660544	0.852710972	0.999677092
3.6.1.26	CDP-diacylglycerol pyrophosphatase	0.081334971	-0.253576038	4.142921482	-0.061207059	0.951194308	0.999677092
2.1.1.290	tRNA wybutosine-synthesizing protein 4	0.052512875	-0.212523182	4.139416356	-0.05134134	0.959053527	0.999677092
2.1.1.229	alkylated DNA repair protein alkB homolog 8	0.035008583	-0.076612487	4.14032189	-0.018503993	0.985236792	0.999677092
2.1.1.294	O-antigen chain-terminating methyltransferase	2.5617666	0.849576774	3.783133496	0.2245696	0.822314117	0.999677092
1.14.14.5	alkanesulfonate monooxygenase	0.151201469	-0.536571892	4.136624996	-0.129712481	0.896793907	0.999677092
1.1.1.41	isocitrate dehydrogenase (NAD+)	37.24360312	-0.071696583	0.397614159	-0.180316976	0.85690373	0.999677092
2.3.1.24	Acyl-CoA-dependent ceramide synthase	1.915657591	-0.331553901	4.097093978	-0.080924163	0.935502264	0.999677092
2.7.1.5	rhamnulokinase	286.9725374	-0.614800182	0.367776267	-1.671668992	0.094589612	0.999677092
2.7.1.189	autoinducer-2 kinase	13.76266685	-0.202070544	0.862245332	-0.234353886	0.814710255	0.999677092
2.4.-.-	#N/A	68.04439895	-0.384075329	0.522428171	-0.735173466	0.462233857	0.999677092
3.2.2.9	adenosylhomocysteine nucleosidase	243.1991387	0.027328416	0.302876564	0.09022955	0.928104803	0.999677092
2.8.3.1	propionate CoA-transferase	89.25752976	-0.467799832	0.527311676	-0.887141045	0.375002968	0.999677092
4.1.1.48	indole-3-glycerol phosphate synthase	96.2007013	-0.210877696	0.474506353	-0.444414905	0.656742639	0.999677092
3.5.3.18	dimethylargininase	1.226516595	-0.178861568	2.733194221	-0.06544049	0.947823287	0.999677092
2.8.1.2	thiosulfate/3-mercaptopyruvate sulfurtransferase	7.144411477	-0.899785302	1.035412019	-0.869011839	0.384840655	0.999677092
2.1.3.-	#N/A	2.822043806	-0.740356973	2.511614451	-0.294773337	0.768167036	0.999677092
2.8.1.-	#N/A	58.92031517	0.776216549	0.694314057	1.117961738	0.263583335	0.999677092
3.6.3.7	#N/A	11.08064655	0.72012335	0.850530284	0.846675731	0.39717589	0.999677092
2.7.1.52	fucokinase	57.1794349	-1.185152043	0.662585057	-1.7886791	0.073666511	0.999677092
3.1.26.8	ribonuclease M5	72.41566023	-0.074558005	0.354778813	-0.210153487	0.833547881	0.999677092
4.1.3.30	methylisocitrate lyase	0.651710156	0.13451143	4.061864374	0.033115687	0.973582333	0.999677092
3.5.1.24	choloylglycine hydrolase	410.6257429	-0.189517427	0.399155819	-0.474795602	0.634932668	0.999677092
2.1.1.35	tRNA (uracil-5-)-methyltransferase	0.644054713	-2.22708864	4.108412173	-0.542080138	0.5877633	0.999677092
5.4.2.6	beta-phosphoglucomutase	14.7895933	-0.795628458	0.772000416	-1.030606256	0.302725501	0.999677092
2.5.1.29	geranylgeranyl diphosphate synthase, type III	364.885785	0.006277111	0.190902923	0.032881168	0.97376935	0.999677092
2.6.1.45	#N/A	1.130264311	1.625242212	3.554124565	0.457283413	0.64746735	0.999677092
1.3.1.93	very-long-chain enoyl-CoA reductase	0.999108385	-0.375581498	4.101773479	-0.091565636	0.927043155	0.999677092
1.1.1.69	gluconate 5-dehydrogenase	308.5135377	0.333537248	0.309868472	1.0763833	0.281755868	0.999677092
1.1.1.-	#N/A	3841.379493	1.142604963	0.786265865	1.453204334	0.146167029	0.999677092
2.7.1.146	ADP-dependent phosphofructokinase/glucokinase	1.596352927	1.859917272	2.884743243	0.644742743	0.519093912	0.999677092
5.4.3.8	glutamate-1-semialdehyde 2,1-aminomutase	119.9576647	-0.123299226	0.42023341	-0.293406527	0.769211437	0.999677092
1.1.99.1	choline dehydrogenase	0.295593336	-0.235135354	4.116822682	-0.057115735	0.954453002	0.999677092
3.6.3.17	simple sugar transport system ATP-binding protein	2360.199292	0.230032415	0.399077501	0.576410384	0.564337828	0.999677092
1.4.1.24	3-dehydroquinate synthase II	3.146725448	0.823999661	1.757091573	0.468956584	0.63910067	0.999677092
4.2.2.17	inulin fructotransferase (DFA-I-forming)	0.177059089	1.378457516	4.125697282	0.334115041	0.738292751	0.999677092
3.6.3.29	molybdate transport system ATP-binding protein	78.3810439	0.067311148	0.536213914	0.125530401	0.900103659	0.999677092
3.1.3.64	myotubularin	3.613934552	0.021085684	2.802326683	0.007524349	0.993996495	0.999677092
3.1.3.3	phosphoserine phosphatase	138.4424214	-0.263826089	0.398863436	-0.661444658	0.508327195	0.999677092
2.7.8.37	alpha-D-ribose 1-methylphosphonate 5-triphosphate synthase	11.54475179	-0.474731578	1.352260112	-0.351065282	0.725539373	0.999677092
2.1.1.215	tRNA (guanine26-N2/guanine27-N2)-dimethyltransferase	2.22570527	0.021617515	3.267377318	0.006616167	0.994721101	0.999677092
2.6.1.51	alanine-glyoxylate transaminase / serine-glyoxylate transamina	1.130264311	1.625242212	3.554124565	0.457283413	0.64746735	0.999677092
1.2.4.4	3-methyl-2-oxobutanoate dehydrogenase (2-methylpropanoyl-	106.8812829	-0.194245972	0.539998754	-0.359715593	0.71905983	0.999677092
3.4.22.15	cathepsin L	89.02316587	3.436265409	1.558586887	2.204731374	0.02747295	0.999677092
3.1.2.28	1,4-dihydroxy-2-naphthoyl-CoA hydrolase	0.020495196	0.108319359	4.142686886	0.026147127	0.979139988	0.999677092
2.7.1.203	PTS system, D-glucosaminate-specific IIA component	3.533453409	-1.583479473	1.332180521	-1.188637311	0.234582419	0.999677092
3.2.1.187	beta-L-arabinobiosidase	5.256782339	0.65012846	1.571020747	0.413825509	0.679001901	0.999677092
3.2.1.80	fructan beta-fructosidase	126.3111575	-0.16672645	0.419671073	-0.397278871	0.691161827	0.999677092
1.12.1.4	iron-hydrogenase subunit alpha	0.249773869	-0.680453166	4.123822835	-0.165005431	0.868939707	0.999677092
5.1.3.32	L-rhamnose mutarotase	119.1287929	-0.304406634	0.370931313	-0.820654992	0.411842814	0.999677092
2.3.1.199	elongation of very long chain fatty acids protein 6	0.416302691	-3.502189481	4.143731151	-0.845177777	0.398011588	0.999677092
2.1.1.184	23S rRNA (adenine-N6)-dimethyltransferase	38.39874982	-0.54128683	0.448810482	-1.206047656	0.227799116	0.999677092
5.4.3.3	beta-lysine 5,6-aminomutase alpha subunit	144.6425607	0.271367564	0.482325095	0.56262377	0.573691102	0.999677092
2.7.1.39	homoserine kinase	148.0990502	-0.06663123	0.294678395	-0.226115086	0.821111912	0.999677092
1.3.1.87	2,3-dihydroxy-2,3-dihydrophenylpropionate dehydrogenase	0.111464417	0.448520484	4.143731151	0.10824073	0.913804737	0.999677092
2.4.1.142	beta-1,4-mannosyltransferase	2.114580348	-0.233971314	4.090419757	-0.05719983	0.954386014	0.999677092
2.4.1.258	alpha-1,3-mannosyltransferase	0.723054713	-0.508569129	4.103305808	-0.123941318	0.901361738	0.999677092
2.1.1.282	tRNA wybutosine-synthesizing protein 3	0.510438089	-0.367669842	4.109866693	-0.089460284	0.928716116	0.999677092
4.-.-.-	#N/A	0.043561417	1.161031658	4.14287533	0.280247791	0.779287403	0.999677092
4.1.99.3	deoxyribodipyrimidine photo-lyase	0.334769309	-1.078136902	4.126864619	-0.26124843	0.793900933	0.999677092
4.1.2.9	xylulose-5-phosphate/fructose-6-phosphate phosphoketolase	113.3229537	0.143142376	0.30375757	0.471238877	0.637470159	0.999677092
2.7.1.43	glucuronokinase	1.764226465	0.030695528	4.096491363	0.007493127	0.994021406	0.999677092
1.1.1.157	3-hydroxybutyryl-CoA dehydrogenase	1028.321371	-0.268831782	0.55833511	-0.481488227	0.630169545	0.999677092
1.1.1.369	myo-inositol 2-dehydrogenase / D-chiro-inositol 1-dehydrogena	75.32692191	-0.418773873	0.481635504	-0.86948298	0.384583013	0.999677092
1.1.1.34	hydroxymethylglutaryl-CoA reductase (NADPH)	5.653749803	-0.264396119	2.571749532	-0.102807881	0.91811545	0.999677092
3.4.22.34	legumain	232.7625314	1.997288503	1.46098728	1.367081377	0.1715998	0.999677092
2.1.1.86	#N/A	31.96680807	2.249722909	1.59692106	1.408787802	0.158897923	0.999677092
1.14.12.17	nitric oxide dioxygenase	9.04465669	0.491236563	1.05383894	0.466140076	0.641115239	0.999677092
2.7.8.-	#N/A	803.4885897	-0.037578022	0.260183897	-0.1444287	0.885161955	0.999677092
3.1.5.1	dGTPase	378.9543563	-0.002529252	0.243855932	-0.010371913	0.991724559	0.999677092
3.4.24.-	#N/A	2958.235347	0.09963354	0.175489165	0.56774753	0.570206413	0.999677092
2.1.2.13	UDP-4-amino-4-deoxy-L-arabinose formyltransferase / UDP-glu	1.285501156	-2.177651273	2.073352696	-1.05030431	0.293578224	0.999677092
3.1.3.24	sucrose-6-phosphatase	973.9245196	0.382037566	0.333691546	1.144882364	0.252257891	0.999677092
2.4.1.241	digalactosyldiacylglycerol synthase	0.104075673	0.43806451	4.143245313	0.105729803	0.915796734	0.999677092
3.2.1.93	trehalose-6-phosphate hydrolase	14.12340887	-0.76906373	0.717598239	-1.071719088	0.283846225	0.999677092
2.5.1.56	N-acetylneuraminate synthase	25.93261642	0.106868074	0.658986439	0.162170369	0.871171693	0.999677092
2.7.1.15	ribokinase	109.9336484	-0.339652084	0.308513633	-1.100930551	0.270926884	0.999677092
3.6.3.24	nickel transport system ATP-binding protein	10.48207584	-2.684292389	1.421011968	-1.88900055	0.058891755	0.999677092
2.3.1.117	2,3,4,5-tetrahydropyridine-2,6-dicarboxylate N-succinyltransfer	34.45067098	0.696272753	0.561000877	1.241125963	0.21455922	0.999677092
2.3.2.-	#N/A	18.67247501	-0.655159485	1.091850993	-0.600044777	0.548476395	0.999677092
2.4.1.46	1,2-diacylglycerol 3-beta-galactosyltransferase	5.249527659	-0.693316627	4.019547446	-0.17248624	0.863055278	0.999677092

1.13.11.15	3,4-dihydroxyphenylacetate 2,3-dioxygenase	0.110533983	-0.307374933	4.142162324	-0.074206395	0.940846158	0.999677092
2.6.1.87	UDP-4-amino-4-deoxy-L-arabinose-oxoglutarate aminotransfera	0.040990391	-0.099691597	4.140771598	-0.024075609	0.980792299	0.999677092
4.1.2.57	sulfofructosephosphate aldolase	0.103724382	0.448526166	4.143731151	0.108242101	0.913803649	0.999677092
5.99.1.-	#N/A	287.1172249	0.167353236	0.344196729	0.486213904	0.626815514	0.999677092
3.6.1.67	dihydroneopterin triphosphate diphosphatase	2.254603037	-1.175425867	1.651977924	-0.711526377	0.476758112	0.999677092
2.2.1.2	transaldolase	460.0355829	-0.354798776	0.604474538	-0.586954046	0.55723457	0.999677092
6.1.1.6	#N/A	607.4222191	-0.121561003	0.288916487	-0.420747893	0.673939184	0.999677092
4.3.1.7	#N/A	47.02469326	-0.779935257	0.478535302	-1.629838496	0.103135635	0.999677092
6.1.1.24	nondiscriminating glutamyl-tRNA synthetase	13.45465817	-0.595595131	0.633062113	-0.940816263	0.346799025	0.999677092
2.4.1.25	4-alpha-glucanotransferase	679.9918035	-0.046454389	0.292037714	-0.159069827	0.87361386	0.999677092
2.4.1.182	lipid-A-disaccharide synthase	104.0503707	-0.386702755	0.573806353	-0.673925537	0.500358656	0.999677092
2.2.1.9	2-succinyl-5-enolpyruvyl-6-hydroxy-3-cyclohexene-1-carboxylat	95.19445505	-0.049167928	0.354134651	-0.138839642	0.889576868	0.999677092
3.2.1.165	exo-1,4-beta-D-glucosaminidase	2.50673581	0.151856824	2.162835246	0.070211924	0.944024984	0.999677092
1.1.1.18	myo-inositol 2-dehydrogenase / D-chiro-inositol 1-dehydrogena	75.32692191	-0.418773873	0.481635504	-0.86948298	0.384583013	0.999677092
2.4.1.7	sucrose phosphorylase	40.52910576	-0.650553142	0.769598611	-0.84531486	0.397935067	0.999677092
3.5.4.2	adenine deaminase	259.7447852	-0.148438131	0.462027465	-0.321275556	0.748001579	0.999677092
5.2.1.8	peptidylprolyl isomerase	2610.435006	0.721635176	0.412040544	1.751369343	0.07988231	0.999677092
5.1.1.18	serine racemase	30.0924605	-0.429623602	0.785541149	-0.546914191	0.584437686	0.999677092
2.6.1.13	ornithine--oxo-acid transaminase	35.59584511	-0.472734384	0.359351184	-1.315521988	0.188334533	0.999677092
1.10.3.10	#N/A	9.229660608	-1.633593663	1.728907026	-0.944870741	0.344724871	0.999677092
2.7.7.6	DNA-directed RNA polymerase	5724.757651	0.300135273	0.344369371	0.87155043	0.383453683	0.999677092
4.1.3.39	4-hydroxy 2-oxovalerate aldolase	57.95554725	-0.358379831	0.441821363	-0.811141926	0.417284172	0.999677092
1.21.4.2	glycine/sarcosine/betaine reductase complex component A	2335.584865	0.904425731	0.537066512	1.684010659	0.092179609	0.999677092
6.4.1.1	pyruvate carboxylase	480.0100183	-0.042485771	0.369863722	-0.114868716	0.908549182	0.999677092
2.7.2.4	aspartate kinase	755.1354989	0.489520702	0.369229808	1.325788686	0.184909702	0.999677092
2.3.1.23	lysophosphatidylcholine acyltransferase / lyso-PAF acetyltransf	1.237997841	-0.211775872	4.10025245	-0.051649472	0.958807999	0.999677092
4.3.1.1	aspartate ammonia-lyase	327.7532297	-0.011906054	0.464654467	-0.025623458	0.979557676	0.999677092
2.3.2.23	ubiquitin-conjugating enzyme E2 R	9.282151261	-0.223989071	4.093737118	-0.05471506	0.956365471	0.999677092
2.7.1.1	hexokinase	23.12651405	-0.587305089	0.792285417	-0.741279691	0.458523873	0.999677092
5.1.3.1	ribulose-phosphate 3-epimerase	405.4522483	0.452615521	0.330641923	1.368899371	0.171030732	0.999677092
1.1.1.302	2,5-diamino-6-(ribosylamino)-4(3H)-pyrimidinone 5'-phosphate	0.582805694	-0.770921528	4.106500735	-0.187731983	0.851086757	0.999677092
3.1.1.-	#N/A	541.0085476	-0.035506286	0.303392901	-0.117030708	0.906835719	0.999677092
1.6.99.3	NADH dehydrogenase	195.4290965	-0.425841756	0.679675779	-0.626536606	0.530963034	0.999677092
1.7.99.4	#N/A	62.74469958	0.813927741	0.572423579	1.421897648	0.155055966	0.999677092
3.5.4.31	5-methylthioadenosine/S-adenosylhomocysteine deaminase	646.4515798	-0.927056953	0.774640676	-1.19675739	0.231401131	0.999677092
3.1.3.90	maltose 6'-phosphate phosphatase	7.623733685	-0.844852251	0.745649027	-1.133042787	0.257196286	0.999677092
4.1.1.15	glutamate decarboxylase	198.5891402	-0.837499699	0.6717038	-1.246828883	0.212460248	0.999677092
4.1.1.5	acetolactate decarboxylase	2.950417394	-0.521588101	1.933397083	-0.269778053	0.787331008	0.999677092
4.6.1.17	cyclic pyranopterin monophosphate synthase	67.32316178	-0.34079917	0.586168542	-0.58140133	0.560970001	0.999677092
2.3.1.29	glycine C-acetyltransferase	448.8884273	0.625360202	0.405056021	1.543885709	0.122616023	0.999677092
2.1.1.190	23S rRNA (uracil1939-C5)-methyltransferase	470.4162853	-0.121589166	0.29397808	-0.413599428	0.679167492	0.999677092
1.4.7.1	glutamate synthase (ferredoxin)	49.96845184	-0.434458101	0.642169784	-0.676547094	0.498693357	0.999677092
3.1.26.-	#N/A	361.3170482	-0.250977713	0.21384424	-1.173647292	0.24053633	0.999677092
3.2.1.156	oligosaccharide reducing-end xylanase	47.24343399	0.25703007	0.695967047	0.369313563	0.711894017	0.999677092
3.6.3.16	arsenite/tail-anchored protein-transporting ATPase	8.08124422	0.262065068	1.046588737	0.250399282	0.802278585	0.999677092
6.3.5.11	cobyrinic acid a,c-diamide synthase	147.2244454	-0.268935886	0.314339714	-0.855558093	0.392242258	0.999677092
2.7.11.5	isocitrate dehydrogenase kinase/phosphatase	0.941316252	-1.600097639	4.096107688	-0.390638567	0.696064414	0.999677092
2.1.2.1	glycine hydroxymethyltransferase	400.2794024	-0.017023354	0.231710514	-0.0734682	0.941433548	0.999677092
2.6.1.44	alanine-glyoxylate transaminase / (R)-3-amino-2-methylpropior	1.130264311	1.625242212	3.554124565	0.457283413	0.64746735	0.999677092
4.2.3.12	6-pyruvoyltetrahydropterin/6-carboxytetrahydropterin synthas	143.680634	0.828945086	0.403172058	2.056057879	0.039776938	0.999677092
3.6.4.13	ATP-dependent helicase HrpA	1266.701025	-0.33115844	0.431180463	-0.76802747	0.442470865	0.999677092
1.1.1.29	glycerate dehydrogenase	213.4454083	0.02641259	0.20062861	0.131649172	0.895261791	0.999677092
4.2.1.83	4-oxalmesaconate hydratase	0.709423283	-2.599668333	3.864105194	-0.672773696	0.501091274	0.999677092
2.3.2.27	protein neuralized	11.57495195	-0.285875107	4.093521669	-0.069835982	0.944324207	0.999677092
1.6.99.1	NADPH2 dehydrogenase	0.530483847	-1.76016459	4.118769147	-0.427352087	0.6691229	0.999677092
2.5.1.87	ditrans,polycis-polyprenyl diphosphate synthase	0.052512875	-0.212523182	4.139416356	-0.05134134	0.959053527	0.999677092
2.7.7.72	tRNA nucleotidyltransferase (CCA-adding enzyme)	197.4056266	0.176045518	0.273471718	0.643743052	0.519742067	0.999677092
3.2.2.1	purine nucleosidase	27.4703256	0.182492379	0.589582331	0.309528236	0.756919737	0.999677092
2.1.1.192	23S rRNA (adenine2503-C2)-methyltransferase	509.0918047	-0.307670923	0.20431813	-1.505842495	0.132107611	0.999677092
1.4.1.4	glutamate dehydrogenase (NADP+)	1096.26824	-0.058970237	0.309899837	-0.190288054	0.849083414	0.999677092
2.7.1.58	2-dehydro-3-deoxygalactonokinase	5.245567172	-1.491650675	1.416722538	-1.052888364	0.29239216	0.999677092
1.5.3.1	sarcosine oxidase	12.21788258	-0.423571142	0.865718291	-0.489271332	0.624649614	0.999677092
2.5.1.77	#N/A	1.536021083	-0.306619509	4.098437413	-0.074813759	0.940362894	0.999677092
5.3.1.31	sulfoquinovose isomerase	0.13101338	-0.281844306	4.138534189	-0.068102447	0.945704082	0.999677092
2.7.4.24	inositol-hexakisphosphate/diphosphoinositol-pentakisphospha	4.421491168	-0.044615756	2.742138043	-0.016270427	0.987018651	0.999677092
4.2.99.18	DNA-(apurinic or apyrimidinic site) lyase	479.2168979	-0.500488354	0.43637307	-1.146927683	0.25141151	0.999677092
3.2.1.172	unsaturated rhamnogalacturonyl hydrolase	155.0754149	0.177094068	0.462666429	0.382768356	0.701891526	0.999677092
2.4.2.8	hypoxanthine phosphoribosyltransferase	321.3459054	-0.10302176	0.25454159	-0.404734487	0.685672689	0.999677092
3.2.1.37	xylan 1,4-beta-xylosidase	187.3640125	0.485736855	0.572248284	0.848821864	0.395980422	0.999677092
1.1.1.307	D-xylose reductase	0.179515503	1.072379495	4.117474528	0.260445933	0.794519816	0.999677092
1.1.1.219	dihydroflavonol-4-reductase	15.39927764	0.72433045	0.905087338	0.800287905	0.423544009	0.999677092
3.6.3.35	manganese/zinc transport system ATP-binding protein	2.734835095	-1.499726557	2.078128438	-0.721671736	0.470496323	0.999677092
1.7.2.4	nitrous-oxide reductase	0.408715984	0.213969555	4.10666973	0.052102937	0.958446673	0.999677092
2.1.1.202	multisite-specific tRNA:(cytosine-C5)-methyltransferase	0.206248383	1.09585394	4.120932953	0.265923749	0.790297934	0.999677092
1.7.7.1	ferredoxin-nitrite reductase	24.30469765	-0.005774888	0.569270832	-0.01014436	0.99190611	0.999677092
2.4.1.227	UDP-N-acetylglucosamine--N-acetylmuramyl-(pentapeptide) py	364.4604836	-0.141178681	0.152364729	-0.92658374	0.3541427	0.999677092
2.6.1.100	L-glutamine:2-deoxy-scyllo-inosose/3-amino-2,3-dideoxy-scyllo	0.025751485	0.087866701	4.143731151	0.02120473	0.983082341	0.999677092
2.4.2.21	nicotinate-nucleotide--dimethylbenzimidazole phosphoribosylt	143.4673169	-0.02642693	0.293904837	-0.08991662	0.928353473	0.999677092
4.2.1.147	5,6,7,8-tetrahydromethanopterin hydro-lyase	3.430205491	0.946113908	3.052945237	0.309902024	0.756635464	0.999677092
2.3.1.1	amino-acid N-acetyltransferase	187.3742114	-0.101592062	0.234225442	-0.433736241	0.664479986	0.999677092
2.1.1.223	tRNA1Val (adenine37-N6)-methyltransferase	87.6555404	-0.642134356	0.571730837	-1.123141021	0.261377607	0.999677092
4.1.1.39	#N/A	0.674912036	0.5900884	4.092750263	0.144178941	0.885359169	0.999677092
2.5.1.105	7,8-dihydropterin-6-yl-methyl-4-(beta-D-ribofuranosyl)aminobe	39.00221093	0.387034721	0.446392312	0.867028197	0.38592656	0.999677092
1.17.4.1	ribonucleotide reductase, class II	441.4559732	-0.260987666	0.39866442	-0.654655024	0.512689889	0.999677092
2.3.1.183	phosphinothricin acetyltransferase	219.4034355	-0.611376863	0.652558143	-0.93689255	0.348813832	0.999677092
6.3.2.9	UDP-N-acetylmuramoylalanine--D-glutamate ligase	401.0932987	0.049115648	0.276490321	0.177639665	0.859005972	0.999677092
6.3.2.17	folylpolyglutamate synthase	408.1969088	-0.130742908	0.196628486	-0.664923537	0.5060994	0.999677092

2.7.8.20	phosphoglycerol transferase	36.26566766	-0.623059022	0.455892471	-1.366679781	0.171725698	0.999677092
2.3.3.10	hydroxymethylglutaryl-CoA synthase	5.711122078	-2.972229279	1.518810621	-1.956945281	0.050353902	0.999677092
2.3.3.15	sulfoacetaldehyde acetyltransferase	1.166067547	3.823076346	4.030985397	0.948422276	0.342914523	0.999677092
3.2.1.28	alpha,alpha-trehalase	2.844993824	-1.208256546	1.233575564	-0.979475098	0.327345287	0.999677092
2.7.2.1	acetate kinase	2424.083986	-1.328491269	0.785108378	-1.69211195	0.09062463	0.999677092
3.5.1.44	chemotaxis protein CheD	22.51654174	-0.568338848	0.605275088	-0.938976111	0.34774301	0.999677092
2.1.1.199	16S rRNA (cytosine1402-N4)-methyltransferase	1164.169383	0.126258485	0.390249754	0.323532517	0.746291985	0.999677092
2.7.2.-	#N/A	0.770139323	-0.598245692	4.103132003	-0.145802205	0.884077535	0.999677092
1.13.11.49	chlorite dismutase	0.051862191	0.448527249	4.143731151	0.108242362	0.913803442	0.999677092
3.6.4.3	#N/A	2.836180328	0.942595933	3.072868718	0.30674787	0.75903529	0.999677092
2.6.1.98	UDP-2-acetamido-2-deoxy-ribo-hexuluronate aminotransferase	0.251634167	-0.385219611	4.135429191	-0.09315106	0.925783554	0.999677092
1.1.1.4	(R,R)-butanediol dehydrogenase / meso-butanediol dehydroger	2.80919775	-0.373009592	1.590975653	-0.234453363	0.814633035	0.999677092
1.8.1.4	dihydrolipoamide dehydrogenase	317.743301	-0.211445972	0.439539425	-0.481062584	0.630472019	0.999677092
3.1.13.5	ribonuclease D	6.041358097	-0.929097193	1.024087385	-0.907244056	0.364277751	0.999677092
1.1.1.305	UDP-4-amino-4-deoxy-L-arabinose formyltransferase / UDP-glu	1.285501156	-2.177651273	2.073352696	-1.05030431	0.293578224	0.999677092
5.4.99.19	16S rRNA pseudouridine516 synthase	318.1196796	-0.444135931	0.498474838	-0.89098967	0.372934712	0.999677092
2.7.1.68	1-phosphatidylinositol-4-phosphate 5-kinase	1.131986754	0.088295069	4.098085842	0.021545441	0.982810555	0.999677092
1.1.1.60	2-hydroxy-3-oxopropionate reductase	9.559379802	-0.293274231	0.733851462	-0.399637047	0.689423866	0.999677092
2.7.1.198	PTS system, glucitol/sorbitol-specific IIA component	10.56111943	-0.924450615	0.728871186	-1.268331953	0.204679431	0.999677092
3.4.21.116	stage IV sporulation protein B	186.7204116	0.528758911	0.659390143	0.801890833	0.422616109	0.999677092
1.6.2.2	cytochrome-b5 reductase	0.078987483	0.401836267	4.14177237	0.097020365	0.922710222	0.999677092
1.2.1.89	D-glyceraldehyde dehydrogenase (NADP+)	0.103005941	-0.436320317	4.137063428	-0.10546619	0.916005897	0.999677092
1.1.1.136	UDP-N-acetyl-D-glucosamine dehydrogenase	13.44204196	0.263430319	0.665522918	0.395824565	0.692234454	0.999677092
2.5.1.128	N4-bis(aminopropyl)spermidine synthase	4.506886474	0.913603563	3.238518358	0.282105414	0.777862682	0.999677092
2.8.4.3	tRNA-2-methylthio-N6-dimethylallyladenosine synthase	406.9485174	0.332743692	0.182270773	1.825546057	0.067918668	0.999677092
3.2.1.40	alpha-L-rhamnosidase	377.6323579	0.164907533	0.480350034	0.343307007	0.73136751	0.999677092
1.1.1.381	3-hydroxy acid dehydrogenase / malonic semialdehyde reducta	0.577711424	-1.794321571	4.109137123	-0.436666268	0.662353396	0.999677092
3.5.2.7	imidazolonepropionase	168.0587454	0.312562185	0.418813979	0.746303134	0.455484319	0.999677092
5.1.99.4	alpha-methylacyl-CoA racemase	1.348104808	-2.212049158	3.585705348	-0.616907678	0.537295622	0.999677092
3.5.3.7	guanidinobutyrase	0.574681685	2.083933171	4.111231316	0.506887842	0.612233517	0.999677092
6.3.4.20	7-cyano-7-deazaguanine synthase	94.67236829	-0.373026838	0.549352944	-0.679029469	0.49711919	0.999677092
6.3.2.14	bifunctional isochorismate lyase / aryl carrier protein	4.385493731	-3.378797069	2.219601223	-1.522254103	0.127945423	0.999677092
1.4.3.19	glycine oxidase	0.103005941	-0.436320317	4.137063428	-0.10546619	0.916005897	0.999677092
3.1.3.8	3-phytase	0.154508912	-0.660754812	4.13282253	-0.159879793	0.872975766	0.999677092
3.1.11.2	exodeoxyribonuclease III	258.3334323	-0.351215482	0.30216667	-1.162323701	0.245104003	0.999677092
2.1.1.33	tRNA (guanine-N7-)-methyltransferase	245.3849363	0.315595944	0.299403884	1.054080995	0.291845837	0.999677092
1.12.5.1	#N/A	0.276334958	-0.696876501	4.119342135	-0.169171795	0.865661515	0.999677092
3.5.1.53	N-carbamoylputrescine amidase	105.5660389	-0.278562462	0.37811967	-0.7367045	0.46130207	0.999677092
1.9.3.1	#N/A	0.209529612	0.448514122	4.143731151	0.108239195	0.913805955	0.999677092
3.2.1.24	alpha-mannosidase	269.881228	-0.199293769	0.445309581	-0.447539819	0.654485345	0.999677092
3.6.3.3	#N/A	575.5346922	0.086770587	0.277384335	0.312817186	0.754419557	0.999677092
4.2.1.40	glucarate dehydratase	7.189205811	-1.568288832	1.075328707	-1.458427384	0.144722777	0.999677092
2.1.1.100	protein-S-isoprenylcysteine O-methyltransferase	0.166515913	-0.468055926	4.138090197	-0.113109165	0.909944009	0.999677092
2.4.2.17	ATP phosphoribosyltransferase	157.6760459	-0.434979924	0.188507056	-2.307499423	0.021026994	0.999677092
1.2.7.3	#N/A	1254.655337	0.221946917	0.302495018	0.733720899	0.463118858	0.999677092
2.7.8.38	archaetidylserine synthase	0.293518541	0.59224539	4.111781154	0.144036214	0.885471873	0.999677092
2.4.2.1	#N/A	957.9806977	0.003161644	0.603197196	0.005241477	0.995817926	0.999677092
2.7.1.73	inosine kinase	0.730107326	-2.580259306	4.105034615	-0.628559695	0.529637354	0.999677092
4.1.1.25	tyrosine decarboxylase	0.874214996	-0.504261795	4.102311327	-0.122921386	0.90216935	0.999677092
3.2.1.54	cyclomaltodextrinase / maltogenic alpha-amylase / neopullulan	0.30127684	-1.332711405	4.125972188	-0.323005426	0.746691133	0.999677092
2.3.1.242	KDO2-lipid IV(A) palmitoleoyltransferase	3.363987485	-2.353586053	2.087398435	-1.127521231	0.259522169	0.999677092
6.3.1.2	glutamine synthetase	935.7833556	-0.225119732	0.219565078	-1.025298437	0.305222393	0.999677092
3.6.3.30	iron(III) transport system ATP-binding protein	97.39821977	-0.309977084	0.395931193	-0.782906449	0.43368205	0.999677092
6.3.2.3	glutathione synthase	1.801797141	-2.730608945	1.92278752	-1.420130366	0.155569731	0.999677092
6.3.2.39	aerobactin synthase	2.405771588	-0.111218006	4.096375455	-0.027150345	0.97833982	0.999677092
3.5.1.82	N-acyl-D-glutamate deacylase	0.439714584	1.627155285	4.115670892	0.395356025	0.692580158	0.999677092
5.1.1.4	proline racemase	6.577327919	0.271213868	1.02955312	0.263428727	0.792220151	0.999677092
2.7.1.60	N-acylmannosamine kinase	0.061485587	-0.248235939	4.139390501	-0.059969201	0.952180165	0.999677092
1.8.5.4	sulfide:quinone oxidoreductase	3.822115518	-0.430353642	1.994098887	-0.215813591	0.829133054	0.999677092
2.1.1.224	23S rRNA (adenine-C8)-methyltransferase	4.139167645	-0.116816194	3.305773184	-0.035337026	0.971810999	0.999677092
6.2.1.30	phenylacetate-CoA ligase	732.7612545	0.758379952	0.589577068	1.286311821	0.198334259	0.999677092
1.1.1.163	cyclopentanol dehydrogenase	0.213055426	2.039247922	4.123748771	0.494513132	0.620943834	0.999677092
3.4.21.88	repressor LexA	498.3446664	0.225334199	0.371767499	0.60611592	0.54443778	0.999677092
2.7.1.89	thiamine kinase	0.567411961	-1.443822676	4.113954887	-0.350957343	0.725620351	0.999677092
2.4.1.64	alpha,alpha-trehalose phosphorylase	4.662255611	-1.6098457	1.512391869	-1.064436892	0.287130829	0.999677092
3.6.1.23	dUTP pyrophosphatase	266.0926212	0.077664839	0.27399378	0.283454753	0.776828262	0.999677092
1.1.1.26	glyoxylate reductase	0.566106049	-0.425800683	4.106517621	-0.103688994	0.917416161	0.999677092
3.5.1.19	nicotinamidase	5.650251342	0.675476056	1.244214301	0.54289366	0.587203021	0.999677092
2.4.1.12	cellulose synthase (UDP-forming)	8.384802002	1.681434508	1.001401962	1.679080501	0.093136356	0.999677092
3.1.21.3	type I restriction enzyme, R subunit	1199.129253	0.030980911	0.252977801	0.12246494	0.902530811	0.999677092
2.1.1.189	23S rRNA (uracil747-C5)-methyltransferase	0.50752299	-1.670391333	4.1193398	-0.405499768	0.685110189	0.999677092
4.2.1.68	L-fuconate dehydratase	0.087521458	-0.402401827	4.138412209	-0.097235801	0.922539137	0.999677092
1.10.9.1	#N/A	0.084668419	1.508835902	4.130322632	0.365307032	0.71488223	0.999677092
4.6.1.12	2-C-methyl-D-erythritol 2,4-cyclodiphosphate synthase	226.1923799	-0.232294624	0.215701007	-1.076928788	0.281512081	0.999677092
1.3.8.4	isovaleryl-CoA dehydrogenase	0.545047208	-1.892569288	4.113322666	-0.460107179	0.645439291	0.999677092
6.3.5.3	phosphoribosylformylglycinamidine synthase	1099.359206	0.092095222	0.221225132	0.416296383	0.677193139	0.999677092
3.2.1.45	glucosylceramidase	63.37924565	-0.237780036	0.781514577	-0.304255408	0.760933315	0.999677092
2.1.1.163	demethylmenaquinone methyltransferase / 2-methoxy-6-polyp	61.69477923	-0.2233348	0.466166708	-0.479087838	0.631876141	0.999677092
3.4.23.-	#N/A	121.6829844	-0.277383804	0.545260351	-0.50871809	0.610949841	0.999677092
1.1.1.339	dTDP-6-deoxy-L-talose 4-dehydrogenase (NAD+)	18.79373412	-0.125702653	0.505046351	-0.248893299	0.803443318	0.999677092
6.5.1.-	#N/A	117.6895471	1.052742393	0.780549263	1.348719988	0.177426923	0.999677092
6.3.1.11	gamma-glutamylputrescine synthase	0.102475978	-0.460139135	4.137494487	-0.111212024	0.911448217	0.999677092
1.5.5.-	#N/A	3.832395075	-0.628868577	1.666729583	-0.377306903	0.705945543	0.999677092
1.1.1.298	malonyl-CoA reductase / 3-hydroxypropionate dehydrogenase	0.201546003	-1.141459061	4.130495086	-0.276349212	0.782279862	0.999677092
2.7.1.72	streptomycin 6-kinase	0.20799384	1.013521217	4.134081795	0.245162352	0.806330725	0.999677092
5.4.99.28	tRNA pseudouridine32 synthase / 23S rRNA pseudouridine746 s	66.23259907	-0.206478592	0.574821053	-0.359204992	0.719441743	0.999677092
2.7.1.156	adenosylcobinamide kinase / adenosylcobinamide-phosphate g	139.991378	-0.073036857	0.41060099	-0.177877936	0.858818839	0.999677092

2.3.1.18	galactoside O-acetyltransferase	43.53976159	0.84397812	0.783998167	1.076505222	0.281701367	0.999677092
4.99.1.4	uroporphyrin-III C-methyltransferase / precorrin-2 dehydrogena	30.31722373	0.024199596	0.835822525	0.028953032	0.97690205	0.999677092
3.2.1.97	endo-alpha-N-acetylgalactosaminidase	605.1706156	-0.599578405	0.749604735	-0.799859416	0.423792254	0.999677092
4.1.1.87	malonate decarboxylase beta subunit	7.252252901	-0.281758147	1.111774037	-0.253431127	0.799935077	0.999677092
3.1.3.95	myotubularin	3.613934552	0.021085684	2.802326683	0.007524349	0.993996495	0.999677092
3.6.1.5	apyrase	0.249773869	-0.680453166	4.123822835	-0.165005431	0.868939707	0.999677092
1.2.99.7	aldehyde oxidoreductase	192.6359077	-0.36075102	0.562383453	-0.641468055	0.52121863	0.999677092
2.4.2.53	undecaprenyl-phosphate 4-deoxy-4-formamido-L-arabinose tra	25.70573289	-0.374796011	0.542288504	-0.691137667	0.489479031	0.999677092
1.3.7.11	digeranylgeranylglycerophospholipid reductase	2.870100187	0.632201588	3.908480759	0.161751235	0.871501756	0.999677092
2.7.1.169	pantoate kinase	0.104075673	0.43806451	4.143245313	0.105729803	0.915796734	0.999677092
3.6.1.40	exopolyphosphatase / guanosine-5'-triphosphate,3'-diphosphat	148.2287987	-0.279660763	0.37191423	-0.751949617	0.452081358	0.999677092
6.1.1.12	aspartyl-tRNA synthetase	692.5248136	0.243380306	0.299738104	0.811976531	0.416805098	0.999677092
3.5.1.3	omega-amidase	2.642522728	-0.273740429	3.821185707	-0.071637562	0.942890347	0.999677092
1.5.98.2	5,10-methylenetetrahydromethanopterin reductase	40.82137094	2.312522247	1.331987473	1.73614414	0.082538351	0.999677092
2.1.1.264	23S rRNA (guanine2445-N2)-methyltransferase / 23S rRNA (gua	1.313961173	-2.947557179	2.492975776	-1.182344894	0.237068879	0.999677092
1.1.1.282	quinate/shikimate dehydrogenase	0.322402874	-0.175669432	4.111922489	-0.042721971	0.965923166	0.999677092
2.4.2.10	orotate phosphoribosyltransferase	556.3995842	-0.28695171	0.278660714	-1.029753013	0.303125963	0.999677092
3.1.7.2	GTP diphosphokinase / guanosine-3',5'-bis(diphosphate) 3'-diph	5.410257227	0.402782991	1.50274421	0.268031637	0.788674966	0.999677092
3.5.4.10	phosphoribosylaminoimidazolecarboxamide formyltransferase	546.3313652	0.038018604	0.183640104	0.207027788	0.835988157	0.999677092
2.4.1.319	beta-1,4-mannooligosaccharide/beta-1,4-mannosyl-N-acetylglu	163.8803014	0.123291893	0.350898711	0.351360349	0.725318026	0.999677092
3.6.3.38	capsular polysaccharide transport system ATP-binding protein	0.528533578	0.395440403	4.090549498	0.096671707	0.922987109	0.999677092
2.3.1.169	#N/A	421.7701187	-0.119616511	0.562762327	-0.212552449	0.831676064	0.999677092
5.4.2.2	phosphoglucomutase	10668.07044	-0.284484548	0.73721156	-0.385892684	0.699576149	0.999677092
5.2.1.1	maleate isomerase	0.8515885	-0.77139525	4.099164452	-0.188183533	0.85073278	0.999677092
2.7.7.62	adenosylcobinamide kinase / adenosylcobinamide-phosphate g	139.991378	-0.073036857	0.41060099	-0.177877936	0.858818839	0.999677092
1.1.1.320	benzil reductase ((S)-benzoin forming)	0.13068425	1.791016996	4.127020683	0.433973351	0.664307793	0.999677092
2.1.1.265	tellurite methyltransferase	1.655407417	-1.87551076	2.012107858	-0.932112437	0.351278421	0.999677092
3.5.1.25	N-acetylglucosamine-6-phosphate deacetylase	970.9548437	-0.425790059	0.479610957	-0.887782176	0.374657933	0.999677092
3.4.24.55	protease III	0.658608599	0.253393841	4.092608421	0.061914998	0.950630524	0.999677092
2.1.1.219	tRNA (adenine57-N1/adenine58-N1)-methyltransferase catalyt	3.95831283	-0.195393474	1.71238583	-0.114105986	0.909153777	0.999677092
4.1.3.4	hydroxymethylglutaryl-CoA lyase	4.714175398	-1.094657121	1.322760434	-0.827555083	0.407922515	0.999677092
1.1.5.4	malate dehydrogenase (quinone)	1.565747164	-1.114289676	2.567004368	-0.434081722	0.664229098	0.999677092
1.7.2.1	nitrite reductase (NO-forming)	0.040667486	0.087870004	4.143731151	0.021205527	0.983081705	0.999677092
4.1.1.47	tartronate-semialdehyde synthase	1.982359021	0.400681845	2.21712595	0.180721282	0.856586353	0.999677092
2.1.2.10	aminomethyltransferase	191.58773	0.027487614	0.343259042	0.080078341	0.936174949	0.999677092
2.7.8.1	ethanolaminephosphotransferase	1.503869701	-0.53731895	4.098356522	-0.131105956	0.89569149	0.999677092
2.7.1.40	pyruvate kinase	528.5119295	0.013519403	0.188347503	0.071779038	0.942777755	0.999677092
2.7.1.11	6-phosphofructokinase 1	1517.868815	0.110452594	0.304191181	0.363102551	0.716528279	0.999677092
3.4.23.1	pepsin A	0.078987483	0.401836267	4.14177237	0.097020365	0.922710222	0.999677092
6.1.1.21	#N/A	372.2009374	0.054799499	0.180076643	0.304312086	0.760890138	0.999677092
1.97.1.4	#N/A	2043.548934	-0.349043366	0.516534451	-0.675740728	0.499205272	0.999677092
1.1.1.11	D-arabinitol 4-dehydrogenase	0.221067967	-0.658961953	4.125613164	-0.15972461	0.873098014	0.999677092
6.3.2.13	UDP-N-acetylmuramoyl-L-alanyl-D-glutamate--2,6-diaminopime	496.0040111	0.13232666	0.183392064	0.721550635	0.470570799	0.999677092
3.2.2.24	ADP-ribosyl-[dinitrogen reductase] hydrolase	2.896195258	-1.172680304	3.131245066	-0.37450927	0.708025461	0.999677092
5.-.-.-	#N/A	559.3196393	-0.455480852	0.739377221	-0.616033114	0.537872665	0.999677092
3.2.2.28	double-stranded uracil-DNA glycosylase	1.308970377	-1.350179922	4.084972029	-0.330523664	0.741004315	0.999677092
2.7.1.4	fructokinase	457.5016156	-0.050206202	0.287209739	-0.174806755	0.861231481	0.999677092
2.7.1.14	sedoheptulokinase	8.938681165	-1.507467767	1.324319543	-1.138296097	0.254996863	0.999677092
1.11.1.-	#N/A	3.659730758	-1.79751141	2.206270897	-0.814728333	0.415227832	0.999677092
4.1.1.11	aspartate 1-decarboxylase	122.8047799	-0.194959026	0.401600016	-0.485455722	0.627353114	0.999677092
3.4.21.81	streptogrisin B	8.877711127	-1.125777035	2.289733686	-0.49166287	0.622957691	0.999677092
2.7.1.165	glycerate 2-kinase	141.9706858	-0.613636338	0.421695376	-1.455164966	0.145623597	0.999677092
4.4.1.1	cystathionine gamma-lyase	3.136372628	2.092553874	2.396939051	0.873010882	0.382657147	0.999677092
1.2.1.87	propionaldehyde dehydrogenase	223.0956313	-0.983864512	0.64763258	-1.519170811	0.128719507	0.999677092
2.8.3.10	#N/A	92.4380191	0.53191065	0.465243268	1.143295749	0.25291582	0.999677092
4.4.1.14	1-aminocyclopropane-1-carboxylate synthase	3.109366266	-0.031980842	2.99757882	-0.010668891	0.991487618	0.999677092
1.3.1.2	dihydropyrimidine dehydrogenase (NADP+)	0.077254456	-0.295989182	4.139694903	-0.071500241	0.942999633	0.999677092
3.10.1.1	N-sulfoglucosamine sulfohydrolase	0.500048027	-1.162291506	4.117739317	-0.28226447	0.777740727	0.999677092
2.7.1.51	L-fuculokinase	17.52662205	0.269000858	0.990028099	0.271710326	0.785844761	0.999677092
4.1.3.44	tRNA wybutosine-synthesizing protein 1	3.850197146	-0.457114555	2.90706168	-0.157242813	0.875053488	0.999677092
1.1.1.30	3-hydroxybutyrate dehydrogenase	0.712468766	-2.93762797	3.837729809	-0.76545982	0.443997783	0.999677092
5.4.99.20	23S rRNA pseudouridine2457 synthase	2.784565633	-2.236850015	1.919712388	-1.165200594	0.243937805	0.999677092
2.4.99.16	starch synthase (maltosyl-transferring)	1.915180048	-0.572809552	3.60218212	-0.159017377	0.873655183	0.999677092
2.7.4.28	[pyruvate, water dikinase]-phosphate phosphotransferase / [py	9.875085199	-0.230823304	0.667810463	-0.345641941	0.729611822	0.999677092
3.4.23.51	hydrogenase 3 maturation protease	1.085645249	-2.639432685	3.542393962	-0.745098573	0.456212132	0.999677092
2.7.1.208	PTS system, alpha-glucoside-specific IIB component	59.53385477	-0.280052055	0.61820656	-0.453007253	0.650543519	0.999677092
3.2.2.3	uridine nucleosidase	0.052512875	-0.212523182	4.139416356	-0.05134134	0.959053527	0.999677092
1.6.2.4	NADPH-ferrihemoprotein reductase	1.311335403	-0.277609262	4.100464027	-0.067701914	0.946022926	0.999677092
6.6.1.1	magnesium chelatase subunit H	18.6825575	-0.434570369	0.702801104	-0.618340475	0.536350926	0.999677092
1.1.1.102	3-dehydrosphinganine reductase	1.670128019	0.628264475	4.098321767	0.153297987	0.878163288	0.999677092
3.6.3.8	Ca2+-transporting ATPase	569.4036309	0.059393263	0.332456753	0.178649592	0.858212852	0.999677092
1.1.1.36	acetoacetyl-CoA reductase	0.578478959	-1.487265979	4.079656594	-0.364556659	0.715442372	0.999677092
3.1.3.11	fructose-1,6-bisphosphatase I / sedoheptulose-1,7-bisphosphat	458.6616046	-0.167895871	0.233473249	-0.719122519	0.472065434	0.999677092
6.1.1.2	tryptophanyl-tRNA synthetase	420.3540249	-0.123066599	0.291584511	-0.422061508	0.672980123	0.999677092
1.1.1.79	glyoxylate/hydroxypyruvate reductase	10.11112133	-1.171900326	1.899259156	-0.617030236	0.537214783	0.999677092
6.3.2.34	coenzyme F420-0:L-glutamate ligase / coenzyme F420-1:gamm	1.257810662	-0.846182438	4.084521864	-0.207168052	0.835878617	0.999677092
1.1.1.374	UDP-N-acetylglucosamine 3-dehydrogenase	1.956313881	1.40309498	4.086328011	0.343363278	0.731325182	0.999677092
2.4.1.21	starch synthase	1092.909783	0.039078747	0.371390819	0.105222706	0.916199094	0.999677092
2.3.2.3	phosphatidylglycerol lysyltransferase	2.629663203	-0.887773831	1.716377761	-0.517236852	0.604990829	0.999677092
3.2.1.58	glucan 1,3-beta-glucosidase	1.646498268	-0.250806296	2.287100823	-0.109661233	0.912678047	0.999677092
1.1.1.81	glyoxylate/hydroxypyruvate reductase	52.56367318	0.230467604	0.430267472	0.53563799	0.592208767	0.999677092
2.7.11.25	mitogen-activated protein kinase kinase kinase 8	0.495257723	0.396549258	4.10450062	0.096613278	0.923033512	0.999677092
1.8.99.5	dissimilatory sulfite reductase alpha subunit	53.43867049	0.392472972	0.632397093	0.620611602	0.534855204	0.999677092
2.1.1.63	methylated-DNA-protein]-cysteine S-methyltransferase	165.7113079	0.052126145	0.340410242	0.153127429	0.878297786	0.999677092
4.2.99.21	salicylate synthetase	0.575016627	-1.416403502	4.120846178	-0.343716664	0.731059378	0.999677092
2.3.1.57	diamine N-acetyltransferase	81.34146399	-0.200643431	0.511270803	-0.392440619	0.694732678	0.999677092
2.4.1.288	galactofuranosylgalactofuranosylrhamnosyl-N-acetylglucosamir	0.870667307	-0.734159188	4.086540138	-0.179652998	0.857424995	0.999677092

2.4.2.14	amidophosphoribosyltransferase	632.8867892	-0.10235966	0.159790734	-0.640585702	0.521791891	0.999677092
2.7.7.39	glycerol-3-phosphate cytidylyltransferase	10.10101612	-0.607628802	1.100307066	-0.552235663	0.580786903	0.999677092
4.2.2.10	pectin lyase	10.9423628	1.470985713	1.157239856	1.271115668	0.203687502	0.999677092
1.3.5.1	#N/A	476.3325581	-0.514199142	0.335333657	-1.533395563	0.125178421	0.999677092
4.2.1.6	galactonate dehydratase	37.59083693	0.369482103	0.451256563	0.818784994	0.412909094	0.999677092
4.3.2.1	argininosuccinate lyase	276.1844737	-0.045564018	0.194840876	-0.233852459	0.815099522	0.999677092
6.1.2.1	D-alanine---(R)-lactate ligase	0.017504292	0.120319428	4.141980666	0.029048766	0.976825697	0.999677092
2.2.1.6	#N/A	949.9615364	-0.177660465	0.208663305	-0.851421699	0.394535142	0.999677092
2.3.1.102	acetyl CoA:N6-hydroxylysine acetyl transferase	1.45851818	0.02136391	4.099676987	0.00521112	0.995842146	0.999677092
2.6.1.17	acetylornithine/N-succinyldiaminopimelate aminotransferase	710.8284773	-0.034236364	0.46655183	-0.073381694	0.941502384	0.999677092
2.1.1.321	type III protein arginine methyltransferase	0.500958201	-0.054243404	4.10524762	-0.013213187	0.989457709	0.999677092
3.4.24.15	thimet oligopeptidase	1.404001712	0.492081423	3.674940217	0.133901885	0.893480159	0.999677092
3.1.1.31	6-phosphogluconolactonase	371.1096164	0.093621494	0.548102042	0.170810336	0.864372901	0.999677092
3.6.1.31	phosphoribosyl-ATP pyrophosphohydrolase	275.8587485	-0.026130014	0.220511411	-0.118497331	0.905673609	0.999677092
1.20.4.1	arsenate reductase (glutaredoxin)	92.4590067	-0.422573163	0.455683219	-0.927339751	0.353750161	0.999677092
2.7.7.60	2-C-methyl-D-erythritol 4-phosphate cytidylyltransferase	341.3139113	-0.011133056	0.229746347	-0.048458035	0.961351209	0.999677092
2.7.7.3	pantetheine-phosphate adenylyltransferase	410.527713	-0.621777643	0.354642446	-1.753252182	0.07955873	0.999677092
2.1.3.6	putrescine carbamoyltransferase	5.844709524	0.37463673	1.243004721	0.301396063	0.763112496	0.999677092
2.4.1.265	alpha-1,3-glucosyltransferase	0.122530041	-0.543453665	4.137516692	-0.131347788	0.89550019	0.999677092
3.5.4.-	#N/A	0.035008583	-0.076612487	4.14032189	-0.018503993	0.985236792	0.999677092
3.6.1.1	inorganic pyrophosphatase	703.8118417	-0.040883519	0.210609749	-0.194119784	0.846082091	0.999677092
2.1.1.107	uroporphyrin-III C-methyltransferase	93.24422569	-0.204532995	0.348355163	-0.587139269	0.557110176	0.999677092
1.1.1.337	L-2-hydroxycarboxylate dehydrogenase (NAD+)	2.293813537	-0.095220776	3.262381481	-0.029187505	0.976715046	0.999677092
3.2.1.143	poly(ADP-ribose) glycohydrolase	0.208151345	0.386925252	4.141249987	0.093431996	0.925560373	0.999677092
1.1.1.42	isocitrate dehydrogenase	498.3051866	-0.130998033	0.396463771	-0.330416152	0.741085539	0.999677092
4.2.1.106	bile-acid 7alpha-dehydratase	5.369375598	-0.011803723	1.143626536	-0.010321309	0.991764933	0.999677092
2.7.7.18	nicotinate-nucleotide adenylyltransferase	289.546131	-0.042040365	0.229625946	-0.183081947	0.854733721	0.999677092
2.8.3.9	acetate CoA/acetoacetate CoA-transferase alpha subunit	181.9968591	0.049320233	0.540973875	0.091169343	0.927358034	0.999677092
2.3.1.225	palmitoyltransferase ZDHHC9/14/18	2.026598504	-0.462460278	4.097073251	-0.112875765	0.91012905	0.999677092
4.1.1.1	pyruvate decarboxylase	0.143466369	-0.612378542	4.136114884	-0.148056464	0.882298208	0.999677092
3.6.4.7	peroxisome-assembly ATPase	9.240900872	-0.268946977	4.093666523	-0.065698311	0.947618017	0.999677092
1.14.-.-	#N/A	8.314588655	0.251122354	1.018977212	0.246445506	0.805337384	0.999677092
2.7.1.35	pyridoxine kinase	248.946753	0.277129902	0.262573055	1.055439227	0.29122449	0.999677092
1.1.1.289	sorbose reductase	0.110533983	-0.307374933	4.142162324	-0.074206395	0.940846158	0.999677092
3.2.1.135	cyclomaltodextrinase / maltogenic alpha-amylase / neopullulan	0.30127684	-1.332711405	4.125972188	-0.323005426	0.746691133	0.999677092
1.1.1.370	scyllo-inositol 2-dehydrogenase (NAD+)	0.128757426	-0.558067872	4.134677189	-0.134972538	0.892633587	0.999677092
3.2.1.1	alpha-amylase	2005.81684	-1.17795114	0.993877165	-1.185207973	0.235935228	0.999677092
5.1.3.20	ADP-L-glycero-D-manno-heptose 6-epimerase	19.91059957	0.535369524	0.657883664	0.813775373	0.415773646	0.999677092
2.-.-.-	#N/A	276.2509304	0.221070309	0.278211135	0.794613449	0.426838393	0.999677092
4.2.1.115	UDP-N-acetylglucosamine 4,6-dehydratase	0.138985024	1.458218115	4.128479	0.353209527	0.723931366	0.999677092
3.2.1.96	mannosyl-glycoprotein endo-beta-N-acetylglucosaminidase	18.59201307	-0.812322384	0.956089666	-0.849629917	0.395530874	0.999677092
2.7.1.200	PTS system, galactitol-specific IIA component	13.58689989	-0.532580149	0.915361505	-0.581824936	0.560684606	0.999677092
3.6.1.-	#N/A	368.9897887	-0.041164831	0.225859444	-0.18225862	0.855379771	0.999677092
2.7.1.90	diphosphate-dependent phosphofructokinase	109.7540539	-0.948167644	0.458996193	-2.065741849	0.038852861	0.999677092
5.3.1.27	6-phospho-3-hexuloisomerase	13.80075987	-0.839723931	0.807455506	-1.039963099	0.298357045	0.999677092
3.2.1.82	exo-poly-alpha-galacturonosidase	1.206697936	-1.226143165	2.735473003	-0.44823808	0.653981383	0.999677092
5.3.1.4	L-arabinose isomerase	262.4860847	0.071695457	0.28929275	0.247830119	0.80426585	0.999677092
2.3.1.40	acyl-[acyl-carrier-protein]-phospholipid O-acyltransferase / long	5.957426123	-1.941111817	1.544530418	-1.25676503	0.208838732	0.999677092
2.4.1.282	3-O-alpha-D-glucosyl-L-rhamnose phosphorylase	0.087521458	-0.402401827	4.138412209	-0.097235801	0.922539137	0.999677092
2.1.1.113	site-specific DNA-methyltransferase (cytosine-N4-specific)	0.549311404	0.349377191	4.104845309	0.085113364	0.932171266	0.999677092
2.7.7.9	UTP--glucose-1-phosphate uridylyltransferase	171.0660117	0.286173613	0.322417491	0.887587123	0.374762883	0.999677092
1.13.11.1	catechol 1,2-dioxygenase	0.055266992	0.087870004	4.143731151	0.021205527	0.983081705	0.999677092
3.1.2.21	medium-chain acyl-[acyl-carrier-protein] hydrolase	2.090803846	0.248854894	2.021248439	0.1231194	0.902012549	0.999677092
0.0.0.0	#N/A	4559514.544	0.224400724	0.629192235	0.356648909	0.721354658	0.999677092
2.7.7.89	[glutamine synthetase] adenylyltransferase / [glutamine synthe	10.5009941	1.114375934	0.970245138	1.148550907	0.25074121	0.999677092
4.2.1.129	squalene-hopene/tetraprenyl-beta-curcumene cyclase	7.194674308	0.00789086	1.41156842	0.005590137	0.99553974	0.999677092
4.1.1.18	lysine decarboxylase	3.388512186	-0.773016609	1.526292696	-0.50646682	0.612528977	0.999677092
5.1.3.2	UDP-glucose 4-epimerase	1242.539518	0.415543514	0.435535967	0.954096895	0.340034598	0.999677092
5.4.99.61	precorrin-8X/cobalt-precorrin-8 methylmutase	102.094744	-0.078547974	0.50764316	-0.154730686	0.877033637	0.999677092
3.1.26.5	ribonuclease P/MRP protein subunit POP1	316.2440482	-0.011638501	0.289480453	-0.040204792	0.967929858	0.999677092
6.3.2.45	UDP-N-acetylmuramate: L-alanyl-gamma-D-glutamyl-meso-diar	13.36009396	-0.312259614	0.89241488	-0.349904087	0.72641068	0.999677092
2.1.1.171	16S rRNA (guanine966-N2)-methyltransferase	10.12371931	0.016441179	0.841553483	0.019536701	0.98441296	0.999677092
2.5.1.7	UDP-N-acetylglucosamine 1-carboxyvinyltransferase	705.9287071	0.137850114	0.169835444	0.811668704	0.416981757	0.999677092
2.3.1.31	homoserine O-acetyltransferase/O-succinyltransferase	18.80023827	0.496619523	0.899515459	0.552096707	0.580882098	0.999677092
2.7.1.181	O-antigen chain-terminating methyltransferase	2.5617666	0.849576774	3.783133496	0.2245696	0.822314117	0.999677092
4.1.1.98	4-hydroxy-3-polyprenylbenzoate decarboxylase	25.44402293	-0.989257465	0.668637554	-1.47951227	0.139003454	0.999677092
2.1.1.144	trans-aconitate 2-methyltransferase	2.013320454	0.756825477	1.328018553	0.569890741	0.568751805	0.999677092
3.5.5.7	aliphatic nitrilase	0.040667486	0.087870004	4.143731151	0.021205527	0.983081705	0.999677092
1.1.1.261	glycerol-1-phosphate dehydrogenase [NAD(P)+]	72.65407799	-0.251715967	0.352117595	-0.71486336	0.47469348	0.999677092
3.2.2.30	adenosylhomocysteine/aminodeoxyfutalosine nucleosidase	0.689389753	4.083601769	4.07606669	1.001848615	0.316416713	0.999677092
3.4.21.112	membrane-bound transcription factor site-1 protease	0.333031825	-0.735914733	4.11703374	-0.178748774	0.85813497	0.999677092
1.21.98.1	cyclic dehypoxanthinyl futalosine synthase	7.265508363	0.311123678	1.250753507	0.248748995	0.803554947	0.999677092
1.6.5.3	#N/A	1547.45876	-0.013260819	0.224937114	-0.058953452	0.952989183	0.999677092
1.3.1.78	arogenate dehydrogenase (NADP+)	0.051502971	-0.135983389	4.141439557	-0.032834812	0.973806317	0.999677092
6.3.5.7	aspartyl-tRNA(Asn)/glutamyl-tRNA(Gln) amidotransferase subu	503.111813	0.029849352	0.458203202	0.065144356	0.948059065	0.999677092
2.6.1.22	4-aminobutyrate aminotransferase / (S)-3-amino-2-methylprop	25.41220286	-0.017135945	0.665929788	-0.025732361	0.979470812	0.999677092
2.1.1.151	precorrin-2/cobalt-factor-2 C20-methyltransferase	73.91478762	-0.275373779	0.440914952	-0.624550785	0.532265929	0.999677092
6.3.4.23	5-formaminoimidazole-4-carboxamide-1-(beta)-D-ribofuranosy	4.800089674	1.863662201	2.989477444	0.623407347	0.533016872	0.999677092
6.3.4.18	5-(carboxyamino)imidazole ribonucleotide synthase	16.67334866	-0.46530084	0.805213499	-0.577860209	0.563358502	0.999677092
3.2.1.85	6-phospho-beta-galactosidase	5.569374415	-1.45865477	1.618112349	-0.901454569	0.367346679	0.999677092
6.3.1.14	diphthine-ammonia ligase	0.663713212	-0.442876204	4.105440294	-0.107875446	0.914094494	0.999677092
2.1.1.287	ribosomal RNA-processing protein 8	0.26066422	-0.05315672	4.132464608	-0.0128632	0.989736934	0.999677092
2.8.1.13	tRNA-uridine 2-sulfurtransferase	511.8885342	-0.197288973	0.28995302	-0.680417029	0.496240442	0.999677092
4.2.3.3	methylglyoxal synthase	510.2834238	-0.018903598	0.291806504	-0.064781276	0.948348149	0.999677092
4.3.1.17	L-serine dehydratase	492.0753982	0.149696505	0.279882296	0.534855212	0.592749981	0.999677092
6.3.3.2	glutamate formiminotransferase / 5-formyltetrahydrofolate cyc	201.089566	-0.158647709	0.220749449	-0.718677712	0.472339519	0.999677092
5.3.3.8	3-hydroxyacyl-CoA dehydrogenase / enoyl-CoA hydratase / 3-h	0.732925299	-0.395816106	4.111982742	-0.096259185	0.923314726	0.999677092

3.5.3.1	arginase	24.36303767	-0.061819834	0.69004191	-0.089588521	0.928614207	0.999677092
4.1.3.27	anthranilate synthase / indole-3-glycerol phosphate synthase	268.9554381	-0.080127628	0.428130197	-0.187157152	0.851537419	0.999677092
6.1.1.17	glutamyl-tRNA synthetase	495.3645692	0.162276095	0.259903286	0.624371078	0.532383915	0.999677092
3.4.23.43	leader peptidase (prepilin peptidase) / N-methyltransferase	105.4913545	0.275234044	0.664007835	0.414504211	0.678504884	0.999677092
3.5.1.6	beta-ureidopropionase	41.72853179	0.083117985	0.488125003	0.170280122	0.864789843	0.999677092
2.1.1.197	malonyl-CoA O-methyltransferase	55.86749342	-0.987051013	0.919220088	-1.073791822	0.282915996	0.999677092
4.1.99.19	2-iminoacetate synthase	230.7809752	-0.202021867	0.353152842	-0.57205222	0.567286598	0.999677092
3.6.1.27	phosphatidylglycerophosphatase B	463.6147504	-0.13671952	0.344852663	-0.396457776	0.691767351	0.999677092
3.5.3.12	agmatine deiminase	62.47918888	0.004770177	0.470333669	0.010142112	0.991907904	0.999677092
2.8.3.17	cinnamoyl-CoA:phenyllactate CoA-transferase	0.78183725	-0.21644436	4.138937811	-0.052294663	0.958293906	0.999677092
2.1.1.260	rRNA small subunit pseudouridine methyltransferase Nep1	2.083596875	-0.011336834	4.096901517	-0.002767173	0.997792118	0.999677092
1.7.1.15	nitrite reductase (NADH) large subunit	3.939076588	-1.57920884	1.624582609	-0.972070507	0.331015473	0.999677092
4.1.3.17	4-hydroxy-4-methyl-2-oxoglutarate aldolase	0.138732965	-0.096080989	4.140702604	-0.02320403	0.981487524	0.999677092
2.7.7.67	CDP-2,3-bis-(O-geranylgeranyl)-sn-glycerol synthase	0.353849542	-0.434874887	4.115972198	-0.105655448	0.91585573	0.999677092
2.5.1.1	farnesyl diphosphate synthase	376.183266	0.037313067	0.196801999	0.18959699	0.849624946	0.999677092
1.14.13.127	3-(3-hydroxy-phenyl)propionate hydroxylase	0.111464417	0.448520484	4.143731151	0.10824073	0.913804737	0.999677092
5.3.1.23	methylthioribose-1-phosphate isomerase	68.25774551	-0.099674972	0.368535196	-0.270462559	0.786804417	0.999677092
1.2.1.41	glutamate-5-semialdehyde dehydrogenase	370.2096274	-0.146264967	0.196185348	-0.745544807	0.455942434	0.999677092
4.2.1.44	inosose dehydratase	44.65464953	-0.567660231	0.543631362	-1.044200669	0.296392558	0.999677092
3.5.4.13	dCTP deaminase	6.87186702	-0.859402156	0.742408012	-1.157587394	0.247032456	0.999677092
2.1.1.228	tRNA (guanine37-N1)-methyltransferase	241.8350834	0.079778496	0.241401277	0.330480836	0.74103667	0.999677092
1.1.1.17	mannitol-1-phosphate 5-dehydrogenase	25.46882439	-0.04773794	0.578822387	-0.082474245	0.934269598	0.999677092
1.2.1.16	succinate-semialdehyde dehydrogenase / glutarate-semialdehy	8.506514607	-0.697295433	0.995233336	-0.700635126	0.483530752	0.999677092
3.1.4.46	glycerophosphoryl diester phosphodiesterase	320.093814	0.045500414	0.443420655	0.102612301	0.91827068	0.999677092
1.1.1.88	hydroxymethylglutaryl-CoA reductase	4.580753034	-1.461807639	1.474851378	-0.991155896	0.321609463	0.999677092
3.5.4.19	phosphoribosyl-AMP cyclohydrolase	258.1677959	-0.070064183	0.212514407	-0.32969145	0.741633115	0.999677092
4.1.1.83	4-hydroxyphenylacetate decarboxylase large subunit	66.87725712	-0.269548601	0.61085038	-0.441267796	0.659019137	0.999677092
1.2.1.12	glyceraldehyde 3-phosphate dehydrogenase	3426.713094	0.063236281	0.56517684	0.111887601	0.910912528	0.999677092
3.6.1.21	ADP-sugar diphosphatase	1.525601218	0.161551236	3.593262062	0.044959492	0.964139597	0.999677092
6.5.1.3	RNA-editing ligase	69.07652995	0.119324474	0.779664748	0.153045876	0.878362098	0.999677092
1.3.8.7	acyl-CoA dehydrogenase	0.07857	0.641246863	4.139364228	0.154914337	0.876888851	0.999677092
3.4.24.78	spore protease	147.416855	0.254718078	0.596772682	0.426825969	0.66950609	0.999677092
3.1.3.2	acid phosphatase	20.82235118	-0.519229573	0.830503497	-0.625198539	0.531840761	0.999677092
3.2.2.20	DNA-3-methyladenine glycosylase I	67.37949366	-0.12063882	0.673149809	-0.179215411	0.857768563	0.999677092
3.5.4.25	GTP cyclohydrolase II	294.5812985	-0.092759948	0.303275453	-0.30586039	0.759710946	0.999677092
2.4.1.16	chitin synthase	0.059838501	0.80969603	4.135350731	0.195798635	0.844767778	0.999677092
3.4.22.40	bleomycin hydrolase	151.4023736	0.167568774	0.294346824	0.569290241	0.569159189	0.999677092
2.7.8.13	phospho-N-acetylmuramoyl-pentapeptide-transferase	347.4877158	-0.163377	0.17439136	-0.936841142	0.348840279	0.999677092
1.1.3.48	3-deoxy-alpha-D-manno-octulosonate 8-oxidase	3.68938889	0.801333607	1.338492722	0.598683574	0.549383913	0.999677092
1.1.1.49	glucose-6-phosphate 1-dehydrogenase	351.7397334	-0.146360707	0.637673798	-0.229522849	0.818462564	0.999677092
5.1.3.15	glucose-6-phosphate 1-epimerase	7.796606696	-0.641818012	1.492230394	-0.430106514	0.667118162	0.999677092
2.7.4.23	ribose 1,5-bisphosphokinase	0.110533983	-0.307374933	4.142162324	-0.074206395	0.940846158	0.999677092
3.1.3.4	phosphatidate phosphatase	1.586426012	-0.809974262	2.817975446	-0.28743127	0.773782121	0.999677092
5.2.1.-	#N/A	2.589767527	0.08355121	1.941437214	0.043035752	0.965673035	0.999677092
4.2.1.53	oleate hydratase	647.2225734	0.476153376	0.476085686	1.00014218	0.317241706	0.999677092
5.1.1.1	alanine racemase	418.4676232	0.0740351	0.206520679	0.358487587	0.719978456	0.999677092
5.4.99.24	23S rRNA pseudouridine955/2504/2580 synthase	151.4035972	-0.174776957	0.320223877	-0.545796143	0.585206075	0.999677092
1.1.1.95	D-3-phosphoglycerate dehydrogenase / 2-oxoglutarate reducta	655.6787153	-0.231866612	0.344112013	-0.673811444	0.500431198	0.999677092
3.4.21.26	prolyl oligopeptidase	48.70671163	-0.093331548	0.674337937	-0.138404712	0.889920573	0.999677092
4.2.1.159	dTDP-4-dehydro-6-deoxy-alpha-D-glucopyranose 2,3-dehydrata	3.686345816	0.235397041	2.49183667	0.094467283	0.92473797	0.999677092
3.4.24.84	STE24 endopeptidase	7.536420039	0.203614034	0.975939551	0.208633859	0.834734077	0.999677092
2.7.8.41	cardiolipin synthase (CMP-forming)	61.66824824	-0.893079679	0.582503605	-1.533174508	0.125232863	0.999677092
2.7.8.15	UDP-N-acetylglucosamine--dolichyl-phosphate N-acetylglucosa	1.224751113	-0.571313376	4.099387842	-0.139365534	0.889161306	0.999677092
3.1.3.45	3-deoxy-D-manno-octulosonate 8-phosphate phosphatase (KD(	69.92550246	-0.239814746	0.488254762	-0.491167243	0.623308165	0.999677092
2.4.1.212	hyaluronan synthase	0.179400451	-0.409862564	4.138953896	-0.099025641	0.921117911	0.999677092
2.4.2.44	5'-methylthioinosine phosphorylase	0.035008583	-0.076612487	4.14032189	-0.018503993	0.985236792	0.999677092
3.1.4.55	phosphoribosyl 1,2-cyclic phosphate phosphodiesterase	141.2270294	-0.205456443	0.373331146	-0.550332982	0.582091006	0.999677092
1.10.3.14	#N/A	364.9435136	0.075431628	0.420952463	0.179192748	0.857786358	0.999677092
6.3.4.13	phosphoribosylamine---glycine ligase	488.5378408	-0.053625443	0.149972385	-0.357568779	0.720666046	0.999677092
2.7.1.22	HTH-type transcriptional regulator, transcriptional repressor of	0.749927376	0.104503285	3.748673904	0.027877401	0.977759933	0.999677092
1.1.1.1	alcohol dehydrogenase	554.2128909	0.009753643	0.430768805	0.022642408	0.981935516	0.999677092
2.7.11.13	classical protein kinase C alpha type	1.08659773	0.473720998	4.099154185	0.115565548	0.907996869	0.999677092
4.2.1.120	4-hydroxybutyryl-CoA dehydratase / vinylacetyl-CoA-Delta-ison	56.64405634	-0.547322816	0.422429928	-1.295653505	0.195094886	0.999677092
5.3.1.1	triosephosphate isomerase (TIM)	980.3903779	-0.600549665	0.457043042	-1.313989295	0.188849807	0.999677092
2.7.1.78	bifunctional polynucleotide phosphatase/kinase	2.130257046	0.233880229	2.520016792	0.092808996	0.926055304	0.999677092
3.4.19.12	ubiquitin carboxyl-terminal hydrolase MINDY-1/2	14.16158211	0.45748649	1.883411037	0.242903159	0.808080419	0.999677092
1.6.5.5	NADPH2:quinone reductase	5.194413046	-0.660961345	3.394857138	-0.194694892	0.845631811	0.999677092
5.4.99.-	#N/A	5.046551887	-0.163030459	2.525417597	-0.064555842	0.948527644	0.999677092
6.1.1.10	methionyl-tRNA synthetase	823.9019929	-0.539431226	0.349791067	-1.542152666	0.123036499	0.999677092
6.2.1.1	acetyl-CoA synthetase	128.7675066	0.341467083	0.292708808	1.166576044	0.243381623	0.999677092
3.2.1.35	hyaluronoglucosaminidase	600.3728921	-0.640306968	0.599090027	-1.068799245	0.285160139	0.999677092
1.3.1.31	2-enoate reductase	39.40553896	-0.00568558	0.706441261	-0.0080482	0.993578535	0.999677092
2.7.7.85	diadenylate cyclase	249.0442064	0.086868149	0.291219139	0.298291345	0.76548081	0.999677092
2.5.1.55	2-dehydro-3-deoxyphosphooctonate aldolase (KDO 8-P synthas	101.1365769	0.028426637	0.39534614	0.07190316	0.942678975	0.999677092
3.1.12.1	CRISPR-associated exonuclease Cas4	70.35374821	-0.271525154	0.388816567	-0.698337409	0.48496621	0.999677092
3.6.1.45	UDP-sugar diphosphatase	164.3997029	0.262065621	0.356256502	0.735609371	0.461968458	0.999677092
1.5.5.1	electron-transferring-flavoprotein dehydrogenase	0.962125619	-0.552326604	4.099370945	-0.134734478	0.892821812	0.999677092
2.7.4.-	#N/A	274.03306	-0.169162406	0.230918834	-0.732562186	0.463825501	0.999677092
6.3.2.5	phosphopantothenoylcysteine decarboxylase / phosphopantot	273.46446	0.040559522	0.157126151	0.258133493	0.796303884	0.999677092
4.2.1.1	carbonic anhydrase	88.34783082	0.146539828	0.313093335	0.468038799	0.639756844	0.999677092
4.2.1.119	(3R)-3-hydroxyacyl-CoA dehydrogenase / 3a,7a,12a-trihydroxy-	0.33160195	-0.713627078	4.114731115	-0.173432251	0.862311677	0.999677092
2.7.11.-	#N/A	267.8154515	-0.123147273	0.221151869	-0.556844824	0.577633455	0.999677092
2.7.7.15	choline-phosphate cytidylyltransferase	8.165197538	-0.234941743	1.110121932	-0.21163598	0.832391037	0.999677092
4.1.2.53	2-dehydro-3-deoxy-L-rhamnonate aldolase	0.020495196	0.108319359	4.142686886	0.026147127	0.979139988	0.999677092
2.3.1.191	UDP-3-O-[3-hydroxymyristoyl] glucosamine N-acyltransferase	114.3878791	-0.07414647	0.311476499	-0.23804836	0.811843587	0.999677092
3.1.31.1	micrococcal nuclease	28.70376923	2.827188071	1.480125601	1.910100109	0.056120325	0.999677092
3.4.21.53	ATP-dependent Lon protease	752.9861725	-0.036830868	0.152883984	-0.240907299	0.809626965	0.999677092

2.7.1.63	polyphosphate glucokinase	1.077532702	-0.803564065	4.086626163	-0.196632634	0.844115031	0.999677092
2.1.1.205	tRNA (cytidine32/guanosine34-2'-O)-methyltransferase	2.365052993	-0.276552588	3.982906536	-0.069434868	0.944643476	0.999677092
4.1.1.50	S-adenosylmethionine decarboxylase	17.97606195	0.182696216	0.643591224	0.283869962	0.776510037	0.999677092
2.4.1.187	N-acetylglucosaminyldiphosphoundecaprenol N-acetyl-beta-D-i	145.0502687	-0.340787814	0.371351764	-0.917695424	0.358778344	0.999677092
6.3.1.20	lipoate---protein ligase	123.1964858	-0.200120778	0.60352561	-0.331586223	0.740201724	0.999677092
2.7.7.38	3-deoxy-manno-octulosonate cytidylyltransferase (CMP-KDO sy	88.70939335	-0.291635654	0.423269314	-0.689007317	0.490818663	0.999677092
2.4.1.279	nigerose phosphorylase	0.936136296	-2.499766096	4.10795708	-0.608518066	0.542843928	0.999677092
3.1.1.85	pimeloyl-[acyl-carrier protein] methyl ester esterase	49.33588044	-0.362753797	0.867604736	-0.418109516	0.675867048	0.999677092
3.2.1.130	glycoprotein endo-alpha-1,2-mannosidase	1.452736613	0.198661977	3.614173061	0.054967478	0.956164374	0.999677092
4.1.1.33	diphosphomevalonate decarboxylase	3.592499223	-1.306430272	1.666165611	-0.784093888	0.432985022	0.999677092
6.3.2.-	#N/A	3.844159933	1.725685729	1.474173355	1.170612481	0.241754578	0.999677092
2.4.1.-	#N/A	1222.267335	-0.34039271	0.395781631	-0.860051816	0.389760481	0.999677092
4.2.3.5	chorismate synthase	374.3081163	0.405027299	0.262776297	1.541338785	0.123234354	0.999677092
3.7.1.22	3D-(3,5/4)-trihydroxycyclohexane-1,2-dione acylhydrolase (dec	50.10551438	-0.170334557	0.400519934	-0.425283593	0.670629952	0.999677092
3.5.4.1	cytosine/creatinine deaminase	18.19951039	0.302094336	0.731073433	0.413220235	0.679445264	0.999677092
4.1.3.38	4-amino-4-deoxychorismate lyase	50.77012324	-0.296470858	0.800504452	-0.370355039	0.711117969	0.999677092
6.4.1.2	acetyl-CoA carboxylase / biotin carboxylase 2	597.5241611	-0.232881174	0.349365472	-0.666583257	0.505038366	0.999677092
6.5.1.2	DNA ligase (NAD+)	464.5080408	0.460865674	0.224034294	2.057121105	0.039674578	0.999677092
1.3.3.3	coproporphyrinogen III oxidase	1.057957104	-0.89928995	4.099619301	-0.21935938	0.826370109	0.999677092
5.1.99.1	methylmalonyl-CoA/ethylmalonyl-CoA epimerase	69.18936228	-0.851019699	0.37301422	-2.281467176	0.022520817	0.999677092
1.7.99.1	hydroxylamine reductase	187.9977427	-0.148089689	0.475945054	-0.311148709	0.755687573	0.999677092
5.3.1.22	hydroxypyruvate isomerase	4.146248837	-0.544316263	1.234091113	-0.441066513	0.659164846	0.999677092
3.2.1.155	xyloglucan-specific exo-beta-1,4-glucanase	0.222928835	-3.519603453	4.143731151	-0.849380263	0.395669732	0.999677092
4.2.1.-	#N/A	0.500212525	0.971295726	4.078988653	0.238121703	0.811786702	0.999677092
2.1.1.56	mRNA (guanine-N7-)-methyltransferase	1.79241483	-0.21415522	4.097804513	-0.052260965	0.958320756	0.999677092
6.2.1.22	[citrate (pro-3S)-lyase] ligase	21.13495036	0.945675329	0.503609186	1.877796029	0.060409074	0.999677092
1.17.2.1	nicotinate dehydrogenase subunit A	0.166515913	-0.468055926	4.138090197	-0.113109165	0.909944009	0.999677092
5.3.2.1	phenylpyruvate tautomerase	0.940931647	-0.275380111	4.103331858	-0.067111343	0.946493064	0.999677092
1.1.1.8	glycerol-3-phosphate dehydrogenase (NAD+)	2.798147256	-0.200255884	3.000628283	-0.066737984	0.946790294	0.999677092
2.7.1.53	L-xylulokinase	7.910272842	0.762710021	0.801455369	0.951656262	0.341271338	0.999677092
2.7.7.77	molybdenum cofactor guanylyltransferase	16.25494552	0.458387611	0.86948412	0.527194919	0.598058237	0.999677092
1.1.1.87	homoisocitrate dehydrogenase	0.166515913	-0.468055926	4.138090197	-0.113109165	0.909944009	0.999677092
6.3.2.31	coenzyme F420-0:L-glutamate ligase / coenzyme F420-1:gamma	1.257810662	-0.846182438	4.084521864	-0.207168052	0.835878617	0.999677092
3.2.1.31	beta-glucuronidase	65.88671643	0.008144059	0.521876324	0.015605342	0.987549244	0.999677092
2.4.1.255	protein O-GlcNAc transferase	1.352957927	-0.402892255	4.099355492	-0.098281853	0.921708487	0.999677092
3.2.-.-	#N/A	49.66509319	-0.062064785	0.670764479	-0.092528431	0.926278204	0.999677092
2.3.1.22	2-acylglycerol O-acyltransferase 3	2.917573992	-0.20402025	4.095313723	-0.049817978	0.96026744	0.999677092
2.4.1.8	maltose phosphorylase	23.28563407	-0.116927889	0.727039151	-0.1608275	0.872229266	0.999677092
3.5.1.96	succinylglutamate desuccinylase	0.040667486	0.087870004	4.143731151	0.021205527	0.983081705	0.999677092
3.4.11.1	leucyl aminopeptidase	44.43899661	0.443871389	0.410130846	1.082267755	0.27913357	0.999677092
2.3.2.26	other hect domain ubiquitin protein ligase E3	3.299592357	-0.248772562	3.030040266	-0.082102065	0.934565551	0.999677092
2.5.1.74	1,4-dihydroxy-2-naphthoate polyprenyltransferase	275.5790845	0.054449972	0.722738471	0.075338416	0.939945457	0.999677092
1.1.1.156	glycerol 2-dehydrogenase (NADP+)	0.687316943	0.074803816	4.119797966	0.018157157	0.985513481	0.999677092
3.5.2.14	#N/A	21.13792353	-0.936990989	1.107484098	-0.846053672	0.397522803	0.999677092
2.3.1.28	chloramphenicol O-acetyltransferase type B	85.27593719	-0.499290226	0.505147413	-0.988404995	0.322954342	0.999677092
2.1.1.174	23S rRNA (guanine1835-N2)-methyltransferase	0.193014423	-0.746692262	4.133883336	-0.180627318	0.856660112	0.999677092
5.1.1.13	aspartate racemase	62.58129594	0.2979078	0.529872839	0.562225081	0.573962675	0.999677092
3.5.2.6	beta-lactamase class C	211.9174155	0.02406323	0.274919736	0.087528203	0.930251669	0.999677092
2.7.1.24	dephospho-CoA kinase	234.9531747	0.074391258	0.248754633	0.299054762	0.764898262	0.999677092
3.6.3.55	#N/A	1.048181113	0.713808709	4.060236128	0.175804728	0.860447355	0.999677092
3.13.1.1	UDP-sulfoquinovose synthase	0.039493742	0.43806451	4.143245313	0.105729803	0.915796734	0.999677092
2.1.1.185	23S rRNA (guanosine2251-2'-O)-methyltransferase	292.4598568	-0.019466433	0.179622865	-0.108373915	0.913699092	0.999677092
3.1.3.25	myo-inositol-1(or 4)-monophosphatase	93.69475451	0.014811794	0.401583439	0.036883479	0.970577912	0.999677092
2.7.7.71	D-glycero-alpha-D-manno-heptose 1-phosphate guanylyltransfe	3.00025743	-0.56463718	2.727914853	-0.206984899	0.836021652	0.999677092
3.4.25.1	#N/A	26.70507566	-0.877561405	2.82327769	-0.310830709	0.755929323	0.999677092
3.6.3.9	sodium/potassium-transporting ATPase subunit alpha	1.356910129	0.625516146	3.685193506	0.169737666	0.86521645	0.999677092
1.5.1.20	methylenetetrahydrofolate reductase (NADPH)	318.0389004	-0.235673028	0.181754629	-1.29665489	0.194749957	0.999677092
1.13.11.4	gentisate 1,2-dioxygenase	0.110533983	-0.307374933	4.142162324	-0.074206395	0.940846158	0.999677092
1.2.1.9	glyceraldehyde-3-phosphate dehydrogenase (NADP+)	5.223169037	0.055079974	1.61959517	0.034008483	0.972870386	0.999677092
1.3.98.1	dihydroorotate dehydrogenase (fumarate)	176.8433461	-0.216605615	0.594856425	-0.364130915	0.715760252	0.999677092
3.1.3.100	ribosome biogenesis GTPase / thiamine phosphate phosphatase	362.0250623	-0.022420567	0.225855972	-0.099269311	0.920924445	0.999677092
2.1.1.176	16S rRNA (cytosine967-C5)-methyltransferase	285.324976	0.153953324	0.206932777	0.743977469	0.456890106	0.999677092
3.5.1.2	glutaminase	188.4444747	-0.00533713	0.471218681	-0.011326228	0.990963171	0.999677092
5.5.1.2	3-carboxy-cis,cis-muconate cycloisomerase	0.801064419	-1.697256549	4.121268123	-0.411828713	0.680464974	0.999677092
2.5.1.134	cystathionine beta-synthase (O-acetyl-L-serine)	0.078537166	0.448527249	4.143731151	0.108242362	0.913803442	0.999677092
5.1.3.29	L-fucose mutarotase	71.42123421	-1.037593374	0.54686664	-1.897342602	0.057782736	0.999677092
1.1.1.276	serine 3-dehydrogenase (NADP+)	0.122131417	1.162845254	4.119480069	0.282279616	0.777729114	0.999677092
3.2.1.41	pullulanase	58.97450458	-0.400102574	0.377927494	-1.058675488	0.289747595	0.999677092
2.7.1.100	5-methylthioribose kinase	21.01730638	-0.652631922	0.678653973	-0.961656379	0.336222241	0.999677092
2.7.1.83	pseudouridine kinase	71.962011	0.298579896	0.382105529	0.7814069	0.434563214	0.999677092
4.2.2.1	hyaluronate lyase	44.64325423	-0.61795801	0.637192659	-0.969813448	0.332139489	0.999677092
2.3.1.187	malonate decarboxylase alpha subunit	9.455022523	0.48197534	1.184113931	0.407034599	0.683982576	0.999677092
2.7.11.22	CTD kinase subunit alpha	8.228143643	-0.31911026	4.093818966	-0.077949285	0.937868395	0.999677092
5.3.3.17	trans-2,3-dihydro-3-hydroxyanthranilate isomerase	0.035008583	-0.076612487	4.14032189	-0.018503993	0.985236792	0.999677092
2.5.1.46	deoxyhypusine synthase	3.308839742	-0.223980263	2.391095881	-0.09367264	0.925369205	0.999677092
2.8.3.21	L-carnitine CoA-transferase	1.461921331	2.12811356	3.098790782	0.686756128	0.49223642	0.999677092
6.3.2.10	UDP-N-acetylmuramoyl-tripeptide--D-alanyl-D-alanine ligase	353.4964146	0.118652106	0.167351356	0.708999966	0.478324498	0.999677092
2.7.1.50	hydroxyethylthiazole kinase	118.3186211	0.084682526	0.368483292	0.229813746	0.818236502	0.999677092
3.4.22.16	cathepsin H	6.140464691	0.363273679	4.135497155	0.087842807	0.930001614	0.999677092
2.1.1.258	5-methyltetrahydrofolate corrinoid/iron sulfur protein methyltr	251.1608261	-0.255717383	0.633362039	-0.403745989	0.686399517	0.999677092
1.7.-.-	#N/A	19.90229137	0.667388829	0.964743902	0.691778229	0.489076611	0.999677092
4.2.2.21	chondroitin-sulfate-ABC endolyase/exolyase	92.73215075	0.350250927	0.633520791	0.552864139	0.580356445	0.999677092
1.1.1.76	meso-butanediol dehydrogenase / (S,S)-butanediol dehydrogen	2.544822965	-3.712303495	2.122457999	-1.749058637	0.080280884	0.999677092
2.4.1.83	dolichol-phosphate mannosyltransferase	101.4455041	-0.56820861	0.342350757	-1.65972646	0.096969494	0.999677092
2.7.1.147	ADP-dependent phosphofructokinase/glucokinase	1.596352927	1.859917272	2.884743243	0.644742743	0.519093912	0.999677092
4.2.2.5	chondroitin AC lyase	46.22719144	-0.569583322	0.865127451	-0.658380822	0.510293456	0.999677092
2.1.2.5	glutamate formiminotransferase / 5-formyltetrahydrofolate cyc	152.7191343	0.166300153	0.47568737	0.349599681	0.72663915	0.999677092

1.3.1.44	enoyl-acyl-carrier protein] reductase / trans-2-enoyl-CoA reduc	11.55160526	0.397718892	0.852278784	0.466653517	0.640747791	0.999677092
1.5.1.41	aquacobalamin reductase / NAD(P)H-flavin reductase	1.401765292	-1.311531389	3.964718721	-0.330800614	0.740795097	0.999677092
3.5.1.38	glutamin-(asparagin-)ase	0.486306948	-0.761617756	4.109267002	-0.185341511	0.852961181	0.999677092
2.7.1.205	PTS system, cellobiose-specific IIA component	42.12668325	-0.81491506	0.501512593	-1.62491445	0.104180789	0.999677092
3.2.1.20	alpha-glucosidase	623.9134192	-0.199501874	0.373976149	-0.533461492	0.593714161	0.999677092
1.13.12.16	nitronate monooxygenase	2.660689283	0.933041548	1.626905795	0.573506807	0.566301591	0.999677092
2.4.1.261	alpha-1,2-mannosyltransferase	1.191163093	0.076473384	4.098417029	0.018659249	0.985112937	0.999677092
2.1.1.191	23S rRNA (cytosine1962-C5)-methyltransferase	406.1474614	0.020488794	0.19627911	0.104386015	0.91686302	0.999677092
3.7.1.3	kynureninase	0.600135268	1.428373868	4.106531697	0.347829744	0.727968051	0.999677092
2.4.2.22	xanthine phosphoribosyltransferase	289.7354219	0.326378078	0.452294907	0.721604583	0.47053762	0.999677092
4.6.1.-	#N/A	0.249773869	-0.680453166	4.123822835	-0.165005431	0.868939707	0.999677092
6.1.1.15	prolyl-tRNA synthetase	485.2734754	-0.075851235	0.152539485	-0.497256397	0.619008256	0.999677092
4.4.1.-	#N/A	0.783122018	-0.385414013	4.10490287	-0.093891141	0.925195631	0.999677092
2.3.2.6	leucyl/phenylalanyl-tRNA---protein transferase	29.52000004	0.247225287	0.661097861	0.37396171	0.708432804	0.999677092
2.3.1.78	heparan-alpha-glucosaminide N-acetyltransferase	0.457925214	-0.329009811	4.112413712	-0.080004064	0.936234024	0.999677092
1.1.1.47	glucose 1-dehydrogenase	18.79187857	0.499400295	1.150849914	0.43394042	0.664331707	0.999677092
3.1.6.1	arylsulfatase	68.00014758	-0.588743902	0.863264517	-0.681997106	0.495240781	0.999677092
6.2.1.45	ubiquitin-activating enzyme E1	6.168451976	-1.49873337	2.120808027	-0.706680355	0.47976514	0.999677092
2.7.1.148	4-diphosphocytidyl-2-C-methyl-D-erythritol kinase	306.3553243	0.024693321	0.265616096	0.092966206	0.925930408	0.999677092
1.2.1.3	aldehyde dehydrogenase (NAD+)	54.50178897	-0.256239171	0.500026287	-0.512451399	0.608335126	0.999677092
3.5.1.81	N-acyl-D-amino-acid deacylase	49.05676068	0.584866963	0.547529548	1.068192512	0.285433682	0.999677092
5.4.99.12	#N/A	317.454115	-0.189224072	0.240217686	-0.787719155	0.430860999	0.999677092
2.3.1.16	acetyl-CoA acyltransferase	1.009606782	-2.095308762	3.553976412	-0.589567436	0.555480689	0.999677092
4.2.1.126	N-acetylmuramic acid 6-phosphate etherase	72.37809151	0.196909289	0.413869401	0.475776388	0.634233694	0.999677092
3.5.3.6	arginine deiminase	75.43333123	0.067540657	0.469140733	0.14396673	0.885526742	0.999677092
2.7.7.56	ribonuclease PH	38.61928976	0.402262378	0.607140051	0.662552861	0.507616969	0.999677092
2.4.1.166	raffinose-raffinose alpha-galactotransferase	0.473040041	1.321365998	4.093846348	0.32276883	0.74687032	0.999677092
2.7.8.39	archaetidylinositol phosphate synthase	0.782416094	-0.236554	4.102808976	-0.057656596	0.954022167	0.999677092
3.1.27.-	#N/A	2.565835127	-0.212304714	3.186783523	-0.066620375	0.946883925	0.999677092
6.3.4.3	methylenetetrahydrofolate dehydrogenase (NADP+) / metheny	810.1039202	-0.093430436	0.289443416	-0.32279344	0.746851681	0.999677092
5.3.2.8	4-oxalomesaconate tautomerase	0.020495196	0.108319359	4.142686886	0.026147127	0.979139988	0.999677092
1.1.3.9	galactose oxidase	0.296485225	0.653179872	4.111279081	0.158875099	0.873767279	0.999677092
2.7.7.59	[protein-PII] uridylyltransferase	10.91023372	0.283598542	1.164249224	0.243589204	0.80754899	0.999677092
3.6.1.54	UDP-2,3-diacylglucosamine hydrolase	93.92320793	-0.68367785	0.516694648	-1.323175792	0.185776924	0.999677092
3.4.11.19	D-aminopeptidase	57.32240294	0.437942929	0.45446368	0.963647808	0.335222529	0.999677092
2.7.1.199	PTS system, glucose-specific IIB component	59.39654169	-0.160957154	0.593194327	-0.271339671	0.786129797	0.999677092
2.7.7.41	phosphatidate cytidylyltransferase	426.9388583	0.001551546	0.232020657	0.006687104	0.994664503	0.999677092
2.1.1.74	methylenetetrahydrofolate--tRNA-(uracil-5-)-methyltransferase	123.201116	0.374948887	0.357957673	1.047467104	0.294884196	0.999677092
2.7.6.2	thiamine pyrophosphokinase	207.1046171	-0.324443904	0.191214622	-1.696752582	0.089743447	0.999677092
4.3.1.18	D-serine dehydratase	35.25449199	0.144565487	0.452302776	0.319621047	0.749255618	0.999677092
2.5.1.108	2-(3-amino-3-carboxypropyl)histidine synthase	0.899296214	-0.42419324	4.102365953	-0.103402096	0.917643848	0.999677092
2.4.1.1	glycogen phosphorylase	967.5698812	-0.066672383	0.194548586	-0.342702994	0.73182191	0.999677092
4.2.1.30	#N/A	13.82081441	-0.241533387	1.058975108	-0.228082214	0.819582327	0.999677092
2.7.1.91	sphingosine kinase	0.070017166	-0.317095317	4.138829611	-0.076614731	0.93893004	0.999677092
2.7.7.65	two-component system, cell cycle response regulator	2.26188146	-1.480890439	2.22367664	-0.665964832	0.50543358	0.999677092
1.3.5.4	succinate dehydrogenase / fumarate reductase, flavoprotein su	851.5573064	0.200227974	0.231558028	0.864698909	0.387204068	0.999677092
3.6.3.4	Cu2+-exporting ATPase	720.9885904	0.891851395	0.45141729	1.975669553	0.048192215	0.999677092
2.1.3.9	N-acetylornithine carbamoyltransferase	0.471782961	0.558567533	4.105970589	0.13603788	0.891791336	0.999677092
1.1.5.6	#N/A	0.834744568	0.63464266	4.101452185	0.154736087	0.87702938	0.999677092
2.4.1.52	poly(glycerol-phosphate) alpha-glucosyltransferase	0.626809568	0.621165548	4.125917992	0.150552083	0.880329063	0.999677092
4.1.1.31	phosphoenolpyruvate carboxylase	14.51864403	-0.648118319	0.730330678	-0.887431322	0.374846726	0.999677092
4.1.2.22	xylulose-5-phosphate/fructose-6-phosphate phosphoketolase	113.3229537	0.143142376	0.30375757	0.471238877	0.637470159	0.999677092
3.6.3.25	sulfate transport system ATP-binding protein	37.65838914	0.147663439	0.774828191	0.190575718	0.848858016	0.999677092
3.6.1.15	#N/A	4.552541307	0.622207091	1.526785802	0.407527428	0.683620653	0.999677092
3.6.3.36	taurine transport system ATP-binding protein	1.346509197	0.341615555	2.66398505	0.128234787	0.897963171	0.999677092
3.2.1.73	licheninase	7.463162102	-0.80951305	1.646150796	-0.491761175	0.622888186	0.999677092
2.1.1.309	18S rRNA (guanine1575-N7)-methyltransferase	2.903351773	-0.539797193	3.779840339	-0.142809522	0.886440617	0.999677092
1.3.8.1	butyryl-CoA dehydrogenase	257.9828952	-0.094169852	0.521132903	-0.18070218	0.856601347	0.999677092
1.8.5.3	anaerobic dimethyl sulfoxide reductase subunit A	14.50986278	0.067378181	0.689501456	0.097720143	0.92215452	0.999677092
5.4.3.2	lysine 2,3-aminomutase	201.7077419	-0.105547964	0.396423814	-0.266250311	0.790046438	0.999677092
2.6.1.2	alanine transaminase	96.41482852	-0.63440644	0.517465036	-1.225988997	0.220202816	0.999677092
1.3.1.74	2-alkenal reductase	0.667842043	-3.29382582	4.143731151	-0.794893708	0.426675334	0.999677092
3.1.3.48	protein-tyrosine phosphatase	496.7287062	-0.034988687	0.298570377	-0.117187403	0.906711549	0.999677092
5.4.99.29	tRNA pseudouridine32 synthase / 23S rRNA pseudouridine746 s	66.23259907	-0.206478592	0.574821053	-0.359204992	0.719441743	0.999677092
3.1.21.4	type II restriction enzyme	19.39455655	-1.407698749	0.730701063	-1.926504313	0.054041434	0.999677092
2.3.1.97	glycylpeptide N-tetradecanoyltransferase	2.404544264	0.064410587	3.865847408	0.016661441	0.986706709	0.999677092
4.6.1.16	tRNA-intron endonuclease, archaea type	0.578535221	-0.530644066	4.105958946	-0.129237548	0.897169686	0.999677092
1.18.1.3	3-phenylpropionate/trans-cinnamate dioxygenase ferredoxin re	0.36309423	-1.683241808	4.119081808	-0.408644908	0.682800269	0.999677092
3.1.6.6	choline-sulfatase	24.56530346	-0.273254521	0.896415241	-0.304830293	0.760495408	0.999677092
1.8.5.2	thiosulfate dehydrogenase [quinone] small subunit	17.43969102	-1.337360008	1.184255142	-1.129283683	0.258778182	0.999677092
6.3.1.8	glutathionylspermidine amidase/synthetase	1.249834218	-1.802092233	3.40217836	-0.529687759	0.596328436	0.999677092
2.3.1.9	acetyl-CoA C-acetyltransferase	806.2977231	-0.261258136	0.572147872	-0.456626947	0.647939207	0.999677092
1.1.1.28	D-lactate dehydrogenase	327.873437	-0.407134563	0.464256757	-0.876959908	0.380508415	0.999677092
2.7.1.167	D-beta-D-heptose 7-phosphate kinase / D-beta-D-heptose 1-ph	30.61416296	-0.374467891	0.695175583	-0.538666634	0.590116901	0.999677092
4.3.2.2	adenylosuccinate lyase	603.0231759	-0.102723204	0.230407031	-0.445833634	0.655717425	0.999677092
6.2.1.31	2-furoate---CoA ligase	0.078987483	0.401836267	4.14177237	0.097020365	0.922710222	0.999677092
3.2.1.139	alpha-glucuronidase	61.56210552	0.167241018	0.669011731	0.249982191	0.802601121	0.999677092
4.2.1.77	trans-L-3-hydroxyproline dehydratase	0.556212935	1.255763788	4.10936953	0.305585511	0.759920254	0.999677092
2.3.1.89	tetrahydrodipicolinate N-acetyltransferase	3.32986813	0.757725759	1.065502851	0.711143812	0.476995123	0.999677092
1.5.1.2	pyrroline-5-carboxylate reductase	402.521796	-0.319208215	0.448205232	-0.712192076	0.476345843	0.999677092
1.7.2.5	nitric oxide reductase subunit B	0.040990391	-0.099691597	4.140771598	-0.024075609	0.980792299	0.999677092
2.4.1.11	glycogen synthase	55.29743903	1.002086565	0.649854237	1.542017437	0.123069356	0.999677092
2.8.1.6	biotin synthase	211.0746319	-0.363904178	0.329669984	-1.103843829	0.269660889	0.999677092
2.7.1.71	shikimate kinase	453.6115401	-0.004648018	0.386985262	-0.01201084	0.990416967	0.999677092
3.5.1.47	N-acetyldiaminopimelate deacetylase	5.108520293	1.666604361	1.420525311	1.173231021	0.240703175	0.999677092
1.2.5.1	pyruvate dehydrogenase (quinone)	71.21907842	-0.245994434	0.651818382	-0.377397203	0.705878445	0.999677092
4.99.1.1	protoporphyrin/coproporphyrin ferrochelatase	7.519813609	-0.121436902	0.785914722	-0.15451664	0.877202392	0.999677092

2.4.2.52	triphosphoribosyl-dephospho-CoA synthase	19.79538825	0.432859697	0.587814471	0.736388297	0.461494425	0.999677092
1.3.4.1	fumarate reductase (CoM/CoB) subunit A	2.34115809	-1.056061154	4.091700464	-0.258098354	0.796331002	0.999677092
6.2.1.5	succinyl-CoA synthetase alpha subunit	166.7141587	-0.360935586	0.641331417	-0.562791056	0.573577171	0.999677092
4.2.1.136	ADP-dependent NAD(P)H-hydrate dehydratase	352.2405352	0.100758297	0.346893909	0.290458536	0.771465468	0.999677092
5.1.3.10	CDP-paratose 2-epimerase	0.695290102	-1.189669212	4.109961181	-0.289459963	0.772229414	0.999677092
2.7.1.153	phosphatidylinositol-4,5-bisphosphate 3-kinase catalytic subuni	1.323599265	-0.290370431	4.098894826	-0.070841152	0.94352418	0.999677092
1.5.1.39	FMN reductase [NAD(P)H]	10.96471843	0.860920804	0.746128534	1.153850528	0.248561454	0.999677092
4.1.99.14	spore photoproduct lyase	6.75289143	0.228802631	1.106805607	0.206723411	0.836225873	0.999677092
1.1.1.281	GDP-4-dehydro-6-deoxy-D-mannose reductase	10.70714889	1.472382444	1.14780922	1.282776283	0.199570466	0.999677092
6.3.1.10	adenosylcobinamide-phosphate synthase	125.3070087	-0.182331237	0.381234447	-0.478265378	0.632461335	0.999677092
5.4.2.7	phosphopentomutase	326.2463797	-0.149908967	0.444143122	-0.337524009	0.735721917	0.999677092
2.7.1.190	aminoglycoside 2''-phosphotransferase	3.45828159	-0.601369737	2.223207555	-0.270496444	0.786778353	0.999677092
1.12.98.2	5,10-methenyltetrahydromethanopterin hydrogenase	10.46252972	-0.622329125	1.813983264	-0.343073245	0.731543358	0.999677092
2.7.1.36	mevalonate kinase	4.067718738	-0.691000973	1.926536955	-0.358675171	0.719838105	0.999677092
4.2.1.91	cyclohexadienyl dehydratase	4.49090516	1.059750772	1.411455453	0.750821268	0.452760228	0.999677092
3.5.4.39	GTP cyclohydrolase IV	1.827417476	-0.453165426	4.097236046	-0.110602714	0.911931395	0.999677092
5.3.1.8	mannose-6-phosphate isomerase	421.1889985	-0.617574594	0.67169762	-0.919423526	0.357874089	0.999677092
3.1.3.71	2-phosphosulfolactate phosphatase	2.504384397	-0.149996648	1.519794848	-0.098695326	0.92138018	0.999677092
2.7.11.33	[pyruvate, water dikinase]-phosphate phosphotransferase / [py	9.875085199	-0.230823304	0.667810463	-0.345641941	0.729611822	0.999677092
2.7.1.19	phosphoribulokinase	0.688126876	-1.981954399	4.109473353	-0.482289147	0.629600556	0.999677092
1.2.1.24	succinate-semialdehyde dehydrogenase	0.649086519	-1.011226587	4.10957706	-0.246065854	0.805631255	0.999677092
2.7.1.168	D-glycero-alpha-D-manno-heptose-7-phosphate kinase	31.41129843	-0.930649191	0.740007668	-1.25762101	0.208528851	0.999677092
4.1.3.42	2-dehydro-3-deoxyphosphogluconate aldolase / (4S)-4-hydroxy	296.8325156	0.102901204	0.293069656	0.351115177	0.725501942	0.999677092
3.1.3.15	imidazoleglycerol-phosphate dehydratase / histidinol-phosphat	717.5286595	-0.677671162	0.457643011	-1.480785557	0.138663726	0.999677092
4.3.1.14	3-aminobutyryl-CoA ammonia-lyase	95.00817285	0.090343333	0.512955079	0.176123284	0.860197089	0.999677092
1.8.2.2	thiosulfate dehydrogenase	0.118481225	0.379366906	4.141001742	0.091612351	0.927006038	0.999677092
2.3.1.54	formate C-acetyltransferase	1844.276914	-0.545113014	0.521345744	-1.045588307	0.29575115	0.999677092
3.5.4.29	GTP cyclohydrolase IIa	1.229475	-0.181264493	4.098714163	-0.044224722	0.964725276	0.999677092
1.5.99.6	spermidine dehydrogenase	0.131528836	-0.465999869	4.137447435	-0.112629798	0.91032406	0.999677092
5.3.4.1	protein disulfide-isomerase	30.55821104	-0.771257644	1.053343504	-0.732199554	0.464046777	0.999677092
2.4.1.117	dolichyl-phosphate beta-glucosyltransferase	0.969773766	0.036996013	4.100733749	0.009021803	0.99280174	0.999677092
4.1.3.40	chorismate--pyruvate lyase	0.460350927	-0.985906724	4.117061643	-0.239468536	0.810742291	0.999677092
3.1.11.1	exodeoxyribonuclease I	5.432575923	0.821960313	0.763251217	1.076919755	0.281516117	0.999677092
2.3.1.235	minimal PKS ketosynthase (KS/KS alpha)	0.207448764	-4.061570657	4.143731151	-0.98017234	0.327001056	0.999677092
4.4.1.24	(2R)-sulfolactate sulfo-lyase subunit alpha	6.567064541	0.52157617	1.063714885	0.49033456	0.623897176	0.999677092
3.5.1.11	penicillin amidase	0.017504292	0.120319428	4.141980666	0.029048766	0.976825697	0.999677092
3.4.14.4	dipeptidyl-peptidase III	511.5424619	-0.638280299	0.689877324	-0.925208405	0.354857512	0.999677092
3.4.21.105	rhomboid-related protein 1/2/3	65.52118998	-0.538736893	0.405061244	-1.330013426	0.183513848	0.999677092
3.1.6.-	#N/A	21.90800047	0.215901446	0.617136209	0.349844075	0.726455719	0.999677092
6.1.1.9	valyl-tRNA synthetase	560.2418703	-0.082033611	0.236792889	-0.346436127	0.729014979	0.999677092
2.7.4.25	CMP/dCMP kinase	352.665934	0.188287952	0.238071498	0.790888254	0.429009203	0.999677092
1.2.1.59	glyceraldehyde-3-phosphate dehydrogenase (NAD(P))	5.22268142	-0.563824259	1.643434735	-0.343076757	0.731540717	0.999677092
1.1.1.25	shikimate dehydrogenase	278.5865007	0.556289107	0.288220422	1.930082204	0.053596653	0.999677092
4.2.1.59	3-hydroxyacyl-[acyl-carrier protein] dehydratase / trans-2-dece	228.3164338	-0.376432819	0.280863061	-1.340271722	0.180157021	0.999677092
2.3.1.182	(R)-citramalate synthase	88.16405049	-0.51391431	0.314055967	-1.636378114	0.101760477	0.999677092
2.7.1.32	choline kinase	2.466264155	-0.420323963	4.096037618	-0.102617212	0.918266781	0.999677092
4.1.1.65	phosphatidylserine decarboxylase	131.5107749	-0.164122034	0.381008154	-0.430757275	0.666644868	0.999677092
3.4.21.92	ATP-dependent Clp protease, protease subunit	546.6920958	0.094496691	0.286426693	0.329915797	0.741463587	0.999677092
2.7.7.61	holo-ACP synthase	18.79323321	0.126173979	0.526798842	0.239510737	0.810709572	0.999677092
2.7.1.157	N-acetylgalactosamine kinase	0.82830909	-0.597337677	4.101940473	-0.145623195	0.884218856	0.999677092
3.3.2.1	bifunctional isochorismate lyase / aryl carrier protein	0.147099389	-0.701105853	4.136777274	-0.16948117	0.865418182	0.999677092
2.7.9.2	pyruvate, water dikinase	53.46043133	0.390459617	0.432467038	0.902865613	0.366597223	0.999677092
1.2.1.10	acetaldehyde dehydrogenase (acetylating)	462.7407636	-0.001183402	0.526165681	-0.002249106	0.998205475	0.999677092
1.3.1.43	cyclohexadieny/prephenate dehydrogenase	0.078537166	0.448527249	4.143731151	0.108242362	0.913803442	0.999677092
4.1.3.3	N-acetylneuraminate lyase	310.9855885	-0.352021513	0.407114992	-0.864673421	0.387218062	0.999677092
5.4.99.1	methylaspartate mutase sigma subunit	88.22815367	1.342568561	0.57832448	2.321479738	0.020260966	0.999677092
2.3.3.14	homocitrate synthase	1.443527753	-1.528051216	3.488035346	-0.438083639	0.661325654	0.999677092
6.3.4.14	acetyl-CoA carboxylase / biotin carboxylase 2	325.4663148	-0.512058258	0.361504529	-1.416464297	0.15663961	0.999677092
1.-.-.-	#N/A	74.03478962	0.220183781	0.397045846	0.554555056	0.579199038	0.999677092
1.16.1.1	mercuric reductase	0.246942505	0.448520513	4.143731151	0.108240737	0.913804731	0.999677092
1.16.3.1	bacterioferritin	121.0308506	-0.042789842	0.493835348	-0.086647994	0.930951315	0.999677092
4.1.2.19	rhamnulose-1-phosphate aldolase	129.9339125	0.060098201	0.427008389	0.140742436	0.88807342	0.999677092
2.5.1.129	flavin prenyltransferase	18.81128005	-0.367705708	0.965180948	-0.380970749	0.703224954	0.999677092
1.1.1.271	GDP-L-fucose synthase	230.4977144	-0.633557659	0.390688054	-1.621645843	0.104879201	0.999677092
3.4.17.11	glutamate carboxypeptidase	31.27839536	-0.004601471	0.525879994	-0.008750039	0.993018568	0.999677092
2.3.1.243	lauroyl-Kdo2-lipid IVA myristoyltransferase	0.925009175	-0.326633932	4.093735839	-0.079788718	0.936405298	0.999677092
1.8.4.14	L-methionine (R)-S-oxide reductase	1.882391337	1.633256683	3.188736828	0.512195509	0.608514186	0.999677092
1.6.3.4	NADH oxidase (H2O-forming)	0.020495196	0.108319359	4.142686886	0.026147127	0.979139988	0.999677092
4.4.1.15	D-cysteine desulfhydrase	23.97297618	0.533534612	0.528162023	1.010172237	0.312412778	0.999677092
2.7.8.42	KDO II ethanolaminephosphotransferase	0.213509619	-0.800234868	4.133062028	-0.193617919	0.846475067	0.999677092
2.6.1.59	dTDP-4-amino-4,6-dideoxygalactose transaminase	60.17477876	-0.176565613	0.311028458	-0.567683145	0.570250138	0.999677092
1.4.1.14	glutamate synthase (NADH)	1037.619229	-0.044246315	0.172034442	-0.257194516	0.797028618	0.999677092
3.1.3.7	3'(2'), 5'-bisphosphate nucleotidase	473.5000343	0.168763229	0.207949259	0.811559658	0.417044348	0.999677092
2.4.1.315	processive 1,2-diacylglycerol beta-glucosyltransferase	64.5026741	0.184619247	0.303862137	0.607575688	0.54346893	0.999677092
3.2.1.84	#N/A	2.28393639	-0.614324349	4.0960782	-0.149978667	0.880781446	0.999677092
1.1.1.61	4-hydroxybutyrate dehydrogenase	27.54769835	-0.232518301	0.512178773	-0.453978793	0.649844091	0.999677092
3.6.3.44	#N/A	6.055494739	-0.38217269	1.96019926	-0.194966246	0.845419373	0.999677092
2.7.7.82	CMP-N,N'-diacetyllegionaminic acid synthase	10.71142702	0.014430738	0.618527639	0.02333079	0.981386412	0.999677092
3.2.1.65	levanase	13.36105166	-0.350995669	0.928091888	-0.378190644	0.705288975	0.999677092
2.7.4.1	polyphosphate kinase	535.6898625	0.018318385	0.194446396	0.094207891	0.924944017	0.999677092
3.1.-.-	#N/A	7256.639758	0.193817583	0.215527634	0.899270222	0.368508744	0.999677092
1.6.4.-	#N/A	210.9950788	-0.349122628	0.601569488	-0.580352951	0.561676626	0.999677092
3.5.1.18	succinyl-diaminopimelate desuccinylase	253.8850799	0.155965528	0.406779794	0.383415132	0.701411985	0.999677092
2.3.3.-	#N/A	0.541183171	-0.384791177	4.108798836	-0.093650527	0.925386771	0.999677092
1.14.13.81	magnesium-protoporphyrin IX monomethyl ester (oxidative) cy	0.020495196	0.108319359	4.142686886	0.026147127	0.979139988	0.999677092
3.1.3.41	4-nitrophenyl phosphatase	2.491093023	2.347440874	2.053504603	1.143138842	0.25298095	0.999677092
2.3.1.48	chromodomain protein Y	6.701815243	-2.786017819	1.762269641	-1.580925957	0.113894968	0.999677092

3.1.3.10	glucose-1-phosphatase	1.299394767	-1.265932927	3.49689789	-0.362015983	0.717340087	0.999677092
6.3.2.29	cyanophycin synthetase	0.53521663	-0.174159011	4.111715597	-0.042356775	0.966214286	0.999677092
2.4.2.18	anthranilate phosphoribosyltransferase	118.0786532	0.314822348	0.525943862	0.598585458	0.549449355	0.999677092
2.4.1.293	GalNAc5-diNAcBac-PP-undecaprenol beta-1,3-glucosyltransfera	1.168164939	1.297010887	4.099647397	0.316371327	0.751720678	0.999677092
3.2.1.23	beta-galactosidase	3081.654415	-0.101678712	0.15376827	-0.661246379	0.508454322	0.999677092
4.2.1.55	3-hydroxybutyryl-CoA dehydratase	11.55891586	-0.442799137	1.039034841	-0.426163897	0.669988423	0.999677092
4.2.1.7	altronate hydrolase	265.8229694	0.143061926	0.259852515	0.550550479	0.581941864	0.999677092
3.5.1.88	peptide deformylase	337.0782091	0.007393578	0.168382026	0.043909547	0.964976505	0.999677092
2.1.1.177	23S rRNA (pseudouridine1915-N3)-methyltransferase	198.3826389	0.787911291	0.365109764	2.158012104	0.030926891	0.999677092
6.5.1.6	DNA ligase 1	6.069130183	-0.528571705	1.508227305	-0.350458915	0.725994319	0.999677092
2.6.1.42	branched-chain amino acid aminotransferase	374.7399444	0.061393525	0.205218658	0.299161515	0.764816812	0.999677092
2.6.1.16	glucosamine---fructose-6-phosphate aminotransferase (isomeri	553.618185	-0.03261222	0.224943527	-0.144979587	0.884726989	0.999677092
4.1.99.20	3-amino-4-hydroxybenzoic acid synthase	0.49136452	-1.98787398	4.113207215	-0.483290502	0.628889483	0.999677092
2.7.7.52	terminal uridylyltransferase	0.442154223	-0.528861534	4.111733935	-0.128622509	0.897656355	0.999677092
5.3.1.13	arabinose 5-phosphate isomerase	85.30070013	-0.175740565	0.437937493	-0.401291434	0.68820557	0.999677092
5.4.99.21	23S rRNA pseudouridine2604 synthase	51.97311598	-0.979385044	0.550355123	-1.779551062	0.075149461	0.999677092
3.1.4.3	phospholipase C	6.386108775	-1.115154182	1.767662287	-0.63086382	0.52812957	0.999677092
4.1.1.73	tartrate dehydrogenase/decarboxylase / D-malate dehydrogen	5.410002282	0.263459365	0.972147822	0.271007514	0.786385253	0.999677092
2.4.2.-	#N/A	195.2219842	-0.186233866	0.262904961	-0.70836954	0.478715803	0.999677092
3.1.1.32	phospholipase A1/A2	2.533868293	-0.613365067	1.908359951	-0.321409526	0.747900065	0.999677092
1.1.1.303	(R,R)-butanediol dehydrogenase / meso-butanediol dehydroger	2.80919775	-0.373009592	1.590975653	-0.234453363	0.814633035	0.999677092
3.8.1.3	haloacetate dehalogenase	2.59668313	0.891256194	2.348068284	0.379569964	0.70426466	0.999677092
1.1.1.2	alcohol dehydrogenase (NADP+)	4.141518092	-0.030809074	2.36914458	-0.013004303	0.98962436	0.999677092
3.4.14.11	X-Pro dipeptidyl-peptidase	18.79872223	-1.706858146	0.92015933	-1.854959343	0.063602033	0.999677092
1.5.1.3	dihydrofolate reductase	135.387892	-0.445237738	0.339035001	-1.31325007	0.189098697	0.999677092
2.7.11.26	glycogen synthase kinase 3 beta	2.032021168	-0.208124362	4.09646171	-0.050805885	0.959480202	0.999677092
2.5.1.17	cob(I)alamin adenosyltransferase	391.6330143	-0.097597058	0.243798823	-0.400318002	0.68892231	0.999677092
2.1.1.62	#N/A	0.71527601	-0.355724565	4.105337639	-0.086649283	0.930950291	0.999677092
2.7.1.48	uridine kinase	2305.548305	1.194403344	0.704643757	1.695045663	0.090066759	0.999677092
2.5.1.30	heptaprenyl diphosphate synthase	94.25819124	-0.221759488	0.350911855	-0.631952114	0.527418168	0.999677092
2.7.7.4	bifunctional enzyme CysN/CysC	241.1413969	-0.580956593	0.420254709	-1.382391631	0.166851484	0.999677092
6.2.1.26	O-succinylbenzoic acid---CoA ligase	92.79033664	-0.626096565	0.561436853	-1.115168273	0.264778319	0.999677092
3.2.2.4	AMP nucleosidase	32.96225727	-0.596263036	0.676350943	-0.881588237	0.377999519	0.999677092
3.4.19.3	pyroglutamyl-peptidase	46.39155512	0.338746199	0.463986235	0.730078123	0.465342433	0.999677092
1.2.7.7	2-oxoisovalerate ferredoxin oxidoreductase, alpha subunit	4.383532921	-1.581902098	2.645642112	-0.597927471	0.549888329	0.999677092
3.6.3.5	Cd2+/Zn2+-exporting ATPase	575.5346922	0.086770587	0.277384335	0.312817186	0.754419557	0.999677092
5.3.1.26	galactose-6-phosphate isomerase	8.787182483	-1.298865642	1.585386076	-0.819274031	0.412630085	0.999677092
5.1.3.4	#N/A	158.5584007	-0.175017517	0.307218731	-0.569683745	0.568892217	0.999677092
1.1.1.264	L-idonate 5-dehydrogenase	0.035008583	-0.076612487	4.14032189	-0.018503993	0.985236792	0.999677092
3.2.1.136	glucuronoarabinoxylan endo-1,4-beta-xylanase	28.86644239	1.419331066	0.767151484	1.850131423	0.06429461	0.999677092
5.1.3.35	2-epi-5-epi-valiolone 7-phosphate 2-epimerase	0.538064334	-2.12362409	4.117049735	-0.515812105	0.605985647	0.999677092
2.7.4.14	UMP-CMP kinase	8.48881237	-0.486141256	2.109290571	-0.230476191	0.817721763	0.999677092
2.7.8.33	UDP-GlcNAc:undecaprenyl-phosphate/decaprenyl-phosphate G	45.98542719	-0.065008415	0.483355017	-0.134494135	0.893011848	0.999677092
2.7.1.28	triose/dihydroxyacetone kinase / FAD-AMP lyase (cyclizing)	1.318253244	-1.117790696	2.748521871	-0.406687939	0.6842372	0.999677092
4.1.1.17	ornithine decarboxylase	76.59343413	0.539947697	0.767340515	0.703661135	0.481643829	0.999677092
5.4.2.12	2,3-bisphosphoglycerate-independent phosphoglycerate mutas	1222.827048	-0.263857914	0.339505205	-0.777183709	0.437050392	0.999677092
2.7.4.7	hydroxymethylpyrimidine/phosphomethylpyrimidine kinase / t	196.5194831	-0.161383665	0.270883478	-0.59576784	0.551330331	0.999677092
1.1.1.330	17beta-estradiol 17-dehydrogenase / very-long-chain 3-oxoacyl	0.771316995	-0.055954859	4.105202021	-0.013630233	0.989124985	0.999677092
2.1.1.43	histone-lysine N-methyltransferase ASH1L	3.621512174	-0.326278797	3.536388429	-0.09226328	0.926488862	0.999677092
2.7.4.29	Kdo2-lipid A phosphotransferase	0.671500197	0.119413979	4.099423786	0.029129454	0.976761344	0.999677092
2.6.1.83	LL-diaminopimelate aminotransferase	298.573527	-0.021069431	0.267800569	-0.078675827	0.937290474	0.999677092
3.1.4.16	2',3'-cyclic-nucleotide 2'-phosphodiesterase / 3'-nucleotidase	285.4621956	-0.168462923	0.44495182	-0.378609358	0.704977972	0.999677092
2.4.1.4	amylosucrase	262.5900682	0.017144109	0.445003743	0.038525764	0.96926849	0.999677092
4.6.1.1	adenylate cyclase	15.07032412	-1.566470323	0.817165517	-1.916955978	0.055243525	0.999677092
2.7.8.29	phosphatidylserine synthase 2	0.372518596	-0.355951547	4.112618094	-0.086551082	0.931028351	0.999677092
3.1.21.-	#N/A	430.4301869	-0.12971496	0.309964654	-0.418483069	0.675593963	0.999677092
4.1.1.55	4,5-dihydroxyphthalate decarboxylase	0.035008583	-0.076612487	4.14032189	-0.018503993	0.985236792	0.999677092
2.5.1.15	dihydropteroate synthase	181.9806537	-0.608412181	0.448882422	-1.355393197	0.175292316	0.999677092
2.7.7.49	RNA-directed DNA polymerase	100.8236658	-0.191226514	0.535768286	-0.356920181	0.721151561	0.999677092
4.2.2.24	rhamnogalacturonan exolyase	2.020556691	0.139036029	2.556363336	0.054388211	0.956625871	0.999677092
2.6.1.55	taurine---2-oxoglutarate transaminase	19.30889591	-0.81074591	0.657859752	-1.232399318	0.217799956	0.999677092
1.8.4.10	phosphoadenosine phosphosulfate reductase	38.80354416	-0.535091701	1.100209927	-0.486354184	0.626716069	0.999677092
3.2.1.91	cellulose 1,4-beta-cellobiosidase	0.566914952	-2.228586564	4.111995012	-0.541972098	0.587837727	0.999677092
1.5.1.5	methylenetetrahydrofolate dehydrogenase (NADP+) / metheny	460.8469926	-0.196276476	0.325606746	-0.602802241	0.546640256	0.999677092
2.8.4.1	#N/A	74.49309531	0.931317349	1.54799309	0.60162888	0.5474212	0.999677092
2.8.1.10	thiazole synthase	168.3003271	-0.210683799	0.621513475	-0.338985086	0.734620965	0.999677092
3.6.3.20	sn-glycerol 3-phosphate transport system ATP-binding protein	3.684835706	0.315421678	1.625746648	0.194016502	0.846162961	0.999677092
2.3.1.41	3-oxoacyl-[acyl-carrier-protein] synthase I	47.54071951	-0.54197665	0.790523065	-0.685592456	0.49297014	0.999677092
2.6.1.11	acetylornithine aminotransferase	772.0904338	-0.02206944	0.402142961	-0.054879587	0.956234394	0.999677092
1.3.5.2	dihydroorotate dehydrogenase	19.86044219	0.8060695	0.697778258	1.155194348	0.248010849	0.999677092
2.7.7.19	poly(A) polymerase	26.75217356	-0.543622244	0.540026717	-1.006658054	0.314099126	0.999677092
4.2.1.70	#N/A	44.63206652	0.913714082	0.535362629	1.706719957	0.087874116	0.999677092
1.2.1.20	succinate-semialdehyde dehydrogenase / glutarate-semialdehy	7.836932892	-0.245768772	0.999556003	-0.245877941	0.80577672	0.999677092
4.3.3.6	pyridoxal 5'-phosphate synthase pdxS subunit	93.34197574	0.761840037	0.53082022	1.435212918	0.151226434	0.999677092
1.1.1.373	4-hydroxybutyrate dehydrogenase / sulfolactaldehyde 3-reduct	0.225362151	-0.685009681	4.134065803	-0.165698785	0.868394002	0.999677092
2.1.1.226	23S rRNA (cytidine1920-2'-O)/16S rRNA (cytidine1409-2'-O)-me	253.4357891	0.456149563	0.285385358	1.598363584	0.109962084	0.999677092
5.4.99.16	maltose alpha-D-glucosyltransferase / alpha-amylase	62.92339829	-0.2564613	0.438567274	-0.584770719	0.558701896	0.999677092
3.1.3.67	phosphatidylinositol-3,4,5-trisphosphate 3-phosphatase and du	1.263287807	-0.37043359	4.099037247	-0.090370877	0.927992499	0.999677092
1.3.1.1	dihydropyrimidine dehydrogenase (NAD+) subunit PreT	44.09819686	0.363494046	0.526065055	0.690967861	0.489585739	0.999677092
4.1.2.25	7,8-dihydroneopterin aldolase/epimerase/oxygenase	127.4383164	-0.875431609	0.452638728	-1.934062542	0.053105439	0.999677092
3.6.3.12	K+-transporting ATPase ATPase A chain	176.7916052	-0.389912899	0.750885893	-0.519270509	0.603572116	0.999677092
3.1.1.1	carboxylesterase 1	4.915211358	-0.475667368	0.87849525	-0.541456961	0.588192655	0.999677092
2.3.1.128	#N/A	187.3750258	0.082884684	0.494265922	0.167692491	0.866825203	0.999677092
2.4.1.80	ceramide glucosyltransferase	1.085351351	1.322718578	4.10241703	0.322424212	0.747131344	0.999677092
2.5.1.120	aminodeoxyfutalosine synthase	9.285788668	0.840355257	1.237095668	0.679296904	0.496949758	0.999677092
6.3.3.3	dethiobiotin synthetase	100.4835213	-0.423309165	0.69715936	-0.607191396	0.543723902	0.999677092
3.6.1.11	exopolyphosphatase	149.2278941	-0.296548914	0.367830589	-0.806210584	0.420121437	0.999677092

2.1.1.37	DNA (cytosine-5)-methyltransferase 1	565.5999211	-0.07616931	0.420207431	-0.181265975	0.856158811	0.999677092
2.4.1.287	rhamnopyranosyl-N-acetylglucosaminyl-diphospho-decaprenol	0.231763367	-0.887011995	4.129302346	-0.214809166	0.829916106	0.999677092
4.3.99.3	7-carboxy-7-deazaguanine synthase	14.00860713	-0.433860667	0.81562089	-0.531939131	0.594768154	0.999677092
3.2.2.23	#N/A	78.49287026	-0.099932329	0.70733236	-0.141280585	0.887648287	0.999677092
4.1.99.2	tyrosine phenol-lyase	3.669322862	-0.572117177	2.265906352	-0.25248933	0.80066286	0.999677092
3.1.22.4	crossover junction endodeoxyribonuclease RuvC	139.4664006	-0.230802656	0.272112386	-0.84818872	0.396332878	0.999677092
2.7.8.5	CDP-diacylglycerol---glycerol-3-phosphate 3-phosphatidyltransf	307.8633605	-0.051268604	0.364473371	-0.140664884	0.888134688	0.999677092
1.4.1.21	aspartate dehydrogenase	40.35370295	0.457682622	0.43687187	1.047635826	0.294806425	0.999677092
2.6.1.102	perosamine synthetase	0.34586761	0.565364571	4.090357283	0.138218872	0.89006744	0.999677092
2.9.1.2	O-phospho-L-seryl-tRNASec:L-selenocysteinyl-tRNA synthase	1.325513361	-0.585912273	4.098806609	-0.14294704	0.886332007	0.999677092
3.2.1.177	alpha-D-xyloside xylohydrolase	417.1300843	0.306391788	0.369046157	0.830226199	0.406410905	0.999677092
1.3.1.9	enoyl-acyl-carrier protein] reductase I	380.3676926	-0.127816397	0.324935698	-0.393359049	0.694054311	0.999677092
2.7.1.12	gluconokinase	3.957617601	0.076348152	1.419620656	0.053780672	0.957109909	0.999677092
2.1.1.266	23S rRNA (adenine2030-N6)-methyltransferase	6.928663293	-2.404488106	1.346608308	-1.785588349	0.074165935	0.999677092
4.1.2.4	deoxyribose-phosphate aldolase	536.7825912	0.341366872	0.454458829	0.75115027	0.452562225	0.999677092
3.4.13.21	dipeptidase E	32.5585104	-0.509198831	0.391274755	-1.301384321	0.193126938	0.999677092
1.2.1.70	glutamyl-tRNA reductase	46.88750121	-0.116500157	0.655033869	-0.177853639	0.858837921	0.999677092
2.5.1.10	farnesyl diphosphate synthase	376.183266	0.037313067	0.196801999	0.18959699	0.849624946	0.999677092
2.8.3.20	succinyl-CoA---D-citramalate CoA-transferase	0.051502971	-0.135983389	4.141439557	-0.032834812	0.973806317	0.999677092
2.4.2.28	5'-methylthioadenosine phosphorylase	9.012879735	0.724224162	0.724308057	0.999884172	0.317366565	0.999677092
6.1.1.23	nondiscriminating aspartyl-tRNA synthetase	40.42760406	-0.341970214	0.758076032	-0.451102791	0.651915466	0.999677092
2.7.2.15	propionate kinase	0.232008508	-1.180783451	4.130245682	-0.285886977	0.774964689	0.999677092
6.1.1.20	#N/A	740.7444539	0.063995936	0.193745696	0.330308943	0.741166536	0.999677092
1.8.99.2	#N/A	49.97111568	-0.239226362	0.753142014	-0.317637787	0.750759708	0.999677092
2.1.1.170	16S rRNA (guanine527-N7)-methyltransferase	266.2469813	-0.226239116	0.342739784	-0.660090035	0.509196053	0.999677092
2.4.1.230	kojibiose phosphorylase	16.45124371	-0.277639129	0.982532945	-0.28257488	0.777502739	0.999677092
1.2.7.-	#N/A	4868.982823	0.030423516	0.51415652	0.059171701	0.952815349	0.999677092
1.2.7.11	2-oxoglutarate/2-oxoacid ferredoxin oxidoreductase subunit al	859.9599729	0.168428801	0.287558023	0.585721097	0.558062955	0.999677092
4.1.2.48	threonine aldolase	275.6256696	0.17198508	0.264634062	0.649897744	0.515758276	0.999677092
2.1.1.319	type I protein arginine methyltransferase	5.72190343	-0.296581372	2.588335826	-0.114583807	0.908775015	0.999677092
2.3.2.2	gamma-glutamyltranspeptidase / glutathione hydrolase	38.94457833	-0.468495095	0.757639381	-0.618361593	0.536337009	0.999677092
3.1.2.12	S-formylglutathione hydrolase	0.959060744	-1.047020005	3.654761651	-0.286481064	0.774509695	0.999677092
3.4.15.5	peptidyl-dipeptidase Dcp	237.1508587	-0.097656253	0.419837715	-0.232604766	0.816068326	0.999677092
2.7.14.1	protein arginine kinase	137.5777148	0.249837263	0.648902897	0.385014867	0.7002264	0.999677092
2.1.1.196	cobalt-precorrin-6B (C15)-methyltransferase	2.601572733	-0.283785228	1.683704542	-0.168548116	0.866152094	0.999677092
5.4.2.3	phosphoacetylglucosamine mutase	1.577647769	-0.410734017	4.097161135	-0.100248441	0.920147089	0.999677092
3.5.1.116	ureidoglycolate amidohydrolase	0.244004913	-0.685560993	4.122492473	-0.166297694	0.86792268	0.999677092
2.8.3.25	bile acid CoA-transferase	12.81360026	-0.082327123	0.878071425	-0.093759028	0.925300579	0.999677092
6.5.1.7	DNA ligase 1	6.069130183	-0.528571705	1.508227305	-0.350458915	0.725994319	0.999677092
3.1.1.4	secretory phospholipase A2	2.533868293	-0.613365067	1.908359951	-0.321409526	0.747900065	0.999677092
2.1.1.227	23S rRNA (cytidine1920-2'-O)/16S rRNA (cytidine1409-2'-O)-me	253.4357891	0.456149563	0.285385358	1.598363584	0.109962084	0.999677092
1.5.3.-	#N/A	0.327782895	-0.788631295	4.134426062	-0.190747466	0.848723449	0.999677092
1.4.5.1	D-amino-acid dehydrogenase	4.746604651	1.173189192	1.629981543	0.719756121	0.471675166	0.999677092
1.17.1.4	xanthine dehydrogenase molybdenum-binding subunit	365.9000832	-0.181318938	0.547565016	-0.33113682	0.74054114	0.999677092
1.1.1.108	carnitine 3-dehydrogenase	0.055503779	-0.375985817	4.137674506	-0.090868872	0.927596784	0.999677092
3.4.11.10	bacterial leucyl aminopeptidase	12.58645714	-1.106351009	1.403234013	-0.788429441	0.430445555	0.999677092
5.3.1.14	L-rhamnose isomerase	106.3100671	-0.021461517	0.308121129	-0.069652859	0.944469964	0.999677092
1.1.1.267	1-deoxy-D-xylulose-5-phosphate reductoisomerase	352.3064475	0.059381425	0.160005611	0.371120891	0.710547492	0.999677092
2.3.1.79	maltose O-acetyltransferase	191.1526115	-0.001594935	0.356202144	-0.004477612	0.996427394	0.999677092
2.3.1.82	aminoglycoside 6'-N-acetyltransferase	9.056722013	-0.071102172	0.628897867	-0.113058377	0.909984273	0.999677092
3.1.4.52	c-di-GMP phosphodiesterase	11.05301254	-0.981807686	0.840716961	-1.167821908	0.242878609	0.999677092
2.5.1.97	pseudaminic acid synthase	14.79377256	-0.038899688	0.929865339	-0.041833679	0.966631286	0.999677092
2.8.2.22	arylsulfate sulfotransferase	22.28707327	-0.873252661	0.757939387	-1.152140495	0.249263345	0.999677092
3.5.4.26	diaminohydroxyphosphoribosylaminopyrimidine deaminase	147.8638147	-0.057169555	0.365483559	-0.156421686	0.875700645	0.999677092
2.7.6.3	2-amino-4-hydroxy-6-hydroxymethyldihydropteridine diphosph	195.0628172	-0.606485941	0.428484301	-1.415421615	0.156944916	0.999677092
5.1.3.22	L-ribulose-5-phosphate 3-epimerase	8.998605211	-0.784142839	0.902136337	-0.869206578	0.38473415	0.999677092
5.99.1.2	#N/A	1392.585077	-0.219075863	0.322804434	-0.678664354	0.497350557	0.999677092
2.1.1.90	methanol---5-hydroxybenzimidazolylcobamide Co-methyltransf	3.288051399	-0.414397456	2.550730747	-0.16246225	0.870941854	0.999677092
3.4.19.11	g-D-glutamyl-meso-diaminopimelate peptidase	119.8936844	-1.247677881	0.835386546	-1.493533606	0.135297564	0.999677092
4.2.1.22	cystathionine beta-synthase	0.792135483	0.154563955	4.048931713	0.038174009	0.969548944	0.999677092
2.4.99.18	dolichyl-diphosphooligosaccharide---protein glycosyltransferase	10.80229488	1.488745272	1.504546434	0.989497724	0.322419683	0.999677092
2.6.1.110	dTDP-4-dehydro-2,3,6-trideoxy-D-glucose 4-aminotransferase	5.438875754	0.257716636	2.360309072	0.109187665	0.913053644	0.999677092
2.7.1.201	PTS system, trehalose-specific IIA component	10.53537854	-0.348438615	1.00179233	-0.347815216	0.727978962	0.999677092
1.1.1.57	fructuronate reductase	266.1380159	0.058723052	0.471141324	0.124639995	0.900808564	0.999677092
3.5.1.-	#N/A	3277.707675	-0.734623963	1.051616325	-0.698566526	0.484822969	0.999677092
1.7.1.7	GMP reductase	8.098065539	0.101425165	1.185283272	0.085570401	0.931807928	0.999677092
2.7.1.23	NAD+ kinase	351.0343927	0.235413875	0.213002285	1.105217602	0.269065312	0.999677092
1.4.1.1	alanine dehydrogenase	202.9207115	0.156789346	0.346004122	0.453143	0.650445774	0.999677092
2.7.1.137	phosphatidylinositol 3-kinase	3.121209164	-0.289663116	3.004320475	-0.096415519	0.923190568	0.999677092
2.4.1.15	trehalose 6-phosphate synthase	39.99829842	-0.389128513	0.595447001	-0.653506546	0.513429771	0.999677092
2.7.7.-	#N/A	737.2154561	0.073720862	0.252535598	0.291922653	0.770345763	0.999677092
2.4.99.15	3-deoxy-D-manno-octulosonic-acid transferase	104.6062867	0.109755847	0.47516922	0.230982653	0.817328279	0.999677092
1.17.7.3	(E)-4-hydroxy-3-methylbut-2-enyl-diphosphate synthase	363.237913	-0.050042854	0.164822796	-0.303616095	0.761420388	0.999677092
5.4.99.25	tRNA pseudouridine55 synthase	354.9260802	0.065143163	0.20126595	0.323667081	0.746190096	0.999677092
2.7.1.163	hygromycin-B 4-O-kinase	4.223768401	1.564544181	1.893288989	0.826363112	0.408598142	0.999677092
3.6.1.9	ectonucleotide pyrophosphatase/phosphodiesterase family me	8.082410966	-0.079078924	0.968039657	-0.081689756	0.934893424	0.999677092
2.5.1.101	N,N'-diacetyllegionaminate synthase	17.87356951	-1.038726039	0.721636253	-1.439403903	0.150036119	0.999677092
2.7.7.76	molybdenum cofactor cytidylyltransferase	3243.651555	1.836466395	1.041478757	1.763325831	0.077845551	0.999677092
3.2.2.27	uracil-DNA glycosylase	212.7511443	0.56739259	0.586257497	0.967821466	0.333133541	0.999677092
1.1.1.308	sulfopropanediol 3-dehydrogenase	0.443584731	-2.012479562	4.113106867	-0.48928453	0.624640272	0.999677092
2.3.3.1	citrate synthase	968.5318437	0.112459447	0.31030189	0.362419473	0.71703859	0.999677092
3.1.26.11	ribonuclease Z	244.95852	-0.551460366	0.302803614	-1.821181585	0.068579259	0.999677092
1.2.1.38	N-acetyl-gamma-glutamyl-phosphate reductase	189.9986926	-0.038723223	0.328952042	-0.117716924	0.906291956	0.999677092
2.3.1.245	3-hydroxy-5-phosphonooxypentane-2,4-dione thiolase	47.6858419	-0.287065102	0.690787772	-0.415561934	0.677730586	0.999677092
3.1.25.-	#N/A	0.802665179	-1.700366076	4.109993876	-0.41371499	0.679082848	0.999677092
6.3.4.10	biotin---protein ligase	1.456275519	-0.290724248	4.097533312	-0.07095104	0.943436722	0.999677092
4.1.2.52	4-hydroxy-2-oxoheptanedioate aldolase	7.602420736	-1.080745683	1.146229915	-0.942869898	0.345747454	0.999677092

3.4.17.4	Gly-Xaa carboxypeptidase	0.887846555	-1.45643044	4.100702437	-0.355166087	0.722465169	0.999677092
3.1.1.84	carboxylesterase 2	0.167196626	-3.703195448	4.143731151	-0.893686225	0.371489798	0.999677092
2.7.1.105	6-phosphofructo-2-kinase	6.021524969	-0.25663573	2.464720432	-0.104123667	0.917071209	0.999677092
5.1.2.3	3-hydroxyacyl-CoA dehydrogenase / enoyl-CoA hydratase / 3-h	1.265356629	-1.10364617	4.097808242	-0.269325968	0.787678851	0.999677092
2.5.1.49	O-acetylhomoserine (thiol)-lyase	646.0339653	-0.058335322	0.223745261	-0.260722044	0.794306866	0.999677092
4.2.1.2	#N/A	775.0896236	0.192229807	0.233272083	0.824058346	0.409906406	0.999677092
3.11.1.1	phosphonoacetaldehyde hydrolase	29.24177546	-0.838540921	0.727731807	-1.152266417	0.249211613	0.999677092
1.8.98.1	#N/A	299.754454	-0.184223766	0.481078602	-0.382939016	0.701764982	0.999677092
3.4.11.4	tripeptide aminopeptidase	303.3330781	0.273320011	0.239024527	1.143481025	0.252838928	0.999677092
1.5.3.19	4-methylaminobutanoate oxidase (formaldehyde-forming)	0.213055426	2.039247922	4.123748771	0.494513132	0.620943834	0.999677092
2.5.1.60	geranylgeranyl transferase type-2 subunit beta	0.326417885	-0.17294237	4.12434128	-0.041932119	0.966552811	0.999677092
2.7.1.193	PTS system, N-acetylglucosamine-specific IIA component	552.7318893	-0.135087894	0.48251155	-0.279968208	0.779501896	0.999677092
2.7.1.95	kanamycin kinase	7.098706671	-0.951358347	1.254634437	-0.758275334	0.44828617	0.999677092
3.4.21.72	IgA-specific serine endopeptidase	1.260296949	1.970816436	4.102796929	0.480359245	0.630971968	0.999677092
4.1.1.22	histidine decarboxylase	0.995281257	-0.111837994	4.081294702	-0.027402577	0.978138642	0.999677092
4.1.1.35	UDP-glucuronate decarboxylase	85.12043626	0.284696263	0.427189558	0.666440126	0.505129822	0.999677092
4.2.1.12	phosphogluconate dehydratase	0.449882367	-1.10482823	4.109574673	-0.268842476	0.788050905	0.999677092
3.4.11.9	Xaa-Pro aminopeptidase	736.9519013	0.115688832	0.210706599	0.549051775	0.582969919	0.999677092
4.1.2.-	#N/A	5.460202902	-2.536084746	1.490815485	-1.701139257	0.088916841	0.999677092
3.2.2.-	#N/A	1975.209521	-0.686311816	1.056592043	-0.649552323	0.515981438	0.999677092
1.1.1.133	dTDP-4-dehydrorhamnose reductase	378.8196254	-0.124515968	0.216848026	-0.574208447	0.565826754	0.999677092
2.6.99.2	pyridoxine 5-phosphate synthase	56.3305921	-0.860812054	0.519195768	-1.657972018	0.097323114	0.999677092
1.2.1.79	succinate-semialdehyde dehydrogenase / glutarate-semialdehy	7.836932892	-0.245768772	0.999556003	-0.245877941	0.80577672	0.999677092
1.2.7.4	#N/A	586.3369208	-0.355666697	0.495466044	-0.717842728	0.472854264	0.999677092
2.5.1.75	tRNA dimethylallyltransferase	650.812732	-0.853063731	0.699910417	-1.218818452	0.222913107	0.999677092
4.2.3.1	threonine synthase	413.1252022	-0.129776992	0.189508716	-0.684807511	0.493465395	0.999677092
2.7.11.21	polo-like kinase 1	1.054442185	0.259472635	4.099536942	0.063293157	0.949533065	0.999677092
1.3.1.14	dihydroorotate dehydrogenase (NAD+) catalytic subunit	331.264359	-0.086202304	0.225739017	-0.381867102	0.702559943	0.999677092
1.1.1.290	erythronate-4-phosphate dehydrogenase	61.10219299	-0.366466082	0.563732104	-0.650071337	0.515646143	0.999677092
4.2.1.79	2-methylcitrate dehydratase	0.764996219	0.88112855	4.109393438	0.214418153	0.830220987	0.999677092
2.3.1.51	1-acyl-sn-glycerol-3-phosphate acyltransferase	441.3538744	-0.075740409	0.317780229	-0.238342105	0.811615767	0.999677092
3.5.2.3	dihydroorotase	378.9687693	-0.044536605	0.168909288	-0.263671734	0.792032878	0.999677092
2.1.1.250	trimethylamine---corrinoid protein Co-methyltransferase	3.364886548	0.982205915	1.705036765	0.576061429	0.564573661	0.999677092
2.7.1.180	FAD:protein FMN transferase	243.1625185	0.206219697	0.34984563	0.589459119	0.555553328	0.999677092
1.6.1.2	H+-translocating NAD(P) transhydrogenase	7.406383389	-0.251568587	1.476343725	-0.17039974	0.864695777	0.999677092
3.2.1.50	alpha-N-acetylglucosaminidase	117.1078347	-0.477498987	0.478525032	-0.997855818	0.318349279	0.999677092
2.1.1.216	tRNA (guanine26-N2/guanine27-N2)-dimethyltransferase	2.22570527	0.021617515	3.267377318	0.006616167	0.994721101	0.999677092
2.4.2.4	thymidine phosphorylase	3.167265103	-0.12912262	1.986891665	-0.064987247	0.948184154	0.999677092
6.1.1.16	#N/A	481.2742586	-0.050292963	0.133753653	-0.376011882	0.706908062	0.999677092
6.3.4.6	urea carboxylase	2.097824574	1.600622719	2.970166714	0.538899959	0.589955886	0.999677092
1.1.1.193	5-amino-6-(5-phosphoribosylamino)uracil reductase	148.3103672	-0.051907162	0.366423835	-0.141658804	0.887349516	0.999677092
6.3.2.43	[lysine-biosynthesis-protein LysW]---L-2-aminoadipate ligase	0.208936523	0.44852495	4.143731151	0.108241808	0.913803882	0.999677092
6.3.4.19	tRNA(Ile)-lysidine synthase	409.2291933	0.020379815	0.163559262	0.124602026	0.900838624	0.999677092
3.6.3.15	#N/A	673.0695012	0.022972198	0.307214006	0.074775882	0.940393031	0.999677092
6.3.2.38	N2-citryl-N6-acetyl-N6-hydroxylysine synthase	1.063733801	-0.595839861	4.100593652	-0.145305756	0.88446947	0.999677092
2.7.7.66	phosphoribosyl-dephospho-CoA transferase	1.683890448	2.144545571	2.741148479	0.78235294	0.434007183	0.999677092
2.3.1.251	lipid IVA palmitoyltransferase	1.352951646	1.071917033	2.890030935	0.370901577	0.71071084	0.999677092
2.1.1.201	demethylmenaquinone methyltransferase / 2-methoxy-6-polyp	61.69477923	-0.2233348	0.466166708	-0.479087838	0.631876141	0.999677092
3.5.4.4	adenosine deaminase	45.49932187	-0.238298856	0.526870977	-0.452290725	0.651059557	0.999677092
1.2.1.11	aspartate-semialdehyde dehydrogenase	340.0143125	-0.200121399	0.18251466	-1.096467535	0.272874223	0.999677092
4.1.3.16	#N/A	1.127391998	0.966587108	4.094550529	0.236066719	0.813380899	0.999677092
3.2.1.55	alpha-N-arabinofuranosidase	371.5198309	-0.148207307	0.517639743	-0.286313617	0.774637929	0.999677092
1.1.1.215	glyoxylate/hydroxypyruvate/2-ketogluconate reductase	0.185410644	-0.336372923	4.140615101	-0.081237429	0.935253134	0.999677092
4.2.1.114	methanogen homoaconitase large subunit	2.400786458	1.170528298	4.068056973	0.287736456	0.773548481	0.999677092
2.7.9.1	pyruvate, orthophosphate dikinase	3849.975539	-0.509751212	0.555553095	-0.917556245	0.358851234	0.999677092
6.2.1.14	6-carboxyhexanoate--CoA ligase	3.108126578	-0.102222901	1.781372224	-0.057384358	0.954239023	0.999677092
2.3.1.179	3-oxoacyl-[acyl-carrier-protein] synthase II	483.3914302	-0.220756395	0.307158875	-0.71870427	0.472323152	0.999677092
2.3.1.203	UDP-N-acetylbacillosamine N-acetyltransferase	3.040640095	-1.528358815	1.729450802	-0.883724945	0.376844711	0.999677092
2.4.2.12	nicotinamide phosphoribosyltransferase	8.025752757	-0.033172202	1.097372849	-0.030228743	0.975884625	0.999677092
3.6.1.22	NAD+ diphosphatase	128.9123178	-0.516406104	0.444548	-1.161643072	0.245380479	0.999677092
3.2.1.70	glucan 1,6-alpha-glucosidase	19.62187726	-0.098905083	0.653503571	-0.151345896	0.879702867	0.999677092
2.1.4.1	glycine amidinotransferase	23.72075067	-0.216439065	0.696020685	-0.310966426	0.755826145	0.999677092
3.3.1.1	adenosylhomocysteinase	99.61581088	0.067257503	0.291811127	0.230482996	0.817716475	0.999677092
2.7.1.140	inositol-polyphosphate multikinase	2.104401683	-0.145249648	4.096480433	-0.035457181	0.97171519	0.999677092
2.3.1.231	tRNA wybutosine-synthesizing protein 4	0.052512875	-0.212523182	4.139416356	-0.05134134	0.959053527	0.999677092
2.1.1.133	precorrin-4/cobalt-precorrin-4 C11-methyltransferase	98.82307915	-0.216409953	0.365395589	-0.592262085	0.553675101	0.999677092
1.1.1.338	(2R)-3-sulfolactate dehydrogenase (NADP+)	0.413530944	3.521515447	4.059461483	0.867483399	0.385677204	0.999677092
1.1.1.346	2,5-diketo-D-gluconate reductase A	4.277529261	-1.628978452	1.415184877	-1.151071127	0.249702975	0.999677092
6.1.1.3	threonyl-tRNA synthetase	879.1230179	0.71274957	0.351927306	2.025274985	0.042839136	0.999677092
6.3.2.6	phosphoribosylaminoimidazole carboxylase / phosphoribosylan	355.7877861	-0.281389413	0.2282892	-1.232600636	0.2177248	0.999677092
1.2.1.88	1-pyrroline-5-carboxylate dehydrogenase	87.99903725	0.148854377	0.39591705	0.375973646	0.706936488	0.999677092
3.4.16.4	D-alanyl-D-alanine carboxypeptidase	6876.188992	0.181863199	0.75684457	0.240291345	0.810104405	0.999677092
2.8.3.18	succinyl-CoA:acetate CoA-transferase	16.37592351	0.269344993	0.824725907	0.326587283	0.743980063	0.999677092
3.-.-.-	#N/A	7.254996096	-0.311436754	1.16636355	-0.267015163	0.789457486	0.999677092
3.5.1.89	N-acetylglucosaminylphosphatidylinositol deacetylase	0.083257956	0.021491745	4.141091794	0.005189874	0.995859098	0.999677092
4.2.1.45	CDP-glucose 4,6-dehydratase	62.0299926	0.272414816	0.422141177	0.645316854	0.518721873	0.999677092
3.5.4.33	tRNA(adenine34) deaminase	428.6671155	1.194427813	0.569226505	2.098334848	0.035875576	0.999677092
3.1.3.83	D-glycero-D-manno-heptose 1,7-bisphosphate phosphatase	27.20891321	-0.354945311	0.546383902	-0.649626224	0.515933689	0.999677092
2.7.2.2	carbamate kinase	196.6123808	-0.27435373	0.348887924	-0.786366368	0.431652881	0.999677092
3.1.2.1	acetyl-CoA hydrolase	33.55176493	4.23E-05	2.134986791	1.98E-05	0.999984202	0.999984202
6.3.2.26	N-(5-amino-5-carboxypentanoyl)-L-cysteinyl-D-valine synthase	0	NA	NA	NA	NA	NA
1.14.19.3	linoleoyl-CoA desaturase	0	NA	NA	NA	NA	NA
2.3.1.37	5-aminolevulinate synthase	0	NA	NA	NA	NA	NA
3.1.3.99	IMP and pyridine-specific 5'-nucleotidase	0	NA	NA	NA	NA	NA
1.3.99.32	glutaryl-CoA dehydrogenase (non-decarboxylating)	0	NA	NA	NA	NA	NA
2.4.1.58	UDP-glucose:(galactosyl)LPS alpha-1,2-glucosyltransferase	0	NA	NA	NA	NA	NA
4.1.1.45	aminocarboxymuconate-semialdehyde decarboxylase	0	NA	NA	NA	NA	NA

1.14.13.89	#N/A	0	NA	NA	NA	NA	NA
1.12.1.2	NAD-reducing hydrogenase large subunit	0	NA	NA	NA	NA	NA
5.4.3.-	#N/A	0	NA	NA	NA	NA	NA
2.7.1.182	phytol kinase	0	NA	NA	NA	NA	NA
1.1.1.53	3alpha(or 20beta)-hydroxysteroid dehydrogenase	0	NA	NA	NA	NA	NA
2.4.1.161	oligosaccharide 4-alpha-D-glucosyltransferase	0	NA	NA	NA	NA	NA
2.1.1.71	phosphatidyl-N-methylethanolamine N-methyltransferase	0	NA	NA	NA	NA	NA
3.1.3.78	phosphatidylinositol-4,5-bisphosphate 4-phosphatase	0	NA	NA	NA	NA	NA
1.5.1.21	delta1-piperideine-2-carboxylate reductase	0	NA	NA	NA	NA	NA
3.2.1.15	polygalacturonase	0	NA	NA	NA	NA	NA
2.7.1.166	3-deoxy-D-manno-octulosonic acid kinase	0	NA	NA	NA	NA	NA
4.1.1.7	benzoylformate decarboxylase	0	NA	NA	NA	NA	NA
3.4.23.40	phytepsin	0	NA	NA	NA	NA	NA
1.13.11.3	#N/A	0	NA	NA	NA	NA	NA
2.5.1.85	all-trans-nonaprenyl-diphosphate synthase	0	NA	NA	NA	NA	NA
2.7.10.2	protein-tyrosine kinase	0	NA	NA	NA	NA	NA
3.5.3.17	guanidinopropionase	0	NA	NA	NA	NA	NA
1.16.1.10	ferric-chelate reductase [NAD(P)H]	0	NA	NA	NA	NA	NA
2.4.1.318	demethyllactenocin mycarosyltransferase	0	NA	NA	NA	NA	NA
3.1.27.1	ribonuclease T2	0	NA	NA	NA	NA	NA
3.7.1.17	4,5:9,10-diseco-3-hydroxy-5,9,17-trioxoandrosta-1(10),2-diene-	0	NA	NA	NA	NA	NA
1.3.5.6	zeta-carotene desaturase	0	NA	NA	NA	NA	NA
2.6.1.108	(5-formylfuran-3-yl)methyl phosphate transaminase	0	NA	NA	NA	NA	NA
1.1.1.197	carbonyl reductase 1	0	NA	NA	NA	NA	NA
1.14.18.6	4-hydroxysphinganine ceramide fatty acyl 2-hydroxylase	0	NA	NA	NA	NA	NA
1.1.1.252	tetrahydroxynaphthalene reductase	0	NA	NA	NA	NA	NA
1.3.99.28	phytoene desaturase	0	NA	NA	NA	NA	NA
3.1.4.37	2',3'-cyclic-nucleotide 3'-phosphodiesterase	0	NA	NA	NA	NA	NA
1.1.1.184	carbonyl reductase 1	0	NA	NA	NA	NA	NA
2.4.1.109	dolichyl-phosphate-mannose-protein mannosyltransferase	0	NA	NA	NA	NA	NA
1.3.1.33	protochlorophyllide reductase	0	NA	NA	NA	NA	NA
3.4.17.22	carboxypeptidase D	0	NA	NA	NA	NA	NA
1.3.1.85	crotonyl-CoA carboxylase/reductase	0	NA	NA	NA	NA	NA
3.2.2.16	5'-methylthioadenosine nucleosidase	0	NA	NA	NA	NA	NA
1.3.99.23	all-trans-retinol 13,14-reductase	0	NA	NA	NA	NA	NA
1.1.1.292	1,5-anhydro-D-fructose reductase (1,5-anhydro-D-mannitol-for	0	NA	NA	NA	NA	NA
1.13.11.53	1,2-dihydroxy-3-keto-5-methylthiopentene dioxygenase	0	NA	NA	NA	NA	NA
3.4.24.75	lysostaphin	0	NA	NA	NA	NA	NA
1.3.1.-	#N/A	0	NA	NA	NA	NA	NA
2.5.1.92	(2Z,6Z)-farnesyl diphosphate synthase	0	NA	NA	NA	NA	NA
2.5.1.68	short-chain Z-isoprenyl diphosphate synthase	0	NA	NA	NA	NA	NA
3.4.14.12	Xaa-Xaa-Pro tripeptidyl-peptidase	0	NA	NA	NA	NA	NA
2.4.1.292	GalNAc-alpha-(1->4)-GalNAc-alpha-(1->3)-diNAcBac-PP-undeca	0	NA	NA	NA	NA	NA
2.7.1.215	erythritol kinase (D-erythritol 1-phosphate-forming)	0	NA	NA	NA	NA	NA
4.1.1.51	3-hydroxy-2-methylpyridine-4,5-dicarboxylate 4-decarboxylase	0	NA	NA	NA	NA	NA
2.7.1.74	deoxycitidine kinase	0	NA	NA	NA	NA	NA
1.10.3.11	ubiquinol oxidase	0	NA	NA	NA	NA	NA
2.4.1.60	abequosyltransferase	0	NA	NA	NA	NA	NA
5.3.3.10	5-carboxymethyl-2-hydroxymuconate isomerase	0	NA	NA	NA	NA	NA
2.7.1.161	riboflavin kinase, archaea type	0	NA	NA	NA	NA	NA
5.1.3.24	N-acetylneuraminate epimerase	0	NA	NA	NA	NA	NA
1.3.7.9	4-hydroxybenzoyl-CoA reductase subunit gamma	0	NA	NA	NA	NA	NA
2.7.8.23	carboxyvinyl-carboxyphosphonate phosphorylmutase	0	NA	NA	NA	NA	NA
6.2.1.16	acetoacetyl-CoA synthetase	0	NA	NA	NA	NA	NA
1.3.99.29	phytoene desaturase	0	NA	NA	NA	NA	NA
5.1.3.37	mannuronan 5-epimerase	0	NA	NA	NA	NA	NA
1.14.11.17	taurine dioxygenase	0	NA	NA	NA	NA	NA
1.10.2.-	#N/A	0	NA	NA	NA	NA	NA
1.14.17.3	peptidylglycine monooxygenase	0	NA	NA	NA	NA	NA
4.3.1.24	phenylalanine ammonia-lyase	0	NA	NA	NA	NA	NA
2.7.1.138	ceramide kinase	0	NA	NA	NA	NA	NA
1.8.7.1	sulfite reductase (ferredoxin)	0	NA	NA	NA	NA	NA
1.1.1.396	bacteriochlorophyllide a dehydrogenase	0	NA	NA	NA	NA	NA
3.6.3.6	#N/A	0	NA	NA	NA	NA	NA
2.5.1.131	(4-(4-[2-(gamma-L-glutamylamino)ethyl]phenoxymethyl)furan-	0	NA	NA	NA	NA	NA
4.1.3.25	malyl-CoA/(S)-citramalyl-CoA lyase	0	NA	NA	NA	NA	NA
2.5.1.44	homospermidine synthase	0	NA	NA	NA	NA	NA
2.7.1.86	NADH kinase	0	NA	NA	NA	NA	NA
1.13.99.1	inositol oxygenase	0	NA	NA	NA	NA	NA
1.5.1.33	pteridine reductase	0	NA	NA	NA	NA	NA
3.1.3.77	enolase-phosphatase E1	0	NA	NA	NA	NA	NA
1.1.99.21	D-sorbitol dehydrogenase (acceptor)	0	NA	NA	NA	NA	NA
4.1.1.79	sulfopyruvate decarboxylase subunit alpha	0	NA	NA	NA	NA	NA
3.6.1.61	diadenosine hexaphosphate hydrolase (ATP-forming)	0	NA	NA	NA	NA	NA
2.7.7.68	2-phospho-L-lactate guanylyltransferase	0	NA	NA	NA	NA	NA
1.1.1.385	dihydroanticapsin dehydrogenase	0	NA	NA	NA	NA	NA
3.2.1.67	galacturan 1,4-alpha-galacturonidase	0	NA	NA	NA	NA	NA
6.2.1.41	HIP---CoA ligase	0	NA	NA	NA	NA	NA
5.1.1.17	isopenicillin-N epimerase	0	NA	NA	NA	NA	NA
4.1.2.29	6-phospho-5-dehydro-2-deoxy-D-gluconate aldolase	0	NA	NA	NA	NA	NA
2.4.1.289	N-acetylglucosaminyl-diphospho-decaprenol L-rhamnosyltransf	0	NA	NA	NA	NA	NA
4.2.1.140	gluconate/galactonate dehydratase	0	NA	NA	NA	NA	NA
2.7.4.12	T-even-induced deoxynucleotide kinase	0	NA	NA	NA	NA	NA
1.1.1.117	D-arabinose 1-dehydrogenase	0	NA	NA	NA	NA	NA
1.14.99.46	pyrimidine oxygenase	0	NA	NA	NA	NA	NA
2.3.2.8	arginyl-tRNA---protein transferase	0	NA	NA	NA	NA	NA
1.1.1.371	scyllo-inositol 2-dehydrogenase (NADP+)	0	NA	NA	NA	NA	NA

1.1.1.378	L-rhamnose 1-dehydrogenase	0	NA	NA	NA	NA	NA
2.1.1.179	16S rRNA (guanine(1405)-N(7))-methyltransferase	0	NA	NA	NA	NA	NA
1.1.1.377	L-rhamnose 1-dehydrogenase	0	NA	NA	NA	NA	NA
1.1.1.244	methanol dehydrogenase	0	NA	NA	NA	NA	NA
3.4.17.14	zinc D-Ala-D-Ala carboxypeptidase	0	NA	NA	NA	NA	NA
3.4.15.6	cyanophycinase	0	NA	NA	NA	NA	NA
3.1.1.24	3-oxoadipate enol-lactonase	0	NA	NA	NA	NA	NA
3.5.1.97	acyl-homoserine-lactone acylase	0	NA	NA	NA	NA	NA
1.3.1.6	NADH-dependent fumarate reductase subunit A	0	NA	NA	NA	NA	NA
2.8.3.6	#N/A	0	NA	NA	NA	NA	NA
5.1.2.2	mandelate racemase	0	NA	NA	NA	NA	NA
1.1.1.275	(+)-trans-carveol dehydrogenase	0	NA	NA	NA	NA	NA
1.1.1.189	carbonyl reductase 1	0	NA	NA	NA	NA	NA
5.1.99.6	NAD(P)H-hydrate epimerase	0	NA	NA	NA	NA	NA
3.7.1.14	2-hydroxy-6-oxonona-2,4-dienedioate hydrolase	0	NA	NA	NA	NA	NA
2.7.7.93	phosphonoformate cytidylyltransferase	0	NA	NA	NA	NA	NA
3.4.24.71	endothelin-converting enzyme	0	NA	NA	NA	NA	NA
2.5.1.42	geranylgeranylglycerol-phosphate geranylgeranyltransferase	0	NA	NA	NA	NA	NA
2.4.1.34	1,3-beta-glucan synthase	0	NA	NA	NA	NA	NA
1.2.1.60	5-carboxymethyl-2-hydroxymuconic-semialdehyde dehydrogen	0	NA	NA	NA	NA	NA
1.3.99.26	phytoene desaturase	0	NA	NA	NA	NA	NA
3.6.1.12	dCTP diphosphatase	0	NA	NA	NA	NA	NA
2.4.1.173	sterol 3beta-glucosyltransferase	0	NA	NA	NA	NA	NA
2.6.1.106	dTDP-3-amino-3,4,6-trideoxy-alpha-D-glucose transaminase	0	NA	NA	NA	NA	NA
1.1.1.173	L-rhamnose 1-dehydrogenase	0	NA	NA	NA	NA	NA
3.1.3.37	fructose-1,6-bisphosphatase I / sedoheptulose-1,7-bisphosphat	0	NA	NA	NA	NA	NA
2.3.1.4	glucosamine-phosphate N-acetyltransferase	0	NA	NA	NA	NA	NA
3.2.1.46	galactosylceramidase	0	NA	NA	NA	NA	NA
3.4.11.24	aminopeptidase S	0	NA	NA	NA	NA	NA
2.4.1.290	N,N'-diacetylbacillosaminyl-diphospho-undecaprenol alpha-1,3-	0	NA	NA	NA	NA	NA
3.1.11.3	exodeoxyribonuclease (lambda-induced)	0	NA	NA	NA	NA	NA
5.1.3.5	UDP-arabinose 4-epimerase	0	NA	NA	NA	NA	NA
1.1.1.21	aldehyde reductase	0	NA	NA	NA	NA	NA
3.5.4.34	tRNA-specific adenosine deaminase 1	0	NA	NA	NA	NA	NA
3.1.1.75	poly(3-hydroxybutyrate) depolymerase	0	NA	NA	NA	NA	NA
2.3.1.86	fatty acid synthase subunit alpha, fungi type	0	NA	NA	NA	NA	NA
3.4.21.68	tissue plasminogen activator	0	NA	NA	NA	NA	NA
6.2.1.17	propionyl-CoA synthetase	0	NA	NA	NA	NA	NA
6.3.4.24	tyramine---L-glutamate ligase	0	NA	NA	NA	NA	NA
4.2.1.161	bisanhydrobacterioruberin hydratase	0	NA	NA	NA	NA	NA
3.4.22.49	separase	0	NA	NA	NA	NA	NA
4.4.1.19	phosphosulfolactate synthase	0	NA	NA	NA	NA	NA
1.3.99.16	isoquinoline 1-oxidoreductase	0	NA	NA	NA	NA	NA
4.4.1.2	cystathionine gamma-lyase / homocysteine desulfhydrase	0	NA	NA	NA	NA	NA
3.1.21.7	deoxyribonuclease V	0	NA	NA	NA	NA	NA
2.5.1.59	protein farnesyltransferase/geranylgeranyltransferase type-1 s	0	NA	NA	NA	NA	NA
2.5.1.84	all-trans-nonaprenyl-diphosphate synthase	0	NA	NA	NA	NA	NA
2.1.1.17	phosphatidylethanolamine/phosphatidyl-N-methylethanolamir	0	NA	NA	NA	NA	NA
3.5.4.30	dCTP deaminase (dUMP-forming)	0	NA	NA	NA	NA	NA
1.3.99.31	phytoene desaturase	0	NA	NA	NA	NA	NA
2.5.1.43	nicotianamine synthase	0	NA	NA	NA	NA	NA
3.1.3.87	2-hydroxy-3-keto-5-methylthiopentenyl-1-phosphate phosphat	0	NA	NA	NA	NA	NA
3.4.21.50	lysyl endopeptidase	0	NA	NA	NA	NA	NA
3.5.2.9	5-oxoprolinase (ATP-hydrolysing)	0	NA	NA	NA	NA	NA
1.1.1.51	aldo-keto reductase family 1 member C3	0	NA	NA	NA	NA	NA
1.2.1.71	succinylglutamic semialdehyde dehydrogenase	0	NA	NA	NA	NA	NA
5.1.99.7	D-erythro-7,8-dihydroneopterin triphosphate epimerase	0	NA	NA	NA	NA	NA
3.4.24.27	thermolysin	0	NA	NA	NA	NA	NA
4.2.1.41	5-dehydro-4-deoxyglucarate dehydratase	0	NA	NA	NA	NA	NA
1.3.7.7	light-independent protochlorophyllide reductase subunit L	0	NA	NA	NA	NA	NA
3.4.24.25	vibriolysin	0	NA	NA	NA	NA	NA
1.14.13.1	salicylate hydroxylase	0	NA	NA	NA	NA	NA
6.2.1.42	3-oxocholest-4-en-26-oate---CoA ligase	0	NA	NA	NA	NA	NA
1.3.1.83	geranylgeranyl diphosphate/geranylgeranyl-bacteriochlorophyl	0	NA	NA	NA	NA	NA
1.1.1.43	phosphogluconate 2-dehydrogenase	0	NA	NA	NA	NA	NA
3.2.1.2	beta-amylase	0	NA	NA	NA	NA	NA
2.4.1.87	N-acetyllactosaminide 3-alpha-galactosyltransferase	0	NA	NA	NA	NA	NA
3.7.1.8	2,6-dioxo-6-phenylhexa-3-enoate hydrolase	0	NA	NA	NA	NA	NA
1.8.-.-	#N/A	0	NA	NA	NA	NA	NA
3.1.3.21	glycerol-1-phosphatase	0	NA	NA	NA	NA	NA
2.3.1.168	2-oxoisovalerate dehydrogenase E2 component (dihydrolipoyl t	0	NA	NA	NA	NA	NA
3.4.23.52	archaeal preflagellin peptidase FlaK	0	NA	NA	NA	NA	NA
2.7.1.3	ketohexokinase	0	NA	NA	NA	NA	NA
1.8.2.3	sulfide dehydrogenase [flavocytochrome c] flavoprotein chain	0	NA	NA	NA	NA	NA
2.3.1.109	arginine N-succinyltransferase	0	NA	NA	NA	NA	NA
2.3.2.16	peptidoglycan pentaglycine glycine transferase (the first glycine	0	NA	NA	NA	NA	NA
2.7.1.76	deoxyadenosine kinase	0	NA	NA	NA	NA	NA
1.13.11.16	2,3-dihydroxyphenylpropionate 1,2-dioxygenase	0	NA	NA	NA	NA	NA
4.1.1.77	2-oxo-3-hexenedioate decarboxylase	0	NA	NA	NA	NA	NA
1.3.8.-	#N/A	0	NA	NA	NA	NA	NA
2.1.1.152	precorrin-6A synthase	0	NA	NA	NA	NA	NA
1.20.4.3	mycoredoxin	0	NA	NA	NA	NA	NA
1.2.1.31	L-aminoadipate-semialdehyde dehydrogenase	0	NA	NA	NA	NA	NA
6.3.4.22	tRNA(Ile2)-agmatinylcytidine synthase	0	NA	NA	NA	NA	NA
1.3.5.5	15-cis-phytoene desaturase	0	NA	NA	NA	NA	NA
4.2.1.25	L-arabonate dehydrase	0	NA	NA	NA	NA	NA
1.2.99.8	glyceraldehyde dehydrogenase large subunit	0	NA	NA	NA	NA	NA

1.13.11.54	1,2-dihydroxy-3-keto-5-methylthiopentene dioxygenase	0	NA	NA	NA	NA	NA

Table S17: Enzymatic Functions (EC_DGE), Standard Diet, Late in Mission

	Description	baseMean	log2FoldChange	lfcSE	stat	pvalue	padj
3.2.1.49	alpha-N-acetylgalactosaminidase	2.267948236	20.83867637	4.094037317	5.090006456	3.58E-07	0.000713238
1.3.1.104	enoyl-[acyl-carrier protein] reductase III	0.779460713	17.96670318	4.087184038	4.395863512	1.10E-05	0.006286032
4.2.1.22	cystathionine beta-synthase	0.792135483	-17.96063534	4.11323436	-4.366548018	1.26E-05	0.006286032
3.4.16.4	D-alanyl-D-alanine carboxypeptidase	6876.188992	3.377516147	0.756547191	4.46438264	8.03E-06	0.006286032
3.1.27.6	ribonuclease I (enterobacter ribonuclease)	0.207448764	17.43232241	4.114794557	4.23649885	2.27E-05	0.007358
2.3.1.235	minimal PKS ketosynthase (KS/KS alpha)	0.207448764	17.43232241	4.114794557	4.23649885	2.27E-05	0.007358
3.2.1.64	levanbiose-producing levanase	0.405616982	17.23563879	4.096709069	4.207191309	2.59E-05	0.007358
3.4.22.48	staphopain A	0.245162986	16.89418185	4.113923895	4.106585897	4.02E-05	0.009998599
3.2.1.155	xyloglucan-specific exo-beta-1,4-glucanase	0.222928835	16.76888646	4.114799066	4.075262533	4.60E-05	0.010173025
3.1.1.56	carboxylesterase 2	0.167196626	16.22657971	4.116249353	3.942078896	8.08E-05	0.014628233
3.1.1.84	carboxylesterase 2	0.167196626	16.22657971	4.116249353	3.942078896	8.08E-05	0.014628233
6.3.1.-	#N/A	0.197426127	-15.13457739	4.141443943	-3.654420438	0.000257764	0.042788754
4.2.1.53	oleate hydratase	647.2225734	1.678465299	0.475340635	3.531078924	0.000413868	0.063417348
2.1.1.182	16S rRNA (adenine1518-N6/adenine1519-N6)-dimethyltransfer	820.9449571	2.199593394	0.640705928	3.433077948	0.000596771	0.084911918
2.7.-.-	#N/A	187.9179305	1.050221421	0.315881617	3.324731051	0.000885039	0.117533134
3.2.1.1	alpha-amylase	2005.81684	3.180150865	0.992966557	3.20267671	0.001361567	0.169515138
2.1.2.9	methionyl-tRNA formyltransferase	346.3261987	0.447820201	0.148512475	3.015370934	0.002566651	0.300751163
3.6.3.54	copper-transporting P-type ATPase V	180.24383	0.887921981	0.300431895	2.955485069	0.003121778	0.345476773
1.18.1.1	rubredoxin---NAD+ reductase	51.1723042	-1.784319658	0.644419916	-2.76887727	0.005624982	0.589734952
5.4.99.1	methylaspartate mutase sigma subunit	88.22815367	1.557385647	0.575864289	2.704431716	0.006842133	0.681476453
4.4.1.11	methionine-gamma-lyase	52.03597116	1.112368129	0.416263103	2.672271744	0.007533961	0.714650017
3.2.1.136	glucuronoarabinoxylan endo-1,4-beta-xylanase	28.86644239	1.994213393	0.76224355	2.616241743	0.008890359	0.804981593
2.7.1.194	PTS system, ascorbate-specific IIA component	22.57368151	-2.147226947	0.826624209	-2.597585365	0.009388178	0.813097865
3.2.1.55	alpha-N-arabinofuranosidase	371.5198309	1.313864528	0.516001474	2.546241811	0.010888973	0.903784757
4.2.1.6	galactonate dehydratase	37.59083693	1.108509415	0.44305997	2.501939894	0.012351489	0.984166664
2.1.1.22	carnosine N-methyltransferase	0.929084248	-0.064397577	4.101182036	-0.015702199	0.987471972	0.999968657
5.3.1.29	ribose 1,5-bisphosphate isomerase	1.243333821	-0.645604461	3.893298044	-0.165824567	0.868295012	0.999968657
4.4.1.25	L-cysteate sulfo-lyase	2.615552796	-0.106643155	2.077157881	-0.0513409	0.959053878	0.999968657
3.2.1.99	arabinan endo-1,5-alpha-L-arabinosidase	139.1340885	0.315290949	0.560883274	0.562132914	0.574025465	0.999968657
2.8.1.7	cysteine desulfurase	1139.077994	0.058145915	0.240003796	0.242270813	0.808570328	0.999968657
3.4.22.-	#N/A	6.960518191	1.80320616	1.907443749	0.945352208	0.344479093	0.999968657
2.3.1.7	carnitine O-acetyltransferase	0.017504292	-0.007393638	4.141980666	-0.001785049	0.998575738	0.999968657
1.14.11.-	#N/A	0.589631369	-1.174760384	4.103133173	-0.28630813	0.774642132	0.999968657
2.5.1.9	#N/A	543.7887389	1.086938364	0.797540464	1.362862969	0.172925701	0.999968657
3.2.1.22	alpha-galactosidase	682.0919527	0.144020475	0.336866552	0.427529758	0.668993515	0.999968657
5.3.1.17	4-deoxy-L-threo-5-hexosulose-uronate ketol-isomerase	242.425249	-0.276950845	0.342133704	-0.809481328	0.418238339	0.999968657
3.2.1.132	chitosanase	0.184986723	-1.272056298	4.127511293	-0.308189659	0.757938021	0.999968657
3.1.3.18	phosphoglycolate phosphatase	780.8428987	-0.505607551	0.460347902	-1.098316182	0.272066448	0.999968657
1.3.8.6	glutaryl-CoA dehydrogenase	0.112273496	-0.541469018	4.138007264	-0.130852602	0.89589191	0.999968657
1.1.1.44	6-phosphogluconate dehydrogenase	386.0194263	-0.45285491	0.651837402	-0.694736001	0.487220762	0.999968657
6.1.1.14	#N/A	630.4145281	-0.020835312	0.23398023	-0.089047316	0.929044306	0.999968657
2.5.1.47	cysteine synthase	480.1082915	-0.156344652	0.377948175	-0.413666904	0.679118068	0.999968657
6.3.2.7	UDP-N-acetylmuramoyl-L-alanyl-D-glutamate-L-lysine ligase	4.22944368	0.353433831	1.722243443	0.205217115	0.837402498	0.999968657
3.5.2.10	creatinine amidohydrolase	77.38836357	0.071526901	0.51299541	0.139429904	0.889110443	0.999968657
4.2.2.20	chondroitin-sulfate-ABC endolyase/exolyase	92.73215075	-0.007293624	0.634134935	-0.011501692	0.99082318	0.999968657
1.2.3.3	pyruvate oxidase	5.87582389	-1.475675038	1.857281463	-0.794534952	0.42688407	0.999968657
1.1.1.6	glycerol dehydrogenase	131.5645382	-0.310730344	0.480953587	-0.646071374	0.518233133	0.999968657
2.7.7.70	D-beta-D-heptose 7-phosphate kinase / D-beta-D-heptose 1-ph	30.61416296	0.660339415	0.682838187	0.967051093	0.333518493	0.999968657
6.1.1.11	seryl-tRNA synthetase	599.7939619	0.17827399	0.213288389	0.835835416	0.403247499	0.999968657
3.1.1.41	cephalosporin-C deacetylase	12.9623897	-0.24267647	0.74043425	-0.327748845	0.743101568	0.999968657
2.5.1.39	4-hydroxybenzoate polyprenyltransferase	7.596073966	0.149595605	1.105620681	0.135304637	0.892371019	0.999968657
2.6.1.37	2-aminoethylphosphonate-pyruvate transaminase	28.43514803	-0.851767223	0.892690326	-0.95415756	0.340003893	0.999968657
2.1.1.-	#N/A	1033.626585	-0.098229207	0.235292746	-0.417476563	0.676329864	0.999968657
1.5.1.43	carboxynorspermidine synthase	0.131959613	1.192435617	4.136414978	0.28827756	0.773134283	0.999968657
2.3.3.13	2-isopropylmalate synthase	379.9594429	0.085181006	0.157733014	0.540032828	0.589174393	0.999968657
3.5.1.28	N-acetylmuramoyl-L-alanine amidase	773.0604699	0.045646692	0.188964429	0.241562354	0.809119295	0.999968657
2.4.2.3	uridine phosphorylase	240.7680276	-0.209378676	0.26584391	-0.787600047	0.430930687	0.999968657
3.4.19.5	beta-aspartyl-peptidase (threonine type)	19.4807214	-0.381763188	0.534370686	-0.714416411	0.474969727	0.999968657
1.15.1.2	superoxide reductase	1449.052551	-0.069347022	0.620288873	-0.111797946	0.910983616	0.999968657
2.4.1.20	cellobiose phosphorylase	219.5275666	0.326126992	0.360012117	0.905877822	0.365000524	0.999968657
3.1.11.6	#N/A	611.926668	-0.061253771	0.179731997	-0.340806154	0.733249517	0.999968657
1.3.1.106	precorrin-6A/cobalt-precorrin-6A reductase	80.75062147	-0.095053102	0.6065588	-0.1567088	0.875474351	0.999968657
2.8.4.5	threonylcarbamoyladenosine tRNA methylthiotransferase CDK/	336.5404218	0.095237964	0.314664562	0.302665046	0.762145138	0.999968657
2.7.7.23	UDP-N-acetylglucosamine/UDP-N-acetylgalactosamine diphosp	466.8862771	0.199172058	0.430548295	0.462600969	0.643650414	0.999968657
4.1.1.23	orotidine-5'-phosphate decarboxylase	361.1107121	-0.17942697	0.220787924	-0.812666594	0.416409239	0.999968657
1.1.1.270	3-keto steroid reductase	0.499547738	-0.899291794	4.109214446	-0.218847618	0.826768752	0.999968657
2.7.4.2	phosphomevalonate kinase	4.874322445	-0.660938026	1.453288534	-0.45478789	0.649261843	0.999968657
3.5.1.54	allophanate hydrolase	0.548905896	-1.826325867	4.11371757	-0.443959955	0.657071536	0.999968657
4.2.1.49	urocanate hydratase	221.4685891	0.320177584	0.429647262	0.745210343	0.456144571	0.999968657
2.4.1.44	UDP-D-galactose:(glucosyl)LPS alpha-1,3-D-galactosyltransferas	0.907193448	-2.2717196	4.100297222	-0.554037787	0.579552985	0.999968657
4.2.1.42	galactarate dehydratase	4.67297282	-0.490660361	1.25573288	-0.390736254	0.695992198	0.999968657
2.5.1.26	alkyldihydroxyacetonephosphate synthase	19.14698164	0.220171334	0.767835352	0.286742898	0.774309188	0.999968657
2.7.7.24	glucose-1-phosphate thymidylyltransferase	376.3120803	-0.147491048	0.237617563	-0.620707688	0.53479197	0.999968657
5.4.99.60	precorrin-8X/cobalt-precorrin-8 methylmutase	102.094744	-0.189965651	0.507681135	-0.374183002	0.708268169	0.999968657
2.4.2.19	nicotinate-nucleotide pyrophosphorylase (carboxylating)	246.5480813	-0.099164864	0.310092431	-0.319791309	0.749126536	0.999968657
2.1.1.187	23S rRNA (guanine745-N1)-methyltransferase	129.5080327	0.693073376	0.358498134	1.933269129	0.053203053	0.999968657
3.4.11.18	methionyl aminopeptidase	1044.301231	0.026080036	0.317865482	0.0820474	0.934609021	0.999968657
2.1.1.204	tRNA (cytosine38-C5)-methyltransferase	0.144519491	0.091882749	4.123514988	0.022282628	0.982222507	0.999968657
1.2.1.43	#N/A	5.915036706	0.319938574	1.09158483	0.293095475	0.769449176	0.999968657
2.7.1.66	undecaprenol kinase	2.161074354	0.690620913	1.783939942	0.387132379	0.698658208	0.999968657
2.6.1.1	aspartate aminotransferase, chloroplastic	520.0808933	-0.02860987	0.259879104	-0.110089152	0.912338671	0.999968657
4.3.1.19	threonine dehydratase	227.9571154	0.00395316	0.295516505	0.013377121	0.98932692	0.999968657
3.5.1.10	formyltetrahydrofolate deformylase	24.4019335	-1.575156132	0.972521193	-1.619662525	0.10530479	0.999968657
3.7.1.12	cobalt-precorrin 5A hydrolase	99.95703726	0.082895657	0.433725122	0.19112487	0.848427764	0.999968657
1.1.1.62	17beta-estradiol 17-dehydrogenase	0.771316995	0.301606091	4.103094002	0.07350699	0.941402682	0.999968657

1.3.3.4	#N/A	12.23533618	-0.964243708	1.174740538	-0.820814194	0.411752112	0.999968657
3.5.4.16	GTP cyclohydrolase IA	161.7547469	-0.549047211	0.457049959	-1.201284891	0.229640709	0.999968657
2.1.1.200	tRNA (cytidine32/uridine32-2'-O)-methyltransferase	3.219890731	1.13596931	2.867617625	0.396136953	0.692003999	0.999968657
5.3.1.6	#N/A	479.29167	-0.069579064	0.222453778	-0.312779873	0.754447908	0.999968657
1.1.1.335	UDP-N-acetyl-2-amino-2-deoxyglucuronate dehydrogenase	3.835544045	2.292010887	1.722668309	1.330500408	0.183353452	0.999968657
1.1.1.350	ureidoglycolate dehydrogenase (NAD+)	2.906536393	-2.135676698	1.943901042	-1.098655051	0.271918556	0.999968657
2.3.1.181	lipoyl(octanoyl) transferase	98.92787013	-0.563064849	0.700790138	-0.803471422	0.421702308	0.999968657
1.2.1.18	Oxidoreductases	18.35639239	0.470259394	0.56351807	0.834506325	0.403995725	0.999968657
2.1.1.104	caffeoyl-CoA O-methyltransferase	0.263418337	-0.135938625	4.140199955	-0.032833831	0.9738071	0.999968657
5.4.2.8	phosphomannomutase	156.1336159	0.019881676	0.322627811	0.061624184	0.950862117	0.999968657
1.2.99.5	#N/A	30.58832785	-0.257162093	0.838395769	-0.306731144	0.759048022	0.999968657
2.7.1.191	PTS system, mannose-specific IIA component	117.6263563	-0.327008481	0.452383535	-0.722856725	0.469767913	0.999968657
2.5.1.63	adenosyl-fluoride synthase	4.321299614	-0.445417374	1.78756883	-0.24917495	0.803225455	0.999968657
2.8.3.-	#N/A	14.81102674	-0.521762169	0.611359985	-0.853445076	0.393412533	0.999968657
3.6.4.12	DNA replication licensing factor MCM5	12313.76222	0.050361053	0.623867053	0.080724014	0.935661439	0.999968657
2.7.8.11	CDP-diacylglycerol--inositol 3-phosphatidyltransferase	0.582805694	-0.907411673	4.106777413	-0.220954676	0.825127724	0.999968657
1.12.98.1	#N/A	28.04594534	0.047259424	1.190969434	0.039681475	0.968347071	0.999968657
3.2.1.106	mannosyl-oligosaccharide glucosidase	0.284782452	-0.847080738	4.120994309	-0.205552513	0.837140474	0.999968657
5.4.99.26	tRNA pseudouridine65 synthase	1.975607773	1.979259369	1.969559572	1.004924856	0.314933035	0.999968657
1.4.1.2	glutamate dehydrogenase	48.68472367	0.146579487	0.8302995	0.176538089	0.859871228	0.999968657
3.1.21.1	deoxyribonuclease I	1.14797295	-0.635422825	4.064128795	-0.156349087	0.875757867	0.999968657
2.7.7.83	UDP-N-acetylglucosamine/UDP-N-acetylgalactosamine diphosp	41.03166629	-0.733393077	0.853636376	-0.859139907	0.390263332	0.999968657
2.6.1.62	adenosylmethionine---8-amino-7-oxononanoate aminotransfer	73.02597664	-0.27425171	0.73246312	-0.374423916	0.708088952	0.999968657
1.8.3.2	thiol oxidase	3.056885843	-0.261778448	3.719958694	-0.070371332	0.943898108	0.999968657
1.1.1.310	(S)-sulfolactate dehydrogenase	0.458766862	0.610221597	4.089309347	0.149223633	0.881377172	0.999968657
2.6.1.9	histidinol-phosphate aminotransferase	241.1110682	-0.140967367	0.194382404	-0.725206419	0.468325405	0.999968657
3.1.4.53	3',5'-cyclic-AMP phosphodiesterase	0.527495585	-0.828122948	4.102330081	-0.201866484	0.840021105	0.999968657
3.2.1.26	beta-fructofuranosidase	668.236276	-0.257950323	0.432433924	-0.596508065	0.550835868	0.999968657
1.4.3.5	pyridoxamine 5'-phosphate oxidase	196.6921172	-1.206468933	0.834939217	-1.44497816	0.14846402	0.999968657
4.2.1.3	#N/A	1254.902064	-0.344314089	0.566635755	-0.607646245	0.543422123	0.999968657
5.1.3.23	UDP-GlcNAc3NAcA epimerase	1.804103656	-2.210217711	2.129393247	-1.03795657	0.299290275	0.999968657
4.2.3.4	3-dehydroquinate synthase	263.3059402	0.133638514	0.306452831	0.436081839	0.662777353	0.999968657
2.4.2.30	poly [ADP-ribose] polymerase 16	1.661425333	-1.186246343	2.315333137	-0.512343699	0.608410487	0.999968657
6.3.2.32	gamma-F420-2:alpha-L-glutamate ligase	0.812016276	-0.233031467	4.100810161	-0.056825714	0.95468403	0.999968657
1.3.98.3	oxygen-independent coproporphyrinogen III oxidase	570.8711187	0.289295777	0.25980659	1.113504381	0.265491868	0.999968657
2.4.1.256	alpha-1,2-glucosyltransferase	0.333031825	-0.872156465	4.117489705	-0.211817522	0.832249398	0.999968657
2.1.1.207	tRNA (cytidine/uridine-2'-O-)-methyltransferase	169.8966336	-0.144025503	0.400456431	-0.359653365	0.719106371	0.999968657
1.1.1.9	D-xylulose reductase	0.801105635	-0.714575664	4.105748513	-0.174042726	0.861831884	0.999968657
3.5.3.9	allantoate deiminase	14.3761206	-0.298270676	0.64571835	-0.461920705	0.644138185	0.999968657
1.4.1.13	glutamate synthase (NADPH) large chain	1037.619229	0.056364126	0.171744182	0.328186523	0.742770637	0.999968657
4.2.1.76	UDP-glucose 4,6-dehydratase	1.498656123	-0.618754892	4.098578593	-0.150968166	0.880000828	0.999968657
1.11.1.9	glutathione peroxidase	87.79499761	-0.052796093	0.499976047	-0.105597245	0.915901911	0.999968657
4.1.3.-	#N/A	225.5498462	-0.172731961	0.249444186	-0.692467376	0.488643867	0.999968657
3.4.23.36	signal peptidase II	249.2065007	-0.223260148	0.243686423	-0.916178035	0.359573524	0.999968657
4.1.1.86	L-2,4-diaminobutyrate decarboxylase	0.110533983	-0.435087999	4.142162324	-0.105038858	0.916344975	0.999968657
2.4.99.-	#N/A	0.084668419	0.274728032	4.141794518	0.066330676	0.947114561	0.999968657
2.4.1.211	1,3-beta-galactosyl-N-acetylhexosamine phosphorylase	886.5408123	-0.335590377	0.634686853	-0.528749533	0.596979211	0.999968657
5.5.1.4	myo-inositol-1-phosphate synthase	93.74847179	-0.17550696	0.36950205	-0.474982371	0.634799538	0.999968657
4.1.1.96	carboxynorspermidine decarboxylase	158.8615104	0.084993184	0.438036239	0.194032313	0.846150581	0.999968657
2.5.1.32	15-cis-phytoene synthase	4.713411545	2.000956657	1.607919105	1.244438635	0.213338171	0.999968657
3.5.1.1	L-asparaginase	366.752951	0.007962515	0.340602426	0.023377741	0.98134896	0.999968657
5.3.1.-	#N/A	0.594915199	-2.271832619	4.097872418	-0.554393204	0.579309776	0.999968657
3.4.17.-	#N/A	57.62759611	0.17693413	0.543187243	0.325733221	0.744626207	0.999968657
3.4.24.37	saccharolysin	0.04233421	0.310351444	4.143245313	0.074905399	0.940289981	0.999968657
6.2.1.-	#N/A	80.6595248	-0.343951978	0.425587418	-0.808181735	0.41898597	0.999968657
3.5.1.42	nicotinamide-nucleotide amidase	507.4585151	0.152204321	0.380013104	0.400523874	0.688770703	0.999968657
6.2.1.4	#N/A	20.93996699	0.040754421	1.502353363	0.027127054	0.978358397	0.999968657
1.17.3.2	xanthine dehydrogenase/oxidase	0.020495196	-0.019393708	4.142686886	-0.004681432	0.996264771	0.999968657
1.17.7.1	(E)-4-hydroxy-3-methylbut-2-enyl-diphosphate synthase	363.237913	0.015197629	0.164166669	0.092574391	0.926241689	0.999968657
4.1.3.41	3-hydroxy-D-aspartate aldolase	0.311717752	0.585663015	4.111801643	0.142434647	0.886736697	0.999968657
4.2.1.51	cyclohexadienyl dehydratase	217.7655411	0.183834594	0.310474714	0.592108104	0.5537782	0.999968657
1.3.1.28	2,3-dihydro-2,3-dihydroxybenzoate dehydrogenase	0.630791762	0.656385842	4.076080263	0.161033591	0.872066944	0.999968657
2.4.1.180	UDP-N-acetyl-D-mannosaminouronate:lipid I N-acetyl-D-manno	4.766590627	0.177445773	1.987770916	0.089268724	0.928868349	0.999968657
3.2.1.18	sialidase-1	722.2496707	-0.859060572	0.633775134	-1.355465884	0.175269171	0.999968657
3.6.1.42	guanosine-diphosphatase	0.312227018	-3.843061402	4.143731151	-0.927439851	0.353698208	0.999968657
6.3.5.5	carbamoyl-phosphate synthase	1356.851513	-0.022326538	0.177235958	-0.1259707	0.899755118	0.999968657
2.1.1.222	2-polyprenyl-6-hydroxyphenyl methylase / 3-demethylubiquino	0.340645926	1.275310444	4.113901968	0.310000203	0.756560802	0.999968657
3.4.25.2	ATP-dependent HslUV protease, peptidase subunit HslV	11.0559166	0.809127149	0.96667095	0.837024376	0.402578866	0.999968657
3.1.22.-	#N/A	0.333031825	-0.872156465	4.117489705	-0.211817522	0.832249398	0.999968657
2.7.1.20	adenosine kinase	2.502983657	-1.735600787	1.834031281	-0.946331071	0.34397975	0.999968657
1.12.7.2	ferredoxin hydrogenase	22.43350305	-0.365239465	0.518747841	-0.704079008	0.481383572	0.999968657
2.8.3.22	succinyl-CoA:(S)-malate CoA-transferase subunit A	0.128757426	-0.685780939	4.134677189	-0.165860817	0.868266483	0.999968657
1.2.1.-	#N/A	0.816141609	-3.17242556	3.863734205	-0.821077588	0.411602075	0.999968657
3.4.14.5	dipeptidyl-peptidase 4	252.4844495	0.076723273	0.519325839	0.14773629	0.88255089	0.999968657
3.1.3.26	4-phytase / acid phosphatase	3.711875381	1.730804624	1.262924347	1.370473717	0.170539074	0.999968657
1.14.11.47	50S ribosomal protein L16 3-hydroxylase	3.642950035	-1.341877359	1.280034437	-1.048313483	0.2944942	0.999968657
2.7.7.64	UDP-sugar pyrophosphorylase	2.795318261	-0.387458753	3.68695525	-0.105089085	0.91630512	0.999968657
2.1.1.77	protein-L-isoaspartate(D-aspartate) O-methyltransferase	1.597836612	0.056625955	2.563440851	0.022089823	0.982376305	0.999968657
4.1.1.36	phosphopantothenoylcysteine decarboxylase	273.1904406	-0.052310444	0.162774094	-0.321368359	0.747931258	0.999968657
1.2.1.2	#N/A	245.2872466	-0.437227625	0.521806805	-0.837910929	0.40208073	0.999968657
1.1.1.67	mannitol 2-dehydrogenase	4.516882265	-0.725805174	1.710872972	-0.424230896	0.671397431	0.999968657
3.2.1.185	non-reducing end beta-L-arabinofuranosidase	0.714364175	-1.732966942	4.117831805	-0.420844518	0.673868621	0.999968657
2.7.8.7	holo-[acyl-carrier protein] synthase	35.60714793	-0.036909719	0.571648835	-0.06456712	0.948518665	0.999968657
2.3.1.201	UDP-2-acetamido-3-amino-2,3-dideoxy-glucuronate N-acetyltra	22.62710242	0.467299871	0.780627084	0.598621135	0.549425559	0.999968657
5.99.1.3	#N/A	3660.059785	-0.369192418	0.536393516	-0.688286504	0.491272379	0.999968657
6.3.2.1	pantoate--beta-alanine ligase	150.1648978	-0.150349151	0.363696279	-0.413392053	0.679319396	0.999968657
4.3.1.12	ornithine cyclodeaminase	24.93366618	0.201885042	0.514700481	0.392237911	0.694882435	0.999968657

2.1.1.14	5-methyltetrahydropteroyltriglutamate--homocysteine methylt	94.21501856	-0.173307552	0.5295894	-0.327248907	0.743479633	0.999968657
2.4.1.131	#N/A	1.764497646	-0.114481035	3.530122682	-0.032429761	0.974129329	0.999968657
2.1.3.3	ornithine carbamoyltransferase	321.7840829	-0.318650645	0.231608038	-1.375818593	0.168877804	0.999968657
2.1.1.246	[methyl-Co(III) methanol-specific corrinoid protein]:coenzyme M	5.57705486	-0.289846739	1.171795985	-0.247352562	0.804635383	0.999968657
2.6.1.81	succinylornithine aminotransferase	0.203337428	-0.843388953	4.134035098	-0.204011077	0.838344843	0.999968657
5.4.99.5	chorismate mutase	299.5030678	-0.017081186	0.274566346	-0.062211505	0.950394401	0.999968657
2.7.7.12	UDPglucose--hexose-1-phosphate uridylyltransferase	348.5210504	0.002834709	0.28878378	0.009816024	0.992168072	0.999968657
5.2.1.13	prolycopene isomerase	1.503143303	-0.154004898	3.101273259	-0.049658603	0.960394446	0.999968657
6.3.4.15	biotin---protein ligase	359.9504088	-0.163400766	0.226023605	-0.722936728	0.469718758	0.999968657
3.1.3.-	#N/A	200.6898365	0.14811745	0.266743619	0.5552802	0.57870302	0.999968657
2.5.1.72	quinolinate synthase	156.0969679	-0.359826434	0.209226617	-1.719792822	0.085470108	0.999968657
1.8.4.8	phosphoadenosine phosphosulfate reductase	38.80354416	0.02284677	1.082183403	0.021111736	0.983156523	0.999968657
2.3.1.20	diacylglycerol O-acyltransferase / wax synthase	1.228064538	-0.634094983	4.099773925	-0.154665841	0.877084761	0.999968657
1.5.1.28	opine dehydrogenase	8.554545849	0.188295649	0.798007587	0.235957216	0.813465869	0.999968657
2.7.4.16	thiamine-monophosphate kinase	118.4753888	-0.158437835	0.3790735	-0.417960726	0.675975833	0.999968657
1.1.1.125	#N/A	21.0930698	-0.617509316	0.642904695	-0.960498999	0.336804135	0.999968657
2.6.1.85	para-aminobenzoate synthetase component II	61.58760241	-0.031004251	0.614575616	-0.050448228	0.959765205	0.999968657
4.1.99.1	tryptophanase	227.0207991	-0.031396397	0.455214617	-0.068970537	0.945013071	0.999968657
4.4.1.16	selenocysteine lyase	255.398513	0.07342789	0.337559154	0.217525991	0.827798454	0.999968657
2.7.1.45	2-dehydro-3-deoxygluconokinase	519.2465247	-0.083974582	0.247395567	-0.339434464	0.734282459	0.999968657
2.4.2.6	nucleoside deoxyribosyltransferase	0.102241287	0.604750838	4.13998296	0.146075683	0.883861643	0.999968657
2.7.1.92	5-dehydro-2-deoxygluconokinase	11.52794472	-1.304651057	0.99371056	-1.312908516	0.189213777	0.999968657
5.3.3.-	#N/A	0.157074332	0.320813212	4.143731151	0.077421338	0.938288366	0.999968657
3.4.22.47	gingipain K	1.00454257	0.320789341	4.143731151	0.077415578	0.938292949	0.999968657
4.3.3.7	4-hydroxy-tetrahydrodipicolinate synthase	488.6728682	-0.270716396	0.215661698	-1.25528269	0.209376157	0.999968657
1.1.1.367	UDP-2-acetamido-2,6-beta-L-arabino-hexul-4-ose reductase	89.12835807	-0.03500323	0.418538526	-0.083632038	0.933348993	0.999968657
1.14.12.19	3-phenylpropionate/trans-cinnamate dioxygenase subunit alph	1.118856663	5.986666777	4.070628575	1.470698362	0.141372709	0.999968657
2.7.7.42	[glutamine synthetase] adenylyltransferase / [glutamine synthe	10.5009941	0.525614227	0.980933908	0.535830419	0.592075757	0.999968657
5.1.3.11	cellobiose epimerase	95.78211526	0.389310362	0.598641009	0.650323576	0.515483231	0.999968657
1.6.3.3	NADH oxidase (H2O2-forming)	0.663509303	-2.148892929	4.11162941	-0.522637795	0.601226334	0.999968657
2.6.1.19	4-aminobutyrate aminotransferase	27.32058772	-0.086565927	0.57148184	-0.151476251	0.879600043	0.999968657
2.1.2.11	3-methyl-2-oxobutanoate hydroxymethyltransferase	194.9990628	-0.260286625	0.328475994	-0.792406841	0.428123492	0.999968657
5.4.3.5	D-ornithine 4,5-aminomutase subunit beta	35.04208565	0.670673429	0.924921383	0.725113984	0.468382106	0.999968657
2.6.1.21	D-alanine transaminase	38.44577382	0.023823649	0.436818866	0.054538966	0.956505765	0.999968657
2.7.8.6	undecaprenyl-phosphate galactose phosphotransferase	5.98517338	0.842857482	0.888405101	0.948731025	0.342757431	0.999968657
5.4.99.22	23S rRNA pseudouridine2605 synthase	322.0947105	-0.220223643	0.297318574	-0.740699244	0.458875818	0.999968657
1.1.1.130	3-dehydro-L-gulonate 2-dehydrogenase	10.5048034	-1.07628206	0.742452752	-1.449630373	0.147161621	0.999968657
2.7.4.26	isopentenyl phosphate kinase	0.374667258	-0.38164273	4.11901771	-0.092653821	0.926178585	0.999968657
1.3.5.3	menaquinone-dependent protoporphyrinogen oxidase	14.33543221	-0.523953588	0.844655427	-0.620316369	0.53504952	0.999968657
1.1.1.90	aryl-alcohol dehydrogenase	0.095768651	-0.751364288	4.136129673	-0.181658784	0.855850513	0.999968657
3.4.11.21	aspartyl aminopeptidase	189.1715901	0.150593619	0.322889474	0.466393708	0.640933714	0.999968657
3.4.21.-	#N/A	392.414596	0.284641093	0.266486844	1.068124373	0.285464413	0.999968657
2.3.1.222	phosphate propanoyltransferase	0.103005941	-0.564033383	4.137063428	-0.136336654	0.891555149	0.999968657
2.4.1.144	beta-1,4-mannosyl-glycoprotein beta-1,4-N-acetylglucosaminyl	0.830970326	-3.426400946	4.095811001	-0.83656227	0.402838661	0.999968657
1.1.1.83	tartrate dehydrogenase/decarboxylase / D-malate dehydrogena	5.410002282	1.5276326	0.913331604	1.672593604	0.094407327	0.999968657
2.1.1.173	23S rRNA (guanine2445-N2)-methyltransferase / 23S rRNA (gua	1.313961173	-0.240705799	2.37803298	-0.101220547	0.919375386	0.999968657
1.1.1.287	D-arabinitol dehydrogenase (NADP+)	0.799170637	-0.465467905	4.104665366	-0.11339972	0.909713661	0.999968657
6.1.1.-	#N/A	6.459165869	1.226006597	1.109374457	1.105133248	0.269101856	0.999968657
6.4.1.3	acetyl-CoA/propionyl-CoA carboxylase	273.9201141	-0.838546143	0.459424357	-1.825210462	0.067969276	0.999968657
1.7.2.2	nitrite reductase (cytochrome c-552)	49.12275699	0.087607284	0.441847072	0.198275126	0.842829812	0.999968657
5.4.4.2	salicylate biosynthesis isochorismate synthase	61.24446731	-0.464289392	0.437829428	-1.060434413	0.288947013	0.999968657
2.7.7.50	mRNA guanylyltransferase	1.392230011	-0.548103357	4.099240114	-0.133708527	0.893633062	0.999968657
3.1.4.14	acyl carrier protein phosphodiesterase	0.051862191	1.042153446	4.143731151	0.251501221	0.801426618	0.999968657
2.3.1.8	phosphate acetyltransferase	571.8730186	-0.489308424	0.397613251	-1.230613978	0.218467275	0.999968657
3.1.13.3	#N/A	386.1764493	0.216522452	0.236694317	0.914776723	0.360308857	0.999968657
4.2.2.2	pectate lyase	2.166420747	2.673006141	2.424795095	1.10236372	0.270303578	0.999968657
2.7.3.9	#N/A	954.3616646	-0.54768663	0.448943296	-1.219946117	0.222485302	0.999968657
4.3.1.3	histidine ammonia-lyase	217.1796459	0.131367352	0.371619888	0.353499251	0.72371419	0.999968657
3.1.13.1	exoribonuclease II	7.538701321	-0.671019427	1.122507714	-0.597786027	0.549982715	0.999968657
2.1.1.297	release factor glutamine methyltransferase	340.1934048	0.262987345	0.175399501	1.499361994	0.133779748	0.999968657
1.3.3.11	pyrroloquinoline-quinone synthase	0.110533983	-0.435087999	4.142162324	-0.105038858	0.916344975	0.999968657
3.6.3.10	H+/K+-exchanging ATPase	3.674943414	0.260846253	3.859443041	0.067586502	0.946114801	0.999968657
3.5.1.87	beta-ureidopropionase / N-carbamoyl-L-amino-acid hydrolase	41.46682057	0.303469246	0.478918978	0.633654668	0.526306215	0.999968657
3.4.21.62	subtilisin	0.102172711	0.014869512	4.139877907	0.003591775	0.997134184	0.999968657
3.1.1.29	peptidyl-tRNA hydrolase, PTH1 family	279.5423892	-0.144178655	0.335265874	-0.430042742	0.66716455	0.999968657
6.4.1.4	#N/A	2.745395653	-0.195146545	3.729739584	-0.052321762	0.958272314	0.999968657
3.5.1.110	ureidoacrylate peracid hydrolase	0.074204671	0.32081088	4.143731151	0.077420776	0.938288814	0.999968657
2.3.1.42	glyceronephosphate O-acyltransferase	0.906836526	0.063392911	4.100376356	0.015460266	0.987664984	0.999968657
1.18.1.2	ferredoxin/flavodoxin---NADP+ reductase	421.7852528	-0.346540372	0.401675159	-0.862737873	0.388281604	0.999968657
1.8.4.12	peptide-methionine (R)-S-oxide reductase	160.8333044	0.164460686	0.434319805	0.378662643	0.704938397	0.999968657
3.5.2.17	5-hydroxyisourate hydrolase	0.196024097	-0.918351418	4.13072455	-0.222322115	0.824063138	0.999968657
5.1.3.7	UDP-N-acetylglucosamine 4-epimerase	42.11554931	0.034246788	0.658431415	0.052012688	0.958518584	0.999968657
3.4.24.28	bacillolysin	0.938853782	0.879437692	4.048122389	0.217245826	0.828016773	0.999968657
1.1.2.4	D-lactate dehydrogenase (cytochrome)	4.173147047	1.272365516	1.621974706	0.784454598	0.432773412	0.999968657
2.7.11.11	protein kinase A	4.556984542	-0.495187698	2.81661103	-0.175809756	0.860443405	0.999968657
5.1.3.26	GlcNAc-P-P-Und epimerase	55.79927746	-0.586203611	0.69103678	-0.848295819	0.396273246	0.999968657
1.3.1.98	UDP-N-acetylmuramate dehydrogenase	388.1332859	-0.02876178	0.255106297	-0.112744296	0.910233282	0.999968657
4.1.3.43	4-hydroxy-2-oxovalerate/4-hydroxy-2-oxohexanoate aldolase	0.165800975	-0.724199525	4.136604711	-0.175071	0.861023845	0.999968657
5.1.3.30	D-psicose/D-tagatose/L-ribulose 3-epimerase	12.77438443	-0.714274394	0.63939757	-1.117105269	0.263949318	0.999968657
4.2.1.93	ATP-dependent NAD(P)H-hydrate dehydratase	0.404452483	0.099575894	4.107060797	0.02424505	0.980657144	0.999968657
4.2.3.153	(5-formylfuran-3-yl)methyl phosphate synthase	1.166323075	-0.638546815	4.0998186	-0.155750017	0.876230071	0.999968657
2.7.1.82	ethanolamine kinase	1.883458461	-0.142714302	4.096532872	-0.034837827	0.972209058	0.999968657
2.4.1.5	dextransucrase	0.839572008	-2.999868243	4.101501618	-0.731407304	0.464530405	0.999968657
2.3.1.75	diacylglycerol O-acyltransferase / wax synthase	1.228064538	-0.634094983	4.099773925	-0.154665841	0.877084761	0.999968657
3.2.1.123	endoglycosylceramidase	0.937054039	1.151522367	3.683541787	0.312612815	0.754574841	0.999968657
2.7.1.25	adenylylsulfate kinase	65.7994572	-0.217021977	0.552319071	-0.392928632	0.694372194	0.999968657
2.9.1.-	#N/A	0.512348345	-1.033592565	4.107223001	-0.25165241	0.801309745	0.999968657

4.2.1.28	propanediol dehydratase large subunit	272.216343	-0.337201166	0.923888259	-0.364980464	0.715125989	0.999968657
5.3.3.18	2-(1,2-epoxy-1,2-dihydrophenyl)acetyl-CoA isomerase	12.98823612	-0.101043092	0.688502532	-0.146757763	0.883323224	0.999968657
3.1.2.1	acetyl-CoA hydrolase	33.55176493	-1.607377283	2.151408807	-0.747127779	0.454986434	0.999968657
2.1.1.213	tRNA (guanine10-N2)-dimethyltransferase	0.249773869	-0.816552953	4.124382892	-0.19798185	0.843059265	0.999968657
1.1.1.178	3-hydroxyacyl-CoA dehydrogenase / 3-hydroxy-2-methylbutyry	0.416289782	-0.888208298	4.112570367	-0.215974006	0.829008009	0.999968657
2.7.1.56	1-phosphofructokinase	313.4501232	-0.905717413	0.381912156	-2.371533346	0.017714448	0.999968657
1.1.1.159	7-alpha-hydroxysteroid dehydrogenase	14.93492507	-1.847128514	1.101602711	-1.676764678	0.093588507	0.999968657
2.7.1.211	PTS system, sucrose-specific IIA component	94.53090177	0.551238319	0.397515851	1.386707769	0.165530891	0.999968657
3.2.1.4	endoglucanase	673.5239206	1.058565487	0.435286295	2.431883338	0.015020544	0.999968657
2.7.7.33	glucose-1-phosphate cytidylyltransferase	43.70030962	-0.206189457	0.416492754	-0.49506133	0.620556829	0.999968657
2.4.1.41	polypeptide N-acetylgalactosaminyltransferase	3.059020224	-0.287319685	2.967706187	-0.096815408	0.922872988	0.999968657
4.2.1.130	D-lactate dehydratase / protein deglycase	1.123723061	-0.278859912	3.245535545	-0.085921078	0.931529156	0.999968657
2.8.1.11	adenylyltransferase and sulfurtransferase	38.27308209	-0.841027281	0.661640726	-1.271123811	0.203684606	0.999968657
2.2.1.7	1-deoxy-D-xylulose-5-phosphate synthase	675.3423551	0.060886984	0.184928687	0.329245747	0.741969947	0.999968657
2.1.1.225	tRNA:m4X modification enzyme	1.426288518	-0.502316234	4.099330551	-0.122536162	0.902474408	0.999968657
3.4.13.22	zinc D-Ala-D-Ala dipeptidase	69.29723343	-0.308200473	0.559457864	-0.550891306	0.581708188	0.999968657
4.4.1.8	#N/A	447.9802593	0.610362628	0.355084019	1.718924524	0.085628116	0.999968657
1.3.1.101	digeranylgeranylglycerophospholipid reductase	2.870100187	-0.347005083	3.909458866	-0.088760387	0.92927234	0.999968657
3.1.3.82	D-glycero-D-manno-heptose 1,7-bisphosphate phosphatase	27.20891321	0.22377455	0.537147564	0.416597905	0.676972542	0.999968657
2.7.1.144	tagatose 6-phosphate kinase	19.2680341	0.110628733	0.649321881	0.170375797	0.864714605	0.999968657
1.5.1.24	N5-(carboxyethyl)ornithine synthase	0.365589093	-1.861607615	4.115987246	-0.452287022	0.651062224	0.999968657
2.1.1.289	cobalt-precorrin-7 (C5)-methyltransferase	1.380155497	1.268176909	4.097889701	0.309470728	0.756963476	0.999968657
3.1.1.97	diphthine methyl ester acylhydrolase	0.368040408	-0.880011888	4.11507336	-0.213850838	0.830663379	0.999968657
4.2.1.134	very-long-chain (3R)-3-hydroxyacyl-CoA dehydratase	0.052512875	-0.340236249	4.139416356	-0.082194256	0.934492241	0.999968657
1.10.3.9	photosystem II P680 reaction center D1 protein	0.896335188	0.050608101	4.070305492	0.01243349	0.990079766	0.999968657
2.5.1.3	thiamine-phosphate pyrophosphorylase	546.7049117	0.161511548	0.537762581	0.300339878	0.763917918	0.999968657
1.16.1.3	aquacobalamin reductase / NAD(P)H-flavin reductase	1.401765292	-0.760728593	3.954415065	-0.192374493	0.847448871	0.999968657
2.1.1.195	cobalt-precorrin-5B (C1)-methyltransferase	157.4125858	-0.030528505	0.314647382	-0.0970245	0.922706938	0.999968657
5.3.1.9	glucose-6-phosphate isomerase	996.9255714	-0.230325863	0.365924142	-0.629436093	0.529063595	0.999968657
2.7.2.3	phosphoglycerate kinase	1220.852491	-0.702786381	0.471947766	-1.489118991	0.136456038	0.999968657
5.3.3.2	isopentenyl-diphosphate Delta-isomerase	23.14189555	-0.727512444	0.742881949	-0.979310973	0.32742635	0.999968657
3.4.18.1	cathepsin X	33.57074902	0.516043958	1.307031252	0.394821437	0.692974673	0.999968657
2.7.1.150	1-phosphatidylinositol-3-phosphate 5-kinase	0.457193986	-0.247299211	4.106982474	-0.060214333	0.95198493	0.999968657
3.5.1.5	urease	390.454859	-0.549163933	0.650266712	-0.84452106	0.3983783	0.999968657
4.2.1.99	aconitate hydratase 2 / 2-methylisocitrate dehydratase	5.473792319	2.258251054	1.910843089	1.181808735	0.237281601	0.999968657
2.3.1.241	Kdo2-lipid IVA lauroyltransferase	89.05169472	0.11585443	0.434442747	0.266673643	0.789720449	0.999968657
1.2.1.19	aminobutyraldehyde dehydrogenase	0.098065194	1.482145147	4.134061847	0.358520313	0.719953969	0.999968657
6.3.5.9	cobyrinic acid a,c-diamide synthase	147.2244454	-0.384044684	0.314207109	-1.222266058	0.22160703	0.999968657
1.1.1.169	2-dehydropantoate 2-reductase	287.7816628	-0.054272631	0.307441006	-0.176530229	0.859877402	0.999968657
3.5.2.2	dihydropyrimidinase	110.1995053	-0.054111673	0.313820895	-0.172428522	0.863100651	0.999968657
1.18.6.1	nitrogenase delta subunit	64.43291247	-0.232355705	0.748895371	-0.310264576	0.756359767	0.999968657
5.4.99.45	tRNA pseudouridine38/39 synthase	0.201524496	-0.770870323	4.129944085	-0.186653937	0.851931974	0.999968657
2.7.7.80	adenylyltransferase and sulfurtransferase	38.27308209	-0.841027281	0.661640726	-1.271123811	0.203684606	0.999968657
5.3.1.25	L-fucose/D-arabinose isomerase	314.2306221	-0.654355001	0.398860898	-1.640559417	0.100888905	0.999968657
3.6.3.14	#N/A	2909.060585	0.038727625	0.276176486	0.140227814	0.888479997	0.999968657
2.1.1.181	23S rRNA (adenine1618-N6)-methyltransferase	5.132909253	-1.678061953	1.273356535	-1.317825689	0.187562006	0.999968657
2.3.1.129	UDP-N-acetylglucosamine acyltransferase	195.8513799	-0.04244456	0.313863528	-0.135232533	0.892428026	0.999968657
2.5.1.58	protein farnesyltransferase subunit beta	0.766825899	-0.919439367	4.103200851	-0.224078567	0.822696169	0.999968657
4.2.1.104	cyanate lyase	0.603088763	1.917912644	4.109169135	0.466739767	0.640686075	0.999968657
3.5.99.7	1-aminocyclopropane-1-carboxylate deaminase	1.103206331	-1.260175899	2.403821981	-0.524238446	0.600112704	0.999968657
1.3.99.4	3-oxosteroid 1-dehydrogenase	0.726380301	0.844071556	4.050802536	0.20837144	0.834938955	0.999968657
2.4.1.198	#N/A	0.270591585	-0.492320533	4.124424015	-0.1193671	0.904984525	0.999968657
1.2.1.22	lactaldehyde dehydrogenase / glycolaldehyde dehydrogenase	20.55117114	-0.433662613	0.555609512	-0.780516898	0.435086685	0.999968657
3.6.1.6	ectonucleoside triphosphate diphosphohydrolase 5/6	3.61936385	-0.425444798	2.860390282	-0.148736626	0.88176146	0.999968657
1.1.1.40	malate dehydrogenase (oxaloacetate-decarboxylating)(NADP+)	103.4381533	0.119385963	0.424226154	0.281420564	0.778387848	0.999968657
2.7.8.17	UDP-N-acetylglucosamine-lysosomal-enzyme	2.981701137	-0.545131692	3.700357749	-0.147318646	0.882880516	0.999968657
3.2.1.51	alpha-L-fucosidase	1731.162235	-0.166917762	0.426818248	-0.391074568	0.695742118	0.999968657
5.4.99.9	UDP-galactopyranose mutase	302.3987479	-0.178416274	0.197032532	-0.905516835	0.365191645	0.999968657
4.1.99.17	phosphomethylpyrimidine synthase	285.8239162	0.57409544	0.272598013	2.106014763	0.03520307	0.999968657
5.1.3.9	N-acylglucosamine-6-phosphate 2-epimerase	224.6239802	0.096422541	0.579676292	0.166338597	0.867890493	0.999968657
1.3.1.10	enoyl-acyl-carrier protein] reductase I	101.9054622	0.074285112	0.396383207	0.187407314	0.851341289	0.999968657
2.7.6.5	GTP pyrophosphokinase	1176.233156	0.183312362	0.139178694	1.317100745	0.187804857	0.999968657
1.2.1.72	D-erythrose 4-phosphate dehydrogenase	1.604490321	2.027157111	3.060723257	0.662313101	0.507770583	0.999968657
5.1.3.25	dTDP-L-rhamnose 4-epimerase	7.819350156	-0.974629513	1.097939432	-0.887689688	0.374707694	0.999968657
4.2.1.32	#N/A	57.89837913	-0.637986458	0.675394919	-0.944612463	0.344856762	0.999968657
4.2.1.149	crotonobetainyl-CoA hydratase	2.640428685	-3.077436734	1.926627645	-1.597317853	0.110194873	0.999968657
2.3.1.88	#N/A	6.11169659	-0.445718765	2.451423813	-0.181820362	0.855723704	0.999968657
5.5.1.1	muconate cycloisomerase	0.112351376	-0.382154715	4.138210726	-0.092347814	0.9264217	0.999968657
3.6.1.55	8-oxo-dGTP diphosphatase	263.2355375	0.042907032	0.288997679	0.148468432	0.881973098	0.999968657
2.7.1.177	L-threonine kinase	0.753503802	-0.260617838	4.10070669	-0.063554372	0.949325064	0.999968657
5.4.2.9	phosphoenolpyruvate phosphomutase	19.55533939	0.928341191	0.862802394	1.075960379	0.281944977	0.999968657
6.3.1.5	NAD+ synthase	24.1008445	1.18961661	0.576499075	2.063518677	0.039063375	0.999968657
1.18.1.-	#N/A	0.842428939	1.293992903	4.07765153	0.317337785	0.75098731	0.999968657
4.2.1.113	O-succinylbenzoate synthase	6.192673817	0.353157575	1.45046275	0.243479245	0.807634162	0.999968657
2.3.1.-	#N/A	294.6393979	-0.17511688	0.325184425	-0.538515581	0.590221151	0.999968657
1.16.3.2	ferritin heavy chain	754.4409733	-0.387943519	0.503951075	-0.769803932	0.441416207	0.999968657
1.11.1.21	catalase-peroxidase	7.96020901	1.549426408	1.984598886	0.780725223	0.434964122	0.999968657
5.1.3.-	#N/A	101.2987499	-0.240128192	0.874336447	-0.274640492	0.783592454	0.999968657
1.2.7.5	aldehyde:ferredoxin oxidoreductase	32.16256784	0.176540424	0.640327722	0.275703234	0.782776012	0.999968657
4.2.1.46	dTDP-glucose 4,6-dehydratase	412.7164931	-0.050887794	0.223273252	-0.22791711	0.819710681	0.999968657
3.5.5.1	nitrilase	6.75173712	0.518491133	1.246175443	0.416065921	0.677361766	0.999968657
1.14.14.9	4-hydroxyphenylacetate 3-monooxygenase	2.25640993	-1.333431742	2.464270358	-0.541106108	0.588434448	0.999968657
6.3.2.35	D-alanine---D-serine ligase	29.18882695	-0.718547252	0.59442271	-1.208815276	0.226733821	0.999968657
2.7.7.1	nicotinamide-nucleotide adenylyltransferase	3.591754005	-1.981993998	1.292933172	-1.532943884	0.125289682	0.999968657
2.3.1.157	bifunctional UDP-N-acetylglucosamine pyrophosphorylase / Glu	425.7922078	0.233161136	0.461495735	0.505229233	0.61339784	0.999968657
3.1.3.6	2',3'-cyclic-nucleotide 2'-phosphodiesterase / 3'-nucleotidase	285.4621956	-0.412867592	0.445149058	-0.927481671	0.353676504	0.999968657
3.5.2.5	allantoinase	7.295496758	-0.450243943	0.949486459	-0.474197329	0.635359198	0.999968657

4.3.1.29	D-glucosaminate-6-phosphate ammonia-lyase	0.040990391	-0.227404664	4.140771598	-0.054918427	0.956203451	0.999968657
3.1.3.27	phosphatidylglycerophosphatase GEP4	37.35501302	-0.011924337	0.877691157	-0.013586029	0.989160251	0.999968657
5.4.2.11	2,3-bisphosphoglycerate-dependent phosphoglycerate mutase	320.0771963	-0.666143932	0.537215249	-1.239994458	0.214977444	0.999968657
2.7.4.6	nucleoside-diphosphate kinase	77.16799679	-1.008721211	0.419010067	-2.407391349	0.016066942	0.999968657
3.1.26.3	#N/A	233.5547131	-0.440161565	0.324510721	-1.356385279	0.174976615	0.999968657
2.1.1.132	precorrin-6Y C5,15-methyltransferase (decarboxylating)	165.8060595	-0.144585804	0.244491162	-0.591374359	0.554269617	0.999968657
2.7.7.27	glucose-1-phosphate adenylyltransferase	2589.01273	-0.283055942	0.488210016	-0.579783151	0.562060861	0.999968657
2.4.1.267	alpha-1,3-glucosyltransferase	0.129855667	-0.427183657	4.134938483	-0.103310765	0.917716332	0.999968657
1.12.1.3	NADP-reducing hydrogenase subunit HndB	567.3491264	-0.822662247	0.516044902	-1.594167957	0.110898421	0.999968657
3.4.24.13	IgA-specific metalloendopeptidase	8.078081151	-3.394934945	2.176908225	-1.559521392	0.118873026	0.999968657
1.11.1.6	#N/A	161.5587878	0.153447374	0.554305353	0.276828238	0.781911998	0.999968657
3.6.5.5	dynamin GTPase	7.357062005	-0.603131539	2.000690296	-0.301461721	0.763062436	0.999968657
2.7.2.8	acetylglutamate kinase	350.0145196	0.034754428	0.396033123	0.087756368	0.930070318	0.999968657
5.4.99.2	methylmalonyl-CoA mutase	436.038468	-0.029463283	0.307374303	-0.095854736	0.923635944	0.999968657
1.1.1.284	S-(hydroxymethyl)glutathione dehydrogenase / alcohol dehydr	2.230351518	1.61183672	4.052123928	0.397775771	0.690795479	0.999968657
2.7.4.21	inositol-hexakisphosphate 5-kinase	0.249773869	-0.816552953	4.124382892	-0.19798185	0.843059265	0.999968657
4.1.1.82	phosphonopyruvate decarboxylase	15.5295624	0.612083797	0.788185694	0.776573086	0.437410686	0.999968657
6.2.1.20	long-chain-fatty-acid--[acyl-carrier-protein] ligase	6.062451873	1.210722589	1.426743065	0.848591887	0.396108423	0.999968657
2.6.1.101	L-glutamine:2-deoxy-scyllo-inosose/3-amino-2,3-dideoxy-scyllo	0.025751485	-0.039846366	4.143731151	-0.00961606	0.992327613	0.999968657
4.3.1.15	diaminopropionate ammonia-lyase	83.25829541	-0.392935314	0.437606396	-0.897919495	0.369228471	0.999968657
3.4.13.19	membrane dipeptidase	203.4932454	0.445914786	0.310061528	1.438149355	0.150391681	0.999968657
4.6.1.15	triose/dihydroxyacetone kinase / FAD-AMP lyase (cyclizing)	1.318253244	0.261360972	2.6827375	0.097423237	0.922390292	0.999968657
3.1.3.43	pyruvate dehydrogenase phosphatase	1.498669032	-0.416012566	4.099640947	-0.101475366	0.919173112	0.999968657
6.2.1.3	long-chain acyl-CoA synthetase	787.0709517	0.102898052	0.239809115	0.429083155	0.66786271	0.999968657
2.7.1.196	PTS system, cellobiose-specific IIA component	42.12668325	-0.706336632	0.49865234	-1.416491159	0.156631751	0.999968657
1.1.1.38	malate dehydrogenase (oxaloacetate-decarboxylating)	352.0587986	0.257977774	0.267054388	0.966012111	0.334038121	0.999968657
6.3.4.21	nicotinate phosphoribosyltransferase	719.7523161	0.198023255	0.580658179	0.341032405	0.733079187	0.999968657
3.6.1.25	triphosphatase	0.176081768	5.048021843	4.093042624	1.233317682	0.217457263	0.999968657
1.5.98.1	methylenetetrahydromethanopterin dehydrogenase	7.559254633	-1.644423249	2.004575879	-0.820334748	0.412025303	0.999968657
4.2.1.17	enoyl-CoA hydratase	991.304438	-0.588724717	0.597559484	-0.98521525	0.324518351	0.999968657
4.4.1.21	S-ribosylhomocysteine lyase	157.4335133	-0.433119949	0.360435777	-1.201656375	0.22949669	0.999968657
3.6.1.62	mRNA-decapping enzyme subunit 2	0.250485555	0.21085775	4.139832929	0.050933879	0.959378209	0.999968657
2.1.1.72	site-specific DNA-methyltransferase (adenine-specific)	1002.074774	0.121196374	0.261159855	0.464069695	0.642597813	0.999968657
5.4.1.4	D-galactarolactone isomerase	0.052512875	-0.340236249	4.139416356	-0.082194256	0.934492241	0.999968657
3.1.13.-	#N/A	15.34080406	2.153440225	1.593499717	1.351390403	0.176570406	0.999968657
5.3.1.3	L-fucose/D-arabinose isomerase	314.2306221	-0.654355001	0.398860898	-1.640559417	0.100888905	0.999968657
4.2.1.11	enolase	1347.16273	-0.051124853	0.424066484	-0.120558581	0.904040677	0.999968657
5.1.3.13	dTDP-4-dehydrorhamnose 3,5-epimerase	198.2590378	-0.246713137	0.303476093	-0.812957406	0.416242479	0.999968657
2.4.2.7	adenine phosphoribosyltransferase	220.4972688	0.057111715	0.342128965	0.166930372	0.867424835	0.999968657
3.4.11.5	proline iminopeptidase	13.29992319	-0.166922521	0.716199611	-0.233067037	0.815709351	0.999968657
1.5.1.-	#N/A	0.239309938	5.358132189	4.085435101	1.311520574	0.189681947	0.999968657
1.1.1.23	histidinol dehydrogenase	231.2619341	-0.093636134	0.159299486	-0.587799344	0.556666985	0.999968657
1.5.1.34	dihydropteridine reductase	16.32083196	0.36008934	1.013259166	0.355377333	0.722306928	0.999968657
1.8.4.11	peptide-methionine (S)-S-oxide reductase	173.9237147	0.219230234	0.411300153	0.533017633	0.594021374	0.999968657
3.4.24.56	insulysin	0.63958207	-0.912061671	4.105390184	-0.222161994	0.824187779	0.999968657
1.1.99.3	#N/A	2.344093746	-0.3020118	2.102349695	-0.143654407	0.885773375	0.999968657
5.3.2.5	2,3-diketo-5-methylthiopentyl-1-phosphate enolase	7.481498469	-0.343703836	0.970539245	-0.354136978	0.723236231	0.999968657
6.3.4.11	biotin---protein ligase	1.456275519	-0.210094117	4.097167016	-0.051277899	0.959104079	0.999968657
3.2.1.196	glycogen debranching enzyme	169.2043627	-0.303058983	0.462493759	-0.655271509	0.512292961	0.999968657
3.1.21.5	type III restriction enzyme	190.996469	0.126688858	0.252190578	0.50235365	0.615418777	0.999968657
3.11.1.2	phosphonoacetate hydrolase	0.557746465	0.34849732	4.104325523	0.084909766	0.932333128	0.999968657
3.5.4.27	methenyltetrahydromethanopterin cyclohydrolase	1.31203418	-0.48034965	4.099554854	-0.117171173	0.90672441	0.999968657
2.4.1.321	cellobionic acid phosphorylase	0.183277685	-0.275202031	4.138609899	-0.066496248	0.946982745	0.999968657
1.1.1.93	tartrate dehydrogenase	5.410002282	1.5276326	0.913331604	1.672593604	0.094407327	0.999968657
2.7.7.75	molybdopterin adenylyltransferase	1.658651571	1.817657665	2.32386627	0.782169649	0.434114879	0.999968657
2.7.7.87	L-threonylcarbamoyladenylate synthase	305.4179164	0.03583115	0.153824673	0.232935001	0.815811879	0.999968657
3.6.3.21	#N/A	551.7754522	-0.056369916	0.333876598	-0.168834582	0.865926756	0.999968657
2.7.12.1	#N/A	7.925832161	-0.246003473	4.093857985	-0.060090866	0.952083264	0.999968657
6.1.1.19	arginyl-tRNA synthetase	514.4061134	-0.355073426	0.274715347	-1.292513977	0.196179209	0.999968657
1.3.1.34	2,4-dienoyl-CoA reductase (NADPH2)	4.426268906	0.524082596	1.200651878	0.436498377	0.662475177	0.999968657
1.3.1.54	precorrin-6A/cobalt-precorrin-6A reductase	80.75062147	-0.095053102	0.6065588	-0.1567088	0.875474351	0.999968657
1.2.4.2	2-oxoglutarate dehydrogenase E1 component	25.41993019	1.006786908	0.754997404	1.333497179	0.182368701	0.999968657
3.2.1.183	UDP-N-acetylglucosamine 2-epimerase (hydrolysing)	12.14589756	2.044672583	1.079923607	1.893349279	0.058311436	0.999968657
2.6.1.82	putrescine aminotransferase	53.88562689	0.1222156	0.555356729	0.22006684	0.8258191	0.999968657
1.11.1.10	non-heme chloroperoxidase	2.224202965	-1.241278596	4.076257987	-0.304514238	0.760736147	0.999968657
2.4.2.29	queuine tRNA-ribosyltransferase	301.7581135	-0.051328766	0.206641719	-0.248394983	0.803828815	0.999968657
3.4.21.102	carboxyl-terminal processing protease	540.2492765	0.040383392	0.212156528	0.190347157	0.849037103	0.999968657
2.1.1.45	thymidylate synthase	183.6399445	0.083367321	0.351773111	0.236991739	0.812663199	0.999968657
2.7.1.6	galactokinase	445.280387	0.074083271	0.199416561	0.371500095	0.710265085	0.999968657
2.6.1.66	valine--pyruvate aminotransferase	70.05961202	0.075790185	0.422872471	0.17922705	0.857759425	0.999968657
2.7.1.16	L-ribulokinase	41.05216264	-0.379937747	0.406033842	-0.935729262	0.349412601	0.999968657
6.3.2.30	cyanophycin synthetase	0.53521663	-0.421178735	4.116752245	-0.102308497	0.918511811	0.999968657
4.1.1.3	#N/A	2726.451397	-0.084366701	0.58239968	-0.144860486	0.884821025	0.999968657
3.2.1.11	dextranase	27.01727601	-0.127691133	0.845416281	-0.151039358	0.879944668	0.999968657
1.8.1.8	thioredoxin 2	156.4033575	0.324163199	0.319228938	1.01545681	0.309888125	0.999968657
3.1.4.-	#N/A	196.6449542	0.174561312	0.276749198	0.630756342	0.528199853	0.999968657
2.3.1.46	homoserine O-acetyltransferase/O-succinyltransferase	218.3870639	-0.264628147	0.32080539	-0.824886848	0.409435834	0.999968657
6.2.1.7	solute carrier family 27 (fatty acid transporter), member 5	0.48997058	0.220618439	4.140088318	0.053288341	0.957502169	0.999968657
2.4.1.280	N,N'-diacetylchitobiose phosphorylase	5.176582286	-1.158413381	1.408333728	-0.822541815	0.410768598	0.999968657
3.4.21.96	lactocepin	235.7046641	-0.630518126	0.738063104	-0.854287557	0.39294568	0.999968657
4.1.1.-	#N/A	0.314148663	-3.866264326	4.143731151	-0.933039376	0.350799638	0.999968657
4.1.1.49	phosphoenolpyruvate carboxykinase (ATP)	1207.42963	-0.758752175	0.458061903	-1.656440253	0.097632695	0.999968657
2.3.1.193	tRNA(Met) cytidine acetyltransferase	0.767393725	0.029172633	4.074252414	0.007160242	0.994287002	0.999968657
1.1.1.77	lactaldehyde reductase	361.3221681	0.117242537	0.272464592	0.430303756	0.666974695	0.999968657
1.6.1.1	NAD(P) transhydrogenase	5.221596558	-0.683203468	2.049115027	-0.333413917	0.738821859	0.999968657
1.1.1.372	L-glyceraldehyde reductase	4.412671685	-0.953738307	4.093449042	-0.232991372	0.815768106	0.999968657
1.5.1.38	FMN reductase	13.56279913	-0.680645693	1.092173175	-0.623203086	0.533151074	0.999968657

1.1.1.137	ribulose-5-phosphate 2-dehydrogenase	24.26923239	-0.261358287	0.601553773	-0.434472027	0.663945704	0.999968657
2.1.1.34	tRNA (guanosine-2'-O-)-methyltransferase	7.652998207	0.363411262	1.423338438	0.255323156	0.798473516	0.999968657
3.2.2.26	futalosine hydrolase	1.673267527	-3.221566499	3.738268949	-0.86178029	0.388808433	0.999968657
3.4.23.49	omptin	0.074204671	0.32081088	4.143731151	0.077420776	0.938288814	0.999968657
2.7.11.23	CTD kinase subunit alpha	1.84467463	-0.801091985	4.096679206	-0.195546672	0.844965004	0.999968657
6.1.1.4	leucyl-tRNA synthetase	687.997849	0.055770602	0.186406751	0.299187672	0.764796855	0.999968657
4.1.2.21	2-dehydro-3-deoxyphosphogalactonate aldolase	0.046509078	-0.039846366	4.143731151	-0.00961606	0.992327613	0.999968657
3.1.1.53	sialate O-acetylesterase	282.7241622	0.325925076	0.448733653	0.726321893	0.467641461	0.999968657
2.6.1.77	taurine-pyruvate aminotransferase	15.705862	1.177824055	0.792917	1.48543171	0.137429501	0.999968657
2.3.1.189	mycothiol synthase	0.446767538	0.014706454	4.139002033	0.00355314	0.99716501	0.999968657
2.1.1.217	tRNA (adenine22-N1)-methyltransferase	250.9195669	-0.550053907	0.455771763	-1.206862626	0.227485053	0.999968657
2.1.1.242	16S rRNA (guanine1516-N2)-methyltransferase	12.35996513	-0.281605899	0.772008007	-0.364770697	0.715282581	0.999968657
3.5.4.3	guanine deaminase	138.5600482	-0.322554302	0.492607594	-0.654789543	0.512603264	0.999968657
4.1.3.1	isocitrate lyase	1.190703912	-0.015989145	3.505494603	-0.004561167	0.996360728	0.999968657
2.7.1.-	#N/A	1909.117588	-0.611830145	0.504379447	-1.21303544	0.225116271	0.999968657
2.2.1.10	fructose-bisphosphate aldolase / 2-amino-3,7-dideoxy-D-threo-	2.56819499	-2.659902525	2.058596183	-1.292095335	0.196324131	0.999968657
1.1.1.100	3-oxoacyl-[acyl-carrier protein] reductase	830.0443141	-0.078078722	0.335483748	-0.232734736	0.815967395	0.999968657
1.17.99.6	epoxyqueuosine reductase	17.31413246	-0.636972895	0.843347062	-0.755291533	0.450074078	0.999968657
2.4.99.14	3-deoxy-D-manno-octulosonic-acid transferase	104.6062867	0.006585804	0.474861753	0.013868887	0.988934584	0.999968657
4.2.1.24	porphobilinogen synthase	56.76727574	0.408725645	0.521960671	0.783058318	0.433592867	0.999968657
2.4.1.260	alpha-1,6-mannosyltransferase	1.51946093	-0.829106211	4.097680269	-0.202335506	0.839654444	0.999968657
6.3.5.4	asparagine synthase (glutamine-hydrolysing)	377.2433377	-0.023441477	0.224381645	-0.104471457	0.916795218	0.999968657
6.1.1.22	asparaginyl-tRNA synthetase	569.499554	-0.252840157	0.350107168	-0.722179321	0.470184236	0.999968657
2.7.1.33	type I pantothenate kinase	366.0985092	-0.032694151	0.388181379	-0.084223904	0.932878413	0.999968657
6.5.1.1	bifunctional non-homologous end joining protein LigD	24.55321329	0.896652475	0.692986808	1.293895446	0.195701538	0.999968657
2.4.1.10	levansucrase	0.401328364	-1.97030358	4.114505788	-0.478867616	0.63203281	0.999968657
3.2.2.8	ribosylpyrimidine nucleosidase	7.613796596	-0.677218351	1.020272049	-0.663762524	0.506842315	0.999968657
4.2.1.35	3-isopropylmalate/(R)-2-methylmalate dehydratase large subur	590.1564115	-0.249592804	0.155679368	-1.603249085	0.108879672	0.999968657
1.1.1.343	6-phosphogluconate dehydrogenase	386.0194263	-0.45285491	0.651837402	-0.694736001	0.487220762	0.999968657
2.7.1.59	N-acetylglucosamine kinase	0.905041437	1.339033662	3.655246098	0.366332013	0.714117344	0.999968657
2.5.1.114	tRNA wybutosine-synthesizing protein 2	0.249773869	-0.816552953	4.124382892	-0.19798185	0.843059265	0.999968657
2.4.1.336	1,2-diacylglycerol 3-beta-glucosyltransferase	0.52830923	-0.756298483	4.110592729	-0.183987695	0.854023108	0.999968657
3.4.21.83	oligopeptidase B	1.520068509	-1.190270057	1.920734776	-0.619695167	0.535458498	0.999968657
2.7.3.-	#N/A	31.26877507	-0.237238062	0.67149924	-0.3532961	0.723866469	0.999968657
3.5.1.49	formamidase	0.917025487	-1.549298947	2.784548358	-0.556391467	0.577943271	0.999968657
2.7.7.53	sulfate adenylyltransferase (ADP) / ATP adenylyltransferase	1.402946654	-1.645477946	2.966726721	-0.55464426	0.579138009	0.999968657
1.2.1.76	malonyl-CoA/succinyl-CoA reductase (NADPH)	28.49464127	0.031091556	0.656522736	0.047357927	0.962227961	0.999968657
4.3.2.3	ureidoglycolate lyase	78.66999186	0.133610524	1.897981009	0.070396133	0.943878369	0.999968657
3.1.1.5	lysophospholipase	20.15479229	-0.732959748	0.629927867	-1.163561395	0.244601804	0.999968657
2.4.1.132	alpha-1,3/alpha-1,6-mannosyltransferase	1.814404281	0.036188386	4.097078152	0.00883273	0.992952593	0.999968657
1.1.1.304	meso-butanediol dehydrogenase / (S,S)-butanediol dehydroger	2.544822965	0.675197034	1.895219804	0.35626318	0.721643481	0.999968657
2.3.1.81	aminoglycoside 3-N-acetyltransferase	34.85777649	-0.94017167	0.887889091	-1.058884133	0.289652551	0.999968657
5.3.1.12	glucuronate isomerase	529.9970512	0.310071318	0.465084963	0.666698222	0.504964915	0.999968657
2.1.1.61	tRNA 5-methylaminomethyl-2-thiouridine biosynthesis bifuncti	3.684858882	0.529427905	1.593596787	0.332221996	0.739721636	0.999968657
3.6.1.63	alpha-D-ribose 1-methylphosphonate 5-triphosphate diphospha	2.990499697	-0.418041523	2.134556758	-0.195844651	0.84473176	0.999968657
3.4.11.26	intermediate cleaving peptidase 55	0.312227018	-3.843061402	4.143731151	-0.927439851	0.353698208	0.999968657
2.7.11.2	pyruvate dehydrogenase kinase 2/3/4	3.406061021	-0.456456756	3.544516423	-0.128778288	0.897533086	0.999968657
3.1.11.-	#N/A	0.311872348	-0.476356442	4.136594413	-0.115156671	0.908320942	0.999968657
2.1.1.249	dimethylamine---corrinoid protein Co-methyltransferase	1.264396432	-2.510995186	3.093916673	-0.811591084	0.417026309	0.999968657
2.3.-.-	#N/A	2.137499889	-0.694228142	2.441892449	-0.28429923	0.776181076	0.999968657
2.5.1.18	glutathione S-transferase	5.747224439	0.530740039	1.720850148	0.308417348	0.757764783	0.999968657
1.3.1.22	steroid 5-alpha-reductase	119.5639343	-1.710983343	0.878321177	-1.948015586	0.051413098	0.999968657
3.1.2.-	#N/A	298.9955864	-0.502090537	0.298876936	-1.679923997	0.092972104	0.999968657
4.2.1.8	mannonate dehydratase	319.1697185	-0.197801048	0.204488642	-0.967296012	0.333396077	0.999968657
4.1.1.94	ethylmalonyl-CoA/methylmalonyl-CoA decarboxylase	2.58784862	-0.391739429	3.146177783	-0.124512807	0.90090926	0.999968657
2.4.1.141	beta-1,4-N-acetylglucosaminyltransferase	0.388858125	-0.595835653	4.114065097	-0.144828932	0.884845939	0.999968657
3.4.11.2	aminopeptidase N	47.09178138	-0.367875616	0.537894686	-0.683917551	0.494027231	0.999968657
3.5.3.26	(S)-ureidoglycine aminohydrolase	8.571204104	-0.119586865	0.821184946	-0.145627201	0.884215693	0.999968657
4.2.2.6	oligogalacturonide lyase	18.28317034	-0.714161427	0.667101396	-1.070544045	0.284374495	0.999968657
2.7.1.162	N-acetylhexosamine 1-kinase	7.442655289	0.014000479	1.749842217	0.008000995	0.993616198	0.999968657
1.1.1.3	homoserine dehydrogenase	435.1502177	-0.296989209	0.214686821	-1.383360221	0.166554441	0.999968657
1.13.11.-	#N/A	0.969176871	0.257001726	4.092461504	0.062798813	0.949926711	0.999968657
3.6.3.1	#N/A	7.365742814	-0.753582174	2.042165171	-0.369011373	0.712119248	0.999968657
2.6.1.-	#N/A	379.879431	-0.101285859	0.301025642	-0.336469208	0.736517068	0.999968657
5.1.3.31	D-psicose/D-tagatose/L-ribulose 3-epimerase	12.77438443	-0.714274394	0.63939757	-1.117105269	0.263949318	0.999968657
3.2.1.141	maltooligosyltrehalose trehalohydrolase	0.465749231	0.019747657	4.106726812	0.004808612	0.996163297	0.999968657
1.2.7.8	indolepyruvate ferredoxin oxidoreductase, alpha subunit	571.351912	-0.105830201	0.314846384	-0.336132814	0.736770715	0.999968657
5.3.3.14	3-hydroxyacyl-[acyl-carrier protein] dehydratase / trans-2-dece	2.895859573	0.747711828	1.756297775	0.425731808	0.670303281	0.999968657
3.1.3.70	mannosyl-3-phosphoglycerate phosphatase	0.122002457	-0.564064771	4.140415365	-0.136233861	0.891636408	0.999968657
2.7.4.8	guanylate kinase	418.9878264	-0.024658618	0.310569013	-0.079398193	0.936715906	0.999968657
3.5.1.108	UDP-3-O-[3-hydroxymyristoyl] N-acetylglucosamine deacetylas	116.3137628	0.092040889	0.291074733	0.316210506	0.751842734	0.999968657
1.97.1.9	Tat-targeted selenate reductase subunit YnfE	165.715164	0.115704798	0.347033096	0.333411422	0.738823742	0.999968657
2.7.1.202	PTS system, fructose-specific IIA component	408.9532809	-0.63395741	0.377127798	-1.6810148	0.09276004	0.999968657
2.7.7.7	#N/A	4748.086639	0.104167436	0.218093913	0.477626517	0.632916054	0.999968657
2.3.1.101	formylmethanofuran--tetrahydromethanopterin N-formyltrans	2.080730589	-0.601987577	4.09654958	-0.146949906	0.88317156	0.999968657
2.5.1.16	spermidine synthase	74.31269831	0.079409203	0.447037151	0.177634461	0.859010059	0.999968657
3.6.3.31	polyamine-transporting ATPase	242.7358801	0.301240289	0.453560084	0.664168431	0.506582516	0.999968657
6.3.4.9	biotin---protein ligase	1.456275519	-0.210094117	4.097167016	-0.051277899	0.959104079	0.999968657
1.1.5.2	quinoprotein glucose dehydrogenase	1.838329898	2.207780411	4.062841038	0.543408022	0.586848904	0.999968657
2.5.1.76	cysteate synthase	1.65237058	-0.545548625	2.788281665	-0.195657645	0.844878138	0.999968657
3.2.1.78	mannan endo-1,4-beta-mannosidase	244.4033901	0.531209415	0.537281043	0.988699343	0.322810264	0.999968657
3.8.1.2	2-haloacid dehalogenase	90.88721424	-0.016785853	0.36807358	-0.045604614	0.963625392	0.999968657
2.1.1.186	23S rRNA (cytidine2498-2'-O)-methyltransferase	1.410587932	0.393063017	2.191692245	0.17934225	0.857668974	0.999968657
3.6.3.33	vitamin B12 transport system ATP-binding protein	0.081334971	-0.381289104	4.142921482	-0.092033872	0.926671128	0.999968657
1.1.1.140	sorbitol-6-phosphate 2-dehydrogenase	9.218496711	0.82830341	1.044014056	0.793383389	0.427554486	0.999968657
3.4.21.19	glutamyl endopeptidase	47.63602797	0.499982651	0.564890521	0.885096549	0.376104561	0.999968657
5.4.99.17	squalene-hopene/tetraprenyl-beta-curcumene cyclase	7.194674308	2.516898369	1.346719826	1.868910163	0.061635314	0.999968657

5.3.3.1	3beta-hydroxy-Delta5-steroid dehydrogenase / steroid Delta-is	0.017504292	-0.007393638	4.141980666	-0.001785049	0.998575738	0.999968657
2.8.1.8	lipoyl synthase	74.59543311	-0.167511385	0.494596638	-0.338682823	0.734848682	0.999968657
2.7.1.29	triose/dihydroxyacetone kinase / FAD-AMP lyase (cyclizing)	1.318253244	0.261360972	2.6827375	0.097423237	0.922390292	0.999968657
3.1.2.23	4-hydroxybenzoyl-CoA thioesterase	0.325679685	0.57336579	4.109995413	0.139505214	0.889050936	0.999968657
3.2.1.180	unsaturated chondroitin disaccharide hydrolase	100.6903551	-0.760911021	0.500275441	-1.52098416	0.128263812	0.999968657
3.4.21.66	thermitase	0.051502971	-0.263696455	4.141439557	-0.063672656	0.949230878	0.999968657
2.3.1.180	3-oxoacyl-[acyl-carrier-protein] synthase III	380.2113221	-0.255491977	0.272435306	-0.937807882	0.34834315	0.999968657
6.3.5.10	adenosylcobyric acid synthase	172.5070828	0.06634628	0.306039174	0.216790155	0.828371885	0.999968657
1.3.1.76	uroporphyrin-III C-methyltransferase / precorrin-2 dehydrogena	30.31722373	-0.041296544	0.835666778	-0.049417477	0.9605866	0.999968657
3.1.3.46	6-phosphofructo-2-kinase / fructose-2,6-biphosphatase 3	3.811408119	-0.550578171	2.830102539	-0.194543541	0.845750306	0.999968657
4.1.1.8	oxalyl-CoA decarboxylase	0.835501983	-1.259965264	3.777844391	-0.333514336	0.73874607	0.999968657
2.1.1.198	16S rRNA (cytidine1402-2'-O)-methyltransferase	604.2734402	-0.139533262	0.39597514	-0.352378844	0.724554166	0.999968657
3.2.1.17	lysozyme	68.4832737	-0.821981106	0.942099738	-0.872499028	0.382936198	0.999968657
3.5.3.3	creatinase	0.347232792	-0.568697592	4.114092176	-0.138231612	0.890057372	0.999968657
2.8.3.12	#N/A	134.3823044	0.297881737	0.504846542	0.590044128	0.555161065	0.999968657
4.2.1.75	uroporphyrinogen-III synthase	157.1676558	0.068772697	0.252673963	0.272179595	0.785483931	0.999968657
2.4.2.43	4-amino-4-deoxy-L-arabinose transferase	0.111464417	1.546221436	4.136560128	0.373794019	0.708557569	0.999968657
3.4.24.70	oligopeptidase A	23.81967512	1.178000137	0.926219746	1.271836561	0.203431196	0.999968657
3.6.4.-	#N/A	1316.288446	0.199374768	0.196305753	1.01563385	0.309803779	0.999968657
1.11.1.1	NADH peroxidase	1.37195059	-2.252865286	3.3598076	-0.670534017	0.502517429	0.999968657
1.3.1.38	mitochondrial trans-2-enoyl-CoA reductase	12.07210064	-0.650323539	1.98909615	-0.326944245	0.743710056	0.999968657
4.2.1.151	chorismate dehydratase	8.95134946	0.1078042	0.878227901	0.122751965	0.902303513	0.999968657
2.4.1.259	alpha-1,2-mannosyltransferase	1.191163093	-0.451800965	4.099581595	-0.110206604	0.912245525	0.999968657
2.5.1.78	6,7-dimethyl-8-ribityllumazine synthase	125.3847375	-0.067943078	0.386753233	-0.175675526	0.860548864	0.999968657
2.7.8.12	CDP-glycerol glycerophosphotransferase	32.67163653	0.07395602	0.698834746	0.105827623	0.91571912	0.999968657
1.1.1.94	glycerol-3-phosphate dehydrogenase (NAD(P)+)	453.6570729	-0.080834301	0.252299392	-0.32039039	0.74867241	0.999968657
3.5.3.8	formiminoglutamase	2.637396381	2.623431782	1.85313158	1.415674855	0.156870724	0.999968657
3.5.99.10	2-iminobutanoate/2-iminopropanoate deaminase	243.9802341	0.086333554	0.296227056	0.291443853	0.77071188	0.999968657
1.1.1.27	L-lactate dehydrogenase	346.4267477	-0.469812561	0.513197756	-0.915461058	0.359949637	0.999968657
3.1.21.2	deoxyribonuclease IV	367.0371785	-0.380522	0.334917037	-1.136167939	0.255886286	0.999968657
2.8.3.5	3-oxoacid CoA-transferase	11.57711064	1.121628384	1.01501185	1.105039693	0.26914239	0.999968657
2.7.8.14	CDP-ribitol ribitolphosphotransferase / teichoic acid ribitol-pho:	27.28268986	1.318523197	0.952470683	1.38431893	0.16626082	0.999968657
6.3.1.19	proteasome accessory factor A	1.381132656	-3.055459592	4.101228294	-0.745010854	0.456265159	0.999968657
3.4.17.13	muramoyltetrapeptide carboxypeptidase	75.16794398	-1.046819403	0.860019031	-1.217204929	0.223526255	0.999968657
3.4.22.70	sortase A	1125.394995	0.093276969	0.342383034	0.272434552	0.785287908	0.999968657
3.5.1.105	chitin disaccharide deacetylase	3.814956233	-0.304169298	1.336714636	-0.227549912	0.819996163	0.999968657
4.1.1.85	3-dehydro-L-gulonate-6-phosphate decarboxylase	0.249683108	-0.122316526	4.131737936	-0.029604135	0.976382768	0.999968657
2.5.1.31	undecaprenyl diphosphate synthase	411.3346321	-0.19189223	0.238536718	-0.804455733	0.421133825	0.999968657
1.5.1.7	saccharopine dehydrogenase (NAD+, L-lysine forming)	197.2445999	0.035843728	0.316935453	0.113094726	0.909955456	0.999968657
3.4.19.1	acylaminoacyl-peptidase	1.868496682	-0.106935145	1.906481484	-0.056090314	0.95526986	0.999968657
3.1.3.73	alpha-ribazole phosphatase	197.9568009	0.049493521	0.25431061	0.194618389	0.845691706	0.999968657
2.1.1.166	23S rRNA (uridine2552-2'-O)-methyltransferase	15.58275964	-0.137837379	1.089306437	-0.126536826	0.899307	0.999968657
1.6.5.2	NAD(P)H dehydrogenase (quinone)	14.05382573	0.314313472	0.660981058	0.475525687	0.634412329	0.999968657
3.4.21.110	C5a peptidase	1.02995056	1.552353668	4.104148113	0.378240167	0.705252189	0.999968657
4.7.1.1	alpha-D-ribose 1-methylphosphonate 5-phosphate C-P lyase	3.093319832	0.697795443	1.954825581	0.356960462	0.721121405	0.999968657
5.1.1.3	glutamate racemase	294.3667194	-0.053999863	0.217303154	-0.248500134	0.803747466	0.999968657
6.3.1.12	D-aspartate ligase	24.89074862	-0.832428192	0.478576782	-1.739382734	0.081967463	0.999968657
1.8.1.2	#N/A	13.3789185	1.908176997	1.10261604	1.730590638	0.083524803	0.999968657
4.1.99.12	3,4-dihydroxy 2-butanone 4-phosphate synthase	303.3060112	-0.007707806	0.299540346	-0.025732113	0.97947101	0.999968657
3.1.3.96	pseudouridine 5'-phosphatase	0.416289782	-0.888208298	4.112570367	-0.215974006	0.829008009	0.999968657
4.1.2.14	2-dehydro-3-deoxyphosphogluconate aldolase / (4S)-4-hydroxy	296.8325156	-0.525551211	0.294307081	-1.785723975	0.074143962	0.999968657
5.4.99.27	tRNA pseudouridine13 synthase	2.123783118	-0.895673607	4.095828827	-0.218679453	0.826899756	0.999968657
1.12.99.6	#N/A	96.42638783	1.108531427	0.557949591	1.986794945	0.046945125	0.999968657
3.6.1.43	dolichyldiphosphatase	0.416289782	-0.888208298	4.112570367	-0.215974006	0.829008009	0.999968657
2.7.1.192	PTS system, N-acetylmuramic acid-specific IIB component	0.136601963	-0.622482464	4.139022345	-0.150393598	0.880454092	0.999968657
1.3.99.33	urocanate reductase	24.00152255	0.549261674	0.649721569	0.845380084	0.397898661	0.999968657
3.6.1.41	bis(5'-nucleosyl)-tetraphosphatase (symmetrical)	3.187241846	0.594768558	1.199930789	0.495669053	0.620127925	0.999968657
6.3.5.2	GMP synthase (glutamine-hydrolysing)	746.5195763	0.270450891	0.246759646	1.096009399	0.273074661	0.999968657
3.6.3.-	#N/A	1695.617713	0.034696285	0.184719027	0.18783276	0.851007754	0.999968657
3.6.1.29	bis(5'-adenosyl)-triphosphatase	0.035008583	-0.204325554	4.14032189	-0.049350161	0.960640245	0.999968657
2.3.1.76	diacylglycerol O-acyltransferase 1	1.228064538	-0.634094983	4.099773925	-0.154665841	0.877084761	0.999968657
3.1.6.8	arylsulfatase A	1.683971084	4.597431543	3.025007636	1.519808244	0.128559177	0.999968657
1.14.13.59	lysine N6-hydroxylase	1.497268624	1.064085555	4.097607218	0.259684616	0.795107061	0.999968657
2.7.1.170	anhydro-N-acetylmuramic acid kinase	25.33708748	0.071811967	0.636373835	0.112845568	0.910152991	0.999968657
2.6.1.57	aromatic-amino-acid transaminase	22.44406187	0.959665518	0.689602615	1.391621054	0.16403718	0.999968657
1.3.99.-	#N/A	44.4194708	-0.844927893	0.558261682	-1.513497916	0.13015323	0.999968657
2.7.8.36	undecaprenyl phosphate N,N'-diacetylbacillosamine 1-phospha	2.256262368	-1.884624195	2.452053636	-0.768590118	0.442136674	0.999968657
5.3.1.16	phosphoribosylformimino-5-aminoimidazole carboxamide ribot	198.1663491	-0.095412485	0.284089343	-0.335853793	0.736981123	0.999968657
3.1.2.6	hydroxyacylglutathione hydrolase	0.647579986	-2.526251467	4.111242941	-0.614473896	0.538902218	0.999968657
1.15.1.1	nickel superoxide dismutase	1042.834107	-0.157247548	0.632923024	-0.248446559	0.803788914	0.999968657
2.4.1.101	alpha-1,3-mannosyl-glycoprotein beta-1,2-N-acetylglucosaminy	4.740996338	-0.606238968	3.29235228	-0.18413551	0.853907149	0.999968657
3.5.4.43	hydroxydechloroatrazine ethylaminohydrolase	1.738495381	0.3216318	1.929625559	0.166680939	0.867621104	0.999968657
2.3.2.17	peptidoglycan pentaglycine glycine transferase (the second and	0.581404477	-1.173565568	4.120689029	-0.284798382	0.775798612	0.999968657
2.4.99.17	S-adenosylmethionine:tRNA ribosyltransferase-isomerase	312.4137439	-0.086680399	0.291780219	-0.297074281	0.7664098	0.999968657
4.2.2.3	poly(beta-D-mannuronate) lyase	4.039561784	-0.63043274	2.25229767	-0.279906492	0.779549246	0.999968657
6.3.2.8	UDP-N-acetylmuramate--alanine ligase	411.158067	0.366132294	0.220747629	1.658601256	0.097196169	0.999968657
2.7.1.184	sulfofructose kinase	3.910939087	0.980372816	1.173168626	0.835662321	0.403344898	0.999968657
3.5.99.6	#N/A	813.8476878	-0.103075042	0.323566343	-0.318559219	0.750060782	0.999968657
3.2.1.10	oligo-1,6-glucosidase	174.9034232	-0.361141737	0.472475936	-0.764360065	0.4446527	0.999968657
2.3.1.39	acyl-carrier-protein] S-malonyltransferase	274.8139792	-0.2013064	0.333405153	-0.603789109	0.545983861	0.999968657
2.4.99.12	3-deoxy-D-manno-octulosonic-acid transferase	104.6062867	0.006585804	0.474861753	0.013868887	0.988934584	0.999968657
3.2.1.113	mannosyl-oligosaccharide alpha-1,2-mannosidase	10.79752778	-0.462884978	4.093587138	-0.113075638	0.909970589	0.999968657
3.1.3.36	inositol polyphosphate 5-phosphatase INPP5B/F	8.344447898	-0.220328889	4.093804789	-0.053820077	0.957078514	0.999968657
1.13.12.-	#N/A	1.914163853	-1.624654046	4.092006378	-0.397031162	0.691344482	0.999968657
2.7.7.47	streptomycin 3''-adenylyltransferase	12.33183838	-0.268090156	1.03348375	-0.259404327	0.795323292	0.999968657
1.14.11.27	F-box and leucine-rich repeat protein 10/11	0.245483854	1.317910345	4.117209018	0.320097993	0.748894047	0.999968657
1.1.3.21	alpha-glycerophosphate oxidase	4.838986957	0.410517244	2.36454597	0.17361356	0.862169174	0.999968657

1.3.1.12	prephenate dehydrogenase	153.5917507	0.083660523	0.288504672	0.289979787	0.771831702	0.999968657
5.3.99.11	2-keto-myo-inositol isomerase	4.341769719	0.540836251	1.159560968	0.466414674	0.64091871	0.999968657
3.2.1.21	beta-glucosidase	2657.934981	-0.316027378	0.577920555	-0.546835331	0.584491867	0.999968657
1.5.5.2	RHH-type transcriptional regulator, proline utilization regulon re	63.59410052	0.049087302	0.480589999	0.102139665	0.918645817	0.999968657
2.4.2.2	pyrimidine-nucleoside phosphorylase	168.5824076	0.114677245	0.336160719	0.341138147	0.732999585	0.999968657
2.7.1.17	xylulokinase	345.6234778	0.063501588	0.305791548	0.207662992	0.835492116	0.999968657
2.7.7.13	mannose-1-phosphate guanylyltransferase	358.2910064	-0.036745923	0.281842603	-0.130377461	0.896267798	0.999968657
4.2.2.8	heparin/heparan-sulfate lyase	26.41907383	-1.029361463	1.081978075	-0.951369983	0.341416592	0.999968657
5.4.3.9	glutamate 2,3-aminomutase	0.081980782	-0.492938804	4.138295101	-0.119116397	0.90518314	0.999968657
1.11.1.15	peroxiredoxin (alkyl hydroperoxide reductase subunit C)	1347.271603	-0.597938358	0.576673623	-1.036874818	0.299794201	0.999968657
3.5.4.12	dCMP deaminase	245.9939325	-0.220496637	0.268270564	-0.821918864	0.411123076	0.999968657
3.6.3.40	teichoic acid transport system ATP-binding protein	74.48854514	-0.030235053	0.544154046	-0.055563408	0.955689616	0.999968657
2.4.1.250	D-inositol-3-phosphate glycosyltransferase	0.122971174	-0.667878002	4.136874465	-0.161445073	0.871742869	0.999968657
2.1.-.-	#N/A	0.255199619	-0.084299126	4.119168732	-0.020465082	0.983672367	0.999968657
3.1.11.5	exodeoxyribonuclease V	537.3560927	0.919993968	0.391208327	2.351672764	0.018689208	0.999968657
1.1.1.336	UDP-N-acetyl-D-mannosaminuronic acid dehydrogenase	25.22143025	-0.79571207	0.817845502	-0.972936903	0.330584662	0.999968657
1.14.13.-	#N/A	2.723150752	0.684631149	2.978377173	0.229867176	0.818194983	0.999968657
3.6.1.65	(d)CTP diphosphatase	3.073861829	3.698527341	2.697711355	1.370987053	0.170378992	0.999968657
1.2.1.81	sulfoacetaldehyde dehydrogenase	9.761748302	0.257491993	0.75686722	0.340207616	0.733700183	0.999968657
4.1.2.17	L-fuculose-phosphate aldolase	163.6600843	-0.842146059	0.444753323	-1.893512684	0.058289723	0.999968657
2.5.1.41	phosphoglycerol geranylgeranyltransferase	0.624441127	-0.562237103	4.106626684	-0.136909718	0.891102157	0.999968657
5.1.99.3	allantoin racemase	5.521276769	0.219328791	1.153413861	0.190156195	0.849186736	0.999968657
1.1.1.205	IMP dehydrogenase	601.6865534	-0.361106925	0.277591425	-1.300857633	0.19330719	0.999968657
2.4.1.325	dTDP-N-acetylfucosamine:lipid II N-acetylfucosaminyltransferas	0.162992848	-1.042372471	4.132822836	-0.252218039	0.800872536	0.999968657
2.1.3.11	N-succinyl-L-ornithine transcarbamylase	88.30516408	0.14623108	0.395448593	0.369785309	0.711542463	0.999968657
3.5.4.9	methylenetetrahydrofolate dehydrogenase (NADP+) / metheny	460.8469926	-0.306571306	0.325530029	-0.941760447	0.3463153	0.999968657
1.1.1.384	dTDP-3,4-didehydro-2,6-dideoxy-alpha-D-glucose 3-reductase	3.094463476	0.531433352	2.291841447	0.231880505	0.816630827	0.999968657
2.1.1.203	tRNA (cytosine34-C5)-methyltransferase	2.749594251	-0.26593215	3.036101553	-0.087590005	0.930202547	0.999968657
2.3.2.10	serine/alanine adding enzyme	5.042106869	-0.578876291	1.818358098	-0.318351095	0.750218631	0.999968657
4.1.2.50	6-pyruvoyltetrahydropterin/6-carboxytetrahydropterin synthas	143.680634	-0.193172864	0.406100767	-0.475677173	0.634304386	0.999968657
2.7.1.49	hydroxymethylpyrimidine/phosphomethylpyrimidine kinase / t	196.5194831	-0.213723551	0.270537986	-0.789994612	0.429530915	0.999968657
3.6.-.-	#N/A	345.8259585	-0.127304773	0.222327516	-0.572600169	0.566915445	0.999968657
6.3.1.1	aspartate--ammonia ligase	200.3070772	-0.203197498	0.282671252	-0.718847412	0.472234941	0.999968657
3.1.1.89	protein phosphatase methylesterase 1	1.451571512	-0.277495037	4.097669038	-0.067720217	0.946008355	0.999968657
2.1.1.298	ribosomal protein L3 glutamine methyltransferase	2.881866914	0.527491294	1.076667509	0.489929611	0.624183709	0.999968657
1.7.5.1	nitrate reductase / nitrite oxidoreductase, alpha subunit	30.68379277	0.643050917	0.899687637	0.714749087	0.4747641	0.999968657
1.1.1.14	L-iditol 2-dehydrogenase	103.1114767	0.160707027	0.462961727	0.347128105	0.72849508	0.999968657
1.3.1.89	tRNA-dihydrouridine synthase 3	2.528684853	-0.22359174	3.931710693	-0.056868818	0.954649694	0.999968657
1.13.11.2	catechol 2,3-dioxygenase	2.031466026	1.394408111	3.432961876	0.40618223	0.68460871	0.999968657
6.4.1.5	geranyl-CoA carboxylase alpha subunit	0.683567942	-0.914768224	4.104585057	-0.22286497	0.823640601	0.999968657
4.3.1.2	methylaspartate ammonia-lyase	56.28752504	1.012131909	0.733261229	1.380315594	0.167489496	0.999968657
3.4.11.7	glutamyl aminopeptidase	3.896020177	1.032203172	2.306107998	0.447595331	0.654445274	0.999968657
3.5.1.98	histone deacetylase 1/2	6.724734731	-0.149772922	4.094023003	-0.036583312	0.970817249	0.999968657
5.1.3.6	UDP-glucuronate 4-epimerase	20.60700147	-0.058638669	0.935176758	-0.0627033	0.95000277	0.999968657
3.1.26.12	ribonuclease E	21.05414725	-0.106144099	0.898659232	-0.118113847	0.905977452	0.999968657
5.4.99.58	methylornithine synthase	1.195008066	-0.959904891	2.715362422	-0.35350894	0.723706928	0.999968657
2.7.1.26	riboflavin kinase	334.3661825	0.077209675	0.228638124	0.33769379	0.735593955	0.999968657
3.2.1.33	glycogen debranching enzyme	2.01001148	0.435712205	4.016299339	0.108485989	0.913610194	0.999968657
3.2.1.14	chitinase	74.35089469	0.245846377	0.671214165	0.366271139	0.714162763	0.999968657
1.1.3.-	#N/A	0.196207128	-0.615882269	4.139032005	-0.148798624	0.881712536	0.999968657
3.5.1.91	N-substituted formamide deformylase	129.8540039	0.247875366	0.272919037	0.90823773	0.363752634	0.999968657
3.1.1.3	triacylglycerol lipase	4.439500809	-0.372720042	1.466427461	-0.254168755	0.799365188	0.999968657
3.5.1.32	hippurate hydrolase	22.867801	0.293521508	0.667394105	0.439802368	0.660080252	0.999968657
3.5.1.9	arylformamidase	136.0042455	0.317982711	0.827723556	0.384165351	0.700855897	0.999968657
3.1.13.4	poly(A)-specific ribonuclease	2.749647617	-0.594031656	4.095851172	-0.14503253	0.884685188	0.999968657
3.5.2.18	enamidase	3.748924151	2.000770736	1.833412066	1.091282627	0.27514854	0.999968657
2.7.1.55	allose kinase	14.51981234	-0.158832038	0.735803979	-0.215861891	0.829095403	0.999968657
4.1.1.19	arginine decarboxylase	178.3389114	0.118407715	0.465664788	0.25427672	0.799281784	0.999968657
4.1.2.40	tagatose 1,6-diphosphate aldolase	239.5719266	-1.004479043	0.672526857	-1.493589486	0.135282949	0.999968657
2.1.1.64	2-polyprenyl-6-hydroxyphenyl methylase / 3-demethylubiquino	0.340645926	1.275310444	4.113901968	0.310000203	0.756560802	0.999968657
2.3.1.61	2-oxoglutarate dehydrogenase E2 component (dihydrolipoamid	116.1652786	-0.069200831	0.676724517	-0.102258495	0.918551498	0.999968657
6.1.1.5	isoleucyl-tRNA synthetase	783.2345106	0.060865454	0.232690948	0.261572074	0.793651376	0.999968657
3.1.1.17	gluconolactonase	0.591879764	-1.074845685	4.108698349	-0.261602482	0.79362793	0.999968657
4.1.2.43	3-hexulose-6-phosphate synthase	9.761425783	-0.934813532	0.926440635	-1.009037706	0.312956551	0.999968657
4.2.1.80	2-keto-4-pentenoate hydratase	0.950669968	2.513664816	4.032449861	0.623359224	0.533048487	0.999968657
2.1.1.245	acetyl-CoA decarbonylase/synthase complex subunit delta	736.5094664	-0.543412272	0.630014389	-0.862539461	0.388390728	0.999968657
3.5.3.11	agmatinase	76.90483775	0.256480248	0.368939484	0.695182433	0.48694098	0.999968657
6.6.1.2	cobaltochelatase CobN	121.2009386	0.550011952	0.585849624	0.938827865	0.34781913	0.999968657
2.1.1.183	18S rRNA (adenine1779-N6/adenine1780-N6)-dimethyltransfer	1.791028411	-0.442827746	4.098374616	-0.108049602	0.913956346	0.999968657
3.5.-.-	#N/A	13.91699383	-0.414751464	0.721931965	-0.574502147	0.565628048	0.999968657
3.7.1.20	fumarylpyruvate hydrolase	5.87380492	2.185807878	1.244293115	1.756666377	0.078974692	0.999968657
2.7.8.28	LPPG:FO 2-phospho-L-lactate transferase	0.874214996	-0.640905496	4.102479006	-0.156223955	0.875856495	0.999968657
1.1.1.85	3-isopropylmalate dehydrogenase	228.1220397	-0.101449018	0.235922374	-0.430010161	0.667188249	0.999968657
3.2.1.122	maltose-6'-phosphate glucosidase	26.0661655	-0.670977227	0.655136103	-1.024179898	0.305750312	0.999968657
2.1.2.3	phosphoribosylaminoimidazolecarboxamide formyltransferase	547.7326221	-0.066403989	0.181472752	-0.365917131	0.714426913	0.999968657
3.2.1.89	arabinogalactan endo-1,4-beta-galactosidase	105.1190644	0.115757574	0.572674179	0.202135137	0.839811079	0.999968657
2.8.3.8	#N/A	183.3888661	0.134405023	0.528448036	0.254339148	0.799233558	0.999968657
2.1.1.271	precorrin-4/cobalt-precorrin-4 C11-methyltransferase	98.82307915	-0.038033584	0.363740947	-0.104562284	0.916723144	0.999968657
1.8.4.-	#N/A	2.424680651	-0.713761729	2.873529919	-0.24839196	0.803831154	0.999968657
2.4.2.9	uracil phosphoribosyltransferase	517.0070671	-0.17632912	0.296351931	-0.594999058	0.5518441	0.999968657
3.4.-.-	#N/A	1952.852084	-0.24282521	0.294197067	-0.825382837	0.409154276	0.999968657
4.1.1.44	4-carboxymuconolactone decarboxylase	73.14122184	-0.477682099	0.527768561	-0.905097678	0.365413642	0.999968657
3.1.1.73	feruloyl esterase	0.78497298	-2.452712363	4.111524215	-0.596545766	0.55081069	0.999968657
2.1.1.130	#N/A	73.91478762	-0.390825113	0.440859334	-0.886507516	0.375344106	0.999968657
4.1.1.12	aspartate 4-decarboxylase	60.2823711	-0.541903437	0.679090697	-0.797983892	0.424879838	0.999968657
1.7.2.3	trimethylamine-N-oxide reductase (cytochrome c)	15.37738031	0.897412939	0.660971774	1.357717492	0.174553344	0.999968657
2.10.1.1	molybdopterin molybdotransferase	116.2328554	-0.116238572	0.468264634	-0.248232653	0.803954404	0.999968657

1.4.3.-	#N/A	0.039493742	1.031690707	4.143245313	0.24900546	0.803356558	0.999968657
2.5.1.19	3-phosphoshikimate 1-carboxyvinyltransferase	298.9208236	-0.07771362	0.34264064	-0.226807946	0.820573084	0.999968657
2.1.3.2	aspartate carbamoyltransferase	349.324848	-0.483092081	0.283726337	-1.702669151	0.088630001	0.999968657
3.6.5.-	#N/A	429.493709	0.133837646	0.201146827	0.665372893	0.505812019	0.999968657
3.5.1.78	glutathionylspermidine amidase/synthetase	1.249834218	1.268291995	3.316259165	0.382446586	0.702130141	0.999968657
3.6.3.28	#N/A	9.816075023	-0.110017006	1.047282006	-0.10505003	0.91633611	0.999968657
2.1.3.1	methylmalonyl-CoA carboxyltransferase 5S subunit	0.495699816	0.376862626	4.105850781	0.091786732	0.926867486	0.999968657
2.1.1.214	tRNA (guanine10-N2)-methyltransferase	2.397965246	-0.47859155	4.096847556	-0.116819468	0.907003116	0.999968657
3.5.1.4	amidase	10.62089373	-0.062969033	0.946239571	-0.066546607	0.946942653	0.999968657
6.3.5.1	NAD+ synthase (glutamine-hydrolysing)	5370.660459	-1.852038962	1.174956989	-1.576261071	0.114965649	0.999968657
6.2.1.13	acetate---CoA ligase (ADP-forming) subunit alpha	21.3186108	0.545638745	0.79486421	0.686455294	0.492426047	0.999968657
1.2.1.27	malonate-semialdehyde dehydrogenase (acetylating) / methylr	18.35639239	0.470259394	0.56351807	0.834506325	0.403995725	0.999968657
2.3.3.9	malate synthase	7.740445911	2.94013762	2.275276958	1.292210871	0.196284128	0.999968657
1.5.99.15	dihydromethanopterin reductase (acceptor)	1.787205415	-0.269684256	4.097160778	-0.065822229	0.947519358	0.999968657
3.1.3.12	trehalose 6-phosphate phosphatase	40.02501221	-0.162500851	0.595113776	-0.273058459	0.784808279	0.999968657
5.3.1.30	5-deoxy-glucuronate isomerase	31.66558741	-0.391878544	0.457113072	-0.857290173	0.391284538	0.999968657
2.1.1.178	16S rRNA (cytosine1407-C5)-methyltransferase	0.283636172	0.072199322	4.113444851	0.017552034	0.985996222	0.999968657
2.7.2.7	butyrate kinase	160.9491394	-0.179477875	0.379214345	-0.473288729	0.636007202	0.999968657
3.2.1.133	cyclomaltodextrinase / maltogenic alpha-amylase / neopullulan	0.30127684	-1.467920402	4.12669228	-0.35571356	0.722055089	0.999968657
1.5.3.22	coenzyme F420H2 oxidase	4.822589886	0.162635822	2.146846738	0.075755674	0.939613482	0.999968657
1.17.4.2	ribonucleoside-triphosphate reductase (thioredoxin)	1225.795642	0.062450225	0.295267844	0.211503643	0.83249429	0.999968657
2.7.2.11	glutamate 5-kinase	246.2464691	0.125031978	0.190196415	0.657383461	0.510934383	0.999968657
2.7.8.8	CDP-diacylglycerol---serine O-phosphatidyltransferase	124.5052398	0.174538988	0.498122838	0.350393466	0.726043429	0.999968657
2.4.1.208	1,2-diacylglycerol-3-alpha-glucose alpha-1,2-glucosyltransferase	10.86455472	-0.327600908	0.61709354	-0.530877229	0.595503858	0.999968657
6.1.1.7	alanyl-tRNA synthetase	1204.488849	-0.055865723	0.303325359	-0.184177554	0.853874167	0.999968657
2.6.1.52	phosphoserine aminotransferase	469.6711707	-0.499682115	0.389522119	-1.282808065	0.199559328	0.999968657
4.99.1.3	sirohydrochlorin cobaltochelatase	153.816797	-0.154435433	0.35500415	-0.435024305	0.663544786	0.999968657
3.1.1.61	two-component system, chemotaxis family, protein-glutamate	50.27352804	0.840149507	0.637731269	1.317403659	0.187703355	0.999968657
2.5.1.90	octaprenyl-diphosphate synthase	86.87419176	-0.169713666	0.471353132	-0.360056303	0.718805029	0.999968657
3.5.1.16	acetylornithine deacetylase	73.83330864	0.348205947	0.413202564	0.842700355	0.399396049	0.999968657
1.1.1.202	1,3-propanediol dehydrogenase	2.374458127	-3.103052095	4.090880792	-0.758529092	0.448134304	0.999968657
1.20.1.1	phosphonate dehydrogenase	0.332585466	-0.329740267	4.112963724	-0.080170964	0.936101283	0.999968657
2.3.1.234	N6-L-threonylcarbamoyladenine synthase	531.8124809	0.282765885	0.314899594	0.897955699	0.369209168	0.999968657
4.1.99.22	GTP 3',8-cyclase	105.6089693	-0.279671988	0.352568237	-0.793242154	0.427636752	0.999968657
1.4.1.3	glutamate dehydrogenase (NAD(P)+)	18.29244421	1.100199311	0.577597158	1.904786571	0.05680782	0.999968657
2.6.1.97	archaeosine synthase	0.7285168	-0.497493078	4.105306542	-0.121182931	0.903546144	0.999968657
2.1.1.193	16S rRNA (uracil1498-N3)-methyltransferase	289.4001831	0.171360679	0.435296636	0.393664148	0.693829014	0.999968657
3.4.22.1	cathepsin B	63.68857474	2.218577301	1.861385702	1.191895532	0.23330221	0.999968657
4.2.1.20	tryptophan synthase	461.3621495	-0.152047245	0.263678853	-0.576637997	0.564184026	0.999968657
1.1.2.3	L-lactate dehydrogenase (cytochrome)	0.529669772	0.351288356	4.087482277	0.085942478	0.931512144	0.999968657
3.1.3.66	inositol polyphosphate-4-phosphatase	1.375871973	-0.760672779	4.09830751	-0.185606565	0.852753305	0.999968657
2.7.11.17	calcium/calmodulin-dependent protein kinase kinase 1	11.32903906	-0.519861033	4.093598465	-0.126993656	0.89894542	0.999968657
2.3.1.210	dTDP-4-amino-4,6-dideoxy-D-galactose acyltransferase	0.061162681	-0.372976067	4.141488419	-0.09005846	0.92824076	0.999968657
2.1.1.221	tRNA (guanine9-N1)-methyltransferase	3.682511275	-0.322456121	2.893116846	-0.111456308	0.911254511	0.999968657
2.3.1.202	UDP-4-amino-4,6-dideoxy-N-acetyl-beta-L-altrosamine N-acety	0.206011882	-0.950885652	4.130228034	-0.230225945	0.817916202	0.999968657
3.4.13.-	#N/A	542.4797353	-0.128153153	0.276836122	-0.462920635	0.643421256	0.999968657
3.4.24.64	mitochondrial-processing peptidase subunit alpha	2.288629601	-0.136713881	3.220594176	-0.042449894	0.966140055	0.999968657
2.7.7.73	sulfur carrier protein ThiS adenylyltransferase	11.94926618	-0.795248012	1.053349986	-0.754970354	0.45026677	0.999968657
2.8.1.1	#N/A	9.932706052	-1.43760597	0.897147556	-1.602418644	0.109063065	0.999968657
3.4.21.107	serine protease Do	631.2321672	0.200535703	0.386019561	0.519496222	0.603414747	0.999968657
3.4.17.19	carboxypeptidase Taq	71.80582444	0.405734738	0.372139588	1.09027567	0.275591729	0.999968657
3.1.26.4	ribonuclease HI	1467.073592	0.23024512	0.642702038	0.358245511	0.720159592	0.999968657
3.7.1.5	acylpyruvate hydrolase	0.424682647	-0.27167959	4.12926869	-0.065793633	0.947542125	0.999968657
2.1.1.13	5-methyltetrahydrofolate--homocysteine methyltransferase	580.1326798	-0.010253881	0.408094758	-0.025126226	0.979954281	0.999968657
1.1.1.103	threonine 3-dehydrogenase	279.8371802	1.048018095	0.58527808	1.790632746	0.073352249	0.999968657
1.8.4.2	protein-disulfide reductase (glutathione)	11.94444886	-0.313704993	4.093546626	-0.076634035	0.938914683	0.999968657
2.1.1.79	cyclopropane-fatty-acyl-phospholipid synthase	18.11968335	0.359738725	0.591339736	0.608345258	0.54295851	0.999968657
5.4.99.23	23S rRNA pseudouridine1911/1915/1917 synthase	11838.22793	1.100914994	1.110632215	0.99125073	0.321563165	0.999968657
1.2.7.1	#N/A	4974.621445	-0.426300614	0.508327584	-0.838633643	0.401674925	0.999968657
2.1.1.248	methylamine---corrinoid protein Co-methyltransferase	0.303919997	-0.62555228	4.133910308	-0.15132217	0.879721581	0.999968657
2.7.13.3	two-component system, sensor histidine kinase PdtaS	2275.191186	-0.161059364	0.274380233	-0.586993321	0.557208193	0.999968657
2.8.3.19	CoA:oxalate CoA-transferase	7.009045476	0.847086408	0.908044339	0.932868993	0.350887614	0.999968657
2.7.1.207	PTS system, lactose-specific IIA component	9.523751013	0.587787213	0.950299385	0.618528458	0.536227045	0.999968657
2.7.1.2	glucokinase	1290.074803	-0.58639941	0.510657391	-1.148322575	0.250835423	0.999968657
2.7.7.58	#N/A	3.116189468	1.542897554	2.537699837	0.607990563	0.543193733	0.999968657
6.3.2.2	glutamate--cysteine ligase	19.66428531	0.133821846	0.566660176	0.236158903	0.813309368	0.999968657
1.4.1.11	L-erythro-3,5-diaminohexanoate dehydrogenase	90.5850665	0.302797072	0.48472272	0.624680997	0.532180448	0.999968657
6.3.4.5	argininosuccinate synthase	292.8803441	-0.138339438	0.190300167	-0.726953842	0.467254232	0.999968657
3.5.1.118	gamma-glutamyl hercynylcysteine S-oxide hydrolase	0.812531649	-0.474917998	3.21830798	-0.147567604	0.882684023	0.999968657
1.5.-.-	#N/A	6.74317746	-0.200587137	0.985894234	-0.203457054	0.838777809	0.999968657
3.4.19.13	gamma-glutamyltranspeptidase / glutathione hydrolase	38.94457833	0.033858965	0.752654655	0.044986057	0.964118423	0.999968657
2.7.7.43	N-acylneuraminate cytidylyltransferase	11.83068856	-1.126234461	1.076859399	-1.045850983	0.295629838	0.999968657
2.7.1.67	phosphatidylinositol 4-kinase A	1.710812699	-0.341956203	3.548640081	-0.096362605	0.923232591	0.999968657
1.1.1.35	3-hydroxyacyl-CoA dehydrogenase	4.746715694	-0.917610434	1.317532339	-0.696461412	0.486139915	0.999968657
2.8.4.4	ribosomal protein S12 methylthiotransferase	623.2588148	-0.153231057	0.274531866	-0.558153994	0.576739234	0.999968657
1.1.1.58	tagaturonate reductase	243.3490786	-0.10415859	0.343944622	-0.302835349	0.762015343	0.999968657
2.2.1.1	transketolase	1381.337546	0.029868419	0.217943849	0.137046397	0.890994122	0.999968657
2.6.1.76	diaminobutyrate-2-oxoglutarate transaminase	5.447023162	-0.804749496	1.455607163	-0.552861731	0.580358094	0.999968657
3.4.24.40	serralysin	0.231763367	-1.014725061	4.129302346	-0.245737652	0.805885324	0.999968657
2.8.3.16	formyl-CoA transferase	2.442937505	-0.743181514	1.859082831	-0.399757075	0.68933545	0.999968657
3.1.3.97	3',5'-nucleoside bisphosphate phosphatase	1311.671976	0.362825873	0.724128338	0.501051891	0.616334604	0.999968657
4.2.1.90	L-rhamnonate dehydratase	2.379476149	-1.53980335	1.893657847	-0.813137047	0.416139488	0.999968657
3.6.1.13	ADP-ribose pyrophosphatase	291.3683185	-0.172240938	0.440036464	-0.39142424	0.695483678	0.999968657
1.1.-.-	#N/A	107.6860614	-0.307868909	0.490407453	-0.627781873	0.530146843	0.999968657
3.1.1.11	pectinesterase	172.979135	0.372828462	0.489793907	0.761194569	0.446540861	0.999968657
4.1.1.81	threonine-phosphate decarboxylase	111.5133339	-0.474062357	0.325140438	-1.458023371	0.144834101	0.999968657
2.3.2.18	peptidoglycan pentaglycine glycine transferase (the fourth and	0.020495196	-0.019393708	4.142686886	-0.004681432	0.996264771	0.999968657

3.5.4.28	5-methylthioadenosine/S-adenosylhomocysteine deaminase	646.4515798	-0.556011874	0.774015127	-0.718347555	0.472543015	0.999968657
1.6.5.8	#N/A	428.1980446	-0.168124771	0.437791485	-0.384029331	0.700956708	0.999968657
5.3.2.6	4-oxalocrotonate tautomerase	9.196708722	-0.157246755	1.277807289	-0.123059836	0.902059716	0.999968657
1.2.4.1	pyruvate dehydrogenase (acetyl-transferring)	104.4906236	0.372526967	0.812937271	0.458248109	0.6467742	0.999968657
3.5.3.23	succinylarginine dihydrolase	0.099032361	-0.030704248	4.143282072	-0.00741061	0.994087243	0.999968657
5.1.99.8	7,8-dihydroneopterin aldolase/epimerase/oxygenase	59.65369116	0.036385595	0.600350766	0.060607227	0.951672018	0.999968657
1.1.1.37	malate dehydrogenase	310.0391925	-0.365363853	0.404825459	-0.902521928	0.366779679	0.999968657
2.7.1.197	PTS system, mannitol-specific IIA component	35.32517345	-0.049185046	0.533728742	-0.092153639	0.926575972	0.999968657
3.6.1.7	acylphosphatase	266.5174633	-0.865633913	0.654442136	-1.322705041	0.185933486	0.999968657
2.1.1.220	tRNA (adenine57-N1/adenine58-N1)-methyltransferase catalyt	3.95831283	-0.54601632	1.721244945	-0.317221742	0.751075353	0.999968657
2.7.4.22	uridylate kinase	517.0140077	-0.006344336	0.265078873	-0.023933769	0.980905438	0.999968657
1.3.1.88	tRNA-dihydrouridine synthase 1	0.249773869	-0.816552953	4.124382892	-0.19798185	0.843059265	0.999968657
1.1.1.22	UDPglucose 6-dehydrogenase	273.6119241	0.099114925	0.34655789	0.285998177	0.774879518	0.999968657
4.2.1.10	#N/A	252.2617019	-0.336552821	0.2761644	-1.218668378	0.222970085	0.999968657
3.4.19.-	#N/A	72.96599458	0.154330446	0.422810403	0.365010996	0.715103198	0.999968657
3.2.1.184	GDP/UDP-N,N'-diacetylbacillosamine 2-epimerase (hydrolysing)	9.274406239	0.037311243	0.793184664	0.047039794	0.962481512	0.999968657
3.4.22.37	gingipain R	0.847080334	4.820162165	4.044817972	1.191688278	0.233383496	0.999968657
6.3.5.6	aspartyl-tRNA(Asn)/glutamyl-tRNA(Gln) amidotransferase subu	500.7375315	0.524623205	0.456939147	1.148124882	0.250917014	0.999968657
2.1.1.320	type II protein arginine methyltransferase	0.416289782	-0.888208298	4.112570367	-0.215974006	0.829008009	0.999968657
4.1.1.4	acetoacetate decarboxylase	0.197769159	0.948748183	4.136917486	0.229336985	0.81860701	0.999968657
4.6.1.13	1-phosphatidylinositol phosphodiesterase	17.26687161	-0.638634781	0.90924899	-0.702376124	0.482444631	0.999968657
3.4.11.6	aminopeptidase B	12.58645714	-1.25979012	1.402412219	-0.898302299	0.369024407	0.999968657
3.2.2.21	DNA-3-methyladenine glycosylase II	50.9815646	0.144941104	0.400995802	0.361452922	0.717760892	0.999968657
2.5.1.61	hydroxymethylbilane synthase	68.00050909	0.283880264	0.366313873	0.774964545	0.43836061	0.999968657
6.1.1.13	D-alanine--poly(phosphoribitol) ligase subunit 1	16.40246966	-0.10565827	0.64243546	-0.16446519	0.869364948	0.999968657
4.1.1.20	diaminopimelate decarboxylase	541.1671212	-0.525657036	0.430023255	-1.222392114	0.22155938	0.999968657
6.3.4.4	adenylosuccinate synthase	684.2271798	-0.14149169	0.296868954	-0.476613295	0.633637514	0.999968657
3.6.5.4	signal recognition particle subunit SRP54	622.0072358	-0.767335237	0.41588003	-1.845087962	0.065024744	0.999968657
1.1.1.132	GDP-mannose 6-dehydrogenase	5.365709567	-0.896085696	2.003220956	-0.447322445	0.654642263	0.999968657
5.3.1.5	xylose isomerase	119.6721669	0.522825796	0.453150314	1.15375799	0.248599402	0.999968657
4.2.2.23	rhamnogalacturonan endolyase	83.38770096	0.126500668	0.561548461	0.225271151	0.821768341	0.999968657
1.17.7.4	4-hydroxy-3-methylbut-2-en-1-yl diphosphate reductase	340.093813	-0.445851213	0.417924952	-1.066821236	0.286052567	0.999968657
4.1.1.74	indolepyruvate decarboxylase	0.623578467	-2.345264141	4.113038938	-0.570202271	0.568540516	0.999968657
4.2.2.7	heparin lyase	6.977243599	-0.640636285	1.236932735	-0.517923301	0.604511784	0.999968657
4.2.1.19	imidazoleglycerol-phosphate dehydratase / histidinol-phosphat	179.9053962	-0.052112545	0.20727111	-0.251422133	0.801487757	0.999968657
6.3.-.-	#N/A	0.185059353	0.747482268	4.125780339	0.181173549	0.856231356	0.999968657
2.7.12.2	mitogen-activated protein kinase kinase 1	0.552060613	-0.904823566	4.107552161	-0.220282916	0.825650825	0.999968657
2.7.1.107	diacylglycerol kinase (ATP)	109.2477564	-0.710073975	0.413883635	-1.715636752	0.086228547	0.999968657
5.1.3.14	UDP-N-acetylglucosamine 2-epimerase (non-hydrolysing)	267.9615043	-0.006829775	0.227167047	-0.03006499	0.976015222	0.999968657
3.4.11.-	#N/A	205.27062	-0.009414306	0.376014091	-0.02503711	0.980025363	0.999968657
3.1.3.89	5'-deoxynucleotidase	496.8947972	-0.633830454	1.075992984	-0.589065601	0.555817268	0.999968657
4.1.1.41	#N/A	2.58784862	-0.391739429	3.146177783	-0.124512807	0.90090926	0.999968657
3.1.3.57	inositol polyphosphate 1-phosphatase	0.363965079	1.678303067	4.112280504	0.408119793	0.68318573	0.999968657
2.4.1.18	1,4-alpha-glucan branching enzyme	1083.344365	-0.080052151	0.287332205	-0.278604867	0.780548077	0.999968657
4.1.1.37	uroporphyrinogen decarboxylase	83.4923204	0.526400278	0.557174618	0.944767153	0.344777765	0.999968657
2.7.1.195	PTS system, 2-O-A-mannosyl-D-glycerate-specific IIA componer	0.248796225	-1.375070263	4.12644119	-0.333233942	0.738957699	0.999968657
1.11.1.5	cytochrome c peroxidase	35.45100758	0.539546828	0.58369134	0.92437011	0.355293651	0.999968657
5.1.1.20	L-Ala-D/L-Glu epimerase	206.6439338	1.3080606	0.643540307	2.032600888	0.042092862	0.999968657
2.6.1.109	8-amino-3,8-dideoxy-alpha-D-manno-octulosonate transaminas	0.052512875	-0.340236249	4.139416356	-0.082194256	0.934492241	0.999968657
3.5.99.2	hydroxymethylpyrimidine/phosphomethylpyrimidine kinase / t	29.66472934	0.271866625	0.728663431	0.373103155	0.70907167	0.999968657
3.2.1.86	#N/A	1540.806296	-0.139703271	0.555160776	-0.251644708	0.801315698	0.999968657
5.4.99.62	D-ribose pyranase	11.97500773	0.62224718	0.99706376	0.624079627	0.532575293	0.999968657
6.3.2.4	D-alanine-D-alanine ligase	331.7670026	-0.077104452	0.189816754	-0.406204669	0.684592224	0.999968657
1.8.1.7	glutathione reductase (NADPH)	5.508827407	-0.44203086	1.259815402	-0.350869548	0.725686218	0.999968657
2.7.7.79	tRNA(His) guanylyltransferase	1.231597149	-0.290559265	4.100102508	-0.070866342	0.943504131	0.999968657
5.1.3.8	N-acylglucosamine 2-epimerase	139.7091188	-0.889442271	0.437369233	-2.033618748	0.04199005	0.999968657
2.7.4.3	adenylate kinase	755.136847	-0.055498861	0.351986663	-0.157673192	0.874714326	0.999968657
2.4.1.247	beta-D-galactosyl-(1->4)-L-rhamnose phosphorylase	2.458672492	-2.069843973	1.904201373	-1.086987964	0.277042129	0.999968657
6.1.1.18	glutaminyl-tRNA synthetase	439.2984639	-0.173299158	0.184083337	-0.941416867	0.346491274	0.999968657
1.1.5.3	glycerol-3-phosphate dehydrogenase	393.6201507	0.542200679	0.331388858	1.636146376	0.101808956	0.999968657
2.3.1.247	3-keto-5-aminohexanoate cleavage enzyme	82.61576769	0.099671229	0.479529246	0.207852243	0.83534434	0.999968657
1.1.1.380	L-gulonate 5-dehydrogenase	17.29678145	-0.469087497	0.766325223	-0.612125874	0.54045448	0.999968657
1.1.1.262	4-hydroxythreonine-4-phosphate dehydrogenase	161.576444	-0.087516465	0.252959458	-0.345970321	0.72936502	0.999968657
2.1.2.2	phosphoribosylglycinamide formyltransferase	412.4568963	-0.1073687	0.117187724	-0.916211161	0.359556153	0.999968657
2.1.1.131	precorrin-3B C17-methyltransferase	100.1396827	-0.200481721	0.357707825	-0.560462218	0.575164204	0.999968657
2.3.1.35	glutamate N-acetyltransferase / amino-acid N-acetyltransferase	176.3579632	0.035473584	0.248150062	0.142952148	0.886327973	0.999968657
2.7.1.151	#N/A	2.104401683	-0.484032733	4.096898427	-0.11814614	0.905951865	0.999968657
2.3.1.12	pyruvate dehydrogenase E2 component (dihydrolipoamide ace	87.93707027	0.568371507	0.850026983	0.668651135	0.503718045	0.999968657
4.1.2.13	fructose 1,6-bisphosphate aldolase/phosphatase	1639.603458	-0.584674798	0.467508035	-1.250619784	0.211073229	0.999968657
2.4.1.186	glycogenin	0.70344152	0.988715026	4.115523145	0.240240424	0.810143877	0.999968657
3.1.3.81	phosphatidylglycerophosphatase B	0.236562323	0.258174385	4.122912961	0.062619412	0.950069571	0.999968657
5.3.3.3	4-hydroxybutyryl-CoA dehydratase / vinylacetyl-CoA-Delta-ison	56.64405634	-0.367234314	0.41910881	-0.876226663	0.380906824	0.999968657
1.2.1.21	lactaldehyde dehydrogenase / glycolaldehyde dehydrogenase	20.27192498	-0.458357	0.556198471	-0.824088925	0.409889032	0.999968657
3.6.3.41	heme exporter protein A	3.666482455	0.282236899	1.49326815	0.189006174	0.850087978	0.999968657
2.7.10.-	#N/A	1.425729739	0.053966034	1.529670241	0.035279522	0.971856853	0.999968657
5.3.1.24	phosphoribosylanthranilate isomerase	103.2707371	-0.198854647	0.490461168	-0.405444223	0.68515101	0.999968657
5.4.99.18	5-(carboxyamino)imidazole ribonucleotide mutase	295.3770587	-0.272905943	0.237422148	-1.149454444	0.250368641	0.999968657
2.6.1.36	L-lysine 6-transaminase	2.906010432	3.798239244	3.757858471	1.010745688	0.312138164	0.999968657
2.5.1.-	#N/A	278.6566298	0.692189787	0.68932614	1.00415427	0.31530426	0.999968657
2.7.7.2	FAD synthetase	334.3661825	0.077209675	0.228638124	0.33769379	0.735593955	0.999968657
2.5.1.21	farnesyl-diphosphate farnesyltransferase	3.986138423	1.660881165	1.655751022	1.003098379	0.315813397	0.999968657
4.2.1.47	GDPmannose 4,6-dehydratase	191.2217744	-0.001052712	0.431295668	-0.002440812	0.998052515	0.999968657
2.3.1.19	phosphate butyryltransferase	55.23559176	-0.709503108	0.477520913	-1.485805311	0.137330625	0.999968657
1.7.1.13	7-cyano-7-deazaguanine reductase	60.68859191	-0.074810341	0.55619846	-0.134502964	0.893004868	0.999968657
6.1.1.1	tyrosyl-tRNA synthetase	369.2065589	0.035972605	0.219521985	0.163867892	0.869835144	0.999968657
4.2.1.33	3-isopropylmalate dehydratase	590.1564115	-0.249592804	0.155679368	-1.603249085	0.108879672	0.999968657
4.1.1.68	#N/A	0.157074332	0.320813212	4.143731151	0.077421338	0.938288366	0.999968657

3.4.11.23	PepB aminopeptidase	3.030118032	-0.201119138	2.72654854	-0.073763271	0.941198753	0.999968657
3.1.3.1	alkaline phosphatase	149.18058	-0.173642826	0.417497936	-0.415913017	0.677473654	0.999968657
2.7.8.26	adenosylcobinamide-GDP ribazoletransferase	117.5949016	-0.144822573	0.394554776	-0.367053149	0.713579374	0.999968657
1.1.3.15	glycolate oxidase	168.3993579	0.336032124	0.413191289	0.813260425	0.416068762	0.999968657
6.3.2.36	4-phosphopantoate---beta-alanine ligase	2.229993848	-0.479696748	4.096620612	-0.117095722	0.9067842	0.999968657
3.1.3.16	protein phosphatase	407.6783791	0.163141185	0.284286804	0.573861265	0.566061686	0.999968657
1.4.1.16	diaminopimelate dehydrogenase	191.3264728	-0.436964793	0.34464582	-1.267866221	0.204845729	0.999968657
2.4.1.337	1,2-diacylglycerol 3-alpha-glucosyltransferase	134.8407682	-0.707645966	0.531823177	-1.330603848	0.183319396	0.999968657
3.1.3.5	5'-nucleotidase	334.6478068	0.618404452	0.321977567	1.920644527	0.054776538	0.999968657
2.7.9.3	selenide, water dikinase	162.5069829	-0.060903475	0.342023632	-0.178068031	0.858669548	0.999968657
3.2.1.52	beta-N-acetylhexosaminidase	1969.843787	-0.521866085	0.405019319	-1.28849677	0.197573092	0.999968657
3.1.1.45	carboxymethylenebutenolidase	0.306481125	1.51577494	4.122138516	0.367715673	0.713085252	0.999968657
3.6.3.27	#N/A	174.0743809	-0.420917801	0.530694566	-0.793145112	0.427693282	0.999968657
5.4.2.10	phosphoglucosamine mutase	492.1079234	0.072768756	0.270959324	0.268559705	0.788268524	0.999968657
3.8.1.5	haloalkane dehalogenase	0.039493742	1.031690707	4.143245313	0.24900546	0.803356558	0.999968657
6.5.1.4	RNA 3'-terminal phosphate cyclase (ATP)	0.559597965	2.393104992	4.067013497	0.588418257	0.556251587	0.999968657
3.4.21.89	signal peptidase I	629.1568785	-0.337116927	0.219648858	-1.534799359	0.12483312	0.999968657
2.7.11.24	mitogen-activated protein kinase 1/3	19.47956978	-0.691685706	4.093067184	-0.16898958	0.865804837	0.999968657
3.6.1.66	XTP/dITP diphosphohydrolase	350.6006094	0.268909449	0.301208864	0.892767383	0.371981757	0.999968657
4.1.3.36	naphthoate synthase	60.70473352	-0.081638333	0.465932047	-0.175215105	0.860910616	0.999968657
2.1.1.172	16S rRNA (guanine1207-N2)-methyltransferase	6.81937072	2.023073128	1.106742326	1.827953155	0.067556582	0.999968657
2.7.11.1	checkpoint serine/threonine-protein kinase	1323.478779	-0.226518255	0.247869147	-0.913862243	0.360789236	0.999968657
3.6.3.34	iron complex transport system ATP-binding protein	291.8109359	-0.014991571	0.379558337	-0.039497408	0.96849382	0.999968657
2.5.1.6	S-adenosylmethionine synthetase	664.9956883	0.328497993	0.406096445	0.808916199	0.41856335	0.999968657
2.5.1.54	3-deoxy-7-phosphoheptulonate synthase	4039.606101	-0.311572063	1.021039342	-0.305151869	0.760250488	0.999968657
2.7.7.8	polyribonucleotide nucleotidyltransferase	1007.659678	0.263675946	0.250370707	1.053142155	0.292275845	0.999968657
3.5.4.5	cytidine deaminase	296.4043363	-0.33163284	0.276057942	-1.201316063	0.229628622	0.999968657
5.5.1.27	D-galactarolactone cycloisomerase	0.422036594	-1.833210578	4.120551487	-0.444894472	0.656396018	0.999968657
3.2.1.25	beta-mannosidase	235.1877339	0.259540442	0.283572327	0.915253067	0.360058791	0.999968657
2.1.1.148	thymidylate synthase (FAD)	157.2032252	-0.070817956	0.317735447	-0.222883398	0.823626258	0.999968657
3.6.3.2	Mg2+-importing ATPase	52.92754109	-0.229974389	0.587196221	-0.391648278	0.695318111	0.999968657
4.1.1.70	glutaconyl-CoA decarboxylase	137.1694256	0.539129867	0.559945147	0.962826217	0.335634742	0.999968657
2.7.4.9	dTMP kinase	164.5735539	0.371993788	0.286702679	1.297489751	0.194462729	0.999968657
6.2.1.34	feruloyl-CoA synthase	0.103724382	1.515369364	4.135487834	0.366430618	0.714043776	0.999968657
2.7.1.176	UDP-N-acetylglucosamine kinase	2.748491948	1.895087744	1.267170996	1.495526452	0.134777102	0.999968657
2.4.1.291	N-acetylgalactosamine-N,N'-diacetylbacillosaminyl-diphospho-u	1.395090128	-1.10456197	4.101124253	-0.269331506	0.78767459	0.999968657
2.7.1.30	glycerol kinase	776.031683	0.194101435	0.35643487	0.544563541	0.586053736	0.999968657
2.9.1.1	L-seryl-tRNA(Ser) seleniumtransferase	31.33378398	0.46425527	0.663771796	0.69942	0.484289592	0.999968657
1.4.3.16	L-aspartate oxidase	271.3226124	-0.118828157	0.276995699	-0.428989179	0.667931099	0.999968657
3.5.1.102	2-amino-5-formylamino-6-ribosylaminopyrimidin-4(3H)-one 5'-r	0.581356147	0.002057609	4.103654208	0.000501409	0.999599934	0.999968657
3.2.1.8	endo-1,4-beta-xylanase	983.5067301	1.422632943	0.802312258	1.773166156	0.076201178	0.999968657
2.4.1.257	alpha-1,3/alpha-1,6-mannosyltransferase	1.814404281	0.036188386	4.097078152	0.00883273	0.992952593	0.999968657
2.7.4.31	5-(aminomethyl)-3-furanmethanol phosphate kinase	2.223575051	0.00800167	4.023257489	0.001988853	0.998413126	0.999968657
2.1.1.10	homocysteine S-methyltransferase	5.268345962	-0.417246766	1.289659401	-0.323532528	0.746291977	0.999968657
3.6.3.32	glycine betaine/proline transport system ATP-binding protein	73.99996966	-0.440826853	0.626904903	-0.703179782	0.481943717	0.999968657
2.5.1.89	tritrans,polycis-undecaprenyl-diphosphate synthase [geranylge	0.762496501	-0.657410435	4.103213124	-0.160218447	0.872708998	0.999968657
1.17.1.8	4-hydroxy-tetrahydrodipicolinate reductase	419.5572778	-0.088754999	0.278852549	-0.318286491	0.750267631	0.999968657
3.1.1.83	epsilon-lactone hydrolase	8.852331657	0.277614705	0.909439646	0.305259075	0.760168842	0.999968657
2.1.1.80	chemotaxis protein methyltransferase CheR	51.42789199	0.422270849	0.62914548	0.671181566	0.50210487	0.999968657
1.3.1.91	tRNA-dihydrouridine synthase 2	0.173834568	0.698060943	4.118491844	0.169494312	0.865407846	0.999968657
2.3.1.15	glycerol-3-phosphate O-acyltransferase 1/2	513.1341149	-0.24592359	0.232506395	-1.057706781	0.290189143	0.999968657
1.17.1.1	CDP-4-dehydro-6-deoxyglucose reductase, E3	51.88480358	0.451595892	0.444970028	1.014890584	0.310157987	0.999968657
1.4.4.2	glycine dehydrogenase	313.8763903	0.154566928	0.41970502	0.368275146	0.712668083	0.999968657
2.8.1.9	molybdenum cofactor sulfurtransferase	0.582805694	-0.907411673	4.106777413	-0.220954676	0.825127724	0.999968657
4.1.1.32	phosphoenolpyruvate carboxykinase (GTP)	256.1271171	0.749432718	0.590366248	1.269436931	0.204285271	0.999968657
1.3.99.5	#N/A	0.280447894	0.178268188	4.134849875	0.043113582	0.965610993	0.999968657
2.3.1.30	serine O-acetyltransferase	298.4087406	-0.23773507	0.281756619	-0.843760375	0.398803322	0.999968657
3.2.1.-	#N/A	106.9534303	0.238017949	0.415104593	0.573392713	0.566378823	0.999968657
5.4.99.15	(1->4)-alpha-D-glucan 1-alpha-D-glucosylmutase	0.103724382	1.515369364	4.135487834	0.366430618	0.714043776	0.999968657
4.2.99.20	2-succinyl-6-hydroxy-2,4-cyclohexadiene-1-carboxylate synthas	4.655924674	0.04564956	1.483353705	0.030774562	0.975449328	0.999968657
4.2.1.9	dihydroxy-acid dehydratase	494.2953836	-0.200004658	0.252361307	-0.79253298	0.42804997	0.999968657
6.3.4.2	CTP synthase	447.5705848	0.116013081	0.229567849	0.505354219	0.613310068	0.999968657
1.12.99.-	#N/A	35.47611908	0.169806344	1.41726206	0.119812947	0.904631326	0.999968657
3.5.1.94	gamma-glutamyl-gamma-aminobutyrate hydrolase	0.020495196	-0.019393708	4.142686886	-0.004681432	0.996264771	0.999968657
1.1.1.363	glucose-6-phosphate 1-dehydrogenase	351.7397334	-0.624512157	0.638090515	-0.978720326	0.327718186	0.999968657
2.4.1.129	monofunctional glycosyltransferase	61.93255458	0.670740452	0.359180779	1.867417443	0.061843316	0.999968657
4.3.1.4	formiminotetrahydrofolate cyclodeaminase	55.2180708	0.600009319	0.662662465	0.905452399	0.365225767	0.999968657
2.1.1.268	tRNAThr (cytosine32-N3)-methyltransferase	0.833542549	-0.539958607	4.103404785	-0.131587946	0.89531022	0.999968657
2.7.1.21	thymidine kinase	135.8458384	0.375675536	0.388687369	0.966523654	0.333782217	0.999968657
3.4.23.5	cathepsin D	0.897866547	0.382515232	4.117358968	0.092903056	0.925980578	0.999968657
2.4.1.281	4-O-beta-D-mannosyl-D-glucose phosphorylase	101.0610914	0.331269176	0.534790595	0.619437176	0.535628397	0.999968657
3.1.3.32	bifunctional polynucleotide phosphatase/kinase	0.714858879	-0.835354423	3.79724853	-0.219989399	0.825879411	0.999968657
5.1.1.7	diaminopimelate epimerase	534.3515027	-0.119366902	0.596703624	-0.20004387	0.841446271	0.999968657
2.1.1.314	diphthine methyl ester synthase	1.408052998	-0.38145816	3.770784821	-0.101161476	0.919422277	0.999968657
2.1.1.206	tRNA (cytidine56-2'-O)-methyltransferase	0.478742931	-0.311534543	4.115866711	-0.075691115	0.939664845	0.999968657
1.1.1.291	2-hydroxymethylglutarate dehydrogenase	1.165624298	-0.557345736	4.100408353	-0.135924446	0.891881009	0.999968657
2.5.1.48	cystathionine gamma-synthase	39.17001989	0.119180674	0.675870138	0.176336648	0.860029473	0.999968657
6.3.3.1	phosphoribosylformylglycinamidine cyclo-ligase	403.1993717	-0.08509727	0.170131923	-0.500184022	0.616945508	0.999968657
6.3.2.12	#N/A	407.8113641	-0.208950715	0.197372649	-1.058660946	0.28975422	0.999968657
2.8.1.12	molybdopterin synthase catalytic subunit	4.69347111	1.30974279	1.46548421	0.893726989	0.371467982	0.999968657
3.6.4.6	vesicle-fusing ATPase	4.693647225	0.898156013	2.028891952	0.442683018	0.657995027	0.999968657
1.21.4.1	#N/A	273.8860975	-0.600233726	0.665124853	-0.902437675	0.366824415	0.999968657
2.7.1.130	tetraacyldisaccharide 4'-kinase	131.049192	-0.409346147	0.490840145	-0.833970388	0.404297671	0.999968657
1.2.1.8	betaine-aldehyde dehydrogenase	10.0171133	1.168329697	1.10151127	1.060660683	0.288844134	0.999968657
1.1.1.31	3-hydroxyisobutyrate dehydrogenase	19.43101592	0.621998753	0.389218987	1.598068887	0.110027647	0.999968657
2.4.1.320	beta-1,4-mannooligosaccharide/beta-1,4-mannosyl-N-acetylglu	163.8803014	0.149150962	0.350301357	0.425779002	0.670268889	0.999968657
2.7.6.1	ribose-phosphate pyrophosphokinase	590.4496245	-0.08440744	0.339082644	-0.248928814	0.803415847	0.999968657

4.1.2.20	2-dehydro-3-deoxyglucarate aldolase	2.922842527	0.217167545	1.3289772	0.163409534	0.870195996	0.999968657
1.1.1.86	ketol-acid reductoisomerase	435.8011911	-0.254124165	0.315455264	-0.80557909	0.420485586	0.999968657
5.1.3.3	aldose 1-epimerase	759.9440507	-0.043636129	0.372605115	-0.117110923	0.906772154	0.999968657
5.3.1.28	D-sedoheptulose 7-phosphate isomerase	48.71117419	0.0457096	0.433290038	0.105494233	0.915983646	0.999968657
2.3.1.47	8-amino-7-oxononanoate synthase	86.46061388	-0.801638673	0.662113862	-1.21072631	0.226000316	0.999968657
2.4.99.13	3-deoxy-D-manno-octulosonic-acid transferase	104.6062867	0.006585804	0.474861753	0.013868887	0.988934584	0.999968657
3.1.3.23	sugar-phosphatase	0.6779552	6.443629386	4.069602763	1.583355861	0.113340377	0.999968657
3.2.1.170	mannosylglycerate hydrolase	9.078498944	0.665102624	1.476342077	0.450507124	0.652344818	0.999968657
3.4.13.9	Xaa-Pro dipeptidase	53.83327966	-1.191207355	0.612769369	-1.943973403	0.051898659	0.999968657
2.4.2.48	7-cyano-7-deazaguanine tRNA-ribosyltransferase	0.499547738	-0.899291794	4.109214446	-0.218847618	0.826768752	0.999968657
3.6.1.26	CDP-diacylglycerol pyrophosphatase	0.081334971	-0.381289104	4.142921482	-0.092033872	0.926671128	0.999968657
2.1.1.290	tRNA wybutosine-synthesizing protein 4	0.052512875	-0.340236249	4.139416356	-0.082194256	0.934492241	0.999968657
2.1.1.229	alkylated DNA repair protein alkB homolog 8	0.035008583	-0.204325554	4.14032189	-0.049350161	0.960640245	0.999968657
2.1.1.294	O-antigen chain-terminating methyltransferase	2.5617666	0.895331743	3.782517315	0.236702616	0.812887505	0.999968657
1.14.14.5	alkanesulfonate monooxygenase	0.151201469	-0.675915724	4.137728292	-0.163354304	0.87023948	0.999968657
1.1.1.41	isocitrate dehydrogenase (NAD+)	37.24360312	-0.213611744	0.397734672	-0.537070966	0.591218595	0.999968657
2.3.1.24	Acyl-CoA-dependent ceramide synthase	1.915657591	-0.550568836	4.097337729	-0.134372334	0.893108157	0.999968657
2.7.1.5	rhamnulokinase	286.9725374	-0.384509674	0.366904237	-1.047983736	0.2946461	0.999968657
2.7.1.189	autoinducer-2 kinase	13.76266685	0.616516247	0.845592686	0.729093638	0.465944386	0.999968657
2.4.-.-	#N/A	68.04439895	0.195108093	0.518339349	0.376409959	0.706612143	0.999968657
3.2.2.9	adenosylhomocysteine nucleosidase	243.1991387	0.180253761	0.301982734	0.596900883	0.550573558	0.999968657
2.8.3.1	propionate CoA-transferase	89.25752976	-0.303108563	0.525582454	-0.576709821	0.564135497	0.999968657
4.1.1.48	indole-3-glycerol phosphate synthase	96.2007013	0.028967358	0.472706681	0.061279772	0.9511364	0.999968657
3.5.3.18	dimethylargininase	1.226516595	0.528507874	2.704691803	0.195404102	0.845076605	0.999968657
2.8.1.2	thiosulfate/3-mercaptopyruvate sulfurtransferase	7.144411477	-1.011965079	1.033463694	-0.979197513	0.327482397	0.999968657
2.1.3.-	#N/A	2.822043806	-2.885482534	2.617206903	-1.102504556	0.270242379	0.999968657
2.8.1.-	#N/A	58.92031517	0.137882107	0.69731154	0.197733867	0.843253291	0.999968657
3.6.3.7	#N/A	11.08064655	0.219378978	0.859962526	0.255102951	0.798643585	0.999968657
2.7.1.52	fucokinase	57.1794349	-0.270357502	0.649779436	-0.416075806	0.677354533	0.999968657
3.1.26.8	ribonuclease M5	72.41566023	0.150789156	0.351901045	0.428498745	0.668288047	0.999968657
4.1.3.30	methylisocitrate lyase	0.651710156	0.161725988	4.059419023	0.039839688	0.968220935	0.999968657
3.5.1.24	choloylglycine hydrolase	410.6257429	0.363230235	0.398342646	0.911853749	0.361845712	0.999968657
2.1.1.35	tRNA (uracil-5-)-methyltransferase	0.644054713	-0.252548732	4.059512584	-0.06221159	0.950394333	0.999968657
5.4.2.6	beta-phosphoglucomutase	14.7895933	-0.622757453	0.765391361	-0.813645783	0.415847902	0.999968657
2.5.1.29	geranylgeranyl diphosphate synthase, type III	364.885785	-0.208400972	0.191136059	-1.090327872	0.275568742	0.999968657
2.6.1.45	#N/A	1.130264311	1.533922882	3.557283952	0.431206196	0.666318449	0.999968657
1.3.1.93	very-long-chain enoyl-CoA reductase	0.999108385	-0.512244356	4.101927615	-0.124878936	0.900619394	0.999968657
1.1.1.69	gluconate 5-dehydrogenase	308.5135377	-0.014663158	0.310241671	-0.047263662	0.962303089	0.999968657
1.1.1.-	#N/A	3841.379493	-0.831931047	0.786554936	-1.057689691	0.290196937	0.999968657
2.7.1.146	ADP-dependent phosphofructokinase/glucokinase	1.596352927	-0.802041453	2.962026581	-0.270774563	0.786564425	0.999968657
5.4.3.8	glutamate-1-semialdehyde 2,1-aminomutase	119.9576647	0.176710948	0.4186557	0.422091347	0.672958344	0.999968657
1.1.99.1	choline dehydrogenase	0.295593336	0.286976019	4.1141196	0.069753932	0.944389515	0.999968657
3.6.3.17	simple sugar transport system ATP-binding protein	2360.199292	-0.068761057	0.399113697	-0.172284384	0.86321396	0.999968657
1.4.1.24	3-dehydroquinate synthase II	3.146725448	0.008688197	1.783888625	0.00487037	0.996114023	0.999968657
4.2.2.17	inulin fructotransferase (DFA-I-forming)	0.177059089	-0.100818958	4.141271084	-0.024344931	0.980577474	0.999968657
3.6.3.29	molybdate transport system ATP-binding protein	78.3810439	-0.126375833	0.536431187	-0.235586289	0.813753714	0.999968657
3.1.3.64	myotubularin	3.613934552	-0.236662799	2.80260551	-0.08444385	0.932703544	0.999968657
3.1.3.3	phosphoserine phosphatase	138.4424214	-0.129361398	0.397742069	-0.325239415	0.74499988	0.999968657
2.7.8.37	alpha-D-ribose 1-methylphosphonate 5-triphosphate synthase	11.54475179	-1.143750184	1.377372755	-0.830385369	0.406320935	0.999968657
2.1.1.215	tRNA (guanine26-N2/guanine27-N2)-dimethyltransferase	2.22570527	0.211161424	3.266798309	0.064638648	0.948461713	0.999968657
2.6.1.51	alanine-glyoxylate transaminase / serine-glyoxylate transamina	1.130264311	1.533922882	3.557283952	0.431206196	0.666318449	0.999968657
1.2.4.4	3-methyl-2-oxobutanoate dehydrogenase (2-methylpropanoyl-	106.8812829	-0.10519811	0.538981108	-0.195179587	0.845252359	0.999968657
3.4.22.15	cathepsin L	89.02316587	3.797623186	1.550755889	2.448885225	0.01432991	0.999968657
3.1.2.28	1,4-dihydroxy-2-naphthoyl-CoA hydrolase	0.020495196	-0.019393708	4.142686886	-0.004681432	0.996264771	0.999968657
2.7.1.203	PTS system, D-glucosaminate-specific IIA component	3.533453409	-0.080754841	1.214216511	-0.066507777	0.946973566	0.999968657
3.2.1.187	beta-L-arabinobiosidase	5.256782339	-2.457577435	1.700871911	-1.444892716	0.148488023	0.999968657
3.2.1.80	fructan beta-fructosidase	126.3111575	-0.076603663	0.418675001	-0.182966891	0.854823997	0.999968657
1.12.1.4	iron-hydrogenase subunit alpha	0.249773869	-0.816552953	4.124382892	-0.19798185	0.843059265	0.999968657
5.1.3.32	L-rhamnose mutarotase	119.1287929	-0.448928025	0.370841144	-1.210566928	0.226061426	0.999968657
2.3.1.199	elongation of very long chain fatty acids protein 6	0.416302691	-3.563547513	4.143731151	-0.859985212	0.389797194	0.999968657
2.1.1.184	23S rRNA (adenine-N6)-dimethyltransferase	38.39874982	0.029114336	0.438387867	0.066412275	0.947049598	0.999968657
5.4.3.3	beta-lysine 5,6-aminomutase alpha subunit	144.6425607	0.473011766	0.481405451	0.98256421	0.325821966	0.999968657
2.7.1.39	homoserine kinase	148.0990502	0.096571521	0.293289667	0.32927011	0.741951534	0.999968657
1.3.1.87	2,3-dihydroxy-2,3-dihydrophenylpropionate dehydrogenase	0.111464417	1.546221436	4.136560128	0.373794019	0.708557569	0.999968657
2.4.1.142	beta-1,4-mannosyltransferase	2.114580348	-0.512426296	4.090741929	-0.12526488	0.900313855	0.999968657
2.4.1.258	alpha-1,3-mannosyltransferase	0.723054713	-0.175355421	4.101419616	-0.042754811	0.965896987	0.999968657
2.1.1.282	tRNA wybutosine-synthesizing protein 3	0.510438089	-0.504141442	4.110156071	-0.122657494	0.902378325	0.999968657
4.-.-.-	#N/A	0.043561417	0.320814203	4.143731151	0.077421578	0.938288176	0.999968657
4.1.99.3	deoxyribodipyrimidine photo-lyase	0.334769309	-1.165785104	4.123165802	-0.282740292	0.777375927	0.999968657
4.1.2.9	xylulose-5-phosphate/fructose-6-phosphate phosphoketolase	113.3229537	0.620463091	0.300501517	2.064758597	0.038945847	0.999968657
2.7.1.43	glucuronokinase	1.764226465	-0.155055124	4.096627725	-0.037849454	0.969807714	0.999968657
1.1.1.157	3-hydroxybutyryl-CoA dehydrogenase	1028.321371	-0.545602776	0.558381499	-0.977114709	0.328512366	0.999968657
1.1.1.369	myo-inositol 2-dehydrogenase / D-chiro-inositol 1-dehydrogena	75.32692191	0.665546849	0.47460571	1.402315301	0.160821113	0.999968657
1.1.1.34	hydroxymethylglutaryl-CoA reductase (NADPH)	5.653749803	-0.310633541	2.571678238	-0.120790205	0.903857209	0.999968657
3.4.22.34	legumain	232.7625314	1.028077837	1.482437666	0.693504935	0.48799273	0.999968657
2.1.1.86	#N/A	31.96680807	0.442546877	1.684335296	0.262742744	0.792748869	0.999968657
1.14.12.17	nitric oxide dioxygenase	9.04465669	0.492367631	1.045013592	0.471159069	0.637527146	0.999968657
2.7.8.-	#N/A	803.4885897	0.029264117	0.259994704	0.112556589	0.910382103	0.999968657
3.1.5.1	dGTPase	378.9543563	-0.247769646	0.244134637	-1.014889364	0.310158569	0.999968657
3.4.24.-	#N/A	2958.235347	0.106020472	0.175433255	0.604335092	0.54562088	0.999968657
2.1.2.13	UDP-4-amino-4-deoxy-L-arabinose formyltransferase / UDP-glu	1.285501156	-0.978818524	2.014779877	-0.485819089	0.627095439	0.999968657
3.1.3.24	sucrose-6-phosphatase	973.9245196	-0.279446271	0.333989039	-0.83669294	0.402765188	0.999968657
2.4.1.241	digalactosyldiacylglycerol synthase	0.104075673	0.310351444	4.143245313	0.074905399	0.940289981	0.999968657
3.2.1.93	trehalose-6-phosphate hydrolase	14.12340887	0.126313329	0.695073274	0.181726637	0.85579726	0.999968657
2.5.1.56	N-acetylneuraminate synthase	25.93261642	0.766275456	0.646929607	1.184480426	0.23622294	0.999968657
2.7.1.15	ribokinase	109.9336484	-0.354109486	0.307856866	-1.150240666	0.250044761	0.999968657
3.6.3.24	nickel transport system ATP-binding protein	10.48207584	-1.317567831	1.347797573	-0.977571007	0.328286543	0.999968657

2.3.1.117	2,3,4,5-tetrahydropyridine-2,6-dicarboxylate N-succinyltransfer	34.45067098	0.402302985	0.561763214	0.716143341	0.473902844	0.999968657
2.3.2.-	#N/A	18.67247501	0.969670925	1.055247541	0.918903752	0.358145917	0.999968657
2.4.1.46	1,2-diacylglycerol 3-beta-galactosyltransferase	5.249527659	-0.830154753	4.019578665	-0.206527804	0.836378649	0.999968657
1.13.11.15	3,4-dihydroxyphenylacetate 2,3-dioxygenase	0.110533983	-0.435087999	4.142162324	-0.105038858	0.916344975	0.999968657
2.6.1.87	UDP-4-amino-4-deoxy-L-arabinose-oxoglutarate aminotransfera	0.040990391	-0.227404664	4.140771598	-0.054918427	0.956203451	0.999968657
4.1.2.57	sulfofructosephosphate aldolase	0.103724382	1.515369364	4.135487834	0.366430618	0.714043776	0.999968657
5.99.1.-	#N/A	287.1172249	0.283954101	0.343562469	0.826499187	0.408520979	0.999968657
3.6.1.67	dihydroneopterin triphosphate diphosphatase	2.254603037	0.846090183	1.493932605	0.566350972	0.571155216	0.999968657
2.2.1.2	transaldolase	460.0355829	-0.431734581	0.604419384	-0.714296385	0.475043927	0.999968657
6.1.1.6	#N/A	607.4222191	-0.177859465	0.288789753	-0.615878724	0.537974564	0.999968657
4.3.1.7	#N/A	47.02469326	-0.817869336	0.477500007	-1.71281534	0.086746517	0.999968657
6.1.1.24	nondiscriminating glutamyl-tRNA synthetase	13.45465817	0.730314194	0.58635149	1.245522876	0.212939612	0.999968657
2.4.1.25	4-alpha-glucanotransferase	679.9918035	-0.05647344	0.291884275	-0.19347887	0.846583952	0.999968657
2.4.1.182	lipid-A-disaccharide synthase	104.0503707	0.683665317	0.569802114	1.19982938	0.230205611	0.999968657
2.2.1.9	2-succinyl-5-enolpyruvyl-6-hydroxy-3-cyclohexene-1-carboxylat	95.19445505	-0.179116209	0.353747152	-0.506339648	0.612618234	0.999968657
3.2.1.165	exo-1,4-beta-D-glucosaminidase	2.50673581	0.364515165	2.158207295	0.168897198	0.865877503	0.999968657
1.1.1.18	myo-inositol 2-dehydrogenase / D-chiro-inositol 1-dehydrogena	75.32692191	0.665546849	0.47460571	1.402315301	0.160821113	0.999968657
2.4.1.7	sucrose phosphorylase	40.52910576	-0.409848518	0.766533812	-0.534677676	0.592872761	0.999968657
3.5.4.2	adenine deaminase	259.7447852	0.926223245	0.459777857	2.014501637	0.043956893	0.999968657
5.2.1.8	peptidylprolyl isomerase	2610.435006	0.804017351	0.412007928	1.951460872	0.051002246	0.999968657
5.1.1.18	serine racemase	30.0924605	0.617031054	0.774255372	0.796934806	0.425488895	0.999968657
2.6.1.13	ornithine--oxo-acid transaminase	35.59584511	-0.13339591	0.348200929	-0.383100384	0.701645335	0.999968657
1.10.3.10	#N/A	9.229660608	0.137331738	1.698044706	0.080876397	0.935540251	0.999968657
2.7.7.6	DNA-directed RNA polymerase	5724.757651	0.049517917	0.344378347	0.143789287	0.885666863	0.999968657
4.1.3.39	4-hydroxy 2-oxovalerate aldolase	57.95554725	0.099432476	0.436490548	0.227799838	0.819801853	0.999968657
1.21.4.2	glycine/sarcosine/betaine reductase complex component A	2335.584865	0.410812463	0.537159399	0.764786884	0.444398459	0.999968657
6.4.1.1	pyruvate carboxylase	480.0100183	0.037484181	0.369582886	0.101422934	0.919214732	0.999968657
2.7.2.4	aspartate kinase	755.1354989	0.314454133	0.369227139	0.851654984	0.394405612	0.999968657
2.3.1.23	lysophosphatidylcholine acyltransferase / lyso-PAF acetyltransf	1.237997841	-0.105565507	4.099608847	-0.025750141	0.97945663	0.999968657
4.3.1.1	aspartate ammonia-lyase	327.7532297	0.124511316	0.464221461	0.268215338	0.788533569	0.999968657
2.3.2.23	ubiquitin-conjugating enzyme E2 R	9.282151261	-0.438082583	4.093783268	-0.10701167	0.914779722	0.999968657
2.7.1.1	hexokinase	23.12651405	-1.194929006	0.80121527	-1.491395698	0.135857637	0.999968657
5.1.3.1	ribulose-phosphate 3-epimerase	405.4522483	0.199652598	0.330800112	0.603544531	0.546146501	0.999968657
1.1.1.302	2,5-diamino-6-(ribosylamino)-4(3H)-pyrimidinone 5'-phosphate	0.582805694	-0.907411673	4.106777413	-0.220954676	0.825127724	0.999968657
3.1.1.-	#N/A	541.0085476	-0.232962503	0.303455975	-0.76769786	0.442666708	0.999968657
1.6.99.3	NADH dehydrogenase	195.4290965	-0.221908004	0.679087028	-0.326774029	0.743838804	0.999968657
1.7.99.4	#N/A	62.74469958	0.409627219	0.574295424	0.713269167	0.475679213	0.999968657
3.5.4.31	5-methylthioadenosine/S-adenosylhomocysteine deaminase	646.4515798	-0.556011874	0.774015127	-0.718347555	0.472543015	0.999968657
3.1.3.90	maltose 6'-phosphate phosphatase	7.623733685	-0.141160407	0.706605519	-0.19977258	0.841658447	0.999968657
4.1.1.15	glutamate decarboxylase	198.5891402	-0.635700041	0.670988977	-0.947407578	0.343431131	0.999968657
4.1.1.5	acetolactate decarboxylase	2.950417394	1.280186706	1.853055251	0.690851881	0.489658628	0.999968657
4.6.1.17	cyclic pyranopterin monophosphate synthase	67.32316178	-0.299901062	0.585178158	-0.512495311	0.608304401	0.999968657
2.3.1.29	glycine C-acetyltransferase	448.8884273	0.393552925	0.405142958	0.971392731	0.331352747	0.999968657
2.1.1.190	23S rRNA (uracil1939-C5)-methyltransferase	470.4162853	0.079177053	0.293436522	0.269826852	0.787293464	0.999968657
1.4.7.1	glutamate synthase (ferredoxin)	49.96845184	-0.093622293	0.639156733	-0.146477832	0.883544188	0.999968657
3.1.26.-	#N/A	361.3170482	-0.176943016	0.213237325	-0.82979383	0.40665536	0.999968657
3.2.1.156	oligosaccharide reducing-end xylanase	47.24343399	0.693785072	0.692367258	1.002047778	0.316320518	0.999968657
3.6.3.16	arsenite/tail-anchored protein-transporting ATPase	8.08124422	-0.955686741	1.079154061	-0.885588792	0.375839153	0.999968657
6.3.5.11	cobyrinic acid a,c-diamide synthase	147.2244454	-0.384044684	0.314207109	-1.222266058	0.22160703	0.999968657
2.7.11.5	isocitrate dehydrogenase kinase/phosphatase	0.941316252	-0.274422658	4.08252682	-0.067218826	0.946407498	0.999968657
2.1.2.1	glycine hydroxymethyltransferase	400.2794024	-0.136080039	0.231656594	-0.587421395	0.556920728	0.999968657
2.6.1.44	alanine-glyoxylate transaminase / (R)-3-amino-2-methylpropior	1.130264311	1.533922882	3.557283952	0.431206196	0.666318449	0.999968657
4.2.3.12	6-pyruvoyltetrahydropterin/6-carboxytetrahydropterin synthas	143.680634	-0.193172864	0.406100767	-0.475677173	0.634304386	0.999968657
3.6.4.13	ATP-dependent helicase HrpA	1266.701025	-0.301385823	0.431088996	-0.699126691	0.48447286	0.999968657
1.1.1.29	glycerate dehydrogenase	213.4454083	0.090914391	0.199727878	0.455191295	0.648971623	0.999968657
4.2.1.83	4-oxalmesaconate hydratase	0.709423283	-2.056774865	3.85831885	-0.5330754	0.593981387	0.999968657
2.3.2.27	protein neuralized	11.57495195	-0.385869948	4.093521609	-0.094263567	0.92489979	0.999968657
1.6.99.1	NADPH2 dehydrogenase	0.530483847	-0.244835421	4.081821294	-0.059981906	0.952170046	0.999968657
2.5.1.87	ditrans,polycis-polyprenyl diphosphate synthase	0.052512875	-0.340236249	4.139416356	-0.082194256	0.934492241	0.999968657
2.7.7.72	tRNA nucleotidyltransferase (CCA-adding enzyme)	197.4056266	0.165181294	0.273044451	0.604961181	0.54520479	0.999968657
4.4.1.5	lactoylglutathione lyase	1030.235915	-0.380143226	0.512920398	-0.741134936	0.458611629	0.999968657
3.2.2.1	purine nucleosidase	27.4703256	-0.206824323	0.591915932	-0.34941503	0.726777751	0.999968657
2.1.1.192	23S rRNA (adenine2503-C2)-methyltransferase	509.0918047	-0.118797786	0.203669452	-0.583287205	0.559699974	0.999968657
1.4.1.4	glutamate dehydrogenase (NADP+)	1096.26824	-0.158628557	0.309865343	-0.511927391	0.608701827	0.999968657
2.7.1.58	2-dehydro-3-deoxygalactonokinase	5.245567172	-1.458258131	1.408605467	-1.035249518	0.300552399	0.999968657
1.5.3.1	sarcosine oxidase	12.21788258	-0.237213856	0.860503	-0.275668831	0.782802437	0.999968657
2.5.1.77	#N/A	1.536021083	-0.249757737	4.09805331	-0.060945458	0.951402646	0.999968657
5.3.1.31	sulfoquinovose isomerase	0.13101338	0.237576814	4.13203787	0.057521339	0.954129908	0.999968657
2.7.4.24	inositol-hexakisphosphate/diphosphoinositol-pentakisphospha	4.421491168	-0.225698592	2.742295297	-0.082302804	0.934405925	0.999968657
4.2.99.18	DNA-(apurinic or apyrimidinic site) lyase	479.2168979	0.018193449	0.435646273	0.041761975	0.966688448	0.999968657
1.16.1.9	ferric-chelate reductase (NADPH)	0.390839791	5.761166087	4.076919095	1.41311759	0.157621152	0.999968657
3.2.1.172	unsaturated rhamnogalacturonyl hydrolase	155.0754149	0.012701897	0.462586718	0.027458414	0.978094109	0.999968657
2.4.2.8	hypoxanthine phosphoribosyltransferase	321.3459054	-0.152453566	0.254352927	-0.599378068	0.548920799	0.999968657
3.2.1.37	xylan 1,4-beta-xylosidase	187.3640125	-0.071942502	0.572886699	-0.125578935	0.900065238	0.999968657
1.1.1.307	D-xylose reductase	0.179515503	-0.181089432	4.132628153	-0.043819435	0.965048335	0.999968657
1.1.1.219	dihydroflavonol-4-reductase	15.39927764	-0.655607554	0.93596258	-0.700463424	0.483637939	0.999968657
3.6.3.35	manganese/zinc transport system ATP-binding protein	2.734835095	-0.773119505	2.068485369	-0.373761161	0.708582013	0.999968657
1.7.2.4	nitrous-oxide reductase	0.408715984	0.086218009	4.106824849	0.020993836	0.983250572	0.999968657
2.1.1.202	multisite-specific tRNA:(cytosine-C5)-methyltransferase	0.206248383	0.060545237	4.134578024	0.014643631	0.98831649	0.999968657
1.7.7.1	ferredoxin-nitrite reductase	24.30469765	-0.342160235	0.572993076	-0.597145497	0.550410245	0.999968657
2.4.1.227	UDP-N-acetylglucosamine--N-acetylmuramyl-(pentapeptide) py	364.4604836	0.056912705	0.151170497	0.376480237	0.706559905	0.999968657
2.6.1.100	L-glutamine:2-deoxy-scyllo-inosose/3-amino-2,3-dideoxy-scyllo	0.025751485	-0.039846366	4.143731151	-0.00961606	0.992327613	0.999968657
2.4.2.21	nicotinate-nucleotide--dimethylbenzimidazole phosphoribosylt	143.4673169	0.012861133	0.293072298	0.043883823	0.96499701	0.999968657
4.2.1.147	5,6,7,8-tetrahydromethanopterin hydro-lyase	3.430205491	0.275117108	3.053614401	0.090095563	0.928211275	0.999968657
2.3.1.1	amino-acid N-acetyltransferase	187.3742114	0.051609497	0.233056009	0.221446753	0.824744592	0.999968657
2.1.1.223	tRNA1Val (adenine37-N6)-methyltransferase	87.6555404	-0.986531365	0.572996384	-1.721706092	0.085122773	0.999968657
4.1.1.39	#N/A	0.674912036	-1.217258185	4.108438138	-0.296282467	0.767014375	0.999968657

2.5.1.105	7,8-dihydropterin-6-yl-methyl-4-(beta-D-ribofuranosyl)aminobe	39.00221093	0.161798704	0.447069643	0.361909395	0.717419739	0.999968657
1.17.4.1	ribonucleotide reductase, class II	441.4559732	-0.647073374	0.398997616	-1.62174747	0.10485743	0.999968657
2.3.1.183	phosphinothricin acetyltransferase	219.4034355	-0.37285703	0.651473757	-0.572328549	0.567099412	0.999968657
6.3.2.9	UDP-N-acetylmuramoylalanine--D-glutamate ligase	401.0932987	-0.054758903	0.276436899	-0.198088257	0.842976013	0.999968657
6.3.2.17	folylpolyglutamate synthase	408.1969088	-0.21224741	0.196438637	-1.080476903	0.279929866	0.999968657
2.7.8.20	phosphoglycerol transferase	36.26566766	-0.386831773	0.451489909	-0.856789411	0.391561279	0.999968657
2.3.3.10	hydroxymethylglutaryl-CoA synthase	5.711122078	-0.951951551	1.401718625	-0.679131698	0.497054419	0.999968657
2.3.3.15	sulfoacetaldehyde acetyltransferase	1.166067547	0.517240815	4.120406745	0.125531494	0.900102794	0.999968657
3.2.1.28	alpha,alpha-trehalase	2.844993824	-1.629560557	1.25776957	-1.29559547	0.195114891	0.999968657
2.7.2.1	acetate kinase	2424.083986	-1.440789468	0.785120581	-1.835118711	0.066488102	0.999968657
3.5.1.44	chemotaxis protein CheD	22.51654174	-0.13296707	0.595968529	-0.223110892	0.823449202	0.999968657
2.1.1.199	16S rRNA (cytosine1402-N4)-methyltransferase	1164.169383	-0.381954095	0.390421451	-0.978312267	0.327919904	0.999968657
2.7.2.-	#N/A	0.770139323	-0.734854313	4.103324454	-0.179087547	0.85786896	0.999968657
1.13.11.49	chlorite dismutase	0.051862191	1.042153446	4.143731151	0.251501221	0.801426618	0.999968657
3.6.4.3	#N/A	2.836180328	0.821323453	3.07243777	0.267319801	0.789222943	0.999968657
2.6.1.98	UDP-2-acetamido-2-deoxy-ribo-hexuluronate aminotransferase	0.251634167	1.223819686	4.101959357	0.298350027	0.765436026	0.999968657
1.1.1.4	(R,R)-butanediol dehydrogenase / meso-butanediol dehydroger	2.80919775	0.114867932	1.53167963	0.074994751	0.940218889	0.999968657
1.8.1.4	dihydrolipoamide dehydrogenase	317.743301	-0.265555096	0.439398824	-0.604360052	0.545604289	0.999968657
3.1.13.5	ribonuclease D	6.041358097	0.493075664	0.976244197	0.505074105	0.613506788	0.999968657
1.1.1.305	UDP-4-amino-4-deoxy-L-arabinose formyltransferase / UDP-glu	1.285501156	-0.978818524	2.014779877	-0.485819089	0.627095439	0.999968657
5.4.99.19	16S rRNA pseudouridine516 synthase	318.1196796	-0.066343127	0.49756989	-0.133334277	0.89392902	0.999968657
2.7.1.68	1-phosphatidylinositol-4-phosphate 5-kinase	1.131986754	-0.195110675	4.098514337	-0.04760522	0.962030872	0.999968657
1.1.1.60	2-hydroxy-3-oxopropionate reductase	9.559379802	-0.025655508	0.722719563	-0.035498566	0.97168219	0.999968657
2.7.1.198	PTS system, glucitol/sorbitol-specific IIA component	10.56111943	-1.002685378	0.728129963	-1.377069245	0.16849084	0.999968657
3.4.21.116	stage IV sporulation protein B	186.7204116	1.237429996	0.658390953	1.879476002	0.060179527	0.999968657
1.6.2.2	cytochrome-b5 reductase	0.078987483	1.38698787	4.130542208	0.33578833	0.737030491	0.999968657
1.2.1.89	D-glyceraldehyde dehydrogenase (NADP+)	0.103005941	-0.564033383	4.137063428	-0.136336654	0.891555149	0.999968657
1.1.1.136	UDP-N-acetyl-D-glucosamine dehydrogenase	13.44204196	-0.045588324	0.672435548	-0.067795826	0.945948166	0.999968657
2.5.1.128	N4-bis(aminopropyl)spermidine synthase	4.506886474	1.11207239	3.23856174	0.34338465	0.731309106	0.999968657
2.8.4.3	tRNA-2-methylthio-N6-dimethylallyladenosine synthase	406.9485174	0.245095507	0.182151193	1.345560808	0.178444198	0.999968657
3.2.1.40	alpha-L-rhamnosidase	377.6323579	0.286972481	0.480070279	0.597771812	0.549992201	0.999968657
1.1.1.381	3-hydroxy acid dehydrogenase / malonic semialdehyde reducta	0.577711424	-0.730910649	4.093275335	-0.178563763	0.85828025	0.999968657
3.5.2.7	imidazolonepropionase	168.0587454	0.481155831	0.417984951	1.151131946	0.249677957	0.999968657
5.1.99.4	alpha-methylacyl-CoA racemase	1.348104808	0.549774512	3.499137115	0.157117167	0.875152508	0.999968657
3.5.3.7	guanidinobutyrase	0.574681685	0.265652333	4.12817948	0.064350965	0.948690773	0.999968657
6.3.4.20	7-cyano-7-deazaguanine synthase	94.67236829	-0.111427924	0.547372907	-0.203568578	0.83869065	0.999968657
6.3.2.14	bifunctional isochorismate lyase / aryl carrier protein	4.385493731	1.489653257	2.028979701	0.734188349	0.462833953	0.999968657
1.4.3.19	glycine oxidase	0.103005941	-0.564033383	4.137063428	-0.136336654	0.891555149	0.999968657
3.1.3.8	3-phytase	0.154508912	-0.788467879	4.13282253	-0.190781935	0.848696443	0.999968657
3.1.11.2	exodeoxyribonuclease III	258.3334323	-0.447218294	0.302075598	-1.480484678	0.138743947	0.999968657
2.1.1.33	tRNA (guanine-N7-)-methyltransferase	245.3849363	0.125021274	0.299483047	0.417456932	0.67634422	0.999968657
1.12.5.1	#N/A	0.276334958	-0.939258932	4.127808109	-0.227544234	0.820000578	0.999968657
3.5.1.53	N-carbamoylputrescine amidase	105.5660389	-0.08506793	0.376401117	-0.226003395	0.82119878	0.999968657
1.9.3.1	#N/A	0.209529612	2.525827219	4.093178059	0.617082175	0.537180525	0.999968657
3.2.1.24	alpha-mannosidase	269.881228	-0.0804374	0.444913688	-0.180793269	0.856529847	0.999968657
3.6.3.3	#N/A	575.5346922	0.02373901	0.277331073	0.085598089	0.931785917	0.999968657
4.2.1.40	glucarate dehydratase	7.189205811	-0.1865274	1.019982476	-0.182873141	0.854897558	0.999968657
2.1.1.100	protein-S-isoprenylcysteine O-methyltransferase	0.166515913	-0.595768993	4.138090197	-0.143971969	0.885522605	0.999968657
2.4.2.17	ATP phosphoribosyltransferase	157.6760459	-0.280436677	0.186440908	-1.504158502	0.13254056	0.999968657
1.2.7.3	#N/A	1254.655337	0.153145407	0.302429628	0.506383609	0.612587379	0.999968657
2.7.8.38	archaetidylserine synthase	0.293518541	-0.405480618	4.120136259	-0.098414371	0.921603264	0.999968657
2.4.2.1	#N/A	957.9806977	-0.621113689	0.603527827	-1.029138444	0.303414623	0.999968657
2.7.1.73	inosine kinase	0.730107326	-2.707972372	4.105034615	-0.65967102	0.509464968	0.999968657
4.1.1.25	tyrosine decarboxylase	0.874214996	-0.640905496	4.102479006	-0.156223955	0.875856495	0.999968657
3.2.1.54	cyclomaltodextrinase / maltogenic alpha-amylase / neopullular	0.30127684	-1.467920402	4.12669228	-0.35571356	0.722055089	0.999968657
2.3.1.242	KDO2-lipid IV(A) palmitoleoyltransferase	3.363987485	0.527418076	1.968595611	0.267915906	0.78876405	0.999968657
6.3.1.2	glutamine synthetase	935.7833556	-0.041307626	0.2192441	-0.188409295	0.850555813	0.999968657
3.6.3.30	iron(III) transport system ATP-binding protein	97.39821977	0.06095087	0.392886674	0.155136007	0.876714097	0.999968657
6.3.2.3	glutathione synthase	1.801797141	-0.770104744	1.84547628	-0.417293222	0.676463945	0.999968657
6.3.2.39	aerobactin synthase	2.405771588	0.762502701	4.095569267	0.186177464	0.852305596	0.999968657
3.5.1.82	N-acyl-D-glutamate deacylase	0.439714584	1.447903803	4.11470372	0.351885312	0.724924275	0.999968657
5.1.1.4	proline racemase	6.577327919	0.343245748	1.02481625	0.334933944	0.737674916	0.999968657
2.7.1.60	N-acylmannosamine kinase	0.061485587	-0.375949006	4.139390501	-0.09082231	0.927633783	0.999968657
1.8.5.4	sulfide:quinone oxidoreductase	3.822115518	0.705341852	1.969518068	0.35812916	0.720246658	0.999968657
2.1.1.224	23S rRNA (adenine-C8)-methyltransferase	4.139167645	-0.787159941	3.306332937	-0.23807643	0.811821816	0.999968657
6.2.1.30	phenylacetate-CoA ligase	732.7612545	0.865803584	0.589492323	1.468727497	0.141906716	0.999968657
1.1.1.163	cyclopentanol dehydrogenase	0.213055426	0.304813327	4.142999974	0.073573094	0.941350081	0.999968657
3.4.21.88	repressor LexA	498.3446664	-0.215553194	0.37206804	-0.579338107	0.562361058	0.999968657
2.7.1.89	thiamine kinase	0.567411961	-0.016346784	4.083966927	-0.004002673	0.996806338	0.999968657
2.4.1.64	alpha,alpha-trehalose phosphorylase	4.662255611	-0.640753552	1.46070093	-0.438661699	0.660906683	0.999968657
3.6.1.23	dUTP pyrophosphatase	266.0926212	-0.149093814	0.274224342	-0.543692851	0.586652853	0.999968657
1.1.1.26	glyoxylate reductase	0.566106049	3.656147329	4.043773762	0.904142404	0.365919896	0.999968657
3.5.1.19	nicotinamidase	5.650251342	-0.050734256	1.261557041	-0.040215586	0.967921252	0.999968657
2.4.1.12	cellulose synthase (UDP-forming)	8.384802002	0.683505711	1.022936471	0.66818002	0.504018688	0.999968657
3.1.21.3	type I restriction enzyme, R subunit	1199.129253	0.110650693	0.252841623	0.43762847	0.66165563	0.999968657
2.1.1.189	23S rRNA (uracil747-C5)-methyltransferase	0.50752299	-0.201414416	4.089080758	-0.049256649	0.960714767	0.999968657
4.2.1.68	L-fuconate dehydratase	0.087521458	-0.530114894	4.138412209	-0.128096204	0.89807284	0.999968657
1.10.9.1	#N/A	0.084668419	0.274728032	4.141794518	0.066330676	0.947114561	0.999968657
4.6.1.12	2-C-methyl-D-erythritol 2,4-cyclodiphosphate synthase	226.1923799	0.194691772	0.213522261	0.911810183	0.361868649	0.999968657
1.3.8.4	isovaleryl-CoA dehydrogenase	0.545047208	-2.028481725	4.114027358	-0.493064715	0.621966862	0.999968657
6.3.5.3	phosphoribosylformylglycinamidine synthase	1099.359206	0.08817538	0.221084079	0.398831887	0.69001708	0.999968657
3.2.1.45	glucosylceramidase	63.37924565	-0.052533204	0.779761589	-0.067370853	0.946286472	0.999968657
2.1.1.163	demethylmenaquinone methyltransferase / 2-methoxy-6-polyp	61.69477923	-0.1001534	0.463592748	-0.21603746	0.828958548	0.999968657
3.4.23.-	#N/A	121.6829844	-0.102801757	0.543962097	-0.188986986	0.850103016	0.999968657
1.1.1.339	dTDP-6-deoxy-L-talose 4-dehydrogenase (NAD+)	18.79373412	-0.052608619	0.49971285	-0.105277698	0.916155459	0.999968657
6.5.1.-	#N/A	117.6895471	0.584472342	0.783486038	0.74598948	0.45567377	0.999968657
6.3.1.11	gamma-glutamylputrescine synthase	0.102475978	-0.587852202	4.137494487	-0.142079271	0.887017391	0.999968657

1.5.5.-	#N/A	3.832395075	-0.852289652	1.669033436	-0.510648639	0.609597111	0.999968657
1.1.1.298	malonyl-CoA reductase / 3-hydroxypropionate dehydrogenase	0.201546003	-1.269172127	4.130495086	-0.307268766	0.758638808	0.999968657
2.7.1.72	streptomycin 6-kinase	0.20799384	1.526539382	4.122404545	0.370303148	0.711156628	0.999968657
5.4.99.28	tRNA pseudouridine32 synthase / 23S rRNA pseudouridine746 s	66.23259907	-0.017930215	0.572869243	-0.031298966	0.975031115	0.999968657
2.7.1.156	adenosylcobinamide kinase / adenosylcobinamide-phosphate g	139.991378	0.109145387	0.409646217	0.266438167	0.789901774	0.999968657
2.3.1.18	galactoside O-acetyltransferase	43.53976159	0.708685986	0.785025576	0.902755283	0.366655789	0.999968657
4.99.1.4	uroporphyrin-III C-methyltransferase / precorrin-2 dehydrogena	30.31722373	-0.041296544	0.835666778	-0.049417477	0.9605866	0.999968657
3.2.1.97	endo-alpha-N-acetylgalactosaminidase	605.1706156	-0.7670626	0.749712989	-1.02314167	0.306240867	0.999968657
4.1.1.87	malonate decarboxylase beta subunit	7.252252901	0.183548583	1.09860082	0.167074865	0.867311143	0.999968657
3.1.3.95	myotubularin	3.613934552	-0.236662799	2.80260551	-0.08444385	0.932703544	0.999968657
4.2.1.5	D-arabinonate dehydratase	0.322417442	6.045641154	4.080458769	1.481608196	0.138444577	0.999968657
3.6.1.5	apyrase	0.249773869	-0.816552953	4.124382892	-0.19798185	0.843059265	0.999968657
1.2.99.7	aldehyde oxidoreductase	192.6359077	-0.600313216	0.562585759	-1.067060811	0.285944377	0.999968657
2.4.2.53	undecaprenyl-phosphate 4-deoxy-4-formamido-L-arabinose tra	25.70573289	-0.308109022	0.539559942	-0.571037614	0.567974146	0.999968657
1.3.7.11	digeranylgeranylglycerophospholipid reductase	2.870100187	-0.347005083	3.909458866	-0.088760387	0.92927234	0.999968657
2.7.1.169	pantoate kinase	0.104075673	0.310351444	4.143245313	0.074905399	0.940289981	0.999968657
3.6.1.40	exopolyphosphatase / guanosine-5'-triphosphate,3'-diphosphat	148.2287987	-0.066075039	0.370536096	-0.178322814	0.858469463	0.999968657
6.1.1.12	aspartyl-tRNA synthetase	692.5248136	-0.24763504	0.300037605	-0.825346678	0.409174798	0.999968657
3.5.1.3	omega-amidase	2.642522728	-0.529519657	3.821438215	-0.138565542	0.889793474	0.999968657
1.5.98.2	5,10-methylenetetrahydromethanopterin reductase	40.82137094	0.449850819	1.365589388	0.329418801	0.741839159	0.999968657
2.1.1.264	23S rRNA (guanine2445-N2)-methyltransferase / 23S rRNA (gua	1.313961173	-0.240705799	2.37803298	-0.101220547	0.919375386	0.999968657
1.1.1.282	quinate/shikimate dehydrogenase	0.322402874	-0.668724377	4.115729047	-0.162480175	0.870927739	0.999968657
2.4.2.10	orotate phosphoribosyltransferase	556.3995842	-0.396418366	0.278582885	-1.42298177	0.15474144	0.999968657
3.1.7.2	GTP diphosphokinase / guanosine-3',5'-bis(diphosphate) 3'-diph	5.410257227	-1.486249931	1.584445868	-0.93802506	0.348231532	0.999968657
3.5.4.10	phosphoribosylaminoimidazolecarboxamide formyltransferase	546.3313652	-0.082785193	0.183591622	-0.450923018	0.652046979	0.999968657
2.4.1.319	beta-1,4-mannooligosaccharide/beta-1,4-mannosyl-N-acetylglu	163.8803014	0.149150962	0.350301357	0.425779002	0.670268889	0.999968657
3.6.3.38	capsular polysaccharide transport system ATP-binding protein	0.528533578	0.192662499	4.090459352	0.047100456	0.962433164	0.999968657
2.3.1.169	#N/A	421.7701187	-0.449567554	0.562923068	-0.798630541	0.424504673	0.999968657
5.4.2.2	phosphoglucomutase	10668.07044	-0.253491348	0.737198024	-0.343857878	0.730953171	0.999968657
5.2.1.1	maleate isomerase	0.8515885	-1.511531057	4.102804423	-0.368414114	0.712564475	0.999968657
2.7.7.62	adenosylcobinamide kinase / adenosylcobinamide-phosphate g	139.991378	0.109145387	0.409646217	0.266438167	0.789901774	0.999968657
1.1.1.320	benzil reductase ((S)-benzoin forming)	0.13068425	0.320812761	4.143731151	0.07742123	0.938288453	0.999968657
2.1.1.265	tellurite methyltransferase	1.655407417	-1.423710729	1.989006209	-0.715789988	0.474121036	0.999968657
3.5.1.25	N-acetylglucosamine-6-phosphate deacetylase	970.9548437	-0.341864814	0.479500356	-0.712960501	0.475870199	0.999968657
3.4.24.55	protease III	0.658608599	1.41270836	4.065902686	0.347452575	0.728251341	0.999968657
2.1.1.219	tRNA (adenine57-N1/adenine58-N1)-methyltransferase catalyti	3.95831283	-0.54601632	1.721244945	-0.317221742	0.751075353	0.999968657
4.1.3.4	hydroxymethylglutaryl-CoA lyase	4.714175398	-3.331746897	1.461236744	-2.280086995	0.022602529	0.999968657
1.1.5.4	malate dehydrogenase (quinone)	1.565747164	-0.449669937	2.529823051	-0.177747585	0.858921213	0.999968657
1.7.2.1	nitrite reductase (NO-forming)	0.040667486	-0.039843063	4.143731151	-0.009615263	0.992328249	0.999968657
4.1.1.47	tartronate-semialdehyde synthase	1.982359021	2.242756744	2.122860842	1.056478456	0.290749679	0.999968657
2.1.2.10	aminomethyltransferase	191.58773	0.248518617	0.342170055	0.726301478	0.467653973	0.999968657
2.7.8.1	ethanolaminephosphotransferase	1.503869701	-0.674060552	4.09845553	-0.164466967	0.869363549	0.999968657
2.7.1.40	pyruvate kinase	528.5119295	-0.172233612	0.188428912	-0.91405088	0.360690111	0.999968657
2.7.1.11	6-phosphofructokinase 1	1517.868815	-0.422067155	0.30435991	-1.386737024	0.165521967	0.999968657
3.4.23.1	pepsin A	0.078987483	1.38698787	4.130542208	0.33578833	0.737030491	0.999968657
6.1.1.21	#N/A	372.2009374	0.063553759	0.179648132	0.35376799	0.723512764	0.999968657
1.97.1.4	#N/A	2043.548934	0.393709604	0.516346087	0.762491698	0.445766596	0.999968657
1.1.1.11	D-arabinitol 4-dehydrogenase	0.221067967	-0.877349477	4.133341393	-0.212261556	0.831902986	0.999968657
6.3.2.13	UDP-N-acetylmuramoyl-L-alanyl-D-glutamate--2,6-diaminopime	496.0040111	0.179422158	0.183018269	0.98035108	0.326912849	0.999968657
3.2.2.24	ADP-ribosyl-[dinitrogen reductase] hydrolase	2.896195258	-0.548023274	3.126363518	-0.175290964	0.860851012	0.999968657
5.-.-.-	#N/A	559.3196393	-0.265679928	0.73918157	-0.359424449	0.719277588	0.999968657
3.2.2.28	double-stranded uracil-DNA glycosylase	1.308970377	-0.039539065	4.065414379	-0.009725716	0.992240124	0.999968657
2.7.1.4	fructokinase	457.5016156	-0.062298149	0.286938201	-0.217113473	0.828119915	0.999968657
2.7.1.14	sedoheptulokinase	8.938681165	-1.641352485	1.323424005	-1.240231762	0.214889684	0.999968657
1.11.1.-	#N/A	3.659730758	0.1743704	2.131309237	0.08181375	0.934794822	0.999968657
4.1.1.11	aspartate 1-decarboxylase	122.8047799	-0.018428782	0.400175308	-0.046051773	0.963268985	0.999968657
3.4.21.81	streptogrisin B	8.877711127	2.398339168	2.179003106	1.100658903	0.271045139	0.999968657
2.7.1.165	glycerate 2-kinase	141.9706858	-0.051379822	0.419272361	-0.122545215	0.902467239	0.999968657
4.4.1.1	cystathionine gamma-lyase	3.136372628	2.988149471	2.361149192	1.265548776	0.205674675	0.999968657
1.2.1.87	propionaldehyde dehydrogenase	223.0956313	-0.98094239	0.647300761	-1.51543525	0.129662216	0.999968657
2.8.3.10	#N/A	92.4380191	0.210562241	0.4661824	0.45167351	0.651504204	0.999968657
4.4.1.14	1-aminocyclopropane-1-carboxylate synthase	3.109366266	-0.315581712	2.997860733	-0.10526897	0.916162384	0.999968657
1.3.1.2	dihydropyrimidine dehydrogenase (NADP+)	0.077254456	-0.423702248	4.139694903	-0.102351081	0.918478011	0.999968657
3.10.1.1	N-sulfoglucosamine sulfohydrolase	0.500048027	-1.222500411	4.113060599	-0.297224021	0.766295484	0.999968657
2.7.1.51	L-fuculokinase	17.52662205	1.938396957	0.965245183	2.008191278	0.04462297	0.999968657
4.1.3.44	tRNA wybutosine-synthesizing protein 1	3.850197146	-0.6723335	2.90724207	-0.231261616	0.817111564	0.999968657
1.1.1.30	3-hydroxybutyrate dehydrogenase	0.712468766	-1.941913003	3.825887342	-0.507571925	0.611753584	0.999968657
5.4.99.20	23S rRNA pseudouridine2457 synthase	2.784565633	1.257933684	1.75421786	0.717090911	0.473318003	0.999968657
2.4.99.16	starch synthase (maltosyl-transferring)	1.915180048	-2.558778749	3.636634079	-0.703611827	0.481674544	0.999968657
2.7.4.28	[pyruvate, water dikinase]-phosphate phosphotransferase / [py	9.875085199	-0.378757976	0.666282196	-0.568464801	0.5697194	0.999968657
3.4.23.51	hydrogenase 3 maturation protease	1.085645249	-1.944137387	3.534335943	-0.550071475	0.58227035	0.999968657
2.7.1.208	PTS system, alpha-glucoside-specific IIB component	59.53385477	-0.307237572	0.616939744	-0.498002561	0.618482237	0.999968657
3.2.2.3	uridine nucleosidase	0.052512875	-0.340236249	4.139416356	-0.082194256	0.934492241	0.999968657
1.6.2.4	NADPH-ferrihemoprotein reductase	1.311335403	-0.414307939	4.100592999	-0.101036104	0.9195218	0.999968657
6.6.1.1	magnesium chelatase subunit H	18.6825575	0.347113069	0.686560527	0.505582618	0.613149686	0.999968657
1.1.1.102	3-dehydrosphinganine reductase	1.670128019	-0.081071251	4.099799701	-0.019774442	0.984223306	0.999968657
3.6.3.8	Ca2+-transporting ATPase	569.4036309	0.42089527	0.331877445	1.26822499	0.204717615	0.999968657
1.1.1.36	acetoacetyl-CoA reductase	0.578478959	-1.609694443	4.079906096	-0.394542032	0.693180901	0.999968657
3.1.3.11	fructose-1,6-bisphosphatase I / sedoheptulose-1,7-bisphosphat	458.6616046	-0.211448425	0.23326388	-0.906477358	0.364683244	0.999968657
6.1.1.2	tryptophanyl-tRNA synthetase	420.3540249	-0.15518813	0.2913945	-0.532570554	0.59433089	0.999968657
1.1.1.79	glyoxylate/hydroxypyruvate reductase	10.11112133	0.799374149	1.833034915	0.436093248	0.662769076	0.999968657
6.3.2.34	coenzyme F420-0:L-glutamate ligase / coenzyme F420-1:gamm	1.257810662	-1.868418675	4.096129299	-0.456142504	0.648287507	0.999968657
1.1.1.374	UDP-N-acetylglucosamine 3-dehydrogenase	1.956313881	-0.49052241	4.096006374	-0.119756261	0.904676231	0.999968657
2.4.1.21	starch synthase	1092.909783	-0.182932965	0.371436029	-0.492501939	0.622364551	0.999968657
2.3.2.3	phosphatidylglycerol lysyltransferase	2.629663203	-1.257304058	1.727901349	-0.727648056	0.466829055	0.999968657
3.2.1.58	glucan 1,3-beta-glucosidase	1.646498268	-0.169605439	2.274162686	-0.07457929	0.940549452	0.999968657
1.1.1.81	glyoxylate/hydroxypyruvate reductase	52.56367318	0.077175795	0.430225871	0.179384365	0.857635907	0.999968657

2.7.11.25	mitogen-activated protein kinase kinase kinase 8	0.495257723	0.27007215	4.104619142	0.065797128	0.947539343	0.999968657
1.8.99.5	dissimilatory sulfite reductase alpha subunit	53.43867049	0.221354887	0.632033905	0.350226286	0.726168881	0.999968657
2.1.1.63	methylated-DNA-protein]-cysteine S-methyltransferase	165.7113079	0.057712504	0.33992592	0.16977965	0.865183431	0.999968657
4.2.99.21	salicylate synthetase	0.575016627	0.355452257	4.079955731	0.087121597	0.930574859	0.999968657
2.3.1.57	diamine N-acetyltransferase	81.34146399	-0.131248036	0.510206454	-0.257244954	0.796989683	0.999968657
2.4.1.288	galactofuranosylgalactofuranosylrhamnosyl-N-acetylglucosamir	0.870667307	-2.173721565	4.104827849	-0.529552431	0.596422283	0.999968657
2.4.2.14	amidophosphoribosyltransferase	632.8867892	-0.120386946	0.159549357	-0.754543598	0.450522877	0.999968657
2.7.7.39	glycerol-3-phosphate cytidylyltransferase	10.10101612	-1.767592774	1.134274154	-1.558347043	0.119151003	0.999968657
4.2.2.10	pectin lyase	10.9423628	0.82029078	1.167004522	0.702902829	0.482116308	0.999968657
1.3.5.1	#N/A	476.3325581	-0.224136045	0.334566147	-0.669930437	0.502902136	0.999968657
4.3.2.1	argininosuccinate lyase	276.1844737	-0.04774882	0.194280218	-0.245772937	0.805858008	0.999968657
6.1.2.1	D-alanine---(R)-lactate ligase	0.017504292	-0.007393638	4.141980666	-0.001785049	0.998575738	0.999968657
2.2.1.6	#N/A	949.9615364	-0.310314459	0.208643683	-1.487293817	0.136937227	0.999968657
2.3.1.102	acetyl CoA:N6-hydroxylysine acetyl transferase	1.45851818	1.144924821	4.097713531	0.279405774	0.77993344	0.999968657
2.6.1.17	acetylornithine/N-succinyldiaminopimelate aminotransferase	710.8284773	-0.524274733	0.466862624	-1.12297431	0.261448405	0.999968657
2.1.1.321	type III protein arginine methyltransferase	0.500958201	-0.641033829	4.108339442	-0.156032343	0.876007527	0.999968657
3.4.24.15	thimet oligopeptidase	1.404001712	0.097388494	3.675621574	0.026495789	0.978861892	0.999968657
3.1.1.31	6-phosphogluconolactonase	371.1096164	-0.057712958	0.548123838	-0.105291823	0.916144251	0.999968657
3.6.1.31	phosphoribosyl-ATP pyrophosphohydrolase	275.8587485	-0.367600389	0.221186125	-1.66195049	0.096522702	0.999968657
1.20.4.1	arsenate reductase (glutaredoxin)	92.4590067	-0.851963266	0.457584284	-1.861871782	0.062621165	0.999968657
2.7.7.60	2-C-methyl-D-erythritol 4-phosphate cytidylyltransferase	341.3139113	0.173363794	0.228999809	0.757047765	0.449021247	0.999968657
2.7.7.3	pantetheine-phosphate adenylyltransferase	410.527713	-0.480499815	0.354159233	-1.356733838	0.174865797	0.999968657
2.1.3.6	putrescine carbamoyltransferase	5.844709524	0.527775316	1.236303351	0.42689791	0.669453688	0.999968657
2.4.1.265	alpha-1,3-glucosyltransferase	0.122530041	-0.671166732	4.137516692	-0.16221487	0.87113665	0.999968657
3.5.4.-	#N/A	0.035008583	-0.204325554	4.14032189	-0.049350161	0.960640245	0.999968657
3.6.1.1	inorganic pyrophosphatase	703.8118417	-0.240279317	0.210692322	-1.140427499	0.254108242	0.999968657
2.1.1.107	uroporphyrin-III C-methyltransferase	93.24422569	0.164443035	0.345601529	0.475816863	0.634204855	0.999968657
1.1.1.337	L-2-hydroxycarboxylate dehydrogenase (NAD+)	2.293813537	-0.130758749	3.262267931	-0.040082161	0.968027624	0.999968657
3.2.1.143	poly(ADP-ribose) glycohydrolase	0.208151345	0.259212185	4.141249987	0.06259274	0.95009081	0.999968657
1.1.1.42	isocitrate dehydrogenase	498.3051866	-0.125567624	0.396274781	-0.316870087	0.751342181	0.999968657
4.2.1.106	bile-acid 7alpha-dehydratase	5.369375598	-0.739349914	1.16830503	-0.632839794	0.526838267	0.999968657
2.7.7.18	nicotinate-nucleotide adenylyltransferase	289.546131	-0.031664271	0.229172089	-0.138168095	0.890107569	0.999968657
2.8.3.9	acetate CoA/acetoacetate CoA-transferase alpha subunit	181.9968591	0.149808666	0.540482646	0.277175718	0.781645184	0.999968657
2.3.1.225	palmitoyltransferase ZDHHC9/14/18	2.026598504	-0.599236794	4.097147887	-0.146257058	0.883718464	0.999968657
4.1.1.1	pyruvate decarboxylase	0.143466369	-0.740091609	4.136114884	-0.178934007	0.85798952	0.999968657
3.6.4.7	peroxisome-assembly ATPase	9.240900872	-0.474250799	4.09370586	-0.115848773	0.907772396	0.999968657
1.14.-.-	#N/A	8.314588655	0.316098786	1.015264835	0.311346139	0.755537495	0.999968657
2.7.1.35	pyridoxine kinase	248.946753	0.251018544	0.262308234	0.956960218	0.338587335	0.999968657
1.1.1.289	sorbose reductase	0.110533983	-0.435087999	4.142162324	-0.105038858	0.916344975	0.999968657
3.2.1.135	cyclomaltodextrinase / maltogenic alpha-amylase / neopullulan	0.30127684	-1.467920402	4.12669228	-0.35571356	0.722055089	0.999968657
1.1.1.370	scyllo-inositol 2-dehydrogenase (NAD+)	0.128757426	-0.685780939	4.134677189	-0.165860817	0.868266483	0.999968657
5.1.3.20	ADP-L-glycero-D-manno-heptose 6-epimerase	19.91059957	1.387632952	0.640527882	2.166389614	0.030281427	0.999968657
2.-.-.-	#N/A	276.2509304	-0.038952926	0.278520643	-0.139856514	0.888773361	0.999968657
4.2.1.115	UDP-N-acetylglucosamine 4,6-dehydratase	0.138985024	0.999767953	4.139395407	0.241525115	0.809148153	0.999968657
3.2.1.96	mannosyl-glycoprotein endo-beta-N-acetylglucosaminidase	18.59201307	-0.270697599	0.946946598	-0.285863637	0.774982565	0.999968657
2.7.1.200	PTS system, galactitol-specific IIA component	13.58689989	-1.316900166	0.934159308	-1.409716902	0.158623292	0.999968657
3.6.1.-	#N/A	368.9897887	-0.16846768	0.225801018	-0.746089109	0.455613588	0.999968657
2.7.1.90	diphosphate-dependent phosphofructokinase	109.7540539	-0.886128955	0.457750921	-1.93583216	0.052888261	0.999968657
5.3.1.27	6-phospho-3-hexuloisomerase	13.80075987	-0.746095786	0.801397039	-0.930993939	0.351856699	0.999968657
3.2.1.82	exo-poly-alpha-galacturonosidase	1.206697936	0.173117613	2.671638516	0.064798292	0.948334601	0.999968657
5.3.1.4	L-arabinose isomerase	262.4860847	-0.00521201	0.288980084	-0.018035878	0.985610232	0.999968657
2.3.1.40	acyl-[acyl-carrier-protein]-phospholipid O-acyltransferase / long	5.957426123	1.250412989	1.431081539	0.873753839	0.382252324	0.999968657
2.4.1.282	3-O-alpha-D-glucosyl-L-rhamnose phosphorylase	0.087521458	-0.530114894	4.138412209	-0.128096204	0.89807284	0.999968657
2.1.1.113	site-specific DNA-methyltransferase (cytosine-N4-specific)	0.549311404	0.323648739	4.102720543	0.078886372	0.937123003	0.999968657
2.7.7.9	UTP--glucose-1-phosphate uridylyltransferase	171.0660117	0.297356823	0.321888063	0.923789532	0.355595906	0.999968657
1.13.11.1	catechol 1,2-dioxygenase	0.055266992	-0.039843063	4.143731151	-0.009615263	0.992328249	0.999968657
3.1.2.21	medium-chain acyl-[acyl-carrier-protein] hydrolase	2.090803846	-1.395181151	2.139113301	-0.652224055	0.514256644	0.999968657
0.0.0.0	#N/A	4559514.544	0.269252443	0.629192221	0.427933521	0.668699521	0.999968657
2.7.7.89	[glutamine synthetase] adenylyltransferase / [glutamine synthe	10.5009941	0.525614227	0.980933908	0.535830419	0.592075757	0.999968657
4.2.1.129	squalene-hopene/tetraprenyl-beta-curcumene cyclase	7.194674308	2.516898369	1.346719826	1.868910163	0.061635314	0.999968657
4.1.1.18	lysine decarboxylase	3.388512186	0.468933483	1.454410532	0.322421677	0.747133264	0.999968657
5.1.3.2	UDP-glucose 4-epimerase	1242.539518	-0.105507026	0.435692348	-0.242159465	0.808656603	0.999968657
5.4.99.61	precorrin-8X/cobalt-precorrin-8 methylmutase	102.094744	-0.189965651	0.507681135	-0.374183002	0.708268169	0.999968657
3.1.26.5	ribonuclease P/MRP protein subunit POP1	316.2440482	-0.508250615	0.29036423	-1.750389898	0.080051058	0.999968657
6.3.2.45	UDP-N-acetylmuramate: L-alanyl-gamma-D-glutamyl-meso-dia	13.36009396	0.496353126	0.87091991	0.569918221	0.568733166	0.999968657
2.1.1.171	16S rRNA (guanine966-N2)-methyltransferase	10.12371931	0.365260265	0.831279532	0.439395234	0.660375179	0.999968657
2.5.1.7	UDP-N-acetylglucosamine 1-carboxyvinyltransferase	705.9287071	0.088796714	0.169715771	0.523208384	0.600829248	0.999968657
2.3.1.31	homoserine O-acetyltransferase/O-succinyltransferase	18.80023827	0.163008037	0.900986727	0.180921686	0.856429047	0.999968657
2.7.1.181	O-antigen chain-terminating methyltransferase	2.5617666	0.895331743	3.782517315	0.236702616	0.812887505	0.999968657
4.1.1.98	4-hydroxy-3-polyprenylbenzoate decarboxylase	25.44402293	-0.510552499	0.656669294	-0.777488004	0.436870909	0.999968657
2.1.1.144	trans-aconitate 2-methyltransferase	2.013320454	0.152228339	1.349543266	0.112799895	0.910189201	0.999968657
3.5.5.7	aliphatic nitrilase	0.040667486	-0.039843063	4.143731151	-0.009615263	0.992328249	0.999968657
1.1.1.261	glycerol-1-phosphate dehydrogenase [NAD(P)+]	72.65407799	-0.545242074	0.353641821	-1.541791843	0.123124185	0.999968657
3.2.2.30	adenosylhomocysteine/aminodeoxyfutalosine nucleosidase	0.689389753	0.005038395	4.118824892	0.00122326	0.99902398	0.999968657
3.4.21.112	membrane-bound transcription factor site-1 protease	0.333031825	-0.872156465	4.117489705	-0.211817522	0.832249398	0.999968657
1.21.98.1	cyclic dehypoxanthinyl futalosine synthase	7.265508363	-0.123847665	1.255379267	-0.098653585	0.921413323	0.999968657
1.6.5.3	#N/A	1547.45876	0.005711861	0.224831844	0.025405037	0.979731894	0.999968657
1.3.1.78	arogenate dehydrogenase (NADP+)	0.051502971	-0.263696455	4.141439557	-0.063672656	0.949230878	0.999968657
6.3.5.7	aspartyl-tRNA(Asn)/glutamyl-tRNA(Gln) amidotransferase subu	503.111813	0.515316573	0.457660134	1.125980907	0.260173601	0.999968657
2.6.1.22	4-aminobutyrate aminotransferase / (S)-3-amino-2-methylprop	25.41220286	0.00864301	0.663325137	0.013029825	0.989603998	0.999968657
2.1.1.151	precorrin-2/cobalt-factor-2 C20-methyltransferase	73.91478762	-0.390825113	0.440859334	-0.886507516	0.375344106	0.999968657
6.3.4.23	5-formaminoimidazole-4-carboxamide-1-(beta)-D-ribofuranosy	4.800089674	-0.597090512	3.023592851	-0.197477154	0.843454158	0.999968657
6.3.4.18	5-(carboxyamino)imidazole ribonucleotide synthase	16.67334866	0.233142584	0.789741699	0.295213719	0.767830622	0.999968657
3.2.1.85	6-phospho-beta-galactosidase	5.569374415	0.98859097	1.521649285	0.649683853	0.515896456	0.999968657
6.3.1.14	diphthine-ammonia ligase	0.663713212	-0.579446809	4.105659502	-0.141133674	0.887764342	0.999968657
2.1.1.287	ribosomal RNA-processing protein 8	0.26066422	-0.189111711	4.133131538	-0.045755067	0.963505473	0.999968657
2.8.1.13	tRNA-uridine 2-sulfurtransferase	511.8885342	-0.250434983	0.289840695	-0.864043551	0.387563967	0.999968657

4.2.3.3	methylglyoxal synthase	510.2834238	-0.200035876	0.291869875	-0.68535979	0.493116911	0.999968657
4.3.1.17	L-serine dehydratase	492.0753982	0.110897329	0.279741121	0.396428414	0.691789008	0.999968657
6.3.3.2	glutamate formiminotransferase / 5-formyltetrahydrofolate cyc	201.089566	-0.030887843	0.219616718	-0.140644314	0.888150938	0.999968657
5.3.3.8	3-hydroxyacyl-CoA dehydrogenase / enoyl-CoA hydratase / 3-h	0.732925299	-0.64195411	4.11766367	-0.155902512	0.876109865	0.999968657
3.5.3.1	arginase	24.36303767	-0.155556759	0.688551497	-0.225918845	0.82126454	0.999968657
4.1.3.27	anthranilate synthase / indole-3-glycerol phosphate synthase	268.9554381	-0.034813458	0.42775011	-0.081387374	0.93513389	0.999968657
6.1.1.17	glutamyl-tRNA synthetase	495.3645692	-0.111728026	0.260043258	-0.429651693	0.667449028	0.999968657
3.4.23.43	leader peptidase (prepilin peptidase) / N-methyltransferase	105.4913545	-0.14167366	0.665009379	-0.213040093	0.831295691	0.999968657
3.5.1.6	beta-ureidopropionase	41.72853179	0.279001283	0.485323581	0.574876832	0.565374599	0.999968657
2.1.1.197	malonyl-CoA O-methyltransferase	55.86749342	-0.777871911	0.915830146	-0.84936264	0.395679535	0.999968657
4.1.99.19	2-iminoacetate synthase	230.7809752	0.124699078	0.35198857	0.354270248	0.723136362	0.999968657
3.6.1.27	phosphatidylglycerophosphatase B	463.6147504	0.053031517	0.344438785	0.153964998	0.877637334	0.999968657
3.5.3.12	agmatine deiminase	62.47918888	0.155303758	0.468559182	0.331449609	0.740304898	0.999968657
2.8.3.17	cinnamoyl-CoA:phenyllactate CoA-transferase	0.78183725	3.161104825	4.046067261	0.781278368	0.43463879	0.999968657
2.1.1.260	rRNA small subunit pseudouridine methyltransferase Nep1	2.083596875	-0.366397	4.097386609	-0.089422121	0.928746444	0.999968657
1.7.1.15	nitrite reductase (NADH) large subunit	3.939076588	1.746669038	1.425842209	1.225008649	0.220571962	0.999968657
4.1.3.17	4-hydroxy-4-methyl-2-oxoglutarate aldolase	0.138732965	1.148719535	4.121679396	0.278701816	0.780473668	0.999968657
2.7.7.67	CDP-2,3-bis-(O-geranylgeranyl)-sn-glycerol synthase	0.353849542	-0.571200653	4.116366261	-0.138763321	0.889637179	0.999968657
2.5.1.1	farnesyl diphosphate synthase	376.183266	-0.19025838	0.19704993	-0.965533862	0.334277484	0.999968657
1.14.13.127	3-(3-hydroxy-phenyl)propionate hydroxylase	0.111464417	1.546221436	4.136560128	0.373794019	0.708557569	0.999968657
5.3.1.23	methylthioribose-1-phosphate isomerase	68.25774551	0.314736637	0.364184851	0.864222209	0.387465834	0.999968657
1.2.1.41	glutamate-5-semialdehyde dehydrogenase	370.2096274	-0.027205578	0.195498628	-0.139159942	0.889323762	0.999968657
4.2.1.44	inosose dehydratase	44.65464953	0.185368122	0.53640579	0.345574424	0.72966257	0.999968657
3.5.4.13	dCTP deaminase	6.87186702	-0.455244784	0.714321758	-0.637310538	0.523922588	0.999968657
2.1.1.228	tRNA (guanine37-N1)-methyltransferase	241.8350834	-0.089513856	0.241481259	-0.370686556	0.710871005	0.999968657
1.1.1.17	mannitol-1-phosphate 5-dehydrogenase	25.46882439	0.622256656	0.568750293	1.094077074	0.273921177	0.999968657
1.2.1.16	succinate-semialdehyde dehydrogenase / glutarate-semialdehy	8.506514607	-0.358479854	0.979397346	-0.366020855	0.714349514	0.999968657
3.1.4.46	glycerophosphoryl diester phosphodiesterase	320.093814	0.369677055	0.442861755	0.834745946	0.403860766	0.999968657
1.1.1.88	hydroxymethylglutaryl-CoA reductase	4.580753034	-0.020290185	1.371541974	-0.014793704	0.988196763	0.999968657
3.5.4.19	phosphoribosyl-AMP cyclohydrolase	258.1677959	-0.321058572	0.212935632	-1.507772882	0.131612665	0.999968657
4.1.1.83	4-hydroxyphenylacetate decarboxylase large subunit	66.87725712	0.167425508	0.607768134	0.275475956	0.782950596	0.999968657
1.2.1.12	glyceraldehyde 3-phosphate dehydrogenase	3426.713094	-0.274243074	0.565205269	-0.485209692	0.627527608	0.999968657
3.6.1.21	ADP-sugar diphosphatase	1.525601218	-0.12167663	3.593717757	-0.033858149	0.972990267	0.999968657
6.5.1.3	RNA-editing ligase	69.07652995	0.561572205	0.774913987	0.724689726	0.468642399	0.999968657
1.3.8.7	acyl-CoA dehydrogenase	0.07857	-0.221522409	4.140659147	-0.05349931	0.95733408	0.999968657
3.4.24.78	spore protease	147.416855	0.453623936	0.59618704	0.760875205	0.44673161	0.999968657
3.1.3.2	acid phosphatase	20.82235118	-0.889362486	0.833873279	-1.066543933	0.28617783	0.999968657
3.2.2.20	DNA-3-methyladenine glycosylase I	67.37949366	-0.122424213	0.672436008	-0.182060763	0.85553504	0.999968657
3.5.4.25	GTP cyclohydrolase II	294.5812985	-0.00959941	0.302701388	-0.031712474	0.974701347	0.999968657
2.4.1.16	chitin synthase	0.059838501	0.010476881	4.140558968	0.002530306	0.99798111	0.999968657
3.4.22.40	bleomycin hydrolase	151.4023736	0.120481441	0.293728512	0.410179592	0.681674211	0.999968657
2.7.8.13	phospho-N-acetylmuramoyl-pentapeptide-transferase	347.4877158	-0.20389153	0.174056519	-1.1714099	0.241434055	0.999968657
1.1.3.48	3-deoxy-alpha-D-manno-octulosonate 8-oxidase	3.68938889	0.809802259	1.333262451	0.607383984	0.543596115	0.999968657
1.1.1.49	glucose-6-phosphate 1-dehydrogenase	351.7397334	-0.624512157	0.638090515	-0.978720326	0.327718186	0.999968657
5.1.3.15	glucose-6-phosphate 1-epimerase	7.796606696	2.16116707	1.421873875	1.519942878	0.128525333	0.999968657
2.7.4.23	ribose 1,5-bisphosphokinase	0.110533983	-0.435087999	4.142162324	-0.105038858	0.916344975	0.999968657
3.1.3.4	phosphatidate phosphatase	1.586426012	-0.10515665	2.803409239	-0.037510275	0.970078148	0.999968657
5.2.1.-	#N/A	2.589767527	-2.763318886	2.104470433	-1.3130709	0.189159058	0.999968657
5.1.1.1	alanine racemase	418.4676232	0.181482429	0.20600061	0.880980059	0.378328613	0.999968657
5.4.99.24	23S rRNA pseudouridine955/2504/2580 synthase	151.4035972	-0.499668325	0.320978829	-1.556701812	0.119541298	0.999968657
1.1.1.95	D-3-phosphoglycerate dehydrogenase / 2-oxoglutarate reducta	655.6787153	-0.36143578	0.34411375	-1.050338091	0.293562698	0.999968657
3.4.21.26	prolyl oligopeptidase	48.70671163	-0.190772529	0.673434953	-0.283282786	0.776960072	0.999968657
4.2.1.159	dTDP-4-dehydro-6-deoxy-alpha-D-glucopyranose 2,3-dehydrata	3.686345816	5.205554315	2.376126076	2.190773616	0.028468179	0.999968657
3.4.24.84	STE24 endopeptidase	7.536420039	0.537019736	0.964063583	0.557037674	0.577501689	0.999968657
2.7.8.41	cardiolipin synthase (CMP-forming)	61.66824824	-0.521889359	0.579307673	-0.900884595	0.367649682	0.999968657
2.7.8.15	UDP-N-acetylglucosamine--dolichyl-phosphate N-acetylglucosa	1.224751113	-0.708022292	4.099509735	-0.172709016	0.862880157	0.999968657
3.1.3.45	3-deoxy-D-manno-octulosonate 8-phosphate phosphatase (KD(	69.92550246	-0.222044019	0.486986492	-0.455955191	0.648422201	0.999968657
2.4.1.212	hyaluronan synthase	0.179400451	0.659028218	4.122714407	0.159852988	0.872996882	0.999968657
2.4.2.44	5'-methylthioinosine phosphorylase	0.035008583	-0.204325554	4.14032189	-0.049350161	0.960640245	0.999968657
3.1.4.55	phosphoribosyl 1,2-cyclic phosphate phosphodiesterase	141.2270294	-0.113814836	0.371803266	-0.306115752	0.759516515	0.999968657
1.10.3.14	#N/A	364.9435136	0.075639355	0.420708437	0.179790439	0.85731709	0.999968657
6.3.4.13	phosphoribosylamine---glycine ligase	488.5378408	-0.140587562	0.149776853	-0.938646791	0.347912121	0.999968657
2.7.1.22	HTH-type transcriptional regulator, transcriptional repressor of	0.749927376	0.308763889	3.7469284	0.082404534	0.934325031	0.999968657
1.1.1.1	alcohol dehydrogenase	554.2128909	-0.270538354	0.430943348	-0.627781714	0.530146947	0.999968657
2.7.11.13	classical protein kinase C alpha type	1.08659773	-0.257229859	4.101020588	-0.062723377	0.949986782	0.999968657
4.2.1.120	4-hydroxybutyryl-CoA dehydratase / vinylacetyl-CoA-Delta-isom	56.64405634	-0.367234314	0.41910881	-0.876226663	0.380906824	0.999968657
5.3.1.1	triosephosphate isomerase (TIM)	980.3903779	-0.711733756	0.457027107	-1.557311909	0.119396449	0.999968657
1.2.1.58	phenylglyoxylate dehydrogenase alpha subunit	0.14203695	-3.689180797	4.143731151	-0.890304091	0.373302625	0.999968657
2.7.1.78	bifunctional polynucleotide phosphatase/kinase	2.130257046	-1.589534365	2.567265359	-0.619154681	0.535814463	0.999968657
3.4.19.12	ubiquitin carboxyl-terminal hydrolase MINDY-1/2	14.16158211	1.533260392	1.860982729	0.823898238	0.409997381	0.999968657
1.6.5.5	NADPH2:quinone reductase	5.194413046	-0.833104825	3.394933975	-0.245396474	0.806149459	0.999968657
5.4.99.-	#N/A	5.046551887	-0.239184106	2.525385047	-0.094711936	0.924543637	0.999968657
6.1.1.10	methionyl-tRNA synthetase	823.9019929	-0.539274534	0.349649022	-1.542331023	0.122993174	0.999968657
6.2.1.1	acetyl-CoA synthetase	128.7675066	0.326625924	0.291980942	1.118654942	0.263287374	0.999968657
3.2.1.35	hyaluronoglucosaminidase	600.3728921	-0.253583526	0.598667276	-0.42358007	0.671872093	0.999968657
1.3.1.31	2-enoate reductase	39.40553896	0.893866046	0.699124641	1.278550338	0.201055448	0.999968657
2.7.7.85	diadenylate cyclase	249.0442064	0.07552646	0.290942775	0.259592148	0.795178394	0.999968657
2.5.1.55	2-dehydro-3-deoxyphosphooctonate aldolase (KDO 8-P synthas	101.1365769	-0.164590959	0.395344525	-0.41632285	0.677173775	0.999968657
3.1.12.1	CRISPR-associated exonuclease Cas4	70.35374821	0.065805695	0.38535688	0.17076559	0.864408087	0.999968657
3.6.1.45	UDP-sugar diphosphatase	164.3997029	0.524334314	0.355002335	1.476988352	0.13967876	0.999968657
1.5.5.1	electron-transferring-flavoprotein dehydrogenase	0.962125619	-0.870644401	4.100391435	-0.212332021	0.831848016	0.999968657
2.7.4.-	#N/A	274.03306	-0.187788612	0.230591901	-0.814376442	0.41542933	0.999968657
6.3.2.5	phosphopantothenoylcysteine decarboxylase / phosphopantot	273.46446	-0.056781766	0.156825203	-0.362070416	0.717299411	0.999968657
4.2.1.1	carbonic anhydrase	88.34783082	-0.049314264	0.313451457	-0.15732664	0.874987426	0.999968657
4.2.1.119	(3R)-3-hydroxyacyl-CoA dehydrogenase / 3a,7a,12a-trihydroxy-	0.33160195	-0.95758384	4.122024005	-0.232309137	0.816297916	0.999968657
2.7.11.-	#N/A	267.8154515	-0.133632744	0.220783576	-0.605265785	0.545002411	0.999968657
2.7.7.15	choline-phosphate cytidylyltransferase	8.165197538	0.421326546	1.085541416	0.388125722	0.697922998	0.999968657

4.1.2.53	2-dehydro-3-deoxy-L-rhamnonate aldolase	0.020495196	-0.019393708	4.142686886	-0.004681432	0.996264771	0.999968657
2.3.1.191	UDP-3-O-[3-hydroxymyristoyl] glucosamine N-acyltransferase	114.3876791	0.185483254	0.30907358	0.600126525	0.548421916	0.999968657
3.1.31.1	micrococcal nuclease	28.70376923	2.958942602	1.478728452	2.001004713	0.045391882	0.999968657
3.4.21.53	ATP-dependent Lon protease	752.9861725	0.151427184	0.152339074	0.994014078	0.320216014	0.999968657
2.7.1.63	polyphosphate glucokinase	1.077532702	-1.772711203	4.094761573	-0.432921715	0.665071644	0.999968657
2.1.1.205	tRNA (cytidine32/guanosine34-2'-O)-methyltransferase	2.365052993	-0.593703947	3.983253934	-0.149049987	0.88151419	0.999968657
4.1.1.50	S-adenosylmethionine decarboxylase	17.97606195	0.581610772	0.634841058	0.916151791	0.359587287	0.999968657
2.4.1.187	N-acetylglucosaminyldiphosphoundecaprenol N-acetyl-beta-D-r	145.0502687	-0.229471539	0.370417277	-0.619494697	0.535590515	0.999968657
6.3.1.20	lipoate---protein ligase	123.1964858	0.126151706	0.602052994	0.209535884	0.834029926	0.999968657
2.7.7.38	3-deoxy-manno-octulosonate cytidylyltransferase (CMP-KDO sy	88.70939335	-0.059739786	0.420932952	-0.141922331	0.887141355	0.999968657
2.4.1.279	nigerose phosphorylase	0.936136296	-1.997473349	4.096902084	-0.487557014	0.625863649	0.999968657
3.1.1.85	pimeloyl-[acyl-carrier protein] methyl ester esterase	49.33588044	-0.403146596	0.866440361	-0.465290647	0.641723332	0.999968657
3.2.1.130	glycoprotein endo-alpha-1,2-mannosidase	1.452736613	0.21173134	3.613897502	0.058588087	0.9532802	0.999968657
4.1.1.33	diphosphomevalonate decarboxylase	3.592499223	0.568359433	1.573957034	0.361102254	0.718023008	0.999968657
6.3.2.-	#N/A	3.844159933	1.842665133	1.467700517	1.255477607	0.209305432	0.999968657
2.4.1.-	#N/A	1222.267335	-0.547100733	0.395808061	-1.382237471	0.166898798	0.999968657
4.2.3.5	chorismate synthase	374.3081163	0.04005902	0.263156057	0.152225339	0.879009209	0.999968657
1.12.2.1	NiFe] hydrogenase large subunit	10.41753835	2.079540177	1.142691554	1.819861335	0.068780124	0.999968657
3.7.1.22	3D-(3,5/4)-trihydroxycyclohexane-1,2-dione acylhydrolase (dec	50.10551438	0.251338707	0.39525495	0.635890094	0.52484806	0.999968657
3.5.4.1	cytosine/creatinine deaminase	18.19951039	-0.050782721	0.736271792	-0.068972791	0.945011277	0.999968657
4.1.3.38	4-amino-4-deoxychorismate lyase	50.77012324	-0.511718537	0.800528224	-0.639226103	0.522675853	0.999968657
6.4.1.2	acetyl-CoA carboxylase / biotin carboxylase 2	597.5241611	-0.091230945	0.349077907	-0.261348378	0.793823862	0.999968657
6.5.1.2	DNA ligase (NAD+)	464.5080408	0.234208478	0.224190495	1.044685135	0.296168517	0.999968657
1.3.3.3	coproporphyrinogen III oxidase	1.057957104	1.104970384	4.068404685	0.271597953	0.785931174	0.999968657
5.1.99.1	methylmalonyl-CoA/ethylmalonyl-CoA epimerase	69.18936228	-0.606887234	0.368571849	-1.646591391	0.099642061	0.999968657
1.7.99.1	hydroxylamine reductase	187.9977427	0.009818134	0.47534325	0.020654831	0.983521001	0.999968657
5.3.1.22	hydroxypyruvate isomerase	4.146248837	0.14205887	1.194516607	0.118925822	0.905334124	0.999968657
4.2.1.-	#N/A	0.500212525	-0.533226887	4.111462545	-0.129692751	0.896809518	0.999968657
2.1.1.56	mRNA (guanine-N7-)-methyltransferase	1.79241483	-0.04070499	4.097245789	-0.00993472	0.992073371	0.999968657
6.2.1.22	[citrate (pro-3S)-lyase] ligase	21.13495036	0.393642596	0.509930229	0.771953835	0.440141771	0.999968657
1.17.2.1	nicotinate dehydrogenase subunit A	0.166515913	-0.595768993	4.138090197	-0.143971969	0.885522605	0.999968657
5.3.2.1	phenylpyruvate tautomerase	0.940931647	-0.412010619	4.103508587	-0.100404473	0.920023218	0.999968657
1.1.1.8	glycerol-3-phosphate dehydrogenase (NAD+)	2.798147256	-0.228600964	3.000485462	-0.076187992	0.939269536	0.999968657
2.7.1.53	L-xylulokinase	7.910272842	0.463049168	0.808244772	0.572907099	0.566707598	0.999968657
2.7.7.77	molybdenum cofactor guanylyltransferase	16.25494552	0.001016038	0.873723943	0.001162882	0.999072155	0.999968657
1.1.1.87	homoisocitrate dehydrogenase	0.166515913	-0.595768993	4.138090197	-0.143971969	0.885522605	0.999968657
6.3.2.31	coenzyme F420-0:L-glutamate ligase / coenzyme F420-1:gamm	1.257810662	-1.868418675	4.096129299	-0.456142504	0.648287507	0.999968657
3.2.1.31	beta-glucuronidase	65.88671643	0.014775487	0.520515062	0.028386282	0.977354065	0.999968657
2.4.1.255	protein O-GlcNAc transferase	1.352957927	-0.539613263	4.099468878	-0.131630043	0.895276922	0.999968657
3.2.-.-	#N/A	49.66509319	-0.207382464	0.670710637	-0.309198114	0.757170829	0.999968657
2.3.1.22	2-acylglycerol O-acyltransferase 3	2.917573992	-0.571537739	4.095638197	-0.139547907	0.889017202	0.999968657
2.4.1.8	maltose phosphorylase	23.28563407	-0.347138168	0.728093132	-0.476777149	0.633520819	0.999968657
3.5.1.96	succinylglutamate desuccinylase	0.040667486	-0.039843063	4.143731151	-0.009615263	0.992328249	0.999968657
3.4.11.1	leucyl aminopeptidase	44.43899661	0.165864547	0.41152855	0.403045056	0.686915079	0.999968657
2.3.2.26	other hect domain ubiquitin protein ligase E3	3.299592357	-0.482292693	3.030278628	-0.15915787	0.873544495	0.999968657
2.5.1.74	1,4-dihydroxy-2-naphthoate polyprenyltransferase	275.5790845	0.713530629	0.720655904	0.990112792	0.322118992	0.999968657
1.1.1.156	glycerol 2-dehydrogenase (NADP+)	0.687316943	2.16129095	4.090324285	0.5283911	0.597227913	0.999968657
3.5.2.14	#N/A	21.13792353	-0.659243256	1.10136771	-0.598567809	0.549461128	0.999968657
2.3.1.28	chloramphenicol O-acetyltransferase type B	85.27593719	-0.30399492	0.503443225	-0.603831585	0.545955618	0.999968657
2.1.1.174	23S rRNA (guanine1835-N2)-methyltransferase	0.193014423	-0.874405328	4.133883336	-0.21152153	0.832480334	0.999968657
5.1.1.13	aspartate racemase	62.58129594	0.511175424	0.52818722	0.96779211	0.333148205	0.999968657
3.5.2.6	beta-lactamase class C	211.9174155	-0.240613264	0.275212867	-0.874280574	0.381965476	0.999968657
2.7.1.24	dephospho-CoA kinase	234.9531747	0.05213163	0.248350863	0.20991121	0.833736973	0.999968657
3.6.3.55	#N/A	1.048181113	-0.930184743	4.081479314	-0.227903824	0.81972101	0.999968657
3.13.1.1	UDP-sulfoquinovose synthase	0.039493742	1.031690707	4.143245313	0.24900546	0.803356558	0.999968657
2.1.1.185	23S rRNA (guanosine2251-2'-O)-methyltransferase	292.4598568	0.091189159	0.178722969	0.510226296	0.609892932	0.999968657
3.1.3.25	myo-inositol-1(or 4)-monophosphatase	93.69475451	0.070451416	0.400323151	0.175986364	0.860304655	0.999968657
2.7.7.71	D-glycero-alpha-D-manno-heptose 1-phosphate guanylyltransfe	3.00025743	-0.640685795	2.724661669	-0.235143248	0.814097552	0.999968657
3.4.25.1	#N/A	26.70507566	-1.008240751	2.823286677	-0.357115967	0.721004992	0.999968657
3.6.3.9	sodium/potassium-transporting ATPase subunit alpha	1.356910129	0.554730174	3.685132124	0.150531963	0.880344935	0.999968657
1.5.1.20	methylenetetrahydrofolate reductase (NADPH)	318.0389004	-0.25811304	0.181267077	-1.423937788	0.154464482	0.999968657
1.13.11.4	gentisate 1,2-dioxygenase	0.110533983	-0.435087999	4.142162324	-0.105038858	0.916344975	0.999968657
1.2.1.9	glyceraldehyde-3-phosphate dehydrogenase (NADP+)	5.223169037	0.308086968	1.610181416	0.191336805	0.848261727	0.999968657
1.3.98.1	dihydroorotate dehydrogenase (fumarate)	176.8433461	-0.274157915	0.594516936	-0.461143994	0.6446953	0.999968657
3.1.3.100	ribosome biogenesis GTPase / thiamine phosphate phosphatase	362.0250623	-0.166260274	0.22583925	-0.736188571	0.461615947	0.999968657
2.1.1.176	16S rRNA (cytosine967-C5)-methyltransferase	285.324976	0.277108858	0.206210125	1.343817908	0.179007277	0.999968657
3.5.1.2	glutaminase	188.4444747	0.525485524	0.469711683	1.118740589	0.263250824	0.999968657
5.5.1.2	3-carboxy-cis,cis-muconate cycloisomerase	0.801064419	0.371702899	4.069895045	0.091329849	0.927230501	0.999968657
1.8.1.9	thioredoxin reductase (NADPH)	2323.064914	0.074827552	0.477876148	0.156583567	0.875573054	0.999968657
2.5.1.134	cystathionine beta-synthase (O-acetyl-L-serine)	0.078537166	0.320814183	4.143731151	0.077421573	0.93828818	0.999968657
5.1.3.29	L-fucose mutarotase	71.42123421	-0.963640828	0.545378757	-1.766920357	0.077241567	0.999968657
1.1.1.276	serine 3-dehydrogenase (NADP+)	0.122131417	-0.222758921	4.140682777	-0.05379763	0.957096397	0.999968657
3.2.1.41	pullulanase	58.97450458	-0.048278029	0.373214031	-0.129357487	0.897074786	0.999968657
2.7.1.100	5-methylthioribose kinase	21.01730638	-0.21030996	0.66890094	-0.314411218	0.753208737	0.999968657
2.7.1.83	pseudouridine kinase	71.962011	0.516012668	0.380194421	1.357233666	0.174706977	0.999968657
4.2.2.1	hyaluronate lyase	44.64325423	1.235369171	0.61707257	2.001983608	0.045286495	0.999968657
2.3.1.187	malonate decarboxylase alpha subunit	9.455022523	0.818149701	1.17529946	0.696120205	0.486353554	0.999968657
2.7.11.22	CTD kinase subunit alpha	8.228143643	-0.507152828	4.093858482	-0.123881378	0.901409198	0.999968657
5.3.3.17	trans-2,3-dihydro-3-hydroxyanthranilate isomerase	0.035008583	-0.204325554	4.14032189	-0.049350161	0.960640245	0.999968657
2.5.1.46	deoxyhypusine synthase	3.308839742	-0.295780831	2.39100777	-0.123705508	0.90154845	0.999968657
2.8.3.21	L-carnitine CoA-transferase	1.461921331	-0.975429775	3.187126037	-0.306053091	0.759564223	0.999968657
6.3.2.10	UDP-N-acetylmuramoyl-tripeptide--D-alanyl-D-alanine ligase	353.4964146	0.031968546	0.167146134	0.191261056	0.848321071	0.999968657
2.7.1.50	hydroxyethylthiazole kinase	118.3186211	-0.02960387	0.368443921	-0.080348373	0.935960187	0.999968657
3.4.22.16	cathepsin H	6.140464691	0.227371019	4.13620262	0.054970958	0.956161601	0.999968657
2.1.1.258	5-methyltetrahydrofolate corrinoid/iron sulfur protein methyltr	251.1608261	-0.576953747	0.633582404	-0.910621481	0.362494848	0.999968657
1.7.-.-	#N/A	19.90229137	0.785709722	0.962502161	0.816319956	0.414317164	0.999968657
4.2.2.21	chondroitin-sulfate-ABC endolyase/exolyase	92.73215075	-0.007293624	0.634134935	-0.011501692	0.99082318	0.999968657

1.1.1.76	meso-butanediol dehydrogenase / (S,S)-butanediol dehydrogen	2.544822965	0.675197034	1.895219804	0.35626318	0.721643481	0.999968657
2.4.1.83	dolichol-phosphate mannosyltransferase	101.4455041	-0.267992831	0.339402744	-0.789601249	0.429760678	0.999968657
2.7.1.147	ADP-dependent phosphofructokinase/glucokinase	1.596352927	-0.802041453	2.962026581	-0.270774563	0.786564425	0.999968657
4.2.2.5	chondroitin AC lyase	46.22719144	-0.293467957	0.861300273	-0.34072665	0.733309374	0.999968657
2.1.2.5	glutamate formiminotransferase / 5-formyltetrahydrofolate cyc	152.7191343	0.173354592	0.47522292	0.36478584	0.715271276	0.999968657
1.3.1.44	enoyl-acyl-carrier protein] reductase / trans-2-enoyl-CoA reduc	11.55160526	0.546020647	0.846576355	0.644975074	0.518943339	0.999968657
1.5.1.41	aquacobalamin reductase / NAD(P)H-flavin reductase	1.401765292	-0.760728593	3.954415065	-0.192374493	0.847448871	0.999968657
3.5.1.38	glutamin-(asparagin-)ase	0.486306948	-0.898040745	4.109591765	-0.218523103	0.82702156	0.999968657
2.7.1.205	PTS system, cellobiose-specific IIA component	42.12668325	-0.706336632	0.49865234	-1.416491159	0.156631751	0.999968657
3.2.1.20	alpha-glucosidase	623.9134192	-0.008577469	0.373691175	-0.022953362	0.981687475	0.999968657
1.13.12.16	nitronate monooxygenase	2.660689283	0.89638067	1.625058477	0.551599024	0.581223105	0.999968657
2.4.1.261	alpha-1,2-mannosyltransferase	1.191163093	-0.451800965	4.099581595	-0.110206604	0.912245525	0.999968657
2.1.1.191	23S rRNA (cytosine1962-C5)-methyltransferase	406.1474614	-0.063540576	0.196123205	-0.323982956	0.745950938	0.999968657
3.7.1.3	kynureninase	0.600135268	-0.245541175	4.11825748	-0.059622589	0.952456227	0.999968657
2.4.2.22	xanthine phosphoribosyltransferase	289.7354219	0.440528399	0.452007446	0.974604296	0.329756582	0.999968657
4.6.1.-	#N/A	0.249773869	-0.816552953	4.124382892	-0.19798185	0.843059265	0.999968657
6.1.1.15	prolyl-tRNA synthetase	485.2734754	0.004185319	0.151958864	0.02754245	0.978027083	0.999968657
4.4.1.-	#N/A	0.783122018	0.785325507	4.100574003	0.191515994	0.848121351	0.999968657
2.3.2.6	leucyl/phenylalanyl-tRNA---protein transferase	29.52000004	0.304595698	0.658478964	0.462574682	0.64366926	0.999968657
2.3.1.78	heparan-alpha-glucosaminide N-acetyltransferase	0.457925214	-0.465426518	4.112742167	-0.113166958	0.909898191	0.999968657
1.1.1.47	glucose 1-dehydrogenase	18.79187857	0.417620533	1.150519129	0.362984432	0.716616514	0.999968657
3.1.6.1	arylsulfatase	68.00014758	-0.38205637	0.861507871	-0.443474033	0.657422898	0.999968657
6.2.1.45	ubiquitin-activating enzyme E1	6.168451976	-1.549613359	2.120681571	-0.730714776	0.464953388	0.999968657
2.7.1.148	4-diphosphocytidyl-2-C-methyl-D-erythritol kinase	306.3553243	-0.017838723	0.265358992	-0.067224869	0.946402688	0.999968657
1.2.1.3	aldehyde dehydrogenase (NAD+)	54.50178897	-0.142601305	0.498331666	-0.286157422	0.774757553	0.999968657
3.5.1.81	N-acyl-D-amino-acid deacylase	49.05676068	0.697733629	0.545968061	1.277975177	0.201258179	0.999968657
5.4.99.12	#N/A	317.454115	-0.090632191	0.239671667	-0.37815146	0.705318082	0.999968657
2.3.1.16	acetyl-CoA acyltransferase	1.009606782	-0.292019991	3.517699667	-0.083014475	0.93384003	0.999968657
4.2.1.126	N-acetylmuramic acid 6-phosphate etherase	72.37809151	0.26028007	0.412518675	0.630953423	0.528070979	0.999968657
3.5.3.6	arginine deiminase	75.43333123	0.233480967	0.467753038	0.499154358	0.617670646	0.999968657
2.7.7.56	ribonuclease PH	38.61928976	0.762750195	0.603948366	1.262939413	0.206610954	0.999968657
2.4.1.166	raffinose-raffinose alpha-galactotransferase	0.473040041	0.936101905	4.099976404	0.228318852	0.81939837	0.999968657
2.7.8.39	archaetidylinositol phosphate synthase	0.782416094	0.16453376	4.100890669	0.04012147	0.967996285	0.999968657
3.1.27.-	#N/A	2.565835127	-0.248089576	3.186670465	-0.077852284	0.937945556	0.999968657
6.3.4.3	methylenetetrahydrofolate dehydrogenase (NADP+) / metheny	810.1039202	-0.185033943	0.289376953	-0.639421837	0.522548547	0.999968657
5.3.2.8	4-oxalomesaconate tautomerase	0.020495196	-0.019393708	4.142686886	-0.004681432	0.996264771	0.999968657
1.1.3.9	galactose oxidase	0.296485225	-0.436149509	4.125686142	-0.10571563	0.915807979	0.999968657
2.7.7.59	[protein-PII] uridylyltransferase	10.91023372	0.244223807	1.158622828	0.210788015	0.833052691	0.999968657
3.6.1.54	UDP-2,3-diacylglucosamine hydrolase	93.92320793	-0.788624222	0.516049087	-1.528196137	0.126463848	0.999968657
3.4.11.19	D-aminopeptidase	57.32240294	0.5663323	0.452841483	1.250619304	0.211073404	0.999968657
2.7.1.199	PTS system, glucose-specific IIB component	59.39654169	-0.278399279	0.592403617	-0.469948649	0.638391706	0.999968657
2.7.7.41	phosphatidate cytidylyltransferase	426.9388583	-0.089297097	0.23195711	-0.38497245	0.700257827	0.999968657
2.1.1.74	methylenetetrahydrofolate--tRNA-(uracil-5-)-methyltransferase	123.201116	0.162508754	0.358344705	0.453498411	0.650189887	0.999968657
2.7.6.2	thiamine pyrophosphokinase	207.1046171	-0.378137442	0.190802304	-1.981828492	0.047498439	0.999968657
4.3.1.18	D-serine dehydratase	35.25449199	-0.067859446	0.453541085	-0.149621386	0.881063335	0.999968657
2.5.1.108	2-(3-amino-3-carboxypropyl)histidine synthase	0.899296214	-0.560839387	4.102531859	-0.136705675	0.891263444	0.999968657
2.4.1.1	glycogen phosphorylase	967.5698812	0.031000058	0.194301748	0.159545956	0.873238754	0.999968657
4.2.1.30	#N/A	13.82081441	-2.321404588	1.11067322	-2.090087837	0.03660991	0.999968657
2.7.1.91	sphingosine kinase	0.070017166	-0.444808383	4.138829611	-0.107472021	0.914414521	0.999968657
2.7.7.65	two-component system, cell cycle response regulator	2.26188146	-2.053799196	2.253701493	-0.911300455	0.362137087	0.999968657
1.3.5.4	succinate dehydrogenase / fumarate reductase, flavoprotein su	851.5573064	0.067680042	0.231506417	0.292346287	0.770021872	0.999968657
3.6.3.4	Cu2+-exporting ATPase	720.9885904	0.378907165	0.451625093	0.838986077	0.401477123	0.999968657
2.1.3.9	N-acetylornithine carbamoyltransferase	0.471782961	-0.832302845	4.130224099	-0.201515178	0.840295762	0.999968657
1.1.5.6	#N/A	0.834744568	-0.719063533	4.11411895	-0.174779471	0.86125292	0.999968657
2.4.1.52	poly(glycerol-phosphate) alpha-glucosyltransferase	0.626809568	1.431366273	4.120341518	0.347390202	0.728298193	0.999968657
4.1.1.31	phosphoenolpyruvate carboxylase	14.51864403	-0.121820886	0.706207125	-0.172500222	0.863044287	0.999968657
4.1.2.22	xylulose-5-phosphate/fructose-6-phosphate phosphoketolase	113.3229537	0.620463091	0.300501517	2.064758597	0.038945847	0.999968657
3.6.3.25	sulfate transport system ATP-binding protein	37.65838914	0.44415855	0.772306169	0.575106826	0.565219052	0.999968657
3.6.1.15	#N/A	4.552541307	0.554924864	1.52320729	0.364313424	0.715623976	0.999968657
3.6.3.36	taurine transport system ATP-binding protein	1.346509197	0.34697269	2.655344128	0.130669576	0.896036701	0.999968657
3.2.1.73	licheninase	7.463162102	-3.238642029	1.741355262	-1.859839918	0.062908178	0.999968657
2.1.1.309	18S rRNA (guanine1575-N7)-methyltransferase	2.903351773	-0.357448523	3.779468215	-0.094576407	0.924651291	0.999968657
1.3.8.1	butyryl-CoA dehydrogenase	257.9828952	0.413981072	0.5201737	0.795851601	0.426118295	0.999968657
1.8.5.3	anaerobic dimethyl sulfoxide reductase subunit A	14.50986278	-0.378387186	0.696342934	-0.543392009	0.586859926	0.999968657
5.4.3.2	lysine 2,3-aminomutase	201.7077419	-0.100041591	0.396004843	-0.25262719	0.800556317	0.999968657
2.6.1.2	alanine transaminase	96.41482852	-0.281238985	0.514193336	-0.546951828	0.584411827	0.999968657
1.3.1.74	2-alkenal reductase	0.667842043	-3.469536213	4.143731151	-0.837297616	0.402425299	0.999968657
3.1.3.48	protein-tyrosine phosphatase	496.7287062	0.050107026	0.298265625	0.167994641	0.866587495	0.999968657
5.4.99.29	tRNA pseudouridine32 synthase / 23S rRNA pseudouridine746 s	66.23259907	-0.017930215	0.572869243	-0.031298966	0.975031115	0.999968657
3.1.21.4	type II restriction enzyme	19.39455655	-1.670507058	0.732940609	-2.27918475	0.022656085	0.999968657
2.3.1.97	glycylpeptide N-tetradecanoyltransferase	2.404544264	-0.469087667	3.866486132	-0.12132144	0.903436438	0.999968657
4.6.1.16	tRNA-intron endonuclease, archaea type	0.578535221	-0.256206494	4.103867421	-0.0624305	0.950220007	0.999968657
1.18.1.3	3-phenylpropionate/trans-cinnamate dioxygenase ferredoxin re	0.36309423	-1.810954875	4.119081808	-0.439650135	0.660190523	0.999968657
3.1.6.6	choline-sulfatase	24.56530346	-0.213639041	0.894633403	-0.238800653	0.811260164	0.999968657
1.8.5.2	thiosulfate dehydrogenase [quinone] small subunit	17.43969102	-1.149323396	1.177892899	-0.975745246	0.329190725	0.999968657
6.3.1.8	glutathionylspermidine amidase/synthetase	1.249834218	1.268291995	3.316259165	0.382446586	0.702130141	0.999968657
2.3.1.9	acetyl-CoA C-acetyltransferase	806.2977231	-0.538599995	0.572199436	-0.941280192	0.346561291	0.999968657
1.1.1.28	D-lactate dehydrogenase	327.873437	-0.23860108	0.463725464	-0.514530898	0.606880864	0.999968657
2.7.1.167	D-beta-D-heptose 7-phosphate kinase / D-beta-D-heptose 1-ph	30.61416296	0.660339415	0.682838187	0.967051093	0.333518493	0.999968657
4.3.2.2	adenylosuccinate lyase	603.0231759	-0.155282329	0.230275527	-0.674332749	0.500099788	0.999968657
6.2.1.31	2-furoate---CoA ligase	0.078987483	1.38698787	4.130542208	0.33578833	0.737030491	0.999968657
3.2.1.139	alpha-glucuronidase	61.56210552	0.402184292	0.666840128	0.60311951	0.546429188	0.999968657
4.2.1.77	trans-L-3-hydroxyproline dehydratase	0.556212935	1.240365209	4.108981499	0.301866828	0.762753585	0.999968657
2.3.1.89	tetrahydrodipicolinate N-acetyltransferase	3.32986813	-3.043370337	1.39041638	-2.188819393	0.02860997	0.999968657
1.5.1.2	pyrroline-5-carboxylate reductase	402.521796	-0.485294936	0.448303066	-1.082515319	0.279023612	0.999968657
1.7.2.5	nitric oxide reductase subunit B	0.040990391	-0.227404664	4.140771598	-0.054918427	0.956203451	0.999968657
2.4.1.11	glycogen synthase	55.29743903	1.055816805	0.647970225	1.62942179	0.103223759	0.999968657

2.8.1.6	biotin synthase	211.0746319	-0.180164719	0.328645676	-0.548203528	0.58355216	0.999968657
2.7.1.71	shikimate kinase	453.6115401	0.121216251	0.386686605	0.313474141	0.753920464	0.999968657
3.5.1.47	N-acetyldiaminopimelate deacetylase	5.108520293	1.953104785	1.398314975	1.396755966	0.162486968	0.999968657
1.2.5.1	pyruvate dehydrogenase (quinone)	71.21907842	0.170515669	0.648043681	0.263123727	0.792455217	0.999968657
4.99.1.1	protoporphyrin/coproporphyrin ferrochelatase	7.519813609	0.301632352	0.764494212	0.394551517	0.6931739	0.999968657
2.4.2.52	triphosphoribosyl-dephospho-CoA synthase	19.79538825	-0.146617228	0.594054013	-0.246807907	0.805056891	0.999968657
1.3.4.1	fumarate reductase (CoM/CoB) subunit A	2.34115809	-1.192819098	4.091787996	-0.291515372	0.770657189	0.999968657
6.2.1.5	succinyl-CoA synthetase alpha subunit	166.7141587	-0.250781386	0.640689837	-0.391424012	0.695483846	0.999968657
4.2.1.136	ADP-dependent NAD(P)H-hydrate dehydratase	352.2405352	0.084264439	0.346721201	0.243032265	0.807980402	0.999968657
5.1.3.10	CDP-paratose 2-epimerase	0.695290102	-1.248364064	4.106414114	-0.304003452	0.761125262	0.999968657
2.7.1.153	phosphatidylinositol-4,5-bisphosphate 3-kinase catalytic subuni	1.323599265	-0.057134885	4.098065915	-0.013941915	0.988876322	0.999968657
1.5.1.39	FMN reductase [NAD(P)H]	10.96471843	-0.297843481	0.792806267	-0.375682551	0.707152912	0.999968657
4.1.99.14	spore photoproduct lyase	6.75289143	-0.724648398	1.129013945	-0.641841848	0.520975875	0.999968657
1.1.1.281	GDP-4-dehydro-6-deoxy-D-mannose reductase	10.70714889	1.015918229	1.151240713	0.882455092	0.377530754	0.999968657
6.3.1.10	adenosylcobinamide-phosphate synthase	125.3070087	0.046401412	0.379809488	0.122170227	0.902764205	0.999968657
5.4.2.7	phosphopentomutase	326.2463797	-0.411160518	0.444380319	-0.925244661	0.354838657	0.999968657
2.7.1.190	aminoglycoside 2''-phosphotransferase	3.45828159	0.295561243	2.207955324	0.133861967	0.893511725	0.999968657
1.12.98.2	5,10-methenyltetrahydromethanopterin hydrogenase	10.46252972	0.311188728	1.731229895	0.179750089	0.857348768	0.999968657
2.7.1.36	mevalonate kinase	4.067718738	0.653842849	1.856117919	0.352263637	0.724640556	0.999968657
4.2.1.91	cyclohexadienyl dehydratase	4.49090516	0.142265422	1.42834464	0.099601608	0.920660617	0.999968657
3.5.4.39	GTP cyclohydrolase IV	1.827417476	-0.429658945	4.096988295	-0.104871899	0.916477457	0.999968657
5.3.1.8	mannose-6-phosphate isomerase	421.1889985	-0.828652407	0.671834605	-1.233417274	0.217420124	0.999968657
3.1.3.71	2-phosphosulfolactate phosphatase	2.504384397	0.923376464	1.448461478	0.637487761	0.52380718	0.999968657
2.7.11.33	[pyruvate, water dikinase]-phosphate phosphotransferase / [py	9.875085199	-0.378757976	0.666282196	-0.568464801	0.5697194	0.999968657
2.7.1.19	phosphoribulokinase	0.688126876	-0.473842034	4.077001561	-0.116223167	0.907475676	0.999968657
1.2.1.24	succinate-semialdehyde dehydrogenase	0.649086519	-1.080841251	4.107072228	-0.263165874	0.792422733	0.999968657
2.7.1.168	D-glycero-alpha-D-manno-heptose-7-phosphate kinase	31.41129843	-0.197995731	0.721949891	-0.274251348	0.78389147	0.999968657
4.1.3.42	2-dehydro-3-deoxyphosphogluconate aldolase / (4S)-4-hydroxy	296.8325156	-0.525551211	0.294307081	-1.785723975	0.074143962	0.999968657
3.1.3.15	imidazoleglycerol-phosphate dehydratase / histidinol-phosphat	717.5286595	-0.427650836	0.457254746	-0.935257292	0.349655719	0.999968657
4.3.1.14	3-aminobutyryl-CoA ammonia-lyase	95.00817285	-0.053120885	0.512932392	-0.103563133	0.917516046	0.999968657
1.8.2.2	thiosulfate dehydrogenase	0.118481225	1.603489503	4.124415214	0.388779844	0.697439012	0.999968657
2.3.1.54	formate C-acetyltransferase	1844.276914	-0.142859063	0.521201555	-0.274095619	0.784011141	0.999968657
3.5.4.29	GTP cyclohydrolase IIa	1.229475	-0.537888679	4.099451397	-0.131209917	0.895609251	0.999968657
1.5.99.6	spermidine dehydrogenase	0.131528836	-0.593712936	4.137447435	-0.143497397	0.885897367	0.999968657
5.3.4.1	protein disulfide-isomerase	30.55821104	-1.083392107	1.056085216	-1.025856712	0.30495913	0.999968657
2.4.1.117	dolichyl-phosphate beta-glucosyltransferase	0.969773766	-0.380014932	4.101917797	-0.092643234	0.926186995	0.999968657
4.1.3.40	chorismate--pyruvate lyase	0.460350927	-1.056716244	4.113677802	-0.256878709	0.797272407	0.999968657
3.1.11.1	exodeoxyribonuclease I	5.432575923	-0.174679332	0.801792953	-0.217860897	0.827537495	0.999968657
4.4.1.24	(2R)-sulfolactate sulfo-lyase subunit alpha	6.567064541	-0.157036903	1.077027538	-0.145805839	0.884074667	0.999968657
3.5.1.11	penicillin amidase	0.017504292	-0.007393638	4.141980666	-0.001785049	0.998575738	0.999968657
3.4.14.4	dipeptidyl-peptidase III	511.5424619	-0.597790682	0.68959309	-0.866874524	0.386010763	0.999968657
3.4.21.105	rhomboid-related protein 1/2/3	65.52118998	-0.740916675	0.405732025	-1.826123229	0.067831702	0.999968657
3.1.6.-	#N/A	21.90800047	-0.132242091	0.622870682	-0.212310669	0.831864672	0.999968657
6.1.1.9	valyl-tRNA synthetase	560.2418703	0.195312659	0.236177498	0.826974037	0.408251777	0.999968657
2.7.4.25	CMP/dCMP kinase	352.665934	-0.005349581	0.238207548	-0.022457646	0.982082897	0.999968657
1.2.1.59	glyceraldehyde-3-phosphate dehydrogenase (NAD(P))	5.22268142	-0.203437816	1.626837489	-0.125051099	0.900483096	0.999968657
1.1.1.25	shikimate dehydrogenase	278.5865007	-0.097914612	0.289360496	-0.33838279	0.735074742	0.999968657
4.2.1.59	3-hydroxyacyl-[acyl-carrier protein] dehydratase / trans-2-dece	228.3164338	-0.212522036	0.279748798	-0.759688828	0.447440608	0.999968657
2.3.1.182	(R)-citramalate synthase	88.16405049	-0.132259521	0.309497133	-0.427336821	0.669134018	0.999968657
2.7.1.32	choline kinase	2.466264155	-0.289218603	4.095715784	-0.07061491	0.943704243	0.999968657
4.1.1.65	phosphatidylserine decarboxylase	131.5107749	0.109187415	0.379210118	0.287933813	0.773397404	0.999968657
3.4.21.92	ATP-dependent Clp protease, protease subunit	546.6920958	0.049953052	0.286329515	0.174460015	0.861503952	0.999968657
2.7.7.61	holo-ACP synthase	18.79323321	0.006305997	0.526417627	0.011979076	0.990442308	0.999968657
2.7.1.157	N-acetylgalactosamine kinase	0.82830909	-0.413948321	4.100744014	-0.100944687	0.919594369	0.999968657
3.3.2.1	bifunctional isochorismate lyase / aryl carrier protein	0.147099389	-0.82881892	4.136777274	-0.200353769	0.841203913	0.999968657
2.7.9.2	pyruvate, water dikinase	53.46043133	0.087988021	0.433288495	0.203070291	0.839080092	0.999968657
[truncated: 979,089 more chars]
